# 8-OxoG in GC-rich Sp1 binding sites enhances gene transcription in adipose tissue of juvenile mice

**DOI:** 10.1038/s41598-019-52139-z

**Published:** 2019-10-30

**Authors:** Jong Woo Park, Young In Han, Sung Woo Kim, Tae Min Kim, Su Cheong Yeom, Jaeku Kang, Joonghoon Park

**Affiliations:** 1 Research Center for Epigenome Regulation, School of Pharmacy, Sungkyunkwan University, Suwon, 16419 Republic of Korea; 2 Institute of Green Bio Science and Technology, Seoul National University, Pyeongchang, 25354 Republic of Korea; 3 Animal Genetic Resources Research Center, National Institute of Animal Science, Rural Development Administration (RDA), Namwon, 55717 Republic of Korea; 4 Department of International Agricultural Technology, Graduate School of International Agricultural Technology, Seoul National University, Pyeongchang, 25354 Republic of Korea; 5 Department of Pharmacology, College of Medicine, Konyang University, Daejeon, 35365 Republic of Korea

**Keywords:** Epigenetics, Gene regulation

## Abstract

The oxidation of guanine to 8-oxoguanine (8-oxoG) is the most common type of oxidative DNA lesion. There is a growing body of evidence indicating that 8-oxoG is not only pre-mutagenic, but also plays an essential role in modulating gene expression along with its cognate repair proteins. In this study, we investigated the relationship between 8-oxoG formed under intrinsic oxidative stress conditions and gene expression in adipose and lung tissues of juvenile mice. We observed that transcriptional activity and the number of active genes were significantly correlated with the distribution of 8-oxoG in gene promoter regions, as determined by reverse-phase liquid chromatography/mass spectrometry (RP-LC/MS), and 8-oxoG and RNA sequencing. Gene regulation by 8-oxoG was not associated with the degree of 8-oxoG formation. Instead, genes with GC-rich transcription factor binding sites in their promoters became more active with increasing 8-oxoG abundance as also demonstrated by specificity protein 1 (Sp1)- and estrogen response element (ERE)-luciferase assays in human embryonic kidney (HEK293T) cells. These results indicate that the occurrence of 8-oxoG in GC-rich Sp1 binding sites is important for gene regulation during adipose tissue development.

## Introduction

Genetic material is constantly exposed to various kinds of deleterious factors. Those include intrinsic and extrinsic reactive oxygen species (ROS), ionizing radiation, ultraviolet light, and other reactive molecules. These can cause DNA lesions that are processed by dedicated repair pathways to protect genome integrity. Unrecovered DNA damages can induce mutations and result in cell death or uncontrolled cell proliferation^1^.

ROS either from oxidative metabolism or exposure to agents can result in oxidative DNA lesions^2^. Over 100 different types of oxidative modification have been identified to date including oxidized pyrimidines and purines, abasic sites as well as single strand breaks (SSBs)^3^. Guanine (G) is the most sensitive to oxidation because of the lowest redox potential. The resultant 8-hydroxyguanine (8-oxoG) is the most plentiful and well-characterized oxidative DNA lesion whose repair is critical since it can pair not only with cytosine (C) but also with adenine (A) during replication, resulting in a G:C to T:A transversion mutation. The major repair pathway for these lesions is base excision-repair (BER)^4,5^. In mammalian cells, 8-oxoG DNA glycosylase 1 (OGG1) is known as a bi-functional glycosylase coupled with lyase activity^6^; however, recent findings indicate that OGG1 is a primary DNA glycosylase to hydrolyze the 8-oxoG from the lesion, generating an abasic site^7,8^. Subsequently, this abasic site was occupied by apurinic/apyrimidinic endodeoxyribonuclease 1 (APEX1) to cleave the 5′ end of this site to induce an SSB having a hydroxyl group at 3′ end and sugar phosphate at 5′ end. DNA polymerase then lyases the latter part and fills the break through templated DNA synthesis. DNA ligase I or III seals the nick and completes the BER process^9^. Unrepaired 8-oxoG modifications have been implicated in cancer, neurodegenerative diseases, and aging^10^.

There is increasing evidence of transcriptional regulation associated with DNA repair. For example, nuclear factor-κ B (NFκB)-mediated pro-inflammatory gene expression is enhanced by 8-oxoG/OGG1-dependent site-specific recruitment of transcription factors^11^. 8-oxoG may also turn on or off the gene transcription depending on the strand on which it is located in the G-quadruplex gene promoter structure^12^. On the other hand, genome-wide profiling of 8-oxoG in rat renal tissues revealed that the lesion is preferentially located in the gene deserts, and that the gene expression is not related with the distribution of 8-oxoGs^13^.

8-OxoG measurement by *in situ* hybridization using an anti-8-oxoG antibody on metaphase chromosomes from human peripheral lymphocytes revealed that 8-oxoG is randomly distributed throughout human genome. Additionally, positive correlation exists between the density of 8-oxoGs and the frequency of DNA recombination and single nucleotide polymorphisms^14^. Therefore, it appears that the gene regulatory activity of 8-oxoG is controversial, and the high-resolution genomic mapping of 8-oxoG is required to address the epigenetic function of 8-oxoG.

In this study we performed genome-wide 8-oxoG profiling of adipose and lung tissues of juvenile female C57BL/6 mice by affinity purification followed by next-generation sequencing in order to clarify the genetic and molecular roles of 8-oxoG beyond its function as a DNA damage mark. We found that transcriptional activity and the number of active genes were correlated with 8-oxoG distribution, especially in gene promoters. A transcription factor binding motif analysis revealed that genes that were highly expressed - especially in adipose tissue - had GC-rich promoters as compared to those were moderately active or inactive genes. Furthermore, genes with GC-rich transcription factor binding sites in their promoters became more active with increasing 8-oxoG abundance as demonstrated by Sp1- and ERE-luciferase assays in HEK293T cells under oxidative stress condition. These results suggest that 8-oxoG promotes transcription during adipose tissue development in mice.

## Results

### Global concentrations of 8-oxoGs in various tissues of juvenile mice

Hydrolyzed genomic DNA samples from lung, liver, and adipose tissues were analyzed by RP-LC/MS to determine 8-oxoG levels. For quality assurance of the procedure, we also measured total dG and dC by HPLC. Representative chromatograms and standard curves generated with various concentrations of 8-oxoG standard are shown in Supplementary Fig. S1.The retention time of 8-oxoG was 2.9 min, and the correlation coefficient (*R*^2^) was 0.999 with 8-oxoG standard. Averaged 8-oxoG levels with standard deviation (SD) in lung, liver, and adipose tissues were 0.025% ± 0.008%, 0.054% ± 0.012%, and 0.070% ± 0.008% of total dG, respectively (Table 1). Coefficient of variation of 8-oxoG levels in different tissues was less than 32%. These global concentrations of 8-oxoG were comparable to the historical range of 8-oxoG levels in pig liver (0.0002% to 0.0441%), HeLa cells (0.0001% to 0.0214%)^15^ or commercially available calf thymus DNA (0.032%)^16^. The ratios of total dG to total dC were 1.03, 1.00, and 0.91 in lung, liver, and adipose tissues, respectively, indicating that there was no detection bias in the analysis. Since we used sexually immature mice, we might be able to avoid the aging effect on 8-oxoG formation. The level of 8-oxoG was lower in lung tissues than in liver or adipose tissues (*p* < 0.01). These results demonstrate that 8-oxoG is present at a low but significantly different level in genomic DNA of adipose and lung tissues of juvenile mice; therefore, these tissues were subjected to further analyses.

**Table 1:** Global concentrations of 8-oxoGs in various tissues of juvenile mice.

Tissue type	No. of animals	%8-oxoG/dG_total_	dG_total_/dC_total_
Lung	5	0.025 ± 0.008	1.032 ± 0.018
Liver	4	0.054 ± 0.012**	1.005 ± 0.051
Adipose	4	0.070 ± 0.008***	0.910 ± 0.088

Mean ± Standard Deviation (SD), **p < 0.01, ***p < 0.001 vs. lung, one-sided Student’s *t*-test.

### Genomic distribution of 8-oxoGs associated with gene expression

**Figure 1:**
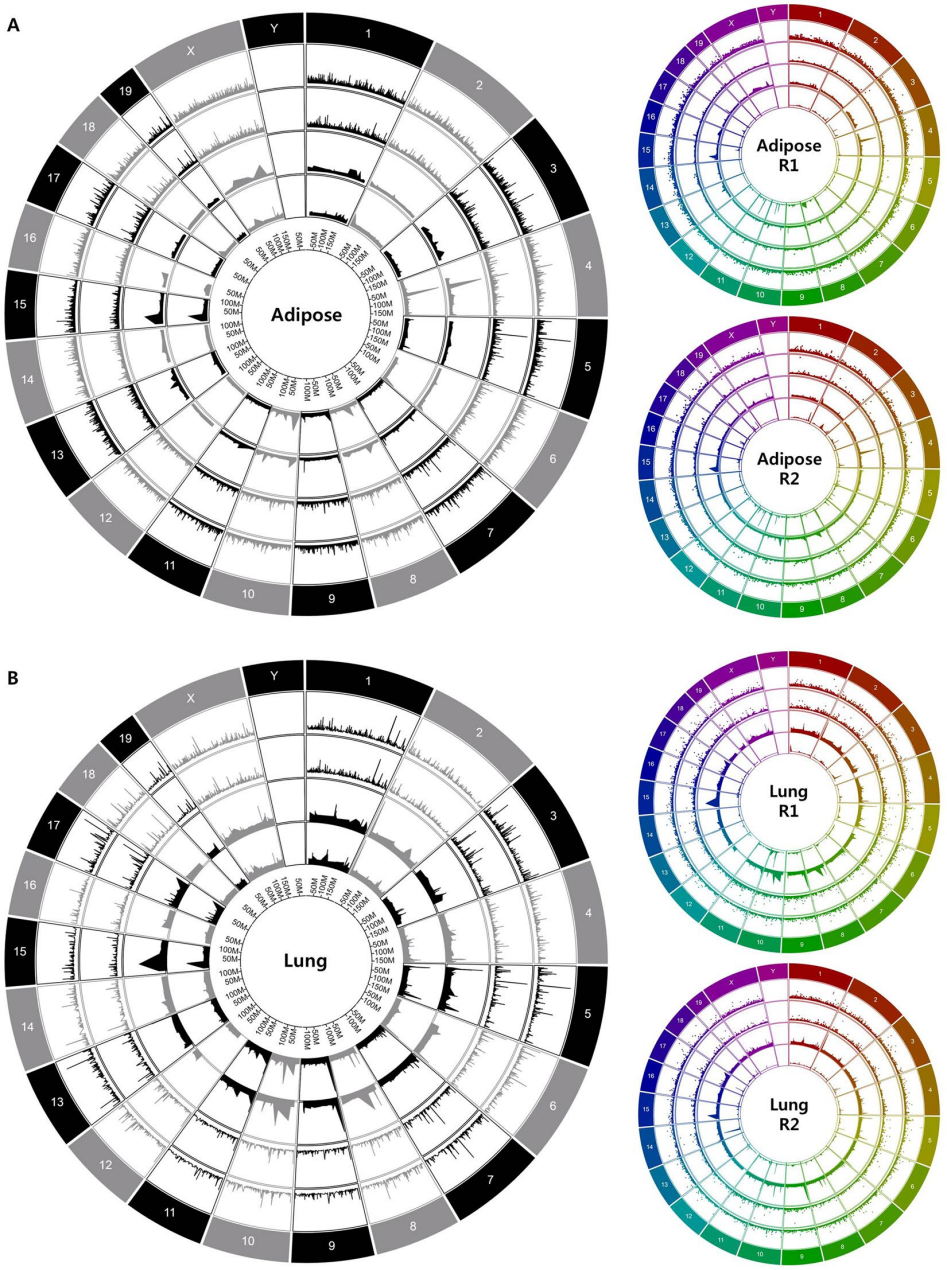
Genomic distribution of 8-oxoGs in association with gene expression in adipose and lung tissues. (**A**) Alternative gray Circos plot compares the biological replicates of adipose tissues. 1^st^ and 2^nd^ inner layers depict genomic distribution of 8-oxoGs in adipose tissue R1 and R2, respectively. 3^rd^ and 4^th^ inner layers depict the 8-oxoG distribution in gene promoters of adipose tissue R1 and R2, respectively. Dark rainbow Circos plots indicate individual adipose tissue with 1^st^ inner layer for genomic distribution of whole 8-oxoGs, 2^nd^ inner layer for global gene expression, 3^rd^ inner layer for promoter distribution of 8-oxoGs, and 4^th^ inner layer for gene expression harboring 8-oxoGs in promoter. (**B**) Genomic distribution of 8-oxoGs and accordant gene expression in lung tissues are depicted as in the upper panel. 8-oxoG peaks are depicted as fold enrichment compared to the input DNA and gene expression as non-log transformed FPKM values.

We performed OG-seq to determine the genomic distribution of 8-oxoGs as well as RNA sequencing to clarify the association between 8-oxoG and gene activity. More than 120 million mappable reads - of which 95.4% had a lower base call accuracy of 99% (Q20) by OG-seq - were generated for each tissue. Averaged 16,230 and 25,313 of 8-oxoG peaks with significant fold enrichment compared to the input DNA (fold enrichment ≥2, *q* < 0.01) were identified in adipose and lung tissues, respectively (Supplementary Tables S1 and S2). The chromosomal locations of the enriched 8-oxoG peaks were annotated in terms of gene symbols and genetic elements. Additionally, a median of 118 million mappable reads (94%) with a lower base call accuracy generated by RNA sequencing were annotated, and 18,975 genes were identified commonly expressed in both tissues. Their expression levels are summarized in Supplementary Table S3 as FPKM values. We integrated the enriched 8-oxoG peaks and gene expression levels in Circos plots, which revealed that 8-oxoGs as well as the active genes were evenly distributed throughout the chromosomes of each tissue (Fig. 1 and Supplementary Fig. S2). Although the total number of 8-oxoG peaks was more in lung tissues than in adipose tissues, the opposite was true for the fold enrichment of 8-oxoGs in adipose tissues (fold enrichment = 4.55 ± 3.14) than in lung tissues (fold enrichment = 2.92 ± 1.35, *p* < 0.0001), which was consistent with the global concentrations of 8-oxoGs determined by LC/MS. Alternative gray Circos plots also demonstrated that genome-wide distribution of 8-oxoG were reproducibly observed between replicates in both tissues, especially in the promoter regions. However, it appeared that the genomic loci having 8-oxoGs were not well correlated with gene activity. As shown in the dark rainbow Circos plots of individual tissues, some genomic loci showed gene expression with 8-oxoGs, whereas others showed gene expression with 8-oxoG independent manner.

**Figure 2:**
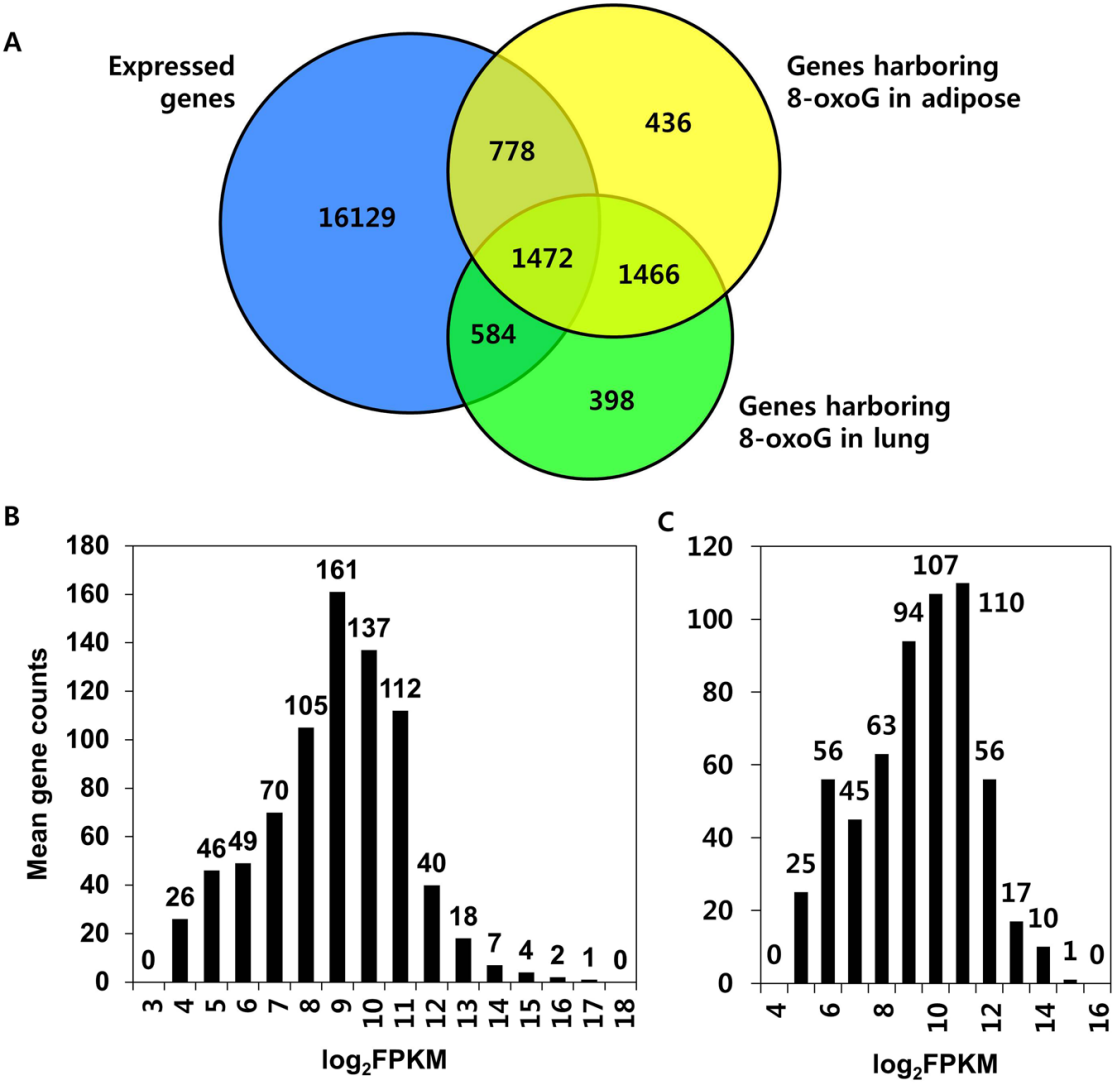
Correlation analysis between gene activity and 8-oxoG formation. (**A**) Venn diagram indicates genes commonly expressed in both of adipose and lung tissues (blue), genes with 8-oxoG formation in adipose tissues (yellow), and in lung tissues (green). (**B**) Histograms show the gene counts according to those expression levels in adipose and (**C**) lung tissues. Gene expression levels are log transformed (base = 2).

We then identified expressed genes harboring 8-oxoGs in their DNA sequence. Among the 18,963 genes commonly expressed in both tissues, 778 genes in adipose and 584 genes in lung tissue had tissue-specific 8-oxoG formation. By contrast, 1,472 genes had 8-oxoGs without tissue specificity (Fig. 2A). There were 436 and 398 genes in adipose and lung tissue, respectively, those were not expressed but had tissue-specific 8-oxoG formation. By contrast, 1,466 genes not expressed in both tissues had 8-oxoGs in common. Genes with 8-oxoGs were classified depending on their expression level as ‘high’ for highly expressed genes (FPKM > 2^9^), ‘low’ for intermediately expressed genes (FPKM ≤ 2^9^), or ‘off’ for genes without transcriptional activities. The number of genes designated as off, low, and high genes was 436, 457 and 321in adipose tissue, respectively (Fig. 2B), and 398, 283, and 301 in lung tissue, respectively (Fig. 2C).

**Figure 3:**
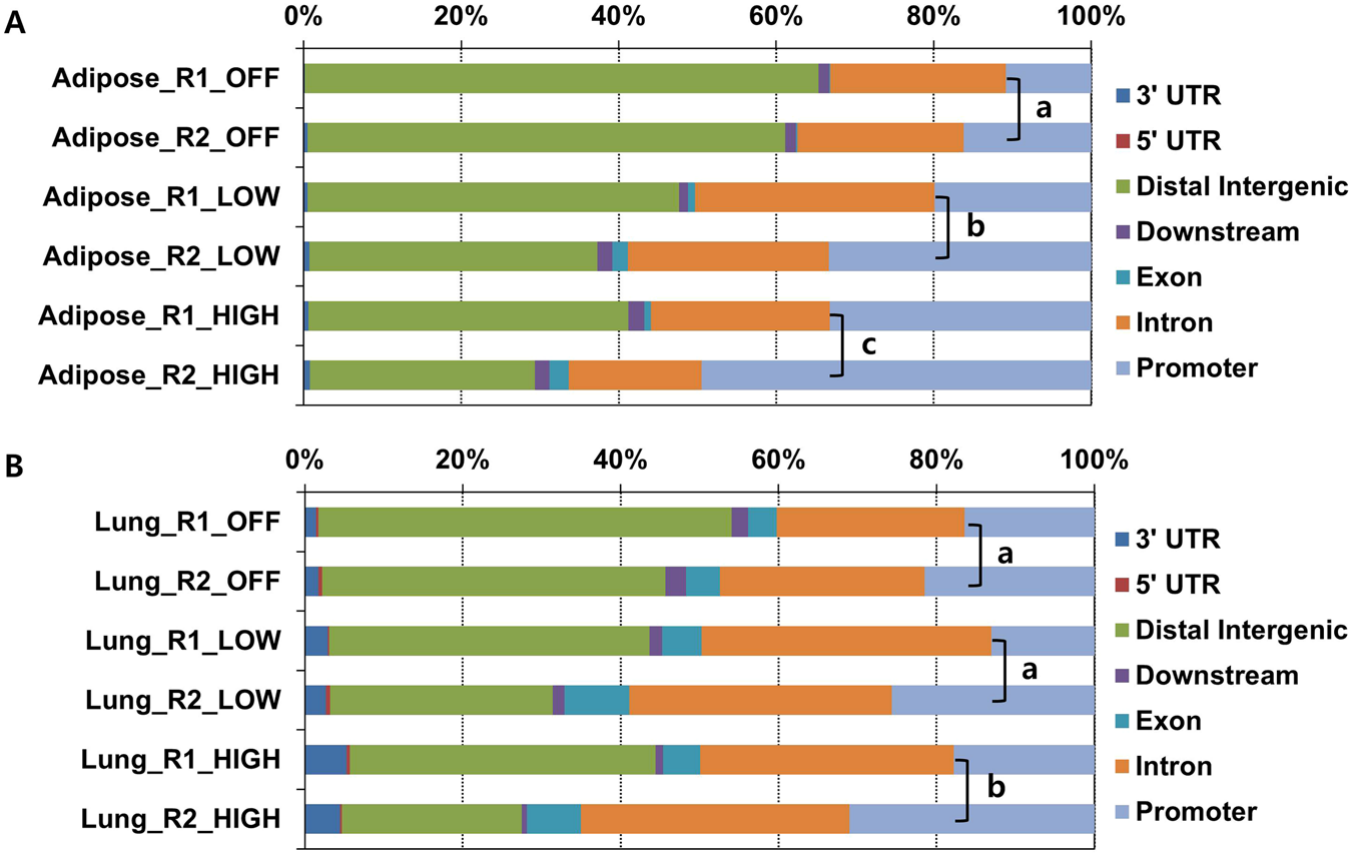
Genomic distribution of 8-oxoGs in adipose and lung tissues. (**A**) Stacked columns indicate the proportions of genomic distribution of 8-oxoGs in each genetic element in adipose tissues according to the associated gene expression levels. (**B**) Stacked columns depict the proportional distribution of 8-oxoGs in each genetic element in lung tissues. X axis denotes the relative proportions of 8-oxoGs in each genetic element; Y axis denotes the categorized genes according to expression levels. Different lowercase letters in the promoter region indicate significant differences with Fisher’s exact test (*p* < 0.01 vs. a).

The location of 8-oxoG peaks in genome was identified as 3′ untranslated region (UTR), 5′ UTR, distal intergenic, downstream, exon, intron, or promoter region with ±3 kb of transcription start site (TSS). When we compared the fraction of 8-oxoG peaks in each genetic element according to gene activity, we found that peaks were less abundant in the distal intergenic regions and more prevalent in promoter regions in association with increased gene expression levels. In adipose tissue, averaged 63.2% of 8-oxoG peaks in intergenic regions were observed from off genes, 41.8% from low genes, and 33.9% from high genes (Fig. 3A). In contrast, 13.2% of peaks were located in the promoter region of off genes, 26.7% in that of low genes, and 42.5% in that of high genes (*p* < 0.01, *R*^2^ = 0.9394). A similar distribution of 8-oxoG was observed in lung tissues (Fig. 3B). The averaged proportion of the 8-oxoG peaks in the promoter regions was 27.1% for high genes, which was significantly higher than the proportion of 8-oxoG peaks in the promoter regions of off and low genes (19% and 20.9%, respectively; *p* < 0.01, *R*^2^ = 0.3668). Read count frequency analysis revealed that the peaks were localized around TSS (Supplementary Figs S3 and S4).

**Figure 4:**
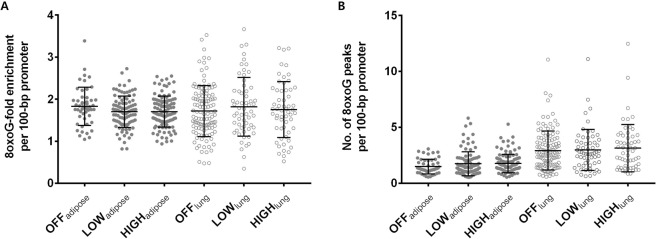
Insignificant correlation between gene expression and 8-oxoG enrichment in promoters in adipose and lung tissues. (**A**) Fold enrichment of 8-oxoG and (**B**) number of 8-oxoG peaks per 100-bp promoter length compared to input DNA were depicted. Closed circles indicate fold enrichment value from adipose tissues, and open circles from lung tissues. Genes are categorized according to expression level from off, low to high. Bar indicates mean ± SD.

The observed density of 8-oxoGs in the gene promoters implied that these modifications are related to gene activity. One possible hypothesis is that promoters have a greater degree of 8-oxoG formation to increase gene activity. Alternatively, promoters may have unique DNA sequence context that employs 8-oxoGs to regulate gene activity in an epigenetic fashion. To test those hypotheses, we normalized the fold enrichment and the frequency of 8-oxoG peaks according to the promoter length of each gene in adipose and lung tissues, and then evaluated the correlation with gene expression. In adipose tissues, the 8-oxoG peaks were enriched 1.8-fold in off genes, 1.7-fold in low genes, and 1.7-fold in high genes per 100-bp promoter length without a significant difference among gene categories (Fig. 4A). Likewise, 1.497, 1.747, and 1.766 of 8-oxoG peaks per 100-bp promoter length were observed in off, low, and high genes, respectively, and there were no differences in the frequency of the 8-oxoG peaks per unit promoter length (Fig. 4B). Similar fold enrichment and peak frequency of 8-oxoG were observed in lung tissues, and they were not significantly different according to the gene expression levels. These results imply that 8-oxoG-associated gene regulation might not be related to 8-oxoG abundance in the promoter regions.

### Transcriptional binding motif in gene promoters harboring 8-oxoG formation

**Figure 5:**
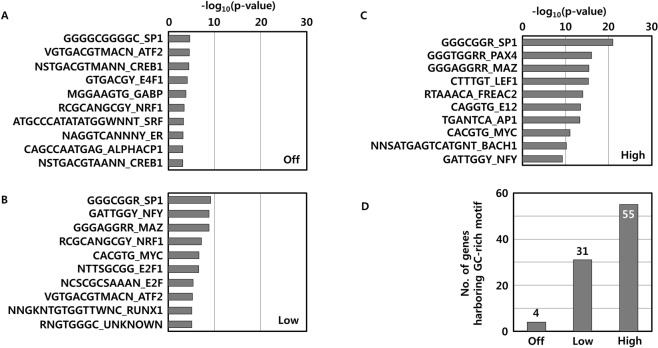
Transcription binding motif of gene promoters harboring 8-oxoGs in adipose tissue. (**A**) Enrichment analysis of transcription factor binding motifs of off genes, (**B**) low genes, and (**C**) high genes are depicted. Enrichment *P* values are determined after log transformation. (**D**) Bars indicate the number of genes with GC-rich transcription factor binding sites such including Sp1, Pax4, and Maz according to gene expression level.

Next, we investigated the feature of the DNA sequence of the promoter regions harboring 8-oxoGs to clarify the significance of 8-oxoG-associated gene regulation. To examine DNA context-dependent gene regulation by 8-oxoG in greater detail, we performed an enrichment analysis of transcription factor binding motifs of the genes. This analysis demonstrated that genes with GC-rich transcription factor binding sites in their promoters became more active, which were correlated with 8-oxoG abundance, especially in adipose tissues. While only four of off genes were enriched in the GC-rich Sp1 binding motif (Fig. 5A; *p* = 2.44E-05), 31 of low genes (Fig. 5B; *p* ≤ 1.44E-09) and 55 of high genes (Fig. 5C; *p* ≤ 3.92E-16) in adipose tissues were enriched in GC-rich Sp1, Paired box 4 (Pax4), or Myc-associated zinc finger protein (Maz) binding sites (Fig. 5D, Supplementary Tables S4–S6). In contrast, there was few enrichment of GC-rich transcription factor binding motifs according to gene activity in the promoter regions in genomic DNA from lung tissue (Supplementary Fig. S5). Therefore, it appears that the regulation of tissue-specific gene activity in mice, especially in adipose tissues, would be closely related to the occurrence of 8-oxoG formation.

We also found that off genes with 8-oxoGs in adipose tissues were functionally enriched in apoptotic process (*p* = 4.87E-06) and cell death (*p* = 5.76E-06), whereas low or high genes with 8-oxoGs were enriched in regulation of gene expression (*p* = 8.36E-13), cell cycle (*p* = 1.01E-08), sequence-specific DNA binding (*p* = 5.79E-09), and catabolic process (*p* = 3.36E-08) (Supplementary Fig. S6). Furthermore, KEGG analysis showed that high genes were specifically enriched in glucagon, insulin, phosphatidylinositol signaling pathway (*q* ≤ 0.04), and regulation of lipolysis in adipocytes (*q* = 0.0472) with adipose tissue-specific genes including Patatin-like phospholipase domain-containing 2 (*Pnpla2*) and Fatty acid binding protein 4 (*Fabp4*) (Supplementary Fig. S7). On the contrary, gene ontologies of off and low genes with 8-oxoGs in lung tissues were related with epigenetic functions including chromatin silencing (*p* = 5.70E-06) of off genes, chromatin assembly or dissembly (*p* = 6.50E-12) of low genes. Genes with high expression level were functionally enriched in regulation of intracellular signal transduction (*p* = 1.06E-06) (Supplementary Table S7). These results demonstrate that genes required for the survival and metabolic function of adipose tissue are active whereas those involved in cell death are silenced in juvenile mice.

**Figure 6:**
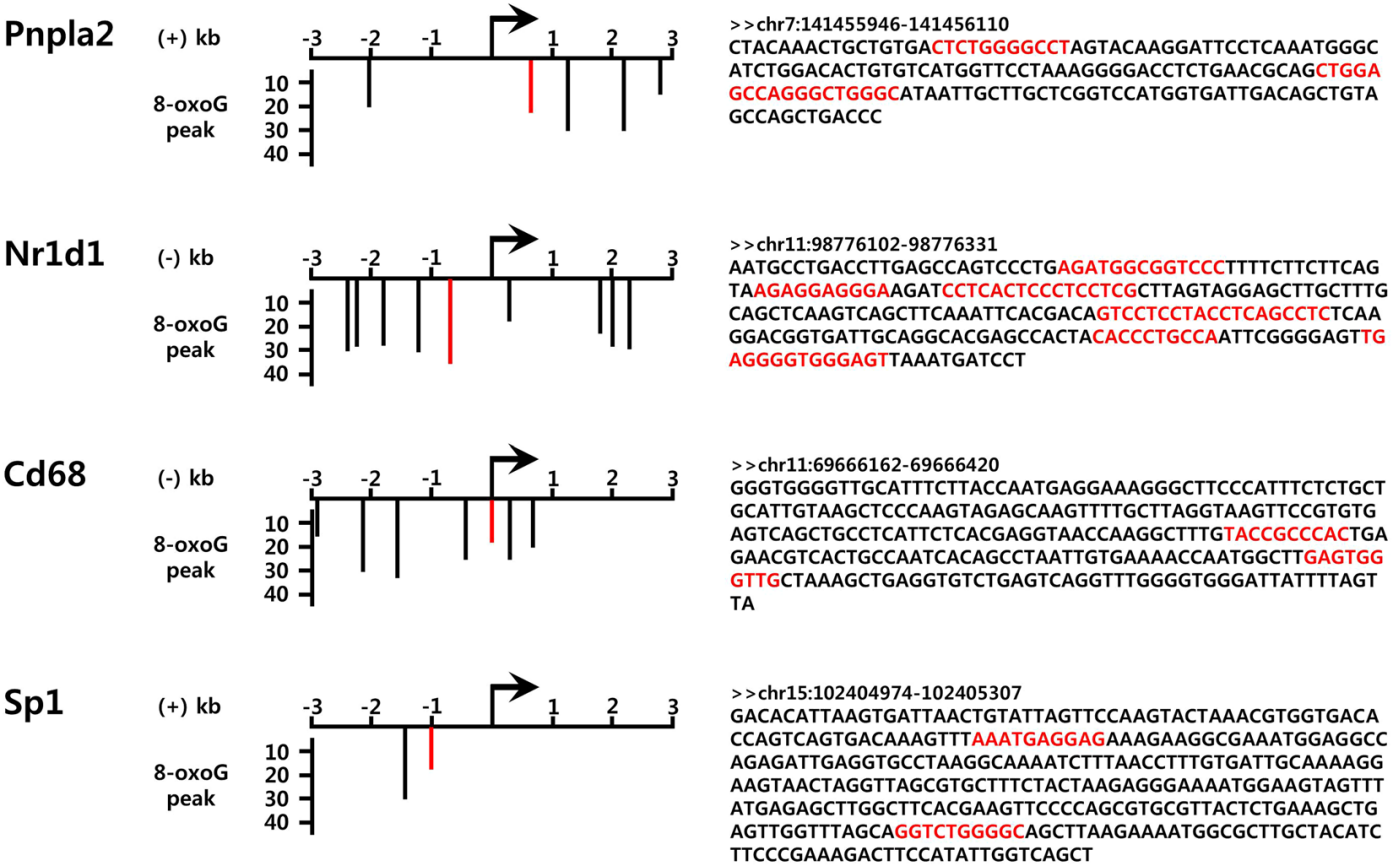
Genomic browser views of 8-oxoG peaks in promoters of genes highly expressed in adipose tissue. Representative adipose tissue-specific genes with 8-oxoGs around the TSS are depicted. Gene direction is indicated by the (+) or (−) strand. Red bars represent 8-oxoG peaks and matched DNA sequence in the right panel. Arrows indicate TSS. Sp1 binding sequences are highlighted in red.

We carried out a literature mining to clarify the biological relevance of genes harboring GC-rich transcription factor binding motifs with 8-oxoGs in adipose tissues. Among the 121 high genes, 47 genes (38.8%) were related with adipose tissue physiology, and 31 genes of them (66.0%) were predicted to have GC-rich transcription factor binding motifs in the promoter (Supplementary Table S8). For example, *Pnpla2* is an adipose triglyceride lipase that regulates lipid metabolism in adipose tissue^17–19^. A genome browsing revealed that there were five 8-oxoG peaks within the 3 kb up- or downstream of TSS of *Pnpla2* gene in adipose tissues, all of which contained several GC-rich Sp1 binding sites (Fig. 6). Likewise, Nuclear receptor subfamily 1 group D member 1 (*Nr1d1*)^20,21^, Cluster of differentiation 68 (*Cd68*)^22,23^ and Sp1^24,25^, which encode regulators of adipocyte differentiation and metabolic function, had several 8-oxoG peaks with GC-rich Sp1 binding sites around the TSS. In contrast to the high genes, only 29 genes of 95 low genes (30.5%) and 14 genes of 50 off genes (28.0%) were related with adipose physiology. Furthermore, only two of the off genes had GC-rich transcription factor binding motifs, which was significantly lower frequency than active genes (*p* < 0.0154). These results indicate that genes essential for the development and function of adipose tissue would be upregulated by 8-oxoG formation, and adipose tissue-specific genes have GC-rich promoters being regulated by the epigenetic function of 8-oxoGs.

### GC-rich transcription factor binding motif-dependent gene activation

**Figure 7:**
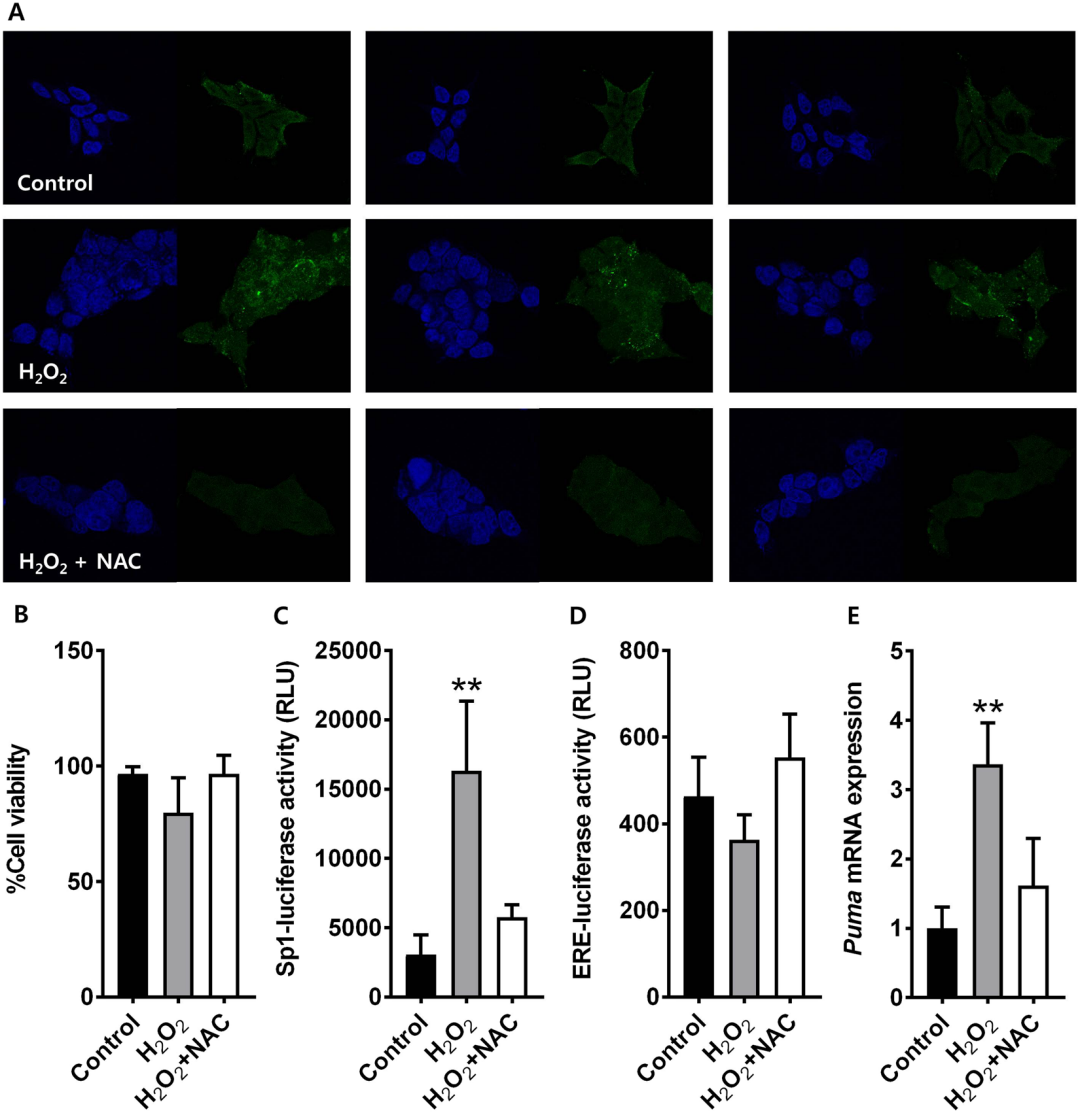
GC-rich transcription factor binding motif-dependent gene regulation in HEK293T cells. (**A**) 8-OxoG formation is regulated by extrinsic H_2_O_2_ and/or NAC treatment. Green signals indicate 8-oxoGs and blue indicates DAPI. Magnification = 200. (**B**) Cell survival is evaluated in response to H_2_O_2_ either in presence or absence of NAC. (**C**) Sp1 site-mediated transcriptional activation is evaluated in response to H_2_O_2_ either in presence or absence of NAC. (**D**) ERE site-mediated transcriptional activation is evaluated in response to H_2_O_2_ either in presence or absence of NAC. (**E**) Oxidative stress and Sp1-dependent expression of *PUMA* genes is evaluated in response to H_2_O_2_ either in presence or absence of NAC. Mean differences between groups are evaluated by analysis of variance followed by Dunnett’s multiple comparison test. **p < 0.01 vs. control.

To verify whether the presence of 8-oxoG formation is associated with GC-rich transcription factor binding motif-dependent gene activation, we carried out luciferase assays using HEK293T cells. Sp1 luciferase vector with high GC contents in the promoter region or ERE-luciferase vector with low GC contents was transfected into the cells. First we tested whether treatment of the H_2_O_2_ to HEK293T cell could induce 8-oxoG formation and it could be inhibited by pre-treatment of N-acetylcysteine (NAC). Immunofluorescent observation revealed that 8-oxoGs was induced by 300 µM H_2_O_2_ treatment and it was inhibited by co-treatment of 500 µM NAC (Fig. 7A). There were no significant cell death upon the concentration of H_2_O_2_ or NAC used either standalone or combinational treatment (Fig. 7B). When cells were subjected to oxidative stress by treatment with 300 µM H_2_O_2_, luciferase activity was significantly increased by 5.4-fold in Sp1-transfected cells compared to the control group (*p* < 0.01). This effect was abrogated by co-administration of 500 µM NAC (Fig. 7C). In contrast, oxidative stress-induced gene activation was not observed in ERE-transfected cells (Fig. 7D). p53 upregulated modulator of apoptosis (*PUMA*) is known to be up-regulated upon oxidative stress via Sp1 dependent manner^26^. We tested the correlation between 8-oxoG formation and *PUMA* gene expression. In accordance with 8-oxoG formation and reporter assay, mRNA level of *PUMA* gene was increased by 3.3-fold upon oxidative stress by treatment with 300 µM H_2_O_2_, which was inhibited by co-treatment of 500 µM NAC (Fig. 7E). Taken together, gene activation in response to 8-oxoG formation appears to be dependent on the DNA context of the transcription factor binding site, and our result shows strong correlation between 8-oxoG formation and the specific gene activation with high-GC contents on their promoter regions such as Sp1 binding sites.

## Discussion

ROS formed as a result of oxidative stress are electron-deficient and readily oxidize proteins, lipids, RNA, and especially DNA^27^. Of the four DNA bases, heterocyclic G is the most susceptible to oxidation and 8-oxoG is considered as the major oxidatively modified product^28^. The long-standing view is that 8-oxoG is mutagenic and detrimental to cellular processes. However, there is increasing evidence that in addition to being a pre-mutagenic DNA lesion, 8-oxoG also plays an essential role in the regulation of gene expression along with the DNA repair protein OGG1^29^.

Perillo and colleagues reported for the first time that 8-oxoG formation triggered by H3K9me2 demethylation facilitates OGG1 recruitment to promote the estrogen-induced gene expression^30^. 8-OxoG formation in promoters followed by OGG1 recruitment may induce NF-κB-driven pro-inflammatory gene expression, too^11^. Furthermore, it was demonstrated that sirtuin 1 (*SIRT1*) gene expression is regulated by APEX1 through oxidation of G to 8-oxoG in negative calcium responsive elements in the *SIRT1* promoter in HeLa cells under oxidative stress condition^31^. Taken together, these findings imply that both the abasic site and BER enzyme are key factors for gene activation in association with 8-oxoG formation in gene promoters. It was also reported that 8-oxoG formation in a putative G-quadruplex-forming sequence in the coding strand of the vascular endothelial growth factor (*VEGF*) promoter increased gene transcription^32^. On the other hand, 8-oxoG in template strands was shown to block the advancement of RNA polymerase II and recruit the transcription-coupled repair machinery, thereby slowing transcription^33,34^. 8-OxoG was also found to inhibit transcription when located in either the coding or template strand in a gene-coding region^35,36^.

Despite the controversy surrounding whether 8-oxoG positively or negatively regulates gene activity, the above studies suggest that 8-oxoG is an important epigenetic regulator of gene expression. However, most studies have focused on single gene under specific conditions, and it remains unclear whether 8-oxoG lesions are formed randomly in the genome or in a sequence-specific manner. Thus, a major challenge for studying the role of 8-oxoG in gene regulation is the development and implementation of genome-wide 8-oxoG sequencing. In this study, we investigated the association between the intrinsic oxidative stress-induced 8-oxoG formation and gene expression. The genome-wide analysis of 8-oxoG was performed with both adipose and lung tissues, which are representative tissues as insensitive and sensitive to oxygen toxicity, respectively. We also used juvenile mice to minimize the aging effect on 8-oxoG formation.

Many laboratories have developed antibody-based 8-oxoG sequencing methods that provide a low-resolution sequence map of 8-oxoG distribution (approximately 10–1000 kb)^13,14^. However, these do not supply information on the sequence-specific distribution of 8-oxoG and its epigenetic functions. In this study, we examined the genome-wide 8-oxoG distribution by applying OG-Seq at ~150-bp resolution using a chemical method to label 8-oxoG with biotin for affinity purification and sample enrichment^37^. Through this advanced approach, we found a correlation between 8-oxoG distribution and gene expression. Transcriptional activity and the number of active genes were significantly correlated with the distribution of 8-oxoG, especially in the gene promoter regions. However, the 8-oxoG peaks and number of 8-oxoG peaks per 100-bp promoter length did not differ among genes categorized as inactive or moderately or high active. Instead, genes with GC-rich transcription factor binding sites in their promoters became more active with increasing 8-oxoG abundance, implying that 8-oxoG-related gene regulation is not associated with the degree of 8-oxoG formation but with DNA context within the promoter. The importance of 8-oxoG formation for GC-rich transcriptional binding site-dependent gene activation was confirmed by Sp1-luciferase assay and *PUMA* gene expression analysis in HEK293T cells. Thus, oxidative stress-mediated gene activation appears to be dependent on the DNA motif of the transcription factor binding site, with GC-rich Sp1 binding sites being a key element in the modulation of gene activity under oxidative stress. However, the mechanism how the formation of 8-OxoG in GC-rich promoter would facilitate the transcription is yet to be discovered. One possible way is that the oxidation of DNA itself may physically facilitate the binding of transcriptional factors to the gene regulatory regions. Secondly, oxidized DNA may be bound by series of repair proteins such as OGG1, then, possibly recruit additional proteins to the locus, such as histone modifying enzymes or other chromatin remodelling proteins, thereby forming active chromatin.

Guanine oxidation does not occur randomly in the genome, but instead shows a strong distributional bias^38^. Furthermore, 8-oxoG substitution in synthetic DNA oligomer affected the binding affinity of Sp1 depending on guanine position^39^. However, the mechanisms by which 8-oxoG formation at GC-rich transcription factor binding sites and regulation of gene expression remain unclear. Guanine oxidation in a GC-rich promoter is suggested to have a *cis* effect in response to an oxidative burst, while OGG1 acts as a *trans*-factor whose oxidation is considered a reversible post-translational modification^38^. Rapid binding of oxidatively inactivated OGG1 and consequent allosteric modification of DNA during BER’s pre-excision stage^40^ promotes homing of transcription factors (e.g., NF-κB and transcription signal transducer and transcription activator) or co-activators (e.g., C-terminal binding protein/p300) to their binding motif, facilitating assembly of the transcriptional machinery. After re-establishing the redox balance, OGG1 regains its enzymatic activity by reducing its oxidized cysteine and then excises the oxidized guanine to avoid mutation(s) in the promoter.

There are some potential pitfalls to consider in this study. First, number of mice used in comprehensive genomic analysis of 8-oxoG was low (n = 2 per each tissues) with limited variety of tissues (lung and adipose tissues). Although we observed the significant enrichment of 8-oxoG in the GC-rich transcription factor binding motif-containing promoter regions of adipose tissue-specific genes according to the gene activity, there was no enrichment like this in genomic DNA from lung tissues. It turns to be elusive if the conclusion drawn in this study is general for other species and tissues. Second, the effect of glycosylase Ogg1 on the formation and distribution of 8-oxoG in genomic DNA was not investigated. We observed significantly reduced level of 8-oxoG in the lung tissues compared to the adipose tissues, which was well explained by differential expression of OGG1 in human lung and adipose tissues (https://www.proteinatlas.org/ENSG00000114026-OGG1/tissue). However, the precise role of Ogg1 in 8-oxoG formation and the transcriptional regulatory activity were beyond the scope of this study, and were remained to be clarified. Third, there would be difference in oxidative stress conditions between *in vivo* and *in vitro* experiments. We applied intrinsic oxidative stress condition in animal experiment and extrinsic oxidation using H_2_O_2_ in cell experiment. Therefore, the role and the mechanism of 8-oxoG formation might be different. However, there are limitations to reproduce the intrinsic oxidative stress condition *in vitro*, and it requires some improvement of cell culture condition.

In conclusion, we demonstrated that the promoter regions of adipose tissue-specific genes are GC-rich, which could be important for the epigenetic function of the 8-oxoG. Furthermore, genes with GC-rich transcription factor binding sites in their promoters became more active with increasing 8-oxoG abundance as demonstrated by Sp1- and ERE-luciferase assays in HEK293T cells under oxidative stress condition. These results suggest that 8-oxoG promotes transcription during adipose tissue development in mice.

## Methods

### Tissue collection

C57BL/6 female mice were obtained from Koatech (Pyeongtaek, South Korea) and were individually housed in ventilated cages with free access to food and water. Mice (n = 5) were humanely sacrificed at 4 weeks old and liver, lung and intercapular fat samples were collected and stored at −80 °C until analyses. The study protocol was approved by the Institutional Animal Care and Use Committee of Seoul National University (SNU-170912-22) and was conducted in accordance with the approved guidelines.

### DNA hydrolysis and quantification of 8-oxoG

Genomic DNA was extracted from animal tissues using the DNeasy Blood and Tissue kit (Qiagen, Valencia, CA, USA). Before extraction, each of 100 µM desferal and butylated hydroxytoluene was added to the DNA extraction solution. The extracted DNA was enzymatically hydrolyzed to generate deoxynucleosides. Briefly, 20 µg of DNA were added to 40 µl hydrolysis buffer (100 mM NH_4_HCO_3_ [pH 7.6], 10 mM MgCl_2_, and 1 mM CaCl_2_) along with 1 U DNase I, 0.2 mU phosphodiesterase I, and 0.1 U alkaline phosphatase, followed by incubation at 37 °C for 6 h. The samples were vacuum-centrifuged at 55 °C until they were completely dried. All reagents including nucleoside standards were purchased from Sigma-Aldrich (St. Louis, MO, USA). The pellets were dissolved in 50 µl of 5% methanol and subjected to RP-LC/MS for 8-oxoG measurement. Samples were spiked with 10 nM of 8-[^15^N_5_]oxoG internal standard with 288.9/173 mass transition (Cambridge Isotope Laboratories, Inc., Tewksbury, MA, USA). Separate samples were subjected to high-performance liquid chromatography (HPLC) for dG and dC measurements as previously described^16^.

The RP-LC/MS system consisted of an Ultimate 3000 RS HPLC instrument (Dionex, Sunnyvale, CA, USA) coupled with a TSQ ENDURA triple-quadrupole mass spectrometer (Thermo Fisher Scientific, Waltham, MA, USA). A Kinetex reversed-phase chromatography column (2.1 × 100 mm, inner diameter = 2.6 µm, Phenomenex, Torrance, CA, USA) was used with an injection volume of 10 µl. For separation and quantification of each nucleoside, a gradient program was established with solvent A (0.1% formic acid in water) and solvent B (0.1% formic acid in methanol), starting with 5% solvent B for 0.5 min, then ramping to 90% solvent B over 6 min, holding at 90% solvent B for 1.5 min, and re-equilibrating with 5% solvent B for 5 min at a flow rate of 300 µl/min. Mass spectrometric detection was performed using positive electrospray ionization in multiple reaction monitoring mode to monitor the 284.1/168.2 mass transition of 8-oxoG. For HPLC analysis, separation was achieved with three mobile phases - i.e., solvent A, deionized water; solvent B, 50 mM (NH_4_)_2_HPO_4_ (pH 4.0 with phosphoric acid); solvent C, methanol. The gradient program for the Shiseido column (Capcell Pak C18 UG120 4.6 × 250 mm, inner diameter = 5 µm, Phenomenex) was 82.5% solvent A, 15% solvent B, and 2.5% solvent C from 0–3 min; 55% solvent A, 15% solvent B, and 30% solvent C from 3–8 min; 82.5% solvent A, 15% solvent B, and 2.5% solvent C from 8–8.5 min; and 82.5% solvent A, 15% solvent B, and 2.5% solvent C from 8.5–10 min at a flow rate of 1.0 mL/min at 40 °C. The injection volume was 10 µl, and diode array detection was set to 280 nm.

### 8-oxoG enrichment by affinity purification

DNA fragments harboring 8-oxoG were enriched as previously described^37^, with minor modifications. Briefly, 5 µg of the genomic DNA extracted from adipose or lung tissues of randomly selected mice (n = 2) were sheared with an S2 ultrasonicator (Covaris, Woburn, MA, USA) in 10 mM Tris buffer (pH 8.0) to obtain ~150-bp fragments. After sonication, the fragmented DNA was concentrated to 20 µl in 100 mM NaPi buffer (pH 8.0) using QIAquick PCR purification kit (Qiagen). A 100-µl volume of 100 mM NaPi buffer containing 20 mM amine-PEG2-biotin (Thermo Fisher Scientific) was added and the mixture was heated to 75 °C for 10 min. After thermal equilibration, 5 mM K_2_IrBr_6_, a mild one electron oxidant, was added for 1 h for 8-oxoG biotinylation through covalent adduction. The DNA fragments biotinylated at 8-oxoG were eluted with 125 µl Tris buffer using the QIAquick PCR purification kit and extracted using Dynabeads MyOne Streptavidin C1 (Thermo Fisher Scientific); strands complementary to those with bound biotinylated 8-oxoG were released by incubation in 150 mM NaOH at 20 °C for 30 min, and concentrated to 10 µl ddH_2_O using ssDNA/RNA Clean & Concentrator kit (Zymo Research, Irvine, CA, USA).

### 8-oxoG sequencing (OG-seq)

The 8-oxoG-enriched DNA fragments obtained by affinity purification were subjected to next-generation sequencing. OG-seq libraries were constructed using the TruSeq Nano DNA kit (Illumina, San Diego, CA, USA) and sequenced with the TruSeq SBS Kit v3-HS on a HiSeq. 2000 sequencer (Illumina) to obtain 101-bp paired-end reads. Image analysis and base calling were performed using the Illumina pipeline (v1.8) with default settings. The reads were aligned to the mouse reference genome (mm10) with Isaac aligner (Illumina). Duplicated reads were identified and removed with Picard (http://broadinstitute.github.io/picard/); enriched peaks were called from the mapped reads using MACS2^41^, and peaks were annotated with ChIPseeker^42^. 8-OxoG lesions differing significantly from those in input DNA (*p* < 10^−4.5^) were selected for further analyses.

### RNA sequencing

Total RNA was extracted from adipose or lung tissue of two randomly selected mice using the RNeasy Mini kit (Qiagen) with DNase I (Qiagen) treatment. RNA integrity was evaluated with a Bioanalyzer (Agilent Technologies, Santa Clara, CA, USA). RNA sequencing libraries were generated using the TruSeq RNA sample Preparation kit (Illumina) and were sequenced on the HiSeq. 2000 sequencer, which yielded ~100 million paired-end reads (2 × 101 bp). TopHat alignment coupled with Cufflinks assembly was used to generate the final transcriptome assembly and examine gene expression levels^43^. The number of reads aligned to each gene was normalized to fragments per kilobase of exon per million (FPKM).

### Functional enrichment and analysis

Genes in each tissue with promoters harboring 8-oxoG lesions were categorized as inactive for FPKM = 0 (designated as an “off” gene), moderately active for FPKM ≤ 2^9^ (designated as a “low” gene), and active for FPKM > 2^9^ (designated as a “high” gene). Biological functions and transcription factor binding motifs of the categorized genes were evaluated by Gene Set Enrichment Analysis (http://software.broadinstitute.org/gsea)^44^. The query gene set was computationally overlapped with the Molecular Signature Database using a false discovery rate *q*-value cutoff of 0.05. Transcription factor binding sites were predicted using AliBaba 2.1^45^ with the read DNA sequence obtained by 8-oxoG sequencing. The biological relevance of the genes of interest was investigated through multiplex literature mining using PubMatrix^46^. A regulatory network was constructed using the STRING protein-protein association network database (https://string-db.org)^47^. Adipose tissue-specific ‘high’ genes harboring 8-oxoGs in promoters were used with more than 0.4 of interaction confidence.

### Luciferase reporter gene assay

HEK293T cells (5 × 10^4^ cells per well) were seeded in a 12-well plate and cultured for 24 h before transfection with Sp1-luciferase reporter plasmid DNA (0.5 g; Panomics, Fremont, CA, USA) or a 3 × ERE TATA luc construct (Addgene, Cambridge, MA, USA) for 24 h. The medium was then replaced and cells were treated with 300 µM H_2_O_2_ with or without pre-treatment of 500 µM N-acetylcysteine (NAC) for 5 min, followed by incubation for 3 h. The luciferase activity of cell lysates was measured using a luciferase assay kit (Promega, Madison, WI, USA) according to the manufacturer’s instructions. Relative luciferase activity was normalized to total protein content of the lysates.

### Immunofluorescence assay

HEK293T cells (5 × 10^4^ cells per well) were seeded onto coverslips in 12-well plate 24 h prior to staining. Cells were treated with 300 µM H_2_O_2_ with or without pre-treatment of 500 µM NAC for 5 min, followed by incubation for 3 h. The cells were then washed with PBS and fixed in 4% paraformaldehyde (Electron Microscopy Sciences, Hatfield, PA, USA) for 15 min at room temperature. The cells were washed twice with PBS and permeabilized with 0.2% Triton X-100 in PBS for 10 min. The cells were incubated with an antibody against 8-oxoG (Merck KGaA, Darmstadt, Germany) diluted 1:200 in 0.05% Tween 20 in PBS for overnight at 4 °C. Nuclei were stained with DAPI (Life technologies, Carlsbad, CA, USA) for 1 hr at room temperature and analyzed using an LSM 700 (Zeiss, Oberkochen, Germany).

### Cytotoxicity assay

HEK293T cells (5 × 10^4^ cells per well) were seeded 6-well plate 24 h prior to cytotoxicity assay. Cells were treated with 300 µM H_2_O_2_ with or without pre-treatment of 500 µM NAC for 5 min, followed by incubation for 3 h. The cells were washed with PBS and collected by incubation with trypsin and the viable cells were counted using a hemocytometer after trypan blue staining (Thermo Fisher Scientific).

### Quantitative real-time PCR (qPCR)

Total RNA was extracted using the easy-BLUE total RNA extraction kit (iNtRON Biotechnology, Seoul, Korea). Then, 1 μg of total RNA was reverse transcribed into cDNA using the ImProm-II reverse transcription system (Promega). qPCR was performed using KAPA SYBR® FAST qPCR kit (KAPABIOSYSTEMS, Wilmington, MA, USA) with the CFX96 Touch real-time PCR detection system (Bio-Rad, Hercules, CA, USA). The relative mRNA levels were normalized to *GAPDH* mRNA levels, and primers used in the qPCR were followed: GAPDH (forward) 5′- TTG ATT TTG GAG GGA TCT CG-3′ and (reverse) 5′- GAG TCA ACG GAT TTG GTC GT-3′, *PUMA* (forward) 5′- GAC CTC AAC GCA CAG TA-3′ and (reverse) 5′- CTA ATT GGG CTC CAT CT-3′.

## Supplementary information

Supplementary Tables

Supplementary Figures

## Data Availability

RNA-seq and OG-seq raw and processed data from this study have been submitted to the NCBI Gene Expression Omnibus (GEO; http://www.ncbi.nlm.nih.gov/geo/) under accession number GSE124359 and GSE124712.
