# Supplementary material for: 8-OxoG in GC-rich Sp1 binding sites enhances gene transcription in adipose tissue of juvenile mice
Source: Sci Rep. 2019 Oct 30;9:15618. doi: 10.1038/s41598-019-52139-z (PMC6821754; doi:10.1038/s41598-019-52139-z)

8-OxoG in GC-rich Sp1 binding sites enhances gene transcription in adipose tissue of juvenile mice

Jong Woo Park[1,#,] Young In Han[2,#], Sung Woo Kim[3], Tae Min Kim[2,4], Su Cheong Yeom[2,4], Jaeku Kang[5,*] and Joonghoon Park[2,4,*]

[1]Research Center for Epigenome Regulation, School of Pharmacy, Sungkyunkwan University, Suwon, 16419, Republic of Korea

[2]Institute of Green Bio Science and Technology, Seoul National University, Pyeongchang, 25354, Republic of Korea

[3]Animal Genetic Resources Research Center, National Institute of Animal Science, Rural Development Administration (RDA), Namwon, 55717, Republic of Korea

[4]Department of International Agricultural Technology, Graduate School of International Agricultural Technology, Seoul National University, Pyeongchang, 25354, Republic of Korea

[5]Department of Pharmacology, College of Medicine, Konyang University, Daejeon, 35365, Republic of Korea

*Correspondence: jaeku@konyang.ac.kr (J.K.), joonghoon@snu.ac.kr (J.P.)

[#]The authors contributed equally to this work.

Supplementary Figures

Supplementary Figure S1. Representative chromatograms and standard curves with various concentrations of 8-oxoG by LC/MS. The retention time of 8-oxoG is 2.9 min, and the correlation coefficient (R^2) is 0.999.

Supplementary Figure S2. Genome-wide coverage plot of 8-oxoGs in adipose and lung tissues. Significant pileups of 8-oxoG are plotted in individual chromosome ($q < 0.01$ vs. input DNA).

Supplementary Figure S3. Heat map of read count frequency of 8-oxoG peaks around gene transcription start site (TSS).

Supplementary Figure S4. Averaged profile of read count frequency of 8-oxoG peaks around gene transcription start site (TSS). Genomic region is denoted from 5’ to 3’ in X-axis, read count frequency in Y-axis.

Supplementary Figure S5. Enrichment analysis of transcription factor binding motifs of lung tissue-specific genes harboring 8-oxoGs in promoters. High (red bars), low (blue bars), or off (grey bars) denote genes with 8-oxoGs classified depending on their expression level. *P*-value was (-)log transformed ($-\log_{10}$).

Supplementary Figure S6. Functional enrichment analyses of adipose tissue-specific genes harboring 8-oxoGs in promoters. High, low, or off indicates genes with 8-oxoGs classified depending on expression levels. *p*-value was (-)log transformed with base = 10.

Supplementary Figure S7. KEGG analysis of adipose tissue-specific ‘high’ genes harboring 8-oxoGs in promoters. Genes enriched in glucagon, insulin, phosphatidylinositol signaling pathway, and regulation of lipolysis in adipocytes were used to construct the regulatory

network with interaction confidence > 0.4. Nodes indicate the enriched genes and different thickness of edge indicates different level of interaction confidence. Red nodes indicate genes enriched in regulation of lipolysis in adipocytes (q = 0.0472).

Supplementary Tables

Supplementary Table S1. 8-oxoG sequencing of the genomic DNA of adipose tissues

Supplementary Table S2. 8-oxoG sequencing of the genomic DNA of lung tissues

Supplementary Table S3. RNA sequencing of adipose and lung tissues

Supplementary Table S4. Transcription binding motif enrichment of off genes in adipose tissues

Supplementary Table S5. Transcription binding motif enrichment of low genes in adipose tissues

Supplementary Table S6. Transcription binding motif enrichment of high genes in adipose tissues

Supplementary Table S7. Functional enrichment analyses of lung tissue-specific genes harboring 8-oxoGs in promoters

Supplementary Table S8. Literature mining of categorized genes in adipose tissues

Supplementary Figure S1

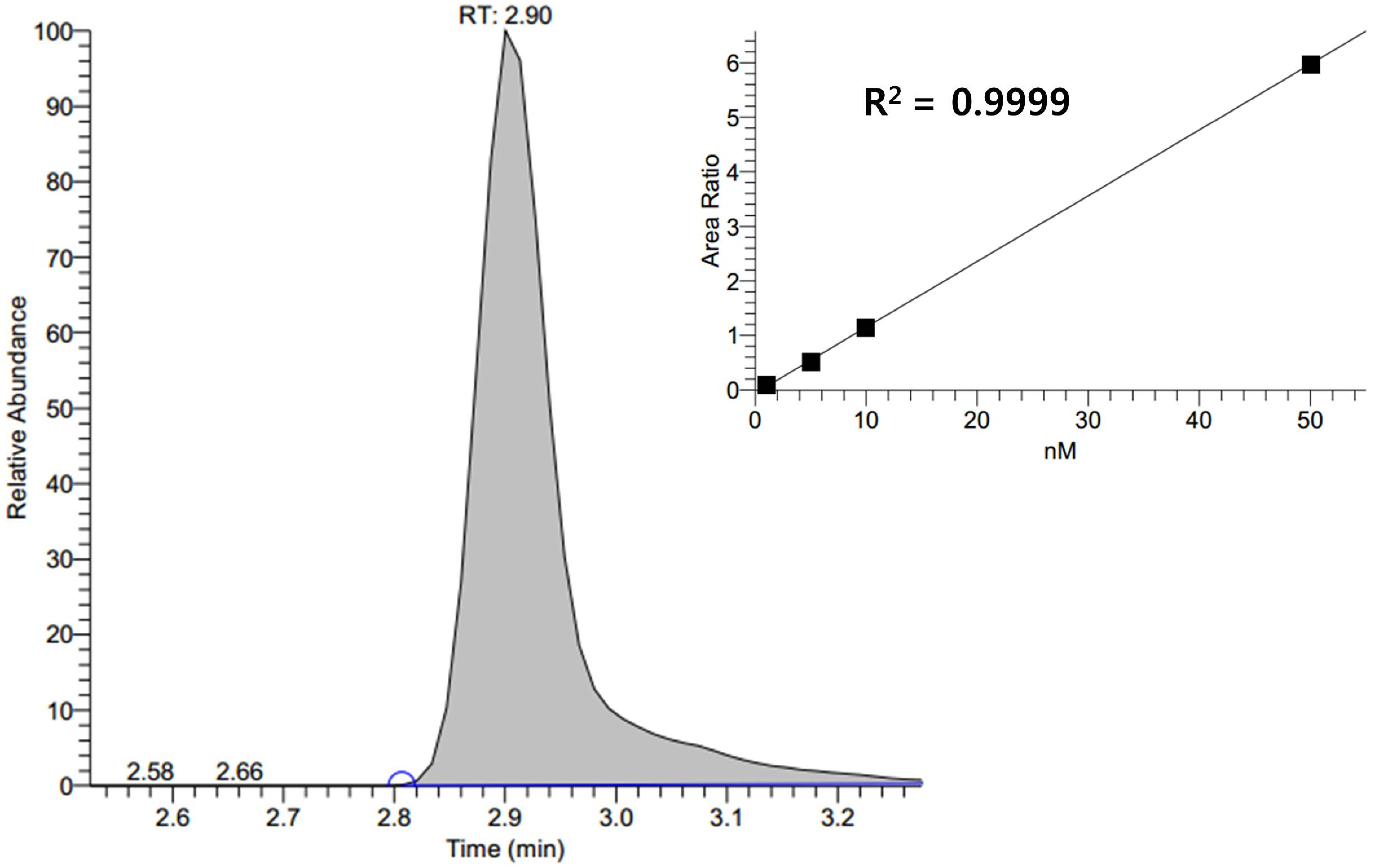

Supplementary Figure S2

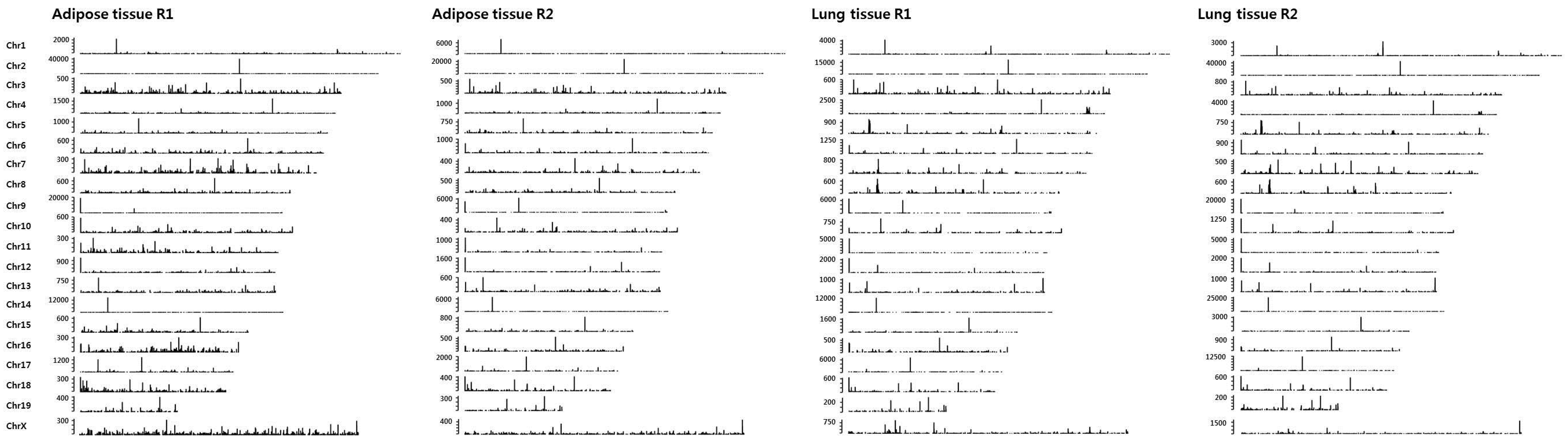

Supplementary Figure S3

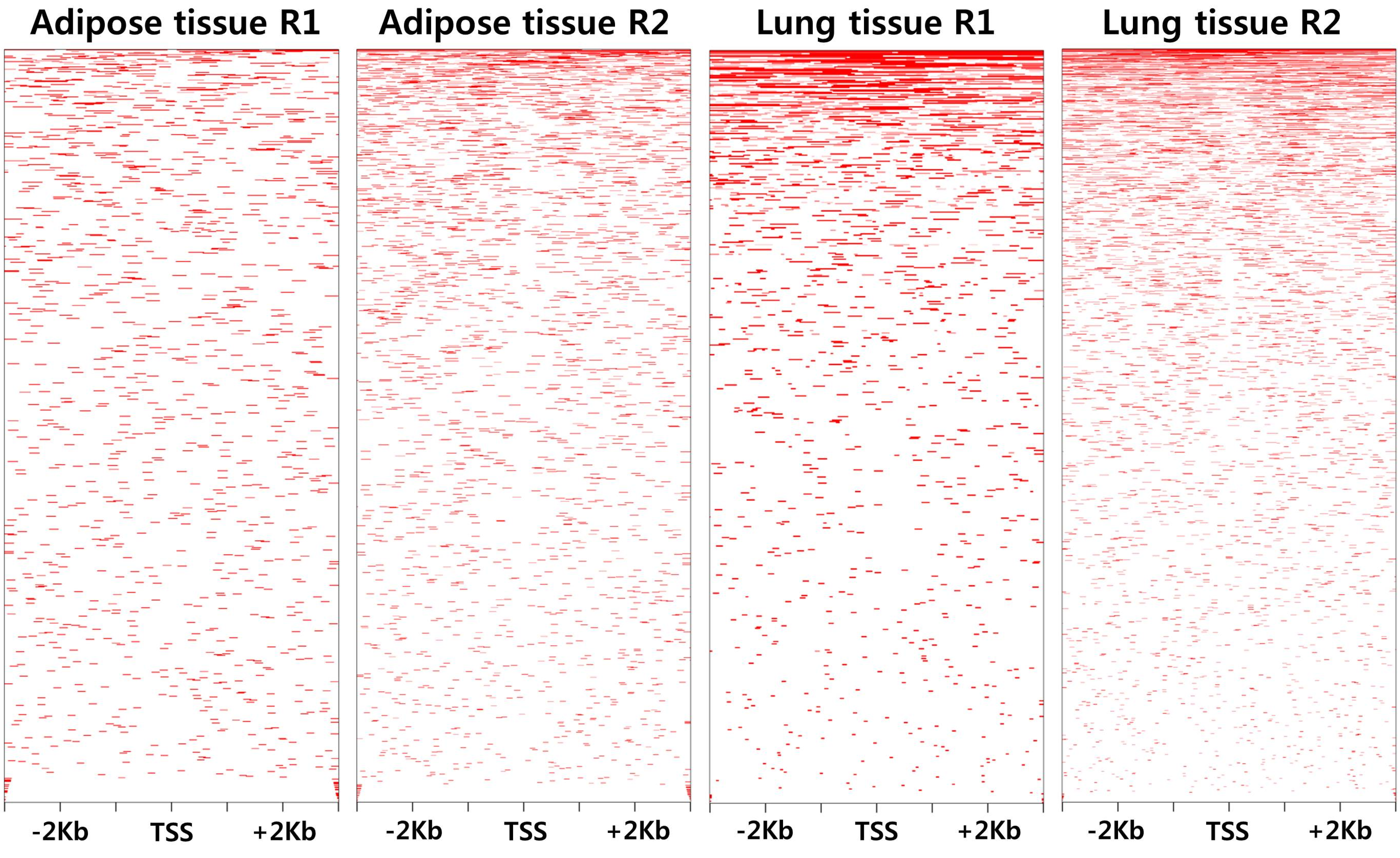

Supplementary Figure S4

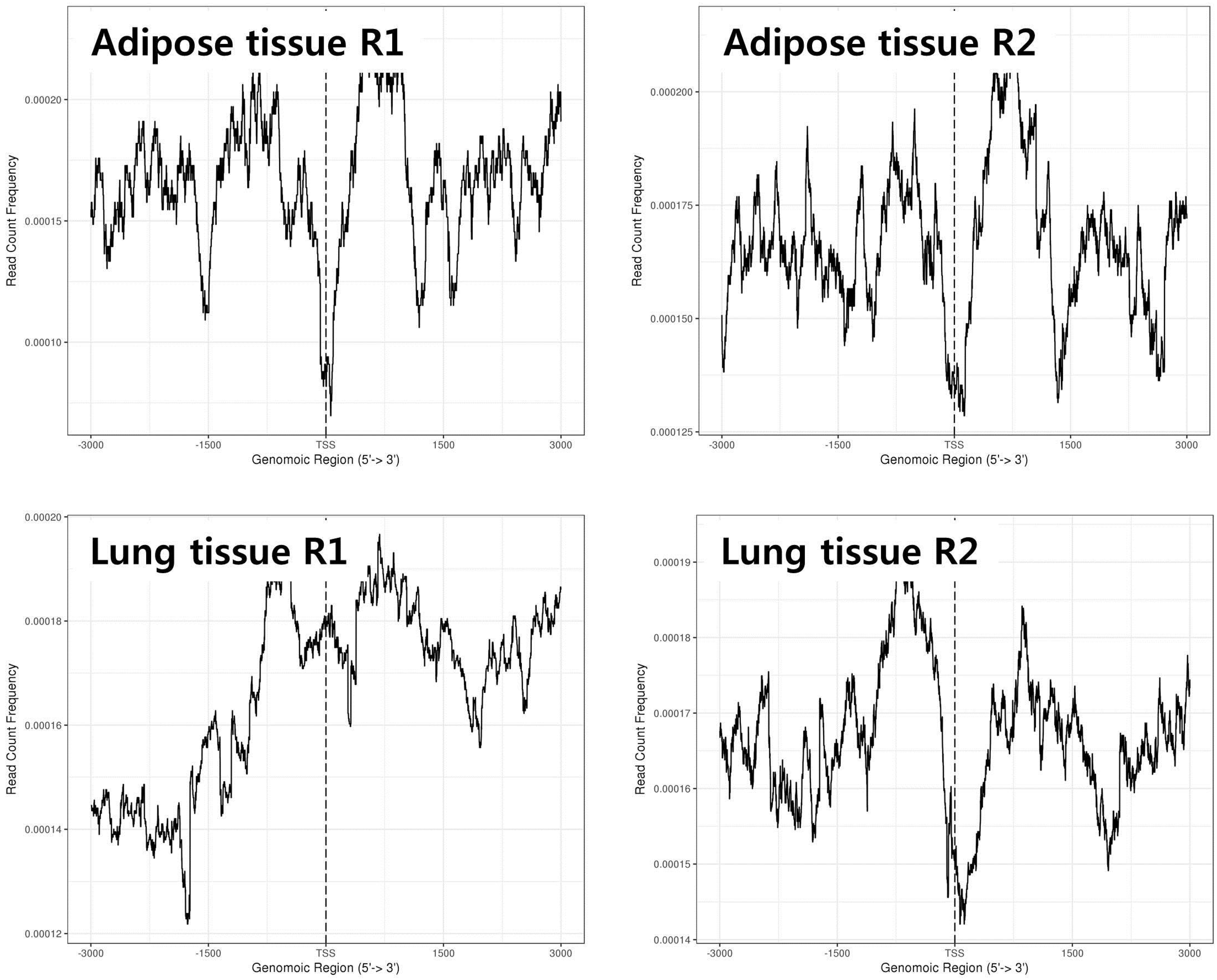

Supplementary Figure S5

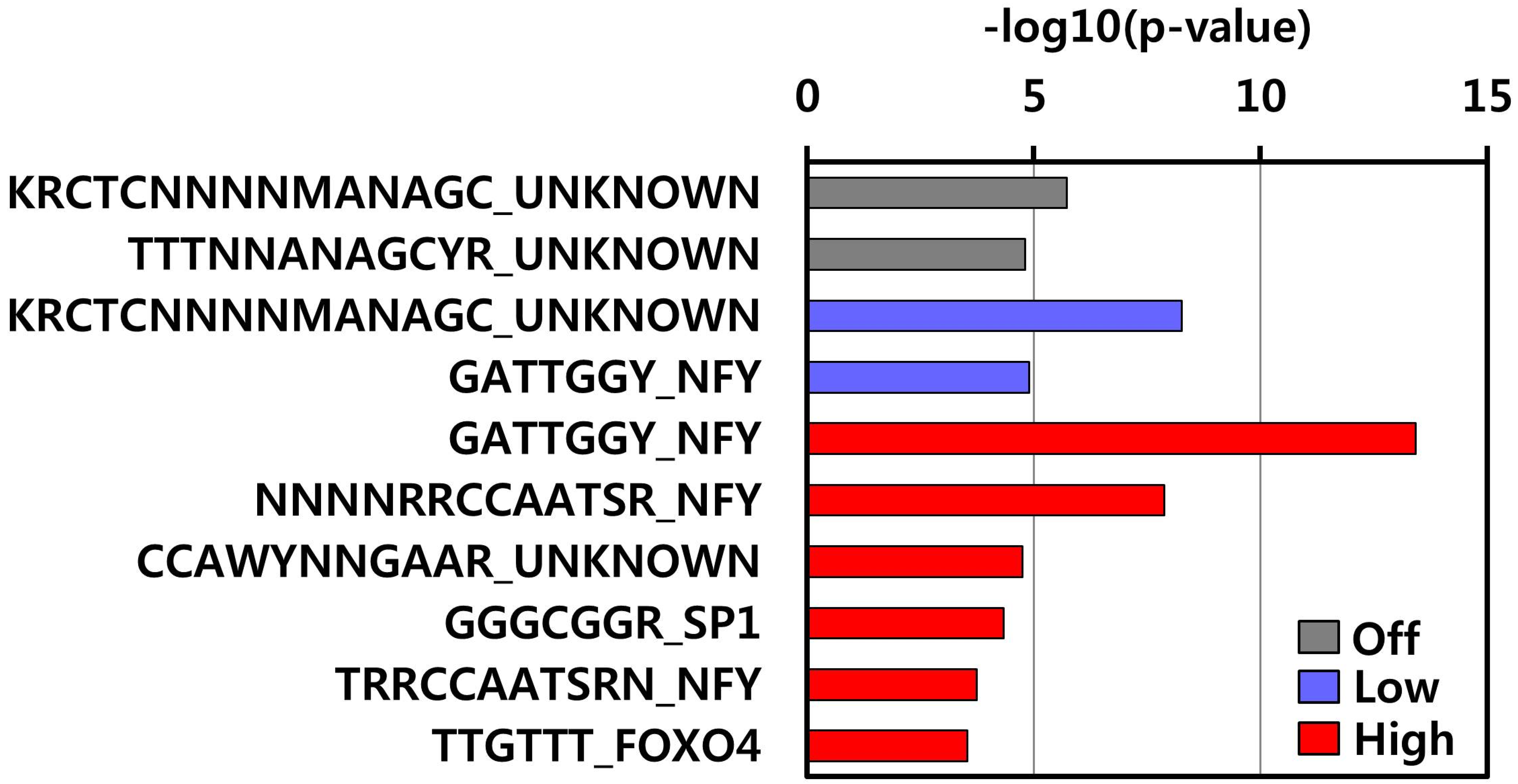

Supplementary Figure S6

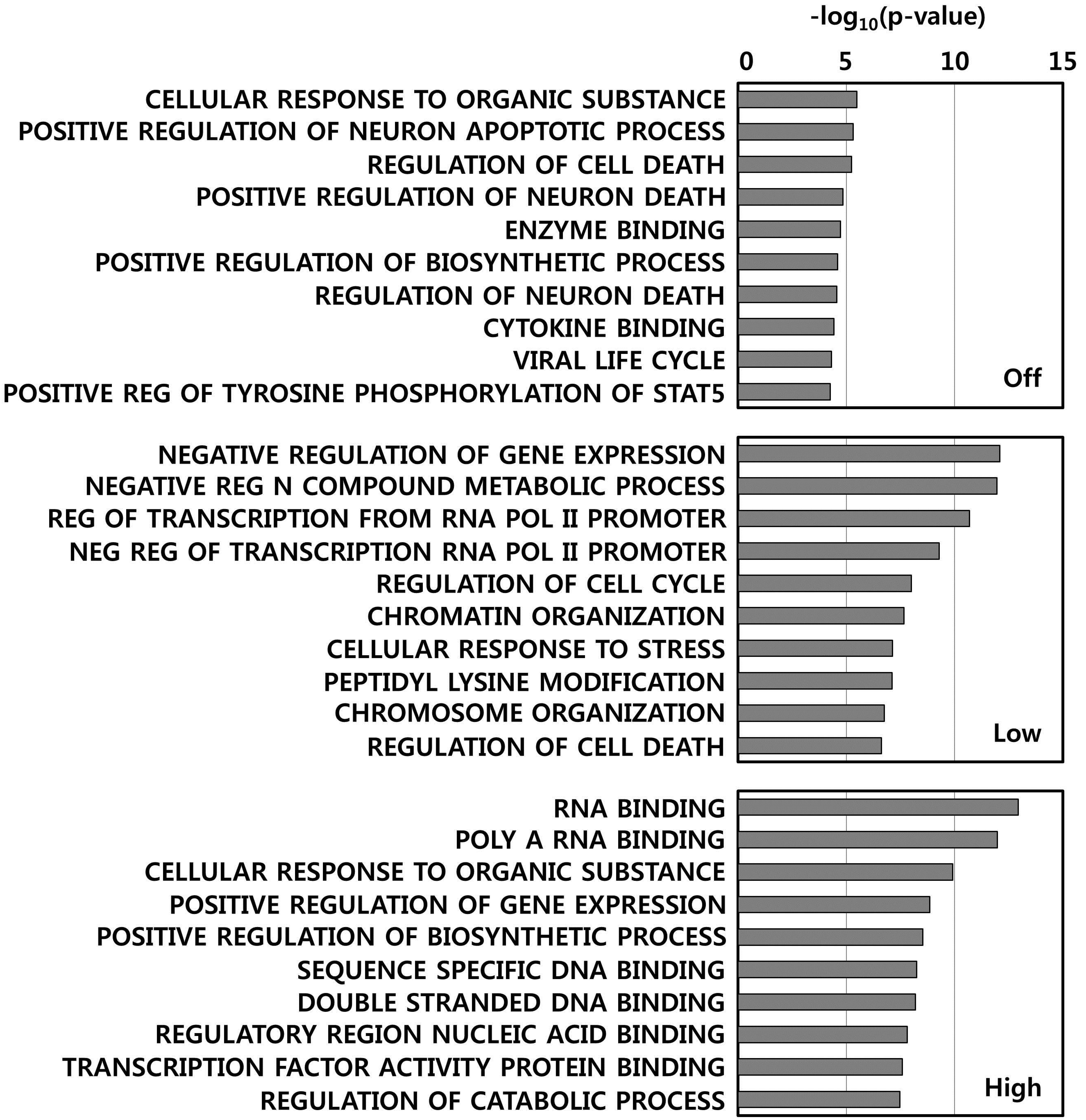

Supplementary Figure S7

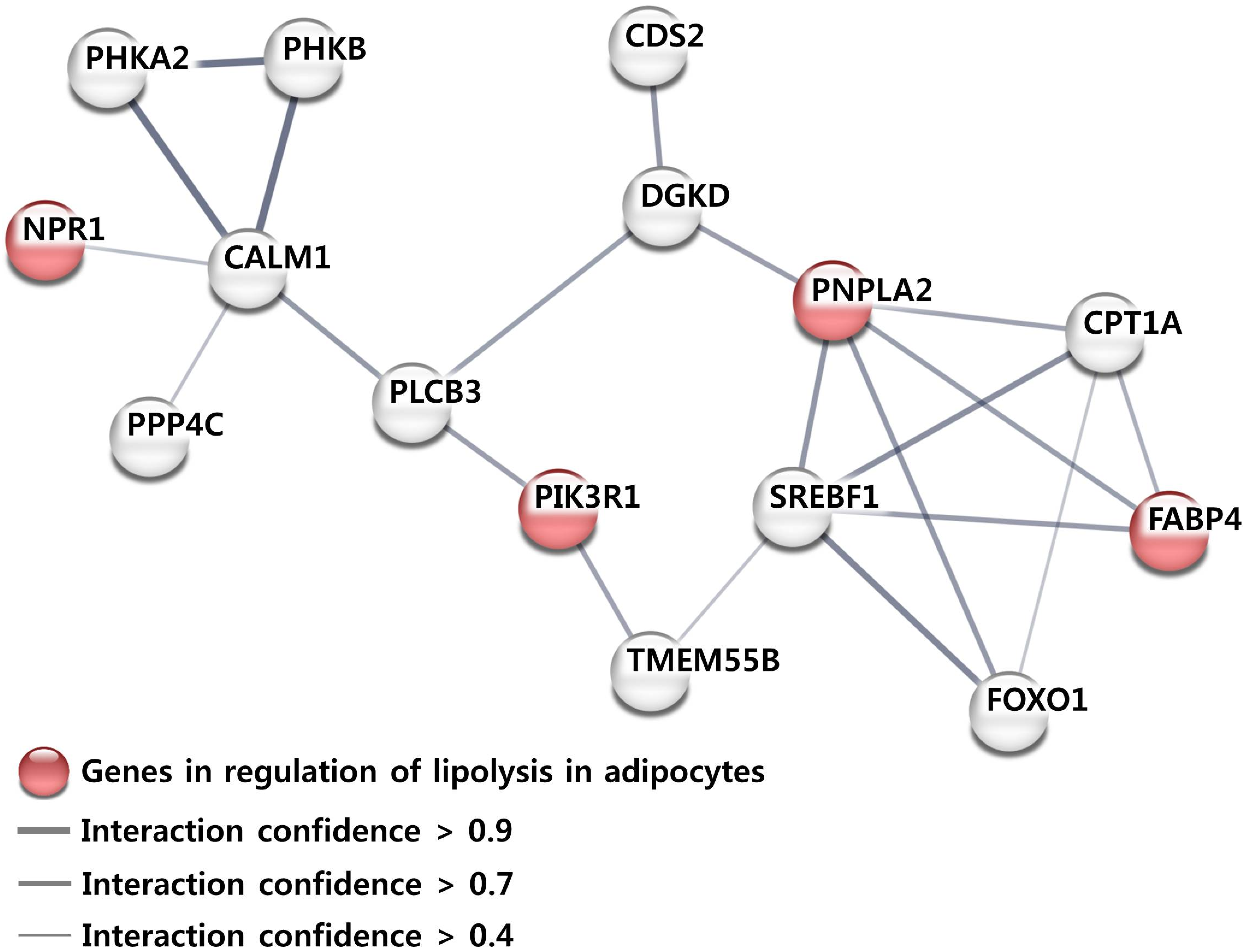

Supplement: Supplementary file 2 — Supplementary Figures [file 41598_2019_52139_MOESM2_ESM.pdf]
