# Supplementary material for: 8-OxoG in GC-rich Sp1 binding sites enhances gene transcription in adipose tissue of juvenile mice
Source: Sci Rep. 2019 Oct 30;9:15618. doi: 10.1038/s41598-019-52139-z (PMC6821754; doi:10.1038/s41598-019-52139-z)
Supplement: Supplementary file 1 — Supplementary Tables [file 41598_2019_52139_MOESM1_ESM.pdf]

8-OxoG in GC-rich Sp1 binding sites enhances gene transcription in adipose tissue of juvenile mice

Jong Woo Park[1,#,] Young In Han[2,#], Sung Woo Kim[3], Tae Min Kim[2,4], Su Cheong Yeom[2,4], Jaeku Kang[5,*] and Joonghoon Park[2,4,*]

[1]Research Center for Epigenome Regulation, School of Pharmacy, Sungkyunkwan University, Suwon, 16419, Republic of Korea

[2]Institute of Green Bio Science and Technology, Seoul National University, Pyeongchang, 25354, Republic of Korea

[3]Animal Genetic Resources Research Center, National Institute of Animal Science, Rural Development Administration (RDA), Namwon, 55717, Republic of Korea

[4]Department of International Agricultural Technology, Graduate School of International Agricultural Technology, Seoul National University, Pyeongchang, 25354, Republic of Korea

[5]Department of Pharmacology, College of Medicine, Konyang University, Daejeon, 35365, Republic of Korea

*Correspondence: jaeku@konyang.ac.kr (J.K.), joonghoon@snu.ac.kr (J.P.)

[#]The authors contributed equally to this work.

Supplementary Table S1. 8-oxoG sequencing of the genomic DNA of adipose tissues

chr	start	end	width	length	abs_summit	pileup	(-)log10(pvalue)	fold_enrichment	(-)log10(qvalue)	MACS_peakID	annotation	geneChr	geneStart	geneEnd	geneLength	geneStrand	geneId	transcriptId	distanceToTSS	ENSEMBL	SYMBOL	GENENAME
chr1	3041947	3042224	278	279	3042090	76	59.98846	12.32963	55.90987	Adipose_R1_peak_	Distal Intergen	1	3214482	3671498	457017	2	497097	uc007aeu.1	629274	ENSMUSG000	Xkr4	X-linked Kx blood group related 4
chr1	3041965	3042218	254	255	3042087	75	39.53023	6.90301	35.50594	Adipose_R2_peak_	Distal Intergen	1	3214482	3671498	457017	2	497097	uc007aeu.1	629280	ENSMUSG000	Xkr4	X-linked Kx blood group related 4
chr1	3043056	3043366	311	312	3043219	76	63.6162	13.57681	59.50595	Adipose_R1_peak_	Distal Intergen	1	3214482	3671498	457017	2	497097	uc007aeu.1	628132	ENSMUSG000	Xkr4	X-linked Kx blood group related 4
chr1	3043066	3043355	290	291	3043219	90	30.28704	4.32934	26.41128	Adipose_R2_peak_	Distal Intergen	1	3214482	3671498	457017	2	497097	uc007aeu.1	628143	ENSMUSG000	Xkr4	X-linked Kx blood group related 4
chr1	3138954	3139215	262	263	3139087	66	33.09721	6.37683	29.34114	Adipose_R1_peak_	Distal Intergen	1	3214482	3671498	457017	2	497097	uc007aeu.1	532283	ENSMUSG000	Xkr4	X-linked Kx blood group related 4
chr1	3138955	3139205	251	252	3139063	58	25.74274	5.44085	21.94821	Adipose_R2_peak_	Distal Intergen	1	3214482	3671498	457017	2	497097	uc007aeu.1	532293	ENSMUSG000	Xkr4	X-linked Kx blood group related 4
chr1	3241475	3241611	137	138	3241539	20	7.74501	3.7745	4.70467	Adipose_R1_peak_	Intron (uc007a	1	3214482	3671498	457017	2	497097	uc007aeu.1	429887	ENSMUSG000	Xkr4	X-linked Kx blood group related 4
chr1	3242711	3243001	291	292	3242860	34	18.86162	6.29083	15.38482	Adipose_R1_peak_	Intron (uc007a	1	3214482	3671498	457017	2	497097	uc007aeu.1	428497	ENSMUSG000	Xkr4	X-linked Kx blood group related 4
chr1	3768731	3768931	201	202	3768825	29	14.55748	5.39214	11.20652	Adipose_R1_peak_	Distal Intergen	1	3214482	3671498	457017	2	497097	uc007aeu.1	-97233	ENSMUSG000	Xkr4	X-linked Kx blood group related 4
chr1	3921695	3921914	220	221	3921799	27	12.93148	5.03266	9.63827	Adipose_R1_peak_	Distal Intergen	1	3214482	3671498	457017	2	497097	uc007aeu.1	-250197	ENSMUSG000	Xkr4	X-linked Kx blood group related 4
chr1	3987323	3987429	107	108	3987363	28	6.25023	2.67431	3.31626	Adipose_R2_peak_	Distal Intergen	1	3214482	3671498	457017	2	497097	uc007aeu.1	-315825	ENSMUSG000	Xkr4	X-linked Kx blood group related 4
chr1	4019983	4020313	331	332	4020148	107	93.40253	15.65045	89.10152	Adipose_R1_peak_	Distal Intergen	1	4343507	4360314	16808	2	19888	uc007aex.2	340001	ENSMUSG000	Rp1	retinitis pigmentosa 1 (human)
chr1	4020043	4020311	269	270	4020160	70	35.62622	6.54746	31.65691	Adipose_R2_peak_	Distal Intergen	1	4343507	4360314	16808	2	19888	uc007aex.2	340003	ENSMUSG000	Rp1	retinitis pigmentosa 1 (human)
chr1	4188191	4188391	201	202	4188258	27	12.93148	5.03266	9.63827	Adipose_R1_peak_	Distal Intergen	1	4343507	4360314	16808	2	19888	uc007aex.2	171923	ENSMUSG000	Rp1	retinitis pigmentosa 1 (human)
chr1	4188192	4188400	209	210	4188288	38	11.72962	3.59649	8.4047	Adipose_R2_peak_	Distal Intergen	1	4343507	4360314	16808	2	19888	uc007aex.2	171914	ENSMUSG000	Rp1	retinitis pigmentosa 1 (human)
chr1	4191322	4191638	317	318	4191485	54	38.78658	9.88558	34.93639	Adipose_R1_peak_	Distal Intergen	1	4343507	4360314	16808	2	19888	uc007aex.2	168676	ENSMUSG000	Rp1	retinitis pigmentosa 1 (human)
chr1	4191373	4191559	187	188	4191490	42	14.2409	3.96536	10.79479	Adipose_R2_peak_	Distal Intergen	1	4343507	4360314	16808	2	19888	uc007aex.2	168755	ENSMUSG000	Rp1	retinitis pigmentosa 1 (human)
chr1	4192285	4192485	201	202	4192384	24	10.60668	4.49345	7.40961	Adipose_R1_peak_	Distal Intergen	1	4343507	4360314	16808	2	19888	uc007aex.2	167829	ENSMUSG000	Rp1	retinitis pigmentosa 1 (human)
chr1	4249721	4249976	256	257	4249874	78	42.70965	7.2852	38.64095	Adipose_R2_peak_	Distal Intergen	1	4343507	4360314	16808	2	19888	uc007aex.2	110338	ENSMUSG000	Rp1	retinitis pigmentosa 1 (human)
chr1	4249725	4250011	287	288	4249876	106	103.5054	19.23195	99.15611	Adipose_R1_peak_	Distal Intergen	1	4343507	4360314	16808	2	19888	uc007aex.2	110303	ENSMUSG000	Rp1	retinitis pigmentosa 1 (human)
chr1	4277759	4277926	168	169	4277920	30	7.24477	2.85875	4.21917	Adipose_R2_peak_	Distal Intergen	1	4343507	4360314	16808	2	19888	uc007aex.2	82388	ENSMUSG000	Rp1	retinitis pigmentosa 1 (human)
chr1	4873232	4873391	160	161	4873309	24	10.60668	4.49345	7.40961	Adipose_R1_peak_	Intron (uc007a	1	4858328	4897909	39582	1	21399	uc011whu.1	14904	ENSMUSG000	Tcea1	transcription elongation factor A (SII) 1
chr1	4873238	4873365	128	129	4873324	26	5.31252	2.48988	2.47999	Adipose_R2_peak_	Intron (uc007a	1	4858328	4897909	39582	1	21399	uc011whu.1	14910	ENSMUSG000	Tcea1	transcription elongation factor A (SII) 1
chr1	5039382	5039596	215	216	5039493	41	25.38991	7.54899	21.76305	Adipose_R1_peak_	Intron (uc007a	1	4909576	5019539	109964	2	58175	uc007afk.3	-19843	ENSMUSG000	Rgs20	regulator of G-protein signaling 20
chr1	5039435	5039572	138	139	5039504	33	8.83596	3.1354	5.68652	Adipose_R2_peak_	Intron (uc007a	1	4909576	5019539	109964	2	58175	uc007afk.3	-19896	ENSMUSG000	Rgs20	regulator of G-protein signaling 20
chr1	5072004	5072464	461	462	5072218	30	15.39182	5.57188	12.01394	Adipose_R1_peak_	Promoter (1-2	1	4909576	5070285	160710	2	58175	uc007afl.2	-1719	ENSMUSG000	Rgs20	regulator of G-protein signaling 20
chr1	5424824	5425015	192	193	5424941	29	14.55748	5.39214	11.20652	Adipose_R1_peak_	Distal Intergen	1	5588493	5606133	17641	1	18387	uc007afo.2	-163478	ENSMUSG000	Oprk1	opioid receptor, kappa 1
chr1	5424910	5425009	100	101	5424956	32	8.29282	3.04319	5.18352	Adipose_R2_peak_	Distal Intergen	1	5588493	5606133	17641	1	18387	uc007afo.2	-163484	ENSMUSG000	Oprk1	opioid receptor, kappa 1
chr1	5431482	5431669	188	189	5431564	26	12.14079	4.85292	8.87838	Adipose_R1_peak_	Distal Intergen	1	5588493	5606133	17641	1	18387	uc007afo.2	-156824	ENSMUSG000	Oprk1	opioid receptor, kappa 1
chr1	5632966	5633132	167	168	5633101	21	8.43286	3.95423	5.34896	Adipose_R1_peak_	Distal Intergen	1	5596518	5606133	9616	1	18387	uc007afq.2	36448	ENSMUSG000	Oprk1	opioid receptor, kappa 1
chr1	5701612	5701829	218	219	5701695	23	9.86443	4.31371	6.70247	Adipose_R1_peak_	Distal Intergen	1	5596518	5606133	9616	1	18387	uc007afq.2	105094	ENSMUSG000	Oprk1	opioid receptor, kappa 1
chr1	5701716	5701841	126	127	5701787	28	6.25023	2.67431	3.31626	Adipose_R2_peak_	Distal Intergen	1	5596518	5606133	9616	1	18387	uc007afq.2	105198	ENSMUSG000	Oprk1	opioid receptor, kappa 1
chr1	5741126	5741270	145	146	5741195	19	7.07687	3.59476	4.08458	Adipose_R1_peak_	Distal Intergen	1	5596518	5606133	9616	1	18387	uc007afq.2	144608	ENSMUSG000	Oprk1	opioid receptor, kappa 1
chr1	5748548	5748878	331	332	5748786	33	17.97508	6.11109	14.52164	Adipose_R1_peak_	Distal Intergen	1	5596518	5606133	9616	1	18387	uc007afq.2	152030	ENSMUSG000	Oprk1	opioid receptor, kappa 1
chr1	5807306	5807461	156	157	5807395	27	5.77405	2.5821	2.88921	Adipose_R2_peak_	Distal Intergen	1	5913707	5917398	3692	2	226304	uc011whw.1	109937	ENSMUSG000	Npbwr1	neuropeptides B/W receptor 1
chr1	5807328	5807547	220	221	5807470	22	9.13959	4.13397	6.01526	Adipose_R1_peak_	Distal Intergen	1	5913707	5917398	3692	2	226304	uc011whw.1	109851	ENSMUSG000	Npbwr1	neuropeptides B/W receptor 1
chr1	5807743	5807955	213	214	5807820	27	12.93148	5.03266	9.63827	Adipose_R1_peak_	Distal Intergen	1	5913707	5917398	3692	2	226304	uc011whw.1	109443	ENSMUSG000	Npbwr1	neuropeptides B/W receptor 1
chr1	5964083	5964281	199	200	5964195	24	10.60668	4.49345	7.40961	Adipose_R1_peak_	Distal Intergen	1	5913707	5917398	3692	2	226304	uc011whw.1	-46685	ENSMUSG000	Npbwr1	neuropeptides B/W receptor 1
chr1	5964162	5964269	108	109	5964243	28	6.25023	2.67431	3.31626	Adipose_R2_peak_	Distal Intergen	1	5913707	5917398	3692	2	226304	uc011whw.1	-46764	ENSMUSG000	Npbwr1	neuropeptides B/W receptor 1
chr1	5993543	5993753	211	212	5993613	26	12.14079	4.85292	8.87838	Adipose_R1_peak_	Distal Intergen	1	5913707	5917398	3692	2	226304	uc011whw.1	-76145	ENSMUSG000	Npbwr1	neuropeptides B/W receptor 1
chr1	5993558	5993694	137	138	5993650	26	5.31252	2.48988	2.47999	Adipose_R2_peak_	Distal Intergen	1	5913707	5917398	3692	2	226304	uc011whw.1	-76160	ENSMUSG000	Npbwr1	neuropeptides B/W receptor 1
chr1	5994649	5994850	202	203	5994747	22	9.13959	4.13397	6.01526	Adipose_R1_peak_	Distal Intergen	1	5913707	5917398	3692	2	226304	uc011whw.1	-77251	ENSMUSG000	Npbwr1	neuropeptides B/W receptor 1
chr1	5996539	5996864	326	327	5996695	69	55.87963	12.58165	51.83661	Adipose_R1_peak_	Distal Intergen	1	5913707	5917398	3692	2	226304	uc011whw.1	-79141	ENSMUSG000	Npbwr1	neuropeptides B/W receptor 1
chr1	5996616	5996813	198	199	5996689	41	13.59774	3.87315	10.1803	Adipose_R2_peak_	Distal Intergen	1	5913707	5917398	3692	2	226304	uc011whw.1	-79218	ENSMUSG000	Npbwr1	neuropeptides B/W receptor 1
chr1	6028713	6028878	166	167	6028757	33	8.83596	3.1354	5.68652	Adipose_R2_peak_	Distal Intergen	1	5913707	5917398	3692	2	226304	uc011whw.1	-111315	ENSMUSG000	Npbwr1	neuropeptides B/W receptor 1
chr1	6436921	6437177	257	258	6437050	40	24.42476	7.36925	20.81738	Adipose_R1_peak_	Distal Intergen	1	6487231	6860940	373710	1	240690	uc007afw.1	-50054	ENSMUSG000	St18	suppression of tumorigenicity 18
chr1	6436988	6437111	124	125	6437037	33	8.83596	3.1354	5.68652	Adipose_R2_peak_	Distal Intergen	1	6487231	6860940	373710	1	240690	uc007afw.1	-50120	ENSMUSG000	St18	suppression of tumorigenicity 18
chr1	6507651	6507817	167	168	6507679	16	5.20017	3.05554	2.38734	Adipose_R1_peak_	Intron (uc007a	1	6487231	6860940	373710	1	240690	uc007afw.1	20420	ENSMUSG000	St18	suppression of tumorigenicity 18
chr1	6537793	6538054	262	263	6537901	33	17.97508	6.11109	14.52164	Adipose_R1_peak_	Intron (uc007a	1	6487231	6860940	373710	1	240690	uc007afw.1	50562	ENSMUSG000	St18	suppression of tumorigenicity 18
chr1	6537847	6537974	128	129	6537936	27	5.77405	2.5821	2.88921	Adipose_R2_peak_	Intron (uc007a	1	6487231	6860940	373710	1	240690	uc007afw.1	50616	ENSMUSG000	St18	suppression of tumorigenicity 18
chr1	6567611	6567773	163	164	6567735	23	9.86443	4.31371	6.70247	Adipose_R1_peak_	Intron (uc007a	1	6487231	6860940	373710	1	240690	uc007afw.1	80380	ENSMUSG000	St18	suppression of tumorigenicity 18
chr1	6629606	6629775	170	171	6629694	25	11.36566	4.67319	8.13482	Adipose_R1_peak_	Intron (uc007a	1	6730051	6860940	130890	1	240690	uc007afy.1	-100276	ENSMUSG000	St18	suppression of tumorigenicity 18
chr1	6629612	6629775	164	165	6629721	41	13.59774	3.87315	10.1803	Adipose_R2_peak_	Intron (uc007a	1	6730051	6860940	130890	1	240690	uc007afy.1	-100276	ENSMUSG000	St18	suppression of tumorigenicity 18
chr1	6917947	6918115	169	170	6918010	20	7.74501	3.7745	4.70467	Adipose_R1_peak_	Distal Intergen	1	7088920	7173628	84709	1	319263	uc007agb.1	-170805	ENSMUSG000	Pcmtd1	protein-L-isoaspartate (D-aspartate) O-methyltransferase domain containing 1
chr1	7081514	7081722	209	210	7081601	25	11.36566	4.67319	8.13482	Adipose_R1_peak_	Distal Intergen	1	7088920	7173628	84709	1	319263	uc007agb.1	-7198	ENSMUSG000	Pcmtd1	protein-L-isoaspartate (D-aspartate) O-methyltransferase domain containing 1
chr1	7322523	7322675	153	154	7322612	19	7.07687	3.59476	4.08458	Adipose_R1_peak_	Distal Intergen	1	7160085	7173628	13544	1	319263	uc007agd.1	162438	ENSMUSG000	Pcmtd1	protein-L-isoaspartate (D-aspartate) O-methyltransferase domain containing 1
chr1	7322545	7322716	172	173	7322621	38	11.72962	3.59649	8.4047	Adipose_R2_peak_	Distal Intergen	1	7160085	7173628	13544	1	319263	uc007agd.1	162460	ENSMUSG000	Pcmtd1	protein-L-isoaspartate (D-aspartate) O-methyltransferase domain containing 1
chr1	7334405	7334572	168	169	7334493	22	6.91715	3.25564	3.97106	Adipose_R1_peak_	Distal Intergen	1	7160085	7173628	13544	1	319263	uc007agd.1	174320	ENSMUSG000	Pcmtd1	protein-L-isoaspartate (D-aspartate) O-methyltransferase domain containing 1
chr1	7334413	7334577	165	166	7334458	39	9.88015	3.09373	6.67659	Adipose_R2_peak_	Distal Intergen	1	7160085	7173628	13544	1	319263	uc007agd.1	174328	ENSMUSG000	Pcmtd1	protein-L-isoaspartate (D-aspartate) O-methyltransferase domain containing 1
chr1	7453723	7453889	167	168	7453864	18	6.42934	3.41502	3.48999	Adipose_R1_peak_	Distal Intergen	1	7160085	7173628	13544	1	319263	uc007agd.1	293638	ENSMUSG000	Pcmtd1	protein-L-isoaspartate (D-aspartate) O-methyltransferase domain containing 1
chr1	7453742	7453873	132	133	7453830	26	5.31252	2.48988	2.47999	Adipose_R2_peak_	Distal Intergen	1	7160085	7173628	13544	1	319263	uc007agd.1	293657	ENSMUSG000	Pcmtd1	protein-L-isoaspartate (D-aspartate) O-methyltransferase domain containing 1
chr1	7480245	7480580	336	337	7480361	41	11.59866	3.39233	8.2923	Adipose_R2_peak_	Distal Intergen	1	7160085	7173628	13544	1	319263	uc007agd.1	320160	ENSMUSG000	Pcmtd1	protein-L-isoaspartate (D-aspartate) O-methyltransferase domain containing 1
chr1	7480304	7480435	132	133	7480353	23	6.81428	3.14204	3.86956	Adipose_R1_peak_	Distal Intergen	1	7160085	7173628	13544	1	319263	uc007agd.1	320219	ENSMUSG000	Pcmtd1	protein-L-isoaspartate (D-aspartate) O-methyltransferase domain containing 1
chr1	7481645	7481784	140	141	7481712	40	12.96465	3.78093	9.57654	Adipose_R2_peak_	Distal Intergen	1	7160085	7173628	13544	1	319263	uc007agd.1	321560	ENSMUSG000	Pcmtd1	protein-L-isoaspartate (D-aspartate) O-methyltransferase domain containing 1
chr1	7485115	7485324	210	211	7485215	27	12.93148	5.03266	9.63827	Adipose_R1_peak_	Distal Intergen	1	7160085	7173628	13544	1	319263	uc007agd.1	325030	ENSMUSG000	Pcmtd1	protein-L-isoaspartate (D-aspartate) O-methyltransferase domain containing 1
chr1	7711122	7711361	240	241	7711243	28	13.73721	5.2124	10.41463	Adipose_R1_peak_	Distal Intergen	1	7160085	7173628	13544	1	319263	uc007agd.1	551037	ENSMUSG000	Pcmtd1	protein-L-isoaspartate (D-aspartate) O-methyltransferase domain containing 1
chr1	7719831	7720022	192	193	7719953	25	11.36566	4.67319	8.13482	Adipose_R1_peak_	Distal Intergen	1	7160085	7173628	13544	1	319263	uc007agd.1	559746	ENSMUSG000	Pcmtd1	protein-L-isoaspartate (D-aspartate) O-methyltransferase domain containing 1
chr1	7997343	7997499	157	158	7997407	19	7.07687	3.59476	4.08458	Adipose_R1_peak_	Distal Intergen	1	8359739	8470519	110781	2	71096	uc007agg.2	473020	ENSMUSG000	Sntg1	syntrophin, gamma 1
chr1	8075769	8075928	160	161	8075864	16	5.20017	3.05554	2.38734	Adipose_R1_peak_	Distal Intergen	1	8359739	8470519	110781	2	71096	uc007agg.2	394591	ENSMUSG000	Sntg1	syntrophin, gamma 1
chr1	8128328	8128462	135	136	8128394	21	8.43286	3.95423	5.34896	Adipose_R1_peak_	Distal Intergen	1	8359739	8470519	110781	2	71096	uc007agg.2	342057	ENSMUSG000	Sntg1	syntrophin, gamma 1
chr1	8162759	8163053	295	296	8162926	35	19.76029	6.47056	16.26028	Adipose_R1_peak_	Distal Intergen	1	8359739	8470519	110781	2	71096	uc007agg.2	307466	ENSMUSG000	Sntg1	syntrophin, gamma 1
chr1	8217410	8217761	352	353	8217571	56	40.97333	10.24506	37.09402	Adipose_R1_peak_	Distal Intergen	1	8359739	8470519	110781	2	71096	uc007agg.2	252758	ENSMUSG000	Sntg1	syntrophin, gamma 1
chr1	8217449	8217663	215	216	8217564	36	10.5378	3.41206	7.27949	Adipose_R2_peak_	Distal Intergen	1	8359739	8470519	110781	2	71096	uc007agg.2	252856	ENSMUSG000	Sntg1	syntrophin, gamma 1
chr1	8240466	8240640	175	176	8240561	27	12.93148	5.03266	9.63827	Adipose_R1_peak_	Distal Intergen	1	8359739	8470519	110781	2	71096	uc007agg.2	229879	ENSMUSG000	Sntg1	syntrophin, gamma 1
chr1	8240480	8240625	146	147	8240545	33	8.83596	3.1354	5.68652	Adipose_R2_peak_	Distal Intergen	1	8359739	8470519	110781	2	71096	uc007agg.2	229894	ENSMUSG000	Sntg1	syntrophin, gamma 1
chr1	8281580	8281745	166	167	8281656	27	12.93148	5.03266	9.63827	Adipose_R1_peak_	Distal Intergen	1	8359739	8470519	110781	2	71096	uc007agg.2	188774	ENSMUSG000	Sntg1	syntrophin, gamma 1
chr1	8376488	8376751	264	265	8376613	34	18.86162	6.29083	15.38482	Adipose_R1_peak_	Intron (uc007a	1	8359739	8470519	110781	2	71096	uc007agg.2	93768	ENSMUSG000	Sntg1	syntrophin, gamma 1
chr1	8376512	8376684	173	174	8376568	28	6.25023	2.67431	3.31626	Adipose_R2_peak_	Intron (uc007a	1	8359739	8470519	110781	2	71096	uc007agg.2	93835	ENSMUSG000	Sntg1	syntrophin, gamma 1
chr1	8519789	8519964	176	177	8519865	25	11.36566	4.67319	8.13482	Adipose_R1_peak_	Intron (uc007a	1	8359739	8470519	110781	2	71096	uc007agg.2	-49270	ENSMUSG000	Sntg1	syntrophin, gamma 1
chr1	8600600	8600864	265	266	8600735	65	51.17258	11.8627	47.17609	Adipose_R1_peak_	Intron (uc007a	1	8359739	8470519	110781	2	71096	uc007agg.2	-130081	ENSMUSG000	Sntg1	syntrophin, gamma 1
chr1	8600654	8600846	193	194	8600737	39	12.34187	3.68871	8.98501	Adipose_R2_peak_	Intron (uc007a	1	8359739	8470519	110781	2	71096	uc007agg.2	-130135	ENSMUSG000	Sntg1	syntrophin, gamma 1
chr1	8635294	8635493	200	201	8635412	25	11.36566	4.67319	8.13482	Adipose_R1_peak_	Intron (uc007a	1	8359739	8470519	110781	2	71096	uc007agg.2	-164775	ENSMUSG000	Sntg1	syntrophin, gamma 1
chr1	8638506	8638637	132	133	8638570	20	7.74501	3.7745	4.70467	Adipose_R1_peak_	Intron (uc007a	1	8359739	8470519	110781	2	71096	uc007agg.2	-167987	ENSMUSG000	Sntg1	syntrophin, gamma 1
chr1	8639042	8639183	142	143	8639112	22	9.13959	4.13397	6.01526	Adipose_R1_peak_	Intron (uc007a	1	8359739	8470519	110781	2	71096	uc007agg.2	-168523	ENSMUSG000	Sntg1	syntrophin, gamma 1
chr1	8668976	8669163	188	189	8669062	22	9.13959	4.13397	6.01526	Adipose_R1_peak_	Intron (uc007a	1	8359739	8470519	110781	2	71096	uc007agg.2	-198457	ENSMUSG000	Sntg1	syntrophin, gamma 1
chr1	8668978	8669138	161	162	8669092	29	6.74062	2.76653	3.75989	Adipose_R2_peak_	Intron (uc007a	1	8359739	8470519	110781	2	71096	uc007agg.2	-198459	ENSMUSG000	Sntg1	syntrophin, gamma 1
chr1	8692458	8692704	247	248	8692578	37	20.03813	6.24876	16.53657	Adipose_R1_peak_	Intron (uc007a	1	8359739	8470519	110781	2	71096	uc007agg.2	-221939	ENSMUSG000	Sntg1	syntrophin, gamma 1
chr1	8692491	8692654	164	165	8692575	39	12.34187	3.68871	8.98501	Adipose_R2_peak_	Intron (uc007a	1	8359739	8470519	110781	2	71096	uc007agg.2	-221972	ENSMUSG000	Sntg1	syntrophin, gamma 1
chr1	8716842	8717192	351	352	8717001	91	83.39809	16.53589	79.14825	Adipose_R1_peak_	Intron (uc007a	1	8359739	8470519	110781	2	71096	uc007agg.2	-246323	ENSMUSG000	Sntg1	syntrophin, gamma 1

chr1	8716867	8717149	283	284	8716995	69	34.76717	6.45524	30.8116	Adipose_R2_peak_6	Intron (uc007a	1	8359739	8470519	110781	2	71096	uc007agg.2	-246348	ENSMUSG000	Sntg1	syntrophin, gamma 1
chr1	8718079	8718369	291	292	8718243	69	45.7172	9.26367	41.78275	Adipose_R1_peak_3	Intron (uc007a	1	8359739	8470519	110781	2	71096	uc007agg.2	-247560	ENSMUSG000	Sntg1	syntrophin, gamma 1
chr1	8718142	8718356	215	216	8718235	56	24.19188	5.25641	20.43163	Adipose_R2_peak_6	Intron (uc007a	1	8359739	8470519	110781	2	71096	uc007agg.2	-247623	ENSMUSG000	Sntg1	syntrophin, gamma 1
chr1	8763203	8763402	200	201	8763307	26	12.14079	4.85292	8.87838	Adipose_R1_peak_3	Intron (uc007a	1	8359739	8470519	110781	2	71096	uc007agg.2	-292684	ENSMUSG000	Sntg1	syntrophin, gamma 1
chr1	8798481	8798655	175	176	8798553	24	10.60668	4.49345	7.40961	Adipose_R1_peak_3	Intron (uc007a	1	8359739	8470519	110781	2	71096	uc007agg.2	-327962	ENSMUSG000	Sntg1	syntrophin, gamma 1
chr1	8798493	8798643	151	152	8798566	31	7.7623	2.95097	4.69388	Adipose_R2_peak_6	Intron (uc007a	1	8359739	8470519	110781	2	71096	uc007agg.2	-327974	ENSMUSG000	Sntg1	syntrophin, gamma 1
chr1	8988972	8989118	147	148	8989031	16	5.20017	3.05554	2.38734	Adipose_R1_peak_3	Intron (uc007a	1	8359739	9299877	940139	2	71096	uc007agh.2	310759	ENSMUSG000	Sntg1	syntrophin, gamma 1
chr1	9028840	9029043	204	205	9028940	29	14.55748	5.39214	11.20652	Adipose_R1_peak_3	Intron (uc007a	1	8359739	9299877	940139	2	71096	uc007agh.2	270834	ENSMUSG000	Sntg1	syntrophin, gamma 1
chr1	9028879	9029022	144	145	9028956	30	7.24477	2.85875	4.21917	Adipose_R2_peak_6	Intron (uc007a	1	8359739	9299877	940139	2	71096	uc007agh.2	270855	ENSMUSG000	Sntg1	syntrophin, gamma 1
chr1	9045028	9045252	225	226	9045126	32	17.10103	5.93135	13.67137	Adipose_R1_peak_3	Intron (uc007a	1	8359739	9299877	940139	2	71096	uc007agh.2	254625	ENSMUSG000	Sntg1	syntrophin, gamma 1
chr1	9048704	9049263	560	561	9048830	31	16.23981	5.75161	12.83549	Adipose_R1_peak_3	Intron (uc007a	1	8359739	9299877	940139	2	71096	uc007agh.2	250614	ENSMUSG000	Sntg1	syntrophin, gamma 1
chr1	9049097	9049209	113	114	9049152	34	9.39139	3.22762	6.20474	Adipose_R2_peak_6	Intron (uc007a	1	8359739	9299877	940139	2	71096	uc007agh.2	250668	ENSMUSG000	Sntg1	syntrophin, gamma 1
chr1	9123505	9123668	164	165	9123566	17	5.80341	3.23528	2.92378	Adipose_R1_peak_3	Intron (uc007a	1	8359739	9299877	940139	2	71096	uc007agh.2	176209	ENSMUSG000	Sntg1	syntrophin, gamma 1
chr1	9176444	9176675	232	233	9176554	50	34.50784	9.16663	30.72138	Adipose_R1_peak_3	Intron (uc007a	1	8359739	9299877	940139	2	71096	uc007agh.2	123202	ENSMUSG000	Sntg1	syntrophin, gamma 1
chr1	9176455	9176659	205	206	9176535	37	11.12817	3.50427	7.83613	Adipose_R2_peak_6	Intron (uc007a	1	8359739	9299877	940139	2	71096	uc007agh.2	123218	ENSMUSG000	Sntg1	syntrophin, gamma 1
chr1	9177843	9178046	204	205	9177929	29	14.55748	5.39214	11.20652	Adipose_R1_peak_3	Intron (uc007a	1	8359739	9299877	940139	2	71096	uc007agh.2	121831	ENSMUSG000	Sntg1	syntrophin, gamma 1
chr1	9180908	9181168	261	262	9181036	41	25.38991	7.54899	21.76305	Adipose_R1_peak_3	Intron (uc007a	1	8359739	9299877	940139	2	71096	uc007agh.2	118709	ENSMUSG000	Sntg1	syntrophin, gamma 1
chr1	9180970	9181079	110	111	9181029	29	6.74062	2.76653	3.75989	Adipose_R2_peak_6	Intron (uc007a	1	8359739	9299877	940139	2	71096	uc007agh.2	118798	ENSMUSG000	Sntg1	syntrophin, gamma 1
chr1	9232649	9232847	199	200	9232753	30	15.39182	5.57188	12.01394	Adipose_R1_peak_3	Intron (uc007a	1	8359739	9299877	940139	2	71096	uc007agh.2	67030	ENSMUSG000	Sntg1	syntrophin, gamma 1
chr1	9241833	9242052	220	221	9241964	40	24.42476	7.36925	20.81738	Adipose_R1_peak_3	Intron (uc007a	1	8359739	9299877	940139	2	71096	uc007agh.2	57825	ENSMUSG000	Sntg1	syntrophin, gamma 1
chr1	10387980	10388280	301	302	10388111	110	106.3556	19.02201	101.9939	Adipose_R1_peak_3	Intron (uc007a	1	10137507	10232670	95164	2	211673	uc007ahn.1	-155310	ENSMUSG000	Arfgef1	ADP-ribosylation factor guanine nucleotide-exchange factor 1(brefeldin A-inhibited)
chr1	10388005	10388246	242	243	10388105	95	58.88104	8.8529	54.63476	Adipose_R2_peak_6	Intron (uc007a	1	10137507	10232670	95164	2	211673	uc007ahn.1	-155335	ENSMUSG000	Arfgef1	ADP-ribosylation factor guanine nucleotide-exchange factor 1(brefeldin A-inhibited)
chr1	10423016	10423294	279	280	10423193	88	79.49821	15.99667	75.27097	Adipose_R1_peak_3	Intron (uc007a	1	10137507	10232670	95164	2	211673	uc007ahn.1	-190346	ENSMUSG000	Arfgef1	ADP-ribosylation factor guanine nucleotide-exchange factor 1(brefeldin A-inhibited)
chr1	10423030	10423289	260	261	10423182	89	53.01237	8.2996	48.82483	Adipose_R2_peak_7	Intron (uc007a	1	10137507	10232670	95164	2	211673	uc007ahn.1	-190360	ENSMUSG000	Arfgef1	ADP-ribosylation factor guanine nucleotide-exchange factor 1(brefeldin A-inhibited)
chr1	10425123	10425435	313	314	10425277	61	46.56983	11.14375	42.62347	Adipose_R1_peak_3	Intron (uc007a	1	10137507	10232670	95164	2	211673	uc007ahn.1	-192453	ENSMUSG000	Arfgef1	ADP-ribosylation factor guanine nucleotide-exchange factor 1(brefeldin A-inhibited)
chr1	10425217	10425414	198	199	10425306	46	16.90986	4.33423	13.35898	Adipose_R2_peak_7	Intron (uc007a	1	10137507	10232670	95164	2	211673	uc007ahn.1	-192547	ENSMUSG000	Arfgef1	ADP-ribosylation factor guanine nucleotide-exchange factor 1(brefeldin A-inhibited)
chr1	10470437	10470618	182	183	10470532	23	9.86443	4.31371	6.70247	Adipose_R1_peak_3	Intron (uc007a	1	10137507	10232670	95164	2	211673	uc007ahn.1	-237767	ENSMUSG000	Arfgef1	ADP-ribosylation factor guanine nucleotide-exchange factor 1(brefeldin A-inhibited)
chr1	10864565	10864862	298	299	10864726	36	20.67077	6.6503	17.14779	Adipose_R1_peak_3	Distal Intergen	1	10993465	11303682	310218	1	109294	uc007ahs.2	-128603	ENSMUSG000	Prex2	phosphatidylinositol-3,4,5-trisphosphate-dependent Rac exchange factor 2
chr1	10864606	10864844	239	240	10864723	41	13.59774	3.87315	10.1803	Adipose_R2_peak_7	Distal Intergen	1	10993465	11303682	310218	1	109294	uc007ahs.2	-128621	ENSMUSG000	Prex2	phosphatidylinositol-3,4,5-trisphosphate-dependent Rac exchange factor 2
chr1	10894717	10894852	136	137	10894779	37	11.12817	3.50427	7.83613	Adipose_R2_peak_7	Distal Intergen	1	10993465	11303682	310218	1	109294	uc007ahs.2	-98613	ENSMUSG000	Prex2	phosphatidylinositol-3,4,5-trisphosphate-dependent Rac exchange factor 2
chr1	10972511	10972666	156	157	10972579	23	9.86443	4.31371	6.70247	Adipose_R1_peak_3	Distal Intergen	1	10993465	11303682	310218	1	109294	uc007ahs.2	-20799	ENSMUSG000	Prex2	phosphatidylinositol-3,4,5-trisphosphate-dependent Rac exchange factor 2
chr1	11070044	11070399	356	357	11070141	26	12.14079	4.85292	8.87838	Adipose_R1_peak_3	Intron (uc007a	1	11098463	11150071	51609	1	109294	uc007aht.1	-28064	ENSMUSG000	Prex2	phosphatidylinositol-3,4,5-trisphosphate-dependent Rac exchange factor 2
chr1	11070190	11070299	110	111	11070267	28	6.25023	2.67431	3.31626	Adipose_R2_peak_7	Intron (uc007a	1	11098463	11150071	51609	1	109294	uc007aht.1	-28164	ENSMUSG000	Prex2	phosphatidylinositol-3,4,5-trisphosphate-dependent Rac exchange factor 2
chr1	11470316	11470633	318	319	11470496	98	81.07878	14.10339	76.84294	Adipose_R1_peak_3	Intron (uc011v	1	11414105	11596317	182213	1	320492	uc011wii.1	56211	ENSMUSG000	A830018L16Ri	RIKEN cDNA A830018L16 gene
chr1	11470343	11470627	285	286	11470486	94	57.89143	8.76068	53.65463	Adipose_R2_peak_7	Intron (uc011v	1	11414105	11596317	182213	1	320492	uc011wii.1	56238	ENSMUSG000	A830018L16Ri	RIKEN cDNA A830018L16 gene
chr1	11725622	11725817	196	197	11725724	29	14.55748	5.39214	11.20652	Adipose_R1_peak_3	Intron (uc007a	1	11596151	11933740	337590	1	320492	uc007ahz.1	129471	ENSMUSG000	A830018L16Ri	RIKEN cDNA A830018L16 gene
chr1	11725667	11725791	125	126	11725712	28	6.25023	2.67431	3.31626	Adipose_R2_peak_7	Intron (uc007a	1	11596151	11933740	337590	1	320492	uc007ahz.1	129516	ENSMUSG000	A830018L16Ri	RIKEN cDNA A830018L16 gene
chr1	11727552	11727742	191	192	11727671	20	7.74501	3.7745	4.70467	Adipose_R1_peak_3	Intron (uc007a	1	11596151	11933740	337590	1	320492	uc007ahz.1	131401	ENSMUSG000	A830018L16Ri	RIKEN cDNA A830018L16 gene
chr1	11728215	11728436	222	223	11728312	25	11.36566	4.67319	8.13482	Adipose_R1_peak_3	Intron (uc007a	1	11596151	11933740	337590	1	320492	uc007ahz.1	132064	ENSMUSG000	A830018L16Ri	RIKEN cDNA A830018L16 gene
chr1	11781725	11782265	541	542	11781817	25	11.36566	4.67319	8.13482	Adipose_R1_peak_3	Intron (uc007a	1	11596151	11933740	337590	1	320492	uc007ahz.1	185574	ENSMUSG000	A830018L16Ri	RIKEN cDNA A830018L16 gene
chr1	11782008	11782225	218	219	11782120	36	10.5378	3.41206	7.27949	Adipose_R2_peak_7	Intron (uc007a	1	11596151	11933740	337590	1	320492	uc007ahz.1	185857	ENSMUSG000	A830018L16Ri	RIKEN cDNA A830018L16 gene
chr1	11782551	11782804	254	255	11782668	34	18.86162	6.29083	15.38482	Adipose_R1_peak_3	Intron (uc007a	1	11596151	11933740	337590	1	320492	uc007ahz.1	186400	ENSMUSG000	A830018L16Ri	RIKEN cDNA A830018L16 gene
chr1	11808106	11808347	242	243	11808235	47	24.45499	6.28408	20.84733	Adipose_R1_peak_3	Intron (uc007a	1	11596151	11933740	337590	1	320492	uc007ahz.1	211955	ENSMUSG000	A830018L16Ri	RIKEN cDNA A830018L16 gene
chr1	11808160	11808318	159	160	11808231	28	6.25023	2.67431	3.31626	Adipose_R2_peak_7	Intron (uc007a	1	11596151	11933740	337590	1	320492	uc007ahz.1	212009	ENSMUSG000	A830018L16Ri	RIKEN cDNA A830018L16 gene
chr1	11810308	11810590	283	284	11810443	41	25.38991	7.54899	21.76305	Adipose_R1_peak_3	Intron (uc007a	1	11596151	11933740	337590	1	320492	uc007ahz.1	214157	ENSMUSG000	A830018L16Ri	RIKEN cDNA A830018L16 gene
chr1	12038976	12039217	242	243	12039085	32	17.10103	5.93135	13.67137	Adipose_R1_peak_3	Distal Intergen	1	12425986	12426106	121	1	1.02E+08	uc033fic.1	-386769	ENSMUSG000	Mir6341	microRNA 6341
chr1	12039018	12039166	149	150	12039098	34	9.39139	3.22762	6.20474	Adipose_R2_peak_7	Distal Intergen	1	12425986	12426106	121	1	1.02E+08	uc033fic.1	-386820	ENSMUSG000	Mir6341	microRNA 6341
chr1	12078348	12078552	205	206	12078451	25	11.36566	4.67319	8.13482	Adipose_R1_peak_3	Distal Intergen	1	12425986	12426106	121	1	1.02E+08	uc033fic.1	-347434	ENSMUSG000	Mir6341	microRNA 6341
chr1	12176034	12176164	131	132	12176099	21	8.43286	3.95423	5.34896	Adipose_R1_peak_3	Distal Intergen	1	12425986	12426106	121	1	1.02E+08	uc033fic.1	-249822	ENSMUSG000	Mir6341	microRNA 6341
chr1	12218956	12219155	200	201	12219039	20	7.74501	3.7745	4.70467	Adipose_R1_peak_3	Distal Intergen	1	12425986	12426106	121	1	1.02E+08	uc033fic.1	-206831	ENSMUSG000	Mir6341	microRNA 6341
chr1	12375472	12375737	266	267	12375596	59	44.30963	10.78427	40.38949	Adipose_R1_peak_3	Distal Intergen	1	12425986	12426106	121	1	1.02E+08	uc033fic.1	-50249	ENSMUSG000	Mir6341	microRNA 6341
chr1	12375488	12375721	234	235	12375605	51	20.44827	4.79532	16.78434	Adipose_R2_peak_7	Distal Intergen	1	12425986	12426106	121	1	1.02E+08	uc033fic.1	-50265	ENSMUSG000	Mir6341	microRNA 6341
chr1	13092773	13092920	148	149	13092833	29	6.74062	2.76653	3.75989	Adipose_R2_peak_7	Distal Intergen	1	13113428	13127163	13736	2	383491	uc007aik.1	34243	ENSMUSG000	Prdm14	PR domain containing 14
chr1	13209399	13209508	110	111	13209451	34	9.39139	3.22762	6.20474	Adipose_R2_peak_7	Intron (uc007a	1	13113428	13127163	13736	2	383491	uc007aik.1	-82236	ENSMUSG000	Prdm14	PR domain containing 14
chr1	13210196	13210394	199	200	13210298	48	16.26567	4.04736	12.73901	Adipose_R2_peak_7	Intron (uc007a	1	13113428	13127163	13736	2	383491	uc007aik.1	-83033	ENSMUSG000	Prdm14	PR domain containing 14
chr1	13210230	13210371	142	143	13210302	28	9.76182	3.75633	6.63205	Adipose_R1_peak_3	Intron (uc007a	1	13113428	13127163	13736	2	383491	uc007aik.1	-83067	ENSMUSG000	Prdm14	PR domain containing 14
chr1	13213342	13213452	111	112	13213395	29	6.74062	2.76653	3.75989	Adipose_R2_peak_7	Intron (uc007a	1	13113428	13127163	13736	2	383491	uc007aik.1	-86179	ENSMUSG000	Prdm14	PR domain containing 14
chr1	13272953	13273100	148	149	13272996	16	5.20017	3.05554	2.38734	Adipose_R1_peak_3	Intron (uc007a	1	13139105	13372429	233325	2	17978	uc007ail.2	99329	ENSMUSG000	Ncoa2	nuclear receptor coactivator 2
chr1	13338427	13338912	486	487	13338695	29	14.55748	5.39214	11.20652	Adipose_R1_peak_3	Intron (uc007a	1	13139105	13372429	233325	2	17978	uc007ail.2	33517	ENSMUSG000	Ncoa2	nuclear receptor coactivator 2
chr1	13471925	13472119	195	196	13472024	38	11.72962	3.59649	8.4047	Adipose_R2_peak_7	Distal Intergen	1	13139105	13374083	234979	2	17978	uc007aim.2	-97842	ENSMUSG000	Ncoa2	nuclear receptor coactivator 2
chr1	13471954	13472168	215	216	13472062	33	17.97508	6.11109	14.52164	Adipose_R1_peak_3	Distal Intergen	1	13139105	13374083	234979	2	17978	uc007aim.2	-97871	ENSMUSG000	Ncoa2	nuclear receptor coactivator 2
chr1	13477698	13477882	185	186	13477813	37	21.59274	6.83004	18.04766	Adipose_R1_peak_3	Distal Intergen	1	13139105	13374083	234979	2	17978	uc007aim.2	-103615	ENSMUSG000	Ncoa2	nuclear receptor coactivator 2
chr1	13477755	13477870	116	117	13477826	28	6.25023	2.67431	3.31626	Adipose_R2_peak_7	Distal Intergen	1	13139105	13374083	234979	2	17978	uc007aim.2	-103672	ENSMUSG000	Ncoa2	nuclear receptor coactivator 2
chr1	13554264	13554509	246	247	13554365	33	17.97508	6.11109	14.52164	Adipose_R1_peak_3	Distal Intergen	1	13564693	13589910	25218	2	72265	uc007ait.2	35401	ENSMUSG000	Tram1	translocating chain-associating membrane protein 1
chr1	13554313	13554467	155	156	13554368	33	8.83596	3.1354	5.68652	Adipose_R2_peak_7	Distal Intergen	1	13564693	13589910	25218	2	72265	uc007ait.2	35443	ENSMUSG000	Tram1	translocating chain-associating membrane protein 1
chr1	13819967	13820160	194	195	13820076	43	14.8939	4.05758	11.42017	Adipose_R2_peak_7	Distal Intergen	1	13785685	13786929	1245	2	433273	uc011wil.1	-33038	ENSMUSG000	Gm5523	glyceraldehyde-3-phosphate dehydrogenase pseudogene
chr1	13819985	13820160	176	177	13820064	25	11.36566	4.67319	8.13482	Adipose_R1_peak_3	Distal Intergen	1	13785685	13786929	1245	2	433273	uc011wil.1	-33056	ENSMUSG000	Gm5523	glyceraldehyde-3-phosphate dehydrogenase pseudogene
chr1	13844900	13845055	156	157	13844989	17	5.80341	3.23528	2.92378	Adipose_R1_peak_3	Distal Intergen	1	13785685	13786929	1245	2	433273	uc011wil.1	-57971	ENSMUSG000	Gm5523	glyceraldehyde-3-phosphate dehydrogenase pseudogene
chr1	13844974	13845093	120	121	13845041	27	5.77405	2.5821	2.88921	Adipose_R2_peak_7	Distal Intergen	1	13785685	13786929	1245	2	433273	uc011wil.1	-58045	ENSMUSG000	Gm5523	glyceraldehyde-3-phosphate dehydrogenase pseudogene
chr1	13961672	13961862	191	192	13961774	21	8.43286	3.95423	5.34896	Adipose_R1_peak_3	Distal Intergen	1	13785685	13786929	1245	2	433273	uc011wil.1	-174743	ENSMUSG000	Gm5523	glyceraldehyde-3-phosphate dehydrogenase pseudogene
chr1	14086959	14087158	200	201	14087110	19	7.07687	3.59476	4.08458	Adipose_R1_peak_3	Distal Intergen	1	14168958	14304570	135613	2	14048	uc007aiw.1	217412	ENSMUSG000	Eya1	EYA transcriptional coactivator and phosphatase 1
chr1	14117880	14118193	314	315	14118012	75	63.12309	13.66008	59.01505	Adipose_R1_peak_3	Distal Intergen	1	14168958	14304570	135613	2	14048	uc007aiw.1	186377	ENSMUSG000	Eya1	EYA transcriptional coactivator and phosphatase 1
chr1	14117891	14118170	280	281	14118015	68	33.91425	6.36302	29.97245	Adipose_R2_peak_7	Distal Intergen	1	14168958	14304570	135613	2	14048	uc007aiw.1	186400	ENSMUSG000	Eya1	EYA transcriptional coactivator and phosphatase 1
chr1	14959432	14959711	280	281	14959564	64	50.01186	11.68296	46.0279	Adipose_R1_peak_3	Distal Intergen	1	14872648	14918862	46215	2	277328	uc007ajc.1	-40570	ENSMUSG000	Trpa1	transient receptor potential cation channel, subfamily A, member 1
chr1	14959439	14959709	271	272	14959558	62	28.93052	5.80972	25.0743	Adipose_R2_peak_7	Distal Intergen	1	14872648	14918862	46215	2	277328	uc007ajc.1	-40577	ENSMUSG000	Trpa1	transient receptor potential cation channel, subfamily A, member 1
chr1	14988786	14988916	131	132	14988850	18	6.42934	3.41502	3.48999	Adipose_R1_peak_3	Distal Intergen	1	14872648	14918862	46215	2	277328	uc007ajc.1	-69924	ENSMUSG000	Trpa1	transient receptor potential cation channel, subfamily A, member 1
chr1	14991107	14991360	254	255	14991230	40	24.42476	7.36925	20.81738	Adipose_R1_peak_3	Distal Intergen	1	14872648	14918862	46215	2	277328	uc007ajc.1	-72245	ENSMUSG000	Trpa1	transient receptor potential cation channel, subfamily A, member 1
chr1	14991138	14991286	149	150	14991218	33	8.83596	3.1354	5.68652	Adipose_R2_peak_7	Distal Intergen	1	14872648	14918862	46215	2	277328	uc007ajc.1	-72276	ENSMUSG000	Trpa1	transient receptor potential cation channel, subfamily A, member 1
chr1	14993292	14993486	195	196	14993403	26	12.14079	4.85292	8.87838	Adipose_R1_peak_3	Distal Intergen	1	14872648	14918862	46215	2	277328	uc007ajc.1	-74430	ENSMUSG000	Trpa1	transient receptor potential cation channel, subfamily A, member 1
chr1	14993946	14994156	211	212	14994065	26	12.14079	4.85292	8.87838	Adipose_R1_peak_3	Distal Intergen	1	14872648	14918862	46215	2	277328	uc007ajc.1	-75084	ENSMUSG000	Trpa1	transient receptor potential cation channel, subfamily A, member 1
chr1	15022059	15022337	279	280	15022200	61	41.62079	9.43261	37.73869	Adipose_R1_peak_3	Distal Intergen	1	14872648	14918862	46215	2	277328	uc007ajc.1	-103197	ENSMUSG000	Trpa1	transient receptor potential cation channel, subfamily A, member 1
chr1	15022063	15022321	259	260	15022196	60	27.32259	5.62528	23.49644	Adipose_R2_peak_7	Distal Intergen	1	14872648	14918862	46215	2	277328	uc007ajc.1	-103201	ENSMUSG000	Trpa1	transient receptor potential cation channel, subfamily A, member 1
chr1	15036506	15036811	306	307	15036652	60	40.5738	9.28047	36.70618	Adipose_R1_peak_3	Distal Intergen	1	14872648	14918862	46215	2	277328	uc007ajc.1	-117644	ENSMUSG000	Trpa1	transient receptor potential cation channel, subfamily A, member 1
chr1	15036594	15036767	174	175	15036658	42	14.2409	3.96536	10.79479	Adipose_R2_peak_7	Distal Intergen	1	14872648	14918862	46215	2	277328	uc007ajc.1	-117732	ENSMUSG000	Trpa1	transient receptor potential cation channel, subfamily A, member 1
chr1	15230780	15231054	275	276	15230919	26	12.14079	4.85292	8.87838	Adipose_R1_peak_3	Distal Intergen	1	15312452	15714214	401763	1	98741	uc007aje.1	-81398	ENSMUSG000	Kcnb2	potassium voltage gated channel, Shab-related subfamily, member 2
chr1	15230818	15230944	127	128	15230871	29	6.74062	2.76653	3.75989	Adipose_R2_peak_7	Distal Intergen	1	15312452	15714214	401763	1	98741	uc007aje.1	-81508	ENSMUSG000	Kcnb2	potassium voltage gated channel, Shab-related subfamily, member 2
chr1	15233031	15233241	211	212	15233119	35	19.76029	6.47056	16.26028	Adipose_R1_peak_3	Distal Intergen	1	15312452	15714214	401763	1	98741	uc007aje.1	-79211	ENSMUSG000	Kcnb2	potassium voltage gated channel, Shab-related subfamily, member 2
chr1	15233034	15233240	207	208	15233114	47	17.60017	4.42645	14.02531	Adipose_R2_peak_7	Distal Intergen	1	15312452	15714214	401763	1	98741	uc007aje.1	-79212	ENSMUSG000	Kcnb2	potassium voltage gated channel, Shab-related subfamily, member 2
chr1	15246266	15246513	248	249	15246390	31	16.23981	5.75161	12.83549	Adipose_R1_peak_3	Distal Intergen	1	15312452	15714214	401763	1	98741	uc007aje.1	-65939	ENSMUSG000	Kcnb2	potassium voltage gated channel, Shab-related subfamily, member 2
chr1	15259861	15260010	150	151	15259927	23	9.86443	4.31371	6.70247	Adipose_R1_peak_4	Distal Intergen	1	15312452	15714214	401763	1	98741	uc007aje.1	-52442	ENSMUSG000	Kcnb2	potassium voltage gated channel, Shab-related subfamily, member 2
chr1	15740711	15740946	236	237	15740832	35	19.76029	6.47056	16.26028	Adipose_R1_peak_4	Distal Intergen	1	15805646	15822013	16368	1	21749	uc007ajf.2	-64700	ENSMUSG000	Terf1	telomeric repeat binding factor 1
chr1	15805290	15805425	136	137	15805383	28	6.25023	2.67431	3.31626	Adipose_R2_peak_7	Promoter (<=1	1	15805646	15822013	16368	1	21749	uc007ajf.2	-221	ENSMUSG000	Terf1	telomeric repeat binding factor 1
chr1	16071712	16072139	428	429	16071888	26	7.48837	3.16164	4.49102	Adipose_R1_peak_4	Intron (uc029c	1	16065979	16093325	27347	2	75799	uc029qmy.2	21186	ENSMUSG000	4930444P10Ri	RIKEN cDNA 4930444P10 gene
chr1	16071866	16072236	371	372	16072110	46	16.63907	4.26897	13.10529	Adipose_R2_peak_7	Intron (uc029c	1	16065979	16093325	27347	2	75799	uc029qmy.2	21089	ENSMUSG000	4930444P10Ri	RIKEN cDNA 4930444P10 gene

chr1	16073523	16073661	139	140	16073612	22	9.13959	4.13397	6.01526	Adipose_R1_peak_4	Intron (uc029c	1	16065979	16093325	27347	2	75799	uc029qmy.2	19664	ENSMUSG000	4930444P10Ri	RIKEN cDNA 4930444P10 gene
chr1	16197071	16197290	220	221	16197166	43	27.35048	7.90847	23.68561	Adipose_R1_peak_4	Distal Intergen	1	16105882	16132550	26669	1	98711	uc007ajm.1	91189	ENSMUSG000	Rdh10	retinol dehydrogenase 10 (all-trans)
chr1	16197090	16197285	196	197	16197216	37	11.12817	3.50427	7.83613	Adipose_R2_peak_7	Distal Intergen	1	16105882	16132550	26669	1	98711	uc007ajm.1	91208	ENSMUSG000	Rdh10	retinol dehydrogenase 10 (all-trans)
chr1	16333499	16333642	144	145	16333563	20	7.74501	3.7745	4.70467	Adipose_R1_peak_4	Intron (uc007a	1	16228810	16519302	290493	2	29819	uc007ajn.2	185660	ENSMUSG000	Stau2	staufen (RNA binding protein) homolog 2 (Drosophila)
chr1	16382040	16382209	170	171	16382109	46	15.991	4.11521	12.48181	Adipose_R2_peak_7	Intron (uc007a	1	16228810	16519302	290493	2	29819	uc007ajn.2	137093	ENSMUSG000	Stau2	staufen (RNA binding protein) homolog 2 (Drosophila)
chr1	16453894	16454109	216	217	16454027	29	14.55748	5.39214	11.20652	Adipose_R1_peak_4	Intron (uc007a	1	16228810	16519302	290493	2	29819	uc007ajn.2	65193	ENSMUSG000	Stau2	staufen (RNA binding protein) homolog 2 (Drosophila)
chr1	16519533	16519678	146	147	16519578	27	5.77405	2.5821	2.88921	Adipose_R2_peak_7	Promoter (<=	1	16228810	16519302	290493	2	29819	uc007ajn.2	-231	ENSMUSG000	Stau2	staufen (RNA binding protein) homolog 2 (Drosophila)
chr1	16833861	16834135	275	276	16834013	51	20.44827	4.79532	16.78434	Adipose_R2_peak_7	Distal Intergen	1	16688456	16709605	21150	1	17087	uc007ajz.2	145405	ENSMUSG000	Ly96	lymphocyte antigen 96
chr1	16834242	16834460	219	220	16834341	46	16.90986	4.33423	13.35898	Adipose_R2_peak_7	Distal Intergen	1	16688456	16709605	21150	1	17087	uc007ajz.2	145786	ENSMUSG000	Ly96	lymphocyte antigen 96
chr1	16834264	16834401	138	139	16834328	27	8.36459	3.37594	5.31771	Adipose_R1_peak_4	Distal Intergen	1	16688456	16709605	21150	1	17087	uc007ajz.2	145808	ENSMUSG000	Ly96	lymphocyte antigen 96
chr1	17253928	17254058	131	132	17253965	19	7.07687	3.59476	4.08458	Adipose_R1_peak_4	Distal Intergen	1	17145373	17158744	13372	1	14545	uc007akb.2	108555	ENSMUSG000	Gdap1	ganglioside-induced differentiation-associated-protein 1
chr1	17289168	17289549	382	383	17289381	109	67.813	9.05657	63.66855	Adipose_R1_peak_4	Distal Intergen	1	17145373	17158744	13372	1	14545	uc007akb.2	143795	ENSMUSG000	Gdap1	ganglioside-induced differentiation-associated-protein 1
chr1	17289242	17289523	282	283	17289384	111	46.25833	5.47118	42.14953	Adipose_R2_peak_7	Distal Intergen	1	17145373	17158744	13372	1	14545	uc007akb.2	143869	ENSMUSG000	Gdap1	ganglioside-induced differentiation-associated-protein 1
chr1	17290371	17290659	289	290	17290509	69	55.21613	12.34256	51.18214	Adipose_R1_peak_4	Distal Intergen	1	17145373	17158744	13372	1	14545	uc007akb.2	144998	ENSMUSG000	Gdap1	ganglioside-induced differentiation-associated-protein 1
chr1	17290376	17290634	259	260	17290511	72	37.3624	6.73189	33.36734	Adipose_R2_peak_7	Distal Intergen	1	17145373	17158744	13372	1	14545	uc007akb.2	145003	ENSMUSG000	Gdap1	ganglioside-induced differentiation-associated-protein 1
chr1	17387077	17387305	229	230	17387177	47	31.38649	8.62742	27.65048	Adipose_R1_peak_4	Distal Intergen	1	17601901	17630939	29039	1	94227	uc007ake.2	-214596	ENSMUSG000	Pi15	peptidase inhibitor 15
chr1	17387119	17387222	104	105	17387164	27	5.77405	2.5821	2.88921	Adipose_R2_peak_7	Distal Intergen	1	17601901	17630939	29039	1	94227	uc007ake.2	-214679	ENSMUSG000	Pi15	peptidase inhibitor 15
chr1	17467555	17468046	492	493	17467659	25	10.51708	4.33387	7.34966	Adipose_R1_peak_4	Distal Intergen	1	17601901	17630939	29039	1	94227	uc007ake.2	-133855	ENSMUSG000	Pi15	peptidase inhibitor 15
chr1	17467760	17468031	272	273	17467914	54	22.67101	5.07198	18.94758	Adipose_R2_peak_7	Distal Intergen	1	17601901	17630939	29039	1	94227	uc007ake.2	-133870	ENSMUSG000	Pi15	peptidase inhibitor 15
chr1	17471412	17471562	151	152	17471476	23	9.86443	4.31371	6.70247	Adipose_R1_peak_4	Distal Intergen	1	17601901	17630939	29039	1	94227	uc007ake.2	-130339	ENSMUSG000	Pi15	peptidase inhibitor 15
chr1	17474569	17474789	221	222	17474687	38	22.52591	7.00978	18.96	Adipose_R1_peak_4	Distal Intergen	1	17601901	17630939	29039	1	94227	uc007ake.2	-127112	ENSMUSG000	Pi15	peptidase inhibitor 15
chr1	17474641	17474765	125	126	17474715	28	6.25023	2.67431	3.31626	Adipose_R2_peak_7	Distal Intergen	1	17601901	17630939	29039	1	94227	uc007ake.2	-127136	ENSMUSG000	Pi15	peptidase inhibitor 15
chr1	17475960	17476237	278	279	17476111	37	21.59274	6.83004	18.04766	Adipose_R1_peak_4	Distal Intergen	1	17601901	17630939	29039	1	94227	uc007ake.2	-125664	ENSMUSG000	Pi15	peptidase inhibitor 15
chr1	17476015	17476183	169	170	17476106	37	11.12817	3.50427	7.83613	Adipose_R2_peak_7	Distal Intergen	1	17601901	17630939	29039	1	94227	uc007ake.2	-125718	ENSMUSG000	Pi15	peptidase inhibitor 15
chr1	17477843	17478104	262	263	17477961	45	29.34988	8.26794	25.64927	Adipose_R1_peak_4	Distal Intergen	1	17601901	17630939	29039	1	94227	uc007ake.2	-123797	ENSMUSG000	Pi15	peptidase inhibitor 15
chr1	17525350	17525495	146	147	17525386	25	4.86612	2.39766	2.09018	Adipose_R2_peak_7	Distal Intergen	1	17601901	17630939	29039	1	94227	uc007ake.2	-76406	ENSMUSG000	Pi15	peptidase inhibitor 15
chr1	17683231	17683428	198	199	17683417	40	12.96465	3.78093	9.57654	Adipose_R2_peak_7	Intron (uc007a	1	17727052	17766207	39156	1	83691	uc007akg.1	-43624	ENSMUSG000	Crispld1	cysteine-rich secretory protein LCCL domain containing 1
chr1	17683241	17683428	188	189	17683334	24	10.60668	4.49345	7.40961	Adipose_R1_peak_4	Intron (uc007a	1	17727052	17766207	39156	1	83691	uc007akg.1	-43624	ENSMUSG000	Crispld1	cysteine-rich secretory protein LCCL domain containing 1
chr1	17781768	17781945	178	179	17781827	31	7.7623	2.95097	4.69388	Adipose_R2_peak_7	Distal Intergen	1	17676027	17727646	51620	2	1.03E+08	uc007akf.1	-54122	ENSMUSG000	Gm16070	predicted gene 16070
chr1	17781771	17781945	175	176	17781841	26	12.14079	4.85292	8.87838	Adipose_R1_peak_4	Distal Intergen	1	17676027	17727646	51620	2	1.03E+08	uc007akf.1	-54125	ENSMUSG000	Gm16070	predicted gene 16070
chr1	17917681	17917975	295	296	17917833	86	76.92245	15.6372	72.71191	Adipose_R1_peak_4	Distal Intergen	1	17676027	17727646	51620	2	1.03E+08	uc007akf.1	-190035	ENSMUSG000	Gm16070	predicted gene 16070
chr1	17917696	17917975	280	281	17917842	84	48.25301	7.83851	44.11785	Adipose_R2_peak_7	Distal Intergen	1	17676027	17727646	51620	2	1.03E+08	uc007akf.1	-190050	ENSMUSG000	Gm16070	predicted gene 16070
chr1	17958155	17958301	147	148	17958244	20	7.74501	3.7745	4.70467	Adipose_R1_peak_4	Distal Intergen	1	18115191	18137052	21862	2	78081	uc007aki.1	178751	ENSMUSG000	Crisp4	cysteine-rich secretory protein 4
chr1	17960772	17960876	105	106	17960819	32	8.29282	3.04319	5.18352	Adipose_R2_peak_7	Distal Intergen	1	18115191	18137052	21862	2	78081	uc007aki.1	176176	ENSMUSG000	Crisp4	cysteine-rich secretory protein 4
chr1	17962780	17963038	259	260	17962893	37	20.03813	6.24876	16.53657	Adipose_R1_peak_4	Distal Intergen	1	18115191	18137052	21862	2	78081	uc007aki.1	174014	ENSMUSG000	Crisp4	cysteine-rich secretory protein 4
chr1	18079423	18079599	177	178	18079508	23	9.86443	4.31371	6.70247	Adipose_R1_peak_4	Distal Intergen	1	18115191	18137052	21862	2	78081	uc007aki.1	57453	ENSMUSG000	Crisp4	cysteine-rich secretory protein 4
chr1	18477843	18477987	145	146	18477907	19	7.07687	3.59476	4.08458	Adipose_R1_peak_4	Distal Intergen	1	18260524	18265138	4615	2	654464	uc007ako.1	-212705	NA	NA	NA
chr1	18480853	18481019	167	168	18480924	23	9.86443	4.31371	6.70247	Adipose_R1_peak_4	Distal Intergen	1	18260524	18265138	4615	2	654464	uc007ako.1	-215715	NA	NA	NA
chr1	18537763	18537992	230	231	18537877	30	15.39182	5.57188	12.01394	Adipose_R1_peak_4	Distal Intergen	1	18260524	18265138	4615	2	654464	uc007ako.1	-272625	NA	NA	NA
chr1	18537806	18537952	147	148	18537876	33	8.83596	3.1354	5.68652	Adipose_R2_peak_7	Distal Intergen	1	18260524	18265138	4615	2	654464	uc007ako.1	-272668	NA	NA	NA
chr1	18815976	18816230	255	256	18816103	38	22.52591	7.00978	18.96	Adipose_R1_peak_4	Distal Intergen	1	19103022	19166332	63311	1	226896	uc007akp.1	-286792	ENSMUSG000	Tfap2d	transcription factor AP-2, delta
chr1	18877804	18878073	270	271	18877923	41	25.38991	7.54899	21.76305	Adipose_R1_peak_4	Distal Intergen	1	19103022	19166332	63311	1	226896	uc007akp.1	-224949	ENSMUSG000	Tfap2d	transcription factor AP-2, delta
chr1	18877853	18878010	158	159	18877932	36	10.5378	3.41206	7.27949	Adipose_R2_peak_7	Distal Intergen	1	19103022	19166332	63311	1	226896	uc007akp.1	-225012	ENSMUSG000	Tfap2d	transcription factor AP-2, delta
chr1	18931987	18932216	230	231	18932096	34	18.86162	6.29083	15.38482	Adipose_R1_peak_4	Distal Intergen	1	19103022	19166332	63311	1	226896	uc007akp.1	-170806	ENSMUSG000	Tfap2d	transcription factor AP-2, delta
chr1	19104780	19105006	227	228	19104856	21	8.43286	3.95423	5.34896	Adipose_R1_peak_4	Promoter (1-2	1	19103022	19166332	63311	1	226896	uc007akp.1	1758	ENSMUSG000	Tfap2d	transcription factor AP-2, delta
chr1	19126035	19126197	163	164	19126116	20	7.74501	3.7745	4.70467	Adipose_R1_peak_4	Intron (uc007a	1	19103022	19166332	63311	1	226896	uc007akp.1	23013	ENSMUSG000	Tfap2d	transcription factor AP-2, delta
chr1	19313473	19313630	158	159	19313566	19	7.07687	3.59476	4.08458	Adipose_R1_peak_4	Distal Intergen	1	19213927	19238845	24919	1	21419	uc007aku.2	99546	ENSMUSG000	Tfap2b	transcription factor AP-2 beta
chr1	19325262	19325427	166	167	19325335	19	7.07687	3.59476	4.08458	Adipose_R1_peak_4	Distal Intergen	1	19213927	19238845	24919	1	21419	uc007aku.2	111335	ENSMUSG000	Tfap2b	transcription factor AP-2 beta
chr1	19378027	19378313	287	288	19378174	31	16.23981	5.75161	12.83549	Adipose_R1_peak_4	Distal Intergen	1	19213927	19238845	24919	1	21419	uc007aku.2	164100	ENSMUSG000	Tfap2b	transcription factor AP-2 beta
chr1	19378137	19378265	129	130	19378179	35	9.95877	3.31984	6.73562	Adipose_R2_peak_7	Distal Intergen	1	19213927	19238845	24919	1	21419	uc007aku.2	164210	ENSMUSG000	Tfap2b	transcription factor AP-2 beta
chr1	19382214	19382377	164	165	19382302	22	9.13959	4.13397	6.01526	Adipose_R1_peak_4	Distal Intergen	1	19213927	19238845	24919	1	21419	uc007aku.2	168287	ENSMUSG000	Tfap2b	transcription factor AP-2 beta
chr1	19382260	19382377	118	119	19382315	30	7.24477	2.85875	4.21917	Adipose_R2_peak_7	Distal Intergen	1	19213927	19238845	24919	1	21419	uc007aku.2	168333	ENSMUSG000	Tfap2b	transcription factor AP-2 beta
chr1	19399737	19400013	277	278	19399868	49	33.45876	8.98689	29.68925	Adipose_R1_peak_4	Distal Intergen	1	19213927	19238845	24919	1	21419	uc007aku.2	185810	ENSMUSG000	Tfap2b	transcription factor AP-2 beta
chr1	19399758	19400008	251	252	19399891	46	16.90986	4.33423	13.35898	Adipose_R2_peak_7	Distal Intergen	1	19213927	19238845	24919	1	21419	uc007aku.2	185831	ENSMUSG000	Tfap2b	transcription factor AP-2 beta
chr1	19434888	19435030	143	144	19435014	40	12.96465	3.78093	9.57654	Adipose_R2_peak_7	Distal Intergen	1	19213927	19238845	24919	1	21419	uc007aku.2	220961	ENSMUSG000	Tfap2b	transcription factor AP-2 beta
chr1	19434901	19435104	204	205	19434993	28	13.73721	5.2124	10.41463	Adipose_R1_peak_4	Distal Intergen	1	19213927	19238845	24919	1	21419	uc007aku.2	220974	ENSMUSG000	Tfap2b	transcription factor AP-2 beta
chr1	19439205	19439358	154	155	19439288	25	11.36566	4.67319	8.13482	Adipose_R1_peak_4	Distal Intergen	1	19213927	19238845	24919	1	21419	uc007aku.2	225278	ENSMUSG000	Tfap2b	transcription factor AP-2 beta
chr1	19439938	19440135	198	199	19440038	29	14.55748	5.39214	11.20652	Adipose_R1_peak_4	Distal Intergen	1	19213927	19238845	24919	1	21419	uc007aku.2	226011	ENSMUSG000	Tfap2b	transcription factor AP-2 beta
chr1	19444636	19444890	255	256	19444767	44	28.34544	8.08821	24.66239	Adipose_R1_peak_4	Distal Intergen	1	19213927	19238845	24919	1	21419	uc007aku.2	230709	ENSMUSG000	Tfap2b	transcription factor AP-2 beta
chr1	19444640	19444879	240	241	19444778	49	19.00719	4.61089	15.38598	Adipose_R2_peak_7	Distal Intergen	1	19213927	19238845	24919	1	21419	uc007aku.2	230713	ENSMUSG000	Tfap2b	transcription factor AP-2 beta
chr1	19447736	19447909	174	175	19447898	43	14.8939	4.05758	11.42017	Adipose_R2_peak_7	Distal Intergen	1	19213927	19238845	24919	1	21419	uc007aku.2	233809	ENSMUSG000	Tfap2b	transcription factor AP-2 beta
chr1	19447751	19447948	198	199	19447854	34	18.86162	6.29083	15.38482	Adipose_R1_peak_4	Distal Intergen	1	19213927	19238845	24919	1	21419	uc007aku.2	233824	ENSMUSG000	Tfap2b	transcription factor AP-2 beta
chr1	19689895	19690169	275	276	19690032	96	59.87514	8.94512	55.61917	Adipose_R2_peak_7	Distal Intergen	1	19213927	19238845	24919	1	21419	uc007aku.2	475968	ENSMUSG000	Tfap2b	transcription factor AP-2 beta
chr1	19689896	19690185	290	291	19690029	91	74.22908	13.4921	70.03769	Adipose_R1_peak_4	Distal Intergen	1	19213927	19238845	24919	1	21419	uc007aku.2	475969	ENSMUSG000	Tfap2b	transcription factor AP-2 beta
chr1	19799465	19799731	267	268	19799595	31	16.23981	5.75161	12.83549	Adipose_R1_peak_4	Distal Intergen	1	20356454	20392300	35847	1	75028	uc029qnd.1	-556723	ENSMUSG000	4930486I03Ri	RIKEN cDNA 4930486I03 gene
chr1	19839767	19839946	180	181	19839843	21	8.43286	3.95423	5.34896	Adipose_R1_peak_4	Distal Intergen	1	20356454	20392300	35847	1	75028	uc029qnd.1	-516508	ENSMUSG000	4930486I03Ri	RIKEN cDNA 4930486I03 gene
chr1	20023090	20023237	148	149	20023181	23	9.86443	4.31371	6.70247	Adipose_R1_peak_4	Distal Intergen	1	20356454	20392300	35847	1	75028	uc029qnd.1	-333217	ENSMUSG000	4930486I03Ri	RIKEN cDNA 4930486I03 gene
chr1	20023109	20023229	121	122	20023168	28	6.25023	2.67431	3.31626	Adipose_R2_peak_7	Distal Intergen	1	20356454	20392300	35847	1	75028	uc029qnd.1	-333225	ENSMUSG000	4930486I03Ri	RIKEN cDNA 4930486I03 gene
chr1	20042138	20042346	209	210	20042253	25	11.36566	4.67319	8.13482	Adipose_R1_peak_4	Distal Intergen	1	20356454	20392300	35847	1	75028	uc029qnd.1	-314108	ENSMUSG000	4930486I03Ri	RIKEN cDNA 4930486I03 gene
chr1	20096718	20096858	141	142	20096790	21	8.43286	3.95423	5.34896	Adipose_R1_peak_4	Intron (uc007a	1	20356454	20392300	35847	1	75028	uc029qnd.1	-259596	ENSMUSG000	4930486I03Ri	RIKEN cDNA 4930486I03 gene
chr1	20127072	20127165	94	95	20127105	27	5.77405	2.5821	2.88921	Adipose_R2_peak_7	Intron (uc007a	1	20356454	20392300	35847	1	75028	uc029qnd.1	-229289	ENSMUSG000	4930486I03Ri	RIKEN cDNA 4930486I03 gene
chr1	20128072	20128202	131	132	20128124	32	8.29282	3.04319	5.18352	Adipose_R2_peak_7	Intron (uc007a	1	20356454	20392300	35847	1	75028	uc029qnd.1	-228252	ENSMUSG000	4930486I03Ri	RIKEN cDNA 4930486I03 gene
chr1	20134530	20134626	97	98	20134575	27	5.77405	2.5821	2.88921	Adipose_R2_peak_7	Intron (uc007a	1	20356454	20392300	35847	1	75028	uc029qnd.1	-221828	ENSMUSG000	4930486I03Ri	RIKEN cDNA 4930486I03 gene
chr1	20166908	20167138	231	232	20167122	30	15.39182	5.57188	12.01394	Adipose_R1_peak_4	Intron (uc007a	1	20356454	20392300	35847	1	75028	uc029qnd.1	-189316	ENSMUSG000	4930486I03Ri	RIKEN cDNA 4930486I03 gene
chr1	20249359	20249695	337	338	20249499	89	64.45456	11.06999	60.33506	Adipose_R1_peak_4	Intron (uc007a	1	20356454	20392300	35847	1	75028	uc029qnd.1	-106759	ENSMUSG000	4930486I03Ri	RIKEN cDNA 4930486I03 gene
chr1	20249366	20249652	287	288	20249494	76	40.9047	7.10077	36.85923	Adipose_R2_peak_7	Intron (uc007a	1	20356454	20392300	35847	1	75028	uc029qnd.1	-106802	ENSMUSG000	4930486I03Ri	RIKEN cDNA 4930486I03 gene
chr1	20282342	20282552	211	212	20282469	26	11.91191	4.7607	8.67723	Adipose_R1_peak_4	Intron (uc007a	1	20356454	20392300	35847	1	75028	uc029qnd.1	-73902	ENSMUSG000	4930486I03Ri	RIKEN cDNA 4930486I03 gene
chr1	20282398	20282544	147	148	20282470	37	11.12817	3.50427	7.83613	Adipose_R2_peak_7	Intron (uc007a	1	20356454	20392300	35847	1	75028	uc029qnd.1	-73910	ENSMUSG000	4930486I03Ri	RIKEN cDNA 4930486I03 gene
chr1	20283119	20283398	280	281	20283254	65	51.17258	11.8627	47.17609	Adipose_R1_peak_4	Intron (uc007a	1	20356454	20392300	35847	1	75028	uc029qnd.1	-73056	ENSMUSG000	4930486I03Ri	RIKEN cDNA 4930486I03 gene
chr1	20283138	20283328	191	192	20283249	39	12.34187	3.68871	8.98501	Adipose_R2_peak_7	Intron (uc007a	1	20356454	20392300	35847	1	75028	uc029qnd.1	-73126	ENSMUSG000	4930486I03Ri	RIKEN cDNA 4930486I03 gene
chr1	20320261	20320491	231	232	20320352	26	12.14079	4.85292	8.87838	Adipose_R1_peak_4	Intron (uc007a	1	20356454	20392300	35847	1	75028	uc029qnd.1	-35963	ENSMUSG000	4930486I03Ri	RIKEN cDNA 4930486I03 gene
chr1	20487587	20487835	249	250	20487723	38	22.52591	7.00978	18.96	Adipose_R1_peak_4	Intron (uc007a	1	20057779	20618057	560279	2	241035	uc007akv.1	130222	ENSMUSG000	Pkhd1	polycystic kidney and hepatic disease 1
chr1	20487639	20487813	175	176	20487724	42	14.2409	3.96536	10.79479	Adipose_R2_peak_7	Intron (uc007a	1	20057779	20618057	560279	2	241035	uc007akv.1	130244	ENSMUSG000	Pkhd1	polycystic kidney and hepatic disease 1
chr1	20489628	20489856	229	230	20489729	29	14.55748	5.39214	11.20652	Adipose_R1_peak_4	Intron (uc007a	1	20057779	20618057	560279	2	241035	uc007akv.1	128201	ENSMUSG000	Pkhd1	polycystic kidney and hepatic disease 1
chr1	20491001	20491287	287	288	20491142	50	34.50784	9.16663	30.72138	Adipose_R1_peak_4	Intron (uc007a	1	20057779	20618057	560279	2	241035	uc007akv.1	126770	ENSMUSG000	Pkhd1	polycystic kidney and hepatic disease 1
chr1	20491093	20491235	143	144	20491153	33	8.83596	3.1354	5.68652	Adipose_R2_peak_7	Intron (uc007a	1	20057779	20618057	560279	2	241035	uc007akv.1	126822	ENSMUSG000	Pkhd1	polycystic kidney and hepatic disease 1
chr1	20576799	20577034	236	237	20576919	33	13.46149	4.4995	10.16491	Adipose_R1_peak_4	Intron (uc007a	1	20057779	20618057	560279	2	241035	uc007akv.1	41023	ENSMUSG000	Pkhd1	polycystic kidney and hepatic disease 1
chr1	20576834	20576977	144	145	20576911	35	9.95877	3.31984	6.73562	Adipose_R2_peak_7	Intron (uc007a	1	20057779	20618057	560279	2	241035	uc007akv.1	41080	ENSMUSG000	Pkhd1	polycystic kidney and hepatic disease 1
chr1	20580482	20580622	141	142	20580554	19	7.07687	3.59476	4.08458	Adipose_R1_peak_4	Intron (uc007a	1	20057779	20618057	560279	2	241035	uc007akv.1	37435	ENSMUSG000	Pkhd1	polycystic kidney and hepatic disease 1
chr1	20705547	20706192	646	647	20705788	38	22.52591	7.00978	18.96	Adipose_R1_peak_4	Distal Intergen	1	20730905	20734496	3592	1	16171	uc007aky.1	-24713	ENSMUSG000	Il17a	interleukin 17A
chr1	20705807	20706130	324	325	20706061	33	8.83596	3.1354	5.68652	Adipose_R2_peak_7	Distal Intergen	1	20730905	20734496	3592	1	16171	uc007aky.1	-24775	ENSMUSG000	Il17a	interleukin 17A
chr1	20708928	20709287	360	361	20709135	67	53.51345	12.22218	49.49424	Adipose_R1_peak_4	Distal Intergen	1	20730905	20734496	3592	1	16171	uc007aky.1	-21618	ENSMUSG000	Il17a	interleukin 17A
chr1	20708982	20709225	244	245	20709135	48	18.29934	4.51867	14.70076	Adipose_R2_peak_7	Distal Intergen	1	20730905	20734496	3592	1	16171	uc007aky.1	-21680	ENSMUSG000	Il17a	interleukin 17A
chr1	21136161	21136446	286	287	21136301	71	50.02111	10.19156	46.03708	Adipose_R1_peak_4	Distal Intergen	1	21001397	21079225	77829	2	170829	uc007alg.2	-56936	ENSMUSG000	Tram2	translocating chain-associating membrane protein 2

chr1	21136174	21136413	240	241	21136305	69	34.76717	6.45524	30.8116	Adipose_R2_peak_7	Distal Interger	1	21001397	21079225	77829	2	170829	uc007alg.2	-56949	ENSMUSG000	Tram2	translocating chain-associating membrane protein 2
chr1	21199957	21200206	250	251	21200084	41	25.38991	7.54899	21.76305	Adipose_R1_peak_4	Distal Interger	1	21218575	21230167	11593	1	75712	uc007ali.2	-18369	ENSMUSG000	Tmem14a	transmembrane protein 14A
chr1	21199995	21200134	140	141	21200065	35	9.95877	3.31984	6.73562	Adipose_R2_peak_7	Distal Interger	1	21218575	21230167	11593	1	75712	uc007ali.2	-18441	ENSMUSG000	Tmem14a	transmembrane protein 14A
chr1	21586157	21586388	232	233	21586249	39	23.47001	7.18952	19.88325	Adipose_R1_peak_4	Intron (uc007a	1	21383556	21386384	2829	1	98582	uc007alp.2	202601	ENSMUSG000	Khdc1b	KH domain containing 1B
chr1	21586189	21586364	176	177	21586252	44	15.55653	4.1498	12.05625	Adipose_R2_peak_7	Intron (uc007a	1	21383556	21386384	2829	1	98582	uc007alp.2	202633	ENSMUSG000	Khdc1b	KH domain containing 1B
chr1	21632749	21632906	158	159	21632803	48	18.29934	4.51867	14.70076	Adipose_R2_peak_7	Intron (uc007a	1	21383556	21386384	2829	1	98582	uc007alp.2	249193	ENSMUSG000	Khdc1b	KH domain containing 1B
chr1	21692782	21692962	181	182	21692865	26	12.14079	4.85292	8.87838	Adipose_R1_peak_4	Intron (uc007a	1	21398399	21962650	564252	2	226922	uc007alq.3	269688	ENSMUSG000	Kcnq5	potassium voltage-gated channel, subfamily Q, member 5
chr1	21779535	21779702	168	169	21779610	19	7.07687	3.59476	4.08458	Adipose_R1_peak_4	Intron (uc007a	1	21398399	21962650	564252	2	226922	uc007alq.3	182948	ENSMUSG000	Kcnq5	potassium voltage-gated channel, subfamily Q, member 5
chr1	21834042	21834288	247	248	21834133	19	7.07687	3.59476	4.08458	Adipose_R1_peak_4	Intron (uc007a	1	21398399	21962650	564252	2	226922	uc007alq.3	128362	ENSMUSG000	Kcnq5	potassium voltage-gated channel, subfamily Q, member 5
chr1	21996642	21996790	149	150	21996715	36	10.5378	3.41206	7.27949	Adipose_R2_peak_7	Distal Interger	1	21398399	21962650	564252	2	226922	uc007alq.3	-33992	ENSMUSG000	Kcnq5	potassium voltage-gated channel, subfamily Q, member 5
chr1	22012375	22012586	212	213	22012448	25	11.36566	4.67319	8.13482	Adipose_R1_peak_4	Distal Interger	1	21398399	21962650	564252	2	226922	uc007alq.3	-49725	ENSMUSG000	Kcnq5	potassium voltage-gated channel, subfamily Q, member 5
chr1	22060851	22061069	219	220	22060962	34	18.86162	6.29083	15.38482	Adipose_R1_peak_4	Distal Interger	1	21398399	21962650	564252	2	226922	uc007alq.3	-98201	ENSMUSG000	Kcnq5	potassium voltage-gated channel, subfamily Q, member 5
chr1	22084577	22084716	140	141	22084689	19	7.07687	3.59476	4.08458	Adipose_R1_peak_4	Distal Interger	1	21398399	21962650	564252	2	226922	uc007alq.3	-121927	ENSMUSG000	Kcnq5	potassium voltage-gated channel, subfamily Q, member 5
chr1	22096687	22096877	191	192	22096790	31	16.23981	5.75161	12.83549	Adipose_R1_peak_4	Distal Interger	1	21398399	21962650	564252	2	226922	uc007alq.3	-134037	ENSMUSG000	Kcnq5	potassium voltage-gated channel, subfamily Q, member 5
chr1	22096738	22096852	115	116	22096795	29	6.74062	2.76653	3.75989	Adipose_R2_peak_7	Distal Interger	1	21398399	21962650	564252	2	226922	uc007alq.3	-134088	ENSMUSG000	Kcnq5	potassium voltage-gated channel, subfamily Q, member 5
chr1	22098254	22098447	194	195	22098394	20	7.74501	3.7745	4.70467	Adipose_R1_peak_4	Distal Interger	1	21398399	21962650	564252	2	226922	uc007alq.3	-135604	ENSMUSG000	Kcnq5	potassium voltage-gated channel, subfamily Q, member 5
chr1	22154953	22155121	169	170	22155017	70	22.80721	4.09998	19.08197	Adipose_R2_peak_7	Distal Interger	1	22288422	22315843	27422	2	116837	uc007alr.1	160722	ENSMUSG000	Rims1	regulating synaptic membrane exocytosis 1
chr1	22162064	22162254	191	192	22162162	26	12.14079	4.85292	8.87838	Adipose_R1_peak_4	Distal Interger	1	22288422	22315843	27422	2	116837	uc007alr.1	153589	ENSMUSG000	Rims1	regulating synaptic membrane exocytosis 1
chr1	22186355	22186575	221	222	22186486	42	14.2409	3.96536	10.79479	Adipose_R2_peak_7	Distal Interger	1	22288422	22315843	27422	2	116837	uc007alr.1	129268	ENSMUSG000	Rims1	regulating synaptic membrane exocytosis 1
chr1	22186360	22186557	198	199	22186469	32	17.10103	5.93135	13.67137	Adipose_R1_peak_4	Distal Interger	1	22288422	22315843	27422	2	116837	uc007alr.1	129286	ENSMUSG000	Rims1	regulating synaptic membrane exocytosis 1
chr1	22230926	22231202	277	278	22231079	61	28.12311	5.7175	24.28203	Adipose_R2_peak_7	Distal Interger	1	22288422	22315843	27422	2	116837	uc007alr.1	84641	ENSMUSG000	Rims1	regulating synaptic membrane exocytosis 1
chr1	22230968	22231208	241	242	22231092	50	34.50784	9.16663	30.72138	Adipose_R1_peak_4	Distal Interger	1	22288422	22315843	27422	2	116837	uc007alr.1	84635	ENSMUSG000	Rims1	regulating synaptic membrane exocytosis 1
chr1	22233425	22233665	241	242	22233521	35	19.76029	6.47056	16.26028	Adipose_R1_peak_4	Distal Interger	1	22288422	22315843	27422	2	116837	uc007alr.1	82178	ENSMUSG000	Rims1	regulating synaptic membrane exocytosis 1
chr1	22233465	22233581	117	118	22233516	30	7.24477	2.85875	4.21917	Adipose_R2_peak_7	Distal Interger	1	22288422	22315843	27422	2	116837	uc007alr.1	82262	ENSMUSG000	Rims1	regulating synaptic membrane exocytosis 1
chr1	22234031	22234213	183	184	22234147	20	7.74501	3.7745	4.70467	Adipose_R1_peak_4	Distal Interger	1	22288422	22315843	27422	2	116837	uc007alr.1	81630	ENSMUSG000	Rims1	regulating synaptic membrane exocytosis 1
chr1	22234114	22234279	166	167	22234174	36	10.5378	3.41206	7.27949	Adipose_R2_peak_7	Distal Interger	1	22288422	22315843	27422	2	116837	uc007alr.1	81564	ENSMUSG000	Rims1	regulating synaptic membrane exocytosis 1
chr1	22236093	22236299	207	208	22236210	24	10.60668	4.49345	7.40961	Adipose_R1_peak_4	Distal Interger	1	22288422	22315843	27422	2	116837	uc007alr.1	79544	ENSMUSG000	Rims1	regulating synaptic membrane exocytosis 1
chr1	22258119	22258282	164	165	22258183	17	5.80341	3.23528	2.92378	Adipose_R1_peak_4	Distal Interger	1	22288422	22315843	27422	2	116837	uc007alr.1	57561	ENSMUSG000	Rims1	regulating synaptic membrane exocytosis 1
chr1	22815864	22816058	195	196	22815978	25	11.36566	4.67319	8.13482	Adipose_R1_peak_4	Distal Interger	1	22468191	22805724	337534	2	116837	uc029qnm.1	-10140	ENSMUSG000	Rims1	regulating synaptic membrane exocytosis 1
chr1	22815914	22816065	152	153	22816003	35	9.95877	3.31984	6.73562	Adipose_R2_peak_7	Distal Interger	1	22468191	22805724	337534	2	116837	uc029qnm.1	-10190	ENSMUSG000	Rims1	regulating synaptic membrane exocytosis 1
chr1	22895938	22896084	147	148	22895998	17	5.80341	3.23528	2.92378	Adipose_R1_peak_4	Distal Interger	1	22468191	22805724	337534	2	116837	uc029qnm.1	-90214	ENSMUSG000	Rims1	regulating synaptic membrane exocytosis 1
chr1	22920126	22920342	217	218	22920221	31	16.23981	5.75161	12.83549	Adipose_R1_peak_4	Distal Interger	1	22468191	22805724	337534	2	116837	uc029qnm.1	-114402	ENSMUSG000	Rims1	regulating synaptic membrane exocytosis 1
chr1	23099043	23099206	164	165	23099142	20	7.74501	3.7745	4.70467	Adipose_R1_peak_4	Downstream (	1	23100474	23102253	1780	2	66755	uc007alz.1	3047	ENSMUSG000	4933415F23Ri	RIKEN cDNA 4933415F23 gene
chr1	23104489	23104647	159	160	23104548	38	22.52591	7.00978	18.96	Adipose_R1_peak_4	Promoter (2-3	1	23100474	23102253	1780	2	66755	uc007alz.1	-2236	ENSMUSG000	4933415F23Ri	RIKEN cDNA 4933415F23 gene
chr1	23104501	23104634	134	135	23104545	34	9.39139	3.22762	6.20474	Adipose_R2_peak_7	Promoter (2-3	1	23100474	23102253	1780	2	66755	uc007alz.1	-2248	ENSMUSG000	4933415F23Ri	RIKEN cDNA 4933415F23 gene
chr1	23304370	23304511	142	143	23304443	38	11.72962	3.59649	8.4047	Adipose_R2_peak_7	Distal Interger	1	23291701	23291784	84	1	723964	uc007ama.2	12669	ENSMUSG000	Mir30c-2	microRNA 30c-2
chr1	23305292	23305633	342	343	23305502	69	34.76717	6.45524	30.8116	Adipose_R2_peak_7	Distal Interger	1	23291701	23291784	84	1	723964	uc007ama.2	13591	ENSMUSG000	Mir30c-2	microRNA 30c-2
chr1	23305313	23305638	326	327	23305502	62	42.31025	9.46672	38.41579	Adipose_R1_peak_4	Distal Interger	1	23291701	23291784	84	1	723964	uc007ama.2	13612	ENSMUSG000	Mir30c-2	microRNA 30c-2
chr1	23617324	23617503	180	181	23617406	36	20.67077	6.6503	17.14779	Adipose_R1_peak_4	Distal Interger	1	23761926	23815740	53815	1	280645	uc007amd.1	-144423	ENSMUSG000	B3gat2	beta-1,3-glucuronyltransferase 2 (glucuronosyltransferase S)
chr1	23617369	23617475	107	108	23617415	28	6.25023	2.67431	3.31626	Adipose_R2_peak_7	Distal Interger	1	23761926	23815740	53815	1	280645	uc007amd.1	-144451	ENSMUSG000	B3gat2	beta-1,3-glucuronyltransferase 2 (glucuronosyltransferase S)
chr1	23667337	23667686	350	351	23667507	160	168.1803	24.19272	163.6215	Adipose_R1_peak_4	Distal Interger	1	23761926	23815740	53815	1	280645	uc007amd.1	-94240	ENSMUSG000	B3gat2	beta-1,3-glucuronyltransferase 2 (glucuronosyltransferase S)
chr1	23667358	23667636	279	280	23667499	132	98.32313	12.26496	93.8217	Adipose_R2_peak_7	Distal Interger	1	23761926	23815740	53815	1	280645	uc007amd.1	-94290	ENSMUSG000	B3gat2	beta-1,3-glucuronyltransferase 2 (glucuronosyltransferase S)
chr1	23680318	23680496	179	180	23680381	20	7.74501	3.7745	4.70467	Adipose_R1_peak_4	Distal Interger	1	23761926	23815740	53815	1	280645	uc007amd.1	-81430	ENSMUSG000	B3gat2	beta-1,3-glucuronyltransferase 2 (glucuronosyltransferase S)
chr1	23698297	23698405	109	110	23698364	26	5.31252	2.48988	2.47999	Adipose_R2_peak_7	Distal Interger	1	23761926	23815740	53815	1	280645	uc007amd.1	-63521	ENSMUSG000	B3gat2	beta-1,3-glucuronyltransferase 2 (glucuronosyltransferase S)
chr1	24075722	24075938	217	218	24075854	25	11.36566	4.67319	8.13482	Adipose_R1_peak_4	Intron (uc007a	1	24010758	24100341	89584	2	68187	uc007aml.2	24403	ENSMUSG000	Fam135a	family with sequence similarity 135, member A
chr1	24080457	24080745	289	290	24080597	57	42.07807	10.4248	38.18469	Adipose_R1_peak_4	Intron (uc007a	1	24010758	24100341	89584	2	68187	uc007aml.2	19596	ENSMUSG000	Fam135a	family with sequence similarity 135, member A
chr1	24080458	24080738	281	282	24080579	65	31.39315	6.08637	27.49408	Adipose_R2_peak_7	Intron (uc007a	1	24010758	24100341	89584	2	68187	uc007aml.2	19603	ENSMUSG000	Fam135a	family with sequence similarity 135, member A
chr1	24464100	24464309	210	211	24464218	31	16.23981	5.75161	12.83549	Adipose_R1_peak_4	Intron (uc007a	1	24257683	24452745	195063	2	12823	uc011wir.1	-11355	ENSMUSG000	Col19a1	collagen, type XIX, alpha 1
chr1	24496824	24497072	249	250	24496939	34	18.86162	6.29083	15.38482	Adipose_R1_peak_4	Intron (uc007a	1	24257683	24452745	195063	2	12823	uc011wir.1	-44079	ENSMUSG000	Col19a1	collagen, type XIX, alpha 1
chr1	24496851	24497020	170	171	24496935	37	11.12817	3.50427	7.83613	Adipose_R2_peak_7	Intron (uc007a	1	24257683	24452745	195063	2	12823	uc011wir.1	-44106	ENSMUSG000	Col19a1	collagen, type XIX, alpha 1
chr1	24611606	24612081	476	477	24611882	7306	1327.327	2.93456	1321.611	Adipose_R2_peak_7	Distal Interger	1	24257683	24587437	329755	2	12823	uc007amq.1	-24169	ENSMUSG000	Col19a1	collagen, type XIX, alpha 1
chr1	24611620	24612050	431	432	24611886	3731	505.0634	2.47388	500.145	Adipose_R1_peak_4	Distal Interger	1	24257683	24587437	329755	2	12823	uc007amq.1	-24183	ENSMUSG000	Col19a1	collagen, type XIX, alpha 1
chr1	24612274	24612749	476	477	24612517	7360	938.6097	2.40255	933.2425	Adipose_R2_peak_7	Distal Interger	1	24257683	24587437	329755	2	12823	uc007amq.1	-24837	ENSMUSG000	Col19a1	collagen, type XIX, alpha 1
chr1	24612310	24612718	409	410	24612507	3899	496.1173	2.39695	491.2052	Adipose_R1_peak_4	Distal Interger	1	24257683	24587437	329755	2	12823	uc007amq.1	-24873	ENSMUSG000	Col19a1	collagen, type XIX, alpha 1
chr1	24612875	24615486	2612	2613	24613006	6559	719.2285	2.23448	714.0114	Adipose_R2_peak_7	Exon (uc007an	1	24257683	24587437	329755	2	12823	uc007amq.1	-25438	ENSMUSG000	Col19a1	collagen, type XIX, alpha 1
chr1	24612902	24614695	1794	1795	24613009	3111	252.355	1.96759	247.6497	Adipose_R1_peak_4	Exon (uc007an	1	24257683	24587437	329755	2	12823	uc007amq.1	-25465	ENSMUSG000	Col19a1	collagen, type XIX, alpha 1
chr1	24614869	24615417	549	550	24615113	4358	669.9269	2.6522	664.8868	Adipose_R1_peak_4	Distal Interger	1	24257683	24587437	329755	2	12823	uc007amq.1	-27432	ENSMUSG000	Col19a1	collagen, type XIX, alpha 1
chr1	24615625	24616145	521	522	24615926	7676	1632.855	3.2615	1626.81	Adipose_R2_peak_7	Distal Interger	1	24257683	24587437	329755	2	12823	uc007amq.1	-28188	ENSMUSG000	Col19a1	collagen, type XIX, alpha 1
chr1	24615656	24616143	488	489	24615934	4274	715.3386	2.78704	710.2551	Adipose_R1_peak_4	Distal Interger	1	24257683	24587437	329755	2	12823	uc007amq.1	-28219	ENSMUSG000	Col19a1	collagen, type XIX, alpha 1
chr1	24640808	24641237	430	431	24641164	21	8.43286	3.95423	5.34896	Adipose_R1_peak_4	Distal Interger	1	24678544	24766301	87758	1	68421	uc007amt.1	-37307	ENSMUSG000	Lmbrd1	LMBR1 domain containing 1
chr1	24684023	24684254	232	233	24684157	91	54.95	8.48403	50.74234	Adipose_R2_peak_7	Intron (uc007a	1	24678918	24766301	87384	1	68421	uc007amu.2	5105	ENSMUSG000	Lmbrd1	LMBR1 domain containing 1
chr1	24684106	24684262	157	158	24684173	50	16.89818	4.06191	13.49049	Adipose_R1_peak_4	Intron (uc007a	1	24678918	24766301	87384	1	68421	uc007amu.2	5188	ENSMUSG000	Lmbrd1	LMBR1 domain containing 1
chr1	24722471	24722621	151	152	24722560	23	9.86443	4.31371	6.70247	Adipose_R1_peak_5	Intron (uc007a	1	24678918	24766301	87384	1	68421	uc007amu.2	43553	ENSMUSG000	Lmbrd1	LMBR1 domain containing 1
chr1	24838545	24838803	259	260	24838657	32	17.10103	5.93135	13.67137	Adipose_R1_peak_5	Distal Interger	1	24678918	24766301	87384	1	68421	uc007amu.2	159627	ENSMUSG000	Lmbrd1	LMBR1 domain containing 1
chr1	24908836	24909138	303	304	24908974	90	53.97882	8.39181	49.78112	Adipose_R2_peak_7	Distal Interger	1	24678918	24766301	87384	1	68421	uc007amu.2	229918	ENSMUSG000	Lmbrd1	LMBR1 domain containing 1
chr1	24908839	24909131	293	294	24908974	87	73.18137	14.091	68.9942	Adipose_R1_peak_5	Distal Interger	1	24678918	24766301	87384	1	68421	uc007amu.2	229921	ENSMUSG000	Lmbrd1	LMBR1 domain containing 1
chr1	25125388	25125572	185	186	25125484	20	7.74501	3.7745	4.70467	Adipose_R1_peak_5	Intron (uc007a	1	25067476	25229100	161625	2	210933	uc007amv.2	103528	ENSMUSG000	Adgrb3	adhesion G protein-coupled receptor B3
chr1	25182975	25183159	185	186	25183092	22	9.13959	4.13397	6.01526	Adipose_R1_peak_5	Intron (uc007a	1	25067476	25229100	161625	2	210933	uc007amv.2	45941	ENSMUSG000	Adgrb3	adhesion G protein-coupled receptor B3
chr1	25603545	25603814	270	271	25603671	48	32.41826	8.80716	28.66523	Adipose_R1_peak_5	Intron (uc007a	1	25067476	25826775	759300	2	210933	uc007amw.2	222961	ENSMUSG000	Adgrb3	adhesion G protein-coupled receptor B3
chr1	25603586	25603765	180	181	25603676	40	12.96465	3.78093	9.57654	Adipose_R2_peak_7	Intron (uc007a	1	25067476	25826775	759300	2	210933	uc007amw.2	223010	ENSMUSG000	Adgrb3	adhesion G protein-coupled receptor B3
chr1	25611412	25611556	145	146	25611520	20	7.74501	3.7745	4.70467	Adipose_R1_peak_5	Intron (uc007a	1	25067476	25826775	759300	2	210933	uc007amw.2	215219	ENSMUSG000	Adgrb3	adhesion G protein-coupled receptor B3
chr1	25668340	25668539	200	201	25668424	25	11.36566	4.67319	8.13482	Adipose_R1_peak_5	Intron (uc007a	1	25067476	25826775	759300	2	210933	uc007amw.2	158236	ENSMUSG000	Adgrb3	adhesion G protein-coupled receptor B3
chr1	25668390	25668493	104	105	25668434	27	5.77405	2.5821	2.88921	Adipose_R2_peak_7	Intron (uc007a	1	25067476	25826775	759300	2	210933	uc007amw.2	158282	ENSMUSG000	Adgrb3	adhesion G protein-coupled receptor B3
chr1	25668999	25669260	262	263	25669114	44	28.34544	8.08821	24.66239	Adipose_R1_peak_5	Intron (uc007a	1	25067476	25826775	759300	2	210933	uc007amw.2	157515	ENSMUSG000	Adgrb3	adhesion G protein-coupled receptor B3
chr1	25669017	25669213	197	198	25669112	45	16.22858	4.24202	12.70278	Adipose_R2_peak_7	Intron (uc007a	1	25067476	25826775	759300	2	210933	uc007amw.2	157562	ENSMUSG000	Adgrb3	adhesion G protein-coupled receptor B3
chr1	26211639	26211803	165	166	26211715	23	9.86443	4.31371	6.70247	Adipose_R1_peak_5	Distal Interger	1	25067476	25829707	762232	2	210933	uc011wis.1	-381932	ENSMUSG000	Adgrb3	adhesion G protein-coupled receptor B3
chr1	26352697	26352919	223	224	26352812	35	9.95877	3.31984	6.73562	Adipose_R2_peak_8	Distal Interger	1	26681801	26687460	5660	2	210940	uc007amz.2	334541	ENSMUSG000	4931408C20Ri	RIKEN cDNA 4931408C20 gene
chr1	26562897	26563046	150	151	26562961	19	7.07687	3.59476	4.08458	Adipose_R1_peak_5	Distal Interger	1	26681801	26687460	5660	2	210940	uc007amz.2	124414	ENSMUSG000	4931408C20Ri	RIKEN cDNA 4931408C20 gene
chr1	26587844	26588025	182	183	26587930	24	10.60668	4.49345	7.40961	Adipose_R1_peak_5	Distal Interger	1	26681801	26687460	5660	2	210940	uc007amz.2	99435	ENSMUSG000	4931408C20Ri	RIKEN cDNA 4931408C20 gene
chr1	26667319	26667589	271	272	26667455	79	43.62032	7.37742	39.5398	Adipose_R2_peak_8	Distal Interger	1	26681801	26687460	5660	2	210940	uc007amz.2	19871	ENSMUSG000	4931408C20Ri	RIKEN cDNA 4931408C20 gene
chr1	26667321	26667589	269	270	26667440	72	59.47482	13.12087	55.39848	Adipose_R1_peak_5	Distal Interger	1	26681801	26687460	5660	2	210940	uc007amz.2	19871	ENSMUSG000	4931408C20Ri	RIKEN cDNA 4931408C20 gene
chr1	26686118	26686223	106	107	26686167	32	8.29282	3.04319	5.18352	Adipose_R2_peak_8	Promoter (1-2	1	26681801	26687460	5660	2	210940	uc007amz.2	1237	ENSMUSG000	4931408C20Ri	RIKEN cDNA 4931408C20 gene
chr1	26686429	26686637	209	210	26686534	56	12.75022	3.00592	9.38183	Adipose_R2_peak_8	Promoter (<=:	1	26681801	26687460	5660	2	210940	uc007amz.2	823	ENSMUSG000	4931408C20Ri	RIKEN cDNA 4931408C20 gene
chr1	26686948	26687725	778	779	26686993	44	4.91194	1.95006	2.13435	Adipose_R2_peak_8	Promoter (<=:	1	26681801	26687460	5660	2	210940	uc007amz.2	0	ENSMUSG000	4931408C20Ri	RIKEN cDNA 4931408C20 gene
chr1	26687896	26688336	441	442	26687940	47	6.333	2.1514	3.3959	Adipose_R2_peak_8	Promoter (<=:	1	26681801	26687460	5660	2	210940	uc007amz.2	-436	ENSMUSG000	4931408C20Ri	RIKEN cDNA 4931408C20 gene
chr1	26803373	26803549	177	178	26803452	42	14.2409	3.96536	10.79479	Adipose_R2_peak_8	Distal Interger	1	26681801	26687460	5660	2	210940	uc007amz.2	-115913	ENSMUSG000	4931408C20Ri	RIKEN cDNA 4931408C20 gene
chr1	26906834	26907203	370	371	26906901	19	6.57303	3.37992	3.63151	Adipose_R1_peak_5	Distal Interger	1	26681801	26687460	5660	2	210940	uc007amz.2	-219374	ENSMUSG000	4931408C20Ri	RIKEN cDNA 4931408C20 gene
chr1	26941309	26941486	178	179	26941463	24	10.60668	4.49345	7.40961	Adipose_R1_peak_5	Distal Interger	1	26681801	26687460	5660	2	210940	uc007amz.2	-253849	ENSMUSG000	4931408C20Ri	RIKEN cDNA 4931408C20 gene
chr1	26941318	26941486	169	170	26941479	38	11.72962	3.59649	8.4047	Adipose_R2_peak_8	Distal Interger	1	26681801	26687460	5660	2	210940	uc007amz.2	-253858	ENSMUSG000	4931408C20Ri	RIKEN cDNA 4931408C20 gene
chr1	27027505	27027639	135	136	27027613	19	7.07687	3.59476	4.08458	Adipose_R1_peak_5	Distal Interger	1	26681801	26687460	5660	2	210940	uc007amz.2	-340045	ENSMUSG000	4931408C20Ri	RIKEN cDNA 4931408C20 gene
chr1	27051237	27051472	236	237	27051352	49	33.45876	8.98689	29.68925	Adipose_R1_peak_5	Distal Interger	1	26681801	26687460	5660	2	210940	uc007amz.2	-363777	ENSMUSG000	4931408C20Ri	RIKEN cDNA 4931408C20 gene
chr1	27051264	27051422	159	160	27051341	37	11.12817	3.50427	7.83613	Adipose_R2_peak_8	Distal Interger	1	26681801	26687460	5660	2	210940	uc007amz.2	-363804	ENSMUSG000	4931408C20Ri	RIKEN cDNA 4931408C20 gene
chr1	27145436	27145636	201	202	27145538	27	12.93148	5.03266	9.63827	Adipose_R1_peak_5	Distal Interger	1	26681801	26687460	5660	2	210940	uc007amz.2	-457976	ENSMUSG000	4931408C20Ri	RIKEN cDNA 4931408C20 gene
chr1	27161078	27161221	144	145	27161137	17	5.80341	3.23528	2.92378	Adipose_R1_peak_5	Distal Interger	1	26681801	26687460	5660	2	210940	uc007amz.2	-473618	ENSMUSG000	4931408C20Ri	RIKEN cDNA 4931408C20 gene

chr1	27183305	27183465	161	162	27183390	23	9.86443	4.31371	6.70247	Adipose_R1_peak_	Distal Interger	1	26681801	26687460	5660	2	210940	uc007amz.2	-495845	ENSMUSG000	4931408C20R	RIKEN cDNA 4931408C20 gene
chr1	27196516	27196646	131	132	27196574	20	7.74501	3.7745	4.70467	Adipose_R1_peak_	Distal Interger	1	26681801	26687460	5660	2	210940	uc007amz.2	-509056	ENSMUSG000	4931408C20R	RIKEN cDNA 4931408C20 gene
chr1	27196517	27196646	130	131	27196560	31	7.7623	2.95097	4.69388	Adipose_R2_peak_	Distal Interger	1	26681801	26687460	5660	2	210940	uc007amz.2	-509057	ENSMUSG000	4931408C20R	RIKEN cDNA 4931408C20 gene
chr1	27202580	27202777	198	199	27202687	25	11.36566	4.67319	8.13482	Adipose_R1_peak_	Distal Interger	1	26681801	26687460	5660	2	210940	uc007amz.2	-515120	ENSMUSG000	4931408C20R	RIKEN cDNA 4931408C20 gene
chr1	27273658	27274017	360	361	27273862	202	202.4776	22.68249	197.8445	Adipose_R1_peak_	Distal Interger	1	26681801	26687460	5660	2	210940	uc007amz.2	-586198	ENSMUSG000	4931408C20R	RIKEN cDNA 4931408C20 gene
chr1	27273665	27274007	343	344	27273861	163	134.8857	15.12371	130.2675	Adipose_R2_peak_	Distal Interger	1	26681801	26687460	5660	2	210940	uc007amz.2	-586205	ENSMUSG000	4931408C20R	RIKEN cDNA 4931408C20 gene
chr1	27293770	27293901	132	133	27293810	27	5.77405	2.5821	2.88921	Adipose_R2_peak_	Distal Interger	1	26681801	26687460	5660	2	210940	uc007amz.2	-606310	ENSMUSG000	4931408C20R	RIKEN cDNA 4931408C20 gene
chr1	27742540	27742713	174	175	27742617	24	10.60668	4.49345	7.40961	Adipose_R1_peak_	Distal Interger	1	28776122	28780252	4131	2	210962	uc007anb.1	1037539	ENSMUSG000	Gm597	predicted gene 597
chr1	27742548	27742684	137	138	27742596	32	8.29282	3.04319	5.18352	Adipose_R2_peak_	Distal Interger	1	28776122	28780252	4131	2	210962	uc007anb.1	1037568	ENSMUSG000	Gm597	predicted gene 597
chr1	27755862	27756053	192	193	27755972	22	9.13959	4.13397	6.01526	Adipose_R1_peak_	Distal Interger	1	28776122	28780252	4131	2	210962	uc007anb.1	1024199	ENSMUSG000	Gm597	predicted gene 597
chr1	27856490	27856659	170	171	27856585	21	8.43286	3.95423	5.34896	Adipose_R1_peak_	Distal Interger	1	28776122	28780252	4131	2	210962	uc007anb.1	923593	ENSMUSG000	Gm597	predicted gene 597
chr1	27856536	27856657	122	123	27856598	34	9.39139	3.22762	6.20474	Adipose_R2_peak_	Distal Interger	1	28776122	28780252	4131	2	210962	uc007anb.1	923595	ENSMUSG000	Gm597	predicted gene 597
chr1	28029912	28030027	116	117	28029972	30	7.24477	2.85875	4.21917	Adipose_R2_peak_	Distal Interger	1	28776122	28780252	4131	2	210962	uc007anb.1	750225	ENSMUSG000	Gm597	predicted gene 597
chr1	28293807	28293949	143	144	28293886	20	7.74501	3.7745	4.70467	Adipose_R1_peak_	Distal Interger	1	28776122	28780252	4131	2	210962	uc007anb.1	486303	ENSMUSG000	Gm597	predicted gene 597
chr1	28293827	28293948	122	123	28293898	32	8.29282	3.04319	5.18352	Adipose_R2_peak_	Distal Interger	1	28776122	28780252	4131	2	210962	uc007anb.1	486304	ENSMUSG000	Gm597	predicted gene 597
chr1	28579174	28579795	622	623	28579246	22	9.13959	4.13397	6.01526	Adipose_R1_peak_	Distal Interger	1	28776122	28780252	4131	2	210962	uc007anb.1	200457	ENSMUSG000	Gm597	predicted gene 597
chr1	28619141	28619280	140	141	28619210	24	10.60668	4.49345	7.40961	Adipose_R1_peak_	Distal Interger	1	28776122	28780252	4131	2	210962	uc007anb.1	160972	ENSMUSG000	Gm597	predicted gene 597
chr1	28855597	28856101	505	506	28855661	22	9.13959	4.13397	6.01526	Adipose_R1_peak_	Distal Interger	1	28776122	28780252	4131	2	210962	uc007anb.1	-75345	ENSMUSG000	Gm597	predicted gene 597
chr1	28855619	28855848	230	231	28855654	27	5.77405	2.5821	2.88921	Adipose_R2_peak_	Distal Interger	1	28776122	28780252	4131	2	210962	uc007anb.1	-75367	ENSMUSG000	Gm597	predicted gene 597
chr1	29246186	29246394	209	210	29246282	40	24.42476	7.36925	20.81738	Adipose_R1_peak_	Distal Interger	1	29421488	29421612	125	1	1.02E+08	uc033fii.1	-175094	ENSMUSG000	Mir6342	microRNA 6342
chr1	29246221	29246374	154	155	29246300	31	7.7623	2.95097	4.69388	Adipose_R2_peak_	Distal Interger	1	29421488	29421612	125	1	1.02E+08	uc033fii.1	-175114	ENSMUSG000	Mir6342	microRNA 6342
chr1	29369363	29369615	253	254	29369471	51	35.56531	9.34637	31.76235	Adipose_R1_peak_	Distal Interger	1	29421488	29421612	125	1	1.02E+08	uc033fii.1	-51873	ENSMUSG000	Mir6342	microRNA 6342
chr1	29370635	29371023	389	390	29370778	83	63.8275	12.0297	59.71627	Adipose_R1_peak_	Distal Interger	1	29421488	29421612	125	1	1.02E+08	uc033fii.1	-50465	ENSMUSG000	Mir6342	microRNA 6342
chr1	29370652	29370986	335	336	29370778	54	22.67101	5.07198	18.94758	Adipose_R2_peak_	Distal Interger	1	29421488	29421612	125	1	1.02E+08	uc033fii.1	-50502	ENSMUSG000	Mir6342	microRNA 6342
chr1	29372033	29372350	318	319	29372170	54	38.78658	9.88558	34.93639	Adipose_R1_peak_	Distal Interger	1	29421488	29421612	125	1	1.02E+08	uc033fii.1	-49138	ENSMUSG000	Mir6342	microRNA 6342
chr1	29372139	29372281	143	144	29372204	33	8.83596	3.1354	5.68652	Adipose_R2_peak_	Distal Interger	1	29421488	29421612	125	1	1.02E+08	uc033fii.1	-49207	ENSMUSG000	Mir6342	microRNA 6342
chr1	29474621	29474959	339	340	29474799	31	16.23981	5.75161	12.83549	Adipose_R1_peak_	Distal Interger	1	29421488	29421612	125	1	1.02E+08	uc033fii.1	53133	ENSMUSG000	Mir6342	microRNA 6342
chr1	29583152	29583341	190	191	29583236	24	10.60668	4.49345	7.40961	Adipose_R1_peak_	Distal Interger	1	29421488	29421612	125	1	1.02E+08	uc033fii.1	161664	ENSMUSG000	Mir6342	microRNA 6342
chr1	29583656	29583987	332	333	29583843	82	71.83084	14.91825	67.65268	Adipose_R1_peak_	Distal Interger	1	29421488	29421612	125	1	1.02E+08	uc033fii.1	162168	ENSMUSG000	Mir6342	microRNA 6342
chr1	29583713	29583932	220	221	29583848	44	15.55653	4.1498	12.05625	Adipose_R2_peak_	Distal Interger	1	29421488	29421612	125	1	1.02E+08	uc033fii.1	162225	ENSMUSG000	Mir6342	microRNA 6342
chr1	29611930	29612132	203	204	29612035	46	16.90986	4.33423	13.35898	Adipose_R2_peak_	Distal Interger	1	29421488	29421612	125	1	1.02E+08	uc033fii.1	190442	ENSMUSG000	Mir6342	microRNA 6342
chr1	29615077	29615211	135	136	29615146	22	9.13959	4.13397	6.01526	Adipose_R1_peak_	Distal Interger	1	29421488	29421612	125	1	1.02E+08	uc033fii.1	193589	ENSMUSG000	Mir6342	microRNA 6342
chr1	29647898	29648266	369	370	29648057	66	21.84026	4.13065	18.14588	Adipose_R2_peak_	Distal Interger	1	29421488	29421612	125	1	1.02E+08	uc033fii.1	226410	ENSMUSG000	Mir6342	microRNA 6342
chr1	29648004	29648199	196	197	29648053	33	6.32349	2.49613	3.43577	Adipose_R1_peak_	Distal Interger	1	29421488	29421612	125	1	1.02E+08	uc033fii.1	226516	ENSMUSG000	Mir6342	microRNA 6342
chr1	29657637	29657819	183	184	29657760	18	6.42934	3.41502	3.48999	Adipose_R1_peak_	Distal Interger	1	29421488	29421612	125	1	1.02E+08	uc033fii.1	236149	ENSMUSG000	Mir6342	microRNA 6342
chr1	29700031	29700287	257	258	29700164	42	26.36523	7.72873	22.71906	Adipose_R1_peak_	Distal Interger	1	29421488	29421612	125	1	1.02E+08	uc033fii.1	278543	ENSMUSG000	Mir6342	microRNA 6342
chr1	29700047	29700279	233	234	29700169	47	17.60017	4.42645	14.02531	Adipose_R2_peak_	Distal Interger	1	29421488	29421612	125	1	1.02E+08	uc033fii.1	278559	ENSMUSG000	Mir6342	microRNA 6342
chr1	29740690	29740874	185	186	29740757	38	11.72962	3.59649	8.4047	Adipose_R2_peak_	Distal Interger	1	29421488	29421612	125	1	1.02E+08	uc033fii.1	319202	ENSMUSG000	Mir6342	microRNA 6342
chr1	29740693	29740893	201	202	29740774	31	16.23981	5.75161	12.83549	Adipose_R1_peak_	Distal Interger	1	29421488	29421612	125	1	1.02E+08	uc033fii.1	319205	ENSMUSG000	Mir6342	microRNA 6342
chr1	29843045	29843267	223	224	29843151	33	17.97508	6.11109	14.52164	Adipose_R1_peak_	Distal Interger	1	29421488	29421612	125	1	1.02E+08	uc033fii.1	421557	ENSMUSG000	Mir6342	microRNA 6342
chr1	29843062	29843230	169	170	29843126	32	8.29282	3.04319	5.18352	Adipose_R2_peak_	Distal Interger	1	29421488	29421612	125	1	1.02E+08	uc033fii.1	421574	ENSMUSG000	Mir6342	microRNA 6342
chr1	29894836	29894959	124	125	29894944	26	5.31252	2.48988	2.47999	Adipose_R2_peak_	Distal Interger	1	29421488	29421612	125	1	1.02E+08	uc033fii.1	473348	ENSMUSG000	Mir6342	microRNA 6342
chr1	30070702	30070889	188	189	30070772	23	9.86443	4.31371	6.70247	Adipose_R1_peak_	Distal Interger	1	29421488	29421612	125	1	1.02E+08	uc033fii.1	649214	ENSMUSG000	Mir6342	microRNA 6342
chr1	30160198	30160492	295	296	30160363	67	53.51345	12.22218	49.49424	Adipose_R1_peak_	Distal Interger	1	30802342	30863256	60915	2	213109	uc007ane.1	702764	ENSMUSG000	Phf3	PHD finger protein 3
chr1	30160219	30160488	270	271	30160366	66	32.22715	6.17859	28.31407	Adipose_R2_peak_	Distal Interger	1	30802342	30863256	60915	2	213109	uc007ane.1	702768	ENSMUSG000	Phf3	PHD finger protein 3
chr1	30162984	30163164	181	182	30163092	33	17.97508	6.11109	14.52164	Adipose_R1_peak_	Distal Interger	1	30802342	30863256	60915	2	213109	uc007ane.1	700092	ENSMUSG000	Phf3	PHD finger protein 3
chr1	30163713	30163930	218	219	30163834	43	14.8939	4.05758	11.42017	Adipose_R2_peak_	Distal Interger	1	30802342	30863256	60915	2	213109	uc007ane.1	699326	ENSMUSG000	Phf3	PHD finger protein 3
chr1	30163715	30163975	261	262	30163838	62	47.71039	11.32349	43.751	Adipose_R1_peak_	Distal Interger	1	30802342	30863256	60915	2	213109	uc007ane.1	699281	ENSMUSG000	Phf3	PHD finger protein 3
chr1	30205637	30205753	117	118	30205672	26	5.31252	2.48988	2.47999	Adipose_R2_peak_	Distal Interger	1	30802342	30863256	60915	2	213109	uc007ane.1	657503	ENSMUSG000	Phf3	PHD finger protein 3
chr1	30266973	30267081	109	110	30267031	31	7.7623	2.95097	4.69388	Adipose_R2_peak_	Distal Interger	1	30802342	30863256	60915	2	213109	uc007ane.1	596175	ENSMUSG000	Phf3	PHD finger protein 3
chr1	30296555	30296712	158	159	30296634	19	7.07687	3.59476	4.08458	Adipose_R1_peak_	Distal Interger	1	30802342	30863256	60915	2	213109	uc007ane.1	566544	ENSMUSG000	Phf3	PHD finger protein 3
chr1	30308855	30309123	269	270	30309000	38	22.52591	7.00978	18.96	Adipose_R1_peak_	Distal Interger	1	30802342	30863256	60915	2	213109	uc007ane.1	554133	ENSMUSG000	Phf3	PHD finger protein 3
chr1	30335148	30335284	137	138	30335223	23	7.85017	3.51968	4.80878	Adipose_R1_peak_	Distal Interger	1	30802342	30863256	60915	2	213109	uc007ane.1	527972	ENSMUSG000	Phf3	PHD finger protein 3
chr1	30611233	30611385	153	154	30611306	24	10.60668	4.49345	7.40961	Adipose_R1_peak_	Distal Interger	1	30802342	30863256	60915	2	213109	uc007ane.1	251871	ENSMUSG000	Phf3	PHD finger protein 3
chr1	30613038	30613203	166	167	30613126	33	8.83596	3.1354	5.68652	Adipose_R2_peak_	Distal Interger	1	30802342	30863256	60915	2	213109	uc007ane.1	250053	ENSMUSG000	Phf3	PHD finger protein 3
chr1	30661161	30661428	268	269	30661303	50	33.00508	8.6188	29.24943	Adipose_R1_peak_	Distal Interger	1	30802342	30863256	60915	2	213109	uc007ane.1	201828	ENSMUSG000	Phf3	PHD finger protein 3
chr1	30661212	30661419	208	209	30661305	54	22.67101	5.07198	18.94758	Adipose_R2_peak_	Distal Interger	1	30802342	30863256	60915	2	213109	uc007ane.1	201837	ENSMUSG000	Phf3	PHD finger protein 3
chr1	30733090	30733414	325	326	30733252	88	79.49821	15.99667	75.27097	Adipose_R1_peak_	Distal Interger	1	30802342	30863256	60915	2	213109	uc007ane.1	129842	ENSMUSG000	Phf3	PHD finger protein 3
chr1	30733115	30733381	267	268	30733239	75	40.01057	7.00855	35.97731	Adipose_R2_peak_	Distal Interger	1	30802342	30863256	60915	2	213109	uc007ane.1	129875	ENSMUSG000	Phf3	PHD finger protein 3
chr1	30734678	30735337	660	661	30734818	103	94.7774	17.10186	90.46938	Adipose_R1_peak_	Distal Interger	1	30802342	30863256	60915	2	213109	uc007ane.1	127919	ENSMUSG000	Phf3	PHD finger protein 3
chr1	30734685	30734942	258	259	30734816	66	32.22715	6.17859	28.31407	Adipose_R2_peak_	Distal Interger	1	30802342	30863256	60915	2	213109	uc007ane.1	128314	ENSMUSG000	Phf3	PHD finger protein 3
chr1	30855639	30855832	194	195	30855727	25	11.36566	4.67319	8.13482	Adipose_R1_peak_	Intron (uc007a	1	30802342	30863256	60915	2	213109	uc007ane.1	7424	ENSMUSG000	Phf3	PHD finger protein 3
chr1	30859206	30859429	224	225	30859279	43	27.35048	7.90847	23.68561	Adipose_R1_peak_	Intron (uc007a	1	30802342	30863256	60915	2	213109	uc007ane.1	3827	ENSMUSG000	Phf3	PHD finger protein 3
chr1	30859234	30859389	156	157	30859295	35	9.95877	3.31984	6.73562	Adipose_R2_peak_	Intron (uc007a	1	30802342	30863256	60915	2	213109	uc007ane.1	3867	ENSMUSG000	Phf3	PHD finger protein 3
chr1	31129772	31130148	377	378	31129970	327	323.4882	23.24131	318.7126	Adipose_R1_peak_	Distal Interger	1	31176435	31204725	28291	1	266744	uc007ank.1	-46287	ENSMUSG000	Lgsn	lengsin, lens protein with glutamine synthetase domain
chr1	31129811	31130124	314	315	31129965	269	243.1332	18.88039	238.3322	Adipose_R2_peak_	Distal Interger	1	31176435	31204725	28291	1	266744	uc007ank.1	-46311	ENSMUSG000	Lgsn	lengsin, lens protein with glutamine synthetase domain
chr1	31281068	31281182	115	116	31281128	28	6.25023	2.67431	3.31626	Adipose_R2_peak_	Distal Interger	1	31264847	31268061	3215	1	226957	uc011wiu.1	16221	NA	Gm4850	THO complex 4 pseudogene
chr1	31296164	31296323	160	161	31296225	20	7.74501	3.7745	4.70467	Adipose_R1_peak_	Distal Interger	1	31264847	31268061	3215	1	226957	uc011wiu.1	31317	NA	Gm4850	THO complex 4 pseudogene
chr1	31385711	31385976	266	267	31385843	52	36.63102	9.52611	32.81188	Adipose_R1_peak_	Distal Interger	1	31264847	31268061	3215	1	226957	uc011wiu.1	120864	NA	Gm4850	THO complex 4 pseudogene
chr1	31385756	31385963	208	209	31385850	44	15.55653	4.1498	12.05625	Adipose_R2_peak_	Distal Interger	1	31264847	31268061	3215	1	226957	uc011wiu.1	120909	NA	Gm4850	THO complex 4 pseudogene
chr1	31386909	31387071	163	164	31386994	22	9.13959	4.13397	6.01526	Adipose_R1_peak_	Distal Interger	1	31264847	31268061	3215	1	226957	uc011wiu.1	122062	NA	Gm4850	THO complex 4 pseudogene
chr1	31414642	31414811	170	171	31414731	26	12.14079	4.85292	8.87838	Adipose_R1_peak_	Distal Interger	1	31264847	31268061	3215	1	226957	uc011wiu.1	149795	NA	Gm4850	THO complex 4 pseudogene
chr1	31472856	31473078	223	224	31472972	32	17.10103	5.93135	13.67137	Adipose_R1_peak_	Distal Interger	1	31264847	31268061	3215	1	226957	uc011wiu.1	208009	NA	Gm4850	THO complex 4 pseudogene
chr1	31814788	31815044	257	258	31814930	25	11.36566	4.67319	8.13482	Adipose_R1_peak_	Distal Interger	1	32172806	32246872	74067	1	170771	uc007ann.1	-357762	ENSMUSG000	Khdrbs2	KH domain containing, RNA binding, signal transduction associated 2
chr1	31958804	31958958	155	156	31958916	28	6.25023	2.67431	3.31626	Adipose_R2_peak_	Distal Interger	1	32172806	32246872	74067	1	170771	uc007ann.1	-213848	ENSMUSG000	Khdrbs2	KH domain containing, RNA binding, signal transduction associated 2
chr1	32191668	32191828	161	162	32191761	22	9.13959	4.13397	6.01526	Adipose_R1_peak_	Intron (uc007a	1	32172806	32246872	74067	1	170771	uc007ann.1	18862	ENSMUSG000	Khdrbs2	KH domain containing, RNA binding, signal transduction associated 2
chr1	32262872	32263139	268	269	32263005	50	34.50784	9.16663	30.72138	Adipose_R1_peak_	Intron (uc007a	1	32172806	32246872	74067	1	170771	uc007ann.1	90066	ENSMUSG000	Khdrbs2	KH domain containing, RNA binding, signal transduction associated 2
chr1	32262893	32263080	188	189	32262991	34	9.39139	3.22762	6.20474	Adipose_R2_peak_	Intron (uc007a	1	32172806	32246872	74067	1	170771	uc007ann.1	90087	ENSMUSG000	Khdrbs2	KH domain containing, RNA binding, signal transduction associated 2
chr1	32263608	32263907	300	301	32263785	42	26.36523	7.72873	22.71906	Adipose_R1_peak_	Intron (uc007a	1	32172806	32246872	74067	1	170771	uc007ann.1	90802	ENSMUSG000	Khdrbs2	KH domain containing, RNA binding, signal transduction associated 2
chr1	32263627	32263897	271	272	32263788	47	17.60017	4.42645	14.02531	Adipose_R2_peak_	Intron (uc007a	1	32172806	32246872	74067	1	170771	uc007ann.1	90821	ENSMUSG000	Khdrbs2	KH domain containing, RNA binding, signal transduction associated 2
chr1	32376548	32376761	214	215	32376668	30	15.39182	5.57188	12.01394	Adipose_R1_peak_	Intron (uc007a	1	32519563	32520999	1437	2	408192	uc011wiv.1	144238	ENSMUSG000	Gm9839	predicted gene 9839
chr1	32391998	32392204	207	208	32392088	33	17.97508	6.11109	14.52164	Adipose_R1_peak_	Intron (uc007a	1	32519563	32520999	1437	2	408192	uc011wiv.1	128795	ENSMUSG000	Gm9839	predicted gene 9839
chr1	32392005	32392182	178	179	32392076	37	11.12817	3.50427	7.83613	Adipose_R2_peak_	Intron (uc007a	1	32519563	32520999	1437	2	408192	uc011wiv.1	128817	ENSMUSG000	Gm9839	predicted gene 9839
chr1	32439686	32439872	187	188	32439793	25	11.36566	4.67319	8.13482	Adipose_R1_peak_	Intron (uc007a	1	32519563	32520999	1437	2	408192	uc011wiv.1	81127	ENSMUSG000	Gm9839	predicted gene 9839
chr1	32442419	32442660	242	243	32442547	32	17.10103	5.93135	13.67137	Adipose_R1_peak_	Intron (uc007a	1	32519563	32520999	1437	2	408192	uc011wiv.1	78339	ENSMUSG000	Gm9839	predicted gene 9839
chr1	32442484	32442599	116	117	32442549	32	8.29282	3.04319	5.18352	Adipose_R2_peak_	Intron (uc007a	1	32519563	32520999	1437	2	408192	uc011wiv.1	78400	ENSMUSG000	Gm9839	predicted gene 9839
chr1	32476091	32476230	140	141	32476170	26	5.31252	2.48988	2.47999	Adipose_R2_peak_	Intron (uc007a	1	32519563	32520999	1437	2	408192	uc011wiv.1	44769	ENSMUSG000	Gm9839	predicted gene 9839
chr1	32667248	32667453	206	207	32667368	52	19.87205	4.5855	16.22688	Adipose_R2_peak_	Distal Interger	1	32543546	32547293	3748	2	408191	uc011wiw.1	-119955	ENSMUSG000	Gm5415	predicted gene 5415
chr1	32667259	32667502	244	245	32667378	44	20.15377	5.37253	16.65174	Adipose_R1_peak_	Distal Interger	1	32543546	32547293	3748	2	408191	uc011wiw.1	-119966	ENSMUSG000	Gm5415	predicted gene 5415
chr1	32757979	32758184	206	207	32758060	23	9.86443	4.31371	6.70247	Adipose_R1_peak_	Distal Interger	1	32543546	32547293	3748	2	408191	uc011wiw.1	-210686	ENSMUSG000	Gm5415	predicted gene 5415
chr1	32759812	32759981	170	171	32759904	21	8.43286	3.95423	5.34896	Adipose_R1_peak_	Distal Interger	1	32543546	32547293	3748	2	408191	uc011wiw.1	-212519	ENSMUSG000	Gm5415	predicted gene 5415
chr1	32976289	32976522	234	235	32976399	34	18.86162	6.29083	15.38482	Adipose_R1_peak_	Distal Interger	1	32543546	32547293	3748	2	408191	uc011wiw.1	-428996	ENSMUSG000	Gm5415	predicted gene 5415
chr1	32976330	32976503	174	175	32976432	38	11.72962	3.59649	8.4047	Adipose_R2_peak_	Distal Interger	1	32543546	32547293	3748	2	408191	uc011wiw.1	-429037	ENSMUSG000	Gm5415	predicted gene 5415
chr1	33018905	33019053	149	150	33018970	22	7.90912	3.63517	4.867	Adipose_R1_peak_	Distal Interger	1	32543546	32547293	3748	2	408191	uc011wiw.1	-471612	ENSMUSG000	Gm5415	predicted gene 5415

chr1	33065396	33065583	188	189	33065511	43	14.8939	4.05758	11.42017	Adipose_R2_peak_	Distal Interger	1	32543546	32547293	3748	2	408191	uc011wiw.1	-518103	ENSMUSG000	Gm5415	predicted gene 5415
chr1	33074020	33074177	158	159	33074116	23	9.86443	4.31371	6.70247	Adipose_R1_peak_	Distal Interger	1	32543546	32547293	3748	2	408191	uc011wiw.1	-526727	ENSMUSG000	Gm5415	predicted gene 5415
chr1	33117353	33117490	138	139	33117436	20	7.74501	3.7745	4.70467	Adipose_R1_peak_	Distal Interger	1	33478942	33622142	143201	2	19076	uc007anr.1	504652	ENSMUSG000	Prim2	DNA primase, p58 subunit
chr1	33230002	33230186	185	186	33230086	28	13.73721	5.2124	10.41463	Adipose_R1_peak_	Distal Interger	1	33478942	33622142	143201	2	19076	uc007anr.1	391956	ENSMUSG000	Prim2	DNA primase, p58 subunit
chr1	33230969	33231152	184	185	33231062	22	9.13959	4.13397	6.01526	Adipose_R1_peak_	Distal Interger	1	33478942	33622142	143201	2	19076	uc007anr.1	390990	ENSMUSG000	Prim2	DNA primase, p58 subunit
chr1	33232716	33233203	488	489	33232856	37	21.59274	6.83004	18.04766	Adipose_R1_peak_	Distal Interger	1	33478942	33622142	143201	2	19076	uc007anr.1	388939	ENSMUSG000	Prim2	DNA primase, p58 subunit
chr1	33252499	33252743	245	246	33252627	35	19.76029	6.47056	16.26028	Adipose_R1_peak_	Distal Interger	1	33478942	33622142	143201	2	19076	uc007anr.1	369399	ENSMUSG000	Prim2	DNA primase, p58 subunit
chr1	33252562	33252668	107	108	33252615	32	8.29282	3.04319	5.18352	Adipose_R2_peak_	Distal Interger	1	33478942	33622142	143201	2	19076	uc007anr.1	369474	ENSMUSG000	Prim2	DNA primase, p58 subunit
chr1	33300457	33300697	241	242	33300550	39	23.47001	7.18952	19.88325	Adipose_R1_peak_	Distal Interger	1	33478942	33622142	143201	2	19076	uc007anr.1	321445	ENSMUSG000	Prim2	DNA primase, p58 subunit
chr1	33300488	33300691	204	205	33300587	44	15.55653	4.1498	12.05625	Adipose_R2_peak_	Distal Interger	1	33478942	33622142	143201	2	19076	uc007anr.1	321451	ENSMUSG000	Prim2	DNA primase, p58 subunit
chr1	33305736	33305925	190	191	33305818	23	9.86443	4.31371	6.70247	Adipose_R1_peak_	Distal Interger	1	33478942	33622142	143201	2	19076	uc007anr.1	316217	ENSMUSG000	Prim2	DNA primase, p58 subunit
chr1	33413833	33414041	209	210	33413918	46	16.90986	4.33423	13.35898	Adipose_R2_peak_	Distal Interger	1	33478942	33622142	143201	2	19076	uc007anr.1	208101	ENSMUSG000	Prim2	DNA primase, p58 subunit
chr1	33413854	33414044	191	192	33413931	38	17.05383	5.10581	13.64521	Adipose_R1_peak_	Distal Interger	1	33478942	33622142	143201	2	19076	uc007anr.1	208098	ENSMUSG000	Prim2	DNA primase, p58 subunit
chr1	33486622	33487149	528	529	33486722	17	5.80341	3.23528	2.92378	Adipose_R1_peak_	Intron (uc007a	1	33478942	33622142	143201	2	19076	uc007anr.1	134993	ENSMUSG000	Prim2	DNA primase, p58 subunit
chr1	33528191	33528303	113	114	33528232	27	5.77405	2.5821	2.88921	Adipose_R2_peak_	Intron (uc007a	1	33478942	33622142	143201	2	19076	uc007anr.1	93839	ENSMUSG000	Prim2	DNA primase, p58 subunit
chr1	33538913	33539218	306	307	33539050	130	132.5257	21.83604	128.0663	Adipose_R1_peak_	Intron (uc007a	1	33478942	33622142	143201	2	19076	uc007anr.1	82924	ENSMUSG000	Prim2	DNA primase, p58 subunit
chr1	33538918	33539201	284	285	33539054	108	72.13875	10.05173	67.7811	Adipose_R2_peak_	Intron (uc007a	1	33478942	33622142	143201	2	19076	uc007anr.1	82941	ENSMUSG000	Prim2	DNA primase, p58 subunit
chr1	33539593	33539924	332	333	33539742	102	85.97438	14.57959	81.71259	Adipose_R1_peak_	Intron (uc007a	1	33478942	33622142	143201	2	19076	uc007anr.1	82218	ENSMUSG000	Prim2	DNA primase, p58 subunit
chr1	33539635	33539870	236	237	33539743	80	44.53636	7.46964	40.44495	Adipose_R2_peak_	Intron (uc007a	1	33478942	33622142	143201	2	19076	uc007anr.1	82272	ENSMUSG000	Prim2	DNA primase, p58 subunit
chr1	33575888	33576243	356	357	33576052	25	11.14684	4.58438	7.94567	Adipose_R1_peak_	Intron (uc007a	1	33478942	33622142	143201	2	19076	uc007anr.1	45899	ENSMUSG000	Prim2	DNA primase, p58 subunit
chr1	33576054	33576244	191	192	33576163	36	10.5378	3.41206	7.27949	Adipose_R2_peak_	Intron (uc007a	1	33478942	33622142	143201	2	19076	uc007anr.1	45898	ENSMUSG000	Prim2	DNA primase, p58 subunit
chr1	34376982	34377279	298	299	34377110	35	19.76029	6.47056	16.26028	Adipose_R1_peak_	Distal Interger	1	34433121	34433199	79	2	1.01E+08	uc029qns.1	55920	ENSMUSG000	Mir5103	microRNA 5103
chr1	34435372	34435578	207	208	34435423	26	5.31252	2.48988	2.47999	Adipose_R2_peak_	Promoter (2-3	1	34433121	34433199	79	2	1.01E+08	uc029qns.1	-2173	ENSMUSG000	Mir5103	microRNA 5103
chr1	35045760	35046003	244	245	35045879	41	25.38991	7.54899	21.76305	Adipose_R1_peak_	Distal Interger	1	34849959	34871557	21599	1	226971	uc007apk.2	195801	ENSMUSG000	Plekhb2	pleckstrin homology domain containing, family B (evectins) member 2
chr1	35045764	35045956	193	194	35045854	38	11.72962	3.59649	8.4047	Adipose_R2_peak_	Distal Interger	1	34849959	34871557	21599	1	226971	uc007apk.2	195805	ENSMUSG000	Plekhb2	pleckstrin homology domain containing, family B (evectins) member 2
chr1	35046920	35047135	216	217	35047045	27	12.93148	5.03266	9.63827	Adipose_R1_peak_	Distal Interger	1	34849959	34871557	21599	1	226971	uc007apk.2	196961	ENSMUSG000	Plekhb2	pleckstrin homology domain containing, family B (evectins) member 2
chr1	35056275	35056415	141	142	35056332	16	5.20017	3.05554	2.38734	Adipose_R1_peak_	Distal Interger	1	34849959	34871557	21599	1	226971	uc007apk.2	206316	ENSMUSG000	Plekhb2	pleckstrin homology domain containing, family B (evectins) member 2
chr1	35408212	35408370	159	160	35408274	21	8.43286	3.95423	5.34896	Adipose_R1_peak_	Distal Interger	1	34849959	34871557	21599	1	226971	uc007apk.2	558253	ENSMUSG000	Plekhb2	pleckstrin homology domain containing, family B (evectins) member 2
chr1	35408716	35408996	281	282	35408853	66	48.58143	10.72838	44.61815	Adipose_R1_peak_	Distal Interger	1	34849959	34871557	21599	1	226971	uc007apk.2	558757	ENSMUSG000	Plekhb2	pleckstrin homology domain containing, family B (evectins) member 2
chr1	35408735	35408999	265	266	35408861	67	33.06755	6.27081	29.1399	Adipose_R2_peak_	Distal Interger	1	34849959	34871557	21599	1	226971	uc007apk.2	558776	ENSMUSG000	Plekhb2	pleckstrin homology domain containing, family B (evectins) member 2
chr1	36287468	36287863	396	397	36287581	28	6.25023	2.67431	3.31626	Adipose_R2_peak_	Distal Interger	1	36264602	36273425	8824	2	214854	uc007aps.1	-14043	ENSMUSG000	Neurl3	neuralized E3 ubiquitin protein ligase 3
chr1	36287506	36287929	424	425	36287815	26	10.9428	4.38087	7.74312	Adipose_R1_peak_	Distal Interger	1	36264602	36273425	8824	2	214854	uc007aps.1	-14081	ENSMUSG000	Neurl3	neuralized E3 ubiquitin protein ligase 3
chr1	36726848	36727167	320	321	36726939	21	8.43286	3.95423	5.34896	Adipose_R1_peak_	Intron (uc007a	1	36714538	36757960	43423	1	71166	uc007aqy.2	12310	ENSMUSG000	4933424G06R	RIKEN cDNA 4933424G06 gene
chr1	36726917	36727082	166	167	36727049	28	6.25023	2.67431	3.31626	Adipose_R2_peak_	Intron (uc007a	1	36714538	36757960	43423	1	71166	uc007aqy.2	12379	ENSMUSG000	4933424G06R	RIKEN cDNA 4933424G06 gene
chr1	37229254	37229438	185	186	37229284	19	7.07687	3.59476	4.08458	Adipose_R1_peak_	Intron (uc033f	1	37219205	37263384	44180	1	12790	uc007ari.2	10049	ENSMUSG000	Cnga3	cyclic nucleotide gated channel alpha 3
chr1	37232884	37233138	255	256	37233019	56	24.19188	5.25641	20.43163	Adipose_R2_peak_	Intron (uc033f	1	37219205	37263384	44180	1	12790	uc007ari.2	13679	ENSMUSG000	Cnga3	cyclic nucleotide gated channel alpha 3
chr1	37232966	37233136	171	172	37233065	31	8.63952	3.19519	5.5536	Adipose_R1_peak_	Intron (uc033f	1	37219205	37263384	44180	1	12790	uc007ari.2	13761	ENSMUSG000	Cnga3	cyclic nucleotide gated channel alpha 3
chr1	37604888	37605113	226	227	37604995	56	17.07464	3.76903	13.52087	Adipose_R2_peak_	Distal Interger	1	37611677	37626068	14392	2	72097	uc007arw.1	20955	ENSMUSG000	2010300C02Ri	RIKEN cDNA 2010300C02 gene
chr1	37679577	37679760	184	185	37679657	33	8.83596	3.1354	5.68652	Adipose_R2_peak_	Intron (uc007a	1	37611676	37719811	108136	2	72097	uc007arx.2	40051	ENSMUSG000	2010300C02Ri	RIKEN cDNA 2010300C02 gene
chr1	37777667	37777886	220	221	37777795	37	21.59274	6.83004	18.04766	Adipose_R1_peak_	Intron (uc007a	1	37611676	37719811	108136	2	72097	uc007arx.2	-57856	ENSMUSG000	2010300C02Ri	RIKEN cDNA 2010300C02 gene
chr1	38536414	38536577	164	165	38536437	18	6.42934	3.41502	3.48999	Adipose_R1_peak_	Intron (uc007a	1	38175991	38627244	451254	2	16764	uc007ast.2	90667	ENSMUSG000	Aff3	AF4/FMR2 family, member 3
chr1	38653377	38653494	118	119	38653421	34	9.39139	3.22762	6.20474	Adipose_R2_peak_	Intron (uc056y	1	38335431	38664955	329525	2	16764	uc056ycp.1	11461	ENSMUSG000	Aff3	AF4/FMR2 family, member 3
chr1	38653379	38653513	135	136	38653456	22	9.13959	4.13397	6.01526	Adipose_R1_peak_	Intron (uc056y	1	38335431	38664955	329525	2	16764	uc056ycp.1	11442	ENSMUSG000	Aff3	AF4/FMR2 family, member 3
chr1	40199665	40199880	216	217	40199791	32	17.10103	5.93135	13.67137	Adipose_R1_peak_	Distal Interger	1	40225080	40316177	91098	1	16177	uc007atv.1	-25200	ENSMUSG000	Il1r1	interleukin 1 receptor, type I
chr1	40238438	40238804	367	368	40238601	43	14.8939	4.05758	11.42017	Adipose_R2_peak_	Intron (uc007a	1	40225080	40316177	91098	1	16177	uc007atv.1	13358	ENSMUSG000	Il1r1	interleukin 1 receptor, type I
chr1	40238446	40238804	359	360	40238587	34	18.86162	6.29083	15.38482	Adipose_R1_peak_	Intron (uc007a	1	40225080	40316177	91098	1	16177	uc007atv.1	13366	ENSMUSG000	Il1r1	interleukin 1 receptor, type I
chr1	40909544	40909691	148	149	40909608	27	5.77405	2.5821	2.88921	Adipose_R2_peak_	Distal Interger	1	40805601	40855267	49667	1	381339	uc007auq.1	103943	ENSMUSG000	Tmem182	transmembrane protein 182
chr1	40915879	40916044	166	167	40915923	17	5.80341	3.23528	2.92378	Adipose_R1_peak_	Distal Interger	1	40805601	40855267	49667	1	381339	uc007auq.1	110278	ENSMUSG000	Tmem182	transmembrane protein 182
chr1	41129198	41129337	140	141	41129263	17	5.80341	3.23528	2.92378	Adipose_R1_peak_	Distal Interger	1	41167560	41181252	13693	2	74897	uc029qny.1	51915	ENSMUSG000	4930448I06Ri	RIKEN cDNA 4930448I06 gene
chr1	41365172	41365627	456	457	41365478	56	40.97333	10.24506	37.09402	Adipose_R1_peak_	Distal Interger	1	41167560	41181252	13693	2	74897	uc029qny.1	-183920	ENSMUSG000	4930448I06Ri	RIKEN cDNA 4930448I06 gene
chr1	41365185	41365567	383	384	41365429	37	11.12817	3.50427	7.83613	Adipose_R2_peak_	Distal Interger	1	41167560	41181252	13693	2	74897	uc029qny.1	-183933	ENSMUSG000	4930448I06Ri	RIKEN cDNA 4930448I06 gene
chr1	41472519	41472886	368	369	41472700	115	116.015	20.8496	111.6139	Adipose_R1_peak_	Distal Interger	1	41167560	41181252	13693	2	74897	uc029qny.1	-291267	ENSMUSG000	4930448I06Ri	RIKEN cDNA 4930448I06 gene
chr1	41472573	41472845	273	274	41472698	65	31.39315	6.08637	27.49408	Adipose_R2_peak_	Distal Interger	1	41167560	41181252	13693	2	74897	uc029qny.1	-291321	ENSMUSG000	4930448I06Ri	RIKEN cDNA 4930448I06 gene
chr1	41576707	41577045	339	340	41576845	25	11.36566	4.67319	8.13482	Adipose_R1_peak_	Distal Interger	1	41167560	41181252	13693	2	74897	uc029qny.1	-395455	ENSMUSG000	4930448I06Ri	RIKEN cDNA 4930448I06 gene
chr1	41679789	41679942	154	155	41679886	24	10.60668	4.49345	7.40961	Adipose_R1_peak_	Distal Interger	1	41167560	41181252	13693	2	74897	uc029qny.1	-498537	ENSMUSG000	4930448I06Ri	RIKEN cDNA 4930448I06 gene
chr1	41917938	41918104	167	168	41918016	38	18.34288	5.52043	14.88761	Adipose_R1_peak_	Distal Interger	1	41167560	41181252	13693	2	74897	uc029qny.1	-736686	ENSMUSG000	4930448I06Ri	RIKEN cDNA 4930448I06 gene
chr1	41917960	41918102	143	144	41918008	30	7.24477	2.85875	4.21917	Adipose_R2_peak_	Distal Interger	1	41167560	41181252	13693	2	74897	uc029qny.1	-736708	ENSMUSG000	4930448I06Ri	RIKEN cDNA 4930448I06 gene
chr1	43226534	43226692	159	160	43226597	23	9.86443	4.31371	6.70247	Adipose_R1_peak_	Distal Interger	1	43123071	43196761	73691	2	14200	uc007avl.2	-29773	ENSMUSG000	Fhl2	four and a half LIM domains 2
chr1	43227103	43227427	325	326	43227281	75	58.34468	12.01187	54.27874	Adipose_R1_peak_	Distal Interger	1	43123071	43196761	73691	2	14200	uc007avl.2	-30342	ENSMUSG000	Fhl2	four and a half LIM domains 2
chr1	43227171	43227427	257	258	43227295	67	33.06755	6.27081	29.1399	Adipose_R2_peak_	Distal Interger	1	43123071	43196761	73691	2	14200	uc007avl.2	-30410	ENSMUSG000	Fhl2	four and a half LIM domains 2
chr1	43640775	43640993	219	220	43640881	27	12.93148	5.03266	9.63827	Adipose_R1_peak_	Distal Interger	1	43730602	43742564	11963	1	78896	uc007avp.2	-89609	ENSMUSG000	1500015O10R	RIKEN cDNA 1500015O10 gene
chr1	43777616	43778107	492	493	43777686	19	7.07687	3.59476	4.08458	Adipose_R1_peak_	Intron (uc007a	1	43730602	43742564	11963	1	78896	uc007avp.2	47014	ENSMUSG000	1500015O10R	RIKEN cDNA 1500015O10 gene
chr1	43777799	43778160	362	363	43777909	47	17.60017	4.42645	14.02531	Adipose_R2_peak_	Intron (uc007a	1	43730602	43742564	11963	1	78896	uc007avp.2	47197	ENSMUSG000	1500015O10R	RIKEN cDNA 1500015O10 gene
chr1	43778404	43778926	523	524	43778567	47	28.20262	7.48943	24.53385	Adipose_R1_peak_	Intron (uc007a	1	43730602	43742564	11963	1	78896	uc007avp.2	47802	ENSMUSG000	1500015O10R	RIKEN cDNA 1500015O10 gene
chr1	43778470	43778887	418	419	43778599	48	18.29934	4.51867	14.70076	Adipose_R2_peak_	Intron (uc007a	1	43730602	43742564	11963	1	78896	uc007avp.2	47868	ENSMUSG000	1500015O10R	RIKEN cDNA 1500015O10 gene
chr1	43835535	43835670	136	137	43835568	26	5.31252	2.48988	2.47999	Adipose_R2_peak_	Distal Interger	1	43750231	43827708	77478	2	67883	uc007avq.1	-7827	ENSMUSG000	Uxs1	UDP-glucuronate decarboxylase 1
chr1	43849608	43850067	460	461	43849837	256	283.6473	29.252	278.9086	Adipose_R1_peak_	Distal Interger	1	43750231	43827708	77478	2	67883	uc007avq.1	-21900	ENSMUSG000	Uxs1	UDP-glucuronate decarboxylase 1
chr1	43849639	43849983	345	346	43849835	221	197.6076	18.13168	192.8695	Adipose_R2_peak_	Distal Interger	1	43750231	43827708	77478	2	67883	uc007avq.1	-21931	ENSMUSG000	Uxs1	UDP-glucuronate decarboxylase 1
chr1	43850353	43850654	302	303	43850520	59	44.30963	10.78427	40.38949	Adipose_R1_peak_	Distal Interger	1	43750231	43827708	77478	2	67883	uc007avq.1	-22645	ENSMUSG000	Uxs1	UDP-glucuronate decarboxylase 1
chr1	43850371	43850617	247	248	43850522	53	21.92215	4.97976	18.21755	Adipose_R2_peak_	Distal Interger	1	43750231	43827708	77478	2	67883	uc007avq.1	-22663	ENSMUSG000	Uxs1	UDP-glucuronate decarboxylase 1
chr1	43905639	43905877	239	240	43905759	36	20.67077	6.6503	17.14779	Adipose_R1_peak_	Distal Interger	1	43933994	43957003	23010	1	22019	uc007avr.2	-28117	ENSMUSG000	Tpp2	tripeptidyl peptidase II
chr1	43909694	43909905	212	213	43909813	29	14.55748	5.39214	11.20652	Adipose_R1_peak_	Distal Interger	1	43933994	43957003	23010	1	22019	uc007avr.2	-24089	ENSMUSG000	Tpp2	tripeptidyl peptidase II
chr1	43909748	43909857	110	111	43909826	30	7.24477	2.85875	4.21917	Adipose_R2_peak_	Distal Interger	1	43933994	43957003	23010	1	22019	uc007avr.2	-24137	ENSMUSG000	Tpp2	tripeptidyl peptidase II
chr1	44245816	44245980	165	166	44245908	24	10.60668	4.49345	7.40961	Adipose_R1_peak_	Distal Interger	1	44204070	44218931	14862	2	403183	uc007awk.1	-26885	ENSMUSG000	Mettl21e	methyltransferase like 21E
chr1	44245874	44245968	95	96	44245922	27	5.77405	2.5821	2.88921	Adipose_R2_peak_	Distal Interger	1	44204070	44218931	14862	2	403183	uc007awk.1	-26943	ENSMUSG000	Mettl21e	methyltransferase like 21E
chr1	44248655	44248793	139	140	44248719	21	8.43286	3.95423	5.34896	Adipose_R1_peak_	Distal Interger	1	44204070	44218931	14862	2	403183	uc007awk.1	-29724	ENSMUSG000	Mettl21e	methyltransferase like 21E
chr1	44272088	44272462	375	376	44272278	87	70.48232	13.22335	66.3181	Adipose_R1_peak_	Distal Interger	1	44204070	44218931	14862	2	403183	uc007awk.1	-53157	ENSMUSG000	Mettl21e	methyltransferase like 21E
chr1	44272120	44272420	301	302	44272288	71	36.49133	6.63968	32.5091	Adipose_R2_peak_	Distal Interger	1	44204070	44218931	14862	2	403183	uc007awk.1	-53189	ENSMUSG000	Mettl21e	methyltransferase like 21E
chr1	44274638	44274845	208	209	44274736	35	19.76029	6.47056	16.26028	Adipose_R1_peak_	Distal Interger	1	44204070	44218931	14862	2	403183	uc007awk.1	-55707	ENSMUSG000	Mettl21e	methyltransferase like 21E
chr1	44283724	44283986	263	264	44283864	44	28.34544	8.08821	24.66239	Adipose_R1_peak_	Distal Interger	1	44204070	44218931	14862	2	403183	uc007awk.1	-64793	ENSMUSG000	Mettl21e	methyltransferase like 21E
chr1	44283763	44283918	156	157	44283861	35	9.95877	3.31984	6.73562	Adipose_R2_peak_	Distal Interger	1	44204070	44218931	14862	2	403183	uc007awk.1	-64832	ENSMUSG000	Mettl21e	methyltransferase like 21E
chr1	44355497	44355684	188	189	44355600	26	12.14079	4.85292	8.87838	Adipose_R1_peak_	Distal Interger	1	44204070	44218931	14862	2	403183	uc007awk.1	-136566	ENSMUSG000	Mettl21e	methyltransferase like 21E
chr1	44355541	44355677	137	138	44355607	31	7.7623	2.95097	4.69388	Adipose_R2_peak_	Distal Interger	1	44204070	44218931	14862	2	403183	uc007awk.1	-136610	ENSMUSG000	Mettl21e	methyltransferase like 21E
chr1	44369500	44369631	132	133	44369562	19	7.07687	3.59476	4.08458	Adipose_R1_peak_	Distal Interger	1	44204070	44218931	14862	2	403183	uc007awk.1	-150569	ENSMUSG000	Mettl21e	methyltransferase like 21E
chr1	44371221	44371550	330	331	44371384	153	122.7887	14.20153	118.202	Adipose_R2_peak_	Distal Interger	1	44204070	44218931	14862	2	403183	uc007awk.1	-152290	ENSMUSG000	Mettl21e	methyltransferase like 21E
chr1	44371237	44371591	355	356	44371387	157	154.8916	21.13869	150.3639	Adipose_R1_peak_	Distal Interger	1	44204070	44218931	14862	2	403183	uc007awk.1	-152306	ENSMUSG000	Mettl21e	methyltransferase like 21E
chr1	44374718	44375026	309	310	44374883	84	74.36649	15.27772	70.17167	Adipose_R1_peak_	Distal Interger	1	44204070	44218931	14862	2	403183	uc007awk.1	-155787	ENSMUSG000	Mettl21e	methyltransferase like 21E
chr1	44374733	44375017	285	286	44374861	78	42.70965	7.2852	38.64095	Adipose_R2_peak_	Distal Interger	1	44204070	44218931	14862	2	403183	uc007awk.1	-155802	ENSMUSG000	Mettl21e	methyltransferase like 21E
chr1	44406274	44406425	152	153	44406338	19	7.07687	3.59476	4.08458	Adipose_R1_peak_	Distal Interger	1	44551671	44788874	237204	1	70676	uc007awl.2	-145246	ENSMUSG000	Gulp1	GULP, engulfment adaptor PTB domain containing 1
chr1	44413292	44413612	321	322	44413437	127	93.17986	11.90405	88.87962	Adipose_R1_peak_	Distal Interger	1	44551671	44788874	237204	1	70676	uc007awl.2	-138059	ENSMUSG000	Gulp1	GULP, engulfment adaptor PTB domain containing 1
chr1	44413305	44413573	269	270	44413442	114	78.48856	10.60504	74.08665	Adipose_R2_peak_	Distal Interger	1	44551671	44788874	237204	1	70676	uc007awl.2	-138098	ENSMUSG000	Gulp1	GULP, engulfment adaptor PTB domain containing 1
chr1	44417948	44418327	380	381	44418159	125	130.2678	22.64698	125.815	Adipose_R1_peak_	Distal Interger	1	44551671	44788874	237204	1	70676	uc007awl.2	-133344	ENSMUSG000	Gulp1	GULP, engulfment adaptor PTB domain containing 1
chr1	44417975	44418321	347	348	44418156	105	69.01683	9.77508	64.68261	Adipose_R2_peak_	Distal Interger	1	44551671	44788874	237204	1	70676	uc007awl.2	-133350	ENSMUSG000	Gulp1	GULP, engulfment adaptor PTB domain containing 1
chr1	44488972	44489211	240	241	44489090	38	22.52591	7.00978	18.96	Adipose_R1_peak_	Distal Interger	1	44551671	44788874	237204	1	70676	uc007awl.2	-62460	ENSMUSG000	Gulp1	GULP, engulfment adaptor PTB domain containing 1

chr1	44488991	44489174	184	185	44489089	40	12.96465	3.78093	9.57654	Adipose_R2_peak_	Distal Interger	1	44551671	44788874	237204	1	70676	uc007awl.2	-62497	ENSMUSG000	Gulp1	GULP, engulfment adaptor PTB domain containing 1
chr1	44511499	44511779	281	282	44511675	27	12.93148	5.03266	9.63827	Adipose_R1_peak_	Distal Interger	1	44551671	44788874	237204	1	70676	uc007awl.2	-39892	ENSMUSG000	Gulp1	GULP, engulfment adaptor PTB domain containing 1
chr1	44608932	44609134	203	204	44609037	25	11.36566	4.67319	8.13482	Adipose_R1_peak_	Intron (uc007a	1	44551671	44788874	237204	1	70676	uc007awl.2	57261	ENSMUSG000	Gulp1	GULP, engulfment adaptor PTB domain containing 1
chr1	44696093	44696273	181	182	44696159	23	9.86443	4.31371	6.70247	Adipose_R1_peak_	Intron (uc007a	1	44739675	44744949	5275	2	75066	uc029qoa.1	48676	ENSMUSG000	4930521E06Ri	RIKEN cDNA 4930521E06 gene
chr1	44825467	44825699	233	234	44825547	40	12.96465	3.78093	9.57654	Adipose_R2_peak_	Distal Interger	1	44739675	44744949	5275	2	75066	uc029qoa.1	-80518	ENSMUSG000	4930521E06Ri	RIKEN cDNA 4930521E06 gene
chr1	44898532	44898702	171	172	44898605	18	6.42934	3.41502	3.48999	Adipose_R1_peak_	Distal Interger	1	44739675	44744949	5275	2	75066	uc029qoa.1	-153583	ENSMUSG000	4930521E06Ri	RIKEN cDNA 4930521E06 gene
chr1	44959598	44959751	154	155	44959622	26	5.31252	2.48988	2.47999	Adipose_R2_peak_	Distal Interger	1	44739675	44744949	5275	2	75066	uc029qoa.1	-214649	ENSMUSG000	4930521E06Ri	RIKEN cDNA 4930521E06 gene
chr1	44960654	44960908	255	256	44960774	35	18.97054	6.1693	15.49305	Adipose_R1_peak_	Distal Interger	1	44739675	44744949	5275	2	75066	uc029qoa.1	-215705	ENSMUSG000	4930521E06Ri	RIKEN cDNA 4930521E06 gene
chr1	44960721	44960822	102	103	44960765	32	8.29282	3.04319	5.18352	Adipose_R2_peak_	Distal Interger	1	44739675	44744949	5275	2	75066	uc029qoa.1	-215772	ENSMUSG000	4930521E06Ri	RIKEN cDNA 4930521E06 gene
chr1	45106896	45107098	203	204	45107015	26	12.14079	4.85292	8.87838	Adipose_R1_peak_	Distal Interger	1	45311538	45325018	13481	1	12825	uc007awp.2	-204440	ENSMUSG000	Col3a1	collagen, type III, alpha 1
chr1	45129668	45129770	103	104	45129708	25	4.86612	2.39766	2.09018	Adipose_R2_peak_	Distal Interger	1	45311538	45325018	13481	1	12825	uc007awp.2	-181768	ENSMUSG000	Col3a1	collagen, type III, alpha 1
chr1	45199226	45199394	169	170	45199328	22	9.13959	4.13397	6.01526	Adipose_R1_peak_	Distal Interger	1	45311538	45325018	13481	1	12825	uc007awp.2	-112144	ENSMUSG000	Col3a1	collagen, type III, alpha 1
chr1	45202028	45202649	622	623	45202195	95	81.34936	14.78974	77.11219	Adipose_R1_peak_	Distal Interger	1	45311538	45325018	13481	1	12825	uc007awp.2	-108889	ENSMUSG000	Col3a1	collagen, type III, alpha 1
chr1	45202050	45202334	285	286	45202192	84	48.25301	7.83851	44.11785	Adipose_R2_peak_	Distal Interger	1	45311538	45325018	13481	1	12825	uc007awp.2	-109204	ENSMUSG000	Col3a1	collagen, type III, alpha 1
chr1	45202443	45202615	173	174	45202521	47	17.60017	4.42645	14.02531	Adipose_R2_peak_	Distal Interger	1	45311538	45325018	13481	1	12825	uc007awp.2	-108923	ENSMUSG000	Col3a1	collagen, type III, alpha 1
chr1	45202940	45203096	157	158	45203037	19	7.07687	3.59476	4.08458	Adipose_R1_peak_	Distal Interger	1	45311538	45325018	13481	1	12825	uc007awp.2	-108442	ENSMUSG000	Col3a1	collagen, type III, alpha 1
chr1	45234207	45234453	247	248	45234309	38	22.52591	7.00978	18.96	Adipose_R1_peak_	Distal Interger	1	45311538	45325018	13481	1	12825	uc007awp.2	-77085	ENSMUSG000	Col3a1	collagen, type III, alpha 1
chr1	45251782	45252037	256	257	45251896	30	15.39182	5.57188	12.01394	Adipose_R1_peak_	Distal Interger	1	45311538	45325018	13481	1	12825	uc007awp.2	-59501	ENSMUSG000	Col3a1	collagen, type III, alpha 1
chr1	45537671	45537828	158	159	45537735	22	9.13959	4.13397	6.01526	Adipose_R1_peak_	Distal Interger	1	45374331	45503282	128952	2	12832	uc007awr.1	-34389	ENSMUSG000	Col5a2	collagen, type V, alpha 2
chr1	45558562	45558794	233	234	45558690	58	25.74274	5.44085	21.94821	Adipose_R2_peak_	Distal Interger	1	45374331	45503282	128952	2	12832	uc007awr.1	-55280	ENSMUSG000	Col5a2	collagen, type V, alpha 2
chr1	45613207	45613376	170	171	45613279	23	9.86443	4.31371	6.70247	Adipose_R1_peak_	Distal Interger	1	45374331	45503282	128952	2	12832	uc007awr.1	-109925	ENSMUSG000	Col5a2	collagen, type V, alpha 2
chr1	45638718	45638935	218	219	45638829	30	15.39182	5.57188	12.01394	Adipose_R1_peak_	Distal Interger	1	45374331	45503282	128952	2	12832	uc007awr.1	-135436	ENSMUSG000	Col5a2	collagen, type V, alpha 2
chr1	45638750	45638926	177	178	45638830	39	12.34187	3.68871	8.98501	Adipose_R2_peak_	Distal Interger	1	45374331	45503282	128952	2	12832	uc007awr.1	-135468	ENSMUSG000	Col5a2	collagen, type V, alpha 2
chr1	45945528	45945760	233	234	45945659	40	24.42476	7.36925	20.81738	Adipose_R1_peak_	Distal Interger	1	45921156	45926519	5364	2	53945	uc011wkh.1	-19009	ENSMUSG000	Slc40a1	solute carrier family 40 (iron-regulated transporter), member 1
chr1	45945579	45945691	113	114	45945646	28	6.25023	2.67431	3.31626	Adipose_R2_peak_	Distal Interger	1	45921156	45926519	5364	2	53945	uc011wkh.1	-19060	ENSMUSG000	Slc40a1	solute carrier family 40 (iron-regulated transporter), member 1
chr1	45973773	45974017	245	246	45973912	29	14.55748	5.39214	11.20652	Adipose_R1_peak_	Distal Interger	1	45921156	45926519	5364	2	53945	uc011wkh.1	-47254	ENSMUSG000	Slc40a1	solute carrier family 40 (iron-regulated transporter), member 1
chr1	45975007	45975162	156	157	45975124	16	5.20017	3.05554	2.38734	Adipose_R1_peak_	Distal Interger	1	45921156	45926519	5364	2	53945	uc011wkh.1	-48488	ENSMUSG000	Slc40a1	solute carrier family 40 (iron-regulated transporter), member 1
chr1	46094031	46094188	158	159	46094170	36	10.5378	3.41206	7.27949	Adipose_R2_peak_	Intron (uc007a	1	46066738	46373550	306813	1	227058	uc007awx.2	27293	ENSMUSG000	Dnah7b	dynein, axonemal, heavy chain 7B
chr1	46094068	46094248	181	182	46094155	22	9.13959	4.13397	6.01526	Adipose_R1_peak_	Intron (uc007a	1	46066738	46373550	306813	1	227058	uc007awx.2	27330	ENSMUSG000	Dnah7b	dynein, axonemal, heavy chain 7B
chr1	46130352	46130517	166	167	46130443	34	9.39139	3.22762	6.20474	Adipose_R2_peak_	Intron (uc007a	1	46066738	46373550	306813	1	227058	uc007awx.2	63614	ENSMUSG000	Dnah7b	dynein, axonemal, heavy chain 7B
chr1	46283271	46283409	139	140	46283334	19	7.07687	3.59476	4.08458	Adipose_R1_peak_	Intron (uc007a	1	46289533	46373550	84018	1	227058	uc007awz.2	-6124	ENSMUSG000	Dnah7b	dynein, axonemal, heavy chain 7B
chr1	46284174	46284304	131	132	46284236	17	5.80341	3.23528	2.92378	Adipose_R1_peak_	Intron (uc007a	1	46289533	46373550	84018	1	227058	uc007awz.2	-5229	ENSMUSG000	Dnah7b	dynein, axonemal, heavy chain 7B
chr1	46319309	46319529	221	222	46319416	41	25.38991	7.54899	21.76305	Adipose_R1_peak_	Intron (uc007a	1	46289533	46373550	84018	1	227058	uc007awz.2	29776	ENSMUSG000	Dnah7b	dynein, axonemal, heavy chain 7B
chr1	46348113	46348374	262	263	46348252	44	28.34544	8.08821	24.66239	Adipose_R1_peak_	Intron (uc007a	1	46289533	46373550	84018	1	227058	uc007awz.2	58580	ENSMUSG000	Dnah7b	dynein, axonemal, heavy chain 7B
chr1	46348176	46348316	141	142	46348254	29	6.74062	2.76653	3.75989	Adipose_R2_peak_	Intron (uc007a	1	46289533	46373550	84018	1	227058	uc007awz.2	58643	ENSMUSG000	Dnah7b	dynein, axonemal, heavy chain 7B
chr1	46349831	46350495	665	666	46349968	64	50.01186	11.68296	46.0279	Adipose_R1_peak_	Intron (uc007a	1	46289533	46373550	84018	1	227058	uc007awz.2	60298	ENSMUSG000	Dnah7b	dynein, axonemal, heavy chain 7B
chr1	46349842	46350065	224	225	46349966	48	18.29934	4.51867	14.70076	Adipose_R2_peak_	Intron (uc007a	1	46289533	46373550	84018	1	227058	uc007awz.2	60309	ENSMUSG000	Dnah7b	dynein, axonemal, heavy chain 7B
chr1	46583415	46583617	203	204	46583560	42	14.2409	3.96536	10.79479	Adipose_R2_peak_	Intron (uc007a	1	46425518	46807477	381960	1	1.00E+08	uc007axb.2	157897	ENSMUSG000	Dnah7c	dynein, axonemal, heavy chain 7C
chr1	46583457	46583601	145	146	46583538	31	16.23981	5.75161	12.83549	Adipose_R1_peak_	Intron (uc007a	1	46425518	46807477	381960	1	1.00E+08	uc007axb.2	157939	ENSMUSG000	Dnah7c	dynein, axonemal, heavy chain 7C
chr1	46704247	46704549	303	304	46704386	66	49.87331	11.16805	45.89693	Adipose_R1_peak_	Intron (uc007a	1	46817602	46836151	18550	2	227059	uc007axd.1	131602	ENSMUSG000	Slc39a10	solute carrier family 39 (zinc transporter), member 10
chr1	46704254	46704503	250	251	46704384	52	21.18119	4.88754	17.49655	Adipose_R2_peak_	Intron (uc007a	1	46817602	46836151	18550	2	227059	uc007axd.1	131648	ENSMUSG000	Slc39a10	solute carrier family 39 (zinc transporter), member 10
chr1	47110231	47110447	217	218	47110334	37	21.59274	6.83004	18.04766	Adipose_R1_peak_	Distal Interger	1	46807544	46853509	45966	2	227059	uc007axc.1	-256722	ENSMUSG000	Slc39a10	solute carrier family 39 (zinc transporter), member 10
chr1	47110680	47110841	162	163	47110806	26	12.14079	4.85292	8.87838	Adipose_R1_peak_	Distal Interger	1	46807544	46853509	45966	2	227059	uc007axc.1	-257171	ENSMUSG000	Slc39a10	solute carrier family 39 (zinc transporter), member 10
chr1	47125894	47125993	100	101	47125938	27	5.77405	2.5821	2.88921	Adipose_R2_peak_	Distal Interger	1	46807544	46853509	45966	2	227059	uc007axc.1	-272385	ENSMUSG000	Slc39a10	solute carrier family 39 (zinc transporter), member 10
chr1	47146210	47146516	307	308	47146342	49	30.12764	7.80149	26.42323	Adipose_R1_peak_	Distal Interger	1	46807544	46853509	45966	2	227059	uc007axc.1	-292701	ENSMUSG000	Slc39a10	solute carrier family 39 (zinc transporter), member 10
chr1	47146220	47146451	232	233	47146334	62	28.93052	5.80972	25.0743	Adipose_R2_peak_	Distal Interger	1	46807544	46853509	45966	2	227059	uc007axc.1	-292711	ENSMUSG000	Slc39a10	solute carrier family 39 (zinc transporter), member 10
chr1	47147769	47148000	232	233	47147882	29	14.55748	5.39214	11.20652	Adipose_R1_peak_	Distal Interger	1	46807544	46853509	45966	2	227059	uc007axc.1	-294260	ENSMUSG000	Slc39a10	solute carrier family 39 (zinc transporter), member 10
chr1	47147840	47147986	147	148	47147894	30	7.24477	2.85875	4.21917	Adipose_R2_peak_	Distal Interger	1	46807544	46853509	45966	2	227059	uc007axc.1	-294331	ENSMUSG000	Slc39a10	solute carrier family 39 (zinc transporter), member 10
chr1	47175354	47175597	244	245	47175467	35	19.76029	6.47056	16.26028	Adipose_R1_peak_	Distal Interger	1	47467021	47467125	105	2	1.02E+08	uc033fiz.1	291528	ENSMUSG000	Mir6350	microRNA 6350
chr1	47175382	47175589	208	209	47175480	45	16.22858	4.24202	12.70278	Adipose_R2_peak_	Distal Interger	1	47467021	47467125	105	2	1.02E+08	uc033fiz.1	291536	ENSMUSG000	Mir6350	microRNA 6350
chr1	47177760	47177957	198	199	47177835	21	8.43286	3.95423	5.34896	Adipose_R1_peak_	Distal Interger	1	47467021	47467125	105	2	1.02E+08	uc033fiz.1	289168	ENSMUSG000	Mir6350	microRNA 6350
chr1	47245152	47245390	239	240	47245281	31	16.23981	5.75161	12.83549	Adipose_R1_peak_	Distal Interger	1	47467021	47467125	105	2	1.02E+08	uc033fiz.1	221735	ENSMUSG000	Mir6350	microRNA 6350
chr1	47245193	47245398	206	207	47245288	40	12.96465	3.78093	9.57654	Adipose_R2_peak_	Distal Interger	1	47467021	47467125	105	2	1.02E+08	uc033fiz.1	221727	ENSMUSG000	Mir6350	microRNA 6350
chr1	47248128	47248235	108	109	47248194	26	5.31252	2.48988	2.47999	Adipose_R2_peak_	Distal Interger	1	47467021	47467125	105	2	1.02E+08	uc033fiz.1	218890	ENSMUSG000	Mir6350	microRNA 6350
chr1	47590711	47590867	157	158	47590785	25	11.36566	4.67319	8.13482	Adipose_R1_peak_	Distal Interger	1	47467021	47467125	105	2	1.02E+08	uc033fiz.1	-123586	ENSMUSG000	Mir6350	microRNA 6350
chr1	47593553	47593888	336	337	47593688	53	21.92215	4.97976	18.21755	Adipose_R2_peak_	Distal Interger	1	47467021	47467125	105	2	1.02E+08	uc033fiz.1	-126428	ENSMUSG000	Mir6350	microRNA 6350
chr1	47593642	47593923	282	283	47593779	30	15.39182	5.57188	12.01394	Adipose_R1_peak_	Distal Interger	1	47467021	47467125	105	2	1.02E+08	uc033fiz.1	-126517	ENSMUSG000	Mir6350	microRNA 6350
chr1	47647816	47647932	117	118	47647872	29	6.74062	2.76653	3.75989	Adipose_R2_peak_	Distal Interger	1	47467021	47467125	105	2	1.02E+08	uc033fiz.1	-180691	ENSMUSG000	Mir6350	microRNA 6350
chr1	47679284	47679515	232	233	47679365	22	9.13959	4.13397	6.01526	Adipose_R1_peak_	Distal Interger	1	47467021	47467125	105	2	1.02E+08	uc033fiz.1	-212159	ENSMUSG000	Mir6350	microRNA 6350
chr1	47680396	47680495	100	101	47680481	28	6.25023	2.67431	3.31626	Adipose_R2_peak_	Distal Interger	1	47467021	47467125	105	2	1.02E+08	uc033fiz.1	-213271	ENSMUSG000	Mir6350	microRNA 6350
chr1	47757022	47757244	223	224	47757131	27	12.93148	5.03266	9.63827	Adipose_R1_peak_	Distal Interger	1	47467021	47467125	105	2	1.02E+08	uc033fiz.1	-289897	ENSMUSG000	Mir6350	microRNA 6350
chr1	47790309	47790481	173	174	47790473	29	6.74062	2.76653	3.75989	Adipose_R2_peak_	Distal Interger	1	47467021	47467125	105	2	1.02E+08	uc033fiz.1	-323184	ENSMUSG000	Mir6350	microRNA 6350
chr1	47790443	47790731	289	290	47790466	16	5.20017	3.05554	2.38734	Adipose_R1_peak_	Distal Interger	1	47467021	47467125	105	2	1.02E+08	uc033fiz.1	-323318	ENSMUSG000	Mir6350	microRNA 6350
chr1	47937608	47937895	288	289	47937751	52	31.00269	7.59017	27.28092	Adipose_R1_peak_	Distal Interger	1	47467021	47467125	105	2	1.02E+08	uc033fiz.1	-470483	ENSMUSG000	Mir6350	microRNA 6350
chr1	47937628	47937894	267	268	47937759	64	30.56565	5.99415	26.6807	Adipose_R2_peak_	Distal Interger	1	47467021	47467125	105	2	1.02E+08	uc033fiz.1	-470503	ENSMUSG000	Mir6350	microRNA 6350
chr1	48049164	48049319	156	157	48049187	16	5.20017	3.05554	2.38734	Adipose_R1_peak_	Distal Interger	1	47467021	47467125	105	2	1.02E+08	uc033fiz.1	-582039	ENSMUSG000	Mir6350	microRNA 6350
chr1	48093308	48093472	165	166	48093386	40	12.96465	3.78093	9.57654	Adipose_R2_peak_	Distal Interger	1	47467021	47467125	105	2	1.02E+08	uc033fiz.1	-626183	ENSMUSG000	Mir6350	microRNA 6350
chr1	48124847	48124998	152	153	48124916	20	7.74501	3.7745	4.70467	Adipose_R1_peak_	Distal Interger	1	47467021	47467125	105	2	1.02E+08	uc033fiz.1	-657722	ENSMUSG000	Mir6350	microRNA 6350
chr1	48365879	48366172	294	295	48366009	60	45.43621	10.96401	41.50272	Adipose_R1_peak_	Distal Interger	1	47467021	47467125	105	2	1.02E+08	uc033fiz.1	-898754	ENSMUSG000	Mir6350	microRNA 6350
chr1	48365902	48366130	229	230	48366013	46	16.90986	4.33423	13.35898	Adipose_R2_peak_	Distal Interger	1	47467021	47467125	105	2	1.02E+08	uc033fiz.1	-898777	ENSMUSG000	Mir6350	microRNA 6350
chr1	48419174	48419379	206	207	48419275	30	15.39182	5.57188	12.01394	Adipose_R1_peak_	Distal Interger	1	47467021	47467125	105	2	1.02E+08	uc033fiz.1	-952049	ENSMUSG000	Mir6350	microRNA 6350
chr1	48484740	48485111	372	373	48484885	254	274.0419	27.48598	269.3132	Adipose_R1_peak_	Distal Interger	1	47467021	47467125	105	2	1.02E+08	uc033fiz.1	-1017615	ENSMUSG000	Mir6350	microRNA 6350
chr1	48484750	48485084	335	336	48484882	236	230.3502	21.8556	225.5661	Adipose_R2_peak_	Distal Interger	1	47467021	47467125	105	2	1.02E+08	uc033fiz.1	-1017625	ENSMUSG000	Mir6350	microRNA 6350
chr1	48498236	48498454	219	220	48498324	33	17.97508	6.11109	14.52164	Adipose_R1_peak_	Distal Interger	1	47467021	47467125	105	2	1.02E+08	uc033fiz.1	-1031111	ENSMUSG000	Mir6350	microRNA 6350
chr1	48507462	48507617	156	157	48507575	27	5.77405	2.5821	2.88921	Adipose_R2_peak_	Distal Interger	1	47467021	47467125	105	2	1.02E+08	uc033fiz.1	-1040337	ENSMUSG000	Mir6350	microRNA 6350
chr1	48546801	48547006	206	207	48546881	20	7.74501	3.7745	4.70467	Adipose_R1_peak_	Distal Interger	1	47467021	47467125	105	2	1.02E+08	uc033fiz.1	-1079676	ENSMUSG000	Mir6350	microRNA 6350
chr1	48549510	48549766	257	258	48549639	44	28.34544	8.08821	24.66239	Adipose_R1_peak_	Distal Interger	1	47467021	47467125	105	2	1.02E+08	uc033fiz.1	-1082385	ENSMUSG000	Mir6350	microRNA 6350
chr1	48549574	48549753	180	181	48549656	33	8.83596	3.1354	5.68652	Adipose_R2_peak_	Distal Interger	1	47467021	47467125	105	2	1.02E+08	uc033fiz.1	-1082449	ENSMUSG000	Mir6350	microRNA 6350
chr1	48816216	48816564	349	350	48816355	92	84.7075	16.71563	80.45023	Adipose_R1_peak_	Distal Interger	1	47467021	47467125	105	2	1.02E+08	uc033fiz.1	-1349091	ENSMUSG000	Mir6350	microRNA 6350
chr1	48816218	48816531	314	315	48816338	103	66.95583	9.59064	62.63862	Adipose_R2_peak_	Distal Interger	1	47467021	47467125	105	2	1.02E+08	uc033fiz.1	-1349093	ENSMUSG000	Mir6350	microRNA 6350
chr1	48817775	48817947	173	174	48817861	26	12.14079	4.85292	8.87838	Adipose_R1_peak_	Distal Interger	1	47467021	47467125	105	2	1.02E+08	uc033fiz.1	-1350650	ENSMUSG000	Mir6350	microRNA 6350
chr1	48824144	48824414	271	272	48824264	38	22.52591	7.00978	18.96	Adipose_R1_peak_	Distal Interger	1	47467021	47467125	105	2	1.02E+08	uc033fiz.1	-1357019	ENSMUSG000	Mir6350	microRNA 6350
chr1	48824169	48824364	196	197	48824279	34	9.39139	3.22762	6.20474	Adipose_R2_peak_	Distal Interger	1	47467021	47467125	105	2	1.02E+08	uc033fiz.1	-1357044	ENSMUSG000	Mir6350	microRNA 6350
chr1	48827186	48827488	303	304	48827307	74	61.90123	13.48034	57.80368	Adipose_R1_peak_	Distal Interger	1	47467021	47467125	105	2	1.02E+08	uc033fiz.1	-1360061	ENSMUSG000	Mir6350	microRNA 6350
chr1	48827191	48827457	267	268	48827304	71	36.49133	6.63968	32.5091	Adipose_R2_peak_	Distal Interger	1	47467021	47467125	105	2	1.02E+08	uc033fiz.1	-1360066	ENSMUSG000	Mir6350	microRNA 6350
chr1	48926279	48926527	249	250	48926405	45	29.34988	8.26794	25.64927	Adipose_R1_peak_	Distal Interger	1	47467021	47467125	105	2	1.02E+08	uc033fiz.1	-1459154	ENSMUSG000	Mir6350	microRNA 6350
chr1	48926317	48926484	168	169	48926406	37	11.12817	3.50427	7.83613	Adipose_R2_peak_	Distal Interger	1	47467021	47467125	105	2	1.02E+08	uc033fiz.1	-1459192	ENSMUSG000	Mir6350	microRNA 6350
chr1	49012222	49012507	286	287	49012361	80	69.31596	14.55877	65.15641	Adipose_R1_peak_	Distal Interger	1	47467021	47467125	105	2	1.02E+08	uc033fiz.1	-1545097	ENSMUSG000	Mir6350	microRNA 6350
chr1	49012323	49012422	100	101	49012368	29	6.74062	2.76653	3.75989	Adipose_R2_peak_	Distal Interger	1	47467021	47467125	105	2	1.02E+08	uc033fiz.1	-1545198	ENSMUSG000	Mir6350	microRNA 6350
chr1	49253185	49253364	180	181	49253286	25	11.36566	4.67319	8.13482	Adipose_R1_peak_	Intron (uc007a	1	50909962	50979920	69959	1	56363	uc007axi.1	-1656598	ENSMUSG000	Tmeff2	transmembrane protein with EGF-like and two follistatin-like domains 2
chr1	49255732	49255985	254	255	49255846	58	43.19021	10.60454	39.28351	Adipose_R1_peak_	Intron (uc007a	1	50909962	50979920	69959	1	56363	uc007axi.1	-1653977	ENSMUSG000	Tmeff2	transmembrane protein with EGF-like and two follistatin-like domains 2
chr1	49255763	49255935	173	174	49255868	29	6.74062	2.76653	3.75989	Adipose_R2_peak_	Intron (uc007a	1	50909962	50979920	69959	1	56363	uc007axi.1	-1654027	ENSMUSG000	Tmeff2	transmembrane protein with EGF-like and two follistatin-like domains 2
chr1	49322346	49322484	139	140	49322413	31	7.7623	2.95097	4.69388	Adipose_R2_peak_	Intron (uc007a	1	50909962	50979920	69959	1	56363	uc007axi.1	-1587478	ENSMUSG000	Tmeff2	transmembrane protein with EGF-like and two follistatin-like domains 2
chr1	49607462	49607863	402	403	49607649	121	99.74705	14.42438	95.4152	Adipose_R1_peak_	Distal Interger	1	50909962	50979920	69959	1	56363	uc007axi.1	-1302099	ENSMUSG000	Tmeff2	transmembrane protein with EGF-like and two follistatin-like domains 2

chr1	49607531	49607831	301	302	49607659	83	31.90153	4.88939	27.99754	Adipose_R2_peak_	Distal Intergen	1	50909962	50979920	69959	1	56363	uc007axi.1	-1302131	ENSMUSG000	Tmeff2	transmembrane protein with EGF-like and two follistatin-like domains 2
chr1	50122910	50123184	275	276	50123055	54	22.67101	5.07198	18.94758	Adipose_R2_peak_	Distal Intergen	1	50909962	50979920	69959	1	56363	uc007axi.1	-786778	ENSMUSG000	Tmeff2	transmembrane protein with EGF-like and two follistatin-like domains 2
chr1	50122927	50123192	266	267	50123052	41	25.38991	7.54899	21.76305	Adipose_R1_peak_	Distal Intergen	1	50909962	50979920	69959	1	56363	uc007axi.1	-786770	ENSMUSG000	Tmeff2	transmembrane protein with EGF-like and two follistatin-like domains 2
chr1	50370540	50371058	519	520	50370896	35	18.51832	6.00074	15.0623	Adipose_R1_peak_	Distal Intergen	1	50909962	50979920	69959	1	56363	uc007axi.1	-538904	ENSMUSG000	Tmeff2	transmembrane protein with EGF-like and two follistatin-like domains 2
chr1	50372018	50372466	449	450	50372155	31	16.23981	5.75161	12.83549	Adipose_R1_peak_	Distal Intergen	1	50909962	50979920	69959	1	56363	uc007axi.1	-537496	ENSMUSG000	Tmeff2	transmembrane protein with EGF-like and two follistatin-like domains 2
chr1	50372123	50372293	171	172	50372189	28	6.25023	2.67431	3.31626	Adipose_R2_peak_	Distal Intergen	1	50909962	50979920	69959	1	56363	uc007axi.1	-537669	ENSMUSG000	Tmeff2	transmembrane protein with EGF-like and two follistatin-like domains 2
chr1	50372421	50372517	97	98	50372462	27	5.77405	2.5821	2.88921	Adipose_R2_peak_	Distal Intergen	1	50909962	50979920	69959	1	56363	uc007axi.1	-537445	ENSMUSG000	Tmeff2	transmembrane protein with EGF-like and two follistatin-like domains 2
chr1	50372841	50373066	226	227	50372967	21	8.43286	3.95423	5.34896	Adipose_R1_peak_	Distal Intergen	1	50909962	50979920	69959	1	56363	uc007axi.1	-536896	ENSMUSG000	Tmeff2	transmembrane protein with EGF-like and two follistatin-like domains 2
chr1	50373713	50373852	140	141	50373741	16	5.20017	3.05554	2.38734	Adipose_R1_peak_	Distal Intergen	1	50909962	50979920	69959	1	56363	uc007axi.1	-536110	ENSMUSG000	Tmeff2	transmembrane protein with EGF-like and two follistatin-like domains 2
chr1	50374336	50374525	190	191	50374440	43	14.8939	4.05758	11.42017	Adipose_R2_peak_	Distal Intergen	1	50909962	50979920	69959	1	56363	uc007axi.1	-535437	ENSMUSG000	Tmeff2	transmembrane protein with EGF-like and two follistatin-like domains 2
chr1	50374344	50374562	219	220	50374449	29	14.55748	5.39214	11.20652	Adipose_R1_peak_	Distal Intergen	1	50909962	50979920	69959	1	56363	uc007axi.1	-535400	ENSMUSG000	Tmeff2	transmembrane protein with EGF-like and two follistatin-like domains 2
chr1	50663086	50663289	204	205	50663160	24	10.60668	4.49345	7.40961	Adipose_R1_peak_	Distal Intergen	1	50909962	50979920	69959	1	56363	uc007axi.1	-246673	ENSMUSG000	Tmeff2	transmembrane protein with EGF-like and two follistatin-like domains 2
chr1	50663147	50663311	165	166	50663190	36	10.5378	3.41206	7.27949	Adipose_R2_peak_	Distal Intergen	1	50909962	50979920	69959	1	56363	uc007axi.1	-246651	ENSMUSG000	Tmeff2	transmembrane protein with EGF-like and two follistatin-like domains 2
chr1	50710436	50710643	208	209	50710510	20	7.74501	3.7745	4.70467	Adipose_R1_peak_	Distal Intergen	1	50909962	50979920	69959	1	56363	uc007axi.1	-199319	ENSMUSG000	Tmeff2	transmembrane protein with EGF-like and two follistatin-like domains 2
chr1	50710519	50710617	99	100	50710564	26	5.31252	2.48988	2.47999	Adipose_R2_peak_	Distal Intergen	1	50909962	50979920	69959	1	56363	uc007axi.1	-199345	ENSMUSG000	Tmeff2	transmembrane protein with EGF-like and two follistatin-like domains 2
chr1	50711499	50711677	179	180	50711586	20	7.74501	3.7745	4.70467	Adipose_R1_peak_	Distal Intergen	1	50909962	50979920	69959	1	56363	uc007axi.1	-198285	ENSMUSG000	Tmeff2	transmembrane protein with EGF-like and two follistatin-like domains 2
chr1	50711930	50712078	149	150	50712021	18	6.42934	3.41502	3.48999	Adipose_R1_peak_	Distal Intergen	1	50909962	50979920	69959	1	56363	uc007axi.1	-197884	ENSMUSG000	Tmeff2	transmembrane protein with EGF-like and two follistatin-like domains 2
chr1	50713635	50713825	191	192	50713666	18	6.42934	3.41502	3.48999	Adipose_R1_peak_	Distal Intergen	1	50909962	50979920	69959	1	56363	uc007axi.1	-196137	ENSMUSG000	Tmeff2	transmembrane protein with EGF-like and two follistatin-like domains 2
chr1	50743651	50743850	200	201	50743770	27	12.93148	5.03266	9.63827	Adipose_R1_peak_	Distal Intergen	1	50909962	50979920	69959	1	56363	uc007axi.1	-166112	ENSMUSG000	Tmeff2	transmembrane protein with EGF-like and two follistatin-like domains 2
chr1	50876152	50876308	157	158	50876234	26	12.14079	4.85292	8.87838	Adipose_R1_peak_	Distal Intergen	1	50909962	50979920	69959	1	56363	uc007axi.1	-33654	ENSMUSG000	Tmeff2	transmembrane protein with EGF-like and two follistatin-like domains 2
chr1	50876168	50876277	110	111	50876218	29	6.74062	2.76653	3.75989	Adipose_R2_peak_	Distal Intergen	1	50909962	50979920	69959	1	56363	uc007axi.1	-33685	ENSMUSG000	Tmeff2	transmembrane protein with EGF-like and two follistatin-like domains 2
chr1	51103145	51103305	161	162	51103209	24	10.60668	4.49345	7.40961	Adipose_R1_peak_	Intron (uc007a	1	50927523	51187270	259748	1	56363	uc007axj.1	175622	ENSMUSG000	Tmeff2	transmembrane protein with EGF-like and two follistatin-like domains 2
chr1	51436603	51436810	208	209	51436747	23	9.86443	4.31371	6.70247	Adipose_R1_peak_	Distal Intergen	1	51466007	51478399	12393	2	109019	uc007axm.1	41589	ENSMUSG000	Nabp1	nucleic acid binding protein 1
chr1	51715794	51715904	111	112	51715850	26	5.31252	2.48988	2.47999	Adipose_R2_peak_	Distal Intergen	1	51755643	51758022	2380	2	17912	uc029qoh.1	42118	ENSMUSG000	Myo1b	myosin IB
chr1	51715802	51715966	165	166	51715866	21	8.43286	3.95423	5.34896	Adipose_R1_peak_	Distal Intergen	1	51755643	51758022	2380	2	17912	uc029qoh.1	42056	ENSMUSG000	Myo1b	myosin IB
chr1	52370960	52371125	166	167	52371022	40	12.96465	3.78093	9.57654	Adipose_R2_peak_	Distal Intergen	1	52455849	52500448	44600	2	17936	uc007ayh.2	129323	ENSMUSG000	Nab1	Ngfi-A binding protein 1
chr1	52403746	52403956	211	212	52403857	33	17.97508	6.11109	14.52164	Adipose_R1_peak_	Distal Intergen	1	52455849	52500448	44600	2	17936	uc007ayh.2	96492	ENSMUSG000	Nab1	Ngfi-A binding protein 1
chr1	52403803	52403925	123	124	52403871	31	7.7623	2.95097	4.69388	Adipose_R2_peak_	Distal Intergen	1	52455849	52500448	44600	2	17936	uc007ayh.2	96523	ENSMUSG000	Nab1	Ngfi-A binding protein 1
chr1	52943109	52943379	271	272	52943238	54	38.78658	9.88558	34.93639	Adipose_R1_peak_	Intron (uc007a	1	52925127	52952840	27714	2	67080	uc007ayr.1	9461	ENSMUSG000	1700019D03R	RIKEN cDNA 1700019D03 gene
chr1	52943174	52943362	189	190	52943256	41	13.59774	3.87315	10.1803	Adipose_R2_peak_	Intron (uc007a	1	52925127	52952840	27714	2	67080	uc007ayr.1	9478	ENSMUSG000	1700019D03R	RIKEN cDNA 1700019D03 gene
chr1	52979303	52979572	270	271	52979445	49	33.45876	8.98689	29.68925	Adipose_R1_peak_	Distal Intergen	1	52925127	52963260	38134	2	67080	uc007ays.1	-16043	ENSMUSG000	1700019D03R	RIKEN cDNA 1700019D03 gene
chr1	52979339	52979507	169	170	52979436	33	8.83596	3.1354	5.68652	Adipose_R2_peak_	Distal Intergen	1	52925127	52963260	38134	2	67080	uc007ays.1	-16079	ENSMUSG000	1700019D03R	RIKEN cDNA 1700019D03 gene
chr1	52980193	52980379	187	188	52980300	23	9.86443	4.31371	6.70247	Adipose_R1_peak_	Distal Intergen	1	52925127	52963260	38134	2	67080	uc007ays.1	-16933	ENSMUSG000	1700019D03R	RIKEN cDNA 1700019D03 gene
chr1	53130794	53131027	234	235	53130909	46	16.90986	4.33423	13.35898	Adipose_R2_peak_	Distal Intergen	1	53158577	53187617	29041	2	69397	uc007ayu.1	56590	ENSMUSG000	1700019A02R	RIKEN cDNA 1700019A02 gene
chr1	53130833	53130977	145	146	53130908	21	8.43286	3.95423	5.34896	Adipose_R1_peak_	Distal Intergen	1	53158577	53187617	29041	2	69397	uc007ayu.1	56640	ENSMUSG000	1700019A02R	RIKEN cDNA 1700019A02 gene
chr1	53359415	53359587	173	174	53359479	20	7.74501	3.7745	4.70467	Adipose_R1_peak_	Distal Intergen	1	53325507	53352752	27246	2	70396	uc007aze.2	-6663	ENSMUSG000	Asnsd1	asparagine synthetase domain containing 1
chr1	53372303	53372538	236	237	53372427	30	15.39182	5.57188	12.01394	Adipose_R1_peak_	Distal Intergen	1	53325507	53352752	27246	2	70396	uc007aze.2	-19551	ENSMUSG000	Asnsd1	asparagine synthetase domain containing 1
chr1	53372323	53372543	221	222	53372431	36	10.5378	3.41206	7.27949	Adipose_R2_peak_	Distal Intergen	1	53325507	53352752	27246	2	70396	uc007aze.2	-19571	ENSMUSG000	Asnsd1	asparagine synthetase domain containing 1
chr1	53489047	53489248	202	203	53489121	22	9.13959	4.13397	6.01526	Adipose_R1_peak_	Intron (uc029c	1	53325507	53352752	27246	2	70396	uc007aze.2	-136295	ENSMUSG000	Asnsd1	asparagine synthetase domain containing 1
chr1	53489048	53489239	192	193	53489105	28	6.25023	2.67431	3.31626	Adipose_R2_peak_	Intron (uc029c	1	53325507	53352752	27246	2	70396	uc007aze.2	-136296	ENSMUSG000	Asnsd1	asparagine synthetase domain containing 1
chr1	53602963	53603155	193	194	53603028	32	17.10103	5.93135	13.67137	Adipose_R1_peak_	Intron (uc029c	1	53397002	53706784	309783	2	627872	uc029qom.1	103629	ENSMUSG000	Dnah7a	dynein, axonemal, heavy chain 7A
chr1	53653827	53654048	222	223	53653973	36	20.67077	6.6503	17.14779	Adipose_R1_peak_	Intron (uc029c	1	53397002	53706784	309783	2	627872	uc029qom.1	52736	ENSMUSG000	Dnah7a	dynein, axonemal, heavy chain 7A
chr1	53653830	53654038	209	210	53653974	38	11.72962	3.59649	8.4047	Adipose_R2_peak_	Intron (uc029c	1	53397002	53706784	309783	2	627872	uc029qom.1	52746	ENSMUSG000	Dnah7a	dynein, axonemal, heavy chain 7A
chr1	54198689	54198938	250	251	54198804	51	35.56531	9.34637	31.76235	Adipose_R1_peak_	Distal Intergen	1	53806874	54195034	388161	2	329152	uc007azk.2	-3655	ENSMUSG000	Hecw2	HECT, C2 and WW domain containing E3 ubiquitin protein ligase 2
chr1	54198704	54198892	189	190	54198821	43	14.8939	4.05758	11.42017	Adipose_R2_peak_	Distal Intergen	1	53806874	54195034	388161	2	329152	uc007azk.2	-3670	ENSMUSG000	Hecw2	HECT, C2 and WW domain containing E3 ubiquitin protein ligase 2
chr1	54268446	54268674	229	230	54268558	31	16.23981	5.75161	12.83549	Adipose_R1_peak_	Intron (uc007a	1	54250683	54278961	28279	1	78016	uc007azn.1	17763	ENSMUSG000	Ccdc150	coiled-coil domain containing 150
chr1	54269334	54269606	273	274	54269461	96	59.87514	8.94512	55.61917	Adipose_R2_peak_	Intron (uc007a	1	54250683	54278961	28279	1	78016	uc007azn.1	18651	ENSMUSG000	Ccdc150	coiled-coil domain containing 150
chr1	54269337	54269652	316	317	54269474	107	74.86324	10.78377	70.66652	Adipose_R1_peak_	Intron (uc007a	1	54250683	54278961	28279	1	78016	uc007azn.1	18654	ENSMUSG000	Ccdc150	coiled-coil domain containing 150
chr1	54316610	54316821	212	213	54316700	80	69.31596	14.55877	65.15641	Adipose_R1_peak_	Intron (uc007a	1	54250683	54278961	28279	1	78016	uc007azn.1	65927	ENSMUSG000	Ccdc150	coiled-coil domain containing 150
chr1	54316619	54316813	195	196	54316683	62	28.93052	5.80972	25.0743	Adipose_R2_peak_	Intron (uc007a	1	54250683	54278961	28279	1	78016	uc007azn.1	65936	ENSMUSG000	Ccdc150	coiled-coil domain containing 150
chr1	55443807	55444217	411	412	55443909	32	7.84946	2.92451	4.77766	Adipose_R2_peak_	Intron (uc007b	1	55405946	55754285	348340	1	227120	uc007ban.2	37861	ENSMUSG000	Plcl1	phospholipase C-like 1
chr1	55477862	55478083	222	223	55477979	50	19.72356	4.7031	16.08069	Adipose_R2_peak_	Intron (uc007b	1	55405946	55754285	348340	1	227120	uc007ban.2	71916	ENSMUSG000	Plcl1	phospholipase C-like 1
chr1	55477871	55478103	233	234	55477998	41	25.38991	7.54899	21.76305	Adipose_R1_peak_	Intron (uc007b	1	55405946	55754285	348340	1	227120	uc007ban.2	71925	ENSMUSG000	Plcl1	phospholipase C-like 1
chr1	55773817	55773989	173	174	55773889	24	10.60668	4.49345	7.40961	Adipose_R1_peak_	Distal Intergen	1	55931241	55972098	40858	1	329159	uc007bao.2	-157252	ENSMUSG000	9130227L01Ri	RIKEN cDNA 9130227L01 gene
chr1	55926033	55926257	225	226	55926127	37	11.12817	3.50427	7.83613	Adipose_R2_peak_	Distal Intergen	1	55931241	55972098	40858	1	329159	uc007bao.2	-4984	ENSMUSG000	9130227L01Ri	RIKEN cDNA 9130227L01 gene
chr1	55927485	55927743	259	260	55927614	45	29.34988	8.26794	25.64927	Adipose_R1_peak_	Distal Intergen	1	55931241	55972098	40858	1	329159	uc007bao.2	-3498	ENSMUSG000	9130227L01Ri	RIKEN cDNA 9130227L01 gene
chr1	55927516	55927678	163	164	55927603	36	10.5378	3.41206	7.27949	Adipose_R2_peak_	Distal Intergen	1	55931241	55972098	40858	1	329159	uc007bao.2	-3563	ENSMUSG000	9130227L01Ri	RIKEN cDNA 9130227L01 gene
chr1	56012427	56012587	161	162	56012496	31	7.7623	2.95097	4.69388	Adipose_R2_peak_	Distal Intergen	1	56018978	56020203	1226	1	69356	uc007baq.1	-6391	ENSMUSG000	1700003I22Ri	RIKEN cDNA 1700003I22 gene
chr1	56138656	56138929	274	275	56138799	44	28.34544	8.08821	24.66239	Adipose_R1_peak_	Distal Intergen	1	56018978	56020203	1226	1	69356	uc007baq.1	119678	ENSMUSG000	1700003I22Ri	RIKEN cDNA 1700003I22 gene
chr1	56138721	56138866	146	147	56138790	34	9.39139	3.22762	6.20474	Adipose_R2_peak_	Distal Intergen	1	56018978	56020203	1226	1	69356	uc007baq.1	119743	ENSMUSG000	1700003I22Ri	RIKEN cDNA 1700003I22 gene
chr1	56143159	56143399	241	242	56143280	113	101.7189	16.71847	97.37908	Adipose_R1_peak_	Distal Intergen	1	56018978	56020203	1226	1	69356	uc007baq.1	124181	ENSMUSG000	1700003I22Ri	RIKEN cDNA 1700003I22 gene
chr1	56143168	56143398	231	232	56143284	84	48.25301	7.83851	44.11785	Adipose_R2_peak_	Distal Intergen	1	56018978	56020203	1226	1	69356	uc007baq.1	124190	ENSMUSG000	1700003I22Ri	RIKEN cDNA 1700003I22 gene
chr1	56261178	56261337	160	161	56261242	19	7.07687	3.59476	4.08458	Adipose_R1_peak_	Distal Intergen	1	56018978	56020203	1226	1	69356	uc007baq.1	242200	ENSMUSG000	1700003I22Ri	RIKEN cDNA 1700003I22 gene
chr1	56451578	56451711	134	135	56451622	31	7.7623	2.95097	4.69388	Adipose_R2_peak_	Distal Intergen	1	56636051	56637451	1401	2	71066	uc007bar.2	185740	ENSMUSG000	Hsfy2	heat shock transcription factor, Y-linked 2
chr1	56461379	56461615	237	238	56461503	32	17.10103	5.93135	13.67137	Adipose_R1_peak_	Distal Intergen	1	56636051	56637451	1401	2	71066	uc007bar.2	175836	ENSMUSG000	Hsfy2	heat shock transcription factor, Y-linked 2
chr1	56462868	56462999	132	133	56462939	23	9.86443	4.31371	6.70247	Adipose_R1_peak_	Distal Intergen	1	56636051	56637451	1401	2	71066	uc007bar.2	174452	ENSMUSG000	Hsfy2	heat shock transcription factor, Y-linked 2
chr1	56716071	56716205	135	136	56716133	20	7.74501	3.7745	4.70467	Adipose_R1_peak_	Distal Intergen	1	56636051	56637451	1401	2	71066	uc007bar.2	-78620	ENSMUSG000	Hsfy2	heat shock transcription factor, Y-linked 2
chr1	56717677	56717834	158	159	56717745	26	12.14079	4.85292	8.87838	Adipose_R1_peak_	Distal Intergen	1	56636051	56637451	1401	2	71066	uc007bar.2	-80226	ENSMUSG000	Hsfy2	heat shock transcription factor, Y-linked 2
chr1	56776460	56776692	233	234	56776568	24	10.60668	4.49345	7.40961	Adipose_R1_peak_	Distal Intergen	1	56636051	56637451	1401	2	71066	uc007bar.2	-139009	ENSMUSG000	Hsfy2	heat shock transcription factor, Y-linked 2
chr1	58939614	58939776	163	164	58939705	21	8.43286	3.95423	5.34896	Adipose_R1_peak_	Intron (uc007b	1	58900450	58973482	73033	2	70827	uc007bcv.2	33706	ENSMUSG000	Trak2	trafficking protein, kinesin binding 2
chr1	58939647	58939744	98	99	58939718	27	5.77405	2.5821	2.88921	Adipose_R2_peak_	Intron (uc007b	1	58900450	58973482	73033	2	70827	uc007bcv.2	33738	ENSMUSG000	Trak2	trafficking protein, kinesin binding 2
chr1	58941875	58942095	221	222	58941978	60	27.32259	5.62528	23.49644	Adipose_R2_peak_	Intron (uc007b	1	58900450	58973482	73033	2	70827	uc007bcv.2	31387	ENSMUSG000	Trak2	trafficking protein, kinesin binding 2
chr1	58941907	58942100	194	195	58941986	41	14.98472	4.22829	11.63136	Adipose_R1_peak_	Intron (uc007b	1	58900450	58973482	73033	2	70827	uc007bcv.2	31382	ENSMUSG000	Trak2	trafficking protein, kinesin binding 2
chr1	60636027	60636269	243	244	60636152	34	18.86162	6.29083	15.38482	Adipose_R1_peak_	Distal Intergen	1	60483185	60566765	83581	2	77300	uc007bet.2	-69262	ENSMUSG000	Raph1	Ras association (RalGDS/AF-6) and pleckstrin homology domains 1
chr1	60636080	60636209	130	131	60636155	28	6.25023	2.67431	3.31626	Adipose_R2_peak_	Distal Intergen	1	60483185	60566765	83581	2	77300	uc007bet.2	-69315	ENSMUSG000	Raph1	Ras association (RalGDS/AF-6) and pleckstrin homology domains 1
chr1	60689883	60690101	219	220	60689981	29	14.55748	5.39214	11.20652	Adipose_R1_peak_	Distal Intergen	1	60746388	60764626	18239	1	12487	uc007bev.2	-56287	ENSMUSG000	Cd28	CD28 antigen
chr1	60690388	60690603	216	217	60690515	43	27.35048	7.90847	23.68561	Adipose_R1_peak_	Distal Intergen	1	60746388	60764626	18239	1	12487	uc007bev.2	-55785	ENSMUSG000	Cd28	CD28 antigen
chr1	60690392	60690599	208	209	60690491	42	14.2409	3.96536	10.79479	Adipose_R2_peak_	Distal Intergen	1	60746388	60764626	18239	1	12487	uc007bev.2	-55789	ENSMUSG000	Cd28	CD28 antigen
chr1	60693003	60693843	841	842	60693152	72	59.47482	13.12087	55.39848	Adipose_R1_peak_	Distal Intergen	1	60746388	60764626	18239	1	12487	uc007bev.2	-52545	ENSMUSG000	Cd28	CD28 antigen
chr1	60693029	60693500	472	473	60693155	64	30.56565	5.99415	26.6807	Adipose_R2_peak_	Distal Intergen	1	60746388	60764626	18239	1	12487	uc007bev.2	-52888	ENSMUSG000	Cd28	CD28 antigen
chr1	61210815	61211066	252	253	61210939	33	17.97508	6.11109	14.52164	Adipose_R1_peak_	Distal Intergen	1	61378432	61395904	17473	1	77341	uc029qot.1	-167366	ENSMUSG000	9530026F06Ri	RIKEN cDNA 9530026F06 gene
chr1	61212924	61213151	228	229	61213033	35	19.76029	6.47056	16.26028	Adipose_R1_peak_	Distal Intergen	1	61378432	61395904	17473	1	77341	uc029qot.1	-165281	ENSMUSG000	9530026F06Ri	RIKEN cDNA 9530026F06 gene
chr1	61212971	61213141	171	172	61213030	35	9.95877	3.31984	6.73562	Adipose_R2_peak_	Distal Intergen	1	61378432	61395904	17473	1	77341	uc029qot.1	-165291	ENSMUSG000	9530026F06Ri	RIKEN cDNA 9530026F06 gene
chr1	61288841	61289013	173	174	61288903	18	6.42934	3.41502	3.48999	Adipose_R1_peak_	Distal Intergen	1	61378432	61395904	17473	1	77341	uc029qot.1	-89419	ENSMUSG000	9530026F06Ri	RIKEN cDNA 9530026F06 gene
chr1	61288892	61289001	110	111	61288936	27	5.77405	2.5821	2.88921	Adipose_R2_peak_	Distal Intergen	1	61378432	61395904	17473	1	77341	uc029qot.1	-89431	ENSMUSG000	9530026F06Ri	RIKEN cDNA 9530026F06 gene
chr1	61364690	61364895	206	207	61364799	30	15.39182	5.57188	12.01394	Adipose_R1_peak_	Distal Intergen	1	61378432	61395904	17473	1	77341	uc029qot.1	-13537	ENSMUSG000	9530026F06Ri	RIKEN cDNA 9530026F06 gene
chr1	61519172	61519340	169	170	61519234	25	11.36566	4.67319	8.13482	Adipose_R1_peak_	Distal Intergen	1	61638824	61769859	131036	1	72823	uc007bfc.2	-119484	ENSMUSG000	Pard3b	par-3 family cell polarity regulator beta
chr1	61526718	61526890	173	174	61526779	16	5.20017	3.05554	2.38734	Adipose_R1_peak_	Distal Intergen	1	61638824	61769859	131036	1	72823	uc007bfc.2	-111934	ENSMUSG000	Pard3b	par-3 family cell polarity regulator beta
chr1	61570054	61570323	270	271	61570213	20	7.74501	3.7745	4.70467	Adipose_R1_peak_	Distal Intergen	1	61638824	61769859	131036	1	72823	uc007bfc.2	-68501	ENSMUSG000	Pard3b	par-3 family cell polarity regulator beta
chr1	61644372	61644543	172	173	61644437	19	7.07687	3.59476	4.08458	Adipose_R1_peak_	Intron (uc007b	1	61638824	61769859	131036	1	72823	uc007bfc.2	5548	ENSMUSG000	Pard3b	par-3 family cell polarity regulator beta
chr1	61671547	61671760	214	215	61671647	29	14.55748	5.39214	11.20652	Adipose_R1_peak_	Intron (uc007b	1	61638824	61769859	131036	1	72823	uc007bfc.2	32723	ENSMUSG000	Pard3b	par-3 family cell polarity regulator beta
chr1	61894812	61895048	237	238	61894926	41	25.38991	7.54899	21.76305	Adipose_R1_peak_	Intron (uc007b	1	62010307	62642284	631978	1	72823	uc011wma.1	-115259	ENSMUSG000	Pard3b	par-3 family cell polarity regulator beta
chr1	62100050	62100326	277	278	62100192	33	17.97508	6.11109	14.52164	Adipose_R1_peak_	Intron (uc007b	1	62010307	62642284	631978	1	72823	uc011wma.1	89743	ENSMUSG000	Pard3b	par-3 family cell polarity regulator beta
chr1	62100140	62100262	123	124	62100194	29	6.74062	2.76653	3.75989	Adipose_R2_peak_	Intron (uc007b	1	62010307	62642284	631978	1	72823	uc011wma.1	89833	ENSMUSG000	Pard3b	par-3 family cell polarity regulator beta

chr1	62221526	62221658	133	134	62221588	20	7.74501	3.7745	4.70467	Adipose_R1_peak_7	Intron (uc007b	1	62010307	62642284	631978	1	72823	uc011wma.1	211219	ENSMUSG000	Pard3b	par-3 family cell polarity regulator beta
chr1	62450883	62451015	133	134	62450908	16	5.20017	3.05554	2.38734	Adipose_R1_peak_7	Intron (uc007b	1	62703317	62739932	36616	1	18187	uc007bfi.1	-252302	ENSMUSG000	Nrp2	neuropilin 2
chr1	62674130	62674391	262	263	62674258	40	24.42476	7.36925	20.81738	Adipose_R1_peak_7	Distal Interger	1	62703317	62739932	36616	1	18187	uc007bfi.1	-28926	ENSMUSG000	Nrp2	neuropilin 2
chr1	62674173	62674327	155	156	62674257	36	10.5378	3.41206	7.27949	Adipose_R2_peak_9	Distal Interger	1	62703317	62739932	36616	1	18187	uc007bfi.1	-28990	ENSMUSG000	Nrp2	neuropilin 2
chr1	62675583	62675851	269	270	62675709	45	29.34988	8.26794	25.64927	Adipose_R1_peak_7	Distal Interger	1	62703317	62739932	36616	1	18187	uc007bfi.1	-27466	ENSMUSG000	Nrp2	neuropilin 2
chr1	62676921	62677130	210	211	62677002	28	13.73721	5.2124	10.41463	Adipose_R1_peak_7	Distal Interger	1	62703317	62739932	36616	1	18187	uc007bfi.1	-26187	ENSMUSG000	Nrp2	neuropilin 2
chr1	62676964	62677090	127	128	62677012	33	8.83596	3.1354	5.68652	Adipose_R2_peak_9	Distal Interger	1	62703317	62739932	36616	1	18187	uc007bfi.1	-26227	ENSMUSG000	Nrp2	neuropilin 2
chr1	62711272	62711473	202	203	62711355	33	17.97508	6.11109	14.52164	Adipose_R1_peak_7	Intron (uc007b	1	62703317	62739932	36616	1	18187	uc007bfi.1	7955	ENSMUSG000	Nrp2	neuropilin 2
chr1	62722437	62722571	135	136	62722480	29	6.74062	2.76653	3.75989	Adipose_R2_peak_9	Intron (uc007b	1	62703317	62739932	36616	1	18187	uc007bfi.1	19120	ENSMUSG000	Nrp2	neuropilin 2
chr1	63629824	63630140	317	318	63630071	36	10.5898	3.42556	7.40961	Adipose_R1_peak_7	Intron (uc007b	1	63622851	63686927	64077	2	241073	uc007bgi.1	56787	ENSMUSG000	Dytn	dystrotelin
chr1	63629879	63630116	238	239	63630049	33	5.92758	2.40055	3.03692	Adipose_R2_peak_9	Intron (uc007b	1	63622851	63686927	64077	2	241073	uc007bgi.1	56811	ENSMUSG000	Dytn	dystrotelin
chr1	63630296	63630708	413	414	63630507	90	20.87999	3.18617	17.20913	Adipose_R2_peak_9	Intron (uc007b	1	63622851	63686927	64077	2	241073	uc007bgi.1	56219	ENSMUSG000	Dytn	dystrotelin
chr1	63630334	63630659	326	327	63630469	50	8.37464	2.43843	5.32764	Adipose_R1_peak_7	Intron (uc007b	1	63622851	63686927	64077	2	241073	uc007bgi.1	56268	ENSMUSG000	Dytn	dystrotelin
chr1	63674178	63674355	178	179	63674259	26	12.14079	4.85292	8.87838	Adipose_R1_peak_7	Intron (uc007b	1	63622851	63686927	64077	2	241073	uc007bgi.1	12572	ENSMUSG000	Dytn	dystrotelin
chr1	64119427	64119563	137	138	64119450	25	4.86612	2.39766	2.09018	Adipose_R2_peak_9	Promoter (1-2	1	64035671	64121389	85719	2	93691	uc007bgo.2	1826	ENSMUSG000	Klf7	Kruppel-like factor 7 (ubiquitous)
chr1	64119571	64119712	142	143	64119702	20	7.74501	3.7745	4.70467	Adipose_R1_peak_7	Promoter (1-2	1	64035671	64121389	85719	2	93691	uc007bgo.2	1677	ENSMUSG000	Klf7	Kruppel-like factor 7 (ubiquitous)
chr1	64162904	64163050	147	148	64162963	21	8.43286	3.95423	5.34896	Adipose_R1_peak_7	Distal Interger	1	64035671	64121426	85756	2	93691	uc007bgp.2	-41478	ENSMUSG000	Klf7	Kruppel-like factor 7 (ubiquitous)
chr1	64261023	64261461	439	440	64261180	54	38.78658	9.88558	34.93639	Adipose_R1_peak_7	Distal Interger	1	64035671	64121426	85756	2	93691	uc007bgp.2	-139597	ENSMUSG000	Klf7	Kruppel-like factor 7 (ubiquitous)
chr1	64261108	64261337	230	231	64261204	38	11.72962	3.59649	8.4047	Adipose_R2_peak_9	Distal Interger	1	64035671	64121426	85756	2	93691	uc007bgp.2	-139682	ENSMUSG000	Klf7	Kruppel-like factor 7 (ubiquitous)
chr1	65413932	65414124	193	194	65414057	37	11.12817	3.50427	7.83613	Adipose_R2_peak_9	Distal Interger	1	65311257	65389244	77988	1	213527	uc007bhu.1	102675	ENSMUSG000	Pth2r	parathyroid hormone 2 receptor
chr1	65417684	65417824	141	142	65417761	31	7.7623	2.95097	4.69388	Adipose_R2_peak_9	Distal Interger	1	65311257	65389244	77988	1	213527	uc007bhu.1	106427	ENSMUSG000	Pth2r	parathyroid hormone 2 receptor
chr1	65470592	65470816	225	226	65470684	50	34.50784	9.16663	30.72138	Adipose_R1_peak_7	Distal Interger	1	65311257	65389244	77988	1	213527	uc007bhu.1	159335	ENSMUSG000	Pth2r	parathyroid hormone 2 receptor
chr1	65528887	65529123	237	238	65529013	63	44.49519	9.98591	40.57404	Adipose_R1_peak_7	Distal Interger	1	65311257	65389244	77988	1	213527	uc007bhu.1	217630	ENSMUSG000	Pth2r	parathyroid hormone 2 receptor
chr1	65528893	65529128	236	237	65529010	60	27.32259	5.62528	23.49644	Adipose_R2_peak_9	Distal Interger	1	65311257	65389244	77988	1	213527	uc007bhu.1	217636	ENSMUSG000	Pth2r	parathyroid hormone 2 receptor
chr1	65678657	65678815	159	160	65678741	17	5.80341	3.23528	2.92378	Adipose_R1_peak_7	Distal Interger	1	65926522	65928304	1783	1	12969	uc007bhv.1	-247707	ENSMUSG000	Crygf	crystallin, gamma F
chr1	65709975	65710144	170	171	65710069	29	14.55748	5.39214	11.20652	Adipose_R1_peak_7	Distal Interger	1	65926522	65928304	1783	1	12969	uc007bhv.1	-216378	ENSMUSG000	Crygf	crystallin, gamma F
chr1	65710036	65710146	111	112	65710100	31	7.7623	2.95097	4.69388	Adipose_R2_peak_9	Distal Interger	1	65926522	65928304	1783	1	12969	uc007bhv.1	-216376	ENSMUSG000	Crygf	crystallin, gamma F
chr1	65910126	65910239	114	115	65910186	28	6.25023	2.67431	3.31626	Adipose_R2_peak_9	Distal Interger	1	65926522	65928304	1783	1	12969	uc007bhv.1	-16283	ENSMUSG000	Crygf	crystallin, gamma F
chr1	65929884	65930076	193	194	65929906	16	5.20017	3.05554	2.38734	Adipose_R1_peak_7	Distal Interger	1	65926522	65928304	1783	1	12969	uc007bhv.1	3362	ENSMUSG000	Crygf	crystallin, gamma F
chr1	65976115	65976369	255	256	65976250	34	18.86162	6.29083	15.38482	Adipose_R1_peak_7	Distal Interger	1	65926522	65928304	1783	1	12969	uc007bhv.1	49593	ENSMUSG000	Crygf	crystallin, gamma F
chr1	65979582	65980006	425	426	65979708	30	15.39182	5.57188	12.01394	Adipose_R1_peak_7	Distal Interger	1	65926522	65928304	1783	1	12969	uc007bhv.1	53060	ENSMUSG000	Crygf	crystallin, gamma F
chr1	66037842	66038113	272	273	66038013	45	29.34988	8.26794	25.64927	Adipose_R1_peak_7	Distal Interger	1	65926522	65928304	1783	1	12969	uc007bhv.1	111320	ENSMUSG000	Crygf	crystallin, gamma F
chr1	66037932	66038081	150	151	66038022	34	9.39139	3.22762	6.20474	Adipose_R2_peak_1	Distal Interger	1	65926522	65928304	1783	1	12969	uc007bhv.1	111410	ENSMUSG000	Crygf	crystallin, gamma F
chr1	66049776	66050044	269	270	66049914	60	45.43621	10.96401	41.50272	Adipose_R1_peak_7	Distal Interger	1	65926522	65928304	1783	1	12969	uc007bhv.1	123254	ENSMUSG000	Crygf	crystallin, gamma F
chr1	66049814	66050005	192	193	66049903	46	16.90986	4.33423	13.35898	Adipose_R2_peak_1	Distal Interger	1	65926522	65928304	1783	1	12969	uc007bhv.1	123292	ENSMUSG000	Crygf	crystallin, gamma F
chr1	66050564	66050811	248	249	66050693	32	17.10103	5.93135	13.67137	Adipose_R1_peak_7	Distal Interger	1	65926522	65928304	1783	1	12969	uc007bhv.1	124042	ENSMUSG000	Crygf	crystallin, gamma F
chr1	66052235	66052550	316	317	66052385	56	39.65759	9.76806	35.80355	Adipose_R1_peak_7	Distal Interger	1	66175329	66442583	267255	1	17756	uc007bhw.1	-122779	ENSMUSG000	Map2	microtubule-associated protein 2
chr1	66052305	66052482	178	179	66052381	46	16.90986	4.33423	13.35898	Adipose_R2_peak_1	Distal Interger	1	66175329	66442583	267255	1	17756	uc007bhw.1	-122847	ENSMUSG000	Map2	microtubule-associated protein 2
chr1	66053476	66053914	439	440	66053567	26	12.14079	4.85292	8.87838	Adipose_R1_peak_7	Distal Interger	1	66175329	66442583	267255	1	17756	uc007bhw.1	-121415	ENSMUSG000	Map2	microtubule-associated protein 2
chr1	66053542	66053659	118	119	66053579	30	7.24477	2.85875	4.21917	Adipose_R2_peak_1	Distal Interger	1	66175329	66442583	267255	1	17756	uc007bhw.1	-121670	ENSMUSG000	Map2	microtubule-associated protein 2
chr1	66071218	66071437	220	221	66071342	39	23.47001	7.18952	19.88325	Adipose_R1_peak_7	Distal Interger	1	66175329	66442583	267255	1	17756	uc007bhw.1	-103892	ENSMUSG000	Map2	microtubule-associated protein 2
chr1	66071259	66071403	145	146	66071326	30	7.24477	2.85875	4.21917	Adipose_R2_peak_1	Distal Interger	1	66175329	66442583	267255	1	17756	uc007bhw.1	-103926	ENSMUSG000	Map2	microtubule-associated protein 2
chr1	66177563	66177778	216	217	66177766	21	8.43286	3.95423	5.34896	Adipose_R1_peak_7	Promoter (2-3	1	66175329	66442583	267255	1	17756	uc007bhw.1	2234	ENSMUSG000	Map2	microtubule-associated protein 2
chr1	66547599	66547848	250	251	66547724	48	32.41826	8.80716	28.66523	Adipose_R1_peak_7	Intron (uc007b	1	66468447	66486190	17744	1	329178	uc007bif.2	79152	ENSMUSG000	Unc80	unc-80, NALCN activator
chr1	66547625	66547801	177	178	66547710	40	12.96465	3.78093	9.57654	Adipose_R2_peak_1	Intron (uc007b	1	66468447	66486190	17744	1	329178	uc007bif.2	79178	ENSMUSG000	Unc80	unc-80, NALCN activator
chr1	66579572	66579964	393	394	66579758	130	80.43127	9.16049	76.01889	Adipose_R2_peak_1	Intron (uc007b	1	66679564	66699148	19585	1	329178	uc007bii.2	-99600	ENSMUSG000	Unc80	unc-80, NALCN activator
chr1	66579586	66579999	414	415	66579778	145	110.4869	12.89032	106.1089	Adipose_R1_peak_7	Intron (uc007b	1	66679564	66699148	19585	1	329178	uc007bii.2	-99565	ENSMUSG000	Unc80	unc-80, NALCN activator
chr1	66583807	66584016	210	211	66583917	34	9.39139	3.22762	6.20474	Adipose_R2_peak_1	Intron (uc007b	1	66679564	66699148	19585	1	329178	uc007bii.2	-95548	ENSMUSG000	Unc80	unc-80, NALCN activator
chr1	66583821	66584093	273	274	66583976	28	13.73721	5.2124	10.41463	Adipose_R1_peak_7	Intron (uc007b	1	66679564	66699148	19585	1	329178	uc007bii.2	-95471	ENSMUSG000	Unc80	unc-80, NALCN activator
chr1	66634613	66634782	170	171	66634686	21	8.43286	3.95423	5.34896	Adipose_R1_peak_7	Intron (uc007b	1	66679564	66699148	19585	1	329178	uc007bii.2	-44782	ENSMUSG000	Unc80	unc-80, NALCN activator
chr1	67168120	67168265	146	147	67168248	16	5.20017	3.05554	2.38734	Adipose_R1_peak_7	Exon (uc007bi	1	67123027	67231267	108241	1	227231	uc007biy.2	45093	ENSMUSG000	Cps1	carbamoyl-phosphate synthetase 1
chr1	67278104	67278347	244	245	67278220	28	13.73721	5.2124	10.41463	Adipose_R1_peak_7	Intron (uc007b	1	67123027	67231267	108241	1	227231	uc007biy.2	155077	ENSMUSG000	Cps1	carbamoyl-phosphate synthetase 1
chr1	67278974	67279239	266	267	67279096	34	18.86162	6.29083	15.38482	Adipose_R1_peak_7	Intron (uc007b	1	67123027	67231267	108241	1	227231	uc007biy.2	155947	ENSMUSG000	Cps1	carbamoyl-phosphate synthetase 1
chr1	67539427	67539684	258	259	67539541	38	22.52591	7.00978	18.96	Adipose_R1_peak_7	Distal Interger	1	67123027	67231267	108241	1	227231	uc007biy.2	416400	ENSMUSG000	Cps1	carbamoyl-phosphate synthetase 1
chr1	67539487	67539657	171	172	67539568	35	9.95877	3.31984	6.73562	Adipose_R2_peak_1	Distal Interger	1	67123027	67231267	108241	1	227231	uc007biy.2	416460	ENSMUSG000	Cps1	carbamoyl-phosphate synthetase 1
chr1	67573061	67573213	153	154	67573105	33	8.83596	3.1354	5.68652	Adipose_R2_peak_1	Distal Interger	1	67123027	67231267	108241	1	227231	uc007biy.2	450034	ENSMUSG000	Cps1	carbamoyl-phosphate synthetase 1
chr1	67587776	67587944	169	170	67587858	30	7.24477	2.85875	4.21917	Adipose_R2_peak_1	Distal Interger	1	67123027	67231267	108241	1	227231	uc007biy.2	464749	ENSMUSG000	Cps1	carbamoyl-phosphate synthetase 1
chr1	67591382	67591571	190	191	67591473	23	9.86443	4.31371	6.70247	Adipose_R1_peak_8	Distal Interger	1	67123027	67231267	108241	1	227231	uc007biy.2	468355	ENSMUSG000	Cps1	carbamoyl-phosphate synthetase 1
chr1	67592133	67592355	223	224	67592221	28	13.73721	5.2124	10.41463	Adipose_R1_peak_8	Distal Interger	1	67123027	67231267	108241	1	227231	uc007biy.2	469106	ENSMUSG000	Cps1	carbamoyl-phosphate synthetase 1
chr1	67592587	67592739	153	154	67592653	20	7.74501	3.7745	4.70467	Adipose_R1_peak_8	Distal Interger	1	67123027	67231267	108241	1	227231	uc007biy.2	469560	ENSMUSG000	Cps1	carbamoyl-phosphate synthetase 1
chr1	67683890	67684111	222	223	67684015	38	22.52591	7.00978	18.96	Adipose_R1_peak_8	Distal Interger	1	68042441	68071688	29248	2	13869	uc007bjc.1	387577	ENSMUSG000	Erbb4	erb-b2 receptor tyrosine kinase 4
chr1	67683932	67684082	151	152	67684033	32	8.29282	3.04319	5.18352	Adipose_R2_peak_1	Distal Interger	1	68042441	68071688	29248	2	13869	uc007bjc.1	387606	ENSMUSG000	Erbb4	erb-b2 receptor tyrosine kinase 4
chr1	67684931	67685329	399	400	67685059	32	17.10103	5.93135	13.67137	Adipose_R1_peak_8	Distal Interger	1	68042441	68071688	29248	2	13869	uc007bjc.1	386359	ENSMUSG000	Erbb4	erb-b2 receptor tyrosine kinase 4
chr1	67685487	67685711	225	226	67685579	32	17.10103	5.93135	13.67137	Adipose_R1_peak_8	Distal Interger	1	68042441	68071688	29248	2	13869	uc007bjc.1	385977	ENSMUSG000	Erbb4	erb-b2 receptor tyrosine kinase 4
chr1	67756248	67756418	171	172	67756322	18	6.42934	3.41502	3.48999	Adipose_R1_peak_8	Distal Interger	1	68042441	68071688	29248	2	13869	uc007bjc.1	315270	ENSMUSG000	Erbb4	erb-b2 receptor tyrosine kinase 4
chr1	68271052	68271182	131	132	68271112	21	8.43286	3.95423	5.34896	Adipose_R1_peak_8	Intron (uc007b	1	68042441	68071688	29248	2	13869	uc007bjc.1	-199364	ENSMUSG000	Erbb4	erb-b2 receptor tyrosine kinase 4
chr1	68406773	68406936	164	165	68406835	23	9.86443	4.31371	6.70247	Adipose_R1_peak_8	Intron (uc007b	1	68042441	68071688	29248	2	13869	uc007bjc.1	-335085	ENSMUSG000	Erbb4	erb-b2 receptor tyrosine kinase 4
chr1	68472704	68472980	277	278	68472853	65	51.17258	11.8627	47.17609	Adipose_R1_peak_8	Intron (uc007b	1	68042441	68071688	29248	2	13869	uc007bjc.1	-401016	ENSMUSG000	Erbb4	erb-b2 receptor tyrosine kinase 4
chr1	68472717	68472958	242	243	68472847	50	19.72356	4.7031	16.08069	Adipose_R2_peak_1	Intron (uc007b	1	68042441	68071688	29248	2	13869	uc007bjc.1	-401029	ENSMUSG000	Erbb4	erb-b2 receptor tyrosine kinase 4
chr1	69456755	69456883	129	130	69456841	29	6.74062	2.76653	3.75989	Adipose_R2_peak_1	Distal Interger	1	69531210	69685960	154751	2	22779	uc007bjd.2	229077	ENSMUSG000	Ikzf2	IKAROS family zinc finger 2
chr1	69661412	69661589	178	179	69661522	22	9.13959	4.13397	6.01526	Adipose_R1_peak_8	Intron (uc007b	1	69531210	69685960	154751	2	22779	uc007bjd.2	24371	ENSMUSG000	Ikzf2	IKAROS family zinc finger 2
chr1	69968045	69968277	233	234	69968165	31	16.23981	5.75161	12.83549	Adipose_R1_peak_8	Intron (uc007b	1	69951615	70725132	773518	1	66722	uc007bji.2	16430	ENSMUSG000	Spag16	sperm associated antigen 16
chr1	70092753	70092936	184	185	70092831	27	12.93148	5.03266	9.63827	Adipose_R1_peak_8	Intron (uc007b	1	69951615	70725132	773518	1	66722	uc007bji.2	141138	ENSMUSG000	Spag16	sperm associated antigen 16
chr1	70119326	70119490	165	166	70119397	23	9.86443	4.31371	6.70247	Adipose_R1_peak_8	Intron (uc007b	1	69951615	70725132	773518	1	66722	uc007bji.2	167711	ENSMUSG000	Spag16	sperm associated antigen 16
chr1	70241119	70241292	174	175	70241201	29	14.55748	5.39214	11.20652	Adipose_R1_peak_8	Intron (uc007b	1	69951615	70725132	773518	1	66722	uc007bji.2	289504	ENSMUSG000	Spag16	sperm associated antigen 16
chr1	70241148	70241251	104	105	70241192	30	7.24477	2.85875	4.21917	Adipose_R2_peak_1	Intron (uc007b	1	69951615	70725132	773518	1	66722	uc007bji.2	289533	ENSMUSG000	Spag16	sperm associated antigen 16
chr1	70546115	70546367	253	254	70546239	34	18.86162	6.29083	15.38482	Adipose_R1_peak_8	Intron (uc007b	1	70725715	70732202	6488	1	320460	uc007bjj.1	-179348	ENSMUSG000	Vwc2l	von Willebrand factor C domain-containing protein 2-like
chr1	70546185	70546291	107	108	70546235	29	6.74062	2.76653	3.75989	Adipose_R2_peak_1	Intron (uc007b	1	70725715	70732202	6488	1	320460	uc007bjj.1	-179424	ENSMUSG000	Vwc2l	von Willebrand factor C domain-containing protein 2-like
chr1	70631865	70632125	261	262	70632007	43	27.35048	7.90847	23.68561	Adipose_R1_peak_8	Intron (uc007b	1	70725715	70732202	6488	1	320460	uc007bjj.1	-93590	ENSMUSG000	Vwc2l	von Willebrand factor C domain-containing protein 2-like
chr1	70631963	70632101	139	140	70632023	33	8.83596	3.1354	5.68652	Adipose_R2_peak_1	Intron (uc007b	1	70725715	70732202	6488	1	320460	uc007bjj.1	-93614	ENSMUSG000	Vwc2l	von Willebrand factor C domain-containing protein 2-like
chr1	70689168	70689369	202	203	70689276	21	8.43286	3.95423	5.34896	Adipose_R1_peak_8	Intron (uc007b	1	70725715	70732202	6488	1	320460	uc007bjj.1	-36346	ENSMUSG000	Vwc2l	von Willebrand factor C domain-containing protein 2-like
chr1	70873274	70873505	232	233	70873375	29	14.55748	5.39214	11.20652	Adipose_R1_peak_8	Intron (uc007b	1	70728699	70885397	156699	1	320460	uc011wmp.1	144575	ENSMUSG000	Vwc2l	von Willebrand factor C domain-containing protein 2-like
chr1	70974643	70974778	136	137	70974702	21	8.43286	3.95423	5.34896	Adipose_R1_peak_8	Distal Interger	1	71027535	71102972	75438	2	12021	uc007bjm.2	128194	ENSMUSG000	Bard1	BRCA1 associated RING domain 1
chr1	70984331	70984834	504	505	70984461	72	59.47482	13.12087	55.39848	Adipose_R1_peak_8	Distal Interger	1	71027535	71102972	75438	2	12021	uc007bjm.2	118138	ENSMUSG000	Bard1	BRCA1 associated RING domain 1
chr1	70984342	70984726	385	386	70984468	57	24.96362	5.34863	21.18596	Adipose_R2_peak_1	Distal Interger	1	71027535	71102972	75438	2	12021	uc007bjm.2	118246	ENSMUSG000	Bard1	BRCA1 associated RING domain 1
chr1	71080031	71080177	147	148	71080144	38	11.72962	3.59649	8.4047	Adipose_R2_peak_1	Intron (uc007b	1	71027535	71102972	75438	2	12021	uc007bjm.2	22795	ENSMUSG000	Bard1	BRCA1 associated RING domain 1
chr1	71080032	71080180	149	150	71080154	26	12.14079	4.85292	8.87838	Adipose_R1_peak_8	Intron (uc007b	1	71027535	71102972	75438	2	12021	uc007bjm.2	22792	ENSMUSG000	Bard1	BRCA1 associated RING domain 1
chr1	71083705	71083852	148	149	71083781	34	9.39139	3.22762	6.20474	Adipose_R2_peak_1	Intron (uc007b	1	71027535	71102972	75438	2	12021	uc007bjm.2	19120	ENSMUSG000	Bard1	BRCA1 associated RING domain 1
chr1	71084333	71084606	274	275	71084452	60	45.43621	10.96401	41.50272	Adipose_R1_peak_8	Intron (uc007b	1	71027535	71102972	75438	2	12021	uc007bjm.2	18366	ENSMUSG000	Bard1	BRCA1 associated RING domain 1
chr1	71084334	71084544	211	212	71084457	53	21.92215	4.97976	18.21755	Adipose_R2_peak_1	Intron (uc007b	1	71027535	71102972	75438	2	12021	uc007bjm.2	18428	ENSMUSG000	Bard1	BRCA1 associated RING domain 1
chr1	71255376	71255605	230	231	71255474	32	17.10103	5.93135	13.67137	Adipose_R1_peak_8	Intron (uc011v	1	71290013	71307126	17114	2	74591	uc007bjo.2	51521	ENSMUSG000	Abca12	ATP-binding cassette, sub-family A (ABC1), member 12
chr1	71255438	71255589	152	153	71255481	39	12.34187	3.68871	8.98501	Adipose_R2_peak_1	Intron (uc011v	1	71290013	71307126	17114	2	74591	uc007bjo.2	51537	ENSMUSG000	Abca12	ATP-binding cassette, sub-family A (ABC1), member 12
chr1	71368075	71368231	157	158	71368160	19	7.07687	3.59476	4.08458	Adipose_R1_peak_8	Intron (uc011v	1	71243090	71414910	171821	2	74591	uc011wmq.1	46679	ENSMUSG000	Abca12	ATP-binding cassette, sub-family A (ABC1), member 12
chr1	71452128	71452396	269	270	71452250	42	20.84566	5.81671	17.32188	Adipose_R1_peak_8	Distal Interger	1	71243090	71414910	171821	2	74591	uc011wmq.1	-37218	ENSMUSG000	Abca12	ATP-binding cassette, sub-family A (ABC1), member 12

chr1	71452208	71452342	135	136	71452264	32	8.29282	3.04319	5.18352	Adipose_R2_peak_1	Distal Interger	1	71243090	71414910	171821	2	74591	uc011wmq.1	-37298	ENSMUSG000	Abca12	ATP-binding cassette, sub-family A (ABC1), member 12
chr1	73294572	73294763	192	193	73294648	26	12.14079	4.85292	8.87838	Adipose_R1_peak_8	Distal Interger	1	73398968	73430779	31812	1	78718	uc029qpc.1	-104205	ENSMUSG000	D530049I02Ri	RIKEN cDNA D530049I02 gene
chr1	73296238	73296487	250	251	73296372	59	39.88495	9.24359	36.02039	Adipose_R1_peak_8	Distal Interger	1	73398968	73430779	31812	1	78718	uc029qpc.1	-102481	ENSMUSG000	D530049I02Ri	RIKEN cDNA D530049I02 gene
chr1	73296272	73296479	208	209	73296361	50	19.72356	4.7031	16.08069	Adipose_R2_peak_1	Distal Interger	1	73398968	73430779	31812	1	78718	uc029qpc.1	-102489	ENSMUSG000	D530049I02Ri	RIKEN cDNA D530049I02 gene
chr1	73297273	73297615	343	344	73297468	80	67.96437	14.07866	63.81916	Adipose_R1_peak_8	Distal Interger	1	73398968	73430779	31812	1	78718	uc029qpc.1	-101353	ENSMUSG000	D530049I02Ri	RIKEN cDNA D530049I02 gene
chr1	73297339	73297565	227	228	73297472	54	22.67101	5.07198	18.94758	Adipose_R2_peak_1	Distal Interger	1	73398968	73430779	31812	1	78718	uc029qpc.1	-101403	ENSMUSG000	D530049I02Ri	RIKEN cDNA D530049I02 gene
chr1	73475218	73475421	204	205	73475338	30	15.39182	5.57188	12.01394	Adipose_R1_peak_8	Distal Interger	1	73391385	73407569	16185	2	723792	uc007blb.1	-67649	NA	Pinc	pregnancy induced noncoding RNA
chr1	73475283	73475400	118	119	73475355	30	7.24477	2.85875	4.21917	Adipose_R2_peak_1	Distal Interger	1	73391385	73407569	16185	2	723792	uc007blb.1	-67714	NA	Pinc	pregnancy induced noncoding RNA
chr1	73549968	73550227	260	261	73550099	39	23.47001	7.18952	19.88325	Adipose_R1_peak_8	Distal Interger	1	73391385	73407569	16185	2	723792	uc007blb.1	-142399	NA	Pinc	pregnancy induced noncoding RNA
chr1	73550038	73550156	119	120	73550100	30	7.24477	2.85875	4.21917	Adipose_R2_peak_1	Distal Interger	1	73391385	73407569	16185	2	723792	uc007blb.1	-142469	NA	Pinc	pregnancy induced noncoding RNA
chr1	73552246	73552451	206	207	73552333	29	14.55748	5.39214	11.20652	Adipose_R1_peak_8	Distal Interger	1	73391385	73407569	16185	2	723792	uc007blb.1	-144677	NA	Pinc	pregnancy induced noncoding RNA
chr1	73886099	73886355	257	258	73886229	35	19.76029	6.47056	16.26028	Adipose_R1_peak_8	Distal Interger	1	73618381	73864466	246086	2	109198	uc007bld.2	-21633	ENSMUSG000	6030407O03R	RIKEN cDNA 6030407O03 gene
chr1	73886165	73886276	112	113	73886220	32	8.29282	3.04319	5.18352	Adipose_R2_peak_1	Distal Interger	1	73618381	73864466	246086	2	109198	uc007bld.2	-21699	ENSMUSG000	6030407O03R	RIKEN cDNA 6030407O03 gene
chr1	73887223	73887376	154	155	73887299	20	7.74501	3.7745	4.70467	Adipose_R1_peak_8	Distal Interger	1	73618381	73864466	246086	2	109198	uc007bld.2	-22757	ENSMUSG000	6030407O03R	RIKEN cDNA 6030407O03 gene
chr1	73960441	73960575	135	136	73960454	27	5.77405	2.5821	2.88921	Adipose_R2_peak_1	Intron (uc007b	1	73910231	73937333	27103	2	21961	uc007blf.2	-23108	ENSMUSG000	Tns1	tensin 1
chr1	73964254	73964374	121	122	73964279	26	5.31252	2.48988	2.47999	Adipose_R2_peak_1	Intron (uc007b	1	73910231	73937333	27103	2	21961	uc007blf.2	-26921	ENSMUSG000	Tns1	tensin 1
chr1	73986812	73987022	211	212	73986903	20	7.74501	3.7745	4.70467	Adipose_R1_peak_8	Intron (uc007b	1	73989722	73998468	8747	2	21961	uc007bli.1	11446	ENSMUSG000	Tns1	tensin 1
chr1	73998042	73998229	188	189	73998160	21	8.43286	3.95423	5.34896	Adipose_R1_peak_8	Promoter (<=1	1	73989722	73998468	8747	2	21961	uc007bli.1	239	ENSMUSG000	Tns1	tensin 1
chr1	74007105	74007275	171	172	74007114	25	4.86612	2.39766	2.09018	Adipose_R2_peak_1	Intron (uc007b	1	73989722	73998468	8747	2	21961	uc007bli.1	-8637	ENSMUSG000	Tns1	tensin 1
chr1	74029756	74029964	209	210	74029800	28	6.25023	2.67431	3.31626	Adipose_R2_peak_1	Intron (uc007b	1	73989722	73998468	8747	2	21961	uc007bli.1	-31288	ENSMUSG000	Tns1	tensin 1
chr1	74050791	74051015	225	226	74050876	18	6.42934	3.41502	3.48999	Adipose_R1_peak_8	Intron (uc007b	1	73989722	73998468	8747	2	21961	uc007bli.1	-52323	ENSMUSG000	Tns1	tensin 1
chr1	74077684	74077933	250	251	74077798	19	7.07687	3.59476	4.08458	Adipose_R1_peak_8	Intron (uc007b	1	73910231	74124449	214219	2	21961	uc007blg.3	46516	ENSMUSG000	Tns1	tensin 1
chr1	74110561	74110694	134	135	74110622	16	5.20017	3.05554	2.38734	Adipose_R1_peak_8	Intron (uc007b	1	73910231	74124449	214219	2	21961	uc007blg.3	13755	ENSMUSG000	Tns1	tensin 1
chr1	74229013	74229278	266	267	74229106	53	21.92215	4.97976	18.21755	Adipose_R2_peak_1	Distal Interger	1	74236518	74268213	31696	1	76709	uc007bln.1	-7240	ENSMUSG000	Arpc2	actin related protein 2/3 complex, subunit 2
chr1	74229015	74229309	295	296	74229128	48	32.41826	8.80716	28.66523	Adipose_R1_peak_8	Distal Interger	1	74236518	74268213	31696	1	76709	uc007bln.1	-7209	ENSMUSG000	Arpc2	actin related protein 2/3 complex, subunit 2
chr1	74388185	74388396	212	213	74388249	32	8.29282	3.04319	5.18352	Adipose_R2_peak_1	Distal Interger	1	74391609	74397285	5677	1	227292	uc007blz.2	-3213	ENSMUSG000	Ctdsp1	CTD (carboxy-terminal domain, RNA polymerase II, polypeptide A) small phosphatase 1
chr1	74388819	74388956	138	139	74388943	16	5.20017	3.05554	2.38734	Adipose_R1_peak_8	Promoter (2-3	1	74391609	74397285	5677	1	227292	uc007blz.2	-2653	ENSMUSG000	Ctdsp1	CTD (carboxy-terminal domain, RNA polymerase II, polypeptide A) small phosphatase 1
chr1	74391041	74391181	141	142	74391086	26	5.31252	2.48988	2.47999	Adipose_R2_peak_1	Promoter (<=1	1	74391609	74397285	5677	1	227292	uc007blz.2	-428	ENSMUSG000	Ctdsp1	CTD (carboxy-terminal domain, RNA polymerase II, polypeptide A) small phosphatase 1
chr1	74394104	74394316	213	214	74394240	30	7.24477	2.85875	4.21917	Adipose_R2_peak_1	Promoter (<=1	1	74394310	74394394	85	1	387219	uc007bma.2	0	ENSMUSG000	Mir26b	microRNA 26b
chr1	74394147	74394310	164	165	74394220	20	7.74501	3.7745	4.70467	Adipose_R1_peak_8	Promoter (<=1	1	74394310	74394394	85	1	387219	uc007bma.2	0	ENSMUSG000	Mir26b	microRNA 26b
chr1	74394531	74394708	178	179	74394560	26	5.31252	2.48988	2.47999	Adipose_R2_peak_1	Promoter (<=1	1	74394310	74394394	85	1	387219	uc007bma.2	221	ENSMUSG000	Mir26b	microRNA 26b
chr1	74396337	74396555	219	220	74396464	26	5.31252	2.48988	2.47999	Adipose_R2_peak_1	Promoter (2-3	1	74394310	74394394	85	1	387219	uc007bma.2	2027	ENSMUSG000	Mir26b	microRNA 26b
chr1	75713461	75713623	163	164	75713528	37	11.12817	3.50427	7.83613	Adipose_R2_peak_1	Distal Interger	1	75547944	75559431	11488	1	20536	uc007bpx.2	165517	ENSMUSG000	Slc4a3	solute carrier family 4 (anion exchanger), member 3
chr1	75740084	75740191	108	109	75740133	27	5.77405	2.5821	2.88921	Adipose_R2_peak_1	Distal Interger	1	75547944	75559431	11488	1	20536	uc007bpx.2	192140	ENSMUSG000	Slc4a3	solute carrier family 4 (anion exchanger), member 3
chr1	75760756	75760939	184	185	75760824	24	10.60668	4.49345	7.40961	Adipose_R1_peak_8	Distal Interger	1	75547944	75559431	11488	1	20536	uc007bpx.2	212812	ENSMUSG000	Slc4a3	solute carrier family 4 (anion exchanger), member 3
chr1	75969449	75969646	198	199	75969540	24	10.60668	4.49345	7.40961	Adipose_R1_peak_8	Distal Interger	1	75547944	75559431	11488	1	20536	uc007bpx.2	421505	ENSMUSG000	Slc4a3	solute carrier family 4 (anion exchanger), member 3
chr1	76057569	76057740	172	173	76057657	25	11.36566	4.67319	8.13482	Adipose_R1_peak_8	Distal Interger	1	76489133	76502028	12896	1	620079	uc007bpy.1	-431393	ENSMUSG000	Gm17751	predicted gene, 17751
chr1	76057615	76057722	108	109	76057660	26	5.31252	2.48988	2.47999	Adipose_R2_peak_1	Distal Interger	1	76489133	76502028	12896	1	620079	uc007bpy.1	-431411	ENSMUSG000	Gm17751	predicted gene, 17751
chr1	76236233	76236422	190	191	76236314	25	11.36566	4.67319	8.13482	Adipose_R1_peak_8	Distal Interger	1	76489133	76502028	12896	1	620079	uc007bpy.1	-252711	ENSMUSG000	Gm17751	predicted gene, 17751
chr1	76314205	76314447	243	244	76314328	36	20.67077	6.6503	17.14779	Adipose_R1_peak_8	Distal Interger	1	76489133	76502028	12896	1	620079	uc007bpy.1	-174686	ENSMUSG000	Gm17751	predicted gene, 17751
chr1	76314262	76314367	106	107	76314309	31	7.7623	2.95097	4.69388	Adipose_R2_peak_1	Distal Interger	1	76489133	76502028	12896	1	620079	uc007bpy.1	-174766	ENSMUSG000	Gm17751	predicted gene, 17751
chr1	76316670	76316902	233	234	76316739	73	38.23935	6.82411	34.23139	Adipose_R2_peak_1	Distal Interger	1	76489133	76502028	12896	1	620079	uc007bpy.1	-172231	ENSMUSG000	Gm17751	predicted gene, 17751
chr1	76316684	76316868	185	186	76316756	38	22.52591	7.00978	18.96	Adipose_R1_peak_8	Distal Interger	1	76489133	76502028	12896	1	620079	uc007bpy.1	-172265	ENSMUSG000	Gm17751	predicted gene, 17751
chr1	76590501	76590661	161	162	76590564	17	5.80341	3.23528	2.92378	Adipose_R1_peak_8	Distal Interger	1	76509560	76509643	84	1	1.02E+08	uc033fjq.1	80941	ENSMUSG000	Mir6343	microRNA 6343
chr1	76618386	76618737	352	353	76618594	72	59.47482	13.12087	55.39848	Adipose_R1_peak_8	Distal Interger	1	76509560	76509643	84	1	1.02E+08	uc033fjq.1	108826	ENSMUSG000	Mir6343	microRNA 6343
chr1	76618454	76618724	271	272	76618578	59	26.5291	5.53306	22.7188	Adipose_R2_peak_1	Distal Interger	1	76509560	76509643	84	1	1.02E+08	uc033fjq.1	108894	ENSMUSG000	Mir6343	microRNA 6343
chr1	76659165	76659306	142	143	76659215	16	5.20017	3.05554	2.38734	Adipose_R1_peak_8	Distal Interger	1	76509560	76509643	84	1	1.02E+08	uc033fjq.1	149605	ENSMUSG000	Mir6343	microRNA 6343
chr1	77785557	77785766	210	211	77785672	38	22.52591	7.00978	18.96	Adipose_R1_peak_8	Distal Interger	1	77367185	77515088	147904	2	13838	uc007bpz.1	-270469	ENSMUSG000	Epha4	Eph receptor A4
chr1	77785579	77785775	197	198	77785670	36	10.5378	3.41206	7.27949	Adipose_R2_peak_1	Distal Interger	1	77367185	77515088	147904	2	13838	uc007bpz.1	-270491	ENSMUSG000	Epha4	Eph receptor A4
chr1	77797600	77797775	176	177	77797675	31	7.7623	2.95097	4.69388	Adipose_R2_peak_1	Distal Interger	1	77367185	77515088	147904	2	13838	uc007bpz.1	-282512	ENSMUSG000	Epha4	Eph receptor A4
chr1	77797626	77797775	150	151	77797772	23	9.86443	4.31371	6.70247	Adipose_R1_peak_8	Distal Interger	1	77367185	77515088	147904	2	13838	uc007bpz.1	-282538	ENSMUSG000	Epha4	Eph receptor A4
chr1	78573735	78574296	562	563	78573825	98	62.23788	9.20704	58.13897	Adipose_R1_peak_8	Intron (uc007b	1	78537672	78538173	502	1	68393	uc007bqm.2	36063	ENSMUSG000	Mogat1	monoacylglycerol O-acyltransferase 1
chr1	78573737	78574186	450	451	78573818	121	80.59243	10.14953	76.17918	Adipose_R2_peak_1	Intron (uc007b	1	78537672	78538173	502	1	68393	uc007bqm.2	36065	ENSMUSG000	Mogat1	monoacylglycerol O-acyltransferase 1
chr1	78574435	78574617	183	184	78574514	38	15.89162	4.74912	12.51103	Adipose_R1_peak_8	Intron (uc007b	1	78537672	78538173	502	1	68393	uc007bqm.2	36763	ENSMUSG000	Mogat1	monoacylglycerol O-acyltransferase 1
chr1	78574447	78574594	148	149	78574512	37	11.12817	3.50427	7.83613	Adipose_R2_peak_1	Intron (uc007b	1	78537672	78538173	502	1	68393	uc007bqm.2	36775	ENSMUSG000	Mogat1	monoacylglycerol O-acyltransferase 1
chr1	78954387	78954547	161	162	78954465	25	11.36566	4.67319	8.13482	Adipose_R1_peak_8	Distal Interger	1	78816949	78820025	3077	1	57814	uc007bqq.1	137438	ENSMUSG000	Kcne4	potassium voltage-gated channel, Isk-related subfamily, gene 4
chr1	78984028	78984284	257	258	78984147	36	20.67077	6.6503	17.14779	Adipose_R1_peak_8	Distal Interger	1	78816949	78820025	3077	1	57814	uc007bqq.1	167079	ENSMUSG000	Kcne4	potassium voltage-gated channel, Isk-related subfamily, gene 4
chr1	78984081	78984214	134	135	78984143	33	8.83596	3.1354	5.68652	Adipose_R2_peak_1	Distal Interger	1	78816949	78820025	3077	1	57814	uc007bqq.1	167132	ENSMUSG000	Kcne4	potassium voltage-gated channel, Isk-related subfamily, gene 4
chr1	78987679	78987931	253	254	78987812	41	25.38991	7.54899	21.76305	Adipose_R1_peak_8	Distal Interger	1	78816949	78820025	3077	1	57814	uc007bqq.1	170730	ENSMUSG000	Kcne4	potassium voltage-gated channel, Isk-related subfamily, gene 4
chr1	78987735	78987853	119	120	78987797	33	8.83596	3.1354	5.68652	Adipose_R2_peak_1	Distal Interger	1	78816949	78820025	3077	1	57814	uc007bqq.1	170786	ENSMUSG000	Kcne4	potassium voltage-gated channel, Isk-related subfamily, gene 4
chr1	79041267	79041520	254	255	79041387	42	26.36523	7.72873	22.71906	Adipose_R1_peak_8	Distal Interger	1	78816949	78820025	3077	1	57814	uc007bqq.1	224318	ENSMUSG000	Kcne4	potassium voltage-gated channel, Isk-related subfamily, gene 4
chr1	79041293	79041477	185	186	79041403	35	9.95877	3.31984	6.73562	Adipose_R2_peak_1	Distal Interger	1	78816949	78820025	3077	1	57814	uc007bqq.1	224344	ENSMUSG000	Kcne4	potassium voltage-gated channel, Isk-related subfamily, gene 4
chr1	79044594	79044860	267	268	79044732	34	18.78671	6.26176	15.32924	Adipose_R1_peak_8	Distal Interger	1	78816949	78820025	3077	1	57814	uc007bqq.1	227645	ENSMUSG000	Kcne4	potassium voltage-gated channel, Isk-related subfamily, gene 4
chr1	79044658	79044805	148	149	79044741	31	7.7623	2.95097	4.69388	Adipose_R2_peak_1	Distal Interger	1	78816949	78820025	3077	1	57814	uc007bqq.1	227709	ENSMUSG000	Kcne4	potassium voltage-gated channel, Isk-related subfamily, gene 4
chr1	79248657	79248845	189	190	79248743	23	9.86443	4.31371	6.70247	Adipose_R1_peak_8	Distal Interger	1	79434669	79440090	5422	2	20254	uc007bqr.1	191245	ENSMUSG000	Scg2	secretogranin II
chr1	79248689	79248797	109	110	79248739	29	6.74062	2.76653	3.75989	Adipose_R2_peak_1	Distal Interger	1	79434669	79440090	5422	2	20254	uc007bqr.1	191293	ENSMUSG000	Scg2	secretogranin II
chr1	79277380	79277514	135	136	79277464	17	5.80341	3.23528	2.92378	Adipose_R1_peak_8	Distal Interger	1	79434669	79440090	5422	2	20254	uc007bqr.1	162576	ENSMUSG000	Scg2	secretogranin II
chr1	79278432	79278595	164	165	79278545	23	9.86443	4.31371	6.70247	Adipose_R1_peak_8	Distal Interger	1	79434669	79440090	5422	2	20254	uc007bqr.1	161495	ENSMUSG000	Scg2	secretogranin II
chr1	79278485	79278583	99	100	79278559	27	5.77405	2.5821	2.88921	Adipose_R2_peak_1	Distal Interger	1	79434669	79440090	5422	2	20254	uc007bqr.1	161507	ENSMUSG000	Scg2	secretogranin II
chr1	79381487	79381626	140	141	79381559	34	9.39139	3.22762	6.20474	Adipose_R2_peak_1	Distal Interger	1	79434669	79440090	5422	2	20254	uc007bqr.1	58464	ENSMUSG000	Scg2	secretogranin II
chr1	79382412	79382586	175	176	79382485	24	10.60668	4.49345	7.40961	Adipose_R1_peak_8	Distal Interger	1	79434669	79440090	5422	2	20254	uc007bqr.1	57504	ENSMUSG000	Scg2	secretogranin II
chr1	79489832	79490013	182	183	79489937	22	9.13959	4.13397	6.01526	Adipose_R1_peak_8	Distal Interger	1	79434669	79440090	5422	2	20254	uc007bqr.1	-49742	ENSMUSG000	Scg2	secretogranin II
chr1	79962787	79963056	270	271	79962912	38	22.52591	7.00978	18.96	Adipose_R1_peak_8	Distal Interger	1	79794321	79858665	64345	2	20720	uc007bqz.2	-104122	ENSMUSG000	Serpine2	serine (or cysteine) peptidase inhibitor, clade E, member 2
chr1	79962800	79963026	227	228	79962919	49	19.00719	4.61089	15.38598	Adipose_R2_peak_1	Distal Interger	1	79794321	79858665	64345	2	20720	uc007bqz.2	-104135	ENSMUSG000	Serpine2	serine (or cysteine) peptidase inhibitor, clade E, member 2
chr1	80004352	80004494	143	144	80004444	17	5.80341	3.23528	2.92378	Adipose_R1_peak_8	Distal Interger	1	79794321	79858665	64345	2	20720	uc007bqz.2	-145687	ENSMUSG000	Serpine2	serine (or cysteine) peptidase inhibitor, clade E, member 2
chr1	80005298	80005518	221	222	80005415	32	17.10103	5.93135	13.67137	Adipose_R1_peak_8	Distal Interger	1	79794321	79858665	64345	2	20720	uc007bqz.2	-146633	ENSMUSG000	Serpine2	serine (or cysteine) peptidase inhibitor, clade E, member 2
chr1	80005353	80005487	135	136	80005436	31	7.7623	2.95097	4.69388	Adipose_R2_peak_1	Distal Interger	1	79794321	79858665	64345	2	20720	uc007bqz.2	-146688	ENSMUSG000	Serpine2	serine (or cysteine) peptidase inhibitor, clade E, member 2
chr1	80138828	80139096	269	270	80138966	45	29.34988	8.26794	25.64927	Adipose_R1_peak_8	Distal Interger	1	80198699	80213944	15246	2	241128	uc007brb.2	74848	ENSMUSG000	Fam124b	family with sequence similarity 124, member B
chr1	80138851	80139034	184	185	80138956	39	12.34187	3.68871	8.98501	Adipose_R2_peak_1	Distal Interger	1	80198699	80213944	15246	2	241128	uc007brb.2	74910	ENSMUSG000	Fam124b	family with sequence similarity 124, member B
chr1	80926838	80927110	273	274	80926980	42	26.36523	7.72873	22.71906	Adipose_R1_peak_8	Distal Interger	1	81076950	81300096	223147	1	241134	uc007brj.1	-149840	ENSMUSG000	Nyap2	neuronal tyrosine-phophorylated phosphoinositide 3-kinase adaptor 2
chr1	80926872	80927057	186	187	80926976	41	13.59774	3.87315	10.1803	Adipose_R2_peak_1	Distal Interger	1	81076950	81300096	223147	1	241134	uc007brj.1	-149893	ENSMUSG000	Nyap2	neuronal tyrosine-phophorylated phosphoinositide 3-kinase adaptor 2
chr1	81425812	81426045	234	235	81425957	35	19.76029	6.47056	16.26028	Adipose_R1_peak_8	Distal Interger	1	81077407	81341764	264358	1	241134	uc007brn.1	348405	ENSMUSG000	Nyap2	neuronal tyrosine-phophorylated phosphoinositide 3-kinase adaptor 2
chr1	81425907	81426032	126	127	81425974	30	7.24477	2.85875	4.21917	Adipose_R2_peak_1	Distal Interger	1	81077407	81341764	264358	1	241134	uc007brn.1	348500	ENSMUSG000	Nyap2	neuronal tyrosine-phophorylated phosphoinositide 3-kinase adaptor 2
chr1	81497154	81497313	160	161	81497218	24	10.60668	4.49345	7.40961	Adipose_R1_peak_8	Distal Interger	1	81077407	81341764	264358	1	241134	uc007brn.1	419747	ENSMUSG000	Nyap2	neuronal tyrosine-phophorylated phosphoinositide 3-kinase adaptor 2
chr1	81500167	81500366	200	201	81500251	23	9.86443	4.31371	6.70247	Adipose_R1_peak_8	Distal Interger	1	81077407	81341764	264358	1	241134	uc007brn.1	422760	ENSMUSG000	Nyap2	neuronal tyrosine-phophorylated phosphoinositide 3-kinase adaptor 2
chr1	81559385	81559766	382	383	81559590	171	195.6953	29.47555	191.0739	Adipose_R1_peak_8	Distal Interger	1	81077407	81341764	264358	1	241134	uc007brn.1	481978	ENSMUSG000	Nyap2	neuronal tyrosine-phophorylated phosphoinositide 3-kinase adaptor 2
chr1	81559433	81559734	302	303	81559590	148	116.8445	13.74044	112.274	Adipose_R2_peak_1	Distal Interger	1	81077407	81341764	264358	1	241134	uc007brn.1	482026	ENSMUSG000	Nyap2	neuronal tyrosine-phophorylated phosphoinositide 3-kinase adaptor 2
chr1	81850534	81850652	119	120	81850562	25	4.86612	2.39766	2.09018	Adipose_R2_peak_1	Distal Interger	1	82131916	82132019	104	1	1.02E+08	uc033fjw.1	-281264	ENSMUSG000	Mir6344	microRNA 6344
chr1	82008484	82008692	209	210	82008594	32	17.10103	5.93135	13.67137	Adipose_R1_peak_8	Distal Interger	1	82131916	82132019	104	1	1.02E+08	uc033fjw.1	-123224	ENSMUSG000	Mir6344	microRNA 6344
chr1	82061774	82061986	213	214	82061891	34	18.86162	6.29083	15.38482	Adipose_R1_peak_8	Distal Interger	1	82131916	82132019	104	1	1.02E+08	uc033fjw.1	-69930	ENSMUSG000	Mir6344	microRNA 6344
chr1	82061821	82061946	126	127	82061904	30	7.24477	2.85875	4.21917	Adipose_R2_peak_1	Distal Interger	1	82131916	82132019	104	1	1.02E+08	uc033fjw.1	-69970	ENSMUSG000	Mir6344	microRNA 6344
chr1	82552129	82552279	151	152	82552205	36	10.5378	3.41206	7.27949	Adipose_R2_peak_1	Intron (uc033fj	1	82450723	82571535	120813	2	12829	uc033fjy.1	19256	ENSMUSG000	Col4a4	collagen, type IV, alpha 4
chr1	82552149	82552306	158	159	82552225	24	10.60668	4.49345	7.40961	Adipose_R1_peak_8	Intron (uc033fj	1	82450723	82571535	120813	2	12829	uc033fjy.1	19229	ENSMUSG000	Col4a4	collagen, type IV, alpha 4
chr1	82705600	82706079	480	481	82705825	61	43.50234	10.05977	39.59415	Adipose_R1_peak_8	Intron (uc007b	1	82724890	82752389	27500	1	75734	uc007brx.2	-18811	ENSMUSG000	Mff	mitochondrial fission factor

chr1	82705670	82705919	250	251	82705784	37	11.12817	3.50427	7.83613	Adipose_R2_peak_1	Intron (uc007t	1	82724890	82752389	27500	1	75734	uc007brx.2	-18971	ENSMUSG000	Mff	mitochondrial fission factor
chr1	83031215	83031327	113	114	83031275	31	7.7623	2.95097	4.69388	Adipose_R2_peak_1	Intron (uc007t	1	83012523	83038448	25926	2	80721	uc007bsi.2	7121	ENSMUSG000	Slc19a3	solute carrier family 19, member 3
chr1	83031473	83031631	159	160	83031558	25	10.1687	4.19851	7.00421	Adipose_R1_peak_8	Intron (uc007t	1	83012523	83038448	25926	2	80721	uc007bsi.2	6817	ENSMUSG000	Slc19a3	solute carrier family 19, member 3
chr1	83031514	83031628	115	116	83031582	31	7.7623	2.95097	4.69388	Adipose_R2_peak_1	Intron (uc007t	1	83012523	83038448	25926	2	80721	uc007bsi.2	6820	ENSMUSG000	Slc19a3	solute carrier family 19, member 3
chr1	83231271	83231501	231	232	83231386	55	23.42763	5.16419	19.68558	Adipose_R2_peak_1	Distal Intergen	1	83255781	83279335	23555	2	77629	uc007bsp.2	47834	ENSMUSG000	Sphkap	SPHK1 interactor, AKAP domain containing
chr1	83421826	83422013	188	189	83421871	27	5.77405	2.5821	2.88921	Adipose_R2_peak_1	Distal Intergen	1	83255781	83408200	152420	2	77629	uc007bsq.2	-13626	ENSMUSG000	Sphkap	SPHK1 interactor, AKAP domain containing
chr1	83467208	83467440	233	234	83467333	29	14.55748	5.39214	11.20652	Adipose_R1_peak_8	Distal Intergen	1	83255781	83408200	152420	2	77629	uc007bsq.2	-59008	ENSMUSG000	Sphkap	SPHK1 interactor, AKAP domain containing
chr1	83467248	83467401	154	155	83467316	34	9.39139	3.22762	6.20474	Adipose_R2_peak_1	Distal Intergen	1	83255781	83408200	152420	2	77629	uc007bsq.2	-59048	ENSMUSG000	Sphkap	SPHK1 interactor, AKAP domain containing
chr1	83820659	83820831	173	174	83820746	43	14.8939	4.05758	11.42017	Adipose_R2_peak_1	Distal Intergen	1	84218849	84218961	113	2	1.02E+08	uc033fka.1	398130	ENSMUSG000	Mir6353	microRNA 6353
chr1	83820703	83820833	131	132	83820764	26	10.08036	4.05716	6.9166	Adipose_R1_peak_8	Distal Intergen	1	84218849	84218961	113	2	1.02E+08	uc033fka.1	398128	ENSMUSG000	Mir6353	microRNA 6353
chr1	83983103	83983212	110	111	83983153	32	8.29282	3.04319	5.18352	Adipose_R2_peak_1	Distal Intergen	1	84218849	84218961	113	2	1.02E+08	uc033fka.1	235749	ENSMUSG000	Mir6353	microRNA 6353
chr1	84161595	84161733	139	140	84161706	26	5.31252	2.48988	2.47999	Adipose_R2_peak_1	Intron (uc007t	1	84218849	84218961	113	2	1.02E+08	uc033fka.1	57228	ENSMUSG000	Mir6353	microRNA 6353
chr1	84162321	84162554	234	235	84162456	62	26.16656	5.20375	22.36708	Adipose_R2_peak_1	Intron (uc007t	1	84218849	84218961	113	2	1.02E+08	uc033fka.1	56407	ENSMUSG000	Mir6353	microRNA 6353
chr1	84264591	84264852	262	263	84264718	46	30.36364	8.44768	26.64488	Adipose_R1_peak_8	Intron (uc007t	1	84036293	84284645	248353	2	98496	uc007bst.2	19793	ENSMUSG000	Pid1	phosphotyrosine interaction domain containing 1
chr1	84264611	84264846	236	237	84264732	54	22.67101	5.07198	18.94758	Adipose_R2_peak_1	Intron (uc007t	1	84036293	84284645	248353	2	98496	uc007bst.2	19799	ENSMUSG000	Pid1	phosphotyrosine interaction domain containing 1
chr1	84267386	84267597	212	213	84267497	37	21.59274	6.83004	18.04766	Adipose_R1_peak_8	Intron (uc007t	1	84036293	84284645	248353	2	98496	uc007bst.2	17048	ENSMUSG000	Pid1	phosphotyrosine interaction domain containing 1
chr1	84457411	84457587	177	178	84457511	47	17.60017	4.42645	14.02531	Adipose_R2_peak_1	Intron (uc007t	1	84115332	84341110	225779	2	98496	uc007bsu.1	-116301	ENSMUSG000	Pid1	phosphotyrosine interaction domain containing 1
chr1	84457413	84457672	260	261	84457522	37	21.59274	6.83004	18.04766	Adipose_R1_peak_8	Intron (uc007t	1	84115332	84341110	225779	2	98496	uc007bsu.1	-116303	ENSMUSG000	Pid1	phosphotyrosine interaction domain containing 1
chr1	84543705	84543910	206	207	84543801	24	10.60668	4.49345	7.40961	Adipose_R1_peak_8	Intron (uc007t	1	84695839	84695915	77	1	1.01E+08	uc029qpm.1	-151929	ENSMUSG000	Mir5126	microRNA 5126
chr1	84543829	84543926	98	99	84543896	27	5.77405	2.5821	2.88921	Adipose_R2_peak_1	Intron (uc007t	1	84695839	84695915	77	1	1.01E+08	uc029qpm.1	-151913	ENSMUSG000	Mir5126	microRNA 5126
chr1	84545289	84545623	335	336	84545454	43	14.8939	4.05758	11.42017	Adipose_R2_peak_1	Intron (uc007t	1	84695839	84695915	77	1	1.01E+08	uc029qpm.1	-150216	ENSMUSG000	Mir5126	microRNA 5126
chr1	84545295	84545621	327	328	84545459	33	13.61287	4.54883	10.31532	Adipose_R1_peak_8	Intron (uc007t	1	84695839	84695915	77	1	1.01E+08	uc029qpm.1	-150218	ENSMUSG000	Mir5126	microRNA 5126
chr1	84545865	84546091	227	228	84545964	31	7.7623	2.95097	4.69388	Adipose_R2_peak_1	Intron (uc007t	1	84695839	84695915	77	1	1.01E+08	uc029qpm.1	-149748	ENSMUSG000	Mir5126	microRNA 5126
chr1	84575882	84576029	148	149	84575950	21	8.43286	3.95423	5.34896	Adipose_R1_peak_8	Intron (uc007t	1	84695839	84695915	77	1	1.01E+08	uc029qpm.1	-119810	ENSMUSG000	Mir5126	microRNA 5126
chr1	84701075	84701242	168	169	84701157	21	8.43286	3.95423	5.34896	Adipose_R1_peak_8	Distal Intergen	1	84369839	84696221	326383	2	227325	uc007bsw.1	-4854	ENSMUSG000	Dner	delta/notch-like EGF repeat containing
chr1	84701077	84701179	103	104	84701138	27	5.77405	2.5821	2.88921	Adipose_R2_peak_1	Distal Intergen	1	84369839	84696221	326383	2	227325	uc007bsw.1	-4856	ENSMUSG000	Dner	delta/notch-like EGF repeat containing
chr1	84953700	84954090	391	392	84953811	94	33.19411	4.54931	29.26523	Adipose_R2_peak_1	Distal Intergen	1	84906705	84935083	28379	2	71781	uc007btd.1	-18617	ENSMUSG000	Slc16a14	solute carrier family 16 (monocarboxylic acid transporters), member 14
chr1	84955118	84955549	432	433	84955337	133	25.57249	2.8718	21.78773	Adipose_R2_peak_1	Distal Intergen	1	84906705	84935083	28379	2	71781	uc007btd.1	-20035	ENSMUSG000	Slc16a14	solute carrier family 16 (monocarboxylic acid transporters), member 14
chr1	84955246	84955513	268	269	84955367	83	12.36224	2.39362	9.09833	Adipose_R1_peak_8	Distal Intergen	1	84906705	84935083	28379	2	71781	uc007btd.1	-20163	ENSMUSG000	Slc16a14	solute carrier family 16 (monocarboxylic acid transporters), member 14
chr1	84955746	84955865	120	121	84955796	55	10.11269	2.60582	6.88452	Adipose_R2_peak_1	Distal Intergen	1	84906705	84935083	28379	2	71781	uc007btd.1	-20663	ENSMUSG000	Slc16a14	solute carrier family 16 (monocarboxylic acid transporters), member 14
chr1	84956447	84956640	194	195	84956553	40	10.63649	3.22069	7.37524	Adipose_R2_peak_1	Distal Intergen	1	84906705	84935083	28379	2	71781	uc007btd.1	-21364	ENSMUSG000	Slc16a14	solute carrier family 16 (monocarboxylic acid transporters), member 14
chr1	84956758	84956956	199	200	84956869	45	11.77553	3.23746	8.44906	Adipose_R2_peak_1	Distal Intergen	1	84906705	84935083	28379	2	71781	uc007btd.1	-21675	ENSMUSG000	Slc16a14	solute carrier family 16 (monocarboxylic acid transporters), member 14
chr1	84957249	84957612	364	365	84957469	63	11.72562	2.66267	8.4047	Adipose_R2_peak_1	Distal Intergen	1	84906705	84935083	28379	2	71781	uc007btd.1	-22166	ENSMUSG000	Slc16a14	solute carrier family 16 (monocarboxylic acid transporters), member 14
chr1	84958543	84958842	300	301	84958729	57	15.93868	3.51633	12.43069	Adipose_R2_peak_1	Distal Intergen	1	84906705	84935083	28379	2	71781	uc007btd.1	-23460	ENSMUSG000	Slc16a14	solute carrier family 16 (monocarboxylic acid transporters), member 14
chr1	84959191	84959481	291	292	84959332	94	29.61907	4.09248	25.75603	Adipose_R2_peak_1	Distal Intergen	1	84906705	84935083	28379	2	71781	uc007btd.1	-24108	ENSMUSG000	Slc16a14	solute carrier family 16 (monocarboxylic acid transporters), member 14
chr1	84959233	84959375	143	144	84959297	51	6.12671	2.05204	3.24208	Adipose_R1_peak_8	Distal Intergen	1	84906705	84935083	28379	2	71781	uc007btd.1	-24150	ENSMUSG000	Slc16a14	solute carrier family 16 (monocarboxylic acid transporters), member 14
chr1	84959887	84960072	186	187	84959990	44	8.48427	2.61338	5.36772	Adipose_R2_peak_1	Distal Intergen	1	84906705	84935083	28379	2	71781	uc007btd.1	-24804	ENSMUSG000	Slc16a14	solute carrier family 16 (monocarboxylic acid transporters), member 14
chr1	84960731	84960852	122	123	84960790	41	8.10253	2.6291	5.02132	Adipose_R2_peak_1	Distal Intergen	1	84906705	84935083	28379	2	71781	uc007btd.1	-25648	ENSMUSG000	Slc16a14	solute carrier family 16 (monocarboxylic acid transporters), member 14
chr1	84961044	84961479	436	437	84961172	59	17.63173	3.7294	14.05622	Adipose_R2_peak_1	Distal Intergen	1	84906705	84935083	28379	2	71781	uc007btd.1	-25961	ENSMUSG000	Slc16a14	solute carrier family 16 (monocarboxylic acid transporters), member 14
chr1	84961117	84961409	293	294	84961376	34	7.16311	2.66362	4.16973	Adipose_R1_peak_9	Distal Intergen	1	84906705	84935083	28379	2	71781	uc007btd.1	-26034	ENSMUSG000	Slc16a14	solute carrier family 16 (monocarboxylic acid transporters), member 14
chr1	84962304	84962787	484	485	84962350	37	6.47049	2.40459	3.52834	Adipose_R2_peak_1	Distal Intergen	1	84906705	84935083	28379	2	71781	uc007btd.1	-27221	ENSMUSG000	Slc16a14	solute carrier family 16 (monocarboxylic acid transporters), member 14
chr1	84962891	84963163	273	274	84963030	103	24.80597	3.30784	21.03806	Adipose_R2_peak_1	Distal Intergen	1	84906705	84935083	28379	2	71781	uc007btd.1	-27808	ENSMUSG000	Slc16a14	solute carrier family 16 (monocarboxylic acid transporters), member 14
chr1	84963763	84964055	293	294	84963949	100	19.52974	2.85175	15.89987	Adipose_R2_peak_1	Distal Intergen	1	84906705	84935083	28379	2	71781	uc007btd.1	-28680	ENSMUSG000	Slc16a14	solute carrier family 16 (monocarboxylic acid transporters), member 14
chr1	84964161	84964478	318	319	84964299	96	17.67672	2.73881	14.10064	Adipose_R2_peak_1	Distal Intergen	1	84906705	84935083	28379	2	71781	uc007btd.1	-29078	ENSMUSG000	Slc16a14	solute carrier family 16 (monocarboxylic acid transporters), member 14
chr1	84964188	84964337	150	151	84964254	60	7.94039	2.18785	4.89803	Adipose_R1_peak_9	Distal Intergen	1	84906705	84935083	28379	2	71781	uc007btd.1	-29105	ENSMUSG000	Slc16a14	solute carrier family 16 (monocarboxylic acid transporters), member 14
chr1	84964820	84965037	218	219	84964931	84	25.643	3.94105	21.85733	Adipose_R2_peak_1	Distal Intergen	1	84906705	84935083	28379	2	71781	uc007btd.1	-29737	ENSMUSG000	Slc16a14	solute carrier family 16 (monocarboxylic acid transporters), member 14
chr1	84965594	84965697	104	105	84965639	36	7.59494	2.69037	4.55612	Adipose_R2_peak_1	Exon (uc007bt	1	84906705	84935083	28379	2	71781	uc007btd.1	-30511	ENSMUSG000	Slc16a14	solute carrier family 16 (monocarboxylic acid transporters), member 14
chr1	84965801	84966298	498	499	84965911	71	25.31956	4.46552	21.53764	Adipose_R2_peak_1	Exon (uc007bt	1	84906705	84935083	28379	2	71781	uc007btd.1	-30718	ENSMUSG000	Slc16a14	solute carrier family 16 (monocarboxylic acid transporters), member 14
chr1	84966805	84966904	100	101	84966831	28	5.43479	2.44572	2.59758	Adipose_R2_peak_1	Distal Intergen	1	84906705	84935083	28379	2	71781	uc007btd.1	-31722	ENSMUSG000	Slc16a14	solute carrier family 16 (monocarboxylic acid transporters), member 14
chr1	84966814	84966947	134	135	84966859	24	5.3874	2.6026	2.57115	Adipose_R1_peak_9	Distal Intergen	1	84906705	84935083	28379	2	71781	uc007btd.1	-31731	ENSMUSG000	Slc16a14	solute carrier family 16 (monocarboxylic acid transporters), member 14
chr1	84967436	84967939	504	505	84967552	108	51.33863	6.34457	47.17002	Adipose_R2_peak_1	Distal Intergen	1	84906705	84935083	28379	2	71781	uc007btd.1	-32353	ENSMUSG000	Slc16a14	solute carrier family 16 (monocarboxylic acid transporters), member 14
chr1	84975150	84975274	125	126	84975243	40	5.81866	2.19388	2.93226	Adipose_R2_peak_1	Distal Intergen	1	84906705	84935083	28379	2	71781	uc007btd.1	-40067	ENSMUSG000	Slc16a14	solute carrier family 16 (monocarboxylic acid transporters), member 14
chr1	84975727	84976086	360	361	84976002	42	13.5027	3.78483	10.10071	Adipose_R2_peak_1	Distal Intergen	1	84906705	84935083	28379	2	71781	uc007btd.1	-40644	ENSMUSG000	Slc16a14	solute carrier family 16 (monocarboxylic acid transporters), member 14
chr1	84976414	84976597	184	185	84976502	71	27.91731	4.91852	24.08529	Adipose_R2_peak_1	Distal Intergen	1	84906705	84935083	28379	2	71781	uc007btd.1	-41331	ENSMUSG000	Slc16a14	solute carrier family 16 (monocarboxylic acid transporters), member 14
chr1	84976700	84976866	167	168	84976769	70	23.62752	4.23408	19.88283	Adipose_R2_peak_1	Distal Intergen	1	84906705	84935083	28379	2	71781	uc007btd.1	-41617	ENSMUSG000	Slc16a14	solute carrier family 16 (monocarboxylic acid transporters), member 14
chr1	84979077	84979328	252	253	84979217	117	22.21325	2.82808	18.50436	Adipose_R2_peak_1	Distal Intergen	1	84906705	84935083	28379	2	71781	uc007btd.1	-43994	ENSMUSG000	Slc16a14	solute carrier family 16 (monocarboxylic acid transporters), member 14
chr1	84979591	84979967	377	378	84979829	144	22.95321	2.5711	19.22588	Adipose_R2_peak_1	Distal Intergen	1	84906705	84935083	28379	2	71781	uc007btd.1	-44508	ENSMUSG000	Slc16a14	solute carrier family 16 (monocarboxylic acid transporters), member 14
chr1	84982523	84982965	443	444	84982809	87	40.74075	6.09517	36.70131	Adipose_R2_peak_1	Distal Intergen	1	84906705	84935083	28379	2	71781	uc007btd.1	-47440	ENSMUSG000	Slc16a14	solute carrier family 16 (monocarboxylic acid transporters), member 14
chr1	84982644	84982790	147	148	84982715	32	5.71176	2.37976	2.88974	Adipose_R1_peak_9	Distal Intergen	1	84906705	84935083	28379	2	71781	uc007btd.1	-47561	ENSMUSG000	Slc16a14	solute carrier family 16 (monocarboxylic acid transporters), member 14
chr1	84983489	84983754	266	267	84983631	55	19.64069	4.33123	16.00896	Adipose_R2_peak_1	Distal Intergen	1	84906705	84935083	28379	2	71781	uc007btd.1	-48406	ENSMUSG000	Slc16a14	solute carrier family 16 (monocarboxylic acid transporters), member 14
chr1	84986256	84986426	171	172	84986338	31	7.7623	2.95097	4.69388	Adipose_R2_peak_1	Distal Intergen	1	84906705	84935083	28379	2	71781	uc007btd.1	-51173	ENSMUSG000	Slc16a14	solute carrier family 16 (monocarboxylic acid transporters), member 14
chr1	84987409	84988022	614	615	84987612	92	25.32641	3.64073	21.54441	Adipose_R2_peak_1	Distal Intergen	1	84906705	84935083	28379	2	71781	uc007btd.1	-52326	ENSMUSG000	Slc16a14	solute carrier family 16 (monocarboxylic acid transporters), member 14
chr1	84988539	84988924	386	387	84988655	44	11.57392	3.23993	8.26827	Adipose_R2_peak_1	Distal Intergen	1	84906705	84935083	28379	2	71781	uc007btd.1	-53456	ENSMUSG000	Slc16a14	solute carrier family 16 (monocarboxylic acid transporters), member 14
chr1	84988688	84988840	153	154	84988798	27	6.46127	2.78386	3.52142	Adipose_R1_peak_9	Distal Intergen	1	84906705	84935083	28379	2	71781	uc007btd.1	-53605	ENSMUSG000	Slc16a14	solute carrier family 16 (monocarboxylic acid transporters), member 14
chr1	84989115	84989361	247	248	84989231	46	13.4862	3.55207	10.08454	Adipose_R2_peak_1	Distal Intergen	1	84906705	84935083	28379	2	71781	uc007btd.1	-54032	ENSMUSG000	Slc16a14	solute carrier family 16 (monocarboxylic acid transporters), member 14
chr1	84989634	84989824	191	192	84989710	47	12.17961	3.23298	8.8416	Adipose_R2_peak_1	Distal Intergen	1	84906705	84935083	28379	2	71781	uc007btd.1	-54551	ENSMUSG000	Slc16a14	solute carrier family 16 (monocarboxylic acid transporters), member 14
chr1	84989946	84990306	361	362	84990172	97	36.95664	4.91908	32.9703	Adipose_R2_peak_1	Distal Intergen	1	84906705	84935083	28379	2	71781	uc007btd.1	-54863	ENSMUSG000	Slc16a14	solute carrier family 16 (monocarboxylic acid transporters), member 14
chr1	84990594	84990774	181	182	84990692	46	8.24597	2.513	5.15948	Adipose_R2_peak_1	Exon (uc007bt	1	84906705	84935083	28379	2	71781	uc007btd.1	-55511	ENSMUSG000	Slc16a14	solute carrier family 16 (monocarboxylic acid transporters), member 14
chr1	84990903	84991375	473	474	84991094	92	23.10656	3.38625	19.37722	Adipose_R2_peak_1	Intron (uc007t	1	84906705	84935083	28379	2	71781	uc007btd.1	-55820	ENSMUSG000	Slc16a14	solute carrier family 16 (monocarboxylic acid transporters), member 14
chr1	84991107	84991249	143	144	84991165	46	6.24581	2.15433	3.35928	Adipose_R1_peak_9	Intron (uc007t	1	84906705	84935083	28379	2	71781	uc007btd.1	-56024	ENSMUSG000	Slc16a14	solute carrier family 16 (monocarboxylic acid transporters), member 14
chr1	84991694	84991933	240	241	84991827	80	26.64449	4.2409	22.83287	Adipose_R2_peak_1	Intron (uc007t	1	84906705	84935083	28379	2	71781	uc007btd.1	-56611	ENSMUSG000	Slc16a14	solute carrier family 16 (monocarboxylic acid transporters), member 14
chr1	84992052	84992337	286	287	84992203	72	23.69461	4.14975	19.94922	Adipose_R2_peak_1	Intron (uc007t	1	84906705	84935083	28379	2	71781	uc007btd.1	-56969	ENSMUSG000	Slc16a14	solute carrier family 16 (monocarboxylic acid transporters), member 14
chr1	84992846	84993577	732	733	84992936	58	12.19092	2.85622	8.85268	Adipose_R2_peak_1	Exon (uc007bt	1	84906705	84935083	28379	2	71781	uc007btd.1	-57763	ENSMUSG000	Slc16a14	solute carrier family 16 (monocarboxylic acid transporters), member 14
chr1	84993122	84993528	407	408	84993212	48	6.75002	2.20459	3.80615	Adipose_R1_peak_9	Intron (uc007t	1	84906705	84935083	28379	2	71781	uc007btd.1	-58039	ENSMUSG000	Slc16a14	solute carrier family 16 (monocarboxylic acid transporters), member 14
chr1	84993799	84993944	146	147	84993854	43	8.6314	2.67278	5.50921	Adipose_R2_peak_1	Intron (uc007t	1	84906705	84935083	28379	2	71781	uc007btd.1	-58716	ENSMUSG000	Slc16a14	solute carrier family 16 (monocarboxylic acid transporters), member 14
chr1	84994298	84994827	530	531	84994424	69	15.82748	3.08849	12.32159	Adipose_R2_peak_1	Intron (uc007t	1	84906705	84935083	28379	2	71781	uc007btd.1	-59215	ENSMUSG000	Slc16a14	solute carrier family 16 (monocarboxylic acid transporters), member 14
chr1	84995060	84995223	164	165	84995138	45	14.93125	3.93321	11.45662	Adipose_R2_peak_1	Intron (uc007t	1	84906705	84935083	28379	2	71781	uc007btd.1	-59977	ENSMUSG000	Slc16a14	solute carrier family 16 (monocarboxylic acid transporters), member 14
chr1	84998762	84998950	189	190	84998843	23	9.86443	4.31371	6.70247	Adipose_R1_peak_9	Intron (uc007t	1	84906705	84935083	28379	2	71781	uc007btd.1	-63679	ENSMUSG000	Slc16a14	solute carrier family 16 (monocarboxylic acid transporters), member 14
chr1	85001244	85001509	266	267	85001377	81	37.48525	5.96274	33.48911	Adipose_R2_peak_1	Intron (uc007t	1	84906705	84935083	28379	2	71781	uc007btd.1	-66161	ENSMUSG000	Slc16a14	solute carrier family 16 (monocarboxylic acid transporters), member 14
chr1	85002105	85002218	114	115	85002165	30	7.24477	2.85875	4.21917	Adipose_R2_peak_1	Exon (uc029qp	1	84906705	84935083	28379	2	71781	uc007btd.1	-67022	ENSMUSG000	Slc16a14	solute carrier family 16 (monocarboxylic acid transporters), member 14
chr1	85002973	85003165	193	194	85003065	38	6.50631	2.38425	3.56239	Adipose_R2_peak_1	Intron (uc007t	1	84906705	84935083	28379	2	71781	uc007btd.1	-67890	ENSMUSG000	Slc16a14	solute carrier family 16 (monocarboxylic acid transporters), member 14
chr1	85004519	85005028	510	511	85004636	31	6.71571	2.67085	3.75989	Adipose_R2_peak_1	Intron (uc007t	1	84906705	84935083	28379	2	71781	uc007btd.1	-69436	ENSMUSG000	Slc16a14	solute carrier family 16 (monocarboxylic acid transporters), member 14
chr1	85005344	85005636	293	294	85005493	105	38.96622	4.82274	34.95184	Adipose_R2_peak_1	Intron (uc007t	1	84906705	84935083	28379	2	71781	uc007btd.1	-70261	ENSMUSG000	Slc16a14	solute carrier family 16 (monocarboxylic acid transporters), member 14
chr1	85005451	85005622	172	173	85005523	50	11.35487	2.96045	8.13482	Adipose_R1_peak_9	Intron (uc007t	1	84906705	84935083	28379	2	71781	uc007btd.1	-70368	ENSMUSG000	Slc16a14	solute carrier family 16 (monocarboxylic acid transporters), member 14
chr1	85007690	85007800	111	112	85007696	34	4.77172	2.10443	2.04601	Adipose_R2_peak_1	Intron (uc007t	1	84906705	84935083	28379	2	71781	uc007btd.1	-72607	ENSMUSG000	Slc16a14	solute carrier family 16 (monocarboxylic acid transporters), member 14
chr1	85008684	85009711	1028	1029	85008817	61	9.86749	2.44111	6.66438	Adipose_R2_peak_1	Intron (uc007t	1	84906705	84935083	28379	2	71781	uc007btd.1	-73601	ENSMUSG000	Slc16a14	solute carrier family 16 (monocarboxylic acid transporters), member 14
chr1	85009209	85009673	465	466	85009263	47	8.19422	2.47818	5.14911	Adipose_R1_peak_9	Intron (uc007t	1	84906705	84935083	28379	2	71781	uc007btd.1	-74126	ENSMUSG000	Slc16a14	solute carrier family 16 (monocarboxylic acid transporters), member 14
chr1	85009938	85010320	383	384	85010153	73	21.64636	3.79272	17.95485	Adipose_R2_peak_1	Intron (uc007t	1	84906705	84935083	28379	2	71781	uc007btd.1	-74855	ENSMUSG000	Slc16a14	solute carrier family 16 (monocarboxylic acid transporters), member 14
chr1	85010800	85011221	422	423	85010916	52	11.76336	2.96659	8.4373	Adipose_R2_peak_1	Intron (uc007t	1	84906705	84935083	28379	2	71781	uc007btd.1	-75717	ENSMUSG000	Slc16a14	solute carrier family 16 (monocarboxylic acid transporters), member 14
chr1	85011845	85012010	166	167	85011942	51	13.94996	3.40752	10.52365	Adipose_R2_peak_1	Intron (uc007t	1	84906705	84935083	28379	2	71781	uc007btd.1	-76762	ENSMUSG000	Slc16a14	solute carrier family 16 (monocarboxylic acid transporters), member 14
chr1	85012811	85013035	225	226	85012936	35	9.95877	3.31984	6.73562	Adipose_R2_peak_1	Intron (uc007t	1	84906705	84935083	28379	2	71781	uc007btd.1	-77728	ENSMUSG000	Slc16a14	solute carrier family 16 (monocarboxylic acid transporters), member 14
chr1	85012814	85012975	162	163	85012884	24	8.10482	3.52581	5.06071	Adipose_R1_peak_9	Intron (uc007t	1	84906705	84935083	28379	2	71781	uc007btd.1	-77731	ENSMUSG000	Slc16a14	solute carrier family 16 (monocarboxylic acid transporters), member 14
chr1	85014186	85014379	194	195	85014291	31	16.23981	5.75161	12.83549	Adipose_R1_peak_9	Intron (uc007t	1	84906705	84935083	28379	2	71781	uc007btd.1	-79103	ENSMUSG000	Slc16a14	solute carrier family 16 (monocarboxylic acid transporters), member 14
chr1	85014228	85014378	151	152	85014293	34	9.39139	3.22762	6.20474	Adipose_R2_peak_1	Intron (uc007t	1	84906705	84935083	28379	2	71781	uc007btd.1	-79145	ENSMUSG000	Slc16a14	solute carrier family 16 (monocarboxylic acid transporters), member 14

chr1	85018636	85018807	172	173	85018730	24	10.60668	4.49345	7.40961	Adipose_R1_peak_9	Intron (uc007bt	1	84906705	84935083	28379	2	71781	uc007btd.1	-83553	ENSMUSG000	Slc16a14	solute carrier family 16 (monocarboxylic acid transporters), member 14
chr1	85019715	85019985	271	272	85019859	42	26.36523	7.72873	22.71906	Adipose_R1_peak_9	Intron (uc007bt	1	84906705	84935083	28379	2	71781	uc007btd.1	-84632	ENSMUSG000	Slc16a14	solute carrier family 16 (monocarboxylic acid transporters), member 14
chr1	85019746	85019931	186	187	85019859	35	9.95877	3.31984	6.73562	Adipose_R2_peak_1	Intron (uc007bt	1	84906705	84935083	28379	2	71781	uc007btd.1	-84663	ENSMUSG000	Slc16a14	solute carrier family 16 (monocarboxylic acid transporters), member 14
chr1	85025988	85026583	596	597	85026131	53	12.27222	3.02256	8.93144	Adipose_R2_peak_1	3' UTR	1	85088139	85109853	21715	2	381287	uc007btj.1	83270	ENSMUSG000	A530032D15R	RIKEN cDNA A530032D15Rik gene
chr1	85027575	85027766	192	193	85027685	77	38.61857	6.51655	34.60739	Adipose_R2_peak_1	3' UTR	1	85088139	85109853	21715	2	381287	uc007btj.1	82087	ENSMUSG000	A530032D15R	RIKEN cDNA A530032D15Rik gene
chr1	85028284	85028472	189	190	85028399	69	27.87358	5.03989	24.04205	Adipose_R2_peak_1	Exon (uc007bt	1	85088139	85109853	21715	2	381287	uc007btj.1	81381	ENSMUSG000	A530032D15R	RIKEN cDNA A530032D15Rik gene
chr1	85028753	85028938	186	187	85028847	41	10.00801	3.03382	6.78338	Adipose_R2_peak_1	5' UTR	1	85088139	85109853	21715	2	381287	uc007btj.1	80915	ENSMUSG000	A530032D15R	RIKEN cDNA A530032D15Rik gene
chr1	85029089	85029714	626	627	85029210	49	6.11136	2.08021	3.21367	Adipose_R2_peak_1	Intron (uc007bt	1	85088139	85109853	21715	2	381287	uc007btj.1	80139	ENSMUSG000	A530032D15R	RIKEN cDNA A530032D15Rik gene
chr1	85030285	85030651	367	368	85030409	45	15.96501	4.17814	12.45636	Adipose_R2_peak_1	Intron (uc007bt	1	85088139	85109853	21715	2	381287	uc007btj.1	79202	ENSMUSG000	A530032D15R	RIKEN cDNA A530032D15Rik gene
chr1	85031500	85031857	358	359	85031575	25	8.15817	3.46091	5.11339	Adipose_R1_peak_9	Intron (uc007bt	1	85088139	85109853	21715	2	381287	uc007btj.1	77996	ENSMUSG000	A530032D15R	RIKEN cDNA A530032D15Rik gene
chr1	85031502	85031869	368	369	85031604	32	7.20996	2.757	4.21168	Adipose_R2_peak_1	Intron (uc007bt	1	85088139	85109853	21715	2	381287	uc007btj.1	77984	ENSMUSG000	A530032D15R	RIKEN cDNA A530032D15Rik gene
chr1	85034657	85034895	239	240	85034766	62	28.93052	5.80972	25.0743	Adipose_R2_peak_1	Intron (uc007bt	1	85088139	85109853	21715	2	381287	uc007btj.1	74958	ENSMUSG000	A530032D15R	RIKEN cDNA A530032D15Rik gene
chr1	85035384	85035687	304	305	85035466	46	9.32664	2.71407	6.15991	Adipose_R2_peak_1	Intron (uc007bt	1	85088139	85109853	21715	2	381287	uc007btj.1	74166	ENSMUSG000	A530032D15R	RIKEN cDNA A530032D15Rik gene
chr1	85036062	85036204	143	144	85036118	34	8.59097	3.0194	5.47032	Adipose_R2_peak_1	Intron (uc007bt	1	85088139	85109853	21715	2	381287	uc007btj.1	73649	ENSMUSG000	A530032D15R	RIKEN cDNA A530032D15Rik gene
chr1	85037294	85037453	160	161	85037395	39	10.66983	3.27868	7.40755	Adipose_R2_peak_1	Intron (uc007bt	1	85088139	85109853	21715	2	381287	uc007btj.1	72400	ENSMUSG000	A530032D15R	RIKEN cDNA A530032D15Rik gene
chr1	85037823	85037986	164	165	85037915	42	12.1839	3.47413	8.84576	Adipose_R2_peak_1	Intron (uc007bt	1	85088139	85109853	21715	2	381287	uc007btj.1	71867	ENSMUSG000	A530032D15R	RIKEN cDNA A530032D15Rik gene
chr1	85038233	85038468	236	237	85038317	40	9.90559	3.05441	6.70155	Adipose_R2_peak_1	Intron (uc007bt	1	85088139	85109853	21715	2	381287	uc007btj.1	71385	ENSMUSG000	A530032D15R	RIKEN cDNA A530032D15Rik gene
chr1	85038791	85039206	416	417	85038906	31	5.13923	2.26878	2.35358	Adipose_R2_peak_1	Intron (uc007bt	1	85088139	85109853	21715	2	381287	uc007btj.1	70647	ENSMUSG000	A530032D15R	RIKEN cDNA A530032D15Rik gene
chr1	85039460	85040179	720	721	85039590	124	37.12991	3.99319	33.14223	Adipose_R2_peak_1	Intron (uc007bt	1	85088139	85109853	21715	2	381287	uc007btj.1	69674	ENSMUSG000	A530032D15R	RIKEN cDNA A530032D15Rik gene
chr1	85040535	85040869	335	336	85040725	55	14.79439	3.39508	11.33562	Adipose_R2_peak_1	Intron (uc007bt	1	85088139	85109853	21715	2	381287	uc007btj.1	68984	ENSMUSG000	A530032D15R	RIKEN cDNA A530032D15Rik gene
chr1	85041093	85041332	240	241	85041228	115	37.49723	4.28794	33.501	Adipose_R2_peak_1	Intron (uc007bt	1	85088139	85109853	21715	2	381287	uc007btj.1	68521	ENSMUSG000	A530032D15R	RIKEN cDNA A530032D15Rik gene
chr1	85041892	85042021	130	131	85041937	38	6.81466	2.4501	3.83168	Adipose_R2_peak_1	5' UTR	1	85088139	85109853	21715	2	381287	uc007btj.1	67832	ENSMUSG000	A530032D15R	RIKEN cDNA A530032D15Rik gene
chr1	85042986	85043359	374	375	85043190	146	43.44638	4.01081	39.37196	Adipose_R2_peak_1	Intron (uc007bt	1	85088139	85109853	21715	2	381287	uc007btj.1	66494	ENSMUSG000	A530032D15R	RIKEN cDNA A530032D15Rik gene
chr1	85043031	85043345	315	316	85043170	81	12.68927	2.45713	9.42332	Adipose_R1_peak_9	Intron (uc007bt	1	85088139	85109853	21715	2	381287	uc007btj.1	66508	ENSMUSG000	A530032D15R	RIKEN cDNA A530032D15Rik gene
chr1	85045807	85046082	276	277	85045950	80	18.40927	3.13728	14.80899	Adipose_R2_peak_1	Intron (uc007bt	1	85088139	85109853	21715	2	381287	uc007btj.1	63771	ENSMUSG000	A530032D15R	RIKEN cDNA A530032D15Rik gene
chr1	85046290	85046587	298	299	85046431	125	33.68754	3.65673	29.75419	Adipose_R2_peak_1	Intron (uc007bt	1	85088139	85109853	21715	2	381287	uc007btj.1	63266	ENSMUSG000	A530032D15R	RIKEN cDNA A530032D15Rik gene
chr1	85046328	85046527	200	201	85046442	64	6.56384	1.95252	3.62255	Adipose_R1_peak_9	Intron (uc007bt	1	85088139	85109853	21715	2	381287	uc007btj.1	63326	ENSMUSG000	A530032D15R	RIKEN cDNA A530032D15Rik gene
chr1	85046975	85047255	281	282	85047126	87	35.62605	5.24787	31.65691	Adipose_R2_peak_1	Intron (uc007bt	1	85088139	85109853	21715	2	381287	uc007btj.1	62598	ENSMUSG000	A530032D15R	RIKEN cDNA A530032D15Rik gene
chr1	85048594	85048775	182	183	85048703	37	11.12817	3.50427	7.83613	Adipose_R2_peak_1	Intron (uc007bt	1	85088139	85109853	21715	2	381287	uc007btj.1	61078	ENSMUSG000	A530032D15R	RIKEN cDNA A530032D15Rik gene
chr1	85048600	85048858	259	260	85048710	41	24.2904	7.13888	20.69974	Adipose_R1_peak_9	Intron (uc007bt	1	85088139	85109853	21715	2	381287	uc007btj.1	60995	ENSMUSG000	A530032D15R	RIKEN cDNA A530032D15Rik gene
chr1	85056394	85056649	256	257	85056509	52	36.63102	9.52611	32.81188	Adipose_R1_peak_9	Intron (uc007bt	1	85088139	85109853	21715	2	381287	uc007btj.1	53204	ENSMUSG000	A530032D15R	RIKEN cDNA A530032D15Rik gene
chr1	85056407	85056614	208	209	85056522	41	13.59774	3.87315	10.1803	Adipose_R2_peak_1	Intron (uc007bt	1	85088139	85109853	21715	2	381287	uc007btj.1	53239	ENSMUSG000	A530032D15R	RIKEN cDNA A530032D15Rik gene
chr1	85061171	85061363	193	194	85061274	35	9.95877	3.31984	6.73562	Adipose_R2_peak_1	Intron (uc007bt	1	85088139	85109853	21715	2	381287	uc007btj.1	48490	ENSMUSG000	A530032D15R	RIKEN cDNA A530032D15Rik gene
chr1	85062191	85062377	187	188	85062327	31	7.7623	2.95097	4.69388	Adipose_R2_peak_1	Intron (uc007bt	1	85088139	85109853	21715	2	381287	uc007btj.1	47476	ENSMUSG000	A530032D15R	RIKEN cDNA A530032D15Rik gene
chr1	85063189	85063552	364	365	85063404	93	26.42502	3.74011	22.62283	Adipose_R2_peak_1	Intron (uc007bt	1	85088139	85109853	21715	2	381287	uc007btj.1	46301	ENSMUSG000	A530032D15R	RIKEN cDNA A530032D15Rik gene
chr1	85064152	85064495	344	345	85064330	119	29.21506	3.38821	25.35594	Adipose_R2_peak_1	Intron (uc007bt	1	85088139	85109853	21715	2	381287	uc007btj.1	45358	ENSMUSG000	A530032D15R	RIKEN cDNA A530032D15Rik gene
chr1	85067475	85067710	236	237	85067564	58	17.39029	3.73208	13.83047	Adipose_R2_peak_1	Intron (uc007bt	1	85088139	85109853	21715	2	381287	uc007btj.1	42143	ENSMUSG000	A530032D15R	RIKEN cDNA A530032D15Rik gene
chr1	85068052	85069094	1043	1044	85068315	67	17.95395	3.46088	14.37299	Adipose_R2_peak_1	Intron (uc007bt	1	85088139	85109853	21715	2	381287	uc007btj.1	40759	ENSMUSG000	A530032D15R	RIKEN cDNA A530032D15Rik gene
chr1	85069929	85070243	315	316	85070057	54	11.38128	2.83886	8.08147	Adipose_R2_peak_1	Intron (uc007bt	1	85088139	85109853	21715	2	381287	uc007btj.1	39610	ENSMUSG000	A530032D15R	RIKEN cDNA A530032D15Rik gene
chr1	85070881	85070997	117	118	85070929	33	5.85011	2.38198	2.96246	Adipose_R2_peak_1	Intron (uc007bt	1	85088139	85109853	21715	2	381287	uc007btj.1	38856	ENSMUSG000	A530032D15R	RIKEN cDNA A530032D15Rik gene
chr1	85071147	85071295	149	150	85071229	45	6.49234	2.21739	3.54917	Adipose_R2_peak_1	Intron (uc007bt	1	85088139	85109853	21715	2	381287	uc007btj.1	38558	ENSMUSG000	A530032D15R	RIKEN cDNA A530032D15Rik gene
chr1	85072141	85072252	112	113	85072214	36	5.80845	2.28702	2.9223	Adipose_R2_peak_1	Intron (uc007bt	1	85088139	85109853	21715	2	381287	uc007btj.1	37601	ENSMUSG000	A530032D15R	RIKEN cDNA A530032D15Rik gene
chr1	85072446	85072635	190	191	85072559	52	13.10157	3.20706	9.7097	Adipose_R2_peak_1	Intron (uc007bt	1	85088139	85109853	21715	2	381287	uc007btj.1	37218	ENSMUSG000	A530032D15R	RIKEN cDNA A530032D15Rik gene
chr1	85072770	85072930	161	162	85072845	48	11.12698	2.9881	7.83613	Adipose_R2_peak_1	Intron (uc007bt	1	85088139	85109853	21715	2	381287	uc007btj.1	36923	ENSMUSG000	A530032D15R	RIKEN cDNA A530032D15Rik gene
chr1	85073438	85074103	666	667	85073644	54	9.12074	2.47153	5.96115	Adipose_R2_peak_1	Intron (uc007bt	1	85088139	85109853	21715	2	381287	uc007btj.1	35750	ENSMUSG000	A530032D15R	RIKEN cDNA A530032D15Rik gene
chr1	85074781	85075336	556	557	85074915	76	13.40777	2.6205	10.0082	Adipose_R2_peak_1	Intron (uc007bt	1	85088139	85109853	21715	2	381287	uc007btj.1	34517	ENSMUSG000	A530032D15R	RIKEN cDNA A530032D15Rik gene
chr1	85075803	85076218	416	417	85076034	105	28.72321	3.65867	24.87598	Adipose_R2_peak_1	Intron (uc007bt	1	85088139	85109853	21715	2	381287	uc007btj.1	33635	ENSMUSG000	A530032D15R	RIKEN cDNA A530032D15Rik gene
chr1	85076677	85076895	219	220	85076775	83	22.92106	3.61862	19.1942	Adipose_R2_peak_1	Intron (uc007bt	1	85088139	85109853	21715	2	381287	uc007btj.1	32958	ENSMUSG000	A530032D15R	RIKEN cDNA A530032D15Rik gene
chr1	85077129	85077462	334	335	85077248	65	13.57268	2.87719	10.16915	Adipose_R2_peak_1	Intron (uc007bt	1	85088139	85109853	21715	2	381287	uc007btj.1	32391	ENSMUSG000	A530032D15R	RIKEN cDNA A530032D15Rik gene
chr1	85077766	85078009	244	245	85077905	64	20.63449	4.02098	16.96753	Adipose_R2_peak_1	Intron (uc007bt	1	85088139	85109853	21715	2	381287	uc007btj.1	31844	ENSMUSG000	A530032D15R	RIKEN cDNA A530032D15Rik gene
chr1	85078246	85078539	294	295	85078374	67	20.71576	3.89589	17.04779	Adipose_R2_peak_1	Intron (uc007bt	1	85088139	85109853	21715	2	381287	uc007btj.1	31314	ENSMUSG000	A530032D15R	RIKEN cDNA A530032D15Rik gene
chr1	85080387	85080661	275	276	85080516	89	33.46841	4.81583	29.53709	Adipose_R2_peak_1	Intron (uc007bt	1	85088139	85109853	21715	2	381287	uc007btj.1	29192	ENSMUSG000	A530032D15R	RIKEN cDNA A530032D15Rik gene
chr1	85080442	85080585	144	145	85080526	51	9.78137	2.65255	6.65148	Adipose_R1_peak_9	Intron (uc007bt	1	85088139	85109853	21715	2	381287	uc007btj.1	29268	ENSMUSG000	A530032D15R	RIKEN cDNA A530032D15Rik gene
chr1	85080868	85081663	796	797	85080933	49	7.98318	2.39438	4.90662	Adipose_R2_peak_1	Intron (uc007bt	1	85088139	85109853	21715	2	381287	uc007btj.1	28190	ENSMUSG000	A530032D15R	RIKEN cDNA A530032D15Rik gene
chr1	85081186	85081454	269	270	85081318	32	4.91346	2.18602	2.17066	Adipose_R1_peak_9	Intron (uc007bt	1	85088139	85109853	21715	2	381287	uc007btj.1	28399	ENSMUSG000	A530032D15R	RIKEN cDNA A530032D15Rik gene
chr1	85082317	85082529	213	214	85082425	44	9.82659	2.87815	6.62479	Adipose_R2_peak_1	Intron (uc007bt	1	85088139	85109853	21715	2	381287	uc007btj.1	27324	ENSMUSG000	A530032D15R	RIKEN cDNA A530032D15Rik gene
chr1	85083128	85083527	400	401	85083238	65	11.56486	2.59769	8.25949	Adipose_R2_peak_1	Intron (uc007bt	1	85088139	85109853	21715	2	381287	uc007btj.1	26326	ENSMUSG000	A530032D15R	RIKEN cDNA A530032D15Rik gene
chr1	85083843	85084014	172	173	85083901	47	9.47767	2.71191	6.28783	Adipose_R2_peak_1	Intron (uc007bt	1	85088139	85109853	21715	2	381287	uc007btj.1	25839	ENSMUSG000	A530032D15R	RIKEN cDNA A530032D15Rik gene
chr1	85085028	85085154	127	128	85085098	43	6.53404	2.26701	3.58922	Adipose_R2_peak_1	3' UTR	1	85088139	85109853	21715	2	381287	uc007btj.1	24699	ENSMUSG000	A530032D15R	RIKEN cDNA A530032D15Rik gene
chr1	85085423	85086001	579	580	85085535	87	28.52865	4.2141	24.6834	Adipose_R2_peak_1	Intron (uc007bt	1	85088139	85109853	21715	2	381287	uc007btj.1	23852	ENSMUSG000	A530032D15R	RIKEN cDNA A530032D15Rik gene
chr1	85086229	85086790	562	563	85086476	70	14.18664	2.84047	10.7549	Adipose_R2_peak_1	Intron (uc007bt	1	85088139	85109853	21715	2	381287	uc007btj.1	23063	ENSMUSG000	A530032D15R	RIKEN cDNA A530032D15Rik gene
chr1	85087030	85087636	607	608	85087106	54	8.39668	2.35777	5.28327	Adipose_R2_peak_1	Intron (uc007bt	1	85088139	85109853	21715	2	381287	uc007btj.1	22217	ENSMUSG000	A530032D15R	RIKEN cDNA A530032D15Rik gene
chr1	85087816	85088122	307	308	85087973	100	24.50088	3.34382	20.73669	Adipose_R2_peak_1	Intron (uc007bt	1	85088139	85109853	21715	2	381287	uc007btj.1	21731	ENSMUSG000	A530032D15R	RIKEN cDNA A530032D15Rik gene
chr1	85088231	85089330	1100	1101	85088389	113	27.43137	3.34844	23.60419	Adipose_R2_peak_1	3' UTR	1	85088139	85109853	21715	2	381287	uc007btj.1	20523	ENSMUSG000	A530032D15R	RIKEN cDNA A530032D15Rik gene
chr1	85089523	85090438	916	917	85089897	136	44.78901	4.37653	40.69547	Adipose_R2_peak_1	Exon (uc007bt	1	85088139	85109853	21715	2	381287	uc007btj.1	19415	ENSMUSG000	A530032D15R	RIKEN cDNA A530032D15Rik gene
chr1	85089759	85090438	680	681	85089900	73	10.65975	2.34406	7.46225	Adipose_R1_peak_9	Intron (uc007bt	1	85088139	85109853	21715	2	381287	uc007btj.1	19415	ENSMUSG000	A530032D15R	RIKEN cDNA A530032D15Rik gene
chr1	85090720	85091127	408	409	85090885	172	53.61337	4.21553	49.42178	Adipose_R2_peak_1	Exon (uc007bt	1	85088139	85109853	21715	2	381287	uc007btj.1	18726	ENSMUSG000	A530032D15R	RIKEN cDNA A530032D15Rik gene
chr1	85090774	85090975	202	203	85090878	80	10.37632	2.22	7.21011	Adipose_R1_peak_9	Intron (uc007bt	1	85088139	85109853	21715	2	381287	uc007btj.1	18878	ENSMUSG000	A530032D15R	RIKEN cDNA A530032D15Rik gene
chr1	85092200	85092643	444	445	85092401	72	11.69523	2.48437	8.38587	Adipose_R2_peak_1	Intron (uc007bt	1	85088139	85109853	21715	2	381287	uc007btj.1	17210	ENSMUSG000	A530032D15R	RIKEN cDNA A530032D15Rik gene
chr1	85092983	85093181	199	200	85093093	66	15.46478	3.1224	11.97875	Adipose_R2_peak_1	Intron (uc007bt	1	85088139	85109853	21715	2	381287	uc007btj.1	16672	ENSMUSG000	A530032D15R	RIKEN cDNA A530032D15Rik gene
chr1	85093406	85093671	266	267	85093589	60	13.04471	2.93306	9.65434	Adipose_R2_peak_1	Intron (uc007bt	1	85088139	85109853	21715	2	381287	uc007btj.1	16182	ENSMUSG000	A530032D15R	RIKEN cDNA A530032D15Rik gene
chr1	85094182	85094994	813	814	85094393	88	16.66451	2.77034	13.1301	Adipose_R2_peak_1	Exon (uc007bt	1	85088139	85109853	21715	2	381287	uc007btj.1	14859	ENSMUSG000	A530032D15R	RIKEN cDNA A530032D15Rik gene
chr1	85095311	85096041	731	732	85095467	121	24.83318	2.98276	21.06494	Adipose_R2_peak_1	Intron (uc007bt	1	85088139	85109853	21715	2	381287	uc007btj.1	13812	ENSMUSG000	A530032D15R	RIKEN cDNA A530032D15Rik gene
chr1	85095674	85095823	150	151	85095761	44	5.88529	2.12656	3.0043	Adipose_R1_peak_9	Intron (uc007bt	1	85088139	85109853	21715	2	381287	uc007btj.1	14030	ENSMUSG000	A530032D15R	RIKEN cDNA A530032D15Rik gene
chr1	85096263	85096398	136	137	85096323	54	11.8352	2.91516	8.50719	Adipose_R2_peak_1	Intron (uc007bt	1	85088139	85109853	21715	2	381287	uc007btj.1	13455	ENSMUSG000	A530032D15R	RIKEN cDNA A530032D15Rik gene
chr1	85097036	85097322	287	288	85097168	64	16.52469	3.34608	12.99299	Adipose_R2_peak_1	Intron (uc007bt	1	85088139	85109853	21715	2	381287	uc007btj.1	12531	ENSMUSG000	A530032D15R	RIKEN cDNA A530032D15Rik gene
chr1	85097530	85097838	309	310	85097688	63	16.91314	3.4433	13.36223	Adipose_R2_peak_1	Intron (uc007bt	1	85088139	85109853	21715	2	381287	uc007btj.1	12015	ENSMUSG000	A530032D15R	RIKEN cDNA A530032D15Rik gene
chr1	85098262	85098415	154	155	85098351	47	11.32342	3.06319	8.12101	Adipose_R1_peak_9	Intron (uc007bt	1	85088139	85109853	21715	2	381287	uc007btj.1	11438	ENSMUSG000	A530032D15R	RIKEN cDNA A530032D15Rik gene
chr1	85098263	85098955	693	694	85098388	85	25.88848	3.93735	22.09218	Adipose_R2_peak_1	Intron (uc007bt	1	85088139	85109853	21715	2	381287	uc007btj.1	10898	ENSMUSG000	A530032D15R	RIKEN cDNA A530032D15Rik gene
chr1	85099129	85100011	883	884	85099259	65	18.33046	3.59397	14.73132	Adipose_R2_peak_1	Exon (uc007bt	1	85088139	85109853	21715	2	381287	uc007btj.1	9842	ENSMUSG000	A530032D15R	RIKEN cDNA A530032D15Rik gene
chr1	85099871	85100128	258	259	85099923	46	6.4324	2.18719	3.49298	Adipose_R1_peak_9	Intron (uc007bt	1	85088139	85109853	21715	2	381287	uc007btj.1	9725	ENSMUSG000	A530032D15R	RIKEN cDNA A530032D15Rik gene
chr1	85101039	85101315	277	278	85101255	72	26.24537	4.56814	22.44517	Adipose_R2_peak_1	Intron (uc007bt	1	85088139	85109853	21715	2	381287	uc007btj.1	8538	ENSMUSG000	A530032D15R	RIKEN cDNA A530032D15Rik gene
chr1	85101160	85101308	149	150	85101280	47	9.32755	2.68415	6.20159	Adipose_R1_peak_9	Intron (uc007bt	1	85088139	85109853	21715	2	381287	uc007btj.1	8545	ENSMUSG000	A530032D15R	RIKEN cDNA A530032D15Rik gene
chr1	85101543	85102222	680	681	85101655	57	10.62982	2.63886	7.36878	Adipose_R2_peak_1	Intron (uc007bt	1	85088139	85109853	21715	2	381287	uc007btj.1	7631	ENSMUSG000	A530032D15R	RIKEN cDNA A530032D15Rik gene
chr1	85101569	85101761	193	194	85101654	40	10.2084	3.12278	7.04365	Adipose_R1_peak_9	Intron (uc007bt	1	85088139	85109853	21715	2	381287	uc007btj.1	8092	ENSMUSG000	A530032D15R	RIKEN cDNA A530032D15Rik gene
chr1	85102851	85103127	277	278	85102948	44	8.70594	2.65639	5.58099	Adipose_R2_peak_1	Intron (uc007bt	1	85088139	85109853	21715	2	381287	uc007btj.1	6726	ENSMUSG000	A530032D15R	RIKEN cDNA A530032D15Rik gene
chr1	85103827	85104092	266	267	85103961	93	33.88626	4.68605	29.95106	Adipose_R2_peak_1	Intron (uc007bt	1	85088139	85109853	21715	2	381287	uc007btj.1	5761	ENSMUSG000	A530032D15R	RIKEN cDNA A530032D15Rik gene
chr1	85104565	85104755	191	192	85104673	78	21.84435	3.63972	18.14987	Adipose_R2_peak_1	Intron (uc007bt	1	85088139	85109853	21715	2	381287	uc007btj.1	5098	ENSMUSG000	A530032D15R	RIKEN cDNA A530032D15Rik gene
chr1	85105383	85106362	980	981	85105538	93	26.42502	3.74011	22.62283	Adipose_R2_peak_1	Exon (uc007bt	1	85088139	85109853	21715	2	381287	uc007btj.1	3491	ENSMUSG000	A530032D15R	RIKEN cDNA A530032D15Rik gene
chr1	85106560	85106719	160	161	85106649	54	13.58349	3.21799	10.17959	Adipose_R2_peak_1	Exon (uc007bt	1	85088139	85109853	21715	2	381287	uc007btj.1	3134	ENSMUSG000	A530032D15R	RIKEN cDNA A530032D15Rik gene
chr1	85107316	85107842	527	528	85107469	136	30.98641	3.23037	27.0973	Adipose_R2_peak_1	Promoter (2-3	1	85088139	85109853	21715	2	381287	uc007btj.1	2011	ENSMUSG000	A530032D15R	RIKEN cDNA A530032D15Rik gene
chr1	85107361	85107803	443	444	85107464	79	12.93047	2.51453	9.63827	Adipose_R1_peak_9	Promoter (2-3	1	85088139	85109853	21715	2	381287	uc007btj.1	2050	ENSMUSG000	A530032D15R	RIKEN cDNA A530032D15Rik gene
chr1	85108160	85109127	968	969	85108300	81	25.44793	4.02808	21.66439	Adipose_R2_peak_1	Promoter (<=1	1	85088139	85109853	21715	2	381287	uc007btj.1	726	ENSMUSG000	A530032D15R	RIKEN cDNA A530032D15Rik gene
chr1	85108651	85108897	247	248	85108767	74	20.87446	3.64303	17.35055	Adipose_R1_peak_9	Promoter (<=1	1	85088139	85109853	21715	2	381287	uc007btj.1	956	ENSMUSG000	A530032D15R	RIKEN cDNA A530032D15Rik gene

chr1	85109767	85110080	314	315	85109850	66	15.08671	3.06747	11.60881	Adipose_R2_peak_1	Promoter (<=	1	85088139	85109853	21715	2	381287	uc007btj.1	0	ENSMUSG000	A530032D15R	RIKEN cDNA A530032D15Rik gene
chr1	85110282	85110387	106	107	85110327	46	8.05635	2.4783	4.97756	Adipose_R2_peak_1	Promoter (<=	1	85088139	85109853	21715	2	381287	uc007btj.1	-429	ENSMUSG000	A530032D15R	RIKEN cDNA A530032D15Rik gene
chr1	85110705	85110906	202	203	85110811	54	12.32038	2.99774	8.97833	Adipose_R2_peak_1	Promoter (<=	1	85088139	85109853	21715	2	381287	uc007btj.1	-852	ENSMUSG000	A530032D15R	RIKEN cDNA A530032D15Rik gene
chr1	85110760	85110908	149	150	85110829	46	12.98217	3.44443	9.68863	Adipose_R1_peak_9	Promoter (<=	1	85088139	85109853	21715	2	381287	uc007btj.1	-907	ENSMUSG000	A530032D15R	RIKEN cDNA A530032D15Rik gene
chr1	85111084	85111681	598	599	85111243	70	13.05632	2.69276	9.66565	Adipose_R2_peak_1	Promoter (1-2	1	85088139	85109853	21715	2	381287	uc007btj.1	-1231	ENSMUSG000	A530032D15R	RIKEN cDNA A530032D15Rik gene
chr1	85112316	85112575	260	261	85112455	75	23.05785	3.92279	19.32927	Adipose_R2_peak_1	Promoter (2-3	1	85088139	85109853	21715	2	381287	uc007btj.1	-2463	ENSMUSG000	A530032D15R	RIKEN cDNA A530032D15Rik gene
chr1	85112906	85113511	606	607	85113017	88	17.34852	2.84315	13.78956	Adipose_R2_peak_1	Exon (uc007bt	1	85088139	85109853	21715	2	381287	uc007btj.1	-3053	ENSMUSG000	A530032D15R	RIKEN cDNA A530032D15Rik gene
chr1	85113253	85113457	205	206	85113317	76	7.2869	1.92449	4.29203	Adipose_R1_peak_9	Exon (uc007bt	1	85088139	85109853	21715	2	381287	uc007btj.1	-3400	ENSMUSG000	A530032D15R	RIKEN cDNA A530032D15Rik gene
chr1	85113944	85114558	615	616	85114037	71	19.93295	3.61402	16.28666	Adipose_R2_peak_1	Exon (uc007bt	1	85088139	85109853	21715	2	381287	uc007btj.1	-4091	ENSMUSG000	A530032D15R	RIKEN cDNA A530032D15Rik gene
chr1	85114349	85114538	190	191	85114413	43	6.82537	2.32213	3.88042	Adipose_R1_peak_9	Intron (uc007b	1	85088139	85109853	21715	2	381287	uc007btj.1	-4496	ENSMUSG000	A530032D15R	RIKEN cDNA A530032D15Rik gene
chr1	85114788	85114969	182	183	85114887	33	8.29963	2.99369	5.25334	Adipose_R1_peak_9	Intron (uc007b	1	85088139	85109853	21715	2	381287	uc007btj.1	-4935	ENSMUSG000	A530032D15R	RIKEN cDNA A530032D15Rik gene
chr1	85114797	85114920	124	125	85114878	36	7.09094	2.5743	4.0968	Adipose_R2_peak_1	Intron (uc007b	1	85088139	85109853	21715	2	381287	uc007btj.1	-4944	ENSMUSG000	A530032D15R	RIKEN cDNA A530032D15Rik gene
chr1	85117599	85117796	198	199	85117680	42	11.90171	3.40954	8.5716	Adipose_R2_peak_1	Intron (uc007b	1	85088139	85109853	21715	2	381287	uc007btj.1	-7746	ENSMUSG000	A530032D15R	RIKEN cDNA A530032D15Rik gene
chr1	85118342	85118473	132	133	85118392	33	8.38285	3.01548	5.26988	Adipose_R2_peak_1	Intron (uc007b	1	85088139	85109853	21715	2	381287	uc007btj.1	-8489	ENSMUSG000	A530032D15R	RIKEN cDNA A530032D15Rik gene
chr1	85119182	85119423	242	243	85119294	54	16.04041	3.67006	12.53012	Adipose_R2_peak_1	Intron (uc007b	1	85088139	85109853	21715	2	381287	uc007btj.1	-9329	ENSMUSG000	A530032D15R	RIKEN cDNA A530032D15Rik gene
chr1	85120534	85121047	514	515	85120827	71	19.11233	3.49378	15.48939	Adipose_R2_peak_1	Intron (uc007b	1	85088139	85109853	21715	2	381287	uc007btj.1	-10681	ENSMUSG000	A530032D15R	RIKEN cDNA A530032D15Rik gene
chr1	85121240	85121546	307	308	85121399	52	11.25863	2.87823	7.96264	Adipose_R2_peak_1	Intron (uc007b	1	85088139	85109853	21715	2	381287	uc007btj.1	-11387	ENSMUSG000	A530032D15R	RIKEN cDNA A530032D15Rik gene
chr1	85123127	85123433	307	308	85123353	84	38.30453	5.88738	34.29595	Adipose_R2_peak_1	Exon (uc029qc	1	85088139	85109853	21715	2	381287	uc007btj.1	-13274	ENSMUSG000	A530032D15R	RIKEN cDNA A530032D15Rik gene
chr1	85123726	85123919	194	195	85123776	27	5.77405	2.5821	2.88921	Adipose_R2_peak_1	Intron (uc007b	1	85088139	85109853	21715	2	381287	uc007btj.1	-13873	ENSMUSG000	A530032D15R	RIKEN cDNA A530032D15Rik gene
chr1	85124644	85124770	127	128	85124703	29	6.74062	2.76653	3.75989	Adipose_R2_peak_1	Intron (uc007b	1	85088139	85109853	21715	2	381287	uc007btj.1	-14791	ENSMUSG000	A530032D15R	RIKEN cDNA A530032D15Rik gene
chr1	85124887	85124997	111	112	85124957	29	6.74062	2.76653	3.75989	Adipose_R2_peak_1	Intron (uc007b	1	85088139	85109853	21715	2	381287	uc007btj.1	-15034	ENSMUSG000	A530032D15R	RIKEN cDNA A530032D15Rik gene
chr1	85126706	85126957	252	253	85126834	42	8.58973	2.6966	5.4691	Adipose_R2_peak_1	Intron (uc007b	1	85088139	85109853	21715	2	381287	uc007btj.1	-16853	ENSMUSG000	A530032D15R	RIKEN cDNA A530032D15Rik gene
chr1	85129525	85129663	139	140	85129588	21	8.25416	3.87909	5.20839	Adipose_R1_peak_9	Intron (uc007b	1	85088139	85109853	21715	2	381287	uc007btj.1	-19672	ENSMUSG000	A530032D15R	RIKEN cDNA A530032D15Rik gene
chr1	85131105	85131343	239	240	85131228	36	10.5378	3.41206	7.27949	Adipose_R2_peak_1	Intron (uc007b	1	85088139	85109853	21715	2	381287	uc007btj.1	-21252	ENSMUSG000	A530032D15R	RIKEN cDNA A530032D15Rik gene
chr1	85132317	85132676	360	361	85132418	38	9.62931	3.08176	6.43417	Adipose_R2_peak_1	Intron (uc007b	1	85088139	85109853	21715	2	381287	uc007btj.1	-22464	ENSMUSG000	A530032D15R	RIKEN cDNA A530032D15Rik gene
chr1	85139452	85139566	115	116	85139487	26	5.31252	2.48988	2.47999	Adipose_R2_peak_1	Intron (uc007b	1	85088139	85109853	21715	2	381287	uc007btj.1	-29599	ENSMUSG000	A530032D15R	RIKEN cDNA A530032D15Rik gene
chr1	85147829	85148069	241	242	85147937	41	10.97089	3.24842	7.69925	Adipose_R2_peak_1	Intron (uc007b	1	85088139	85109853	21715	2	381287	uc007btj.1	-37976	ENSMUSG000	A530032D15R	RIKEN cDNA A530032D15Rik gene
chr1	85153605	85154011	407	408	85153720	41	13.17256	3.76789	9.77902	Adipose_R2_peak_1	Intron (uc007b	1	85088139	85109853	21715	2	381287	uc007btj.1	-43752	ENSMUSG000	A530032D15R	RIKEN cDNA A530032D15Rik gene
chr1	85154650	85154849	200	201	85154740	35	9.95877	3.31984	6.73562	Adipose_R2_peak_1	Intron (uc007b	1	85199611	85213762	14152	1	665378	uc007btq.1	-44762	ENSMUSG000	Gm7609	predicted pseudogene 7609
chr1	85156959	85157141	183	184	85157054	31	7.7623	2.95097	4.69388	Adipose_R2_peak_1	Intron (uc007b	1	85199611	85213762	14152	1	665378	uc007btq.1	-42470	ENSMUSG000	Gm7609	predicted pseudogene 7609
chr1	85158000	85158179	180	181	85158095	45	16.22858	4.24202	12.70278	Adipose_R2_peak_1	Intron (uc007b	1	85199611	85213762	14152	1	665378	uc007btq.1	-41432	ENSMUSG000	Gm7609	predicted pseudogene 7609
chr1	85158747	85158930	184	185	85158859	34	7.58054	2.76568	4.54248	Adipose_R2_peak_1	Intron (uc007b	1	85199611	85213762	14152	1	665378	uc007btq.1	-40681	ENSMUSG000	Gm7609	predicted pseudogene 7609
chr1	85160976	85161139	164	165	85161085	31	7.7623	2.95097	4.69388	Adipose_R2_peak_1	Intron (uc007b	1	85199611	85213762	14152	1	665378	uc007btq.1	-38472	ENSMUSG000	Gm7609	predicted pseudogene 7609
chr1	85161520	85161760	241	242	85161609	46	11.77934	3.19469	8.45267	Adipose_R2_peak_1	Intron (uc007b	1	85199611	85213762	14152	1	665378	uc007btq.1	-37851	ENSMUSG000	Gm7609	predicted pseudogene 7609
chr1	85162129	85162346	218	219	85162240	34	8.44544	2.98225	5.36134	Adipose_R1_peak_9	Intron (uc007b	1	85199611	85213762	14152	1	665378	uc007btq.1	-37265	ENSMUSG000	Gm7609	predicted pseudogene 7609
chr1	85162152	85162341	190	191	85162214	56	18.81401	4.10391	15.20665	Adipose_R2_peak_1	Intron (uc007b	1	85199611	85213762	14152	1	665378	uc007btq.1	-37270	ENSMUSG000	Gm7609	predicted pseudogene 7609
chr1	85163005	85163280	276	277	85163141	62	20.06823	4.01997	16.42013	Adipose_R2_peak_1	Intron (uc007b	1	85199611	85213762	14152	1	665378	uc007btq.1	-36331	ENSMUSG000	Gm7609	predicted pseudogene 7609
chr1	85163099	85163236	138	139	85163159	31	7.85376	2.97601	4.81233	Adipose_R1_peak_9	Intron (uc007b	1	85199611	85213762	14152	1	665378	uc007btq.1	-36375	ENSMUSG000	Gm7609	predicted pseudogene 7609
chr1	85164198	85164644	447	448	85164456	126	34.60415	3.71532	30.65611	Adipose_R2_peak_1	Intron (uc007b	1	85199611	85213762	14152	1	665378	uc007btq.1	-34967	ENSMUSG000	Gm7609	predicted pseudogene 7609
chr1	85165022	85165174	153	154	85165067	36	7.28361	2.61844	4.25666	Adipose_R2_peak_1	Intron (uc007b	1	85199611	85213762	14152	1	665378	uc007btq.1	-34437	ENSMUSG000	Gm7609	predicted pseudogene 7609
chr1	85165346	85165469	124	125	85165395	35	8.81087	3.0261	5.68248	Adipose_R2_peak_1	Intron (uc007b	1	85199611	85213762	14152	1	665378	uc007btq.1	-34142	ENSMUSG000	Gm7609	predicted pseudogene 7609
chr1	85165809	85165927	119	120	85165880	30	6.28187	2.59727	3.34646	Adipose_R2_peak_1	Intron (uc007b	1	85199611	85213762	14152	1	665378	uc007btq.1	-33684	ENSMUSG000	Gm7609	predicted pseudogene 7609
chr1	85166650	85166791	142	143	85166713	28	6.57715	2.76807	3.6356	Adipose_R1_peak_9	Intron (uc007b	1	85199611	85213762	14152	1	665378	uc007btq.1	-32820	ENSMUSG000	Gm7609	predicted pseudogene 7609
chr1	85166674	85167386	713	714	85166739	43	12.44224	3.47949	9.0824	Adipose_R2_peak_1	Intron (uc007b	1	85199611	85213762	14152	1	665378	uc007btq.1	-32225	ENSMUSG000	Gm7609	predicted pseudogene 7609
chr1	85168134	85168361	228	229	85168226	42	12.89241	3.63921	9.52013	Adipose_R2_peak_1	Intron (uc007b	1	85199611	85213762	14152	1	665378	uc007btq.1	-31250	ENSMUSG000	Gm7609	predicted pseudogene 7609
chr1	85168530	85168953	424	425	85168607	34	7.84824	2.83196	4.77676	Adipose_R2_peak_1	Intron (uc007b	1	85199611	85213762	14152	1	665378	uc007btq.1	-30658	ENSMUSG000	Gm7609	predicted pseudogene 7609
chr1	85169402	85169793	392	393	85169596	59	16.11365	3.46477	12.60214	Adipose_R2_peak_1	Intron (uc007b	1	85199611	85213762	14152	1	665378	uc007btq.1	-29818	ENSMUSG000	Gm7609	predicted pseudogene 7609
chr1	85170055	85170191	137	138	85170116	35	9.93889	3.31464	6.73324	Adipose_R2_peak_1	Intron (uc007b	1	85199611	85213762	14152	1	665378	uc007btq.1	-29420	ENSMUSG000	Gm7609	predicted pseudogene 7609
chr1	85170695	85171305	611	612	85170746	31	7.7623	2.95097	4.69388	Adipose_R2_peak_1	Intron (uc007b	1	85199611	85213762	14152	1	665378	uc007btq.1	-28306	ENSMUSG000	Gm7609	predicted pseudogene 7609
chr1	85172458	85172750	293	294	85172552	33	8.65855	3.08819	5.53548	Adipose_R2_peak_1	Intron (uc007b	1	85199611	85213762	14152	1	665378	uc007btq.1	-26861	ENSMUSG000	Gm7609	predicted pseudogene 7609
chr1	85175851	85176144	294	295	85175991	69	34.76717	6.45524	30.8116	Adipose_R2_peak_1	Intron (uc007b	1	85199611	85213762	14152	1	665378	uc007btq.1	-23467	ENSMUSG000	Gm7609	predicted pseudogene 7609
chr1	85175856	85176172	317	318	85175994	49	28.44708	7.24558	24.76371	Adipose_R1_peak_9	Intron (uc007b	1	85199611	85213762	14152	1	665378	uc007btq.1	-23439	ENSMUSG000	Gm7609	predicted pseudogene 7609
chr1	85176313	85176427	115	116	85176394	28	6.25023	2.67431	3.31626	Adipose_R2_peak_1	Intron (uc007b	1	85199611	85213762	14152	1	665378	uc007btq.1	-23184	ENSMUSG000	Gm7609	predicted pseudogene 7609
chr1	85177795	85178055	261	262	85177922	40	21.58633	6.33838	18.04766	Adipose_R1_peak_9	Intron (uc007b	1	85199611	85213762	14152	1	665378	uc007btq.1	-21556	ENSMUSG000	Gm7609	predicted pseudogene 7609
chr1	85177855	85178050	196	197	85177935	38	11.72962	3.59649	8.4047	Adipose_R2_peak_1	Intron (uc007b	1	85199611	85213762	14152	1	665378	uc007btq.1	-21561	ENSMUSG000	Gm7609	predicted pseudogene 7609
chr1	85179622	85180165	544	545	85179699	38	11.64014	3.57371	8.33251	Adipose_R2_peak_1	Intron (uc007b	1	85199611	85213762	14152	1	665378	uc007btq.1	-19446	ENSMUSG000	Gm7609	predicted pseudogene 7609
chr1	85180430	85180612	183	184	85180530	62	20.67122	4.12834	17.00375	Adipose_R2_peak_1	Exon (uc007bt	1	85199611	85213762	14152	1	665378	uc007btq.1	-18999	ENSMUSG000	Gm7609	predicted pseudogene 7609
chr1	85181629	85182188	560	561	85181774	50	11.52988	2.99244	8.22533	Adipose_R2_peak_1	Intron (uc007b	1	85199611	85213762	14152	1	665378	uc007btq.1	-17423	ENSMUSG000	Gm7609	predicted pseudogene 7609
chr1	85182811	85183320	510	511	85182943	96	36.99084	4.97154	33.00424	Adipose_R2_peak_1	Intron (uc007b	1	85199611	85213762	14152	1	665378	uc007btq.1	-16291	ENSMUSG000	Gm7609	predicted pseudogene 7609
chr1	85183438	85183727	290	291	85183602	103	32.39195	4.11554	28.47724	Adipose_R2_peak_1	Intron (uc007b	1	85199611	85213762	14152	1	665378	uc007btq.1	-15884	ENSMUSG000	Gm7609	predicted pseudogene 7609
chr1	85184037	85184163	127	128	85184091	31	6.41306	2.59195	3.47278	Adipose_R2_peak_1	Intron (uc007b	1	85199611	85213762	14152	1	665378	uc007btq.1	-15448	ENSMUSG000	Gm7609	predicted pseudogene 7609
chr1	85185196	85185345	150	151	85185275	39	8.68771	2.82417	5.56386	Adipose_R2_peak_1	Intron (uc007b	1	85199611	85213762	14152	1	665378	uc007btq.1	-14266	ENSMUSG000	Gm7609	predicted pseudogene 7609
chr1	85185568	85186281	714	715	85185636	31	6.50874	2.6168	3.56471	Adipose_R2_peak_1	Exon (uc007bt	1	85199611	85213762	14152	1	665378	uc007btq.1	-13330	ENSMUSG000	Gm7609	predicted pseudogene 7609
chr1	85186494	85186680	187	188	85186585	45	13.44397	3.59593	10.04346	Adipose_R2_peak_1	Intron (uc007b	1	85199611	85213762	14152	1	665378	uc007btq.1	-12931	ENSMUSG000	Gm7609	predicted pseudogene 7609
chr1	85187487	85187664	178	179	85187560	49	16.92144	4.12996	13.37038	Adipose_R2_peak_1	Intron (uc007b	1	85199611	85213762	14152	1	665378	uc007btq.1	-11947	ENSMUSG000	Gm7609	predicted pseudogene 7609
chr1	85187783	85188002	220	221	85187914	55	22.55115	4.96281	18.83775	Adipose_R2_peak_1	Exon (uc007bt	1	85199611	85213762	14152	1	665378	uc007btq.1	-11609	ENSMUSG000	Gm7609	predicted pseudogene 7609
chr1	85189296	85189439	144	145	85189353	41	13.59774	3.87315	10.1803	Adipose_R2_peak_1	Intron (uc007b	1	85199611	85213762	14152	1	665378	uc007btq.1	-10172	ENSMUSG000	Gm7609	predicted pseudogene 7609
chr1	85195125	85195521	397	398	85195390	71	14.66673	2.88048	11.21091	Adipose_R2_peak_1	Intron (uc007b	1	85199611	85213762	14152	1	665378	uc007btq.1	-4090	ENSMUSG000	Gm7609	predicted pseudogene 7609
chr1	85196260	85196360	101	102	85196271	27	5.03438	2.37103	2.25232	Adipose_R2_peak_1	Intron (uc007b	1	85199611	85213762	14152	1	665378	uc007btq.1	-3251	ENSMUSG000	Gm7609	predicted pseudogene 7609
chr1	85196887	85196996	110	111	85196906	27	5.04834	2.37496	2.26591	Adipose_R2_peak_1	Promoter (2-3	1	85199611	85213762	14152	1	665378	uc007btq.1	-2615	ENSMUSG000	Gm7609	predicted pseudogene 7609
chr1	85197341	85197898	558	559	85197411	37	6.37156	2.38305	3.43281	Adipose_R2_peak_1	Promoter (1-2	1	85199611	85213762	14152	1	665378	uc007btq.1	-1713	ENSMUSG000	Gm7609	predicted pseudogene 7609
chr1	85198404	85198774	371	372	85198611	112	27.03728	3.33249	23.2214	Adipose_R2_peak_1	Promoter (<=	1	85199611	85213762	14152	1	665378	uc007btq.1	-837	ENSMUSG000	Gm7609	predicted pseudogene 7609
chr1	85198979	85199533	555	556	85199082	64	11.44535	2.60193	8.14345	Adipose_R2_peak_1	Promoter (<=	1	85199611	85213762	14152	1	665378	uc007btq.1	-78	ENSMUSG000	Gm7609	predicted pseudogene 7609
chr1	85199960	85200210	251	252	85200087	92	37.46188	5.24578	33.46593	Adipose_R2_peak_1	Promoter (<=	1	85199611	85213762	14152	1	665378	uc007btq.1	349	ENSMUSG000	Gm7609	predicted pseudogene 7609
chr1	85202576	85202749	174	175	85202637	68	27.66478	5.06796	23.83529	Adipose_R2_peak_1	Promoter (2-3	1	85199611	85213762	14152	1	665378	uc007btq.1	2965	ENSMUSG000	Gm7609	predicted pseudogene 7609
chr1	85204666	85204923	258	259	85204804	49	17.56671	4.27523	14.00387	Adipose_R2_peak_1	Intron (uc007b	1	85199611	85213762	14152	1	665378	uc007btq.1	5055	ENSMUSG000	Gm7609	predicted pseudogene 7609
chr1	85206550	85206969	420	421	85206630	33	7.09729	2.68666	4.1027	Adipose_R2_peak_1	Intron (uc007b	1	85199611	85213762	14152	1	665378	uc007btq.1	6939	ENSMUSG000	Gm7609	predicted pseudogene 7609
chr1	85207234	85207333	100	101	85207285	27	5.77405	2.5821	2.88921	Adipose_R2_peak_1	Intron (uc007b	1	85199611	85213762	14152	1	665378	uc007btq.1	7623	ENSMUSG000	Gm7609	predicted pseudogene 7609
chr1	85207929	85208235	307	308	85208102	104	46.76157	5.92266	42.64501	Adipose_R2_peak_1	5' UTR	1	85199611	85213762	14152	1	665378	uc007btq.1	8318	ENSMUSG000	Gm7609	predicted pseudogene 7609
chr1	85209464	85209563	100	101	85209509	31	7.7623	2.95097	4.69388	Adipose_R2_peak_1	Intron (uc007b	1	85199611	85213762	14152	1	665378	uc007btq.1	9853	ENSMUSG000	Gm7609	predicted pseudogene 7609
chr1	85210650	85210782	133	134	85210722	31	7.7623	2.95097	4.69388	Adipose_R2_peak_1	Intron (uc007b	1	85199611	85213762	14152	1	665378	uc007btq.1	11039	ENSMUSG000	Gm7609	predicted pseudogene 7609
chr1	85212929	85213115	187	188	85213057	37	11.12817	3.50427	7.83613	Adipose_R2_peak_1	Exon (uc007bt	1	85199611	85213762	14152	1	665378	uc007btq.1	13318	ENSMUSG000	Gm7609	predicted pseudogene 7609
chr1	85213824	85214001	178	179	85213914	48	18.29934	4.51867	14.70076	Adipose_R2_peak_1	Exon (uc007bt	1	85199611	85213762	14152	1	665378	uc007btq.1	14213	ENSMUSG000	Gm7609	predicted pseudogene 7609
chr1	85214968	85215257	290	291	85215147	41	13.59774	3.87315	10.1803	Adipose_R2_peak_1	Exon (uc007bt	1	85199611	85213762	14152	1	665378	uc007btq.1	15357	ENSMUSG000	Gm7609	predicted pseudogene 7609
chr1	85216176	85216358	183	184	85216276	39	12.34187	3.68871	8.98501	Adipose_R2_peak_1	Intron (uc007b	1	85199611	85213762	14152	1	665378	uc007btq.1	16565	ENSMUSG000	Gm7609	predicted pseudogene 7609
chr1	85226173	85226408	236	237	85226307	52	36.63102	9.52611	32.81188	Adipose_R1_peak_9	Intron (uc007b	1	85199611	85213762	14152	1	665378	uc007btq.1	26562	ENSMUSG000	Gm7609	predicted pseudogene 7609
chr1	85226544	85226798	255	256	85226687	42	26.36523	7.72873	22.71906	Adipose_R1_peak_9	Intron (uc007b	1	85199611	85213762	14152	1	665378	uc007btq.1	26933	ENSMUSG000	Gm7609	predicted pseudogene 7609
chr1	85227912	85228180	269	270	85228048	56	40.97333	10.24506	37.09402	Adipose_R1_peak_9	Intron (uc007b	1	85199611	85213762	14152	1	665378	uc007btq.1	28301	ENSMUSG000	Gm7609	predicted pseudogene 7609
chr1	85227937	85228175	239	240	85228052	52	21.18119	4.88754	17.49655	Adipose_R2_peak_1	Intron (uc007b	1	85199611	85213762	14152	1	665378	uc007btq.1	28326	ENSMUSG000	Gm7609	predicted pseudogene 7609
chr1	85231013	85231197	185	186	85231107	35	9.95877	3.31984	6.73562	Adipose_R2_peak_1	Intron (uc007b	1	85112418	85261886	149469	2	620078	uc007btn.1	30689	ENSMUSG000	C130026I21Ril	RIKEN cDNA C130026I21 gene
chr1	85231600	85231790	191	192	85231657	42	9.1787	2.81775	6.01749	Adipose_R2_peak_1	Intron (uc007b	1	85112418	85261886	149469	2	620078	uc007btn.1	30096	ENSMUSG000	C130026I21Ril	RIKEN cDNA C130026I21 gene
chr1	85232522	85232945	424	425	85232573	47	7.09435	2.28361	4.10012	Adipose_R2_peak_1	Intron (uc007b	1	85112418	85261886	149469	2	620078	uc007btn.1	28941	ENSMUSG000	C130026I21Ril	RIKEN cDNA C130026I21 gene
chr1	85232729	85232862	134	135	85232828	38	7.75898	2.6555	4.71856	Adipose_R1_peak_9	Intron (uc007b	1	85112418	85261886	149469	2	620078	uc007btn.1	29024	ENSMUSG000	C130026I21Ril	RIKEN cDNA C130026I21 gene
chr1	85233209	85233571	363	364	85233340	44	8.62365	2.64039	5.50183	Adipose_R2_peak_1	Intron (uc007b	1	85112418	85261886	149469	2	620078	uc007btn.1	28315	ENSMUSG000	C130026I21Ril	RIKEN cDNA C130026I21 gene

chr1	85233783	85233979	197	198	85233839	50	17.25488	4.13966	13.69774	Adipose_R2_peak_1	Intron (uc007bt	1	85112418	85261886	149469	2	620078	uc007btn.1	27907	ENSMUSG000	C130026I21Ril	RIKEN cDNA C130026I21 gene
chr1	85234320	85234453	134	135	85234369	37	7.36756	2.60283	4.33723	Adipose_R2_peak_1	Intron (uc007bt	1	85112418	85261886	149469	2	620078	uc007btn.1	27433	ENSMUSG000	C130026I21Ril	RIKEN cDNA C130026I21 gene
chr1	85234584	85234880	297	298	85234722	74	22.43948	3.87118	18.72743	Adipose_R2_peak_1	Intron (uc007bt	1	85112418	85261886	149469	2	620078	uc007btn.1	27006	ENSMUSG000	C130026I21Ril	RIKEN cDNA C130026I21 gene
chr1	85234677	85234817	141	142	85234736	39	10.1878	3.16516	7.02316	Adipose_R1_peak_	Intron (uc007bt	1	85112418	85261886	149469	2	620078	uc007btn.1	27069	ENSMUSG000	C130026I21Ril	RIKEN cDNA C130026I21 gene
chr1	85235261	85235443	183	184	85235350	58	19.57535	4.13987	15.9448	Adipose_R2_peak_1	Intron (uc007bt	1	85112418	85261886	149469	2	620078	uc007btn.1	26443	ENSMUSG000	C130026I21Ril	RIKEN cDNA C130026I21 gene
chr1	85235672	85235765	94	95	85235724	37	7.22086	2.57004	4.21917	Adipose_R2_peak_1	Intron (uc007bt	1	85112418	85261886	149469	2	620078	uc007btn.1	26121	ENSMUSG000	C130026I21Ril	RIKEN cDNA C130026I21 gene
chr1	85236119	85236423	305	306	85236272	63	13.08716	2.85835	9.69588	Adipose_R2_peak_1	Intron (uc007bt	1	85112418	85261886	149469	2	620078	uc007btn.1	25463	ENSMUSG000	C130026I21Ril	RIKEN cDNA C130026I21 gene
chr1	85237654	85237801	148	149	85237740	49	7.11169	2.24684	4.11655	Adipose_R2_peak_1	Intron (uc007bt	1	85112418	85261886	149469	2	620078	uc007btn.1	24085	ENSMUSG000	C130026I21Ril	RIKEN cDNA C130026I21 gene
chr1	85238563	85239272	710	711	85238666	48	12.11627	3.17874	8.78012	Adipose_R2_peak_1	Intron (uc007bt	1	85112418	85261886	149469	2	620078	uc007btn.1	22614	ENSMUSG000	C130026I21Ril	RIKEN cDNA C130026I21 gene
chr1	85239492	85240539	1048	1049	85239526	36	5.11938	2.13661	2.33409	Adipose_R2_peak_1	Intron (uc007bt	1	85112418	85261886	149469	2	620078	uc007btn.1	21347	ENSMUSG000	C130026I21Ril	RIKEN cDNA C130026I21 gene
chr1	85240665	85241139	475	476	85240756	52	12.42861	3.08497	9.06911	Adipose_R2_peak_1	Intron (uc007bt	1	85112418	85261886	149469	2	620078	uc007btn.1	20747	ENSMUSG000	C130026I21Ril	RIKEN cDNA C130026I21 gene
chr1	85241317	85241605	289	290	85241463	37	5.33328	2.16015	2.49977	Adipose_R2_peak_1	Exon (uc029qc	1	85112418	85261886	149469	2	620078	uc007btn.1	20281	ENSMUSG000	C130026I21Ril	RIKEN cDNA C130026I21 gene
chr1	85241930	85242097	168	169	85242027	56	16.27111	3.62002	12.74431	Adipose_R2_peak_1	Intron (uc007bt	1	85112418	85261886	149469	2	620078	uc007btn.1	19789	ENSMUSG000	C130026I21Ril	RIKEN cDNA C130026I21 gene
chr1	85242700	85243707	1008	1009	85242862	51	11.9099	3.02677	8.57954	Adipose_R2_peak_1	Intron (uc007bt	1	85112418	85261886	149469	2	620078	uc007btn.1	18179	ENSMUSG000	C130026I21Ril	RIKEN cDNA C130026I21 gene
chr1	85243376	85243615	240	241	85243448	31	5.5753	2.37792	2.75554	Adipose_R1_peak_	Intron (uc007bt	1	85112418	85261886	149469	2	620078	uc007btn.1	18271	ENSMUSG000	C130026I21Ril	RIKEN cDNA C130026I21 gene
chr1	85244233	85245405	1173	1174	85244364	67	20.23336	3.8177	16.58251	Adipose_R2_peak_1	Intron (uc007bt	1	85112418	85261886	149469	2	620078	uc007btn.1	16481	ENSMUSG000	C130026I21Ril	RIKEN cDNA C130026I21 gene
chr1	85244575	85244931	357	358	85244721	54	18.95049	4.25042	15.47325	Adipose_R1_peak_	Intron (uc007bt	1	85112418	85261886	149469	2	620078	uc007btn.1	16955	ENSMUSG000	C130026I21Ril	RIKEN cDNA C130026I21 gene
chr1	85245749	85245984	236	237	85245858	98	39.85149	5.26985	35.82449	Adipose_R2_peak_1	Intron (uc007bt	1	85112418	85261886	149469	2	620078	uc007btn.1	15902	ENSMUSG000	C130026I21Ril	RIKEN cDNA C130026I21 gene
chr1	85245766	85245910	145	146	85245849	54	12.45139	3.02023	9.18694	Adipose_R1_peak_	Intron (uc007bt	1	85112418	85261886	149469	2	620078	uc007btn.1	15976	ENSMUSG000	C130026I21Ril	RIKEN cDNA C130026I21 gene
chr1	85246216	85247868	1653	1654	85246373	86	26.13402	3.93374	22.33496	Adipose_R2_peak_1	3' UTR	1	85112418	85261886	149469	2	620078	uc007btn.1	14018	ENSMUSG000	C130026I21Ril	RIKEN cDNA C130026I21 gene
chr1	85246225	85246563	339	340	85246281	32	5.63957	2.36206	2.81893	Adipose_R1_peak_	3' UTR	1	85112418	85261886	149469	2	620078	uc007btn.1	15323	ENSMUSG000	C130026I21Ril	RIKEN cDNA C130026I21 gene
chr1	85247614	85247827	214	215	85247668	61	6.32857	1.95379	3.44082	Adipose_R1_peak_	Intron (uc007bt	1	85112418	85261886	149469	2	620078	uc007btn.1	14059	ENSMUSG000	C130026I21Ril	RIKEN cDNA C130026I21 gene
chr1	85248043	85248184	142	143	85248120	49	8.136	2.42052	5.05355	Adipose_R2_peak_1	Intron (uc007bt	1	85112418	85261886	149469	2	620078	uc007btn.1	13702	ENSMUSG000	C130026I21Ril	RIKEN cDNA C130026I21 gene
chr1	85248561	85249822	1262	1263	85248792	90	24.00924	3.54342	20.25982	Adipose_R2_peak_1	Intron (uc007bt	1	85112418	85261886	149469	2	620078	uc007btn.1	12064	ENSMUSG000	C130026I21Ril	RIKEN cDNA C130026I21 gene
chr1	85249950	85251154	1205	1206	85250267	77	17.00653	3.03721	13.45392	Adipose_R2_peak_1	Intron (uc007bt	1	85112418	85261886	149469	2	620078	uc007btn.1	10732	ENSMUSG000	C130026I21Ril	RIKEN cDNA C130026I21 gene
chr1	85251589	85251927	339	340	85251739	102	34.69074	4.41941	30.74185	Adipose_R2_peak_1	Intron (uc007bt	1	85112418	85261886	149469	2	620078	uc007btn.1	9959	ENSMUSG000	C130026I21Ril	RIKEN cDNA C130026I21 gene
chr1	85251762	85251907	146	147	85251821	43	8.12562	2.57286	5.08122	Adipose_R1_peak_	Intron (uc007bt	1	85112418	85261886	149469	2	620078	uc007btn.1	9979	ENSMUSG000	C130026I21Ril	RIKEN cDNA C130026I21 gene
chr1	85252067	85252220	154	155	85252142	53	8.13222	2.33577	5.04985	Adipose_R2_peak_1	Intron (uc007bt	1	85112418	85261886	149469	2	620078	uc007btn.1	9666	ENSMUSG000	C130026I21Ril	RIKEN cDNA C130026I21 gene
chr1	85252436	85252662	227	228	85252553	72	16.56853	3.10803	13.03607	Adipose_R2_peak_1	Intron (uc007bt	1	85112418	85261886	149469	2	620078	uc007btn.1	9224	ENSMUSG000	C130026I21Ril	RIKEN cDNA C130026I21 gene
chr1	85252895	85253229	335	336	85253113	52	8.29917	2.3826	5.1897	Adipose_R2_peak_1	Intron (uc007bt	1	85112418	85261886	149469	2	620078	uc007btn.1	8657	ENSMUSG000	C130026I21Ril	RIKEN cDNA C130026I21 gene
chr1	85253345	85253506	162	163	85253434	66	11.39152	2.55434	8.09125	Adipose_R2_peak_1	Intron (uc007bt	1	85112418	85261886	149469	2	620078	uc007btn.1	8380	ENSMUSG000	C130026I21Ril	RIKEN cDNA C130026I21 gene
chr1	85254329	85254967	639	640	85254387	55	9.94193	2.57877	6.73562	Adipose_R2_peak_1	Exon (uc007bt	1	85112418	85261886	149469	2	620078	uc007btn.1	6919	ENSMUSG000	C130026I21Ril	RIKEN cDNA C130026I21 gene
chr1	85255069	85255479	411	412	85255203	139	39.72453	3.86318	35.69863	Adipose_R2_peak_1	Intron (uc007bt	1	85112418	85261886	149469	2	620078	uc007btn.1	6407	ENSMUSG000	C130026I21Ril	RIKEN cDNA C130026I21 gene
chr1	85255645	85255997	353	354	85255808	82	15.24156	2.72304	11.7602	Adipose_R2_peak_1	Intron (uc007bt	1	85112418	85261886	149469	2	620078	uc007btn.1	5889	ENSMUSG000	C130026I21Ril	RIKEN cDNA C130026I21 gene
chr1	85256107	85256648	542	543	85256219	71	13.31801	2.70578	9.92087	Adipose_R2_peak_1	Intron (uc007bt	1	85112418	85261886	149469	2	620078	uc007btn.1	5238	ENSMUSG000	C130026I21Ril	RIKEN cDNA C130026I21 gene
chr1	85257141	85257609	469	470	85257408	81	26.47351	4.17341	22.67081	Adipose_R2_peak_1	Exon (uc007bt	1	85112418	85261886	149469	2	620078	uc007btn.1	4277	ENSMUSG000	C130026I21Ril	RIKEN cDNA C130026I21 gene
chr1	85257731	85257943	213	214	85257836	70	20.79955	3.78306	17.13021	Adipose_R2_peak_1	Exon (uc007bt	1	85112418	85261886	149469	2	620078	uc007btn.1	3943	ENSMUSG000	C130026I21Ril	RIKEN cDNA C130026I21 gene
chr1	85258342	85258913	572	573	85258467	49	13.10245	3.33016	9.80824	Adipose_R1_peak_	Promoter (2-3	1	85112418	85261886	149469	2	620078	uc007btn.1	2973	ENSMUSG000	C130026I21Ril	RIKEN cDNA C130026I21 gene
chr1	85258361	85260003	1643	1644	85258462	50	9.61708	2.65116	6.42234	Adipose_R2_peak_1	Promoter (1-2	1	85112418	85261886	149469	2	620078	uc007btn.1	1883	ENSMUSG000	C130026I21Ril	RIKEN cDNA C130026I21 gene
chr1	85259526	85259932	407	408	85259804	54	9.73418	2.56928	6.60457	Adipose_R1_peak_	Promoter (1-2	1	85112418	85261886	149469	2	620078	uc007btn.1	1954	ENSMUSG000	C130026I21Ril	RIKEN cDNA C130026I21 gene
chr1	85260388	85260765	378	379	85260415	38	5.95832	2.26854	3.06659	Adipose_R2_peak_1	Promoter (1-2	1	85112418	85261886	149469	2	620078	uc007btn.1	1121	ENSMUSG000	C130026I21Ril	RIKEN cDNA C130026I21 gene
chr1	85261346	85261520	175	176	85261422	49	13.16346	3.34218	9.77018	Adipose_R2_peak_1	Promoter (<=	1	85112418	85261886	149469	2	620078	uc007btn.1	366	ENSMUSG000	C130026I21Ril	RIKEN cDNA C130026I21 gene
chr1	85263204	85263474	271	272	85263360	61	15.68108	3.31757	12.17824	Adipose_R2_peak_1	Promoter (1-2	1	85112418	85261886	149469	2	620078	uc007btn.1	-1318	ENSMUSG000	C130026I21Ril	RIKEN cDNA C130026I21 gene
chr1	85263986	85264144	159	160	85264040	42	11.38181	3.29223	8.082	Adipose_R2_peak_1	Promoter (2-3	1	85112418	85261886	149469	2	620078	uc007btn.1	-2100	ENSMUSG000	C130026I21Ril	RIKEN cDNA C130026I21 gene
chr1	85265275	85265628	354	355	85265346	48	7.94199	2.40978	4.86755	Adipose_R2_peak_1	Intron (uc007bt	1	85112418	85261886	149469	2	620078	uc007btn.1	-3389	ENSMUSG000	C130026I21Ril	RIKEN cDNA C130026I21 gene
chr1	85265867	85266002	136	137	85265889	25	4.93383	2.41801	2.19067	Adipose_R1_peak_	Intron (uc007bt	1	85112418	85261886	149469	2	620078	uc007btn.1	-3981	ENSMUSG000	C130026I21Ril	RIKEN cDNA C130026I21 gene
chr1	85266488	85266765	278	279	85266652	48	14.71533	3.70895	11.36359	Adipose_R1_peak_	Intron (uc007bt	1	85083075	85270566	187492	2	381287	uc007bti.1	3801	ENSMUSG000	A530032D15R	RIKEN cDNA A530032D15Rik gene
chr1	85266610	85266741	132	133	85266668	40	8.45142	2.73586	5.33621	Adipose_R2_peak_1	Intron (uc007bt	1	85083075	85270566	187492	2	381287	uc007bti.1	3825	ENSMUSG000	A530032D15R	RIKEN cDNA A530032D15Rik gene
chr1	85267497	85268217	721	722	85267568	45	12.05341	3.29577	8.71884	Adipose_R2_peak_1	Promoter (2-3	1	85083075	85270566	187492	2	381287	uc007bti.1	2349	ENSMUSG000	A530032D15R	RIKEN cDNA A530032D15Rik gene
chr1	85268436	85268769	334	335	85268641	47	12.7146	3.34146	9.34736	Adipose_R2_peak_1	Promoter (1-2	1	85083075	85270566	187492	2	381287	uc007bti.1	1797	ENSMUSG000	A530032D15R	RIKEN cDNA A530032D15Rik gene
chr1	85268592	85268736	145	146	85268671	33	9.4275	3.29534	6.30061	Adipose_R1_peak_	Promoter (1-2	1	85083075	85270566	187492	2	381287	uc007bti.1	1830	ENSMUSG000	A530032D15R	RIKEN cDNA A530032D15Rik gene
chr1	85269548	85270111	564	565	85269626	52	11.36268	2.89634	8.06336	Adipose_R2_peak_1	Promoter (<=	1	85083075	85270566	187492	2	381287	uc007bti.1	455	ENSMUSG000	A530032D15R	RIKEN cDNA A530032D15Rik gene
chr1	85269557	85269723	167	168	85269617	30	6.34282	2.61353	3.45471	Adipose_R1_peak_	Promoter (<=	1	85083075	85270566	187492	2	381287	uc007bti.1	843	ENSMUSG000	A530032D15R	RIKEN cDNA A530032D15Rik gene
chr1	85270723	85270823	101	102	85270799	43	7.46552	2.44453	4.43163	Adipose_R2_peak_1	Promoter (<=	1	85083075	85270566	187492	2	381287	uc007bti.1	-157	ENSMUSG000	A530032D15R	RIKEN cDNA A530032D15Rik gene
chr1	85271038	85271474	437	438	85271306	187	75.27257	5.45607	70.89455	Adipose_R2_peak_1	Promoter (<=	1	85083075	85270566	187492	2	381287	uc007bti.1	-472	ENSMUSG000	A530032D15R	RIKEN cDNA A530032D15Rik gene
chr1	85271189	85271411	223	224	85271295	69	7.3959	2.00404	4.3996	Adipose_R1_peak_	Promoter (<=	1	85083075	85270566	187492	2	381287	uc007bti.1	-623	ENSMUSG000	A530032D15R	RIKEN cDNA A530032D15Rik gene
chr1	85273371	85273845	475	476	85273501	108	20.75164	2.83607	17.08316	Adipose_R2_peak_1	Promoter (2-3	1	85083075	85270566	187492	2	381287	uc007bti.1	-2805	ENSMUSG000	A530032D15R	RIKEN cDNA A530032D15Rik gene
chr1	85273406	85273547	142	143	85273476	63	7.75808	2.12089	4.71768	Adipose_R1_peak_	Promoter (2-3	1	85083075	85270566	187492	2	381287	uc007bti.1	-2840	ENSMUSG000	A530032D15R	RIKEN cDNA A530032D15Rik gene
chr1	85274027	85274905	879	880	85274102	58	9.36474	2.42628	6.19665	Adipose_R2_peak_1	Intron (uc007bt	1	85083075	85270566	187492	2	381287	uc007bti.1	-3461	ENSMUSG000	A530032D15R	RIKEN cDNA A530032D15Rik gene
chr1	85275174	85275625	452	453	85275439	132	39.83717	4.03657	35.81035	Adipose_R2_peak_1	Exon (uc007bt	1	85083075	85270566	187492	2	381287	uc007bti.1	-4608	ENSMUSG000	A530032D15R	RIKEN cDNA A530032D15Rik gene
chr1	85275803	85277246	1444	1445	85275904	89	25.58973	3.7636	21.80471	Adipose_R2_peak_1	Exon (uc007bt	1	85083075	85270566	187492	2	381287	uc007bti.1	-5237	ENSMUSG000	A530032D15R	RIKEN cDNA A530032D15Rik gene
chr1	85277462	85277905	444	445	85277556	75	21.56494	3.70583	17.87457	Adipose_R2_peak_1	Intron (uc007bt	1	85083075	85270566	187492	2	381287	uc007bti.1	-6896	ENSMUSG000	A530032D15R	RIKEN cDNA A530032D15Rik gene
chr1	85277503	85277635	133	134	85277563	47	8.5613	2.54423	5.47609	Adipose_R1_peak_	Intron (uc007bt	1	85083075	85270566	187492	2	381287	uc007bti.1	-6937	ENSMUSG000	A530032D15R	RIKEN cDNA A530032D15Rik gene
chr1	85278263	85278832	570	571	85278491	57	11.93013	2.84276	8.5992	Adipose_R2_peak_1	Intron (uc007bt	1	85083075	85270566	187492	2	381287	uc007bti.1	-7697	ENSMUSG000	A530032D15R	RIKEN cDNA A530032D15Rik gene
chr1	85279082	85279414	333	334	85279184	81	27.33367	4.29787	23.50735	Adipose_R2_peak_1	Intron (uc007bt	1	85083075	85270566	187492	2	381287	uc007bti.1	-8516	ENSMUSG000	A530032D15R	RIKEN cDNA A530032D15Rik gene
chr1	85279797	85279967	171	172	85279867	46	8.80012	2.61537	5.67226	Adipose_R2_peak_1	Intron (uc007bt	1	85083075	85270566	187492	2	381287	uc007bti.1	-9231	ENSMUSG000	A530032D15R	RIKEN cDNA A530032D15Rik gene
chr1	85280485	85280654	170	171	85280573	63	18.84392	3.76091	15.23619	Adipose_R2_peak_1	Intron (uc007bt	1	85083075	85270566	187492	2	381287	uc007bti.1	-9919	ENSMUSG000	A530032D15R	RIKEN cDNA A530032D15Rik gene
chr1	85281021	85281514	494	495	85281152	77	24.50659	4.05472	20.74229	Adipose_R2_peak_1	Intron (uc007bt	1	85083075	85270566	187492	2	381287	uc007bti.1	-10455	ENSMUSG000	A530032D15R	RIKEN cDNA A530032D15Rik gene
chr1	85281720	85281965	246	247	85281848	112	33.26036	3.93755	29.3309	Adipose_R2_peak_1	Intron (uc007bt	1	85083075	85270566	187492	2	381287	uc007bti.1	-11154	ENSMUSG000	A530032D15R	RIKEN cDNA A530032D15Rik gene
chr1	85281738	85281933	196	197	85281845	71	16.4056	3.11313	13.00041	Adipose_R1_peak_	Intron (uc007bt	1	85083075	85270566	187492	2	381287	uc007bti.1	-11172	ENSMUSG000	A530032D15R	RIKEN cDNA A530032D15Rik gene
chr1	85282163	85282437	275	276	85282260	62	12.49828	2.79758	9.13681	Adipose_R2_peak_1	Intron (uc007bt	1	85083075	85270566	187492	2	381287	uc007bti.1	-11597	ENSMUSG000	A530032D15R	RIKEN cDNA A530032D15Rik gene
chr1	85282178	85282384	207	208	85282272	43	7.31813	2.41618	4.32286	Adipose_R1_peak_	Intron (uc007bt	1	85083075	85270566	187492	2	381287	uc007bti.1	-11612	ENSMUSG000	A530032D15R	RIKEN cDNA A530032D15Rik gene
chr1	85283182	85283291	110	111	85283231	35	6.67727	2.5127	3.72687	Adipose_R2_peak_1	Intron (uc007bt	1	85083075	85270566	187492	2	381287	uc007bti.1	-12616	ENSMUSG000	A530032D15R	RIKEN cDNA A530032D15Rik gene
chr1	85284557	85284743	187	188	85284632	32	6.18262	2.49622	3.28225	Adipose_R2_peak_1	Intron (uc007bt	1	85083075	85270566	187492	2	381287	uc007bti.1	-13991	ENSMUSG000	A530032D15R	RIKEN cDNA A530032D15Rik gene
chr1	85285000	85285234	235	236	85285104	61	23.19561	4.66642	19.46509	Adipose_R2_peak_1	Intron (uc007bt	1	85083075	85270566	187492	2	381287	uc007bti.1	-14434	ENSMUSG000	A530032D15R	RIKEN cDNA A530032D15Rik gene
chr1	85285462	85285821	360	361	85285655	79	17.89328	3.09855	14.31321	Adipose_R2_peak_1	Exon (uc029qc	1	85083075	85270566	187492	2	381287	uc007bti.1	-14896	ENSMUSG000	A530032D15R	RIKEN cDNA A530032D15Rik gene
chr1	85286017	85286379	363	364	85286132	44	12.73705	3.49273	9.36907	Adipose_R2_peak_1	Intron (uc007bt	1	85083075	85270566	187492	2	381287	uc007bti.1	-15451	ENSMUSG000	A530032D15R	RIKEN cDNA A530032D15Rik gene
chr1	85286584	85286788	205	206	85286699	48	16.04076	3.99718	12.53046	Adipose_R2_peak_1	Intron (uc007bt	1	85083075	85270566	187492	2	381287	uc007bti.1	-16018	ENSMUSG000	A530032D15R	RIKEN cDNA A530032D15Rik gene
chr1	85287293	85287579	287	288	85287422	32	6.40688	2.55233	3.46688	Adipose_R2_peak_1	Intron (uc007bt	1	85083075	85270566	187492	2	381287	uc007bti.1	-16727	ENSMUSG000	A530032D15R	RIKEN cDNA A530032D15Rik gene
chr1	85287723	85288276	554	555	85287893	40	6.77555	2.38649	3.79376	Adipose_R2_peak_1	Intron (uc007bt	1	85083075	85270566	187492	2	381287	uc007bti.1	-17157	ENSMUSG000	A530032D15R	RIKEN cDNA A530032D15Rik gene
chr1	85288529	85288652	124	125	85288567	32	5.97985	2.44585	3.08744	Adipose_R2_peak_1	Intron (uc007bt	1	85083075	85270566	187492	2	381287	uc007bti.1	-17963	ENSMUSG000	A530032D15R	RIKEN cDNA A530032D15Rik gene
chr1	85288792	85289089	298	299	85288928	63	17.43854	3.52814	13.87809	Adipose_R2_peak_1	Intron (uc007bt	1	85083075	85270566	187492	2	381287	uc007bti.1	-18226	ENSMUSG000	A530032D15R	RIKEN cDNA A530032D15Rik gene
chr1	85288802	85289075	274	275	85288940	36	8.06585	2.80063	5.02218	Adipose_R1_peak_	Intron (uc007bt	1	85083075	85270566	187492	2	381287	uc007bti.1	-18236	ENSMUSG000	A530032D15R	RIKEN cDNA A530032D15Rik gene
chr1	85289443	85289614	172	173	85289537	47	15.67802	3.97817	12.17525	Adipose_R2_peak_1	Exon (uc029qc	1	85083075	85270566	187492	2	381287	uc007bti.1	-18877	ENSMUSG000	A530032D15R	RIKEN cDNA A530032D15Rik gene
chr1	85289766	85289995	230	231	85289879	43	13.95147	3.82934	10.6277	Adipose_R1_peak_	Exon (uc029qc	1	85083075	85270566	187492	2	381287	uc007bti.1	-19200	ENSMUSG000	A530032D15R	RIKEN cDNA A530032D15Rik gene
chr1	85289793	85290034	242	243	85289890	56	18.01537	3.94801	14.43328	Adipose_R2_peak_1	Intron (uc007bt	1	85083075	85270566	187492	2	381287	uc007bti.1	-19227	ENSMUSG000	A530032D15R	RIKEN cDNA A530032D15Rik gene
chr1	85290181	85290462	282	283	85290272	31	5.75604	2.42358	2.88921	Adipose_R2_peak_1	Intron (uc007bt	1	85083075	85270566	187492	2	381287	uc007bti.1	-19615	ENSMUSG000	A530032D15R	RIKEN cDNA A530032D15Rik gene
chr1	85291009	85291300	292	293	85291182	44	13.80675	3.73437	10.38404	Adipose_R2_peak_1	Intron (uc007bt	1	85083075	85270566	187492	2	381287	uc007bti.1	-20443	ENSMUSG000	A530032D15R	RIKEN cDNA A530032D15Rik gene
chr1	85292127	85292328	202	203	85292245	45	10.55496	2.9875	7.2961	Adipose_R2_peak_1	Intron (uc007bt	1	85083075	85270566	187492	2	381287	uc007bti.1	-21561	ENSMUSG000	A530032D15R	RIKEN cDNA A530032D15Rik gene
chr1	85292464	85292680	217	218	85292629	42	8.79064	2.73768	5.66306	Adipose_R2_peak_1	Intron (uc007bt	1	85083075	85270566	187492	2	381287	uc007bti.1	-21898	ENSMUSG000	A530032D15R	RIKEN cDNA A530032D15Rik gene
chr1	85292960	85293073	114	115	85293055	38	6.68685	2.42274	3.73613	Adipose_R2_peak_1	Intron (uc007bt	1	85083075	85270566	187492	2	381287	uc007bti.1	-22394	ENSMUSG000	A530032D15R	RIKEN cDNA A530032D15Rik gene
chr1	85293254	85293619	366	367	85293355	42	6.95188	2.37047	3.96363	Adipose_R2_peak_1	Intron (uc007bt	1	85083075	85270566	187492	2	381287	uc007bti.1	-22688	ENSMUSG000	A530032D15R	RIKEN cDNA A530032D15Rik gene
chr1	85293903	85294214	312	313	85294035	58	12.23383	2.86296	8.89444	Adipose_R2_peak_1	Intron (uc007bt	1	85083075	85270566	187492	2	381287	uc007bti.1	-23337	ENSMUSG000	A530032D15R	RIKEN cDNA A530032D15Rik gene
chr1	85294067	85294211	145	146	85294127	33	6.38561	2.51124	3.48999	Adipose_R1_peak_	Intron (uc007bt	1	85083075	85270566	187492	2	381287	uc007bti.1	-23501	ENSMUSG000	A530032D15R	RIKEN cDNA A530032D15Rik gene

chr1	85294656	85294845	190	191	85294725	60	12.36004	2.82828	9.00239	Adipose_R2_peak_1	Intron (uc007b	1	85083075	85270566	187492	2	381287	uc007bti.1	-24090	ENSMUSG000	A530032D15R	RIKEN cDNA A530032D15Rik gene
chr1	85295166	85295329	164	165	85295208	32	6.05665	2.46489	3.16115	Adipose_R2_peak_1	Intron (uc007b	1	85083075	85270566	187492	2	381287	uc007bti.1	-24600	ENSMUSG000	A530032D15R	RIKEN cDNA A530032D15Rik gene
chr1	85296088	85296274	187	188	85296130	32	5.80138	2.40179	2.91536	Adipose_R2_peak_1	Intron (uc007b	1	85083075	85270566	187492	2	381287	uc007bti.1	-25522	ENSMUSG000	A530032D15R	RIKEN cDNA A530032D15Rik gene
chr1	85296379	85296675	297	298	85296436	36	6.5266	2.44656	3.58192	Adipose_R2_peak_1	Intron (uc007b	1	85083075	85270566	187492	2	381287	uc007bti.1	-25813	ENSMUSG000	A530032D15R	RIKEN cDNA A530032D15Rik gene
chr1	85296869	85297235	367	368	85297073	50	13.07917	3.28299	9.68807	Adipose_R2_peak_1	Intron (uc007b	1	85083075	85270566	187492	2	381287	uc007bti.1	-26303	ENSMUSG000	A530032D15R	RIKEN cDNA A530032D15Rik gene
chr1	85297479	85297653	175	176	85297606	28	6.25023	2.67431	3.31626	Adipose_R2_peak_1	Intron (uc007b	1	85083075	85270566	187492	2	381287	uc007bti.1	-26913	ENSMUSG000	A530032D15R	RIKEN cDNA A530032D15Rik gene
chr1	85298287	85298423	137	138	85298348	37	11.12817	3.50427	7.83613	Adipose_R2_peak_1	Intron (uc007b	1	85083075	85270566	187492	2	381287	uc007bti.1	-27721	ENSMUSG000	A530032D15R	RIKEN cDNA A530032D15Rik gene
chr1	85299248	85299540	293	294	85299390	58	23.44759	4.92918	19.70531	Adipose_R2_peak_1	Intron (uc007b	1	85083075	85270566	187492	2	381287	uc007bti.1	-28682	ENSMUSG000	A530032D15R	RIKEN cDNA A530032D15Rik gene
chr1	85299303	85299466	164	165	85299392	34	11.48715	3.80621	8.25548	Adipose_R1_peak_9	Intron (uc007b	1	85083075	85270566	187492	2	381287	uc007bti.1	-28737	ENSMUSG000	A530032D15R	RIKEN cDNA A530032D15Rik gene
chr1	85304500	85304664	165	166	85304571	33	8.83596	3.1354	5.68652	Adipose_R2_peak_1	Intron (uc007b	1	85083075	85270566	187492	2	381287	uc007bti.1	-33934	ENSMUSG000	A530032D15R	RIKEN cDNA A530032D15Rik gene
chr1	85305164	85305330	167	168	85305234	26	5.29021	2.48331	2.47999	Adipose_R2_peak_1	Intron (uc007b	1	85083075	85270566	187492	2	381287	uc007bti.1	-34598	ENSMUSG000	A530032D15R	RIKEN cDNA A530032D15Rik gene
chr1	85305462	85305662	201	202	85305584	31	7.59917	2.90653	4.55975	Adipose_R2_peak_1	Intron (uc007b	1	85083075	85270566	187492	2	381287	uc007bti.1	-34896	ENSMUSG000	A530032D15R	RIKEN cDNA A530032D15Rik gene
chr1	85305763	85306293	531	532	85305953	36	7.36814	2.6379	4.33764	Adipose_R2_peak_1	Intron (uc007b	1	85083075	85270566	187492	2	381287	uc007bti.1	-35197	ENSMUSG000	A530032D15R	RIKEN cDNA A530032D15Rik gene
chr1	85305913	85306276	364	365	85305971	24	6.38485	2.92745	3.48999	Adipose_R1_peak_9	Intron (uc007b	1	85083075	85270566	187492	2	381287	uc007bti.1	-35347	ENSMUSG000	A530032D15R	RIKEN cDNA A530032D15Rik gene
chr1	85306947	85307269	323	324	85307081	44	13.91593	3.75955	10.49063	Adipose_R2_peak_1	Intron (uc007b	1	85083075	85270566	187492	2	381287	uc007bti.1	-36381	ENSMUSG000	A530032D15R	RIKEN cDNA A530032D15Rik gene
chr1	85307905	85308247	343	344	85308098	86	27.481	4.11221	23.65325	Adipose_R2_peak_1	Intron (uc007b	1	85083075	85270566	187492	2	381287	uc007bti.1	-37339	ENSMUSG000	A530032D15R	RIKEN cDNA A530032D15Rik gene
chr1	85307987	85308202	216	217	85308123	47	14.00429	3.61086	10.68021	Adipose_R1_peak_9	Intron (uc007b	1	85083075	85270566	187492	2	381287	uc007bti.1	-37421	ENSMUSG000	A530032D15R	RIKEN cDNA A530032D15Rik gene
chr1	85308653	85309146	494	495	85308719	43	6.72816	2.3037	3.75989	Adipose_R2_peak_1	5' UTR	1	85083075	85270566	187492	2	381287	uc007bti.1	-38087	ENSMUSG000	A530032D15R	RIKEN cDNA A530032D15Rik gene
chr1	85308821	85309058	238	239	85308897	39	9.11316	2.91906	6.01526	Adipose_R1_peak_9	Intron (uc007b	1	85083075	85270566	187492	2	381287	uc007bti.1	-38255	ENSMUSG000	A530032D15R	RIKEN cDNA A530032D15Rik gene
chr1	85309501	85309877	377	378	85309630	58	11.87613	2.80693	8.54691	Adipose_R2_peak_1	Intron (uc007b	1	85083075	85270566	187492	2	381287	uc007bti.1	-38935	ENSMUSG000	A530032D15R	RIKEN cDNA A530032D15Rik gene
chr1	85310059	85310480	422	423	85310136	39	9.10305	2.9168	5.94392	Adipose_R2_peak_1	Intron (uc007b	1	85083075	85270566	187492	2	381287	uc007bti.1	-39493	ENSMUSG000	A530032D15R	RIKEN cDNA A530032D15Rik gene
chr1	85310897	85311223	327	328	85311090	65	21.19476	4.069	17.50992	Adipose_R2_peak_1	Intron (uc007b	1	85083075	85270566	187492	2	381287	uc007bti.1	-40331	ENSMUSG000	A530032D15R	RIKEN cDNA A530032D15Rik gene
chr1	85310920	85311119	200	201	85311022	34	7.5236	2.75167	4.52572	Adipose_R1_peak_9	Intron (uc007b	1	85083075	85270566	187492	2	381287	uc007bti.1	-40354	ENSMUSG000	A530032D15R	RIKEN cDNA A530032D15Rik gene
chr1	85311419	85311772	354	355	85311499	49	9.27212	2.61784	6.10745	Adipose_R2_peak_1	Intron (uc007b	1	85083075	85270566	187492	2	381287	uc007bti.1	-40853	ENSMUSG000	A530032D15R	RIKEN cDNA A530032D15Rik gene
chr1	85311892	85312083	192	193	85312008	65	14.31863	2.98422	10.87063	Adipose_R2_peak_1	Intron (uc007b	1	85083075	85270566	187492	2	381287	uc007bti.1	-41326	ENSMUSG000	A530032D15R	RIKEN cDNA A530032D15Rik gene
chr1	85312668	85312816	149	150	85312748	44	10.71956	3.06046	7.45555	Adipose_R2_peak_1	Intron (uc007b	1	85083075	85270566	187492	2	381287	uc007bti.1	-42102	ENSMUSG000	A530032D15R	RIKEN cDNA A530032D15Rik gene
chr1	85312966	85313160	195	196	85313016	39	7.86941	2.64541	4.79686	Adipose_R2_peak_1	Intron (uc007b	1	85083075	85270566	187492	2	381287	uc007bti.1	-42400	ENSMUSG000	A530032D15R	RIKEN cDNA A530032D15Rik gene
chr1	85314073	85314815	743	744	85314175	52	11.38262	2.89982	8.08273	Adipose_R2_peak_1	5' UTR	1	85083075	85270566	187492	2	381287	uc007bti.1	-43507	ENSMUSG000	A530032D15R	RIKEN cDNA A530032D15Rik gene
chr1	85315637	85316172	536	537	85315655	40	6.4765	2.32583	3.53392	Adipose_R2_peak_1	Intron (uc007b	1	85083075	85270566	187492	2	381287	uc007bti.1	-45071	ENSMUSG000	A530032D15R	RIKEN cDNA A530032D15Rik gene
chr1	85317104	85317577	474	475	85317200	55	10.8101	2.71739	7.5433	Adipose_R2_peak_1	5' UTR	1	85083075	85270566	187492	2	381287	uc007bti.1	-46538	ENSMUSG000	A530032D15R	RIKEN cDNA A530032D15Rik gene
chr1	85317986	85318281	296	297	85318096	78	14.40509	2.70118	10.9553	Adipose_R2_peak_1	Intron (uc007b	1	85083075	85270566	187492	2	381287	uc007bti.1	-47420	ENSMUSG000	A530032D15R	RIKEN cDNA A530032D15Rik gene
chr1	85318678	85318998	321	322	85318753	68	12.16191	2.6169	8.82438	Adipose_R2_peak_1	Intron (uc007b	1	85083075	85270566	187492	2	381287	uc007bti.1	-48112	ENSMUSG000	A530032D15R	RIKEN cDNA A530032D15Rik gene
chr1	85319250	85320238	989	990	85319362	81	15.55309	2.77706	12.05625	Adipose_R2_peak_1	Intron (uc007b	1	85083075	85270566	187492	2	381287	uc007bti.1	-48684	ENSMUSG000	A530032D15R	RIKEN cDNA A530032D15Rik gene
chr1	85320554	85320687	134	135	85320625	42	9.97785	2.98582	6.81489	Adipose_R1_peak_9	Intron (uc007b	1	85083075	85270566	187492	2	381287	uc007bti.1	-49988	ENSMUSG000	A530032D15R	RIKEN cDNA A530032D15Rik gene
chr1	85320573	85320687	115	116	85320636	56	10.79056	2.68853	7.52431	Adipose_R2_peak_1	Intron (uc007b	1	85083075	85270566	187492	2	381287	uc007bti.1	-50007	ENSMUSG000	A530032D15R	RIKEN cDNA A530032D15Rik gene
chr1	85320932	85321127	196	197	85321022	73	19.94743	3.54548	16.301	Adipose_R2_peak_1	Intron (uc007b	1	85083075	85270566	187492	2	381287	uc007bti.1	-50366	ENSMUSG000	A530032D15R	RIKEN cDNA A530032D15Rik gene
chr1	85321315	85321577	263	264	85321456	65	11.46247	2.58375	8.16016	Adipose_R2_peak_1	Intron (uc007b	1	85083075	85270566	187492	2	381287	uc007bti.1	-50749	ENSMUSG000	A530032D15R	RIKEN cDNA A530032D15Rik gene
chr1	85321879	85322239	361	362	85321935	48	5.63112	2.01561	2.78694	Adipose_R2_peak_1	Intron (uc007b	1	85083075	85270566	187492	2	381287	uc007bti.1	-51313	ENSMUSG000	A530032D15R	RIKEN cDNA A530032D15Rik gene
chr1	85322695	85322867	173	174	85322751	49	8.04387	2.40475	4.96534	Adipose_R2_peak_1	Intron (uc007b	1	85083075	85270566	187492	2	381287	uc007bti.1	-52129	ENSMUSG000	A530032D15R	RIKEN cDNA A530032D15Rik gene
chr1	85323059	85323177	119	120	85323078	40	4.89523	2.01134	2.11809	Adipose_R2_peak_1	Intron (uc007b	1	85083075	85270566	187492	2	381287	uc007bti.1	-52493	ENSMUSG000	A530032D15R	RIKEN cDNA A530032D15Rik gene
chr1	85323456	85323690	235	236	85323563	57	10.8452	2.6722	7.57739	Adipose_R2_peak_1	Intron (uc007b	1	85083075	85270566	187492	2	381287	uc007bti.1	-52890	ENSMUSG000	A530032D15R	RIKEN cDNA A530032D15Rik gene
chr1	85324456	85324594	139	140	85324514	73	11.0032	2.38486	7.73083	Adipose_R2_peak_1	Intron (uc007b	1	85083075	85270566	187492	2	381287	uc007bti.1	-53890	ENSMUSG000	A530032D15R	RIKEN cDNA A530032D15Rik gene
chr1	85324800	85324920	121	122	85324845	54	7.86509	2.27525	4.79272	Adipose_R2_peak_1	Intron (uc007b	1	85083075	85270566	187492	2	381287	uc007bti.1	-54234	ENSMUSG000	A530032D15R	RIKEN cDNA A530032D15Rik gene
chr1	85325288	85325519	232	233	85325390	78	25.67048	4.18434	21.88444	Adipose_R2_peak_1	Intron (uc007b	1	85083075	85270566	187492	2	381287	uc007bti.1	-54722	ENSMUSG000	A530032D15R	RIKEN cDNA A530032D15Rik gene
chr1	85326115	85326516	402	403	85326218	56	12.11666	2.90157	8.7805	Adipose_R2_peak_1	Intron (uc007b	1	85083075	85270566	187492	2	381287	uc007bti.1	-55549	ENSMUSG000	A530032D15R	RIKEN cDNA A530032D15Rik gene
chr1	85327149	85327909	761	762	85327274	56	7.41187	2.17309	4.38008	Adipose_R2_peak_1	5' UTR	1	85083075	85270566	187492	2	381287	uc007bti.1	-56583	ENSMUSG000	A530032D15R	RIKEN cDNA A530032D15Rik gene
chr1	85328961	85329142	182	183	85329030	71	21.22083	3.80753	17.53564	Adipose_R2_peak_1	Intron (uc007b	1	85083075	85270566	187492	2	381287	uc007bti.1	-58395	ENSMUSG000	A530032D15R	RIKEN cDNA A530032D15Rik gene
chr1	85329255	85329378	124	125	85329329	42	5.78085	2.14523	2.89577	Adipose_R2_peak_1	Intron (uc007b	1	85083075	85270566	187492	2	381287	uc007bti.1	-58689	ENSMUSG000	A530032D15R	RIKEN cDNA A530032D15Rik gene
chr1	85329666	85330941	1276	1277	85329782	95	24.46449	3.46093	20.70089	Adipose_R2_peak_1	Intron (uc007b	1	85083075	85270566	187492	2	381287	uc007bti.1	-59100	ENSMUSG000	A530032D15R	RIKEN cDNA A530032D15Rik gene
chr1	85331230	85331434	205	206	85331251	43	6.63318	2.28573	3.68434	Adipose_R2_peak_1	Intron (uc007b	1	85083075	85270566	187492	2	381287	uc007bti.1	-60664	ENSMUSG000	A530032D15R	RIKEN cDNA A530032D15Rik gene
chr1	85332133	85332424	292	293	85332165	38	6.56864	2.39752	3.62256	Adipose_R2_peak_1	Intron (uc007b	1	85083075	85270566	187492	2	381287	uc007bti.1	-61567	ENSMUSG000	A530032D15R	RIKEN cDNA A530032D15Rik gene
chr1	85332596	85332766	171	172	85332686	54	12.86333	3.09146	9.49188	Adipose_R2_peak_1	Intron (uc007b	1	85083075	85270566	187492	2	381287	uc007bti.1	-62030	ENSMUSG000	A530032D15R	RIKEN cDNA A530032D15Rik gene
chr1	85332958	85333568	611	612	85333170	74	18.32999	3.28919	14.73085	Adipose_R2_peak_1	Intron (uc007b	1	85083075	85270566	187492	2	381287	uc007bti.1	-62392	ENSMUSG000	A530032D15R	RIKEN cDNA A530032D15Rik gene
chr1	85334309	85334585	277	278	85334420	39	6.54786	2.36605	3.60245	Adipose_R2_peak_1	Intron (uc007b	1	85083075	85270566	187492	2	381287	uc007bti.1	-63743	ENSMUSG000	A530032D15R	RIKEN cDNA A530032D15Rik gene
chr1	85338346	85338619	274	275	85338491	70	26.08403	4.65257	22.28555	Adipose_R2_peak_1	Intron (uc007b	1	85083075	85270566	187492	2	381287	uc007bti.1	-67780	ENSMUSG000	A530032D15R	RIKEN cDNA A530032D15Rik gene
chr1	85338349	85338617	269	270	85338506	58	21.50455	4.52198	17.97772	Adipose_R1_peak_9	Intron (uc007b	1	85083075	85270566	187492	2	381287	uc007bti.1	-67783	ENSMUSG000	A530032D15R	RIKEN cDNA A530032D15Rik gene
chr1	85339961	85340475	515	516	85340129	49	5.69437	2.01137	2.84761	Adipose_R2_peak_1	Exon (uc007bt	1	85083075	85270566	187492	2	381287	uc007bti.1	-69395	ENSMUSG000	A530032D15R	RIKEN cDNA A530032D15Rik gene
chr1	85341084	85341398	315	316	85341252	64	10.54218	2.47809	7.28371	Adipose_R2_peak_1	Exon (uc007bt	1	85083075	85270566	187492	2	381287	uc007bti.1	-70518	ENSMUSG000	A530032D15R	RIKEN cDNA A530032D15Rik gene
chr1	85342083	85342581	499	500	85342376	89	16.79061	2.76605	13.25378	Adipose_R2_peak_1	Exon (uc007bt	1	85083075	85270566	187492	2	381287	uc007bti.1	-71517	ENSMUSG000	A530032D15R	RIKEN cDNA A530032D15Rik gene
chr1	85342413	85342560	148	149	85342474	51	7.39827	2.25637	4.40193	Adipose_R1_peak_9	Exon (uc007bt	1	85083075	85270566	187492	2	381287	uc007bti.1	-71847	ENSMUSG000	A530032D15R	RIKEN cDNA A530032D15Rik gene
chr1	85342888	85344276	1389	1390	85343004	62	7.7563	2.13409	4.69388	Adipose_R2_peak_1	Distal Intergen	1	85083075	85270566	187492	2	381287	uc007bti.1	-72322	ENSMUSG000	A530032D15R	RIKEN cDNA A530032D15Rik gene
chr1	85344723	85345198	476	477	85344875	57	6.74094	2.06042	3.7602	Adipose_R2_peak_1	Distal Intergen	1	85083075	85270566	187492	2	381287	uc007bti.1	-74157	ENSMUSG000	A530032D15R	RIKEN cDNA A530032D15Rik gene
chr1	85345297	85345587	291	292	85345424	91	30.84557	4.36297	26.95795	Adipose_R2_peak_1	Distal Intergen	1	85083075	85270566	187492	2	381287	uc007bti.1	-74731	ENSMUSG000	A530032D15R	RIKEN cDNA A530032D15Rik gene
chr1	85345879	85346204	326	327	85346123	54	9.69123	2.56239	6.49407	Adipose_R2_peak_1	Distal Intergen	1	85083075	85270566	187492	2	381287	uc007bti.1	-75313	ENSMUSG000	A530032D15R	RIKEN cDNA A530032D15Rik gene
chr1	85346327	85346993	667	668	85346458	127	43.97548	4.5694	39.89233	Adipose_R2_peak_1	Distal Intergen	1	85083075	85270566	187492	2	381287	uc007bti.1	-75761	ENSMUSG000	A530032D15R	RIKEN cDNA A530032D15Rik gene
chr1	85346691	85346904	214	215	85346751	42	7.47242	2.47254	4.47523	Adipose_R1_peak_9	Distal Intergen	1	85083075	85270566	187492	2	381287	uc007bti.1	-76125	ENSMUSG000	A530032D15R	RIKEN cDNA A530032D15Rik gene
chr1	85448294	85448701	408	409	85448362	29	6.74062	2.76653	3.75989	Adipose_R2_peak_1	Distal Intergen	1	85594253	85598779	4527	2	109032	uc029qre.1	150078	ENSMUSG000	Sp110	Sp110 nuclear body protein
chr1	85449322	85449768	447	448	85449432	30	7.24477	2.85875	4.21917	Adipose_R2_peak_1	Distal Intergen	1	85594253	85598779	4527	2	109032	uc029qre.1	149011	ENSMUSG000	Sp110	Sp110 nuclear body protein
chr1	85451903	85452038	136	137	85451965	44	15.55653	4.1498	12.05625	Adipose_R2_peak_1	Distal Intergen	1	85594253	85598779	4527	2	109032	uc029qre.1	146741	ENSMUSG000	Sp110	Sp110 nuclear body protein
chr1	85452471	85452600	130	131	85452590	33	8.80499	3.12713	5.6768	Adipose_R2_peak_1	Distal Intergen	1	85594253	85598779	4527	2	109032	uc029qre.1	146179	ENSMUSG000	Sp110	Sp110 nuclear body protein
chr1	85453538	85454226	689	690	85453749	32	8.15464	3.00597	5.07178	Adipose_R2_peak_1	Distal Intergen	1	85594253	85598779	4527	2	109032	uc029qre.1	144553	ENSMUSG000	Sp110	Sp110 nuclear body protein
chr1	85454866	85455114	249	250	85454967	44	15.55653	4.1498	12.05625	Adipose_R2_peak_1	Exon (uc029qc	1	85594253	85598779	4527	2	109032	uc029qre.1	143665	ENSMUSG000	Sp110	Sp110 nuclear body protein
chr1	85455412	85456039	628	629	85455535	33	8.83596	3.1354	5.68652	Adipose_R2_peak_1	Distal Intergen	1	85594253	85598779	4527	2	109032	uc029qre.1	142740	ENSMUSG000	Sp110	Sp110 nuclear body protein
chr1	85456232	85456613	382	383	85456365	44	14.29495	3.8477	10.84762	Adipose_R2_peak_1	Distal Intergen	1	85594253	85598779	4527	2	109032	uc029qre.1	142166	ENSMUSG000	Sp110	Sp110 nuclear body protein
chr1	85456371	85456585	215	216	85456484	34	14.8941	4.86196	11.54137	Adipose_R1_peak_9	Distal Intergen	1	85594253	85598779	4527	2	109032	uc029qre.1	142194	ENSMUSG000	Sp110	Sp110 nuclear body protein
chr1	85456808	85456967	160	161	85456943	29	5.57962	2.44803	2.73723	Adipose_R2_peak_1	Distal Intergen	1	85594253	85598779	4527	2	109032	uc029qre.1	141812	ENSMUSG000	Sp110	Sp110 nuclear body protein
chr1	85457166	85457373	208	209	85457269	43	11.47383	3.26462	8.1712	Adipose_R2_peak_1	Distal Intergen	1	85594253	85598779	4527	2	109032	uc029qre.1	141406	ENSMUSG000	Sp110	Sp110 nuclear body protein
chr1	85457770	85457876	107	108	85457840	27	5.59171	2.52951	2.74887	Adipose_R2_peak_1	Distal Intergen	1	85594253	85598779	4527	2	109032	uc029qre.1	140903	ENSMUSG000	Sp110	Sp110 nuclear body protein
chr1	85458745	85458999	255	256	85458870	85	22.12982	3.45955	18.42221	Adipose_R2_peak_1	Distal Intergen	1	85594253	85598779	4527	2	109032	uc029qre.1	139780	ENSMUSG000	Sp110	Sp110 nuclear body protein
chr1	85458798	85458991	194	195	85458864	47	16.16915	4.08994	12.78724	Adipose_R1_peak_9	Distal Intergen	1	85594253	85598779	4527	2	109032	uc029qre.1	139788	ENSMUSG000	Sp110	Sp110 nuclear body protein
chr1	85460350	85460612	263	264	85460516	87	35.62605	5.24787	31.65691	Adipose_R2_peak_1	Distal Intergen	1	85594253	85598779	4527	2	109032	uc029qre.1	138167	ENSMUSG000	Sp110	Sp110 nuclear body protein
chr1	85461627	85461759	133	134	85461694	31	7.7623	2.95097	4.69388	Adipose_R2_peak_1	Distal Intergen	1	85594253	85598779	4527	2	109032	uc029qre.1	137020	ENSMUSG000	Sp110	Sp110 nuclear body protein
chr1	85466172	85466392	221	222	85466272	31	16.23981	5.75161	12.83549	Adipose_R1_peak_9	Distal Intergen	1	85594253	85598779	4527	2	109032	uc029qre.1	132387	ENSMUSG000	Sp110	Sp110 nuclear body protein
chr1	85466201	85466364	164	165	85466272	36	10.5378	3.41206	7.27949	Adipose_R2_peak_1	Distal Intergen	1	85594253	85598779	4527	2	109032	uc029qre.1	132415	ENSMUSG000	Sp110	Sp110 nuclear body protein
chr1	85478919	85479190	272	273	85479110	44	8.40763	2.59858	5.2939	Adipose_R2_peak_1	Distal Intergen	1	85594253	85598779	4527	2	109032	uc029qre.1	119589	ENSMUSG000	Sp110	Sp110 nuclear body protein
chr1	85479404	85479515	112	113	85479466	47	8.90528	2.60669	5.75368	Adipose_R2_peak_1	Distal Intergen	1	85594253	85598779	4527	2	109032	uc029qre.1	119264	ENSMUSG000	Sp110	Sp110 nuclear body protein
chr1	85480814	85480967	154	155	85480887	23	9.86443	4.31371	6.70247	Adipose_R1_peak_9	Distal Intergen	1	85594253	85598779	4527	2	109032	uc029qre.1	117812	ENSMUSG000	Sp110	Sp110 nuclear body protein
chr1	85481535	85481676	142	143	85481580	47	10.5313	2.91007	7.27949	Adipose_R2_peak_1	Distal Intergen	1	85594253	85598779	4527	2	109032	uc029qre.1	117103	ENSMUSG000	Sp110	Sp110 nuclear body protein
chr1	85481934	85482067	134	135	85481985	28	5.44941	2.44976	2.61182	Adipose_R2_peak_1	Distal Intergen	1	85594253	85598779	4527	2	109032	uc029qre.1	116712	ENSMUSG000	Sp110	Sp110 nuclear body protein
chr1	85482426	85482600	175	176	85482513	27	5.77405	2.5821	2.88921	Adipose_R2_peak_1	Distal Intergen	1	85594253	85598779	4527	2	109032	uc029qre.1	116179	ENSMUSG000	Sp110	Sp110 nuclear body protein
chr1	85483001	85483251	251	252	85483133	39	12.34187	3.68871	8.98501	Adipose_R2_peak_1	Distal Intergen	1	85594253	85598779	4527	2	109032	uc029qre.1	115528	ENSMUSG000	Sp110	Sp110 nuclear body protein
chr1	85483603	85483753	151	152	85483684	19	7.07687	3.59476	4.08458	Adipose_R1_peak_9	Distal Intergen	1	85594253	85598779	4527	2	109032	uc029qre.1	115026	ENSMUSG000	Sp110	Sp110 nuclear body protein
chr1	85484344	85484500	157	158	85484389	38	11.72962	3.59649	8.4047	Adipose_R2_peak_1	Distal Intergen	1	85594253	85598779	4527	2	109032	uc029qre.1	114279	ENSMUSG000	Sp110	Sp110 nuclear body protein
chr1	85485118	85485328	211	212	85485233	65	31.39315	6.08637	27.49408	Adipose_R2_peak_1	Distal Intergen	1	85594253	85598779	4527	2	109032	uc029qre.1	113451	ENSMUSG000	Sp110	Sp110 nuclear body protein

chr1	85489443	85489593	151	152	85489488	38	11.72962	3.59649	8.4047	Adipose_R2_peak_1	Distal Interger	1	85594253	85598779	4527	2	109032	uc029qre.1	109186	ENSMUSG000	Sp110	Sp110 nuclear body protein
chr1	85490334	85490494	161	162	85490408	37	11.12817	3.50427	7.83613	Adipose_R2_peak_1	Distal Interger	1	85594253	85598779	4527	2	109032	uc029qre.1	108285	ENSMUSG000	Sp110	Sp110 nuclear body protein
chr1	85490343	85490486	144	145	85490413	24	10.60668	4.49345	7.40961	Adipose_R1_peak_9	Distal Interger	1	85594253	85598779	4527	2	109032	uc029qre.1	108293	ENSMUSG000	Sp110	Sp110 nuclear body protein
chr1	85492310	85492527	218	219	85492408	48	16.68162	4.14117	13.14689	Adipose_R2_peak_1	Distal Interger	1	85594253	85598779	4527	2	109032	uc029qre.1	106252	ENSMUSG000	Sp110	Sp110 nuclear body protein
chr1	85492743	85492900	158	159	85492875	28	6.25023	2.67431	3.31626	Adipose_R2_peak_1	Distal Interger	1	85594253	85598779	4527	2	109032	uc029qre.1	105879	ENSMUSG000	Sp110	Sp110 nuclear body protein
chr1	85495034	85495190	157	158	85495096	52	21.18119	4.88754	17.49655	Adipose_R2_peak_1	Distal Interger	1	85594253	85598779	4527	2	109032	uc029qre.1	103589	ENSMUSG000	Sp110	Sp110 nuclear body protein
chr1	85495354	85495620	267	268	85495455	26	10.16927	4.08992	7.00478	Adipose_R1_peak_9	Distal Interger	1	85594253	85598779	4527	2	109032	uc029qre.1	103159	ENSMUSG000	Sp110	Sp110 nuclear body protein
chr1	85496005	85496273	269	270	85496127	79	37.49879	6.12254	33.50252	Adipose_R2_peak_1	Distal Interger	1	85594253	85598779	4527	2	109032	uc029qre.1	102506	ENSMUSG000	Sp110	Sp110 nuclear body protein
chr1	85496986	85497324	339	340	85497066	68	31.46892	5.83144	27.56914	Adipose_R2_peak_1	Distal Interger	1	85594253	85598779	4527	2	109032	uc029qre.1	101455	ENSMUSG000	Sp110	Sp110 nuclear body protein
chr1	85497512	85497655	144	145	85497560	20	5.25588	2.77934	2.4422	Adipose_R1_peak_9	Distal Interger	1	85594253	85598779	4527	2	109032	uc029qre.1	101124	ENSMUSG000	Sp110	Sp110 nuclear body protein
chr1	85499051	85499211	161	162	85499162	69	16.79647	3.22507	13.25943	Adipose_R2_peak_1	Distal Interger	1	85594253	85598779	4527	2	109032	uc029qre.1	99568	ENSMUSG000	Sp110	Sp110 nuclear body protein
chr1	85500008	85500247	240	241	85500130	44	13.37007	3.63464	9.97125	Adipose_R2_peak_1	Distal Interger	1	85594253	85598779	4527	2	109032	uc029qre.1	98532	ENSMUSG000	Sp110	Sp110 nuclear body protein
chr1	85501268	85501482	215	216	85501320	36	7.59551	2.6905	4.55644	Adipose_R2_peak_1	Distal Interger	1	85594253	85598779	4527	2	109032	uc029qre.1	97297	ENSMUSG000	Sp110	Sp110 nuclear body protein
chr1	85502952	85503352	401	402	85503085	114	37.88499	4.36151	33.88536	Adipose_R2_peak_1	Distal Interger	1	85594253	85598779	4527	2	109032	uc029qre.1	95427	ENSMUSG000	Sp110	Sp110 nuclear body protein
chr1	85503000	85503188	189	190	85503095	59	8.42704	2.27221	5.34896	Adipose_R1_peak_9	Distal Interger	1	85594253	85598779	4527	2	109032	uc029qre.1	95591	ENSMUSG000	Sp110	Sp110 nuclear body protein
chr1	85503700	85503889	190	191	85503751	36	9.72196	3.20382	6.52369	Adipose_R2_peak_1	Distal Interger	1	85594253	85598779	4527	2	109032	uc029qre.1	94890	ENSMUSG000	Sp110	Sp110 nuclear body protein
chr1	85504902	85504998	97	98	85504974	27	5.77405	2.5821	2.88921	Adipose_R2_peak_1	Distal Interger	1	85594253	85598779	4527	2	109032	uc029qre.1	93781	ENSMUSG000	Sp110	Sp110 nuclear body protein
chr1	85505730	85505886	157	158	85505781	33	8.51488	3.0502	5.39709	Adipose_R2_peak_1	Distal Interger	1	85594253	85598779	4527	2	109032	uc029qre.1	92893	ENSMUSG000	Sp110	Sp110 nuclear body protein
chr1	85506453	85506746	294	295	85506596	100	30.31655	3.97525	26.44047	Adipose_R2_peak_1	Distal Interger	1	85594253	85598779	4527	2	109032	uc029qre.1	92033	ENSMUSG000	Sp110	Sp110 nuclear body protein
chr1	85507002	85507211	210	211	85507095	41	10.10034	3.05408	6.87239	Adipose_R2_peak_1	Distal Interger	1	85594253	85598779	4527	2	109032	uc029qre.1	91568	ENSMUSG000	Sp110	Sp110 nuclear body protein
chr1	85507940	85508091	152	153	85507999	32	8.29282	3.04319	5.18352	Adipose_R2_peak_1	Distal Interger	1	85594253	85598779	4527	2	109032	uc029qre.1	90688	ENSMUSG000	Sp110	Sp110 nuclear body protein
chr1	85508205	85508420	216	217	85508307	55	23.42763	5.16419	19.68558	Adipose_R2_peak_1	Distal Interger	1	85594253	85598779	4527	2	109032	uc029qre.1	90359	ENSMUSG000	Sp110	Sp110 nuclear body protein
chr1	85508845	85509029	185	186	85508932	44	15.55653	4.1498	12.05625	Adipose_R2_peak_1	Distal Interger	1	85594253	85598779	4527	2	109032	uc029qre.1	89750	ENSMUSG000	Sp110	Sp110 nuclear body protein
chr1	85510177	85510423	247	248	85510297	46	16.86262	4.3228	13.32444	Adipose_R2_peak_1	Distal Interger	1	85594253	85598779	4527	2	109032	uc029qre.1	88356	ENSMUSG000	Sp110	Sp110 nuclear body protein
chr1	85513467	85513567	101	102	85513503	29	6.74062	2.76653	3.75989	Adipose_R2_peak_1	Distal Interger	1	85594253	85598779	4527	2	109032	uc029qre.1	85212	ENSMUSG000	Sp110	Sp110 nuclear body protein
chr1	85525022	85525348	327	328	85525243	36	10.5378	3.41206	7.27949	Adipose_R2_peak_1	Distal Interger	1	85594253	85598779	4527	2	109032	uc029qre.1	73431	ENSMUSG000	Sp110	Sp110 nuclear body protein
chr1	85525873	85526061	189	190	85525940	54	21.81344	4.87419	18.11944	Adipose_R2_peak_1	Distal Interger	1	85594253	85598779	4527	2	109032	uc029qre.1	72718	ENSMUSG000	Sp110	Sp110 nuclear body protein
chr1	85525891	85526164	274	275	85525967	27	12.5942	4.89795	9.32889	Adipose_R1_peak_9	Distal Interger	1	85594253	85598779	4527	2	109032	uc029qre.1	72615	ENSMUSG000	Sp110	Sp110 nuclear body protein
chr1	85526821	85527042	222	223	85526931	40	12.96465	3.78093	9.57654	Adipose_R2_peak_1	Distal Interger	1	85594253	85598779	4527	2	109032	uc029qre.1	71737	ENSMUSG000	Sp110	Sp110 nuclear body protein
chr1	85527649	85527967	319	320	85527754	44	15.55653	4.1498	12.05625	Adipose_R2_peak_1	Distal Interger	1	85594253	85598779	4527	2	109032	uc029qre.1	70812	ENSMUSG000	Sp110	Sp110 nuclear body protein
chr1	85527701	85527881	181	182	85527782	24	7.81045	3.41972	4.7694	Adipose_R1_peak_9	Distal Interger	1	85594253	85598779	4527	2	109032	uc029qre.1	70898	ENSMUSG000	Sp110	Sp110 nuclear body protein
chr1	85528079	85528220	142	143	85528141	30	7.24477	2.85875	4.21917	Adipose_R2_peak_1	Distal Interger	1	85594253	85598779	4527	2	109032	uc029qre.1	70559	ENSMUSG000	Sp110	Sp110 nuclear body protein
chr1	85528825	85529113	289	290	85528946	59	19.33831	4.04067	15.71162	Adipose_R2_peak_1	Distal Interger	1	85594253	85598779	4527	2	109032	uc029qre.1	69666	ENSMUSG000	Sp110	Sp110 nuclear body protein
chr1	85528860	85529033	174	175	85528968	33	8.84604	3.13809	5.75797	Adipose_R1_peak_9	Distal Interger	1	85594253	85598779	4527	2	109032	uc029qre.1	69746	ENSMUSG000	Sp110	Sp110 nuclear body protein
chr1	85529453	85529577	125	126	85529528	36	10.5378	3.41206	7.27949	Adipose_R2_peak_1	Distal Interger	1	85594253	85598779	4527	2	109032	uc029qre.1	69202	ENSMUSG000	Sp110	Sp110 nuclear body protein
chr1	85530091	85530340	250	251	85530223	56	24.19188	5.25641	20.43163	Adipose_R2_peak_1	Distal Interger	1	85594253	85598779	4527	2	109032	uc029qre.1	68439	ENSMUSG000	Sp110	Sp110 nuclear body protein
chr1	85533371	85533500	130	131	85533486	29	6.74062	2.76653	3.75989	Adipose_R2_peak_1	Distal Interger	1	85594253	85598779	4527	2	109032	uc029qre.1	65279	ENSMUSG000	Sp110	Sp110 nuclear body protein
chr1	85536658	85536915	258	259	85536762	75	24.21855	4.09676	20.45806	Adipose_R2_peak_1	Intron (uc007b	1	85594253	85598779	4527	2	109032	uc029qre.1	61864	ENSMUSG000	Sp110	Sp110 nuclear body protein
chr1	85537881	85538070	190	191	85538013	32	8.29282	3.04319	5.18352	Adipose_R2_peak_1	Intron (uc007b	1	85594253	85598779	4527	2	109032	uc029qre.1	60709	ENSMUSG000	Sp110	Sp110 nuclear body protein
chr1	85539288	85539488	201	202	85539381	45	16.22858	4.24202	12.70278	Adipose_R2_peak_1	Intron (uc007b	1	85594253	85598779	4527	2	109032	uc029qre.1	59291	ENSMUSG000	Sp110	Sp110 nuclear body protein
chr1	85540294	85540420	127	128	85540367	31	7.7623	2.95097	4.69388	Adipose_R2_peak_1	Intron (uc007b	1	85594253	85598779	4527	2	109032	uc029qre.1	58359	ENSMUSG000	Sp110	Sp110 nuclear body protein
chr1	85546976	85547131	156	157	85547090	22	9.13959	4.13397	6.01526	Adipose_R1_peak_9	Intron (uc007b	1	85594253	85598779	4527	2	109032	uc029qre.1	51648	ENSMUSG000	Sp110	Sp110 nuclear body protein
chr1	85549644	85549769	126	127	85549701	31	7.7623	2.95097	4.69388	Adipose_R2_peak_1	5' UTR	1	85594253	85598779	4527	2	109032	uc029qre.1	49010	ENSMUSG000	Sp110	Sp110 nuclear body protein
chr1	85549659	85549793	135	136	85549722	24	10.3979	4.40806	7.23136	Adipose_R1_peak_9	5' UTR	1	85594253	85598779	4527	2	109032	uc029qre.1	48986	ENSMUSG000	Sp110	Sp110 nuclear body protein
chr1	85550453	85550739	287	288	85550569	80	24.47353	3.93051	20.70972	Adipose_R2_peak_1	3' UTR	1	85594253	85598779	4527	2	109032	uc029qre.1	48040	ENSMUSG000	Sp110	Sp110 nuclear body protein
chr1	85550532	85550763	232	233	85550601	44	11.9645	3.3237	8.72945	Adipose_R1_peak_9	3' UTR	1	85594253	85598779	4527	2	109032	uc029qre.1	48016	ENSMUSG000	Sp110	Sp110 nuclear body protein
chr1	85551703	85551935	233	234	85551810	125	48.57777	5.12518	44.4403	Adipose_R2_peak_1	3' UTR	1	85594253	85598779	4527	2	109032	uc029qre.1	46844	ENSMUSG000	Sp110	Sp110 nuclear body protein
chr1	85553318	85553558	241	242	85553417	72	27.49929	4.78363	23.67126	Adipose_R2_peak_1	Distal Interger	1	85594253	85598779	4527	2	109032	uc029qre.1	45221	ENSMUSG000	Sp110	Sp110 nuclear body protein
chr1	85555341	85555570	230	231	85555455	58	16.35434	3.54747	12.82603	Adipose_R2_peak_1	Distal Interger	1	85594253	85598779	4527	2	109032	uc029qre.1	43209	ENSMUSG000	Sp110	Sp110 nuclear body protein
chr1	85563157	85563334	178	179	85563279	61	28.12311	5.7175	24.28203	Adipose_R2_peak_1	Distal Interger	1	85594253	85598779	4527	2	109032	uc029qre.1	35445	ENSMUSG000	Sp110	Sp110 nuclear body protein
chr1	85563173	85563323	151	152	85563265	29	14.29841	5.28967	10.97253	Adipose_R1_peak_9	Distal Interger	1	85594253	85598779	4527	2	109032	uc029qre.1	35456	ENSMUSG000	Sp110	Sp110 nuclear body protein
chr1	85565486	85565667	182	183	85565574	39	12.34187	3.68871	8.98501	Adipose_R2_peak_1	Distal Interger	1	85594253	85598779	4527	2	109032	uc029qre.1	33112	ENSMUSG000	Sp110	Sp110 nuclear body protein
chr1	85565502	85565648	147	148	85565597	21	8.43286	3.95423	5.34896	Adipose_R1_peak_9	Distal Interger	1	85594253	85598779	4527	2	109032	uc029qre.1	33131	ENSMUSG000	Sp110	Sp110 nuclear body protein
chr1	85572069	85572183	115	116	85572083	25	4.86612	2.39766	2.09018	Adipose_R2_peak_1	Distal Interger	1	85594253	85598779	4527	2	109032	uc029qre.1	26596	ENSMUSG000	Sp110	Sp110 nuclear body protein
chr1	85572654	85572796	143	144	85572746	33	6.3281	2.49725	3.39123	Adipose_R2_peak_1	Distal Interger	1	85594253	85598779	4527	2	109032	uc029qre.1	25983	ENSMUSG000	Sp110	Sp110 nuclear body protein
chr1	85573645	85573798	154	155	85573740	41	13.59774	3.87315	10.1803	Adipose_R2_peak_1	Distal Interger	1	85594253	85598779	4527	2	109032	uc029qre.1	24981	ENSMUSG000	Sp110	Sp110 nuclear body protein
chr1	85575211	85575311	101	102	85575269	30	7.24477	2.85875	4.21917	Adipose_R2_peak_1	Downstream (	1	85594253	85598779	4527	2	109032	uc029qre.1	23468	ENSMUSG000	Sp110	Sp110 nuclear body protein
chr1	85578281	85578447	167	168	85578333	35	9.95877	3.31984	6.73562	Adipose_R2_peak_1	Intron (uc007b	1	85594253	85598779	4527	2	109032	uc029qre.1	20332	ENSMUSG000	Sp110	Sp110 nuclear body protein
chr1	85578698	85579122	425	426	85578840	45	13.27089	3.55779	9.87466	Adipose_R2_peak_1	Exon (uc007bt	1	85594253	85598779	4527	2	109032	uc029qre.1	19657	ENSMUSG000	Sp110	Sp110 nuclear body protein
chr1	85578893	85579046	154	155	85578897	29	6.04569	2.57419	3.16225	Adipose_R1_peak_9	Intron (uc007b	1	85594253	85598779	4527	2	109032	uc029qre.1	19733	ENSMUSG000	Sp110	Sp110 nuclear body protein
chr1	85580186	85580489	304	305	85580335	56	20.54981	4.45583	16.8844	Adipose_R2_peak_1	Intron (uc007b	1	85594253	85598779	4527	2	109032	uc029qre.1	18290	ENSMUSG000	Sp110	Sp110 nuclear body protein
chr1	85582691	85583229	539	540	85582805	36	10.5378	3.41206	7.27949	Adipose_R2_peak_1	Intron (uc007b	1	85594253	85598779	4527	2	109032	uc029qre.1	15550	ENSMUSG000	Sp110	Sp110 nuclear body protein
chr1	85583039	85583197	159	160	85583098	27	7.58971	3.12862	4.59104	Adipose_R1_peak_9	Intron (uc007b	1	85594253	85598779	4527	2	109032	uc029qre.1	15582	ENSMUSG000	Sp110	Sp110 nuclear body protein
chr1	85583897	85584332	436	437	85583976	34	9.39139	3.22762	6.20474	Adipose_R2_peak_1	Intron (uc007b	1	85594253	85598779	4527	2	109032	uc029qre.1	14447	ENSMUSG000	Sp110	Sp110 nuclear body protein
chr1	85584650	85584874	225	226	85584778	51	14.60349	3.53455	11.14904	Adipose_R2_peak_1	Intron (uc007b	1	85594253	85598779	4527	2	109032	uc029qre.1	13905	ENSMUSG000	Sp110	Sp110 nuclear body protein
chr1	85585711	85586158	448	449	85585894	79	22.26244	3.66265	18.55287	Adipose_R2_peak_1	Intron (uc007b	1	85594253	85598779	4527	2	109032	uc029qre.1	12621	ENSMUSG000	Sp110	Sp110 nuclear body protein
chr1	85585725	85585891	167	168	85585853	41	7.33805	2.47332	4.3424	Adipose_R1_peak_9	Intron (uc007b	1	85594253	85598779	4527	2	109032	uc029qre.1	12888	ENSMUSG000	Sp110	Sp110 nuclear body protein
chr1	85586508	85586778	271	272	85586651	43	12.43054	3.47685	9.07089	Adipose_R2_peak_1	Intron (uc007b	1	85594253	85598779	4527	2	109032	uc029qre.1	12001	ENSMUSG000	Sp110	Sp110 nuclear body protein
chr1	85588643	85588789	147	148	85588725	39	8.20839	2.71889	5.12338	Adipose_R2_peak_1	Intron (uc007b	1	85594253	85598779	4527	2	109032	uc029qre.1	9990	ENSMUSG000	Sp110	Sp110 nuclear body protein
chr1	85589026	85589193	168	169	85589115	39	6.01528	2.25625	3.12117	Adipose_R2_peak_1	Exon (uc007bt	1	85594253	85598779	4527	2	109032	uc029qre.1	9586	ENSMUSG000	Sp110	Sp110 nuclear body protein
chr1	85589536	85589661	126	127	85589591	35	9.90854	3.3067	6.70445	Adipose_R2_peak_1	Intron (uc007b	1	85594253	85598779	4527	2	109032	uc029qre.1	9118	ENSMUSG000	Sp110	Sp110 nuclear body protein
chr1	85590325	85590501	177	178	85590436	63	28.53901	5.6326	24.69371	Adipose_R2_peak_1	Intron (uc007b	1	85594253	85598779	4527	2	109032	uc029qre.1	8278	ENSMUSG000	Sp110	Sp110 nuclear body protein
chr1	85590969	85591231	263	264	85591103	41	13.0575	3.73969	9.66681	Adipose_R2_peak_1	Intron (uc007b	1	85594253	85598779	4527	2	109032	uc029qre.1	7548	ENSMUSG000	Sp110	Sp110 nuclear body protein
chr1	85591362	85591504	143	144	85591450	35	9.39596	3.17416	6.20924	Adipose_R2_peak_1	Intron (uc007b	1	85594253	85598779	4527	2	109032	uc029qre.1	7275	ENSMUSG000	Sp110	Sp110 nuclear body protein
chr1	85591629	85591788	160	161	85591727	39	10.40552	3.21618	7.16778	Adipose_R2_peak_1	Exon (uc007bt	1	85594253	85598779	4527	2	109032	uc029qre.1	6991	ENSMUSG000	Sp110	Sp110 nuclear body protein
chr1	85591891	85592150	260	261	85591996	70	29.16198	5.21485	25.30336	Adipose_R2_peak_1	Intron (uc007b	1	85594253	85598779	4527	2	109032	uc029qre.1	6629	ENSMUSG000	Sp110	Sp110 nuclear body protein
chr1	85593492	85593647	156	157	85593519	33	7.9432	2.90118	4.86875	Adipose_R2_peak_1	Intron (uc007b	1	85594253	85598779	4527	2	109032	uc029qre.1	5132	ENSMUSG000	Sp110	Sp110 nuclear body protein
chr1	85594332	85594894	563	564	85594474	57	21.47979	4.58312	17.79067	Adipose_R2_peak_1	Exon (uc007bt	1	85594253	85598779	4527	2	109032	uc029qre.1	3885	ENSMUSG000	Sp110	Sp110 nuclear body protein
chr1	85595090	85595466	377	378	85595193	39	5.43845	2.13878	2.62143	Adipose_R1_peak_9	Exon (uc029qr	1	85594253	85598779	4527	2	109032	uc029qre.1	3313	ENSMUSG000	Sp110	Sp110 nuclear body protein
chr1	85595108	85595570	463	464	85595414	93	23.96667	3.45719	20.21787	Adipose_R2_peak_1	Exon (uc029qr	1	85594253	85598779	4527	2	109032	uc029qre.1	3209	ENSMUSG000	Sp110	Sp110 nuclear body protein
chr1	85596051	85596240	190	191	85596157	48	12.28036	3.21093	8.93934	Adipose_R2_peak_1	Promoter (2-3	1	85594253	85598779	4527	2	109032	uc029qre.1	2539	ENSMUSG000	Sp110	Sp110 nuclear body protein
chr1	85596473	85596658	186	187	85596569	34	10.94571	3.65187	7.74602	Adipose_R1_peak_9	Promoter (2-3	1	85594253	85598779	4527	2	109032	uc029qre.1	2121	ENSMUSG000	Sp110	Sp110 nuclear body protein
chr1	85596492	85596642	151	152	85596568	48	14.62512	3.68979	11.17022	Adipose_R2_peak_1	Promoter (2-3	1	85594253	85598779	4527	2	109032	uc029qre.1	2137	ENSMUSG000	Sp110	Sp110 nuclear body protein
chr1	85597746	85597957	212	213	85597879	59	22.6682	4.69351	18.94758	Adipose_R2_peak_1	Promoter (<=	1	85594253	85598779	4527	2	109032	uc029qre.1	822	ENSMUSG000	Sp110	Sp110 nuclear body protein
chr1	85598388	85598855	468	469	85598514	65	21.59911	4.13897	17.90824	Adipose_R2_peak_1	Promoter (<=	1	85576899	85598810	21912	2	109032	uc007btz.2	0	ENSMUSG000	Sp110	Sp110 nuclear body protein
chr1	85599160	85599496	337	338	85599303	78	25.14488	4.1067	21.36509	Adipose_R2_peak_1	Promoter (<=	1	85576899	85598810	21912	2	109032	uc007btz.2	-350	ENSMUSG000	Sp110	Sp110 nuclear body protein
chr1	85599956	85600579	624	625	85600057	51	11.02329	2.86824	7.75021	Adipose_R2_peak_1	Promoter (<=	1	85600703	85645036	44334	1	434484	uc007buc.1	-124	ENSMUSG000	Sp140	Sp140 nuclear body protein
chr1	85600265	85600470	206	207	85600356	47	6.47335	2.17564	3.53321	Adipose_R1_peak_9	Promoter (<=	1	85600703	85645036	44334	1	434484	uc007buc.1	-233	ENSMUSG000	Sp140	Sp140 nuclear body protein
chr1	85600776	85600904	129	130	85600853	43	6.68246	2.29505	3.73188	Adipose_R2_peak_1	Promoter (<=	1	85600703	85645036	44334	1	434484	uc007buc.1	73	ENSMUSG000	Sp140	Sp140 nuclear body protein
chr1	85601170	85601534	365	366	85601285	62	20.87721	4.16577	17.2064	Adipose_R2_peak_1	Promoter (<=	1	85600703	85645036	44334	1	434484	uc007buc.1	467	ENSMUSG000	Sp140	Sp140 nuclear body protein
chr1	85601257	85601496	240	241	85601370	29	5.07539	2.31385	2.32922	Adipose_R1_peak_9	Promoter (<=	1	85600703	85645036	44334	1	434484	uc007buc.1	554	ENSMUSG000	Sp140	Sp140 nuclear body protein
chr1	85603724	85604036	313	314	85603842	54	16.57901	3.77364	13.04635	Adipose_R2_peak_1	Promoter (<=	1	85576899	85603891	26993	2	109032	uc007bub.2	0	ENSMUSG000	Sp110	Sp110 nuclear body protein
chr1	85604283	85604919	637	638	85604490	112	33.81807	3.99481	29.88355	Adipose_R2_peak_1	Promoter (<=	1	85576899	85603891	26993	2	109032	uc007bub.2	-392	ENSMUSG000	Sp110	Sp110 nuclear body protein
chr1	85604462	85604848	387	388	85604532	56	9.2933	2.45586	6.16763	Adipose_R1_peak_9	Promoter (<=	1	85576899	85603891	26993	2	109032	uc007bub.2	-571	ENSMUSG000	Sp110	Sp110 nuclear body protein

chr1	85605132	85605575	444	445	85605265	92	24.66857	3.5642	20.90225	Adipose_R2_peak_1	Promoter (1-2	1	85576899	85603891	26993	2	109032	uc007bub.2	-1241	ENSMUSG000	Sp110	Sp110 nuclear body protein
chr1	85605187	85605326	140	141	85605285	40	6.74353	2.37997	3.79971	Adipose_R1_peak_9	Promoter (1-2	1	85576899	85603891	26993	2	109032	uc007bub.2	-1296	ENSMUSG000	Sp110	Sp110 nuclear body protein
chr1	85606460	85606711	252	253	85606572	58	23.18764	4.87336	19.45721	Adipose_R2_peak_1	Promoter (2-3	1	85576899	85603891	26993	2	109032	uc007bub.2	-2569	ENSMUSG000	Sp110	Sp110 nuclear body protein
chr1	85607284	85607630	347	348	85607346	36	9.59771	3.17269	6.40381	Adipose_R2_peak_1	Intron (uc007b	1	85576899	85603891	26993	2	109032	uc007bub.2	-3393	ENSMUSG000	Sp110	Sp110 nuclear body protein
chr1	85607891	85608062	172	173	85607974	60	22.67682	4.62836	18.95329	Adipose_R2_peak_1	Intron (uc007b	1	85576899	85603891	26993	2	109032	uc007bub.2	-4000	ENSMUSG000	Sp110	Sp110 nuclear body protein
chr1	85608203	85608495	293	294	85608365	65	21.59911	4.13897	17.90824	Adipose_R2_peak_1	Intron (uc007b	1	85576899	85603891	26993	2	109032	uc007bub.2	-4312	ENSMUSG000	Sp110	Sp110 nuclear body protein
chr1	85608292	85608495	204	205	85608384	28	6.62473	2.78182	3.68265	Adipose_R1_peak_1	Intron (uc007b	1	85576899	85603891	26993	2	109032	uc007bub.2	-4401	ENSMUSG000	Sp110	Sp110 nuclear body protein
chr1	85608678	85609118	441	442	85608747	39	9.15254	2.92792	5.99214	Adipose_R2_peak_1	Intron (uc007b	1	85576899	85603891	26993	2	109032	uc007bub.2	-4787	ENSMUSG000	Sp110	Sp110 nuclear body protein
chr1	85609395	85609562	168	169	85609448	53	19.73004	4.4827	16.08712	Adipose_R2_peak_1	Intron (uc007b	1	85576899	85603891	26993	2	109032	uc007bub.2	-5504	ENSMUSG000	Sp110	Sp110 nuclear body protein
chr1	85610271	85610386	116	117	85610316	31	7.7623	2.95097	4.69388	Adipose_R2_peak_1	Intron (uc007b	1	85576899	85603891	26993	2	109032	uc007bub.2	-6380	ENSMUSG000	Sp110	Sp110 nuclear body protein
chr1	85610661	85611035	375	376	85610808	84	29.08609	4.41861	25.22825	Adipose_R2_peak_1	Exon (uc007bu	1	85576899	85603891	26993	2	109032	uc007bub.2	-6770	ENSMUSG000	Sp110	Sp110 nuclear body protein
chr1	85610735	85611001	267	268	85610788	35	7.72193	2.75917	4.70467	Adipose_R1_peak_1	Exon (uc007bu	1	85576899	85603891	26993	2	109032	uc007bub.2	-6844	ENSMUSG000	Sp110	Sp110 nuclear body protein
chr1	85611196	85611449	254	255	85611312	58	25.74274	5.44085	21.94821	Adipose_R2_peak_1	Intron (uc007b	1	85576899	85603891	26993	2	109032	uc007bub.2	-7305	ENSMUSG000	Sp110	Sp110 nuclear body protein
chr1	85611209	85611344	136	137	85611265	21	5.61957	2.84963	2.79916	Adipose_R1_peak_1	Intron (uc007b	1	85576899	85603891	26993	2	109032	uc007bub.2	-7318	ENSMUSG000	Sp110	Sp110 nuclear body protein
chr1	85611980	85612252	273	274	85612122	24	9.42183	4.02018	6.29496	Adipose_R1_peak_1	Intron (uc007b	1	85576899	85603891	26993	2	109032	uc007bub.2	-8089	ENSMUSG000	Sp110	Sp110 nuclear body protein
chr1	85614916	85615146	231	232	85615024	46	16.90986	4.33423	13.35898	Adipose_R2_peak_1	Intron (uc007b	1	85576899	85603891	26993	2	109032	uc007bub.2	-11025	ENSMUSG000	Sp110	Sp110 nuclear body protein
chr1	85615447	85615610	164	165	85615514	39	12.34187	3.68871	8.98501	Adipose_R2_peak_1	Intron (uc007b	1	85576899	85603891	26993	2	109032	uc007bub.2	-11556	ENSMUSG000	Sp110	Sp110 nuclear body protein
chr1	85615871	85616550	680	681	85615921	39	8.81214	2.85178	5.68359	Adipose_R2_peak_1	Intron (uc007b	1	85576899	85603891	26993	2	109032	uc007bub.2	-11980	ENSMUSG000	Sp110	Sp110 nuclear body protein
chr1	85616759	85616962	204	205	85616861	50	14.35315	3.53243	10.90435	Adipose_R2_peak_1	Intron (uc007b	1	85576899	85603891	26993	2	109032	uc007bub.2	-12868	ENSMUSG000	Sp110	Sp110 nuclear body protein
chr1	85617282	85617988	707	708	85617367	56	14.48287	3.30055	11.03118	Adipose_R2_peak_1	Intron (uc007b	1	85576899	85603891	26993	2	109032	uc007bub.2	-13391	ENSMUSG000	Sp110	Sp110 nuclear body protein
chr1	85617589	85617946	358	359	85617678	50	11.43239	2.9746	8.20102	Adipose_R1_peak_1	Intron (uc007b	1	85576899	85603891	26993	2	109032	uc007bub.2	-13698	ENSMUSG000	Sp110	Sp110 nuclear body protein
chr1	85619504	85619626	123	124	85619579	39	9.73917	3.06127	6.54041	Adipose_R2_peak_1	Intron (uc007b	1	85576899	85603891	26993	2	109032	uc007bub.2	-15613	ENSMUSG000	Sp110	Sp110 nuclear body protein
chr1	85620863	85621477	615	616	85620941	41	11.2327	3.30804	7.93736	Adipose_R2_peak_1	Intron (uc007b	1	85576899	85603891	26993	2	109032	uc007bub.2	-16972	ENSMUSG000	Sp110	Sp110 nuclear body protein
chr1	85620880	85621027	148	149	85620956	30	8.40916	3.18916	5.34896	Adipose_R1_peak_1	Intron (uc007b	1	85576899	85603891	26993	2	109032	uc007bub.2	-16989	ENSMUSG000	Sp110	Sp110 nuclear body protein
chr1	85621605	85621706	102	103	85621678	43	6.55181	2.27037	3.6063	Adipose_R2_peak_1	Exon (uc007bu	1	85576899	85603891	26993	2	109032	uc007bub.2	-17714	ENSMUSG000	Sp110	Sp110 nuclear body protein
chr1	85621907	85622272	366	367	85622072	170	70.81177	5.63548	66.46535	Adipose_R2_peak_1	Intron (uc007b	1	85576899	85603891	26993	2	109032	uc007bub.2	-18016	ENSMUSG000	Sp110	Sp110 nuclear body protein
chr1	85621946	85622134	189	190	85622082	71	11.52414	2.48031	8.29215	Adipose_R1_peak_1	Intron (uc007b	1	85576899	85603891	26993	2	109032	uc007bub.2	-18055	ENSMUSG000	Sp110	Sp110 nuclear body protein
chr1	85623855	85624071	217	218	85623981	59	25.34163	5.26487	21.55944	Adipose_R2_peak_1	Intron (uc007b	1	85576899	85603891	26993	2	109032	uc007bub.2	-19964	ENSMUSG000	Sp110	Sp110 nuclear body protein
chr1	85624926	85625953	1028	1029	85625050	73	12.11487	2.51841	8.77876	Adipose_R2_peak_1	Intron (uc007b	1	85576899	85603891	26993	2	109032	uc007bub.2	-21035	ENSMUSG000	Sp110	Sp110 nuclear body protein
chr1	85626641	85626930	290	291	85626778	57	24.89964	5.33385	21.13078	Adipose_R2_peak_1	Intron (uc007b	1	85576899	85603891	26993	2	109032	uc007bub.2	-22750	ENSMUSG000	Sp110	Sp110 nuclear body protein
chr1	85626748	85626980	233	234	85626829	34	13.159	4.30552	9.86435	Adipose_R1_peak_1	Intron (uc007b	1	85576899	85603891	26993	2	109032	uc007bub.2	-22857	ENSMUSG000	Sp110	Sp110 nuclear body protein
chr1	85627223	85627417	195	196	85627305	46	16.90986	4.33423	13.35898	Adipose_R2_peak_1	Intron (uc007b	1	85649988	85683118	33131	1	20684	uc007bud.2	-22571	ENSMUSG000	Sp100	nuclear antigen Sp100
chr1	85627473	85627643	171	172	85627537	26	9.48464	3.84128	6.35734	Adipose_R1_peak_1	Intron (uc007b	1	85649988	85683118	33131	1	20684	uc007bud.2	-22345	ENSMUSG000	Sp100	nuclear antigen Sp100
chr1	85630151	85630295	145	146	85630234	30	7.24477	2.85875	4.21917	Adipose_R2_peak_1	Intron (uc007b	1	85649988	85683118	33131	1	20684	uc007bud.2	-19693	ENSMUSG000	Sp100	nuclear antigen Sp100
chr1	85630792	85631039	248	249	85630896	39	10.77605	3.30397	7.51045	Adipose_R2_peak_1	Intron (uc007b	1	85649988	85683118	33131	1	20684	uc007bud.2	-18949	ENSMUSG000	Sp100	nuclear antigen Sp100
chr1	85631314	85631487	174	175	85631412	35	9.95877	3.31984	6.73562	Adipose_R2_peak_1	Intron (uc007b	1	85649988	85683118	33131	1	20684	uc007bud.2	-18501	ENSMUSG000	Sp100	nuclear antigen Sp100
chr1	85631749	85631867	119	120	85631772	25	4.86612	2.39766	2.09018	Adipose_R2_peak_1	Intron (uc007b	1	85649988	85683118	33131	1	20684	uc007bud.2	-18121	ENSMUSG000	Sp100	nuclear antigen Sp100
chr1	85632574	85632955	382	383	85632712	42	14.2409	3.96536	10.79479	Adipose_R2_peak_1	Exon (uc007bu	1	85649988	85683118	33131	1	20684	uc007bud.2	-17033	ENSMUSG000	Sp100	nuclear antigen Sp100
chr1	85634154	85634256	103	104	85634197	27	5.77405	2.5821	2.88921	Adipose_R2_peak_1	Intron (uc007b	1	85649988	85683118	33131	1	20684	uc007bud.2	-15732	ENSMUSG000	Sp100	nuclear antigen Sp100
chr1	85634464	85634669	206	207	85634546	37	8.96473	2.97055	5.8106	Adipose_R2_peak_1	Intron (uc007b	1	85649988	85683118	33131	1	20684	uc007bud.2	-15319	ENSMUSG000	Sp100	nuclear antigen Sp100
chr1	85634988	85635134	147	148	85635033	26	5.31252	2.48988	2.47999	Adipose_R2_peak_1	Intron (uc007b	1	85649988	85683118	33131	1	20684	uc007bud.2	-14854	ENSMUSG000	Sp100	nuclear antigen Sp100
chr1	85636845	85637095	251	252	85636901	28	6.10844	2.63404	3.21082	Adipose_R2_peak_1	Intron (uc007b	1	85649988	85683118	33131	1	20684	uc007bud.2	-12893	ENSMUSG000	Sp100	nuclear antigen Sp100
chr1	85638103	85638457	355	356	85638305	46	14.41184	3.75459	10.96188	Adipose_R2_peak_1	Intron (uc007b	1	85649988	85683118	33131	1	20684	uc007bud.2	-11531	ENSMUSG000	Sp100	nuclear antigen Sp100
chr1	85638177	85638358	182	183	85638314	30	6.94832	2.77718	4.0017	Adipose_R1_peak_1	Intron (uc007b	1	85649988	85683118	33131	1	20684	uc007bud.2	-11630	ENSMUSG000	Sp100	nuclear antigen Sp100
chr1	85638665	85638841	177	178	85638695	25	4.86612	2.39766	2.09018	Adipose_R2_peak_1	Intron (uc007b	1	85649988	85683118	33131	1	20684	uc007bud.2	-11147	ENSMUSG000	Sp100	nuclear antigen Sp100
chr1	85639647	85639808	162	163	85639772	31	7.7623	2.95097	4.69388	Adipose_R2_peak_1	Intron (uc007b	1	85649988	85683118	33131	1	20684	uc007bud.2	-10180	ENSMUSG000	Sp100	nuclear antigen Sp100
chr1	85643464	85643571	108	109	85643557	28	6.25023	2.67431	3.31626	Adipose_R2_peak_1	Intron (uc007b	1	85649988	85683118	33131	1	20684	uc007bud.2	-6417	ENSMUSG000	Sp100	nuclear antigen Sp100
chr1	85643939	85644154	216	217	85644042	32	8.29282	3.04319	5.18352	Adipose_R2_peak_1	Intron (uc007b	1	85649988	85683118	33131	1	20684	uc007bud.2	-5834	ENSMUSG000	Sp100	nuclear antigen Sp100
chr1	85645938	85646104	167	168	85646020	38	11.72962	3.59649	8.4047	Adipose_R2_peak_1	Distal Interger	1	85649988	85683118	33131	1	20684	uc007bud.2	-3884	ENSMUSG000	Sp100	nuclear antigen Sp100
chr1	85648360	85648566	207	208	85648480	49	19.00719	4.61089	15.38598	Adipose_R2_peak_1	Promoter (1-2	1	85649988	85683118	33131	1	20684	uc007bud.2	-1422	ENSMUSG000	Sp100	nuclear antigen Sp100
chr1	85650628	85651162	535	536	85650738	42	10.21238	3.03599	6.98067	Adipose_R2_peak_1	Promoter (<=	1	85649988	85683118	33131	1	20684	uc007bud.2	640	ENSMUSG000	Sp100	nuclear antigen Sp100
chr1	85651551	85651668	118	119	85651618	30	7.24477	2.85875	4.21917	Adipose_R2_peak_1	Promoter (1-2	1	85649988	85683118	33131	1	20684	uc007bud.2	1563	ENSMUSG000	Sp100	nuclear antigen Sp100
chr1	85651948	85652173	226	227	85652054	25	11.36566	4.67319	8.13482	Adipose_R1_peak_1	Promoter (1-2	1	85649988	85683118	33131	1	20684	uc007bud.2	1960	ENSMUSG000	Sp100	nuclear antigen Sp100
chr1	85652292	85652480	189	190	85652426	31	7.7623	2.95097	4.69388	Adipose_R2_peak_1	Promoter (2-3	1	85649988	85683118	33131	1	20684	uc007bud.2	2304	ENSMUSG000	Sp100	nuclear antigen Sp100
chr1	85653425	85653632	208	209	85653527	40	12.19307	3.58986	8.85454	Adipose_R2_peak_1	Intron (uc007b	1	85649988	85683118	33131	1	20684	uc007bud.2	3437	ENSMUSG000	Sp100	nuclear antigen Sp100
chr1	85657295	85657437	143	144	85657380	28	6.25023	2.67431	3.31626	Adipose_R2_peak_1	Intron (uc007b	1	85649988	85683118	33131	1	20684	uc007bud.2	7307	ENSMUSG000	Sp100	nuclear antigen Sp100
chr1	85668019	85668322	304	305	85668190	18	6.42934	3.41502	3.48999	Adipose_R1_peak_1	Intron (uc007b	1	85649988	85683118	33131	1	20684	uc007bud.2	18031	ENSMUSG000	Sp100	nuclear antigen Sp100
chr1	86435209	86435573	365	366	86435341	19	7.07687	3.59476	4.08458	Adipose_R1_peak_1	Distal Interger	1	86426329	86428049	1721	1	71863	uc007bvo.2	8880	ENSMUSG000	1700019O17R	RIKEN cDNA 1700019O17 gene
chr1	86435633	86435820	188	189	86435760	32	8.29282	3.04319	5.18352	Adipose_R2_peak_1	Distal Interger	1	86426329	86428049	1721	1	71863	uc007bvo.2	9304	ENSMUSG000	1700019O17R	RIKEN cDNA 1700019O17 gene
chr1	86441370	86441514	145	146	86441454	16	5.20017	3.05554	2.38734	Adipose_R1_peak_1	Distal Interger	1	86426329	86428049	1721	1	71863	uc007bvo.2	15041	ENSMUSG000	1700019O17R	RIKEN cDNA 1700019O17 gene
chr1	86461265	86461400	136	137	86461342	19	7.07687	3.59476	4.08458	Adipose_R1_peak_1	Distal Interger	1	86426329	86428049	1721	1	71863	uc007bvo.2	34936	ENSMUSG000	1700019O17R	RIKEN cDNA 1700019O17 gene
chr1	86465727	86465869	143	144	86465842	19	7.07687	3.59476	4.08458	Adipose_R1_peak_1	Distal Interger	1	86426329	86428049	1721	1	71863	uc007bvo.2	39398	ENSMUSG000	1700019O17R	RIKEN cDNA 1700019O17 gene
chr1	86489921	86490105	185	186	86489984	19	7.07687	3.59476	4.08458	Adipose_R1_peak_1	Distal Interger	1	86526736	86530698	3963	1	19231	uc007bvp.1	-36631	ENSMUSG000	Ptma	prothymosin alpha
chr1	86498983	86499126	144	145	86499019	25	4.86612	2.39766	2.09018	Adipose_R2_peak_1	Distal Interger	1	86526736	86530698	3963	1	19231	uc007bvp.1	-27610	ENSMUSG000	Ptma	prothymosin alpha
chr1	86506963	86507137	175	176	86507010	28	6.25023	2.67431	3.31626	Adipose_R2_peak_1	Distal Interger	1	86526736	86530698	3963	1	19231	uc007bvp.1	-19599	ENSMUSG000	Ptma	prothymosin alpha
chr1	86520984	86521150	167	168	86521127	20	7.74501	3.7745	4.70467	Adipose_R1_peak_1	Distal Interger	1	86526736	86530698	3963	1	19231	uc007bvp.1	-5586	ENSMUSG000	Ptma	prothymosin alpha
chr1	86523626	86523787	162	163	86523781	21	8.43286	3.95423	5.34896	Adipose_R1_peak_1	Promoter (2-3	1	86526736	86530698	3963	1	19231	uc007bvp.1	-2949	ENSMUSG000	Ptma	prothymosin alpha
chr1	86527872	86528010	139	140	86527891	25	4.86612	2.39766	2.09018	Adipose_R2_peak_1	Promoter (1-2	1	86526736	86530698	3963	1	19231	uc007bvp.1	1136	ENSMUSG000	Ptma	prothymosin alpha
chr1	86530022	86530152	131	132	86530067	26	5.31252	2.48988	2.47999	Adipose_R2_peak_1	3' UTR	1	86526736	86530698	3963	1	19231	uc007bvp.1	3286	ENSMUSG000	Ptma	prothymosin alpha
chr1	87847156	87847337	182	183	87847240	26	12.14079	4.85292	8.87838	Adipose_R1_peak_1	Distal Interger	1	87853287	87944489	91203	1	227333	uc011wos.1	-5950	ENSMUSG000	Dgkd	diacylglycerol kinase, delta
chr1	87868137	87868294	158	159	87868244	19	7.07687	3.59476	4.08458	Adipose_R1_peak_1	Intron (uc011v	1	87853287	87944489	91203	1	227333	uc011wos.1	14850	ENSMUSG000	Dgkd	diacylglycerol kinase, delta
chr1	87873632	87873771	140	141	87873676	29	6.74062	2.76653	3.75989	Adipose_R2_peak_1	Intron (uc011v	1	87853287	87944489	91203	1	227333	uc011wos.1	20345	ENSMUSG000	Dgkd	diacylglycerol kinase, delta
chr1	87873633	87873783	151	152	87873721	34	18.86162	6.29083	15.38482	Adipose_R1_peak_1	Intron (uc011v	1	87853287	87944489	91203	1	227333	uc011wos.1	20346	ENSMUSG000	Dgkd	diacylglycerol kinase, delta
chr1	87972086	87972311	226	227	87972193	26	12.14079	4.85292	8.87838	Adipose_R1_peak_1	Intron (uc007b	1	87949932	87975931	26000	2	227334	uc011wot.1	3620	ENSMUSG000	Usp40	ubiquitin specific peptidase 40
chr1	88029141	88029404	264	265	88029261	46	30.36364	8.44768	26.64488	Adipose_R1_peak_1	Distal Interger	1	87945121	88008551	63431	2	227334	uc007bxw.1	-20590	ENSMUSG000	Usp40	ubiquitin specific peptidase 40
chr1	88029161	88029348	188	189	88029261	34	9.39139	3.22762	6.20474	Adipose_R2_peak_1	Distal Interger	1	87945121	88008551	63431	2	227334	uc007bxw.1	-20610	ENSMUSG000	Usp40	ubiquitin specific peptidase 40
chr1	88029928	88030222	295	296	88030076	81	53.27079	9.42121	49.25919	Adipose_R1_peak_1	Distal Interger	1	87945121	88008551	63431	2	227334	uc007bxw.1	-21377	ENSMUSG000	Usp40	ubiquitin specific peptidase 40
chr1	88029943	88030213	271	272	88030074	62	26.73374	5.32436	22.92123	Adipose_R2_peak_1	Distal Interger	1	87945121	88008551	63431	2	227334	uc007bxw.1	-21392	ENSMUSG000	Usp40	ubiquitin specific peptidase 40
chr1	88040996	88041288	293	294	88041169	32	7.9847	2.96049	4.90786	Adipose_R2_peak_1	Distal Interger	1	88055411	88220002	164592	1	394430	uc007bya.1	-14123	ENSMUSG000	Ugt1a10	UDP glycosyltransferase 1 family, polypeptide A10
chr1	88052124	88052360	237	238	88052262	32	17.10103	5.93135	13.67137	Adipose_R1_peak_1	Distal Interger	1	88055411	88220002	164592	1	394430	uc007bya.1	-3051	ENSMUSG000	Ugt1a10	UDP glycosyltransferase 1 family, polypeptide A10
chr1	88052175	88052347	173	174	88052276	36	10.5378	3.41206	7.27949	Adipose_R2_peak_1	Distal Interger	1	88055411	88220002	164592	1	394430	uc007bya.1	-3064	ENSMUSG000	Ugt1a10	UDP glycosyltransferase 1 family, polypeptide A10
chr1	88210302	88210413	112	113	88210347	42	11.12667	3.23544	7.83613	Adipose_R2_peak_1	Promoter (1-2	1	88211959	88220002	8044	1	394436	uc007byk.1	-1546	ENSMUSG000	Ugt1a1	UDP glucuronosyltransferase 1 family, polypeptide A1
chr1	88211420	88211578	159	160	88211464	41	8.11919	2.63253	5.03754	Adipose_R2_peak_1	Promoter (<=	1	88211959	88220002	8044	1	394436	uc007byk.1	-381	ENSMUSG000	Ugt1a1	UDP glucuronosyltransferase 1 family, polypeptide A1
chr1	88211700	88212886	1187	1188	88211777	50	6.1012	2.06297	3.20409	Adipose_R2_peak_1	Promoter (<=	1	88211959	88220002	8044	1	394436	uc007byk.1	0	ENSMUSG000	Ugt1a1	UDP glucuronosyltransferase 1 family, polypeptide A1
chr1	88213127	88213268	142	143	88213200	53	6.08179	2.01655	3.18542	Adipose_R2_peak_1	Promoter (1-2	1	88211959	88220002	8044	1	394436	uc007byk.1	1168	ENSMUSG000	Ugt1a1	UDP glucuronosyltransferase 1 family, polypeptide A1
chr1	88213505	88215260	1756	1757	88213753	78	16.00163	2.89093	12.49231	Adipose_R2_peak_1	Promoter (1-2	1	88211959	88220002	8044	1	394436	uc007byk.1	1546	ENSMUSG000	Ugt1a1	UDP glucuronosyltransferase 1 family, polypeptide A1
chr1	88215554	88216290	737	738	88215639	66	13.24669	2.80684	9.85106	Adipose_R2_peak_1	3' UTR	1	88211959	88220002	8044	1	394436	uc007byk.1	3595	ENSMUSG000	Ugt1a1	UDP glucuronosyltransferase 1 family, polypeptide A1
chr1	88215932	88216154	223	224	88216081	45	9.19062	2.71922	6.06589	Adipose_R1_peak_1	3' UTR	1	88211959	88220002	8044	1	394436	uc007byk.1	3973	ENSMUSG000	Ugt1a1	UDP glucuronosyltransferase 1 family, polypeptide A1
chr1	88216582	88217180	599	600	88216680	67	11.9423	2.60793	8.61094	Adipose_R2_peak_1	3' UTR	1	88211959	88220002	8044	1	394436	uc007byk.1	4623	ENSMUSG000	Ugt1a1	UDP glucuronosyltransferase 1 family, polypeptide A1
chr1	88217344	88217871	528	529	88217615	74	14.91578	2.84442	11.44156	Adipose_R2_peak_1	3' UTR	1	88211959	88220002	8044	1	394436	uc007byk.1	5385	ENSMUSG000	Ugt1a1	UDP glucuronosyltransferase 1 family, polypeptide A1
chr1	88218039	88218443	405	406	88218157	73	14.32105	2.79119	10.873	Adipose_R2_peak_1	3' UTR	1	88211959	88220002	8044	1	394436	uc007byk.1	6080	ENSMUSG000	Ugt1a1	UDP glucuronosyltransferase 1 family, polypeptide A1
chr1	88218647	88218871	225	226	88218688	51	5.41876	1.93946	2.58244	Adipose_R2_peak_1	3' UTR	1	88211959	88220002	8044	1	394436	uc007byk.1	6688	ENSMUSG000	Ugt1a1	UDP glucuronosyltransferase 1 family, polypeptide A1
chr1	88219193	88219496	304	305	88219311	92	16.09999	2.64636	12.58876	Adipose_R2_peak_1	3' UTR	1	88211959	88220002	8044	1	394436	uc007byk.1	7234	ENSMUSG000	Ugt1a1	UDP glucuronosyltransferase 1 family, polypeptide A1
chr1	88219665	88220231	567	568	88219835	98	21.63865	3.09482	17.94724	Adipose_R2_peak_1	3' UTR	1	88227020	88262289	35270	1	1.00E+08	uc033fki.1	-6789	ENSMUSG000	Mroh2a	maestro heat-like repeat family member 2A

chr1	88219711	88219870	160	161	88219782	45	6.29728	2.18227	3.40986	Adipose_R1_peak_1	3' UTR	1	88227020	88262289	35270	1	1.00E+08	uc033fki.1	-7150	ENSMUSG000	Mroh2a	maestro heat-like repeat family member 2A
chr1	88220342	88221720	1379	1380	88220464	86	16.75351	2.81627	13.21736	Adipose_R2_peak_1	Distal Intergen	1	88227020	88262289	35270	1	1.00E+08	uc033fki.1	-5300	ENSMUSG000	Mroh2a	maestro heat-like repeat family member 2A
chr1	88220715	88220955	241	242	88220885	42	5.18106	2.03167	2.38734	Adipose_R1_peak_1	Distal Intergen	1	88227020	88262289	35270	1	1.00E+08	uc033fki.1	-6065	ENSMUSG000	Mroh2a	maestro heat-like repeat family member 2A
chr1	88222229	88222528	300	301	88222355	82	13.98535	2.58357	10.55821	Adipose_R2_peak_1	Distal Intergen	1	88227020	88262289	35270	1	1.00E+08	uc033fki.1	-4492	ENSMUSG000	Mroh2a	maestro heat-like repeat family member 2A
chr1	88222834	88223462	629	630	88223126	92	21.77115	3.23816	18.07783	Adipose_R2_peak_1	Distal Intergen	1	88227020	88262289	35270	1	1.00E+08	uc033fki.1	-3558	ENSMUSG000	Mroh2a	maestro heat-like repeat family member 2A
chr1	88223569	88224772	1204	1205	88223604	63	7.13124	2.03799	4.13544	Adipose_R2_peak_1	Promoter (2-3	1	88227020	88262289	35270	1	1.00E+08	uc033fki.1	-2248	ENSMUSG000	Mroh2a	maestro heat-like repeat family member 2A
chr1	88224950	88225124	175	176	88225057	69	13.32253	2.74876	9.92534	Adipose_R2_peak_1	Promoter (1-2	1	88227020	88262289	35270	1	1.00E+08	uc033fki.1	-1896	ENSMUSG000	Mroh2a	maestro heat-like repeat family member 2A
chr1	88225448	88225886	439	440	88225491	50	5.87378	2.02609	2.98532	Adipose_R2_peak_1	Promoter (1-2	1	88227020	88262289	35270	1	1.00E+08	uc033fki.1	-1134	ENSMUSG000	Mroh2a	maestro heat-like repeat family member 2A
chr1	88226039	88226160	122	123	88226113	53	7.17674	2.18588	4.17957	Adipose_R2_peak_1	Promoter (<=	1	88227020	88262289	35270	1	1.00E+08	uc033fki.1	-860	ENSMUSG000	Mroh2a	maestro heat-like repeat family member 2A
chr1	88226488	88227218	731	732	88226622	58	9.51114	2.44791	6.32032	Adipose_R2_peak_1	Promoter (<=	1	88227020	88262289	35270	1	1.00E+08	uc033fki.1	0	ENSMUSG000	Mroh2a	maestro heat-like repeat family member 2A
chr1	88227391	88229276	1886	1887	88227621	59	9.41031	2.41364	6.22322	Adipose_R2_peak_1	Promoter (<=	1	88227020	88262289	35270	1	1.00E+08	uc033fki.1	371	ENSMUSG000	Mroh2a	maestro heat-like repeat family member 2A
chr1	88229381	88229712	332	333	88229502	58	10.70704	2.6271	7.44352	Adipose_R2_peak_1	Promoter (2-3	1	88227020	88262289	35270	1	1.00E+08	uc033fki.1	2361	ENSMUSG000	Mroh2a	maestro heat-like repeat family member 2A
chr1	88229841	88230229	389	390	88230027	61	11.90875	2.73552	8.57848	Adipose_R2_peak_1	Promoter (2-3	1	88227020	88262289	35270	1	1.00E+08	uc033fki.1	2821	ENSMUSG000	Mroh2a	maestro heat-like repeat family member 2A
chr1	88230363	88232034	1672	1673	88230572	53	8.53496	2.39973	5.41656	Adipose_R2_peak_1	Exon (uc033fki	1	88227020	88262289	35270	1	1.00E+08	uc033fki.1	3343	ENSMUSG000	Mroh2a	maestro heat-like repeat family member 2A
chr1	88231344	88231516	173	174	88231366	35	5.06841	2.14814	2.3227	Adipose_R1_peak_1	Intron (uc033f	1	88227020	88262289	35270	1	1.00E+08	uc033fki.1	4324	ENSMUSG000	Mroh2a	maestro heat-like repeat family member 2A
chr1	88232147	88234172	2026	2027	88232216	49	5.86007	2.03869	2.97208	Adipose_R2_peak_1	Exon (uc033fki	1	88227020	88262289	35270	1	1.00E+08	uc033fki.1	5127	ENSMUSG000	Mroh2a	maestro heat-like repeat family member 2A
chr1	88234355	88235022	668	669	88234527	69	15.82949	3.08877	12.32358	Adipose_R2_peak_1	Exon (uc033fki	1	88227020	88262289	35270	1	1.00E+08	uc033fki.1	7335	ENSMUSG000	Mroh2a	maestro heat-like repeat family member 2A
chr1	88235532	88235753	222	223	88235700	54	8.49003	2.37234	5.37308	Adipose_R2_peak_1	Intron (uc033f	1	88227020	88262289	35270	1	1.00E+08	uc033fki.1	8512	ENSMUSG000	Mroh2a	maestro heat-like repeat family member 2A
chr1	88235868	88236291	424	425	88235963	62	12.20717	2.75503	8.86841	Adipose_R2_peak_1	Intron (uc033f	1	88227020	88262289	35270	1	1.00E+08	uc033fki.1	8848	ENSMUSG000	Mroh2a	maestro heat-like repeat family member 2A
chr1	88236667	88237616	950	951	88236813	59	9.41031	2.41364	6.22322	Adipose_R2_peak_1	Exon (uc033fki	1	88227020	88262289	35270	1	1.00E+08	uc033fki.1	9647	ENSMUSG000	Mroh2a	maestro heat-like repeat family member 2A
chr1	88237763	88239133	1371	1372	88237938	67	15.10289	3.04178	11.62461	Adipose_R2_peak_1	Exon (uc033fki	1	88227020	88262289	35270	1	1.00E+08	uc033fki.1	10743	ENSMUSG000	Mroh2a	maestro heat-like repeat family member 2A
chr1	88239418	88240556	1139	1140	88239585	69	13.48368	2.77007	10.08205	Adipose_R2_peak_1	Exon (uc033fki	1	88227020	88262289	35270	1	1.00E+08	uc033fki.1	12398	ENSMUSG000	Mroh2a	maestro heat-like repeat family member 2A
chr1	88240777	88241818	1042	1043	88240923	62	9.90164	2.42722	6.69766	Adipose_R2_peak_1	Exon (uc033fki	1	88227020	88262289	35270	1	1.00E+08	uc033fki.1	13757	ENSMUSG000	Mroh2a	maestro heat-like repeat family member 2A
chr1	88241954	88242541	588	589	88242041	60	11.42211	2.68747	8.12111	Adipose_R2_peak_1	Exon (uc033fki	1	88227020	88262289	35270	1	1.00E+08	uc033fki.1	14934	ENSMUSG000	Mroh2a	maestro heat-like repeat family member 2A
chr1	88242695	88243296	602	603	88242959	65	11.98355	2.655	8.65121	Adipose_R2_peak_1	Exon (uc033fki	1	88227020	88262289	35270	1	1.00E+08	uc033fki.1	15675	ENSMUSG000	Mroh2a	maestro heat-like repeat family member 2A
chr1	88243080	88243280	201	202	88243104	36	5.07751	2.12754	2.33131	Adipose_R1_peak_1	Intron (uc033f	1	88227020	88262289	35270	1	1.00E+08	uc033fki.1	16060	ENSMUSG000	Mroh2a	maestro heat-like repeat family member 2A
chr1	88243589	88244211	623	624	88243637	52	7.68792	2.28428	4.64587	Adipose_R2_peak_1	Exon (uc033fki	1	88227020	88262289	35270	1	1.00E+08	uc033fki.1	16569	ENSMUSG000	Mroh2a	maestro heat-like repeat family member 2A
chr1	88244373	88244751	379	380	88244556	66	12.37938	2.6876	9.02111	Adipose_R2_peak_1	Intron (uc033f	1	88227020	88262289	35270	1	1.00E+08	uc033fki.1	17353	ENSMUSG000	Mroh2a	maestro heat-like repeat family member 2A
chr1	88244852	88245272	421	422	88244889	47	4.78809	1.88699	2.06164	Adipose_R2_peak_1	Exon (uc033fki	1	88227020	88262289	35270	1	1.00E+08	uc033fki.1	17832	ENSMUSG000	Mroh2a	maestro heat-like repeat family member 2A
chr1	88245392	88245885	494	495	88245423	49	5.36645	1.95738	2.53195	Adipose_R2_peak_1	Intron (uc033f	1	88227020	88262289	35270	1	1.00E+08	uc033fki.1	18372	ENSMUSG000	Mroh2a	maestro heat-like repeat family member 2A
chr1	88246028	88246261	234	235	88246126	59	8.12491	2.2292	5.04284	Adipose_R2_peak_1	Exon (uc033fki	1	88227020	88262289	35270	1	1.00E+08	uc033fki.1	19008	ENSMUSG000	Mroh2a	maestro heat-like repeat family member 2A
chr1	88246406	88246797	392	393	88246550	54	7.61817	2.23718	4.57823	Adipose_R2_peak_1	Intron (uc033f	1	88227020	88262289	35270	1	1.00E+08	uc033fki.1	19386	ENSMUSG000	Mroh2a	maestro heat-like repeat family member 2A
chr1	88247071	88247740	670	671	88247211	52	7.51407	2.25652	4.4783	Adipose_R2_peak_1	Exon (uc033fki	1	88227020	88262289	35270	1	1.00E+08	uc033fki.1	20051	ENSMUSG000	Mroh2a	maestro heat-like repeat family member 2A
chr1	88247883	88249044	1162	1163	88248092	71	12.14695	2.55776	8.80975	Adipose_R2_peak_1	Exon (uc033fki	1	88258856	88269413	10558	2	381280	uc011wow.1	20369	ENSMUSG000	Hjurp	Holliday junction recognition protein
chr1	88249175	88251104	1930	1931	88249220	61	10.24116	2.49409	7.00858	Adipose_R2_peak_1	Exon (uc033fki	1	88258856	88269413	10558	2	381280	uc011wow.1	18309	ENSMUSG000	Hjurp	Holliday junction recognition protein
chr1	88251270	88251801	532	533	88251363	53	8.425	2.38222	5.31093	Adipose_R2_peak_1	Exon (uc033fki	1	88258856	88269413	10558	2	381280	uc011wow.1	17612	ENSMUSG000	Hjurp	Holliday junction recognition protein
chr1	88252114	88252754	641	642	88252282	64	13.32594	2.86732	9.92871	Adipose_R2_peak_1	Exon (uc033fki	1	88258856	88269413	10558	2	381280	uc011wow.1	16659	ENSMUSG000	Hjurp	Holliday junction recognition protein
chr1	88252994	88254458	1465	1466	88253333	69	16.16698	3.13599	12.65447	Adipose_R2_peak_1	Intron (uc033f	1	88258856	88269413	10558	2	381280	uc011wow.1	14955	ENSMUSG000	Hjurp	Holliday junction recognition protein
chr1	88253668	88253885	218	219	88253820	45	9.70707	2.81947	6.57765	Adipose_R1_peak_1	Intron (uc033f	1	88258856	88269413	10558	2	381280	uc011wow.1	15528	ENSMUSG000	Hjurp	Holliday junction recognition protein
chr1	88254774	88255348	575	576	88254931	57	9.81005	2.51342	6.6089	Adipose_R2_peak_1	Exon (uc033fki	1	88258856	88269413	10558	2	381280	uc011wow.1	14065	ENSMUSG000	Hjurp	Holliday junction recognition protein
chr1	88255539	88255841	303	304	88255630	56	7.3355	2.16179	4.30663	Adipose_R2_peak_1	Exon (uc033fki	1	88258856	88269413	10558	2	381280	uc011wow.1	13572	ENSMUSG000	Hjurp	Holliday junction recognition protein
chr1	88255948	88257749	1802	1803	88256099	62	10.69114	2.53775	7.42799	Adipose_R2_peak_1	Exon (uc033fki	1	88258856	88269413	10558	2	381280	uc011wow.1	11664	ENSMUSG000	Hjurp	Holliday junction recognition protein
chr1	88258036	88258204	169	170	88258096	45	5.81561	2.09602	2.92932	Adipose_R2_peak_1	Intron (uc033f	1	88258856	88269413	10558	2	381280	uc011wow.1	11209	ENSMUSG000	Hjurp	Holliday junction recognition protein
chr1	88258353	88259583	1231	1232	88258505	42	4.80549	1.96096	2.07848	Adipose_R2_peak_1	3' UTR	1	88258856	88269413	10558	2	381280	uc011wow.1	9830	ENSMUSG000	Hjurp	Holliday junction recognition protein
chr1	88258914	88259113	200	201	88259025	53	14.41752	3.40951	11.09092	Adipose_R1_peak_1	3' UTR	1	88258856	88269413	10558	2	381280	uc011wow.1	10300	ENSMUSG000	Hjurp	Holliday junction recognition protein
chr1	88259809	88262467	2659	2660	88259960	60	11.67835	2.72564	8.36968	Adipose_R2_peak_1	3' UTR	1	88258856	88269413	10558	2	381280	uc011wow.1	6946	ENSMUSG000	Hjurp	Holliday junction recognition protein
chr1	88262612	88264768	2157	2158	88262774	70	16.99257	3.22292	13.44021	Adipose_R2_peak_1	3' UTR	1	88258856	88269413	10558	2	381280	uc011wow.1	4645	ENSMUSG000	Hjurp	Holliday junction recognition protein
chr1	88263998	88264190	193	194	88264023	34	5.13815	2.18781	2.38734	Adipose_R1_peak_1	3' UTR	1	88258856	88269413	10558	2	381280	uc011wow.1	5223	ENSMUSG000	Hjurp	Holliday junction recognition protein
chr1	88264885	88266228	1344	1345	88265005	61	11.05733	2.61119	7.78311	Adipose_R2_peak_1	3' UTR	1	88258856	88269413	10558	2	381280	uc011wow.1	3185	ENSMUSG000	Hjurp	Holliday junction recognition protein
chr1	88266363	88266870	508	509	88266438	60	11.33951	2.67521	8.04101	Adipose_R2_peak_1	Promoter (2-3	1	88258856	88269413	10558	2	381280	uc011wow.1	2543	ENSMUSG000	Hjurp	Holliday junction recognition protein
chr1	88267012	88268321	1310	1311	88267065	47	6.59257	2.19628	3.64566	Adipose_R2_peak_1	Promoter (1-2	1	88258856	88269413	10558	2	381280	uc011wow.1	1092	ENSMUSG000	Hjurp	Holliday junction recognition protein
chr1	88268479	88269038	560	561	88268624	61	13.59261	2.98865	10.1803	Adipose_R2_peak_1	Promoter (<=	1	88258856	88269413	10558	2	381280	uc011wow.1	375	ENSMUSG000	Hjurp	Holliday junction recognition protein
chr1	88268752	88268889	138	139	88268774	31	4.77692	2.17912	2.0371	Adipose_R1_peak_1	Promoter (<=	1	88258856	88269413	10558	2	381280	uc011wow.1	524	ENSMUSG000	Hjurp	Holliday junction recognition protein
chr1	88269231	88270261	1031	1032	88269390	53	11.21196	2.8398	7.91711	Adipose_R2_peak_1	Promoter (<=	1	88258856	88269413	10558	2	381280	uc011wow.1	0	ENSMUSG000	Hjurp	Holliday junction recognition protein
chr1	88271514	88272245	732	733	88271724	54	12.35397	3.0035	8.99658	Adipose_R2_peak_1	Promoter (2-3	1	88258856	88269413	10558	2	381280	uc011wow.1	-2101	ENSMUSG000	Hjurp	Holliday junction recognition protein
chr1	88272596	88273062	467	468	88272710	52	11.40297	2.90337	8.10245	Adipose_R2_peak_1	Exon (uc007by	1	88258856	88269413	10558	2	381280	uc011wow.1	-3183	ENSMUSG000	Hjurp	Holliday junction recognition protein
chr1	88273364	88277438	4075	4076	88273476	51	8.72229	2.47391	5.59675	Adipose_R2_peak_1	Promoter (<=	1	88258856	88277579	18724	2	381280	uc007bym.2	141	ENSMUSG000	Hjurp	Holliday junction recognition protein
chr1	88275298	88275464	167	168	88275361	34	5.94798	2.37466	3.06609	Adipose_R1_peak_1	Promoter (2-3	1	88258856	88277579	18724	2	381280	uc007bym.2	2115	ENSMUSG000	Hjurp	Holliday junction recognition protein
chr1	88277693	88278590	898	899	88277818	59	12.69067	2.90646	9.32404	Adipose_R2_peak_1	Promoter (<=	1	88258856	88277579	18724	2	381280	uc007bym.2	-114	ENSMUSG000	Hjurp	Holliday junction recognition protein
chr1	88278103	88278235	133	134	88278161	34	5.0893	2.17665	2.3428	Adipose_R1_peak_1	Promoter (<=	1	88258856	88277579	18724	2	381280	uc007bym.2	-524	ENSMUSG000	Hjurp	Holliday junction recognition protein
chr1	88279003	88280929	1927	1928	88279045	47	5.99084	2.0925	3.09801	Adipose_R2_peak_1	Promoter (1-2	1	88258856	88277579	18724	2	381280	uc007bym.2	-1424	ENSMUSG000	Hjurp	Holliday junction recognition protein
chr1	88281082	88281663	582	583	88281147	51	9.02499	2.52451	5.86903	Adipose_R2_peak_1	Distal Intergen	1	88258856	88277579	18724	2	381280	uc007bym.2	-3503	ENSMUSG000	Hjurp	Holliday junction recognition protein
chr1	88281782	88282328	547	548	88281906	63	13.32938	2.89379	9.93207	Adipose_R2_peak_1	Distal Intergen	1	88258856	88277579	18724	2	381280	uc007bym.2	-4203	ENSMUSG000	Hjurp	Holliday junction recognition protein
chr1	88282762	88285535	2774	2775	88282906	49	7.98318	2.39438	4.90662	Adipose_R2_peak_1	Distal Intergen	1	88258856	88277579	18724	2	381280	uc007bym.2	-5183	ENSMUSG000	Hjurp	Holliday junction recognition protein
chr1	88284150	88284423	274	275	88284172	34	4.83828	2.11953	2.09712	Adipose_R1_peak_1	Distal Intergen	1	88258856	88277579	18724	2	381280	uc007bym.2	-6571	ENSMUSG000	Hjurp	Holliday junction recognition protein
chr1	88285691	88285886	196	197	88285760	50	8.75768	2.50335	5.63102	Adipose_R2_peak_1	Distal Intergen	1	88258856	88277579	18724	2	381280	uc007bym.2	-8112	ENSMUSG000	Hjurp	Holliday junction recognition protein
chr1	88286086	88286745	660	661	88286275	63	11.40814	2.61787	8.10746	Adipose_R2_peak_1	Distal Intergen	1	88258856	88277579	18724	2	381280	uc007bym.2	-8507	ENSMUSG000	Hjurp	Holliday junction recognition protein
chr1	88286869	88288708	1840	1841	88286953	67	15.6159	3.11511	12.1145	Adipose_R2_peak_1	Distal Intergen	1	88258856	88277579	18724	2	381280	uc007bym.2	-9290	ENSMUSG000	Hjurp	Holliday junction recognition protein
chr1	88288863	88289010	148	149	88288939	45	6.3317	2.18846	3.39472	Adipose_R2_peak_1	Distal Intergen	1	88258856	88277579	18724	2	381280	uc007bym.2	-11284	ENSMUSG000	Hjurp	Holliday junction recognition protein
chr1	88289329	88289433	105	106	88289372	41	6.32927	2.27257	3.39239	Adipose_R2_peak_1	Distal Intergen	1	88258856	88277579	18724	2	381280	uc007bym.2	-11750	ENSMUSG000	Hjurp	Holliday junction recognition protein
chr1	88289627	88290834	1208	1209	88289769	47	8.79951	2.58743	5.67166	Adipose_R2_peak_1	Distal Intergen	1	88258856	88277579	18724	2	381280	uc007bym.2	-12048	ENSMUSG000	Hjurp	Holliday junction recognition protein
chr1	88291123	88292290	1168	1169	88291234	64	14.99629	3.11269	11.52024	Adipose_R2_peak_1	Distal Intergen	1	88303283	88388851	85569	1	171382	uc029qrk.1	-10993	ENSMUSG000	Trpm8	transient receptor potential cation channel, subfamily M, member 8
chr1	88291545	88291935	391	392	88291765	39	8.73343	2.83431	5.64648	Adipose_R1_peak_1	Distal Intergen	1	88303283	88388851	85569	1	171382	uc029qrk.1	-11348	ENSMUSG000	Trpm8	transient receptor potential cation channel, subfamily M, member 8
chr1	88294394	88295003	610	611	88294434	37	5.91217	2.28374	3.02211	Adipose_R2_peak_1	Distal Intergen	1	88303283	88388851	85569	1	171382	uc029qrk.1	-8280	ENSMUSG000	Trpm8	transient receptor potential cation channel, subfamily M, member 8
chr1	88295446	88295977	532	533	88295613	50	11.49099	2.98532	8.18798	Adipose_R2_peak_1	Distal Intergen	1	88303283	88388851	85569	1	171382	uc029qrk.1	-7306	ENSMUSG000	Trpm8	transient receptor potential cation channel, subfamily M, member 8
chr1	88296316	88296786	471	472	88296446	62	10.37341	2.49307	7.13663	Adipose_R2_peak_1	Distal Intergen	1	88303283	88388851	85569	1	171382	uc029qrk.1	-6497	ENSMUSG000	Trpm8	transient receptor potential cation channel, subfamily M, member 8
chr1	88297308	88297832	525	526	88297397	57	15.3218	3.4078	11.83878	Adipose_R2_peak_1	Distal Intergen	1	88303283	88388851	85569	1	171382	uc029qrk.1	-5451	ENSMUSG000	Trpm8	transient receptor potential cation channel, subfamily M, member 8
chr1	88297343	88297623	281	282	88297434	33	7.26475	2.72859	4.27013	Adipose_R1_peak_1	Distal Intergen	1	88303283	88388851	85569	1	171382	uc029qrk.1	-5660	ENSMUSG000	Trpm8	transient receptor potential cation channel, subfamily M, member 8
chr1	88297943	88299145	1203	1204	88298049	53	9.28183	2.51974	6.11692	Adipose_R2_peak_1	Distal Intergen	1	88303283	88388851	85569	1	171382	uc029qrk.1	-4138	ENSMUSG000	Trpm8	transient receptor potential cation channel, subfamily M, member 8
chr1	88299306	88301912	2607	2608	88299478	58	12.9358	2.97438	9.56246	Adipose_R2_peak_1	Promoter (1-2	1	88303283	88388851	85569	1	171382	uc029qrk.1	-1371	ENSMUSG000	Trpm8	transient receptor potential cation channel, subfamily M, member 8
chr1	88301360	88301537	178	179	88301452	39	6.38839	2.33302	3.48999	Adipose_R1_peak_1	Promoter (1-2	1	88303283	88388851	85569	1	171382	uc029qrk.1	-1746	ENSMUSG000	Trpm8	transient receptor potential cation channel, subfamily M, member 8
chr1	88302024	88302831	808	809	88302133	60	13.44514	2.99516	10.04461	Adipose_R2_peak_1	Promoter (<=	1	88303283	88388851	85569	1	171382	uc029qrk.1	-452	ENSMUSG000	Trpm8	transient receptor potential cation channel, subfamily M, member 8
chr1	88302931	88303349	419	420	88303003	55	10.50849	2.66891	7.26747	Adipose_R2_peak_1	Promoter (<=	1	88303283	88388851	85569	1	171382	uc029qrk.1	0	ENSMUSG000	Trpm8	transient receptor potential cation channel, subfamily M, member 8
chr1	88303762	88304846	1085	1086	88304057	75	20.44009	3.54721	16.78434	Adipose_R2_peak_1	Promoter (<=	1	88303283	88388851	85569	1	171382	uc029qrk.1	479	ENSMUSG000	Trpm8	transient receptor potential cation channel, subfamily M, member 8
chr1	88305148	88306301	1154	1155	88305245	53	9.77778	2.60055	6.57776	Adipose_R2_peak_1	Promoter (<=	1	88306662	88388851	82190	1	171382	uc029qrl.1	-361	ENSMUSG000	Trpm8	transient receptor potential cation channel, subfamily M, member 8
chr1	88305596	88305864	269	270	88305692	41	9.04891	2.82698	5.95902	Adipose_R1_peak_1	Promoter (<=	1	88306662	88388851	82190	1	171382	uc029qrl.1	-798	ENSMUSG000	Trpm8	transient receptor potential cation channel, subfamily M, member 8
chr1	88306533	88306965	433	434	88306868	47	6.6802	2.21147	3.72971	Adipose_R2_peak_1	Promoter (<=	1	88306662	88388851	82190	1	171382	uc029qrl.1	0	ENSMUSG000	Trpm8	transient receptor potential cation channel, subfamily M, member 8
chr1	88307106	88307397	292	293	88307241	52	7.59711	2.26977	4.55795	Adipose_R2_peak_1	Promoter (<=	1	88306662	88388851	82190	1	171382	uc029qrl.1	444	ENSMUSG000	Trpm8	transient receptor potential cation channel, subfamily M, member 8
chr1	88307611	88308878	1268	1269	88307876	64	16.82322	3.39272	13.28592	Adipose_R2_peak_1	Promoter (<=	1	88306662	88388851	82190	1	171382	uc029qrl.1	949	ENSMUSG000	Trpm8	transient receptor potential cation channel, subfamily M, member 8
chr1	88308654	88308871	218	219	88308756	44	12.21458	3.37793	8.95167	Adipose_R1_peak_1	Promoter (1-2	1	88306662	88388851	82190	1	171382	uc029qrl.1	1992	ENSMUSG000	Trpm8	transient receptor potential cation channel, subfamily M, member 8
chr1	88309021	88309557	537	538	88309247	50	8.61034	2.47831	5.4889	Adipose_R2_peak_1	Promoter (2-3	1	88306662	88388851	82190	1	171382	uc029qrl.1	2359	ENSMUSG000	Trpm8	transient receptor potential cation channel, subfamily M, member 8
chr1	88309720	88310319	600	601	88309748	44	5.81185	2.11317	2.92565	Adipose_R2_peak_1	Intron (uc029c	1	88306662	88388851	82190	1	171382	uc029qrl.1	3058	ENSMUSG000	Trpm8	transient receptor potential cation channel, subfamily M, member 8
chr1	88310501	88310810	310	311	88310594	44	6.43946	2.22809	3.49831	Adipose_R2_peak_1	Intron (uc029c	1	88306662	88388851	82190	1	171382	uc029qrl.1	3839	ENSMUSG000	Trpm8	transient receptor potential cation channel, subfamily M, member 8
chr1	88611587	88611760	174	175	88611651	22	9.13959	4.13397	6.01526	Adipose_R1_peak_1	Distal Intergen	1	88551517	88562166	10650	1	1.01E+08	uc007bys.2	60070	ENSMUSG000	Gm19589	predicted gene, 19589

chr1	88615055	88615165	111	112	88615092	26	5.31252	2.48988	2.47999	Adipose_R2_peak_1	Distal Interger	1	88551517	88562166	10650	1	1.01E+08	uc007bys.2	63538	ENSMUSG000	Gm19589	predicted gene, 19589
chr1	90714368	90714509	142	143	90714450	21	8.43286	3.95423	5.34896	Adipose_R1_peak_1	Distal Interger	1	90791467	90808076	16610	2	12835	uc007bzk.1	93567	ENSMUSG000	Col6a3	collagen, type VI, alpha 3
chr1	90743731	90743864	134	135	90743762	28	6.25023	2.67431	3.31626	Adipose_R2_peak_1	Distal Interger	1	90791467	90808076	16610	2	12835	uc007bzk.1	64212	ENSMUSG000	Col6a3	collagen, type VI, alpha 3
chr1	90839239	90839346	108	109	90839290	30	7.24477	2.85875	4.21917	Adipose_R2_peak_1	Intron (uc011v	1	90766860	90843971	77112	2	12835	uc011wpc.2	4625	ENSMUSG000	Col6a3	collagen, type VI, alpha 3
chr1	92416261	92416474	214	215	92416373	30	15.39182	5.57188	12.01394	Adipose_R1_peak_1	Distal Interger	1	92464483	92464542	60	2	1.02E+08	uc033fko.1	48068	ENSMUSG000	Mir6900	microRNA 6900
chr1	92416314	92416413	100	101	92416364	31	7.7623	2.95097	4.69388	Adipose_R2_peak_1	Distal Interger	1	92464483	92464542	60	2	1.02E+08	uc033fko.1	48129	ENSMUSG000	Mir6900	microRNA 6900
chr1	92722537	92722751	215	216	92722601	50	34.50784	9.16663	30.72138	Adipose_R1_peak_1	Distal Interger	1	92644218	92648841	4624	2	260301	uc007cbr.1	-73696	ENSMUSG000	Otos	otospiralin
chr1	93870454	93870601	148	149	93870521	22	9.13959	4.13397	6.01526	Adipose_R1_peak_1	Intron (uc007c	1	93861344	93876494	15151	1	381334	uc007ces.1	9110	ENSMUSG000	Gal3st2	galactose-3-O-sulfotransferase 2
chr1	93875056	93875289	234	235	93875158	30	7.24477	2.85875	4.21917	Adipose_R2_peak_1	3' UTR	1	93861344	93876494	15151	1	381334	uc007ces.1	13712	ENSMUSG000	Gal3st2	galactose-3-O-sulfotransferase 2
chr1	93959837	93960000	164	165	93959901	39	12.34187	3.68871	8.98501	Adipose_R2_peak_1	Distal Interger	1	93990509	94011539	21031	1	619597	uc007cet.1	-30509	ENSMUSG000	Gm6086	predicted gene 6086
chr1	93959841	93960011	171	172	93959911	25	11.36566	4.67319	8.13482	Adipose_R1_peak_1	Distal Interger	1	93990509	94011539	21031	1	619597	uc007cet.1	-30498	ENSMUSG000	Gm6086	predicted gene 6086
chr1	94107415	94107611	197	198	94107513	25	11.36566	4.67319	8.13482	Adipose_R1_peak_1	Distal Interger	1	94038305	94052553	14249	2	18566	uc007cev.1	-54862	ENSMUSG000	Pdcd1	programmed cell death 1
chr1	94107445	94107586	142	143	94107529	29	6.74062	2.76653	3.75989	Adipose_R2_peak_1	Distal Interger	1	94038305	94052553	14249	2	18566	uc007cev.1	-54892	ENSMUSG000	Pdcd1	programmed cell death 1
chr1	94240464	94240694	231	232	94240595	32	17.10103	5.93135	13.67137	Adipose_R1_peak_1	Distal Interger	1	94038305	94052553	14249	2	18566	uc007cev.1	-187911	ENSMUSG000	Pdcd1	programmed cell death 1
chr1	94391502	94391742	241	242	94391629	36	20.67077	6.6503	17.14779	Adipose_R1_peak_1	Distal Interger	1	94038305	94052553	14249	2	18566	uc007cev.1	-338949	ENSMUSG000	Pdcd1	programmed cell death 1
chr1	94391560	94391688	129	130	94391627	32	8.29282	3.04319	5.18352	Adipose_R2_peak_1	Distal Interger	1	94038305	94052553	14249	2	18566	uc007cev.1	-339007	ENSMUSG000	Pdcd1	programmed cell death 1
chr1	94392240	94392514	275	276	94392382	51	31.78214	8.01111	28.0443	Adipose_R1_peak_1	Distal Interger	1	94038305	94052553	14249	2	18566	uc007cev.1	-339687	ENSMUSG000	Pdcd1	programmed cell death 1
chr1	94392250	94392492	243	244	94392374	56	24.19188	5.25641	20.43163	Adipose_R2_peak_1	Distal Interger	1	94038305	94052553	14249	2	18566	uc007cev.1	-339697	ENSMUSG000	Pdcd1	programmed cell death 1
chr1	94476713	94476906	194	195	94476827	25	11.36566	4.67319	8.13482	Adipose_R1_peak_1	Distal Interger	1	94797442	94802472	5031	1	74689	uc007cew.1	-320536	ENSMUSG000	4930440C22Ri	RIKEN cDNA 4930440C22 gene
chr1	94476757	94476921	165	166	94476837	39	12.34187	3.68871	8.98501	Adipose_R2_peak_1	Distal Interger	1	94797442	94802472	5031	1	74689	uc007cew.1	-320521	ENSMUSG000	4930440C22Ri	RIKEN cDNA 4930440C22 gene
chr1	94479719	94480060	342	343	94479868	136	134.0556	20.58635	129.5917	Adipose_R1_peak_1	Distal Interger	1	94797442	94802472	5031	1	74689	uc007cew.1	-317382	ENSMUSG000	4930440C22Ri	RIKEN cDNA 4930440C22 gene
chr1	94479727	94480006	280	281	94479867	106	70.05344	9.8673	65.71089	Adipose_R2_peak_1	Distal Interger	1	94797442	94802472	5031	1	74689	uc007cew.1	-317436	ENSMUSG000	4930440C22Ri	RIKEN cDNA 4930440C22 gene
chr1	94648926	94649109	184	185	94649002	24	10.60668	4.49345	7.40961	Adipose_R1_peak_1	Distal Interger	1	94797442	94802472	5031	1	74689	uc007cew.1	-148333	ENSMUSG000	4930440C22Ri	RIKEN cDNA 4930440C22 gene
chr1	94664286	94664451	166	167	94664349	19	7.07687	3.59476	4.08458	Adipose_R1_peak_1	Distal Interger	1	94797442	94802472	5031	1	74689	uc007cew.1	-132991	ENSMUSG000	4930440C22Ri	RIKEN cDNA 4930440C22 gene
chr1	94728467	94728642	176	177	94728560	40	12.96465	3.78093	9.57654	Adipose_R2_peak_1	Distal Interger	1	94797442	94802472	5031	1	74689	uc007cew.1	-68800	ENSMUSG000	4930440C22Ri	RIKEN cDNA 4930440C22 gene
chr1	94728475	94728649	175	176	94728569	23	8.69536	3.843	5.60874	Adipose_R1_peak_1	Distal Interger	1	94797442	94802472	5031	1	74689	uc007cew.1	-68793	ENSMUSG000	4930440C22Ri	RIKEN cDNA 4930440C22 gene
chr1	94922848	94923113	266	267	94922947	40	24.42476	7.36925	20.81738	Adipose_R1_peak_1	Distal Interger	1	94797442	94802472	5031	1	74689	uc007cew.1	125406	ENSMUSG000	4930440C22Ri	RIKEN cDNA 4930440C22 gene
chr1	94922900	94923035	136	137	94922957	31	7.7623	2.95097	4.69388	Adipose_R2_peak_1	Distal Interger	1	94797442	94802472	5031	1	74689	uc007cew.1	125458	ENSMUSG000	4930440C22Ri	RIKEN cDNA 4930440C22 gene
chr1	94925387	94925634	248	249	94925505	36	20.67077	6.6503	17.14779	Adipose_R1_peak_1	Distal Interger	1	94797442	94802472	5031	1	74689	uc007cew.1	127945	ENSMUSG000	4930440C22Ri	RIKEN cDNA 4930440C22 gene
chr1	94925397	94925607	211	212	94925515	42	14.2409	3.96536	10.79479	Adipose_R2_peak_1	Distal Interger	1	94797442	94802472	5031	1	74689	uc007cew.1	127955	ENSMUSG000	4930440C22Ri	RIKEN cDNA 4930440C22 gene
chr1	94926848	94927040	193	194	94926953	25	11.36566	4.67319	8.13482	Adipose_R1_peak_1	Distal Interger	1	94797442	94802472	5031	1	74689	uc007cew.1	129406	ENSMUSG000	4930440C22Ri	RIKEN cDNA 4930440C22 gene
chr1	94941826	94942054	229	230	94941953	31	16.23981	5.75161	12.83549	Adipose_R1_peak_1	Distal Interger	1	94797442	94802472	5031	1	74689	uc007cew.1	144384	ENSMUSG000	4930440C22Ri	RIKEN cDNA 4930440C22 gene
chr1	95156456	95156811	356	357	95156594	47	31.38649	8.62742	27.65048	Adipose_R1_peak_1	Distal Interger	1	95313628	95327945	14318	1	67698	uc007cex.2	-156817	ENSMUSG000	Fam174a	family with sequence similarity 174, member A
chr1	95156493	95156702	210	211	95156591	38	11.72962	3.59649	8.4047	Adipose_R2_peak_1	Distal Interger	1	95313628	95327945	14318	1	67698	uc007cex.2	-156926	ENSMUSG000	Fam174a	family with sequence similarity 174, member A
chr1	95228913	95229090	178	179	95229006	25	11.36566	4.67319	8.13482	Adipose_R1_peak_1	Distal Interger	1	95313628	95327945	14318	1	67698	uc007cex.2	-84538	ENSMUSG000	Fam174a	family with sequence similarity 174, member A
chr1	95238159	95238610	452	453	95238257	31	16.23981	5.75161	12.83549	Adipose_R1_peak_1	Distal Interger	1	95313628	95327945	14318	1	67698	uc007cex.2	-75018	ENSMUSG000	Fam174a	family with sequence similarity 174, member A
chr1	95238208	95238585	378	379	95238278	33	8.83596	3.1354	5.68652	Adipose_R2_peak_1	Distal Interger	1	95313628	95327945	14318	1	67698	uc007cex.2	-75043	ENSMUSG000	Fam174a	family with sequence similarity 174, member A
chr1	95239029	95239317	289	290	95239235	23	9.86443	4.31371	6.70247	Adipose_R1_peak_1	Distal Interger	1	95313628	95327945	14318	1	67698	uc007cex.2	-74311	ENSMUSG000	Fam174a	family with sequence similarity 174, member A
chr1	95451936	95452070	135	136	95452002	43	14.8939	4.05758	11.42017	Adipose_R2_peak_1	Distal Interger	1	95313628	95327945	14318	1	67698	uc007cex.2	138308	ENSMUSG000	Fam174a	family with sequence similarity 174, member A
chr1	95787045	95787145	101	102	95787090	28	6.25023	2.67431	3.31626	Adipose_R2_peak_1	Distal Interger	1	95701184	95716518	15335	2	75381	uc029qsf.1	-70527	ENSMUSG000	4930598F16Ri	RIKEN cDNA 4930598F16 gene
chr1	95787047	95787222	176	177	95787114	23	9.86443	4.31371	6.70247	Adipose_R1_peak_1	Distal Interger	1	95701184	95716518	15335	2	75381	uc029qsf.1	-70529	ENSMUSG000	4930598F16Ri	RIKEN cDNA 4930598F16 gene
chr1	95787852	95788032	181	182	95787922	22	9.13959	4.13397	6.01526	Adipose_R1_peak_1	Distal Interger	1	95701184	95716518	15335	2	75381	uc029qsf.1	-71334	ENSMUSG000	4930598F16Ri	RIKEN cDNA 4930598F16 gene
chr1	95810199	95810458	260	261	95810330	52	36.63102	9.52611	32.81188	Adipose_R1_peak_1	Distal Interger	1	95701184	95716518	15335	2	75381	uc029qsf.1	-93681	ENSMUSG000	4930598F16Ri	RIKEN cDNA 4930598F16 gene
chr1	95810240	95810458	219	220	95810321	47	17.60017	4.42645	14.02531	Adipose_R2_peak_1	Distal Interger	1	95701184	95716518	15335	2	75381	uc029qsf.1	-93722	ENSMUSG000	4930598F16Ri	RIKEN cDNA 4930598F16 gene
chr1	95814039	95814359	321	322	95814094	17	5.80341	3.23528	2.92378	Adipose_R1_peak_1	Distal Interger	1	95701184	95716518	15335	2	75381	uc029qsf.1	-97521	ENSMUSG000	4930598F16Ri	RIKEN cDNA 4930598F16 gene
chr1	95830802	95831141	340	341	95831016	92	34.13638	4.76652	30.19255	Adipose_R2_peak_1	Distal Interger	1	95701184	95716518	15335	2	75381	uc029qsf.1	-114284	ENSMUSG000	4930598F16Ri	RIKEN cDNA 4930598F16 gene
chr1	95830850	95831075	226	227	95830955	36	5.07751	2.12754	2.33131	Adipose_R1_peak_1	Distal Interger	1	95701184	95716518	15335	2	75381	uc029qsf.1	-114332	ENSMUSG000	4930598F16Ri	RIKEN cDNA 4930598F16 gene
chr1	95986809	95986946	138	139	95986881	18	6.42934	3.41502	3.48999	Adipose_R1_peak_1	Distal Interger	1	95990659	96021758	31100	2	73454	uc029qsg.1	34812	ENSMUSG000	1700063A18R	RIKEN cDNA 1700063A18 gene
chr1	96079275	96079466	192	193	96079390	22	9.13959	4.13397	6.01526	Adipose_R1_peak_1	Distal Interger	1	95990659	96021758	31100	2	73454	uc029qsg.1	-57517	ENSMUSG000	1700063A18R	RIKEN cDNA 1700063A18 gene
chr1	96191852	96192091	240	241	96191982	47	17.60017	4.42645	14.02531	Adipose_R2_peak_1	Distal Interger	1	95990659	96021758	31100	2	73454	uc029qsg.1	-170094	ENSMUSG000	1700063A18R	RIKEN cDNA 1700063A18 gene
chr1	96191854	96192124	271	272	96191991	45	29.34988	8.26794	25.64927	Adipose_R1_peak_1	Distal Interger	1	95990659	96021758	31100	2	73454	uc029qsg.1	-170096	ENSMUSG000	1700063A18R	RIKEN cDNA 1700063A18 gene
chr1	96195864	96196046	183	184	96195949	25	11.36566	4.67319	8.13482	Adipose_R1_peak_1	Distal Interger	1	95990659	96021758	31100	2	73454	uc029qsg.1	-174106	ENSMUSG000	1700063A18R	RIKEN cDNA 1700063A18 gene
chr1	96206197	96206339	143	144	96206285	18	6.42934	3.41502	3.48999	Adipose_R1_peak_1	Distal Interger	1	95990659	96021758	31100	2	73454	uc029qsg.1	-184439	ENSMUSG000	1700063A18R	RIKEN cDNA 1700063A18 gene
chr1	96245458	96245619	162	163	96245528	17	5.80341	3.23528	2.92378	Adipose_R1_peak_1	Distal Interger	1	95990659	96021758	31100	2	73454	uc029qsg.1	-223700	ENSMUSG000	1700063A18R	RIKEN cDNA 1700063A18 gene
chr1	96443409	96443569	161	162	96443494	23	9.86443	4.31371	6.70247	Adipose_R1_peak_1	Distal Interger	1	96612057	96662573	50517	2	75159	uc029qsi.1	219004	ENSMUSG000	4930533P14Ri	RIKEN cDNA 4930533P14 gene
chr1	96537340	96537451	112	113	96537406	27	5.77405	2.5821	2.88921	Adipose_R2_peak_1	Distal Interger	1	96612057	96662573	50517	2	75159	uc029qsi.1	125122	ENSMUSG000	4930533P14Ri	RIKEN cDNA 4930533P14 gene
chr1	96572483	96572869	387	388	96572755	37	21.59274	6.83004	18.04766	Adipose_R1_peak_1	Distal Interger	1	96612057	96662573	50517	2	75159	uc029qsi.1	89704	ENSMUSG000	4930533P14Ri	RIKEN cDNA 4930533P14 gene
chr1	96572705	96572822	118	119	96572754	29	6.74062	2.76653	3.75989	Adipose_R2_peak_1	Distal Interger	1	96612057	96662573	50517	2	75159	uc029qsi.1	89751	ENSMUSG000	4930533P14Ri	RIKEN cDNA 4930533P14 gene
chr1	96575212	96575588	377	378	96575434	50	34.50784	9.16663	30.72138	Adipose_R1_peak_1	Distal Interger	1	96612057	96662573	50517	2	75159	uc029qsi.1	86985	ENSMUSG000	4930533P14Ri	RIKEN cDNA 4930533P14 gene
chr1	96575277	96575565	289	290	96575446	48	18.29934	4.51867	14.70076	Adipose_R2_peak_1	Distal Interger	1	96612057	96662573	50517	2	75159	uc029qsi.1	87008	ENSMUSG000	4930533P14Ri	RIKEN cDNA 4930533P14 gene
chr1	96575951	96576255	305	306	96576103	112	111.8104	20.31038	107.4269	Adipose_R1_peak_1	Distal Interger	1	96612057	96662573	50517	2	75159	uc029qsi.1	86318	ENSMUSG000	4930533P14Ri	RIKEN cDNA 4930533P14 gene
chr1	96575993	96576221	229	230	96576104	69	34.76717	6.45524	30.8116	Adipose_R2_peak_1	Distal Interger	1	96612057	96662573	50517	2	75159	uc029qsi.1	86352	ENSMUSG000	4930533P14Ri	RIKEN cDNA 4930533P14 gene
chr1	96670148	96670302	155	156	96670228	24	10.60668	4.49345	7.40961	Adipose_R1_peak_1	Distal Interger	1	96612057	96662573	50517	2	75159	uc029qsi.1	-7575	ENSMUSG000	4930533P14Ri	RIKEN cDNA 4930533P14 gene
chr1	96680668	96680768	101	102	96680718	27	5.77405	2.5821	2.88921	Adipose_R2_peak_1	Distal Interger	1	96612057	96662573	50517	2	75159	uc029qsi.1	-18095	ENSMUSG000	4930533P14Ri	RIKEN cDNA 4930533P14 gene
chr1	96680673	96680805	133	134	96680740	20	7.74501	3.7745	4.70467	Adipose_R1_peak_1	Distal Interger	1	96612057	96662573	50517	2	75159	uc029qsi.1	-18100	ENSMUSG000	4930533P14Ri	RIKEN cDNA 4930533P14 gene
chr1	96732941	96733161	221	222	96733023	18	6.42934	3.41502	3.48999	Adipose_R1_peak_1	Distal Interger	1	96612057	96662573	50517	2	75159	uc029qsi.1	-70368	ENSMUSG000	4930533P14Ri	RIKEN cDNA 4930533P14 gene
chr1	96744428	96744742	315	316	96744571	53	37.70483	9.70585	33.87	Adipose_R1_peak_1	Distal Interger	1	96612057	96662573	50517	2	75159	uc029qsi.1	-81855	ENSMUSG000	4930533P14Ri	RIKEN cDNA 4930533P14 gene
chr1	96744466	96744724	259	260	96744569	56	24.19188	5.25641	20.43163	Adipose_R2_peak_1	Distal Interger	1	96612057	96662573	50517	2	75159	uc029qsi.1	-81893	ENSMUSG000	4930533P14Ri	RIKEN cDNA 4930533P14 gene
chr1	96983171	96983478	308	309	96983305	48	31.30305	8.3971	27.57971	Adipose_R1_peak_1	Intron (uc007c	1	96906176	96997560	91385	2	67854	uc007cfe.2	14082	ENSMUSG000	Slco6b1	solute carrier organic anion transporter family, member 6b1
chr1	96983183	96983428	246	247	96983311	48	18.29934	4.51867	14.70076	Adipose_R2_peak_1	Intron (uc007c	1	96906176	96997560	91385	2	67854	uc007cfe.2	14132	ENSMUSG000	Slco6b1	solute carrier organic anion transporter family, member 6b1
chr1	97025113	97025455	343	344	97025202	31	7.7623	2.95097	4.69388	Adipose_R2_peak_1	Distal Interger	1	96906176	96997560	91385	2	67854	uc007cfe.2	-27553	ENSMUSG000	Slco6b1	solute carrier organic anion transporter family, member 6b1
chr1	97147073	97147236	164	165	97147103	27	5.77405	2.5821	2.88921	Adipose_R2_peak_1	Distal Interger	1	97059449	97128303	68855	2	74441	uc007cff.1	-18770	ENSMUSG000	Slco6c1	solute carrier organic anion transporter family, member 6c1
chr1	97197964	97198163	200	201	97198050	27	12.93148	5.03266	9.63827	Adipose_R1_peak_1	Distal Interger	1	97059449	97128303	68855	2	74441	uc007cff.1	-69661	ENSMUSG000	Slco6c1	solute carrier organic anion transporter family, member 6c1
chr1	97275197	97275423	227	228	97275334	30	15.39182	5.57188	12.01394	Adipose_R1_peak_1	Distal Interger	1	97059449	97128303	68855	2	74441	uc007cff.1	-146894	ENSMUSG000	Slco6c1	solute carrier organic anion transporter family, member 6c1
chr1	97275253	97275357	105	106	97275347	28	6.25023	2.67431	3.31626	Adipose_R2_peak_1	Distal Interger	1	97059449	97128303	68855	2	74441	uc007cff.1	-146950	ENSMUSG000	Slco6c1	solute carrier organic anion transporter family, member 6c1
chr1	97392601	97392886	286	287	97392752	69	55.71914	12.52353	51.68306	Adipose_R1_peak_1	Distal Interger	1	97059449	97128303	68855	2	74441	uc007cff.1	-264298	ENSMUSG000	Slco6c1	solute carrier organic anion transporter family, member 6c1
chr1	97392618	97392889	272	273	97392759	63	29.74474	5.90194	25.87429	Adipose_R2_peak_1	Distal Interger	1	97059449	97128303	68855	2	74441	uc007cff.1	-264315	ENSMUSG000	Slco6c1	solute carrier organic anion transporter family, member 6c1
chr1	97420589	97420804	216	217	97420690	50	19.72356	4.7031	16.08069	Adipose_R2_peak_1	Distal Interger	1	97643902	97661676	17775	2	52392	uc007cfg.2	240872	ENSMUSG000	D1Ertd622e	DNA segment, Chr 1, ERATO Doi 622, expressed
chr1	97420603	97420809	207	208	97420694	40	18.81518	5.42586	15.35762	Adipose_R1_peak_1	Distal Interger	1	97643902	97661676	17775	2	52392	uc007cfg.2	240867	ENSMUSG000	D1Ertd622e	DNA segment, Chr 1, ERATO Doi 622, expressed
chr1	97451578	97451814	237	238	97451697	38	22.52591	7.00978	18.96	Adipose_R1_peak_1	Distal Interger	1	97643902	97661676	17775	2	52392	uc007cfg.2	209862	ENSMUSG000	D1Ertd622e	DNA segment, Chr 1, ERATO Doi 622, expressed
chr1	97451606	97451795	190	191	97451695	40	12.96465	3.78093	9.57654	Adipose_R2_peak_1	Distal Interger	1	97643902	97661676	17775	2	52392	uc007cfg.2	209881	ENSMUSG000	D1Ertd622e	DNA segment, Chr 1, ERATO Doi 622, expressed
chr1	97509751	97509901	151	152	97509824	21	8.43286	3.95423	5.34896	Adipose_R1_peak_1	Distal Interger	1	97643902	97661676	17775	2	52392	uc007cfg.2	151775	ENSMUSG000	D1Ertd622e	DNA segment, Chr 1, ERATO Doi 622, expressed
chr1	97511454	97511588	135	136	97511518	17	5.80341	3.23528	2.92378	Adipose_R1_peak_1	Distal Interger	1	97643902	97661676	17775	2	52392	uc007cfg.2	150088	ENSMUSG000	D1Ertd622e	DNA segment, Chr 1, ERATO Doi 622, expressed
chr1	97579581	97579737	157	158	97579699	19	7.07687	3.59476	4.08458	Adipose_R1_peak_1	Distal Interger	1	97643902	97661676	17775	2	52392	uc007cfg.2	81939	ENSMUSG000	D1Ertd622e	DNA segment, Chr 1, ERATO Doi 622, expressed
chr1	98364224	98364402	179	180	98364289	26	12.14079	4.85292	8.87838	Adipose_R1_peak_1	Distal Interger	1	98421124	98509380	88257	1	70866	uc007cfu.2	-56722	ENSMUSG000	Slco6d1	solute carrier organic anion transporter family, member 6d1
chr1	98620289	98620540	252	253	98620425	51	20.44827	4.79532	16.78434	Adipose_R2_peak_1	Intron (uc007c	1	98421124	98509380	88257	1	70866	uc007cfu.2	199165	ENSMUSG000	Slco6d1	solute carrier organic anion transporter family, member 6d1
chr1	98620295	98620553	259	260	98620429	50	34.50784	9.16663	30.72138	Adipose_R1_peak_1	Intron (uc007c	1	98421124	98509380	88257	1	70866	uc007cfu.2	199171	ENSMUSG000	Slco6d1	solute carrier organic anion transporter family, member 6d1
chr1	98648922	98649065	144	145	98648990	20	7.74501	3.7745	4.70467	Adipose_R1_peak_1	Intron (uc007c	1	98421124	98509380	88257	1	70866	uc007cfu.2	227798	ENSMUSG000	Slco6d1	solute carrier organic anion transporter family, member 6d1
chr1	98650659	98650861	203	204	98650783	25	11.36566	4.67319	8.13482	Adipose_R1_peak_1	Intron (uc007c	1	98421124	98509380	88257	1	70866	uc007cfu.2	229535	ENSMUSG000	Slco6d1	solute carrier organic anion transporter family, member 6d1
chr1	98651050	98651214	165	166	98651137	24	10.60668	4.49345	7.40961	Adipose_R1_peak_1	Intron (uc007c	1	98421124	98509380	88257	1	70866	uc007cfu.2	229926	ENSMUSG000	Slco6d1	solute carrier organic anion transporter family, member 6d1
chr1	98767790	98767984	195	196	98767815	17	5.80341	3.23528	2.92378	Adipose_R1_peak_1	Distal Interger	1	98421124	98509380	88257	1	70866	uc007cfu.2	346666	ENSMUSG000	Slco6d1	solute carrier organic anion transporter family, member 6d1
chr1	98767934	98768064	131	132	98767986	32	8.29282	3.04319	5.18352	Adipose_R2_peak_1	Distal Interger	1	98421124	98509380	88257	1	70866	uc007cfu.2	346810	ENSMUSG000	Slco6d1	solute carrier organic anion transporter family, member 6d1
chr1	98768274	98768402	129	130	98768348	34	9.39139	3.22762	6.20474	Adipose_R2_peak_1	Distal Interger	1	98421124	98509380	88257	1	70866	uc007cfu.2	347150	ENSMUSG000	Slco6d1	solute carrier organic anion transporter family, member 6d1
chr1	98768771	98768912	142	143	98768831	18	6.42934	3.41502	3.48999	Adipose_R1_peak_1	Distal Interger	1	98421124	98509380	88257	1	70866	uc007cfu.2	347647	ENSMUSG000	Slco6d1	solute carrier organic anion transporter family, member 6d1

chr1	98796803	98797474	672	673	98796933	35	19.76029	6.47056	16.26028	Adipose_R1_peak_1	Distal Interger	1	98421124	98509380	88257	1	70866	uc007cfu.2	375679	ENSMUSG000	Slco6d1	solute carrier organic anion transporter family, member 6d1
chr1	98796882	98797025	144	145	98796958	34	9.39139	3.22762	6.20474	Adipose_R2_peak_1	Distal Interger	1	98421124	98509380	88257	1	70866	uc007cfu.2	375758	ENSMUSG000	Slco6d1	solute carrier organic anion transporter family, member 6d1
chr1	98797218	98797430	213	214	98797314	48	18.29934	4.51867	14.70076	Adipose_R2_peak_1	Distal Interger	1	98421124	98509380	88257	1	70866	uc007cfu.2	376094	ENSMUSG000	Slco6d1	solute carrier organic anion transporter family, member 6d1
chr1	98800068	98800320	253	254	98800197	44	24.61781	6.76194	21.00949	Adipose_R1_peak_1	Distal Interger	1	98421124	98509380	88257	1	70866	uc007cfu.2	378944	ENSMUSG000	Slco6d1	solute carrier organic anion transporter family, member 6d1
chr1	98800117	98800313	197	198	98800209	37	11.12817	3.50427	7.83613	Adipose_R2_peak_1	Distal Interger	1	98421124	98509380	88257	1	70866	uc007cfu.2	378993	ENSMUSG000	Slco6d1	solute carrier organic anion transporter family, member 6d1
chr1	98805346	98805496	151	152	98805416	21	8.43286	3.95423	5.34896	Adipose_R1_peak_1	Distal Interger	1	98421124	98509380	88257	1	70866	uc007cfu.2	384222	ENSMUSG000	Slco6d1	solute carrier organic anion transporter family, member 6d1
chr1	98811842	98812068	227	228	98811932	33	17.97508	6.11109	14.52164	Adipose_R1_peak_1	Distal Interger	1	98421124	98509380	88257	1	70866	uc007cfu.2	390718	ENSMUSG000	Slco6d1	solute carrier organic anion transporter family, member 6d1
chr1	98843274	98843470	197	198	98843356	22	9.13959	4.13397	6.01526	Adipose_R1_peak_1	Distal Interger	1	98421124	98509380	88257	1	70866	uc007cfu.2	422150	ENSMUSG000	Slco6d1	solute carrier organic anion transporter family, member 6d1
chr1	98845616	98845760	145	146	98845680	19	7.07687	3.59476	4.08458	Adipose_R1_peak_1	Distal Interger	1	98421124	98509380	88257	1	70866	uc007cfu.2	424492	ENSMUSG000	Slco6d1	solute carrier organic anion transporter family, member 6d1
chr1	98886456	98886622	167	168	98886538	20	7.74501	3.7745	4.70467	Adipose_R1_peak_1	Distal Interger	1	98421124	98509380	88257	1	70866	uc007cfu.2	465332	ENSMUSG000	Slco6d1	solute carrier organic anion transporter family, member 6d1
chr1	98888791	98888964	174	175	98888871	25	11.31267	4.6516	8.11033	Adipose_R1_peak_1	Distal Interger	1	98421124	98509380	88257	1	70866	uc007cfu.2	467667	ENSMUSG000	Slco6d1	solute carrier organic anion transporter family, member 6d1
chr1	99070173	99070277	105	106	99070191	26	5.31252	2.48988	2.47999	Adipose_R2_peak_1	Distal Interger	1	98421124	98509380	88257	1	70866	uc007cfu.2	649049	ENSMUSG000	Slco6d1	solute carrier organic anion transporter family, member 6d1
chr1	99184470	99184628	159	160	99184534	22	9.13959	4.13397	6.01526	Adipose_R1_peak_1	Distal Interger	1	99772765	1.00E+08	713178	1	241175	uc007cfy.1	-588137	ENSMUSG000	Cntnap5b	contactin associated protein-like 5B
chr1	99187481	99187647	167	168	99187573	24	10.60668	4.49345	7.40961	Adipose_R1_peak_1	Distal Interger	1	99772765	1.00E+08	713178	1	241175	uc007cfy.1	-585118	ENSMUSG000	Cntnap5b	contactin associated protein-like 5B
chr1	99203934	99204158	225	226	99204079	23	9.86443	4.31371	6.70247	Adipose_R1_peak_1	Distal Interger	1	99772765	1.00E+08	713178	1	241175	uc007cfy.1	-568607	ENSMUSG000	Cntnap5b	contactin associated protein-like 5B
chr1	99205736	99205949	214	215	99205848	36	20.67077	6.6503	17.14779	Adipose_R1_peak_1	Distal Interger	1	99772765	1.00E+08	713178	1	241175	uc007cfy.1	-566816	ENSMUSG000	Cntnap5b	contactin associated protein-like 5B
chr1	99249583	99249800	218	219	99249714	23	9.86443	4.31371	6.70247	Adipose_R1_peak_1	Distal Interger	1	99772765	1.00E+08	713178	1	241175	uc007cfy.1	-522965	ENSMUSG000	Cntnap5b	contactin associated protein-like 5B
chr1	99265417	99265698	282	283	99265562	45	29.34988	8.26794	25.64927	Adipose_R1_peak_1	Distal Interger	1	99772765	1.00E+08	713178	1	241175	uc007cfy.1	-507067	ENSMUSG000	Cntnap5b	contactin associated protein-like 5B
chr1	99265490	99265658	169	170	99265563	36	10.5378	3.41206	7.27949	Adipose_R2_peak_1	Distal Interger	1	99772765	1.00E+08	713178	1	241175	uc007cfy.1	-507107	ENSMUSG000	Cntnap5b	contactin associated protein-like 5B
chr1	99322715	99322888	174	175	99322847	27	12.93148	5.03266	9.63827	Adipose_R1_peak_1	Distal Interger	1	99772765	1.00E+08	713178	1	241175	uc007cfy.1	-449877	ENSMUSG000	Cntnap5b	contactin associated protein-like 5B
chr1	99341596	99341867	272	273	99341690	28	13.73721	5.2124	10.41463	Adipose_R1_peak_1	Distal Interger	1	99772765	1.00E+08	713178	1	241175	uc007cfy.1	-430898	ENSMUSG000	Cntnap5b	contactin associated protein-like 5B
chr1	99345966	99346180	215	216	99346082	26	12.14079	4.85292	8.87838	Adipose_R1_peak_1	Distal Interger	1	99772765	1.00E+08	713178	1	241175	uc007cfy.1	-426585	ENSMUSG000	Cntnap5b	contactin associated protein-like 5B
chr1	99346019	99346151	133	134	99346094	32	8.29282	3.04319	5.18352	Adipose_R2_peak_1	Distal Interger	1	99772765	1.00E+08	713178	1	241175	uc007cfy.1	-426614	ENSMUSG000	Cntnap5b	contactin associated protein-like 5B
chr1	99651304	99651520	217	218	99651439	22	9.13959	4.13397	6.01526	Adipose_R1_peak_1	Distal Interger	1	99772765	1.00E+08	713178	1	241175	uc007cfy.1	-121245	ENSMUSG000	Cntnap5b	contactin associated protein-like 5B
chr1	99820338	99820644	307	308	99820530	31	15.96059	5.64231	12.57961	Adipose_R1_peak_1	Intron (uc007c	1	99772765	1.00E+08	713178	1	241175	uc007cfy.1	47573	ENSMUSG000	Cntnap5b	contactin associated protein-like 5B
chr1	99820474	99820594	121	122	99820542	32	8.29282	3.04319	5.18352	Adipose_R2_peak_1	Intron (uc007c	1	99772765	1.00E+08	713178	1	241175	uc007cfy.1	47709	ENSMUSG000	Cntnap5b	contactin associated protein-like 5B
chr1	99823014	99823260	247	248	99823151	31	16.23981	5.75161	12.83549	Adipose_R1_peak_1	Intron (uc007c	1	99772765	1.00E+08	713178	1	241175	uc007cfy.1	50249	ENSMUSG000	Cntnap5b	contactin associated protein-like 5B
chr1	99923977	99924075	99	100	99924035	27	5.77405	2.5821	2.88921	Adipose_R2_peak_1	Intron (uc007c	1	99772765	1.00E+08	713178	1	241175	uc007cfy.1	151212	ENSMUSG000	Cntnap5b	contactin associated protein-like 5B
chr1	100135398	100135554	157	158	100135457	29	14.55748	5.39214	11.20652	Adipose_R1_peak_1	Intron (uc007c	1	1.00E+08	1.00E+08	388182	1	241175	uc007cfz.1	37637	ENSMUSG000	Cntnap5b	contactin associated protein-like 5B
chr1	100224404	100224498	95	96	100224459	29	6.74062	2.76653	3.75989	Adipose_R2_peak_1	Intron (uc007c	1	1.00E+08	1.00E+08	388182	1	241175	uc007cfz.1	126643	ENSMUSG000	Cntnap5b	contactin associated protein-like 5B
chr1	100225868	100226034	167	168	100225912	29	6.74062	2.76653	3.75989	Adipose_R2_peak_1	Intron (uc007c	1	1.00E+08	1.00E+08	388182	1	241175	uc007cfz.1	128107	ENSMUSG000	Cntnap5b	contactin associated protein-like 5B
chr1	100227369	100227558	190	191	100227453	32	7.71675	2.8894	4.67293	Adipose_R2_peak_1	Intron (uc007c	1	1.00E+08	1.00E+08	388182	1	241175	uc007cfz.1	129608	ENSMUSG000	Cntnap5b	contactin associated protein-like 5B
chr1	100228705	100228844	140	141	100228780	30	7.24477	2.85875	4.21917	Adipose_R2_peak_1	Intron (uc007c	1	1.00E+08	1.00E+08	388182	1	241175	uc007cfz.1	130944	ENSMUSG000	Cntnap5b	contactin associated protein-like 5B
chr1	100230165	100230292	128	129	100230218	31	7.7623	2.95097	4.69388	Adipose_R2_peak_1	Intron (uc007c	1	1.00E+08	1.00E+08	388182	1	241175	uc007cfz.1	132404	ENSMUSG000	Cntnap5b	contactin associated protein-like 5B
chr1	100230891	100231250	360	361	100231115	63	17.11398	3.47559	13.55924	Adipose_R2_peak_1	Intron (uc007c	1	1.00E+08	1.00E+08	388182	1	241175	uc007cfz.1	133130	ENSMUSG000	Cntnap5b	contactin associated protein-like 5B
chr1	100315217	100315478	262	263	100315343	41	25.38991	7.54899	21.76305	Adipose_R1_peak_1	Intron (uc007c	1	1.00E+08	1.00E+08	388182	1	241175	uc007cfz.1	217456	ENSMUSG000	Cntnap5b	contactin associated protein-like 5B
chr1	100315282	100315440	159	160	100315359	35	9.95877	3.31984	6.73562	Adipose_R2_peak_1	Intron (uc007c	1	1.00E+08	1.00E+08	388182	1	241175	uc007cfz.1	217521	ENSMUSG000	Cntnap5b	contactin associated protein-like 5B
chr1	100319311	100319471	161	162	100319399	19	7.07687	3.59476	4.08458	Adipose_R1_peak_1	Intron (uc007c	1	1.00E+08	1.00E+08	388182	1	241175	uc007cfz.1	221550	ENSMUSG000	Cntnap5b	contactin associated protein-like 5B
chr1	100561887	100562042	156	157	100561949	23	9.86443	4.31371	6.70247	Adipose_R1_peak_1	Distal Interger	1	1.00E+08	1.00E+08	388182	1	241175	uc007cfz.1	464126	ENSMUSG000	Cntnap5b	contactin associated protein-like 5B
chr1	100561896	100562051	156	157	100561941	27	5.77405	2.5821	2.88921	Adipose_R2_peak_1	Distal Interger	1	1.00E+08	1.00E+08	388182	1	241175	uc007cfz.1	464135	ENSMUSG000	Cntnap5b	contactin associated protein-like 5B
chr1	100562831	100562997	167	168	100562913	23	9.86443	4.31371	6.70247	Adipose_R1_peak_1	Distal Interger	1	1.00E+08	1.00E+08	388182	1	241175	uc007cfz.1	465070	ENSMUSG000	Cntnap5b	contactin associated protein-like 5B
chr1	100637344	100637509	166	167	100637426	21	8.43286	3.95423	5.34896	Adipose_R1_peak_1	Distal Interger	1	1.01E+08	1.01E+08	19435	1	1.01E+08	uc007cgb.2	-518259	ENSMUSG000	Gm20268	predicted gene, 20268
chr1	100640789	100640971	183	184	100640881	25	11.36566	4.67319	8.13482	Adipose_R1_peak_1	Distal Interger	1	1.01E+08	1.01E+08	19435	1	1.01E+08	uc007cgb.2	-514797	ENSMUSG000	Gm20268	predicted gene, 20268
chr1	100757499	100757637	139	140	100757573	18	6.42934	3.41502	3.48999	Adipose_R1_peak_1	Distal Interger	1	1.01E+08	1.01E+08	19435	1	1.01E+08	uc007cgb.2	-398131	ENSMUSG000	Gm20268	predicted gene, 20268
chr1	100980797	100980911	115	116	100980842	29	6.74062	2.76653	3.75989	Adipose_R2_peak_1	Distal Interger	1	1.01E+08	1.01E+08	19435	1	1.01E+08	uc007cgb.2	-174857	ENSMUSG000	Gm20268	predicted gene, 20268
chr1	100983087	100983279	193	194	100983190	22	9.13959	4.13397	6.01526	Adipose_R1_peak_1	Distal Interger	1	1.01E+08	1.01E+08	19435	1	1.01E+08	uc007cgb.2	-172489	ENSMUSG000	Gm20268	predicted gene, 20268
chr1	100985099	100985234	136	137	100985171	23	9.86443	4.31371	6.70247	Adipose_R1_peak_1	Distal Interger	1	1.01E+08	1.01E+08	19435	1	1.01E+08	uc007cgb.2	-170534	ENSMUSG000	Gm20268	predicted gene, 20268
chr1	100985652	100985821	170	171	100985742	21	8.43286	3.95423	5.34896	Adipose_R1_peak_1	Distal Interger	1	1.01E+08	1.01E+08	19435	1	1.01E+08	uc007cgb.2	-169947	ENSMUSG000	Gm20268	predicted gene, 20268
chr1	100985659	100985821	163	164	100985719	33	8.83596	3.1354	5.68652	Adipose_R2_peak_1	Distal Interger	1	1.01E+08	1.01E+08	19435	1	1.01E+08	uc007cgb.2	-169947	ENSMUSG000	Gm20268	predicted gene, 20268
chr1	100993626	100993956	331	332	100993760	50	34.50784	9.16663	30.72138	Adipose_R1_peak_1	Distal Interger	1	1.01E+08	1.01E+08	19435	1	1.01E+08	uc007cgb.2	-161812	ENSMUSG000	Gm20268	predicted gene, 20268
chr1	100993678	100993883	206	207	100993756	37	11.12817	3.50427	7.83613	Adipose_R2_peak_1	Distal Interger	1	1.01E+08	1.01E+08	19435	1	1.01E+08	uc007cgb.2	-161885	ENSMUSG000	Gm20268	predicted gene, 20268
chr1	101042316	101042479	164	165	101042400	24	10.60668	4.49345	7.40961	Adipose_R1_peak_1	Distal Interger	1	1.01E+08	1.01E+08	19435	1	1.01E+08	uc007cgb.2	-113289	ENSMUSG000	Gm20268	predicted gene, 20268
chr1	101045182	101045535	354	355	101045387	114	114.6097	20.66986	110.214	Adipose_R1_peak_1	Distal Interger	1	1.01E+08	1.01E+08	19435	1	1.01E+08	uc007cgb.2	-110233	ENSMUSG000	Gm20268	predicted gene, 20268
chr1	101045229	101045524	296	297	101045377	90	53.97882	8.39181	49.78112	Adipose_R2_peak_1	Distal Interger	1	1.01E+08	1.01E+08	19435	1	1.01E+08	uc007cgb.2	-110244	ENSMUSG000	Gm20268	predicted gene, 20268
chr1	101103398	101103656	259	260	101103478	34	8.48629	2.99266	5.36945	Adipose_R2_peak_1	Distal Interger	1	1.01E+08	1.01E+08	19435	1	1.01E+08	uc007cgb.2	-52112	ENSMUSG000	Gm20268	predicted gene, 20268
chr1	101105334	101105475	142	143	101105413	32	8.29282	3.04319	5.18352	Adipose_R2_peak_1	Distal Interger	1	1.01E+08	1.01E+08	19435	1	1.01E+08	uc007cgb.2	-50293	ENSMUSG000	Gm20268	predicted gene, 20268
chr1	101263895	101264044	150	151	101263959	20	7.74501	3.7745	4.70467	Adipose_R1_peak_1	Distal Interger	1	1.01E+08	1.01E+08	19435	1	1.01E+08	uc007cgb.2	108127	ENSMUSG000	Gm20268	predicted gene, 20268
chr1	101265250	101265362	113	114	101265346	28	6.25023	2.67431	3.31626	Adipose_R2_peak_1	Distal Interger	1	1.01E+08	1.01E+08	19435	1	1.01E+08	uc007cgb.2	109482	ENSMUSG000	Gm20268	predicted gene, 20268
chr1	101308168	101308560	393	394	101308315	203	205.662	23.21948	201.0231	Adipose_R1_peak_1	Distal Interger	1	1.01E+08	1.01E+08	19435	1	1.01E+08	uc007cgb.2	152400	ENSMUSG000	Gm20268	predicted gene, 20268
chr1	101308175	101308531	357	358	101308319	168	141.0342	15.5848	136.4003	Adipose_R2_peak_1	Distal Interger	1	1.01E+08	1.01E+08	19435	1	1.01E+08	uc007cgb.2	152407	ENSMUSG000	Gm20268	predicted gene, 20268
chr1	101364983	101365126	144	145	101365064	20	7.74501	3.7745	4.70467	Adipose_R1_peak_1	Distal Interger	1	1.01E+08	1.01E+08	19435	1	1.01E+08	uc007cgb.2	209215	ENSMUSG000	Gm20268	predicted gene, 20268
chr1	101375642	101375792	151	152	101375695	20	7.74501	3.7745	4.70467	Adipose_R1_peak_1	Distal Interger	1	1.01E+08	1.01E+08	19435	1	1.01E+08	uc007cgb.2	219874	ENSMUSG000	Gm20268	predicted gene, 20268
chr1	101406039	101406253	215	216	101406158	27	12.93148	5.03266	9.63827	Adipose_R1_peak_1	Distal Interger	1	1.01E+08	1.01E+08	19435	1	1.01E+08	uc007cgb.2	250271	ENSMUSG000	Gm20268	predicted gene, 20268
chr1	101456242	101456505	264	265	101456366	27	12.93148	5.03266	9.63827	Adipose_R1_peak_1	Distal Interger	1	1.01E+08	1.01E+08	19435	1	1.01E+08	uc007cgb.2	300474	ENSMUSG000	Gm20268	predicted gene, 20268
chr1	101606793	101607254	462	463	101606946	31	16.23981	5.75161	12.83549	Adipose_R1_peak_1	Distal Interger	1	1.01E+08	1.01E+08	19435	1	1.01E+08	uc007cgb.2	451025	ENSMUSG000	Gm20268	predicted gene, 20268
chr1	101606883	101607014	132	133	101606941	29	6.74062	2.76653	3.75989	Adipose_R2_peak_1	Distal Interger	1	1.01E+08	1.01E+08	19435	1	1.01E+08	uc007cgb.2	451115	ENSMUSG000	Gm20268	predicted gene, 20268
chr1	101760350	101760524	175	176	101760436	23	9.86443	4.31371	6.70247	Adipose_R1_peak_1	Distal Interger	1	1.01E+08	1.01E+08	19435	1	1.01E+08	uc007cgb.2	604582	ENSMUSG000	Gm20268	predicted gene, 20268
chr1	101830879	101831111	233	234	101831103	50	19.72356	4.7031	16.08069	Adipose_R2_peak_1	Distal Interger	1	1.01E+08	1.01E+08	19435	1	1.01E+08	uc007cgb.2	675111	ENSMUSG000	Gm20268	predicted gene, 20268
chr1	101830913	101831111	199	200	101830978	22	9.13959	4.13397	6.01526	Adipose_R1_peak_1	Distal Interger	1	1.01E+08	1.01E+08	19435	1	1.01E+08	uc007cgb.2	675145	ENSMUSG000	Gm20268	predicted gene, 20268
chr1	101842278	101842545	268	269	101842398	35	19.76029	6.47056	16.26028	Adipose_R1_peak_1	Distal Interger	1	1.01E+08	1.01E+08	19435	1	1.01E+08	uc007cgb.2	686510	ENSMUSG000	Gm20268	predicted gene, 20268
chr1	101898401	101898588	188	189	101898488	22	9.13959	4.13397	6.01526	Adipose_R1_peak_1	Distal Interger	1	1.01E+08	1.01E+08	19435	1	1.01E+08	uc007cgb.2	742633	ENSMUSG000	Gm20268	predicted gene, 20268
chr1	101929635	101929929	295	296	101929772	83	50.76875	8.52693	46.78164	Adipose_R1_peak_1	Distal Interger	1	1.01E+08	1.01E+08	19435	1	1.01E+08	uc007cgb.2	773867	ENSMUSG000	Gm20268	predicted gene, 20268
chr1	101929642	101929918	277	278	101929782	76	40.9047	7.10077	36.85923	Adipose_R2_peak_1	Distal Interger	1	1.01E+08	1.01E+08	19435	1	1.01E+08	uc007cgb.2	773874	ENSMUSG000	Gm20268	predicted gene, 20268
chr1	101932738	101933007	270	271	101932873	62	38.95704	8.42872	35.10588	Adipose_R1_peak_1	Distal Interger	1	1.01E+08	1.01E+08	19435	1	1.01E+08	uc007cgb.2	776970	ENSMUSG000	Gm20268	predicted gene, 20268
chr1	101932775	101932958	184	185	101932873	40	12.96465	3.78093	9.57654	Adipose_R2_peak_1	Distal Interger	1	1.01E+08	1.01E+08	19435	1	1.01E+08	uc007cgb.2	777007	ENSMUSG000	Gm20268	predicted gene, 20268
chr1	101933760	101934014	255	256	101933881	51	35.56531	9.34637	31.76235	Adipose_R1_peak_1	Distal Interger	1	1.01E+08	1.01E+08	19435	1	1.01E+08	uc007cgb.2	777992	ENSMUSG000	Gm20268	predicted gene, 20268
chr1	101933770	101933947	178	179	101933869	37	11.12817	3.50427	7.83613	Adipose_R2_peak_1	Distal Interger	1	1.01E+08	1.01E+08	19435	1	1.01E+08	uc007cgb.2	778002	ENSMUSG000	Gm20268	predicted gene, 20268
chr1	101935657	101935931	275	276	101935809	51	35.56531	9.34637	31.76235	Adipose_R1_peak_1	Distal Interger	1	1.01E+08	1.01E+08	19435	1	1.01E+08	uc007cgb.2	779889	ENSMUSG000	Gm20268	predicted gene, 20268
chr1	101935705	101935877	173	174	101935800	40	12.96465	3.78093	9.57654	Adipose_R2_peak_1	Distal Interger	1	1.01E+08	1.01E+08	19435	1	1.01E+08	uc007cgb.2	779937	ENSMUSG000	Gm20268	predicted gene, 20268
chr1	102002146	102002277	132	133	102002191	27	5.77405	2.5821	2.88921	Adipose_R2_peak_1	Distal Interger	1	1.01E+08	1.01E+08	19435	1	1.01E+08	uc007cgb.2	846378	ENSMUSG000	Gm20268	predicted gene, 20268
chr1	102002152	102002322	171	172	102002215	18	6.42934	3.41502	3.48999	Adipose_R1_peak_1	Distal Interger	1	1.01E+08	1.01E+08	19435	1	1.01E+08	uc007cgb.2	846384	ENSMUSG000	Gm20268	predicted gene, 20268
chr1	102003599	102003800	202	203	102003717	26	12.14079	4.85292	8.87838	Adipose_R1_peak_1	Distal Interger	1	1.01E+08	1.01E+08	19435	1	1.01E+08	uc007cgb.2	847831	ENSMUSG000	Gm20268	predicted gene, 20268
chr1	102005630	102005936	307	308	102005753	66	52.33981	12.04244	48.33179	Adipose_R1_peak_1	Distal Interger	1	1.01E+08	1.01E+08	19435	1	1.01E+08	uc007cgb.2	849862	ENSMUSG000	Gm20268	predicted gene, 20268
chr1	102005640	102005913	274	275	102005771	62	28.93052	5.80972	25.0743	Adipose_R2_peak_1	Distal Interger	1	1.01E+08	1.01E+08	19435	1	1.01E+08	uc007cgb.2	849872	ENSMUSG000	Gm20268	predicted gene, 20268
chr1	102006574	102006819	246	247	102006690	64	50.01186	11.68296	46.0279	Adipose_R1_peak_1	Distal Interger	1	1.01E+08	1.01E+08	19435	1	1.01E+08	uc007cgb.2	850806	ENSMUSG000	Gm20268	predicted gene, 20268
chr1	102006604	102006800	197	198	102006707	38	11.72962	3.59649	8.4047	Adipose_R2_peak_1	Distal Interger	1	1.01E+08	1.01E+08	19435	1	1.01E+08	uc007cgb.2	850836	ENSMUSG000	Gm20268	predicted gene, 20268
chr1	102015449	102015608	160	161	102015526	19	7.07687	3.59476	4.08458	Adipose_R1_peak_1	Distal Interger	1	1.01E+08	1.01E+08	19435	1	1.01E+08	uc007cgb.2	859681	ENSMUSG000	Gm20268	predicted gene, 20268
chr1	102018190	102018475	286	287	102018331	54	38.78658	9.88558	34.93639	Adipose_R1_peak_1	Distal Interger	1	1.01E+08	1.01E+08	19435	1	1.01E+08	uc007cgb.2	862422	ENSMUSG000	Gm20268	predicted gene, 20268
chr1	102018196	102018447	252	253	102018326	47	17.60017	4.42645	14.02531	Adipose_R2_peak_1	Distal Interger	1	1.01E+08	1.01E+08	19435	1	1.01E+08	uc007cgb.2	862428	ENSMUSG000	Gm20268	predicted gene, 20268
chr1	102088180	102088389	210	211	102088263	30	15.39182	5.57188	12.01394	Adipose_R1_peak_1	Distal Interger	1	1.01E+08	1.01E+08	19435	1	1.01E+08	uc007cgb.2	932412	ENSMUSG000	Gm20268	predicted gene, 20268
chr1	102088233	102088356	124	125	102088290	29	6.74062	2.76653	3.75989	Adipose_R2_peak_1	Distal Interger	1	1.01E+08	1.01E+08	19435	1	1.01E+08	uc007cgb.2	932465	ENSMUSG000	Gm20268	predicted gene, 20268
chr1	102123454	102123715	262	263	102123664	18	6.42934	3.41502	3.48999	Adipose_R1_peak_1	Distal Interger	1	1.01E+08	1.01E+08	19435	1	1.01E+08	uc007cgb.2	967686	ENSMUSG000	Gm20268	predicted gene, 20268
chr1	102125041	102125338	298	299	102125216	50	34.50784	9.16663	30.72138	Adipose_R1_peak_1	Distal Interger	1	1.01E+08	1.01E+08	19435	1	1.01E+08	uc007cgb.2	969273	ENSMUSG000	Gm20268	predicted gene, 20268
chr1	102125100	102125265	166	167	102125192	37	11.12817	3.50427	7.83613	Adipose_R2_peak_1	Distal Interger	1	1.01E+08	1.01E+08	19435	1	1.01E+08	uc007cgb.2	969332	ENSMUSG000	Gm20268	predicted gene, 20268

chr1	102175525	102175699	175	176	102175671	18	6.42934	3.41502	3.48999	Adipose_R1_peak_1	Distal Interger	1	1.01E+08	1.01E+08	19435	1	1.01E+08	uc007cgb.2	1019757	ENSMUSG000	Gm20268	predicted gene, 20268
chr1	102245433	102245622	190	191	102245543	24	10.60668	4.49345	7.40961	Adipose_R1_peak_1	Distal Interger	1	1.01E+08	1.01E+08	19435	1	1.01E+08	uc007cgb.2	1089665	ENSMUSG000	Gm20268	predicted gene, 20268
chr1	102251355	102251559	205	206	102251458	27	12.93148	5.03266	9.63827	Adipose_R1_peak_1	Distal Interger	1	1.01E+08	1.01E+08	19435	1	1.01E+08	uc007cgb.2	1095587	ENSMUSG000	Gm20268	predicted gene, 20268
chr1	102384923	102385214	292	293	102385065	71	58.27042	12.94113	54.20487	Adipose_R1_peak_1	Distal Interger	1	1.01E+08	1.01E+08	19435	1	1.01E+08	uc007cgb.2	1229155	ENSMUSG000	Gm20268	predicted gene, 20268
chr1	102384933	102385179	247	248	102385063	67	33.06755	6.27081	29.1399	Adipose_R2_peak_1	Distal Interger	1	1.01E+08	1.01E+08	19435	1	1.01E+08	uc007cgb.2	1229165	ENSMUSG000	Gm20268	predicted gene, 20268
chr1	102388889	102389168	280	281	102389032	37	21.59274	6.83004	18.04766	Adipose_R1_peak_1	Distal Interger	1	1.01E+08	1.01E+08	19435	1	1.01E+08	uc007cgb.2	1233121	ENSMUSG000	Gm20268	predicted gene, 20268
chr1	102388982	102389105	124	125	102389044	31	7.7623	2.95097	4.69388	Adipose_R2_peak_1	Distal Interger	1	1.01E+08	1.01E+08	19435	1	1.01E+08	uc007cgb.2	1233214	ENSMUSG000	Gm20268	predicted gene, 20268
chr1	102390739	102390929	191	192	102390837	23	9.86443	4.31371	6.70247	Adipose_R1_peak_1	Distal Interger	1	1.01E+08	1.01E+08	19435	1	1.01E+08	uc007cgb.2	1234971	ENSMUSG000	Gm20268	predicted gene, 20268
chr1	102595404	102595725	322	323	102595588	39	23.47001	7.18952	19.88325	Adipose_R1_peak_1	Distal Interger	1	1.01E+08	1.01E+08	19435	1	1.01E+08	uc007cgb.2	1439636	ENSMUSG000	Gm20268	predicted gene, 20268
chr1	102595465	102595694	230	231	102595570	37	11.12817	3.50427	7.83613	Adipose_R2_peak_1	Distal Interger	1	1.01E+08	1.01E+08	19435	1	1.01E+08	uc007cgb.2	1439697	ENSMUSG000	Gm20268	predicted gene, 20268
chr1	102603226	102603429	204	205	102603331	24	10.60668	4.49345	7.40961	Adipose_R1_peak_1	Distal Interger	1	1.01E+08	1.01E+08	19435	1	1.01E+08	uc007cgb.2	1447458	ENSMUSG000	Gm20268	predicted gene, 20268
chr1	102605066	102605361	296	297	102605196	43	27.35048	7.90847	23.68561	Adipose_R1_peak_1	Distal Interger	1	1.01E+08	1.01E+08	19435	1	1.01E+08	uc007cgb.2	1449298	ENSMUSG000	Gm20268	predicted gene, 20268
chr1	102605902	102606166	265	266	102606027	39	23.47001	7.18952	19.88325	Adipose_R1_peak_1	Distal Interger	1	1.01E+08	1.01E+08	19435	1	1.01E+08	uc007cgb.2	1450134	ENSMUSG000	Gm20268	predicted gene, 20268
chr1	102605906	102606083	178	179	102606015	42	14.2409	3.96536	10.79479	Adipose_R2_peak_1	Distal Interger	1	1.01E+08	1.01E+08	19435	1	1.01E+08	uc007cgb.2	1450138	ENSMUSG000	Gm20268	predicted gene, 20268
chr1	102628105	102628318	214	215	102628251	34	10.07551	3.411	6.9119	Adipose_R1_peak_1	Distal Interger	1	1.01E+08	1.01E+08	19435	1	1.01E+08	uc007cgb.2	1472337	ENSMUSG000	Gm20268	predicted gene, 20268
chr1	102629493	102629690	198	199	102629599	49	19.00719	4.61089	15.38598	Adipose_R2_peak_1	Distal Interger	1	1.01E+08	1.01E+08	19435	1	1.01E+08	uc007cgb.2	1473725	ENSMUSG000	Gm20268	predicted gene, 20268
chr1	102629502	102629710	209	210	102629603	31	16.23981	5.75161	12.83549	Adipose_R1_peak_1	Distal Interger	1	1.01E+08	1.01E+08	19435	1	1.01E+08	uc007cgb.2	1473734	ENSMUSG000	Gm20268	predicted gene, 20268
chr1	102642574	102642763	190	191	102642637	34	18.86162	6.29083	15.38482	Adipose_R1_peak_1	Distal Interger	1	1.01E+08	1.01E+08	19435	1	1.01E+08	uc007cgb.2	1486806	ENSMUSG000	Gm20268	predicted gene, 20268
chr1	102777694	102777955	262	263	102777818	52	21.18119	4.88754	17.49655	Adipose_R2_peak_1	Distal Interger	1	1.01E+08	1.01E+08	19435	1	1.01E+08	uc007cgb.2	1621926	ENSMUSG000	Gm20268	predicted gene, 20268
chr1	102777700	102777963	264	265	102777819	35	19.76029	6.47056	16.26028	Adipose_R1_peak_1	Distal Interger	1	1.01E+08	1.01E+08	19435	1	1.01E+08	uc007cgb.2	1621932	ENSMUSG000	Gm20268	predicted gene, 20268
chr1	102890513	102890689	177	178	102890575	20	7.74501	3.7745	4.70467	Adipose_R1_peak_1	Distal Interger	1	1.01E+08	1.01E+08	19435	1	1.01E+08	uc007cgb.2	1734745	ENSMUSG000	Gm20268	predicted gene, 20268
chr1	102966126	102966400	275	276	102966279	43	27.35048	7.90847	23.68561	Adipose_R1_peak_1	Distal Interger	1	1.05E+08	1.05E+08	226663	1	23836	uc007cgc.1	-1802419	ENSMUSG000	Cdh20	cadherin 20
chr1	102966150	102966383	234	235	102966266	50	19.72356	4.7031	16.08069	Adipose_R2_peak_1	Distal Interger	1	1.05E+08	1.05E+08	226663	1	23836	uc007cgc.1	-1802436	ENSMUSG000	Cdh20	cadherin 20
chr1	103005279	103005506	228	229	103005393	31	16.23981	5.75161	12.83549	Adipose_R1_peak_1	Distal Interger	1	1.05E+08	1.05E+08	226663	1	23836	uc007cgc.1	-1763313	ENSMUSG000	Cdh20	cadherin 20
chr1	103005304	103005485	182	183	103005394	38	11.72962	3.59649	8.4047	Adipose_R2_peak_1	Distal Interger	1	1.05E+08	1.05E+08	226663	1	23836	uc007cgc.1	-1763334	ENSMUSG000	Cdh20	cadherin 20
chr1	103005733	103006033	301	302	103005876	72	59.47482	13.12087	55.39848	Adipose_R1_peak_1	Distal Interger	1	1.05E+08	1.05E+08	226663	1	23836	uc007cgc.1	-1762786	ENSMUSG000	Cdh20	cadherin 20
chr1	103005784	103006028	245	246	103005881	54	22.67101	5.07198	18.94758	Adipose_R2_peak_1	Distal Interger	1	1.05E+08	1.05E+08	226663	1	23836	uc007cgc.1	-1762791	ENSMUSG000	Cdh20	cadherin 20
chr1	103037716	103037880	165	166	103037815	39	12.34187	3.68871	8.98501	Adipose_R2_peak_1	Distal Interger	1	1.05E+08	1.05E+08	226663	1	23836	uc007cgc.1	-1730939	ENSMUSG000	Cdh20	cadherin 20
chr1	103037758	103037916	159	160	103037839	30	15.39182	5.57188	12.01394	Adipose_R1_peak_1	Distal Interger	1	1.05E+08	1.05E+08	226663	1	23836	uc007cgc.1	-1730903	ENSMUSG000	Cdh20	cadherin 20
chr1	103038276	103038487	212	213	103038389	25	10.2208	4.21861	7.05584	Adipose_R1_peak_1	Distal Interger	1	1.05E+08	1.05E+08	226663	1	23836	uc007cgc.1	-1730332	ENSMUSG000	Cdh20	cadherin 20
chr1	103038327	103038471	145	146	103038421	31	7.7623	2.95097	4.69388	Adipose_R2_peak_1	Distal Interger	1	1.05E+08	1.05E+08	226663	1	23836	uc007cgc.1	-1730348	ENSMUSG000	Cdh20	cadherin 20
chr1	103206688	103206914	227	228	103206800	38	22.52591	7.00978	18.96	Adipose_R1_peak_1	Distal Interger	1	1.05E+08	1.05E+08	226663	1	23836	uc007cgc.1	-1561905	ENSMUSG000	Cdh20	cadherin 20
chr1	103206697	103206908	212	213	103206824	45	16.22858	4.24202	12.70278	Adipose_R2_peak_1	Distal Interger	1	1.05E+08	1.05E+08	226663	1	23836	uc007cgc.1	-1561911	ENSMUSG000	Cdh20	cadherin 20
chr1	103408767	103408942	176	177	103408856	22	9.13959	4.13397	6.01526	Adipose_R1_peak_1	Distal Interger	1	1.05E+08	1.05E+08	226663	1	23836	uc007cgc.1	-1359877	ENSMUSG000	Cdh20	cadherin 20
chr1	103408795	103408929	135	136	103408901	27	5.77405	2.5821	2.88921	Adipose_R2_peak_1	Distal Interger	1	1.05E+08	1.05E+08	226663	1	23836	uc007cgc.1	-1359890	ENSMUSG000	Cdh20	cadherin 20
chr1	103409096	103409359	264	265	103409238	42	26.36523	7.72873	22.71906	Adipose_R1_peak_1	Distal Interger	1	1.05E+08	1.05E+08	226663	1	23836	uc007cgc.1	-1359460	ENSMUSG000	Cdh20	cadherin 20
chr1	103409101	103409329	229	230	103409233	48	18.29934	4.51867	14.70076	Adipose_R2_peak_1	Distal Interger	1	1.05E+08	1.05E+08	226663	1	23836	uc007cgc.1	-1359490	ENSMUSG000	Cdh20	cadherin 20
chr1	103418335	103418645	311	312	103418509	46	30.36364	8.44768	26.64488	Adipose_R1_peak_1	Distal Interger	1	1.05E+08	1.05E+08	226663	1	23836	uc007cgc.1	-1350174	ENSMUSG000	Cdh20	cadherin 20
chr1	103418380	103418620	241	242	103418513	40	12.96465	3.78093	9.57654	Adipose_R2_peak_1	Distal Interger	1	1.05E+08	1.05E+08	226663	1	23836	uc007cgc.1	-1350199	ENSMUSG000	Cdh20	cadherin 20
chr1	103591015	103591116	102	103	103591061	27	5.77405	2.5821	2.88921	Adipose_R2_peak_1	Distal Interger	1	1.05E+08	1.05E+08	226663	1	23836	uc007cgc.1	-1177703	ENSMUSG000	Cdh20	cadherin 20
chr1	103591024	103591171	148	149	103591098	22	9.13959	4.13397	6.01526	Adipose_R1_peak_1	Distal Interger	1	1.05E+08	1.05E+08	226663	1	23836	uc007cgc.1	-1177648	ENSMUSG000	Cdh20	cadherin 20
chr1	103593492	103593740	249	250	103593591	37	11.12817	3.50427	7.83613	Adipose_R2_peak_1	Distal Interger	1	1.05E+08	1.05E+08	226663	1	23836	uc007cgc.1	-1175079	ENSMUSG000	Cdh20	cadherin 20
chr1	103614905	103615082	178	179	103614968	21	8.43286	3.95423	5.34896	Adipose_R1_peak_1	Distal Interger	1	1.05E+08	1.05E+08	226663	1	23836	uc007cgc.1	-1153737	ENSMUSG000	Cdh20	cadherin 20
chr1	103679797	103679927	131	132	103679862	20	7.74501	3.7745	4.70467	Adipose_R1_peak_1	Distal Interger	1	1.05E+08	1.05E+08	226663	1	23836	uc007cgc.1	-1088892	ENSMUSG000	Cdh20	cadherin 20
chr1	103680633	103680885	253	254	103680718	37	21.59274	6.83004	18.04766	Adipose_R1_peak_1	Distal Interger	1	1.05E+08	1.05E+08	226663	1	23836	uc007cgc.1	-1087934	ENSMUSG000	Cdh20	cadherin 20
chr1	103680656	103680881	226	227	103680711	38	11.72962	3.59649	8.4047	Adipose_R2_peak_1	Distal Interger	1	1.05E+08	1.05E+08	226663	1	23836	uc007cgc.1	-1087938	ENSMUSG000	Cdh20	cadherin 20
chr1	103713092	103713364	273	274	103713217	52	36.63102	9.52611	32.81188	Adipose_R1_peak_1	Distal Interger	1	1.05E+08	1.05E+08	226663	1	23836	uc007cgc.1	-1055455	ENSMUSG000	Cdh20	cadherin 20
chr1	103713093	103713365	273	274	103713220	59	26.5291	5.53306	22.7188	Adipose_R2_peak_1	Distal Interger	1	1.05E+08	1.05E+08	226663	1	23836	uc007cgc.1	-1055454	ENSMUSG000	Cdh20	cadherin 20
chr1	103771205	103771459	255	256	103771332	62	42.6747	9.58475	38.7786	Adipose_R1_peak_1	Distal Interger	1	1.05E+08	1.05E+08	226663	1	23836	uc007cgc.1	-997360	ENSMUSG000	Cdh20	cadherin 20
chr1	103771213	103771450	238	239	103771333	54	22.67101	5.07198	18.94758	Adipose_R2_peak_1	Distal Interger	1	1.05E+08	1.05E+08	226663	1	23836	uc007cgc.1	-997369	ENSMUSG000	Cdh20	cadherin 20
chr1	103893117	103893375	259	260	103893231	81	70.57077	14.73851	66.40193	Adipose_R1_peak_1	Distal Interger	1	1.05E+08	1.05E+08	226663	1	23836	uc007cgc.1	-875444	ENSMUSG000	Cdh20	cadherin 20
chr1	103924597	103924846	250	251	103924721	49	33.45876	8.98689	29.68925	Adipose_R1_peak_1	Distal Interger	1	1.05E+08	1.05E+08	226663	1	23836	uc007cgc.1	-843973	ENSMUSG000	Cdh20	cadherin 20
chr1	103924598	103924778	181	182	103924757	50	19.72356	4.7031	16.08069	Adipose_R2_peak_1	Distal Interger	1	1.05E+08	1.05E+08	226663	1	23836	uc007cgc.1	-844041	ENSMUSG000	Cdh20	cadherin 20
chr1	104203558	104203807	250	251	104203674	37	21.59274	6.83004	18.04766	Adipose_R1_peak_1	Distal Interger	1	1.05E+08	1.05E+08	226663	1	23836	uc007cgc.1	-565012	ENSMUSG000	Cdh20	cadherin 20
chr1	104203588	104203764	177	178	104203685	33	8.83596	3.1354	5.68652	Adipose_R2_peak_1	Distal Interger	1	1.05E+08	1.05E+08	226663	1	23836	uc007cgc.1	-565055	ENSMUSG000	Cdh20	cadherin 20
chr1	104271658	104272056	399	400	104271915	152	142.5876	19.01063	138.0964	Adipose_R1_peak_1	Distal Interger	1	1.05E+08	1.05E+08	226663	1	23836	uc007cgc.1	-496763	ENSMUSG000	Cdh20	cadherin 20
chr1	104271682	104272044	363	364	104271904	133	97.10199	11.87529	92.60667	Adipose_R2_peak_1	Distal Interger	1	1.05E+08	1.05E+08	226663	1	23836	uc007cgc.1	-496775	ENSMUSG000	Cdh20	cadherin 20
chr1	104293736	104293890	155	156	104293789	36	10.5378	3.41206	7.27949	Adipose_R2_peak_1	Distal Interger	1	1.05E+08	1.05E+08	226663	1	23836	uc007cgc.1	-474929	ENSMUSG000	Cdh20	cadherin 20
chr1	104345478	104345731	254	255	104345600	33	17.97508	6.11109	14.52164	Adipose_R1_peak_1	Distal Interger	1	1.05E+08	1.05E+08	226663	1	23836	uc007cgc.1	-423088	ENSMUSG000	Cdh20	cadherin 20
chr1	104385425	104385664	240	241	104385550	44	28.34544	8.08821	24.66239	Adipose_R1_peak_1	Distal Interger	1	1.05E+08	1.05E+08	226663	1	23836	uc007cgc.1	-383155	ENSMUSG000	Cdh20	cadherin 20
chr1	104385427	104385645	219	220	104385577	42	14.2409	3.96536	10.79479	Adipose_R2_peak_1	Distal Interger	1	1.05E+08	1.05E+08	226663	1	23836	uc007cgc.1	-383174	ENSMUSG000	Cdh20	cadherin 20
chr1	104509846	104510039	194	195	104509999	32	8.29282	3.04319	5.18352	Adipose_R2_peak_1	Distal Interger	1	1.05E+08	1.05E+08	226663	1	23836	uc007cgc.1	-258780	ENSMUSG000	Cdh20	cadherin 20
chr1	104534778	104534927	150	151	104534805	16	5.20017	3.05554	2.38734	Adipose_R1_peak_1	Distal Interger	1	1.05E+08	1.05E+08	226663	1	23836	uc007cgc.1	-233892	ENSMUSG000	Cdh20	cadherin 20
chr1	104965633	104965862	230	231	104965738	31	16.23981	5.75161	12.83549	Adipose_R1_peak_1	Intron (uc007c	1	1.05E+08	1.05E+08	226663	1	23836	uc007cgc.1	196814	ENSMUSG000	Cdh20	cadherin 20
chr1	105014280	105014552	273	274	105014417	50	21.65698	5.17707	18.11176	Adipose_R1_peak_1	Distal Interger	1	1.05E+08	1.05E+08	226663	1	23836	uc007cgc.1	245461	ENSMUSG000	Cdh20	cadherin 20
chr1	105014325	105014555	231	232	105014437	63	28.40722	5.60369	24.56317	Adipose_R2_peak_1	Distal Interger	1	1.05E+08	1.05E+08	226663	1	23836	uc007cgc.1	245506	ENSMUSG000	Cdh20	cadherin 20
chr1	105019203	105019395	193	194	105019327	31	12.1794	4.28125	8.9167	Adipose_R1_peak_1	Distal Interger	1	1.05E+08	1.05E+08	226663	1	23836	uc007cgc.1	250384	ENSMUSG000	Cdh20	cadherin 20
chr1	105019271	105019413	143	144	105019348	34	8.21596	2.92409	5.13058	Adipose_R2_peak_1	Distal Interger	1	1.05E+08	1.05E+08	226663	1	23836	uc007cgc.1	250452	ENSMUSG000	Cdh20	cadherin 20
chr1	105019614	105019745	132	133	105019677	23	9.86443	4.31371	6.70247	Adipose_R1_peak_1	Distal Interger	1	1.05E+08	1.05E+08	226663	1	23836	uc007cgc.1	250795	ENSMUSG000	Cdh20	cadherin 20
chr1	105043246	105043467	222	223	105043358	40	23.50992	7.02615	19.92289	Adipose_R1_peak_1	Distal Interger	1	1.05E+08	1.05E+08	226663	1	23836	uc007cgc.1	274427	ENSMUSG000	Cdh20	cadherin 20
chr1	105043267	105043447	181	182	105043376	35	9.95877	3.31984	6.73562	Adipose_R2_peak_1	Distal Interger	1	1.05E+08	1.05E+08	226663	1	23836	uc007cgc.1	274448	ENSMUSG000	Cdh20	cadherin 20
chr1	105047746	105047916	171	172	105047845	26	12.14079	4.85292	8.87838	Adipose_R1_peak_1	Distal Interger	1	1.05E+08	1.05E+08	226663	1	23836	uc007cgc.1	278927	ENSMUSG000	Cdh20	cadherin 20
chr1	105047760	105047910	151	152	105047854	31	7.7623	2.95097	4.69388	Adipose_R2_peak_1	Distal Interger	1	1.05E+08	1.05E+08	226663	1	23836	uc007cgc.1	278941	ENSMUSG000	Cdh20	cadherin 20
chr1	105389659	105389793	135	136	105389737	35	9.61173	3.22962	6.4172	Adipose_R2_peak_1	Distal Interger	1	1.05E+08	1.05E+08	79794	2	320311	uc011wpy.1	-32949	ENSMUSG000	Rnf152	ring finger protein 152
chr1	105427063	105427248	186	187	105427125	25	11.36566	4.67319	8.13482	Adipose_R1_peak_1	Distal Interger	1	1.05E+08	1.05E+08	79794	2	320311	uc011wpy.1	-70353	ENSMUSG000	Rnf152	ring finger protein 152
chr1	105474794	105474937	144	145	105474857	19	7.07687	3.59476	4.08458	Adipose_R1_peak_1	Distal Interger	1	1.06E+08	1.06E+08	50744	2	27392	uc011wqa.2	94228	ENSMUSG000	Pign	phosphatidylinositol glycan anchor biosynthesis, class N
chr1	105536508	105536638	131	132	105536569	22	9.13959	4.13397	6.01526	Adipose_R1_peak_1	Intron (uc011v	1	1.06E+08	1.06E+08	50744	2	27392	uc011wqa.2	32527	ENSMUSG000	Pign	phosphatidylinositol glycan anchor biosynthesis, class N
chr1	105606516	105606783	268	269	105606653	53	37.70483	9.70585	33.87	Adipose_R1_peak_1	Intron (uc007c	1	1.06E+08	1.06E+08	50744	2	27392	uc011wqa.2	-37351	ENSMUSG000	Pign	phosphatidylinositol glycan anchor biosynthesis, class N
chr1	105606522	105606774	253	254	105606651	59	26.5291	5.53306	22.7188	Adipose_R2_peak_1	Intron (uc007c	1	1.06E+08	1.06E+08	50744	2	27392	uc011wqa.2	-37357	ENSMUSG000	Pign	phosphatidylinositol glycan anchor biosynthesis, class N
chr1	105611728	105611896	169	170	105611773	31	7.7623	2.95097	4.69388	Adipose_R2_peak_1	Intron (uc007c	1	1.06E+08	1.06E+08	50744	2	27392	uc011wqa.2	-42563	ENSMUSG000	Pign	phosphatidylinositol glycan anchor biosynthesis, class N
chr1	105611749	105611927	179	180	105611812	16	5.20017	3.05554	2.38734	Adipose_R1_peak_1	Intron (uc007c	1	1.06E+08	1.06E+08	50744	2	27392	uc011wqa.2	-42584	ENSMUSG000	Pign	phosphatidylinositol glycan anchor biosynthesis, class N
chr1	105897651	105897839	189	190	105897749	22	9.13959	4.13397	6.01526	Adipose_R1_peak_1	Distal Interger	1	1.06E+08	1.06E+08	43674	1	227449	uc007cgq.3	-92567	ENSMUSG000	Zcchc2	zinc finger, CCHC domain containing 2
chr1	105935118	105935261	144	145	105935193	20	7.74501	3.7745	4.70467	Adipose_R1_peak_1	Distal Interger	1	1.06E+08	1.06E+08	43674	1	227449	uc007cgq.3	-55145	ENSMUSG000	Zcchc2	zinc finger, CCHC domain containing 2
chr1	105973266	105973435	170	171	105973326	18	6.42934	3.41502	3.48999	Adipose_R1_peak_1	Distal Interger	1	1.06E+08	1.06E+08	43674	1	227449	uc007cgq.3	-16971	ENSMUSG000	Zcchc2	zinc finger, CCHC domain containing 2
chr1	105988446	105988555	110	111	105988495	28	6.25023	2.67431	3.31626	Adipose_R2_peak_1	Promoter (1-2	1	1.06E+08	1.06E+08	43674	1	227449	uc007cgq.3	-1851	ENSMUSG000	Zcchc2	zinc finger, CCHC domain containing 2
chr1	106080138	106080245	108	109	106080185	33	8.83596	3.1354	5.68652	Adipose_R2_peak_1	Distal Interger	1	1.06E+08	1.06E+08	28065	1	227449	uc007cgs.3	74123	ENSMUSG000	Zcchc2	zinc finger, CCHC domain containing 2
chr1	106294045	106294240	196	197	106294146	26	12.14079	4.85292	8.87838	Adipose_R1_peak_1	Intron (uc007c	1	1.06E+08	1.06E+08	115538	1	98432	uc007cgu.2	122176	ENSMUSG000	Phlpp1	PH domain and leucine rich repeat protein phosphatase 1
chr1	106294088	106294194	107	108	106294135	30	7.24477	2.85875	4.21917	Adipose_R2_peak_1	Intron (uc007c	1	1.06E+08	1.06E+08	115538	1	98432	uc007cgu.2	122219	ENSMUSG000	Phlpp1	PH domain and leucine rich repeat protein phosphatase 1
chr1	106298970	106299188	219	220	106299079	40	24.42476	7.36925	20.81738	Adipose_R1_peak_1	Intron (uc007c	1	1.06E+08	1.06E+08	115538	1	98432	uc007cgu.2	127101	ENSMUSG000	Phlpp1	PH domain and leucine rich repeat protein phosphatase 1
chr1	106923169	106923333	165	166	106923262	19	6.91816	3.52644	3.97188	Adipose_R1_peak_1	Distal Interger	1	1.07E+08	1.07E+08	22632	1	71869	uc007chc.2	-11116	ENSMUSG000	Serpinb12	serine (or cysteine) peptidase inhibitor, clade B (ovalbumin), member 12
chr1	106923234	106923344	111	112	106923281	32	8.29282	3.04319	5.18352	Adipose_R2_peak_1	Distal Interger	1	1.07E+08	1.07E+08	22632	1	71869	uc007chc.2	-11105	ENSMUSG000	Serpinb12	serine (or cysteine) peptidase inhibitor, clade B (ovalbumin), member 12
chr1	107263006	107263236	231	232	107263111	42	14.2409	3.96536	10.79479	Adipose_R2_peak_1	Distal Interger	1	1.07E+08	1.07E+08	7171	2	381286	uc007chk.2	15135	ENSMUSG000	Serpinb3c	serine (or cysteine) peptidase inhibitor, clade B, member 3C
chr1	107263018	107263236	219	220	107263194	25	11.36566	4.67319	8.13482	Adipose_R1_peak_1	Distal Interger	1	1.07E+08	1.07E+08	7171	2	381286	uc007chk.2	15135	ENSMUSG000	Serpinb3c	serine (or cysteine) peptidase inhibitor, clade B, member 3C
chr1	107315807	107315975	169	170	107315899	16	5.20017	3.05554	2.38734	Adipose_R1_peak_1	Distal Interger	1	1.07E+08	1.07E+08	7171	2	381286	uc007chk.2	-37436	ENSMUSG000	Serpinb3c	serine (or cysteine) peptidase inhibitor, clade B, member 3C

chr1	107396725	107396944	220	221	107396839	34	15.51782	5.07191	12.1393	Adipose_R1_peak_1	Distal Interger	1	1.07E+08	1.07E+08	30001	1	116872	uc007chm.2	-25745	ENSMUSG000	Serpinb7	serine (or cysteine) peptidase inhibitor, clade B, member 7
chr1	107396753	107396991	239	240	107396867	44	15.55653	4.1498	12.05625	Adipose_R2_peak_1	Distal Interger	1	1.07E+08	1.07E+08	30001	1	116872	uc007chm.2	-25698	ENSMUSG000	Serpinb7	serine (or cysteine) peptidase inhibitor, clade B, member 7
chr1	107754608	107754948	341	342	107754802	45	26.58351	7.27034	22.93646	Adipose_R1_peak_1	Distal Interger	1	1.08E+08	1.08E+08	33689	1	319596	uc007chu.1	-162311	ENSMUSG000	D830032E09R	RIKEN cDNA D830032E09 gene
chr1	107754692	107754894	203	204	107754780	42	14.2409	3.96536	10.79479	Adipose_R2_peak_1	Distal Interger	1	1.08E+08	1.08E+08	33689	1	319596	uc007chu.1	-162365	ENSMUSG000	D830032E09R	RIKEN cDNA D830032E09 gene
chr1	107823469	107823768	300	301	107823559	27	12.93148	5.03266	9.63827	Adipose_R1_peak_1	Distal Interger	1	1.08E+08	1.08E+08	33689	1	319596	uc007chu.1	-93491	ENSMUSG000	D830032E09R	RIKEN cDNA D830032E09 gene
chr1	107882521	107882727	207	208	107882620	33	17.97508	6.11109	14.52164	Adipose_R1_peak_1	Distal Interger	1	1.08E+08	1.08E+08	33689	1	319596	uc007chu.1	-34532	ENSMUSG000	D830032E09R	RIKEN cDNA D830032E09 gene
chr1	107882988	107883251	264	265	107883126	56	40.97333	10.24506	37.09402	Adipose_R1_peak_1	Distal Interger	1	1.08E+08	1.08E+08	33689	1	319596	uc007chu.1	-34008	ENSMUSG000	D830032E09R	RIKEN cDNA D830032E09 gene
chr1	107883023	107883235	213	214	107883119	49	19.00719	4.61089	15.38598	Adipose_R2_peak_1	Distal Interger	1	1.08E+08	1.08E+08	33689	1	319596	uc007chu.1	-34024	ENSMUSG000	D830032E09R	RIKEN cDNA D830032E09 gene
chr1	108139238	108139511	274	275	108139398	34	18.86162	6.29083	15.38482	Adipose_R1_peak_1	Distal Interger	1	1.08E+08	1.08E+08	33689	1	319596	uc007chu.1	221979	ENSMUSG000	D830032E09R	RIKEN cDNA D830032E09 gene
chr1	108139322	108139548	227	228	108139419	48	18.29934	4.51867	14.70076	Adipose_R2_peak_1	Distal Interger	1	1.08E+08	1.08E+08	33689	1	319596	uc007chu.1	222063	ENSMUSG000	D830032E09R	RIKEN cDNA D830032E09 gene
chr1	108291091	108291332	242	243	108291241	27	12.93148	5.03266	9.63827	Adipose_R1_peak_1	Distal Interger	1	1.08E+08	1.08E+08	33689	1	319596	uc007chu.1	373832	ENSMUSG000	D830032E09R	RIKEN cDNA D830032E09 gene
chr1	108327746	108327898	153	154	108327811	18	6.42934	3.41502	3.48999	Adipose_R1_peak_1	Distal Interger	1	1.08E+08	1.08E+08	33689	1	319596	uc007chu.1	410487	ENSMUSG000	D830032E09R	RIKEN cDNA D830032E09 gene
chr1	108413854	108414071	218	219	108413960	32	17.10103	5.93135	13.67137	Adipose_R1_peak_1	Distal Interger	1	1.08E+08	1.08E+08	33689	1	319596	uc007chu.1	496595	ENSMUSG000	D830032E09R	RIKEN cDNA D830032E09 gene
chr1	108461127	108461276	150	151	108461199	21	8.43286	3.95423	5.34896	Adipose_R1_peak_1	Distal Interger	1	1.08E+08	1.08E+08	33689	1	319596	uc007chu.1	543868	ENSMUSG000	D830032E09R	RIKEN cDNA D830032E09 gene
chr1	108712653	108712932	280	281	108712654	23	9.86443	4.31371	6.70247	Adipose_R1_peak_1	Distal Interger	1	1.08E+08	1.08E+08	33689	1	319596	uc007chu.1	795394	ENSMUSG000	D830032E09R	RIKEN cDNA D830032E09 gene
chr1	108782183	108782509	327	328	108782316	73	60.68511	13.3006	56.59814	Adipose_R1_peak_1	Distal Interger	1	1.08E+08	1.08E+08	33689	1	319596	uc007chu.1	864924	ENSMUSG000	D830032E09R	RIKEN cDNA D830032E09 gene
chr1	108782232	108782419	188	189	108782323	44	15.55653	4.1498	12.05625	Adipose_R2_peak_1	Distal Interger	1	1.08E+08	1.08E+08	33689	1	319596	uc007chu.1	864973	ENSMUSG000	D830032E09R	RIKEN cDNA D830032E09 gene
chr1	108784598	108784805	208	209	108784720	34	18.86162	6.29083	15.38482	Adipose_R1_peak_1	Distal Interger	1	1.08E+08	1.08E+08	33689	1	319596	uc007chu.1	867339	ENSMUSG000	D830032E09R	RIKEN cDNA D830032E09 gene
chr1	108846434	108846687	254	255	108846573	34	18.86162	6.29083	15.38482	Adipose_R1_peak_1	Distal Interger	1	1.08E+08	1.08E+08	33689	1	319596	uc007chu.1	929175	ENSMUSG000	D830032E09R	RIKEN cDNA D830032E09 gene
chr1	108846503	108846639	137	138	108846567	31	7.7623	2.95097	4.69388	Adipose_R2_peak_1	Distal Interger	1	1.08E+08	1.08E+08	33689	1	319596	uc007chu.1	929244	ENSMUSG000	D830032E09R	RIKEN cDNA D830032E09 gene
chr1	108956236	108956403	168	169	108956275	16	5.20017	3.05554	2.38734	Adipose_R1_peak_1	Distal Interger	1	1.10E+08	1.10E+08	83471	1	241201	uc007chv.1	-1025924	ENSMUSG000	Cdh7	cadherin 7, type 2
chr1	109035139	109035324	186	187	109035203	20	7.74501	3.7745	4.70467	Adipose_R1_peak_1	Distal Interger	1	1.10E+08	1.10E+08	83471	1	241201	uc007chv.1	-947003	ENSMUSG000	Cdh7	cadherin 7, type 2
chr1	109037145	109037380	236	237	109037275	33	17.97508	6.11109	14.52164	Adipose_R1_peak_1	Distal Interger	1	1.10E+08	1.10E+08	83471	1	241201	uc007chv.1	-944947	ENSMUSG000	Cdh7	cadherin 7, type 2
chr1	109037206	109037349	144	145	109037262	32	8.29282	3.04319	5.18352	Adipose_R2_peak_1	Distal Interger	1	1.10E+08	1.10E+08	83471	1	241201	uc007chv.1	-944978	ENSMUSG000	Cdh7	cadherin 7, type 2
chr1	109039188	109039503	316	317	109039355	107	104.8797	19.41169	100.5245	Adipose_R1_peak_1	Distal Interger	1	1.10E+08	1.10E+08	83471	1	241201	uc007chv.1	-942824	ENSMUSG000	Cdh7	cadherin 7, type 2
chr1	109039201	109039470	270	271	109039360	92	55.92586	8.57625	51.70821	Adipose_R2_peak_1	Distal Interger	1	1.10E+08	1.10E+08	83471	1	241201	uc007chv.1	-942857	ENSMUSG000	Cdh7	cadherin 7, type 2
chr1	109040893	109041097	205	206	109040969	27	12.93148	5.03266	9.63827	Adipose_R1_peak_1	Distal Interger	1	1.10E+08	1.10E+08	83471	1	241201	uc007chv.1	-941230	ENSMUSG000	Cdh7	cadherin 7, type 2
chr1	109069363	109069639	277	278	109069489	58	43.19021	10.60454	39.28351	Adipose_R1_peak_1	Distal Interger	1	1.10E+08	1.10E+08	83471	1	241201	uc007chv.1	-912688	ENSMUSG000	Cdh7	cadherin 7, type 2
chr1	109069425	109069636	212	213	109069508	46	16.90986	4.33423	13.35898	Adipose_R2_peak_1	Distal Interger	1	1.10E+08	1.10E+08	83471	1	241201	uc007chv.1	-912691	ENSMUSG000	Cdh7	cadherin 7, type 2
chr1	109071611	109071797	187	188	109071677	25	11.36566	4.67319	8.13482	Adipose_R1_peak_1	Distal Interger	1	1.10E+08	1.10E+08	83471	1	241201	uc007chv.1	-910530	ENSMUSG000	Cdh7	cadherin 7, type 2
chr1	109131251	109131526	276	277	109131398	57	34.95329	8.02371	31.16506	Adipose_R1_peak_1	Distal Interger	1	1.10E+08	1.10E+08	83471	1	241201	uc007chv.1	-850801	ENSMUSG000	Cdh7	cadherin 7, type 2
chr1	109131280	109131507	228	229	109131382	55	23.42763	5.16419	19.68558	Adipose_R2_peak_1	Distal Interger	1	1.10E+08	1.10E+08	83471	1	241201	uc007chv.1	-850820	ENSMUSG000	Cdh7	cadherin 7, type 2
chr1	109254236	109254505	270	271	109254371	52	36.63102	9.52611	32.81188	Adipose_R1_peak_1	Distal Interger	1	1.10E+08	1.10E+08	83471	1	241201	uc007chv.1	-727822	ENSMUSG000	Cdh7	cadherin 7, type 2
chr1	109254251	109254464	214	215	109254367	45	16.22858	4.24202	12.70278	Adipose_R2_peak_1	Distal Interger	1	1.10E+08	1.10E+08	83471	1	241201	uc007chv.1	-727863	ENSMUSG000	Cdh7	cadherin 7, type 2
chr1	109335623	109336009	387	388	109335873	101	96.69529	18.33327	92.3773	Adipose_R1_peak_1	Distal Interger	1	1.10E+08	1.10E+08	83471	1	241201	uc007chv.1	-646318	ENSMUSG000	Cdh7	cadherin 7, type 2
chr1	109335739	109336008	270	271	109335868	97	60.87369	9.03734	56.60858	Adipose_R2_peak_1	Distal Interger	1	1.10E+08	1.10E+08	83471	1	241201	uc007chv.1	-646319	ENSMUSG000	Cdh7	cadherin 7, type 2
chr1	109343752	109344082	331	332	109343939	55	39.87613	10.06532	36.01168	Adipose_R1_peak_1	Distal Interger	1	1.10E+08	1.10E+08	83471	1	241201	uc007chv.1	-638245	ENSMUSG000	Cdh7	cadherin 7, type 2
chr1	109343824	109344030	207	208	109343952	41	13.59774	3.87315	10.1803	Adipose_R2_peak_1	Distal Interger	1	1.10E+08	1.10E+08	83471	1	241201	uc007chv.1	-638297	ENSMUSG000	Cdh7	cadherin 7, type 2
chr1	109407506	109407734	229	230	109407594	33	17.97508	6.11109	14.52164	Adipose_R1_peak_1	Distal Interger	1	1.10E+08	1.10E+08	83471	1	241201	uc007chv.1	-574593	ENSMUSG000	Cdh7	cadherin 7, type 2
chr1	109407533	109407701	169	170	109407604	33	8.83596	3.1354	5.68652	Adipose_R2_peak_1	Distal Interger	1	1.10E+08	1.10E+08	83471	1	241201	uc007chv.1	-574626	ENSMUSG000	Cdh7	cadherin 7, type 2
chr1	109776831	109777208	378	379	109776995	69	54.23783	11.99586	50.21558	Adipose_R1_peak_1	Distal Interger	1	1.10E+08	1.10E+08	83471	1	241201	uc007chv.1	-205119	ENSMUSG000	Cdh7	cadherin 7, type 2
chr1	109776854	109777123	270	271	109777001	61	28.12311	5.7175	24.28203	Adipose_R2_peak_1	Distal Interger	1	1.10E+08	1.10E+08	83471	1	241201	uc007chv.1	-205204	ENSMUSG000	Cdh7	cadherin 7, type 2
chr1	109877283	109877557	275	276	109877424	60	45.43621	10.96401	41.50272	Adipose_R1_peak_1	Distal Interger	1	1.10E+08	1.10E+08	83471	1	241201	uc007chv.1	-104770	ENSMUSG000	Cdh7	cadherin 7, type 2
chr1	109877313	109877509	197	198	109877418	39	12.34187	3.68871	8.98501	Adipose_R2_peak_1	Distal Interger	1	1.10E+08	1.10E+08	83471	1	241201	uc007chv.1	-104818	ENSMUSG000	Cdh7	cadherin 7, type 2
chr1	110171776	110171896	121	122	110171828	32	8.29282	3.04319	5.18352	Adipose_R2_peak_1	Distal Interger	1	1.10E+08	1.10E+08	40676	1	241201	uc011wqk.2	72548	ENSMUSG000	Cdh7	cadherin 7, type 2
chr1	110176980	110177092	113	114	110177025	29	6.74062	2.76653	3.75989	Adipose_R2_peak_1	Distal Interger	1	1.10E+08	1.10E+08	40676	1	241201	uc011wqk.2	77752	ENSMUSG000	Cdh7	cadherin 7, type 2
chr1	110331892	110332123	232	233	110331999	30	15.39182	5.57188	12.01394	Adipose_R1_peak_1	Distal Interger	1	1.10E+08	1.10E+08	40676	1	241201	uc011wqk.2	232664	ENSMUSG000	Cdh7	cadherin 7, type 2
chr1	110542023	110542265	243	244	110542138	29	14.55748	5.39214	11.20652	Adipose_R1_peak_1	Distal Interger	1	1.11E+08	1.11E+08	88178	2	227485	uc007chx.1	435107	ENSMUSG000	Cdh19	cadherin 19, type 2
chr1	110543802	110543938	137	138	110543892	16	5.20017	3.05554	2.38734	Adipose_R1_peak_1	Distal Interger	1	1.11E+08	1.11E+08	88178	2	227485	uc007chx.1	433434	ENSMUSG000	Cdh19	cadherin 19, type 2
chr1	110575498	110575758	261	262	110575618	42	26.36523	7.72873	22.71906	Adipose_R1_peak_1	Distal Interger	1	1.11E+08	1.11E+08	88178	2	227485	uc007chx.1	401614	ENSMUSG000	Cdh19	cadherin 19, type 2
chr1	110575510	110575704	195	196	110575620	39	12.34187	3.68871	8.98501	Adipose_R2_peak_1	Distal Interger	1	1.11E+08	1.11E+08	88178	2	227485	uc007chx.1	401668	ENSMUSG000	Cdh19	cadherin 19, type 2
chr1	110621544	110621868	325	326	110621742	26	12.14079	4.85292	8.87838	Adipose_R1_peak_1	Distal Interger	1	1.11E+08	1.11E+08	88178	2	227485	uc007chx.1	355504	ENSMUSG000	Cdh19	cadherin 19, type 2
chr1	110679087	110679275	189	190	110679272	47	17.60017	4.42645	14.02531	Adipose_R2_peak_1	Distal Interger	1	1.11E+08	1.11E+08	88178	2	227485	uc007chx.1	298097	ENSMUSG000	Cdh19	cadherin 19, type 2
chr1	110679105	110679311	207	208	110679223	31	16.23981	5.75161	12.83549	Adipose_R1_peak_1	Distal Interger	1	1.11E+08	1.11E+08	88178	2	227485	uc007chx.1	298061	ENSMUSG000	Cdh19	cadherin 19, type 2
chr1	110694024	110694238	215	216	110694133	39	23.47001	7.18952	19.88325	Adipose_R1_peak_1	Distal Interger	1	1.11E+08	1.11E+08	88178	2	227485	uc007chx.1	283134	ENSMUSG000	Cdh19	cadherin 19, type 2
chr1	111104773	111104941	169	170	111104873	25	11.36566	4.67319	8.13482	Adipose_R1_peak_1	Distal Interger	1	1.11E+08	1.11E+08	88178	2	227485	uc007chx.1	-127401	ENSMUSG000	Cdh19	cadherin 19, type 2
chr1	111104813	111104914	102	103	111104865	29	6.74062	2.76653	3.75989	Adipose_R2_peak_1	Distal Interger	1	1.11E+08	1.11E+08	88178	2	227485	uc007chx.1	-127441	ENSMUSG000	Cdh19	cadherin 19, type 2
chr1	111411880	111412170	291	292	111412029	42	26.36523	7.72873	22.71906	Adipose_R1_peak_1	Distal Interger	1	1.11E+08	1.11E+08	88178	2	227485	uc007chx.1	-434508	ENSMUSG000	Cdh19	cadherin 19, type 2
chr1	111411985	111412082	98	99	111412030	27	5.77405	2.5821	2.88921	Adipose_R2_peak_1	Distal Interger	1	1.11E+08	1.11E+08	88178	2	227485	uc007chx.1	-434613	ENSMUSG000	Cdh19	cadherin 19, type 2
chr1	111433523	111433771	249	250	111433627	43	27.35048	7.90847	23.68561	Adipose_R1_peak_1	Distal Interger	1	1.12E+08	1.12E+08	6217	2	319901	uc007chy.1	431147	ENSMUSG000	Dsel	dermatan sulfate epimerase-like
chr1	111491362	111491558	197	198	111491450	24	10.60668	4.49345	7.40961	Adipose_R1_peak_1	Distal Interger	1	1.12E+08	1.12E+08	6217	2	319901	uc007chy.1	373360	ENSMUSG000	Dsel	dermatan sulfate epimerase-like
chr1	111491373	111491516	144	145	111491440	31	7.7623	2.95097	4.69388	Adipose_R2_peak_1	Distal Interger	1	1.12E+08	1.12E+08	6217	2	319901	uc007chy.1	373402	ENSMUSG000	Dsel	dermatan sulfate epimerase-like
chr1	111575469	111575695	227	228	111575587	27	12.93148	5.03266	9.63827	Adipose_R1_peak_1	Distal Interger	1	1.12E+08	1.12E+08	6217	2	319901	uc007chy.1	289223	ENSMUSG000	Dsel	dermatan sulfate epimerase-like
chr1	111890772	111890950	179	180	111890857	26	12.14079	4.85292	8.87838	Adipose_R1_peak_1	Distal Interger	1	1.12E+08	1.12E+08	6217	2	319901	uc007chy.1	-25854	ENSMUSG000	Dsel	dermatan sulfate epimerase-like
chr1	111961042	111961251	210	211	111961137	25	10.66971	4.3939	7.472	Adipose_R1_peak_1	Distal Interger	1	1.12E+08	1.12E+08	6217	2	319901	uc007chy.1	-96124	ENSMUSG000	Dsel	dermatan sulfate epimerase-like
chr1	111962378	111962687	310	311	111962518	67	53.51345	12.22218	49.49424	Adipose_R1_peak_1	Distal Interger	1	1.12E+08	1.12E+08	6217	2	319901	uc007chy.1	-97460	ENSMUSG000	Dsel	dermatan sulfate epimerase-like
chr1	111962400	111962668	269	270	111962521	66	32.22715	6.17859	28.31407	Adipose_R2_peak_1	Distal Interger	1	1.12E+08	1.12E+08	6217	2	319901	uc007chy.1	-97482	ENSMUSG000	Dsel	dermatan sulfate epimerase-like
chr1	111963029	111963183	155	156	111963163	28	6.25023	2.67431	3.31626	Adipose_R2_peak_1	Distal Interger	1	1.12E+08	1.12E+08	6217	2	319901	uc007chy.1	-98111	ENSMUSG000	Dsel	dermatan sulfate epimerase-like
chr1	112027694	112027798	105	106	112027739	27	5.77405	2.5821	2.88921	Adipose_R2_peak_1	Distal Interger	1	1.12E+08	1.12E+08	6217	2	319901	uc007chy.1	-162776	ENSMUSG000	Dsel	dermatan sulfate epimerase-like
chr1	112137293	112137484	192	193	112137409	31	16.23981	5.75161	12.83549	Adipose_R1_peak_1	Distal Interger	1	1.12E+08	1.12E+08	6217	2	319901	uc007chy.1	-272375	ENSMUSG000	Dsel	dermatan sulfate epimerase-like
chr1	112137310	112137484	175	176	112137416	37	11.12817	3.50427	7.83613	Adipose_R2_peak_1	Distal Interger	1	1.12E+08	1.12E+08	6217	2	319901	uc007chy.1	-272392	ENSMUSG000	Dsel	dermatan sulfate epimerase-like
chr1	112218121	112218370	250	251	112218240	37	21.59274	6.83004	18.04766	Adipose_R1_peak_1	Distal Interger	1	1.12E+08	1.12E+08	6217	2	319901	uc007chy.1	-353203	ENSMUSG000	Dsel	dermatan sulfate epimerase-like
chr1	112218176	112218288	113	114	112218242	29	6.74062	2.76653	3.75989	Adipose_R2_peak_1	Distal Interger	1	1.12E+08	1.12E+08	6217	2	319901	uc007chy.1	-353258	ENSMUSG000	Dsel	dermatan sulfate epimerase-like
chr1	112219014	112219249	236	237	112219138	29	14.55748	5.39214	11.20652	Adipose_R1_peak_1	Distal Interger	1	1.12E+08	1.12E+08	6217	2	319901	uc007chy.1	-354096	ENSMUSG000	Dsel	dermatan sulfate epimerase-like
chr1	112219056	112219184	129	130	112219150	29	6.74062	2.76653	3.75989	Adipose_R2_peak_1	Distal Interger	1	1.12E+08	1.12E+08	6217	2	319901	uc007chy.1	-354138	ENSMUSG000	Dsel	dermatan sulfate epimerase-like
chr1	112234024	112234247	224	225	112234073	30	5.82459	2.47643	2.93775	Adipose_R2_peak_1	Distal Interger	1	1.12E+08	1.12E+08	6217	2	319901	uc007chy.1	-369106	ENSMUSG000	Dsel	dermatan sulfate epimerase-like
chr1	112268720	112268855	136	137	112268725	17	5.80341	3.23528	2.92378	Adipose_R1_peak_1	Distal Interger	1	1.12E+08	1.12E+08	6217	2	319901	uc007chy.1	-403802	ENSMUSG000	Dsel	dermatan sulfate epimerase-like
chr1	112533538	112533742	205	206	112533644	31	16.23981	5.75161	12.83549	Adipose_R1_peak_1	Distal Interger	1	1.12E+08	1.12E+08	6217	2	319901	uc007chy.1	-668620	ENSMUSG000	Dsel	dermatan sulfate epimerase-like
chr1	112533571	112533686	116	117	112533618	29	6.74062	2.76653	3.75989	Adipose_R2_peak_1	Distal Interger	1	1.12E+08	1.12E+08	6217	2	319901	uc007chy.1	-668653	ENSMUSG000	Dsel	dermatan sulfate epimerase-like
chr1	112536741	112536913	173	174	112536824	25	11.36566	4.67319	8.13482	Adipose_R1_peak_1	Distal Interger	1	1.12E+08	1.12E+08	6217	2	319901	uc007chy.1	-671823	ENSMUSG000	Dsel	dermatan sulfate epimerase-like
chr1	112553879	112554203	325	326	112554061	87	70.48232	13.22335	66.3181	Adipose_R1_peak_1	Distal Interger	1	1.12E+08	1.12E+08	6217	2	319901	uc007chy.1	-688961	ENSMUSG000	Dsel	dermatan sulfate epimerase-like
chr1	112553919	112554200	282	283	112554061	86	50.14197	8.02294	45.98602	Adipose_R2_peak_1	Distal Interger	1	1.12E+08	1.12E+08	6217	2	319901	uc007chy.1	-689001	ENSMUSG000	Dsel	dermatan sulfate epimerase-like
chr1	112555918	112556210	293	294	112556072	53	37.70483	9.70585	33.87	Adipose_R1_peak_1	Distal Interger	1	1.12E+08	1.12E+08	6217	2	319901	uc007chy.1	-691000	ENSMUSG000	Dsel	dermatan sulfate epimerase-like
chr1	112555951	112556161	211	212	112556054	44	15.55653	4.1498	12.05625	Adipose_R2_peak_1	Distal Interger	1	1.12E+08	1.12E+08	6217	2	319901	uc007chy.1	-691033	ENSMUSG000	Dsel	dermatan sulfate epimerase-like
chr1	112654109	112654255	147	148	112654192	22	9.13959	4.13397	6.01526	Adipose_R1_peak_1	Distal Interger	1	1.12E+08	1.12E+08	6217	2	319901	uc007chy.1	-789191	ENSMUSG000	Dsel	dermatan sulfate epimerase-like
chr1	112658007	112658263	257	258	112658123	50	19.72356	4.7031	16.08069	Adipose_R2_peak_1	Distal Interger	1	1.12E+08	1.12E+08	6217	2	319901	uc007chy.1	-793089	ENSMUSG000	Dsel	dermatan sulfate epimerase-like
chr1	112658018	112658294	277	278	112658141	46	30.36364	8.44768	26.64488	Adipose_R1_peak_1	Distal Interger	1	1.12E+08	1.12E+08	6217	2	319901	uc007chy.1	-793100	ENSMUSG000	Dsel	dermatan sulfate epimerase-like
chr1	112739773	112740030	258	259	112739887	62	47.71039	11.32349	43.751	Adipose_R1_peak_1	Distal Interger	1	1.12E+08	1.12E+08	6217	2	319901	uc007chy.1	-874855	ENSMUSG000	Dsel	dermatan sulfate epimerase-like
chr1	112739793	112740018	226	227	112739884	51	20.44827	4.79532	16.78434	Adipose_R2_peak_1	Distal Interger	1	1.12E+08	1.12E+08	6217	2	319901	uc007chy.1	-874875	ENSMUSG000	Dsel	dermatan sulfate epimerase-like
chr1	112820073	112820248	176	177	112820172	19	7.07687	3.59476	4.08458	Adipose_R1_peak_1	Distal Interger	1	1.12E+08	1.12E+08	6217	2	319901	uc007chy.1	-955155	ENSMUSG000	Dsel	dermatan sulfate epimerase-like
chr1	112821319	112821496	178	179	112821428	20	7.74501	3.7745	4.70467	Adipose_R1_peak_1	Distal Interger	1	1.12E+08	1.12E+08	6217	2	319901	uc007chy.1	-956401	ENSMUSG000	Dsel	dermatan sulfate epimerase-like
chr1	112865299	112865511	213	214	112865404	28	13.73721	5.2124	10.41463	Adipose_R1_peak_1	Distal Interger	1	1.12E+08	1.12E+08	6217	2	319901	uc007chy.1	-1000381	ENSMUSG000	Dsel	dermatan sulfate epimerase-like
chr1	113051883	113052111	229	230	113052020	28	13.73721	5.2124	10.41463	Adipose_R1_peak_1	Distal Interger	1	1.12E+08	1.12E+08	6217	2	319901	uc007chy.1	-1186965	ENSMUSG000	Dsel	dermatan sulfate epimerase-like
chr1	113053100	113053296	197	198	113053186	23	9.86443	4.31371	6.70247	Adipose_R1_peak_1	Distal Interger	1	1.12E+08	1.12E+08	6217	2	319901	uc007chy.1	-1188182	ENSMUSG000	Dsel	dermatan sulfate epimerase-like

chr1	113053132	113053303	172	173	113053175	35	9.95877	3.31984	6.73562	Adipose_R2_peak_1	Distal Interger	1	1.12E+08	1.12E+08	6217	2	319901	uc007chy.1	-1188214	ENSMUSG000	Dsel	dermatan sulfate epimerase-like
chr1	113187863	113188157	295	296	113188006	81	64.88108	12.79445	60.7598	Adipose_R1_peak_1	Distal Interger	1	1.12E+08	1.12E+08	6217	2	319901	uc007chy.1	-1322945	ENSMUSG000	Dsel	dermatan sulfate epimerase-like
chr1	113187871	113188134	264	265	113188004	70	35.62622	6.54746	31.65691	Adipose_R2_peak_1	Distal Interger	1	1.12E+08	1.12E+08	6217	2	319901	uc007chy.1	-1322953	ENSMUSG000	Dsel	dermatan sulfate epimerase-like
chr1	113226993	113227256	264	265	113227086	28	13.73721	5.2124	10.41463	Adipose_R1_peak_1	Distal Interger	1	1.12E+08	1.12E+08	6217	2	319901	uc007chy.1	-1362075	ENSMUSG000	Dsel	dermatan sulfate epimerase-like
chr1	113331021	113331204	184	185	113331175	20	7.74501	3.7745	4.70467	Adipose_R1_peak_1	Distal Interger	1	1.12E+08	1.12E+08	6217	2	319901	uc007chy.1	-1466103	ENSMUSG000	Dsel	dermatan sulfate epimerase-like
chr1	113338738	113338881	144	145	113338799	39	12.34187	3.68871	8.98501	Adipose_R2_peak_1	Distal Interger	1	1.12E+08	1.12E+08	6217	2	319901	uc007chy.1	-1473820	ENSMUSG000	Dsel	dermatan sulfate epimerase-like
chr1	113366884	113367080	197	198	113366990	36	20.67077	6.6503	17.14779	Adipose_R1_peak_1	Distal Interger	1	1.12E+08	1.12E+08	6217	2	319901	uc007chy.1	-1501966	ENSMUSG000	Dsel	dermatan sulfate epimerase-like
chr1	113366887	113367049	163	164	113366985	30	7.24477	2.85875	4.21917	Adipose_R2_peak_1	Distal Interger	1	1.12E+08	1.12E+08	6217	2	319901	uc007chy.1	-1501969	ENSMUSG000	Dsel	dermatan sulfate epimerase-like
chr1	113368365	113368557	193	194	113368455	29	14.55748	5.39214	11.20652	Adipose_R1_peak_1	Distal Interger	1	1.12E+08	1.12E+08	6217	2	319901	uc007chy.1	-1503447	ENSMUSG000	Dsel	dermatan sulfate epimerase-like
chr1	113403095	113403281	187	188	113403182	26	12.14079	4.85292	8.87838	Adipose_R1_peak_1	Distal Interger	1	1.12E+08	1.12E+08	6217	2	319901	uc007chy.1	-1538177	ENSMUSG000	Dsel	dermatan sulfate epimerase-like
chr1	113603642	113604115	474	475	113603854	225	247.9493	28.36995	243.249	Adipose_R1_peak_1	Distal Interger	1	1.12E+08	1.12E+08	6217	2	319901	uc007chy.1	-1738724	ENSMUSG000	Dsel	dermatan sulfate epimerase-like
chr1	113603653	113604098	446	447	113603849	189	144.1956	13.2862	139.5543	Adipose_R2_peak_1	Distal Interger	1	1.12E+08	1.12E+08	6217	2	319901	uc007chy.1	-1738735	ENSMUSG000	Dsel	dermatan sulfate epimerase-like
chr1	113612204	113612395	192	193	113612374	40	12.96465	3.78093	9.57654	Adipose_R2_peak_1	Distal Interger	1	1.12E+08	1.12E+08	6217	2	319901	uc007chy.1	-1747286	ENSMUSG000	Dsel	dermatan sulfate epimerase-like
chr1	113618381	113618511	131	132	113618445	28	13.73721	5.2124	10.41463	Adipose_R1_peak_1	Distal Interger	1	1.12E+08	1.12E+08	6217	2	319901	uc007chy.1	-1753463	ENSMUSG000	Dsel	dermatan sulfate epimerase-like
chr1	113618382	113618499	118	119	113618442	33	8.83596	3.1354	5.68652	Adipose_R2_peak_1	Distal Interger	1	1.12E+08	1.12E+08	6217	2	319901	uc007chy.1	-1753464	ENSMUSG000	Dsel	dermatan sulfate epimerase-like
chr1	113621830	113621935	106	107	113621884	26	5.31252	2.48988	2.47999	Adipose_R2_peak_1	Distal Interger	1	1.12E+08	1.12E+08	6217	2	319901	uc007chy.1	-1756912	ENSMUSG000	Dsel	dermatan sulfate epimerase-like
chr1	113744746	113745002	257	258	113744876	49	33.45876	8.98689	29.68925	Adipose_R1_peak_1	Distal Interger	1	1.12E+08	1.12E+08	6217	2	319901	uc007chy.1	-1879828	ENSMUSG000	Dsel	dermatan sulfate epimerase-like
chr1	113744751	113745011	261	262	113744881	56	24.19188	5.25641	20.43163	Adipose_R2_peak_1	Distal Interger	1	1.12E+08	1.12E+08	6217	2	319901	uc007chy.1	-1879833	ENSMUSG000	Dsel	dermatan sulfate epimerase-like
chr1	113761966	113762123	158	159	113762028	20	7.74501	3.7745	4.70467	Adipose_R1_peak_1	Distal Interger	1	1.12E+08	1.12E+08	6217	2	319901	uc007chy.1	-1897048	ENSMUSG000	Dsel	dermatan sulfate epimerase-like
chr1	113805650	113805910	261	262	113805743	80	44.53636	7.46964	40.44495	Adipose_R2_peak_1	Distal Interger	1	1.16E+08	1.17E+08	895538	1	636808	uc007cia.1	-1879227	ENSMUSG000	Cntnap5a	contactin associated protein-like 5A
chr1	113805662	113805923	262	263	113805758	74	61.90123	13.48034	57.80368	Adipose_R1_peak_1	Distal Interger	1	1.16E+08	1.17E+08	895538	1	636808	uc007cia.1	-1879214	ENSMUSG000	Cntnap5a	contactin associated protein-like 5A
chr1	113937492	113937663	172	173	113937624	31	7.7623	2.95097	4.69388	Adipose_R2_peak_1	Distal Interger	1	1.16E+08	1.17E+08	895538	1	636808	uc007cia.1	-1747474	ENSMUSG000	Cntnap5a	contactin associated protein-like 5A
chr1	114032143	114032427	285	286	114032281	63	48.85777	11.50323	44.88581	Adipose_R1_peak_1	Distal Interger	1	1.16E+08	1.17E+08	895538	1	636808	uc007cia.1	-1652710	ENSMUSG000	Cntnap5a	contactin associated protein-like 5A
chr1	114032212	114032406	195	196	114032286	51	20.44827	4.79532	16.78434	Adipose_R2_peak_1	Distal Interger	1	1.16E+08	1.17E+08	895538	1	636808	uc007cia.1	-1652731	ENSMUSG000	Cntnap5a	contactin associated protein-like 5A
chr1	114066004	114066264	261	262	114066123	41	25.38991	7.54899	21.76305	Adipose_R1_peak_1	Distal Interger	1	1.16E+08	1.17E+08	895538	1	636808	uc007cia.1	-1618873	ENSMUSG000	Cntnap5a	contactin associated protein-like 5A
chr1	114066052	114066265	214	215	114066155	42	14.2409	3.96536	10.79479	Adipose_R2_peak_1	Distal Interger	1	1.16E+08	1.17E+08	895538	1	636808	uc007cia.1	-1618872	ENSMUSG000	Cntnap5a	contactin associated protein-like 5A
chr1	114130900	114131092	193	194	114130963	24	10.60668	4.49345	7.40961	Adipose_R1_peak_1	Distal Interger	1	1.16E+08	1.17E+08	895538	1	636808	uc007cia.1	-1554045	ENSMUSG000	Cntnap5a	contactin associated protein-like 5A
chr1	114180743	114180887	145	146	114180801	36	10.5378	3.41206	7.27949	Adipose_R2_peak_1	Distal Interger	1	1.16E+08	1.17E+08	895538	1	636808	uc007cia.1	-1504250	ENSMUSG000	Cntnap5a	contactin associated protein-like 5A
chr1	114183441	114183572	132	133	114183459	17	5.80341	3.23528	2.92378	Adipose_R1_peak_1	Distal Interger	1	1.16E+08	1.17E+08	895538	1	636808	uc007cia.1	-1501565	ENSMUSG000	Cntnap5a	contactin associated protein-like 5A
chr1	114228158	114228401	244	245	114228269	30	15.39182	5.57188	12.01394	Adipose_R1_peak_1	Distal Interger	1	1.16E+08	1.17E+08	895538	1	636808	uc007cia.1	-1456736	ENSMUSG000	Cntnap5a	contactin associated protein-like 5A
chr1	114273250	114273519	270	271	114273376	54	38.78658	9.88558	34.93639	Adipose_R1_peak_1	Distal Interger	1	1.16E+08	1.17E+08	895538	1	636808	uc007cia.1	-1411618	ENSMUSG000	Cntnap5a	contactin associated protein-like 5A
chr1	114273257	114273490	234	235	114273396	45	16.22858	4.24202	12.70278	Adipose_R2_peak_1	Distal Interger	1	1.16E+08	1.17E+08	895538	1	636808	uc007cia.1	-1411647	ENSMUSG000	Cntnap5a	contactin associated protein-like 5A
chr1	114310205	114310402	198	199	114310309	25	11.36566	4.67319	8.13482	Adipose_R1_peak_1	Distal Interger	1	1.16E+08	1.17E+08	895538	1	636808	uc007cia.1	-1374735	ENSMUSG000	Cntnap5a	contactin associated protein-like 5A
chr1	114380614	114380841	228	229	114380732	75	53.97238	10.63439	49.95136	Adipose_R1_peak_1	Distal Interger	1	1.16E+08	1.17E+08	895538	1	636808	uc007cia.1	-1304296	ENSMUSG000	Cntnap5a	contactin associated protein-like 5A
chr1	114380641	114380817	177	178	114380745	45	16.22858	4.24202	12.70278	Adipose_R2_peak_1	Distal Interger	1	1.16E+08	1.17E+08	895538	1	636808	uc007cia.1	-1304320	ENSMUSG000	Cntnap5a	contactin associated protein-like 5A
chr1	114381730	114381920	191	192	114381810	26	12.14079	4.85292	8.87838	Adipose_R1_peak_1	Distal Interger	1	1.16E+08	1.17E+08	895538	1	636808	uc007cia.1	-1303217	ENSMUSG000	Cntnap5a	contactin associated protein-like 5A
chr1	114404466	114404695	230	231	114404587	29	14.55748	5.39214	11.20652	Adipose_R1_peak_1	Distal Interger	1	1.16E+08	1.17E+08	895538	1	636808	uc007cia.1	-1280442	ENSMUSG000	Cntnap5a	contactin associated protein-like 5A
chr1	114404506	114404684	179	180	114404592	37	11.12817	3.50427	7.83613	Adipose_R2_peak_1	Distal Interger	1	1.16E+08	1.17E+08	895538	1	636808	uc007cia.1	-1280453	ENSMUSG000	Cntnap5a	contactin associated protein-like 5A
chr1	114503046	114503292	247	248	114503165	41	25.38991	7.54899	21.76305	Adipose_R1_peak_1	Distal Interger	1	1.16E+08	1.17E+08	895538	1	636808	uc007cia.1	-1181845	ENSMUSG000	Cntnap5a	contactin associated protein-like 5A
chr1	114503076	114503292	217	218	114503173	43	14.8939	4.05758	11.42017	Adipose_R2_peak_1	Distal Interger	1	1.16E+08	1.17E+08	895538	1	636808	uc007cia.1	-1181845	ENSMUSG000	Cntnap5a	contactin associated protein-like 5A
chr1	114558260	114558561	302	303	114558408	63	48.85777	11.50323	44.88581	Adipose_R1_peak_1	Distal Interger	1	1.16E+08	1.17E+08	895538	1	636808	uc007cia.1	-1126576	ENSMUSG000	Cntnap5a	contactin associated protein-like 5A
chr1	114558330	114558529	200	201	114558404	41	13.59774	3.87315	10.1803	Adipose_R2_peak_1	Distal Interger	1	1.16E+08	1.17E+08	895538	1	636808	uc007cia.1	-1126608	ENSMUSG000	Cntnap5a	contactin associated protein-like 5A
chr1	114639523	114639825	303	304	114639715	17	5.80341	3.23528	2.92378	Adipose_R1_peak_1	Distal Interger	1	1.16E+08	1.17E+08	895538	1	636808	uc007cia.1	-1045312	ENSMUSG000	Cntnap5a	contactin associated protein-like 5A
chr1	114659007	114659257	251	252	114659119	35	19.76029	6.47056	16.26028	Adipose_R1_peak_1	Distal Interger	1	1.16E+08	1.17E+08	895538	1	636808	uc007cia.1	-1025880	ENSMUSG000	Cntnap5a	contactin associated protein-like 5A
chr1	114731911	114732129	219	220	114732030	31	16.23981	5.75161	12.83549	Adipose_R1_peak_1	Distal Interger	1	1.16E+08	1.17E+08	895538	1	636808	uc007cia.1	-953008	ENSMUSG000	Cntnap5a	contactin associated protein-like 5A
chr1	114770269	114770498	230	231	114770392	49	33.45876	8.98689	29.68925	Adipose_R1_peak_1	Distal Interger	1	1.16E+08	1.17E+08	895538	1	636808	uc007cia.1	-914639	ENSMUSG000	Cntnap5a	contactin associated protein-like 5A
chr1	114770329	114770460	132	133	114770400	35	9.95877	3.31984	6.73562	Adipose_R2_peak_1	Distal Interger	1	1.16E+08	1.17E+08	895538	1	636808	uc007cia.1	-914677	ENSMUSG000	Cntnap5a	contactin associated protein-like 5A
chr1	115102212	115102579	368	369	115102354	175	173.2304	21.64799	168.6593	Adipose_R1_peak_1	Distal Interger	1	1.16E+08	1.17E+08	895538	1	636808	uc007cia.1	-582558	ENSMUSG000	Cntnap5a	contactin associated protein-like 5A
chr1	115102213	115102539	327	328	115102356	179	154.7836	16.59919	150.1206	Adipose_R2_peak_1	Distal Interger	1	1.16E+08	1.17E+08	895538	1	636808	uc007cia.1	-582598	ENSMUSG000	Cntnap5a	contactin associated protein-like 5A
chr1	115105511	115105729	219	220	115105633	33	17.97508	6.11109	14.52164	Adipose_R1_peak_1	Distal Interger	1	1.16E+08	1.17E+08	895538	1	636808	uc007cia.1	-579408	ENSMUSG000	Cntnap5a	contactin associated protein-like 5A
chr1	115105521	115105696	176	177	115105621	32	8.29282	3.04319	5.18352	Adipose_R2_peak_1	Distal Interger	1	1.16E+08	1.17E+08	895538	1	636808	uc007cia.1	-579441	ENSMUSG000	Cntnap5a	contactin associated protein-like 5A
chr1	115106426	115106580	155	156	115106563	18	6.42934	3.41502	3.48999	Adipose_R1_peak_1	Distal Interger	1	1.16E+08	1.17E+08	895538	1	636808	uc007cia.1	-578557	ENSMUSG000	Cntnap5a	contactin associated protein-like 5A
chr1	115108610	115108863	254	255	115108727	36	20.67077	6.6503	17.14779	Adipose_R1_peak_1	Distal Interger	1	1.16E+08	1.17E+08	895538	1	636808	uc007cia.1	-576274	ENSMUSG000	Cntnap5a	contactin associated protein-like 5A
chr1	115108653	115108805	153	154	115108725	36	10.5378	3.41206	7.27949	Adipose_R2_peak_1	Distal Interger	1	1.16E+08	1.17E+08	895538	1	636808	uc007cia.1	-576332	ENSMUSG000	Cntnap5a	contactin associated protein-like 5A
chr1	115110292	115110447	156	157	115110318	18	6.42934	3.41502	3.48999	Adipose_R1_peak_1	Distal Interger	1	1.16E+08	1.17E+08	895538	1	636808	uc007cia.1	-574690	ENSMUSG000	Cntnap5a	contactin associated protein-like 5A
chr1	115305098	115305350	253	254	115305213	29	14.55748	5.39214	11.20652	Adipose_R1_peak_1	Distal Interger	1	1.16E+08	1.17E+08	895538	1	636808	uc007cia.1	-379787	ENSMUSG000	Cntnap5a	contactin associated protein-like 5A
chr1	115306230	115306408	179	180	115306301	23	9.86443	4.31371	6.70247	Adipose_R1_peak_1	Distal Interger	1	1.16E+08	1.17E+08	895538	1	636808	uc007cia.1	-378729	ENSMUSG000	Cntnap5a	contactin associated protein-like 5A
chr1	115306626	115306799	174	175	115306730	20	7.74501	3.7745	4.70467	Adipose_R1_peak_1	Distal Interger	1	1.16E+08	1.17E+08	895538	1	636808	uc007cia.1	-378338	ENSMUSG000	Cntnap5a	contactin associated protein-like 5A
chr1	115307752	115307907	156	157	115307874	22	9.13959	4.13397	6.01526	Adipose_R1_peak_1	Distal Interger	1	1.16E+08	1.17E+08	895538	1	636808	uc007cia.1	-377230	ENSMUSG000	Cntnap5a	contactin associated protein-like 5A
chr1	115307790	115307931	142	143	115307872	31	7.7623	2.95097	4.69388	Adipose_R2_peak_1	Distal Interger	1	1.16E+08	1.17E+08	895538	1	636808	uc007cia.1	-377206	ENSMUSG000	Cntnap5a	contactin associated protein-like 5A
chr1	115375170	115375557	388	389	115375433	47	17.60017	4.42645	14.02531	Adipose_R2_peak_1	Distal Interger	1	1.16E+08	1.17E+08	895538	1	636808	uc007cia.1	-309580	ENSMUSG000	Cntnap5a	contactin associated protein-like 5A
chr1	115375282	115375554	273	274	115375414	35	19.20331	6.25718	15.72474	Adipose_R1_peak_1	Distal Interger	1	1.16E+08	1.17E+08	895538	1	636808	uc007cia.1	-309583	ENSMUSG000	Cntnap5a	contactin associated protein-like 5A
chr1	115457742	115457908	167	168	115457841	22	9.13959	4.13397	6.01526	Adipose_R1_peak_1	Distal Interger	1	1.16E+08	1.17E+08	895538	1	636808	uc007cia.1	-227229	ENSMUSG000	Cntnap5a	contactin associated protein-like 5A
chr1	115478650	115478862	213	214	115478740	29	14.55748	5.39214	11.20652	Adipose_R1_peak_1	Distal Interger	1	1.16E+08	1.17E+08	895538	1	636808	uc007cia.1	-206275	ENSMUSG000	Cntnap5a	contactin associated protein-like 5A
chr1	115482041	115482388	348	349	115482173	44	28.34544	8.08821	24.66239	Adipose_R1_peak_1	Distal Interger	1	1.16E+08	1.17E+08	895538	1	636808	uc007cia.1	-202749	ENSMUSG000	Cntnap5a	contactin associated protein-like 5A
chr1	115482049	115482295	247	248	115482166	55	23.42763	5.16419	19.68558	Adipose_R2_peak_1	Distal Interger	1	1.16E+08	1.17E+08	895538	1	636808	uc007cia.1	-202842	ENSMUSG000	Cntnap5a	contactin associated protein-like 5A
chr1	115611818	115612005	188	189	115611878	32	17.10103	5.93135	13.67137	Adipose_R1_peak_1	Distal Interger	1	1.16E+08	1.17E+08	895538	1	636808	uc007cia.1	-73132	ENSMUSG000	Cntnap5a	contactin associated protein-like 5A
chr1	115613710	115613910	201	202	115613815	31	16.23981	5.75161	12.83549	Adipose_R1_peak_1	Distal Interger	1	1.16E+08	1.17E+08	895538	1	636808	uc007cia.1	-71227	ENSMUSG000	Cntnap5a	contactin associated protein-like 5A
chr1	115801473	115801623	151	152	115801543	23	9.86443	4.31371	6.70247	Adipose_R1_peak_1	Intron (uc007c	1	1.16E+08	1.17E+08	895538	1	636808	uc007cia.1	116336	ENSMUSG000	Cntnap5a	contactin associated protein-like 5A
chr1	115887146	115887291	146	147	115887207	21	8.43286	3.95423	5.34896	Adipose_R1_peak_1	Intron (uc007c	1	1.16E+08	1.17E+08	895538	1	636808	uc007cia.1	202009	ENSMUSG000	Cntnap5a	contactin associated protein-like 5A
chr1	115954371	115954505	135	136	115954484	16	5.20017	3.05554	2.38734	Adipose_R1_peak_1	Intron (uc007c	1	1.16E+08	1.17E+08	895538	1	636808	uc007cia.1	269234	ENSMUSG000	Cntnap5a	contactin associated protein-like 5A
chr1	116456701	116456831	131	132	116456741	27	5.77405	2.5821	2.88921	Adipose_R2_peak_1	Intron (uc007c	1	1.16E+08	1.17E+08	895538	1	636808	uc007cia.1	771564	ENSMUSG000	Cntnap5a	contactin associated protein-like 5A
chr1	116493100	116493309	210	211	116493199	39	12.34187	3.68871	8.98501	Adipose_R2_peak_1	Intron (uc007c	1	1.16E+08	1.17E+08	895538	1	636808	uc007cia.1	807963	ENSMUSG000	Cntnap5a	contactin associated protein-like 5A
chr1	116493103	116493314	212	213	116493204	44	28.34544	8.08821	24.66239	Adipose_R1_peak_1	Intron (uc007c	1	1.16E+08	1.17E+08	895538	1	636808	uc007cia.1	807966	ENSMUSG000	Cntnap5a	contactin associated protein-like 5A
chr1	116519143	116519319	177	178	116519268	45	16.22858	4.24202	12.70278	Adipose_R2_peak_1	Intron (uc007c	1	1.16E+08	1.17E+08	895538	1	636808	uc007cia.1	834006	ENSMUSG000	Cntnap5a	contactin associated protein-like 5A
chr1	116635725	116635915	191	192	116635825	20	7.74501	3.7745	4.70467	Adipose_R1_peak_1	Distal Interger	1	1.16E+08	1.17E+08	895538	1	636808	uc007cia.1	950588	ENSMUSG000	Cntnap5a	contactin associated protein-like 5A
chr1	116650396	116650542	147	148	116650453	38	11.72962	3.59649	8.4047	Adipose_R2_peak_1	Distal Interger	1	1.16E+08	1.17E+08	895538	1	636808	uc007cia.1	965259	ENSMUSG000	Cntnap5a	contactin associated protein-like 5A
chr1	116652907	116653131	225	226	116653025	28	13.73721	5.2124	10.41463	Adipose_R1_peak_1	Distal Interger	1	1.16E+08	1.17E+08	895538	1	636808	uc007cia.1	967770	ENSMUSG000	Cntnap5a	contactin associated protein-like 5A
chr1	116652911	116653098	188	189	116653011	39	12.34187	3.68871	8.98501	Adipose_R2_peak_1	Distal Interger	1	1.16E+08	1.17E+08	895538	1	636808	uc007cia.1	967774	ENSMUSG000	Cntnap5a	contactin associated protein-like 5A
chr1	116773256	116773406	151	152	116773318	18	6.42934	3.41502	3.48999	Adipose_R1_peak_1	Distal Interger	1	1.16E+08	1.17E+08	895538	1	636808	uc007cia.1	1088119	ENSMUSG000	Cntnap5a	contactin associated protein-like 5A
chr1	116838146	116838311	166	167	116838210	20	7.74501	3.7745	4.70467	Adipose_R1_peak_1	Distal Interger	1	1.16E+08	1.17E+08	895538	1	636808	uc007cia.1	1153009	ENSMUSG000	Cntnap5a	contactin associated protein-like 5A
chr1	116856880	116857247	368	369	116856941	20	7.74501	3.7745	4.70467	Adipose_R1_peak_1	Distal Interger	1	1.16E+08	1.17E+08	895538	1	636808	uc007cia.1	1171743	ENSMUSG000	Cntnap5a	contactin associated protein-like 5A
chr1	116857573	116857712	140	141	116857641	19	7.07687	3.59476	4.08458	Adipose_R1_peak_1	Distal Interger	1	1.16E+08	1.17E+08	895538	1	636808	uc007cia.1	1172436	ENSMUSG000	Cntnap5a	contactin associated protein-like 5A
chr1	116858418	116858760	343	344	116858575	110	107.9475	19.57177	103.5795	Adipose_R1_peak_1	Distal Interger	1	1.16E+08	1.17E+08	895538	1	636808	uc007cia.1	1173281	ENSMUSG000	Cntnap5a	contactin associated protein-like 5A
chr1	116858438	116858732	295	296	116858573	93	56.90635	8.66847	52.67918	Adipose_R2_peak_1	Distal Interger	1	1.16E+08	1.17E+08	895538	1	636808	uc007cia.1	1173301	ENSMUSG000	Cntnap5a	contactin associated protein-like 5A
chr1	116897992	116898190	199	200	116898090	43	14.8939	4.05758	11.42017	Adipose_R2_peak_1	Distal Interger	1	1.16E+08	1.17E+08	895538	1	636808	uc007cia.1	1212855	ENSMUSG000	Cntnap5a	contactin associated protein-like 5A
chr1	116898019	116898204	186	187	116898100	31	16.23981	5.75161	12.83549	Adipose_R1_peak_1	Distal Interger	1	1.16E+08	1.17E+08	895538	1	636808	uc007cia.1	1212882	ENSMUSG000	Cntnap5a	contactin associated protein-like 5A
chr1	116963427	116963577	151	152	116963568	18	6.42934	3.41502	3.48999	Adipose_R1_peak_1	Distal Interger	1	1.16E+08	1.17E+08	895538	1	636808	uc007cia.1	1278290	ENSMUSG000	Cntnap5a	contactin associated protein-like 5A
chr1	117096579	117096862	284	285	117096712	39	23.47001	7.18952	19.88325	Adipose_R1_peak_1	Distal Interger	1	1.18E+08	1.18E+08	12615	2	22099	uc007cib.1	1214270	ENSMUSG000	Tsn	translin
chr1	117096631	117096792	162	163	117096705	36	10.5378	3.41206	7.27949	Adipose_R2_peak_1	Distal Interger	1	1.18E+08	1.18E+08	12615	2	22099	uc007cib.1	1214340	ENSMUSG000	Tsn	translin
chr1	117168234	117168328	95	96	117168279	30	7.24477	2.85875	4.21917	Adipose_R2_peak_1	Distal Interger	1	1.18E+08	1.18E+08	12615	2	22099	uc007cib.1	1142804	ENSMUSG000	Tsn	translin
chr1	117206672	117206957	286	287	117206825	72	59.47482	13.12087	55.39848	Adipose_R1_peak_1	Distal Interger	1	1.18E+08	1.18E+08	12615	2	22099	uc007cib.1	1104175	ENSMUSG000	Tsn	translin
chr1	117206776	117206907	132	133	117206841	33	8.83596	3.1354	5.68652	Adipose_R2_peak_1	Distal Interger	1	1.18E+08	1.18E+08	12615	2	22099	uc007cib.1	1104225	ENSMUSG000	Tsn	translin
chr1	117239990	117240189	200	201	117240055	19	7.07687	3.59476	4.08458	Adipose_R1_peak_1	Distal Interger	1	1.18E+08	1.18E+08	12615	2	22099	uc007cib.1	1070943	ENSMUSG000	Tsn	translin

chr1	117370053	117370323	271	272	117370185	53	37.70483	9.70585	33.87	Adipose_R1_peak_1	Distal Interger	1	1.18E+08	1.18E+08	12615	2	22099	uc007cib.1	940809	ENSMUSG000	Tsn	translin
chr1	117370074	117370325	252	253	117370196	61	28.12311	5.7175	24.28203	Adipose_R2_peak_1	Distal Interger	1	1.18E+08	1.18E+08	12615	2	22099	uc007cib.1	940807	ENSMUSG000	Tsn	translin
chr1	117375250	117375518	269	270	117375384	35	19.76029	6.47056	16.26028	Adipose_R1_peak_1	Distal Interger	1	1.18E+08	1.18E+08	12615	2	22099	uc007cib.1	935614	ENSMUSG000	Tsn	translin
chr1	117375327	117375443	117	118	117375382	28	6.25023	2.67431	3.31626	Adipose_R2_peak_1	Distal Interger	1	1.18E+08	1.18E+08	12615	2	22099	uc007cib.1	935689	ENSMUSG000	Tsn	translin
chr1	117403569	117403707	139	140	117403641	21	8.43286	3.95423	5.34896	Adipose_R1_peak_1	Distal Interger	1	1.18E+08	1.18E+08	12615	2	22099	uc007cib.1	907425	ENSMUSG000	Tsn	translin
chr1	117484306	117484487	182	183	117484416	24	10.60668	4.49345	7.40961	Adipose_R1_peak_1	Distal Interger	1	1.18E+08	1.18E+08	12615	2	22099	uc007cib.1	826645	ENSMUSG000	Tsn	translin
chr1	117588619	117588757	139	140	117588695	18	6.42934	3.41502	3.48999	Adipose_R1_peak_1	Distal Interger	1	1.18E+08	1.18E+08	12615	2	22099	uc007cib.1	722375	ENSMUSG000	Tsn	translin
chr1	117590286	117590447	162	163	117590363	21	8.43286	3.95423	5.34896	Adipose_R1_peak_1	Distal Interger	1	1.18E+08	1.18E+08	12615	2	22099	uc007cib.1	720685	ENSMUSG000	Tsn	translin
chr1	117590300	117590447	148	149	117590363	31	7.7623	2.95097	4.69388	Adipose_R2_peak_1	Distal Interger	1	1.18E+08	1.18E+08	12615	2	22099	uc007cib.1	720685	ENSMUSG000	Tsn	translin
chr1	117590668	117590886	219	220	117590761	28	13.73721	5.2124	10.41463	Adipose_R1_peak_1	Distal Interger	1	1.18E+08	1.18E+08	12615	2	22099	uc007cib.1	720246	ENSMUSG000	Tsn	translin
chr1	117639634	117639909	276	277	117639774	54	38.78658	9.88558	34.93639	Adipose_R1_peak_1	Distal Interger	1	1.18E+08	1.18E+08	12615	2	22099	uc007cib.1	671223	ENSMUSG000	Tsn	translin
chr1	117639689	117639882	194	195	117639767	40	12.96465	3.78093	9.57654	Adipose_R2_peak_1	Distal Interger	1	1.18E+08	1.18E+08	12615	2	22099	uc007cib.1	671250	ENSMUSG000	Tsn	translin
chr1	117686846	117687011	166	167	117686930	34	9.39139	3.22762	6.20474	Adipose_R2_peak_1	Distal Interger	1	1.18E+08	1.18E+08	12615	2	22099	uc007cib.1	624121	ENSMUSG000	Tsn	translin
chr1	117720698	117720890	193	194	117720812	39	12.34187	3.68871	8.98501	Adipose_R2_peak_1	Distal Interger	1	1.18E+08	1.18E+08	12615	2	22099	uc007cib.1	590242	ENSMUSG000	Tsn	translin
chr1	117720706	117720965	260	261	117720827	44	28.34544	8.08821	24.66239	Adipose_R1_peak_1	Distal Interger	1	1.18E+08	1.18E+08	12615	2	22099	uc007cib.1	590167	ENSMUSG000	Tsn	translin
chr1	117722433	117722607	175	176	117722505	23	9.86443	4.31371	6.70247	Adipose_R1_peak_1	Distal Interger	1	1.18E+08	1.18E+08	12615	2	22099	uc007cib.1	588525	ENSMUSG000	Tsn	translin
chr1	117723082	117723213	132	133	117723154	19	7.07687	3.59476	4.08458	Adipose_R1_peak_1	Distal Interger	1	1.18E+08	1.18E+08	12615	2	22099	uc007cib.1	587919	ENSMUSG000	Tsn	translin
chr1	117824463	117824616	154	155	117824540	23	9.86443	4.31371	6.70247	Adipose_R1_peak_1	Distal Interger	1	1.18E+08	1.18E+08	12615	2	22099	uc007cib.1	486516	ENSMUSG000	Tsn	translin
chr1	117839757	117839962	206	207	117839921	43	14.8939	4.05758	11.42017	Adipose_R2_peak_1	Distal Interger	1	1.18E+08	1.18E+08	12615	2	22099	uc007cib.1	471170	ENSMUSG000	Tsn	translin
chr1	117877055	117877277	223	224	117877229	57	24.96362	5.34863	21.18596	Adipose_R2_peak_1	Distal Interger	1	1.18E+08	1.18E+08	12615	2	22099	uc007cib.1	433855	ENSMUSG000	Tsn	translin
chr1	117877098	117877277	180	181	117877208	26	12.14079	4.85292	8.87838	Adipose_R1_peak_1	Distal Interger	1	1.18E+08	1.18E+08	12615	2	22099	uc007cib.1	433855	ENSMUSG000	Tsn	translin
chr1	117877678	117877880	203	204	117877786	34	18.86162	6.29083	15.38482	Adipose_R1_peak_1	Distal Interger	1	1.18E+08	1.18E+08	12615	2	22099	uc007cib.1	433252	ENSMUSG000	Tsn	translin
chr1	117924393	117924551	159	160	117924474	25	11.36566	4.67319	8.13482	Adipose_R1_peak_1	Distal Interger	1	1.18E+08	1.18E+08	12615	2	22099	uc007cib.1	386581	ENSMUSG000	Tsn	translin
chr1	117957735	117957946	212	213	117957854	30	15.39182	5.57188	12.01394	Adipose_R1_peak_1	Distal Interger	1	1.18E+08	1.18E+08	12615	2	22099	uc007cib.1	353186	ENSMUSG000	Tsn	translin
chr1	117957768	117957889	122	123	117957879	31	7.7623	2.95097	4.69388	Adipose_R2_peak_1	Distal Interger	1	1.18E+08	1.18E+08	12615	2	22099	uc007cib.1	353243	ENSMUSG000	Tsn	translin
chr1	118055184	118055445	262	263	118055307	37	21.59274	6.83004	18.04766	Adipose_R1_peak_1	Distal Interger	1	1.18E+08	1.18E+08	12615	2	22099	uc007cib.1	255687	ENSMUSG000	Tsn	translin
chr1	118068860	118069012	153	154	118068918	18	6.42934	3.41502	3.48999	Adipose_R1_peak_1	Distal Interger	1	1.18E+08	1.18E+08	12615	2	22099	uc007cib.1	242120	ENSMUSG000	Tsn	translin
chr1	118091539	118091830	292	293	118091689	55	39.35435	9.87404	35.5015	Adipose_R1_peak_1	Distal Interger	1	1.18E+08	1.18E+08	12615	2	22099	uc007cib.1	219302	ENSMUSG000	Tsn	translin
chr1	118091595	118091805	211	212	118091666	47	17.60017	4.42645	14.02531	Adipose_R2_peak_1	Distal Interger	1	1.18E+08	1.18E+08	12615	2	22099	uc007cib.1	219327	ENSMUSG000	Tsn	translin
chr1	118097221	118097648	428	429	118097488	61	46.56983	11.14375	42.62347	Adipose_R1_peak_1	Distal Interger	1	1.18E+08	1.18E+08	12615	2	22099	uc007cib.1	213484	ENSMUSG000	Tsn	translin
chr1	118097235	118097636	402	403	118097510	57	24.96362	5.34863	21.18596	Adipose_R2_peak_1	Distal Interger	1	1.18E+08	1.18E+08	12615	2	22099	uc007cib.1	213496	ENSMUSG000	Tsn	translin
chr1	118098497	118098690	194	195	118098608	26	12.14079	4.85292	8.87838	Adipose_R1_peak_1	Distal Interger	1	1.18E+08	1.18E+08	12615	2	22099	uc007cib.1	212442	ENSMUSG000	Tsn	translin
chr1	118109691	118109872	182	183	118109758	17	5.80341	3.23528	2.92378	Adipose_R1_peak_1	Distal Interger	1	1.18E+08	1.18E+08	12615	2	22099	uc007cib.1	201260	ENSMUSG000	Tsn	translin
chr1	118117515	118117702	188	189	118117601	23	9.86443	4.31371	6.70247	Adipose_R1_peak_1	Distal Interger	1	1.18E+08	1.18E+08	12615	2	22099	uc007cib.1	193430	ENSMUSG000	Tsn	translin
chr1	118122939	118123072	134	135	118123011	22	9.13959	4.13397	6.01526	Adipose_R1_peak_1	Distal Interger	1	1.18E+08	1.18E+08	12615	2	22099	uc007cib.1	188060	ENSMUSG000	Tsn	translin
chr1	118123980	118124227	248	249	118124104	40	24.42476	7.36925	20.81738	Adipose_R1_peak_1	Distal Interger	1	1.18E+08	1.18E+08	12615	2	22099	uc007cib.1	186905	ENSMUSG000	Tsn	translin
chr1	118124046	118124190	145	146	118124112	34	9.39139	3.22762	6.20474	Adipose_R2_peak_1	Distal Interger	1	1.18E+08	1.18E+08	12615	2	22099	uc007cib.1	186942	ENSMUSG000	Tsn	translin
chr1	118160081	118160282	202	203	118160176	24	10.60668	4.49345	7.40961	Adipose_R1_peak_1	Distal Interger	1	1.18E+08	1.18E+08	12615	2	22099	uc007cib.1	150850	ENSMUSG000	Tsn	translin
chr1	118193404	118193605	202	203	118193512	20	7.74501	3.7745	4.70467	Adipose_R1_peak_1	Distal Interger	1	1.18E+08	1.18E+08	12615	2	22099	uc007cib.1	117527	ENSMUSG000	Tsn	translin
chr1	118246526	118246720	195	196	118246611	21	8.43286	3.95423	5.34896	Adipose_R1_peak_1	Distal Interger	1	1.18E+08	1.18E+08	12615	2	22099	uc007cib.1	64412	ENSMUSG000	Tsn	translin
chr1	118247417	118247634	218	219	118247505	28	13.73721	5.2124	10.41463	Adipose_R1_peak_1	Distal Interger	1	1.18E+08	1.18E+08	12615	2	22099	uc007cib.1	63498	ENSMUSG000	Tsn	translin
chr1	118585587	118585831	245	246	118585714	40	24.42476	7.36925	20.81738	Adipose_R1_peak_1	Intron (uc007c	1	1.19E+08	1.19E+08	9822	1	81879	uc007cik.1	-42114	ENSMUSG000	Tfcp2l1	transcription factor CP2-like 1
chr1	118585600	118585832	233	234	118585708	54	22.67101	5.07198	18.94758	Adipose_R2_peak_1	Intron (uc007c	1	1.19E+08	1.19E+08	9822	1	81879	uc007cik.1	-42113	ENSMUSG000	Tfcp2l1	transcription factor CP2-like 1
chr1	120572231	120572393	163	164	120572274	28	6.25023	2.67431	3.31626	Adipose_R2_peak_1	Distal Interger	1	1.21E+08	1.21E+08	5505	1	13798	uc007cjm.2	-30094	ENSMUSG000	En1	engrailed 1
chr1	120572486	120572693	208	209	120572617	23	9.86443	4.31371	6.70247	Adipose_R1_peak_1	Distal Interger	1	1.21E+08	1.21E+08	5505	1	13798	uc007cjm.2	-29794	ENSMUSG000	En1	engrailed 1
chr1	121622956	121623128	173	174	121623051	36	10.5378	3.41206	7.27949	Adipose_R2_peak_1	Distal Interger	1	1.22E+08	1.22E+08	14146	2	66942	uc007cjw.2	-54976	ENSMUSG000	Ddx18	DEAD (Asp-Glu-Ala-Asp) box polypeptide 18
chr1	121622963	121623161	199	200	121623063	27	12.93148	5.03266	9.63827	Adipose_R1_peak_1	Distal Interger	1	1.22E+08	1.22E+08	14146	2	66942	uc007cjw.2	-54983	ENSMUSG000	Ddx18	DEAD (Asp-Glu-Ala-Asp) box polypeptide 18
chr1	121668188	121668338	151	152	121668302	18	6.42934	3.41502	3.48999	Adipose_R1_peak_1	Distal Interger	1	1.22E+08	1.22E+08	14146	2	66942	uc007cjw.2	-100208	ENSMUSG000	Ddx18	DEAD (Asp-Glu-Ala-Asp) box polypeptide 18
chr1	121674971	121675162	192	193	121675032	31	16.23981	5.75161	12.83549	Adipose_R1_peak_1	Distal Interger	1	1.22E+08	1.22E+08	14146	2	66942	uc007cjw.2	-106991	ENSMUSG000	Ddx18	DEAD (Asp-Glu-Ala-Asp) box polypeptide 18
chr1	121804028	121804174	147	148	121804091	17	5.80341	3.23528	2.92378	Adipose_R1_peak_1	Distal Interger	1	1.22E+08	1.22E+08	14146	2	66942	uc007cjw.2	-236048	ENSMUSG000	Ddx18	DEAD (Asp-Glu-Ala-Asp) box polypeptide 18
chr1	121838321	121838495	175	176	121838424	25	11.36566	4.67319	8.13482	Adipose_R1_peak_1	Distal Interger	1	1.22E+08	1.22E+08	14146	2	66942	uc007cjw.2	-270341	ENSMUSG000	Ddx18	DEAD (Asp-Glu-Ala-Asp) box polypeptide 18
chr1	122012056	122012223	168	169	122012080	17	5.80341	3.23528	2.92378	Adipose_R1_peak_1	Distal Interger	1	1.22E+08	1.22E+08	14146	2	66942	uc007cjw.2	-444076	ENSMUSG000	Ddx18	DEAD (Asp-Glu-Ala-Asp) box polypeptide 18
chr1	122032868	122033167	300	301	122032985	115	69.41998	8.72623	65.25997	Adipose_R1_peak_1	Distal Interger	1	1.22E+08	1.22E+08	14146	2	66942	uc007cjw.2	-464888	ENSMUSG000	Ddx18	DEAD (Asp-Glu-Ala-Asp) box polypeptide 18
chr1	122032915	122033132	218	219	122033001	67	28.56607	5.31572	24.72042	Adipose_R2_peak_1	Distal Interger	1	1.22E+08	1.22E+08	14146	2	66942	uc007cjw.2	-464935	ENSMUSG000	Ddx18	DEAD (Asp-Glu-Ala-Asp) box polypeptide 18
chr1	122036670	122036899	230	231	122036782	40	24.42476	7.36925	20.81738	Adipose_R1_peak_1	Distal Interger	1	1.22E+08	1.22E+08	14146	2	66942	uc007cjw.2	-468690	ENSMUSG000	Ddx18	DEAD (Asp-Glu-Ala-Asp) box polypeptide 18
chr1	122036696	122036881	186	187	122036797	36	10.5378	3.41206	7.27949	Adipose_R2_peak_1	Distal Interger	1	1.22E+08	1.22E+08	14146	2	66942	uc007cjw.2	-468716	ENSMUSG000	Ddx18	DEAD (Asp-Glu-Ala-Asp) box polypeptide 18
chr1	122087250	122087420	171	172	122087335	21	8.43286	3.95423	5.34896	Adipose_R1_peak_1	Distal Interger	1	1.22E+08	1.22E+08	14146	2	66942	uc007cjw.2	-519270	ENSMUSG000	Ddx18	DEAD (Asp-Glu-Ala-Asp) box polypeptide 18
chr1	122138371	122138646	276	277	122138498	68	54.69342	12.40192	50.66183	Adipose_R1_peak_1	Distal Interger	1	1.22E+08	1.22E+08	14146	2	66942	uc007cjw.2	-570391	ENSMUSG000	Ddx18	DEAD (Asp-Glu-Ala-Asp) box polypeptide 18
chr1	122138383	122138601	219	220	122138504	62	28.93052	5.80972	25.0743	Adipose_R2_peak_1	Distal Interger	1	1.22E+08	1.22E+08	14146	2	66942	uc007cjw.2	-570403	ENSMUSG000	Ddx18	DEAD (Asp-Glu-Ala-Asp) box polypeptide 18
chr1	122317657	122317913	257	258	122317792	42	26.36523	7.72873	22.71906	Adipose_R1_peak_1	Distal Interger	1	1.22E+08	1.22E+08	14146	2	66942	uc007cjw.2	-749677	ENSMUSG000	Ddx18	DEAD (Asp-Glu-Ala-Asp) box polypeptide 18
chr1	122317715	122317863	149	150	122317790	30	7.24477	2.85875	4.21917	Adipose_R2_peak_1	Distal Interger	1	1.22E+08	1.22E+08	14146	2	66942	uc007cjw.2	-749735	ENSMUSG000	Ddx18	DEAD (Asp-Glu-Ala-Asp) box polypeptide 18
chr1	122447591	122447862	272	273	122447756	30	15.39182	5.57188	12.01394	Adipose_R1_peak_1	Distal Interger	1	1.22E+08	1.22E+08	14146	2	66942	uc007cjw.2	-879611	ENSMUSG000	Ddx18	DEAD (Asp-Glu-Ala-Asp) box polypeptide 18
chr1	122449847	122450038	192	193	122449941	41	13.59774	3.87315	10.1803	Adipose_R2_peak_1	Distal Interger	1	1.22E+08	1.22E+08	14146	2	66942	uc007cjw.2	-881867	ENSMUSG000	Ddx18	DEAD (Asp-Glu-Ala-Asp) box polypeptide 18
chr1	122449848	122450047	200	201	122449923	28	13.73721	5.2124	10.41463	Adipose_R1_peak_1	Distal Interger	1	1.22E+08	1.22E+08	14146	2	66942	uc007cjw.2	-881868	ENSMUSG000	Ddx18	DEAD (Asp-Glu-Ala-Asp) box polypeptide 18
chr1	122655533	122655766	234	235	122655645	35	19.76029	6.47056	16.26028	Adipose_R1_peak_1	Distal Interger	1	1.22E+08	1.22E+08	14146	2	66942	uc007cjw.2	-1087553	ENSMUSG000	Ddx18	DEAD (Asp-Glu-Ala-Asp) box polypeptide 18
chr1	122673265	122673598	334	335	122673469	165	151.928	18.54824	147.409	Adipose_R1_peak_1	Distal Interger	1	1.22E+08	1.22E+08	14146	2	66942	uc007cjw.2	-1105285	ENSMUSG000	Ddx18	DEAD (Asp-Glu-Ala-Asp) box polypeptide 18
chr1	122673334	122673598	265	266	122673476	139	106.3294	12.91048	101.798	Adipose_R2_peak_1	Distal Interger	1	1.22E+08	1.22E+08	14146	2	66942	uc007cjw.2	-1105354	ENSMUSG000	Ddx18	DEAD (Asp-Glu-Ala-Asp) box polypeptide 18
chr1	122674788	122675078	291	292	122674923	69	55.87963	12.58165	51.83661	Adipose_R1_peak_1	Distal Interger	1	1.22E+08	1.22E+08	14146	2	66942	uc007cjw.2	-1106808	ENSMUSG000	Ddx18	DEAD (Asp-Glu-Ala-Asp) box polypeptide 18
chr1	122677137	122677345	209	210	122677256	26	12.14079	4.85292	8.87838	Adipose_R1_peak_1	Distal Interger	1	1.22E+08	1.22E+08	14146	2	66942	uc007cjw.2	-1109157	ENSMUSG000	Ddx18	DEAD (Asp-Glu-Ala-Asp) box polypeptide 18
chr1	122767412	122767689	278	279	122767568	62	47.71039	11.32349	43.751	Adipose_R1_peak_1	Distal Interger	1	1.22E+08	1.22E+08	14146	2	66942	uc007cjw.2	-1199432	ENSMUSG000	Ddx18	DEAD (Asp-Glu-Ala-Asp) box polypeptide 18
chr1	122767467	122767674	208	209	122767559	45	16.22858	4.24202	12.70278	Adipose_R2_peak_1	Distal Interger	1	1.22E+08	1.22E+08	14146	2	66942	uc007cjw.2	-1199487	ENSMUSG000	Ddx18	DEAD (Asp-Glu-Ala-Asp) box polypeptide 18
chr1	122771444	122771705	262	263	122771581	47	31.38649	8.62742	27.65048	Adipose_R1_peak_1	Distal Interger	1	1.22E+08	1.22E+08	14146	2	66942	uc007cjw.2	-1203464	ENSMUSG000	Ddx18	DEAD (Asp-Glu-Ala-Asp) box polypeptide 18
chr1	122772874	122773162	289	290	122772937	32	8.29282	3.04319	5.18352	Adipose_R2_peak_1	Distal Interger	1	1.22E+08	1.22E+08	14146	2	66942	uc007cjw.2	-1204894	ENSMUSG000	Ddx18	DEAD (Asp-Glu-Ala-Asp) box polypeptide 18
chr1	122772898	122773232	335	336	122773146	26	12.14079	4.85292	8.87838	Adipose_R1_peak_1	Distal Interger	1	1.22E+08	1.22E+08	14146	2	66942	uc007cjw.2	-1204918	ENSMUSG000	Ddx18	DEAD (Asp-Glu-Ala-Asp) box polypeptide 18
chr1	122955135	122955268	134	135	122955202	22	9.13959	4.13397	6.01526	Adipose_R1_peak_1	Distal Interger	1	1.23E+08	1.24E+08	713422	2	269109	uc007cjx.2	1090291	ENSMUSG000	Dpp10	dipeptidylpeptidase 10
chr1	123015934	123016146	213	214	123016020	33	17.97508	6.11109	14.52164	Adipose_R1_peak_1	Distal Interger	1	1.23E+08	1.24E+08	713422	2	269109	uc007cjx.2	1029413	ENSMUSG000	Dpp10	dipeptidylpeptidase 10
chr1	123015951	123016114	164	165	123016029	34	9.39139	3.22762	6.20474	Adipose_R2_peak_1	Distal Interger	1	1.23E+08	1.24E+08	713422	2	269109	uc007cjx.2	1029445	ENSMUSG000	Dpp10	dipeptidylpeptidase 10
chr1	123214253	123214425	173	174	123214267	17	5.80341	3.23528	2.92378	Adipose_R1_peak_1	Distal Interger	1	1.23E+08	1.24E+08	713422	2	269109	uc007cjx.2	831134	ENSMUSG000	Dpp10	dipeptidylpeptidase 10
chr1	123857128	123857531	404	405	123857390	55	39.87613	10.06532	36.01168	Adipose_R1_peak_1	Intron (uc007c	1	1.23E+08	1.24E+08	713422	2	269109	uc007cjx.2	188028	ENSMUSG000	Dpp10	dipeptidylpeptidase 10
chr1	123857249	123857497	249	250	123857370	47	17.60017	4.42645	14.02531	Adipose_R2_peak_1	Intron (uc007c	1	1.23E+08	1.24E+08	713422	2	269109	uc007cjx.2	188062	ENSMUSG000	Dpp10	dipeptidylpeptidase 10
chr1	124007164	124007408	245	246	124007276	33	17.97508	6.11109	14.52164	Adipose_R1_peak_1	Intron (uc007c	1	1.23E+08	1.24E+08	713422	2	269109	uc007cjx.2	38151	ENSMUSG000	Dpp10	dipeptidylpeptidase 10
chr1	124088101	124088302	202	203	124088172	28	13.73721	5.2124	10.41463	Adipose_R1_peak_1	Distal Interger	1	1.23E+08	1.24E+08	713422	2	269109	uc007cjx.2	-42542	ENSMUSG000	Dpp10	dipeptidylpeptidase 10
chr1	124088807	124088939	133	134	124088888	18	6.42934	3.41502	3.48999	Adipose_R1_peak_1	Distal Interger	1	1.23E+08	1.24E+08	713422	2	269109	uc007cjx.2	-43248	ENSMUSG000	Dpp10	dipeptidylpeptidase 10
chr1	124090509	124090776	268	269	124090623	54	27.83723	6.37917	24.17007	Adipose_R1_peak_1	Distal Interger	1	1.23E+08	1.24E+08	713422	2	269109	uc007cjx.2	-44950	ENSMUSG000	Dpp10	dipeptidylpeptidase 10
chr1	124090524	124090739	216	217	124090633	46	16.90986	4.33423	13.35898	Adipose_R2_peak_1	Distal Interger	1	1.23E+08	1.24E+08	713422	2	269109	uc007cjx.2	-44965	ENSMUSG000	Dpp10	dipeptidylpeptidase 10
chr1	124143001	124143323	323	324	124143175	128	127.9553	20.9308	123.5116	Adipose_R1_peak_1	Distal Interger	1	1.23E+08	1.24E+08	713422	2	269109	uc007cjx.2	-97442	ENSMUSG000	Dpp10	dipeptidylpeptidase 10
chr1	124143032	124143321	290	291	124143172	115	79.5602	10.69726	75.15137	Adipose_R2_peak_1	Distal Interger	1	1.23E+08	1.24E+08	713422	2	269109	uc007cjx.2	-97473	ENSMUSG000	Dpp10	dipeptidylpeptidase 10
chr1	124146933	124147129	197	198	124147013	27	12.93148	5.03266	9.63827	Adipose_R1_peak_1	Distal Interger	1	1.23E+08	1.24E+08	713422	2	269109	uc007cjx.2	-101374	ENSMUSG000	Dpp10	dipeptidylpeptidase 10
chr1	124660798	124661017	220	221	124660910	29	14.55748	5.39214	11.20652	Adipose_R1_peak_1	Intron (uc007c	1	1.23E+08	1.24E+08	713422	2	269109	uc007cjx.2	-615239	ENSMUSG000	Dpp10	dipeptidylpeptidase 10
chr1	124742430	124742642	213	214	124742560	27	12.93148	5.03266	9.63827	Adipose_R1_peak_1	Intron (uc007c	1	1.25E+08	1.25E+08	42823	2	74117	uc007cke.2	693085	ENSMUSG000	Actr3	ARP3 actin-related protein 3
chr1	124742488	124742618	131	132	124742573	28	6.25023	2.67431	3.31626	Adipose_R2_peak_1	Intron (uc007c	1	1.25E+08	1.25E+08	42823	2	74117	uc007cke.2	693109	ENSMUSG000	Actr3	ARP3 actin-related protein 3
chr1	124881984	124882207	224	225	124882089	33	17.97508	6.11109	14.52164	Adipose_R1_peak_1	Distal Interger	1	1.25E+08	1.25E+08	42823	2	74117	uc007cke.2	553520	ENSMUSG000	Actr3	ARP3 actin-related protein 3
chr1	124881991	124882165	175	176	124882073	34	9.39139	3.22762	6.20474	Adipose_R2_peak_1	Distal Interger	1	1.25E+08	1.25E+08	42823	2	74117	uc007cke.2	553562	ENSMUSG000	Actr3	ARP3 actin-related protein 3
chr1	124966309	124966504	196	197	124966424	27	12.93148	5.03266	9.63827	Adipose_R1_peak_1	Distal Interger	1	1.25E+08	1.25E+08	42823	2	74117	uc007cke.2	469223	ENSMUSG000	Actr3	ARP3 actin-related protein 3

chr1	124967762	124968044	283	284	124967903	57	40.73726	9.93943	36.86884	Adipose_R1_peak_1	Distal Interger	1	1.25E+08	1.25E+08	42823	2	74117	uc007cke.2	467683	ENSMUSG000	Actr3	ARP3 actin-related protein 3
chr1	124967779	124968041	263	264	124967905	65	31.39315	6.08637	27.49408	Adipose_R2_peak_1	Distal Interger	1	1.25E+08	1.25E+08	42823	2	74117	uc007cke.2	467686	ENSMUSG000	Actr3	ARP3 actin-related protein 3
chr1	124968405	124968534	130	131	124968461	31	7.7623	2.95097	4.69388	Adipose_R2_peak_1	Distal Interger	1	1.25E+08	1.25E+08	42823	2	74117	uc007cke.2	467193	ENSMUSG000	Actr3	ARP3 actin-related protein 3
chr1	125007834	125008018	185	186	125008008	34	9.39139	3.22762	6.20474	Adipose_R2_peak_1	Distal Interger	1	1.25E+08	1.25E+08	42823	2	74117	uc007cke.2	427709	ENSMUSG000	Actr3	ARP3 actin-related protein 3
chr1	125007865	125008018	154	155	125007946	18	6.42934	3.41502	3.48999	Adipose_R1_peak_1	Distal Interger	1	1.25E+08	1.25E+08	42823	2	74117	uc007cke.2	427709	ENSMUSG000	Actr3	ARP3 actin-related protein 3
chr1	125094818	125095028	211	212	125094943	29	14.55748	5.39214	11.20652	Adipose_R1_peak_1	Distal Interger	1	1.25E+08	1.25E+08	42823	2	74117	uc007cke.2	340699	ENSMUSG000	Actr3	ARP3 actin-related protein 3
chr1	125133744	125133962	219	220	125133876	30	15.39182	5.57188	12.01394	Adipose_R1_peak_1	Distal Interger	1	1.25E+08	1.25E+08	42823	2	74117	uc007cke.2	301765	ENSMUSG000	Actr3	ARP3 actin-related protein 3
chr1	125140679	125140783	105	106	125140720	25	4.86612	2.39766	2.09018	Adipose_R2_peak_1	Distal Interger	1	1.25E+08	1.25E+08	42823	2	74117	uc007cke.2	294944	ENSMUSG000	Actr3	ARP3 actin-related protein 3
chr1	125347273	125347455	183	184	125347392	24	10.60668	4.49345	7.40961	Adipose_R1_peak_1	Distal Interger	1	1.25E+08	1.25E+08	42823	2	74117	uc007cke.2	88272	ENSMUSG000	Actr3	ARP3 actin-related protein 3
chr1	125348869	125348986	118	119	125348950	29	6.74062	2.76653	3.75989	Adipose_R2_peak_1	Distal Interger	1	1.25E+08	1.25E+08	42823	2	74117	uc007cke.2	86741	ENSMUSG000	Actr3	ARP3 actin-related protein 3
chr1	125512796	125512946	151	152	125512871	19	7.07687	3.59476	4.08458	Adipose_R1_peak_1	Distal Interger	1	1.26E+08	1.26E+08	34669	1	74150	uc007ckg.1	-48070	ENSMUSG000	Slc35f5	solute carrier family 35, member F5
chr1	125618164	125618371	208	209	125618273	55	23.42763	5.16419	19.68558	Adipose_R2_peak_1	Distal Interger	1	1.26E+08	1.26E+08	34669	1	74150	uc007ckg.1	57148	ENSMUSG000	Slc35f5	solute carrier family 35, member F5
chr1	125618170	125618435	266	267	125618279	52	36.63102	9.52611	32.81188	Adipose_R1_peak_1	Distal Interger	1	1.26E+08	1.26E+08	34669	1	74150	uc007ckg.1	57154	ENSMUSG000	Slc35f5	solute carrier family 35, member F5
chr1	126275384	126275853	470	471	126275730	26	12.14079	4.85292	8.87838	Adipose_R1_peak_1	Intron (uc011v	1	1.26E+08	1.26E+08	468550	2	210356	uc011wqy.1	106332	ENSMUSG000	Nckap5	NCK-associated protein 5
chr1	126275398	126275779	382	383	126275643	40	12.96465	3.78093	9.57654	Adipose_R2_peak_1	Intron (uc011v	1	1.26E+08	1.26E+08	468550	2	210356	uc011wqy.1	106406	ENSMUSG000	Nckap5	NCK-associated protein 5
chr1	126668246	126668508	263	264	126668384	37	21.59274	6.83004	18.04766	Adipose_R1_peak_1	Intron (uc007c	1	1.26E+08	1.27E+08	916997	2	210356	uc007ckm.1	162124	ENSMUSG000	Nckap5	NCK-associated protein 5
chr1	126668268	126668498	231	232	126668380	44	15.55653	4.1498	12.05625	Adipose_R2_peak_1	Intron (uc007c	1	1.26E+08	1.27E+08	916997	2	210356	uc007ckm.1	162134	ENSMUSG000	Nckap5	NCK-associated protein 5
chr1	126769279	126769581	303	304	126769432	76	46.32772	8.37366	42.39051	Adipose_R1_peak_1	Intron (uc007c	1	1.26E+08	1.27E+08	916997	2	210356	uc007ckm.1	61051	ENSMUSG000	Nckap5	NCK-associated protein 5
chr1	126769293	126769563	271	272	126769399	55	17.77062	3.95384	14.19289	Adipose_R2_peak_1	Intron (uc007c	1	1.26E+08	1.27E+08	916997	2	210356	uc007ckm.1	61069	ENSMUSG000	Nckap5	NCK-associated protein 5
chr1	126794152	126794254	103	104	126794212	28	6.25023	2.67431	3.31626	Adipose_R2_peak_1	Intron (uc007c	1	1.26E+08	1.27E+08	916997	2	210356	uc007ckm.1	36378	ENSMUSG000	Nckap5	NCK-associated protein 5
chr1	126797650	126797929	280	281	126797780	72	37.3624	6.73189	33.36734	Adipose_R2_peak_1	Intron (uc007c	1	1.26E+08	1.27E+08	916997	2	210356	uc007ckm.1	32703	ENSMUSG000	Nckap5	NCK-associated protein 5
chr1	126797664	126797943	280	281	126797790	60	35.81745	7.81824	32.01317	Adipose_R1_peak_1	Intron (uc007c	1	1.26E+08	1.27E+08	916997	2	210356	uc007ckm.1	32689	ENSMUSG000	Nckap5	NCK-associated protein 5
chr1	126940242	126940472	231	232	126940375	25	8.20857	3.47852	5.16326	Adipose_R1_peak_1	Distal Interger	1	1.26E+08	1.27E+08	916997	2	210356	uc007ckm.1	-109610	ENSMUSG000	Nckap5	NCK-associated protein 5
chr1	126940359	126940565	207	208	126940457	34	9.39139	3.22762	6.20474	Adipose_R2_peak_1	Distal Interger	1	1.26E+08	1.27E+08	916997	2	210356	uc007ckm.1	-109727	ENSMUSG000	Nckap5	NCK-associated protein 5
chr1	126943531	126943787	257	258	126943652	33	17.97508	6.11109	14.52164	Adipose_R1_peak_1	Distal Interger	1	1.26E+08	1.27E+08	916997	2	210356	uc007ckm.1	-112899	ENSMUSG000	Nckap5	NCK-associated protein 5
chr1	128449090	128449349	260	261	128449227	33	17.97508	6.11109	14.52164	Adipose_R1_peak_1	Distal Interger	1	1.28E+08	1.28E+08	53710	2	226414	uc007clp.2	-31674	ENSMUSG000	Dars	aspartyl-tRNA synthetase
chr1	128449135	128449343	209	210	128449226	34	9.39139	3.22762	6.20474	Adipose_R2_peak_1	Distal Interger	1	1.28E+08	1.28E+08	53710	2	226414	uc007clp.2	-31719	ENSMUSG000	Dars	aspartyl-tRNA synthetase
chr1	128484340	128484531	192	193	128484451	25	11.36566	4.67319	8.13482	Adipose_R1_peak_1	Distal Interger	1	1.28E+08	1.28E+08	53710	2	226414	uc007clp.2	-66924	ENSMUSG000	Dars	aspartyl-tRNA synthetase
chr1	128501274	128501424	151	152	128501341	32	8.29282	3.04319	5.18352	Adipose_R2_peak_1	Distal Interger	1	1.28E+08	1.28E+08	53710	2	226414	uc007clp.2	-83858	ENSMUSG000	Dars	aspartyl-tRNA synthetase
chr1	128501295	128501485	191	192	128501365	25	11.36566	4.67319	8.13482	Adipose_R1_peak_1	Distal Interger	1	1.28E+08	1.28E+08	53710	2	226414	uc007clp.2	-83879	ENSMUSG000	Dars	aspartyl-tRNA synthetase
chr1	128502375	128502561	187	188	128502489	23	9.86443	4.31371	6.70247	Adipose_R1_peak_1	Distal Interger	1	1.28E+08	1.28E+08	53710	2	226414	uc007clp.2	-84959	ENSMUSG000	Dars	aspartyl-tRNA synthetase
chr1	128590355	128590538	184	185	128590393	26	5.31252	2.48988	2.47999	Adipose_R2_peak_1	Promoter (1-2	1	1.29E+08	1.29E+08	4095	2	12767	uc007cls.1	1755	ENSMUSG000	Cxcr4	chemokine (C-X-C motif) receptor 4
chr1	128746476	128746579	104	105	128746563	26	5.31252	2.48988	2.47999	Adipose_R2_peak_1	Distal Interger	1	1.29E+08	1.29E+08	4101	2	12767	uc007clt.1	-154177	ENSMUSG000	Cxcr4	chemokine (C-X-C motif) receptor 4
chr1	128775733	128776036	304	305	128775896	34	18.86162	6.29083	15.38482	Adipose_R1_peak_1	Distal Interger	1	1.29E+08	1.29E+08	4101	2	12767	uc007clt.1	-183434	ENSMUSG000	Cxcr4	chemokine (C-X-C motif) receptor 4
chr1	128775848	128775979	132	133	128775909	36	10.5378	3.41206	7.27949	Adipose_R2_peak_1	Distal Interger	1	1.29E+08	1.29E+08	4101	2	12767	uc007clt.1	-183549	ENSMUSG000	Cxcr4	chemokine (C-X-C motif) receptor 4
chr1	128852042	128852255	214	215	128852157	31	16.17222	5.72504	12.79011	Adipose_R1_peak_1	Distal Interger	1	1.29E+08	1.29E+08	4101	2	12767	uc007clt.1	-259743	ENSMUSG000	Cxcr4	chemokine (C-X-C motif) receptor 4
chr1	128852716	128852893	178	179	128852816	26	12.14079	4.85292	8.87838	Adipose_R1_peak_1	Distal Interger	1	1.29E+08	1.29E+08	4101	2	12767	uc007clt.1	-260417	ENSMUSG000	Cxcr4	chemokine (C-X-C motif) receptor 4
chr1	128947080	128947265	186	187	128947189	24	10.60668	4.49345	7.40961	Adipose_R1_peak_1	Distal Interger	1	1.29E+08	1.29E+08	157750	1	210417	uc007clu.2	-326039	ENSMUSG000	Thsd7b	thrombospondin, type I, domain containing 7B
chr1	129091996	129092304	309	310	129092157	76	64.35061	13.83982	60.23161	Adipose_R1_peak_1	Distal Interger	1	1.29E+08	1.29E+08	157750	1	210417	uc007clu.2	-181000	ENSMUSG000	Thsd7b	thrombospondin, type I, domain containing 7B
chr1	129092028	129092265	238	239	129092159	52	21.18119	4.88754	17.49655	Adipose_R2_peak_1	Distal Interger	1	1.29E+08	1.29E+08	157750	1	210417	uc007clu.2	-181039	ENSMUSG000	Thsd7b	thrombospondin, type I, domain containing 7B
chr1	129270627	129270788	162	163	129270784	22	9.13959	4.13397	6.01526	Adipose_R1_peak_1	Promoter (2-3	1	1.29E+08	1.29E+08	157750	1	210417	uc007clu.2	-2516	ENSMUSG000	Thsd7b	thrombospondin, type I, domain containing 7B
chr1	129332060	129332197	138	139	129332167	21	8.43286	3.95423	5.34896	Adipose_R1_peak_1	Intron (uc007c	1	1.29E+08	1.29E+08	157750	1	210417	uc007clu.2	58756	ENSMUSG000	Thsd7b	thrombospondin, type I, domain containing 7B
chr1	129437478	129437749	272	273	129437618	54	38.78658	9.88558	34.93639	Adipose_R1_peak_1	Intron (uc007c	1	1.29E+08	1.29E+08	157750	1	210417	uc007clu.2	164174	ENSMUSG000	Thsd7b	thrombospondin, type I, domain containing 7B
chr1	129437506	129437742	237	238	129437605	44	15.55653	4.1498	12.05625	Adipose_R2_peak_1	Intron (uc007c	1	1.29E+08	1.29E+08	157750	1	210417	uc007clu.2	164202	ENSMUSG000	Thsd7b	thrombospondin, type I, domain containing 7B
chr1	129768000	129768386	387	388	129768185	28	13.73721	5.2124	10.41463	Adipose_R1_peak_1	Intron (uc007c	1	1.30E+08	1.30E+08	267643	1	210417	uc007clw.1	-147153	ENSMUSG000	Thsd7b	thrombospondin, type I, domain containing 7B
chr1	129768093	129768429	337	338	129768210	44	15.55653	4.1498	12.05625	Adipose_R2_peak_1	Intron (uc007c	1	1.30E+08	1.30E+08	267643	1	210417	uc007clw.1	-147110	ENSMUSG000	Thsd7b	thrombospondin, type I, domain containing 7B
chr1	129770302	129770566	265	266	129770454	40	24.42476	7.36925	20.81738	Adipose_R1_peak_1	Intron (uc007c	1	1.30E+08	1.30E+08	267643	1	210417	uc007clw.1	-144973	ENSMUSG000	Thsd7b	thrombospondin, type I, domain containing 7B
chr1	129770345	129770504	160	161	129770442	35	9.95877	3.31984	6.73562	Adipose_R2_peak_1	Intron (uc007c	1	1.30E+08	1.30E+08	267643	1	210417	uc007clw.1	-145035	ENSMUSG000	Thsd7b	thrombospondin, type I, domain containing 7B
chr1	129773592	129773860	269	270	129773729	43	27.35048	7.90847	23.68561	Adipose_R1_peak_1	Intron (uc007c	1	1.30E+08	1.30E+08	267643	1	210417	uc007clw.1	-141679	ENSMUSG000	Thsd7b	thrombospondin, type I, domain containing 7B
chr1	129773647	129773859	213	214	129773744	44	15.55653	4.1498	12.05625	Adipose_R2_peak_1	Intron (uc007c	1	1.30E+08	1.30E+08	267643	1	210417	uc007clw.1	-141680	ENSMUSG000	Thsd7b	thrombospondin, type I, domain containing 7B
chr1	130033866	130034079	214	215	130033965	30	15.39182	5.57188	12.01394	Adipose_R1_peak_1	Intron (uc007c	1	1.30E+08	1.30E+08	267643	1	210417	uc007clw.1	118327	ENSMUSG000	Thsd7b	thrombospondin, type I, domain containing 7B
chr1	130204324	130204959	636	637	130204495	40	24.42476	7.36925	20.81738	Adipose_R1_peak_1	Intron (uc007c	1	1.30E+08	1.30E+08	34516	2	13137	uc007clx.2	218084	ENSMUSG000	Cd55b	CD55 molecule, decay accelerating factor for complement B
chr1	130204745	130204962	218	219	130204841	48	18.29934	4.51867	14.70076	Adipose_R2_peak_1	Intron (uc007c	1	1.30E+08	1.30E+08	34516	2	13137	uc007clx.2	218081	ENSMUSG000	Cd55b	CD55 molecule, decay accelerating factor for complement B
chr1	130205166	130205395	230	231	130205271	26	12.14079	4.85292	8.87838	Adipose_R1_peak_1	Intron (uc007c	1	1.30E+08	1.30E+08	34516	2	13137	uc007clx.2	217648	ENSMUSG000	Cd55b	CD55 molecule, decay accelerating factor for complement B
chr1	131375290	131375558	269	270	131375423	50	34.50784	9.16663	30.72138	Adipose_R1_peak_1	Intron (uc007c	1	1.31E+08	1.31E+08	38083	2	14270	uc007cni.1	-42220	ENSMUSG000	Srgap2	SLIT-ROBO Rho GTPase activating protein 2
chr1	131375302	131375505	204	205	131375427	44	15.55653	4.1498	12.05625	Adipose_R2_peak_1	Intron (uc007c	1	1.31E+08	1.31E+08	38083	2	14270	uc007cni.1	-42232	ENSMUSG000	Srgap2	SLIT-ROBO Rho GTPase activating protein 2
chr1	131680630	131680904	275	276	131680752	44	28.34544	8.08821	24.66239	Adipose_R1_peak_1	Distal Interger	1	1.32E+08	1.32E+08	26745	1	226421	uc007cnp.2	-7791	ENSMUSG000	Rab7b	RAB7B, member RAS oncogene family
chr1	131680695	131680829	135	136	131680763	32	8.29282	3.04319	5.18352	Adipose_R2_peak_1	Distal Interger	1	1.32E+08	1.32E+08	26745	1	226421	uc007cnp.2	-7866	ENSMUSG000	Rab7b	RAB7B, member RAS oncogene family
chr1	131681507	131681740	234	235	131681613	45	16.22858	4.24202	12.70278	Adipose_R2_peak_1	Distal Interger	1	1.32E+08	1.32E+08	26745	1	226421	uc007cnp.2	-6955	ENSMUSG000	Rab7b	RAB7B, member RAS oncogene family
chr1	131681517	131681747	231	232	131681633	41	24.45004	7.19752	20.84242	Adipose_R1_peak_1	Distal Interger	1	1.32E+08	1.32E+08	26745	1	226421	uc007cnp.2	-6948	ENSMUSG000	Rab7b	RAB7B, member RAS oncogene family
chr1	131686206	131686525	320	321	131686343	62	47.71039	11.32349	43.751	Adipose_R1_peak_1	Promoter (2-3	1	1.32E+08	1.32E+08	26745	1	226421	uc007cnp.2	-2170	ENSMUSG000	Rab7b	RAB7B, member RAS oncogene family
chr1	131686211	131686485	275	276	131686323	68	33.91425	6.36302	29.97245	Adipose_R2_peak_1	Promoter (2-3	1	1.32E+08	1.32E+08	26745	1	226421	uc007cnp.2	-2210	ENSMUSG000	Rab7b	RAB7B, member RAS oncogene family
chr1	131694371	131694466	96	97	131694415	26	5.31252	2.48988	2.47999	Adipose_R2_peak_1	Intron (uc007c	1	1.32E+08	1.32E+08	26745	1	226421	uc007cnp.2	5676	ENSMUSG000	Rab7b	RAB7B, member RAS oncogene family
chr1	132131158	132131695	538	539	132131372	46	30.36364	8.44768	26.64488	Adipose_R1_peak_1	Intron (uc007c	1	1.32E+08	1.32E+08	26139	2	18557	uc007coh.2	7990	ENSMUSG000	Cdk18	cyclin-dependent kinase 18
chr1	132131315	132131673	359	360	132131534	33	8.83596	3.1354	5.68652	Adipose_R2_peak_1	Intron (uc007c	1	1.32E+08	1.32E+08	26139	2	18557	uc007coh.2	8012	ENSMUSG000	Cdk18	cyclin-dependent kinase 18
chr1	133594628	133596111	1484	1485	133594684	55	9.84745	2.56386	6.6451	Adipose_R2_peak_1	Distal Interger	1	1.34E+08	1.34E+08	6410	2	20643	uc007cql.2	14169	ENSMUSG000	Snrpe	small nuclear ribonucleoprotein E
chr1	133594682	133595398	717	718	133594769	43	12.2396	3.43393	8.97653	Adipose_R1_peak_1	Distal Interger	1	1.34E+08	1.34E+08	6410	2	20643	uc007cql.2	14882	ENSMUSG000	Snrpe	small nuclear ribonucleoprotein E
chr1	134061882	134062739	858	859	134061943	18	6.42934	3.41502	3.48999	Adipose_R1_peak_1	Distal Interger	1	1.34E+08	1.34E+08	4291	2	12227	uc007cre.1	16416	ENSMUSG000	Btg2	B cell translocation gene 2, anti-proliferative
chr1	134063844	134063969	126	127	134063896	29	6.74062	2.76653	3.75989	Adipose_R2_peak_1	Distal Interger	1	1.34E+08	1.34E+08	4291	2	12227	uc007cre.1	15186	ENSMUSG000	Btg2	B cell translocation gene 2, anti-proliferative
chr1	134066139	134066272	134	135	134066145	25	4.86612	2.39766	2.09018	Adipose_R2_peak_1	Distal Interger	1	1.34E+08	1.34E+08	4291	2	12227	uc007cre.1	12883	ENSMUSG000	Btg2	B cell translocation gene 2, anti-proliferative
chr1	134068954	134069122	169	170	134069019	29	6.74062	2.76653	3.75989	Adipose_R2_peak_1	Distal Interger	1	1.34E+08	1.34E+08	4291	2	12227	uc007cre.1	10033	ENSMUSG000	Btg2	B cell translocation gene 2, anti-proliferative
chr1	134070841	134070934	94	95	134070895	27	5.77405	2.5821	2.88921	Adipose_R2_peak_1	Distal Interger	1	1.34E+08	1.34E+08	4291	2	12227	uc007cre.1	8221	ENSMUSG000	Btg2	B cell translocation gene 2, anti-proliferative
chr1	134077464	134077725	262	263	134077529	31	7.7623	2.95097	4.69388	Adipose_R2_peak_1	Promoter (1-2	1	1.34E+08	1.34E+08	4291	2	12227	uc007cre.1	1430	ENSMUSG000	Btg2	B cell translocation gene 2, anti-proliferative
chr1	134077828	134077941	114	115	134077870	25	4.86612	2.39766	2.09018	Adipose_R2_peak_1	Promoter (1-2	1	1.34E+08	1.34E+08	4291	2	12227	uc007cre.1	1214	ENSMUSG000	Btg2	B cell translocation gene 2, anti-proliferative
chr1	134078900	134079047	148	149	134078983	27	5.77405	2.5821	2.88921	Adipose_R2_peak_1	Promoter (<=	1	1.34E+08	1.34E+08	4291	2	12227	uc007cre.1	108	ENSMUSG000	Btg2	B cell translocation gene 2, anti-proliferative
chr1	134082584	134082903	320	321	134082627	27	5.77405	2.5821	2.88921	Adipose_R2_peak_1	Distal Interger	1	1.34E+08	1.34E+08	4291	2	12227	uc007cre.1	-3429	ENSMUSG000	Btg2	B cell translocation gene 2, anti-proliferative
chr1	134083785	134083941	157	158	134083877	22	9.13959	4.13397	6.01526	Adipose_R1_peak_1	Distal Interger	1	1.34E+08	1.34E+08	4291	2	12227	uc007cre.1	-4630	ENSMUSG000	Btg2	B cell translocation gene 2, anti-proliferative
chr1	134083859	134084030	172	173	134083907	26	5.31252	2.48988	2.47999	Adipose_R2_peak_1	Distal Interger	1	1.34E+08	1.34E+08	4291	2	12227	uc007cre.1	-4704	ENSMUSG000	Btg2	B cell translocation gene 2, anti-proliferative
chr1	134085682	134085880	199	200	134085784	16	5.20017	3.05554	2.38734	Adipose_R1_peak_1	Distal Interger	1	1.34E+08	1.34E+08	4291	2	12227	uc007cre.1	-6527	ENSMUSG000	Btg2	B cell translocation gene 2, anti-proliferative
chr1	134087204	134087355	152	153	134087239	29	6.74062	2.76653	3.75989	Adipose_R2_peak_1	Distal Interger	1	1.34E+08	1.34E+08	4291	2	12227	uc007cre.1	-8049	ENSMUSG000	Btg2	B cell translocation gene 2, anti-proliferative
chr1	134088349	134088528	180	181	134088396	25	4.86612	2.39766	2.09018	Adipose_R2_peak_1	Distal Interger	1	1.34E+08	1.34E+08	4291	2	12227	uc007cre.1	-9194	ENSMUSG000	Btg2	B cell translocation gene 2, anti-proliferative
chr1	134088362	134088505	144	145	134088477	18	6.42934	3.41502	3.48999	Adipose_R1_peak_1	Distal Interger	1	1.34E+08	1.34E+08	4291	2	12227	uc007cre.1	-9207	ENSMUSG000	Btg2	B cell translocation gene 2, anti-proliferative
chr1	134090784	134091010	227	228	134090892	24	10.60668	4.49345	7.40961	Adipose_R1_peak_1	Distal Interger	1	1.34E+08	1.34E+08	4291	2	12227	uc007cre.1	-11629	ENSMUSG000	Btg2	B cell translocation gene 2, anti-proliferative
chr1	134090805	134091043	239	240	134090890	31	7.7623	2.95097	4.69388	Adipose_R2_peak_1	Distal Interger	1	1.34E+08	1.34E+08	4291	2	12227	uc007cre.1	-11650	ENSMUSG000	Btg2	B cell translocation gene 2, anti-proliferative
chr1	134092154	134092409	256	257	134092303	32	8.26309	3.03516	5.17585	Adipose_R2_peak_1	Distal Interger	1	1.34E+08	1.34E+08	4291	2	12227	uc007cre.1	-12999	ENSMUSG000	Btg2	B cell translocation gene 2, anti-proliferative
chr1	134092599	134092825	227	228	134092727	19	7.07687	3.59476	4.08458	Adipose_R1_peak_1	Distal Interger	1	1.34E+08	1.34E+08	4291	2	12227	uc007cre.1	-13444	ENSMUSG000	Btg2	B cell translocation gene 2, anti-proliferative
chr1	134094549	134094799	251	252	134094655	32	8.29282	3.04319	5.18352	Adipose_R2_peak_1	Distal Interger	1	1.34E+08	1.34E+08	4291	2	12227	uc007cre.1	-15394	ENSMUSG000	Btg2	B cell translocation gene 2, anti-proliferative
chr1	134096001	134096097	97	98	134096052	28	6.25023	2.67431	3.31626	Adipose_R2_peak_1	Distal Interger	1	1.34E+08	1.34E+08	40299	1	71884	uc007crf.2	-15145	ENSMUSG000	Chit1	chitinase 1 (chitotriosidase)
chr1	134096075	134096250	176	177	134096139	19	6.91816	3.52644	3.97188	Adipose_R1_peak_1	Distal Interger	1	1.34E+08	1.34E+08	40299	1	71884	uc007crf.2	-14992	ENSMUSG000	Chit1	chitinase 1 (chitotriosidase)
chr1	134097061	134097260	200	201	134097122	27	5.77405	2.5821	2.88921	Adipose_R2_peak_1	Distal Interger	1	1.34E+08	1.34E+08	40299	1	71884	uc007crf.2	-13982	ENSMUSG000	Chit1	chitinase 1 (chitotriosidase)
chr1	134097403	134097643	241	242	134097485	26	5.31252	2.48988	2.47999	Adipose_R2_peak_1	Distal Interger	1	1.34E+08	1.34E+08	40299	1	71884	uc007crf.2	-13599	ENSMUSG000	Chit1	chitinase 1 (chitotriosidase)
chr1	134098059	134098264	206	207	134098182	32	8.29282	3.04319	5.18352	Adipose_R2_peak_1	Distal Interger	1	1.34E+08	1.34E+08	40299	1	71884	uc007crf.2	-12978	ENSMUSG000	Chit1	chitinase 1 (chitotriosidase)
chr1	134127698	134127796	99	100	134127743	31	7.7623	2.95097	4.69388	Adipose_R2_peak_1	Intron (uc007c	1	1.34E+08	1.34E+08	40299	1	71884	uc007crf.2	16456	ENSMUSG000	Chit1	chitinase 1 (chitotriosidase)
chr1	134348587	134348737	151	152	134348650	23	9.86443	4.31371	6.70247	Adipose_R1_peak_1	Intron (uc007c	1	1.34E+08	1.34E+08	15903	2	57439	uc007crq.1	13262	ENSMUSG000	Tmem183a	transmembrane protein 183A

chr1	137063632	137064016	385	386	137063674	28	6.25023	2.67431	3.31626	Adipose_R2_peak_1	Distal Interger	1	1.37E+08	1.37E+08	116865	2	26424	uc007cvc.2	-103184	ENSMUSG000	Nr5a2	nuclear receptor subfamily 5, group A, member 2
chr1	137063847	137064021	175	176	137063911	33	12.46333	4.182	9.19881	Adipose_R1_peak_1	Distal Interger	1	1.37E+08	1.37E+08	116865	2	26424	uc007cvc.2	-103399	ENSMUSG000	Nr5a2	nuclear receptor subfamily 5, group A, member 2
chr1	137067820	137067988	169	170	137067928	32	12.04647	4.14252	8.81093	Adipose_R1_peak_1	Distal Interger	1	1.37E+08	1.37E+08	116865	2	26424	uc007cvc.2	-107372	ENSMUSG000	Nr5a2	nuclear receptor subfamily 5, group A, member 2
chr1	137067864	137068063	200	201	137067950	35	9.16253	3.11468	6.00174	Adipose_R2_peak_1	Distal Interger	1	1.37E+08	1.37E+08	116865	2	26424	uc007cvc.2	-107416	ENSMUSG000	Nr5a2	nuclear receptor subfamily 5, group A, member 2
chr1	137152392	137152883	492	493	137152527	35	19.76029	6.47056	16.26028	Adipose_R1_peak_1	Distal Interger	1	1.37E+08	1.37E+08	116865	2	26424	uc007cvc.2	-191944	ENSMUSG000	Nr5a2	nuclear receptor subfamily 5, group A, member 2
chr1	137275730	137275950	221	222	137275839	41	25.38991	7.54899	21.76305	Adipose_R1_peak_1	Distal Interger	1	1.37E+08	1.37E+08	116865	2	26424	uc007cvc.2	-315282	ENSMUSG000	Nr5a2	nuclear receptor subfamily 5, group A, member 2
chr1	137275794	137275907	114	115	137275838	33	8.83596	3.1354	5.68652	Adipose_R2_peak_1	Distal Interger	1	1.37E+08	1.37E+08	116865	2	26424	uc007cvc.2	-315346	ENSMUSG000	Nr5a2	nuclear receptor subfamily 5, group A, member 2
chr1	137392741	137392913	173	174	137392840	22	9.13959	4.13397	6.01526	Adipose_R1_peak_1	Distal Interger	1	1.37E+08	1.37E+08	116865	2	26424	uc007cvc.2	-432293	ENSMUSG000	Nr5a2	nuclear receptor subfamily 5, group A, member 2
chr1	137411955	137412104	150	151	137412061	20	7.74501	3.7745	4.70467	Adipose_R1_peak_1	Distal Interger	1	1.37E+08	1.37E+08	116865	2	26424	uc007cvc.2	-451507	ENSMUSG000	Nr5a2	nuclear receptor subfamily 5, group A, member 2
chr1	137455729	137455894	166	167	137455802	31	7.7623	2.95097	4.69388	Adipose_R2_peak_1	Distal Interger	1	1.37E+08	1.37E+08	116865	2	26424	uc007cvc.2	-495281	ENSMUSG000	Nr5a2	nuclear receptor subfamily 5, group A, member 2
chr1	137455735	137455877	143	144	137455808	19	7.07687	3.59476	4.08458	Adipose_R1_peak_1	Distal Interger	1	1.37E+08	1.37E+08	116865	2	26424	uc007cvc.2	-495287	ENSMUSG000	Nr5a2	nuclear receptor subfamily 5, group A, member 2
chr1	137531534	137531826	293	294	137531661	38	22.52591	7.00978	18.96	Adipose_R1_peak_1	Distal Interger	1	1.38E+08	1.38E+08	264	1	735252	uc007cve.2	-434629	ENSMUSG000	Mir181a-1	microRNA 181a-1
chr1	137531598	137531729	132	133	137531648	30	7.24477	2.85875	4.21917	Adipose_R2_peak_1	Distal Interger	1	1.38E+08	1.38E+08	264	1	735252	uc007cve.2	-434726	ENSMUSG000	Mir181a-1	microRNA 181a-1
chr1	137700972	137701104	133	134	137701025	18	6.42934	3.41502	3.48999	Adipose_R1_peak_1	Distal Interger	1	1.38E+08	1.38E+08	264	1	735252	uc007cve.2	-265351	ENSMUSG000	Mir181a-1	microRNA 181a-1
chr1	137713442	137713653	212	213	137713555	28	13.73721	5.2124	10.41463	Adipose_R1_peak_1	Distal Interger	1	1.38E+08	1.38E+08	264	1	735252	uc007cve.2	-252802	ENSMUSG000	Mir181a-1	microRNA 181a-1
chr1	138452470	138452620	151	152	138452540	20	7.74501	3.7745	4.70467	Adipose_R1_peak_1	Distal Interger	1	1.38E+08	1.39E+08	135921	2	59125	uc007cvn.2	167137	ENSMUSG000	Nek7	NIMA (never in mitosis gene a)-related expressed kinase 7
chr1	138527498	138527665	168	169	138527595	32	8.29282	3.04319	5.18352	Adipose_R2_peak_1	Intron (uc007c	1	1.38E+08	1.39E+08	135921	2	59125	uc007cvn.2	92092	ENSMUSG000	Nek7	NIMA (never in mitosis gene a)-related expressed kinase 7
chr1	138599011	138599157	147	148	138599077	21	8.43286	3.95423	5.34896	Adipose_R1_peak_1	Intron (uc007c	1	1.38E+08	1.39E+08	135921	2	59125	uc007cvn.2	20600	ENSMUSG000	Nek7	NIMA (never in mitosis gene a)-related expressed kinase 7
chr1	138602103	138602217	115	116	138602211	30	7.24477	2.85875	4.21917	Adipose_R2_peak_1	Intron (uc007c	1	1.38E+08	1.39E+08	135921	2	59125	uc007cvn.2	17540	ENSMUSG000	Nek7	NIMA (never in mitosis gene a)-related expressed kinase 7
chr1	138790987	138791124	138	139	138791062	20	7.74501	3.7745	4.70467	Adipose_R1_peak_1	Distal Interger	1	1.39E+08	1.39E+08	17259	2	16876	uc007cvr.1	51320	ENSMUSG000	Lhx9	LIM homeobox protein 9
chr1	138795088	138795282	195	196	138795164	29	14.55748	5.39214	11.20652	Adipose_R1_peak_1	Distal Interger	1	1.39E+08	1.39E+08	17259	2	16876	uc007cvr.1	47162	ENSMUSG000	Lhx9	LIM homeobox protein 9
chr1	138795101	138795280	180	181	138795165	38	11.72962	3.59649	8.4047	Adipose_R2_peak_1	Distal Interger	1	1.39E+08	1.39E+08	17259	2	16876	uc007cvr.1	47164	ENSMUSG000	Lhx9	LIM homeobox protein 9
chr1	138804808	138804924	117	118	138804863	31	7.7623	2.95097	4.69388	Adipose_R2_peak_1	Distal Interger	1	1.39E+08	1.39E+08	17259	2	16876	uc007cvr.1	37520	ENSMUSG000	Lhx9	LIM homeobox protein 9
chr1	138868298	138868458	161	162	138868327	17	5.80341	3.23528	2.92378	Adipose_R1_peak_1	Distal Interger	1	1.39E+08	1.39E+08	4876	2	69549	uc007cvw.1	-11444	ENSMUSG000	2310009B15Ri	RIKEN cDNA 2310009B15 gene
chr1	138868878	138869030	153	154	138868940	20	7.74501	3.7745	4.70467	Adipose_R1_peak_1	Distal Interger	1	1.39E+08	1.39E+08	4876	2	69549	uc007cvw.1	-12024	ENSMUSG000	2310009B15Ri	RIKEN cDNA 2310009B15 gene
chr1	138869873	138870380	508	509	138869968	28	13.73721	5.2124	10.41463	Adipose_R1_peak_1	Distal Interger	1	1.39E+08	1.39E+08	4876	2	69549	uc007cvw.1	-13019	ENSMUSG000	2310009B15Ri	RIKEN cDNA 2310009B15 gene
chr1	138870092	138870347	256	257	138870254	48	18.29934	4.51867	14.70076	Adipose_R2_peak_1	Distal Interger	1	1.39E+08	1.39E+08	4876	2	69549	uc007cvw.1	-13238	ENSMUSG000	2310009B15Ri	RIKEN cDNA 2310009B15 gene
chr1	138870904	138871117	214	215	138871020	27	12.93148	5.03266	9.63827	Adipose_R1_peak_1	Distal Interger	1	1.39E+08	1.39E+08	4876	2	69549	uc007cvw.1	-14050	ENSMUSG000	2310009B15Ri	RIKEN cDNA 2310009B15 gene
chr1	138870957	138871092	136	137	138871035	29	6.74062	2.76653	3.75989	Adipose_R2_peak_1	Distal Interger	1	1.39E+08	1.39E+08	4876	2	69549	uc007cvw.1	-14103	ENSMUSG000	2310009B15Ri	RIKEN cDNA 2310009B15 gene
chr1	138871818	138872292	475	476	138871957	52	36.63102	9.52611	32.81188	Adipose_R1_peak_1	Distal Interger	1	1.39E+08	1.39E+08	4876	2	69549	uc007cvw.1	-14964	ENSMUSG000	2310009B15Ri	RIKEN cDNA 2310009B15 gene
chr1	138871884	138872129	246	247	138871978	44	15.55653	4.1498	12.05625	Adipose_R2_peak_1	Distal Interger	1	1.39E+08	1.39E+08	4876	2	69549	uc007cvw.1	-15030	ENSMUSG000	2310009B15Ri	RIKEN cDNA 2310009B15 gene
chr1	139545997	139546145	149	150	139546070	23	9.86443	4.31371	6.70247	Adipose_R1_peak_1	Downstream (	1	1.40E+08	1.40E+08	13159	2	50702	uc007cwl.1	14077	ENSMUSG000	Cfhr1	complement factor H-related 1
chr1	139614057	139614209	153	154	139614173	16	5.20017	3.05554	2.38734	Adipose_R1_peak_1	Intron (uc007c	1	1.40E+08	1.40E+08	13159	2	50702	uc007cwl.1	-53835	ENSMUSG000	Cfhr1	complement factor H-related 1
chr1	139619972	139620120	149	150	139620054	21	8.43286	3.95423	5.34896	Adipose_R1_peak_1	Intron (uc007c	1	1.40E+08	1.40E+08	13159	2	50702	uc007cwl.1	-59750	ENSMUSG000	Cfhr1	complement factor H-related 1
chr1	139651181	139651342	162	163	139651281	18	6.42934	3.41502	3.48999	Adipose_R1_peak_1	Distal Interger	1	1.40E+08	1.40E+08	13159	2	50702	uc007cwl.1	-90959	ENSMUSG000	Cfhr1	complement factor H-related 1
chr1	139652911	139653055	145	146	139652973	20	7.74501	3.7745	4.70467	Adipose_R1_peak_1	Distal Interger	1	1.40E+08	1.40E+08	13159	2	50702	uc007cwl.1	-92689	ENSMUSG000	Cfhr1	complement factor H-related 1
chr1	139669474	139669665	192	193	139669572	40	12.96465	3.78093	9.57654	Adipose_R2_peak_1	Distal Interger	1	1.40E+08	1.40E+08	13159	2	50702	uc007cwl.1	-109252	ENSMUSG000	Cfhr1	complement factor H-related 1
chr1	139669475	139669686	212	213	139669580	33	17.97508	6.11109	14.52164	Adipose_R1_peak_1	Distal Interger	1	1.40E+08	1.40E+08	13159	2	50702	uc007cwl.1	-109253	ENSMUSG000	Cfhr1	complement factor H-related 1
chr1	139718815	139719012	198	199	139718921	30	15.39182	5.57188	12.01394	Adipose_R1_peak_1	Intron (uc007c	1	1.40E+08	1.40E+08	83321	2	214403	uc007cwn.2	62227	ENSMUSG000	Gm4788	predicted gene 4788
chr1	139718875	139719001	127	128	139718934	29	6.74062	2.76653	3.75989	Adipose_R2_peak_1	Intron (uc007c	1	1.40E+08	1.40E+08	83321	2	214403	uc007cwn.2	62238	ENSMUSG000	Gm4788	predicted gene 4788
chr1	139719216	139719410	195	196	139719312	37	11.12817	3.50427	7.83613	Adipose_R2_peak_1	Intron (uc007c	1	1.40E+08	1.40E+08	83321	2	214403	uc007cwn.2	61829	ENSMUSG000	Gm4788	predicted gene 4788
chr1	139719229	139719398	170	171	139719320	25	11.36566	4.67319	8.13482	Adipose_R1_peak_1	Intron (uc007c	1	1.40E+08	1.40E+08	83321	2	214403	uc007cwn.2	61841	ENSMUSG000	Gm4788	predicted gene 4788
chr1	139721679	139722089	411	412	139721784	24	10.3979	4.40806	7.23136	Adipose_R1_peak_1	Intron (uc007c	1	1.40E+08	1.40E+08	83321	2	214403	uc007cwn.2	59150	ENSMUSG000	Gm4788	predicted gene 4788
chr1	139722594	139722747	154	155	139722663	28	13.73721	5.2124	10.41463	Adipose_R1_peak_1	Intron (uc007c	1	1.40E+08	1.40E+08	83321	2	214403	uc007cwn.2	58492	ENSMUSG000	Gm4788	predicted gene 4788
chr1	139845626	139845923	298	299	139845778	29	14.55748	5.39214	11.20652	Adipose_R1_peak_1	Intron (uc007c	1	1.40E+08	1.40E+08	48408	2	545366	uc007cwp.2	12776	ENSMUSG000	Cfhr2	complement factor H-related 2
chr1	139845654	139845887	234	235	139845776	36	10.5378	3.41206	7.27949	Adipose_R2_peak_1	Intron (uc007c	1	1.40E+08	1.40E+08	48408	2	545366	uc007cwp.2	12812	ENSMUSG000	Cfhr2	complement factor H-related 2
chr1	139938982	139939145	164	165	139939048	25	11.36566	4.67319	8.13482	Adipose_R1_peak_1	Intron (uc007c	1	1.40E+08	1.40E+08	208630	2	545366	uc007cwq.1	79776	ENSMUSG000	Cfhr2	complement factor H-related 2
chr1	139975022	139975292	271	272	139975141	55	39.87613	10.06532	36.01168	Adipose_R1_peak_1	Intron (uc007c	1	1.40E+08	1.40E+08	208630	2	545366	uc007cwq.1	43629	ENSMUSG000	Cfhr2	complement factor H-related 2
chr1	139975042	139975283	242	243	139975172	45	16.22858	4.24202	12.70278	Adipose_R2_peak_1	Intron (uc007c	1	1.40E+08	1.40E+08	208630	2	545366	uc007cwq.1	43638	ENSMUSG000	Cfhr2	complement factor H-related 2
chr1	140025059	140025408	350	351	140025227	108	92.25082	15.07903	87.95564	Adipose_R1_peak_1	Distal Interger	1	1.40E+08	1.40E+08	208630	2	545366	uc007cwq.1	-6138	ENSMUSG000	Cfhr2	complement factor H-related 2
chr1	140025072	140025393	322	323	140025238	108	72.13875	10.05173	67.7811	Adipose_R2_peak_1	Distal Interger	1	1.40E+08	1.40E+08	208630	2	545366	uc007cwq.1	-6151	ENSMUSG000	Cfhr2	complement factor H-related 2
chr1	140645711	140645870	160	161	140645805	33	8.83596	3.1354	5.68652	Adipose_R2_peak_1	Intron (uc029c	1	1.41E+08	1.41E+08	86227	2	75910	uc029qtp.1	10532	ENSMUSG000	4930590L20Ri	RIKEN cDNA 4930590L20 gene
chr1	140645714	140645912	199	200	140645812	23	9.86443	4.31371	6.70247	Adipose_R1_peak_1	Intron (uc029c	1	1.41E+08	1.41E+08	86227	2	75910	uc029qtp.1	10490	ENSMUSG000	4930590L20Ri	RIKEN cDNA 4930590L20 gene
chr1	140715751	140715968	218	219	140715850	28	13.73721	5.2124	10.41463	Adipose_R1_peak_1	Distal Interger	1	1.41E+08	1.41E+08	86227	2	75910	uc029qtp.1	-59349	ENSMUSG000	4930590L20Ri	RIKEN cDNA 4930590L20 gene
chr1	140715791	140715887	97	98	140715836	26	5.31252	2.48988	2.47999	Adipose_R2_peak_1	Distal Interger	1	1.41E+08	1.41E+08	86227	2	75910	uc029qtp.1	-59389	ENSMUSG000	4930590L20Ri	RIKEN cDNA 4930590L20 gene
chr1	140953246	140953395	150	151	140953321	23	9.86443	4.31371	6.70247	Adipose_R1_peak_1	Distal Interger	1	1.41E+08	1.41E+08	86227	2	75910	uc029qtp.1	-296844	ENSMUSG000	4930590L20Ri	RIKEN cDNA 4930590L20 gene
chr1	140960260	140960566	307	308	140960402	69	55.87963	12.58165	51.83661	Adipose_R1_peak_1	Distal Interger	1	1.41E+08	1.41E+08	86227	2	75910	uc029qtp.1	-303858	ENSMUSG000	4930590L20Ri	RIKEN cDNA 4930590L20 gene
chr1	140960275	140960562	288	289	140960428	65	31.39315	6.08637	27.49408	Adipose_R2_peak_1	Distal Interger	1	1.41E+08	1.41E+08	86227	2	75910	uc029qtp.1	-303873	ENSMUSG000	4930590L20Ri	RIKEN cDNA 4930590L20 gene
chr1	140963429	140963602	174	175	140963492	20	7.74501	3.7745	4.70467	Adipose_R1_peak_1	Distal Interger	1	1.41E+08	1.41E+08	86227	2	75910	uc029qtp.1	-307027	ENSMUSG000	4930590L20Ri	RIKEN cDNA 4930590L20 gene
chr1	140978639	140978752	114	115	140978684	28	6.25023	2.67431	3.31626	Adipose_R2_peak_1	Distal Interger	1	1.41E+08	1.41E+08	86227	2	75910	uc029qtp.1	-322237	ENSMUSG000	4930590L20Ri	RIKEN cDNA 4930590L20 gene
chr1	141191609	141191810	202	203	141191688	25	11.36566	4.67319	8.13482	Adipose_R1_peak_1	Distal Interger	1	1.41E+08	1.41E+08	86227	2	75910	uc029qtp.1	-535207	ENSMUSG000	4930590L20Ri	RIKEN cDNA 4930590L20 gene
chr1	141191622	141191724	103	104	141191673	28	6.25023	2.67431	3.31626	Adipose_R2_peak_1	Distal Interger	1	1.41E+08	1.41E+08	86227	2	75910	uc029qtp.1	-535220	ENSMUSG000	4930590L20Ri	RIKEN cDNA 4930590L20 gene
chr1	141248093	141248294	202	203	141248156	32	17.10103	5.93135	13.67137	Adipose_R1_peak_1	Distal Interger	1	1.41E+08	1.41E+08	86227	2	75910	uc029qtp.1	-591691	ENSMUSG000	4930590L20Ri	RIKEN cDNA 4930590L20 gene
chr1	141248094	141248264	171	172	141248139	40	12.96465	3.78093	9.57654	Adipose_R2_peak_1	Distal Interger	1	1.41E+08	1.41E+08	86227	2	75910	uc029qtp.1	-591692	ENSMUSG000	4930590L20Ri	RIKEN cDNA 4930590L20 gene
chr1	141557744	141557944	201	202	141557841	33	17.97508	6.11109	14.52164	Adipose_R1_peak_1	Distal Interger	1	1.41E+08	1.41E+08	86227	2	75910	uc029qtp.1	-901342	ENSMUSG000	4930590L20Ri	RIKEN cDNA 4930590L20 gene
chr1	141702691	141702900	210	211	141702786	24	10.60668	4.49345	7.40961	Adipose_R1_peak_1	Distal Interger	1	1.41E+08	1.41E+08	86227	2	75910	uc029qtp.1	-1046289	ENSMUSG000	4930590L20Ri	RIKEN cDNA 4930590L20 gene
chr1	141703844	141704041	198	199	141703937	35	14.61661	4.66302	11.26529	Adipose_R1_peak_1	Distal Interger	1	1.41E+08	1.41E+08	86227	2	75910	uc029qtp.1	-1047442	ENSMUSG000	4930590L20Ri	RIKEN cDNA 4930590L20 gene
chr1	141703867	141704038	172	173	141703944	38	11.72962	3.59649	8.4047	Adipose_R2_peak_1	Distal Interger	1	1.41E+08	1.41E+08	86227	2	75910	uc029qtp.1	-1047465	ENSMUSG000	4930590L20Ri	RIKEN cDNA 4930590L20 gene
chr1	141925134	141925367	234	235	141925246	28	13.73721	5.2124	10.41463	Adipose_R1_peak_1	Distal Interger	1	1.41E+08	1.41E+08	86227	2	75910	uc029qtp.1	-1268732	ENSMUSG000	4930590L20Ri	RIKEN cDNA 4930590L20 gene
chr1	141925174	141925328	155	156	141925245	38	11.72962	3.59649	8.4047	Adipose_R2_peak_1	Distal Interger	1	1.41E+08	1.41E+08	86227	2	75910	uc029qtp.1	-1268772	ENSMUSG000	4930590L20Ri	RIKEN cDNA 4930590L20 gene
chr1	142031485	142031624	140	141	142031552	20	7.74501	3.7745	4.70467	Adipose_R1_peak_1	Distal Interger	1	1.41E+08	1.41E+08	86227	2	75910	uc029qtp.1	-1375083	ENSMUSG000	4930590L20Ri	RIKEN cDNA 4930590L20 gene
chr1	142356954	142357107	154	155	142357036	41	13.59774	3.87315	10.1803	Adipose_R2_peak_1	Distal Interger	1	1.44E+08	1.44E+08	9241	1	26878	uc007cwy.2	-1283590	ENSMUSG000	B3galt2	UDP-Gal:betaGlcNAc beta 1,3-galactosyltransferase, polypeptide 2
chr1	142357447	142357794	348	349	142357572	55	22.55115	4.96281	18.83775	Adipose_R2_peak_1	Distal Interger	1	1.44E+08	1.44E+08	9241	1	26878	uc007cwy.2	-1282903	ENSMUSG000	B3galt2	UDP-Gal:betaGlcNAc beta 1,3-galactosyltransferase, polypeptide 2
chr1	142357455	142357808	354	355	142357595	34	12.74512	4.17863	9.47868	Adipose_R1_peak_1	Distal Interger	1	1.44E+08	1.44E+08	9241	1	26878	uc007cwy.2	-1282889	ENSMUSG000	B3galt2	UDP-Gal:betaGlcNAc beta 1,3-galactosyltransferase, polypeptide 2
chr1	142498020	142498224	205	206	142498128	25	10.51708	4.33387	7.34966	Adipose_R1_peak_1	Distal Interger	1	1.44E+08	1.44E+08	9241	1	26878	uc007cwy.2	-1142473	ENSMUSG000	B3galt2	UDP-Gal:betaGlcNAc beta 1,3-galactosyltransferase, polypeptide 2
chr1	142585859	142586002	144	145	142585916	17	5.80341	3.23528	2.92378	Adipose_R1_peak_1	Distal Interger	1	1.44E+08	1.44E+08	9241	1	26878	uc007cwy.2	-1054695	ENSMUSG000	B3galt2	UDP-Gal:betaGlcNAc beta 1,3-galactosyltransferase, polypeptide 2
chr1	142587502	142587752	251	252	142587625	40	24.42476	7.36925	20.81738	Adipose_R1_peak_1	Distal Interger	1	1.44E+08	1.44E+08	9241	1	26878	uc007cwy.2	-1052945	ENSMUSG000	B3galt2	UDP-Gal:betaGlcNAc beta 1,3-galactosyltransferase, polypeptide 2
chr1	142587513	142587760	248	249	142587641	49	19.00719	4.61089	15.38598	Adipose_R2_peak_2	Distal Interger	1	1.44E+08	1.44E+08	9241	1	26878	uc007cwy.2	-1052937	ENSMUSG000	B3galt2	UDP-Gal:betaGlcNAc beta 1,3-galactosyltransferase, polypeptide 2
chr1	142667607	142667807	201	202	142667686	24	10.60668	4.49345	7.40961	Adipose_R1_peak_1	Distal Interger	1	1.44E+08	1.44E+08	9241	1	26878	uc007cwy.2	-972890	ENSMUSG000	B3galt2	UDP-Gal:betaGlcNAc beta 1,3-galactosyltransferase, polypeptide 2
chr1	142672434	142672599	166	167	142672511	24	10.60668	4.49345	7.40961	Adipose_R1_peak_1	Distal Interger	1	1.44E+08	1.44E+08	9241	1	26878	uc007cwy.2	-968098	ENSMUSG000	B3galt2	UDP-Gal:betaGlcNAc beta 1,3-galactosyltransferase, polypeptide 2
chr1	142920806	142921005	200	201	142920808	38	11.72962	3.59649	8.4047	Adipose_R2_peak_2	Distal Interger	1	1.44E+08	1.44E+08	9241	1	26878	uc007cwy.2	-719692	ENSMUSG000	B3galt2	UDP-Gal:betaGlcNAc beta 1,3-galactosyltransferase, polypeptide 2
chr1	142980407	142980569	163	164	142980558	21	8.43286	3.95423	5.34896	Adipose_R1_peak_1	Distal Interger	1	1.44E+08	1.44E+08	9241	1	26878	uc007cwy.2	-660128	ENSMUSG000	B3galt2	UDP-Gal:betaGlcNAc beta 1,3-galactosyltransferase, polypeptide 2
chr1	142980423	142980569	147	148	142980553	26	5.31252	2.48988	2.47999	Adipose_R2_peak_2	Distal Interger	1	1.44E+08	1.44E+08	9241	1	26878	uc007cwy.2	-660128	ENSMUSG000	B3galt2	UDP-Gal:betaGlcNAc beta 1,3-galactosyltransferase, polypeptide 2
chr1	143022392	143022747	356	357	143022595	151	160.9818	24.66265	156.4396	Adipose_R1_peak_1	Distal Interger	1	1.44E+08	1.44E+08	9241	1	26878	uc007cwy.2	-617950	ENSMUSG000	B3galt2	UDP-Gal:betaGlcNAc beta 1,3-galactosyltransferase, polypeptide 2
chr1	143022458	143022735	278	279	143022595	145	113.3126	13.46379	108.7548	Adipose_R2_peak_2	Distal Interger	1	1.44E+08	1.44E+08	9241	1	26878	uc007cwy.2	-617962	ENSMUSG000	B3galt2	UDP-Gal:betaGlcNAc beta 1,3-galactosyltransferase, polypeptide 2
chr1	143023444	143023680	237	238	143023539	40	24.42476	7.36925	20.81738	Adipose_R1_peak_1	Distal Interger	1	1.44E+08	1.44E+08	9241	1	26878	uc007cwy.2	-617017	ENSMUSG000	B3galt2	UDP-Gal:betaGlcNAc beta 1,3-galactosyltransferase, polypeptide 2
chr1	143023462	143023655	194	195	143023533	42	14.2409	3.96536	10.79479	Adipose_R2_peak_2	Distal Interger	1	1.44E+08	1.44E+08	9241	1	26878	uc007cwy.2	-617042	ENSMUSG000	B3galt2	UDP-Gal:betaGlcNAc beta 1,3-galactosyltransferase, polypeptide 2
chr1	143033292	143033501	210	211	143033410	34	18.86162	6.29083	15.38482	Adipose_R1_peak_1	Distal Interger	1	1.44E+08	1.44E+08	9241	1	26878	uc007cwy.2	-607196	ENSMUSG000	B3galt2	UDP-Gal:betaGlcNAc beta 1,3-galactosyltransferase, polypeptide 2
chr1	143033316	143033475	160	161	143033413	31	7.7623	2.95097	4.69388	Adipose_R2_peak_2	Distal Interger	1	1.44E+08	1.44E+08	9241	1	26878	uc007cwy.2	-607222	ENSMUSG000	B3galt2	UDP-Gal:betaGlcNAc beta 1,3-galactosyltransferase, polypeptide 2
chr1	143114856	143115116	261	262	143114989	44	28.34544	8.08821	24.66239	Adipose_R1_peak_1	Distal Interger	1	1.44E+08	1.44E+08	9241	1	26878	uc007cwy.2	-525581	ENSMUSG000	B3galt2	UDP-Gal:betaGlcNAc beta 1,3-galactosyltransferase, polypeptide 2
chr1	143114858	143115039	182	183	143114968	42	14.2409	3.96536	10.79479	Adipose_R2_peak_2	Distal Interger	1	1.44E+08	1.44E+08	9241	1	26878	uc007cwy.2	-525658	ENSMUSG000	B3galt2	UDP-Gal:betaGlcNAc beta 1,3-galactosyltransferase, polypeptide 2
chr1	143164951	143165145	195	196	143165031	25	11.36566	4.67319	8.13482	Adipose_R1_peak_1	Distal Interger	1	1.44E+08	1.44E+08	9241	1	26878	uc007cwy.2	-475552	ENSMUSG000	B3galt2	UDP-Gal:betaGlcNAc beta 1,3-galactosyltransferase, polypeptide 2
chr1	143242519	143242709	191	192	143242598	23	9.86443	4.31371	6.70247	Adipose_R1_peak_1	Distal Interger	1	1.44E+08	1.44E+08	9241	1	26878	uc007cwy.2	-397988	ENSMUSG000	B3galt2	UDP-Gal:betaGlcNAc beta 1,3-galactosyltransferase, polypeptide 2
chr1	143393724	143394057	334	335	143393912	79	68.06648	14.37903	63.91661	Adipose_R1_peak_1	Distal Interger	1	1.44E+08	1.44E+08	9241	1	26878	uc007cwy.2	-246640	ENSMUSG000	B3galt2	UDP-Gal:betaGlcNAc beta 1,3-galactosyltransferase, polypeptide 2

chr1	143393782	143394035	254	255	143393910	49	19.00719	4.61089	15.38598	Adipose_R2_peak_2	Distal Intergen	1	1.44E+08	1.44E+08	9241	1	26878	uc007cwy.2	-246662	ENSMUSG000	B3galt2	UDP-Gal:betaGlcNAc beta 1,3-galactosyltransferase, polypeptide 2
chr1	143394900	143395139	240	241	143395020	30	15.39182	5.57188	12.01394	Adipose_R1_peak_1	Distal Intergen	1	1.44E+08	1.44E+08	9241	1	26878	uc007cwy.2	-245558	ENSMUSG000	B3galt2	UDP-Gal:betaGlcNAc beta 1,3-galactosyltransferase, polypeptide 2
chr1	143394955	143395074	120	121	143395027	29	6.74062	2.76653	3.75989	Adipose_R2_peak_2	Distal Intergen	1	1.44E+08	1.44E+08	9241	1	26878	uc007cwy.2	-245623	ENSMUSG000	B3galt2	UDP-Gal:betaGlcNAc beta 1,3-galactosyltransferase, polypeptide 2
chr1	143511044	143511296	253	254	143511153	50	34.50784	9.16663	30.72138	Adipose_R1_peak_1	Distal Intergen	1	1.44E+08	1.44E+08	9241	1	26878	uc007cwy.2	-129401	ENSMUSG000	B3galt2	UDP-Gal:betaGlcNAc beta 1,3-galactosyltransferase, polypeptide 2
chr1	143511061	143511240	180	181	143511151	34	9.39139	3.22762	6.20474	Adipose_R2_peak_2	Distal Intergen	1	1.44E+08	1.44E+08	9241	1	26878	uc007cwy.2	-129457	ENSMUSG000	B3galt2	UDP-Gal:betaGlcNAc beta 1,3-galactosyltransferase, polypeptide 2
chr1	143545497	143545646	150	151	143545560	19	7.07687	3.59476	4.08458	Adipose_R1_peak_1	Distal Intergen	1	1.44E+08	1.44E+08	9241	1	26878	uc007cwy.2	-95051	ENSMUSG000	B3galt2	UDP-Gal:betaGlcNAc beta 1,3-galactosyltransferase, polypeptide 2
chr1	143579968	143580131	164	165	143580098	17	5.80341	3.23528	2.92378	Adipose_R1_peak_1	Distal Intergen	1	1.44E+08	1.44E+08	9241	1	26878	uc007cwy.2	-60566	ENSMUSG000	B3galt2	UDP-Gal:betaGlcNAc beta 1,3-galactosyltransferase, polypeptide 2
chr1	143711608	143711881	274	275	143711753	53	37.70483	9.70585	33.87	Adipose_R1_peak_1	Distal Intergen	1	1.44E+08	1.44E+08	99618	2	214498	uc007cwx.2	-8715	ENSMUSG000	Cdc73	cell division cycle 73, Paf1/RNA polymerase II complex component
chr1	143711644	143711858	215	216	143711754	53	21.92215	4.97976	18.21755	Adipose_R2_peak_2	Distal Intergen	1	1.44E+08	1.44E+08	99618	2	214498	uc007cwx.2	-8751	ENSMUSG000	Cdc73	cell division cycle 73, Paf1/RNA polymerase II complex component
chr1	143862235	143862670	436	437	143862497	69	55.87963	12.58165	51.83661	Adipose_R1_peak_1	Distal Intergen	1	1.44E+08	1.44E+08	30189	1	56207	uc007cxe.2	84957	ENSMUSG000	Uchl5	ubiquitin carboxyl-terminal esterase L5
chr1	143862358	143862643	286	287	143862500	59	26.5291	5.53306	22.7188	Adipose_R2_peak_2	Distal Intergen	1	1.44E+08	1.44E+08	30189	1	56207	uc007cxe.2	85080	ENSMUSG000	Uchl5	ubiquitin carboxyl-terminal esterase L5
chr1	143895564	143895837	274	275	143895687	45	23.3927	6.22253	19.82251	Adipose_R1_peak_1	Distal Intergen	1	1.44E+08	1.44E+08	4812	2	19735	uc007cxg.2	108312	ENSMUSG000	Rgs2	regulator of G-protein signaling 2
chr1	143895574	143895811	238	239	143895701	50	19.72356	4.7031	16.08069	Adipose_R2_peak_2	Distal Intergen	1	1.44E+08	1.44E+08	4812	2	19735	uc007cxg.2	108338	ENSMUSG000	Rgs2	regulator of G-protein signaling 2
chr1	143966610	143966888	279	280	143966756	66	52.33981	12.04244	48.33179	Adipose_R1_peak_1	Distal Intergen	1	1.44E+08	1.44E+08	4812	2	19735	uc007cxg.2	37261	ENSMUSG000	Rgs2	regulator of G-protein signaling 2
chr1	143966614	143966871	258	259	143966759	64	30.56565	5.99415	26.6807	Adipose_R2_peak_2	Distal Intergen	1	1.44E+08	1.44E+08	4812	2	19735	uc007cxg.2	37278	ENSMUSG000	Rgs2	regulator of G-protein signaling 2
chr1	144067520	144067675	156	157	144067599	23	9.86443	4.31371	6.70247	Adipose_R1_peak_1	Distal Intergen	1	1.44E+08	1.44E+08	4812	2	19735	uc007cxg.2	-63371	ENSMUSG000	Rgs2	regulator of G-protein signaling 2
chr1	144294386	144294600	215	216	144294505	104	96.79909	17.50217	92.48058	Adipose_R1_peak_1	Distal Intergen	1	1.44E+08	1.44E+08	4436	2	50778	uc007cxj.2	-45282	ENSMUSG000	Rgs1	regulator of G-protein signaling 1
chr1	144294391	144294600	210	211	144294519	93	56.90635	8.66847	52.67918	Adipose_R2_peak_2	Distal Intergen	1	1.44E+08	1.44E+08	4436	2	50778	uc007cxj.2	-45287	ENSMUSG000	Rgs1	regulator of G-protein signaling 1
chr1	144325450	144325610	161	162	144325535	28	11.34507	4.30468	8.13482	Adipose_R1_peak_1	Distal Intergen	1	1.44E+08	1.44E+08	4436	2	50778	uc007cxj.2	-76346	ENSMUSG000	Rgs1	regulator of G-protein signaling 1
chr1	144386405	144386674	270	271	144386548	54	38.78658	9.88558	34.93639	Adipose_R1_peak_1	Distal Intergen	1	1.44E+08	1.44E+08	4436	2	50778	uc007cxj.2	-137301	ENSMUSG000	Rgs1	regulator of G-protein signaling 1
chr1	144386430	144386622	193	194	144386534	42	14.2409	3.96536	10.79479	Adipose_R2_peak_2	Distal Intergen	1	1.44E+08	1.44E+08	4436	2	50778	uc007cxj.2	-137326	ENSMUSG000	Rgs1	regulator of G-protein signaling 1
chr1	144396648	144396812	165	166	144396722	19	7.07687	3.59476	4.08458	Adipose_R1_peak_1	Distal Intergen	1	1.44E+08	1.44E+08	4436	2	50778	uc007cxj.2	-147544	ENSMUSG000	Rgs1	regulator of G-protein signaling 1
chr1	144458391	144458703	313	314	144458519	27	12.93148	5.03266	9.63827	Adipose_R1_peak_1	Distal Intergen	1	1.45E+08	1.45E+08	47978	2	624910	uc056yee.1	108964	ENSMUSG000	Rgs21	regulator of G-protein signalling 21
chr1	144458430	144458697	268	269	144458530	36	10.5378	3.41206	7.27949	Adipose_R2_peak_2	Distal Intergen	1	1.45E+08	1.45E+08	47978	2	624910	uc056yee.1	108970	ENSMUSG000	Rgs21	regulator of G-protein signalling 21
chr1	144741496	144741788	293	294	144741631	57	42.07807	10.4248	38.18469	Adipose_R1_peak_1	Distal Intergen	1	1.45E+08	1.45E+08	22581	2	64214	uc007cxl.1	33633	ENSMUSG000	Rgs18	regulator of G-protein signaling 18
chr1	144741538	144741768	231	232	144741646	45	16.22858	4.24202	12.70278	Adipose_R2_peak_2	Distal Intergen	1	1.45E+08	1.45E+08	22581	2	64214	uc007cxl.1	33653	ENSMUSG000	Rgs18	regulator of G-protein signaling 18
chr1	144742845	144743136	292	293	144742969	71	58.27042	12.94113	54.20487	Adipose_R1_peak_1	Distal Intergen	1	1.45E+08	1.45E+08	22581	2	64214	uc007cxl.1	32285	ENSMUSG000	Rgs18	regulator of G-protein signaling 18
chr1	144742859	144743075	217	218	144742971	45	16.22858	4.24202	12.70278	Adipose_R2_peak_2	Distal Intergen	1	1.45E+08	1.45E+08	22581	2	64214	uc007cxl.1	32346	ENSMUSG000	Rgs18	regulator of G-protein signaling 18
chr1	144881893	144882189	297	298	144882082	37	21.59274	6.83004	18.04766	Adipose_R1_peak_1	Distal Intergen	1	1.45E+08	1.45E+08	22581	2	64214	uc007cxl.1	-106472	ENSMUSG000	Rgs18	regulator of G-protein signaling 18
chr1	144881977	144882193	217	218	144882095	52	21.18119	4.88754	17.49655	Adipose_R2_peak_2	Distal Intergen	1	1.45E+08	1.45E+08	22581	2	64214	uc007cxl.1	-106556	ENSMUSG000	Rgs18	regulator of G-protein signaling 18
chr1	144987983	144988149	167	168	144988057	19	7.07687	3.59476	4.08458	Adipose_R1_peak_1	Distal Intergen	1	1.45E+08	1.45E+08	22581	2	64214	uc007cxl.1	-212562	ENSMUSG000	Rgs18	regulator of G-protein signaling 18
chr1	145110903	145111113	211	212	145110965	19	7.07687	3.59476	4.08458	Adipose_R1_peak_1	Distal Intergen	1	1.45E+08	1.45E+08	22581	2	64214	uc007cxl.1	-335482	ENSMUSG000	Rgs18	regulator of G-protein signaling 18
chr1	145110943	145111143	201	202	145111057	28	6.25023	2.67431	3.31626	Adipose_R2_peak_2	Distal Intergen	1	1.45E+08	1.45E+08	22581	2	64214	uc007cxl.1	-335522	ENSMUSG000	Rgs18	regulator of G-protein signaling 18
chr1	145176146	145176286	141	142	145176213	23	9.86443	4.31371	6.70247	Adipose_R1_peak_1	Distal Intergen	1	1.45E+08	1.45E+08	22581	2	64214	uc007cxl.1	-400725	ENSMUSG000	Rgs18	regulator of G-protein signaling 18
chr1	145442607	145442788	182	183	145442651	29	6.74062	2.76653	3.75989	Adipose_R2_peak_2	Distal Intergen	1	1.45E+08	1.45E+08	22581	2	64214	uc007cxl.1	-667186	ENSMUSG000	Rgs18	regulator of G-protein signaling 18
chr1	145475574	145475718	145	146	145475652	20	7.74501	3.7745	4.70467	Adipose_R1_peak_1	Distal Intergen	1	1.45E+08	1.45E+08	22581	2	64214	uc007cxl.1	-700153	ENSMUSG000	Rgs18	regulator of G-protein signaling 18
chr1	145733471	145733866	396	397	145733708	76	38.16592	6.51545	34.32908	Adipose_R1_peak_1	Distal Intergen	1	1.46E+08	1.47E+08	406807	1	215378	uc007cxo.2	-761800	ENSMUSG000	Brinp3	bone morphogenetic protein/retinoic acid inducible neural specific 3
chr1	145733494	145733852	359	360	145733601	53	16.89356	3.88791	13.35463	Adipose_R2_peak_2	Distal Intergen	1	1.46E+08	1.47E+08	406807	1	215378	uc007cxo.2	-761814	ENSMUSG000	Brinp3	bone morphogenetic protein/retinoic acid inducible neural specific 3
chr1	145819981	145820179	199	200	145820072	24	10.60668	4.49345	7.40961	Adipose_R1_peak_1	Distal Intergen	1	1.46E+08	1.47E+08	406807	1	215378	uc007cxo.2	-675487	ENSMUSG000	Brinp3	bone morphogenetic protein/retinoic acid inducible neural specific 3
chr1	145821636	145821816	181	182	145821749	25	11.36566	4.67319	8.13482	Adipose_R1_peak_1	Distal Intergen	1	1.46E+08	1.47E+08	406807	1	215378	uc007cxo.2	-673850	ENSMUSG000	Brinp3	bone morphogenetic protein/retinoic acid inducible neural specific 3
chr1	145853181	145853350	170	171	145853289	22	9.13959	4.13397	6.01526	Adipose_R1_peak_1	Distal Intergen	1	1.46E+08	1.47E+08	406807	1	215378	uc007cxo.2	-642316	ENSMUSG000	Brinp3	bone morphogenetic protein/retinoic acid inducible neural specific 3
chr1	145965990	145966114	125	126	145966034	29	6.74062	2.76653	3.75989	Adipose_R2_peak_2	Distal Intergen	1	1.46E+08	1.47E+08	406807	1	215378	uc007cxo.2	-529552	ENSMUSG000	Brinp3	bone morphogenetic protein/retinoic acid inducible neural specific 3
chr1	146009190	146009438	249	250	146009306	41	25.38991	7.54899	21.76305	Adipose_R1_peak_1	Distal Intergen	1	1.46E+08	1.47E+08	406807	1	215378	uc007cxo.2	-486228	ENSMUSG000	Brinp3	bone morphogenetic protein/retinoic acid inducible neural specific 3
chr1	146023233	146023384	152	153	146023303	20	7.74501	3.7745	4.70467	Adipose_R1_peak_1	Distal Intergen	1	1.46E+08	1.47E+08	406807	1	215378	uc007cxo.2	-472282	ENSMUSG000	Brinp3	bone morphogenetic protein/retinoic acid inducible neural specific 3
chr1	146023235	146023386	152	153	146023314	33	8.83596	3.1354	5.68652	Adipose_R2_peak_2	Distal Intergen	1	1.46E+08	1.47E+08	406807	1	215378	uc007cxo.2	-472280	ENSMUSG000	Brinp3	bone morphogenetic protein/retinoic acid inducible neural specific 3
chr1	146447870	146448403	534	535	146447971	27	12.93148	5.03266	9.63827	Adipose_R1_peak_1	Intron (uc007c	1	1.46E+08	1.47E+08	406807	1	215378	uc007cxo.2	-47263	ENSMUSG000	Brinp3	bone morphogenetic protein/retinoic acid inducible neural specific 3
chr1	146448216	146448391	176	177	146448285	32	8.29282	3.04319	5.18352	Adipose_R2_peak_2	Intron (uc007c	1	1.46E+08	1.47E+08	406807	1	215378	uc007cxo.2	-47275	ENSMUSG000	Brinp3	bone morphogenetic protein/retinoic acid inducible neural specific 3
chr1	146451519	146451664	146	147	146451581	19	7.07687	3.59476	4.08458	Adipose_R1_peak_1	Intron (uc007c	1	1.46E+08	1.47E+08	406807	1	215378	uc007cxo.2	-44002	ENSMUSG000	Brinp3	bone morphogenetic protein/retinoic acid inducible neural specific 3
chr1	146726097	146726381	285	286	146726210	27	5.77405	2.5821	2.88921	Adipose_R2_peak_2	Intron (uc007c	1	1.46E+08	1.47E+08	19817	1	215378	uc007cxr.1	228359	ENSMUSG000	Brinp3	bone morphogenetic protein/retinoic acid inducible neural specific 3
chr1	146727922	146728469	548	549	146728083	32	8.29282	3.04319	5.18352	Adipose_R2_peak_2	Intron (uc007c	1	1.46E+08	1.47E+08	19817	1	215378	uc007cxr.1	230184	ENSMUSG000	Brinp3	bone morphogenetic protein/retinoic acid inducible neural specific 3
chr1	146728662	146729033	372	373	146728954	31	7.7623	2.95097	4.69388	Adipose_R2_peak_2	Intron (uc007c	1	1.46E+08	1.47E+08	19817	1	215378	uc007cxr.1	230924	ENSMUSG000	Brinp3	bone morphogenetic protein/retinoic acid inducible neural specific 3
chr1	146729154	146729247	94	95	146729190	25	4.86612	2.39766	2.09018	Adipose_R2_peak_2	Intron (uc007c	1	1.46E+08	1.47E+08	19817	1	215378	uc007cxr.1	231416	ENSMUSG000	Brinp3	bone morphogenetic protein/retinoic acid inducible neural specific 3
chr1	146868540	146868711	172	173	146868642	21	8.43286	3.95423	5.34896	Adipose_R1_peak_1	Intron (uc007c	1	1.46E+08	1.47E+08	19817	1	215378	uc007cxr.1	370802	ENSMUSG000	Brinp3	bone morphogenetic protein/retinoic acid inducible neural specific 3
chr1	146906362	146906634	273	274	146906490	61	46.56983	11.14375	42.62347	Adipose_R1_peak_1	Distal Intergen	1	1.46E+08	1.47E+08	19817	1	215378	uc007cxr.1	408624	ENSMUSG000	Brinp3	bone morphogenetic protein/retinoic acid inducible neural specific 3
chr1	146906373	146906625	253	254	146906503	62	28.93052	5.80972	25.0743	Adipose_R2_peak_2	Distal Intergen	1	1.46E+08	1.47E+08	19817	1	215378	uc007cxr.1	408635	ENSMUSG000	Brinp3	bone morphogenetic protein/retinoic acid inducible neural specific 3
chr1	147041235	147041399	165	166	147041320	20	7.74501	3.7745	4.70467	Adipose_R1_peak_1	Distal Intergen	1	1.46E+08	1.47E+08	19817	1	215378	uc007cxr.1	543497	ENSMUSG000	Brinp3	bone morphogenetic protein/retinoic acid inducible neural specific 3
chr1	147285835	147286144	310	311	147285988	91	83.39809	16.53589	79.14825	Adipose_R1_peak_1	Distal Intergen	1	1.46E+08	1.47E+08	19817	1	215378	uc007cxr.1	788097	ENSMUSG000	Brinp3	bone morphogenetic protein/retinoic acid inducible neural specific 3
chr1	147285860	147286138	279	280	147285998	80	44.53636	7.46964	40.44495	Adipose_R2_peak_2	Distal Intergen	1	1.46E+08	1.47E+08	19817	1	215378	uc007cxr.1	788122	ENSMUSG000	Brinp3	bone morphogenetic protein/retinoic acid inducible neural specific 3
chr1	147335012	147335180	169	170	147335094	27	12.93148	5.03266	9.63827	Adipose_R1_peak_1	Distal Intergen	1	1.46E+08	1.47E+08	19817	1	215378	uc007cxr.1	837274	ENSMUSG000	Brinp3	bone morphogenetic protein/retinoic acid inducible neural specific 3
chr1	147335026	147335162	137	138	147335090	33	8.83596	3.1354	5.68652	Adipose_R2_peak_2	Distal Intergen	1	1.46E+08	1.47E+08	19817	1	215378	uc007cxr.1	837288	ENSMUSG000	Brinp3	bone morphogenetic protein/retinoic acid inducible neural specific 3
chr1	147337179	147337440	262	263	147337302	42	26.36523	7.72873	22.71906	Adipose_R1_peak_1	Distal Intergen	1	1.46E+08	1.47E+08	19817	1	215378	uc007cxr.1	839441	ENSMUSG000	Brinp3	bone morphogenetic protein/retinoic acid inducible neural specific 3
chr1	147337190	147337374	185	186	147337297	42	14.2409	3.96536	10.79479	Adipose_R2_peak_2	Distal Intergen	1	1.46E+08	1.47E+08	19817	1	215378	uc007cxr.1	839452	ENSMUSG000	Brinp3	bone morphogenetic protein/retinoic acid inducible neural specific 3
chr1	147595373	147595842	470	471	147595611	107	80.3042	12.06753	76.0739	Adipose_R1_peak_1	Distal Intergen	1	1.46E+08	1.47E+08	19817	1	215378	uc007cxr.1	1097635	ENSMUSG000	Brinp3	bone morphogenetic protein/retinoic acid inducible neural specific 3
chr1	147595382	147595787	406	407	147595607	96	57.23095	8.39233	53.00177	Adipose_R2_peak_2	Distal Intergen	1	1.46E+08	1.47E+08	19817	1	215378	uc007cxr.1	1097644	ENSMUSG000	Brinp3	bone morphogenetic protein/retinoic acid inducible neural specific 3
chr1	147644504	147644704	201	202	147644597	32	17.10103	5.93135	13.67137	Adipose_R1_peak_1	Distal Intergen	1	1.46E+08	1.47E+08	19817	1	215378	uc007cxr.1	1146766	ENSMUSG000	Brinp3	bone morphogenetic protein/retinoic acid inducible neural specific 3
chr1	147668780	147668944	165	166	147668843	20	7.74501	3.7745	4.70467	Adipose_R1_peak_1	Distal Intergen	1	1.46E+08	1.47E+08	19817	1	215378	uc007cxr.1	1171042	ENSMUSG000	Brinp3	bone morphogenetic protein/retinoic acid inducible neural specific 3
chr1	147668791	147668903	113	114	147668850	30	7.24477	2.85875	4.21917	Adipose_R2_peak_2	Distal Intergen	1	1.46E+08	1.47E+08	19817	1	215378	uc007cxr.1	1171053	ENSMUSG000	Brinp3	bone morphogenetic protein/retinoic acid inducible neural specific 3
chr1	147675805	147676065	261	262	147675941	48	32.41826	8.80716	28.66523	Adipose_R1_peak_1	Distal Intergen	1	1.46E+08	1.47E+08	19817	1	215378	uc007cxr.1	1178067	ENSMUSG000	Brinp3	bone morphogenetic protein/retinoic acid inducible neural specific 3
chr1	147675867	147676050	184	185	147675940	39	12.34187	3.68871	8.98501	Adipose_R2_peak_2	Distal Intergen	1	1.46E+08	1.47E+08	19817	1	215378	uc007cxr.1	1178129	ENSMUSG000	Brinp3	bone morphogenetic protein/retinoic acid inducible neural specific 3
chr1	147771727	147771918	192	193	147771842	25	11.36566	4.67319	8.13482	Adipose_R1_peak_1	Distal Intergen	1	1.46E+08	1.47E+08	19817	1	215378	uc007cxr.1	1273989	ENSMUSG000	Brinp3	bone morphogenetic protein/retinoic acid inducible neural specific 3
chr1	147772492	147772885	394	395	147772644	153	140.3243	18.20782	135.8409	Adipose_R1_peak_1	Distal Intergen	1	1.46E+08	1.47E+08	19817	1	215378	uc007cxr.1	1274754	ENSMUSG000	Brinp3	bone morphogenetic protein/retinoic acid inducible neural specific 3
chr1	147772500	147772821	322	323	147772645	150	119.2136	13.92488	114.6368	Adipose_R2_peak_2	Distal Intergen	1	1.46E+08	1.47E+08	19817	1	215378	uc007cxr.1	1274762	ENSMUSG000	Brinp3	bone morphogenetic protein/retinoic acid inducible neural specific 3
chr1	147829138	147829367	230	231	147829247	40	24.42476	7.36925	20.81738	Adipose_R1_peak_1	Distal Intergen	1	1.46E+08	1.47E+08	19817	1	215378	uc007cxr.1	1331400	ENSMUSG000	Brinp3	bone morphogenetic protein/retinoic acid inducible neural specific 3
chr1	147829143	147829314	172	173	147829248	36	10.5378	3.41206	7.27949	Adipose_R2_peak_2	Distal Intergen	1	1.46E+08	1.47E+08	19817	1	215378	uc007cxr.1	1331405	ENSMUSG000	Brinp3	bone morphogenetic protein/retinoic acid inducible neural specific 3
chr1	147989027	147989170	144	145	147989098	22	9.13959	4.13397	6.01526	Adipose_R1_peak_1	Distal Intergen	1	1.46E+08	1.47E+08	19817	1	215378	uc007cxr.1	1491289	ENSMUSG000	Brinp3	bone morphogenetic protein/retinoic acid inducible neural specific 3
chr1	147998206	147998401	196	197	147998310	29	14.55748	5.39214	11.20652	Adipose_R1_peak_1	Distal Intergen	1	1.46E+08	1.47E+08	19817	1	215378	uc007cxr.1	1500468	ENSMUSG000	Brinp3	bone morphogenetic protein/retinoic acid inducible neural specific 3
chr1	147998241	147998358	118	119	147998309	28	6.25023	2.67431	3.31626	Adipose_R2_peak_2	Distal Intergen	1	1.46E+08	1.47E+08	19817	1	215378	uc007cxr.1	1500503	ENSMUSG000	Brinp3	bone morphogenetic protein/retinoic acid inducible neural specific 3
chr1	148080603	148080741	139	140	148080661	18	6.42934	3.41502	3.48999	Adipose_R1_peak_1	Distal Intergen	1	1.46E+08	1.47E+08	19817	1	215378	uc007cxr.1	1582865	ENSMUSG000	Brinp3	bone morphogenetic protein/retinoic acid inducible neural specific 3
chr1	148129479	148129723	245	246	148129605	32	17.10103	5.93135	13.67137	Adipose_R1_peak_1	Distal Intergen	1	1.46E+08	1.47E+08	19817	1	215378	uc007cxr.1	1631741	ENSMUSG000	Brinp3	bone morphogenetic protein/retinoic acid inducible neural specific 3
chr1	148129494	148129675	182	183	148129597	34	9.39139	3.22762	6.20474	Adipose_R2_peak_2	Distal Intergen	1	1.46E+08	1.47E+08	19817	1	215378	uc007cxr.1	1631756	ENSMUSG000	Brinp3	bone morphogenetic protein/retinoic acid inducible neural specific 3
chr1	148129922	148130169	248	249	148130038	34	18.86162	6.29083	15.38482	Adipose_R1_peak_1	Distal Intergen	1	1.46E+08	1.47E+08	19817	1	215378	uc007cxr.1	1632184	ENSMUSG000	Brinp3	bone morphogenetic protein/retinoic acid inducible neural specific 3
chr1	148300348	148300481	134	135	148300419	21	8.43286	3.95423	5.34896	Adipose_R1_peak_1	Distal Intergen	1	1.50E+08	1.50E+08	92821	2	18783	uc056yeg.1	1621958	ENSMUSG000	Pla2g4a	phospholipase A2, group IVA (cytosolic, calcium-dependent)
chr1	148301897	148302155	259	260	148302031	43	27.35048	7.90847	23.68561	Adipose_R1_peak_1	Distal Intergen	1	1.50E+08	1.50E+08	92821	2	18783	uc056yeg.1	1620284	ENSMUSG000	Pla2g4a	phospholipase A2, group IVA (cytosolic, calcium-dependent)
chr1	148301927	148302079	153	154	148302013	37	11.12817	3.50427	7.83613	Adipose_R2_peak_2	Distal Intergen	1	1.50E+08	1.50E+08	92821	2	18783	uc056yeg.1	1620360	ENSMUSG000	Pla2g4a	phospholipase A2, group IVA (cytosolic, calcium-dependent)
chr1	148378808	148379037	230	231	148378895	26	12.14079	4.85292	8.87838	Adipose_R1_peak_1	Distal Intergen	1	1.50E+08	1.50E+08	92821	2	18783	uc056yeg.1	1543402	ENSMUSG000	Pla2g4a	phospholipase A2, group IVA (cytosolic, calcium-dependent)
chr1	148545654	148545808	155	156	148545722	20	7.74501	3.7745	4.70467	Adipose_R1_peak_1	Distal Intergen	1	1.50E+08	1.50E+08	92821	2	18783	uc056yeg.1	1376631	ENSMUSG000	Pla2g4a	phospholipase A2, group IVA (cytosolic, calcium-dependent)
chr1	148554296	148554472	177	178	148554386	30	15.39182	5.57188	12.01394	Adipose_R1_peak_1	Distal Intergen	1	1.50E+08	1.50E+08	92821	2	18783	uc056yeg.1	1367967	ENSMUSG000	Pla2g4a	phospholipase A2, group IVA (cytosolic, calcium-dependent)
chr1	148558112	148558397	286	287	148558253	27	12.93148	5.03266	9.63827	Adipose_R1_peak_1	Distal Intergen	1	1.50E+08	1.50E+08	92821	2	18783	uc056yeg.1	1364042	ENSMUSG000	Pla2g4a	phospholipase A2, group IVA (cytosolic, calcium-dependent)
chr1	148558203	148558405	203	204	148558291	30	7.24477	2.85875	4.21917	Adipose_R2_peak_2	Distal Intergen	1	1.50E+08	1.50E+08	92821	2	18783	uc056yeg.1	1364034	ENSMUSG000	Pla2g4a	phospholipase A2, group IVA (cytosolic, calcium-dependent)
chr1	148578308	148578486	179	180	148578375	33	8.83596	3.1354	5.68652	Adipose_R2_peak_2	Distal Intergen	1	1.50E+08	1.50E+08	92821	2	18783	uc056yeg.1	1343953	ENSMUSG000	Pla2g4a	phospholipase A2, group IVA (cytosolic, calcium-dependent)
chr1	148578319	148578568	250	251	148578437	46	30.36364	8.44768	26.64488	Adipose_R1_peak_1	Distal Intergen	1	1.50E+08	1.50E+08	92821	2	18783	uc056yeg.1	1343871	ENSMUSG000	Pla2g4a	phospholipase A2, group IVA (cytosolic, calcium-dependent)
chr1	148613190	148613495	306	307	148613357	35	19.76029	6.47056	16.26028	Adipose_R1_peak_1	Distal Intergen	1	1.50E+08	1.50E+08	92821	2	18783	uc056yeg.1	1308944	ENSMUSG000	Pla2g4a	phospholipase A2, group IVA (cytosolic, calcium-dependent)
chr1	148619791	148619937	147	148	148619879	20	7.74501	3.7745	4.70467	Adipose_R1_peak_1	Distal Intergen	1	1.50E+08	1.50E+08	92821	2	18783	uc056yeg.1	1302502	ENSMUSG000	Pla2g4a	phospholipase A2, group IVA (cytosolic, calcium-dependent)
chr1	148760824	148761057	234	235	148760956	32	17.10103	5.93135	13.67137	Adipose_R1_peak_1	Distal Intergen	1	1.50E+08	1.50E+08	92821	2	18783	uc056yeg.1	1161382	ENSMUSG000	Pla2g4a	phospholipase A2, group IVA (cytosolic, calcium-dependent)

chr1	148760918	148761024	107	108	148760963	28	6.25023	2.67431	3.31626	Adipose_R2_peak_2	Distal Interger	1	1.50E+08	1.50E+08	92821	2	18783	uc056yeg.1	1161415	ENSMUSG000	Pla2g4a	phospholipase A2, group IVA (cytosolic, calcium-dependent)
chr1	148882453	148882707	255	256	148882584	38	22.52591	7.00978	18.96	Adipose_R1_peak_1	Distal Interger	1	1.50E+08	1.50E+08	92821	2	18783	uc056yeg.1	1039732	ENSMUSG000	Pla2g4a	phospholipase A2, group IVA (cytosolic, calcium-dependent)
chr1	148937780	148937922	143	144	148937870	29	6.74062	2.76653	3.75989	Adipose_R2_peak_2	Distal Interger	1	1.50E+08	1.50E+08	92821	2	18783	uc056yeg.1	984517	ENSMUSG000	Pla2g4a	phospholipase A2, group IVA (cytosolic, calcium-dependent)
chr1	149296919	149297106	188	189	149297025	31	16.23981	5.75161	12.83549	Adipose_R1_peak_1	Distal Interger	1	1.50E+08	1.50E+08	92821	2	18783	uc056yeg.1	625333	ENSMUSG000	Pla2g4a	phospholipase A2, group IVA (cytosolic, calcium-dependent)
chr1	149296951	149297124	174	175	149297028	38	11.72962	3.59649	8.4047	Adipose_R2_peak_2	Distal Interger	1	1.50E+08	1.50E+08	92821	2	18783	uc056yeg.1	625315	ENSMUSG000	Pla2g4a	phospholipase A2, group IVA (cytosolic, calcium-dependent)
chr1	149466506	149466652	147	148	149466576	20	7.74501	3.7745	4.70467	Adipose_R1_peak_1	Distal Interger	1	1.50E+08	1.50E+08	92821	2	18783	uc056yeg.1	455787	ENSMUSG000	Pla2g4a	phospholipase A2, group IVA (cytosolic, calcium-dependent)
chr1	149507211	149507368	158	159	149507285	36	10.5378	3.41206	7.27949	Adipose_R2_peak_2	Distal Interger	1	1.50E+08	1.50E+08	92821	2	18783	uc056yeg.1	415071	ENSMUSG000	Pla2g4a	phospholipase A2, group IVA (cytosolic, calcium-dependent)
chr1	149604376	149604564	189	190	149604446	42	14.2409	3.96536	10.79479	Adipose_R2_peak_2	Distal Interger	1	1.50E+08	1.50E+08	92821	2	18783	uc056yeg.1	317875	ENSMUSG000	Pla2g4a	phospholipase A2, group IVA (cytosolic, calcium-dependent)
chr1	149604383	149604572	190	191	149604464	31	16.23981	5.75161	12.83549	Adipose_R1_peak_1	Distal Interger	1	1.50E+08	1.50E+08	92821	2	18783	uc056yeg.1	317867	ENSMUSG000	Pla2g4a	phospholipase A2, group IVA (cytosolic, calcium-dependent)
chr1	149749766	149750052	287	288	149749967	31	16.23981	5.75161	12.83549	Adipose_R1_peak_1	Distal Interger	1	1.50E+08	1.50E+08	92821	2	18783	uc056yeg.1	172387	ENSMUSG000	Pla2g4a	phospholipase A2, group IVA (cytosolic, calcium-dependent)
chr1	149979499	149979685	187	188	149979563	25	11.36566	4.67319	8.13482	Adipose_R1_peak_1	Distal Interger	1	1.50E+08	1.50E+08	131672	2	18783	uc007cxt.2	-18209	ENSMUSG000	Pla2g4a	phospholipase A2, group IVA (cytosolic, calcium-dependent)
chr1	150238659	150238852	194	195	150238728	24	10.60668	4.49345	7.40961	Adipose_R1_peak_1	Distal Interger	1	1.50E+08	1.50E+08	5906	2	1.03E+08	uc033fmh.1	-73711	ENSMUSG000	Ptgs2os2	prostaglandin-endoperoxide synthase 2, opposite strand 2
chr1	150238670	150238826	157	158	150238734	33	8.83596	3.1354	5.68652	Adipose_R2_peak_2	Distal Interger	1	1.50E+08	1.50E+08	5906	2	1.03E+08	uc033fmh.1	-73722	ENSMUSG000	Ptgs2os2	prostaglandin-endoperoxide synthase 2, opposite strand 2
chr1	150275431	150275756	326	327	150275600	91	83.39809	16.53589	79.14825	Adipose_R1_peak_1	Distal Interger	1	1.50E+08	1.50E+08	14487	1	20028	uc007cxx.2	-43661	ENSMUSG000	Pdc	phosducin
chr1	150275463	150275747	285	286	150275599	73	38.23935	6.82411	34.23139	Adipose_R2_peak_2	Distal Interger	1	1.50E+08	1.50E+08	14487	1	20028	uc007cxx.2	-43670	ENSMUSG000	Pdc	phosducin
chr1	150297240	150297405	166	167	150297288	36	10.5378	3.41206	7.27949	Adipose_R2_peak_2	Distal Interger	1	1.50E+08	1.50E+08	14487	1	20028	uc007cxx.2	-22012	ENSMUSG000	Pdc	phosducin
chr1	150297676	150297905	230	231	150297784	39	23.47001	7.18952	19.88325	Adipose_R1_peak_1	Distal Interger	1	1.50E+08	1.50E+08	14487	1	20028	uc007cxx.2	-21512	ENSMUSG000	Pdc	phosducin
chr1	150297686	150297867	182	183	150297787	41	13.59774	3.87315	10.1803	Adipose_R2_peak_2	Distal Interger	1	1.50E+08	1.50E+08	14487	1	20028	uc007cxx.2	-21550	ENSMUSG000	Pdc	phosducin
chr1	150340606	150340779	174	175	150340677	27	12.93148	5.03266	9.63827	Adipose_R1_peak_1	Intron (uc029c	1	1.50E+08	1.50E+08	26091	2	75584	uc029qty.1	5992	ENSMUSG000	2310030A07R	RIKEN cDNA 2310030A07 gene
chr1	150342010	150342167	158	159	150342072	22	9.13959	4.13397	6.01526	Adipose_R1_peak_1	Intron (uc029c	1	1.50E+08	1.50E+08	26091	2	75584	uc029qty.1	4604	ENSMUSG000	2310030A07R	RIKEN cDNA 2310030A07 gene
chr1	150343950	150344210	261	262	150344109	65	51.17258	11.8627	47.17609	Adipose_R1_peak_1	Promoter (2-3	1	1.50E+08	1.50E+08	26091	2	75584	uc029qty.1	2561	ENSMUSG000	2310030A07R	RIKEN cDNA 2310030A07 gene
chr1	150343984	150344180	197	198	150344125	58	25.74274	5.44085	21.94821	Adipose_R2_peak_2	Promoter (2-3	1	1.50E+08	1.50E+08	26091	2	75584	uc029qty.1	2591	ENSMUSG000	2310030A07R	RIKEN cDNA 2310030A07 gene
chr1	150358569	150358836	268	269	150358688	41	25.38991	7.54899	21.76305	Adipose_R1_peak_1	Downstream (	1	1.50E+08	1.50E+08	26091	2	75584	uc029qty.1	-11798	ENSMUSG000	2310030A07R	RIKEN cDNA 2310030A07 gene
chr1	151182673	151182958	286	287	151182820	38	9.62931	3.08176	6.43417	Adipose_R2_peak_2	Distal Interger	1	1.51E+08	1.51E+08	6062	1	402734	uc029qtz.1	44639	NA	C730036E19Ri	RIKEN cDNA C730036E19 gene
chr1	151188635	151188765	131	132	151188668	17	5.80341	3.23528	2.92378	Adipose_R1_peak_1	Distal Interger	1	1.51E+08	1.51E+08	6062	1	402734	uc029qtz.1	50601	NA	C730036E19Ri	RIKEN cDNA C730036E19 gene
chr1	151194317	151194471	155	156	151194391	32	8.29282	3.04319	5.18352	Adipose_R2_peak_2	Distal Interger	1	1.51E+08	1.51E+08	6062	1	402734	uc029qtz.1	56283	NA	C730036E19Ri	RIKEN cDNA C730036E19 gene
chr1	151195192	151195366	175	176	151195267	35	9.95877	3.31984	6.73562	Adipose_R2_peak_2	Distal Interger	1	1.51E+08	1.51E+08	6062	1	402734	uc029qtz.1	57158	NA	C730036E19Ri	RIKEN cDNA C730036E19 gene
chr1	151199655	151199833	179	180	151199750	35	9.95877	3.31984	6.73562	Adipose_R2_peak_2	Distal Interger	1	1.51E+08	1.51E+08	6062	1	402734	uc029qtz.1	61621	NA	C730036E19Ri	RIKEN cDNA C730036E19 gene
chr1	151201541	151201856	316	317	151201738	48	17.1112	4.23943	13.5566	Adipose_R2_peak_2	Distal Interger	1	1.51E+08	1.51E+08	6062	1	402734	uc029qtz.1	63507	NA	C730036E19Ri	RIKEN cDNA C730036E19 gene
chr1	151201599	151201752	154	155	151201637	24	5.31854	2.58072	2.5036	Adipose_R1_peak_1	Distal Interger	1	1.51E+08	1.51E+08	6062	1	402734	uc029qtz.1	63565	NA	C730036E19Ri	RIKEN cDNA C730036E19 gene
chr1	151202183	151202291	109	110	151202232	30	7.24477	2.85875	4.21917	Adipose_R2_peak_2	Distal Interger	1	1.51E+08	1.51E+08	6062	1	402734	uc029qtz.1	64149	NA	C730036E19Ri	RIKEN cDNA C730036E19 gene
chr1	151202828	151202930	103	104	151202878	27	5.77405	2.5821	2.88921	Adipose_R2_peak_2	Distal Interger	1	1.51E+08	1.51E+08	6062	1	402734	uc029qtz.1	64794	NA	C730036E19Ri	RIKEN cDNA C730036E19 gene
chr1	151203404	151203815	412	413	151203441	30	7.24477	2.85875	4.21917	Adipose_R2_peak_2	Distal Interger	1	1.51E+08	1.51E+08	6062	1	402734	uc029qtz.1	65370	NA	C730036E19Ri	RIKEN cDNA C730036E19 gene
chr1	152022025	152022517	493	494	152022134	26	12.08537	4.8305	8.84945	Adipose_R1_peak_1	Intron (uc007c	1	1.52E+08	1.52E+08	237918	2	69399	uc007czd.2	67803	ENSMUSG000	1700025G04R	RIKEN cDNA 1700025G04 gene
chr1	152146209	152146350	142	143	152146318	17	5.80341	3.23528	2.92378	Adipose_R1_peak_1	Distal Interger	1	1.52E+08	1.52E+08	237918	2	69399	uc007czd.2	-55889	ENSMUSG000	1700025G04R	RIKEN cDNA 1700025G04 gene
chr1	152179542	152179672	131	132	152179594	21	8.43286	3.95423	5.34896	Adipose_R1_peak_1	Distal Interger	1	1.52E+08	1.52E+08	237918	2	69399	uc007czd.2	-89222	ENSMUSG000	1700025G04R	RIKEN cDNA 1700025G04 gene
chr1	152180356	152180472	117	118	152180362	25	4.86612	2.39766	2.09018	Adipose_R2_peak_2	Distal Interger	1	1.52E+08	1.52E+08	237918	2	69399	uc007czd.2	-90036	ENSMUSG000	1700025G04R	RIKEN cDNA 1700025G04 gene
chr1	152181115	152181265	151	152	152181187	37	11.12817	3.50427	7.83613	Adipose_R2_peak_2	Distal Interger	1	1.52E+08	1.52E+08	237918	2	69399	uc007czd.2	-90795	ENSMUSG000	1700025G04R	RIKEN cDNA 1700025G04 gene
chr1	152559770	152559925	156	157	152559847	17	5.80341	3.23528	2.92378	Adipose_R1_peak_1	Intron (uc007c	1	1.53E+08	1.53E+08	107582	2	19731	uc007czh.1	65186	ENSMUSG000	Rgl1	ral guanine nucleotide dissociation stimulator,-like 1
chr1	152666860	152667014	155	156	152666921	41	13.59774	3.87315	10.1803	Adipose_R2_peak_2	Distal Interger	1	1.53E+08	1.53E+08	107582	2	19731	uc007czh.1	-41749	ENSMUSG000	Rgl1	ral guanine nucleotide dissociation stimulator,-like 1
chr1	154372452	154372626	175	176	154372526	25	11.36566	4.67319	8.13482	Adipose_R1_peak_1	Distal Interger	1	1.54E+08	1.54E+08	15386	2	78112	uc007dat.1	-115546	ENSMUSG000	4930452N14R	RIKEN cDNA 4930452N14 gene
chr1	154454798	154455008	211	212	154454886	29	14.55748	5.39214	11.20652	Adipose_R1_peak_1	Intron (uc007c	1	1.54E+08	1.54E+08	15386	2	78112	uc007dat.1	-197892	ENSMUSG000	4930452N14R	RIKEN cDNA 4930452N14 gene
chr1	154454824	154454938	115	116	154454879	31	7.7623	2.95097	4.69388	Adipose_R2_peak_2	Intron (uc007c	1	1.54E+08	1.54E+08	15386	2	78112	uc007dat.1	-197918	ENSMUSG000	4930452N14R	RIKEN cDNA 4930452N14 gene
chr1	154455193	154455330	138	139	154455256	19	7.07687	3.59476	4.08458	Adipose_R1_peak_1	Intron (uc007c	1	1.54E+08	1.54E+08	15386	2	78112	uc007dat.1	-198287	ENSMUSG000	4930452N14R	RIKEN cDNA 4930452N14 gene
chr1	154456097	154456319	223	224	154456212	33	17.97508	6.11109	14.52164	Adipose_R1_peak_1	Intron (uc007c	1	1.54E+08	1.54E+08	15386	2	78112	uc007dat.1	-199191	ENSMUSG000	4930452N14R	RIKEN cDNA 4930452N14 gene
chr1	154456110	154456273	164	165	154456203	36	10.5378	3.41206	7.27949	Adipose_R2_peak_2	Intron (uc007c	1	1.54E+08	1.54E+08	15386	2	78112	uc007dat.1	-199204	ENSMUSG000	4930452N14R	RIKEN cDNA 4930452N14 gene
chr1	155070608	155070744	137	138	155070681	22	9.13959	4.13397	6.01526	Adipose_R1_peak_1	Intron (uc007c	1	1.55E+08	1.55E+08	3270	2	15939	uc007dax.2	28892	ENSMUSG000	Ier5	immediate early response 5
chr1	155082427	155082600	174	175	155082448	16	5.20017	3.05554	2.38734	Adipose_R1_peak_1	Exon (uc007da	1	1.55E+08	1.55E+08	3270	2	15939	uc007dax.2	17036	ENSMUSG000	Ier5	immediate early response 5
chr1	155084715	155085191	477	478	155084818	32	8.29282	3.04319	5.18352	Adipose_R2_peak_2	Distal Interger	1	1.55E+08	1.55E+08	3270	2	15939	uc007dax.2	14445	ENSMUSG000	Ier5	immediate early response 5
chr1	155087460	155087674	215	216	155087501	26	5.31252	2.48988	2.47999	Adipose_R2_peak_2	Distal Interger	1	1.55E+08	1.55E+08	3270	2	15939	uc007dax.2	11962	ENSMUSG000	Ier5	immediate early response 5
chr1	155089489	155089794	306	307	155089738	28	6.25023	2.67431	3.31626	Adipose_R2_peak_2	Distal Interger	1	1.55E+08	1.55E+08	3270	2	15939	uc007dax.2	9842	ENSMUSG000	Ier5	immediate early response 5
chr1	155090392	155090616	225	226	155090549	21	8.43286	3.95423	5.34896	Adipose_R1_peak_1	Distal Interger	1	1.55E+08	1.55E+08	3270	2	15939	uc007dax.2	9020	ENSMUSG000	Ier5	immediate early response 5
chr1	155091723	155091860	138	139	155091859	27	5.77405	2.5821	2.88921	Adipose_R2_peak_2	Distal Interger	1	1.55E+08	1.55E+08	3270	2	15939	uc007dax.2	7776	ENSMUSG000	Ier5	immediate early response 5
chr1	155099341	155099579	239	240	155099422	26	5.31252	2.48988	2.47999	Adipose_R2_peak_2	Promoter (<=	1	1.55E+08	1.55E+08	3270	2	15939	uc007dax.2	57	ENSMUSG000	Ier5	immediate early response 5
chr1	155109224	155109361	138	139	155109331	18	6.42934	3.41502	3.48999	Adipose_R1_peak_1	Distal Interger	1	1.55E+08	1.55E+08	3270	2	15939	uc007dax.2	-9588	ENSMUSG000	Ier5	immediate early response 5
chr1	156725522	156725798	277	278	156725648	82	46.38432	7.65407	42.27038	Adipose_R2_peak_2	Distal Interger	1	1.57E+08	1.57E+08	40340	2	215015	uc007dcp.1	-6612	ENSMUSG000	Fam20b	family with sequence similarity 20, member B
chr1	156725524	156725823	300	301	156725653	75	63.12309	13.66008	59.01505	Adipose_R1_peak_1	Distal Interger	1	1.57E+08	1.57E+08	40340	2	215015	uc007dcp.1	-6614	ENSMUSG000	Fam20b	family with sequence similarity 20, member B
chr1	156786918	156787057	140	141	156786961	16	5.20017	3.05554	2.38734	Adipose_R1_peak_1	Distal Interger	1	1.57E+08	1.57E+08	9403	2	78255	uc007dcs.2	26511	ENSMUSG000	Ralgps2	Ral GEF with PH domain and SH3 binding motif 2
chr1	156849833	156850114	282	283	156849975	54	38.78658	9.88558	34.93639	Adipose_R1_peak_1	Intron (uc007c	1	1.57E+08	1.57E+08	6593	1	72713	uc007ddc.1	10810	ENSMUSG000	Angptl1	angiopoietin-like 1
chr1	156912012	156912137	126	127	156912066	31	7.7623	2.95097	4.69388	Adipose_R2_peak_2	Intron (uc011v	1	1.57E+08	1.57E+08	119942	2	78255	uc007dda.1	26681	ENSMUSG000	Ralgps2	Ral GEF with PH domain and SH3 binding motif 2
chr1	156912029	156912213	185	186	156912115	25	11.36566	4.67319	8.13482	Adipose_R1_peak_1	Intron (uc011v	1	1.57E+08	1.57E+08	119942	2	78255	uc007dda.1	26605	ENSMUSG000	Ralgps2	Ral GEF with PH domain and SH3 binding motif 2
chr1	157203689	157203844	156	157	157203764	24	10.60668	4.49345	7.40961	Adipose_R1_peak_1	Intron (uc007c	1	1.57E+08	1.57E+08	9534	2	73435	uc056yek.1	-95009	ENSMUSG000	Tex35	testis expressed 35
chr1	157203721	157203842	122	123	157203788	32	8.29282	3.04319	5.18352	Adipose_R2_peak_2	Intron (uc007c	1	1.57E+08	1.57E+08	9534	2	73435	uc056yek.1	-95041	ENSMUSG000	Tex35	testis expressed 35
chr1	157599249	157599585	337	338	157599488	939	970.4103	27.31566	965.0153	Adipose_R2_peak_2	Distal Interger	1	1.58E+08	1.58E+08	26338	1	89867	uc007ddo.1	63397	ENSMUSG000	Sec16b	SEC16 homolog B (S. cerevisiae)
chr1	157599269	157599586	318	319	157599476	815	681.4011	17.10177	676.3491	Adipose_R1_peak_1	Distal Interger	1	1.58E+08	1.58E+08	26338	1	89867	uc007ddo.1	63417	ENSMUSG000	Sec16b	SEC16 homolog B (S. cerevisiae)
chr1	157690654	157690858	205	206	157690738	21	8.43286	3.95423	5.34896	Adipose_R1_peak_1	Distal Interger	1	1.58E+08	1.58E+08	26338	1	89867	uc007ddo.1	154802	ENSMUSG000	Sec16b	SEC16 homolog B (S. cerevisiae)
chr1	157846999	157847145	147	148	157847088	24	10.60668	4.49345	7.40961	Adipose_R1_peak_1	Distal Interger	1	1.58E+08	1.58E+08	26338	1	89867	uc007ddo.1	311147	ENSMUSG000	Sec16b	SEC16 homolog B (S. cerevisiae)
chr1	157877239	157877547	309	310	157877349	456	442.5454	22.83452	437.6724	Adipose_R1_peak_1	Distal Interger	1	1.58E+08	1.58E+08	26338	1	89867	uc007ddo.1	341387	ENSMUSG000	Sec16b	SEC16 homolog B (S. cerevisiae)
chr1	157877242	157877473	232	233	157877346	438	425.452	22.8208	420.4668	Adipose_R2_peak_2	Distal Interger	1	1.58E+08	1.58E+08	26338	1	89867	uc007ddo.1	341390	ENSMUSG000	Sec16b	SEC16 homolog B (S. cerevisiae)
chr1	157977400	157977932	533	534	157977768	74	38.91365	6.8703	35.0626	Adipose_R1_peak_1	Distal Interger	1	1.58E+08	1.58E+08	110961	2	240843	uc007ddp.2	378297	ENSMUSG000	Brinp2	bone morphogenic protein/retinoic acid inducible neural-specific 2
chr1	157977573	157977883	311	312	157977752	56	18.01537	3.94801	14.43328	Adipose_R2_peak_2	Distal Interger	1	1.58E+08	1.58E+08	110961	2	240843	uc007ddp.2	378346	ENSMUSG000	Brinp2	bone morphogenic protein/retinoic acid inducible neural-specific 2
chr1	158587623	158587813	191	192	158587707	30	15.39182	5.57188	12.01394	Adipose_R1_peak_1	Intron (uc007c	1	1.59E+08	1.59E+08	109	1	735253	uc011wuk.1	81998	ENSMUSG000	Mir488	microRNA 488
chr1	158587655	158587774	120	121	158587708	32	8.29282	3.04319	5.18352	Adipose_R2_peak_2	Intron (uc007c	1	1.59E+08	1.59E+08	109	1	735253	uc011wuk.1	82030	ENSMUSG000	Mir488	microRNA 488
chr1	159025989	159026290	302	303	159026123	94	57.89143	8.76068	53.65463	Adipose_R2_peak_2	Distal Interger	1	1.59E+08	1.59E+08	245708	2	23850	uc007ddv.1	-68551	ENSMUSG000	Pappa2	pappalysin 2
chr1	159025990	159026327	338	339	159026126	103	99.40691	18.69274	95.07599	Adipose_R1_peak_1	Distal Interger	1	1.59E+08	1.59E+08	245708	2	23850	uc007ddv.1	-68552	ENSMUSG000	Pappa2	pappalysin 2
chr1	159026638	159027018	381	382	159026793	153	145.4302	19.53269	140.93	Adipose_R1_peak_1	Distal Interger	1	1.59E+08	1.59E+08	245708	2	23850	uc007ddv.1	-69200	ENSMUSG000	Pappa2	pappalysin 2
chr1	159026649	159026991	343	344	159026799	131	97.19221	12.17274	92.69543	Adipose_R2_peak_2	Distal Interger	1	1.59E+08	1.59E+08	245708	2	23850	uc007ddv.1	-69211	ENSMUSG000	Pappa2	pappalysin 2
chr1	159625999	159626209	211	212	159626114	42	26.36523	7.72873	22.71906	Adipose_R1_peak_1	Intron (uc007c	1	1.60E+08	1.60E+08	401154	1	21960	uc007dea.2	102230	ENSMUSG000	Tnr	tenascin R
chr1	159626001	159626204	204	205	159626118	44	15.55653	4.1498	12.05625	Adipose_R2_peak_2	Intron (uc007c	1	1.60E+08	1.60E+08	401154	1	21960	uc007dea.2	102232	ENSMUSG000	Tnr	tenascin R
chr1	160351771	160351924	154	155	160351829	21	8.43286	3.95423	5.34896	Adipose_R1_peak_1	Promoter (<=	1	1.60E+08	1.60E+08	132398	2	29809	uc007deh.1	-200	ENSMUSG000	Rabgap1l	RAB GTPase activating protein 1-like
chr1	160423794	160424113	320	321	160423974	131	108.6284	14.74921	104.2584	Adipose_R1_peak_1	Intron (uc007c	1	1.60E+08	1.60E+08	132398	2	29809	uc007deh.1	-72223	ENSMUSG000	Rabgap1l	RAB GTPase activating protein 1-like
chr1	160423832	160424098	267	268	160423963	97	52.32477	7.34593	48.1459	Adipose_R2_peak_2	Intron (uc007c	1	1.60E+08	1.60E+08	132398	2	29809	uc007deh.1	-72261	ENSMUSG000	Rabgap1l	RAB GTPase activating protein 1-like
chr1	160427543	160427700	158	159	160427624	22	9.13959	4.13397	6.01526	Adipose_R1_peak_1	Intron (uc007c	1	1.60E+08	1.60E+08	132398	2	29809	uc007deh.1	-75972	ENSMUSG000	Rabgap1l	RAB GTPase activating protein 1-like
chr1	160427578	160427673	96	97	160427631	30	7.24477	2.85875	4.21917	Adipose_R2_peak_2	Intron (uc007c	1	1.60E+08	1.60E+08	132398	2	29809	uc007deh.1	-76007	ENSMUSG000	Rabgap1l	RAB GTPase activating protein 1-like
chr1	160638614	160638871	258	259	160638744	39	23.47001	7.18952	19.88325	Adipose_R1_peak_1	Intron (uc007c	1	1.61E+08	1.61E+08	1086	2	620246	uc011wum.1	-60861	NA	Gpr52	G protein-coupled receptor 52
chr1	160638634	160638864	231	232	160638742	51	20.44827	4.79532	16.78434	Adipose_R2_peak_2	Intron (uc007c	1	1.61E+08	1.61E+08	1086	2	620246	uc011wum.1	-60881	NA	Gpr52	G protein-coupled receptor 52
chr1	161679491	161679870	380	381	161679651	126	123.5099	20.07247	119.082	Adipose_R1_peak_1	Distal Interger	1	1.62E+08	1.62E+08	7804	2	14103	uc007dfr.2	108625	ENSMUSG000	Fasl	Fas ligand (TNF superfamily, member 6)
chr1	161679511	161679870	360	361	161679675	106	70.05344	9.8673	65.71089	Adipose_R2_peak_2	Distal Interger	1	1.62E+08	1.62E+08	7804	2	14103	uc007dfr.2	108625	ENSMUSG000	Fasl	Fas ligand (TNF superfamily, member 6)
chr1	161726009	161726155	147	148	161726116	26	5.31252	2.48988	2.47999	Adipose_R2_peak_2	Exon (uc007df	1	1.62E+08	1.62E+08	7804	2	14103	uc007dfr.2	62340	ENSMUSG000	Fasl	Fas ligand (TNF superfamily, member 6)
chr1	161933524	161933754	231	232	161933649	33	17.97508	6.11109	14.52164	Adipose_R1_peak_1	Distal Interger	1	1.62E+08	1.62E+08	27060	2	75368	uc007dfw.2	35394	ENSMUSG000	4930558K02Ri	RIKEN cDNA 4930558K02 gene
chr1	161986860	161986998	139	140	161986891	25	4.86612	2.39766	2.09018	Adipose_R2_peak_2	Intron (uc007c	1	1.62E+08	1.62E+08	37548	2	75368	uc029qum.1	-7224	ENSMUSG000	4930558K02Ri	RIKEN cDNA 4930558K02 gene
chr1	162117306	162117605	300	301	162117451	92	84.7075	16.71563	80.45023	Adipose_R1_peak_1	Intron (uc007c	1	1.62E+08	1.62E+08	118300	2	103967	uc007dge.1	17147	ENSMUSG000	Dnm3	dynamin 3
chr1	162117312	162117568	257	258	162117440	61	28.12311	5.7175	24.28203	Adipose_R2_peak_2	Intron (uc007c	1	1.62E+08	1.62E+08	118300	2	103967	uc007dge.1	17184	ENSMUSG000	Dnm3	dynamin 3
chr1	162265839	162266121	283	284	162266007	54	38.78658	9.88558	34.93639	Adipose_R1_peak_1	Intron (uc007c	1	1.62E+08	1.62E+08	313183	2	103967	uc007dfz.1	29517	ENSMUSG000	Dnm3	dynamin 3

chr1	162265925	162266109	185	186	162266004	39	12.34187	3.68871	8.98501	Adipose_R2_peak_2	Intron (uc007c	1	1.62E+08	1.62E+08	313183	2	103967	uc007dfz.1	29529	ENSMUSG000	Dnm3	dynamin 3
chr1	162490092	162490348	257	258	162490216	89	62.05884	10.43864	57.96054	Adipose_R1_peak_1	Distal Interger	1	1.62E+08	1.62E+08	490733	2	103967	uc007dga.2	-12058	ENSMUSG000	Dnm3	dynamin 3
chr1	162491887	162492108	222	223	162492008	33	17.97508	6.11109	14.52164	Adipose_R1_peak_1	Distal Interger	1	1.62E+08	1.62E+08	490733	2	103967	uc007dga.2	-13853	ENSMUSG000	Dnm3	dynamin 3
chr1	162491910	162492101	192	193	162492022	35	9.95877	3.31984	6.73562	Adipose_R2_peak_2	Distal Interger	1	1.62E+08	1.62E+08	490733	2	103967	uc007dga.2	-13876	ENSMUSG000	Dnm3	dynamin 3
chr1	162493086	162493315	230	231	162493187	41	25.38991	7.54899	21.76305	Adipose_R1_peak_1	Distal Interger	1	1.62E+08	1.62E+08	490733	2	103967	uc007dga.2	-15052	ENSMUSG000	Dnm3	dynamin 3
chr1	162493088	162493317	230	231	162493211	48	18.29934	4.51867	14.70076	Adipose_R2_peak_2	Distal Interger	1	1.62E+08	1.62E+08	490733	2	103967	uc007dga.2	-15054	ENSMUSG000	Dnm3	dynamin 3
chr1	162811734	162811999	266	267	162811911	66	52.33981	12.04244	48.33179	Adipose_R1_peak_1	Promoter (<=	1	1.63E+08	1.63E+08	18771	2	226564	uc007dgv.1	654	ENSMUSG000	Fmo4	flavin containing monooxygenase 4
chr1	162811741	162811997	257	258	162811920	63	29.74474	5.90194	25.87429	Adipose_R2_peak_2	Promoter (<=	1	1.63E+08	1.63E+08	18771	2	226564	uc007dgv.1	656	ENSMUSG000	Fmo4	flavin containing monooxygenase 4
chr1	162824922	162825066	145	146	162824990	21	8.43286	3.95423	5.34896	Adipose_R1_peak_1	Distal Interger	1	1.63E+08	1.63E+08	10820	2	226564	uc007dgw.1	-11250	ENSMUSG000	Fmo4	flavin containing monooxygenase 4
chr1	163013588	163013752	165	166	163013673	21	8.43286	3.95423	5.34896	Adipose_R1_peak_1	Distal Interger	1	1.63E+08	1.63E+08	30730	2	14262	uc007dhe.2	-29060	ENSMUSG000	Fmo3	flavin containing monooxygenase 3
chr1	163017239	163017534	296	297	163017395	66	52.33981	12.04244	48.33179	Adipose_R1_peak_1	Distal Interger	1	1.63E+08	1.63E+08	30730	2	14262	uc007dhe.2	-32711	ENSMUSG000	Fmo3	flavin containing monooxygenase 3
chr1	163017329	163017490	162	163	163017412	36	10.5378	3.41206	7.27949	Adipose_R2_peak_2	Distal Interger	1	1.63E+08	1.63E+08	30730	2	14262	uc007dhe.2	-32801	ENSMUSG000	Fmo3	flavin containing monooxygenase 3
chr1	163774668	163774896	229	230	163774796	28	13.73721	5.2124	10.41463	Adipose_R1_peak_1	Distal Interger	1	1.64E+08	1.64E+08	137525	1	16579	uc007dho.2	-4687	ENSMUSG000	Kifap3	kinesin-associated protein 3
chr1	163775905	163776077	173	174	163775997	39	12.34187	3.68871	8.98501	Adipose_R2_peak_2	Distal Interger	1	1.64E+08	1.64E+08	137525	1	16579	uc007dho.2	-3506	ENSMUSG000	Kifap3	kinesin-associated protein 3
chr1	163775940	163776128	189	190	163776020	31	16.23981	5.75161	12.83549	Adipose_R1_peak_1	Distal Interger	1	1.64E+08	1.64E+08	137525	1	16579	uc007dho.2	-3455	ENSMUSG000	Kifap3	kinesin-associated protein 3
chr1	163812270	163812521	252	253	163812467	30	7.24477	2.85875	4.21917	Adipose_R2_peak_2	Intron (uc007c	1	1.64E+08	1.64E+08	137525	1	16579	uc007dho.2	32687	ENSMUSG000	Kifap3	kinesin-associated protein 3
chr1	164351913	164352077	165	166	164352023	18	6.42934	3.41502	3.48999	Adipose_R1_peak_1	Intron (uc007c	1	1.64E+08	1.64E+08	17685	2	66352	uc007dig.1	-44429	ENSMUSG000	Blzf1	basic leucine zipper nuclear factor 1
chr1	164351928	164352077	150	151	164352070	30	7.24477	2.85875	4.21917	Adipose_R2_peak_2	Intron (uc007c	1	1.64E+08	1.64E+08	17685	2	66352	uc007dig.1	-44444	ENSMUSG000	Blzf1	basic leucine zipper nuclear factor 1
chr1	164535491	164535872	382	383	164535609	19	7.07687	3.59476	4.08458	Adipose_R1_peak_1	Distal Interger	1	1.64E+08	1.64E+08	21257	2	11931	uc007dip.2	-77136	ENSMUSG000	Atp1b1	ATPase, Na+/K+ transporting, beta 1 polypeptide
chr1	164539673	164540065	393	394	164539830	24	10.60668	4.49345	7.40961	Adipose_R1_peak_1	Distal Interger	1	1.64E+08	1.64E+08	21257	2	11931	uc007dip.2	-81318	ENSMUSG000	Atp1b1	ATPase, Na+/K+ transporting, beta 1 polypeptide
chr1	164539754	164540155	402	403	164539845	37	11.12817	3.50427	7.83613	Adipose_R2_peak_2	Distal Interger	1	1.64E+08	1.64E+08	21257	2	11931	uc007dip.2	-81399	ENSMUSG000	Atp1b1	ATPase, Na+/K+ transporting, beta 1 polypeptide
chr1	164577765	164577919	155	156	164577852	18	6.42934	3.41502	3.48999	Adipose_R1_peak_1	Distal Interger	1	1.64E+08	1.64E+08	21257	2	11931	uc007dip.2	-119410	ENSMUSG000	Atp1b1	ATPase, Na+/K+ transporting, beta 1 polypeptide
chr1	164727144	164727513	370	371	164727322	138	147.9434	24.50879	143.4355	Adipose_R1_peak_1	Distal Interger	1	1.65E+08	1.65E+08	27535	1	56429	uc007diq.1	-69219	ENSMUSG000	Dpt	dermatopontin
chr1	164727170	164727465	296	297	164727318	106	70.05344	9.8673	65.71089	Adipose_R2_peak_2	Distal Interger	1	1.65E+08	1.65E+08	27535	1	56429	uc007diq.1	-69267	ENSMUSG000	Dpt	dermatopontin
chr1	165027628	165027975	348	349	165027767	93	86.02155	16.89536	81.75695	Adipose_R1_peak_1	Distal Interger	1	1.65E+08	1.65E+08	25314	2	75857	uc007div.2	63955	ENSMUSG000	4930568G15R	RIKEN cDNA 4930568G15 gene
chr1	165027640	165027920	281	282	165027770	80	44.53636	7.46964	40.44495	Adipose_R2_peak_2	Distal Interger	1	1.65E+08	1.65E+08	25314	2	75857	uc007div.2	64010	ENSMUSG000	4930568G15R	RIKEN cDNA 4930568G15 gene
chr1	165408732	165408907	176	177	165408819	26	12.14079	4.85292	8.87838	Adipose_R1_peak_1	Intron (uc007c	1	1.65E+08	1.65E+08	130963	2	74106	uc007djb.2	51556	ENSMUSG000	Dcaf6	DDB1 and CUL4 associated factor 6
chr1	165448432	165448668	237	238	165448569	35	19.76029	6.47056	16.26028	Adipose_R1_peak_1	Intron (uc007c	1	1.65E+08	1.65E+08	130963	2	74106	uc007djb.2	11795	ENSMUSG000	Dcaf6	DDB1 and CUL4 associated factor 6
chr1	165448492	165448639	148	149	165448564	29	6.74062	2.76653	3.75989	Adipose_R2_peak_2	Intron (uc007c	1	1.65E+08	1.65E+08	130963	2	74106	uc007djb.2	11824	ENSMUSG000	Dcaf6	DDB1 and CUL4 associated factor 6
chr1	165533474	165533784	311	312	165533640	90	53.97882	8.39181	49.78112	Adipose_R2_peak_2	Intron (uc007c	1	1.66E+08	1.66E+08	33417	1	271639	uc011wva.1	23563	ENSMUSG000	Adcy10	adenylate cyclase 10
chr1	165533478	165533790	313	314	165533633	72	49.20463	9.76661	45.23124	Adipose_R1_peak_1	Intron (uc007c	1	1.66E+08	1.66E+08	33417	1	271639	uc011wva.1	23567	ENSMUSG000	Adcy10	adenylate cyclase 10
chr1	165533945	165534123	179	180	165533966	16	5.20017	3.05554	2.38734	Adipose_R1_peak_1	Intron (uc007c	1	1.66E+08	1.66E+08	33417	1	271639	uc011wva.1	24034	ENSMUSG000	Adcy10	adenylate cyclase 10
chr1	165635431	165635626	196	197	165635513	32	8.29282	3.04319	5.18352	Adipose_R2_peak_2	Promoter (<=	1	1.66E+08	1.66E+08	42361	2	68481	uc007djh.1	-890	ENSMUSG000	Mpzl1	myelin protein zero-like 1
chr1	165638173	165638466	294	295	165638354	21	8.43286	3.95423	5.34896	Adipose_R1_peak_1	Distal Interger	1	1.66E+08	1.66E+08	42361	2	68481	uc007djh.1	-3632	ENSMUSG000	Mpzl1	myelin protein zero-like 1
chr1	165638561	165638672	112	113	165638570	26	5.31252	2.48988	2.47999	Adipose_R2_peak_2	Distal Interger	1	1.66E+08	1.66E+08	42361	2	68481	uc007djh.1	-4020	ENSMUSG000	Mpzl1	myelin protein zero-like 1
chr1	165759599	165759772	174	175	165759697	22	9.13959	4.13397	6.01526	Adipose_R1_peak_1	Distal Interger	1	1.66E+08	1.66E+08	11525	1	433375	uc007djm.1	-4008	ENSMUSG000	Creg1	cellular repressor of E1A-stimulated genes 1
chr1	165767474	165768223	750	751	165767675	19	7.07687	3.59476	4.08458	Adipose_R1_peak_1	Intron (uc007c	1	1.66E+08	1.66E+08	11525	1	433375	uc007djm.1	3694	ENSMUSG000	Creg1	cellular repressor of E1A-stimulated genes 1
chr1	165767782	165767981	200	201	165767842	29	6.74062	2.76653	3.75989	Adipose_R2_peak_2	Intron (uc007c	1	1.66E+08	1.66E+08	11525	1	433375	uc007djm.1	4002	ENSMUSG000	Creg1	cellular repressor of E1A-stimulated genes 1
chr1	165768165	165768264	100	101	165768214	27	5.77405	2.5821	2.88921	Adipose_R2_peak_2	Intron (uc007c	1	1.66E+08	1.66E+08	11525	1	433375	uc007djm.1	4385	ENSMUSG000	Creg1	cellular repressor of E1A-stimulated genes 1
chr1	166541275	166541701	427	428	166541427	51	35.56531	9.34637	31.76235	Adipose_R1_peak_1	Distal Interger	1	1.66E+08	1.66E+08	13976	2	226601	uc011wvl.1	-43687	ENSMUSG000	Gm4846	predicted gene 4846
chr1	166541288	166541538	251	252	166541408	49	19.00719	4.61089	15.38598	Adipose_R2_peak_2	Distal Interger	1	1.66E+08	1.66E+08	13976	2	226601	uc011wvl.1	-43700	ENSMUSG000	Gm4846	predicted gene 4846
chr1	166551762	166551996	235	236	166551874	33	17.97508	6.11109	14.52164	Adipose_R1_peak_1	Distal Interger	1	1.66E+08	1.66E+08	13976	2	226601	uc011wvl.1	-54174	ENSMUSG000	Gm4846	predicted gene 4846
chr1	166583652	166583785	134	135	166583716	21	8.43286	3.95423	5.34896	Adipose_R1_peak_1	Distal Interger	1	1.67E+08	1.67E+08	18723	2	226604	uc011wvm.1	63908	ENSMUSG000	Gm4847	predicted gene 4847
chr1	166622104	166622270	167	168	166622198	21	8.43286	3.95423	5.34896	Adipose_R1_peak_1	Distal Interger	1	1.67E+08	1.67E+08	18723	2	226604	uc011wvm.1	25423	ENSMUSG000	Gm4847	predicted gene 4847
chr1	166622136	166622268	133	134	166622189	34	9.39139	3.22762	6.20474	Adipose_R2_peak_2	Distal Interger	1	1.67E+08	1.67E+08	18723	2	226604	uc011wvm.1	25425	ENSMUSG000	Gm4847	predicted gene 4847
chr1	166622679	166622904	226	227	166622849	31	7.7623	2.95097	4.69388	Adipose_R2_peak_2	Distal Interger	1	1.67E+08	1.67E+08	18723	2	226604	uc011wvm.1	24789	ENSMUSG000	Gm4847	predicted gene 4847
chr1	166622688	166622973	286	287	166622827	24	10.60668	4.49345	7.40961	Adipose_R1_peak_1	Distal Interger	1	1.67E+08	1.67E+08	18723	2	226604	uc011wvm.1	24720	ENSMUSG000	Gm4847	predicted gene 4847
chr1	166872722	166872911	190	191	166872830	29	14.55748	5.39214	11.20652	Adipose_R1_peak_1	Distal Interger	1	1.67E+08	1.67E+08	25708	2	1.01E+08	uc007dkr.2	126819	ENSMUSG000	Gm16701	predicted gene, 16701
chr1	166872787	166872897	111	112	166872839	28	6.25023	2.67431	3.31626	Adipose_R2_peak_2	Distal Interger	1	1.67E+08	1.67E+08	25708	2	1.01E+08	uc007dkr.2	126833	ENSMUSG000	Gm16701	predicted gene, 16701
chr1	166964277	166964466	190	191	166964449	30	15.39182	5.57188	12.01394	Adipose_R1_peak_1	Distal Interger	1	1.67E+08	1.67E+08	25708	2	1.01E+08	uc007dkr.2	35264	ENSMUSG000	Gm16701	predicted gene, 16701
chr1	167199438	167199614	177	178	167199521	28	13.73721	5.2124	10.41463	Adipose_R1_peak_1	Distal Interger	1	1.67E+08	1.67E+08	59044	2	80914	uc007dku.2	85513	ENSMUSG000	Uck2	uridine-cytidine kinase 2
chr1	167199458	167199588	131	132	167199511	31	7.7623	2.95097	4.69388	Adipose_R2_peak_2	Distal Interger	1	1.67E+08	1.67E+08	59044	2	80914	uc007dku.2	85539	ENSMUSG000	Uck2	uridine-cytidine kinase 2
chr1	167340222	167340375	154	155	167340290	22	8.53987	3.88691	5.45492	Adipose_R1_peak_1	Distal Interger	1	1.67E+08	1.67E+08	18540	1	56752	uc007dkx.1	-9616	ENSMUSG000	Aldh9a1	aldehyde dehydrogenase 9, subfamily A1
chr1	167824114	167824499	386	387	167824244	23	9.86443	4.31371	6.70247	Adipose_R1_peak_1	Intron (uc007c	1	1.68E+08	1.68E+08	159176	1	110648	uc007dlc.1	134556	ENSMUSG000	Lmx1a	LIM homeobox transcription factor 1 alpha
chr1	167824309	167824444	136	137	167824409	28	6.25023	2.67431	3.31626	Adipose_R2_peak_2	Intron (uc007c	1	1.68E+08	1.68E+08	159176	1	110648	uc007dlc.1	134751	ENSMUSG000	Lmx1a	LIM homeobox transcription factor 1 alpha
chr1	167932948	167933168	221	222	167933068	39	12.34187	3.68871	8.98501	Adipose_R2_peak_2	Distal Interger	1	1.68E+08	1.68E+08	159176	1	110648	uc007dlc.1	243390	ENSMUSG000	Lmx1a	LIM homeobox transcription factor 1 alpha
chr1	167932949	167933182	234	235	167933070	49	32.31849	8.56847	28.57812	Adipose_R1_peak_1	Distal Interger	1	1.68E+08	1.68E+08	159176	1	110648	uc007dlc.1	243391	ENSMUSG000	Lmx1a	LIM homeobox transcription factor 1 alpha
chr1	168502075	168502249	175	176	168502236	18	6.42934	3.41502	3.48999	Adipose_R1_peak_1	Distal Interger	1	1.68E+08	1.68E+08	120	1	1.02E+08	uc033fmv.1	11432	ENSMUSG000	Mir6348	microRNA 6348
chr1	168853713	168853904	192	193	168853797	24	10.60668	4.49345	7.40961	Adipose_R1_peak_1	Distal Interger	1	1.68E+08	1.68E+08	120	1	1.02E+08	uc033fmv.1	363070	ENSMUSG000	Mir6348	microRNA 6348
chr1	168854636	168855045	410	411	168854830	119	98.41029	14.4683	94.08471	Adipose_R1_peak_1	Distal Interger	1	1.68E+08	1.68E+08	120	1	1.02E+08	uc033fmv.1	363993	ENSMUSG000	Mir6348	microRNA 6348
chr1	168854679	168855024	346	347	168854846	95	57.7475	8.61233	53.51472	Adipose_R2_peak_2	Distal Interger	1	1.68E+08	1.68E+08	120	1	1.02E+08	uc033fmv.1	364036	ENSMUSG000	Mir6348	microRNA 6348
chr1	168988755	168988950	196	197	168988879	38	11.72962	3.59649	8.4047	Adipose_R2_peak_2	Distal Interger	1	1.68E+08	1.68E+08	120	1	1.02E+08	uc033fmv.1	498112	ENSMUSG000	Mir6348	microRNA 6348
chr1	168988770	168988974	205	206	168988879	26	12.14079	4.85292	8.87838	Adipose_R1_peak_1	Distal Interger	1	1.68E+08	1.68E+08	120	1	1.02E+08	uc033fmv.1	498127	ENSMUSG000	Mir6348	microRNA 6348
chr1	169059270	169059405	136	137	169059351	26	5.31252	2.48988	2.47999	Adipose_R2_peak_2	Distal Interger	1	1.69E+08	1.70E+08	33531	2	66977	uc007dlj.1	472059	ENSMUSG000	Nuf2	NUF2, NDC80 kinetochore complex component
chr1	169102569	169102734	166	167	169102703	19	7.07687	3.59476	4.08458	Adipose_R1_peak_1	Distal Interger	1	1.69E+08	1.70E+08	33531	2	66977	uc007dlj.1	428730	ENSMUSG000	Nuf2	NUF2, NDC80 kinetochore complex component
chr1	169170121	169170255	135	136	169170232	28	6.25023	2.67431	3.31626	Adipose_R2_peak_2	Distal Interger	1	1.69E+08	1.70E+08	33531	2	66977	uc007dlj.1	361209	ENSMUSG000	Nuf2	NUF2, NDC80 kinetochore complex component
chr1	169176678	169176816	139	140	169176744	21	8.43286	3.95423	5.34896	Adipose_R1_peak_1	Distal Interger	1	1.69E+08	1.70E+08	33531	2	66977	uc007dlj.1	354648	ENSMUSG000	Nuf2	NUF2, NDC80 kinetochore complex component
chr1	169197525	169197702	178	179	169197611	33	8.83596	3.1354	5.68652	Adipose_R2_peak_2	Distal Interger	1	1.69E+08	1.70E+08	33531	2	66977	uc007dlj.1	333762	ENSMUSG000	Nuf2	NUF2, NDC80 kinetochore complex component
chr1	169427710	169427874	165	166	169427795	22	9.13959	4.13397	6.01526	Adipose_R1_peak_1	Distal Interger	1	1.69E+08	1.70E+08	33531	2	66977	uc007dlj.1	103590	ENSMUSG000	Nuf2	NUF2, NDC80 kinetochore complex component
chr1	169535872	169536059	188	189	169535977	27	12.93148	5.03266	9.63827	Adipose_R1_peak_1	Distal Interger	1	1.69E+08	1.70E+08	33531	2	66977	uc007dlj.1	-4408	ENSMUSG000	Nuf2	NUF2, NDC80 kinetochore complex component
chr1	169535913	169536060	148	149	169535997	35	9.95877	3.31984	6.73562	Adipose_R2_peak_2	Distal Interger	1	1.69E+08	1.70E+08	33531	2	66977	uc007dlj.1	-4449	ENSMUSG000	Nuf2	NUF2, NDC80 kinetochore complex component
chr1	169701882	169702003	122	123	169701940	27	5.77405	2.5821	2.88921	Adipose_R2_peak_2	Distal Interger	1	1.70E+08	1.70E+08	6166	2	19736	uc007dln.1	45639	ENSMUSG000	Rgs4	regulator of G-protein signaling 4
chr1	169701886	169702036	151	152	169701955	21	8.43286	3.95423	5.34896	Adipose_R1_peak_1	Distal Interger	1	1.70E+08	1.70E+08	6166	2	19736	uc007dln.1	45606	ENSMUSG000	Rgs4	regulator of G-protein signaling 4
chr1	170061896	170062029	134	135	170062012	21	8.43286	3.95423	5.34896	Adipose_R1_peak_1	Intron (uc011v	1	1.70E+08	1.70E+08	116638	2	18214	uc011wvr.1	26915	ENSMUSG000	Ddr2	discoidin domain receptor family, member 2
chr1	170116481	170116746	266	267	170116628	57	41.13291	10.08101	37.25286	Adipose_R1_peak_1	Distal Interger	1	1.70E+08	1.70E+08	138253	2	18214	uc007dlu.2	-5922	ENSMUSG000	Ddr2	discoidin domain receptor family, member 2
chr1	170116535	170116742	208	209	170116641	52	21.12641	4.87465	17.45173	Adipose_R2_peak_2	Distal Interger	1	1.70E+08	1.70E+08	138253	2	18214	uc007dlu.2	-5976	ENSMUSG000	Ddr2	discoidin domain receptor family, member 2
chr1	170169073	170169268	196	197	170169187	29	6.74062	2.76653	3.75989	Adipose_R2_peak_2	Intron (uc007c	1	1.70E+08	1.70E+08	33742	2	107652	uc007dlw.2	5696	ENSMUSG000	Uap1	UDP-N-acetylglucosamine pyrophosphorylase 1
chr1	170169100	170169234	135	136	170169125	16	5.20017	3.05554	2.38734	Adipose_R1_peak_1	Intron (uc007c	1	1.70E+08	1.70E+08	33742	2	107652	uc007dlw.2	5730	ENSMUSG000	Uap1	UDP-N-acetylglucosamine pyrophosphorylase 1
chr1	171063770	171064885	1116	1117	171063970	64	15.98501	3.26265	12.47609	Adipose_R2_peak_2	Promoter (<=	1	1.71E+08	1.71E+08	62	2	1.02E+08	uc033fnc.1	0	ENSMUSG000	Mir6546	microRNA 6546
chr1	171065266	171065535	270	271	171065417	72	11.69523	2.48437	8.38587	Adipose_R2_peak_2	Promoter (<=	1	1.71E+08	1.71E+08	62	2	1.02E+08	uc033fnc.1	-614	ENSMUSG000	Mir6546	microRNA 6546
chr1	171065383	171065518	136	137	171065439	44	7.12152	2.35453	4.12865	Adipose_R1_peak_1	Promoter (<=	1	1.71E+08	1.71E+08	62	2	1.02E+08	uc033fnc.1	-731	ENSMUSG000	Mir6546	microRNA 6546
chr1	171065996	171066311	316	317	171066130	72	17.28061	3.20457	13.72302	Adipose_R2_peak_2	Promoter (1-2	1	1.71E+08	1.71E+08	62	2	1.02E+08	uc033fnc.1	-1344	ENSMUSG000	Mir6546	microRNA 6546
chr1	171066600	171067263	664	665	171066718	60	11.89539	2.75814	8.56537	Adipose_R2_peak_2	Promoter (1-2	1	1.71E+08	1.71E+08	62	2	1.02E+08	uc033fnc.1	-1948	ENSMUSG000	Mir6546	microRNA 6546
chr1	171068319	171068664	346	347	171068511	78	19.03414	3.26809	15.41245	Adipose_R2_peak_2	Distal Interger	1	1.71E+08	1.71E+08	62	2	1.02E+08	uc033fnc.1	-3667	ENSMUSG000	Mir6546	microRNA 6546
chr1	171068874	171069427	554	555	171069165	103	20.13266	2.85897	16.48328	Adipose_R2_peak_2	Distal Interger	1	1.71E+08	1.71E+08	62	2	1.02E+08	uc033fnc.1	-4222	ENSMUSG000	Mir6546	microRNA 6546
chr1	171069591	171070150	560	561	171069691	77	19.35562	3.33816	15.72872	Adipose_R2_peak_2	Distal Interger	1	1.71E+08	1.71E+08	62	2	1.02E+08	uc033fnc.1	-4939	ENSMUSG000	Mir6546	microRNA 6546
chr1	171069622	171069778	157	158	171069739	40	7.00556	2.43346	4.05838	Adipose_R1_peak_1	Distal Interger	1	1.71E+08	1.71E+08	62	2	1.02E+08	uc033fnc.1	-4970	ENSMUSG000	Mir6546	microRNA 6546
chr1	171070625	171070962	338	339	171070796	69	23.81464	4.3156	20.06764	Adipose_R2_peak_2	Distal Interger	1	1.71E+08	1.71E+08	62	2	1.02E+08	uc033fnc.1	-5973	ENSMUSG000	Mir6546	microRNA 6546
chr1	171071156	171071464	309	310	171071330	65	21.59911	4.13897	17.90824	Adipose_R2_peak_2	Distal Interger	1	1.71E+08	1.71E+08	62	2	1.02E+08	uc033fnc.1	-6504	ENSMUSG000	Mir6546	microRNA 6546
chr1	171073911	171074111	201	202	171073966	33	8.14106	2.95238	5.05855	Adipose_R2_peak_2	Distal Interger	1	1.71E+08	1.71E+08	62	2	1.02E+08	uc033fnc.1	-9259	ENSMUSG000	Mir6546	microRNA 6546
chr1	171075241	171075925	685	686	171075382	69	26.0114	4.69828	22.21377	Adipose_R2_peak_2	Distal Interger	1	1.71E+08	1.71E+08	62	2	1.02E+08	uc033fnc.1	-10589	ENSMUSG000	Mir6546	microRNA 6546
chr1	171075257	171075664	408	409	171075350	51	19.29939	4.52855	15.82055	Adipose_R1_peak_1	Distal Interger	1	1.71E+08	1.71E+08	62	2	1.02E+08	uc033fnc.1	-10605	ENSMUSG000	Mir6546	microRNA 6546
chr1	171076335	171076548	214	215	171076409	59	26.5291	5.53306	22.7188	Adipose_R2_peak_2	Distal Interger	1	1.71E+08	1.71E+08	62	2	1.02E+08	uc033fnc.1	-11683	ENSMUSG000	Mir6546	microRNA 6546
chr1	171076347	171076487	141	142	171076409	32	9.50133	3.37797	6.37392	Adipose_R1_peak_1	Distal Interger	1	1.71E+08	1.71E+08	62	2	1.02E+08	uc033fnc.1	-11695	ENSMUSG000	Mir6546	microRNA 6546

chr1	171077208	171077376	169	170	171077282	31	7.7623	2.95097	4.69388	Adipose_R2_peak_2	Distal Intergen	1	1.71E+08	1.71E+08	62	2	1.02E+08	uc033fnc.1	-12556	ENSMUSG000	Mir6546	microRNA 6546
chr1	171078111	171078252	142	143	171078175	33	10.62926	3.63271	7.43208	Adipose_R1_peak_1	Distal Intergen	1	1.71E+08	1.71E+08	62	2	1.02E+08	uc033fnc.1	-13459	ENSMUSG000	Mir6546	microRNA 6546
chr1	171078141	171078295	155	156	171078194	42	14.2409	3.96536	10.79479	Adipose_R2_peak_2	Distal Intergen	1	1.71E+08	1.71E+08	62	2	1.02E+08	uc033fnc.1	-13489	ENSMUSG000	Mir6546	microRNA 6546
chr1	171078661	171078877	217	218	171078764	59	21.47193	4.45175	17.78305	Adipose_R2_peak_2	Distal Intergen	1	1.71E+08	1.71E+08	62	2	1.02E+08	uc033fnc.1	-14009	ENSMUSG000	Mir6546	microRNA 6546
chr1	171081613	171081805	193	194	171081648	28	5.55735	2.47963	2.71575	Adipose_R2_peak_2	Distal Intergen	1	1.71E+08	1.71E+08	62	2	1.02E+08	uc033fnc.1	-16961	ENSMUSG000	Mir6546	microRNA 6546
chr1	171830886	171831081	196	197	171830977	31	16.23981	5.75161	12.83549	Adipose_R1_peak_1	Distal Intergen	1	1.72E+08	1.72E+08	15401	1	12523	uc007dpd.3	-8616	ENSMUSG000	Cd84	CD84 antigen
chr1	171858848	171859087	240	241	171858965	42	23.79978	6.79619	20.21142	Adipose_R1_peak_1	Intron (uc007c	1	1.72E+08	1.72E+08	15401	1	12523	uc007dpd.3	19151	ENSMUSG000	Cd84	CD84 antigen
chr1	171858886	171859046	161	162	171858964	40	12.96465	3.78093	9.57654	Adipose_R2_peak_2	Intron (uc007c	1	1.72E+08	1.72E+08	15401	1	12523	uc007dpd.3	19189	ENSMUSG000	Cd84	CD84 antigen
chr1	171875711	171875850	140	141	171875790	24	10.60668	4.49345	7.40961	Adipose_R1_peak_1	Intron (uc007c	1	1.72E+08	1.72E+08	15401	1	12523	uc007dpd.3	36014	ENSMUSG000	Cd84	CD84 antigen
chr1	171876749	171876899	151	152	171876887	19	6.91816	3.52644	3.97188	Adipose_R1_peak_1	Intron (uc007c	1	1.72E+08	1.72E+08	15401	1	12523	uc007dpd.3	37052	ENSMUSG000	Cd84	CD84 antigen
chr1	171878028	171878267	240	241	171878174	26	12.14079	4.85292	8.87838	Adipose_R1_peak_1	Intron (uc007c	1	1.72E+08	1.72E+08	15401	1	12523	uc007dpd.3	38331	ENSMUSG000	Cd84	CD84 antigen
chr1	171878068	171878223	156	157	171878169	30	7.24477	2.85875	4.21917	Adipose_R2_peak_2	Intron (uc007c	1	1.72E+08	1.72E+08	15401	1	12523	uc007dpd.3	38371	ENSMUSG000	Cd84	CD84 antigen
chr1	172717859	172717998	140	141	172717922	20	7.74501	3.7745	4.70467	Adipose_R1_peak_1	Distal Intergen	1	1.73E+08	1.73E+08	1911	1	12944	uc011wwq.1	19803	ENSMUSG000	Crp	C-reactive protein, pentraxin-related
chr1	172721624	172722061	438	439	172721824	391	363.4793	20.59895	358.6709	Adipose_R1_peak_1	Distal Intergen	1	1.73E+08	1.73E+08	1911	1	12944	uc011wwq.1	23568	ENSMUSG000	Crp	C-reactive protein, pentraxin-related
chr1	172721681	172722044	364	365	172721827	308	222.1924	12.49919	217.4199	Adipose_R2_peak_2	Distal Intergen	1	1.73E+08	1.73E+08	1911	1	12944	uc011wwq.1	23625	ENSMUSG000	Crp	C-reactive protein, pentraxin-related
chr1	172878737	172879106	370	371	172878873	26	12.14079	4.85292	8.87838	Adipose_R1_peak_1	Distal Intergen	1	1.73E+08	1.73E+08	1094	2	20219	uc007dqy.2	15948	ENSMUSG000	Apcs	serum amyloid P-component
chr1	172879625	172879767	143	144	172879709	32	8.29282	3.04319	5.18352	Adipose_R2_peak_2	Distal Intergen	1	1.73E+08	1.73E+08	1094	2	20219	uc007dqy.2	15287	ENSMUSG000	Apcs	serum amyloid P-component
chr1	172879637	172879789	153	154	172879715	25	11.36566	4.67319	8.13482	Adipose_R1_peak_1	Distal Intergen	1	1.73E+08	1.73E+08	1094	2	20219	uc007dqy.2	15265	ENSMUSG000	Apcs	serum amyloid P-component
chr1	172883826	172884054	229	230	172883930	42	26.36523	7.72873	22.71906	Adipose_R1_peak_1	Distal Intergen	1	1.73E+08	1.73E+08	1094	2	20219	uc007dqy.2	11000	ENSMUSG000	Apcs	serum amyloid P-component
chr1	172883878	172883974	97	98	172883928	30	7.24477	2.85875	4.21917	Adipose_R2_peak_2	Distal Intergen	1	1.73E+08	1.73E+08	1094	2	20219	uc007dqy.2	11080	ENSMUSG000	Apcs	serum amyloid P-component
chr1	173260175	173260430	256	257	173260288	45	29.34988	8.26794	25.64927	Adipose_R1_peak_1	Distal Intergen	1	1.73E+08	1.73E+08	991	1	258645	uc007drf.2	-9717	ENSMUSG000	Olfr418	olfactory receptor 418
chr1	173260216	173260368	153	154	173260292	35	9.95877	3.31984	6.73562	Adipose_R2_peak_2	Distal Intergen	1	1.73E+08	1.73E+08	991	1	258645	uc007drf.2	-9779	ENSMUSG000	Olfr418	olfactory receptor 418
chr1	173837864	173838128	265	266	173838001	38	22.52591	7.00978	18.96	Adipose_R1_peak_1	Intron (uc029c	1	1.74E+08	1.74E+08	11541	2	1.00E+08	uc007dry.2	30632	ENSMUSG000	Mndal	myeloid nuclear differentiation antigen like
chr1	173837874	173838096	223	224	173837981	44	15.55653	4.1498	12.05625	Adipose_R2_peak_2	Intron (uc029c	1	1.74E+08	1.74E+08	11541	2	1.00E+08	uc007dry.2	30664	ENSMUSG000	Mndal	myeloid nuclear differentiation antigen like
chr1	174309786	174310089	304	305	174309913	47	31.38649	8.62742	27.65048	Adipose_R1_peak_1	Distal Intergen	1	1.74E+08	1.74E+08	2360	1	66289	uc007dsy.2	-20429	ENSMUSG000	Mptx1	mucosal pentraxin 1
chr1	174309873	174310041	169	170	174309918	29	6.74062	2.76653	3.75989	Adipose_R2_peak_2	Distal Intergen	1	1.74E+08	1.74E+08	2360	1	66289	uc007dsy.2	-20477	ENSMUSG000	Mptx1	mucosal pentraxin 1
chr1	174361291	174361631	341	342	174361456	61	46.56983	11.14375	42.62347	Adipose_R1_peak_1	Distal Intergen	1	1.74E+08	1.74E+08	930	1	258238	uc007dsz.1	-7288	ENSMUSG000	Olfr417	olfactory receptor 417
chr1	174361311	174361591	281	282	174361464	57	24.96362	5.34863	21.18596	Adipose_R2_peak_2	Distal Intergen	1	1.74E+08	1.74E+08	930	1	258238	uc007dsz.1	-7328	ENSMUSG000	Olfr417	olfactory receptor 417
chr1	174379874	174380003	130	131	174379927	32	8.29282	3.04319	5.18352	Adipose_R2_peak_2	Distal Intergen	1	1.74E+08	1.74E+08	930	1	258238	uc007dsz.1	10955	ENSMUSG000	Olfr417	olfactory receptor 417
chr1	174631460	174631641	182	183	174631607	30	7.24477	2.85875	4.21917	Adipose_R2_peak_2	Intron (uc007c	1	1.75E+08	1.75E+08	87902	1	54418	uc007dte.1	18953	ENSMUSG000	Fmn2	formin 2
chr1	174631468	174631666	199	200	174631610	21	8.43286	3.95423	5.34896	Adipose_R1_peak_1	Intron (uc007c	1	1.75E+08	1.75E+08	87902	1	54418	uc007dte.1	18961	ENSMUSG000	Fmn2	formin 2
chr1	174707886	174708182	297	298	174707997	88	71.15864	13.21092	66.98766	Adipose_R1_peak_1	Intron (uc007c	1	1.75E+08	1.75E+08	87902	1	54418	uc007dte.1	95379	ENSMUSG000	Fmn2	formin 2
chr1	174707895	174708172	278	279	174707996	74	39.12209	6.91633	35.10124	Adipose_R2_peak_2	Intron (uc007c	1	1.75E+08	1.75E+08	87902	1	54418	uc007dte.1	95388	ENSMUSG000	Fmn2	formin 2
chr1	174710706	174710976	271	272	174710843	43	27.35048	7.90847	23.68561	Adipose_R1_peak_1	Intron (uc007c	1	1.75E+08	1.75E+08	87902	1	54418	uc007dte.1	98199	ENSMUSG000	Fmn2	formin 2
chr1	174710747	174710948	202	203	174710841	44	15.55653	4.1498	12.05625	Adipose_R2_peak_2	Intron (uc007c	1	1.75E+08	1.75E+08	87902	1	54418	uc007dte.1	98240	ENSMUSG000	Fmn2	formin 2
chr1	174741469	174741706	238	239	174741613	25	11.36566	4.67319	8.13482	Adipose_R1_peak_1	Intron (uc007c	1	1.75E+08	1.75E+08	87902	1	54418	uc007dte.1	128962	ENSMUSG000	Fmn2	formin 2
chr1	174784648	174785046	399	400	174784879	64	41.94308	8.99208	38.05985	Adipose_R1_peak_1	Intron (uc007c	1	1.75E+08	1.75E+08	88035	2	23893	uc007dtf.1	136773	ENSMUSG000	Grem2	gremlin 2, DAN family BMP antagonist
chr1	174784725	174785029	305	306	174784923	54	22.67101	5.07198	18.94758	Adipose_R2_peak_2	Intron (uc007c	1	1.75E+08	1.75E+08	88035	2	23893	uc007dtf.1	136790	ENSMUSG000	Grem2	gremlin 2, DAN family BMP antagonist
chr1	175046955	175047112	158	159	175047049	27	12.93148	5.03266	9.63827	Adipose_R1_peak_1	Distal Intergen	1	1.75E+08	1.75E+08	20516	2	24012	uc007dtg.2	32479	ENSMUSG000	Rgs7	regulator of G protein signaling 7
chr1	175100903	175101004	102	103	175100954	28	6.25023	2.67431	3.31626	Adipose_R2_peak_2	Intron (uc007c	1	1.75E+08	1.75E+08	27151	2	24012	uc007dtj.2	-14677	ENSMUSG000	Rgs7	regulator of G protein signaling 7
chr1	175223435	175223634	200	201	175223518	23	9.86443	4.31371	6.70247	Adipose_R1_peak_1	Intron (uc007c	1	1.75E+08	1.75E+08	27151	2	24012	uc007dtj.2	-137209	ENSMUSG000	Rgs7	regulator of G protein signaling 7
chr1	175234810	175235092	283	284	175234964	57	42.07807	10.4248	38.18469	Adipose_R1_peak_1	Intron (uc007c	1	1.75E+08	1.75E+08	27151	2	24012	uc007dtj.2	-148584	ENSMUSG000	Rgs7	regulator of G protein signaling 7
chr1	175234818	175235090	273	274	175234946	56	24.19188	5.25641	20.43163	Adipose_R2_peak_2	Intron (uc007c	1	1.75E+08	1.75E+08	27151	2	24012	uc007dtj.2	-148592	ENSMUSG000	Rgs7	regulator of G protein signaling 7
chr1	175398891	175399058	168	169	175398952	20	7.74501	3.7745	4.70467	Adipose_R1_peak_1	Intron (uc007c	1	1.75E+08	1.75E+08	433470	2	24012	uc007dtk.2	93487	ENSMUSG000	Rgs7	regulator of G protein signaling 7
chr1	175454005	175454202	198	199	175454026	18	6.42934	3.41502	3.48999	Adipose_R1_peak_1	Intron (uc007c	1	1.75E+08	1.75E+08	433470	2	24012	uc007dtk.2	38343	ENSMUSG000	Rgs7	regulator of G protein signaling 7
chr1	176551648	176551843	196	197	176551762	27	10.92219	4.25989	7.72273	Adipose_R1_peak_1	Distal Intergen	1	1.77E+08	1.77E+08	16859	2	545389	uc007dub.1	198668	ENSMUSG000	Cep170	centrosomal protein 170
chr1	176551700	176551859	160	161	176551780	36	10.5378	3.41206	7.27949	Adipose_R2_peak_2	Distal Intergen	1	1.77E+08	1.77E+08	16859	2	545389	uc007dub.1	198652	ENSMUSG000	Cep170	centrosomal protein 170
chr1	176893444	176893660	217	218	176893551	30	15.39182	5.57188	12.01394	Adipose_R1_peak_1	Intron (uc011v	1	1.77E+08	1.77E+08	1510	2	15365	uc029quu.1	-57311	NA	Hmga2-ps1	high mobility group AT-hook 2, pseudogene 1
chr1	176898482	176898727	246	247	176898605	45	29.34988	8.26794	25.64927	Adipose_R1_peak_1	Intron (uc011v	1	1.77E+08	1.77E+08	1510	2	15365	uc029quu.1	-62349	NA	Hmga2-ps1	high mobility group AT-hook 2, pseudogene 1
chr1	176898507	176898696	190	191	176898635	38	11.72962	3.59649	8.4047	Adipose_R2_peak_2	Intron (uc011v	1	1.77E+08	1.77E+08	1510	2	15365	uc029quu.1	-62374	NA	Hmga2-ps1	high mobility group AT-hook 2, pseudogene 1
chr1	177899706	177899911	206	207	177899831	27	12.93148	5.03266	9.63827	Adipose_R1_peak_1	Distal Intergen	1	1.78E+08	1.78E+08	166540	1	545391	uc011wxj.1	-83512	ENSMUSG000	Gm16432	predicted gene 16432
chr1	177920362	177920510	149	150	177920448	19	7.07687	3.59476	4.08458	Adipose_R1_peak_1	Distal Intergen	1	1.78E+08	1.78E+08	166540	1	545391	uc011wxj.1	-62913	ENSMUSG000	Gm16432	predicted gene 16432
chr1	177983913	177984041	129	130	177984021	26	5.31252	2.48988	2.47999	Adipose_R2_peak_2	Promoter (<=1	1	1.78E+08	1.78E+08	166540	1	545391	uc011wxj.1	490	ENSMUSG000	Gm16432	predicted gene 16432
chr1	178008952	178009152	201	202	178009058	26	12.14079	4.85292	8.87838	Adipose_R1_peak_1	Intron (uc011v	1	1.78E+08	1.78E+08	56857	1	545391	uc007duv.2	17517	ENSMUSG000	Gm16432	predicted gene 16432
chr1	178083080	178083284	205	206	178083198	49	33.45876	8.98689	29.68925	Adipose_R1_peak_1	Intron (uc011v	1	1.78E+08	1.78E+08	56857	1	545391	uc007duv.2	91645	ENSMUSG000	Gm16432	predicted gene 16432
chr1	178083093	178083279	187	188	178083213	44	15.55653	4.1498	12.05625	Adipose_R2_peak_2	Intron (uc011v	1	1.78E+08	1.78E+08	56857	1	545391	uc007duv.2	91658	ENSMUSG000	Gm16432	predicted gene 16432
chr1	178131740	178131928	189	190	178131858	22	9.13959	4.13397	6.01526	Adipose_R1_peak_1	Intron (uc011v	1	1.78E+08	1.78E+08	65181	1	78825	uc007dux.1	-55489	ENSMUSG000	Desi2	desumoylating isopeptidase 2
chr1	178174521	178174720	200	201	178174612	51	20.44827	4.79532	16.78434	Adipose_R2_peak_2	Distal Intergen	1	1.78E+08	1.78E+08	65181	1	78825	uc007dux.1	-12697	ENSMUSG000	Desi2	desumoylating isopeptidase 2
chr1	178499241	178499529	289	290	178499379	79	43.62032	7.37742	39.5398	Adipose_R2_peak_2	Distal Intergen	1	1.79E+08	1.79E+08	187376	1	269152	uc007dvf.1	-29596	ENSMUSG000	Kif26b	kinesin family member 26B
chr1	178499243	178499511	269	270	178499375	49	32.65486	8.69053	28.9009	Adipose_R1_peak_1	Distal Intergen	1	1.79E+08	1.79E+08	187376	1	269152	uc007dvf.1	-29614	ENSMUSG000	Kif26b	kinesin family member 26B
chr1	178499926	178500097	172	173	178500032	20	7.74501	3.7745	4.70467	Adipose_R1_peak_1	Distal Intergen	1	1.79E+08	1.79E+08	187376	1	269152	uc007dvf.1	-29028	ENSMUSG000	Kif26b	kinesin family member 26B
chr1	178499964	178500058	95	96	178500009	25	4.86612	2.39766	2.09018	Adipose_R2_peak_2	Distal Intergen	1	1.79E+08	1.79E+08	187376	1	269152	uc007dvf.1	-29067	ENSMUSG000	Kif26b	kinesin family member 26B
chr1	178501312	178501460	149	150	178501401	23	9.86443	4.31371	6.70247	Adipose_R1_peak_1	Distal Intergen	1	1.79E+08	1.79E+08	187376	1	269152	uc007dvf.1	-27665	ENSMUSG000	Kif26b	kinesin family member 26B
chr1	178739817	178739977	161	162	178739879	18	6.42934	3.41502	3.48999	Adipose_R1_peak_1	Intron (uc007c	1	1.79E+08	1.79E+08	187376	1	269152	uc007dvf.1	210692	ENSMUSG000	Kif26b	kinesin family member 26B
chr1	179896737	179896914	178	179	179896840	27	11.52295	4.4835	8.29096	Adipose_R1_peak_1	Distal Intergen	1	1.80E+08	1.80E+08	140184	1	226751	uc007dvs.2	-64175	ENSMUSG000	Cdc42bpa	CDC42 binding protein kinase alpha
chr1	179896806	179896902	97	98	179896851	32	8.29282	3.04319	5.18352	Adipose_R2_peak_2	Distal Intergen	1	1.80E+08	1.80E+08	140184	1	226751	uc007dvs.2	-64187	ENSMUSG000	Cdc42bpa	CDC42 binding protein kinase alpha
chr1	179898933	179899125	193	194	179899070	19	7.07687	3.59476	4.08458	Adipose_R1_peak_1	Distal Intergen	1	1.80E+08	1.80E+08	140184	1	226751	uc007dvs.2	-61964	ENSMUSG000	Cdc42bpa	CDC42 binding protein kinase alpha
chr1	179899616	179899766	151	152	179899683	20	7.74501	3.7745	4.70467	Adipose_R1_peak_1	Distal Intergen	1	1.80E+08	1.80E+08	140184	1	226751	uc007dvs.2	-61323	ENSMUSG000	Cdc42bpa	CDC42 binding protein kinase alpha
chr1	180242290	180242803	514	515	180242363	20	7.74501	3.7745	4.70467	Adipose_R1_peak_1	Promoter (2-3	1	1.80E+08	1.80E+08	18754	2	19165	uc007dwb.2	2954	ENSMUSG000	Psen2	presenilin 2
chr1	180242464	180242657	194	195	180242565	35	8.87632	3.04249	5.72549	Adipose_R2_peak_2	Intron (uc007c	1	1.80E+08	1.80E+08	18754	2	19165	uc007dwb.2	3100	ENSMUSG000	Psen2	presenilin 2
chr1	180244163	180244370	208	209	180244263	23	8.56759	3.79322	5.48223	Adipose_R1_peak_1	Promoter (1-2	1	1.80E+08	1.80E+08	18754	2	19165	uc007dwb.2	1387	ENSMUSG000	Psen2	presenilin 2
chr1	180244623	180244750	128	129	180244714	29	6.74062	2.76653	3.75989	Adipose_R2_peak_2	Promoter (1-2	1	1.80E+08	1.80E+08	18754	2	19165	uc007dwb.2	1007	ENSMUSG000	Psen2	presenilin 2
chr1	180342529	180342691	163	164	180342607	17	5.80341	3.23528	2.92378	Adipose_R1_peak_1	Intron (uc007c	1	1.80E+08	1.80E+08	3581	2	277333	uc007dwf.1	-11980	NA	Gm5069	glyceraldehyde-3-phosphate dehydrogenase pseudogene
chr1	180390812	180390958	147	148	180390909	29	6.74062	2.76653	3.75989	Adipose_R2_peak_2	Intron (uc007c	1	1.80E+08	1.80E+08	3581	2	277333	uc007dwf.1	-60263	NA	Gm5069	glyceraldehyde-3-phosphate dehydrogenase pseudogene
chr1	180392824	180392967	144	145	180392878	25	4.86612	2.39766	2.09018	Adipose_R2_peak_2	Intron (uc007c	1	1.80E+08	1.80E+08	3581	2	277333	uc007dwf.1	-62275	NA	Gm5069	glyceraldehyde-3-phosphate dehydrogenase pseudogene
chr1	181495283	181495601	319	320	181495452	55	39.87613	10.06532	36.01168	Adipose_R1_peak_1	Distal Intergen	1	1.82E+08	1.82E+08	1819	2	403180	uc011wxt.1	15850	ENSMUSG000	Ccdc121	coiled-coil domain containing 121
chr1	181495284	181495599	316	317	181495444	74	39.12209	6.91633	35.10124	Adipose_R2_peak_2	Distal Intergen	1	1.82E+08	1.82E+08	1819	2	403180	uc011wxt.1	15852	ENSMUSG000	Ccdc121	coiled-coil domain containing 121
chr1	181504727	181504866	140	141	181504837	18	6.42934	3.41502	3.48999	Adipose_R1_peak_1	Distal Intergen	1	1.82E+08	1.82E+08	1819	2	403180	uc011wxt.1	6585	ENSMUSG000	Ccdc121	coiled-coil domain containing 121
chr1	181743026	181743211	186	187	181743118	51	17.30509	4.08819	13.87435	Adipose_R1_peak_1	Intron (uc007c	1	1.82E+08	1.82E+08	4738	2	98386	uc007dxn.2	76841	ENSMUSG000	Lbr	lamin B receptor
chr1	181743118	181743214	97	98	181743158	51	9.63868	2.62821	6.44334	Adipose_R2_peak_2	Intron (uc007c	1	1.82E+08	1.82E+08	4738	2	98386	uc007dxn.2	76838	ENSMUSG000	Lbr	lamin B receptor
chr1	181743501	181743761	261	262	181743636	38	7.4307	2.58342	4.43407	Adipose_R1_peak_1	Intron (uc007c	1	1.82E+08	1.82E+08	4738	2	98386	uc007dxn.2	76291	ENSMUSG000	Lbr	lamin B receptor
chr1	181743534	181743740	207	208	181743636	65	16.23434	3.26786	12.70833	Adipose_R2_peak_2	Intron (uc007c	1	1.82E+08	1.82E+08	4738	2	98386	uc007dxn.2	76312	ENSMUSG000	Lbr	lamin B receptor
chr1	182629724	182629910	187	188	182629834	40	24.42476	7.36925	20.81738	Adipose_R1_peak_1	Intron (uc007c	1	1.83E+08	1.83E+08	41520	1	170725	uc007dyh.2	64717	ENSMUSG000	Capn8	calpain 8
chr1	182629755	182629909	155	156	182629860	37	11.12817	3.50427	7.83613	Adipose_R2_peak_2	Intron (uc007c	1	1.83E+08	1.83E+08	41520	1	170725	uc007dyh.2	64748	ENSMUSG000	Capn8	calpain 8
chr1	183174425	183174593	169	170	183174503	29	14.55748	5.39214	11.20652	Adipose_R1_peak_1	Intron (uc008i	1	1.83E+08	1.83E+08	61294	2	68897	uc008ick.2	-26868	ENSMUSG000	Disp1	dispatched homolog 1 (Drosophila)
chr1	183174456	183174632	177	178	183174530	40	12.96465	3.78093	9.57654	Adipose_R2_peak_2	Intron (uc008i	1	1.83E+08	1.83E+08	61294	2	68897	uc008ick.2	-26899	ENSMUSG000	Disp1	dispatched homolog 1 (Drosophila)
chr1	183299136	183299581	446	447	183299335	454	47.52753	2.15004	43.57695	Adipose_R1_peak_1	Promoter (1-2	1	1.83E+08	1.83E+08	20915	2	71678	uc008ics.1	-1880	ENSMUSG000	Brox	BRO1 domain and CAAX motif containing
chr1	183299146	183299571	426	427	183299354	983	306.7464	4.40722	301.8696	Adipose_R2_peak_2	Promoter (1-2	1	1.83E+08	1.83E+08	20915	2	71678	uc008ics.1	-1890	ENSMUSG000	Brox	BRO1 domain and CAAX motif containing
chr1	183567586	183567752	167	168	183567664	23	9.86443	4.31371	6.70247	Adipose_R1_peak_1	Distal Intergen	1	1.83E+08	1.83E+08	17806	1	78772	uc057awa.1	148338	ENSMUSG000	Hhipl2	hedgehog interacting protein-like 2
chr1	183570452	183570658	207	208	183570569	34	18.55215	6.17128	15.09587	Adipose_R1_peak_1	Distal Intergen	1	1.83E+08	1.83E+08	17806	1	78772	uc057awa.1	151204	ENSMUSG000	Hhipl2	hedgehog interacting protein-like 2
chr1	183570509	183570658	150	151	183570633	26	5.31252	2.48988	2.47999	Adipose_R2_peak_2	Distal Intergen	1	1.83E+08	1.83E+08	17806	1	78772	uc057awa.1	151261	ENSMUSG000	Hhipl2	hedgehog interacting protein-like 2
chr1	183617543	183617706	164	165	183617652	17	5.80341	3.23528	2.92378	Adipose_R1_peak_1	Distal Intergen	1	1.83E+08	1.83E+08	17806	1	78772	uc057awa.1	198295	ENSMUSG000	Hhipl2	hedgehog interacting protein-like 2
chr1	184635982	184636255	274	275	184636096	33	17.97508	6.11109	14.52164	Adipose_R1_peak_1	Distal Intergen	1	1.85E+08	1.85E+08	6493	1	74971	uc029qvc.1	-76242	ENSMUSG000	4930488B22Ri	RIKEN cDNA 4930488B22 gene
chr1	184636013	184636121	109	110	184636061	28	6.25023	2.67431	3.31626	Adipose_R2_peak_2	Distal Intergen	1	1.85E+08	1.85E+08	6493	1	74971	uc029qvc.1	-76376	ENSMUSG000	4930488B22Ri	RIKEN cDNA 4930488B22 gene

chr1	184697296	184697455	160	161	184697373	20	7.74501	3.7745	4.70467	Adipose_R1_peak_1	Distal Interger	1	1.85E+08	1.85E+08	6493	1	74971	uc029qvc.1	-15042	ENSMUSG000	4930488B22Ri	RIKEN cDNA 4930488B22 gene
chr1	184732383	184732555	173	174	184732483	19	7.07687	3.59476	4.08458	Adipose_R1_peak_1	Promoter (<=1	1	1.85E+08	1.85E+08	5349	2	15284	uc007dyn.2	0	ENSMUSG000	Hlx	H2.0-like homeobox
chr1	184733954	184734079	126	127	184733992	26	5.31252	2.48988	2.47999	Adipose_R2_peak_2	Promoter (1-2	1	1.85E+08	1.85E+08	5349	2	15284	uc007dyn.2	-1461	ENSMUSG000	Hlx	H2.0-like homeobox
chr1	184734234	184734329	96	97	184734277	30	7.24477	2.85875	4.21917	Adipose_R2_peak_2	Promoter (1-2	1	1.85E+08	1.85E+08	5349	2	15284	uc007dyn.2	-1741	ENSMUSG000	Hlx	H2.0-like homeobox
chr1	184753305	184753768	464	465	184753430	38	22.52591	7.00978	18.96	Adipose_R1_peak_1	Distal Interger	1	1.85E+08	1.85E+08	5349	2	15284	uc007dyn.2	-20812	ENSMUSG000	Hlx	H2.0-like homeobox
chr1	185031213	185031469	257	258	185031314	17	5.80341	3.23528	2.92378	Adipose_R1_peak_1	Distal Interger	1	1.85E+08	1.85E+08	103126	2	226778	uc007dyt.2	-31664	ENSMUSG000	Mark1	MAP/microtubule affinity regulating kinase 1
chr1	185050464	185050724	261	262	185050495	30	7.24477	2.85875	4.21917	Adipose_R2_peak_2	Distal Interger	1	1.85E+08	1.85E+08	103126	2	226778	uc007dyt.2	-50915	ENSMUSG000	Mark1	MAP/microtubule affinity regulating kinase 1
chr1	185566843	185567036	194	195	185566921	48	18.29934	4.51867	14.70076	Adipose_R2_peak_2	Distal Interger	1	1.85E+08	1.85E+08	709	1	114668	uc011wyd.1	69537	ENSMUSG000	5033404E19Ri	NSA2 ribosome biogenesis homolog pseudogene
chr1	185568190	185568385	196	197	185568267	19	7.07687	3.59476	4.08458	Adipose_R1_peak_1	Distal Interger	1	1.85E+08	1.85E+08	709	1	114668	uc011wyd.1	70884	ENSMUSG000	5033404E19Ri	NSA2 ribosome biogenesis homolog pseudogene
chr1	185605195	185605362	168	169	185605354	31	7.7623	2.95097	4.69388	Adipose_R2_peak_2	Distal Interger	1	1.85E+08	1.85E+08	709	1	114668	uc011wyd.1	107889	ENSMUSG000	5033404E19Ri	NSA2 ribosome biogenesis homolog pseudogene
chr1	185605198	185605362	165	166	185605235	18	6.42934	3.41502	3.48999	Adipose_R1_peak_1	Distal Interger	1	1.85E+08	1.85E+08	709	1	114668	uc011wyd.1	107892	ENSMUSG000	5033404E19Ri	NSA2 ribosome biogenesis homolog pseudogene
chr1	187064785	187065039	255	256	187064912	50	34.50784	9.16663	30.72138	Adipose_R1_peak_1	Intron (uc007c	1	1.87E+08	1.87E+08	2715	1	69308	uc007dzu.1	-60099	ENSMUSG000	1700007P06Ri	RIKEN cDNA 1700007P06 gene
chr1	187064828	187064974	147	148	187064902	38	11.72962	3.59649	8.4047	Adipose_R2_peak_2	Intron (uc007c	1	1.87E+08	1.87E+08	2715	1	69308	uc007dzu.1	-60164	ENSMUSG000	1700007P06Ri	RIKEN cDNA 1700007P06 gene
chr1	187068977	187069310	334	335	187069205	225	265.1175	33.14363	260.3986	Adipose_R1_peak_1	Intron (uc007c	1	1.87E+08	1.87E+08	2715	1	69308	uc007dzu.1	-55828	ENSMUSG000	1700007P06Ri	RIKEN cDNA 1700007P06 gene
chr1	187069006	187069301	296	297	187069221	198	179.2087	18.35133	174.4999	Adipose_R2_peak_2	Intron (uc007c	1	1.87E+08	1.87E+08	2715	1	69308	uc007dzu.1	-55837	ENSMUSG000	1700007P06Ri	RIKEN cDNA 1700007P06 gene
chr1	187196872	187197108	237	238	187197005	30	15.39182	5.57188	12.01394	Adipose_R1_peak_1	Intron (uc007c	1	1.87E+08	1.87E+08	167040	2	74717	uc007dzt.2	18338	ENSMUSG000	Spata17	spermatogenesis associated 17
chr1	187196954	187197074	121	122	187197006	27	5.77405	2.5821	2.88921	Adipose_R2_peak_2	Intron (uc007c	1	1.87E+08	1.87E+08	167040	2	74717	uc007dzt.2	18372	ENSMUSG000	Spata17	spermatogenesis associated 17
chr1	187591063	187591340	278	279	187591203	38	22.52591	7.00978	18.96	Adipose_R1_peak_1	Distal Interger	1	1.88E+08	1.88E+08	605879	1	26381	uc007eaa.2	-17666	ENSMUSG000	Esrrg	estrogen-related receptor gamma
chr1	187591132	187591314	183	184	187591215	33	8.83596	3.1354	5.68652	Adipose_R2_peak_2	Distal Interger	1	1.88E+08	1.88E+08	605879	1	26381	uc007eaa.2	-17692	ENSMUSG000	Esrrg	estrogen-related receptor gamma
chr1	188324681	188324875	195	196	188324722	17	5.80341	3.23528	2.92378	Adipose_R1_peak_1	Intron (uc007e	1	1.88E+08	1.89E+08	702202	1	22283	uc007eaf.1	61843	ENSMUSG000	Ush2a	Usher syndrome 2A
chr1	188611125	188611252	128	129	188611190	32	8.29282	3.04319	5.18352	Adipose_R2_peak_2	Intron (uc007e	1	1.89E+08	1.89E+08	140990	1	22283	uc007eag.1	73427	ENSMUSG000	Ush2a	Usher syndrome 2A
chr1	188982707	188982844	138	139	188982782	20	7.74501	3.7745	4.70467	Adipose_R1_peak_1	Intron (uc007e	1	1.89E+08	1.89E+08	5612	2	226823	uc029qve.1	-6000	ENSMUSG000	Kctd3	potassium channel tetramerisation domain containing 3
chr1	189098307	189098445	139	140	189098413	16	5.20017	3.05554	2.38734	Adipose_R1_peak_1	Distal Interger	1	1.89E+08	1.89E+08	36743	2	226823	uc007eai.1	-90467	ENSMUSG000	Kctd3	potassium channel tetramerisation domain containing 3
chr1	189108083	189108203	121	122	189108143	27	5.77405	2.5821	2.88921	Adipose_R2_peak_2	Distal Interger	1	1.89E+08	1.89E+08	36743	2	226823	uc007eai.1	-100243	ENSMUSG000	Kctd3	potassium channel tetramerisation domain containing 3
chr1	189392115	189392250	136	137	189392191	23	9.86443	4.31371	6.70247	Adipose_R1_peak_1	Intron (uc007e	1	1.89E+08	1.89E+08	194344	2	16526	uc007ean.2	10023	ENSMUSG000	Kcnk2	potassium channel, subfamily K, member 2
chr1	189392705	189393004	300	301	189392838	89	72.88177	13.52388	68.69965	Adipose_R1_peak_1	Intron (uc007e	1	1.89E+08	1.89E+08	194344	2	16526	uc007ean.2	9269	ENSMUSG000	Kcnk2	potassium channel, subfamily K, member 2
chr1	189392716	189392952	237	238	189392831	56	24.19188	5.25641	20.43163	Adipose_R2_peak_2	Intron (uc007e	1	1.89E+08	1.89E+08	194344	2	16526	uc007ean.2	9321	ENSMUSG000	Kcnk2	potassium channel, subfamily K, member 2
chr1	189447945	189448186	242	243	189448069	26	12.14079	4.85292	8.87838	Adipose_R1_peak_1	Distal Interger	1	1.89E+08	1.89E+08	194344	2	16526	uc007ean.2	-45672	ENSMUSG000	Kcnk2	potassium channel, subfamily K, member 2
chr1	189517008	189517191	184	185	189517032	16	5.20017	3.05554	2.38734	Adipose_R1_peak_1	Distal Interger	1	1.89E+08	1.89E+08	194344	2	16526	uc007ean.2	-114735	ENSMUSG000	Kcnk2	potassium channel, subfamily K, member 2
chr1	190058472	190058606	135	136	190058584	19	7.07687	3.59476	4.08458	Adipose_R1_peak_1	Distal Interger	1	1.90E+08	1.90E+08	8878	2	19130	uc007ebc.2	110793	ENSMUSG000	Prox1	prospero homeobox 1
chr1	190300410	190300583	174	175	190300511	32	8.29282	3.04319	5.18352	Adipose_R2_peak_2	Distal Interger	1	1.90E+08	1.90E+08	48904	2	19130	uc033fol.1	-129730	ENSMUSG000	Prox1	prospero homeobox 1
chr1	190300415	190300561	147	148	190300512	25	8.77725	3.68028	5.68994	Adipose_R1_peak_1	Distal Interger	1	1.90E+08	1.90E+08	48904	2	19130	uc033fol.1	-129735	ENSMUSG000	Prox1	prospero homeobox 1
chr1	190301566	190301985	420	421	190301887	26	6.14996	2.7408	3.26482	Adipose_R1_peak_1	Distal Interger	1	1.90E+08	1.90E+08	48904	2	19130	uc033fol.1	-130886	ENSMUSG000	Prox1	prospero homeobox 1
chr1	190301754	190301956	203	204	190301880	34	8.91061	3.10176	5.75872	Adipose_R2_peak_2	Distal Interger	1	1.90E+08	1.90E+08	48904	2	19130	uc033fol.1	-131074	ENSMUSG000	Prox1	prospero homeobox 1
chr1	190302285	190302396	112	113	190302363	31	6.60994	2.64318	3.66189	Adipose_R2_peak_2	Distal Interger	1	1.90E+08	1.90E+08	48904	2	19130	uc033fol.1	-131605	ENSMUSG000	Prox1	prospero homeobox 1
chr1	190302549	190302688	140	141	190302616	31	9.62237	3.47996	6.49386	Adipose_R1_peak_1	Distal Interger	1	1.90E+08	1.90E+08	48904	2	19130	uc033fol.1	-131869	ENSMUSG000	Prox1	prospero homeobox 1
chr1	190302556	190302699	144	145	190302628	36	9.41432	3.12701	6.22695	Adipose_R2_peak_2	Distal Interger	1	1.90E+08	1.90E+08	48904	2	19130	uc033fol.1	-131876	ENSMUSG000	Prox1	prospero homeobox 1
chr1	190303070	190303373	304	305	190303306	26	5.78826	2.63134	2.92378	Adipose_R1_peak_1	Distal Interger	1	1.90E+08	1.90E+08	48904	2	19130	uc033fol.1	-132390	ENSMUSG000	Prox1	prospero homeobox 1
chr1	190303214	190303324	111	112	190303274	36	9.71152	3.2012	6.51365	Adipose_R2_peak_2	Distal Interger	1	1.90E+08	1.90E+08	48904	2	19130	uc033fol.1	-132534	ENSMUSG000	Prox1	prospero homeobox 1
chr1	190756210	190756373	164	165	190756272	20	7.74501	3.7745	4.70467	Adipose_R1_peak_1	Distal Interger	1	1.91E+08	1.91E+08	61220	2	320119	uc007ebf.1	77725	ENSMUSG000	Rps6kc1	ribosomal protein S6 kinase polypeptide 1
chr1	190902733	190903180	448	449	190902850	91	54.95	8.48403	50.74234	Adipose_R2_peak_2	Intron (uc007e	1	1.91E+08	1.91E+08	138892	2	320119	uc007ebi.2	8590	ENSMUSG000	Rps6kc1	ribosomal protein S6 kinase polypeptide 1
chr1	191147568	191147871	304	305	191147723	30	11.08307	4.01538	7.88242	Adipose_R1_peak_1	Distal Interger	1	1.91E+08	1.91E+08	2234	2	619288	uc011wyr.1	16946	ENSMUSG000	Fam71a	family with sequence similarity 71, member A
chr1	191147597	191147877	281	282	191147781	37	11.12817	3.50427	7.83613	Adipose_R2_peak_2	Distal Interger	1	1.91E+08	1.91E+08	2234	2	619288	uc011wyr.1	16940	ENSMUSG000	Fam71a	family with sequence similarity 71, member A
chr1	191387496	191387824	329	330	191387586	31	16.23981	5.75161	12.83549	Adipose_R1_peak_1	Intron (uc007e	1	1.91E+08	1.91E+08	45061	2	226849	uc007eci.1	9217	ENSMUSG000	Ppp2r5a	protein phosphatase 2, regulatory subunit B', alpha
chr1	191387529	191387751	223	224	191387614	43	14.8939	4.05758	11.42017	Adipose_R2_peak_2	Intron (uc007e	1	1.91E+08	1.91E+08	45061	2	226849	uc007eci.1	9290	ENSMUSG000	Ppp2r5a	protein phosphatase 2, regulatory subunit B', alpha
chr1	191401953	191402114	162	163	191402046	19	7.07687	3.59476	4.08458	Adipose_R1_peak_1	Distal Interger	1	1.91E+08	1.91E+08	45061	2	226849	uc007eci.1	-4912	ENSMUSG000	Ppp2r5a	protein phosphatase 2, regulatory subunit B', alpha
chr1	191417444	191417584	141	142	191417501	16	5.20017	3.05554	2.38734	Adipose_R1_peak_1	Distal Interger	1	1.91E+08	1.91E+08	45061	2	226849	uc007eci.1	-20403	ENSMUSG000	Ppp2r5a	protein phosphatase 2, regulatory subunit B', alpha
chr1	191653666	191653928	263	264	191653796	53	37.70483	9.70585	33.87	Adipose_R1_peak_1	Distal Interger	1	1.92E+08	1.92E+08	66234	1	226856	uc007ecs.3	-64096	ENSMUSG000	Lpgat1	lysophosphatidylglycerol acyltransferase 1
chr1	191653727	191653919	193	194	191653801	41	13.59774	3.87315	10.1803	Adipose_R2_peak_2	Distal Interger	1	1.92E+08	1.92E+08	66234	1	226856	uc007ecs.3	-64105	ENSMUSG000	Lpgat1	lysophosphatidylglycerol acyltransferase 1
chr1	193470616	193470849	234	235	193470730	27	12.93148	5.03266	9.63827	Adipose_R1_peak_1	Distal Interger	1	1.94E+08	1.94E+08	68	2	387201	uc011wzc.1	36681	ENSMUSG000	Mir205	microRNA 205
chr1	193470669	193470799	131	132	193470739	32	8.29282	3.04319	5.18352	Adipose_R2_peak_2	Distal Interger	1	1.94E+08	1.94E+08	68	2	387201	uc011wzc.1	36731	ENSMUSG000	Mir205	microRNA 205
chr1	193804176	193804672	497	498	193804297	38	21.66112	6.68341	18.11576	Adipose_R1_peak_1	Distal Interger	1	1.94E+08	1.94E+08	68	2	387201	uc011wzc.1	-296646	ENSMUSG000	Mir205	microRNA 205
chr1	193804189	193804657	469	470	193804304	39	12.34187	3.68871	8.98501	Adipose_R2_peak_2	Distal Interger	1	1.94E+08	1.94E+08	68	2	387201	uc011wzc.1	-296659	ENSMUSG000	Mir205	microRNA 205
chr1	194082802	194083072	271	272	194082945	47	31.38649	8.62742	27.65048	Adipose_R1_peak_1	Distal Interger	1	1.94E+08	1.94E+08	10573	2	74942	uc029qvi.1	139687	ENSMUSG000	4930503O07R	RIKEN cDNA 4930503O07 gene
chr1	194082836	194083058	223	224	194082925	44	15.55653	4.1498	12.05625	Adipose_R2_peak_2	Distal Interger	1	1.94E+08	1.94E+08	10573	2	74942	uc029qvi.1	139701	ENSMUSG000	4930503O07R	RIKEN cDNA 4930503O07 gene
chr1	195052094	195052317	224	225	195052202	37	21.59274	6.83004	18.04766	Adipose_R1_peak_1	Intron (uc007e	1	1.95E+08	1.95E+08	88	1	387224	uc011wzg.1	14547	ENSMUSG000	Mir29c	microRNA 29c
chr1	195052132	195052306	175	176	195052199	36	10.5378	3.41206	7.27949	Adipose_R2_peak_2	Intron (uc007e	1	1.95E+08	1.95E+08	88	1	387224	uc011wzg.1	14585	ENSMUSG000	Mir29c	microRNA 29c
chr1	195241694	195241893	200	201	195241748	78	23.05506	3.80702	19.48666	Adipose_R1_peak_1	Distal Interger	1	1.95E+08	1.95E+08	39905	2	12902	uc007eez.1	-64979	ENSMUSG000	Cr2	complement receptor 2
chr1	195324151	195324283	133	134	195324221	21	7.74089	3.66712	4.70467	Adipose_R1_peak_1	Distal Interger	1	1.95E+08	1.95E+08	39905	2	12902	uc007eez.1	-147436	ENSMUSG000	Cr2	complement receptor 2
chr1	195371656	195371802	147	148	195371787	17	5.80341	3.23528	2.92378	Adipose_R1_peak_1	Distal Interger	1	1.95E+08	1.95E+08	39905	2	12902	uc007eez.1	-194941	ENSMUSG000	Cr2	complement receptor 2
chr10	3109485	3109756	272	273	3109619	339	70.03564	3.11784	65.69584	Adipose_R2_peak_2	Distal Interger	10	3125091	3133193	8103	2	619314	uc029qvj.1	23437	NA	B020014A21R	RIKEN cDNA B020014A21 gene
chr10	3109518	3109730	213	214	3109621	227	32.08158	2.44852	28.34244	Adipose_R1_peak_1	Distal Interger	10	3125091	3133193	8103	2	619314	uc029qvj.1	23463	NA	B020014A21R	RIKEN cDNA B020014A21 gene
chr10	3110035	3110379	345	346	3110207	123	14.42363	2.17232	10.97348	Adipose_R2_peak_2	Distal Interger	10	3125091	3133193	8103	2	619314	uc029qvj.1	22814	NA	B020014A21R	RIKEN cDNA B020014A21 gene
chr10	3110062	3110375	314	315	3110238	84	20.59765	3.30092	17.09339	Adipose_R1_peak_1	Distal Interger	10	3125091	3133193	8103	2	619314	uc029qvj.1	22818	NA	B020014A21R	RIKEN cDNA B020014A21 gene
chr10	3111399	3116286	4888	4889	3111604	173	64.57305	5.01679	60.45304	Adipose_R1_peak_1	Distal Interger	10	3125091	3133193	8103	2	619314	uc029qvj.1	16907	NA	B020014A21R	RIKEN cDNA B020014A21 gene
chr10	3111515	3111926	412	413	3111609	215	79.93205	5.04963	75.52167	Adipose_R2_peak_2	Distal Interger	10	3125091	3133193	8103	2	619314	uc029qvj.1	21267	NA	B020014A21R	RIKEN cDNA B020014A21 gene
chr10	3112197	3112638	442	443	3112242	73	12.7179	2.59188	9.35063	Adipose_R2_peak_2	Distal Interger	10	3125091	3133193	8103	2	619314	uc029qvj.1	20555	NA	B020014A21R	RIKEN cDNA B020014A21 gene
chr10	3112771	3113269	499	500	3112912	100	15.57476	2.48601	12.07428	Adipose_R2_peak_2	Distal Interger	10	3125091	3133193	8103	2	619314	uc029qvj.1	19924	NA	B020014A21R	RIKEN cDNA B020014A21 gene
chr10	3113421	3114359	939	940	3113606	79	10.70293	2.26759	7.43949	Adipose_R2_peak_2	Distal Interger	10	3125091	3133193	8103	2	619314	uc029qvj.1	18834	NA	B020014A21R	RIKEN cDNA B020014A21 gene
chr10	3114519	3114650	132	133	3114583	91	23.89141	3.50195	20.1434	Adipose_R2_peak_2	Distal Interger	10	3125091	3133193	8103	2	619314	uc029qvj.1	18543	NA	B020014A21R	RIKEN cDNA B020014A21 gene
chr10	3115179	3115659	481	482	3115450	184	121.9687	10.49849	117.3846	Adipose_R2_peak_2	Distal Interger	10	3125091	3133193	8103	2	619314	uc029qvj.1	17534	NA	B020014A21R	RIKEN cDNA B020014A21 gene
chr10	3116097	3116242	146	147	3116148	64	16.88996	3.40319	13.35114	Adipose_R2_peak_2	Distal Interger	10	3125091	3133193	8103	2	619314	uc029qvj.1	16951	NA	B020014A21R	RIKEN cDNA B020014A21 gene
chr10	3116712	3117100	389	390	3116957	70	28.18107	5.03089	24.51249	Adipose_R1_peak_1	Distal Interger	10	3125091	3133193	8103	2	619314	uc029qvj.1	16093	NA	B020014A21R	RIKEN cDNA B020014A21 gene
chr10	3116756	3117045	290	291	3116948	101	46.58397	6.08276	42.46864	Adipose_R2_peak_2	Distal Interger	10	3125091	3133193	8103	2	619314	uc029qvj.1	16148	NA	B020014A21R	RIKEN cDNA B020014A21 gene
chr10	3246825	3247016	192	193	3246871	29	6.74062	2.76653	3.75989	Adipose_R2_peak_2	Distal Interger	10	3256208	3267771	11564	2	670558	uc011wzv.1	20755	ENSMUSG000	H60c	histocompatibility 60c
chr10	3288634	3288897	264	265	3288788	36	20.67077	6.6503	17.14779	Adipose_R1_peak_1	Distal Interger	10	3256208	3267771	11564	2	670558	uc011wzv.1	-20863	ENSMUSG000	H60c	histocompatibility 60c
chr10	3288635	3288906	272	273	3288782	56	24.19188	5.25641	20.43163	Adipose_R2_peak_2	Distal Interger	10	3256208	3267771	11564	2	670558	uc011wzv.1	-20864	ENSMUSG000	H60c	histocompatibility 60c
chr10	3316932	3317071	140	141	3316996	21	8.43286	3.95423	5.34896	Adipose_R1_peak_1	Distal Interger	10	3366150	3464975	98826	1	76142	uc007ehq.1	-49079	ENSMUSG000	Ppp1r14c	protein phosphatase 1, regulatory (inhibitor) subunit 14c
chr10	3400302	3400454	153	154	3400426	30	7.24477	2.85875	4.21917	Adipose_R2_peak_2	Intron (uc007e	10	3366150	3464975	98826	1	76142	uc007ehq.1	34152	ENSMUSG000	Ppp1r14c	protein phosphatase 1, regulatory (inhibitor) subunit 14c
chr10	3506219	3506356	138	139	3506228	18	6.42934	3.41502	3.48999	Adipose_R1_peak_1	Distal Interger	10	3540279	3554877	14599	1	70337	uc007ehp.1	-33923	ENSMUSG000	Iyd	iodotyrosine deiodinase
chr10	3580344	3580594	251	252	3580459	42	26.36523	7.72873	22.71906	Adipose_R1_peak_1	Distal Interger	10	3540279	3554877	14599	1	70337	uc007ehp.1	40065	ENSMUSG000	Iyd	iodotyrosine deiodinase
chr10	3580364	3580514	151	152	3580465	29	6.74062	2.76653	3.75989	Adipose_R2_peak_2	Distal Interger	10	3540279	3554877	14599	1	70337	uc007ehp.1	40085	ENSMUSG000	Iyd	iodotyrosine deiodinase
chr10	3987030	3987230	201	202	3987146	29	14.55748	5.39214	11.20652	Adipose_R1_peak_1	Intron (uc007e	10	3973075	4156130	183056	1	270685	uc007ehj.2	13955	ENSMUSG000	Mthfd1l	methylenetetrahydrofolate dehydrogenase (NADP+ dependent) 1-like
chr10	4055268	4055522	255	256	4055448	38	11.72962	3.59649	8.4047	Adipose_R2_peak_2	Intron (uc007e	10	3973075	4156130	183056	1	270685	uc007ehj.2	82193	ENSMUSG000	Mthfd1l	methylenetetrahydrofolate dehydrogenase (NADP+ dependent) 1-like
chr10	4194487	4194683	197	198	4194573	24	10.60668	4.49345	7.40961	Adipose_R1_peak_1	Distal Interger	10	4266329	4359471	93143	1	83397	uc007ehi.2	-71646	ENSMUSG000	Akap12	A kinase (PRKA) anchor protein (gravin) 12
chr10	4313987	4314168	182	183	4314070	19	7.07687	3.59476	4.08458	Adipose_R1_peak_1	Intron (uc007e	10	4347768	4359471	11704	1	83397	uc007ehh.1	-33600	ENSMUSG000	Akap12	A kinase (PRKA) anchor protein (gravin) 12
chr10	4342939	4343177	239	240	4343068	32	17.10103	5.93135	13.67137	Adipose_R1_peak_1	Intron (uc007e	10	4347768	4359471	11704	1	83397	uc007ehh.1	-4591	ENSMUSG000	Akap12	A kinase (PRKA) anchor protein (gravin) 12
chr10	4343019	4343146	128	129	4343091	30	7.24477	2.85875	4.21917	Adipose_R2_peak_2	Intron (uc007e	10	4347768	4359471	11704	1	83397	uc007ehh.1	-4622	ENSMUSG000	Akap12	A kinase (PRKA) anchor protein (gravin) 12
chr10	4385927	4386108	182	183	4385964	26	5.31252	2.48988	2.47999	Adipose_R2_peak_2	Promoter (1-2	10	4367074	4387462	20389	2	381990	uc007ehg.2	1354	ENSMUSG000	Zbtb2	zinc finger and BTB domain containing 2
chr10	4385944	4386126	183	184	4386057	24	10.60668	4.49345	7.40961	Adipose_R1_peak_1	Promoter (1-2	10	4367074	4387462	20389	2	381990	uc007ehg.2	1336	ENSMUSG000	Zbtb2	zinc finger and BTB domain containing 2
chr10	4501516	4501704	189	190	4501598	37	11.12817	3.50427	7.83613	Adipose_R2_peak_2	Distal Interger	10	4509872	4561111	51240	1	1.01E+08	uc011wzs.1	-8168	ENSMUSG000	Ccdc170	coiled-coil domain containing 170
chr10	4501517	4501709	193	194	4501609	30	15.39182	5.57188	12.01394	Adipose_R1_peak_1	Distal Interger	10	4509872	4561111	51240	1	1.01E+08	uc011wzs.1	-8163	ENSMUSG000	Ccdc170	coiled-coil domain containing 170
chr10	4730218	4730474	257	258	4730380	54	25.94448	5.87644	22.31506	Adipose_R1_peak_1	Intron (uc007e	10	4712313	4893083	180771	1	13982	uc011wzq.2	17905	ENSMUSG000	Esr1	estrogen receptor 1 (alpha)
chr10	4730223	4730439	217	218	4730376	40	12.96465	3.78093	9.57654	Adipose_R2_peak_2	Intron (uc007e	10	4712313	4893083	180771	1	13982	uc011wzq.2	17910	ENSMUSG000	Esr1	estrogen receptor 1 (alpha)

chr10	4831786	4832085	300	301	4831952	76	40.9047	7.10077	36.85923	Adipose_R2_peak_2	Intron (uc007e	10	4936523	5005633	69111	1	13982	uc011wzo.2	-104438	ENSMUSG000	Esr1	estrogen receptor 1 (alpha)
chr10	4831800	4832088	289	290	4831951	61	46.56983	11.14375	42.62347	Adipose_R1_peak_1	Intron (uc007e	10	4936523	5005633	69111	1	13982	uc011wzo.2	-104435	ENSMUSG000	Esr1	estrogen receptor 1 (alpha)
chr10	4916504	4916734	231	232	4916604	33	17.97508	6.11109	14.52164	Adipose_R1_peak_1	Intron (uc007e	10	4936523	5005633	69111	1	13982	uc011wzo.2	-19789	ENSMUSG000	Esr1	estrogen receptor 1 (alpha)
chr10	4947003	4947168	166	167	4947074	18	6.42934	3.41502	3.48999	Adipose_R1_peak_1	Intron (uc007e	10	4936523	5005633	69111	1	13982	uc011wzo.2	10480	ENSMUSG000	Esr1	estrogen receptor 1 (alpha)
chr10	5283533	5283684	152	153	5283596	16	5.20017	3.05554	2.38734	Adipose_R1_peak_1	Distal Interger	10	5324385	5367659	43275	2	64009	uc007egp.1	83975	ENSMUSG000	Syne1	spectrin repeat containing, nuclear envelope 1
chr10	5390087	5390294	208	209	5390184	33	17.97508	6.11109	14.52164	Adipose_R1_peak_1	Intron (uc007e	10	5336949	5368486	31538	2	64009	uc007ego.1	-21601	ENSMUSG000	Syne1	spectrin repeat containing, nuclear envelope 1
chr10	5390145	5390282	138	139	5390189	34	9.39139	3.22762	6.20474	Adipose_R2_peak_2	Intron (uc007e	10	5336949	5368486	31538	2	64009	uc007ego.1	-21659	ENSMUSG000	Syne1	spectrin repeat containing, nuclear envelope 1
chr10	5395204	5395387	184	185	5395323	31	16.23981	5.75161	12.83549	Adipose_R1_peak_1	Intron (uc007e	10	5336949	5368486	31538	2	64009	uc007ego.1	-26718	ENSMUSG000	Syne1	spectrin repeat containing, nuclear envelope 1
chr10	5395223	5395382	160	161	5395332	32	8.29282	3.04319	5.18352	Adipose_R2_peak_2	Intron (uc007e	10	5336949	5368486	31538	2	64009	uc007ego.1	-26737	ENSMUSG000	Syne1	spectrin repeat containing, nuclear envelope 1
chr10	5458957	5459148	192	193	5459062	24	10.60668	4.49345	7.40961	Adipose_R1_peak_1	Intron (uc007e	10	5336949	5368486	31538	2	64009	uc007ego.1	-90471	ENSMUSG000	Syne1	spectrin repeat containing, nuclear envelope 1
chr10	5479994	5480163	170	171	5480096	22	9.13959	4.13397	6.01526	Adipose_R1_peak_1	Intron (uc007e	10	5336949	5550692	213744	2	64009	uc007egn.1	70529	ENSMUSG000	Syne1	spectrin repeat containing, nuclear envelope 1
chr10	5537995	5538156	162	163	5538064	18	6.42934	3.41502	3.48999	Adipose_R1_peak_1	Intron (uc007e	10	5336949	5550692	213744	2	64009	uc007egn.1	12536	ENSMUSG000	Syne1	spectrin repeat containing, nuclear envelope 1
chr10	6274817	6274951	135	136	6274871	27	5.77405	2.5821	2.88921	Adipose_R2_peak_2	Distal Interger	10	5825664	5922400	96737	2	56533	uc007egg.2	-352417	ENSMUSG000	Rgs17	regulator of G-protein signaling 17
chr10	6281491	6281664	174	175	6281588	25	11.36566	4.67319	8.13482	Adipose_R1_peak_1	Distal Interger	10	5825664	5922400	96737	2	56533	uc007egg.2	-359091	ENSMUSG000	Rgs17	regulator of G-protein signaling 17
chr10	6282448	6282711	264	265	6282593	54	38.78658	9.88558	34.93639	Adipose_R1_peak_1	Distal Interger	10	5825664	5922400	96737	2	56533	uc007egg.2	-360048	ENSMUSG000	Rgs17	regulator of G-protein signaling 17
chr10	6282499	6282660	162	163	6282594	39	12.34187	3.68871	8.98501	Adipose_R2_peak_2	Distal Interger	10	5825664	5922400	96737	2	56533	uc007egg.2	-360099	ENSMUSG000	Rgs17	regulator of G-protein signaling 17
chr10	6623913	6624217	305	306	6624045	81	70.57077	14.73851	66.40193	Adipose_R1_peak_1	Distal Interger	10	6758593	6850107	91515	1	18390	uc007efy.1	-134376	ENSMUSG000	Oprm1	opioid receptor, mu 1
chr10	6623924	6624224	301	302	6624052	71	36.49133	6.63968	32.5091	Adipose_R2_peak_2	Distal Interger	10	6758593	6850107	91515	1	18390	uc007efy.1	-134369	ENSMUSG000	Oprm1	opioid receptor, mu 1
chr10	6630586	6630732	147	148	6630664	20	7.74501	3.7745	4.70467	Adipose_R1_peak_1	Distal Interger	10	6758593	6850107	91515	1	18390	uc007efy.1	-127861	ENSMUSG000	Oprm1	opioid receptor, mu 1
chr10	6642853	6643135	283	284	6642968	57	42.07807	10.4248	38.18469	Adipose_R1_peak_1	Distal Interger	10	6758593	6850107	91515	1	18390	uc007efy.1	-115458	ENSMUSG000	Oprm1	opioid receptor, mu 1
chr10	6642874	6643071	198	199	6642958	36	10.5378	3.41206	7.27949	Adipose_R2_peak_2	Distal Interger	10	6758593	6850107	91515	1	18390	uc007efy.1	-115522	ENSMUSG000	Oprm1	opioid receptor, mu 1
chr10	6660481	6660664	184	185	6660547	24	10.60668	4.49345	7.40961	Adipose_R1_peak_1	Distal Interger	10	6758593	6850107	91515	1	18390	uc007efy.1	-97929	ENSMUSG000	Oprm1	opioid receptor, mu 1
chr10	6741086	6741181	96	97	6741131	31	7.7623	2.95097	4.69388	Adipose_R2_peak_2	Distal Interger	10	6758593	6850107	91515	1	18390	uc007efy.1	-17412	ENSMUSG000	Oprm1	opioid receptor, mu 1
chr10	6741106	6741241	136	137	6741174	32	15.39232	5.28413	12.01435	Adipose_R1_peak_1	Distal Interger	10	6758593	6850107	91515	1	18390	uc007efy.1	-17352	ENSMUSG000	Oprm1	opioid receptor, mu 1
chr10	7400237	7400614	378	379	7400377	32	17.10103	5.93135	13.67137	Adipose_R1_peak_1	Distal Interger	10	7444873	7473477	28605	2	77777	uc029qvm.1	72863	ENSMUSG000	Ulbp1	UL16 binding protein 1
chr10	7400362	7400618	257	258	7400492	47	17.60017	4.42645	14.02531	Adipose_R2_peak_2	Distal Interger	10	7444873	7473477	28605	2	77777	uc029qvm.1	72859	ENSMUSG000	Ulbp1	UL16 binding protein 1
chr10	8135340	8135744	405	406	8135589	20	7.74501	3.7745	4.70467	Adipose_R1_peak_1	Distal Interger	10	8204753	8302242	97490	2	338362	uc011xaa.1	166498	ENSMUSG000	Ust	uronyl-2-sulfotransferase
chr10	8150063	8150307	245	246	8150207	35	19.20331	6.25718	15.72474	Adipose_R1_peak_1	Distal Interger	10	8204753	8302242	97490	2	338362	uc011xaa.1	151935	ENSMUSG000	Ust	uronyl-2-sulfotransferase
chr10	8151762	8151960	199	200	8151867	24	10.60668	4.49345	7.40961	Adipose_R1_peak_1	Distal Interger	10	8204753	8302242	97490	2	338362	uc011xaa.1	150282	ENSMUSG000	Ust	uronyl-2-sulfotransferase
chr10	8316363	8316541	179	180	8316464	24	10.60668	4.49345	7.40961	Adipose_R1_peak_1	Intron (uc007e	10	8204753	8302242	97490	2	338362	uc011xaa.1	-14121	ENSMUSG000	Ust	uronyl-2-sulfotransferase
chr10	8318676	8318865	190	191	8318755	30	15.39182	5.57188	12.01394	Adipose_R1_peak_1	Intron (uc007e	10	8204753	8302242	97490	2	338362	uc011xaa.1	-16434	ENSMUSG000	Ust	uronyl-2-sulfotransferase
chr10	8454138	8454593	456	457	8454336	31	15.15541	5.334	11.80103	Adipose_R1_peak_1	Intron (uc007e	10	8204753	8518825	314073	2	338362	uc007eit.1	64232	ENSMUSG000	Ust	uronyl-2-sulfotransferase
chr10	8454188	8454591	404	405	8454325	36	10.5378	3.41206	7.27949	Adipose_R2_peak_2	Intron (uc007e	10	8204753	8518825	314073	2	338362	uc007eit.1	64234	ENSMUSG000	Ust	uronyl-2-sulfotransferase
chr10	9709061	9709163	103	104	9709145	27	5.77405	2.5821	2.88921	Adipose_R2_peak_2	Distal Interger	10	9684722	9691909	7188	1	620417	uc007eix.2	24339	NA	Gm6150	predicted gene 6150
chr10	9711570	9711732	163	164	9711634	24	10.60668	4.49345	7.40961	Adipose_R1_peak_1	Distal Interger	10	9684722	9691909	7188	1	620417	uc007eix.2	26848	NA	Gm6150	predicted gene 6150
chr10	10567591	10567798	208	209	10567702	23	9.86443	4.31371	6.70247	Adipose_R1_peak_1	Distal Interger	10	10545039	10558207	13169	2	67844	uc007ejg.1	-9384	ENSMUSG000	Rab32	RAB32, member RAS oncogene family
chr10	10567619	10567743	125	126	10567672	30	7.24477	2.85875	4.21917	Adipose_R2_peak_2	Distal Interger	10	10545039	10558207	13169	2	67844	uc007ejg.1	-9412	ENSMUSG000	Rab32	RAB32, member RAS oncogene family
chr10	10571954	10572121	168	169	10572038	22	9.13959	4.13397	6.01526	Adipose_R1_peak_1	Distal Interger	10	10545039	10558207	13169	2	67844	uc007ejg.1	-13747	ENSMUSG000	Rab32	RAB32, member RAS oncogene family
chr10	10585925	10586220	296	297	10586070	78	66.82238	14.19929	62.68234	Adipose_R1_peak_1	Distal Interger	10	10605945	10616313	10369	1	382450	uc011xac.1	-19725	ENSMUSG000	Gm5177	glyceraldehyde-3-phosphate dehydrogenase pseudogene
chr10	10585990	10586198	209	210	10586080	43	14.8939	4.05758	11.42017	Adipose_R2_peak_2	Distal Interger	10	10605945	10616313	10369	1	382450	uc011xac.1	-19747	ENSMUSG000	Gm5177	glyceraldehyde-3-phosphate dehydrogenase pseudogene
chr10	10667807	10668086	280	281	10667950	46	30.36364	8.44768	26.64488	Adipose_R1_peak_1	Distal Interger	10	10605945	10616313	10369	1	382450	uc011xac.1	61862	ENSMUSG000	Gm5177	glyceraldehyde-3-phosphate dehydrogenase pseudogene
chr10	10667898	10668025	128	129	10667956	31	7.7623	2.95097	4.69388	Adipose_R2_peak_2	Distal Interger	10	10605945	10616313	10369	1	382450	uc011xac.1	61953	ENSMUSG000	Gm5177	glyceraldehyde-3-phosphate dehydrogenase pseudogene
chr10	10672567	10672775	209	210	10672674	26	12.14079	4.85292	8.87838	Adipose_R1_peak_1	Distal Interger	10	10605945	10616313	10369	1	382450	uc011xac.1	66622	ENSMUSG000	Gm5177	glyceraldehyde-3-phosphate dehydrogenase pseudogene
chr10	11337892	11338078	187	188	11338008	20	7.74501	3.7745	4.70467	Adipose_R1_peak_1	Distal Interger	10	11343445	11457477	114033	1	13853	uc007ejv.1	-5367	ENSMUSG000	Epm2a	epilepsy, progressive myoclonic epilepsy, type 2 gene alpha
chr10	11352691	11352897	207	208	11352770	32	14.31418	4.89843	10.98818	Adipose_R1_peak_1	Intron (uc007e	10	11343445	11457477	114033	1	13853	uc007ejv.1	9246	ENSMUSG000	Epm2a	epilepsy, progressive myoclonic epilepsy, type 2 gene alpha
chr10	11352712	11352838	127	128	11352787	33	8.83596	3.1354	5.68652	Adipose_R2_peak_2	Intron (uc007e	10	11343445	11457477	114033	1	13853	uc007ejv.1	9267	ENSMUSG000	Epm2a	epilepsy, progressive myoclonic epilepsy, type 2 gene alpha
chr10	11373439	11373605	167	168	11373525	21	8.43286	3.95423	5.34896	Adipose_R1_peak_1	Intron (uc007e	10	11343445	11457477	114033	1	13853	uc007ejv.1	29994	ENSMUSG000	Epm2a	epilepsy, progressive myoclonic epilepsy, type 2 gene alpha
chr10	11373963	11374163	201	202	11374098	44	15.55653	4.1498	12.05625	Adipose_R2_peak_2	Intron (uc007e	10	11343445	11457477	114033	1	13853	uc007ejv.1	30518	ENSMUSG000	Epm2a	epilepsy, progressive myoclonic epilepsy, type 2 gene alpha
chr10	11373999	11374163	165	166	11374153	26	12.14079	4.85292	8.87838	Adipose_R1_peak_1	Intron (uc007e	10	11343445	11457477	114033	1	13853	uc007ejv.1	30554	ENSMUSG000	Epm2a	epilepsy, progressive myoclonic epilepsy, type 2 gene alpha
chr10	11492089	11492265	177	178	11492175	63	29.74474	5.90194	25.87429	Adipose_R2_peak_2	Distal Interger	10	11609224	11610438	1215	1	30058	uc057awg.1	-116959	ENSMUSG000	Timm8a1	translocase of inner mitochondrial membrane 8A1
chr10	11492109	11492244	136	137	11492176	45	15.85417	4.15145	12.47378	Adipose_R1_peak_1	Distal Interger	10	11609224	11610438	1215	1	30058	uc057awg.1	-116980	ENSMUSG000	Timm8a1	translocase of inner mitochondrial membrane 8A1
chr10	11852269	11852471	203	204	11852374	24	10.60668	4.49345	7.40961	Adipose_R1_peak_1	Distal Interger	10	11609224	11610438	1215	1	30058	uc057awg.1	243045	ENSMUSG000	Timm8a1	translocase of inner mitochondrial membrane 8A1
chr10	11902413	11902617	205	206	11902509	31	16.23981	5.75161	12.83549	Adipose_R1_peak_1	Distal Interger	10	11609224	11610438	1215	1	30058	uc057awg.1	293189	ENSMUSG000	Timm8a1	translocase of inner mitochondrial membrane 8A1
chr10	11902436	11902572	137	138	11902499	29	6.74062	2.76653	3.75989	Adipose_R2_peak_2	Distal Interger	10	11609224	11610438	1215	1	30058	uc057awg.1	293212	ENSMUSG000	Timm8a1	translocase of inner mitochondrial membrane 8A1
chr10	12073319	12073589	271	272	12073452	69	55.87963	12.58165	51.83661	Adipose_R1_peak_1	Distal Interger	10	11609224	11610438	1215	1	30058	uc057awg.1	464095	ENSMUSG000	Timm8a1	translocase of inner mitochondrial membrane 8A1
chr10	12073344	12073527	184	185	12073456	41	13.59774	3.87315	10.1803	Adipose_R2_peak_2	Distal Interger	10	11609224	11610438	1215	1	30058	uc057awg.1	464120	ENSMUSG000	Timm8a1	translocase of inner mitochondrial membrane 8A1
chr10	12356062	12356266	205	206	12356142	23	9.86443	4.31371	6.70247	Adipose_R1_peak_1	Distal Interger	10	12385518	12614891	229374	2	22288	uc007ejx.1	258625	ENSMUSG000	Utrn	utrophin
chr10	12358379	12358538	160	161	12358441	20	7.74501	3.7745	4.70467	Adipose_R1_peak_1	Distal Interger	10	12385518	12614891	229374	2	22288	uc007ejx.1	256353	ENSMUSG000	Utrn	utrophin
chr10	12391901	12392123	223	224	12392005	47	17.60017	4.42645	14.02531	Adipose_R2_peak_2	Intron (uc007e	10	12385518	12614891	229374	2	22288	uc007ejx.1	222768	ENSMUSG000	Utrn	utrophin
chr10	12391951	12392118	168	169	12392020	20	7.74501	3.7745	4.70467	Adipose_R1_peak_1	Intron (uc007e	10	12385518	12614891	229374	2	22288	uc007ejx.1	222773	ENSMUSG000	Utrn	utrophin
chr10	13896781	13897022	242	243	13896901	30	15.39182	5.57188	12.01394	Adipose_R1_peak_1	Distal Interger	10	13647055	13868830	221776	2	66253	uc007elb.1	-27951	ENSMUSG000	Aig1	androgen-induced 1
chr10	13896860	13896970	111	112	13896905	28	6.25023	2.67431	3.31626	Adipose_R2_peak_2	Distal Interger	10	13647055	13868830	221776	2	66253	uc007elb.1	-28030	ENSMUSG000	Aig1	androgen-induced 1
chr10	14292569	14292728	160	161	14292643	20	7.74501	3.7745	4.70467	Adipose_R1_peak_1	Distal Interger	10	14402585	14545036	142452	2	215798	uc007elk.1	252308	ENSMUSG000	Adgrg6	adhesion G protein-coupled receptor G6
chr10	14395119	14395365	247	248	14395197	41	13.59774	3.87315	10.1803	Adipose_R2_peak_2	Distal Interger	10	14402585	14545036	142452	2	215798	uc007elk.1	149671	ENSMUSG000	Adgrg6	adhesion G protein-coupled receptor G6
chr10	14395122	14395480	359	360	14395253	39	14.02653	4.12915	10.70233	Adipose_R1_peak_1	Distal Interger	10	14402585	14545036	142452	2	215798	uc007elk.1	149556	ENSMUSG000	Adgrg6	adhesion G protein-coupled receptor G6
chr10	14485392	14485732	341	342	14485532	51	35.56531	9.34637	31.76235	Adipose_R1_peak_1	Intron (uc007e	10	14402585	14545036	142452	2	215798	uc007elk.1	59304	ENSMUSG000	Adgrg6	adhesion G protein-coupled receptor G6
chr10	14499501	14499719	219	220	14499598	29	14.55748	5.39214	11.20652	Adipose_R1_peak_1	Intron (uc007e	10	14402585	14545036	142452	2	215798	uc007elk.1	45317	ENSMUSG000	Adgrg6	adhesion G protein-coupled receptor G6
chr10	14499531	14499674	144	145	14499602	38	11.72962	3.59649	8.4047	Adipose_R2_peak_2	Intron (uc007e	10	14402585	14545036	142452	2	215798	uc007elk.1	45362	ENSMUSG000	Adgrg6	adhesion G protein-coupled receptor G6
chr10	14866047	14866470	424	425	14866112	23	9.86443	4.31371	6.70247	Adipose_R1_peak_1	Distal Interger	10	14760289	14770556	10268	1	18101	uc007elp.1	105758	ENSMUSG000	Nmbr	neuromedin B receptor
chr10	14866069	14866258	190	191	14866124	31	7.7623	2.95097	4.69388	Adipose_R2_peak_2	Distal Interger	10	14760289	14770556	10268	1	18101	uc007elp.1	105780	ENSMUSG000	Nmbr	neuromedin B receptor
chr10	14924316	14924503	188	189	14924390	25	11.36566	4.67319	8.13482	Adipose_R1_peak_1	Distal Interger	10	14760289	14770556	10268	1	18101	uc007elp.1	164027	ENSMUSG000	Nmbr	neuromedin B receptor
chr10	14927357	14927631	275	276	14927487	48	32.41826	8.80716	28.66523	Adipose_R1_peak_1	Distal Interger	10	14760289	14770556	10268	1	18101	uc007elp.1	167068	ENSMUSG000	Nmbr	neuromedin B receptor
chr10	14927431	14927532	102	103	14927485	31	7.7623	2.95097	4.69388	Adipose_R2_peak_2	Distal Interger	10	14760289	14770556	10268	1	18101	uc007elp.1	167142	ENSMUSG000	Nmbr	neuromedin B receptor
chr10	15001515	15001700	186	187	15001579	20	7.74501	3.7745	4.70467	Adipose_R1_peak_1	Distal Interger	10	14760289	14770556	10268	1	18101	uc007elp.1	241226	ENSMUSG000	Nmbr	neuromedin B receptor
chr10	15048421	15048655	235	236	15048536	36	20.67077	6.6503	17.14779	Adipose_R1_peak_1	Distal Interger	10	14760289	14770556	10268	1	18101	uc007elp.1	288132	ENSMUSG000	Nmbr	neuromedin B receptor
chr10	15048469	15048632	164	165	15048533	35	9.95877	3.31984	6.73562	Adipose_R2_peak_2	Distal Interger	10	14760289	14770556	10268	1	18101	uc007elp.1	288180	ENSMUSG000	Nmbr	neuromedin B receptor
chr10	15069485	15069721	237	238	15069592	23	9.86443	4.31371	6.70247	Adipose_R1_peak_1	Distal Interger	10	14760289	14770556	10268	1	18101	uc007elp.1	309196	ENSMUSG000	Nmbr	neuromedin B receptor
chr10	15125505	15125814	310	311	15125666	41	25.38991	7.54899	21.76305	Adipose_R1_peak_1	Distal Interger	10	14760289	14770556	10268	1	18101	uc007elp.1	365216	ENSMUSG000	Nmbr	neuromedin B receptor
chr10	15125594	15125748	155	156	15125670	37	11.12817	3.50427	7.83613	Adipose_R2_peak_2	Distal Interger	10	14760289	14770556	10268	1	18101	uc007elp.1	365305	ENSMUSG000	Nmbr	neuromedin B receptor
chr10	15201724	15201876	153	154	15201787	20	7.74501	3.7745	4.70467	Adipose_R1_peak_1	Distal Interger	10	14760289	14770556	10268	1	18101	uc007elp.1	441435	ENSMUSG000	Nmbr	neuromedin B receptor
chr10	15348267	15348479	213	214	15348369	31	16.23981	5.75161	12.83549	Adipose_R1_peak_1	Distal Interger	10	15791777	15807065	15289	1	77538	uc007elr.1	-443298	NA	C330004P14R	RIKEN cDNA C330004P14 gene
chr10	15348330	15348434	105	106	15348375	29	6.74062	2.76653	3.75989	Adipose_R2_peak_2	Distal Interger	10	15791777	15807065	15289	1	77538	uc007elr.1	-443343	NA	C330004P14R	RIKEN cDNA C330004P14 gene
chr10	15349013	15349163	151	152	15349076	18	6.42934	3.41502	3.48999	Adipose_R1_peak_1	Distal Interger	10	15791777	15807065	15289	1	77538	uc007elr.1	-442614	NA	C330004P14R	RIKEN cDNA C330004P14 gene
chr10	15467752	15467999	248	249	15467861	44	28.34544	8.08821	24.66239	Adipose_R1_peak_1	Distal Interger	10	15791777	15807065	15289	1	77538	uc007elr.1	-323778	NA	C330004P14R	RIKEN cDNA C330004P14 gene
chr10	15467779	15467967	189	190	15467859	33	8.83596	3.1354	5.68652	Adipose_R2_peak_2	Distal Interger	10	15791777	15807065	15289	1	77538	uc007elr.1	-323810	NA	C330004P14R	RIKEN cDNA C330004P14 gene
chr10	15468943	15469158	216	217	15469055	34	18.86162	6.29083	15.38482	Adipose_R1_peak_1	Distal Interger	10	15791777	15807065	15289	1	77538	uc007elr.1	-322619	NA	C330004P14R	RIKEN cDNA C330004P14 gene
chr10	15533482	15533716	235	236	15533608	25	11.36566	4.67319	8.13482	Adipose_R1_peak_1	Distal Interger	10	15791777	15807065	15289	1	77538	uc007elr.1	-258061	NA	C330004P14R	RIKEN cDNA C330004P14 gene
chr10	15571834	15571981	148	149	15571903	19	7.07687	3.59476	4.08458	Adipose_R1_peak_1	Distal Interger	10	15791777	15807065	15289	1	77538	uc007elr.1	-219796	NA	C330004P14R	RIKEN cDNA C330004P14 gene
chr10	15577947	15578131	185	186	15578062	40	12.96465	3.78093	9.57654	Adipose_R2_peak_2	Distal Interger	10	15791777	15807065	15289	1	77538	uc007elr.1	-213646	NA	C330004P14R	RIKEN cDNA C330004P14 gene
chr10	15577954	15578192	239	240	15578052	27	12.34148	4.79835	9.07769	Adipose_R1_peak_1	Distal Interger	10	15791777	15807065	15289	1	77538	uc007elr.1	-213585	NA	C330004P14R	RIKEN cDNA C330004P14 gene
chr10	15599486	15599736	251	252	15599621	39	23.47001	7.18952	19.88325	Adipose_R1_peak_1	Distal Interger	10	15791777	15807065	15289	1	77538	uc007elr.1	-192041	NA	C330004P14R	RIKEN cDNA C330004P14 gene
chr10	15778961	15779129	169	170	15779061	32	8.29282	3.04319	5.18352	Adipose_R2_peak_2	Distal Interger	10	15791777	15807065	15289	1	77538	uc007elr.1	-12648	NA	C330004P14R	RIKEN cDNA C330004P14 gene
chr10	16191166	16191333	168	169	16191241	24	10.60668	4.49345	7.40961	Adipose_R1_peak_1	Distal Interger	10	15791777	15807065	15289	1	77538	uc007elr.1	399389	NA	C330004P14R	RIKEN cDNA C330004P14 gene
chr10	16256590	16256771	182	183	16256709	21	8.43286	3.95423	5.34896	Adipose_R1_peak_1	Distal Interger	10	15791777	15807065	15289	1	77538	uc007elr.1	464813	NA	C330004P14R	RIKEN cDNA C330004P14 gene

chr10	16522765	16522920	156	157	16522809	19	7.07687	3.59476	4.08458	Adipose_R1_peak_1	Distal Intergen	10	16951094	17095782	144689	2	1.01E+08	uc007els.2	572862	ENSMUSG000	Gm20125	predicted gene, 20125
chr10	16693162	16693332	171	172	16693236	24	10.60668	4.49345	7.40961	Adipose_R1_peak_1	Distal Intergen	10	16951094	17095782	144689	2	1.01E+08	uc007els.2	402450	ENSMUSG000	Gm20125	predicted gene, 20125
chr10	16697139	16697282	144	145	16697211	22	9.13959	4.13397	6.01526	Adipose_R1_peak_1	Distal Intergen	10	16951094	17095782	144689	2	1.01E+08	uc007els.2	398500	ENSMUSG000	Gm20125	predicted gene, 20125
chr10	16744711	16744934	224	225	16744818	26	12.14079	4.85292	8.87838	Adipose_R1_peak_1	Distal Intergen	10	16951094	17095782	144689	2	1.01E+08	uc007els.2	350848	ENSMUSG000	Gm20125	predicted gene, 20125
chr10	16813223	16813407	185	186	16813278	16	5.20017	3.05554	2.38734	Adipose_R1_peak_1	Distal Intergen	10	16951094	17095782	144689	2	1.01E+08	uc007els.2	282375	ENSMUSG000	Gm20125	predicted gene, 20125
chr10	16945850	16946005	156	157	16945914	19	7.07687	3.59476	4.08458	Adipose_R1_peak_1	Distal Intergen	10	16951094	17095782	144689	2	1.01E+08	uc007els.2	149777	ENSMUSG000	Gm20125	predicted gene, 20125
chr10	17141270	17141452	183	184	17141349	22	9.13959	4.13397	6.01526	Adipose_R1_peak_1	Distal Intergen	10	16951094	17095782	144689	2	1.01E+08	uc007els.2	-45488	ENSMUSG000	Gm20125	predicted gene, 20125
chr10	17310429	17310648	220	221	17310479	31	7.7623	2.95097	4.69388	Adipose_R2_peak_2	Distal Intergen	10	16951094	17095782	144689	2	1.01E+08	uc007els.2	-214647	ENSMUSG000	Gm20125	predicted gene, 20125
chr10	17358909	17359060	152	153	17358951	33	8.83596	3.1354	5.68652	Adipose_R2_peak_2	Distal Intergen	10	16951094	17095782	144689	2	1.01E+08	uc007els.2	-263127	ENSMUSG000	Gm20125	predicted gene, 20125
chr10	17821855	17822062	208	209	17821967	35	19.76029	6.47056	16.26028	Adipose_R1_peak_1	Intron (uc007e	10	17796219	17845663	49445	1	378431	uc007elv.1	25636	ENSMUSG000	Txlnb	taxilin beta
chr10	17821876	17822040	165	166	17821948	31	7.7623	2.95097	4.69388	Adipose_R2_peak_2	Intron (uc007e	10	17796219	17845663	49445	1	378431	uc007elv.1	25657	ENSMUSG000	Txlnb	taxilin beta
chr10	19529429	19529606	178	179	19529528	56	39.27646	9.63277	35.42397	Adipose_R1_peak_1	Distal Intergen	10	19591958	19610225	18268	1	15979	uc007eng.1	-62352	ENSMUSG000	Ifngr1	interferon gamma receptor 1
chr10	19529466	19529601	136	137	19529535	36	10.5378	3.41206	7.27949	Adipose_R2_peak_2	Distal Intergen	10	19591958	19610225	18268	1	15979	uc007eng.1	-62357	ENSMUSG000	Ifngr1	interferon gamma receptor 1
chr10	19776049	19776315	267	268	19776173	47	29.67098	8.00099	25.96867	Adipose_R1_peak_1	Distal Intergen	10	19712587	19755925	43339	1	237313	uc011xax.1	63462	ENSMUSG000	Il20ra	interleukin 20 receptor, alpha
chr10	19776100	19776278	179	180	19776173	39	12.34187	3.68871	8.98501	Adipose_R2_peak_2	Distal Intergen	10	19712587	19755925	43339	1	237313	uc011xax.1	63513	ENSMUSG000	Il20ra	interleukin 20 receptor, alpha
chr10	19777259	19777501	243	244	19777351	30	15.39182	5.57188	12.01394	Adipose_R1_peak_1	Distal Intergen	10	19712587	19755925	43339	1	237313	uc011xax.1	64672	ENSMUSG000	Il20ra	interleukin 20 receptor, alpha
chr10	19783052	19783346	295	296	19783195	48	32.41826	8.80716	28.66523	Adipose_R1_peak_1	Distal Intergen	10	19847917	19851459	3543	2	76157	uc007enk.1	68113	ENSMUSG000	Slc35d3	solute carrier family 35, member D3
chr10	19783100	19783352	253	254	19783197	48	18.29934	4.51867	14.70076	Adipose_R2_peak_2	Distal Intergen	10	19847917	19851459	3543	2	76157	uc007enk.1	68107	ENSMUSG000	Slc35d3	solute carrier family 35, member D3
chr10	19996740	19996948	209	210	19996856	36	20.67077	6.6503	17.14779	Adipose_R1_peak_1	Intron (uc007e	10	19934526	20142750	208225	1	26408	uc007enm.2	62214	ENSMUSG000	Map3k5	mitogen-activated protein kinase kinase kinase 5
chr10	19996785	19996907	123	124	19996847	30	7.24477	2.85875	4.21917	Adipose_R2_peak_2	Intron (uc007e	10	19934526	20142750	208225	1	26408	uc007enm.2	62259	ENSMUSG000	Map3k5	mitogen-activated protein kinase kinase kinase 5
chr10	21130539	21130696	158	159	21130660	18	6.42934	3.41502	3.48999	Adipose_R1_peak_1	Intron (uc011x	10	21124930	21143092	18163	2	17863	uc011xbd.1	12396	ENSMUSG000	Myb	myeloblastosis oncogene
chr10	21209205	21209432	228	229	21209330	31	16.23981	5.75161	12.83549	Adipose_R1_peak_1	Distal Intergen	10	21124930	21160984	36055	2	17863	uc007eog.1	-48221	ENSMUSG000	Myb	myeloblastosis oncogene
chr10	21209222	21209408	187	188	21209341	39	12.34187	3.68871	8.98501	Adipose_R2_peak_2	Distal Intergen	10	21124930	21160984	36055	2	17863	uc007eog.1	-48238	ENSMUSG000	Myb	myeloblastosis oncogene
chr10	21645030	21645234	205	206	21645145	36	20.67077	6.6503	17.14779	Adipose_R1_peak_1	Distal Intergen	10	21690845	21693978	3134	1	432436	uc007eot.1	-45611	NA	Gm5420	predicted gene 5420
chr10	21645097	21645203	107	108	21645143	29	6.74062	2.76653	3.75989	Adipose_R2_peak_2	Distal Intergen	10	21690845	21693978	3134	1	432436	uc007eot.1	-45642	NA	Gm5420	predicted gene 5420
chr10	21734731	21734907	177	178	21734835	40	12.96465	3.78093	9.57654	Adipose_R2_peak_2	Distal Intergen	10	21690845	21693978	3134	1	432436	uc007eot.1	43886	NA	Gm5420	predicted gene 5420
chr10	21735562	21735664	103	104	21735603	28	6.25023	2.67431	3.31626	Adipose_R2_peak_2	Distal Intergen	10	21690845	21693978	3134	1	432436	uc007eot.1	44717	NA	Gm5420	predicted gene 5420
chr10	22138399	22138505	107	108	22138446	31	6.06203	2.50152	3.16628	Adipose_R2_peak_2	Intron (uc029c	10	22113050	22149270	36221	2	319887	uc029qwb.1	10765	ENSMUSG000	E030030I06Rik	RIKEN cDNA E030030I06 gene
chr10	22142291	22143124	834	835	22142449	198	44.08068	3.23237	40.16991	Adipose_R1_peak_1	Intron (uc029c	10	22113050	22149270	36221	2	319887	uc029qwb.1	6146	ENSMUSG000	E030030I06Rik	RIKEN cDNA E030030I06 gene
chr10	22247815	22248093	279	280	22247974	35	19.76029	6.47056	16.26028	Adipose_R1_peak_1	Intron (uc011x	10	22273474	22371114	97641	1	56554	uc011xbo.1	-25381	ENSMUSG000	Raet1d	retinoic acid early transcript delta
chr10	22256908	22257023	116	117	22256950	25	4.86612	2.39766	2.09018	Adipose_R2_peak_2	Intron (uc011x	10	22273474	22371114	97641	1	56554	uc011xbo.1	-16451	ENSMUSG000	Raet1d	retinoic acid early transcript delta
chr10	22290072	22290378	307	308	22290218	71	58.27042	12.94113	54.20487	Adipose_R1_peak_1	Intron (uc007e	10	22306720	22312942	6223	1	606736	uc007epk.2	-16342	ENSMUSG000	C920009B18Ri	RIKEN cDNA C920009B18 gene
chr10	22290077	22290342	266	267	22290209	67	33.06755	6.27081	29.1399	Adipose_R2_peak_2	Intron (uc007e	10	22306720	22312942	6223	1	606736	uc007epk.2	-16378	ENSMUSG000	C920009B18Ri	RIKEN cDNA C920009B18 gene
chr10	22437194	22437365	172	173	22437258	19	7.07687	3.59476	4.08458	Adipose_R1_peak_1	Distal Intergen	10	22402813	22407470	4658	1	1.00E+08	uc007epn.2	34381	ENSMUSG000	Gm10825	predicted gene 10825
chr10	22448260	22448371	112	113	22448313	32	8.29282	3.04319	5.18352	Adipose_R2_peak_2	Distal Intergen	10	22402813	22407470	4658	1	1.00E+08	uc007epn.2	45447	ENSMUSG000	Gm10825	predicted gene 10825
chr10	22470424	22471450	1027	1028	22470866	47	21.99208	5.56727	18.44527	Adipose_R1_peak_1	Distal Intergen	10	22402813	22407470	4658	1	1.00E+08	uc007epn.2	67611	ENSMUSG000	Gm10825	predicted gene 10825
chr10	22470438	22470955	518	519	22470538	57	21.71959	4.63333	18.02697	Adipose_R2_peak_2	Distal Intergen	10	22402813	22407470	4658	1	1.00E+08	uc007epn.2	67625	ENSMUSG000	Gm10825	predicted gene 10825
chr10	22802827	22803060	234	235	22802957	39	23.47001	7.18952	19.88325	Adipose_R1_peak_1	Distal Intergen	10	22817275	22820128	2854	2	21412	uc007eps.2	17068	ENSMUSG000	Tcf21	transcription factor 21
chr10	22802855	22803047	193	194	22802965	45	16.22858	4.24202	12.70278	Adipose_R2_peak_2	Distal Intergen	10	22817275	22820128	2854	2	21412	uc007eps.2	17081	ENSMUSG000	Tcf21	transcription factor 21
chr10	22967417	22967576	160	161	22967499	23	9.86443	4.31371	6.70247	Adipose_R1_peak_1	Distal Intergen	10	22817275	22820128	2854	2	21412	uc007eps.2	-147289	ENSMUSG000	Tcf21	transcription factor 21
chr10	22967426	22967594	169	170	22967511	32	8.29282	3.04319	5.18352	Adipose_R2_peak_2	Distal Intergen	10	22817275	22820128	2854	2	21412	uc007eps.2	-147298	ENSMUSG000	Tcf21	transcription factor 21
chr10	23475867	23475984	118	119	23475982	27	5.77405	2.5821	2.88921	Adipose_R2_peak_2	Distal Intergen	10	23104168	23350786	246619	2	14051	uc007epu.1	-125081	ENSMUSG000	Eya4	EYA transcriptional coactivator and phosphatase 4
chr10	23481531	23481830	300	301	23481669	64	50.01186	11.68296	46.0279	Adipose_R1_peak_1	Distal Intergen	10	23104168	23350786	246619	2	14051	uc007epu.1	-130745	ENSMUSG000	Eya4	EYA transcriptional coactivator and phosphatase 4
chr10	23481584	23481794	211	212	23481679	40	12.96465	3.78093	9.57654	Adipose_R2_peak_2	Distal Intergen	10	23104168	23350786	246619	2	14051	uc007epu.1	-130798	ENSMUSG000	Eya4	EYA transcriptional coactivator and phosphatase 4
chr10	23482956	23483224	269	270	23483083	49	33.45876	8.98689	29.68925	Adipose_R1_peak_1	Distal Intergen	10	23104168	23350786	246619	2	14051	uc007epu.1	-132170	ENSMUSG000	Eya4	EYA transcriptional coactivator and phosphatase 4
chr10	23482965	23483234	270	271	23483103	68	33.91425	6.36302	29.97245	Adipose_R2_peak_2	Distal Intergen	10	23104168	23350786	246619	2	14051	uc007epu.1	-132179	ENSMUSG000	Eya4	EYA transcriptional coactivator and phosphatase 4
chr10	23506798	23506972	175	176	23506880	23	9.86443	4.31371	6.70247	Adipose_R1_peak_1	Distal Intergen	10	23104168	23350786	246619	2	14051	uc007epu.1	-156012	ENSMUSG000	Eya4	EYA transcriptional coactivator and phosphatase 4
chr10	23527957	23528257	301	302	23528088	57	42.07807	10.4248	38.18469	Adipose_R1_peak_1	Distal Intergen	10	23104168	23350786	246619	2	14051	uc007epu.1	-177171	ENSMUSG000	Eya4	EYA transcriptional coactivator and phosphatase 4
chr10	23988585	23988759	175	176	23988673	159	128.9982	14.54742	124.5501	Adipose_R1_peak_1	Promoter (2-3	10	23984609	23985646	1038	2	215855	uc007eqi.1	-2939	ENSMUSG000	Taar6	trace amine-associated receptor 6
chr10	24224163	24224350	188	189	24224264	27	12.93148	5.03266	9.63827	Adipose_R1_peak_1	Promoter (<=1	10	24223517	24302783	79267	1	59012	uc007equ.1	646	ENSMUSG000	Moxd1	monooxygenase, DBH-like 1
chr10	24400525	24400642	118	119	24400628	29	6.74062	2.76653	3.75989	Adipose_R2_peak_2	Distal Intergen	10	24223517	24302783	79267	1	59012	uc007equ.1	177008	ENSMUSG000	Moxd1	monooxygenase, DBH-like 1
chr10	24600605	24601075	471	472	24600744	20	7.74501	3.7745	4.70467	Adipose_R1_peak_1	Distal Intergen	10	24595442	24598682	3241	1	14219	uc011xbr.1	5163	ENSMUSG000	Ctgf	connective tissue growth factor
chr10	24600709	24601055	347	348	24600834	35	9.95877	3.31984	6.73562	Adipose_R2_peak_2	Distal Intergen	10	24595442	24598682	3241	1	14219	uc011xbr.1	5267	ENSMUSG000	Ctgf	connective tissue growth factor
chr10	25420953	25421131	179	180	25421015	20	7.74501	3.7745	4.70467	Adipose_R1_peak_1	Intron (uc007e	10	25408368	25523518	115151	1	13822	uc007ern.3	12585	ENSMUSG000	Epb41l2	erythrocyte membrane protein band 4.1 like 2
chr10	25458906	25459048	143	144	25458970	19	7.07687	3.59476	4.08458	Adipose_R1_peak_1	Intron (uc007e	10	25466973	25502138	35166	1	13822	uc007err.1	-7925	ENSMUSG000	Epb41l2	erythrocyte membrane protein band 4.1 like 2
chr10	25474727	25474942	216	217	25474892	34	9.39139	3.22762	6.20474	Adipose_R2_peak_2	Intron (uc007e	10	25466973	25502138	35166	1	13822	uc007err.1	7754	ENSMUSG000	Epb41l2	erythrocyte membrane protein band 4.1 like 2
chr10	25669041	25669231	191	192	25669153	31	16.23981	5.75161	12.83549	Adipose_R1_peak_1	Distal Intergen	10	25527943	25536272	8330	2	1.01E+08	uc007erv.2	-132769	ENSMUSG000	Smlr1	small leucine-rich protein 1
chr10	25669081	25669212	132	133	25669165	29	6.74062	2.76653	3.75989	Adipose_R2_peak_2	Distal Intergen	10	25527943	25536272	8330	2	1.01E+08	uc007erv.2	-132809	ENSMUSG000	Smlr1	small leucine-rich protein 1
chr10	25941795	25942043	249	250	25941933	38	22.52591	7.00978	18.96	Adipose_R1_peak_1	Distal Intergen	10	25779314	25837437	58124	2	73803	uc029qwl.1	-104358	NA	4930401C15Ri	RIKEN cDNA 4930401C15 gene
chr10	25941831	25942010	180	181	25941934	34	9.39139	3.22762	6.20474	Adipose_R2_peak_2	Distal Intergen	10	25779314	25837437	58124	2	73803	uc029qwl.1	-104394	NA	4930401C15Ri	RIKEN cDNA 4930401C15 gene
chr10	25971045	25971291	247	248	25971152	33	17.97508	6.11109	14.52164	Adipose_R1_peak_1	Distal Intergen	10	26078255	26079447	1193	1	1.00E+08	uc011xbw.1	-106964	ENSMUSG000	Gm9767	predicted gene 9767
chr10	25971110	25971210	101	102	25971155	30	7.24477	2.85875	4.21917	Adipose_R2_peak_2	Distal Intergen	10	26078255	26079447	1193	1	1.00E+08	uc011xbw.1	-107045	ENSMUSG000	Gm9767	predicted gene 9767
chr10	26732491	26732809	319	320	26732682	42	26.36523	7.72873	22.71906	Adipose_R1_peak_1	Distal Intergen	10	26772512	26918648	146137	1	73910	uc007ese.1	-39703	ENSMUSG000	Arhgap18	Rho GTPase activating protein 18
chr10	26732545	26732796	252	253	26732679	49	19.00719	4.61089	15.38598	Adipose_R2_peak_2	Distal Intergen	10	26772512	26918648	146137	1	73910	uc007ese.1	-39716	ENSMUSG000	Arhgap18	Rho GTPase activating protein 18
chr10	28911157	28911302	146	147	28911224	20	7.74501	3.7745	4.70467	Adipose_R1_peak_1	Distal Intergen	10	28921963	28923507	1545	2	114676	uc011xcc.1	12205	NA	4930519F09Ri	pyruvate dehydrogenase E1 alpha 1 pseudogene
chr10	28911183	28911314	132	133	28911228	29	6.74062	2.76653	3.75989	Adipose_R2_peak_2	Distal Intergen	10	28921963	28923507	1545	2	114676	uc011xcc.1	12193	NA	4930519F09Ri	pyruvate dehydrogenase E1 alpha 1 pseudogene
chr10	28918995	28919247	253	254	28919116	58	43.19021	10.60454	39.28351	Adipose_R1_peak_1	Downstream (	10	28921963	28923507	1545	2	114676	uc011xcc.1	4260	NA	4930519F09Ri	pyruvate dehydrogenase E1 alpha 1 pseudogene
chr10	28919005	28919242	238	239	28919115	60	27.32259	5.62528	23.49644	Adipose_R2_peak_2	Downstream (	10	28921963	28923507	1545	2	114676	uc011xcc.1	4265	NA	4930519F09Ri	pyruvate dehydrogenase E1 alpha 1 pseudogene
chr10	28937547	28937727	181	182	28937618	23	9.86443	4.31371	6.70247	Adipose_R1_peak_1	Distal Intergen	10	28921963	28923507	1545	2	114676	uc011xcc.1	-14040	NA	4930519F09Ri	pyruvate dehydrogenase E1 alpha 1 pseudogene
chr10	28939058	28939310	253	254	28939173	47	31.38649	8.62742	27.65048	Adipose_R1_peak_1	Distal Intergen	10	28921963	28923507	1545	2	114676	uc011xcc.1	-15551	NA	4930519F09Ri	pyruvate dehydrogenase E1 alpha 1 pseudogene
chr10	28939075	28939256	182	183	28939165	45	16.22858	4.24202	12.70278	Adipose_R2_peak_2	Distal Intergen	10	28921963	28923507	1545	2	114676	uc011xcc.1	-15568	NA	4930519F09Ri	pyruvate dehydrogenase E1 alpha 1 pseudogene
chr10	29081607	29081917	311	312	29081733	53	37.70483	9.70585	33.87	Adipose_R1_peak_1	Distal Intergen	10	29143996	29199628	55633	1	67412	uc007est.1	-62079	ENSMUSG000	Soga3	SOGA family member 3
chr10	29081615	29081883	269	270	29081741	51	20.44827	4.79532	16.78434	Adipose_R2_peak_2	Distal Intergen	10	29143996	29199628	55633	1	67412	uc007est.1	-62113	ENSMUSG000	Soga3	SOGA family member 3
chr10	29104325	29104475	151	152	29104382	19	7.07687	3.59476	4.08458	Adipose_R1_peak_1	Distal Intergen	10	29143996	29199628	55633	1	67412	uc007est.1	-39521	ENSMUSG000	Soga3	SOGA family member 3
chr10	29327334	29327543	210	211	29327427	28	13.73721	5.2124	10.41463	Adipose_R1_peak_1	Intron (uc007e	10	29313500	29346661	33162	1	52665	uc007esx.2	13834	ENSMUSG000	Echdc1	enoyl Coenzyme A hydratase domain containing 1
chr10	29327367	29327513	147	148	29327453	32	8.29282	3.04319	5.18352	Adipose_R2_peak_2	Intron (uc007e	10	29313500	29346661	33162	1	52665	uc007esx.2	13867	ENSMUSG000	Echdc1	enoyl Coenzyme A hydratase domain containing 1
chr10	29518859	29519045	187	188	29518963	19	7.07687	3.59476	4.08458	Adipose_R1_peak_1	Intron (uc007e	10	29453107	29535867	82761	2	72780	uc007etb.2	16822	ENSMUSG000	Rspo3	R-spondin 3
chr10	29762304	29762444	141	142	29762367	23	9.86443	4.31371	6.70247	Adipose_R1_peak_1	Distal Intergen	10	29453107	29535867	82761	2	72780	uc007etb.2	-226437	ENSMUSG000	Rspo3	R-spondin 3
chr10	29762305	29762478	174	175	29762372	28	6.25023	2.67431	3.31626	Adipose_R2_peak_2	Distal Intergen	10	29453107	29535867	82761	2	72780	uc007etb.2	-226438	ENSMUSG000	Rspo3	R-spondin 3
chr10	29771232	29771559	328	329	29771378	73	60.68511	13.3006	56.59814	Adipose_R1_peak_1	Distal Intergen	10	29453107	29535867	82761	2	72780	uc007etb.2	-235365	ENSMUSG000	Rspo3	R-spondin 3
chr10	29771303	29771516	214	215	29771396	40	12.96465	3.78093	9.57654	Adipose_R2_peak_2	Distal Intergen	10	29453107	29535867	82761	2	72780	uc007etb.2	-235436	ENSMUSG000	Rspo3	R-spondin 3
chr10	29956224	29956423	200	201	29956297	20	7.74501	3.7745	4.70467	Adipose_R1_peak_1	Distal Intergen	10	30194580	30200540	5961	2	66311	uc007etc.3	244117	ENSMUSG000	Cenpw	centromere protein W
chr10	30060134	30060431	298	299	30060296	63	48.25501	11.28462	44.29316	Adipose_R1_peak_1	Distal Intergen	10	30194580	30200540	5961	2	66311	uc007etc.3	140109	ENSMUSG000	Cenpw	centromere protein W
chr10	30060151	30060431	281	282	30060293	58	25.74274	5.44085	21.94821	Adipose_R2_peak_2	Distal Intergen	10	30194580	30200540	5961	2	66311	uc007etc.3	140109	ENSMUSG000	Cenpw	centromere protein W
chr10	30575508	30575707	200	201	30575608	26	12.14079	4.85292	8.87838	Adipose_R1_peak_1	Intron (uc007e	10	30534225	30600749	66525	2	73681	uc007ete.1	25042	ENSMUSG000	Trmt11	tRNA methyltransferase 11
chr10	30575554	30575669	116	117	30575612	31	7.7623	2.95097	4.69388	Adipose_R2_peak_2	Intron (uc007e	10	30534225	30600749	66525	2	73681	uc007ete.1	25080	ENSMUSG000	Trmt11	tRNA methyltransferase 11
chr10	31409124	31409367	244	245	31409261	35	19.76029	6.47056	16.26028	Adipose_R1_peak_1	Intron (uc007e	10	31413726	31414435	710	2	1.00E+08	uc011xck.1	5068	ENSMUSG000	Supt4b	suppressor of Ty 4B
chr10	31409169	31409367	199	200	31409284	42	14.2409	3.96536	10.79479	Adipose_R2_peak_2	Intron (uc007e	10	31413726	31414435	710	2	1.00E+08	uc011xck.1	5068	ENSMUSG000	Supt4b	suppressor of Ty 4B
chr10	31667828	31668313	486	487	31668151	65	51.17258	11.8627	47.17609	Adipose_R1_peak_1	Distal Intergen	10	31501887	31609725	107839	2	268291	uc007etu.2	-58103	ENSMUSG000	Rnf217	ring finger protein 217
chr10	31667861	31668302	442	443	31667972	37	11.12817	3.50427	7.83613	Adipose_R2_peak_2	Distal Intergen	10	31501887	31609725	107839	2	268291	uc007etu.2	-58136	ENSMUSG000	Rnf217	ring finger protein 217
chr10	31716364	31716516	153	154	31716427	19	7.07687	3.59476	4.08458	Adipose_R1_peak_1	Intron (uc007e	10	31501887	31609725	107839	2	268291	uc007etu.2	-106639	ENSMUSG000	Rnf217	ring finger protein 217
chr10	31760260	31760507	248	249	31760390	40	24.42476	7.36925	20.81738	Adipose_R1_peak_1	Intron (uc007e	10	31501887	31609725	107839	2	268291	uc007etu.2	-150535	ENSMUSG000	Rnf217	ring finger protein 217
chr10	31760287	31760459	173	174	31760389	38	11.72962	3.59649	8.4047	Adipose_R2_peak_2	Intron (uc007e	10	31501887	31609725	107839	2	268291	uc007etu.2	-150562	ENSMUSG000	Rnf217	ring finger protein 217
chr10	31982782	31982913	132	133	31982831	33	8.83596	3.1354	5.68652	Adipose_R2_peak_2	Intron (uc007e	10	31501887	31609725	107839	2	268291	uc007etu.2	-373057	ENSMUSG000	Rnf217	ring finger protein 217

chr10	31983394	31983657	264	265	31983525	36	20.67077	6.6503	17.14779	Adipose_R1_peak_1	Intron (uc007e	10	31501887	31609725	107839	2	268291	uc007etu.2	-373669	ENSMUSG000	Rnf217	ring finger protein 217
chr10	31983449	31983634	186	187	31983525	38	11.72962	3.59649	8.4047	Adipose_R2_peak_1	Intron (uc007e	10	31501887	31609725	107839	2	268291	uc007etu.2	-373724	ENSMUSG000	Rnf217	ring finger protein 217
chr10	32180647	32180927	281	282	32180785	53	37.70483	9.70585	33.87	Adipose_R1_peak_1	Intron (uc007e	10	31693637	32402373	708737	2	432450	uc007etx.1	221446	ENSMUSG000	Nkain2	Na+/K+ transporting ATPase interacting 2
chr10	32180686	32180908	223	224	32180799	52	21.18119	4.88754	17.49655	Adipose_R2_peak_2	Intron (uc007e	10	31693637	32402373	708737	2	432450	uc007etx.1	221465	ENSMUSG000	Nkain2	Na+/K+ transporting ATPase interacting 2
chr10	32838659	32839354	696	697	32838793	89	66.15358	11.53511	62.02182	Adipose_R1_peak_1	Intron (uc007e	10	31689319	32889915	1200597	2	432450	uc007etw.2	50561	ENSMUSG000	Nkain2	Na+/K+ transporting ATPase interacting 2
chr10	32838662	32838931	270	271	32838788	90	53.97882	8.39181	49.78112	Adipose_R2_peak_2	Intron (uc007e	10	31689319	32889915	1200597	2	432450	uc007etw.2	50984	ENSMUSG000	Nkain2	Na+/K+ transporting ATPase interacting 2
chr10	32839131	32839321	191	192	32839212	38	11.72962	3.59649	8.4047	Adipose_R2_peak_2	Intron (uc007e	10	31689319	32889915	1200597	2	432450	uc007etw.2	50594	ENSMUSG000	Nkain2	Na+/K+ transporting ATPase interacting 2
chr10	32938181	32938313	133	134	32938244	23	9.86443	4.31371	6.70247	Adipose_R1_peak_2	Distal Interger	10	31689319	32889915	1200597	2	432450	uc007etw.2	-48266	ENSMUSG000	Nkain2	Na+/K+ transporting ATPase interacting 2
chr10	34438420	34438738	319	320	34438567	70	57.07198	12.76139	53.01762	Adipose_R1_peak_2	Distal Interger	10	34303612	34418528	114917	2	319638	uc007euv.1	-19892	ENSMUSG000	Nt5dc1	5'-nucleotidase domain containing 1
chr10	34438456	34438699	244	245	34438573	53	21.92215	4.97976	18.21755	Adipose_R2_peak_2	Distal Interger	10	34303612	34418528	114917	2	319638	uc007euv.1	-19928	ENSMUSG000	Nt5dc1	5'-nucleotidase domain containing 1
chr10	34565590	34565751	162	163	34565667	31	16.23981	5.75161	12.83549	Adipose_R1_peak_2	Intron (uc007e	10	34483400	34606280	122881	1	14302	uc007eva.2	82190	ENSMUSG000	Frk	fyn-related kinase
chr10	34668658	34669066	409	410	34668754	28	13.73721	5.2124	10.41463	Adipose_R1_peak_2	Distal Interger	10	34483400	34606280	122881	1	14302	uc007eva.2	185258	ENSMUSG000	Frk	fyn-related kinase
chr10	34745981	34746538	558	559	34746102	35	19.76029	6.47056	16.26028	Adipose_R1_peak_2	Distal Interger	10	34483400	34606280	122881	1	14302	uc007eva.2	262581	ENSMUSG000	Frk	fyn-related kinase
chr10	34746006	34746205	200	201	34746099	41	13.59774	3.87315	10.1803	Adipose_R2_peak_2	Distal Interger	10	34483400	34606280	122881	1	14302	uc007eva.2	262606	ENSMUSG000	Frk	fyn-related kinase
chr10	34854971	34855153	183	184	34854992	16	5.20017	3.05554	2.38734	Adipose_R1_peak_2	Distal Interger	10	34483400	34606280	122881	1	14302	uc007eva.2	371571	ENSMUSG000	Frk	fyn-related kinase
chr10	34959688	34959818	131	132	34959757	26	5.31252	2.48988	2.47999	Adipose_R2_peak_2	Distal Interger	10	34483400	34606280	122881	1	14302	uc007eva.2	476288	ENSMUSG000	Frk	fyn-related kinase
chr10	35221366	35221497	132	133	35221388	16	5.20017	3.05554	2.38734	Adipose_R1_peak_2	Distal Interger	10	34483400	34606280	122881	1	14302	uc007eva.2	737966	ENSMUSG000	Frk	fyn-related kinase
chr10	35615286	35615450	165	166	35615447	34	9.39139	3.22762	6.20474	Adipose_R2_peak_2	Distal Interger	10	36506807	36834394	327588	1	319415	uc007evc.1	-891357	ENSMUSG000	Hs3st5	heparan sulfate (glucosamine) 3-O-sulfotransferase 5
chr10	35635582	35635832	251	252	35635686	27	12.93148	5.03266	9.63827	Adipose_R1_peak_2	Distal Interger	10	36506807	36834394	327588	1	319415	uc007evc.1	-870975	ENSMUSG000	Hs3st5	heparan sulfate (glucosamine) 3-O-sulfotransferase 5
chr10	35637111	35637328	218	219	35637246	21	8.43286	3.95423	5.34896	Adipose_R1_peak_2	Distal Interger	10	36506807	36834394	327588	1	319415	uc007evc.1	-869479	ENSMUSG000	Hs3st5	heparan sulfate (glucosamine) 3-O-sulfotransferase 5
chr10	35671122	35671562	441	442	35671364	77	65.58373	14.01956	61.45456	Adipose_R1_peak_2	Distal Interger	10	36506807	36834394	327588	1	319415	uc007evc.1	-835245	ENSMUSG000	Hs3st5	heparan sulfate (glucosamine) 3-O-sulfotransferase 5
chr10	35671213	35671424	212	213	35671299	41	13.59774	3.87315	10.1803	Adipose_R2_peak_2	Distal Interger	10	36506807	36834394	327588	1	319415	uc007evc.1	-835383	ENSMUSG000	Hs3st5	heparan sulfate (glucosamine) 3-O-sulfotransferase 5
chr10	35672669	35672887	219	220	35672757	35	19.76029	6.47056	16.26028	Adipose_R1_peak_2	Distal Interger	10	36506807	36834394	327588	1	319415	uc007evc.1	-833920	ENSMUSG000	Hs3st5	heparan sulfate (glucosamine) 3-O-sulfotransferase 5
chr10	35806344	35806476	133	134	35806454	19	7.07687	3.59476	4.08458	Adipose_R1_peak_2	Distal Interger	10	36506807	36834394	327588	1	319415	uc007evc.1	-700331	ENSMUSG000	Hs3st5	heparan sulfate (glucosamine) 3-O-sulfotransferase 5
chr10	35942590	35942844	255	256	35942736	50	34.50784	9.16663	30.72138	Adipose_R1_peak_2	Distal Interger	10	36506807	36834394	327588	1	319415	uc007evc.1	-563963	ENSMUSG000	Hs3st5	heparan sulfate (glucosamine) 3-O-sulfotransferase 5
chr10	35942615	35942804	190	191	35942737	44	15.55653	4.1498	12.05625	Adipose_R2_peak_2	Distal Interger	10	36506807	36834394	327588	1	319415	uc007evc.1	-564003	ENSMUSG000	Hs3st5	heparan sulfate (glucosamine) 3-O-sulfotransferase 5
chr10	36015501	36015719	219	220	36015565	49	33.45876	8.98689	29.68925	Adipose_R1_peak_2	Distal Interger	10	36506807	36834394	327588	1	319415	uc007evc.1	-491088	ENSMUSG000	Hs3st5	heparan sulfate (glucosamine) 3-O-sulfotransferase 5
chr10	36130452	36130690	239	240	36130584	50	34.50784	9.16663	30.72138	Adipose_R1_peak_2	Distal Interger	10	36506807	36834394	327588	1	319415	uc007evc.1	-376117	ENSMUSG000	Hs3st5	heparan sulfate (glucosamine) 3-O-sulfotransferase 5
chr10	36130477	36130686	210	211	36130597	43	14.8939	4.05758	11.42017	Adipose_R2_peak_2	Distal Interger	10	36506807	36834394	327588	1	319415	uc007evc.1	-376121	ENSMUSG000	Hs3st5	heparan sulfate (glucosamine) 3-O-sulfotransferase 5
chr10	36134383	36134631	249	250	36134513	52	36.63102	9.52611	32.81188	Adipose_R1_peak_2	Distal Interger	10	36506807	36834394	327588	1	319415	uc007evc.1	-372176	ENSMUSG000	Hs3st5	heparan sulfate (glucosamine) 3-O-sulfotransferase 5
chr10	36134385	36134608	224	225	36134512	50	19.72356	4.7031	16.08069	Adipose_R2_peak_2	Distal Interger	10	36506807	36834394	327588	1	319415	uc007evc.1	-372199	ENSMUSG000	Hs3st5	heparan sulfate (glucosamine) 3-O-sulfotransferase 5
chr10	36161376	36161591	216	217	36161532	27	5.77405	2.5821	2.88921	Adipose_R2_peak_2	Distal Interger	10	36506807	36834394	327588	1	319415	uc007evc.1	-345216	ENSMUSG000	Hs3st5	heparan sulfate (glucosamine) 3-O-sulfotransferase 5
chr10	36163005	36163206	202	203	36163116	25	11.36566	4.67319	8.13482	Adipose_R1_peak_2	Distal Interger	10	36506807	36834394	327588	1	319415	uc007evc.1	-343601	ENSMUSG000	Hs3st5	heparan sulfate (glucosamine) 3-O-sulfotransferase 5
chr10	36223624	36223726	103	104	36223662	26	5.31252	2.48988	2.47999	Adipose_R2_peak_2	Distal Interger	10	36506807	36834394	327588	1	319415	uc007evc.1	-283081	ENSMUSG000	Hs3st5	heparan sulfate (glucosamine) 3-O-sulfotransferase 5
chr10	36277150	36277341	192	193	36277240	22	9.13959	4.13397	6.01526	Adipose_R1_peak_2	Distal Interger	10	36506807	36834394	327588	1	319415	uc007evc.1	-229466	ENSMUSG000	Hs3st5	heparan sulfate (glucosamine) 3-O-sulfotransferase 5
chr10	36278049	36278216	168	169	36278119	23	9.86443	4.31371	6.70247	Adipose_R1_peak_2	Distal Interger	10	36506807	36834394	327588	1	319415	uc007evc.1	-228591	ENSMUSG000	Hs3st5	heparan sulfate (glucosamine) 3-O-sulfotransferase 5
chr10	36278736	36278894	159	160	36278819	18	6.42934	3.41502	3.48999	Adipose_R1_peak_2	Distal Interger	10	36506807	36834394	327588	1	319415	uc007evc.1	-227913	ENSMUSG000	Hs3st5	heparan sulfate (glucosamine) 3-O-sulfotransferase 5
chr10	36780415	36780718	304	305	36780581	73	60.68511	13.3006	56.59814	Adipose_R1_peak_2	Intron (uc007e	10	36974544	36996111	21568	1	15182	uc007eve.1	-193826	ENSMUSG000	Hdac2	histone deacetylase 2
chr10	36780466	36780676	211	212	36780575	52	21.18119	4.88754	17.49655	Adipose_R2_peak_2	Intron (uc007e	10	36974544	36996111	21568	1	15182	uc007eve.1	-193868	ENSMUSG000	Hdac2	histone deacetylase 2
chr10	36822788	36823044	257	258	36822925	36	20.67077	6.6503	17.14779	Adipose_R1_peak_2	Intron (uc007e	10	36974544	36996111	21568	1	15182	uc007eve.1	-151500	ENSMUSG000	Hdac2	histone deacetylase 2
chr10	37137312	37137497	186	187	37137363	27	5.77405	2.5821	2.88921	Adipose_R2_peak_2	Promoter (1-2	10	37133243	37138926	5684	2	17118	uc007evg.1	1429	ENSMUSG000	Marcks	myristoylated alanine rich protein kinase C substrate
chr10	37137364	37137517	154	155	37137428	21	8.43286	3.95423	5.34896	Adipose_R1_peak_2	Promoter (1-2	10	37133243	37138926	5684	2	17118	uc007evg.1	1409	ENSMUSG000	Marcks	myristoylated alanine rich protein kinase C substrate
chr10	37139413	37139596	184	185	37139508	20	7.74501	3.7745	4.70467	Adipose_R1_peak_2	Promoter (<=:	10	37133243	37138926	5684	2	17118	uc007evg.1	-487	ENSMUSG000	Marcks	myristoylated alanine rich protein kinase C substrate
chr10	37139523	37139879	357	358	37139637	30	7.24477	2.85875	4.21917	Adipose_R2_peak_2	Promoter (<=:	10	37133243	37138926	5684	2	17118	uc007evg.1	-597	ENSMUSG000	Marcks	myristoylated alanine rich protein kinase C substrate
chr10	37140201	37140474	274	275	37140358	36	10.5378	3.41206	7.27949	Adipose_R2_peak_2	Promoter (1-2	10	37133243	37138926	5684	2	17118	uc007evg.1	-1275	ENSMUSG000	Marcks	myristoylated alanine rich protein kinase C substrate
chr10	37142347	37142492	146	147	37142419	26	5.31252	2.48988	2.47999	Adipose_R2_peak_2	Intron (uc007e	10	37133243	37138926	5684	2	17118	uc007evg.1	-3421	ENSMUSG000	Marcks	myristoylated alanine rich protein kinase C substrate
chr10	37186660	37186853	194	195	37186723	44	28.34544	8.08821	24.66239	Adipose_R1_peak_2	Distal Interger	10	37133243	37138926	5684	2	17118	uc007evg.1	-47734	ENSMUSG000	Marcks	myristoylated alanine rich protein kinase C substrate
chr10	37187398	37187593	196	197	37187504	23	9.86443	4.31371	6.70247	Adipose_R1_peak_2	Distal Interger	10	37133243	37138926	5684	2	17118	uc007evg.1	-48472	ENSMUSG000	Marcks	myristoylated alanine rich protein kinase C substrate
chr10	37187432	37187571	140	141	37187511	34	9.39139	3.22762	6.20474	Adipose_R2_peak_2	Distal Interger	10	37133243	37138926	5684	2	17118	uc007evg.1	-48506	ENSMUSG000	Marcks	myristoylated alanine rich protein kinase C substrate
chr10	37577579	37578007	429	430	37577717	341	270.4196	14.89291	265.6947	Adipose_R1_peak_2	Distal Interger	10	37133243	37138926	5684	2	17118	uc007evg.1	-438653	ENSMUSG000	Marcks	myristoylated alanine rich protein kinase C substrate
chr10	37577583	37577968	386	387	37577723	222	121.268	7.9999	116.6863	Adipose_R2_peak_2	Distal Interger	10	37133243	37138926	5684	2	17118	uc007evg.1	-438657	ENSMUSG000	Marcks	myristoylated alanine rich protein kinase C substrate
chr10	37706663	37706820	158	159	37706727	19	7.07687	3.59476	4.08458	Adipose_R1_peak_2	Distal Interger	10	38175774	38181903	6130	1	75877	uc007evj.2	-468954	NA	4930591E09Ri	RIKEN cDNA 4930591E09 gene
chr10	38028076	38028271	196	197	38028210	22	9.13959	4.13397	6.01526	Adipose_R1_peak_2	Distal Interger	10	38175774	38181903	6130	1	75877	uc007evj.2	-147503	NA	4930591E09Ri	RIKEN cDNA 4930591E09 gene
chr10	38106937	38107071	135	136	38106967	26	5.31252	2.48988	2.47999	Adipose_R2_peak_2	Distal Interger	10	38175774	38181903	6130	1	75877	uc007evj.2	-68703	NA	4930591E09Ri	RIKEN cDNA 4930591E09 gene
chr10	38603381	38603577	197	198	38603470	21	8.43286	3.95423	5.34896	Adipose_R1_peak_2	Distal Interger	10	38820541	38821779	1239	2	215919	uc011xct.1	218202	ENSMUSG000	Rfpl4b	ret finger protein-like 4B
chr10	38603391	38603560	170	171	38603462	31	7.7623	2.95097	4.69388	Adipose_R2_peak_2	Distal Interger	10	38820541	38821779	1239	2	215919	uc011xct.1	218219	ENSMUSG000	Rfpl4b	ret finger protein-like 4B
chr10	38605733	38606063	331	332	38605873	93	86.02155	16.89536	81.75695	Adipose_R1_peak_2	Distal Interger	10	38820541	38821779	1239	2	215919	uc011xct.1	215716	ENSMUSG000	Rfpl4b	ret finger protein-like 4B
chr10	38605736	38606026	291	292	38605865	78	42.70965	7.2852	38.64095	Adipose_R2_peak_2	Distal Interger	10	38820541	38821779	1239	2	215919	uc011xct.1	215753	ENSMUSG000	Rfpl4b	ret finger protein-like 4B
chr10	38641100	38641310	211	212	38641195	27	12.93148	5.03266	9.63827	Adipose_R1_peak_2	Distal Interger	10	38820541	38821779	1239	2	215919	uc011xct.1	180469	ENSMUSG000	Rfpl4b	ret finger protein-like 4B
chr10	38756006	38756223	218	219	38756120	33	17.97508	6.11109	14.52164	Adipose_R1_peak_2	Distal Interger	10	38820541	38821779	1239	2	215919	uc011xct.1	65556	ENSMUSG000	Rfpl4b	ret finger protein-like 4B
chr10	38764572	38764882	311	312	38764673	236	204.7146	17.19263	200.077	Adipose_R1_peak_2	Distal Interger	10	38820541	38821779	1239	2	215919	uc011xct.1	56897	ENSMUSG000	Rfpl4b	ret finger protein-like 4B
chr10	38764573	38764864	292	293	38764678	144	102.9725	11.58332	98.45319	Adipose_R2_peak_2	Distal Interger	10	38820541	38821779	1239	2	215919	uc011xct.1	56915	ENSMUSG000	Rfpl4b	ret finger protein-like 4B
chr10	38770025	38770196	172	173	38770108	17	5.80341	3.23528	2.92378	Adipose_R1_peak_2	Distal Interger	10	38820541	38821779	1239	2	215919	uc011xct.1	51583	ENSMUSG000	Rfpl4b	ret finger protein-like 4B
chr10	38772485	38772711	227	228	38772597	22	9.13959	4.13397	6.01526	Adipose_R1_peak_2	Distal Interger	10	38820541	38821779	1239	2	215919	uc011xct.1	49068	ENSMUSG000	Rfpl4b	ret finger protein-like 4B
chr10	38814375	38814506	132	133	38814454	17	5.80341	3.23528	2.92378	Adipose_R1_peak_2	Distal Interger	10	38820541	38821779	1239	2	215919	uc011xct.1	7273	ENSMUSG000	Rfpl4b	ret finger protein-like 4B
chr10	39161833	39162043	211	212	39161989	22	9.13959	4.13397	6.01526	Adipose_R1_peak_2	Promoter (1-2	10	39150971	39163794	12824	2	327743	uc011xcu.1	1751	ENSMUSG000	Wisp3	WNT1 inducible signaling pathway protein 3
chr10	39198581	39198787	207	208	39198687	36	10.5378	3.41206	7.27949	Adipose_R2_peak_2	Distal Interger	10	39150971	39163794	12824	2	327743	uc011xcu.1	-34787	ENSMUSG000	Wisp3	WNT1 inducible signaling pathway protein 3
chr10	39919884	39920105	222	223	39919993	35	19.76029	6.47056	16.26028	Adipose_R1_peak_2	Downstream (	10	39920382	39926923	6542	2	72045	uc007ewh.1	6818	ENSMUSG000	Mfsd4b2	major facilitator superfamily domain containing 4B2
chr10	39919906	39920079	174	175	39919998	31	7.7623	2.95097	4.69388	Adipose_R2_peak_2	Downstream (	10	39920382	39926923	6542	2	72045	uc007ewh.1	6844	ENSMUSG000	Mfsd4b2	major facilitator superfamily domain containing 4B2
chr10	40645979	40647126	1148	1149	40646124	68	43.69006	8.84359	39.7811	Adipose_R1_peak_2	Intron (uc007e	10	40630011	40641568	11558	1	70503	uc057awo.1	15968	ENSMUSG000	Ddo	D-aspartate oxidase
chr10	40646641	40646764	124	125	40646710	28	6.25023	2.67431	3.31626	Adipose_R2_peak_2	Intron (uc007e	10	40630011	40641568	11558	1	70503	uc057awo.1	16630	ENSMUSG000	Ddo	D-aspartate oxidase
chr10	42998535	42998906	372	373	42998647	30	15.39182	5.57188	12.01394	Adipose_R1_peak_2	Distal Interger	10	42939881	42960782	20902	1	268297	uc007eze.2	58654	ENSMUSG000	Scml4	sex comb on midleg-like 4 (Drosophila)
chr10	43055335	43055620	286	287	43055479	32	17.10103	5.93135	13.67137	Adipose_R1_peak_2	Intron (uc007e	10	42939881	42960782	20902	1	268297	uc007eze.2	115454	ENSMUSG000	Scml4	sex comb on midleg-like 4 (Drosophila)
chr10	43055354	43055655	302	303	43055525	48	18.29934	4.51867	14.70076	Adipose_R2_peak_2	Intron (uc007e	10	42939881	42960782	20902	1	268297	uc007eze.2	115473	ENSMUSG000	Scml4	sex comb on midleg-like 4 (Drosophila)
chr10	46090279	46090636	358	359	46090487	28	13.73721	5.2124	10.41463	Adipose_R1_peak_2	Distal Interger	10	46244748	46244770	23	1	1.00E+08	uc029qxb.1	-154112	ENSMUSG000	Snord14d	small nucleolar RNA, C/D box 14D
chr10	46136891	46137120	230	231	46137009	43	27.35048	7.90847	23.68561	Adipose_R1_peak_2	Distal Interger	10	46244748	46244770	23	1	1.00E+08	uc029qxb.1	-107628	ENSMUSG000	Snord14d	small nucleolar RNA, C/D box 14D
chr10	46136924	46137081	158	159	46137013	38	11.72962	3.59649	8.4047	Adipose_R2_peak_2	Distal Interger	10	46244748	46244770	23	1	1.00E+08	uc029qxb.1	-107667	ENSMUSG000	Snord14d	small nucleolar RNA, C/D box 14D
chr10	46302575	46302723	149	150	46302636	18	6.42934	3.41502	3.48999	Adipose_R1_peak_2	Distal Interger	10	46244748	46244770	23	1	1.00E+08	uc029qxb.1	57827	ENSMUSG000	Snord14d	small nucleolar RNA, C/D box 14D
chr10	46601875	46602018	144	145	46601995	19	7.07687	3.59476	4.08458	Adipose_R1_peak_2	Distal Interger	10	46244748	46244770	23	1	1.00E+08	uc029qxb.1	357127	ENSMUSG000	Snord14d	small nucleolar RNA, C/D box 14D
chr10	46703761	46703919	159	160	46703845	18	6.42934	3.41502	3.48999	Adipose_R1_peak_2	Distal Interger	10	46244748	46244770	23	1	1.00E+08	uc029qxb.1	459013	ENSMUSG000	Snord14d	small nucleolar RNA, C/D box 14D
chr10	46753780	46753941	162	163	46753840	22	9.13959	4.13397	6.01526	Adipose_R1_peak_2	Distal Interger	10	46244748	46244770	23	1	1.00E+08	uc029qxb.1	509032	ENSMUSG000	Snord14d	small nucleolar RNA, C/D box 14D
chr10	46765720	46765940	221	222	46765820	48	27.78074	7.18601	24.11389	Adipose_R1_peak_2	Distal Interger	10	46244748	46244770	23	1	1.00E+08	uc029qxb.1	520972	ENSMUSG000	Snord14d	small nucleolar RNA, C/D box 14D
chr10	46765722	46765915	194	195	46765808	40	12.96465	3.78093	9.57654	Adipose_R2_peak_2	Distal Interger	10	46244748	46244770	23	1	1.00E+08	uc029qxb.1	520974	ENSMUSG000	Snord14d	small nucleolar RNA, C/D box 14D
chr10	46811298	46811581	284	285	46811430	64	48.05606	10.98474	44.09508	Adipose_R1_peak_2	Distal Interger	10	46244748	46244770	23	1	1.00E+08	uc029qxb.1	566550	ENSMUSG000	Snord14d	small nucleolar RNA, C/D box 14D
chr10	46811325	46811575	251	252	46811443	56	24.19188	5.25641	20.43163	Adipose_R2_peak_2	Distal Interger	10	46244748	46244770	23	1	1.00E+08	uc029qxb.1	566577	ENSMUSG000	Snord14d	small nucleolar RNA, C/D box 14D
chr10	46811847	46812108	262	263	46811972	39	23.47001	7.18952	19.88325	Adipose_R1_peak_2	Distal Interger	10	46244748	46244770	23	1	1.00E+08	uc029qxb.1	567099	ENSMUSG000	Snord14d	small nucleolar RNA, C/D box 14D
chr10	46811887	46812066	180	181	46811971	40	12.96465	3.78093	9.57654	Adipose_R2_peak_2	Distal Interger	10	46244748	46244770	23	1	1.00E+08	uc029qxb.1	567139	ENSMUSG000	Snord14d	small nucleolar RNA, C/D box 14D
chr10	47167652	47167840	189	190	47167714	21	8.43286	3.95423	5.34896	Adipose_R1_peak_2	Distal Interger	10	46244748	46244770	23	1	1.00E+08	uc029qxb.1	922904	ENSMUSG000	Snord14d	small nucleolar RNA, C/D box 14D
chr10	47221912	47222222	311	312	47222045	54	38.78658	9.88558	34.93639	Adipose_R1_peak_2	Distal Interger	10	46244748	46244770	23	1	1.00E+08	uc029qxb.1	977164	ENSMUSG000	Snord14d	small nucleolar RNA, C/D box 14D
chr10	47221925	47222171	247	248	47222053	47	17.60017	4.42645	14.02531	Adipose_R2_peak_2	Distal Interger	10	46244748	46244770	23	1	1.00E+08	uc029qxb.1	977177	ENSMUSG000	Snord14d	small nucleolar RNA, C/D box 14D
chr10	47331678	47331925	248	249	47331798	32	17.10103	5.93135	13.67137	Adipose_R1_peak_2	Distal Interger	10	46244748	46244770	23	1	1.00E+08	uc029qxb.1	1086930	ENSMUSG000	Snord14d	small nucleolar RNA, C/D box 14D
chr10	47442045	47442204	160	161	47442122	23	9.86443	4.31371	6.70247	Adipose_R1_peak_2	Distal Interger	10	46244748	46244770	23	1	1.00E+08	uc029qxb.1	1197297	ENSMUSG000	Snord14d	small nucleolar RNA, C/D box 14D
chr10	47576948	47577079	132	133	47577008	21	8.43286	3.95423	5.34896	Adipose_R1_peak_2	Distal Interger	10	46244748	46244770	23	1	1.00E+08	uc029qxb.1	1332200	ENSMUSG000	Snord14d	small nucleolar RNA, C/D box 14D
chr10	47577613	47577893	281	282	47577742	71	58.27042	12.94113	54.20487	Adipose_R1_peak_2	Distal Interger	10	46244748	46244770	23	1	1.00E+08	uc029qxb.1	1332865	ENSMUSG000	Snord14d	small nucleolar RNA, C/D box 14D

chr10	47577630	47577880	251	252	47577759	61	28.12311	5.7175	24.28203	Adipose_R2_peak_2	Distal Intergen	10	46244748	46244770	23	1	1.00E+08	uc029qxb.1	1332882	ENSMUSG000	Snord14d	small nucleolar RNA, C/D box 14D
chr10	47611199	47611340	142	143	47611267	21	8.43286	3.95423	5.34896	Adipose_R1_peak_2	Distal Intergen	10	46244748	46244770	23	1	1.00E+08	uc029qxb.1	1366451	ENSMUSG000	Snord14d	small nucleolar RNA, C/D box 14D
chr10	47664440	47664718	279	280	47664584	69	55.87963	12.58165	51.83661	Adipose_R1_peak_2	Distal Intergen	10	46244748	46244770	23	1	1.00E+08	uc029qxb.1	1419692	ENSMUSG000	Snord14d	small nucleolar RNA, C/D box 14D
chr10	47757543	47757814	272	273	47757674	38	22.52591	7.00978	18.96	Adipose_R1_peak_2	Distal Intergen	10	49093681	49095843	2163	2	1.00E+08	uc029qxc.1	1338029	NA	C130030K03R	proteasome (prosome, macropain) subunit, alpha type 6 pseudogene
chr10	47757544	47757737	194	195	47757665	39	12.34187	3.68871	8.98501	Adipose_R2_peak_2	Distal Intergen	10	49093681	49095843	2163	2	1.00E+08	uc029qxc.1	1338106	NA	C130030K03R	proteasome (prosome, macropain) subunit, alpha type 6 pseudogene
chr10	47865319	47865542	224	225	47865441	51	20.44827	4.79532	16.78434	Adipose_R2_peak_2	Distal Intergen	10	49093681	49095843	2163	2	1.00E+08	uc029qxc.1	1230301	NA	C130030K03R	proteasome (prosome, macropain) subunit, alpha type 6 pseudogene
chr10	47865325	47865542	218	219	47865439	34	18.86162	6.29083	15.38482	Adipose_R1_peak_2	Distal Intergen	10	49093681	49095843	2163	2	1.00E+08	uc029qxc.1	1230301	NA	C130030K03R	proteasome (prosome, macropain) subunit, alpha type 6 pseudogene
chr10	47876433	47876790	358	359	47876562	27	12.93148	5.03266	9.63827	Adipose_R1_peak_2	Distal Intergen	10	49093681	49095843	2163	2	1.00E+08	uc029qxc.1	1219053	NA	C130030K03R	proteasome (prosome, macropain) subunit, alpha type 6 pseudogene
chr10	47877438	47877583	146	147	47877483	26	5.31252	2.48988	2.47999	Adipose_R2_peak_2	Distal Intergen	10	49093681	49095843	2163	2	1.00E+08	uc029qxc.1	1218260	NA	C130030K03R	proteasome (prosome, macropain) subunit, alpha type 6 pseudogene
chr10	47877443	47877588	146	147	47877507	17	5.80341	3.23528	2.92378	Adipose_R1_peak_2	Distal Intergen	10	49093681	49095843	2163	2	1.00E+08	uc029qxc.1	1218255	NA	C130030K03R	proteasome (prosome, macropain) subunit, alpha type 6 pseudogene
chr10	47914549	47914722	174	175	47914691	23	9.86443	4.31371	6.70247	Adipose_R1_peak_2	Distal Intergen	10	49093681	49095843	2163	2	1.00E+08	uc029qxc.1	1181121	NA	C130030K03R	proteasome (prosome, macropain) subunit, alpha type 6 pseudogene
chr10	48175438	48175591	154	155	48175505	22	9.13959	4.13397	6.01526	Adipose_R1_peak_2	Distal Intergen	10	49093681	49095843	2163	2	1.00E+08	uc029qxc.1	920252	NA	C130030K03R	proteasome (prosome, macropain) subunit, alpha type 6 pseudogene
chr10	48203298	48203448	151	152	48203362	25	11.36566	4.67319	8.13482	Adipose_R1_peak_2	Distal Intergen	10	49093681	49095843	2163	2	1.00E+08	uc029qxc.1	892395	NA	C130030K03R	proteasome (prosome, macropain) subunit, alpha type 6 pseudogene
chr10	48232715	48233020	306	307	48232873	101	91.48549	16.54994	87.19329	Adipose_R1_peak_2	Distal Intergen	10	49093681	49095843	2163	2	1.00E+08	uc029qxc.1	862823	NA	C130030K03R	proteasome (prosome, macropain) subunit, alpha type 6 pseudogene
chr10	48232732	48233019	288	289	48232874	89	53.01237	8.2996	48.82483	Adipose_R2_peak_2	Distal Intergen	10	49093681	49095843	2163	2	1.00E+08	uc029qxc.1	862824	NA	C130030K03R	proteasome (prosome, macropain) subunit, alpha type 6 pseudogene
chr10	48235017	48235217	201	202	48235127	31	16.23981	5.75161	12.83549	Adipose_R1_peak_2	Distal Intergen	10	49093681	49095843	2163	2	1.00E+08	uc029qxc.1	860626	NA	C130030K03R	proteasome (prosome, macropain) subunit, alpha type 6 pseudogene
chr10	48293351	48293576	226	227	48293439	31	16.23981	5.75161	12.83549	Adipose_R1_peak_2	Distal Intergen	10	49093681	49095843	2163	2	1.00E+08	uc029qxc.1	802267	NA	C130030K03R	proteasome (prosome, macropain) subunit, alpha type 6 pseudogene
chr10	48293380	48293509	130	131	48293443	30	7.24477	2.85875	4.21917	Adipose_R2_peak_2	Distal Intergen	10	49093681	49095843	2163	2	1.00E+08	uc029qxc.1	802334	NA	C130030K03R	proteasome (prosome, macropain) subunit, alpha type 6 pseudogene
chr10	48312046	48312335	290	291	48312196	48	32.41826	8.80716	28.66523	Adipose_R1_peak_2	Distal Intergen	10	49093681	49095843	2163	2	1.00E+08	uc029qxc.1	783508	NA	C130030K03R	proteasome (prosome, macropain) subunit, alpha type 6 pseudogene
chr10	48312062	48312305	244	245	48312173	40	12.96465	3.78093	9.57654	Adipose_R2_peak_2	Distal Intergen	10	49093681	49095843	2163	2	1.00E+08	uc029qxc.1	783538	NA	C130030K03R	proteasome (prosome, macropain) subunit, alpha type 6 pseudogene
chr10	48383150	48383426	277	278	48383283	57	42.07807	10.4248	38.18469	Adipose_R1_peak_2	Distal Intergen	10	49093681	49095843	2163	2	1.00E+08	uc029qxc.1	712417	NA	C130030K03R	proteasome (prosome, macropain) subunit, alpha type 6 pseudogene
chr10	48383163	48383421	259	260	48383295	64	30.56565	5.99415	26.6807	Adipose_R2_peak_2	Distal Intergen	10	49093681	49095843	2163	2	1.00E+08	uc029qxc.1	712422	NA	C130030K03R	proteasome (prosome, macropain) subunit, alpha type 6 pseudogene
chr10	48383658	48383855	198	199	48383784	23	9.86443	4.31371	6.70247	Adipose_R1_peak_2	Distal Intergen	10	49093681	49095843	2163	2	1.00E+08	uc029qxc.1	711988	NA	C130030K03R	proteasome (prosome, macropain) subunit, alpha type 6 pseudogene
chr10	48383718	48383849	132	133	48383806	29	6.74062	2.76653	3.75989	Adipose_R2_peak_2	Distal Intergen	10	49093681	49095843	2163	2	1.00E+08	uc029qxc.1	711994	NA	C130030K03R	proteasome (prosome, macropain) subunit, alpha type 6 pseudogene
chr10	48385598	48385804	207	208	48385690	28	13.73721	5.2124	10.41463	Adipose_R1_peak_2	Distal Intergen	10	49093681	49095843	2163	2	1.00E+08	uc029qxc.1	710039	NA	C130030K03R	proteasome (prosome, macropain) subunit, alpha type 6 pseudogene
chr10	48420928	48421151	224	225	48421043	26	12.14079	4.85292	8.87838	Adipose_R1_peak_2	Distal Intergen	10	49093681	49095843	2163	2	1.00E+08	uc029qxc.1	674692	NA	C130030K03R	proteasome (prosome, macropain) subunit, alpha type 6 pseudogene
chr10	48420977	48421092	116	117	48421041	29	6.74062	2.76653	3.75989	Adipose_R2_peak_2	Distal Intergen	10	49093681	49095843	2163	2	1.00E+08	uc029qxc.1	674751	NA	C130030K03R	proteasome (prosome, macropain) subunit, alpha type 6 pseudogene
chr10	48534205	48534461	257	258	48534347	49	19.00719	4.61089	15.38598	Adipose_R2_peak_2	Distal Intergen	10	49093681	49095843	2163	2	1.00E+08	uc029qxc.1	561382	NA	C130030K03R	proteasome (prosome, macropain) subunit, alpha type 6 pseudogene
chr10	48534211	48534432	222	223	48534312	25	11.36566	4.67319	8.13482	Adipose_R1_peak_2	Distal Intergen	10	49093681	49095843	2163	2	1.00E+08	uc029qxc.1	561411	NA	C130030K03R	proteasome (prosome, macropain) subunit, alpha type 6 pseudogene
chr10	48611959	48612167	209	210	48612123	24	10.60668	4.49345	7.40961	Adipose_R1_peak_2	Distal Intergen	10	49093681	49095843	2163	2	1.00E+08	uc029qxc.1	483676	NA	C130030K03R	proteasome (prosome, macropain) subunit, alpha type 6 pseudogene
chr10	48678321	48678521	201	202	48678415	23	9.86443	4.31371	6.70247	Adipose_R1_peak_2	Distal Intergen	10	49093681	49095843	2163	2	1.00E+08	uc029qxc.1	417322	NA	C130030K03R	proteasome (prosome, macropain) subunit, alpha type 6 pseudogene
chr10	48678821	48679111	291	292	48678970	46	30.36364	8.44768	26.64488	Adipose_R1_peak_2	Distal Intergen	10	49093681	49095843	2163	2	1.00E+08	uc029qxc.1	416732	NA	C130030K03R	proteasome (prosome, macropain) subunit, alpha type 6 pseudogene
chr10	48678831	48679061	231	232	48678963	48	18.29934	4.51867	14.70076	Adipose_R2_peak_2	Distal Intergen	10	49093681	49095843	2163	2	1.00E+08	uc029qxc.1	416782	NA	C130030K03R	proteasome (prosome, macropain) subunit, alpha type 6 pseudogene
chr10	48679560	48679775	216	217	48679674	23	9.86443	4.31371	6.70247	Adipose_R1_peak_2	Distal Intergen	10	49093681	49095843	2163	2	1.00E+08	uc029qxc.1	416068	NA	C130030K03R	proteasome (prosome, macropain) subunit, alpha type 6 pseudogene
chr10	48679606	48679750	145	146	48679679	32	8.29282	3.04319	5.18352	Adipose_R2_peak_2	Distal Intergen	10	49093681	49095843	2163	2	1.00E+08	uc029qxc.1	416093	NA	C130030K03R	proteasome (prosome, macropain) subunit, alpha type 6 pseudogene
chr10	48682478	48682787	310	311	48682619	85	75.64196	15.45746	71.43914	Adipose_R1_peak_2	Distal Intergen	10	49093681	49095843	2163	2	1.00E+08	uc029qxc.1	413056	NA	C130030K03R	proteasome (prosome, macropain) subunit, alpha type 6 pseudogene
chr10	48682491	48682744	254	255	48682624	60	27.32259	5.62528	23.49644	Adipose_R2_peak_2	Distal Intergen	10	49093681	49095843	2163	2	1.00E+08	uc029qxc.1	413099	NA	C130030K03R	proteasome (prosome, macropain) subunit, alpha type 6 pseudogene
chr10	48683027	48683253	227	228	48683132	28	13.73721	5.2124	10.41463	Adipose_R1_peak_2	Distal Intergen	10	49093681	49095843	2163	2	1.00E+08	uc029qxc.1	412590	NA	C130030K03R	proteasome (prosome, macropain) subunit, alpha type 6 pseudogene
chr10	48751347	48751469	123	124	48751413	28	6.25023	2.67431	3.31626	Adipose_R2_peak_2	Distal Intergen	10	49093681	49095843	2163	2	1.00E+08	uc029qxc.1	344374	NA	C130030K03R	proteasome (prosome, macropain) subunit, alpha type 6 pseudogene
chr10	48992417	48992634	218	219	48992513	22	9.13959	4.13397	6.01526	Adipose_R1_peak_2	Distal Intergen	10	49093681	49095843	2163	2	1.00E+08	uc029qxc.1	103209	NA	C130030K03R	proteasome (prosome, macropain) subunit, alpha type 6 pseudogene
chr10	48992477	48992646	170	171	48992589	30	7.24477	2.85875	4.21917	Adipose_R2_peak_2	Distal Intergen	10	49093681	49095843	2163	2	1.00E+08	uc029qxc.1	103197	NA	C130030K03R	proteasome (prosome, macropain) subunit, alpha type 6 pseudogene
chr10	49000420	49000679	260	261	49000556	39	23.47001	7.18952	19.88325	Adipose_R1_peak_2	Distal Intergen	10	49093681	49095843	2163	2	1.00E+08	uc029qxc.1	95164	NA	C130030K03R	proteasome (prosome, macropain) subunit, alpha type 6 pseudogene
chr10	49000425	49000664	240	241	49000554	44	15.55653	4.1498	12.05625	Adipose_R2_peak_2	Distal Intergen	10	49093681	49095843	2163	2	1.00E+08	uc029qxc.1	95179	NA	C130030K03R	proteasome (prosome, macropain) subunit, alpha type 6 pseudogene
chr10	49119915	49120311	397	398	49119978	21	8.43286	3.95423	5.34896	Adipose_R1_peak_2	Intron (uc007f	10	49093681	49095843	2163	2	1.00E+08	uc029qxc.1	-24072	NA	C130030K03R	proteasome (prosome, macropain) subunit, alpha type 6 pseudogene
chr10	49229218	49229487	270	271	49229348	50	34.50784	9.16663	30.72138	Adipose_R1_peak_2	Intron (uc007f	10	49093681	49095843	2163	2	1.00E+08	uc029qxc.1	-133375	NA	C130030K03R	proteasome (prosome, macropain) subunit, alpha type 6 pseudogene
chr10	49229267	49229431	165	166	49229354	34	9.39139	3.22762	6.20474	Adipose_R2_peak_2	Intron (uc007f	10	49093681	49095843	2163	2	1.00E+08	uc029qxc.1	-133424	NA	C130030K03R	proteasome (prosome, macropain) subunit, alpha type 6 pseudogene
chr10	49278787	49278993	207	208	49278882	25	11.36566	4.67319	8.13482	Adipose_R1_peak_2	Intron (uc007f	10	49093681	49095843	2163	2	1.00E+08	uc029qxc.1	-182944	NA	C130030K03R	proteasome (prosome, macropain) subunit, alpha type 6 pseudogene
chr10	49763983	49764143	161	162	49764132	36	10.5378	3.41206	7.27949	Adipose_R2_peak_2	Intron (uc007f	10	49099463	49788754	689292	2	14806	uc007fag.2	24611	ENSMUSG000	Grik2	glutamate receptor, ionotropic, kainate 2 (beta 2)
chr10	49763996	49764142	147	148	49764127	18	6.42934	3.41502	3.48999	Adipose_R1_peak_2	Intron (uc007f	10	49099463	49788754	689292	2	14806	uc007fag.2	24612	ENSMUSG000	Grik2	glutamate receptor, ionotropic, kainate 2 (beta 2)
chr10	49787921	49788018	98	99	49787960	26	5.31252	2.48988	2.47999	Adipose_R2_peak_2	Promoter (<=1	10	49099463	49788754	689292	2	14806	uc007fag.2	736	ENSMUSG000	Grik2	glutamate receptor, ionotropic, kainate 2 (beta 2)
chr10	49841472	49841629	158	159	49841559	23	9.86443	4.31371	6.70247	Adipose_R1_peak_2	Distal Intergen	10	49099463	49788754	689292	2	14806	uc007fag.2	-52718	ENSMUSG000	Grik2	glutamate receptor, ionotropic, kainate 2 (beta 2)
chr10	49968389	49968635	247	248	49968531	47	31.38649	8.62742	27.65048	Adipose_R1_peak_2	Distal Intergen	10	49099463	49788754	689292	2	14806	uc007fag.2	-179635	ENSMUSG000	Grik2	glutamate receptor, ionotropic, kainate 2 (beta 2)
chr10	49968419	49968619	201	202	49968547	40	12.96465	3.78093	9.57654	Adipose_R2_peak_2	Distal Intergen	10	49099463	49788754	689292	2	14806	uc007fag.2	-179665	ENSMUSG000	Grik2	glutamate receptor, ionotropic, kainate 2 (beta 2)
chr10	49969874	49970195	322	323	49970020	118	115.7292	19.8358	111.3302	Adipose_R1_peak_2	Distal Intergen	10	49099463	49788754	689292	2	14806	uc007fag.2	-181120	ENSMUSG000	Grik2	glutamate receptor, ionotropic, kainate 2 (beta 2)
chr10	49969885	49970170	286	287	49970026	97	60.87369	9.03734	56.60858	Adipose_R2_peak_2	Distal Intergen	10	49099463	49788754	689292	2	14806	uc007fag.2	-181131	ENSMUSG000	Grik2	glutamate receptor, ionotropic, kainate 2 (beta 2)
chr10	49971076	49971220	145	146	49971137	26	12.14079	4.85292	8.87838	Adipose_R1_peak_2	Distal Intergen	10	49099463	49788754	689292	2	14806	uc007fag.2	-182322	ENSMUSG000	Grik2	glutamate receptor, ionotropic, kainate 2 (beta 2)
chr10	50029534	50029810	277	278	50029661	50	34.50784	9.16663	30.72138	Adipose_R1_peak_2	Distal Intergen	10	49099463	49788754	689292	2	14806	uc007fag.2	-240780	ENSMUSG000	Grik2	glutamate receptor, ionotropic, kainate 2 (beta 2)
chr10	50029550	50029774	225	226	50029648	43	14.8939	4.05758	11.42017	Adipose_R2_peak_2	Distal Intergen	10	49099463	49788754	689292	2	14806	uc007fag.2	-240796	ENSMUSG000	Grik2	glutamate receptor, ionotropic, kainate 2 (beta 2)
chr10	50031372	50031636	265	266	50031514	44	28.34544	8.08821	24.66239	Adipose_R1_peak_2	Distal Intergen	10	49099463	49788754	689292	2	14806	uc007fag.2	-242618	ENSMUSG000	Grik2	glutamate receptor, ionotropic, kainate 2 (beta 2)
chr10	50031396	50031611	216	217	50031499	48	18.29934	4.51867	14.70076	Adipose_R2_peak_2	Distal Intergen	10	49099463	49788754	689292	2	14806	uc007fag.2	-242642	ENSMUSG000	Grik2	glutamate receptor, ionotropic, kainate 2 (beta 2)
chr10	50031794	50031945	152	153	50031890	21	8.43286	3.95423	5.34896	Adipose_R1_peak_2	Distal Intergen	10	49099463	49788754	689292	2	14806	uc007fag.2	-243040	ENSMUSG000	Grik2	glutamate receptor, ionotropic, kainate 2 (beta 2)
chr10	50033577	50033801	225	226	50033689	38	22.52591	7.00978	18.96	Adipose_R1_peak_2	Distal Intergen	10	49099463	49788754	689292	2	14806	uc007fag.2	-244823	ENSMUSG000	Grik2	glutamate receptor, ionotropic, kainate 2 (beta 2)
chr10	50033588	50033752	165	166	50033676	36	10.5378	3.41206	7.27949	Adipose_R2_peak_2	Distal Intergen	10	49099463	49788754	689292	2	14806	uc007fag.2	-244834	ENSMUSG000	Grik2	glutamate receptor, ionotropic, kainate 2 (beta 2)
chr10	50110273	50110460	188	189	50110346	26	12.14079	4.85292	8.87838	Adipose_R1_peak_2	Distal Intergen	10	49099463	49788754	689292	2	14806	uc007fag.2	-321519	ENSMUSG000	Grik2	glutamate receptor, ionotropic, kainate 2 (beta 2)
chr10	50178478	50178946	469	470	50178805	159	181.1528	28.75806	176.5634	Adipose_R1_peak_2	Distal Intergen	10	49099463	49788754	689292	2	14806	uc007fag.2	-389724	ENSMUSG000	Grik2	glutamate receptor, ionotropic, kainate 2 (beta 2)
chr10	50178500	50178942	443	444	50178807	154	123.9861	14.29375	119.3959	Adipose_R2_peak_2	Distal Intergen	10	49099463	49788754	689292	2	14806	uc007fag.2	-389746	ENSMUSG000	Grik2	glutamate receptor, ionotropic, kainate 2 (beta 2)
chr10	50184456	50184711	256	257	50184589	44	28.34544	8.08821	24.66239	Adipose_R1_peak_2	Distal Intergen	10	49099463	49788754	689292	2	14806	uc007fag.2	-395702	ENSMUSG000	Grik2	glutamate receptor, ionotropic, kainate 2 (beta 2)
chr10	50184465	50184713	249	250	50184585	56	24.19188	5.25641	20.43163	Adipose_R2_peak_2	Distal Intergen	10	49099463	49788754	689292	2	14806	uc007fag.2	-395711	ENSMUSG000	Grik2	glutamate receptor, ionotropic, kainate 2 (beta 2)
chr10	50254111	50254333	223	224	50254214	34	18.86162	6.29083	15.38482	Adipose_R1_peak_2	Distal Intergen	10	50592669	50851202	258534	1	77987	uc007fak.2	-338336	ENSMUSG000	Ascc3	activating signal cointegrator 1 complex subunit 3
chr10	50257354	50257568	215	216	50257438	33	17.97508	6.11109	14.52164	Adipose_R1_peak_2	Distal Intergen	10	50592669	50851202	258534	1	77987	uc007fak.2	-335101	ENSMUSG000	Ascc3	activating signal cointegrator 1 complex subunit 3
chr10	50257389	50257502	114	115	50257436	31	7.7623	2.95097	4.69388	Adipose_R2_peak_2	Distal Intergen	10	50592669	50851202	258534	1	77987	uc007fak.2	-335167	ENSMUSG000	Ascc3	activating signal cointegrator 1 complex subunit 3
chr10	50301057	50301232	176	177	50301148	25	11.36566	4.67319	8.13482	Adipose_R1_peak_2	Distal Intergen	10	50592669	50851202	258534	1	77987	uc007fak.2	-291437	ENSMUSG000	Ascc3	activating signal cointegrator 1 complex subunit 3
chr10	50549516	50549681	166	167	50549617	20	7.74501	3.7745	4.70467	Adipose_R1_peak_2	Distal Intergen	10	50592669	50851202	258534	1	77987	uc007fak.2	-42988	ENSMUSG000	Ascc3	activating signal cointegrator 1 complex subunit 3
chr10	50565819	50565951	133	134	50565904	17	5.80341	3.23528	2.92378	Adipose_R1_peak_2	Distal Intergen	10	50592669	50851202	258534	1	77987	uc007fak.2	-26718	ENSMUSG000	Ascc3	activating signal cointegrator 1 complex subunit 3
chr10	50675523	50675735	213	214	50675611	29	14.55748	5.39214	11.20652	Adipose_R1_peak_2	Intron (uc007f	10	50592669	50851202	258534	1	77987	uc007fak.2	82854	ENSMUSG000	Ascc3	activating signal cointegrator 1 complex subunit 3
chr10	50675564	50675714	151	152	50675618	31	7.7623	2.95097	4.69388	Adipose_R2_peak_2	Intron (uc007f	10	50592669	50851202	258534	1	77987	uc007fak.2	82895	ENSMUSG000	Ascc3	activating signal cointegrator 1 complex subunit 3
chr10	51437883	51438049	167	168	51437947	18	6.42934	3.41502	3.48999	Adipose_R1_peak_2	Distal Intergen	10	51480612	51485780	5169	1	14727	uc007fan.2	-42563	ENSMUSG000	Lilr4b	leukocyte immunoglobulin-like receptor, subfamily B, member 4B
chr10	51438382	51438504	123	124	51438445	30	7.24477	2.85875	4.21917	Adipose_R2_peak_2	Distal Intergen	10	51480612	51485780	5169	1	14727	uc007fan.2	-42108	ENSMUSG000	Lilr4b	leukocyte immunoglobulin-like receptor, subfamily B, member 4B
chr10	51438952	51439376	425	426	51439080	29	9.47571	3.58168	6.34846	Adipose_R1_peak_2	Distal Intergen	10	51480612	51485780	5169	1	14727	uc007fan.2	-41236	ENSMUSG000	Lilr4b	leukocyte immunoglobulin-like receptor, subfamily B, member 4B
chr10	51438994	51439340	347	348	51439074	36	10.5378	3.41206	7.27949	Adipose_R2_peak_2	Distal Intergen	10	51480612	51485780	5169	1	14727	uc007fan.2	-41272	ENSMUSG000	Lilr4b	leukocyte immunoglobulin-like receptor, subfamily B, member 4B
chr10	51445274	51445391	118	119	51445340	27	5.77405	2.5821	2.88921	Adipose_R2_peak_2	Distal Intergen	10	51480612	51485780	5169	1	14727	uc007fan.2	-35221	ENSMUSG000	Lilr4b	leukocyte immunoglobulin-like receptor, subfamily B, member 4B
chr10	51577742	51577953	212	213	51577853	32	17.10103	5.93135	13.67137	Adipose_R1_peak_2	Distal Intergen	10	51585450	51590460	5011	2	77296	uc007fat.2	12507	ENSMUSG000	Fam162b	family with sequence similarity 162, member B
chr10	51577777	51577924	148	149	51577854	33	8.83596	3.1354	5.68652	Adipose_R2_peak_2	Distal Intergen	10	51585450	51590460	5011	2	77296	uc007fat.2	12536	ENSMUSG000	Fam162b	family with sequence similarity 162, member B
chr10	51580409	51580724	316	317	51580578	48	32.41826	8.80716	28.66523	Adipose_R1_peak_2	Distal Intergen	10	51585450	51590460	5011	2	77296	uc007fat.2	9736	ENSMUSG000	Fam162b	family with sequence similarity 162, member B
chr10	51580435	51580660	226	227	51580558	33	8.83596	3.1354	5.68652	Adipose_R2_peak_2	Distal Intergen	10	51585450	51590460	5011	2	77296	uc007fat.2	9800	ENSMUSG000	Fam162b	family with sequence similarity 162, member B
chr10	51581576	51581781	206	207	51581694	30	15.39182	5.57188	12.01394	Adipose_R1_peak_2	Distal Intergen	10	51585450	51590460	5011	2	77296	uc007fat.2	8679	ENSMUSG000	Fam162b	family with sequence similarity 162, member B
chr10	51921018	51921175	158	159	51921095	22	9.13959	4.13397	6.01526	Adipose_R1_peak_2	Distal Intergen	10	52022502	52028471	5970	1	215031	uc007faz.2	-101327	ENSMUSG000	Vgll2	vestigial like family member 2
chr10	51985695	51985911	217	218	51985794	31	16.23981	5.75161	12.83549	Adipose_R1_peak_2	Distal Intergen	10	52022502	52028471	5970	1	215031	uc007faz.2	-36591	ENSMUSG000	Vgll2	vestigial like family member 2
chr10	51985732	51985897	166	167	51985793	32	8.29282	3.04319	5.18352	Adipose_R2_peak_2	Distal Intergen	10	52022502	52028471	5970	1	215031	uc007faz.2	-36605	ENSMUSG000	Vgll2	vestigial like family member 2
chr10	51988787	51989071	285	286	51988918	48	32.41826	8.80716	28.66523	Adipose_R1_peak_2	Distal Intergen	10	52022502	52028471	5970	1	215031	uc007faz.2	-33431	ENSMUSG000	Vgll2	vestigial like family member 2
chr10	51988805	51989024	220	221	51988915	44	15.55653	4.1498	12.05625	Adipose_R2_peak_2	Distal Intergen	10	52022502	52028471	5970	1	215031	uc007faz.2	-33478	ENSMUSG000	Vgll2	vestigial like family member 2
chr10	52054418	52054562	145	146	52054491	23	9.86443	4.31371	6.70247	Adipose_R1_peak_2	Intron (uc007f	10	52022502	52028471	5970	1	215031	uc007faz.2	31916	ENSMUSG000	Vgll2	vestigial like family member 2
chr10	52054426	52054548	123	124	52054496	33	8.83596	3.1354	5.68652	Adipose_R2_peak_2	Intron (uc007f	10	52022502	52028471	5970	1	215031	uc007faz.2	31924	ENSMUSG000	Vgll2	vestigial like family member 2
chr10	52114664	52114860	197	198	52114752	26	12.14079	4.85292	8.87838	Adipose_R1_peak_2	Intron (uc007f	10	52045926	52195244	149319	2	19886	uc007fbb.1	80384	ENSMUSG000	Ros1	Ros1 proto-oncogene

chr10	52116619	52116951	333	334	52116753	38	22.52591	7.00978	18.96	Adipose_R1_peak_2	Intron (uc007f	10	52045926	52195244	149319	2	19886	uc007fbb.1	78293	ENSMUSG000	Ros1	Ros1 proto-oncogene
chr10	52116674	52116911	238	239	52116783	38	11.72962	3.59649	8.4047	Adipose_R2_peak_2	Intron (uc007f	10	52045926	52195244	149319	2	19886	uc007fbb.1	78333	ENSMUSG000	Ros1	Ros1 proto-oncogene
chr10	52138049	52138230	182	183	52138159	23	9.86443	4.31371	6.70247	Adipose_R1_peak_2	Intron (uc007f	10	52045926	52195244	149319	2	19886	uc007fbb.1	57014	ENSMUSG000	Ros1	Ros1 proto-oncogene
chr10	52500108	52500313	206	207	52500178	24	10.60668	4.49345	7.40961	Adipose_R1_peak_2	Distal Interger	10	52542320	52545739	3420	2	22639	uc029qxe.1	45426	ENSMUSG000	Zfa-ps	zinc finger protein, autosomal, pseudogene
chr10	52522218	52522402	185	186	52522344	20	7.74501	3.7745	4.70467	Adipose_R1_peak_2	Distal Interger	10	52542320	52545739	3420	2	22639	uc029qxe.1	23337	ENSMUSG000	Zfa-ps	zinc finger protein, autosomal, pseudogene
chr10	52569312	52569540	229	230	52569420	39	12.34187	3.68871	8.98501	Adipose_R2_peak_2	Distal Interger	10	52542320	52545739	3420	2	22639	uc029qxe.1	-23573	ENSMUSG000	Zfa-ps	zinc finger protein, autosomal, pseudogene
chr10	52569344	52569552	209	210	52569436	31	16.23981	5.75161	12.83549	Adipose_R1_peak_2	Distal Interger	10	52542320	52545739	3420	2	22639	uc029qxe.1	-23605	ENSMUSG000	Zfa-ps	zinc finger protein, autosomal, pseudogene
chr10	54145192	54145486	295	296	54145335	52	36.63102	9.52611	32.81188	Adipose_R1_peak_2	Distal Interger	10	54075922	54081081	5160	1	1.01E+08	uc007fbz.1	69270	ENSMUSG000	Gm16998	predicted gene, 16998
chr10	54145202	54145475	274	275	54145348	71	36.49133	6.63968	32.5091	Adipose_R2_peak_2	Distal Interger	10	54075922	54081081	5160	1	1.01E+08	uc007fbz.1	69280	ENSMUSG000	Gm16998	predicted gene, 16998
chr10	54223344	54223501	158	159	54223429	26	12.14079	4.85292	8.87838	Adipose_R1_peak_2	Distal Interger	10	54075922	54081081	5160	1	1.01E+08	uc007fbz.1	147422	ENSMUSG000	Gm16998	predicted gene, 16998
chr10	54591490	54591714	225	226	54591601	50	19.72356	4.7031	16.08069	Adipose_R2_peak_2	Distal Interger	10	54075922	54081081	5160	1	1.01E+08	uc007fbz.1	515568	ENSMUSG000	Gm16998	predicted gene, 16998
chr10	54591504	54591700	197	198	54591602	28	13.73721	5.2124	10.41463	Adipose_R1_peak_2	Distal Interger	10	54075922	54081081	5160	1	1.01E+08	uc007fbz.1	515582	ENSMUSG000	Gm16998	predicted gene, 16998
chr10	54618361	54618532	172	173	54618446	21	8.43286	3.95423	5.34896	Adipose_R1_peak_2	Distal Interger	10	54075922	54081081	5160	1	1.01E+08	uc007fbz.1	542439	ENSMUSG000	Gm16998	predicted gene, 16998
chr10	54619196	54619335	140	141	54619333	36	10.5378	3.41206	7.27949	Adipose_R2_peak_2	Distal Interger	10	54075922	54081081	5160	1	1.01E+08	uc007fbz.1	543274	ENSMUSG000	Gm16998	predicted gene, 16998
chr10	54696843	54697103	261	262	54696962	36	20.67077	6.6503	17.14779	Adipose_R1_peak_2	Distal Interger	10	54075922	54081081	5160	1	1.01E+08	uc007fbz.1	620921	ENSMUSG000	Gm16998	predicted gene, 16998
chr10	54696875	54697054	180	181	54696982	36	10.5378	3.41206	7.27949	Adipose_R2_peak_2	Distal Interger	10	54075922	54081081	5160	1	1.01E+08	uc007fbz.1	620953	ENSMUSG000	Gm16998	predicted gene, 16998
chr10	55119584	55119896	313	314	55119761	63	48.85777	11.50323	44.88581	Adipose_R1_peak_2	Distal Interger	10	56106917	56116880	9964	1	73390	uc007fcb.1	-987021	ENSMUSG000	Msl3l2	male-specific lethal 3-like 2 (Drosophila)
chr10	55119628	55119842	215	216	55119759	46	16.90986	4.33423	13.35898	Adipose_R2_peak_2	Distal Interger	10	56106917	56116880	9964	1	73390	uc007fcb.1	-987075	ENSMUSG000	Msl3l2	male-specific lethal 3-like 2 (Drosophila)
chr10	55140217	55140569	353	354	55140377	41	13.59774	3.87315	10.1803	Adipose_R2_peak_2	Distal Interger	10	56106917	56116880	9964	1	73390	uc007fcb.1	-966348	ENSMUSG000	Msl3l2	male-specific lethal 3-like 2 (Drosophila)
chr10	55140218	55140533	316	317	55140336	29	14.55748	5.39214	11.20652	Adipose_R1_peak_2	Distal Interger	10	56106917	56116880	9964	1	73390	uc007fcb.1	-966384	ENSMUSG000	Msl3l2	male-specific lethal 3-like 2 (Drosophila)
chr10	55248461	55248672	212	213	55248564	35	19.76029	6.47056	16.26028	Adipose_R1_peak_2	Distal Interger	10	56106917	56116880	9964	1	73390	uc007fcb.1	-858245	ENSMUSG000	Msl3l2	male-specific lethal 3-like 2 (Drosophila)
chr10	55248467	55248659	193	194	55248580	43	14.8939	4.05758	11.42017	Adipose_R2_peak_2	Distal Interger	10	56106917	56116880	9964	1	73390	uc007fcb.1	-858258	ENSMUSG000	Msl3l2	male-specific lethal 3-like 2 (Drosophila)
chr10	55500941	55501207	267	268	55501075	41	25.38991	7.54899	21.76305	Adipose_R1_peak_2	Distal Interger	10	56106917	56116880	9964	1	73390	uc007fcb.1	-605710	ENSMUSG000	Msl3l2	male-specific lethal 3-like 2 (Drosophila)
chr10	55500963	55501168	206	207	55501064	44	15.55653	4.1498	12.05625	Adipose_R2_peak_2	Distal Interger	10	56106917	56116880	9964	1	73390	uc007fcb.1	-605749	ENSMUSG000	Msl3l2	male-specific lethal 3-like 2 (Drosophila)
chr10	55543185	55543536	352	353	55543348	364	335.5093	20.13394	330.7236	Adipose_R1_peak_2	Distal Interger	10	56106917	56116880	9964	1	73390	uc007fcb.1	-563381	ENSMUSG000	Msl3l2	male-specific lethal 3-like 2 (Drosophila)
chr10	55543212	55543521	310	311	55543351	341	315.7294	20.22972	310.8435	Adipose_R2_peak_2	Distal Interger	10	56106917	56116880	9964	1	73390	uc007fcb.1	-563396	ENSMUSG000	Msl3l2	male-specific lethal 3-like 2 (Drosophila)
chr10	55580781	55581074	294	295	55580952	54	38.78658	9.88558	34.93639	Adipose_R1_peak_2	Distal Interger	10	56106917	56116880	9964	1	73390	uc007fcb.1	-525843	ENSMUSG000	Msl3l2	male-specific lethal 3-like 2 (Drosophila)
chr10	55580848	55581015	168	169	55580962	36	10.5378	3.41206	7.27949	Adipose_R2_peak_2	Distal Interger	10	56106917	56116880	9964	1	73390	uc007fcb.1	-525902	ENSMUSG000	Msl3l2	male-specific lethal 3-like 2 (Drosophila)
chr10	55734711	55734944	234	235	55734861	20	7.74501	3.7745	4.70467	Adipose_R1_peak_2	Distal Interger	10	56106917	56116880	9964	1	73390	uc007fcb.1	-371973	ENSMUSG000	Msl3l2	male-specific lethal 3-like 2 (Drosophila)
chr10	55736475	55736654	180	181	55736538	24	10.60668	4.49345	7.40961	Adipose_R1_peak_2	Distal Interger	10	56106917	56116880	9964	1	73390	uc007fcb.1	-370263	ENSMUSG000	Msl3l2	male-specific lethal 3-like 2 (Drosophila)
chr10	55738619	55738787	169	170	55738689	22	9.13959	4.13397	6.01526	Adipose_R1_peak_2	Distal Interger	10	56106917	56116880	9964	1	73390	uc007fcb.1	-368130	ENSMUSG000	Msl3l2	male-specific lethal 3-like 2 (Drosophila)
chr10	55842542	55842756	215	216	55842606	30	15.39182	5.57188	12.01394	Adipose_R1_peak_2	Distal Interger	10	56106917	56116880	9964	1	73390	uc007fcb.1	-264161	ENSMUSG000	Msl3l2	male-specific lethal 3-like 2 (Drosophila)
chr10	55900152	55900324	173	174	55900222	23	9.86443	4.31371	6.70247	Adipose_R1_peak_2	Distal Interger	10	56106917	56116880	9964	1	73390	uc007fcb.1	-206593	ENSMUSG000	Msl3l2	male-specific lethal 3-like 2 (Drosophila)
chr10	56031150	56031321	172	173	56031250	19	7.07687	3.59476	4.08458	Adipose_R1_peak_2	Intron (uc007f	10	56106917	56116880	9964	1	73390	uc007fcb.1	-75596	ENSMUSG000	Msl3l2	male-specific lethal 3-like 2 (Drosophila)
chr10	56036751	56036887	137	138	56036837	19	7.07687	3.59476	4.08458	Adipose_R1_peak_2	Intron (uc007f	10	56106917	56116880	9964	1	73390	uc007fcb.1	-70030	ENSMUSG000	Msl3l2	male-specific lethal 3-like 2 (Drosophila)
chr10	56142204	56142355	152	153	56142291	22	9.13959	4.13397	6.01526	Adipose_R1_peak_2	Intron (uc007f	10	56106917	56116880	9964	1	73390	uc007fcb.1	35287	ENSMUSG000	Msl3l2	male-specific lethal 3-like 2 (Drosophila)
chr10	56250852	56251250	399	400	56251103	67	53.51345	12.22218	49.49424	Adipose_R1_peak_2	Distal Interger	10	56014294	56228689	214396	2	544696	uc007fca.1	-22163	ENSMUSG000	Tbc1d32	TBC1 domain family, member 32
chr10	56250923	56251187	265	266	56251095	43	14.8939	4.05758	11.42017	Adipose_R2_peak_2	Distal Interger	10	56014294	56228689	214396	2	544696	uc007fca.1	-22234	ENSMUSG000	Tbc1d32	TBC1 domain family, member 32
chr10	56288452	56288696	245	246	56288536	27	12.93148	5.03266	9.63827	Adipose_R1_peak_2	Distal Interger	10	56014294	56228689	214396	2	544696	uc007fca.1	-59763	ENSMUSG000	Tbc1d32	TBC1 domain family, member 32
chr10	56701552	56701820	269	270	56701683	44	28.34544	8.08821	24.66239	Adipose_R1_peak_2	Distal Interger	10	56379088	56390419	11332	1	14609	uc007fch.2	322464	ENSMUSG000	Gja1	gap junction protein, alpha 1
chr10	56701597	56701753	157	158	56701685	37	11.12817	3.50427	7.83613	Adipose_R2_peak_2	Distal Interger	10	56379088	56390419	11332	1	14609	uc007fch.2	322509	ENSMUSG000	Gja1	gap junction protein, alpha 1
chr10	56707683	56708117	435	436	56707977	40	24.42476	7.36925	20.81738	Adipose_R1_peak_2	Distal Interger	10	56379088	56390419	11332	1	14609	uc007fch.2	328595	ENSMUSG000	Gja1	gap junction protein, alpha 1
chr10	56707917	56708106	190	191	56707984	31	7.7623	2.95097	4.69388	Adipose_R2_peak_2	Distal Interger	10	56379088	56390419	11332	1	14609	uc007fch.2	328829	ENSMUSG000	Gja1	gap junction protein, alpha 1
chr10	56953447	56953696	250	251	56953562	35	19.76029	6.47056	16.26028	Adipose_R1_peak_2	Distal Interger	10	57478383	57486353	7971	2	74928	uc029qxg.1	532657	ENSMUSG000	4930467K11Ri	RIKEN cDNA 4930467K11 gene
chr10	56953531	56953690	160	161	56953634	32	8.29282	3.04319	5.18352	Adipose_R2_peak_2	Distal Interger	10	57478383	57486353	7971	2	74928	uc029qxg.1	532663	ENSMUSG000	4930467K11Ri	RIKEN cDNA 4930467K11 gene
chr10	56977209	56977374	166	167	56977294	25	11.36566	4.67319	8.13482	Adipose_R1_peak_2	Distal Interger	10	57478383	57486353	7971	2	74928	uc029qxg.1	508979	ENSMUSG000	4930467K11Ri	RIKEN cDNA 4930467K11 gene
chr10	57023669	57024019	351	352	57023883	78	66.82238	14.19929	62.68234	Adipose_R1_peak_2	Distal Interger	10	57478383	57486353	7971	2	74928	uc029qxg.1	462334	ENSMUSG000	4930467K11Ri	RIKEN cDNA 4930467K11 gene
chr10	57023707	57024020	314	315	57023887	71	36.49133	6.63968	32.5091	Adipose_R2_peak_2	Distal Interger	10	57478383	57486353	7971	2	74928	uc029qxg.1	462333	ENSMUSG000	4930467K11Ri	RIKEN cDNA 4930467K11 gene
chr10	57073509	57073657	149	150	57073575	25	11.36566	4.67319	8.13482	Adipose_R1_peak_2	Distal Interger	10	57478383	57486353	7971	2	74928	uc029qxg.1	412696	ENSMUSG000	4930467K11Ri	RIKEN cDNA 4930467K11 gene
chr10	57099634	57099908	275	276	57099754	40	24.42476	7.36925	20.81738	Adipose_R1_peak_2	Distal Interger	10	57478383	57486353	7971	2	74928	uc029qxg.1	386445	ENSMUSG000	4930467K11Ri	RIKEN cDNA 4930467K11 gene
chr10	57099642	57099892	251	252	57099757	51	20.44827	4.79532	16.78434	Adipose_R2_peak_2	Distal Interger	10	57478383	57486353	7971	2	74928	uc029qxg.1	386461	ENSMUSG000	4930467K11Ri	RIKEN cDNA 4930467K11 gene
chr10	57226830	57227001	172	173	57226934	27	12.93148	5.03266	9.63827	Adipose_R1_peak_2	Distal Interger	10	57478383	57486353	7971	2	74928	uc029qxg.1	259352	ENSMUSG000	4930467K11Ri	RIKEN cDNA 4930467K11 gene
chr10	57226854	57226985	132	133	57226904	29	6.74062	2.76653	3.75989	Adipose_R2_peak_2	Distal Interger	10	57478383	57486353	7971	2	74928	uc029qxg.1	259368	ENSMUSG000	4930467K11Ri	RIKEN cDNA 4930467K11 gene
chr10	57240127	57240380	254	255	57240213	63	29.74474	5.90194	25.87429	Adipose_R2_peak_2	Distal Interger	10	57478383	57486353	7971	2	74928	uc029qxg.1	245973	ENSMUSG000	4930467K11Ri	RIKEN cDNA 4930467K11 gene
chr10	57240128	57240380	253	254	57240221	38	22.52591	7.00978	18.96	Adipose_R1_peak_2	Distal Interger	10	57478383	57486353	7971	2	74928	uc029qxg.1	245973	ENSMUSG000	4930467K11Ri	RIKEN cDNA 4930467K11 gene
chr10	57245140	57245375	236	237	57245248	31	16.23981	5.75161	12.83549	Adipose_R1_peak_2	Distal Interger	10	57478383	57486353	7971	2	74928	uc029qxg.1	240978	ENSMUSG000	4930467K11Ri	RIKEN cDNA 4930467K11 gene
chr10	57258875	57259108	234	235	57258939	32	17.10103	5.93135	13.67137	Adipose_R1_peak_2	Distal Interger	10	57478383	57486353	7971	2	74928	uc029qxg.1	227245	ENSMUSG000	4930467K11Ri	RIKEN cDNA 4930467K11 gene
chr10	57327129	57327315	187	188	57327223	22	9.13959	4.13397	6.01526	Adipose_R1_peak_2	Distal Interger	10	57478383	57486353	7971	2	74928	uc029qxg.1	159038	ENSMUSG000	4930467K11Ri	RIKEN cDNA 4930467K11 gene
chr10	57330699	57330922	224	225	57330820	38	22.52591	7.00978	18.96	Adipose_R1_peak_2	Distal Interger	10	57478383	57486353	7971	2	74928	uc029qxg.1	155431	ENSMUSG000	4930467K11Ri	RIKEN cDNA 4930467K11 gene
chr10	57330785	57330896	112	113	57330840	30	7.24477	2.85875	4.21917	Adipose_R2_peak_2	Distal Interger	10	57478383	57486353	7971	2	74928	uc029qxg.1	155457	ENSMUSG000	4930467K11Ri	RIKEN cDNA 4930467K11 gene
chr10	57434991	57435144	154	155	57435076	26	7.974	3.321	4.93129	Adipose_R1_peak_2	Distal Interger	10	57478383	57486353	7971	2	74928	uc029qxg.1	51209	ENSMUSG000	4930467K11Ri	RIKEN cDNA 4930467K11 gene
chr10	57435078	57435263	186	187	57435113	29	6.74062	2.76653	3.75989	Adipose_R2_peak_2	Distal Interger	10	57478383	57486353	7971	2	74928	uc029qxg.1	51090	ENSMUSG000	4930467K11Ri	RIKEN cDNA 4930467K11 gene
chr10	57435744	57435928	185	186	57435848	39	12.34187	3.68871	8.98501	Adipose_R2_peak_2	Distal Interger	10	57478383	57486353	7971	2	74928	uc029qxg.1	50425	ENSMUSG000	4930467K11Ri	RIKEN cDNA 4930467K11 gene
chr10	57437637	57437860	224	225	57437750	31	11.69687	4.12303	8.46369	Adipose_R1_peak_2	Distal Interger	10	57478383	57486353	7971	2	74928	uc029qxg.1	48493	ENSMUSG000	4930467K11Ri	RIKEN cDNA 4930467K11 gene
chr10	57437639	57437869	231	232	57437764	33	8.00477	2.91707	4.92731	Adipose_R2_peak_2	Distal Interger	10	57478383	57486353	7971	2	74928	uc029qxg.1	48484	ENSMUSG000	4930467K11Ri	RIKEN cDNA 4930467K11 gene
chr10	57438897	57438995	99	100	57438942	26	5.31252	2.48988	2.47999	Adipose_R2_peak_2	Distal Interger	10	57478383	57486353	7971	2	74928	uc029qxg.1	47358	ENSMUSG000	4930467K11Ri	RIKEN cDNA 4930467K11 gene
chr10	57440633	57440803	171	172	57440701	33	7.83542	2.87346	4.76432	Adipose_R2_peak_2	Distal Interger	10	57478383	57486353	7971	2	74928	uc029qxg.1	45550	ENSMUSG000	4930467K11Ri	RIKEN cDNA 4930467K11 gene
chr10	57441212	57441461	250	251	57441352	32	7.20996	2.757	4.21168	Adipose_R2_peak_2	Distal Interger	10	57478383	57486353	7971	2	74928	uc029qxg.1	44892	ENSMUSG000	4930467K11Ri	RIKEN cDNA 4930467K11 gene
chr10	57442642	57442845	204	205	57442742	32	7.45866	2.82165	4.42524	Adipose_R2_peak_2	Distal Interger	10	57478383	57486353	7971	2	74928	uc029qxg.1	43508	ENSMUSG000	4930467K11Ri	RIKEN cDNA 4930467K11 gene
chr10	57443036	57443431	396	397	57443288	31	7.7623	2.95097	4.69388	Adipose_R2_peak_2	Distal Interger	10	57478383	57486353	7971	2	74928	uc029qxg.1	42922	ENSMUSG000	4930467K11Ri	RIKEN cDNA 4930467K11 gene
chr10	57445036	57445259	224	225	57445187	34	6.35907	2.47115	3.42099	Adipose_R2_peak_2	Distal Interger	10	57478383	57486353	7971	2	74928	uc029qxg.1	41094	ENSMUSG000	4930467K11Ri	RIKEN cDNA 4930467K11 gene
chr10	57445526	57445733	208	209	57445723	26	5.18489	2.45238	2.39747	Adipose_R2_peak_2	Distal Interger	10	57478383	57486353	7971	2	74928	uc029qxg.1	40620	ENSMUSG000	4930467K11Ri	RIKEN cDNA 4930467K11 gene
chr10	57446857	57447033	177	178	57446940	32	8.29282	3.04319	5.18352	Adipose_R2_peak_2	Distal Interger	10	57478383	57486353	7971	2	74928	uc029qxg.1	39320	ENSMUSG000	4930467K11Ri	RIKEN cDNA 4930467K11 gene
chr10	57684385	57684725	341	342	57684547	189	202.6993	25.98989	198.0657	Adipose_R1_peak_2	Intron (uc007f	10	57650981	57741112	90132	1	18768	uc007fcs.1	33404	ENSMUSG000	Pkib	protein kinase inhibitor beta, cAMP dependent, testis specific
chr10	57684397	57684682	286	287	57684552	171	144.7542	15.86145	140.1113	Adipose_R2_peak_2	Intron (uc007f	10	57650981	57741112	90132	1	18768	uc007fcs.1	33416	ENSMUSG000	Pkib	protein kinase inhibitor beta, cAMP dependent, testis specific
chr10	58206566	58206700	135	136	58206599	16	5.20017	3.05554	2.38734	Adipose_R1_peak_2	Distal Interger	10	58230651	58232675	2025	2	664783	uc007fcv.3	25975	ENSMUSG000	Dux	double homeobox
chr10	58206668	58206844	177	178	58206713	27	5.77405	2.5821	2.88921	Adipose_R2_peak_2	Distal Interger	10	58230651	58232675	2025	2	664783	uc007fcv.3	25831	ENSMUSG000	Dux	double homeobox
chr10	58208690	58208829	140	141	58208757	28	6.25023	2.67431	3.31626	Adipose_R2_peak_2	Distal Interger	10	58230651	58232675	2025	2	664783	uc007fcv.3	23846	ENSMUSG000	Dux	double homeobox
chr10	58209973	58210069	97	98	58210060	26	5.31252	2.48988	2.47999	Adipose_R2_peak_2	Distal Interger	10	58230651	58232675	2025	2	664783	uc007fcv.3	22606	ENSMUSG000	Dux	double homeobox
chr10	58221381	58222426	1046	1047	58221532	173	50.2453	3.9619	46.08862	Adipose_R2_peak_2	Distal Interger	10	58230651	58232675	2025	2	664783	uc007fcv.3	10249	ENSMUSG000	Dux	double homeobox
chr10	58221982	58222344	363	364	58222090	119	12.46251	2.05879	9.19801	Adipose_R1_peak_2	Distal Interger	10	58230651	58232675	2025	2	664783	uc007fcv.3	10331	ENSMUSG000	Dux	double homeobox
chr10	58222681	58222787	107	108	58222726	54	6.06966	2.00127	3.17355	Adipose_R2_peak_2	Distal Interger	10	58230651	58232675	2025	2	664783	uc007fcv.3	9888	ENSMUSG000	Dux	double homeobox
chr10	58223152	58223405	254	255	58223277	203	79.08231	5.289	74.67767	Adipose_R2_peak_2	Distal Interger	10	58230651	58232675	2025	2	664783	uc007fcv.3	9270	ENSMUSG000	Dux	double homeobox
chr10	58223210	58223344	135	136	58223277	102	13.39462	2.27184	10.09842	Adipose_R1_peak_2	Distal Interger	10	58230651	58232675	2025	2	664783	uc007fcv.3	9331	ENSMUSG000	Dux	double homeobox
chr10	58223930	58224188	259	260	58224048	217	52.74066	3.46154	48.55907	Adipose_R2_peak_2	Distal Interger	10	58230651	58232675	2025	2	664783	uc007fcv.3	8487	ENSMUSG000	Dux	double homeobox
chr10	58223957	58224139	183	184	58224046	135	18.61333	2.36658	15.15684	Adipose_R1_peak_2	Distal Interger	10	58230651	58232675	2025	2	664783	uc007fcv.3	8536	ENSMUSG000	Dux	double homeobox
chr10	58224317	58224809	493	494	58224434	155	38.15586	3.4455	34.15394	Adipose_R2_peak_2	Distal Interger	10	58230651	58232675	2025	2	664783	uc007fcv.3	7866	ENSMUSG000	Dux	double homeobox
chr10	58224355	58224771	417	418	58224419	82	9.99213	2.15671	6.82904	Adipose_R1_peak_2	Distal Interger	10	58230651	58232675	2025	2	664783	uc007fcv.3	7904	ENSMUSG000	Dux	double homeobox
chr10	58224911	58225648	738	739	58225020	153	26.77641	2.72542	22.96334	Adipose_R2_peak_2	Distal Interger	10	58230651	58232675	2025	2	664783	uc007fcv.3	7027	ENSMUSG000	Dux	double homeobox
chr10	58225432	58225625	194	195	58225512	119	13.27429	2.11837	9.97901	Adipose_R1_peak_2	Distal Interger	10	58230651	58232675	2025	2	664783	uc007fcv.3	7050	ENSMUSG000	Dux	double homeobox
chr10	58225969	58226928	960	961	58226084	155	38.44428	3.46549	34.43449	Adipose_R2_peak_2	Distal Interger	10	58230651	58232675	2025	2	664783	uc007fcv.3	5747	ENSMUSG000	Dux	double homeobox
chr10	58226744	58226902	159	160	58226817	71	17.24222	3.22828	13.81178	Adipose_R1_peak_2	Distal Interger	10	58230651	58232675	2025	2	664783	uc007fcv.3	5773	ENSMUSG000	Dux	double homeobox
chr10	58227864	58228539	676	677	58227991	173	72.66975	5.69433	68.30927	Adipose_R2_peak_2	Downstream (	10	58230651	58232675	2025	2	664783	uc007fcv.3	4136	ENSMUSG000	Dux	double homeobox
chr10	58228205	58228487	283	284	58228238	62	8.44238	2.22686	5.35835	Adipose_R1_peak_2	Downstream (	10	58230651	58232675	2025	2	664783	uc007fcv.3	4188	ENSMUSG000	Dux	double homeobox

chr10	58229844	58230315	472	473	58229921	72	17.0041	3.1669	13.45153	Adipose_R2_peak_2	Promoter (2-3	10	58230651	58232675	2025	2	664783	uc007fcv.3	2360	ENSMUSG000	Dux	double homeobox
chr10	58231186	58231494	309	310	58231342	135	72.12402	7.49729	67.76875	Adipose_R2_peak_2	Promoter (1-2	10	58230651	58232675	2025	2	664783	uc007fcv.3	1181	ENSMUSG000	Dux	double homeobox
chr10	58231275	58231422	148	149	58231341	70	20.77816	3.77977	17.25463	Adipose_R1_peak_2	Promoter (1-2	10	58230651	58232675	2025	2	664783	uc007fcv.3	1253	ENSMUSG000	Dux	double homeobox
chr10	58231822	58232310	489	490	58231947	193	86.48045	6.1704	82.03255	Adipose_R2_peak_2	Promoter (<=1	10	58230651	58232675	2025	2	664783	uc007fcv.3	365	ENSMUSG000	Dux	double homeobox
chr10	58231901	58232870	970	971	58231964	70	10.30908	2.34649	7.14343	Adipose_R1_peak_2	Promoter (<=1	10	58230651	58232675	2025	2	664783	uc007fcv.3	0	ENSMUSG000	Dux	double homeobox
chr10	58232424	58232895	472	473	58232526	155	64.67969	5.61513	60.38341	Adipose_R2_peak_2	Promoter (<=1	10	58230651	58232675	2025	2	664783	uc007fcv.3	0	ENSMUSG000	Dux	double homeobox
chr10	58233270	58233433	164	165	58233338	74	19.79341	3.4902	16.29315	Adipose_R1_peak_2	Promoter (<=1	10	58230651	58232675	2025	2	664783	uc007fcv.3	-595	ENSMUSG000	Dux	double homeobox
chr10	58233294	58233459	166	167	58233351	100	30.77854	4.0283	26.89157	Adipose_R2_peak_2	Promoter (<=1	10	58230651	58232675	2025	2	664783	uc007fcv.3	-619	ENSMUSG000	Dux	double homeobox
chr10	58233815	58234128	314	315	58233918	138	61.56289	6.00907	57.29351	Adipose_R2_peak_2	Promoter (1-2	10	58230651	58232675	2025	2	664783	uc007fcv.3	-1140	ENSMUSG000	Dux	double homeobox
chr10	58233818	58233985	168	169	58233910	81	26.66161	4.20043	23.01419	Adipose_R1_peak_2	Promoter (1-2	10	58230651	58232675	2025	2	664783	uc007fcv.3	-1143	ENSMUSG000	Dux	double homeobox
chr10	58235111	58235284	174	175	58235197	70	28.00233	4.99786	24.16931	Adipose_R2_peak_2	Promoter (1-2	10	58234848	58236660	1813	2	245263	uc007fcw.1	1376	ENSMUSG000	Gm4981	predicted gene 4981
chr10	58235132	58235284	153	154	58235209	65	25.45447	4.84543	21.82727	Adipose_R1_peak_2	Promoter (1-2	10	58234848	58236660	1813	2	245263	uc007fcw.1	1376	ENSMUSG000	Gm4981	predicted gene 4981
chr10	58237824	58238114	291	292	58238000	37	11.24358	3.53401	8.04177	Adipose_R1_peak_2	Promoter (1-2	10	58234848	58236660	1813	2	245263	uc007fcw.1	-1164	ENSMUSG000	Gm4981	predicted gene 4981
chr10	58237994	58238139	146	147	58238044	51	18.93731	4.4465	15.32803	Adipose_R2_peak_2	Promoter (1-2	10	58234848	58236660	1813	2	245263	uc007fcw.1	-1334	ENSMUSG000	Gm4981	predicted gene 4981
chr10	58238468	58238661	194	195	58238540	32	8.74157	3.16552	5.65454	Adipose_R1_peak_2	Promoter (1-2	10	58234848	58236660	1813	2	245263	uc007fcw.1	-1808	ENSMUSG000	Gm4981	predicted gene 4981
chr10	58238506	58238679	174	175	58238560	37	11.09221	3.49503	7.81702	Adipose_R2_peak_2	Promoter (1-2	10	58234848	58236660	1813	2	245263	uc007fcw.1	-1846	ENSMUSG000	Gm4981	predicted gene 4981
chr10	58239428	58239693	266	267	58239546	172	91.00612	7.54172	86.53687	Adipose_R2_peak_2	Promoter (2-3	10	58234848	58236660	1813	2	245263	uc007fcw.1	-2768	ENSMUSG000	Gm4981	predicted gene 4981
chr10	58239459	58239658	200	201	58239546	96	24.8623	3.47904	21.25288	Adipose_R1_peak_2	Promoter (2-3	10	58234848	58236660	1813	2	245263	uc007fcw.1	-2799	ENSMUSG000	Gm4981	predicted gene 4981
chr10	59867775	59867907	133	134	59867820	29	6.74062	2.76653	3.75989	Adipose_R2_peak_2	Distal Intergen	10	59879591	59896775	17185	1	56709	uc007fdz.1	-11684	ENSMUSG000	Dnajb12	DnaJ heat shock protein family (Hsp40) member B12
chr10	59867802	59867975	174	175	59867919	24	10.60668	4.49345	7.40961	Adipose_R1_peak_2	Distal Intergen	10	59879591	59896775	17185	1	56709	uc007fdz.1	-11616	ENSMUSG000	Dnajb12	DnaJ heat shock protein family (Hsp40) member B12
chr10	62611151	62611411	261	262	62611277	43	27.35048	7.90847	23.68561	Adipose_R1_peak_2	Distal Intergen	10	62580247	62602298	22052	2	56200	uc007fhn.1	-8853	ENSMUSG000	Ddx21	DEAD (Asp-Glu-Ala-Asp) box polypeptide 21
chr10	62611165	62611405	241	242	62611284	53	21.92215	4.97976	18.21755	Adipose_R2_peak_2	Distal Intergen	10	62580247	62602298	22052	2	56200	uc007fhn.1	-8867	ENSMUSG000	Ddx21	DEAD (Asp-Glu-Ala-Asp) box polypeptide 21
chr10	63450982	63451153	172	173	63451055	25	11.36566	4.67319	8.13482	Adipose_R1_peak_2	Intron (uc007f	10	63457511	65003667	1546157	1	216033	uc007fki.2	-6358	ENSMUSG000	Ctnna3	catenin (cadherin associated protein), alpha 3
chr10	63451021	63451127	107	108	63451066	29	6.74062	2.76653	3.75989	Adipose_R2_peak_2	Intron (uc007f	10	63457511	65003667	1546157	1	216033	uc007fki.2	-6384	ENSMUSG000	Ctnna3	catenin (cadherin associated protein), alpha 3
chr10	63593149	63593482	334	335	63593374	22	9.13959	4.13397	6.01526	Adipose_R1_peak_2	Intron (uc007f	10	63457511	65003667	1546157	1	216033	uc007fki.2	135638	ENSMUSG000	Ctnna3	catenin (cadherin associated protein), alpha 3
chr10	64394878	64395088	211	212	64394974	27	12.93148	5.03266	9.63827	Adipose_R1_peak_2	Intron (uc007f	10	63928497	64090255	161759	2	216028	uc007fkk.1	-304623	ENSMUSG000	Lrrtm3	leucine rich repeat transmembrane neuronal 3
chr10	64394926	64395033	108	109	64394972	30	7.24477	2.85875	4.21917	Adipose_R2_peak_2	Intron (uc007f	10	63928497	64090255	161759	2	216028	uc007fkk.1	-304671	ENSMUSG000	Lrrtm3	leucine rich repeat transmembrane neuronal 3
chr10	64937883	64938193	311	312	64938036	93	86.02155	16.89536	81.75695	Adipose_R1_peak_2	Intron (uc007f	10	63928497	64090255	161759	2	216028	uc007fkk.1	-847628	ENSMUSG000	Lrrtm3	leucine rich repeat transmembrane neuronal 3
chr10	64937897	64938166	270	271	64938035	72	37.3624	6.73189	33.36734	Adipose_R2_peak_2	Intron (uc007f	10	63928497	64090255	161759	2	216028	uc007fkk.1	-847642	ENSMUSG000	Lrrtm3	leucine rich repeat transmembrane neuronal 3
chr10	65094314	65094510	197	198	65094417	36	20.67077	6.6503	17.14779	Adipose_R1_peak_2	Distal Intergen	10	63928497	64090255	161759	2	216028	uc007fkk.1	-1004059	ENSMUSG000	Lrrtm3	leucine rich repeat transmembrane neuronal 3
chr10	65098378	65098556	179	180	65098462	19	7.07687	3.59476	4.08458	Adipose_R1_peak_2	Distal Intergen	10	63928497	64090255	161759	2	216028	uc007fkk.1	-1008123	ENSMUSG000	Lrrtm3	leucine rich repeat transmembrane neuronal 3
chr10	65099950	65100051	102	103	65100036	30	7.24477	2.85875	4.21917	Adipose_R2_peak_2	Distal Intergen	10	63928497	64090255	161759	2	216028	uc007fkk.1	-1009695	ENSMUSG000	Lrrtm3	leucine rich repeat transmembrane neuronal 3
chr10	65167073	65167373	301	302	65167212	75	61.33073	13.02408	57.24107	Adipose_R1_peak_2	Distal Intergen	10	66120609	66124064	3456	2	69433	uc007fkn.2	956691	ENSMUSG000	1700023F02Ri	RIKEN cDNA 1700023F02 gene
chr10	65167079	65167342	264	265	65167203	75	40.01057	7.00855	35.97731	Adipose_R2_peak_2	Distal Intergen	10	66120609	66124064	3456	2	69433	uc007fkn.2	956722	ENSMUSG000	1700023F02Ri	RIKEN cDNA 1700023F02 gene
chr10	65201285	65201564	280	281	65201425	51	35.56531	9.34637	31.76235	Adipose_R1_peak_2	Distal Intergen	10	66120609	66124064	3456	2	69433	uc007fkn.2	922500	ENSMUSG000	1700023F02Ri	RIKEN cDNA 1700023F02 gene
chr10	65201324	65201526	203	204	65201424	45	16.22858	4.24202	12.70278	Adipose_R2_peak_2	Distal Intergen	10	66120609	66124064	3456	2	69433	uc007fkn.2	922538	ENSMUSG000	1700023F02Ri	RIKEN cDNA 1700023F02 gene
chr10	65658371	65658504	134	135	65658433	21	8.43286	3.95423	5.34896	Adipose_R1_peak_2	Distal Intergen	10	66120609	66124064	3456	2	69433	uc007fkn.2	465560	ENSMUSG000	1700023F02Ri	RIKEN cDNA 1700023F02 gene
chr10	65660283	65660594	312	313	65660464	68	54.69342	12.40192	50.66183	Adipose_R1_peak_2	Distal Intergen	10	66120609	66124064	3456	2	69433	uc007fkn.2	463470	ENSMUSG000	1700023F02Ri	RIKEN cDNA 1700023F02 gene
chr10	65660328	65660585	258	259	65660455	59	26.5291	5.53306	22.7188	Adipose_R2_peak_2	Distal Intergen	10	66120609	66124064	3456	2	69433	uc007fkn.2	463479	ENSMUSG000	1700023F02Ri	RIKEN cDNA 1700023F02 gene
chr10	65662698	65662965	268	269	65662831	41	25.38991	7.54899	21.76305	Adipose_R1_peak_2	Distal Intergen	10	66120609	66124064	3456	2	69433	uc007fkn.2	461099	ENSMUSG000	1700023F02Ri	RIKEN cDNA 1700023F02 gene
chr10	65662705	65662954	250	251	65662833	55	23.42763	5.16419	19.68558	Adipose_R2_peak_2	Distal Intergen	10	66120609	66124064	3456	2	69433	uc007fkn.2	461110	ENSMUSG000	1700023F02Ri	RIKEN cDNA 1700023F02 gene
chr10	65825571	65825748	178	179	65825633	26	12.14079	4.85292	8.87838	Adipose_R1_peak_2	Distal Intergen	10	66120609	66124064	3456	2	69433	uc007fkn.2	298316	ENSMUSG000	1700023F02Ri	RIKEN cDNA 1700023F02 gene
chr10	65905441	65905600	160	161	65905531	22	9.13959	4.13397	6.01526	Adipose_R1_peak_2	Intron (uc007f	10	66120609	66124064	3456	2	69433	uc007fkn.2	218464	ENSMUSG000	1700023F02Ri	RIKEN cDNA 1700023F02 gene
chr10	65941535	65941781	247	248	65941657	41	25.38991	7.54899	21.76305	Adipose_R1_peak_2	Exon (uc029qy	10	66120609	66124064	3456	2	69433	uc007fkn.2	182283	ENSMUSG000	1700023F02Ri	RIKEN cDNA 1700023F02 gene
chr10	65941592	65941772	181	182	65941665	38	11.72962	3.59649	8.4047	Adipose_R2_peak_2	Exon (uc029qy	10	66120609	66124064	3456	2	69433	uc007fkn.2	182292	ENSMUSG000	1700023F02Ri	RIKEN cDNA 1700023F02 gene
chr10	65944383	65944548	166	167	65944449	21	8.43286	3.95423	5.34896	Adipose_R1_peak_2	3' UTR	10	66120609	66124064	3456	2	69433	uc007fkn.2	179516	ENSMUSG000	1700023F02Ri	RIKEN cDNA 1700023F02 gene
chr10	66156695	66156872	178	179	66156758	24	10.60668	4.49345	7.40961	Adipose_R1_peak_2	Intron (uc007f	10	66145296	66179514	34219	1	77198	uc007fko.1	11399	NA	4930407I19Ri	RIKEN cDNA 4930407I19 gene
chr10	66208684	66208923	240	241	66208786	34	18.86162	6.29083	15.38482	Adipose_R1_peak_2	Distal Intergen	10	66145296	66179514	34219	1	77198	uc007fko.1	63388	NA	4930407I19Ri	RIKEN cDNA 4930407I19 gene
chr10	66410452	66410586	135	136	66410515	19	7.07687	3.59476	4.08458	Adipose_R1_peak_2	Distal Intergen	10	66145296	66179514	34219	1	77198	uc007fko.1	265156	NA	4930407I19Ri	RIKEN cDNA 4930407I19 gene
chr10	66463685	66464017	333	334	66463854	91	83.39809	16.53589	79.14825	Adipose_R1_peak_2	Distal Intergen	10	66145296	66179514	34219	1	77198	uc007fko.1	318389	NA	4930407I19Ri	RIKEN cDNA 4930407I19 gene
chr10	66463701	66463968	268	269	66463857	57	24.96362	5.34863	21.18596	Adipose_R2_peak_2	Distal Intergen	10	66145296	66179514	34219	1	77198	uc007fko.1	318405	NA	4930407I19Ri	RIKEN cDNA 4930407I19 gene
chr10	66466170	66466304	135	136	66466238	20	7.74501	3.7745	4.70467	Adipose_R1_peak_2	Distal Intergen	10	66145296	66179514	34219	1	77198	uc007fko.1	320874	NA	4930407I19Ri	RIKEN cDNA 4930407I19 gene
chr10	66466188	66466307	120	121	66466254	28	6.25023	2.67431	3.31626	Adipose_R2_peak_2	Distal Intergen	10	66145296	66179514	34219	1	77198	uc007fko.1	320892	NA	4930407I19Ri	RIKEN cDNA 4930407I19 gene
chr10	66527626	66527821	196	197	66527696	27	12.93148	5.03266	9.63827	Adipose_R1_peak_2	Distal Intergen	10	66145296	66179514	34219	1	77198	uc007fko.1	382330	NA	4930407I19Ri	RIKEN cDNA 4930407I19 gene
chr10	66553788	66553926	139	140	66553873	18	6.42934	3.41502	3.48999	Adipose_R1_peak_2	Distal Intergen	10	66145296	66179514	34219	1	77198	uc007fko.1	408492	NA	4930407I19Ri	RIKEN cDNA 4930407I19 gene
chr10	66570269	66570432	164	165	66570314	34	9.39139	3.22762	6.20474	Adipose_R2_peak_2	Distal Intergen	10	66145296	66179514	34219	1	77198	uc007fko.1	424973	NA	4930407I19Ri	RIKEN cDNA 4930407I19 gene
chr10	66570275	66570456	182	183	66570346	24	10.60668	4.49345	7.40961	Adipose_R1_peak_2	Distal Intergen	10	66145296	66179514	34219	1	77198	uc007fko.1	424979	NA	4930407I19Ri	RIKEN cDNA 4930407I19 gene
chr10	66633328	66633525	198	199	66633434	48	18.29934	4.51867	14.70076	Adipose_R2_peak_2	Distal Intergen	10	67005075	67041326	36252	1	1.03E+08	uc007flp.1	-371550	NA	Gm31763	predicted gene, 31763
chr10	66633329	66633591	263	264	66633448	53	37.70483	9.70585	33.87	Adipose_R1_peak_2	Distal Intergen	10	67005075	67041326	36252	1	1.03E+08	uc007flp.1	-371484	NA	Gm31763	predicted gene, 31763
chr10	66677553	66677852	300	301	66677707	92	84.7075	16.71563	80.45023	Adipose_R1_peak_2	Distal Intergen	10	67005075	67041326	36252	1	1.03E+08	uc007flp.1	-327223	NA	Gm31763	predicted gene, 31763
chr10	66677568	66677826	259	260	66677699	70	35.62622	6.54746	31.65691	Adipose_R2_peak_2	Distal Intergen	10	67005075	67041326	36252	1	1.03E+08	uc007flp.1	-327249	NA	Gm31763	predicted gene, 31763
chr10	66724326	66724471	146	147	66724407	22	9.13959	4.13397	6.01526	Adipose_R1_peak_2	Distal Intergen	10	67005075	67041326	36252	1	1.03E+08	uc007flp.1	-280604	NA	Gm31763	predicted gene, 31763
chr10	66807883	66808049	167	168	66807959	21	8.43286	3.95423	5.34896	Adipose_R1_peak_2	Distal Intergen	10	67005075	67041326	36252	1	1.03E+08	uc007flp.1	-197026	NA	Gm31763	predicted gene, 31763
chr10	68375336	68375962	627	628	68375655	72	57.75775	12.50997	53.70046	Adipose_R1_peak_2	Distal Intergen	10	68319065	68321142	2078	2	75148	uc007fmk.2	-54194	NA	4930545H06R	RIKEN cDNA 4930545H06 gene
chr10	68375458	68375917	460	461	68375635	45	16.22858	4.24202	12.70278	Adipose_R2_peak_2	Distal Intergen	10	68319065	68321142	2078	2	75148	uc007fmk.2	-54316	NA	4930545H06R	RIKEN cDNA 4930545H06 gene
chr10	68685702	68685844	143	144	68685723	16	5.20017	3.05554	2.38734	Adipose_R1_peak_2	Distal Intergen	10	68723746	68782654	58909	1	327766	uc007fmm.2	-37902	ENSMUSG000	Tmem26	transmembrane protein 26
chr10	68769472	68769784	313	314	68769648	70	57.07198	12.76139	53.01762	Adipose_R1_peak_2	Intron (uc007f	10	68723746	68782654	58909	1	327766	uc007fmm.2	45726	ENSMUSG000	Tmem26	transmembrane protein 26
chr10	68769505	68769769	265	266	68769649	52	21.18119	4.88754	17.49655	Adipose_R2_peak_2	Intron (uc007f	10	68723746	68782654	58909	1	327766	uc007fmm.2	45759	ENSMUSG000	Tmem26	transmembrane protein 26
chr10	69050512	69050760	249	250	69050681	38	22.52591	7.00978	18.96	Adipose_R1_peak_2	Distal Intergen	10	69208552	69291784	83233	1	69288	uc029qyr.1	-157792	ENSMUSG000	Rhobtb1	Rho-related BTB domain containing 1
chr10	69050522	69050751	230	231	69050669	39	12.34187	3.68871	8.98501	Adipose_R2_peak_2	Distal Intergen	10	69208552	69291784	83233	1	69288	uc029qyr.1	-157801	ENSMUSG000	Rhobtb1	Rho-related BTB domain containing 1
chr10	69055370	69055545	176	177	69055455	23	9.86443	4.31371	6.70247	Adipose_R1_peak_2	Distal Intergen	10	69208552	69291784	83233	1	69288	uc029qyr.1	-153007	ENSMUSG000	Rhobtb1	Rho-related BTB domain containing 1
chr10	69196869	69197121	253	254	69196994	39	23.47001	7.18952	19.88325	Adipose_R1_peak_2	Distal Intergen	10	69208552	69291784	83233	1	69288	uc029qyr.1	-11431	ENSMUSG000	Rhobtb1	Rho-related BTB domain containing 1
chr10	69196880	69197102	223	224	69197000	49	19.00719	4.61089	15.38598	Adipose_R2_peak_2	Distal Intergen	10	69208552	69291784	83233	1	69288	uc029qyr.1	-11450	ENSMUSG000	Rhobtb1	Rho-related BTB domain containing 1
chr10	70222366	70222514	149	150	70222423	75	20.75724	3.59152	17.23376	Adipose_R1_peak_2	Distal Intergen	10	70204664	70219711	15048	1	69563	uc007fnp.4	17702	NA	Mrln	myoregulin
chr10	70762764	70762861	98	99	70762807	26	5.31252	2.48988	2.47999	Adipose_R2_peak_2	Distal Intergen	10	70601049	70655963	54915	2	319646	uc007fod.1	-106801	ENSMUSG000	D630013N20R	RIKEN cDNA D630013N20 gene
chr10	70787788	70787970	183	184	70787873	25	11.36566	4.67319	8.13482	Adipose_R1_peak_2	Distal Intergen	10	70601049	70655963	54915	2	319646	uc007fod.1	-131825	ENSMUSG000	D630013N20R	RIKEN cDNA D630013N20 gene
chr10	71509898	71510055	158	159	71509964	22	9.13959	4.13397	6.01526	Adipose_R1_peak_2	Distal Intergen	10	71347793	71377414	29622	1	69718	uc007for.1	162105	ENSMUSG000	Ipmk	inositol polyphosphate multikinase
chr10	71510414	71510683	270	271	71510551	51	35.56531	9.34637	31.76235	Adipose_R1_peak_2	Distal Intergen	10	71347793	71377414	29622	1	69718	uc007for.1	162621	ENSMUSG000	Ipmk	inositol polyphosphate multikinase
chr10	71510419	71510669	251	252	71510551	56	24.19188	5.25641	20.43163	Adipose_R2_peak_2	Distal Intergen	10	71347793	71377414	29622	1	69718	uc007for.1	162626	ENSMUSG000	Ipmk	inositol polyphosphate multikinase
chr10	71562710	71563064	355	356	71562907	104	96.09283	17.2663	91.77853	Adipose_R1_peak_2	Distal Intergen	10	71347793	71377414	29622	1	69718	uc007for.1	214917	ENSMUSG000	Ipmk	inositol polyphosphate multikinase
chr10	71562763	71563044	282	283	71562910	84	48.25301	7.83851	44.11785	Adipose_R2_peak_2	Distal Intergen	10	71347793	71377414	29622	1	69718	uc007for.1	214970	ENSMUSG000	Ipmk	inositol polyphosphate multikinase
chr10	71608305	71608515	211	212	71608390	30	15.39182	5.57188	12.01394	Adipose_R1_peak_2	Distal Intergen	10	71347793	71377414	29622	1	69718	uc007for.1	260512	ENSMUSG000	Ipmk	inositol polyphosphate multikinase
chr10	71608326	71608439	114	115	71608384	29	6.74062	2.76653	3.75989	Adipose_R2_peak_2	Distal Intergen	10	71347793	71377414	29622	1	69718	uc007for.1	260533	ENSMUSG000	Ipmk	inositol polyphosphate multikinase
chr10	71629986	71630163	178	179	71630059	27	12.93148	5.03266	9.63827	Adipose_R1_peak_2	Distal Intergen	10	71347793	71377414	29622	1	69718	uc007for.1	282193	ENSMUSG000	Ipmk	inositol polyphosphate multikinase
chr10	71631190	71631392	203	204	71631319	35	19.76029	6.47056	16.26028	Adipose_R1_peak_2	Distal Intergen	10	71347793	71377414	29622	1	69718	uc007for.1	283397	ENSMUSG000	Ipmk	inositol polyphosphate multikinase
chr10	71631954	71632263	310	311	71632091	71	58.27042	12.94113	54.20487	Adipose_R1_peak_2	Distal Intergen	10	71347793	71377414	29622	1	69718	uc007for.1	284161	ENSMUSG000	Ipmk	inositol polyphosphate multikinase
chr10	71631966	71632256	291	292	71632087	72	37.3624	6.73189	33.36734	Adipose_R2_peak_2	Distal Intergen	10	71347793	71377414	29622	1	69718	uc007for.1	284173	ENSMUSG000	Ipmk	inositol polyphosphate multikinase
chr10	71634181	71634398	218	219	71634291	26	12.14079	4.85292	8.87838	Adipose_R1_peak_2	Distal Intergen	10	71347793	71377414	29622	1	69718	uc007for.1	286388	ENSMUSG000	Ipmk	inositol polyphosphate multikinase
chr10	71682358	71682497	140	141	71682430	30	7.24477	2.85875	4.21917	Adipose_R2_peak_2	Distal Intergen	10	71979885	71980690	806	2	108950	uc007fox.1	298193	ENSMUSG000	1700049L16Ri	hematological and neurological expressed 1-like pseudogene
chr10	71718524	71718661	138	139	71718588	20	7.74501	3.7745	4.70467	Adipose_R1_peak_2	Distal Intergen	10	71979885	71980690	806	2	108950	uc007fox.1	262029	ENSMUSG000	1700049L16Ri	hematological and neurological expressed 1-like pseudogene
chr10	71842549	71842730	182	183	71842667	27	12.93148	5.03266	9.63827	Adipose_R1_peak_2	Distal Intergen	10	71979885	71980690	806	2	108950	uc007fox.1	137960	ENSMUSG000	1700049L16Ri	hematological and neurological expressed 1-like pseudogene
chr10	71930255	71930422	168	169	71930329	23	9.86443	4.31371	6.70247	Adipose_R1_peak_2	Distal Intergen	10	71979885	71980690	806	2	108950	uc007fox.1	50268	ENSMUSG000	1700049L16Ri	hematological and neurological expressed 1-like pseudogene
chr10	72009326	72009505	180	181	72009417	25	11.36566	4.67319	8.13482	Adipose_R1_peak_2	Distal Intergen	10	71979885	71980690	806	2	108950	uc007fox.1	-28636	ENSMUSG000	1700049L16Ri	hematological and neurological expressed 1-like pseudogene
chr10	72052271	72052403	133	134	72052334	17	5.80341	3.23528	2.92378	Adipose_R1_peak_2	Distal Intergen	10	71979885	71980690	806	2	108950	uc007fox.1	-71581	ENSMUSG000	1700049L16Ri	hematological and neurological expressed 1-like pseudogene

chr10	72122291	72122430	140	141	72122392	19	7.07687	3.59476	4.08458	Adipose_R1_peak_	Distal Intergen	10	71979885	71980690	806	2	108950	uc007fox.1	-141601	ENSMUSG000	1700049L16Ri	hematological and neurological expressed 1-like pseudogene
chr10	72297196	72297372	177	178	72297317	29	14.55748	5.39214	11.20652	Adipose_R1_peak_	Distal Intergen	10	71979885	71980690	806	2	108950	uc007fox.1	-316506	ENSMUSG000	1700049L16Ri	hematological and neurological expressed 1-like pseudogene
chr10	72461445	72461689	245	246	72461568	41	25.38991	7.54899	21.76305	Adipose_R1_peak_	Distal Intergen	10	72654846	72669792	14947	1	52696	uc007foy.2	-193157	ENSMUSG000	Zwint	ZW10 interactor
chr10	72461455	72461661	207	208	72461571	43	14.8939	4.05758	11.42017	Adipose_R2_peak_	Distal Intergen	10	72654846	72669792	14947	1	52696	uc007foy.2	-193185	ENSMUSG000	Zwint	ZW10 interactor
chr10	72498258	72498570	313	314	72498427	126	131.7124	22.82671	127.2552	Adipose_R1_peak_	Distal Intergen	10	72654846	72669792	14947	1	52696	uc007foy.2	-156276	ENSMUSG000	Zwint	ZW10 interactor
chr10	72498363	72498564	202	203	72498430	49	19.00719	4.61089	15.38598	Adipose_R2_peak_	Distal Intergen	10	72654846	72669792	14947	1	52696	uc007foy.2	-156282	ENSMUSG000	Zwint	ZW10 interactor
chr10	72695133	72695232	100	101	72695178	28	6.25023	2.67431	3.31626	Adipose_R2_peak_	Distal Intergen	10	72654846	72669792	14947	1	52696	uc007foy.2	40287	ENSMUSG000	Zwint	ZW10 interactor
chr10	72695148	72695304	157	158	72695211	17	5.80341	3.23528	2.92378	Adipose_R1_peak_	Distal Intergen	10	72654846	72669792	14947	1	52696	uc007foy.2	40302	ENSMUSG000	Zwint	ZW10 interactor
chr10	72733112	72733317	206	207	72733212	32	17.10103	5.93135	13.67137	Adipose_R1_peak_	Distal Intergen	10	72654846	72669792	14947	1	52696	uc007foy.2	78266	ENSMUSG000	Zwint	ZW10 interactor
chr10	72849718	72850127	410	411	72849830	26	12.14079	4.85292	8.87838	Adipose_R1_peak_	Distal Intergen	10	72654846	72669792	14947	1	52696	uc007foy.2	194872	ENSMUSG000	Zwint	ZW10 interactor
chr10	72849886	72850118	233	234	72850042	38	11.72962	3.59649	8.4047	Adipose_R2_peak_	Distal Intergen	10	72654846	72669792	14947	1	52696	uc007foy.2	195040	ENSMUSG000	Zwint	ZW10 interactor
chr10	72932329	72932544	216	217	72932457	35	19.76029	6.47056	16.26028	Adipose_R1_peak_	Distal Intergen	10	72973303	73086705	113403	2	327768	uc007fpb.1	154161	NA	A330049N07R	RIKEN cDNA A330049N07 gene
chr10	72932361	72932537	177	178	72932479	29	6.74062	2.76653	3.75989	Adipose_R2_peak_	Distal Intergen	10	72973303	73086705	113403	2	327768	uc007fpb.1	154168	NA	A330049N07R	RIKEN cDNA A330049N07 gene
chr10	72967101	72967394	294	295	72967245	32	17.10103	5.93135	13.67137	Adipose_R1_peak_	Distal Intergen	10	72973303	73086705	113403	2	327768	uc007fpb.1	119311	NA	A330049N07R	RIKEN cDNA A330049N07 gene
chr10	73159139	73159243	105	106	73159178	25	4.86612	2.39766	2.09018	Adipose_R2_peak_	Intron (uc007f	10	73099342	74317264	1217923	1	11994	uc007fpe.1	59797	ENSMUSG000	Pcdh15	protocadherin 15
chr10	73221589	73221759	171	172	73221690	21	8.43286	3.95423	5.34896	Adipose_R1_peak_	Intron (uc007f	10	73099342	74317264	1217923	1	11994	uc007fpe.1	122247	ENSMUSG000	Pcdh15	protocadherin 15
chr10	73221646	73221770	125	126	73221730	28	6.25023	2.67431	3.31626	Adipose_R2_peak_	Intron (uc007f	10	73099342	74317264	1217923	1	11994	uc007fpe.1	122304	ENSMUSG000	Pcdh15	protocadherin 15
chr10	73441137	73441377	241	242	73441247	32	17.10103	5.93135	13.67137	Adipose_R1_peak_	Intron (uc007f	10	73099342	74317264	1217923	1	11994	uc007fpe.1	341795	ENSMUSG000	Pcdh15	protocadherin 15
chr10	73576523	73576815	293	294	73576678	59	44.30963	10.78427	40.38949	Adipose_R1_peak_	Intron (uc007f	10	73821867	74507600	685734	1	11994	uc007fpf.2	-245052	ENSMUSG000	Pcdh15	protocadherin 15
chr10	73576567	73576767	201	202	73576686	49	19.00719	4.61089	15.38598	Adipose_R2_peak_	Intron (uc007f	10	73821867	74507600	685734	1	11994	uc007fpf.2	-245100	ENSMUSG000	Pcdh15	protocadherin 15
chr10	73597743	73597894	152	153	73597807	21	8.43286	3.95423	5.34896	Adipose_R1_peak_	Intron (uc007f	10	73821867	74507600	685734	1	11994	uc007fpf.2	-223973	ENSMUSG000	Pcdh15	protocadherin 15
chr10	74244093	74244360	268	269	74244233	48	30.35202	8.05761	26.64488	Adipose_R1_peak_	Intron (uc007f	10	73947028	74634765	687738	1	11994	uc033fpz.1	297065	ENSMUSG000	Pcdh15	protocadherin 15
chr10	74244125	74244310	186	187	74244194	36	10.5378	3.41206	7.27949	Adipose_R2_peak_	Intron (uc007f	10	73947028	74634765	687738	1	11994	uc033fpz.1	297097	ENSMUSG000	Pcdh15	protocadherin 15
chr10	74245436	74245578	143	144	74245498	19	7.07687	3.59476	4.08458	Adipose_R1_peak_	Intron (uc007f	10	73947028	74634765	687738	1	11994	uc033fpz.1	298408	ENSMUSG000	Pcdh15	protocadherin 15
chr10	74344658	74344909	252	253	74344785	31	16.23981	5.75161	12.83549	Adipose_R1_peak_	Intron (uc007f	10	74624205	74649732	25528	1	11994	uc029qyt.1	-279296	ENSMUSG000	Pcdh15	protocadherin 15
chr10	74434950	74435233	284	285	74435114	75	40.01057	7.00855	35.97731	Adipose_R2_peak_	Intron (uc007f	10	74624205	74649732	25528	1	11994	uc029qyt.1	-188972	ENSMUSG000	Pcdh15	protocadherin 15
chr10	74434963	74435273	311	312	74435131	70	57.07198	12.76139	53.01762	Adipose_R1_peak_	Intron (uc007f	10	74624205	74649732	25528	1	11994	uc029qyt.1	-188932	ENSMUSG000	Pcdh15	protocadherin 15
chr10	74768313	74768534	222	223	74768453	25	11.36566	4.67319	8.13482	Adipose_R1_peak_	Distal Intergen	10	74624205	74649732	25528	1	11994	uc029qyt.1	144108	ENSMUSG000	Pcdh15	protocadherin 15
chr10	74768322	74768569	248	249	74768459	40	12.96465	3.78093	9.57654	Adipose_R2_peak_	Distal Intergen	10	74624205	74649732	25528	1	11994	uc029qyt.1	144117	ENSMUSG000	Pcdh15	protocadherin 15
chr10	74770802	74771011	210	211	74770880	22	9.13959	4.13397	6.01526	Adipose_R1_peak_	Distal Intergen	10	74624205	74649732	25528	1	11994	uc029qyt.1	146597	ENSMUSG000	Pcdh15	protocadherin 15
chr10	74770814	74770971	158	159	74770866	31	7.7623	2.95097	4.69388	Adipose_R2_peak_	Distal Intergen	10	74624205	74649732	25528	1	11994	uc029qyt.1	146609	ENSMUSG000	Pcdh15	protocadherin 15
chr10	74771190	74771380	191	192	74771273	29	14.55748	5.39214	11.20652	Adipose_R1_peak_	Distal Intergen	10	74624205	74649732	25528	1	11994	uc029qyt.1	146985	ENSMUSG000	Pcdh15	protocadherin 15
chr10	75505976	75506246	271	272	75506103	17	5.80341	3.23528	2.92378	Adipose_R1_peak_	Intron (uc007f	10	75416467	75511286	94820	2	68778	uc011xgn.1	5040	ENSMUSG000	Gucd1	guanylyl cyclase domain containing 1
chr10	75513034	75513169	136	137	75513053	25	4.86612	2.39766	2.09018	Adipose_R2_peak_	Promoter (1-2	10	75416467	75511286	94820	2	68778	uc011xgn.1	-1748	ENSMUSG000	Gucd1	guanylyl cyclase domain containing 1
chr10	75516094	75516277	184	185	75516155	29	6.74062	2.76653	3.75989	Adipose_R2_peak_	Promoter (1-2	10	75351111	75517322	166212	2	68778	uc007fqi.1	1045	ENSMUSG000	Gucd1	guanylyl cyclase domain containing 1
chr10	75764644	75764847	204	205	75764679	26	5.31252	2.48988	2.47999	Adipose_R2_peak_	Promoter (<=	10	75646110	75764357	118248	2	104248	uc007fqx.1	-287	ENSMUSG000	Cabin1	calcineurin binding protein 1
chr10	75765081	75765303	223	224	75765240	28	6.25023	2.67431	3.31626	Adipose_R2_peak_	Promoter (<=	10	75646110	75764357	118248	2	104248	uc007fqx.1	-724	ENSMUSG000	Cabin1	calcineurin binding protein 1
chr10	75766727	75766912	186	187	75766762	16	5.0713	2.99748	2.32518	Adipose_R1_peak_	Promoter (2-3	10	75646110	75764357	118248	2	104248	uc007fqx.1	-2370	ENSMUSG000	Cabin1	calcineurin binding protein 1
chr10	75766756	75766858	103	104	75766778	25	4.86612	2.39766	2.09018	Adipose_R2_peak_	Promoter (2-3	10	75646110	75764357	118248	2	104248	uc007fqx.1	-2399	ENSMUSG000	Cabin1	calcineurin binding protein 1
chr10	75767129	75767318	190	191	75767239	30	6.96253	2.78106	3.97376	Adipose_R2_peak_	Promoter (2-3	10	75646110	75764357	118248	2	104248	uc007fqx.1	-2772	ENSMUSG000	Cabin1	calcineurin binding protein 1
chr10	75767979	75768146	168	169	75768050	16	5.20017	3.05554	2.38734	Adipose_R1_peak_	Distal Intergen	10	75646110	75764357	118248	2	104248	uc007fqx.1	-3622	ENSMUSG000	Cabin1	calcineurin binding protein 1
chr10	75770444	75770831	388	389	75770593	27	5.77405	2.5821	2.88921	Adipose_R2_peak_	Promoter (2-3	10	75771233	75773374	2142	2	13202	uc007fra.1	2543	ENSMUSG000	Ddt	D-dopachrome tautomerase
chr10	75771599	75771776	178	179	75771670	30	6.03544	2.5319	3.14051	Adipose_R2_peak_	Promoter (1-2	10	75771233	75773374	2142	2	13202	uc007fra.1	1598	ENSMUSG000	Ddt	D-dopachrome tautomerase
chr10	75771802	75772029	228	229	75771915	17	5.76977	3.22034	2.92378	Adipose_R1_peak_	Promoter (1-2	10	75771233	75773374	2142	2	13202	uc007fra.1	1345	ENSMUSG000	Ddt	D-dopachrome tautomerase
chr10	75771957	75772118	162	163	75772045	30	7.24477	2.85875	4.21917	Adipose_R2_peak_	Promoter (1-2	10	75771233	75773374	2142	2	13202	uc007fra.1	1256	ENSMUSG000	Ddt	D-dopachrome tautomerase
chr10	75772403	75772663	261	262	75772494	24	10.60668	4.49345	7.40961	Adipose_R1_peak_	Promoter (<=	10	75771233	75773374	2142	2	13202	uc007fra.1	711	ENSMUSG000	Ddt	D-dopachrome tautomerase
chr10	75774748	75774930	183	184	75774771	27	5.77405	2.5821	2.88921	Adipose_R2_peak_	Promoter (1-2	10	75771233	75773374	2142	2	13202	uc007fra.1	-1374	ENSMUSG000	Ddt	D-dopachrome tautomerase
chr10	75982344	75982600	257	258	75982461	52	36.63102	9.52611	32.81188	Adipose_R1_peak_	Intron (uc007f	10	75974181	75998274	24094	1	333669	uc011xgu.2	8163	ENSMUSG000	Gm5134	predicted gene 5134
chr10	75982359	75982573	215	216	75982476	47	17.60017	4.42645	14.02531	Adipose_R2_peak_	Intron (uc007f	10	75974181	75998274	24094	1	333669	uc011xgu.2	8178	ENSMUSG000	Gm5134	predicted gene 5134
chr10	77963258	77963411	154	155	77963373	21	8.43286	3.95423	5.34896	Adipose_R1_peak_	Intron (uc007f	10	77907722	77969872	62151	2	28240	uc007fwl.2	6461	ENSMUSG000	Trpm2	transient receptor potential cation channel, subfamily M, member 2
chr10	77973832	77973968	137	138	77973914	26	5.31252	2.48988	2.47999	Adipose_R2_peak_	Distal Intergen	10	77907722	77969872	62151	2	28240	uc007fwl.2	-3960	ENSMUSG000	Trpm2	transient receptor potential cation channel, subfamily M, member 2
chr10	78155202	78155366	165	166	78155309	19	7.07687	3.59476	4.08458	Adipose_R1_peak_	Distal Intergen	10	78162067	78169768	7702	2	28295	uc007fxj.1	14402	ENSMUSG000	D10Jhu81e	DNA segment, Chr 10, Johns Hopkins University 81 expressed
chr10	78171627	78171745	119	120	78171735	25	4.86612	2.39766	2.09018	Adipose_R2_peak_	Promoter (1-2	10	78162067	78169768	7702	2	28295	uc007fxj.1	-1859	ENSMUSG000	D10Jhu81e	DNA segment, Chr 10, Johns Hopkins University 81 expressed
chr10	78945492	78945750	259	260	78945632	50	34.50784	9.16663	30.72138	Adipose_R1_peak_	Distal Intergen	10	78955207	78956139	933	1	18372	uc007fyj.1	-9457	ENSMUSG000	Olfr8	olfactory receptor 8
chr10	78945523	78945745	223	224	78945627	41	13.59774	3.87315	10.1803	Adipose_R2_peak_	Distal Intergen	10	78955207	78956139	933	1	18372	uc007fyj.1	-9462	ENSMUSG000	Olfr8	olfactory receptor 8
chr10	78953538	78953692	155	156	78953613	25	11.36566	4.67319	8.13482	Adipose_R1_peak_	Promoter (1-2	10	78955207	78956139	933	1	18372	uc007fyj.1	-1515	ENSMUSG000	Olfr8	olfactory receptor 8
chr10	79080333	79080613	281	282	79080457	75	54.7943	10.88402	50.76215	Adipose_R1_peak_	Intron (uc007f	10	79064374	79097600	33227	2	72128	uc007fyo.2	16987	ENSMUSG000	2610008E11Ri	RIKEN cDNA 2610008E11 gene
chr10	79080335	79080612	278	279	79080465	87	51.09389	8.11516	46.92713	Adipose_R2_peak_	Intron (uc007f	10	79064374	79097600	33227	2	72128	uc007fyo.2	16988	ENSMUSG000	2610008E11Ri	RIKEN cDNA 2610008E11 gene
chr10	79209308	79209752	445	446	79209415	32	17.10103	5.93135	13.67137	Adipose_R1_peak_	Distal Intergen	10	79247777	79294535	46759	1	216144	uc007fyp.1	-38025	ENSMUSG000	Vmn2r81	vomeronasal 2, receptor 81
chr10	79209929	79210081	153	154	79210001	33	8.83596	3.1354	5.68652	Adipose_R2_peak_	Distal Intergen	10	79247777	79294535	46759	1	216144	uc007fyp.1	-37696	ENSMUSG000	Vmn2r81	vomeronasal 2, receptor 81
chr10	79226105	79226346	242	243	79226265	61	46.56983	11.14375	42.62347	Adipose_R1_peak_	Distal Intergen	10	79247777	79294535	46759	1	216144	uc007fyp.1	-21431	ENSMUSG000	Vmn2r81	vomeronasal 2, receptor 81
chr10	79226134	79226334	201	202	79226280	41	13.59774	3.87315	10.1803	Adipose_R2_peak_	Distal Intergen	10	79247777	79294535	46759	1	216144	uc007fyp.1	-21443	ENSMUSG000	Vmn2r81	vomeronasal 2, receptor 81
chr10	79285103	79285260	158	159	79285167	21	8.43286	3.95423	5.34896	Adipose_R1_peak_	Intron (uc007f	10	79247777	79294535	46759	1	216144	uc007fyp.1	37326	ENSMUSG000	Vmn2r81	vomeronasal 2, receptor 81
chr10	79287707	79287917	211	212	79287816	32	17.10103	5.93135	13.67137	Adipose_R1_peak_	Intron (uc007f	10	79247777	79294535	46759	1	216144	uc007fyp.1	39930	ENSMUSG000	Vmn2r81	vomeronasal 2, receptor 81
chr10	79287741	79287886	146	147	79287787	32	8.29282	3.04319	5.18352	Adipose_R2_peak_	Intron (uc007f	10	79247777	79294535	46759	1	216144	uc007fyp.1	39964	ENSMUSG000	Vmn2r81	vomeronasal 2, receptor 81
chr10	79315166	79315286	121	122	79315203	26	5.31252	2.48988	2.47999	Adipose_R2_peak_	Distal Intergen	10	79356591	79396766	40176	1	624845	uc009vaa.1	-41305	ENSMUSG000	Vmn2r82	vomeronasal 2, receptor 82
chr10	79352972	79353106	135	136	79353041	21	8.43286	3.95423	5.34896	Adipose_R1_peak_	Distal Intergen	10	79356591	79396766	40176	1	624845	uc009vaa.1	-3485	ENSMUSG000	Vmn2r82	vomeronasal 2, receptor 82
chr10	79352998	79353097	100	101	79353043	30	7.24477	2.85875	4.21917	Adipose_R2_peak_	Distal Intergen	10	79356591	79396766	40176	1	624845	uc009vaa.1	-3494	ENSMUSG000	Vmn2r82	vomeronasal 2, receptor 82
chr10	79398367	79398553	187	188	79398467	23	9.86443	4.31371	6.70247	Adipose_R1_peak_	Distal Intergen	10	79356591	79396766	40176	1	624845	uc009vaa.1	41776	ENSMUSG000	Vmn2r82	vomeronasal 2, receptor 82
chr10	79447926	79448189	264	265	79448068	50	34.50784	9.16663	30.72138	Adipose_R1_peak_	Distal Intergen	10	79468958	79492154	23197	1	625029	uc009vab.1	-20769	ENSMUSG000	Vmn2r83	vomeronasal 2, receptor 83
chr10	79447987	79448167	181	182	79448064	35	9.95877	3.31984	6.73562	Adipose_R2_peak_	Distal Intergen	10	79468958	79492154	23197	1	625029	uc009vab.1	-20791	ENSMUSG000	Vmn2r83	vomeronasal 2, receptor 83
chr10	79847308	79847461	154	155	79847368	20	7.74501	3.7745	4.70467	Adipose_R1_peak_	Intron (uc029c	10	79854432	79864435	10004	1	19205	uc007gab.2	-6971	ENSMUSG000	Ptbp1	polypyrimidine tract binding protein 1
chr10	79853780	79853961	182	183	79853819	28	6.25023	2.67431	3.31626	Adipose_R2_peak_	Promoter (<=	10	79854432	79864435	10004	1	19205	uc007gab.2	-471	ENSMUSG000	Ptbp1	polypyrimidine tract binding protein 1
chr10	79870868	79871063	196	197	79870961	51	35.56531	9.34637	31.76235	Adipose_R1_peak_	Intron (uc029c	10	79864781	79874366	9586	2	216152	uc007gae.1	3303	ENSMUSG000	Plppr3	phospholipid phosphatase related 3
chr10	79870870	79871064	195	196	79870954	40	12.96465	3.78093	9.57654	Adipose_R2_peak_	Intron (uc029c	10	79864781	79874366	9586	2	216152	uc007gae.1	3302	ENSMUSG000	Plppr3	phospholipid phosphatase related 3
chr10	79959284	79959456	173	174	79959306	16	5.20017	3.05554	2.38734	Adipose_R1_peak_	Promoter (<=	10	79960152	79962355	2204	1	216156	uc007gas.1	-696	ENSMUSG000	Wdr18	WD repeat domain 18
chr10	79961248	79961348	101	102	79961267	26	5.31252	2.48988	2.47999	Adipose_R2_peak_	Promoter (1-2	10	79960152	79962355	2204	1	216156	uc007gas.1	1096	ENSMUSG000	Wdr18	WD repeat domain 18
chr10	79986637	79986765	129	130	79986682	27	5.77405	2.5821	2.88921	Adipose_R2_peak_	Promoter (1-2	10	79988600	79995400	6801	1	12798	uc007gaz.1	-1835	ENSMUSG000	Cnn2	calponin 2
chr10	79987727	79987918	192	193	79987827	28	6.25023	2.67431	3.31626	Adipose_R2_peak_	Promoter (<=	10	79988600	79995400	6801	1	12798	uc007gaz.1	-682	ENSMUSG000	Cnn2	calponin 2
chr10	79990504	79990669	166	167	79990593	20	7.74501	3.7745	4.70467	Adipose_R1_peak_	Promoter (1-2	10	79988600	79995400	6801	1	12798	uc007gaz.1	1904	ENSMUSG000	Cnn2	calponin 2
chr10	79990853	79990995	143	144	79990964	17	5.80341	3.23528	2.92378	Adipose_R1_peak_	Promoter (2-3	10	79988600	79995400	6801	1	12798	uc007gaz.1	2253	ENSMUSG000	Cnn2	calponin 2
chr10	79992226	79992321	96	97	79992250	26	5.31252	2.48988	2.47999	Adipose_R2_peak_	Exon (uc007ga	10	79988600	79995400	6801	1	12798	uc007gaz.1	3626	ENSMUSG000	Cnn2	calponin 2
chr10	79993991	79994346	356	357	79994216	18	6.42934	3.41502	3.48999	Adipose_R1_peak_	Intron (uc007g	10	79997615	80015572	17958	1	27403	uc007gba.1	-3269	ENSMUSG000	Abca7	ATP-binding cassette, sub-family A (ABC1), member 7
chr10	79994166	79994358	193	194	79994311	30	7.24477	2.85875	4.21917	Adipose_R2_peak_	Exon (uc007ga	10	79997615	80015572	17958	1	27403	uc007gba.1	-3257	ENSMUSG000	Abca7	ATP-binding cassette, sub-family A (ABC1), member 7
chr10	79995488	79995600	113	114	79995583	27	5.77405	2.5821	2.88921	Adipose_R2_peak_	Promoter (2-3	10	79997615	80015572	17958	1	27403	uc007gba.1	-2015	ENSMUSG000	Abca7	ATP-binding cassette, sub-family A (ABC1), member 7
chr10	79996933	79997064	132	133	79996976	26	5.31252	2.48988	2.47999	Adipose_R2_peak_	Promoter (<=	10	79997615	80015572	17958	1	27403	uc007gba.1	-551	ENSMUSG000	Abca7	ATP-binding cassette, sub-family A (ABC1), member 7
chr10	80038592	80038720	129	130	80038604	26	5.31252	2.48988	2.47999	Adipose_R2_peak_	Promoter (<=	10	80035953	80039659	3707	2	66420	uc007gbi.1	939	ENSMUSG000	Polr2e	polymerase (RNA) II (DNA directed) polypeptide E
chr10	80041268	80041649	382	383	80041298	27	5.77405	2.5821	2.88921	Adipose_R2_peak_	Promoter (1-2	10	80035953	80039659	3707	2	66420	uc007gbi.1	-1609	ENSMUSG000	Polr2e	polymerase (RNA) II (DNA directed) polypeptide E
chr10	80042099	80042269	171	172	80042123	16	5.20017	3.05554	2.38734	Adipose_R1_peak_	Promoter (2-3	10	80035953	80039659	3707	2	66420	uc007gbi.1	-2440	ENSMUSG000	Polr2e	polymerase (RNA) II (DNA directed) polypeptide E
chr10	80042862	80043012	151	152	80042907	29	6.74062	2.76653	3.75989	Adipose_R2_peak_	Intron (uc029c	10	80035953	80039659	3707	2	66420	uc007gbi.1	-3203	ENSMUSG000	Polr2e	polymerase (RNA) II (DNA directed) polypeptide E
chr10	80044807	80044901	95	96	80044852	27	5.77405	2.5821	2.88921	Adipose_R2_peak_	Promoter (2-3	10	80047166	80056439	9274	1	625249	uc007gbj.1	-2265	ENSMUSG000	Gpx4	glutathione peroxidase 4
chr10	80045806	80045973	168	169	80045886	27	5.77405	2.5821	2.88921	Adipose_R2_peak_	Promoter (1-2	10	80047166	80056439	9274	1	625249	uc007gbj.1	-1193	ENSMUSG000	Gpx4	glutathione peroxidase 4
chr10	80046333	80046502	170	171	80046453	27	5.77405	2.5821	2.88921	Adipose_R2_peak_	Promoter (<=	10	80047166	80056439	9274	1	625249	uc007gbj.1	-664	ENSMUSG000	Gpx4	glutathione peroxidase 4
chr10	80049274	80049410	137	138	80049333	17	5.80341	3.23528	2.92378	Adipose_R1_peak_	Promoter (2-3	10	80047166	80056439	9274	1	625249	uc007gbj.1	2108	ENSMUSG000	Gpx4	glutathione peroxidase 4

chr10	80052635	80052786	152	153	80052695	18	6.42934	3.41502	3.48999	Adipose_R1_peak_	Promoter (<=1	10	80053488	80056439	2952	1	625249	uc007gbk.2	-702	ENSMUSG000	Gpx4	glutathione peroxidase 4
chr10	80055974	80056187	214	215	80056014	26	5.31252	2.48988	2.47999	Adipose_R2_peak_	Promoter (1-2	10	80054019	80056439	2421	1	625249	uc007gbl.2	1955	ENSMUSG000	Gpx4	glutathione peroxidase 4
chr10	80142502	80142596	95	96	80142508	29	5.0715	2.31282	2.28811	Adipose_R2_peak_	Promoter (<=1	10	80142315	80145811	3497	1	66043	uc007gbz.2	187	ENSMUSG000	Atp5d	ATP synthase, H+ transporting, mitochondrial F1 complex, delta subunit
chr10	80143413	80143571	159	160	80143461	21	8.37049	3.92793	5.3235	Adipose_R1_peak_	Promoter (1-2	10	80142315	80145811	3497	1	66043	uc007gbz.2	1098	ENSMUSG000	Atp5d	ATP synthase, H+ transporting, mitochondrial F1 complex, delta subunit
chr10	80143792	80144289	498	499	80143907	31	6.06203	2.50152	3.16628	Adipose_R2_peak_	Promoter (1-2	10	80142315	80145811	3497	1	66043	uc007gbz.2	1477	ENSMUSG000	Atp5d	ATP synthase, H+ transporting, mitochondrial F1 complex, delta subunit
chr10	80144413	80144727	315	316	80144546	22	8.03025	3.68288	4.98681	Adipose_R1_peak_	Promoter (2-3	10	80142315	80145811	3497	1	66043	uc007gbz.2	2098	ENSMUSG000	Atp5d	ATP synthase, H+ transporting, mitochondrial F1 complex, delta subunit
chr10	80145708	80145841	134	135	80145748	30	5.23612	2.32371	2.44684	Adipose_R2_peak_	Promoter (2-3	10	80148272	80158368	10097	1	59090	uc007gca.2	-2431	ENSMUSG000	Midn	midnolin
chr10	80149646	80149799	154	155	80149656	31	5.8988	2.45984	3.00933	Adipose_R2_peak_	Promoter (<=1	10	80149682	80158368	8687	1	59090	uc007gcd.2	0	ENSMUSG000	Midn	midnolin
chr10	80153216	80153507	292	293	80153467	32	5.9608	2.44114	3.06895	Adipose_R2_peak_	Intron (uc007g	10	80149682	80158368	8687	1	59090	uc007gcd.2	3534	ENSMUSG000	Midn	midnolin
chr10	80154139	80154728	590	591	80154287	21	7.15643	3.43266	4.1631	Adipose_R1_peak_	Exon (uc007gc	10	80149682	80158368	8687	1	59090	uc007gcd.2	4457	ENSMUSG000	Midn	midnolin
chr10	80154146	80154488	343	344	80154293	33	6.3281	2.49725	3.39123	Adipose_R2_peak_	Exon (uc007gc	10	80149682	80158368	8687	1	59090	uc007gcd.2	4464	ENSMUSG000	Midn	midnolin
chr10	80154727	80155254	528	529	80154830	38	9.36199	3.0191	6.19395	Adipose_R2_peak_	Exon (uc007gc	10	80149682	80158368	8687	1	59090	uc007gcd.2	5045	ENSMUSG000	Midn	midnolin
chr10	80155751	80156080	330	331	80155985	23	7.73817	3.47788	4.70467	Adipose_R1_peak_	Intron (uc007g	10	80149682	80158368	8687	1	59090	uc007gcd.2	6069	ENSMUSG000	Midn	midnolin
chr10	80158143	80158396	254	255	80158326	18	5.93054	3.19949	3.04898	Adipose_R1_peak_	3' UTR	10	80149682	80158368	8687	1	59090	uc007gcd.2	8461	ENSMUSG000	Midn	midnolin
chr10	80158620	80158886	267	268	80158754	21	7.69435	3.64818	4.69459	Adipose_R1_peak_	Intron (uc029c	10	80149682	80158368	8687	1	59090	uc007gcd.2	8938	ENSMUSG000	Midn	midnolin
chr10	80158669	80158798	130	131	80158689	28	4.87027	2.29141	2.09426	Adipose_R2_peak_	Intron (uc029c	10	80149682	80158368	8687	1	59090	uc007gcd.2	8987	ENSMUSG000	Midn	midnolin
chr10	80159009	80159197	189	190	80159131	35	7.25428	2.64775	4.22846	Adipose_R2_peak_	Intron (uc029c	10	80167841	80171653	3813	1	12696	uc007gce.1	-8644	ENSMUSG000	Cirbp	cold inducible RNA binding protein
chr10	80159436	80159676	241	242	80159590	25	9.52833	3.95561	6.40046	Adipose_R1_peak_	Intron (uc029c	10	80167841	80171653	3813	1	12696	uc007gce.1	-8165	ENSMUSG000	Cirbp	cold inducible RNA binding protein
chr10	80160364	80160650	287	288	80160472	34	6.41246	2.48378	3.47228	Adipose_R2_peak_	Intron (uc029c	10	80167841	80171653	3813	1	12696	uc007gce.1	-7191	ENSMUSG000	Cirbp	cold inducible RNA binding protein
chr10	80161270	80161493	224	225	80161401	20	6.98029	3.45447	4.03326	Adipose_R1_peak_	Intron (uc029c	10	80167841	80171653	3813	1	12696	uc007gce.1	-6348	ENSMUSG000	Cirbp	cold inducible RNA binding protein
chr10	80161679	80161876	198	199	80161808	24	8.85521	3.80347	5.76704	Adipose_R1_peak_	Intron (uc029c	10	80167841	80171653	3813	1	12696	uc007gce.1	-5965	ENSMUSG000	Cirbp	cold inducible RNA binding protein
chr10	80161929	80162141	213	214	80162020	34	7.20088	2.67279	4.20298	Adipose_R2_peak_	Intron (uc029c	10	80167841	80171653	3813	1	12696	uc007gce.1	-5700	ENSMUSG000	Cirbp	cold inducible RNA binding protein
chr10	80162161	80162488	328	329	80162223	17	4.93119	2.85676	2.18808	Adipose_R1_peak_	Intron (uc029c	10	80167841	80171653	3813	1	12696	uc007gce.1	-5353	ENSMUSG000	Cirbp	cold inducible RNA binding protein
chr10	80163115	80163450	336	337	80163288	32	6.09349	2.47404	3.19669	Adipose_R2_peak_	Intron (uc029c	10	80167841	80171653	3813	1	12696	uc007gce.1	-4391	ENSMUSG000	Cirbp	cold inducible RNA binding protein
chr10	80163683	80163944	262	263	80163852	51	15.83584	3.78125	12.32975	Adipose_R2_peak_	Intron (uc029c	10	80167841	80171653	3813	1	12696	uc007gce.1	-3897	ENSMUSG000	Cirbp	cold inducible RNA binding protein
chr10	80164082	80164494	413	414	80164223	46	11.21012	3.07987	7.91535	Adipose_R2_peak_	Intron (uc029c	10	80167841	80171653	3813	1	12696	uc007gce.1	-3347	ENSMUSG000	Cirbp	cold inducible RNA binding protein
chr10	80166451	80166774	324	325	80166530	38	8.60055	2.84388	5.47946	Adipose_R2_peak_	Promoter (1-2	10	80167841	80171653	3813	1	12696	uc007gce.1	-1067	ENSMUSG000	Cirbp	cold inducible RNA binding protein
chr10	80166503	80166801	299	300	80166583	19	6.64025	3.40824	3.69794	Adipose_R1_peak_	Promoter (1-2	10	80167841	80171653	3813	1	12696	uc007gce.1	-1040	ENSMUSG000	Cirbp	cold inducible RNA binding protein
chr10	80166983	80167100	118	119	80166989	31	4.76558	2.17633	2.04003	Adipose_R2_peak_	Promoter (<=1	10	80167841	80171653	3813	1	12696	uc007gce.1	-741	ENSMUSG000	Cirbp	cold inducible RNA binding protein
chr10	80167166	80167462	297	298	80167172	21	5.29844	2.73355	2.48386	Adipose_R1_peak_	Promoter (<=1	10	80167841	80171653	3813	1	12696	uc007gce.1	-379	ENSMUSG000	Cirbp	cold inducible RNA binding protein
chr10	80168036	80168181	146	147	80168116	19	6.42167	3.31653	3.48999	Adipose_R1_peak_	Promoter (<=1	10	80167841	80171653	3813	1	12696	uc007gce.1	195	ENSMUSG000	Cirbp	cold inducible RNA binding protein
chr10	80168320	80168514	195	196	80168385	36	9.65604	3.18728	6.46036	Adipose_R2_peak_	Promoter (<=1	10	80167841	80171653	3813	1	12696	uc007gce.1	479	ENSMUSG000	Cirbp	cold inducible RNA binding protein
chr10	80168431	80168935	505	506	80168513	19	5.7532	3.04278	2.92378	Adipose_R1_peak_	Promoter (<=1	10	80167841	80171653	3813	1	12696	uc007gce.1	590	ENSMUSG000	Cirbp	cold inducible RNA binding protein
chr10	80168676	80169101	426	427	80168814	29	5.69097	2.47798	2.8443	Adipose_R2_peak_	Promoter (<=1	10	80167841	80171653	3813	1	12696	uc007gce.1	835	ENSMUSG000	Cirbp	cold inducible RNA binding protein
chr10	80169467	80169665	199	200	80169588	32	8.21331	3.02175	5.12817	Adipose_R2_peak_	Promoter (1-2	10	80167841	80171653	3813	1	12696	uc007gce.1	1626	ENSMUSG000	Cirbp	cold inducible RNA binding protein
chr10	80169542	80169867	326	327	80169602	19	7.07687	3.59476	4.08458	Adipose_R1_peak_	Promoter (1-2	10	80167841	80171653	3813	1	12696	uc007gce.1	1701	ENSMUSG000	Cirbp	cold inducible RNA binding protein
chr10	80170582	80170712	131	132	80170602	27	5.77405	2.5821	2.88921	Adipose_R2_peak_	Promoter (2-3	10	80172944	80175119	2176	1	69770	uc007gcf.1	-2232	ENSMUSG000	1600002K03Ri	RIKEN cDNA 1600002K03 gene
chr10	80171296	80171539	244	245	80171413	19	7.07687	3.59476	4.08458	Adipose_R1_peak_	Promoter (1-2	10	80172944	80175119	2176	1	69770	uc007gcf.1	-1405	ENSMUSG000	1600002K03Ri	RIKEN cDNA 1600002K03 gene
chr10	80393430	80393615	186	187	80393504	32	8.29282	3.04319	5.18352	Adipose_R2_peak_	Promoter (1-2	10	80395180	80395595	416	2	17192	uc007gdc.1	1980	ENSMUSG000	Mbd3	methyl-CpG binding domain protein 3
chr10	80394096	80394360	265	266	80394253	26	5.31252	2.48988	2.47999	Adipose_R2_peak_	Promoter (1-2	10	80395180	80395595	416	2	17192	uc007gdc.1	1235	ENSMUSG000	Mbd3	methyl-CpG binding domain protein 3
chr10	80395742	80395879	138	139	80395768	25	4.86612	2.39766	2.09018	Adipose_R2_peak_	Promoter (<=1	10	80395180	80395595	416	2	17192	uc007gdc.1	-147	ENSMUSG000	Mbd3	methyl-CpG binding domain protein 3
chr10	80397678	80397918	241	242	80397812	33	7.45652	2.77692	4.42341	Adipose_R2_peak_	Promoter (1-2	10	80392539	80399531	6993	2	17192	uc007gda.2	1613	ENSMUSG000	Mbd3	methyl-CpG binding domain protein 3
chr10	80398050	80398399	350	351	80398129	29	6.74062	2.76653	3.75989	Adipose_R2_peak_	Promoter (1-2	10	80392539	80399531	6993	2	17192	uc007gda.2	1132	ENSMUSG000	Mbd3	methyl-CpG binding domain protein 3
chr10	80398064	80398277	214	215	80398191	20	7.74501	3.7745	4.70467	Adipose_R1_peak_	Promoter (1-2	10	80392539	80399531	6993	2	17192	uc007gda.2	1254	ENSMUSG000	Mbd3	methyl-CpG binding domain protein 3
chr10	80398496	80398655	160	161	80398538	17	5.80341	3.23528	2.92378	Adipose_R1_peak_	Promoter (<=1	10	80392539	80399531	6993	2	17192	uc007gda.2	876	ENSMUSG000	Mbd3	methyl-CpG binding domain protein 3
chr10	80401808	80401903	96	97	80401835	28	6.25023	2.67431	3.31626	Adipose_R2_peak_	Promoter (2-3	10	80392539	80399531	6993	2	17192	uc007gda.2	-2277	ENSMUSG000	Mbd3	methyl-CpG binding domain protein 3
chr10	80402130	80402435	306	307	80402221	19	7.07687	3.59476	4.08458	Adipose_R1_peak_	Promoter (2-3	10	80392539	80399531	6993	2	17192	uc007gda.2	-2599	ENSMUSG000	Mbd3	methyl-CpG binding domain protein 3
chr10	80578224	80578376	153	154	80578345	28	6.25023	2.67431	3.31626	Adipose_R2_peak_	Promoter (<=1	10	80567121	80577296	10176	2	118445	uc007gdt.1	-928	ENSMUSG000	Klf16	Kruppel-like factor 16
chr10	80578310	80578457	148	149	80578424	19	7.07687	3.59476	4.08458	Adipose_R1_peak_	Promoter (1-2	10	80567121	80577296	10176	2	118445	uc007gdt.1	-1014	ENSMUSG000	Klf16	Kruppel-like factor 16
chr10	80579069	80579308	240	241	80579300	27	5.77405	2.5821	2.88921	Adipose_R2_peak_	Promoter (1-2	10	80567121	80577296	10176	2	118445	uc007gdt.1	-1773	ENSMUSG000	Klf16	Kruppel-like factor 16
chr10	80579717	80579841	125	126	80579767	29	6.74062	2.76653	3.75989	Adipose_R2_peak_	Promoter (2-3	10	80567121	80577296	10176	2	118445	uc007gdt.1	-2421	ENSMUSG000	Klf16	Kruppel-like factor 16
chr10	80580070	80580216	147	148	80580162	18	6.42934	3.41502	3.48999	Adipose_R1_peak_	Promoter (2-3	10	80567121	80577296	10176	2	118445	uc007gdt.1	-2774	ENSMUSG000	Klf16	Kruppel-like factor 16
chr10	80581230	80581475	246	247	80581384	30	7.24477	2.85875	4.21917	Adipose_R2_peak_	Downstream (	10	80567121	80577296	10176	2	118445	uc007gdt.1	-3934	ENSMUSG000	Klf16	Kruppel-like factor 16
chr10	80583280	80583388	109	110	80583307	26	5.31252	2.48988	2.47999	Adipose_R2_peak_	Downstream (	10	80567121	80577296	10176	2	118445	uc007gdt.1	-5984	ENSMUSG000	Klf16	Kruppel-like factor 16
chr10	80617748	80617956	209	210	80617825	25	4.86612	2.39766	2.09018	Adipose_R2_peak_	Distal Intergen	10	80622780	80640771	17992	1	103236	uc007gea.2	-4824	ENSMUSG000	Csnk1g2	casein kinase 1, gamma 2
chr10	80622192	80622330	139	140	80622241	18	6.42934	3.41502	3.48999	Adipose_R1_peak_	Promoter (<=1	10	80622780	80640771	17992	1	103236	uc007gea.2	-450	ENSMUSG000	Csnk1g2	casein kinase 1, gamma 2
chr10	80669578	80669720	143	144	80669625	26	5.31252	2.48988	2.47999	Adipose_R2_peak_	Exon (uc007ge	10	80671736	80675940	4205	2	17347	uc007geg.1	6220	ENSMUSG000	Mknk2	MAP kinase-interacting serine/threonine kinase 2
chr10	80670840	80670979	140	141	80670941	25	4.86612	2.39766	2.09018	Adipose_R2_peak_	Intron (uc007g	10	80671736	80675940	4205	2	17347	uc007geg.1	4961	ENSMUSG000	Mknk2	MAP kinase-interacting serine/threonine kinase 2
chr10	80671410	80671552	143	144	80671520	18	6.42934	3.41502	3.48999	Adipose_R1_peak_	Intron (uc007g	10	80671736	80675940	4205	2	17347	uc007geg.1	4388	ENSMUSG000	Mknk2	MAP kinase-interacting serine/threonine kinase 2
chr10	80672737	80672914	178	179	80672824	30	7.24477	2.85875	4.21917	Adipose_R2_peak_	Intron (uc007g	10	80671736	80675940	4205	2	17347	uc007geg.1	3026	ENSMUSG000	Mknk2	MAP kinase-interacting serine/threonine kinase 2
chr10	80675370	80675711	342	343	80675480	29	6.74062	2.76653	3.75989	Adipose_R2_peak_	Promoter (<=1	10	80671736	80675940	4205	2	17347	uc007geg.1	229	ENSMUSG000	Mknk2	MAP kinase-interacting serine/threonine kinase 2
chr10	80676776	80676993	218	219	80676861	36	10.5378	3.41206	7.27949	Adipose_R2_peak_	Promoter (<=1	10	80665318	80676293	10976	2	17347	uc007gef.3	-483	ENSMUSG000	Mknk2	MAP kinase-interacting serine/threonine kinase 2
chr10	80677341	80677576	236	237	80677488	31	7.7623	2.95097	4.69388	Adipose_R2_peak_	Promoter (1-2	10	80665318	80676293	10976	2	17347	uc007gef.3	-1048	ENSMUSG000	Mknk2	MAP kinase-interacting serine/threonine kinase 2
chr10	80677909	80678159	251	252	80678070	37	11.12817	3.50427	7.83613	Adipose_R2_peak_	Promoter (1-2	10	80665318	80676293	10976	2	17347	uc007gef.3	-1616	ENSMUSG000	Mknk2	MAP kinase-interacting serine/threonine kinase 2
chr10	80677922	80678146	225	226	80678017	23	8.56759	3.79322	5.48223	Adipose_R1_peak_	Promoter (1-2	10	80665318	80676293	10976	2	17347	uc007gef.3	-1629	ENSMUSG000	Mknk2	MAP kinase-interacting serine/threonine kinase 2
chr10	80753263	80753533	271	272	80753348	17	5.80341	3.23528	2.92378	Adipose_R1_peak_	Promoter (1-2	10	80755206	80792773	37568	1	208266	uc033frf.1	-1673	ENSMUSG000	Dot1l	DOT1-like, histone H3 methyltransferase (S. cerevisiae)
chr10	80753811	80754131	321	322	80753872	22	9.13959	4.13397	6.01526	Adipose_R1_peak_	Promoter (1-2	10	80755206	80792773	37568	1	208266	uc033frf.1	-1075	ENSMUSG000	Dot1l	DOT1-like, histone H3 methyltransferase (S. cerevisiae)
chr10	80756266	80756424	159	160	80756337	29	6.74062	2.76653	3.75989	Adipose_R2_peak_	Promoter (1-2	10	80755206	80792773	37568	1	208266	uc033frf.1	1060	ENSMUSG000	Dot1l	DOT1-like, histone H3 methyltransferase (S. cerevisiae)
chr10	80928457	80928575	119	120	80928513	25	4.86612	2.39766	2.09018	Adipose_R2_peak_	Promoter (1-2	10	80930091	80932204	2114	1	17873	uc007gfl.1	-1516	ENSMUSG000	Gadd45b	growth arrest and DNA-damage-inducible 45 beta
chr10	80934221	80934393	173	174	80934338	19	7.07687	3.59476	4.08458	Adipose_R1_peak_	Distal Intergen	10	80930091	80932204	2114	1	17873	uc007gfl.1	4130	ENSMUSG000	Gadd45b	growth arrest and DNA-damage-inducible 45 beta
chr10	80934819	80934990	172	173	80934937	31	7.7623	2.95097	4.69388	Adipose_R2_peak_	Distal Intergen	10	80930091	80932204	2114	1	17873	uc007gfl.1	4728	ENSMUSG000	Gadd45b	growth arrest and DNA-damage-inducible 45 beta
chr10	80936911	80937058	148	149	80937032	27	5.77405	2.5821	2.88921	Adipose_R2_peak_	Distal Intergen	10	80930091	80932204	2114	1	17873	uc007gfl.1	6820	ENSMUSG000	Gadd45b	growth arrest and DNA-damage-inducible 45 beta
chr10	80937264	80937409	146	147	80937318	29	6.74062	2.76653	3.75989	Adipose_R2_peak_	Distal Intergen	10	80930091	80932204	2114	1	17873	uc007gfl.1	7173	ENSMUSG000	Gadd45b	growth arrest and DNA-damage-inducible 45 beta
chr10	81062610	81062714	105	106	81062627	25	4.86612	2.39766	2.09018	Adipose_R2_peak_	Promoter (2-3	10	81044073	81060154	16082	2	52551	uc007gft.1	-2456	ENSMUSG000	Sgta	small glutamine-rich tetratricopeptide repeat (TPR)-containing, alpha
chr10	81062812	81062953	142	143	81062837	16	5.20017	3.05554	2.38734	Adipose_R1_peak_	Promoter (2-3	10	81044073	81060154	16082	2	52551	uc007gft.1	-2658	ENSMUSG000	Sgta	small glutamine-rich tetratricopeptide repeat (TPR)-containing, alpha
chr10	81064194	81064362	169	170	81064311	30	7.24477	2.85875	4.21917	Adipose_R2_peak_	Distal Intergen	10	81044073	81060154	16082	2	52551	uc007gft.1	-4040	ENSMUSG000	Sgta	small glutamine-rich tetratricopeptide repeat (TPR)-containing, alpha
chr10	81066049	81066306	258	259	81066058	25	4.86612	2.39766	2.09018	Adipose_R2_peak_	Distal Intergen	10	81070083	81074859	4777	1	50492	uc057axa.1	-3777	ENSMUSG000	Thop1	thimet oligopeptidase 1
chr10	81066734	81066847	114	115	81066769	28	6.25023	2.67431	3.31626	Adipose_R2_peak_	Distal Intergen	10	81070083	81074859	4777	1	50492	uc057axa.1	-3236	ENSMUSG000	Thop1	thimet oligopeptidase 1
chr10	81066912	81067049	138	139	81066947	17	5.80341	3.23528	2.92378	Adipose_R1_peak_	Distal Intergen	10	81070083	81074859	4777	1	50492	uc057axa.1	-3034	ENSMUSG000	Thop1	thimet oligopeptidase 1
chr10	81069129	81069381	253	254	81069265	34	9.39139	3.22762	6.20474	Adipose_R2_peak_	Promoter (<=1	10	81070083	81074859	4777	1	50492	uc057axa.1	-702	ENSMUSG000	Thop1	thimet oligopeptidase 1
chr10	81071416	81071569	154	155	81071441	26	5.31252	2.48988	2.47999	Adipose_R2_peak_	Promoter (1-2	10	81070083	81074859	4777	1	50492	uc057axa.1	1333	ENSMUSG000	Thop1	thimet oligopeptidase 1
chr10	81160179	81160295	117	118	81160185	25	4.86612	2.39766	2.09018	Adipose_R2_peak_	Intron (uc007g	10	81153966	81167720	13755	2	59004	uc007ggc.3	7425	ENSMUSG000	Pias4	protein inhibitor of activated STAT 4
chr10	81165152	81165251	100	101	81165169	26	5.31252	2.48988	2.47999	Adipose_R2_peak_	Promoter (2-3	10	81153966	81167720	13755	2	59004	uc007ggc.3	2469	ENSMUSG000	Pias4	protein inhibitor of activated STAT 4
chr10	81168038	81168238	201	202	81168154	21	8.43286	3.95423	5.34896	Adipose_R1_peak_	Promoter (<=1	10	81153966	81167720	13755	2	59004	uc007ggc.3	-318	ENSMUSG000	Pias4	protein inhibitor of activated STAT 4
chr10	81168056	81168174	119	120	81168123	27	5.77405	2.5821	2.88921	Adipose_R2_peak_	Promoter (<=1	10	81153966	81167720	13755	2	59004	uc007ggc.3	-336	ENSMUSG000	Pias4	protein inhibitor of activated STAT 4
chr10	81169131	81169779	649	650	81169235	32	15.02004	5.14898	11.66647	Adipose_R1_peak_	Promoter (1-2	10	81153966	81167720	13755	2	59004	uc007ggc.3	-1411	ENSMUSG000	Pias4	protein inhibitor of activated STAT 4
chr10	81169582	81169789	208	209	81169707	31	7.7623	2.95097	4.69388	Adipose_R2_peak_	Promoter (1-2	10	81153966	81167720	13755	2	59004	uc007ggc.3	-1862	ENSMUSG000	Pias4	protein inhibitor of activated STAT 4
chr10	81170020	81170367	348	349	81170150	18	6.42934	3.41502	3.48999	Adipose_R1_peak_	Promoter (2-3	10	81153966	81167720	13755	2	59004	uc007ggc.3	-2300	ENSMUSG000	Pias4	protein inhibitor of activated STAT 4
chr10	81171643	81171870	228	229	81171707	28	6.25023	2.67431	3.31626	Adipose_R2_peak_	Distal Intergen	10	81153966	81167720	13755	2	59004	uc007ggc.3	-3923	ENSMUSG000	Pias4	protein inhibitor of activated STAT 4
chr10	81174023	81174168	146	147	81174087	20	7.74501	3.7745	4.70467	Adipose_R1_peak_	Promoter (2-3	10	81176631	81182509	5879	1	13629	uc007gge.2	-2463	ENSMUSG000	Eef2	eukaryotic translation elongation factor 2
chr10	81174386	81174501	116	117	81174392	27	5.42376	2.4814	2.58701	Adipose_R2_peak_	Promoter (2-3	10	81176631	81182509	5879	1	13629	uc007gge.2	-2130	ENSMUSG000	Eef2	eukaryotic translation elongation factor 2
chr10	81174573	81174829	257	258	81174674	17	5.76977	3.22034	2.92378	Adipose_R1_peak_	Promoter (1-2	10	81176631	81182509	5879	1	13629	uc007gge.2	-1802	ENSMUSG000	Eef2	eukaryotic translation elongation factor 2
chr10	81174651	81174821	171	172	81174666	27	5.77405	2.5821	2.88921	Adipose_R2_peak_	Promoter (1-2	10	81176631	81182509	5879	1	13629	uc007gge.2	-1810	ENSMUSG000	Eef2	eukaryotic translation elongation factor 2
chr10	81175257	81175496	240	241	81175393	31	7.68997	2.93123	4.64789	Adipose_R2_peak_	Promoter (1-2	10	81176631	81182509	5879	1	13629	uc007gge.2	-1135	ENSMUSG000	Eef2	eukaryotic translation elongation factor 2

chr10	81175291	81175631	341	342	81175468	38	22.52591	7.00978	18.96	Adipose_R1_peak_	Promoter (<=1	10	81176631	81182509	5879	1	13629	uc007gge.2	-1000	ENSMUSG000	Eef2	eukaryotic translation elongation factor 2
chr10	81175707	81176130	424	425	81175802	29	6.59544	2.72591	3.64829	Adipose_R2_peak_	Promoter (<=1	10	81176631	81182509	5879	1	13629	uc007gge.2	-501	ENSMUSG000	Eef2	eukaryotic translation elongation factor 2
chr10	81177579	81177751	173	174	81177647	35	7.22215	2.64016	4.21917	Adipose_R2_peak_	Promoter (<=1	10	81177656	81178234	579	1	13629	uc007ggf.1	0	ENSMUSG000	Eef2	eukaryotic translation elongation factor 2
chr10	81178079	81178235	157	158	81178175	52	16.63719	3.89062	13.10354	Adipose_R2_peak_	Promoter (<=1	10	81177656	81178234	579	1	13629	uc007ggf.1	423	ENSMUSG000	Eef2	eukaryotic translation elongation factor 2
chr10	81178436	81178580	145	146	81178501	31	6.16503	2.52794	3.26524	Adipose_R2_peak_	Promoter (<=1	10	81178961	81179013	53	1	1.00E+08	uc011xjd.1	-381	ENSMUSG000	Snord37	small nucleolar RNA, C/D box 37
chr10	81179202	81179354	153	154	81179286	33	7.70684	2.84055	4.66368	Adipose_R2_peak_	Promoter (<=1	10	81178961	81179013	53	1	1.00E+08	uc011xjd.1	241	ENSMUSG000	Snord37	small nucleolar RNA, C/D box 37
chr10	81179999	81180139	141	142	81180088	19	7.07687	3.59476	4.08458	Adipose_R1_peak_	Promoter (1-2	10	81178961	81179013	53	1	1.00E+08	uc011xjd.1	1038	ENSMUSG000	Snord37	small nucleolar RNA, C/D box 37
chr10	81181232	81181447	216	217	81181289	29	5.28962	2.37058	2.47999	Adipose_R2_peak_	Promoter (1-2	10	81183007	81192182	9176	1	13144	uc007ggg.2	-1560	ENSMUSG000	Dapk3	death-associated protein kinase 3
chr10	81182644	81182741	98	99	81182677	30	4.79142	2.21013	2.06474	Adipose_R2_peak_	Promoter (<=1	10	81183007	81192182	9176	1	13144	uc007ggg.2	-266	ENSMUSG000	Dapk3	death-associated protein kinase 3
chr10	81183312	81183486	175	176	81183333	26	5.31252	2.48988	2.47999	Adipose_R2_peak_	Promoter (<=1	10	81183263	81193197	9935	1	13144	uc007ggk.2	49	ENSMUSG000	Dapk3	death-associated protein kinase 3
chr10	81185880	81186059	180	181	81185940	30	7.24477	2.85875	4.21917	Adipose_R2_peak_	Promoter (1-2	10	81183918	81193197	9280	1	13144	uc007ggj.2	1962	ENSMUSG000	Dapk3	death-associated protein kinase 3
chr10	81186613	81186777	165	166	81186682	17	5.80341	3.23528	2.92378	Adipose_R1_peak_	Promoter (2-3	10	81183918	81193197	9280	1	13144	uc007ggj.2	2695	ENSMUSG000	Dapk3	death-associated protein kinase 3
chr10	81189075	81189207	133	134	81189098	16	5.20017	3.05554	2.38734	Adipose_R1_peak_	Intron (uc007g	10	81183918	81193197	9280	1	13144	uc007ggj.2	5157	ENSMUSG000	Dapk3	death-associated protein kinase 3
chr10	81378938	81379177	240	241	81378967	25	4.86612	2.39766	2.09018	Adipose_R2_peak_	Promoter (<=1	10	81366879	81378370	11492	2	56371	uc007ght.1	-568	ENSMUSG000	Fzr1	fizzy/cell division cycle 20 related 1 (Drosophila)
chr10	81380069	81380205	137	138	81380137	28	6.25023	2.67431	3.31626	Adipose_R2_peak_	Promoter (1-2	10	81366879	81378370	11492	2	56371	uc007ght.1	-1699	ENSMUSG000	Fzr1	fizzy/cell division cycle 20 related 1 (Drosophila)
chr10	81380451	81380623	173	174	81380495	30	7.24477	2.85875	4.21917	Adipose_R2_peak_	Promoter (2-3	10	81366879	81378370	11492	2	56371	uc007ght.1	-2081	ENSMUSG000	Fzr1	fizzy/cell division cycle 20 related 1 (Drosophila)
chr10	81381230	81381378	149	150	81381286	16	5.20017	3.05554	2.38734	Adipose_R1_peak_	Promoter (2-3	10	81366879	81378370	11492	2	56371	uc007ght.1	-2860	ENSMUSG000	Fzr1	fizzy/cell division cycle 20 related 1 (Drosophila)
chr10	81381680	81381880	201	202	81381785	22	9.13959	4.13397	6.01526	Adipose_R1_peak_	Promoter (2-3	10	81384428	81388352	3925	1	102115	uc007ghu.2	-2548	ENSMUSG000	Dohh	deoxyhypusine hydroxylase/monooxygenase
chr10	81381899	81382065	167	168	81381922	25	4.86612	2.39766	2.09018	Adipose_R2_peak_	Promoter (2-3	10	81384428	81388352	3925	1	102115	uc007ghu.2	-2363	ENSMUSG000	Dohh	deoxyhypusine hydroxylase/monooxygenase
chr10	81382076	81382212	137	138	81382156	22	8.81095	3.99764	5.72328	Adipose_R1_peak_	Promoter (2-3	10	81384428	81388352	3925	1	102115	uc007ghu.2	-2216	ENSMUSG000	Dohh	deoxyhypusine hydroxylase/monooxygenase
chr10	81382727	81382851	125	126	81382820	30	7.24477	2.85875	4.21917	Adipose_R2_peak_	Promoter (1-2	10	81384428	81388352	3925	1	102115	uc007ghu.2	-1577	ENSMUSG000	Dohh	deoxyhypusine hydroxylase/monooxygenase
chr10	81383428	81383657	230	231	81383539	30	7.24477	2.85875	4.21917	Adipose_R2_peak_	Promoter (<=1	10	81384428	81388352	3925	1	102115	uc007ghu.2	-771	ENSMUSG000	Dohh	deoxyhypusine hydroxylase/monooxygenase
chr10	81383484	81383656	173	174	81383596	20	7.74501	3.7745	4.70467	Adipose_R1_peak_	Promoter (<=1	10	81384428	81388352	3925	1	102115	uc007ghu.2	-772	ENSMUSG000	Dohh	deoxyhypusine hydroxylase/monooxygenase
chr10	81383866	81384142	277	278	81384058	23	9.86443	4.31371	6.70247	Adipose_R1_peak_	Promoter (<=1	10	81384428	81388352	3925	1	102115	uc007ghu.2	-286	ENSMUSG000	Dohh	deoxyhypusine hydroxylase/monooxygenase
chr10	81407241	81407378	138	139	81407294	31	7.7623	2.95097	4.69388	Adipose_R2_peak_	Intron (uc007g	10	81416240	81416297	58	2	1.02E+08	uc033frn.1	8919	ENSMUSG000	Mir1191b	microRNA 1191b
chr10	81410201	81410585	385	386	81410424	20	7.74501	3.7745	4.70467	Adipose_R1_peak_	Intron (uc007g	10	81416240	81416297	58	2	1.02E+08	uc033frn.1	5712	ENSMUSG000	Mir1191b	microRNA 1191b
chr10	81414556	81414722	167	168	81414675	20	7.20926	3.54892	4.21524	Adipose_R1_peak_	Promoter (1-2	10	81416240	81416297	58	2	1.02E+08	uc033frn.1	1575	ENSMUSG000	Mir1191b	microRNA 1191b
chr10	81414631	81414753	123	124	81414667	26	5.31252	2.48988	2.47999	Adipose_R2_peak_	Promoter (1-2	10	81416240	81416297	58	2	1.02E+08	uc033frn.1	1544	ENSMUSG000	Mir1191b	microRNA 1191b
chr10	81415861	81415991	131	132	81415921	26	5.31252	2.48988	2.47999	Adipose_R2_peak_	Promoter (<=1	10	81416240	81416297	58	2	1.02E+08	uc033frn.1	306	ENSMUSG000	Mir1191b	microRNA 1191b
chr10	81416251	81416441	191	192	81416295	28	6.25023	2.67431	3.31626	Adipose_R2_peak_	Promoter (<=1	10	81416240	81416297	58	2	1.02E+08	uc033frn.1	0	ENSMUSG000	Mir1191b	microRNA 1191b
chr10	81417830	81417991	162	163	81417924	32	8.29282	3.04319	5.18352	Adipose_R2_peak_	Promoter (1-2	10	81416240	81416297	58	2	1.02E+08	uc033frn.1	-1533	ENSMUSG000	Mir1191b	microRNA 1191b
chr10	81419453	81419676	224	225	81419578	30	7.24477	2.85875	4.21917	Adipose_R2_peak_	Promoter (1-2	10	81396191	81420798	24608	2	18029	uc007ghx.1	1122	ENSMUSG000	Nfic	nuclear factor I/C
chr10	81421210	81421402	193	194	81421252	29	6.74062	2.76653	3.75989	Adipose_R2_peak_	Promoter (<=1	10	81396191	81420798	24608	2	18029	uc007ghx.1	-412	ENSMUSG000	Nfic	nuclear factor I/C
chr10	81421554	81421709	156	157	81421596	26	5.31252	2.48988	2.47999	Adipose_R2_peak_	Promoter (<=1	10	81396191	81420798	24608	2	18029	uc007ghx.1	-756	ENSMUSG000	Nfic	nuclear factor I/C
chr10	81421597	81421759	163	164	81421657	19	6.24956	3.24509	3.36288	Adipose_R1_peak_	Promoter (<=1	10	81396191	81420798	24608	2	18029	uc007ghx.1	-799	ENSMUSG000	Nfic	nuclear factor I/C
chr10	81422127	81422279	153	154	81422176	28	6.25023	2.67431	3.31626	Adipose_R2_peak_	Promoter (1-2	10	81396191	81420798	24608	2	18029	uc007ghx.1	-1329	ENSMUSG000	Nfic	nuclear factor I/C
chr10	81422848	81423109	262	263	81423001	28	6.25023	2.67431	3.31626	Adipose_R2_peak_	Promoter (2-3	10	81396191	81420798	24608	2	18029	uc007ghx.1	-2050	ENSMUSG000	Nfic	nuclear factor I/C
chr10	81423718	81424008	291	292	81423817	33	8.83596	3.1354	5.68652	Adipose_R2_peak_	Promoter (2-3	10	81396191	81420798	24608	2	18029	uc007ghx.1	-2920	ENSMUSG000	Nfic	nuclear factor I/C
chr10	81424832	81425011	180	181	81424851	25	4.86612	2.39766	2.09018	Adipose_R2_peak_	Promoter (2-3	10	81396191	81427173	30983	2	18029	uc007ghy.1	2162	ENSMUSG000	Nfic	nuclear factor I/C
chr10	81427666	81427949	284	285	81427714	28	6.25023	2.67431	3.31626	Adipose_R2_peak_	Promoter (<=1	10	81396191	81427173	30983	2	18029	uc007ghy.1	-493	ENSMUSG000	Nfic	nuclear factor I/C
chr10	81428262	81428390	129	130	81428286	28	6.25023	2.67431	3.31626	Adipose_R2_peak_	Promoter (1-2	10	81396191	81427173	30983	2	18029	uc007ghy.1	-1089	ENSMUSG000	Nfic	nuclear factor I/C
chr10	81428523	81428709	187	188	81428592	26	5.31252	2.48988	2.47999	Adipose_R2_peak_	Promoter (1-2	10	81396191	81427173	30983	2	18029	uc007ghy.1	-1350	ENSMUSG000	Nfic	nuclear factor I/C
chr10	81428859	81429094	236	237	81428947	31	7.7623	2.95097	4.69388	Adipose_R2_peak_	Promoter (1-2	10	81396191	81427173	30983	2	18029	uc007ghy.1	-1686	ENSMUSG000	Nfic	nuclear factor I/C
chr10	81429401	81429533	133	134	81429423	17	5.1732	2.95994	2.38734	Adipose_R1_peak_	Promoter (1-2	10	81396191	81430794	34604	2	18029	uc007gia.1	1261	ENSMUSG000	Nfic	nuclear factor I/C
chr10	81432186	81432354	169	170	81432269	20	7.74501	3.7745	4.70467	Adipose_R1_peak_	Promoter (1-2	10	81396191	81430794	34604	2	18029	uc007gia.1	-1392	ENSMUSG000	Nfic	nuclear factor I/C
chr10	81432204	81432426	223	224	81432322	33	8.83596	3.1354	5.68652	Adipose_R2_peak_	Promoter (1-2	10	81396191	81430794	34604	2	18029	uc007gia.1	-1410	ENSMUSG000	Nfic	nuclear factor I/C
chr10	81432685	81432972	288	289	81432844	33	8.83596	3.1354	5.68652	Adipose_R2_peak_	Promoter (1-2	10	81396191	81430794	34604	2	18029	uc007gia.1	-1891	ENSMUSG000	Nfic	nuclear factor I/C
chr10	81432749	81432988	240	241	81432883	24	10.09137	4.28424	6.92742	Adipose_R1_peak_	Promoter (1-2	10	81396191	81430794	34604	2	18029	uc007gia.1	-1955	ENSMUSG000	Nfic	nuclear factor I/C
chr10	81543478	81543684	207	208	81543571	30	7.24477	2.85875	4.21917	Adipose_R2_peak_	Promoter (1-2	10	81528732	81545046	16315	2	14672	uc007gil.1	1362	ENSMUSG000	Gna11	guanine nucleotide binding protein, alpha 11
chr10	81546623	81546744	122	123	81546634	25	4.86612	2.39766	2.09018	Adipose_R2_peak_	Promoter (1-2	10	81528732	81545046	16315	2	14672	uc007gil.1	-1577	ENSMUSG000	Gna11	guanine nucleotide binding protein, alpha 11
chr10	81548613	81548895	283	284	81548701	28	13.73721	5.2124	10.41463	Adipose_R1_peak_	Distal Intergen	10	81528732	81545046	16315	2	14672	uc007gil.1	-3567	ENSMUSG000	Gna11	guanine nucleotide binding protein, alpha 11
chr10	81550007	81550222	216	217	81550123	30	7.24477	2.85875	4.21917	Adipose_R2_peak_	Distal Intergen	10	81528732	81545046	16315	2	14672	uc007gil.1	-4961	ENSMUSG000	Gna11	guanine nucleotide binding protein, alpha 11
chr10	81551181	81551319	139	140	81551264	27	5.77405	2.5821	2.88921	Adipose_R2_peak_	Distal Intergen	10	81528732	81545046	16315	2	14672	uc007gil.1	-6135	ENSMUSG000	Gna11	guanine nucleotide binding protein, alpha 11
chr10	81568774	81568945	172	173	81568808	17	4.9032	2.84492	2.16055	Adipose_R1_peak_	Distal Intergen	10	81575287	81590473	15187	1	21886	uc007gio.2	-6342	ENSMUSG000	Tle2	transducin-like enhancer of split 2
chr10	81569651	81569754	104	105	81569696	25	4.86612	2.39766	2.09018	Adipose_R2_peak_	Distal Intergen	10	81575287	81590473	15187	1	21886	uc007gio.2	-5533	ENSMUSG000	Tle2	transducin-like enhancer of split 2
chr10	81570235	81570556	322	323	81570302	31	7.7623	2.95097	4.69388	Adipose_R2_peak_	Distal Intergen	10	81575287	81590473	15187	1	21886	uc007gio.2	-4731	ENSMUSG000	Tle2	transducin-like enhancer of split 2
chr10	81571064	81571279	216	217	81571169	35	9.95877	3.31984	6.73562	Adipose_R2_peak_	Distal Intergen	10	81575287	81590473	15187	1	21886	uc007gio.2	-4008	ENSMUSG000	Tle2	transducin-like enhancer of split 2
chr10	81571444	81571587	144	145	81571494	16	5.20017	3.05554	2.38734	Adipose_R1_peak_	Distal Intergen	10	81575287	81590473	15187	1	21886	uc007gio.2	-3700	ENSMUSG000	Tle2	transducin-like enhancer of split 2
chr10	81601173	81601516	344	345	81601215	20	7.74501	3.7745	4.70467	Adipose_R1_peak_	Promoter (<=1	10	81590905	81600900	9996	2	114606	uc007giu.1	-273	ENSMUSG000	Tle6	transducin-like enhancer of split 6
chr10	81601876	81602100	225	226	81601955	36	8.32333	2.8617	5.21282	Adipose_R2_peak_	Promoter (<=1	10	81590905	81600900	9996	2	114606	uc007giu.1	-976	ENSMUSG000	Tle6	transducin-like enhancer of split 6
chr10	81601926	81602133	208	209	81602030	22	9.13959	4.13397	6.01526	Adipose_R1_peak_	Promoter (1-2	10	81590905	81600900	9996	2	114606	uc007giu.1	-1026	ENSMUSG000	Tle6	transducin-like enhancer of split 6
chr10	81602678	81602813	136	137	81602732	33	8.83596	3.1354	5.68652	Adipose_R2_peak_	Promoter (1-2	10	81590905	81600900	9996	2	114606	uc007giu.1	-1778	ENSMUSG000	Tle6	transducin-like enhancer of split 6
chr10	81602783	81603231	449	450	81602943	22	9.13959	4.13397	6.01526	Adipose_R1_peak_	Promoter (1-2	10	81590905	81600900	9996	2	114606	uc007giu.1	-1883	ENSMUSG000	Tle6	transducin-like enhancer of split 6
chr10	81603601	81603822	222	223	81603711	28	6.25023	2.67431	3.31626	Adipose_R2_peak_	Promoter (2-3	10	81606308	81609836	3529	1	268319	uc007giw.2	-2486	ENSMUSG000	BC025920	zinc finger protein pseudogene
chr10	81605769	81606002	234	235	81605840	20	7.74501	3.7745	4.70467	Adipose_R1_peak_	Promoter (<=1	10	81606308	81609836	3529	1	268319	uc007giw.2	-306	ENSMUSG000	BC025920	zinc finger protein pseudogene
chr10	81662003	81662173	171	172	81662085	34	9.39139	3.22762	6.20474	Adipose_R2_peak_	Exon (uc007gj	10	81649069	81667361	18293	1	1.00E+08	uc007gje.1	12934	ENSMUSG000	Gm10778	predicted gene 10778
chr10	81663335	81663508	174	175	81663408	32	6.85346	2.6654	3.86863	Adipose_R2_peak_	Exon (uc007gj	10	81649069	81667361	18293	1	1.00E+08	uc007gje.1	14266	ENSMUSG000	Gm10778	predicted gene 10778
chr10	81675169	81675307	139	140	81675219	36	10.5378	3.41206	7.27949	Adipose_R2_peak_	Distal Intergen	10	81649069	81667361	18293	1	1.00E+08	uc007gje.1	26100	ENSMUSG000	Gm10778	predicted gene 10778
chr10	81680945	81681145	201	202	81681022	22	8.62	3.91948	5.53422	Adipose_R1_peak_	Distal Intergen	10	81704825	81721976	17152	1	73610	uc007gjf.2	-23680	ENSMUSG000	Zfp433	zinc finger protein 433
chr10	81681370	81681634	265	266	81681431	30	5.82459	2.47643	2.93775	Adipose_R2_peak_	Distal Intergen	10	81704825	81721976	17152	1	73610	uc007gjf.2	-23191	ENSMUSG000	Zfp433	zinc finger protein 433
chr10	81682211	81682400	190	191	81682321	34	9.39139	3.22762	6.20474	Adipose_R2_peak_	Distal Intergen	10	81704825	81721976	17152	1	73610	uc007gjf.2	-22425	ENSMUSG000	Zfp433	zinc finger protein 433
chr10	81686649	81686803	155	156	81686688	26	5.31252	2.48988	2.47999	Adipose_R2_peak_	Distal Intergen	10	81704825	81721976	17152	1	73610	uc007gjf.2	-18022	ENSMUSG000	Zfp433	zinc finger protein 433
chr10	81688744	81689162	419	420	81688833	39	12.34187	3.68871	8.98501	Adipose_R2_peak_	Distal Intergen	10	81704825	81721976	17152	1	73610	uc007gjf.2	-15663	ENSMUSG000	Zfp433	zinc finger protein 433
chr10	81689668	81690290	623	624	81689914	56	16.19221	3.60557	12.67912	Adipose_R2_peak_	Distal Intergen	10	81704825	81721976	17152	1	73610	uc007gjf.2	-14535	ENSMUSG000	Zfp433	zinc finger protein 433
chr10	81690400	81691168	769	770	81690706	27	5.31922	2.45161	2.4863	Adipose_R2_peak_	Distal Intergen	10	81704825	81721976	17152	1	73610	uc007gjf.2	-13657	ENSMUSG000	Zfp433	zinc finger protein 433
chr10	81692020	81692198	179	180	81692056	24	7.78129	3.4093	4.74063	Adipose_R1_peak_	Distal Intergen	10	81704825	81721976	17152	1	73610	uc007gjf.2	-12627	ENSMUSG000	Zfp433	zinc finger protein 433
chr10	81693879	81693983	105	106	81693885	25	4.86612	2.39766	2.09018	Adipose_R2_peak_	Distal Intergen	10	81704825	81721976	17152	1	73610	uc007gjf.2	-10842	ENSMUSG000	Zfp433	zinc finger protein 433
chr10	81694523	81694688	166	167	81694585	39	7.6479	2.59782	4.60695	Adipose_R2_peak_	Distal Intergen	10	81704825	81721976	17152	1	73610	uc007gjf.2	-10137	ENSMUSG000	Zfp433	zinc finger protein 433
chr10	81695034	81695552	519	520	81695181	37	6.96264	2.51268	3.97376	Adipose_R2_peak_	Distal Intergen	10	81704825	81721976	17152	1	73610	uc007gjf.2	-9273	ENSMUSG000	Zfp433	zinc finger protein 433
chr10	81695871	81695998	128	129	81695928	30	7.24477	2.85875	4.21917	Adipose_R2_peak_	Distal Intergen	10	81704825	81721976	17152	1	73610	uc007gjf.2	-8827	ENSMUSG000	Zfp433	zinc finger protein 433
chr10	81696628	81696850	223	224	81696749	28	6.25023	2.67431	3.31626	Adipose_R2_peak_	Distal Intergen	10	81704825	81721976	17152	1	73610	uc007gjf.2	-7975	ENSMUSG000	Zfp433	zinc finger protein 433
chr10	81696952	81697219	268	269	81697115	37	11.12817	3.50427	7.83613	Adipose_R2_peak_	Distal Intergen	10	81704825	81721976	17152	1	73610	uc007gjf.2	-7606	ENSMUSG000	Zfp433	zinc finger protein 433
chr10	81697484	81697644	161	162	81697578	35	9.45925	3.19038	6.26992	Adipose_R2_peak_	Distal Intergen	10	81704825	81721976	17152	1	73610	uc007gjf.2	-7181	ENSMUSG000	Zfp433	zinc finger protein 433
chr10	81697753	81697896	144	145	81697814	32	7.08897	2.72578	4.09488	Adipose_R2_peak_	Distal Intergen	10	81704825	81721976	17152	1	73610	uc007gjf.2	-6929	ENSMUSG000	Zfp433	zinc finger protein 433
chr10	81698312	81698495	184	185	81698427	34	7.45994	2.73603	4.42632	Adipose_R2_peak_	Distal Intergen	10	81704825	81721976	17152	1	73610	uc007gjf.2	-6330	ENSMUSG000	Zfp433	zinc finger protein 433
chr10	81699334	81699658	325	326	81699441	32	5.89068	2.42381	3.00134	Adipose_R2_peak_	Distal Intergen	10	81704825	81721976	17152	1	73610	uc007gjf.2	-5167	ENSMUSG000	Zfp433	zinc finger protein 433
chr10	81702750	81702986	237	238	81702865	45	12.76725	3.44809	9.39843	Adipose_R2_peak_	Promoter (1-2	10	81704825	81721976	17152	1	73610	uc007gjf.2	-1839	ENSMUSG000	Zfp433	zinc finger protein 433
chr10	81703246	81703778	533	534	81703358	30	5.9359	2.50566	3.04483	Adipose_R2_peak_	Promoter (1-2	10	81704825	81721976	17152	1	73610	uc007gjf.2	-1047	ENSMUSG000	Zfp433	zinc finger protein 433
chr10	81704390	81704548	159	160	81704441	27	5.76007	2.57805	2.88921	Adipose_R2_peak_	Promoter (<=1	10	81704825	81721976	17152	1	73610	uc007gjf.2	-277	ENSMUSG000	Zfp433	zinc finger protein 433
chr10	81710394	81710640	247	248	81710445	25	4.86612	2.39766	2.09018	Adipose_R2_peak_	Intron (uc007g	10	81704825	81721976	17152	1	73610	uc007gjf.2	5569	ENSMUSG000	Zfp433	zinc finger protein 433
chr10	81715150	81715371	222	223	81715227	31	7.7623	2.95097	4.69388	Adipose_R2_peak_	Intron (uc007g	10	81704825	81721976	17152	1	73610	uc007gjf.2	10325	ENSMUSG000	Zfp433	zinc finger protein 433
chr10	81717684	81717821	138	139	81717773	27	5.77405	2.5821	2.88921	Adipose_R2_peak_	Intron (uc007g	10	81704825	81721976	17152	1	73610	uc007gjf.2	12859	ENSMUSG000	Zfp433	zinc finger protein 433
chr10	81720559	81720668	110	111	81720592	27	5.77405	2.5821	2.88921	Adipose_R2_peak_	Exon (uc007gj	10	81704825	81721976	17152	1	73610	uc007gjf.2	15734	ENSMUSG000	Zfp433	zinc finger protein 433
chr10	81730022	81730159	138	139	81730075	27	5.53046	2.51193	2.68965	Adipose_R2_peak_	Distal Intergen	10	81704825	81721976	17152	1	73610	uc007gjf.2	25197	ENSMUSG000	Zfp433	zinc finger protein 433

chr10	81730491	81730681	191	192	81730518	26	5.31252	2.48988	2.47999	Adipose_R2_peak_	Distal Interger	10	81704825	81721976	17152	1	73610	uc007gjf.2	25666	ENSMUSG000	Zfp433	zinc finger protein 433
chr10	81734326	81734501	176	177	81734441	21	8.43286	3.95423	5.34896	Adipose_R1_peak_	Distal Interger	10	81704825	81721976	17152	1	73610	uc007gjf.2	29501	ENSMUSG000	Zfp433	zinc finger protein 433
chr10	81755089	81755300	212	213	81755189	32	8.29282	3.04319	5.18352	Adipose_R2_peak_	Intron (uc011x	10	81742821	81771142	28322	2	331188	uc011xjl.1	15842	ENSMUSG000	Zfp781	zinc finger protein 781
chr10	81769720	81769861	142	143	81769777	33	8.83596	3.1354	5.68652	Adipose_R2_peak_	Promoter (1-2	10	81742821	81771142	28322	2	331188	uc011xjl.1	1281	ENSMUSG000	Zfp781	zinc finger protein 781
chr10	81775385	81775582	198	199	81775426	28	6.25023	2.67431	3.31626	Adipose_R2_peak_	Distal Interger	10	81742821	81771142	28322	2	331188	uc011xjl.1	-4243	ENSMUSG000	Zfp781	zinc finger protein 781
chr10	81785244	81785481	238	239	81785369	36	20.67077	6.6503	17.14779	Adipose_R1_peak_	Distal Interger	10	81742821	81771142	28322	2	331188	uc011xjl.1	-14102	ENSMUSG000	Zfp781	zinc finger protein 781
chr10	81785264	81785437	174	175	81785360	38	11.72962	3.59649	8.4047	Adipose_R2_peak_	Distal Interger	10	81742821	81771142	28322	2	331188	uc011xjl.1	-14122	ENSMUSG000	Zfp781	zinc finger protein 781
chr10	81799358	81799528	171	172	81799428	24	10.60668	4.49345	7.40961	Adipose_R1_peak_	Distal Interger	10	81812186	81826490	14305	1	1.00E+08	uc007gjh.1	-12658	ENSMUSG000	Gm10778	predicted gene 10778
chr10	81799361	81799475	115	116	81799417	31	7.7623	2.95097	4.69388	Adipose_R2_peak_	Distal Interger	10	81812186	81826490	14305	1	1.00E+08	uc007gjh.1	-12711	ENSMUSG000	Gm10778	predicted gene 10778
chr10	81806219	81806376	158	159	81806296	20	7.74501	3.7745	4.70467	Adipose_R1_peak_	Distal Interger	10	81812186	81826490	14305	1	1.00E+08	uc007gjh.1	-5810	ENSMUSG000	Gm10778	predicted gene 10778
chr10	81808810	81808994	185	186	81808877	34	9.39139	3.22762	6.20474	Adipose_R2_peak_	Distal Interger	10	81812186	81826490	14305	1	1.00E+08	uc007gjh.1	-3192	ENSMUSG000	Gm10778	predicted gene 10778
chr10	81816504	81816670	167	168	81816604	33	8.83596	3.1354	5.68652	Adipose_R2_peak_	Intron (uc007g	10	81812186	81826490	14305	1	1.00E+08	uc007gjh.1	4318	ENSMUSG000	Gm10778	predicted gene 10778
chr10	81831445	81831646	202	203	81831524	27	12.93148	5.03266	9.63827	Adipose_R1_peak_	Distal Interger	10	81812186	81826490	14305	1	1.00E+08	uc007gjh.1	19259	ENSMUSG000	Gm10778	predicted gene 10778
chr10	81831483	81831600	118	119	81831528	29	6.74062	2.76653	3.75989	Adipose_R2_peak_	Distal Interger	10	81812186	81826490	14305	1	1.00E+08	uc007gjh.1	19297	ENSMUSG000	Gm10778	predicted gene 10778
chr10	81911044	81911189	146	147	81911118	20	7.74501	3.7745	4.70467	Adipose_R1_peak_	Intron (uc011x	10	81901911	81930480	28570	2	331188	uc011xjn.1	19291	ENSMUSG000	Zfp781	zinc finger protein 781
chr10	81911068	81911165	98	99	81911130	26	5.31252	2.48988	2.47999	Adipose_R2_peak_	Intron (uc011x	10	81901911	81930480	28570	2	331188	uc011xjn.1	19315	ENSMUSG000	Zfp781	zinc finger protein 781
chr10	81983126	81983417	292	293	81983212	26	12.14079	4.85292	8.87838	Adipose_R1_peak_	Distal Interger	10	81901911	81930480	28570	2	331188	uc011xjn.1	-52646	ENSMUSG000	Zfp781	zinc finger protein 781
chr10	81983148	81983417	270	271	81983264	44	15.55653	4.1498	12.05625	Adipose_R2_peak_	Distal Interger	10	81901911	81930480	28570	2	331188	uc011xjn.1	-52668	ENSMUSG000	Zfp781	zinc finger protein 781
chr10	82205444	82205574	131	132	82205566	19	7.07687	3.59476	4.08458	Adipose_R1_peak_	Distal Interger	10	82224856	82241275	16420	2	237411	uc007gjl.3	35701	ENSMUSG000	Zfp938	zinc finger protein 938
chr10	82347522	82347642	121	122	82347628	27	5.77405	2.5821	2.88921	Adipose_R2_peak_	Distal Interger	10	82224856	82241275	16420	2	237411	uc007gjl.3	-106247	ENSMUSG000	Zfp938	zinc finger protein 938
chr10	82393453	82393673	221	222	82393562	35	19.76029	6.47056	16.26028	Adipose_R1_peak_	Intron (uc007g	10	82486533	82492618	6086	2	432480	uc007gjo.2	98945	ENSMUSG000	Gm1553	predicted gene 1553
chr10	82537173	82537449	277	278	82537316	51	34.3749	8.91121	30.60162	Adipose_R1_peak_	Distal Interger	10	82486533	82492618	6086	2	432480	uc007gjo.2	-44555	ENSMUSG000	Gm1553	predicted gene 1553
chr10	82537233	82537439	207	208	82537318	42	14.2409	3.96536	10.79479	Adipose_R2_peak_	Distal Interger	10	82486533	82492618	6086	2	432480	uc007gjo.2	-44615	ENSMUSG000	Gm1553	predicted gene 1553
chr10	82628170	82628303	134	135	82628213	25	4.86612	2.39766	2.09018	Adipose_R2_peak_	Promoter (1-2	10	82629838	82650494	20657	1	21665	uc007gjr.2	-1535	ENSMUSG000	Tdg	thymine DNA glycosylase
chr10	82629483	82629687	205	206	82629602	34	9.39139	3.22762	6.20474	Adipose_R2_peak_	Promoter (<=1	10	82629838	82650494	20657	1	21665	uc007gjr.2	-151	ENSMUSG000	Tdg	thymine DNA glycosylase
chr10	82629533	82629723	191	192	82629626	22	9.13959	4.13397	6.01526	Adipose_R1_peak_	Promoter (<=1	10	82629838	82650494	20657	1	21665	uc007gjr.2	-115	ENSMUSG000	Tdg	thymine DNA glycosylase
chr10	83466859	83467274	416	417	83466943	23	9.86443	4.31371	6.70247	Adipose_R1_peak_	Distal Interger	10	83487447	83534140	46694	2	216188	uc007gkh.2	66866	ENSMUSG000	Aldh1l2	aldehyde dehydrogenase 1 family, member L2
chr10	83466875	83467007	133	134	83466920	28	6.25023	2.67431	3.31626	Adipose_R2_peak_	Distal Interger	10	83487447	83534140	46694	2	216188	uc007gkh.2	67133	ENSMUSG000	Aldh1l2	aldehyde dehydrogenase 1 family, member L2
chr10	84161307	84161539	233	234	84161424	31	16.23981	5.75161	12.83549	Adipose_R1_peak_	Distal Interger	10	84371319	84440471	69153	2	77976	uc007gko.1	278932	ENSMUSG000	Nuak1	NUAK family, SNF1-like kinase, 1
chr10	84205092	84205276	185	186	84205268	27	12.93148	5.03266	9.63827	Adipose_R1_peak_	Distal Interger	10	84371319	84440471	69153	2	77976	uc007gko.1	235195	ENSMUSG000	Nuak1	NUAK family, SNF1-like kinase, 1
chr10	84205097	84205276	180	181	84205272	38	11.72962	3.59649	8.4047	Adipose_R2_peak_	Distal Interger	10	84371319	84440471	69153	2	77976	uc007gko.1	235195	ENSMUSG000	Nuak1	NUAK family, SNF1-like kinase, 1
chr10	84376755	84376959	205	206	84376878	41	13.59774	3.87315	10.1803	Adipose_R2_peak_	Intron (uc007g	10	84371319	84440471	69153	2	77976	uc007gko.1	63512	ENSMUSG000	Nuak1	NUAK family, SNF1-like kinase, 1
chr10	84456425	84456590	166	167	84456447	17	5.80341	3.23528	2.92378	Adipose_R1_peak_	Distal Interger	10	84371319	84440471	69153	2	77976	uc007gko.1	-15954	ENSMUSG000	Nuak1	NUAK family, SNF1-like kinase, 1
chr10	85281192	85281632	441	442	85281511	30	15.39182	5.57188	12.01394	Adipose_R1_peak_	Distal Interger	10	85131700	85185054	53355	2	12952	uc007gle.1	-96138	ENSMUSG000	Cry1	cryptochrome 1 (photolyase-like)
chr10	85281356	85281557	202	203	85281403	32	8.29282	3.04319	5.18352	Adipose_R2_peak_	Distal Interger	10	85131700	85185054	53355	2	12952	uc007gle.1	-96302	ENSMUSG000	Cry1	cryptochrome 1 (photolyase-like)
chr10	85367791	85367929	139	140	85367854	19	7.07687	3.59476	4.08458	Adipose_R1_peak_	Distal Interger	10	85386814	85660292	273479	1	74007	uc007glg.2	-18885	ENSMUSG000	Btbd11	BTB (POZ) domain containing 11
chr10	85754548	85754765	218	219	85754664	24	10.60668	4.49345	7.40961	Adipose_R1_peak_	Distal Interger	10	85645203	85660292	15090	1	74007	uc007gli.1	109345	ENSMUSG000	Btbd11	BTB (POZ) domain containing 11
chr10	85754688	85754824	137	138	85754758	33	8.83596	3.1354	5.68652	Adipose_R2_peak_	Distal Interger	10	85645203	85660292	15090	1	74007	uc007gli.1	109485	ENSMUSG000	Btbd11	BTB (POZ) domain containing 11
chr10	85755678	85755970	293	294	85755822	39	12.34187	3.68871	8.98501	Adipose_R2_peak_	Distal Interger	10	85645203	85660292	15090	1	74007	uc007gli.1	110475	ENSMUSG000	Btbd11	BTB (POZ) domain containing 11
chr10	85755753	85756353	601	602	85755894	24	10.60668	4.49345	7.40961	Adipose_R1_peak_	Distal Interger	10	85645203	85660292	15090	1	74007	uc007gli.1	110550	ENSMUSG000	Btbd11	BTB (POZ) domain containing 11
chr10	85756134	85756360	227	228	85756214	40	12.96465	3.78093	9.57654	Adipose_R2_peak_	Distal Interger	10	85645203	85660292	15090	1	74007	uc007gli.1	110931	ENSMUSG000	Btbd11	BTB (POZ) domain containing 11
chr10	85780284	85780431	148	149	85780364	19	7.07687	3.59476	4.08458	Adipose_R1_peak_	Distal Interger	10	85871831	85884688	12858	1	103136	uc007gnc.2	-91400	ENSMUSG000	Pwp1	PWP1 homolog, endonuclein
chr10	85794494	85794646	153	154	85794573	21	8.43286	3.95423	5.34896	Adipose_R1_peak_	Distal Interger	10	85871831	85884688	12858	1	103136	uc007gnc.2	-77185	ENSMUSG000	Pwp1	PWP1 homolog, endonuclein
chr10	85857642	85857992	351	352	85857775	39	17.65731	5.18114	14.22497	Adipose_R1_peak_	Distal Interger	10	85871831	85884688	12858	1	103136	uc007gnc.2	-13839	ENSMUSG000	Pwp1	PWP1 homolog, endonuclein
chr10	85857703	85857979	277	278	85857824	46	16.90986	4.33423	13.35898	Adipose_R2_peak_	Distal Interger	10	85871831	85884688	12858	1	103136	uc007gnc.2	-13852	ENSMUSG000	Pwp1	PWP1 homolog, endonuclein
chr10	85866671	85867003	333	334	85866762	23	9.86443	4.31371	6.70247	Adipose_R1_peak_	Distal Interger	10	85871831	85884688	12858	1	103136	uc007gnc.2	-4828	ENSMUSG000	Pwp1	PWP1 homolog, endonuclein
chr10	85866690	85866883	194	195	85866750	31	7.7623	2.95097	4.69388	Adipose_R2_peak_	Distal Interger	10	85871831	85884688	12858	1	103136	uc007gnc.2	-4948	ENSMUSG000	Pwp1	PWP1 homolog, endonuclein
chr10	85867389	85867527	139	140	85867493	19	7.07687	3.59476	4.08458	Adipose_R1_peak_	Distal Interger	10	85871831	85884688	12858	1	103136	uc007gnc.2	-4304	ENSMUSG000	Pwp1	PWP1 homolog, endonuclein
chr10	86561914	86562054	141	142	86561978	16	5.20017	3.05554	2.38734	Adipose_R1_peak_	Distal Interger	10	86048746	86498896	450151	2	27204	uc007gnn.2	-63018	ENSMUSG000	Syn3	synapsin III
chr10	86562745	86562981	237	238	86562857	59	26.5291	5.53306	22.7188	Adipose_R2_peak_	Distal Interger	10	86048746	86498896	450151	2	27204	uc007gnn.2	-63849	ENSMUSG000	Syn3	synapsin III
chr10	86562746	86563028	283	284	86562869	60	45.43621	10.96401	41.50272	Adipose_R1_peak_	Distal Interger	10	86048746	86498896	450151	2	27204	uc007gnn.2	-63850	ENSMUSG000	Syn3	synapsin III
chr10	86596781	86597021	241	242	86596909	35	19.76029	6.47056	16.26028	Adipose_R1_peak_	Distal Interger	10	86685527	86689954	4428	1	66263	uc007gqe.2	-88506	NA	1810014B01Ri	RIKEN cDNA 1810014B01 gene
chr10	86596792	86596994	203	204	86596916	38	11.72962	3.59649	8.4047	Adipose_R2_peak_	Distal Interger	10	86685527	86689954	4428	1	66263	uc007gqe.2	-88533	NA	1810014B01Ri	RIKEN cDNA 1810014B01 gene
chr10	87179157	87179324	168	169	87179239	19	7.07687	3.59476	4.08458	Adipose_R1_peak_	Intron (uc011x	10	87205979	87230599	24621	1	76640	uc007gqq.2	-26655	ENSMUSG000	1700113H08R	RIKEN cDNA 1700113H08 gene
chr10	87179794	87180077	284	285	87179938	52	36.63102	9.52611	32.81188	Adipose_R1_peak_	Intron (uc011x	10	87205979	87230599	24621	1	76640	uc007gqq.2	-25902	ENSMUSG000	1700113H08R	RIKEN cDNA 1700113H08 gene
chr10	87179814	87180048	235	236	87179933	55	23.42763	5.16149	19.68558	Adipose_R2_peak_	Intron (uc011x	10	87205979	87230599	24621	1	76640	uc007gqq.2	-25931	ENSMUSG000	1700113H08R	RIKEN cDNA 1700113H08 gene
chr10	87400719	87401233	515	516	87400848	34	18.86162	6.29083	15.38482	Adipose_R1_peak_	Distal Interger	10	87491041	87493660	2620	2	17172	uc007gqs.1	92427	ENSMUSG000	Ascl1	achaete-scute family bHLH transcription factor 1
chr10	87400789	87400927	139	140	87400865	30	7.24477	2.85875	4.21917	Adipose_R2_peak_	Distal Interger	10	87491041	87493660	2620	2	17172	uc007gqs.1	92733	ENSMUSG000	Ascl1	achaete-scute family bHLH transcription factor 1
chr10	87401905	87402091	187	188	87402015	24	10.60668	4.49345	7.40961	Adipose_R1_peak_	Distal Interger	10	87491041	87493660	2620	2	17172	uc007gqs.1	91569	ENSMUSG000	Ascl1	achaete-scute family bHLH transcription factor 1
chr10	87442255	87442403	149	150	87442318	18	6.42934	3.41502	3.48999	Adipose_R1_peak_	Distal Interger	10	87491041	87493660	2620	2	17172	uc007gqs.1	51257	ENSMUSG000	Ascl1	achaete-scute family bHLH transcription factor 1
chr10	87633849	87634175	327	328	87633987	71	58.27042	12.94113	54.20487	Adipose_R1_peak_	Distal Interger	10	87521795	87584137	62343	1	18478	uc007gqt.2	112054	ENSMUSG000	Pah	phenylalanine hydroxylase
chr10	87633874	87634161	288	289	87633980	69	34.76717	6.45524	30.8116	Adipose_R2_peak_	Distal Interger	10	87521795	87584137	62343	1	18478	uc007gqt.2	112079	ENSMUSG000	Pah	phenylalanine hydroxylase
chr10	87634511	87634786	276	277	87634643	67	53.51345	12.22218	49.49424	Adipose_R1_peak_	Distal Interger	10	87521795	87584137	62343	1	18478	uc007gqt.2	112716	ENSMUSG000	Pah	phenylalanine hydroxylase
chr10	87634524	87634731	208	209	87634634	49	19.00719	4.61089	15.38598	Adipose_R2_peak_	Distal Interger	10	87521795	87584137	62343	1	18478	uc007gqt.2	112729	ENSMUSG000	Pah	phenylalanine hydroxylase
chr10	87686949	87687159	211	212	87687025	20	7.74501	3.7745	4.70467	Adipose_R1_peak_	Distal Interger	10	87521795	87584137	62343	1	18478	uc007gqt.2	165154	ENSMUSG000	Pah	phenylalanine hydroxylase
chr10	87990731	87990986	256	257	87990857	39	23.47001	7.18952	19.88325	Adipose_R1_peak_	Distal Interger	10	87966671	87968017	1347	1	22172	uc007grb.1	24060	NA	Tyms-ps	thymidylate synthase, pseudogene
chr10	87990781	87990906	126	127	87990861	30	7.24477	2.85875	4.21917	Adipose_R2_peak_	Distal Interger	10	87966671	87968017	1347	1	22172	uc007grb.1	24110	NA	Tyms-ps	thymidylate synthase, pseudogene
chr10	88189828	88189974	147	148	88189892	16	5.20017	3.05554	2.38734	Adipose_R1_peak_	Distal Interger	10	88201097	88225362	24266	1	67282	uc007grg.2	-11123	ENSMUSG000	Ccdc53	coiled-coil domain containing 53
chr10	88507234	88507333	100	101	88507279	27	5.77405	2.5821	2.88921	Adipose_R2_peak_	Distal Interger	10	88472813	88503970	31158	2	212862	uc007grq.2	-3264	ENSMUSG000	Chpt1	choline phosphotransferase 1
chr10	88509689	88509836	148	149	88509767	20	7.74501	3.7745	4.70467	Adipose_R1_peak_	Distal Interger	10	88472813	88503970	31158	2	212862	uc007grq.2	-5719	ENSMUSG000	Chpt1	choline phosphotransferase 1
chr10	88651043	88651180	138	139	88651130	37	11.12817	3.50427	7.83613	Adipose_R2_peak_	Distal Interger	10	88675270	88683023	7754	2	20728	uc007grv.2	31843	ENSMUSG000	Spic	Spi-C transcription factor (Spi-1/PU.1 related)
chr10	88653827	88654049	223	224	88653930	30	15.39182	5.57188	12.01394	Adipose_R1_peak_	Distal Interger	10	88675270	88683023	7754	2	20728	uc007grv.2	28974	ENSMUSG000	Spic	Spi-C transcription factor (Spi-1/PU.1 related)
chr10	88653840	88654015	176	177	88653915	34	9.39139	3.22762	6.20474	Adipose_R2_peak_	Distal Interger	10	88675270	88683023	7754	2	20728	uc007grv.2	29008	ENSMUSG000	Spic	Spi-C transcription factor (Spi-1/PU.1 related)
chr10	89878577	89878743	167	168	89878641	18	6.42934	3.41502	3.48999	Adipose_R1_peak_	Intron (uc033f	10	89873509	90972984	1099476	1	77531	uc033frv.1	5068	ENSMUSG000	Anks1b	ankyrin repeat and sterile alpha motif domain containing 1B
chr10	89954684	89955002	319	320	89954855	76	64.35061	13.83982	60.23161	Adipose_R1_peak_	Intron (uc033f	10	89873509	90972984	1099476	1	77531	uc033frv.1	81175	ENSMUSG000	Anks1b	ankyrin repeat and sterile alpha motif domain containing 1B
chr10	89954697	89955000	304	305	89954866	64	30.56565	5.99415	26.6807	Adipose_R2_peak_	Intron (uc033f	10	89873509	90972984	1099476	1	77531	uc033frv.1	81188	ENSMUSG000	Anks1b	ankyrin repeat and sterile alpha motif domain containing 1B
chr10	90216723	90216989	267	268	90216853	46	30.36364	8.44768	26.64488	Adipose_R1_peak_	Intron (uc033f	10	89873509	90972984	1099476	1	77531	uc033frv.1	343214	ENSMUSG000	Anks1b	ankyrin repeat and sterile alpha motif domain containing 1B
chr10	90216738	90216985	248	249	90216858	51	20.44827	4.79532	16.78434	Adipose_R2_peak_	Intron (uc033f	10	89873509	90972984	1099476	1	77531	uc033frv.1	343229	ENSMUSG000	Anks1b	ankyrin repeat and sterile alpha motif domain containing 1B
chr10	90275871	90276034	164	165	90275936	37	11.12817	3.50427	7.83613	Adipose_R2_peak_	Intron (uc033f	10	90575727	90972984	397258	1	77531	uc033frw.1	-299693	ENSMUSG000	Anks1b	ankyrin repeat and sterile alpha motif domain containing 1B
chr10	90275893	90276040	148	149	90275974	21	8.43286	3.95423	5.34896	Adipose_R1_peak_	Intron (uc033f	10	90575727	90972984	397258	1	77531	uc033frw.1	-299687	ENSMUSG000	Anks1b	ankyrin repeat and sterile alpha motif domain containing 1B
chr10	91172603	91172946	344	345	91172682	29	6.74062	2.76653	3.75989	Adipose_R2_peak_	Promoter (<=1	10	91147571	91171619	24049	2	21917	uc007gtq.2	-984	ENSMUSG000	Tmpo	thymopoietin
chr10	91174475	91175000	526	527	91174554	35	9.95877	3.31984	6.73562	Adipose_R2_peak_	Promoter (2-3	10	91147571	91171619	24049	2	21917	uc007gtq.2	-2856	ENSMUSG000	Tmpo	thymopoietin
chr10	91176442	91176661	220	221	91176476	28	6.25023	2.67431	3.31626	Adipose_R2_peak_	Distal Interger	10	91147571	91171619	24049	2	21917	uc007gtq.2	-4823	ENSMUSG000	Tmpo	thymopoietin
chr10	91177286	91177455	170	171	91177360	29	6.74062	2.76653	3.75989	Adipose_R2_peak_	Distal Interger	10	91147571	91171619	24049	2	21917	uc007gtq.2	-5667	ENSMUSG000	Tmpo	thymopoietin
chr10	91177999	91178178	180	181	91178044	28	6.25023	2.67431	3.31626	Adipose_R2_peak_	Distal Interger	10	91147571	91171619	24049	2	21917	uc007gtq.2	-6380	ENSMUSG000	Tmpo	thymopoietin
chr10	91181311	91181449	139	140	91181396	26	5.31252	2.48988	2.47999	Adipose_R2_peak_	Exon (uc033fs	10	91147571	91171619	24049	2	21917	uc007gtq.2	-9692	ENSMUSG000	Tmpo	thymopoietin
chr10	91252528	91252762	235	236	91252670	37	21.59274	6.83004	18.04766	Adipose_R1_peak_	Distal Interger	10	91147571	91171619	24049	2	21917	uc007gtq.2	-80909	ENSMUSG000	Tmpo	thymopoietin
chr10	91252585	91252759	175	176	91252666	33	8.83596	3.1354	5.68652	Adipose_R2_peak_	Distal Interger	10	91147571	91171619	24049	2	21917	uc007gtq.2	-80966	ENSMUSG000	Tmpo	thymopoietin
chr10	91254079	91254358	280	281	91254222	66	52.33981	12.04244	48.33179	Adipose_R1_peak_	Distal Interger	10	91147571	91171619	24049	2	21917	uc007gtq.2	-82460	ENSMUSG000	Tmpo	thymopoietin
chr10	91254126	91254344	219	220	91254223	54	22.67101	5.07198	18.94758	Adipose_R2_peak_	Distal Interger	10	91147571	91171619	24049	2	21917	uc007gtq.2	-82507	ENSMUSG000	Tmpo	thymopoietin
chr10	91931042	91931344	303	304	91931208	67	53.51345	12.22218	49.49424	Adipose_R1_peak_	Distal Interger	10	92072086	92072185	100	2	723955	uc007gty.2	140841	ENSMUSG000	Mir135a-2	microRNA 135a-2
chr10	91931058	91931335	278	279	91931206	75	40.01057	7.00855	35.97731	Adipose_R2_peak_	Distal Interger	10	92072086	92072185	100	2	723955	uc007gty.2	140850	ENSMUSG000	Mir135a-2	microRNA 135a-2
chr10	91977571	91977741	171	172	91977636	20	7.74501	3.7745	4.70467	Adipose_R1_peak_	Distal Interger	10	92072086	92072185	100	2	723955	uc007gty.2	94444	ENSMUSG000	Mir135a-2	microRNA 135a-2
chr10	92753168	92753308	141	142	92753242	20	7.74501	3.7745	4.70467	Adipose_R1_peak_	Distal Interger	10	92684745	92722418	37674	2	17997	uc007gud.2	-30750	ENSMUSG000	Nedd1	neural precursor cell expressed, developmentally down-regulated gene 1

chr10	92769981	92770169	189	190	92770075	27	12.93148	5.03266	9.63827	Adipose_R1_peak_	Distal Intergen	10	92684745	92722418	37674	2	17997	uc007gud.2	-47563	ENSMUSG000	Nedd1	neural precursor cell expressed, developmentally down-regulated gene 1
chr10	92909522	92909790	269	270	92909649	52	36.63102	9.52611	32.81188	Adipose_R1_peak_	Intron (uc007g	10	92684745	92722418	37674	2	17997	uc007gud.2	-187104	ENSMUSG000	Nedd1	neural precursor cell expressed, developmentally down-regulated gene 1
chr10	92909533	92909740	208	209	92909664	40	12.96465	3.78093	9.57654	Adipose_R2_peak_	Intron (uc007g	10	92684745	92722418	37674	2	17997	uc007gud.2	-187115	ENSMUSG000	Nedd1	neural precursor cell expressed, developmentally down-regulated gene 1
chr10	92910520	92910874	355	356	92910733	77	65.58373	14.01956	61.45456	Adipose_R1_peak_	Intron (uc007g	10	92684745	92722418	37674	2	17997	uc007gud.2	-188102	ENSMUSG000	Nedd1	neural precursor cell expressed, developmentally down-regulated gene 1
chr10	92910539	92910874	336	337	92910741	74	39.12209	6.91633	35.10124	Adipose_R2_peak_	Intron (uc007g	10	92684745	92722418	37674	2	17997	uc007gud.2	-188121	ENSMUSG000	Nedd1	neural precursor cell expressed, developmentally down-regulated gene 1
chr10	92914782	92915043	262	263	92914915	43	27.35048	7.90847	23.68561	Adipose_R1_peak_	Intron (uc007g	10	92684745	92722418	37674	2	17997	uc007gud.2	-192364	ENSMUSG000	Nedd1	neural precursor cell expressed, developmentally down-regulated gene 1
chr10	92914836	92915009	174	175	92914924	39	12.34187	3.68871	8.98501	Adipose_R2_peak_	Intron (uc007g	10	92684745	92722418	37674	2	17997	uc007gud.2	-192418	ENSMUSG000	Nedd1	neural precursor cell expressed, developmentally down-regulated gene 1
chr10	93302330	93302463	134	135	93302394	17	5.80341	3.23528	2.92378	Adipose_R1_peak_	Intron (uc011x	10	93247416	93311159	63744	2	13713	uc011xlt.1	8696	ENSMUSG000	Elk3	ELK3, member of ETS oncogene family
chr10	93308636	93308806	171	172	93308685	16	5.20017	3.05554	2.38734	Adipose_R1_peak_	Promoter (2-3	10	93247416	93311159	63744	2	13713	uc011xlt.1	2353	ENSMUSG000	Elk3	ELK3, member of ETS oncogene family
chr10	93312888	93313058	171	172	93312962	34	9.39139	3.22762	6.20474	Adipose_R2_peak_	Promoter (1-2	10	93247416	93311159	63744	2	13713	uc011xlt.1	-1729	ENSMUSG000	Elk3	ELK3, member of ETS oncogene family
chr10	93409677	93409820	144	145	93409748	19	7.07687	3.59476	4.08458	Adipose_R1_peak_	Distal Intergen	10	93433107	93433162	56	1	1.02E+08	uc056yhu.1	-23287	ENSMUSG000	Mir7688	microRNA 7688
chr10	93410954	93411310	357	358	93411159	175	168.3451	20.41334	163.7859	Adipose_R1_peak_	Distal Intergen	10	93433107	93433162	56	1	1.02E+08	uc056yhu.1	-21797	ENSMUSG000	Mir7688	microRNA 7688
chr10	93411012	93411299	288	289	93411165	133	92.97763	11.0683	88.49943	Adipose_R2_peak_	Distal Intergen	10	93433107	93433162	56	1	1.02E+08	uc056yhu.1	-21808	ENSMUSG000	Mir7688	microRNA 7688
chr10	93729920	93730117	198	199	93730039	31	7.7623	2.95097	4.69388	Adipose_R2_peak_	Intron (uc007g	10	93674218	93683322	9105	2	1.01E+08	uc029reo.1	-46598	ENSMUSG000	Gm15915	predicted gene 15915
chr10	93729957	93730094	138	139	93730018	18	6.42934	3.41502	3.48999	Adipose_R1_peak_	Intron (uc007g	10	93674218	93683322	9105	2	1.01E+08	uc029reo.1	-46635	ENSMUSG000	Gm15915	predicted gene 15915
chr10	95901082	95901213	132	133	95901183	30	7.24477	2.85875	4.21917	Adipose_R2_peak_	Distal Intergen	10	95940663	96003003	62341	1	216238	uc011xmf.1	-39450	ENSMUSG000	Eea1	early endosome antigen 1
chr10	95901091	95901239	149	150	95901171	20	7.74501	3.7745	4.70467	Adipose_R1_peak_	Distal Intergen	10	95940663	96003003	62341	1	216238	uc011xmf.1	-39424	ENSMUSG000	Eea1	early endosome antigen 1
chr10	96070892	96071043	152	153	96070977	21	8.43286	3.95423	5.34896	Adipose_R1_peak_	Distal Intergen	10	95940663	96003003	62341	1	216238	uc011xmf.1	130229	ENSMUSG000	Eea1	early endosome antigen 1
chr10	96505654	96505804	151	152	96505716	19	7.07687	3.59476	4.08458	Adipose_R1_peak_	Distal Intergen	10	96617001	96622811	5811	1	12226	uc007gww.2	-111197	ENSMUSG000	Btg1	B cell translocation gene 1, anti-proliferative
chr10	96709059	96709312	254	255	96709123	20	7.74501	3.7745	4.70467	Adipose_R1_peak_	Distal Intergen	10	96617001	96622811	5811	1	12226	uc007gww.2	92058	ENSMUSG000	Btg1	B cell translocation gene 1, anti-proliferative
chr10	96713296	96713389	94	95	96713353	28	6.25023	2.67431	3.31626	Adipose_R2_peak_	Distal Intergen	10	96617001	96622811	5811	1	12226	uc007gww.2	96295	ENSMUSG000	Btg1	B cell translocation gene 1, anti-proliferative
chr10	97321725	97321941	217	218	97321833	28	13.73721	5.2124	10.41463	Adipose_R1_peak_	Distal Intergen	10	97423478	97423562	85	1	1.01E+08	uc029ret.1	-101537	ENSMUSG000	Mir3966	microRNA 3966
chr10	97321810	97321904	95	96	97321860	31	7.7623	2.95097	4.69388	Adipose_R2_peak_	Distal Intergen	10	97423478	97423562	85	1	1.01E+08	uc029ret.1	-101574	ENSMUSG000	Mir3966	microRNA 3966
chr10	97349834	97349984	151	152	97349950	18	6.42934	3.41502	3.48999	Adipose_R1_peak_	Distal Intergen	10	97423478	97423562	85	1	1.01E+08	uc029ret.1	-73494	ENSMUSG000	Mir3966	microRNA 3966
chr10	97897415	97897743	329	330	97897607	30	15.39182	5.57188	12.01394	Adipose_R1_peak_	Intron (uc011x	10	97681414	97967090	285677	2	1.00E+08	uc011xmh.1	69347	NA	Gm10754	predicted gene 10754
chr10	97897469	97897604	136	137	97897509	29	6.74062	2.76653	3.75989	Adipose_R2_peak_	Intron (uc011x	10	97681414	97967090	285677	2	1.00E+08	uc011xmh.1	69486	NA	Gm10754	predicted gene 10754
chr10	97918563	97918720	158	159	97918674	22	9.13959	4.13397	6.01526	Adipose_R1_peak_	Intron (uc011x	10	97681414	97967090	285677	2	1.00E+08	uc011xmh.1	48370	NA	Gm10754	predicted gene 10754
chr10	98051288	98051433	146	147	98051363	22	9.13959	4.13397	6.01526	Adipose_R1_peak_	Distal Intergen	10	97681414	97967090	285677	2	1.00E+08	uc011xmh.1	-84198	NA	Gm10754	predicted gene 10754
chr10	98223198	98223355	158	159	98223322	29	6.74062	2.76653	3.75989	Adipose_R2_peak_	Distal Intergen	10	98299927	98351867	51941	2	667438	uc029rev.1	128512	NA	Gm8633	predicted gene 8633
chr10	98442477	98442641	165	166	98442540	22	9.13959	4.13397	6.01526	Adipose_R1_peak_	Distal Intergen	10	98299927	98351867	51941	2	667438	uc029rev.1	-90610	NA	Gm8633	predicted gene 8633
chr10	98644186	98644379	194	195	98644342	17	5.80341	3.23528	2.92378	Adipose_R1_peak_	Distal Intergen	10	98915152	99026143	110992	1	67972	uc007gxf.2	-270773	ENSMUSG000	Atp2b1	ATPase, Ca++ transporting, plasma membrane 1
chr10	98851422	98851672	251	252	98851491	33	8.83596	3.1354	5.68652	Adipose_R2_peak_	Distal Intergen	10	98915152	99026143	110992	1	67972	uc007gxf.2	-63480	ENSMUSG000	Atp2b1	ATPase, Ca++ transporting, plasma membrane 1
chr10	99021223	99021483	261	262	99021360	42	26.36523	7.72873	22.71906	Adipose_R1_peak_	Intron (uc007g	10	99107171	99197988	90818	1	382406	uc007gxh.1	-85688	ENSMUSG000	Poc1b	POC1 centriolar protein B
chr10	99021288	99021421	134	135	99021362	34	9.39139	3.22762	6.20474	Adipose_R2_peak_	Intron (uc007g	10	99107171	99197988	90818	1	382406	uc007gxh.1	-85750	ENSMUSG000	Poc1b	POC1 centriolar protein B
chr10	99955808	99955942	135	136	99955885	29	6.74062	2.76653	3.75989	Adipose_R2_peak_	Distal Intergen	10	1.00E+08	1.00E+08	84589	1	17311	uc007gxp.1	-59882	ENSMUSG000	Kitl	kit ligand
chr10	100194443	100194595	153	154	100194540	21	8.43286	3.95423	5.34896	Adipose_R1_peak_	Distal Intergen	10	1.00E+08	1.00E+08	8741	1	17311	uc007gxs.1	102771	ENSMUSG000	Kitl	kit ligand
chr10	100328767	100328954	188	189	100328877	33	8.83596	3.1354	5.68652	Adipose_R2_peak_	Distal Intergen	10	1.00E+08	1.00E+08	914	1	1.00E+08	uc011xml.1	-6722	ENSMUSG000	Gm4312	predicted gene 4312
chr10	100386546	100386815	270	271	100386676	78	66.82238	14.19929	62.68234	Adipose_R1_peak_	Distal Intergen	10	1.00E+08	1.00E+08	914	2	1.00E+08	uc011xmq.1	-3986	ENSMUSG000	Gm4312	predicted gene 4312
chr10	100386552	100386804	253	254	100386678	80	44.53636	7.46964	40.44495	Adipose_R2_peak_	Distal Intergen	10	1.00E+08	1.00E+08	914	2	1.00E+08	uc011xmq.1	-3992	ENSMUSG000	Gm4312	predicted gene 4312
chr10	100387019	100387360	342	343	100387148	63	48.85777	11.50323	44.88581	Adipose_R1_peak_	Distal Intergen	10	1.00E+08	1.00E+08	914	2	1.00E+08	uc011xmq.1	-4459	ENSMUSG000	Gm4312	predicted gene 4312
chr10	100387071	100387340	270	271	100387174	77	41.80442	7.19298	37.74731	Adipose_R2_peak_	Distal Intergen	10	1.00E+08	1.00E+08	914	2	1.00E+08	uc011xmq.1	-4511	ENSMUSG000	Gm4312	predicted gene 4312
chr10	100661387	100661542	156	157	100661447	19	7.07687	3.59476	4.08458	Adipose_R1_peak_	Distal Intergen	10	1.01E+08	1.01E+08	26006	1	66605	uc007gxz.1	69001	ENSMUSG000	1700017N19R	RIKEN cDNA 1700017N19 gene
chr10	100676900	100677093	194	195	100677018	28	13.73721	5.2124	10.41463	Adipose_R1_peak_	Distal Intergen	10	1.01E+08	1.01E+08	26006	1	66605	uc007gxz.1	84514	ENSMUSG000	1700017N19R	RIKEN cDNA 1700017N19 gene
chr10	100679751	100679992	242	243	100679900	33	17.97508	6.11109	14.52164	Adipose_R1_peak_	Distal Intergen	10	1.01E+08	1.01E+08	26006	1	66605	uc007gxz.1	87365	ENSMUSG000	1700017N19R	RIKEN cDNA 1700017N19 gene
chr10	100679796	100679995	200	201	100679881	34	9.39139	3.22762	6.20474	Adipose_R2_peak_	Distal Intergen	10	1.01E+08	1.01E+08	26006	1	66605	uc007gxz.1	87410	ENSMUSG000	1700017N19R	RIKEN cDNA 1700017N19 gene
chr10	100734799	100734957	159	160	100734935	23	9.86443	4.31371	6.70247	Adipose_R1_peak_	Distal Intergen	10	1.01E+08	1.01E+08	26006	1	66605	uc007gxz.1	142413	ENSMUSG000	1700017N19R	RIKEN cDNA 1700017N19 gene
chr10	101002025	101002156	132	133	101002086	17	5.80341	3.23528	2.92378	Adipose_R1_peak_	Distal Intergen	10	1.01E+08	1.01E+08	26006	1	66605	uc007gxz.1	409639	ENSMUSG000	1700017N19R	RIKEN cDNA 1700017N19 gene
chr10	101007859	101007988	130	131	101007928	29	6.74062	2.76653	3.75989	Adipose_R2_peak_	Distal Intergen	10	1.01E+08	1.01E+08	26006	1	66605	uc007gxz.1	415473	ENSMUSG000	1700017N19R	RIKEN cDNA 1700017N19 gene
chr10	101342935	101343071	137	138	101343057	18	6.42934	3.41502	3.48999	Adipose_R1_peak_	Distal Intergen	10	1.02E+08	1.02E+08	709982	1	67569	uc029rfb.1	-338416	ENSMUSG000	Mgat4c	MGAT4 family, member C
chr10	101628149	101628425	277	278	101628288	66	52.33981	12.04244	48.33179	Adipose_R1_peak_	Distal Intergen	10	1.02E+08	1.02E+08	709982	1	67569	uc029rfb.1	-53062	ENSMUSG000	Mgat4c	MGAT4 family, member C
chr10	101628161	101628418	258	259	101628295	55	23.42763	5.16419	19.68558	Adipose_R2_peak_	Distal Intergen	10	1.02E+08	1.02E+08	709982	1	67569	uc029rfb.1	-53069	ENSMUSG000	Mgat4c	MGAT4 family, member C
chr10	101632839	101633062	224	225	101632975	38	11.72962	3.59649	8.4047	Adipose_R2_peak_	Distal Intergen	10	1.02E+08	1.02E+08	709982	1	67569	uc029rfb.1	-48425	ENSMUSG000	Mgat4c	MGAT4 family, member C
chr10	101656488	101656635	148	149	101656573	20	7.74501	3.7745	4.70467	Adipose_R1_peak_	Distal Intergen	10	1.02E+08	1.02E+08	709982	1	67569	uc029rfb.1	-24852	ENSMUSG000	Mgat4c	MGAT4 family, member C
chr10	101744898	101745029	132	133	101744989	18	6.42934	3.41502	3.48999	Adipose_R1_peak_	Intron (uc029r	10	1.02E+08	1.02E+08	81	1	1.01E+08	uc011xms.1	-27663	ENSMUSG000	Mir3059	microRNA 3059
chr10	101960141	101960299	159	160	101960203	22	9.13959	4.13397	6.01526	Adipose_R1_peak_	Intron (uc029r	10	1.02E+08	1.02E+08	81	1	1.01E+08	uc011xms.1	187449	ENSMUSG000	Mir3059	microRNA 3059
chr10	101960144	101960271	128	129	101960189	28	6.25023	2.67431	3.31626	Adipose_R2_peak_	Intron (uc029r	10	1.02E+08	1.02E+08	81	1	1.01E+08	uc011xms.1	187452	ENSMUSG000	Mir3059	microRNA 3059
chr10	102173546	102173777	232	233	102173679	42	26.36523	7.72873	22.71906	Adipose_R1_peak_	Intron (uc029r	10	1.02E+08	1.02E+08	232390	1	67569	uc007gyc.2	14467	ENSMUSG000	Mgat4c	MGAT4 family, member C
chr10	102173572	102173770	199	200	102173656	43	14.8939	4.05758	11.42017	Adipose_R2_peak_	Intron (uc029r	10	1.02E+08	1.02E+08	232390	1	67569	uc007gyc.2	14493	ENSMUSG000	Mgat4c	MGAT4 family, member C
chr10	102324580	102324769	190	191	102324684	19	7.07687	3.59476	4.08458	Adipose_R1_peak_	Intron (uc029r	10	1.02E+08	1.02E+08	17032	1	67569	uc007gyd.2	-49668	ENSMUSG000	Mgat4c	MGAT4 family, member C
chr10	102326392	102326510	119	120	102326419	26	5.31252	2.48988	2.47999	Adipose_R2_peak_	Intron (uc029r	10	1.02E+08	1.02E+08	17032	1	67569	uc007gyd.2	-47927	ENSMUSG000	Mgat4c	MGAT4 family, member C
chr10	102326394	102326570	177	178	102326466	24	10.60668	4.49345	7.40961	Adipose_R1_peak_	Intron (uc029r	10	1.02E+08	1.02E+08	17032	1	67569	uc007gyd.2	-47867	ENSMUSG000	Mgat4c	MGAT4 family, member C
chr10	102327052	102327255	204	205	102327171	26	12.14079	4.85292	8.87838	Adipose_R1_peak_	Intron (uc029r	10	1.02E+08	1.02E+08	17032	1	67569	uc007gyd.2	-47182	ENSMUSG000	Mgat4c	MGAT4 family, member C
chr10	102327107	102327206	100	101	102327156	26	5.31252	2.48988	2.47999	Adipose_R2_peak_	Intron (uc029r	10	1.02E+08	1.02E+08	17032	1	67569	uc007gyd.2	-47231	ENSMUSG000	Mgat4c	MGAT4 family, member C
chr10	102327621	102327792	172	173	102327695	20	7.74501	3.7745	4.70467	Adipose_R1_peak_	Intron (uc029r	10	1.02E+08	1.02E+08	17032	1	67569	uc007gyd.2	-46645	ENSMUSG000	Mgat4c	MGAT4 family, member C
chr10	103211094	103211265	172	173	103211205	33	8.83596	3.1354	5.68652	Adipose_R2_peak_	Intron (uc011x	10	1.03E+08	1.03E+08	173125	2	74978	uc011xmu.1	25057	ENSMUSG000	Lrriq1	leucine-rich repeats and IQ motif containing 1
chr10	103211103	103211302	200	201	103211208	30	15.39182	5.57188	12.01394	Adipose_R1_peak_	Intron (uc011x	10	1.03E+08	1.03E+08	173125	2	74978	uc011xmu.1	25020	ENSMUSG000	Lrriq1	leucine-rich repeats and IQ motif containing 1
chr10	103237515	103237740	226	227	103237611	82	71.83084	14.91825	67.65268	Adipose_R1_peak_	Promoter (1-2	10	1.03E+08	1.03E+08	173125	2	74978	uc011xmu.1	-1193	ENSMUSG000	Lrriq1	leucine-rich repeats and IQ motif containing 1
chr10	103237523	103237736	214	215	103237622	71	36.49133	6.63968	32.5091	Adipose_R2_peak_	Promoter (1-2	10	1.03E+08	1.03E+08	173125	2	74978	uc011xmu.1	-1201	ENSMUSG000	Lrriq1	leucine-rich repeats and IQ motif containing 1
chr10	103479334	103479489	156	157	103479396	18	6.42934	3.41502	3.48999	Adipose_R1_peak_	Distal Intergen	10	1.03E+08	1.03E+08	51572	1	103098	uc007gyj.2	111526	ENSMUSG000	Slc6a15	solute carrier family 6 (neurotransmitter transporter), member 15
chr10	103501470	103501777	308	309	103501616	54	38.78658	9.88558	34.93639	Adipose_R1_peak_	Distal Intergen	10	1.03E+08	1.03E+08	51572	1	103098	uc007gyj.2	133662	ENSMUSG000	Slc6a15	solute carrier family 6 (neurotransmitter transporter), member 15
chr10	103501506	103501747	242	243	103501625	45	16.22858	4.24202	12.70278	Adipose_R2_peak_	Distal Intergen	10	1.03E+08	1.03E+08	51572	1	103098	uc007gyj.2	133698	ENSMUSG000	Slc6a15	solute carrier family 6 (neurotransmitter transporter), member 15
chr10	103518325	103518670	346	347	103518389	21	8.43286	3.95423	5.34896	Adipose_R1_peak_	Intron (uc007g	10	1.03E+08	1.03E+08	51572	1	103098	uc007gyj.2	150517	ENSMUSG000	Slc6a15	solute carrier family 6 (neurotransmitter transporter), member 15
chr10	103633071	103633338	268	269	103633198	58	43.19021	10.60454	39.28351	Adipose_R1_peak_	Distal Intergen	10	1.03E+08	1.03E+08	51572	1	103098	uc007gyj.2	265263	ENSMUSG000	Slc6a15	solute carrier family 6 (neurotransmitter transporter), member 15
chr10	103633094	103633298	205	206	103633206	43	14.8939	4.05758	11.42017	Adipose_R2_peak_	Distal Intergen	10	1.03E+08	1.03E+08	51572	1	103098	uc007gyj.2	265286	ENSMUSG000	Slc6a15	solute carrier family 6 (neurotransmitter transporter), member 15
chr10	103637160	103637337	178	179	103637256	24	10.60668	4.49345	7.40961	Adipose_R1_peak_	Distal Intergen	10	1.03E+08	1.03E+08	51572	1	103098	uc007gyj.2	269352	ENSMUSG000	Slc6a15	solute carrier family 6 (neurotransmitter transporter), member 15
chr10	103637165	103637327	163	164	103637271	33	8.83596	3.1354	5.68652	Adipose_R2_peak_	Distal Intergen	10	1.03E+08	1.03E+08	51572	1	103098	uc007gyj.2	269357	ENSMUSG000	Slc6a15	solute carrier family 6 (neurotransmitter transporter), member 15
chr10	103657060	103657178	119	120	103657135	28	6.25023	2.67431	3.31626	Adipose_R2_peak_	Distal Intergen	10	1.03E+08	1.03E+08	51572	1	103098	uc007gyj.2	289252	ENSMUSG000	Slc6a15	solute carrier family 6 (neurotransmitter transporter), member 15
chr10	103679547	103679834	288	289	103679696	109	73.18735	10.14395	68.8215	Adipose_R2_peak_	Distal Intergen	10	1.03E+08	1.03E+08	51572	1	103098	uc007gyj.2	311739	ENSMUSG000	Slc6a15	solute carrier family 6 (neurotransmitter transporter), member 15
chr10	103679551	103679836	286	287	103679693	114	114.6097	20.66986	110.214	Adipose_R1_peak_	Distal Intergen	10	1.03E+08	1.03E+08	51572	1	103098	uc007gyj.2	311743	ENSMUSG000	Slc6a15	solute carrier family 6 (neurotransmitter transporter), member 15
chr10	103706341	103706642	302	303	103706490	59	44.30963	10.78427	40.38949	Adipose_R1_peak_	Distal Intergen	10	1.03E+08	1.03E+08	51572	1	103098	uc007gyj.2	338533	ENSMUSG000	Slc6a15	solute carrier family 6 (neurotransmitter transporter), member 15
chr10	103706403	103706591	189	190	103706481	38	11.72962	3.59649	8.4047	Adipose_R2_peak_	Distal Intergen	10	1.03E+08	1.03E+08	51572	1	103098	uc007gyj.2	338595	ENSMUSG000	Slc6a15	solute carrier family 6 (neurotransmitter transporter), member 15
chr10	103709627	103709776	150	151	103709707	18	6.42934	3.41502	3.48999	Adipose_R1_peak_	Distal Intergen	10	1.03E+08	1.03E+08	51572	1	103098	uc007gyj.2	341819	ENSMUSG000	Slc6a15	solute carrier family 6 (neurotransmitter transporter), member 15
chr10	103937980	103938186	207	208	103938096	37	21.59274	6.83004	18.04766	Adipose_R1_peak_	Distal Intergen	10	1.04E+08	1.04E+08	10979	1	1.00E+08	uc011xmv.1	-204801	ENSMUSG000	Gm4340	predicted gene 4340
chr10	103938041	103938174	134	135	103938137	27	5.77405	2.5821	2.88921	Adipose_R2_peak_	Distal Intergen	10	1.04E+08	1.04E+08	10979	1	1.00E+08	uc011xmv.1	-204813	ENSMUSG000	Gm4340	predicted gene 4340
chr10	104141912	104142021	110	111	104141989	28	6.03868	2.61431	3.14369	Adipose_R2_peak_	Promoter (<=	10	1.04E+08	1.04E+08	10979	1	1.00E+08	uc011xmv.1	-966	ENSMUSG000	Gm4340	predicted gene 4340
chr10	104142339	104142609	271	272	104142359	33	5.64399	2.3328	2.79912	Adipose_R2_peak_	Promoter (<=	10	1.04E+08	1.04E+08	10979	1	1.00E+08	uc011xmv.1	-378	ENSMUSG000	Gm4340	predicted gene 4340
chr10	104142943	104143171	229	230	104143089	47	15.88364	4.02474	12.50309	Adipose_R1_peak_	Promoter (<=	10	1.04E+08	1.04E+08	10979	1	1.00E+08	uc011xmv.1	0	ENSMUSG000	Gm4340	predicted gene 4340
chr10	104142962	104143197	236	237	104143099	73	21.46769	3.76625	17.77891	Adipose_R2_peak_	Promoter (<=	10	1.04E+08	1.04E+08	10979	1	1.00E+08	uc011xmv.1	0	ENSMUSG000	Gm4340	predicted gene 4340
chr10	104143353	104143825	473	474	104143446	59	14.45025	3.18717	10.99921	Adipose_R2_peak_	Promoter (<=	10	1.04E+08	1.04E+08	10979	1	1.00E+08	uc011xmv.1	366	ENSMUSG000	Gm4340	predicted gene 4340
chr10	104143573	104143738	166	167	104143598	27	4.85601	2.32097	2.11444	Adipose_R1_peak_	Promoter (<=	10	1.04E+08	1.04E+08	10979	1	1.00E+08	uc011xmv.1	586	ENSMUSG000	Gm4340	predicted gene 4340
chr10	104144311	104144483	173	174	104144418	52	10.06314	2.67366	6.83642	Adipose_R2_peak_	Promoter (1-2	10	1.04E+08	1.04E+08	10979	1	1.00E+08	uc011xmv.1	1324	ENSMUSG000	Gm4340	predicted gene 4340
chr10	104146409	104146706	298	299	104146601	52	10.54376	2.75513	7.28522	Adipose_R2_peak_	Intron (uc011x	10	1.04E+08	1.04E+08	10979	1	1.00E+08	uc011xmv.1	3422	ENSMUSG000	Gm4340	predicted gene 4340
chr10	104146926	104147078	153	154	104146992	48	9.20241	2.63277	6.03996	Adipose_R2_peak_	Intron (uc011x	10	1.04E+08	1.04E+08	10979	1	1.00E+08	uc011xmv.1	3939	ENSMUSG000	Gm4340	predicted gene 4340
chr10	104147525	104148042	518	519	104147728	52	9.25012	2.53804	6.08604	Adipose_R2_peak_	Intron (uc011x	10	1.04E+08	1.04E+08	10977	1	1.00E+08	uc011xmx.1	-3455	ENSMUSG000	Gm4340	predicted gene 4340

chr10	104148708	104148824	117	118	104148760	35	7.95007	2.81421	4.87526	Adipose_R2_peak_	Promoter (2-3	10	1.04E+08	1.04E+08	10977	1	1.00E+08	uc011xmx.1	-2673	ENSMUSG000	Gm4340	predicted gene 4340
chr10	104149509	104149697	189	190	104149554	34	4.92712	2.13971	2.14873	Adipose_R2_peak_	Promoter (1-2	10	1.04E+08	1.04E+08	10977	1	1.00E+08	uc011xmx.1	-1800	ENSMUSG000	Gm4340	predicted gene 4340
chr10	104150108	104150201	94	95	104150156	39	6.54786	2.36605	3.60245	Adipose_R2_peak_	Promoter (1-2	10	1.04E+08	1.04E+08	10977	1	1.00E+08	uc011xmx.1	-1296	ENSMUSG000	Gm4340	predicted gene 4340
chr10	104493796	104494023	228	229	104493910	25	11.36566	4.67319	8.13482	Adipose_R1_peak_	Distal Intergen	10	1.04E+08	1.04E+08	108	1	1.02E+08	uc033fso.1	206527	ENSMUSG000	Mir6411	microRNA 6411
chr10	104499442	104499730	289	290	104499582	51	35.56531	9.34637	31.76235	Adipose_R1_peak_	Distal Intergen	10	1.04E+08	1.04E+08	108	1	1.02E+08	uc033fso.1	212173	ENSMUSG000	Mir6411	microRNA 6411
chr10	104499455	104499719	265	266	104499587	44	15.55653	4.1498	12.05625	Adipose_R2_peak_	Distal Intergen	10	1.04E+08	1.04E+08	108	1	1.02E+08	uc033fso.1	212186	ENSMUSG000	Mir6411	microRNA 6411
chr10	104558550	104558732	183	184	104558616	24	10.60668	4.49345	7.40961	Adipose_R1_peak_	Distal Intergen	10	1.04E+08	1.04E+08	108	1	1.02E+08	uc033fso.1	271281	ENSMUSG000	Mir6411	microRNA 6411
chr10	104558576	104558693	118	119	104558624	27	5.77405	2.5821	2.88921	Adipose_R2_peak_	Distal Intergen	10	1.04E+08	1.04E+08	108	1	1.02E+08	uc033fso.1	271307	ENSMUSG000	Mir6411	microRNA 6411
chr10	104576819	104577025	207	208	104576901	36	20.67077	6.6503	17.14779	Adipose_R1_peak_	Distal Intergen	10	1.04E+08	1.04E+08	108	1	1.02E+08	uc033fso.1	289550	ENSMUSG000	Mir6411	microRNA 6411
chr10	104621662	104621901	240	241	104621774	29	14.55748	5.39214	11.20652	Adipose_R1_peak_	Distal Intergen	10	1.04E+08	1.04E+08	108	1	1.02E+08	uc033fso.1	334393	ENSMUSG000	Mir6411	microRNA 6411
chr10	104696321	104696525	205	206	104696402	30	7.24477	2.85875	4.21917	Adipose_R2_peak_	Distal Intergen	10	1.04E+08	1.04E+08	108	1	1.02E+08	uc033fso.1	409052	ENSMUSG000	Mir6411	microRNA 6411
chr10	104749402	104749621	220	221	104749542	29	14.55748	5.39214	11.20652	Adipose_R1_peak_	Distal Intergen	10	1.04E+08	1.04E+08	108	1	1.02E+08	uc033fso.1	462133	ENSMUSG000	Mir6411	microRNA 6411
chr10	104751804	104751943	140	141	104751886	18	6.42934	3.41502	3.48999	Adipose_R1_peak_	Distal Intergen	10	1.04E+08	1.04E+08	108	1	1.02E+08	uc033fso.1	464535	ENSMUSG000	Mir6411	microRNA 6411
chr10	104834880	104835086	207	208	104834995	24	10.60668	4.49345	7.40961	Adipose_R1_peak_	Distal Intergen	10	1.04E+08	1.04E+08	108	1	1.02E+08	uc033fso.1	547611	ENSMUSG000	Mir6411	microRNA 6411
chr10	105238337	105238549	213	214	105238475	156	126.3891	14.47818	121.7923	Adipose_R2_peak_	Intron (uc007g	10	1.05E+08	1.06E+08	386816	2	278279	uc007gym.1	335930	ENSMUSG000	Tmtc2	transmembrane and tetratricopeptide repeat containing 2
chr10	105238341	105238549	209	210	105238470	119	87.67443	11.88471	83.40215	Adipose_R1_peak_	Intron (uc007g	10	1.05E+08	1.06E+08	386816	2	278279	uc007gym.1	335930	ENSMUSG000	Tmtc2	transmembrane and tetratricopeptide repeat containing 2
chr10	105248394	105248599	206	207	105248493	38	11.72962	3.59649	8.4047	Adipose_R2_peak_	Intron (uc007g	10	1.05E+08	1.06E+08	386816	2	278279	uc007gym.1	325880	ENSMUSG000	Tmtc2	transmembrane and tetratricopeptide repeat containing 2
chr10	105248413	105248554	142	143	105248489	21	8.43286	3.95423	5.34896	Adipose_R1_peak_	Intron (uc007g	10	1.05E+08	1.06E+08	386816	2	278279	uc007gym.1	325925	ENSMUSG000	Tmtc2	transmembrane and tetratricopeptide repeat containing 2
chr10	105907215	105907401	187	188	105907291	23	9.86443	4.31371	6.70247	Adipose_R1_peak_	Distal Intergen	10	1.06E+08	1.06E+08	6032	1	52713	uc007gyp.1	65736	ENSMUSG000	Ccdc59	coiled-coil domain containing 59
chr10	105924682	105924824	143	144	105924754	21	8.43286	3.95423	5.34896	Adipose_R1_peak_	Distal Intergen	10	1.06E+08	1.06E+08	6032	1	52713	uc007gyp.1	83203	ENSMUSG000	Ccdc59	coiled-coil domain containing 59
chr10	105931449	105931700	252	253	105931572	51	35.56531	9.34637	31.76235	Adipose_R1_peak_	Distal Intergen	10	1.06E+08	1.06E+08	6032	1	52713	uc007gyp.1	89970	ENSMUSG000	Ccdc59	coiled-coil domain containing 59
chr10	105931500	105931659	160	161	105931594	35	9.95877	3.31984	6.73562	Adipose_R2_peak_	Distal Intergen	10	1.06E+08	1.06E+08	6032	1	52713	uc007gyp.1	90021	ENSMUSG000	Ccdc59	coiled-coil domain containing 59
chr10	105967663	105967918	256	257	105967798	33	17.97508	6.11109	14.52164	Adipose_R1_peak_	Distal Intergen	10	1.06E+08	1.06E+08	6032	1	52713	uc007gyp.1	126184	ENSMUSG000	Ccdc59	coiled-coil domain containing 59
chr10	105967671	105967889	219	220	105967803	47	17.60017	4.42645	14.02531	Adipose_R2_peak_	Distal Intergen	10	1.06E+08	1.06E+08	6032	1	52713	uc007gyp.1	126192	ENSMUSG000	Ccdc59	coiled-coil domain containing 59
chr10	106191661	106191841	181	182	106191766	36	10.5378	3.41206	7.27949	Adipose_R2_peak_	Distal Intergen	10	1.06E+08	1.07E+08	463159	1	327814	uc007gyr.2	-278469	ENSMUSG000	Ppfia2	protein tyrosine phosphatase, receptor type, f polypeptide (PTPRF), interacting protein (liprin), alpha 2
chr10	106191668	106191832	165	166	106191758	22	9.13959	4.13397	6.01526	Adipose_R1_peak_	Distal Intergen	10	1.06E+08	1.07E+08	463159	1	327814	uc007gyr.2	-278478	ENSMUSG000	Ppfia2	protein tyrosine phosphatase, receptor type, f polypeptide (PTPRF), interacting protein (liprin), alpha 2
chr10	106218757	106219025	269	270	106218879	44	28.34544	8.08821	24.66239	Adipose_R1_peak_	Distal Intergen	10	1.06E+08	1.07E+08	463159	1	327814	uc007gyr.2	-251285	ENSMUSG000	Ppfia2	protein tyrosine phosphatase, receptor type, f polypeptide (PTPRF), interacting protein (liprin), alpha 2
chr10	106218770	106218999	230	231	106218877	45	16.22858	4.24202	12.70278	Adipose_R2_peak_	Distal Intergen	10	1.06E+08	1.07E+08	463159	1	327814	uc007gyr.2	-251311	ENSMUSG000	Ppfia2	protein tyrosine phosphatase, receptor type, f polypeptide (PTPRF), interacting protein (liprin), alpha 2
chr10	106285902	106286131	230	231	106286016	28	13.73721	5.2124	10.41463	Adipose_R1_peak_	Distal Intergen	10	1.06E+08	1.07E+08	463159	1	327814	uc007gyr.2	-184179	ENSMUSG000	Ppfia2	protein tyrosine phosphatase, receptor type, f polypeptide (PTPRF), interacting protein (liprin), alpha 2
chr10	106454465	106454605	141	142	106454530	20	7.74501	3.7745	4.70467	Adipose_R1_peak_	Distal Intergen	10	1.06E+08	1.07E+08	463159	1	327814	uc007gyr.2	-15705	ENSMUSG000	Ppfia2	protein tyrosine phosphatase, receptor type, f polypeptide (PTPRF), interacting protein (liprin), alpha 2
chr10	106507764	106507942	179	180	106507828	20	7.74501	3.7745	4.70467	Adipose_R1_peak_	Intron (uc007g	10	1.06E+08	1.07E+08	463159	1	327814	uc007gyr.2	37454	ENSMUSG000	Ppfia2	protein tyrosine phosphatase, receptor type, f polypeptide (PTPRF), interacting protein (liprin), alpha 2
chr10	106530617	106530801	185	186	106530687	23	9.86443	4.31371	6.70247	Adipose_R1_peak_	Intron (uc007g	10	1.06E+08	1.07E+08	463159	1	327814	uc007gyr.2	60307	ENSMUSG000	Ppfia2	protein tyrosine phosphatase, receptor type, f polypeptide (PTPRF), interacting protein (liprin), alpha 2
chr10	106883441	106883580	140	141	106883505	22	9.13959	4.13397	6.01526	Adipose_R1_peak_	Intron (uc007g	10	1.07E+08	1.07E+08	40463	1	327814	uc007gyu.2	-9426	ENSMUSG000	Ppfia2	protein tyrosine phosphatase, receptor type, f polypeptide (PTPRF), interacting protein (liprin), alpha 2
chr10	107329438	107329610	173	174	107329536	31	7.7623	2.95097	4.69388	Adipose_R2_peak_	Intron (uc007g	10	1.07E+08	1.07E+08	152750	1	108030	uc033fsp.1	57044	ENSMUSG000	Lin7a	lin-7 homolog A (C. elegans)
chr10	107329441	107329624	184	185	107329537	29	14.55748	5.39214	11.20652	Adipose_R1_peak_	Intron (uc007g	10	1.07E+08	1.07E+08	152750	1	108030	uc033fsp.1	57047	ENSMUSG000	Lin7a	lin-7 homolog A (C. elegans)
chr10	107557919	107558175	257	258	107558055	45	29.34988	8.26794	25.64927	Adipose_R1_peak_	Intron (uc007g	10	1.07E+08	1.07E+08	1870	2	17878	uc007gza.1	-63190	ENSMUSG000	Myf6	myogenic factor 6
chr10	107557960	107558173	214	215	107558056	47	17.60017	4.42645	14.02531	Adipose_R2_peak_	Intron (uc007g	10	1.07E+08	1.07E+08	1870	2	17878	uc007gza.1	-63231	ENSMUSG000	Myf6	myogenic factor 6
chr10	108003966	108004283	318	319	108004181	23	9.86443	4.31371	6.70247	Adipose_R1_peak_	Distal Intergen	10	1.08E+08	1.08E+08	31188	1	628877	uc056yid.1	57321	NA	Gm29685	predicted gene, 29685
chr10	108004133	108004243	111	112	108004191	32	8.29282	3.04319	5.18352	Adipose_R2_peak_	Distal Intergen	10	1.08E+08	1.08E+08	31188	1	628877	uc056yid.1	57488	NA	Gm29685	predicted gene, 29685
chr10	108889972	108890238	267	268	108890031	20	7.74501	3.7745	4.70467	Adipose_R1_peak_	Intron (uc007g	10	1.08E+08	1.09E+08	513326	2	20979	uc007gzg.2	120737	ENSMUSG000	Syt1	synaptotagmin I
chr10	108890395	108890598	204	205	108890424	18	6.42934	3.41502	3.48999	Adipose_R1_peak_	Intron (uc007g	10	1.08E+08	1.09E+08	513326	2	20979	uc007gzg.2	120377	ENSMUSG000	Syt1	synaptotagmin I
chr10	108996964	108997149	186	187	108997023	32	8.29282	3.04319	5.18352	Adipose_R2_peak_	Intron (uc007g	10	1.08E+08	1.09E+08	513326	2	20979	uc007gzg.2	13826	ENSMUSG000	Syt1	synaptotagmin I
chr10	109088561	109088799	239	240	109088675	37	21.59274	6.83004	18.04766	Adipose_R1_peak_	Distal Intergen	10	1.08E+08	1.09E+08	513326	2	20979	uc007gzg.2	-77586	ENSMUSG000	Syt1	synaptotagmin I
chr10	109088605	109088794	190	191	109088680	40	12.96465	3.78093	9.57654	Adipose_R2_peak_	Distal Intergen	10	1.08E+08	1.09E+08	513326	2	20979	uc007gzg.2	-77630	ENSMUSG000	Syt1	synaptotagmin I
chr10	109215637	109215828	192	193	109215728	33	8.83596	3.1354	5.68652	Adipose_R2_peak_	Distal Intergen	10	1.08E+08	1.09E+08	513326	2	20979	uc007gzg.2	-204662	ENSMUSG000	Syt1	synaptotagmin I
chr10	109215748	109215971	224	225	109215841	22	9.13959	4.13397	6.01526	Adipose_R1_peak_	Distal Intergen	10	1.08E+08	1.09E+08	513326	2	20979	uc007gzg.2	-204773	ENSMUSG000	Syt1	synaptotagmin I
chr10	109372936	109373247	312	313	109373156	41	25.38991	7.54899	21.76305	Adipose_R1_peak_	Distal Intergen	10	1.08E+08	1.09E+08	513326	2	20979	uc007gzg.2	-361961	ENSMUSG000	Syt1	synaptotagmin I
chr10	109407806	109407972	167	168	109407873	19	7.07687	3.59476	4.08458	Adipose_R1_peak_	Distal Intergen	10	1.08E+08	1.09E+08	513326	2	20979	uc007gzg.2	-396831	ENSMUSG000	Syt1	synaptotagmin I
chr10	109850669	109850821	153	154	109850737	33	8.83596	3.1354	5.68652	Adipose_R2_peak_	Intron (uc007g	10	1.10E+08	1.10E+08	140181	2	260315	uc007gzk.1	-27050	ENSMUSG000	Nav3	neuron navigator 3
chr10	110009267	110009506	240	241	110009399	52	31.86404	7.86718	28.12587	Adipose_R1_peak_	Distal Intergen	10	1.10E+08	1.10E+08	316781	2	260315	uc007gzl.1	-9048	ENSMUSG000	Nav3	neuron navigator 3
chr10	110009282	110009523	242	243	110009396	66	32.22715	6.17859	28.31407	Adipose_R2_peak_	Distal Intergen	10	1.10E+08	1.10E+08	316781	2	260315	uc007gzl.1	-9063	ENSMUSG000	Nav3	neuron navigator 3
chr10	110012315	110012484	170	171	110012367	30	7.24477	2.85875	4.21917	Adipose_R2_peak_	Distal Intergen	10	1.10E+08	1.10E+08	316781	2	260315	uc007gzl.1	-12096	ENSMUSG000	Nav3	neuron navigator 3
chr10	110844412	110844573	162	163	110844480	24	10.60668	4.49345	7.40961	Adipose_R1_peak_	Intron (uc007g	10	1.11E+08	1.11E+08	95334	2	75630	uc007gzq.2	51934	ENSMUSG000	1700020G17R	RIKEN cDNA 1700020G17 gene
chr10	110848642	110848849	208	209	110848737	30	15.39182	5.57188	12.01394	Adipose_R1_peak_	Intron (uc007g	10	1.11E+08	1.11E+08	95334	2	75630	uc007gzq.2	47658	ENSMUSG000	1700020G17R	RIKEN cDNA 1700020G17 gene
chr10	110848646	110848791	146	147	110848716	33	8.83596	3.1354	5.68652	Adipose_R2_peak_	Intron (uc007g	10	1.11E+08	1.11E+08	95334	2	75630	uc007gzq.2	47716	ENSMUSG000	1700020G17R	RIKEN cDNA 1700020G17 gene
chr10	112015901	112016076	176	177	112015997	26	12.14079	4.85292	8.87838	Adipose_R1_peak_	Distal Intergen	10	1.12E+08	1.12E+08	11817	2	73690	uc007haj.2	-18637	ENSMUSG000	Glipr1	GLI pathogenesis-related 1 (glioma)
chr10	112015933	112016055	123	124	112015995	28	6.25023	2.67431	3.31626	Adipose_R2_peak_	Distal Intergen	10	1.12E+08	1.12E+08	11817	2	73690	uc007haj.2	-18669	ENSMUSG000	Glipr1	GLI pathogenesis-related 1 (glioma)
chr10	112016293	112016435	143	144	112016355	21	8.43286	3.95423	5.34896	Adipose_R1_peak_	Distal Intergen	10	1.12E+08	1.12E+08	11817	2	73690	uc007haj.2	-19029	ENSMUSG000	Glipr1	GLI pathogenesis-related 1 (glioma)
chr10	112019097	112019460	364	365	112019313	146	114.4869	13.55601	109.9246	Adipose_R2_peak_	Distal Intergen	10	1.12E+08	1.12E+08	11817	2	73690	uc007haj.2	-21833	ENSMUSG000	Glipr1	GLI pathogenesis-related 1 (glioma)
chr10	112019106	112019473	368	369	112019300	140	152.2829	25.34304	147.7627	Adipose_R1_peak_	Distal Intergen	10	1.12E+08	1.12E+08	11817	2	73690	uc007haj.2	-21842	ENSMUSG000	Glipr1	GLI pathogenesis-related 1 (glioma)
chr10	112305641	112305910	270	271	112305761	53	37.70483	9.70585	33.87	Adipose_R1_peak_	Intron (uc007h	10	1.12E+08	1.12E+08	194752	1	268345	uc007hao.1	34518	ENSMUSG000	Kcnc2	potassium voltage gated channel, Shaw-related subfamily, member 2
chr10	112305702	112305840	139	140	112305754	32	8.29282	3.04319	5.18352	Adipose_R2_peak_	Intron (uc007h	10	1.12E+08	1.12E+08	194752	1	268345	uc007hao.1	34579	ENSMUSG000	Kcnc2	potassium voltage gated channel, Shaw-related subfamily, member 2
chr10	112327005	112327289	285	286	112327139	106	103.5054	19.23195	99.15611	Adipose_R1_peak_	Intron (uc007h	10	1.12E+08	1.12E+08	194752	1	268345	uc007hao.1	55882	ENSMUSG000	Kcnc2	potassium voltage gated channel, Shaw-related subfamily, member 2
chr10	112327023	112327278	256	257	112327135	70	35.62622	6.54746	31.65691	Adipose_R2_peak_	Intron (uc007h	10	1.12E+08	1.12E+08	194752	1	268345	uc007hao.1	55900	ENSMUSG000	Kcnc2	potassium voltage gated channel, Shaw-related subfamily, member 2
chr10	112508055	112508213	159	160	112508124	25	11.36566	4.67319	8.13482	Adipose_R1_peak_	Distal Intergen	10	1.12E+08	1.12E+08	10267	1	268345	uc011xnj.1	52447	ENSMUSG000	Kcnc2	potassium voltage gated channel, Shaw-related subfamily, member 2
chr10	112511845	112511982	138	139	112511906	20	7.74501	3.7745	4.70467	Adipose_R1_peak_	Distal Intergen	10	1.12E+08	1.12E+08	10267	1	268345	uc011xnj.1	56237	ENSMUSG000	Kcnc2	potassium voltage gated channel, Shaw-related subfamily, member 2
chr10	112599809	112600385	577	578	112599893	23	9.86443	4.31371	6.70247	Adipose_R1_peak_	Distal Intergen	10	1.13E+08	1.13E+08	58674	1	69355	uc007haq.1	-126541	ENSMUSG000	1700010J16Ri	RIKEN cDNA 1700010J16 gene
chr10	112600099	112600380	282	283	112600239	51	20.44827	4.79532	16.78434	Adipose_R2_peak_	Distal Intergen	10	1.13E+08	1.13E+08	58674	1	69355	uc007haq.1	-126546	ENSMUSG000	1700010J16Ri	RIKEN cDNA 1700010J16 gene
chr10	112603993	112604124	132	133	112604080	29	6.74062	2.76653	3.75989	Adipose_R2_peak_	Distal Intergen	10	1.13E+08	1.13E+08	58674	1	69355	uc007haq.1	-122802	ENSMUSG000	1700010J16Ri	RIKEN cDNA 1700010J16 gene
chr10	112604043	112604202	160	161	112604102	19	7.07687	3.59476	4.08458	Adipose_R1_peak_	Distal Intergen	10	1.13E+08	1.13E+08	58674	1	69355	uc007haq.1	-122724	ENSMUSG000	1700010J16Ri	RIKEN cDNA 1700010J16 gene
chr10	112687388	112687555	168	169	112687475	27	5.77405	2.5821	2.88921	Adipose_R2_peak_	Distal Intergen	10	1.13E+08	1.13E+08	58674	1	69355	uc007haq.1	-39371	ENSMUSG000	1700010J16Ri	RIKEN cDNA 1700010J16 gene
chr10	113920064	113920236	173	174	113920161	23	9.86443	4.31371	6.70247	Adipose_R1_peak_	Distal Intergen	10	1.14E+08	1.15E+08	402550	2	237553	uc007hat.2	881134	ENSMUSG000	Trhde	TRH-degrading enzyme
chr10	114100359	114100490	132	133	114100421	19	7.07687	3.59476	4.08458	Adipose_R1_peak_	Distal Intergen	10	1.14E+08	1.15E+08	402550	2	237553	uc007hat.2	700880	ENSMUSG000	Trhde	TRH-degrading enzyme
chr10	114167963	114168133	171	172	114168028	21	8.43286	3.95423	5.34896	Adipose_R1_peak_	Distal Intergen	10	1.14E+08	1.15E+08	402550	2	237553	uc007hat.2	633237	ENSMUSG000	Trhde	TRH-degrading enzyme
chr10	114167965	114168076	112	113	114168014	27	5.77405	2.5821	2.88921	Adipose_R2_peak_	Distal Intergen	10	1.14E+08	1.15E+08	402550	2	237553	uc007hat.2	633294	ENSMUSG000	Trhde	TRH-degrading enzyme
chr10	114171503	114171647	145	146	114171540	17	5.80341	3.23528	2.92378	Adipose_R1_peak_	Distal Intergen	10	1.14E+08	1.15E+08	402550	2	237553	uc007hat.2	629723	ENSMUSG000	Trhde	TRH-degrading enzyme
chr10	114333035	114333286	252	253	114333157	35	19.76029	6.47056	16.26028	Adipose_R1_peak_	Distal Intergen	10	1.14E+08	1.15E+08	402550	2	237553	uc007hat.2	468084	ENSMUSG000	Trhde	TRH-degrading enzyme
chr10	114333052	114333279	228	229	114333160	46	16.90986	4.33423	13.35898	Adipose_R2_peak_	Distal Intergen	10	1.14E+08	1.15E+08	402550	2	237553	uc007hat.2	468091	ENSMUSG000	Trhde	TRH-degrading enzyme
chr10	114536859	114537172	314	315	114537051	88	79.49821	15.99667	75.27097	Adipose_R1_peak_	Intron (uc007h	10	1.14E+08	1.15E+08	402550	2	237553	uc007hat.2	264198	ENSMUSG000	Trhde	TRH-degrading enzyme
chr10	114536943	114537167	225	226	114537056	49	19.00719	4.61089	15.38598	Adipose_R2_peak_	Intron (uc007h	10	1.14E+08	1.15E+08	402550	2	237553	uc007hat.2	264203	ENSMUSG000	Trhde	TRH-degrading enzyme
chr10	114598925	114599059	135	136	114598975	25	4.86612	2.39766	2.09018	Adipose_R2_peak_	Intron (uc007h	10	1.14E+08	1.15E+08	402550	2	237553	uc007hat.2	202311	ENSMUSG000	Trhde	TRH-degrading enzyme
chr10	114861929	114862271	343	344	114862130	97	86.97346	16.11521	82.70567	Adipose_R1_peak_	Distal Intergen	10	1.14E+08	1.15E+08	402550	2	237553	uc007hat.2	-60559	ENSMUSG000	Trhde	TRH-degrading enzyme
chr10	114861996	114862255	260	261	114862124	62	28.93052	5.80972	25.0743	Adipose_R2_peak_	Distal Intergen	10	1.14E+08	1.15E+08	402550	2	237553	uc007hat.2	-60626	ENSMUSG000	Trhde	TRH-degrading enzyme
chr10	114884204	114884489	286	287	114884394	92	84.7075	16.71563	80.45023	Adipose_R1_peak_	Distal Intergen	10	1.14E+08	1.15E+08	402550	2	237553	uc007hat.2	-82834	ENSMUSG000	Trhde	TRH-degrading enzyme
chr10	114884240	114884484	245	246	114884398	84	48.25301	7.83851	44.11785	Adipose_R2_peak_	Distal Intergen	10	1.14E+08	1.15E+08	402550	2	237553	uc007hat.2	-82870	ENSMUSG000	Trhde	TRH-degrading enzyme
chr10	117025622	117026205	584	585	117025888	47	29.36679	7.89317	25.66602	Adipose_R1_peak_	Distal Intergen	10	1.17E+08	1.17E+08	1428	1	237560	uc007hcr.1	-19136	ENSMUSG000	Lrrc10	leucine rich repeat containing 10
chr10	117025663	117026168	506	507	117025717	30	7.24477	2.85875	4.21917	Adipose_R2_peak_	Distal Intergen	10	1.17E+08	1.17E+08	1428	1	237560	uc007hcr.1	-19173	ENSMUSG000	Lrrc10	leucine rich repeat containing 10
chr10	118071250	118071505	256	257	118071376	52	36.63102	9.52611	32.81188	Adipose_R1_peak_	Distal Intergen	10	1.18E+08	1.18E+08	6353	1	17245	uc007hds.2	-70282	ENSMUSG000	Mdm1	transformed mouse 3T3 cell double minute 1
chr10	118071254	118071503	250	251	118071361	67	33.06755	6.27081	29.1399	Adipose_R2_peak_	Distal Intergen	10	1.18E+08	1.18E+08	6353	1	17245	uc007hds.2	-70284	ENSMUSG000	Mdm1	transformed mouse 3T3 cell double minute 1
chr10	118200510	118200605	96	97	118200582	27	5.77405	2.5821	2.88921	Adipose_R2_peak_	Distal Intergen	10	1.18E+08	1.18E+08	5083	1	50929	uc007hdv.2	-4359	ENSMUSG000	Il22	interleukin 22
chr10	118220556	118220687	132	133	118220592	25	4.86612	2.39766	2.09018	Adipose_R2_peak_	Distal Intergen	10	1.18E+08	1.18E+08	5083	1	50929	uc007hdv.2	15592	ENSMUSG000	Il22	interleukin 22
chr10	118228926	118229488	563	564	118229113	27	5.77405	2.5821	2.88921	Adipose_R2_peak_	Distal Intergen	10	1.18E+08	1.18E+08	5083	1	50929	uc007hdv.2	23962	ENSMUSG000	Il22	interleukin 22
chr10	118230745	118230898	154	155	118230782	26	5.31252	2.48988	2.47999	Adipose_R2_peak_	Distal Intergen	10	1.18E+08	1.18E+08	5083	1	50929	uc007hdv.2	25781	ENSMUSG000	Il22	interleukin 22
chr10	118231793	118232211	419	420	118232063	30	7.24477	2.85875	4.21917	Adipose_R2_peak_	Exon (uc011xn	10	1.18E+08	1.18E+08	5083	1	50929	uc007hdv.2	26829	ENSMUSG000	Il22	interleukin 22

chr10	118232331	118232524	194	195	118232415	31	7.73382	2.94319	4.68914	Adipose_R2_peak_	Exon (uc029rf	10	1.18E+08	1.18E+08	5083	1	50929	uc007hdv.2	27367	ENSMUSG000	Il22	interleukin 22
chr10	118239181	118239286	106	107	118239224	25	4.86612	2.39766	2.09018	Adipose_R2_peak_	Distal Interger	10	1.18E+08	1.18E+08	5083	1	50929	uc007hdv.2	34217	ENSMUSG000	Il22	interleukin 22
chr10	118243870	118244010	141	142	118243947	45	13.61973	3.63489	10.20153	Adipose_R2_peak_	Distal Interger	10	1.18E+08	1.18E+08	5083	1	50929	uc007hdv.2	38906	ENSMUSG000	Il22	interleukin 22
chr10	118244731	118244891	161	162	118244828	36	10.5378	3.41206	7.27949	Adipose_R2_peak_	Distal Interger	10	1.18E+08	1.18E+08	5083	1	50929	uc007hdv.2	39767	ENSMUSG000	Il22	interleukin 22
chr10	118245568	118245742	175	176	118245633	25	9.01874	3.7677	5.92906	Adipose_R1_peak_	Distal Interger	10	1.18E+08	1.18E+08	5083	1	50929	uc007hdv.2	40604	ENSMUSG000	Il22	interleukin 22
chr10	118307281	118307450	170	171	118307373	21	8.43286	3.95423	5.34896	Adipose_R1_peak_	Distal Interger	10	1.18E+08	1.18E+08	5409	2	116849	uc007hdx.1	-12243	ENSMUSG000	Iltifb	interleukin 10-related T cell-derived inducible factor beta
chr10	118324941	118325388	448	449	118324979	36	5.60871	2.24318	2.7652	Adipose_R2_peak_	Exon (uc029rh	10	1.18E+08	1.18E+08	5409	2	116849	uc007hdx.1	-29903	ENSMUSG000	Iltifb	interleukin 10-related T cell-derived inducible factor beta
chr10	118325829	118326282	454	455	118325906	33	7.09729	2.68666	4.1027	Adipose_R2_peak_	Exon (uc029rh	10	1.18E+08	1.18E+08	5409	2	116849	uc007hdx.1	-30791	ENSMUSG000	Iltifb	interleukin 10-related T cell-derived inducible factor beta
chr10	118423993	118424292	300	301	118424135	36	10.5378	3.41206	7.27949	Adipose_R2_peak_	Distal Interger	10	1.18E+08	1.18E+08	4849	1	15978	uc007hdy.2	-16754	ENSMUSG000	Ifng	interferon gamma
chr10	118424977	118425230	254	255	118425122	51	20.44827	4.79532	16.78434	Adipose_R2_peak_	Distal Interger	10	1.18E+08	1.18E+08	4849	1	15978	uc007hdy.2	-15816	ENSMUSG000	Ifng	interferon gamma
chr10	118425023	118425223	201	202	118425123	25	11.36566	4.67319	8.13482	Adipose_R1_peak_	Distal Interger	10	1.18E+08	1.18E+08	4849	1	15978	uc007hdy.2	-15823	ENSMUSG000	Ifng	interferon gamma
chr10	118427222	118427370	149	150	118427298	27	5.77405	2.5821	2.88921	Adipose_R2_peak_	Distal Interger	10	1.18E+08	1.18E+08	4849	1	15978	uc007hdy.2	-13676	ENSMUSG000	Ifng	interferon gamma
chr10	119236578	119236793	216	217	119236679	27	12.93148	5.03266	9.63827	Adipose_R1_peak_	Intron (uc007h	10	1.19E+08	1.19E+08	41244	2	71902	uc007hec.2	3262	ENSMUSG000	Cand1	cullin associated and neddylation disassociated 1
chr10	119236606	119236754	149	150	119236697	38	11.72962	3.59649	8.4047	Adipose_R2_peak_	Intron (uc007h	10	1.19E+08	1.19E+08	41244	2	71902	uc007hec.2	3301	ENSMUSG000	Cand1	cullin associated and neddylation disassociated 1
chr10	119642915	119643141	227	228	119642997	27	5.77405	2.5821	2.88921	Adipose_R2_peak_	Intron (uc007h	10	1.20E+08	1.20E+08	15638	2	75321	uc029rjh.1	118558	ENSMUSG000	Grip1os2	glutamate receptor interacting protein 1, opposite strand 2
chr10	119769633	119769841	209	210	119769726	30	15.39182	5.57188	12.01394	Adipose_R1_peak_	Intron (uc007h	10	1.20E+08	1.20E+08	15638	2	75321	uc029rjh.1	-7934	ENSMUSG000	Grip1os2	glutamate receptor interacting protein 1, opposite strand 2
chr10	120975759	120976216	458	459	120975906	21	8.43286	3.95423	5.34896	Adipose_R1_peak_	Intron (uc007h	10	1.21E+08	1.21E+08	55920	2	380664	uc007hfi.1	3114	ENSMUSG000	Lemd3	LEM domain containing 3
chr10	120975854	120976007	154	155	120975895	30	7.24477	2.85875	4.21917	Adipose_R2_peak_	Intron (uc007h	10	1.21E+08	1.21E+08	55920	2	380664	uc007hfi.1	3323	ENSMUSG000	Lemd3	LEM domain containing 3
chr10	120996313	120996505	193	194	120996400	26	12.14079	4.85292	8.87838	Adipose_R1_peak_	Distal Interger	10	1.21E+08	1.21E+08	55920	2	380664	uc007hfi.1	-16983	ENSMUSG000	Lemd3	LEM domain containing 3
chr10	121209814	121210022	209	210	121209891	36	10.5378	3.41206	7.27949	Adipose_R2_peak_	Distal Interger	10	1.21E+08	1.21E+08	30938	2	74694	uc007hfl.2	84735	ENSMUSG000	Tbc1d30	TBC1 domain family, member 30
chr10	121209831	121210022	192	193	121209917	23	9.86443	4.31371	6.70247	Adipose_R1_peak_	Distal Interger	10	1.21E+08	1.21E+08	30938	2	74694	uc007hfl.2	84735	ENSMUSG000	Tbc1d30	TBC1 domain family, member 30
chr10	121355040	121355224	185	186	121355203	22	6.91715	3.25564	3.97106	Adipose_R1_peak_	Distal Interger	10	1.21E+08	1.21E+08	7161	1	75612	uc007hfn.1	-9866	ENSMUSG000	Gns	glucosamine (N-acetyl)-6-sulfatase
chr10	121355082	121355294	213	214	121355215	36	10.5378	3.41206	7.27949	Adipose_R2_peak_	Distal Interger	10	1.21E+08	1.21E+08	7161	1	75612	uc007hfn.1	-9796	ENSMUSG000	Gns	glucosamine (N-acetyl)-6-sulfatase
chr10	121426587	121426817	231	232	121426746	31	7.7623	2.95097	4.69388	Adipose_R2_peak_	Intron (uc007h	10	1.21E+08	1.21E+08	14131	1	75612	uc007hfr.1	43472	ENSMUSG000	Gns	glucosamine (N-acetyl)-6-sulfatase
chr10	121428367	121428515	149	150	121428427	31	7.7623	2.95097	4.69388	Adipose_R2_peak_	Intron (uc007h	10	1.21E+08	1.21E+08	14131	1	75612	uc007hfr.1	45252	ENSMUSG000	Gns	glucosamine (N-acetyl)-6-sulfatase
chr10	121767850	121768036	187	188	121767932	23	9.86443	4.31371	6.70247	Adipose_R1_peak_	Distal Interger	10	1.22E+08	1.22E+08	12923	1	270802	uc007hfx.1	27913	ENSMUSG000	BC048403	cDNA sequence BC048403
chr10	121767918	121768047	130	131	121767986	28	6.25023	2.67431	3.31626	Adipose_R2_peak_	Distal Interger	10	1.22E+08	1.22E+08	12923	1	270802	uc007hfx.1	27981	ENSMUSG000	BC048403	cDNA sequence BC048403
chr10	122424311	122424628	318	319	122424484	60	45.43621	10.96401	41.50272	Adipose_R1_peak_	Distal Interger	10	1.22E+08	1.22E+08	4955	1	54140	uc007hge.1	-23871	ENSMUSG000	Avpr1a	arginine vasopressin receptor 1A
chr10	122424327	122424618	292	293	122424495	86	50.14197	8.02294	45.98602	Adipose_R2_peak_	Distal Interger	10	1.22E+08	1.22E+08	4955	1	54140	uc007hge.1	-23881	ENSMUSG000	Avpr1a	arginine vasopressin receptor 1A
chr10	122507844	122508141	298	299	122508010	67	33.06755	6.27081	29.1399	Adipose_R2_peak_	Distal Interger	10	1.22E+08	1.22E+08	4955	1	54140	uc007hge.1	59345	ENSMUSG000	Avpr1a	arginine vasopressin receptor 1A
chr10	122507850	122508132	283	284	122508001	69	51.50386	11.06356	47.5059	Adipose_R1_peak_	Distal Interger	10	1.22E+08	1.22E+08	4955	1	54140	uc007hge.1	59351	ENSMUSG000	Avpr1a	arginine vasopressin receptor 1A
chr10	122833597	122833754	158	159	122833693	23	9.86443	4.31371	6.70247	Adipose_R1_peak_	Intron (uc007h	10	1.23E+08	1.23E+08	85	2	387251	uc011xpd.1	151970	ENSMUSG000	Mirlet7i	microRNA let7i
chr10	122834896	122835025	130	131	122834948	31	7.7623	2.95097	4.69388	Adipose_R2_peak_	Intron (uc007h	10	1.23E+08	1.23E+08	85	2	387251	uc011xpd.1	150699	ENSMUSG000	Mirlet7i	microRNA let7i
chr10	122839253	122839503	251	252	122839382	44	28.34544	8.08821	24.66239	Adipose_R1_peak_	Intron (uc007h	10	1.23E+08	1.23E+08	85	2	387251	uc011xpd.1	146221	ENSMUSG000	Mirlet7i	microRNA let7i
chr10	122839307	122839456	150	151	122839390	38	11.72962	3.59649	8.4047	Adipose_R2_peak_	Intron (uc007h	10	1.23E+08	1.23E+08	85	2	387251	uc011xpd.1	146268	ENSMUSG000	Mirlet7i	microRNA let7i
chr10	122936544	122936741	198	199	122936621	30	7.24477	2.85875	4.21917	Adipose_R2_peak_	Intron (uc007h	10	1.23E+08	1.23E+08	85	2	387251	uc011xpd.1	48983	ENSMUSG000	Mirlet7i	microRNA let7i
chr10	122953766	122954001	236	237	122953904	20	7.74501	3.7745	4.70467	Adipose_R1_peak_	Distal Interger	10	1.23E+08	1.23E+08	85	2	387251	uc011xpd.1	31723	ENSMUSG000	Mirlet7i	microRNA let7i
chr10	122987680	122987818	139	140	122987704	25	4.86612	2.39766	2.09018	Adipose_R2_peak_	Promoter (1-2	10	1.23E+08	1.23E+08	85	2	387251	uc011xpd.1	-1956	ENSMUSG000	Mirlet7i	microRNA let7i
chr10	123324282	123324493	212	213	123324486	57	24.96362	5.34863	21.18596	Adipose_R2_peak_	Intron (uc007h	10	1.23E+08	1.24E+08	477129	1	268354	uc007hgu.2	60206	ENSMUSG000	Fam19a2	family with sequence similarity 19, member A2
chr10	123324318	123324493	176	177	123324490	24	10.60668	4.49345	7.40961	Adipose_R1_peak_	Intron (uc007h	10	1.23E+08	1.24E+08	477129	1	268354	uc007hgu.2	60242	ENSMUSG000	Fam19a2	family with sequence similarity 19, member A2
chr10	123332930	123333108	179	180	123332994	19	7.07687	3.59476	4.08458	Adipose_R1_peak_	Intron (uc007h	10	1.23E+08	1.24E+08	477129	1	268354	uc007hgu.2	68854	ENSMUSG000	Fam19a2	family with sequence similarity 19, member A2
chr10	123795960	123796246	287	288	123796115	57	42.07807	10.4248	38.18469	Adipose_R1_peak_	Distal Interger	10	1.23E+08	1.24E+08	477129	1	268354	uc007hgu.2	531884	ENSMUSG000	Fam19a2	family with sequence similarity 19, member A2
chr10	123795984	123796212	229	230	123796104	58	25.74274	5.44085	21.94821	Adipose_R2_peak_	Distal Interger	10	1.23E+08	1.24E+08	477129	1	268354	uc007hgu.2	531908	ENSMUSG000	Fam19a2	family with sequence similarity 19, member A2
chr10	123862516	123862752	237	238	123862629	30	15.39182	5.57188	12.01394	Adipose_R1_peak_	Distal Interger	10	1.23E+08	1.24E+08	477129	1	268354	uc007hgu.2	598440	ENSMUSG000	Fam19a2	family with sequence similarity 19, member A2
chr10	123862532	123862740	209	210	123862631	39	12.34187	3.68871	8.98501	Adipose_R2_peak_	Distal Interger	10	1.23E+08	1.24E+08	477129	1	268354	uc007hgu.2	598456	ENSMUSG000	Fam19a2	family with sequence similarity 19, member A2
chr10	123964612	123964861	250	251	123964738	30	15.39182	5.57188	12.01394	Adipose_R1_peak_	Distal Interger	10	1.24E+08	1.25E+08	55415	1	75030	uc011xpg.1	-504213	NA	4930503E24Ri	eukaryotic translation initiation factor 3, subunit I pseudogene
chr10	123964675	123964877	203	204	123964820	39	12.34187	3.68871	8.98501	Adipose_R2_peak_	Distal Interger	10	1.24E+08	1.25E+08	55415	1	75030	uc011xpg.1	-504197	NA	4930503E24Ri	eukaryotic translation initiation factor 3, subunit I pseudogene
chr10	124207718	124208083	366	367	124207799	53	37.70483	9.70585	33.87	Adipose_R1_peak_	Distal Interger	10	1.24E+08	1.25E+08	55415	1	75030	uc011xpg.1	-260991	NA	4930503E24Ri	eukaryotic translation initiation factor 3, subunit I pseudogene
chr10	124232728	124233059	332	333	124232869	142	155.2721	25.70252	150.7431	Adipose_R1_peak_	Distal Interger	10	1.24E+08	1.25E+08	55415	1	75030	uc011xpg.1	-236015	NA	4930503E24Ri	eukaryotic translation initiation factor 3, subunit I pseudogene
chr10	124235376	124235562	187	188	124235549	42	14.2409	3.96536	10.79479	Adipose_R2_peak_	Distal Interger	10	1.24E+08	1.25E+08	55415	1	75030	uc011xpg.1	-233512	NA	4930503E24Ri	eukaryotic translation initiation factor 3, subunit I pseudogene
chr10	124235404	124235561	158	159	124235533	22	9.13959	4.13397	6.01526	Adipose_R1_peak_	Distal Interger	10	1.24E+08	1.25E+08	55415	1	75030	uc011xpg.1	-233513	NA	4930503E24Ri	eukaryotic translation initiation factor 3, subunit I pseudogene
chr10	124430014	124430222	209	210	124430118	27	12.93148	5.03266	9.63827	Adipose_R1_peak_	Distal Interger	10	1.24E+08	1.25E+08	55415	1	75030	uc011xpg.1	-38852	NA	4930503E24Ri	eukaryotic translation initiation factor 3, subunit I pseudogene
chr10	124467849	124468056	208	209	124467954	39	23.47001	7.18952	19.88325	Adipose_R1_peak_	Promoter (1-2	10	1.24E+08	1.25E+08	55415	1	75030	uc011xpg.1	-1018	NA	4930503E24Ri	eukaryotic translation initiation factor 3, subunit I pseudogene
chr10	124467853	124468046	194	195	124467975	37	11.12817	3.50427	7.83613	Adipose_R2_peak_	Promoter (1-2	10	1.24E+08	1.25E+08	55415	1	75030	uc011xpg.1	-1028	NA	4930503E24Ri	eukaryotic translation initiation factor 3, subunit I pseudogene
chr10	124558137	124558283	147	148	124558201	16	5.20017	3.05554	2.38734	Adipose_R1_peak_	Distal Interger	10	1.24E+08	1.25E+08	55415	1	75030	uc011xpg.1	89063	NA	4930503E24Ri	eukaryotic translation initiation factor 3, subunit I pseudogene
chr10	124760733	124760902	170	171	124760805	27	12.93148	5.03266	9.63827	Adipose_R1_peak_	Distal Interger	10	1.24E+08	1.25E+08	55415	1	75030	uc011xpg.1	291659	NA	4930503E24Ri	eukaryotic translation initiation factor 3, subunit I pseudogene
chr10	124931100	124931248	149	150	124931164	20	7.74501	3.7745	4.70467	Adipose_R1_peak_	Distal Interger	10	1.25E+08	1.25E+08	81389	2	20503	uc007hgx.1	377625	ENSMUSG000	Slc16a7	solute carrier family 16 (monocarboxylic acid transporters), member 7
chr10	125410675	125410893	219	220	125410748	19	7.07687	3.59476	4.08458	Adipose_R1_peak_	Distal Interger	10	1.25E+08	1.25E+08	101051	2	20503	uc007hgy.1	-82140	ENSMUSG000	Slc16a7	solute carrier family 16 (monocarboxylic acid transporters), member 7
chr10	125635801	125636322	522	523	125635953	51	36.63102	9.52611	32.81188	Adipose_R1_peak_	Distal Interger	10	1.25E+08	1.25E+08	101051	2	20503	uc007hgy.1	-307266	ENSMUSG000	Slc16a7	solute carrier family 16 (monocarboxylic acid transporters), member 7
chr10	125635841	125636221	381	382	125635928	34	9.39139	3.22762	6.20474	Adipose_R2_peak_	Distal Interger	10	1.25E+08	1.25E+08	101051	2	20503	uc007hgy.1	-307306	ENSMUSG000	Slc16a7	solute carrier family 16 (monocarboxylic acid transporters), member 7
chr10	125715694	125716058	365	366	125715806	19	7.07687	3.59476	4.08458	Adipose_R1_peak_	Distal Interger	10	1.26E+08	1.26E+08	31795	1	320398	uc007hhb.2	-250161	ENSMUSG000	Lrig3	leucine-rich repeats and immunoglobulin-like domains 3
chr10	125716276	125716470	195	196	125716341	29	14.55748	5.39214	11.20652	Adipose_R1_peak_	Distal Interger	10	1.26E+08	1.26E+08	31795	1	320398	uc007hhb.2	-249749	ENSMUSG000	Lrig3	leucine-rich repeats and immunoglobulin-like domains 3
chr10	125824999	125825257	259	260	125825122	49	33.45876	8.98689	29.68925	Adipose_R1_peak_	Distal Interger	10	1.26E+08	1.26E+08	31795	1	320398	uc007hhb.2	-140962	ENSMUSG000	Lrig3	leucine-rich repeats and immunoglobulin-like domains 3
chr10	125825044	125825192	149	150	125825136	34	9.39139	3.22762	6.20474	Adipose_R2_peak_	Distal Interger	10	1.26E+08	1.26E+08	31795	1	320398	uc007hhb.2	-141027	ENSMUSG000	Lrig3	leucine-rich repeats and immunoglobulin-like domains 3
chr10	126111572	126111676	105	106	126111603	28	6.25023	2.67431	3.31626	Adipose_R2_peak_	Distal Interger	10	1.26E+08	1.26E+08	31795	1	320398	uc007hhb.2	145353	ENSMUSG000	Lrig3	leucine-rich repeats and immunoglobulin-like domains 3
chr10	126287867	126288293	427	428	126287967	21	8.43286	3.95423	5.34896	Adipose_R1_peak_	Distal Interger	10	1.26E+08	1.26E+08	31795	1	320398	uc007hhb.2	321648	ENSMUSG000	Lrig3	leucine-rich repeats and immunoglobulin-like domains 3
chr10	126288125	126288245	121	122	126288169	28	6.25023	2.67431	3.31626	Adipose_R2_peak_	Distal Interger	10	1.26E+08	1.26E+08	31795	1	320398	uc007hhb.2	321906	ENSMUSG000	Lrig3	leucine-rich repeats and immunoglobulin-like domains 3
chr10	126866654	126866846	193	194	126866686	16	5.20017	3.05554	2.38734	Adipose_R1_peak_	Downstream (	10	1.27E+08	1.27E+08	32944	2	68876	uc007hhf.2	34525	ENSMUSG000	Xrcc6bp1	XRCC6 binding protein 1
chr10	126871567	126871698	132	133	126871672	33	8.83596	3.1354	5.68652	Adipose_R2_peak_	Intron (uc007h	10	1.27E+08	1.27E+08	32944	2	68876	uc007hhf.2	29673	ENSMUSG000	Xrcc6bp1	XRCC6 binding protein 1
chr10	126970702	126970860	159	160	126970776	16	5.20017	3.05554	2.38734	Adipose_R1_peak_	Distal Interger	10	1.27E+08	1.27E+08	21259	1	52468	uc007hhj.2	-7857	ENSMUSG000	Ctdsp2	CTD (carboxy-terminal domain, RNA polymerase II, polypeptide A) small phosphatase 2
chr10	126980426	126980626	201	202	126980543	29	6.74062	2.76653	3.75989	Adipose_R2_peak_	Promoter (1-2	10	1.27E+08	1.27E+08	21259	1	52468	uc007hhj.2	1709	ENSMUSG000	Ctdsp2	CTD (carboxy-terminal domain, RNA polymerase II, polypeptide A) small phosphatase 2
chr10	127289691	127290329	639	640	127289836	21	7.61919	3.6177	4.62019	Adipose_R1_peak_	Promoter (<=	10	1.27E+08	1.27E+08	5496	1	13198	uc007hiy.2	-464	ENSMUSG000	Ddit3	DNA-damage inducible transcript 3
chr10	127290179	127290412	234	235	127290264	32	8.29282	3.04319	5.18352	Adipose_R2_peak_	Promoter (<=	10	1.27E+08	1.27E+08	5496	1	13198	uc007hiy.2	-381	ENSMUSG000	Ddit3	DNA-damage inducible transcript 3
chr10	127290805	127290943	139	140	127290862	19	7.07687	3.59476	4.08458	Adipose_R1_peak_	Promoter (<=	10	1.27E+08	1.27E+08	5496	1	13198	uc007hiy.2	12	ENSMUSG000	Ddit3	DNA-damage inducible transcript 3
chr10	127294312	127294418	107	108	127294324	25	4.86612	2.39766	2.09018	Adipose_R2_peak_	Exon (uc057ax	10	1.27E+08	1.27E+08	5496	1	13198	uc007hiy.2	3519	ENSMUSG000	Ddit3	DNA-damage inducible transcript 3
chr10	127669625	127669914	290	291	127669863	31	7.7623	2.95097	4.69388	Adipose_R2_peak_	Promoter (2-3	10	1.28E+08	1.28E+08	33925	1	210035	uc007hke.2	2502	ENSMUSG000	Nemp1	nuclear envelope integral membrane protein 1
chr10	127669676	127669960	285	286	127669844	19	7.07687	3.59476	4.08458	Adipose_R1_peak_	Promoter (2-3	10	1.28E+08	1.28E+08	33925	1	210035	uc007hke.2	2553	ENSMUSG000	Nemp1	nuclear envelope integral membrane protein 1
chr10	127670820	127670959	140	141	127670863	26	5.31252	2.48988	2.47999	Adipose_R2_peak_	Intron (uc007h	10	1.28E+08	1.28E+08	33925	1	210035	uc007hke.2	3697	ENSMUSG000	Nemp1	nuclear envelope integral membrane protein 1
chr10	127672404	127672678	275	276	127672565	28	6.25023	2.67431	3.31626	Adipose_R2_peak_	Intron (uc007h	10	1.28E+08	1.28E+08	18102	1	210035	uc007hkf.2	-4387	ENSMUSG000	Nemp1	nuclear envelope integral membrane protein 1
chr10	127674987	127675278	292	293	127675064	29	6.74062	2.76653	3.75989	Adipose_R2_peak_	Promoter (1-2	10	1.28E+08	1.28E+08	18102	1	210035	uc007hkf.2	-1787	ENSMUSG000	Nemp1	nuclear envelope integral membrane protein 1
chr10	127675277	127675609	333	334	127675514	21	8.43286	3.95423	5.34896	Adipose_R1_peak_	Promoter (1-2	10	1.28E+08	1.28E+08	18102	1	210035	uc007hkf.2	-1456	ENSMUSG000	Nemp1	nuclear envelope integral membrane protein 1
chr10	127676174	127676385	212	213	127676260	28	6.25023	2.67431	3.31626	Adipose_R2_peak_	Promoter (<=	10	1.28E+08	1.28E+08	18102	1	210035	uc007hkf.2	-680	ENSMUSG000	Nemp1	nuclear envelope integral membrane protein 1
chr10	127932187	127932385	199	200	127932321	36	20.67077	6.6503	17.14779	Adipose_R1_peak_	Distal Interger	10	1.28E+08	1.28E+08	9685	1	70061	uc007hkx.1	30112	ENSMUSG000	Sdr9c7	4short chain dehydrogenase/reductase family 9C, member 7
chr10	127932233	127932340	108	109	127932299	28	6.25023	2.67431	3.31626	Adipose_R2_peak_	Distal Interger	10	1.28E+08	1.28E+08	9685	1	70061	uc007hkx.1	30158	ENSMUSG000	Sdr9c7	4short chain dehydrogenase/reductase family 9C, member 7
chr10	128085998	128086213	216	217	128086045	27	5.77405	2.5821	2.88921	Adipose_R2_peak_	Promoter (<=	10	1.28E+08	1.28E+08	78	1	751518	uc011xpy.2	712	ENSMUSG000	Mir677	microRNA 677
chr10	128086388	128086545	158	159	128086436	19	7.07687	3.59476	4.08458	Adipose_R1_peak_	Promoter (1-2	10	1.28E+08	1.28E+08	78	1	751518	uc011xpy.2	1102	ENSMUSG000	Mir677	microRNA 677
chr10	128087790	128087916	127	128	128087835	29	6.74062	2.76653	3.75989	Adipose_R2_peak_	Promoter (2-3	10	1.28E+08	1.28E+08	78	1	751518	uc011xpy.2	2504	ENSMUSG000	Mir677	microRNA 677
chr10	128088322	128088458	137	138	128088384	18	6.42934	3.41502	3.48999	Adipose_R1_peak_	Exon (uc007hl	10	1.28E+08	1.28E+08	78	1	751518	uc011xpy.2	3036	ENSMUSG000	Mir677	microRNA 677
chr10	128492658	128492769	112	113	128492701	26	5.31252	2.48988	2.47999	Adipose_R2_peak_	Promoter (<=	10	1.28E+08	1.28E+08	2151	2	17904	uc007hnb.2	240	ENSMUSG000	Myl6	myosin, light polypeptide 6, alkali, smooth muscle and non-muscle
chr10	128494388	128495326	939	940	128494523	33	8.83596	3.1354	5.68652	Adipose_R2_peak_	Promoter (<=	10	1.28E+08	1.28E+08	3028	2	17904	uc007hnc.2	-502	ENSMUSG000	Myl6	myosin, light polypeptide 6, alkali, smooth muscle and non-muscle
chr10	128495137	128495409	273	274	128495219	20	7.4905	3.66653	4.49313	Adipose_R1_peak_	Promoter (1-2	10	1.28E+08	1.28E+08	3028	2	17904	uc007hnc.2	-1251	ENSMUSG000	Myl6	myosin, light polypeptide 6, alkali, smooth muscle and non-muscle
chr10	128495564	128495749	186	187	128495650	21	8.43286	3.95423	5.34896	Adipose_R1_peak_	Promoter (1-2	10	1.28E+08	1.28E+08	3028	2	17904	uc007hnc.2	-1678	ENSMUSG000	Myl6	myosin, light polypeptide 6, alkali, smooth muscle and non-muscle
chr10	128695131	128695315	185	186	128695262	17	5.80341	3.23528	2.92378	Adipose_R1_peak_	Promoter (<=	10	1.29E+08	1.29E+08	14430	2	19344	uc007hnw.1	917	ENSMUSG000	Rab5b	RAB5B, member RAS oncogene family
chr10	128696964	128697075	112	113	128697034	27	5.77405	2.5821	2.88921	Adipose_R2_peak_	Promoter (<=	10	1.29E+08	1.29E+08	19086	2	19344	uc007hnv.2	-696	ENSMUSG000	Rab5b	RAB5B, member RAS oncogene family

chr10	128699126	128699377	252	253	128699282	29	6.74062	2.76653	3.75989	Adipose_R2_peak_	Promoter (2-3	10	1.29E+08	1.29E+08	19086	2	19344	uc007hnv.2	-2858	ENSMUSG000	Rab5b	RAB5B, member RAS oncogene family
chr10	128700193	128700377	185	186	128700271	27	5.77405	2.5821	2.88921	Adipose_R2_peak_	Intron (uc007h	10	1.29E+08	1.29E+08	19086	2	19344	uc007hnv.2	-3925	ENSMUSG000	Rab5b	RAB5B, member RAS oncogene family
chr10	128701178	128701271	94	95	128701266	29	6.74062	2.76653	3.75989	Adipose_R2_peak_	Intron (uc007h	10	1.29E+08	1.29E+08	7088	2	12566	uc007hoa.1	3755	ENSMUSG000	Cdk2	cyclin-dependent kinase 2
chr10	128701186	128701324	139	140	128701285	19	7.07687	3.59476	4.08458	Adipose_R1_peak_	Intron (uc007h	10	1.29E+08	1.29E+08	7088	2	12566	uc007hoa.1	3702	ENSMUSG000	Cdk2	cyclin-dependent kinase 2
chr10	128701718	128701905	188	189	128701800	19	7.07687	3.59476	4.08458	Adipose_R1_peak_	Intron (uc007h	10	1.29E+08	1.29E+08	7088	2	12566	uc007hoa.1	3121	ENSMUSG000	Cdk2	cyclin-dependent kinase 2
chr10	128701875	128702069	195	196	128701973	27	5.77405	2.5821	2.88921	Adipose_R2_peak_	Promoter (2-3	10	1.29E+08	1.29E+08	7088	2	12566	uc007hoa.1	2957	ENSMUSG000	Cdk2	cyclin-dependent kinase 2
chr10	128703047	128703250	204	205	128703128	34	9.39139	3.22762	6.20474	Adipose_R2_peak_	Promoter (1-2	10	1.29E+08	1.29E+08	7088	2	12566	uc007hoa.1	1776	ENSMUSG000	Cdk2	cyclin-dependent kinase 2
chr10	128703622	128703756	135	136	128703686	26	5.31252	2.48988	2.47999	Adipose_R2_peak_	Promoter (1-2	10	1.29E+08	1.29E+08	7088	2	12566	uc007hoa.1	1270	ENSMUSG000	Cdk2	cyclin-dependent kinase 2
chr10	128808946	128809091	146	147	128808968	17	5.80341	3.23528	2.92378	Adipose_R1_peak_	Intron (uc007h	10	1.29E+08	1.29E+08	13771	1	74330	uc007hom.1	3270	ENSMUSG000	Dnajc14	DnaJ heat shock protein family (Hsp40) member C14
chr10	128811252	128811462	211	212	128811326	20	7.74501	3.7745	4.70467	Adipose_R1_peak_	Intron (uc007h	10	1.29E+08	1.29E+08	13771	1	74330	uc007hom.1	5576	ENSMUSG000	Dnajc14	DnaJ heat shock protein family (Hsp40) member C14
chr10	128811267	128811418	152	153	128811296	28	5.45352	2.4509	2.61564	Adipose_R2_peak_	Intron (uc007h	10	1.29E+08	1.29E+08	13771	1	74330	uc007hom.1	5591	ENSMUSG000	Dnajc14	DnaJ heat shock protein family (Hsp40) member C14
chr10	128814706	128814850	145	146	128814765	19	7.07687	3.59476	4.08458	Adipose_R1_peak_	3' UTR	10	1.29E+08	1.29E+08	4175	2	66844	uc007hoo.2	6781	ENSMUSG000	Ormdl2	ORM1-like 2 (S. cerevisiae)
chr10	128820641	128820786	146	147	128820685	30	7.24477	2.85875	4.21917	Adipose_R2_peak_	Promoter (<=	10	1.29E+08	1.29E+08	4175	2	66844	uc007hoo.2	845	ENSMUSG000	Ormdl2	ORM1-like 2 (S. cerevisiae)
chr10	129190660	129190936	277	278	129190813	82	71.83084	14.91825	67.65268	Adipose_R1_peak_	Distal Intergen	10	1.29E+08	1.29E+08	936	2	257664	uc007hpo.1	-3241	ENSMUSG000	Olfr773	olfactory receptor 773
chr10	129190703	129190928	226	227	129190804	54	22.67101	5.07198	18.94758	Adipose_R2_peak_	Distal Intergen	10	1.29E+08	1.29E+08	936	2	257664	uc007hpo.1	-3284	ENSMUSG000	Olfr773	olfactory receptor 773
chr10	129212636	129212833	198	199	129212731	24	10.60668	4.49345	7.40961	Adipose_R1_peak_	Distal Intergen	10	1.29E+08	1.29E+08	936	2	257664	uc007hpo.1	-25217	ENSMUSG000	Olfr773	olfactory receptor 773
chr10	129241079	129241344	266	267	129241203	50	34.50784	9.16663	30.72138	Adipose_R1_peak_	Promoter (2-3	10	1.29E+08	1.29E+08	939	1	258232	uc007hpp.1	2928	ENSMUSG000	Olfr774	olfactory receptor 774
chr10	129241096	129241318	223	224	129241192	50	19.72356	4.7031	16.08069	Adipose_R2_peak_	Promoter (2-3	10	1.29E+08	1.29E+08	939	1	258232	uc007hpp.1	2945	ENSMUSG000	Olfr774	olfactory receptor 774
chr10	129342018	129342314	297	298	129342151	57	42.07807	10.4248	38.18469	Adipose_R1_peak_	Distal Intergen	10	1.29E+08	1.29E+08	945	1	257985	uc007hpv.1	-8251	ENSMUSG000	Olfr782	olfactory receptor 782
chr10	129342066	129342251	186	187	129342144	40	12.96465	3.78093	9.57654	Adipose_R2_peak_	Distal Intergen	10	1.29E+08	1.29E+08	945	1	257985	uc007hpv.1	-8314	ENSMUSG000	Olfr782	olfactory receptor 782
chr10	129450871	129451144	274	275	129450983	48	32.41826	8.80716	28.66523	Adipose_R1_peak_	Distal Intergen	10	1.29E+08	1.29E+08	939	1	258069	uc007hpy.1	-11534	ENSMUSG000	Olfr787	olfactory receptor 787
chr10	129450921	129451070	150	151	129450988	33	8.83596	3.1354	5.68652	Adipose_R2_peak_	Distal Intergen	10	1.29E+08	1.29E+08	939	1	258069	uc007hpy.1	-11608	ENSMUSG000	Olfr787	olfactory receptor 787
chr10	129453324	129453518	195	196	129453405	18	6.42934	3.41502	3.48999	Adipose_R1_peak_	Distal Intergen	10	1.29E+08	1.29E+08	939	1	258069	uc007hpy.1	-9160	ENSMUSG000	Olfr787	olfactory receptor 787
chr10	129971238	129971382	145	146	129971297	27	10.3635	4.05752	7.19744	Adipose_R1_peak_	Distal Intergen	10	1.30E+08	1.30E+08	963	2	1.00E+08	uc011xqm.1	-4540	ENSMUSG000	Olfr819	olfactory receptor 819
chr10	130043083	130043262	180	181	130043197	25	11.36566	4.67319	8.13482	Adipose_R1_peak_	Distal Intergen	10	1.30E+08	1.30E+08	933	1	258772	uc007hrb.1	9455	ENSMUSG000	Olfr821	olfactory receptor 821
chr10	130043139	130043266	128	129	130043205	31	7.7623	2.95097	4.69388	Adipose_R2_peak_	Distal Intergen	10	1.30E+08	1.30E+08	933	1	258772	uc007hrb.1	9511	ENSMUSG000	Olfr821	olfactory receptor 821
chr10	130051450	130051613	164	165	130051535	23	9.86443	4.31371	6.70247	Adipose_R1_peak_	Distal Intergen	10	1.30E+08	1.30E+08	933	1	258772	uc007hrb.1	17822	ENSMUSG000	Olfr821	olfactory receptor 821
chr10	130219028	130219269	242	243	130219147	45	29.34988	8.26794	25.64927	Adipose_R1_peak_	Distal Intergen	10	1.30E+08	1.30E+08	969	2	258297	uc007hrh.1	-7900	ENSMUSG000	Olfr827	olfactory receptor 827
chr10	130219036	130219272	237	238	130219151	65	31.39315	6.08637	27.49408	Adipose_R2_peak_	Distal Intergen	10	1.30E+08	1.30E+08	969	2	258297	uc007hrh.1	-7908	ENSMUSG000	Olfr827	olfactory receptor 827
chr10	130310863	130310994	132	133	130310926	20	7.74501	3.7745	4.70467	Adipose_R1_peak_	Distal Intergen	10	1.30E+08	1.30E+08	39791	1	67596	uc007hrj.3	-11858	ENSMUSG000	Tespa1	thymocyte expressed, positive selection associated 1
chr10	130333191	130333374	184	185	130333292	46	30.36364	8.44768	26.64488	Adipose_R1_peak_	Intron (uc007h	10	1.30E+08	1.30E+08	39791	1	67596	uc007hrj.3	10339	ENSMUSG000	Tespa1	thymocyte expressed, positive selection associated 1
chr10	130333234	130333376	143	144	130333294	35	9.95877	3.31984	6.73562	Adipose_R2_peak_	Intron (uc007h	10	1.30E+08	1.30E+08	39791	1	67596	uc007hrj.3	10382	ENSMUSG000	Tespa1	thymocyte expressed, positive selection associated 1
chr10	130564629	130564812	184	185	130564752	39	12.34187	3.68871	8.98501	Adipose_R2_peak_	Intron (uc029r	10	1.30E+08	1.30E+08	25559	2	625131	uc009vae.1	-67250	ENSMUSG000	Vmn2r87	vomeronasal 2, receptor 87
chr10	130564636	130564859	224	225	130564756	29	14.55748	5.39214	11.20652	Adipose_R1_peak_	Intron (uc029r	10	1.30E+08	1.30E+08	25559	2	625131	uc009vae.1	-67257	ENSMUSG000	Vmn2r87	vomeronasal 2, receptor 87
chr10	130594408	130594992	585	586	130594532	36	10.52404	3.40849	7.35657	Adipose_R1_peak_	Distal Intergen	10	1.30E+08	1.30E+08	25559	2	625131	uc009vae.1	-97029	ENSMUSG000	Vmn2r87	vomeronasal 2, receptor 87
chr10	130594780	130594988	209	210	130594880	201	135.7383	10.88876	131.1185	Adipose_R2_peak_	Distal Intergen	10	1.30E+08	1.30E+08	25559	2	625131	uc009vae.1	-97401	ENSMUSG000	Vmn2r87	vomeronasal 2, receptor 87
chr11	3100384	3100574	191	192	3100475	135	30.8783	3.2382	26.99038	Adipose_R2_peak_	Distal Intergen	11	3124021	3131944	7924	1	236604	uc007hrp.1	-23447	NA	Pisd-ps1	phosphatidylserine decarboxylase, pseudogene 1
chr11	3101097	3101383	287	288	3101130	31	5.53258	2.36716	2.69172	Adipose_R2_peak_	Distal Intergen	11	3124021	3131944	7924	1	236604	uc007hrp.1	-22638	NA	Pisd-ps1	phosphatidylserine decarboxylase, pseudogene 1
chr11	3101483	3101750	268	269	3101577	33	6.83901	2.62249	3.89387	Adipose_R1_peak_	Distal Intergen	11	3124021	3131944	7924	1	236604	uc007hrp.1	-22271	NA	Pisd-ps1	phosphatidylserine decarboxylase, pseudogene 1
chr11	3101521	3101620	100	101	3101590	42	6.59328	2.3009	3.6463	Adipose_R2_peak_	Distal Intergen	11	3124021	3131944	7924	1	236604	uc007hrp.1	-22401	NA	Pisd-ps1	phosphatidylserine decarboxylase, pseudogene 1
chr11	3102778	3102980	203	204	3102829	39	7.1266	2.48708	4.13112	Adipose_R2_peak_	Distal Intergen	11	3124021	3131944	7924	1	236604	uc007hrp.1	-21041	NA	Pisd-ps1	phosphatidylserine decarboxylase, pseudogene 1
chr11	3103927	3104063	137	138	3103987	101	32.10507	4.14896	28.19931	Adipose_R2_peak_	Distal Intergen	11	3124021	3131944	7924	1	236604	uc007hrp.1	-19958	NA	Pisd-ps1	phosphatidylserine decarboxylase, pseudogene 1
chr11	3103935	3104065	131	132	3103997	75	14.06112	2.71786	10.7367	Adipose_R1_peak_	Distal Intergen	11	3124021	3131944	7924	1	236604	uc007hrp.1	-19956	NA	Pisd-ps1	phosphatidylserine decarboxylase, pseudogene 1
chr11	3104547	3104754	208	209	3104605	42	12.51936	3.55177	9.15719	Adipose_R2_peak_	Distal Intergen	11	3124021	3131944	7924	1	236604	uc007hrp.1	-19267	NA	Pisd-ps1	phosphatidylserine decarboxylase, pseudogene 1
chr11	3104574	3104757	184	185	3104686	35	15.7706	5.03734	12.39067	Adipose_R1_peak_	Distal Intergen	11	3124021	3131944	7924	1	236604	uc007hrp.1	-19264	NA	Pisd-ps1	phosphatidylserine decarboxylase, pseudogene 1
chr11	3115451	3115554	104	105	3115504	30	7.24477	2.85875	4.21917	Adipose_R2_peak_	Distal Intergen	11	3124021	3131944	7924	1	236604	uc007hrp.1	-8467	NA	Pisd-ps1	phosphatidylserine decarboxylase, pseudogene 1
chr11	3118847	3118994	148	149	3118921	28	13.73721	5.2124	10.41463	Adipose_R1_peak_	Distal Intergen	11	3124021	3131944	7924	1	236604	uc007hrp.1	-5027	NA	Pisd-ps1	phosphatidylserine decarboxylase, pseudogene 1
chr11	3118855	3118993	139	140	3118923	38	11.72962	3.59649	8.4047	Adipose_R2_peak_	Distal Intergen	11	3124021	3131944	7924	1	236604	uc007hrp.1	-5028	NA	Pisd-ps1	phosphatidylserine decarboxylase, pseudogene 1
chr11	3122154	3122286	133	134	3122215	18	6.42934	3.41502	3.48999	Adipose_R1_peak_	Promoter (1-2	11	3124021	3131944	7924	1	236604	uc007hrp.1	-1735	NA	Pisd-ps1	phosphatidylserine decarboxylase, pseudogene 1
chr11	3122971	3123066	96	97	3123003	29	6.55855	2.71562	3.61288	Adipose_R2_peak_	Promoter (<=	11	3124021	3131944	7924	1	236604	uc007hrp.1	-955	NA	Pisd-ps1	phosphatidylserine decarboxylase, pseudogene 1
chr11	3125828	3125988	161	162	3125909	56	15.50545	3.48123	12.12701	Adipose_R1_peak_	Promoter (1-2	11	3124021	3131944	7924	1	236604	uc007hrp.1	1807	NA	Pisd-ps1	phosphatidylserine decarboxylase, pseudogene 1
chr11	3125845	3125978	134	135	3125921	50	8.28452	2.42324	5.18352	Adipose_R2_peak_	Promoter (1-2	11	3124021	3131944	7924	1	236604	uc007hrp.1	1824	NA	Pisd-ps1	phosphatidylserine decarboxylase, pseudogene 1
chr11	3126984	3127553	570	571	3127305	274	58.62506	3.1769	54.38387	Adipose_R2_peak_	Promoter (2-3	11	3124021	3131944	7924	1	236604	uc007hrp.1	2963	NA	Pisd-ps1	phosphatidylserine decarboxylase, pseudogene 1
chr11	3127034	3127177	144	145	3127085	84	5.86629	1.71657	2.98556	Adipose_R1_peak_	Intron (uc007h	11	3124021	3131944	7924	1	236604	uc007hrp.1	3013	NA	Pisd-ps1	phosphatidylserine decarboxylase, pseudogene 1
chr11	3127384	3127538	155	156	3127445	108	4.91591	1.52662	2.1731	Adipose_R1_peak_	Exon (uc007hr	11	3124021	3131944	7924	1	236604	uc007hrp.1	3363	NA	Pisd-ps1	phosphatidylserine decarboxylase, pseudogene 1
chr11	3127857	3128806	950	951	3128052	352	87.51447	3.57411	83.0611	Adipose_R2_peak_	Intron (uc007h	11	3124021	3131944	7924	1	236604	uc007hrp.1	3836	NA	Pisd-ps1	phosphatidylserine decarboxylase, pseudogene 1
chr11	3127929	3128755	827	828	3128254	174	13.06214	1.83257	9.76812	Adipose_R1_peak_	Intron (uc007h	11	3124021	3131944	7924	1	236604	uc007hrp.1	3908	NA	Pisd-ps1	phosphatidylserine decarboxylase, pseudogene 1
chr11	3129936	3130184	249	250	3130065	156	60.19966	5.16286	55.94165	Adipose_R2_peak_	Exon (uc007hr	11	3131887	3133886	2000	2	78887	uc007hrv.1	3702	ENSMUSG000	Sfi1	Sfi1 homolog, spindle assembly associated (yeast)
chr11	3130007	3130143	137	138	3130077	74	16.53518	3.05138	13.12949	Adipose_R1_peak_	Intron (uc007h	11	3131887	3133886	2000	2	78887	uc007hrv.1	3743	ENSMUSG000	Sfi1	Sfi1 homolog, spindle assembly associated (yeast)
chr11	3132046	3132601	556	557	3132217	250	65.14041	3.67359	60.83907	Adipose_R2_peak_	Promoter (1-2	11	3131887	3133886	2000	2	78887	uc007hrv.1	1285	ENSMUSG000	Sfi1	Sfi1 homolog, spindle assembly associated (yeast)
chr11	3132112	3132535	424	425	3132183	112	10.09337	1.92516	6.9294	Adipose_R1_peak_	Promoter (1-2	11	3131887	3133886	2000	2	78887	uc007hrv.1	1351	ENSMUSG000	Sfi1	Sfi1 homolog, spindle assembly associated (yeast)
chr11	3132935	3133061	127	128	3133015	63	6.99779	2.0204	4.00709	Adipose_R2_peak_	Promoter (<=	11	3131887	3133886	2000	2	78887	uc007hrv.1	825	ENSMUSG000	Sfi1	Sfi1 homolog, spindle assembly associated (yeast)
chr11	3133221	3133916	696	697	3133281	107	18.51524	2.65499	14.91303	Adipose_R2_peak_	Promoter (<=	11	3131887	3133886	2000	2	78887	uc007hrv.1	0	ENSMUSG000	Sfi1	Sfi1 homolog, spindle assembly associated (yeast)
chr11	3133509	3133898	390	391	3133647	152	15.74862	2.07384	12.36893	Adipose_R1_peak_	Promoter (<=	11	3131887	3133886	2000	2	78887	uc007hrv.1	0	ENSMUSG000	Sfi1	Sfi1 homolog, spindle assembly associated (yeast)
chr11	3135399	3135766	368	369	3135581	126	52.30171	5.50301	48.12296	Adipose_R2_peak_	Promoter (<=	11	3133886	3136017	2132	2	78887	uc007hrw.1	251	ENSMUSG000	Sfi1	Sfi1 homolog, spindle assembly associated (yeast)
chr11	3135410	3135638	229	230	3135504	71	10.34047	2.33526	7.17466	Adipose_R1_peak_	Promoter (<=	11	3133886	3136017	2132	2	78887	uc007hrw.1	379	ENSMUSG000	Sfi1	Sfi1 homolog, spindle assembly associated (yeast)
chr11	3136364	3136495	132	133	3136449	56	6.81281	2.08472	3.82989	Adipose_R2_peak_	Promoter (<=	11	3133886	3136017	2132	2	78887	uc007hrw.1	-347	ENSMUSG000	Sfi1	Sfi1 homolog, spindle assembly associated (yeast)
chr11	3136819	3136974	156	157	3136900	83	14.28286	2.59981	10.83583	Adipose_R2_peak_	Promoter (<=	11	3133886	3136017	2132	2	78887	uc007hrw.1	-802	ENSMUSG000	Sfi1	Sfi1 homolog, spindle assembly associated (yeast)
chr11	3137188	3137587	400	401	3137364	294	89.15706	4.19958	84.69674	Adipose_R2_peak_	Promoter (1-2	11	3133886	3136017	2132	2	78887	uc007hrw.1	-1171	ENSMUSG000	Sfi1	Sfi1 homolog, spindle assembly associated (yeast)
chr11	3137232	3137507	276	277	3137366	137	10.82124	1.84795	7.62255	Adipose_R1_peak_	Promoter (1-2	11	3133886	3136017	2132	2	78887	uc007hrw.1	-1215	ENSMUSG000	Sfi1	Sfi1 homolog, spindle assembly associated (yeast)
chr11	3138296	3138570	275	276	3138434	243	111.1918	6.39934	106.6429	Adipose_R2_peak_	Promoter (2-3	11	3133886	3136017	2132	2	78887	uc007hrw.1	-2279	ENSMUSG000	Sfi1	Sfi1 homolog, spindle assembly associated (yeast)
chr11	3138359	3138531	173	174	3138438	105	17.71384	2.61185	14.281	Adipose_R1_peak_	Promoter (2-3	11	3133886	3136017	2132	2	78887	uc007hrw.1	-2342	ENSMUSG000	Sfi1	Sfi1 homolog, spindle assembly associated (yeast)
chr11	3138999	3139260	262	263	3139126	168	47.40255	3.86011	43.27742	Adipose_R2_peak_	Promoter (2-3	11	3133886	3136017	2132	2	78887	uc007hrw.1	-2982	ENSMUSG000	Sfi1	Sfi1 homolog, spindle assembly associated (yeast)
chr11	3139054	3139215	162	163	3139133	104	19.9588	2.82698	16.45779	Adipose_R1_peak_	Intron (uc007h	11	3133886	3136017	2132	2	78887	uc007hrw.1	-3037	ENSMUSG000	Sfi1	Sfi1 homolog, spindle assembly associated (yeast)
chr11	3139907	3140049	143	144	3139981	84	12.46248	2.39067	9.10208	Adipose_R2_peak_	Intron (uc007h	11	3133886	3136017	2132	2	78887	uc007hrw.1	-3890	ENSMUSG000	Sfi1	Sfi1 homolog, spindle assembly associated (yeast)
chr11	3140674	3140948	275	276	3140811	267	95.22911	4.88269	90.74104	Adipose_R2_peak_	Intron (uc007h	11	3133886	3136017	2132	2	78887	uc007hrw.1	-4657	ENSMUSG000	Sfi1	Sfi1 homolog, spindle assembly associated (yeast)
chr11	3140703	3140905	203	204	3140808	126	17.26777	2.35077	13.83719	Adipose_R1_peak_	Intron (uc007h	11	3133886	3136017	2132	2	78887	uc007hrw.1	-4686	ENSMUSG000	Sfi1	Sfi1 homolog, spindle assembly associated (yeast)
chr11	3141246	3141912	667	668	3141553	389	78.35668	3.07388	73.95808	Adipose_R2_peak_	Intron (uc007h	11	3133886	3136017	2132	2	78887	uc007hrw.1	-5229	ENSMUSG000	Sfi1	Sfi1 homolog, spindle assembly associated (yeast)
chr11	3141290	3141854	565	566	3141495	173	13.34775	1.85069	10.05186	Adipose_R1_peak_	Intron (uc007h	11	3133886	3136017	2132	2	78887	uc007hrw.1	-5273	ENSMUSG000	Sfi1	Sfi1 homolog, spindle assembly associated (yeast)
chr11	3142088	3142850	763	764	3142440	361	55.653	2.60028	51.44104	Adipose_R2_peak_	Exon (uc007hr	11	3133886	3136017	2132	2	78887	uc007hrw.1	-6071	ENSMUSG000	Sfi1	Sfi1 homolog, spindle assembly associated (yeast)
chr11	3142249	3142797	549	550	3142367	162	9.14131	1.6599	6.01697	Adipose_R1_peak_	Exon (uc007hr	11	3133886	3136017	2132	2	78887	uc007hrw.1	-6232	ENSMUSG000	Sfi1	Sfi1 homolog, spindle assembly associated (yeast)
chr11	3143259	3143524	266	267	3143394	250	106.922	5.91842	102.389	Adipose_R2_peak_	Exon (uc007hr	11	3133886	3136017	2132	2	78887	uc007hrw.1	-7242	ENSMUSG000	Sfi1	Sfi1 homolog, spindle assembly associated (yeast)
chr11	3143316	3143498	183	184	3143404	113	17.71886	2.51447	14.28602	Adipose_R1_peak_	Exon (uc007hr	11	3133886	3136017	2132	2	78887	uc007hrw.1	-7299	ENSMUSG000	Sfi1	Sfi1 homolog, spindle assembly associated (yeast)
chr11	3145240	3145423	184	185	3145314	97	19.31369	2.88206	15.68733	Adipose_R2_peak_	Exon (uc007hr	11	3133886	3136017	2132	2	78887	uc007hrw.1	-9223	ENSMUSG000	Sfi1	Sfi1 homolog, spindle assembly associated (yeast)
chr11	3146116	3146628	513	514	3146462	355	93.23125	3.73409	88.75223	Adipose_R2_peak_	Exon (uc007hr	11	3133886	3136017	2132	2	78887	uc007hrw.1	-10099	ENSMUSG000	Sfi1	Sfi1 homolog, spindle assembly associated (yeast)
chr11	3146158	3146588	431	432	3146427	185	20.04123	2.13262	16.53966	Adipose_R1_peak_	Exon (uc007hr	11	3133886	3136017	2132	2	78887	uc007hrw.1	-10141	ENSMUSG000	Sfi1	Sfi1 homolog, spindle assembly associated (yeast)
chr11	3147667	3147845	179	180	3147760	95	41.79743	5.72774	37.74482	Adipose_R2_peak_	Intron (uc007h	11	3131850	3157936	26087	2	78887	uc007hrs.1	10091	ENSMUSG000	Sfi1	Sfi1 homolog, spindle assembly associated (yeast)
chr11	3148433	3148801	369	370	3148514	36	5.27779	2.17099	2.47999	Adipose_R2_peak_	Intron (uc007h	11	3131850	3157936	26087	2	78887	uc007hrs.1	9135	ENSMUSG000	Sfi1	Sfi1 homolog, spindle assembly associated (yeast)
chr11	3148618	3148807	190	191	3148720	103	39.15589	4.93383	35.30423	Adipose_R1_peak_	Intron (uc007h	11	3131850	3157936	26087	2	78887	uc007hrs.1	9129	ENSMUSG000	Sfi1	Sfi1 homolog, spindle assembly associated (yeast)
chr11	3150946	3151586	641	642	3151055	180	94.50318	7.48765	90.01902	Adipose_R2_peak_	Intron (uc007h	11	3131850	3157936	26087	2	78887	uc007hrs.1	6350	ENSMUSG000	Sfi1	Sfi1 homolog, spindle assembly associated (yeast)
chr11	3150993	3151127	135	136	3151057	87	16.52345	2.77313	13.11779	Adipose_R1_peak_	Intron (uc007h	11	3131850	3157936	26087	2	78887	uc007hrs.1	6809	ENSMUSG000	Sfi1	Sfi1 homolog, spindle assembly associated (yeast)
chr11	3153683	3154611	929	930	3153882	351	107.6137	4.26223	103.0778	Adipose_R2_peak_	Intron (uc007h	11	3131850	3157936	26087	2	78887	uc007hrs.1	3325	ENSMUSG000	Sfi1	Sfi1 homolog, spindle assembly associated (yeast)
chr11	3153752	3154332	581	582	3153887	153	19.2925	2.27436	15.81369	Adipose_R1_peak_	Intron (uc007h	11	3131850	3157936	26087	2	78887	uc007hrs.1	3604	ENSMUSG000	Sfi1	Sfi1 homolog, spindle assembly associated (yeast)
chr11	3154780	3155111	332	333	3155037	136	54.39196	5.31501	50.19154	Adipose_R2_peak_	Promoter (2-3	11	3131850	3157936	26087	2	78887	uc007hrs.1	2825	ENSMUSG000	Sfi1	Sfi1 homolog, spindle assembly associated (yeast)

chr11	3155505	3155693	189	190	3155617	86	27.78774	4.15356	23.95697	Adipose_R2_peak_	Promoter (2-3	11	3131850	3157936	26087	2	78887	uc007hrs.1	2243	ENSMUSG000	Sfi1	Sfi1 homolog, spindle assembly associated (yeast)
chr11	3156420	3156611	192	193	3156520	133	58.8581	5.93736	54.61522	Adipose_R2_peak_	Promoter (1-2	11	3131850	3157936	26087	2	78887	uc007hrs.1	1325	ENSMUSG000	Sfi1	Sfi1 homolog, spindle assembly associated (yeast)
chr11	3157010	3157661	652	653	3157171	269	94.27442	4.79923	89.79094	Adipose_R2_peak_	Promoter (<=1	11	3131850	3157936	26087	2	78887	uc007hrs.1	275	ENSMUSG000	Sfi1	Sfi1 homolog, spindle assembly associated (yeast)
chr11	3157056	3157397	342	343	3157177	133	21.15617	2.55795	17.63095	Adipose_R1_peak_	Promoter (<=1	11	3131850	3157936	26087	2	78887	uc007hrs.1	539	ENSMUSG000	Sfi1	Sfi1 homolog, spindle assembly associated (yeast)
chr11	3158088	3158239	152	153	3158151	68	9.8647	2.32164	6.66163	Adipose_R2_peak_	Promoter (<=1	11	3131850	3157936	26087	2	78887	uc007hrs.1	-152	ENSMUSG000	Sfi1	Sfi1 homolog, spindle assembly associated (yeast)
chr11	3158539	3158738	200	201	3158634	117	34.59983	3.93515	30.65186	Adipose_R2_peak_	Promoter (<=1	11	3131850	3157936	26087	2	78887	uc007hrs.1	-603	ENSMUSG000	Sfi1	Sfi1 homolog, spindle assembly associated (yeast)
chr11	3158565	3158724	160	161	3158645	63	11.62283	2.64813	8.39017	Adipose_R1_peak_	Promoter (<=1	11	3131850	3157936	26087	2	78887	uc007hrs.1	-629	ENSMUSG000	Sfi1	Sfi1 homolog, spindle assembly associated (yeast)
chr11	3159427	3159604	178	179	3159520	107	26.17367	3.3556	22.37414	Adipose_R2_peak_	Promoter (1-2	11	3131850	3157936	26087	2	78887	uc007hrs.1	-1491	ENSMUSG000	Sfi1	Sfi1 homolog, spindle assembly associated (yeast)
chr11	3159774	3160063	290	291	3159918	296	7.29472	4.02383	4.26752	Adipose_R2_peak_	Promoter (1-2	11	3131850	3157936	26087	2	78887	uc007hrs.1	-1838	ENSMUSG000	Sfi1	Sfi1 homolog, spindle assembly associated (yeast)
chr11	3159818	3160013	196	197	3159919	122	8.4372	1.75084	5.35329	Adipose_R1_peak_	Promoter (1-2	11	3131850	3157936	26087	2	78887	uc007hrs.1	-1882	ENSMUSG000	Sfi1	Sfi1 homolog, spindle assembly associated (yeast)
chr11	3160972	3161271	300	301	3161126	315	117.7695	5.15225	113.1972	Adipose_R2_peak_	Intron (uc007h	11	3131850	3157936	26087	2	78887	uc007hrs.1	-3036	ENSMUSG000	Sfi1	Sfi1 homolog, spindle assembly associated (yeast)
chr11	3161037	3161238	202	203	3161144	139	15.92303	2.1618	12.54237	Adipose_R1_peak_	Intron (uc007h	11	3131850	3157936	26087	2	78887	uc007hrs.1	-3101	ENSMUSG000	Sfi1	Sfi1 homolog, spindle assembly associated (yeast)
chr11	3161493	3162216	724	725	3161735	361	96.3821	3.78613	91.88908	Adipose_R2_peak_	Intron (uc007h	11	3131850	3157936	26087	2	78887	uc007hrs.1	-3557	ENSMUSG000	Sfi1	Sfi1 homolog, spindle assembly associated (yeast)
chr11	3161541	3162181	641	642	3161674	153	18.73608	2.2418	15.27911	Adipose_R1_peak_	Intron (uc007h	11	3131850	3157936	26087	2	78887	uc007hrs.1	-3605	ENSMUSG000	Sfi1	Sfi1 homolog, spindle assembly associated (yeast)
chr11	3162582	3162888	307	308	3162724	282	138.2221	7.01308	133.5963	Adipose_R2_peak_	Intron (uc007h	11	3131850	3157936	26087	2	78887	uc007hrs.1	-4646	ENSMUSG000	Sfi1	Sfi1 homolog, spindle assembly associated (yeast)
chr11	3162633	3162837	205	206	3162729	133	32.31389	3.38377	28.5737	Adipose_R1_peak_	Intron (uc007h	11	3131850	3157936	26087	2	78887	uc007hrs.1	-4697	ENSMUSG000	Sfi1	Sfi1 homolog, spindle assembly associated (yeast)
chr11	3163255	3163358	104	105	3163306	51	5.35734	1.92971	2.52307	Adipose_R2_peak_	Intron (uc007h	11	3131850	3157936	26087	2	78887	uc007hrs.1	-5319	ENSMUSG000	Sfi1	Sfi1 homolog, spindle assembly associated (yeast)
chr11	3164720	3164971	252	253	3164841	168	47.57909	3.87224	43.45282	Adipose_R2_peak_	Intron (uc007h	11	3131850	3157936	26087	2	78887	uc007hrs.1	-6784	ENSMUSG000	Sfi1	Sfi1 homolog, spindle assembly associated (yeast)
chr11	3165259	3165398	140	141	3165324	72	10.94355	2.39308	7.67266	Adipose_R2_peak_	Intron (uc007h	11	3131850	3157936	26087	2	78887	uc007hrs.1	-7323	ENSMUSG000	Sfi1	Sfi1 homolog, spindle assembly associated (yeast)
chr11	3165513	3165724	212	213	3165619	168	76.67455	6.25491	72.28603	Adipose_R2_peak_	5' UTR	11	3131850	3157936	26087	2	78887	uc007hrs.1	-7577	ENSMUSG000	Sfi1	Sfi1 homolog, spindle assembly associated (yeast)
chr11	3166059	3166265	207	208	3166211	109	35.77486	4.29706	31.80429	Adipose_R2_peak_	Intron (uc007h	11	3131850	3157936	26087	2	78887	uc007hrs.1	-8123	ENSMUSG000	Sfi1	Sfi1 homolog, spindle assembly associated (yeast)
chr11	3166412	3166606	195	196	3166506	127	49.95797	5.19697	45.80711	Adipose_R2_peak_	Intron (uc007h	11	3131850	3157936	26087	2	78887	uc007hrs.1	-8476	ENSMUSG000	Sfi1	Sfi1 homolog, spindle assembly associated (yeast)
chr11	3167129	3167719	591	592	3167181	89	25.22654	3.71903	21.44561	Adipose_R2_peak_	Intron (uc007h	11	3131850	3157936	26087	2	78887	uc007hrs.1	-9193	ENSMUSG000	Sfi1	Sfi1 homolog, spindle assembly associated (yeast)
chr11	3168036	3168215	180	181	3168113	73	18.3688	3.32431	14.76904	Adipose_R2_peak_	Intron (uc007h	11	3131850	3157936	26087	2	78887	uc007hrs.1	-10100	ENSMUSG000	Sfi1	Sfi1 homolog, spindle assembly associated (yeast)
chr11	3168485	3168585	101	102	3168547	57	9.92991	2.53162	6.72448	Adipose_R2_peak_	Intron (uc007h	11	3131850	3157936	26087	2	78887	uc007hrs.1	-10549	ENSMUSG000	Sfi1	Sfi1 homolog, spindle assembly associated (yeast)
chr11	3168816	3169287	472	473	3169119	205	75.93245	5.01964	71.54903	Adipose_R2_peak_	Intron (uc007h	11	3131850	3157936	26087	2	78887	uc007hrs.1	-10880	ENSMUSG000	Sfi1	Sfi1 homolog, spindle assembly associated (yeast)
chr11	3168881	3169285	405	406	3169022	76	10.21969	2.252	7.05482	Adipose_R1_peak_	Intron (uc007h	11	3131850	3157936	26087	2	78887	uc007hrs.1	-10945	ENSMUSG000	Sfi1	Sfi1 homolog, spindle assembly associated (yeast)
chr11	3169467	3169946	480	481	3169594	226	76.70343	4.62051	72.31472	Adipose_R2_peak_	Intron (uc007h	11	3131850	3157936	26087	2	78887	uc007hrs.1	-11531	ENSMUSG000	Sfi1	Sfi1 homolog, spindle assembly associated (yeast)
chr11	3169515	3169946	432	433	3169597	115	17.37369	2.46501	13.94271	Adipose_R1_peak_	Intron (uc007h	11	3131850	3157936	26087	2	78887	uc007hrs.1	-11579	ENSMUSG000	Sfi1	Sfi1 homolog, spindle assembly associated (yeast)
chr11	3171398	3171611	214	215	3171497	135	64.96152	6.56477	60.66124	Adipose_R2_peak_	Intron (uc007h	11	3131850	3157936	26087	2	78887	uc007hrs.1	-13462	ENSMUSG000	Sfi1	Sfi1 homolog, spindle assembly associated (yeast)
chr11	3171421	3171555	135	136	3171487	76	17.21705	3.08854	13.78695	Adipose_R1_peak_	Intron (uc007h	11	3131850	3157936	26087	2	78887	uc007hrs.1	-13485	ENSMUSG000	Sfi1	Sfi1 homolog, spindle assembly associated (yeast)
chr11	3172079	3172295	217	218	3172178	180	76.78374	5.80706	72.39474	Adipose_R2_peak_	Intron (uc007h	11	3131850	3157936	26087	2	78887	uc007hrs.1	-14143	ENSMUSG000	Sfi1	Sfi1 homolog, spindle assembly associated (yeast)
chr11	3172101	3172262	162	163	3172169	98	25.89772	3.54037	22.26865	Adipose_R1_peak_	Intron (uc007h	11	3131850	3157936	26087	2	78887	uc007hrs.1	-14165	ENSMUSG000	Sfi1	Sfi1 homolog, spindle assembly associated (yeast)
chr11	3172446	3173144	699	700	3172586	259	100.7629	5.3369	96.25179	Adipose_R2_peak_	Intron (uc007h	11	3131850	3157936	26087	2	78887	uc007hrs.1	-14510	ENSMUSG000	Sfi1	Sfi1 homolog, spindle assembly associated (yeast)
chr11	3172484	3172860	377	378	3172589	128	38.12514	3.98286	34.28862	Adipose_R1_peak_	Intron (uc007h	11	3131850	3157936	26087	2	78887	uc007hrs.1	-14548	ENSMUSG000	Sfi1	Sfi1 homolog, spindle assembly associated (yeast)
chr11	3173484	3173706	223	224	3173590	137	59.53635	5.828	55.28603	Adipose_R2_peak_	Intron (uc007h	11	3131850	3157936	26087	2	78887	uc007hrs.1	-15548	ENSMUSG000	Sfi1	Sfi1 homolog, spindle assembly associated (yeast)
chr11	3173518	3173662	145	146	3173588	83	20.40616	3.30415	16.90293	Adipose_R1_peak_	Intron (uc007h	11	3131850	3157936	26087	2	78887	uc007hrs.1	-15582	ENSMUSG000	Sfi1	Sfi1 homolog, spindle assembly associated (yeast)
chr11	3174044	3174138	95	96	3174089	39	5.66537	2.18483	2.81955	Adipose_R2_peak_	Intron (uc007h	11	3131850	3157936	26087	2	78887	uc007hrs.1	-16108	ENSMUSG000	Sfi1	Sfi1 homolog, spindle assembly associated (yeast)
chr11	3175189	3175395	207	208	3175288	124	50.51118	5.38485	46.35275	Adipose_R2_peak_	Intron (uc007h	11	3131850	3157936	26087	2	78887	uc007hrs.1	-17253	ENSMUSG000	Sfi1	Sfi1 homolog, spindle assembly associated (yeast)
chr11	3175563	3175714	152	153	3175627	49	10.4987	2.83743	7.25809	Adipose_R2_peak_	Intron (uc007h	11	3131850	3157936	26087	2	78887	uc007hrs.1	-17627	ENSMUSG000	Sfi1	Sfi1 homolog, spindle assembly associated (yeast)
chr11	3176510	3176938	429	430	3176635	154	56.14488	4.86629	51.92609	Adipose_R2_peak_	Intron (uc007h	11	3131850	3193463	61614	2	78887	uc007hrt.1	16525	ENSMUSG000	Sfi1	Sfi1 homolog, spindle assembly associated (yeast)
chr11	3176548	3176903	356	357	3176663	82	10.97238	2.25959	7.77253	Adipose_R1_peak_	Intron (uc007h	11	3131850	3193463	61614	2	78887	uc007hrt.1	16560	ENSMUSG000	Sfi1	Sfi1 homolog, spindle assembly associated (yeast)
chr11	3177473	3177588	116	117	3177540	39	6.90088	2.43965	3.91429	Adipose_R2_peak_	5' UTR	11	3131850	3193463	61614	2	78887	uc007hrt.1	15875	ENSMUSG000	Sfi1	Sfi1 homolog, spindle assembly associated (yeast)
chr11	3179781	3180109	329	330	3179958	261	135.6632	7.54363	131.0434	Adipose_R2_peak_	Intron (uc007h	11	3131850	3193463	61614	2	78887	uc007hrt.1	13354	ENSMUSG000	Sfi1	Sfi1 homolog, spindle assembly associated (yeast)
chr11	3179846	3180090	245	246	3179962	117	41.01064	4.59708	37.13109	Adipose_R1_peak_	Intron (uc007h	11	3131850	3193463	61614	2	78887	uc007hrt.1	13373	ENSMUSG000	Sfi1	Sfi1 homolog, spindle assembly associated (yeast)
chr11	3180928	3181091	164	165	3181003	71	11.79797	2.51426	8.47076	Adipose_R2_peak_	Intron (uc007h	11	3131850	3193463	61614	2	78887	uc007hrt.1	12372	ENSMUSG000	Sfi1	Sfi1 homolog, spindle assembly associated (yeast)
chr11	3181203	3181571	369	370	3181465	162	62.37802	5.16247	58.10073	Adipose_R2_peak_	Intron (uc007h	11	3131850	3193463	61614	2	78887	uc007hrt.1	11892	ENSMUSG000	Sfi1	Sfi1 homolog, spindle assembly associated (yeast)
chr11	3181384	3181557	174	175	3181464	107	39.22329	4.77114	35.37125	Adipose_R1_peak_	Intron (uc007h	11	3131850	3193463	61614	2	78887	uc007hrt.1	11906	ENSMUSG000	Sfi1	Sfi1 homolog, spindle assembly associated (yeast)
chr11	3184305	3190751	6447	6448	3184488	296	53.03196	2.83029	48.8443	Adipose_R2_peak_	Promoter (2-3	11	3131850	3193463	61614	2	78887	uc007hrt.1	2712	ENSMUSG000	Sfi1	Sfi1 homolog, spindle assembly associated (yeast)
chr11	3184370	3189428	5059	5060	3184502	129	9.59693	1.80213	6.46855	Adipose_R1_peak_	5' UTR	11	3131850	3193463	61614	2	78887	uc007hrt.1	4035	ENSMUSG000	Sfi1	Sfi1 homolog, spindle assembly associated (yeast)
chr11	3189572	3191597	2026	2027	3189667	139	15.41405	2.12946	12.03599	Adipose_R1_peak_	Promoter (1-2	11	3131850	3193463	61614	2	78887	uc007hrt.1	1866	ENSMUSG000	Sfi1	Sfi1 homolog, spindle assembly associated (yeast)
chr11	3190855	3195381	4527	4528	3190941	189	17.93777	2.0148	14.35715	Adipose_R2_peak_	Promoter (<=1	11	3131850	3193463	61614	2	78887	uc007hrt.1	0	ENSMUSG000	Sfi1	Sfi1 homolog, spindle assembly associated (yeast)
chr11	3191886	3193370	1485	1486	3192023	111	9.94737	1.91981	6.7846	Adipose_R1_peak_	Promoter (<=1	11	3131850	3193463	61614	2	78887	uc007hrt.1	93	ENSMUSG000	Sfi1	Sfi1 homolog, spindle assembly associated (yeast)
chr11	3193535	3195358	1824	1825	3193802	149	17.29097	2.18205	13.86031	Adipose_R1_peak_	Promoter (<=1	11	3131850	3193463	61614	2	78887	uc007hrt.1	-72	ENSMUSG000	Sfi1	Sfi1 homolog, spindle assembly associated (yeast)
chr11	3195496	3197119	1624	1625	3195699	312	67.38693	3.20943	63.06729	Adipose_R2_peak_	Promoter (2-3	11	3131850	3193463	61614	2	78887	uc007hrt.1	-2033	ENSMUSG000	Sfi1	Sfi1 homolog, spindle assembly associated (yeast)
chr11	3195560	3197097	1538	1539	3195678	138	17.54696	2.27291	14.115	Adipose_R1_peak_	Promoter (2-3	11	3131850	3193463	61614	2	78887	uc007hrt.1	-2097	ENSMUSG000	Sfi1	Sfi1 homolog, spindle assembly associated (yeast)
chr11	3197302	3198543	1242	1243	3197419	97	14.02469	2.3814	10.70051	Adipose_R1_peak_	Distal Intergen	11	3202353	3244588	42236	1	74203	uc007hsc.2	-3810	ENSMUSG000	Eif4enif1	eukaryotic translation initiation factor 4E nuclear import factor 1
chr11	3197319	3198621	1303	1304	3197443	168	25.13483	2.49794	21.35518	Adipose_R2_peak_	Distal Intergen	11	3202353	3244588	42236	1	74203	uc007hsc.2	-3732	ENSMUSG000	Eif4enif1	eukaryotic translation initiation factor 4E nuclear import factor 1
chr11	3198759	3200061	1303	1304	3198963	205	26.87698	2.34634	23.0629	Adipose_R2_peak_	Promoter (2-3	11	3202353	3244588	42236	1	74203	uc007hsc.2	-2292	ENSMUSG000	Eif4enif1	eukaryotic translation initiation factor 4E nuclear import factor 1
chr11	3198841	3199120	280	281	3198993	125	17.9018	2.40523	14.46812	Adipose_R1_peak_	Distal Intergen	11	3202353	3244588	42236	1	74203	uc007hsc.2	-3233	ENSMUSG000	Eif4enif1	eukaryotic translation initiation factor 4E nuclear import factor 1
chr11	3199500	3200029	530	531	3199685	113	12.91301	2.13606	9.63827	Adipose_R1_peak_	Promoter (2-3	11	3202353	3244588	42236	1	74203	uc007hsc.2	-2324	ENSMUSG000	Eif4enif1	eukaryotic translation initiation factor 4E nuclear import factor 1
chr11	3217293	3217446	154	155	3217363	35	19.76029	6.47056	16.26028	Adipose_R1_peak_	Intron (uc007h	11	3224632	3230186	5555	1	74203	uc007hsg.1	-7186	ENSMUSG000	Eif4enif1	eukaryotic translation initiation factor 4E nuclear import factor 1
chr11	3217294	3217445	152	153	3217355	33	8.83596	3.1354	5.68652	Adipose_R2_peak_	Intron (uc007h	11	3224632	3230186	5555	1	74203	uc007hsg.1	-7187	ENSMUSG000	Eif4enif1	eukaryotic translation initiation factor 4E nuclear import factor 1
chr11	3275308	3275564	257	258	3275458	31	16.23981	5.75161	12.83549	Adipose_R1_peak_	Intron (uc029r	11	3249922	3266386	16465	2	13494	uc007hsh.1	-8922	ENSMUSG000	Drg1	developmentally regulated GTP binding protein 1
chr11	3275340	3275544	205	206	3275464	36	10.5378	3.41206	7.27949	Adipose_R2_peak_	Intron (uc029r	11	3249922	3266386	16465	2	13494	uc007hsh.1	-8954	ENSMUSG000	Drg1	developmentally regulated GTP binding protein 1
chr11	4664620	4664873	254	255	4664745	47	31.38649	8.62742	27.65048	Adipose_R1_peak_	Intron (uc007h	11	4637793	4683386	45594	1	75452	uc007hva.2	26827	ENSMUSG000	Ascc2	activating signal cointegrator 1 complex subunit 2
chr11	4664689	4664811	123	124	4664743	32	8.29282	3.04319	5.18352	Adipose_R2_peak_	Intron (uc007h	11	4637793	4683386	45594	1	75452	uc007hva.2	26896	ENSMUSG000	Ascc2	activating signal cointegrator 1 complex subunit 2
chr11	5168524	5168836	313	314	5168709	46	16.86262	4.3228	13.32444	Adipose_R2_peak_	Distal Intergen	11	5106266	5152222	45957	2	140703	uc007hwg.1	-16302	ENSMUSG000	Emid1	EMI domain containing 1
chr11	5168548	5168774	227	228	5168695	24	9.44711	4.02999	6.32006	Adipose_R1_peak_	Distal Intergen	11	5106266	5152222	45957	2	140703	uc007hwg.1	-16326	ENSMUSG000	Emid1	EMI domain containing 1
chr11	5169273	5169572	300	301	5169326	30	6.96253	2.78106	3.97376	Adipose_R2_peak_	Distal Intergen	11	5106266	5152222	45957	2	140703	uc007hwg.1	-17051	ENSMUSG000	Emid1	EMI domain containing 1
chr11	5169387	5169648	262	263	5169511	25	8.43029	3.55651	5.34896	Adipose_R1_peak_	Distal Intergen	11	5106266	5152222	45957	2	140703	uc007hwg.1	-17165	ENSMUSG000	Emid1	EMI domain containing 1
chr11	6382856	6383187	332	333	6382973	22	9.13959	4.13397	6.01526	Adipose_R1_peak_	Distal Intergen	11	6395125	6406162	11038	1	52915	uc007hym.1	-11938	ENSMUSG000	Zmiz2	zinc finger, MIZ-type containing 2
chr11	6383045	6383224	180	181	6383140	29	6.74062	2.76653	3.75989	Adipose_R2_peak_	Distal Intergen	11	6395125	6406162	11038	1	52915	uc007hym.1	-11901	ENSMUSG000	Zmiz2	zinc finger, MIZ-type containing 2
chr11	6385650	6385881	232	233	6385775	26	12.14079	4.85292	8.87838	Adipose_R1_peak_	Distal Intergen	11	6395125	6406162	11038	1	52915	uc007hym.1	-9244	ENSMUSG000	Zmiz2	zinc finger, MIZ-type containing 2
chr11	7582307	7582501	195	196	7582428	30	15.39182	5.57188	12.01394	Adipose_R1_peak_	Distal Intergen	11	7206091	7213923	7833	2	16009	uc007hzi.2	-368384	ENSMUSG000	Igfbp3	insulin-like growth factor binding protein 3
chr11	7582388	7582496	109	110	7582467	28	6.25023	2.67431	3.31626	Adipose_R2_peak_	Distal Intergen	11	7206091	7213923	7833	2	16009	uc007hzi.2	-368465	ENSMUSG000	Igfbp3	insulin-like growth factor binding protein 3
chr11	7618032	7618306	275	276	7618164	69	55.87963	12.58165	51.83661	Adipose_R1_peak_	Distal Intergen	11	7206091	7213923	7833	2	16009	uc007hzi.2	-404109	ENSMUSG000	Igfbp3	insulin-like growth factor binding protein 3
chr11	7618042	7618295	254	255	7618167	60	27.32259	5.62528	23.49644	Adipose_R2_peak_	Distal Intergen	11	7206091	7213923	7833	2	16009	uc007hzi.2	-404119	ENSMUSG000	Igfbp3	insulin-like growth factor binding protein 3
chr11	7753702	7753801	100	101	7753755	30	7.24477	2.85875	4.21917	Adipose_R2_peak_	Distal Intergen	11	7206091	7213923	7833	2	16009	uc007hzi.2	-539779	ENSMUSG000	Igfbp3	insulin-like growth factor binding protein 3
chr11	7754496	7754601	106	107	7754568	30	7.24477	2.85875	4.21917	Adipose_R2_peak_	Distal Intergen	11	7206091	7213923	7833	2	16009	uc007hzi.2	-540573	ENSMUSG000	Igfbp3	insulin-like growth factor binding protein 3
chr11	7874256	7874535	280	281	7874384	66	32.22715	6.17859	28.31407	Adipose_R2_peak_	Distal Intergen	11	8431652	8468503	36852	2	319939	uc007hzj.1	593968	ENSMUSG000	Tns3	tensin 3
chr11	7874276	7874558	283	284	7874404	59	44.30963	10.78427	40.38949	Adipose_R1_peak_	Distal Intergen	11	8431652	8468503	36852	2	319939	uc007hzj.1	593945	ENSMUSG000	Tns3	tensin 3
chr11	7874905	7875053	149	150	7874993	21	7.86515	3.71791	4.82354	Adipose_R1_peak_	Distal Intergen	11	8431652	8468503	36852	2	319939	uc007hzj.1	593450	ENSMUSG000	Tns3	tensin 3
chr11	8671304	8671501	198	199	8671367	18	6.42934	3.41502	3.48999	Adipose_R1_peak_	Distal Intergen	11	8431652	8664535	232884	2	319939	uc007hzk.1	-6769	ENSMUSG000	Tns3	tensin 3
chr11	9086391	9086638	248	249	9086508	44	28.34544	8.08821	24.66239	Adipose_R1_peak_	Distal Intergen	11	9118008	9136170	18163	1	22271	uc007hzx.2	-31370	ENSMUSG000	Upp1	uridine phosphorylase 1
chr11	9086442	9086608	167	168	9086502	43	14.8939	4.05758	11.42017	Adipose_R2_peak_	Distal Intergen	11	9118008	9136170	18163	1	22271	uc007hzx.2	-31400	ENSMUSG000	Upp1	uridine phosphorylase 1
chr11	9103536	9103816	281	282	9103696	56	40.97333	10.24506	37.09402	Adipose_R1_peak_	Distal Intergen	11	9118008	9136170	18163	1	22271	uc007hzx.2	-14192	ENSMUSG000	Upp1	uridine phosphorylase 1
chr11	9103551	9103795	245	246	9103687	48	18.29934	4.51867	14.70076	Adipose_R2_peak_	Distal Intergen	11	9118008	9136170	18163	1	22271	uc007hzx.2	-14213	ENSMUSG000	Upp1	uridine phosphorylase 1
chr11	9245224	9245332	109	110	9245290	30	7.24477	2.85875	4.21917	Adipose_R2_peak_	Intron (uc007i	11	9191942	9224540	32599	1	268379	uc007hzz.1	53282	ENSMUSG000	Abca13	ATP-binding cassette, sub-family A (ABC1), member 13
chr11	9361534	9361863	330	331	9361683	116	103.6213	16.56128	99.27169	Adipose_R1_peak_	Intron (uc007i	11	9191942	9224540	32599	1	268379	uc007hzz.1	169592	ENSMUSG000	Abca13	ATP-binding cassette, sub-family A (ABC1), member 13
chr11	9361556	9361821	266	267	9361683	75	40.01057	7.00855	35.97731	Adipose_R2_peak_	Intron (uc007i	11	9191942	9224540	32599	1	268379	uc007hzz.1	169614	ENSMUSG000	Abca13	ATP-binding cassette, sub-family A (ABC1), member 13
chr11	9422153	9422253	101	102	9422197	26	5.31252	2.48988	2.47999	Adipose_R2_peak_	Intron (uc007i	11	9191942	9224540	32599	1	268379	uc007hzz.1	230211	ENSMUSG000	Abca13	ATP-binding cassette, sub-family A (ABC1), member 13
chr11	9825088	9825220	133	134	9825133	26	5.31252	2.48988	2.47999	Adipose_R2_peak_	Distal Intergen	11	9191942	9224540	32599	1	268379	uc007hzz.1	633146	ENSMUSG000	Abca13	ATP-binding cassette, sub-family A (ABC1), member 13
chr11	9946561	9946720	160	161	9946692	33	8.83596	3.1354	5.68652	Adipose_R2_peak_	Distal Intergen	11	9191942	9224540	32599	1	268379	uc007hzz.1	754619	ENSMUSG000	Abca13	ATP-binding cassette, sub-family A (ABC1), member 13

chr11	10093423	10093683	261	262	10093545	42	26.36523	7.72873	22.71906	Adipose_R1_peak_	Distal Intergen	11	9191942	9224540	32599	1	268379	uc007hzz.1	901481	ENSMUSG000	Abca13	ATP-binding cassette, sub-family A (ABC1), member 13
chr11	10093437	10093593	157	158	10093544	30	7.24477	2.85875	4.21917	Adipose_R2_peak_	Distal Intergen	11	9191942	9224540	32599	1	268379	uc007hzz.1	901495	ENSMUSG000	Abca13	ATP-binding cassette, sub-family A (ABC1), member 13
chr11	10251374	10251604	231	232	10251507	35	19.76029	6.47056	16.26028	Adipose_R1_peak_	Distal Intergen	11	11114016	11263526	149511	1	319922	uc007iac.1	-862412	ENSMUSG000	Vwc2	von Willebrand factor C domain containing 2
chr11	10251422	10251526	105	106	10251467	26	5.31252	2.48988	2.47999	Adipose_R2_peak_	Distal Intergen	11	11114016	11263526	149511	1	319922	uc007iac.1	-862490	ENSMUSG000	Vwc2	von Willebrand factor C domain containing 2
chr11	10255369	10255571	203	204	10255454	30	15.39182	5.57188	12.01394	Adipose_R1_peak_	Distal Intergen	11	11114016	11263526	149511	1	319922	uc007iac.1	-858445	ENSMUSG000	Vwc2	von Willebrand factor C domain containing 2
chr11	10255376	10255515	140	141	10255444	30	7.24477	2.85875	4.21917	Adipose_R2_peak_	Distal Intergen	11	11114016	11263526	149511	1	319922	uc007iac.1	-858501	ENSMUSG000	Vwc2	von Willebrand factor C domain containing 2
chr11	10669799	10670203	405	406	10669958	288	334.3947	33.204	329.61	Adipose_R1_peak_	Distal Intergen	11	11114016	11263526	149511	1	319922	uc007iac.1	-443813	ENSMUSG000	Vwc2	von Willebrand factor C domain containing 2
chr11	10669804	10670152	349	350	10669960	176	145.928	15.31384	141.2831	Adipose_R2_peak_	Distal Intergen	11	11114016	11263526	149511	1	319922	uc007iac.1	-443864	ENSMUSG000	Vwc2	von Willebrand factor C domain containing 2
chr11	10726583	10726764	182	183	10726633	17	5.80341	3.23528	2.92378	Adipose_R1_peak_	Distal Intergen	11	11114016	11263526	149511	1	319922	uc007iac.1	-387252	ENSMUSG000	Vwc2	von Willebrand factor C domain containing 2
chr11	10827991	10828257	267	268	10828117	45	29.34988	8.26794	25.64927	Adipose_R1_peak_	Distal Intergen	11	11114016	11263526	149511	1	319922	uc007iac.1	-285759	ENSMUSG000	Vwc2	von Willebrand factor C domain containing 2
chr11	10828046	10828240	195	196	10828143	39	12.34187	3.68871	8.98501	Adipose_R2_peak_	Distal Intergen	11	11114016	11263526	149511	1	319922	uc007iac.1	-285776	ENSMUSG000	Vwc2	von Willebrand factor C domain containing 2
chr11	10939485	10939644	160	161	10939568	23	9.86443	4.31371	6.70247	Adipose_R1_peak_	Distal Intergen	11	11114016	11263526	149511	1	319922	uc007iac.1	-174372	ENSMUSG000	Vwc2	von Willebrand factor C domain containing 2
chr11	11288522	11288884	363	364	11288688	121	91.27221	12.38435	86.9832	Adipose_R1_peak_	Intron (uc007i	11	11280040	11462419	182380	2	53604	uc007iae.2	173535	ENSMUSG000	Zpbp	zona pellucida binding protein
chr11	11288524	11288870	347	348	11288704	101	63.16329	9.0394	58.87888	Adipose_R2_peak_	Intron (uc007i	11	11280040	11462419	182380	2	53604	uc007iae.2	173549	ENSMUSG000	Zpbp	zona pellucida binding protein
chr11	11382801	11382931	131	132	11382862	24	10.60668	4.49345	7.40961	Adipose_R1_peak_	Intron (uc007i	11	11280040	11462419	182380	2	53604	uc007iae.2	79488	ENSMUSG000	Zpbp	zona pellucida binding protein
chr11	12230690	12230837	148	149	12230826	30	7.24477	2.85875	4.21917	Adipose_R2_peak_	Distal Intergen	11	12236676	12412140	175465	2	12808	uc033ftw.1	181303	ENSMUSG000	Cobl	cordon-bleu WH2 repeat
chr11	12330224	12330372	149	150	12330287	18	6.42934	3.41502	3.48999	Adipose_R1_peak_	Intron (uc007il	11	12236676	12412140	175465	2	12808	uc033ftw.1	81768	ENSMUSG000	Cobl	cordon-bleu WH2 repeat
chr11	12566830	12567065	236	237	12566947	33	17.97508	6.11109	14.52164	Adipose_R1_peak_	Distal Intergen	11	12236676	12464960	228285	2	12808	uc007ibd.2	-101870	ENSMUSG000	Cobl	cordon-bleu WH2 repeat
chr11	12750908	12751047	140	141	12750978	22	9.13959	4.13397	6.01526	Adipose_R1_peak_	Distal Intergen	11	12236676	12464960	228285	2	12808	uc007ibd.2	-285948	ENSMUSG000	Cobl	cordon-bleu WH2 repeat
chr11	12751352	12751544	193	194	12751455	26	12.14079	4.85292	8.87838	Adipose_R1_peak_	Distal Intergen	11	12236676	12464960	228285	2	12808	uc007ibd.2	-286392	ENSMUSG000	Cobl	cordon-bleu WH2 repeat
chr11	12756550	12756705	156	157	12756614	17	5.80341	3.23528	2.92378	Adipose_R1_peak_	Distal Intergen	11	12236676	12464960	228285	2	12808	uc007ibd.2	-291590	ENSMUSG000	Cobl	cordon-bleu WH2 repeat
chr11	12791328	12791614	287	288	12791458	57	42.07807	10.4248	38.18469	Adipose_R1_peak_	Distal Intergen	11	12236676	12464960	228285	2	12808	uc007ibd.2	-326368	ENSMUSG000	Cobl	cordon-bleu WH2 repeat
chr11	12791334	12791547	214	215	12791446	54	22.67101	5.07198	18.94758	Adipose_R2_peak_	Distal Intergen	11	12236676	12464960	228285	2	12808	uc007ibd.2	-326374	ENSMUSG000	Cobl	cordon-bleu WH2 repeat
chr11	12857488	12857688	201	202	12857555	29	6.74062	2.76653	3.75989	Adipose_R2_peak_	Distal Intergen	11	12236676	12464960	228285	2	12808	uc007ibd.2	-392528	ENSMUSG000	Cobl	cordon-bleu WH2 repeat
chr11	12858355	12858640	286	287	12858514	31	7.7623	2.95097	4.69388	Adipose_R2_peak_	Distal Intergen	11	12236676	12464960	228285	2	12808	uc007ibd.2	-393395	ENSMUSG000	Cobl	cordon-bleu WH2 repeat
chr11	12861153	12861410	258	259	12861272	32	8.29282	3.04319	5.18352	Adipose_R2_peak_	Distal Intergen	11	12236676	12464960	228285	2	12808	uc007ibd.2	-396193	ENSMUSG000	Cobl	cordon-bleu WH2 repeat
chr11	12862279	12862437	159	160	12862336	31	7.7623	2.95097	4.69388	Adipose_R2_peak_	Distal Intergen	11	12236676	12464960	228285	2	12808	uc007ibd.2	-397319	ENSMUSG000	Cobl	cordon-bleu WH2 repeat
chr11	12897991	12898299	309	310	12898160	51	35.56531	9.34637	31.76235	Adipose_R1_peak_	Distal Intergen	11	12236676	12464960	228285	2	12808	uc007ibd.2	-433031	ENSMUSG000	Cobl	cordon-bleu WH2 repeat
chr11	12898065	12898255	191	192	12898160	51	20.44827	4.79532	16.78434	Adipose_R2_peak_	Distal Intergen	11	12236676	12464960	228285	2	12808	uc007ibd.2	-433105	ENSMUSG000	Cobl	cordon-bleu WH2 repeat
chr11	12898803	12899095	293	294	12898952	86	74.25419	14.70265	70.06258	Adipose_R1_peak_	Distal Intergen	11	12236676	12464960	228285	2	12808	uc007ibd.2	-433843	ENSMUSG000	Cobl	cordon-bleu WH2 repeat
chr11	12898852	12899057	206	207	12898963	46	16.90986	4.33423	13.35898	Adipose_R2_peak_	Distal Intergen	11	12236676	12464960	228285	2	12808	uc007ibd.2	-433892	ENSMUSG000	Cobl	cordon-bleu WH2 repeat
chr11	12902783	12902952	170	171	12902913	23	9.86443	4.31371	6.70247	Adipose_R1_peak_	Distal Intergen	11	12236676	12464960	228285	2	12808	uc007ibd.2	-437823	ENSMUSG000	Cobl	cordon-bleu WH2 repeat
chr11	12904727	12905016	290	291	12904860	60	40.93132	9.39765	37.06203	Adipose_R1_peak_	Distal Intergen	11	12236676	12464960	228285	2	12808	uc007ibd.2	-439767	ENSMUSG000	Cobl	cordon-bleu WH2 repeat
chr11	12904735	12904994	260	261	12904861	58	25.74274	5.44085	21.94821	Adipose_R2_peak_	Distal Intergen	11	12236676	12464960	228285	2	12808	uc007ibd.2	-439775	ENSMUSG000	Cobl	cordon-bleu WH2 repeat
chr11	13289652	13289862	211	212	13289768	32	17.10103	5.93135	13.67137	Adipose_R1_peak_	Distal Intergen	11	12236676	12464960	228285	2	12808	uc007ibd.2	-824692	ENSMUSG000	Cobl	cordon-bleu WH2 repeat
chr11	13289663	13289874	212	213	13289769	40	12.96465	3.78093	9.57654	Adipose_R2_peak_	Distal Intergen	11	12236676	12464960	228285	2	12808	uc007ibd.2	-824703	ENSMUSG000	Cobl	cordon-bleu WH2 repeat
chr11	13312262	13312395	134	135	13312352	20	7.74501	3.7745	4.70467	Adipose_R1_peak_	Distal Intergen	11	12236676	12464960	228285	2	12808	uc007ibd.2	-847302	ENSMUSG000	Cobl	cordon-bleu WH2 repeat
chr11	13354825	13355096	272	273	13354966	56	24.19188	5.25641	20.43163	Adipose_R2_peak_	Distal Intergen	11	14072103	14179041	106939	2	75280	uc056yju.1	823945	ENSMUSG000	4930554G24R	RIKEN cDNA 4930554G24 gene
chr11	13354830	13355133	304	305	13354969	50	34.50784	9.16663	30.72138	Adipose_R1_peak_	Distal Intergen	11	14072103	14179041	106939	2	75280	uc056yju.1	823908	ENSMUSG000	4930554G24R	RIKEN cDNA 4930554G24 gene
chr11	13379348	13379570	223	224	13379470	24	10.60668	4.49345	7.40961	Adipose_R1_peak_	Distal Intergen	11	14072103	14179041	106939	2	75280	uc056yju.1	799471	ENSMUSG000	4930554G24R	RIKEN cDNA 4930554G24 gene
chr11	13384240	13384405	166	167	13384329	23	9.86443	4.31371	6.70247	Adipose_R1_peak_	Distal Intergen	11	14072103	14179041	106939	2	75280	uc056yju.1	794636	ENSMUSG000	4930554G24R	RIKEN cDNA 4930554G24 gene
chr11	13546914	13547070	157	158	13546986	19	7.07687	3.59476	4.08458	Adipose_R1_peak_	Distal Intergen	11	14072103	14179041	106939	2	75280	uc056yju.1	631971	ENSMUSG000	4930554G24R	RIKEN cDNA 4930554G24 gene
chr11	13728142	13728300	159	160	13728230	21	8.43286	3.95423	5.34896	Adipose_R1_peak_	Distal Intergen	11	14072103	14179041	106939	2	75280	uc056yju.1	450741	ENSMUSG000	4930554G24R	RIKEN cDNA 4930554G24 gene
chr11	13758095	13758231	137	138	13758172	22	9.13959	4.13397	6.01526	Adipose_R1_peak_	Distal Intergen	11	14072103	14179041	106939	2	75280	uc056yju.1	420810	ENSMUSG000	4930554G24R	RIKEN cDNA 4930554G24 gene
chr11	13813042	13813341	300	301	13813192	41	25.38991	7.54899	21.76305	Adipose_R1_peak_	Distal Intergen	11	14072103	14179041	106939	2	75280	uc056yju.1	365700	ENSMUSG000	4930554G24R	RIKEN cDNA 4930554G24 gene
chr11	13813137	13813257	121	122	13813195	32	8.29282	3.04319	5.18352	Adipose_R2_peak_	Distal Intergen	11	14072103	14179041	106939	2	75280	uc056yju.1	365784	ENSMUSG000	4930554G24R	RIKEN cDNA 4930554G24 gene
chr11	13821230	13821470	241	242	13821350	36	20.67077	6.6503	17.14779	Adipose_R1_peak_	Distal Intergen	11	14072103	14179041	106939	2	75280	uc056yju.1	357571	ENSMUSG000	4930554G24R	RIKEN cDNA 4930554G24 gene
chr11	13821265	13821419	155	156	13821357	36	10.5378	3.41206	7.27949	Adipose_R2_peak_	Distal Intergen	11	14072103	14179041	106939	2	75280	uc056yju.1	357622	ENSMUSG000	4930554G24R	RIKEN cDNA 4930554G24 gene
chr11	13821824	13822087	264	265	13821956	38	22.52591	7.00978	18.96	Adipose_R1_peak_	Distal Intergen	11	14072103	14179041	106939	2	75280	uc056yju.1	356954	ENSMUSG000	4930554G24R	RIKEN cDNA 4930554G24 gene
chr11	13821882	13821982	101	102	13821949	27	5.77405	2.5821	2.88921	Adipose_R2_peak_	Distal Intergen	11	14072103	14179041	106939	2	75280	uc056yju.1	357059	ENSMUSG000	4930554G24R	RIKEN cDNA 4930554G24 gene
chr11	13950309	13950568	260	261	13950442	37	21.59274	6.83004	18.04766	Adipose_R1_peak_	Distal Intergen	11	14072103	14179041	106939	2	75280	uc056yju.1	228473	ENSMUSG000	4930554G24R	RIKEN cDNA 4930554G24 gene
chr11	13952243	13952478	236	237	13952356	28	13.73721	5.2124	10.41463	Adipose_R1_peak_	Distal Intergen	11	14072103	14179041	106939	2	75280	uc056yju.1	226563	ENSMUSG000	4930554G24R	RIKEN cDNA 4930554G24 gene
chr11	13952357	13952530	174	175	13952414	28	6.25023	2.67431	3.31626	Adipose_R2_peak_	Distal Intergen	11	14072103	14179041	106939	2	75280	uc056yju.1	226511	ENSMUSG000	4930554G24R	RIKEN cDNA 4930554G24 gene
chr11	14390237	14390437	201	202	14390328	34	9.39139	3.22762	6.20474	Adipose_R2_peak_	Distal Intergen	11	14560336	14599225	38890	2	73307	uc007ibh.2	208788	ENSMUSG000	1700046C09Ri	RIKEN cDNA 1700046C09 gene
chr11	14390244	14390456	213	214	14390360	29	14.55748	5.39214	11.20652	Adipose_R1_peak_	Distal Intergen	11	14560336	14599225	38890	2	73307	uc007ibh.2	208769	ENSMUSG000	1700046C09Ri	RIKEN cDNA 1700046C09 gene
chr11	14474772	14474947	176	177	14474833	27	12.93148	5.03266	9.63827	Adipose_R1_peak_	Distal Intergen	11	14560336	14599225	38890	2	73307	uc007ibh.2	124278	ENSMUSG000	1700046C09Ri	RIKEN cDNA 1700046C09 gene
chr11	14896965	14897166	202	203	14897072	24	10.60668	4.49345	7.40961	Adipose_R1_peak_	Distal Intergen	11	14599240	14600316	1077	1	432536	uc007ibi.2	297725	ENSMUSG000	Pom121l12	POM121 membrane glycoprotein-like 12
chr11	14921541	14921820	280	281	14921684	58	43.19021	10.60454	39.28351	Adipose_R1_peak_	Distal Intergen	11	14599240	14600316	1077	1	432536	uc007ibi.2	322301	ENSMUSG000	Pom121l12	POM121 membrane glycoprotein-like 12
chr11	14921609	14921810	202	203	14921680	43	14.8939	4.05758	11.42017	Adipose_R2_peak_	Distal Intergen	11	14599240	14600316	1077	1	432536	uc007ibi.2	322369	ENSMUSG000	Pom121l12	POM121 membrane glycoprotein-like 12
chr11	15079832	15080137	306	307	15079991	77	63.74121	13.36682	59.63028	Adipose_R1_peak_	Distal Intergen	11	14599240	14600316	1077	1	432536	uc007ibi.2	480592	ENSMUSG000	Pom121l12	POM121 membrane glycoprotein-like 12
chr11	15079838	15080146	309	310	15079988	78	42.70965	7.2852	38.64095	Adipose_R2_peak_	Distal Intergen	11	14599240	14600316	1077	1	432536	uc007ibi.2	480598	ENSMUSG000	Pom121l12	POM121 membrane glycoprotein-like 12
chr11	15266501	15266659	159	160	15266555	38	11.72962	3.59649	8.4047	Adipose_R2_peak_	Distal Intergen	11	14599240	14600316	1077	1	432536	uc007ibi.2	667261	ENSMUSG000	Pom121l12	POM121 membrane glycoprotein-like 12
chr11	15279513	15279784	272	273	15279648	37	21.59274	6.83004	18.04766	Adipose_R1_peak_	Distal Intergen	11	14599240	14600316	1077	1	432536	uc007ibi.2	680273	ENSMUSG000	Pom121l12	POM121 membrane glycoprotein-like 12
chr11	15279584	15279776	193	194	15279654	40	12.96465	3.78093	9.57654	Adipose_R2_peak_	Distal Intergen	11	14599240	14600316	1077	1	432536	uc007ibi.2	680344	ENSMUSG000	Pom121l12	POM121 membrane glycoprotein-like 12
chr11	15280151	15280412	262	263	15280284	35	19.76029	6.47056	16.26028	Adipose_R1_peak_	Distal Intergen	11	14599240	14600316	1077	1	432536	uc007ibi.2	680911	ENSMUSG000	Pom121l12	POM121 membrane glycoprotein-like 12
chr11	15281211	15281429	219	220	15281321	31	16.23981	5.75161	12.83549	Adipose_R1_peak_	Distal Intergen	11	14599240	14600316	1077	1	432536	uc007ibi.2	681971	ENSMUSG000	Pom121l12	POM121 membrane glycoprotein-like 12
chr11	15303960	15304215	256	257	15304083	32	17.10103	5.93135	13.67137	Adipose_R1_peak_	Distal Intergen	11	14599240	14600316	1077	1	432536	uc007ibi.2	704720	ENSMUSG000	Pom121l12	POM121 membrane glycoprotein-like 12
chr11	15596189	15596361	173	174	15596257	23	9.86443	4.31371	6.70247	Adipose_R1_peak_	Distal Intergen	11	16257724	16284551	26828	1	211739	uc007ibk.2	-661363	ENSMUSG000	Vstm2a	V-set and transmembrane domain containing 2A
chr11	15856818	15857022	205	206	15857017	45	16.22858	4.24202	12.70278	Adipose_R2_peak_	Distal Intergen	11	16257724	16284551	26828	1	211739	uc007ibk.2	-400702	ENSMUSG000	Vstm2a	V-set and transmembrane domain containing 2A
chr11	15856876	15857022	147	148	15857010	21	8.43286	3.95423	5.34896	Adipose_R1_peak_	Distal Intergen	11	16257724	16284551	26828	1	211739	uc007ibk.2	-400702	ENSMUSG000	Vstm2a	V-set and transmembrane domain containing 2A
chr11	15859150	15859289	140	141	15859281	18	6.42934	3.41502	3.48999	Adipose_R1_peak_	Distal Intergen	11	16257724	16284551	26828	1	211739	uc007ibk.2	-398435	ENSMUSG000	Vstm2a	V-set and transmembrane domain containing 2A
chr11	15939502	15939699	198	199	15939629	27	12.93148	5.03266	9.63827	Adipose_R1_peak_	Distal Intergen	11	16257724	16284551	26828	1	211739	uc007ibk.2	-318025	ENSMUSG000	Vstm2a	V-set and transmembrane domain containing 2A
chr11	15939528	15939741	214	215	15939628	41	13.59774	3.87315	10.1803	Adipose_R2_peak_	Distal Intergen	11	16257724	16284551	26828	1	211739	uc007ibk.2	-317983	ENSMUSG000	Vstm2a	V-set and transmembrane domain containing 2A
chr11	16023880	16024041	162	163	16023946	22	9.13959	4.13397	6.01526	Adipose_R1_peak_	Distal Intergen	11	16257724	16284551	26828	1	211739	uc007ibk.2	-233683	ENSMUSG000	Vstm2a	V-set and transmembrane domain containing 2A
chr11	16024968	16025195	228	229	16025115	23	9.86443	4.31371	6.70247	Adipose_R1_peak_	Distal Intergen	11	16257724	16284551	26828	1	211739	uc007ibk.2	-232529	ENSMUSG000	Vstm2a	V-set and transmembrane domain containing 2A
chr11	16308668	16308824	157	158	16308728	16	5.20017	3.05554	2.38734	Adipose_R1_peak_	Distal Intergen	11	16262914	16284551	21638	1	211739	uc033ftx.1	45754	ENSMUSG000	Vstm2a	V-set and transmembrane domain containing 2A
chr11	16326976	16327216	241	242	16327092	26	12.14079	4.85292	8.87838	Adipose_R1_peak_	Distal Intergen	11	16262914	16284551	21638	1	211739	uc033ftx.1	64062	ENSMUSG000	Vstm2a	V-set and transmembrane domain containing 2A
chr11	16330686	16330844	159	160	16330747	25	9.93588	4.10932	6.77336	Adipose_R1_peak_	Distal Intergen	11	16262914	16284551	21638	1	211739	uc033ftx.1	67772	ENSMUSG000	Vstm2a	V-set and transmembrane domain containing 2A
chr11	16689092	16689286	195	196	16689211	38	11.72962	3.59649	8.4047	Adipose_R2_peak_	Distal Intergen	11	16752203	16887658	135456	1	13649	uc007ibn.1	-62917	ENSMUSG000	Egfr	epidermal growth factor receptor
chr11	16689159	16689301	143	144	16689222	24	10.60668	4.49345	7.40961	Adipose_R1_peak_	Distal Intergen	11	16752203	16887658	135456	1	13649	uc007ibn.1	-62902	ENSMUSG000	Egfr	epidermal growth factor receptor
chr11	16730888	16731102	215	216	16730974	24	10.60668	4.49345	7.40961	Adipose_R1_peak_	Distal Intergen	11	16752203	16887658	135456	1	13649	uc007ibn.1	-21101	ENSMUSG000	Egfr	epidermal growth factor receptor
chr11	17099389	17099576	188	189	17099493	33	17.97508	6.11109	14.52164	Adipose_R1_peak_	Distal Intergen	11	16971206	17052381	81176	2	56193	uc007ibw.2	-47008	ENSMUSG000	Plek	pleckstrin
chr11	17099408	17099584	177	178	17099527	40	12.96465	3.78093	9.57654	Adipose_R2_peak_	Distal Intergen	11	16971206	17052381	81176	2	56193	uc007ibw.2	-47027	ENSMUSG000	Plek	pleckstrin
chr11	17404058	17404329	272	273	17404186	47	31.38649	8.62742	27.65048	Adipose_R1_peak_	Distal Intergen	11	17257618	17269176	11559	1	57316	uc007ice.1	146440	ENSMUSG000	C1d	C1D nuclear receptor co-repressor
chr11	17404102	17404305	204	205	17404179	43	14.8939	4.05758	11.42017	Adipose_R2_peak_	Distal Intergen	11	17257618	17269176	11559	1	57316	uc007ice.1	146484	ENSMUSG000	C1d	C1D nuclear receptor co-repressor
chr11	17406880	17407267	388	389	17407021	91	79.94264	15.33524	75.71372	Adipose_R1_peak_	Distal Intergen	11	17257618	17269176	11559	1	57316	uc007ice.1	149262	ENSMUSG000	C1d	C1D nuclear receptor co-repressor
chr11	17406889	17407213	325	326	17407031	82	46.38432	7.65407	42.27038	Adipose_R2_peak_	Distal Intergen	11	17257618	17269176	11559	1	57316	uc007ice.1	149271	ENSMUSG000	C1d	C1D nuclear receptor co-repressor
chr11	17486216	17486770	555	556	17486484	49	33.45876	8.98689	29.68925	Adipose_R1_peak_	Distal Intergen	11	17257618	17269176	11559	1	57316	uc007ice.1	228598	ENSMUSG000	C1d	C1D nuclear receptor co-repressor
chr11	17486485	17486628	144	145	17486494	26	5.31252	2.48988	2.47999	Adipose_R2_peak_	Distal Intergen	11	17257618	17269176	11559	1	57316	uc007ice.1	228867	ENSMUSG000	C1d	C1D nuclear receptor co-repressor
chr11	17517268	17517492	225	226	17517381	27	10.77876	4.20743	7.58042	Adipose_R1_peak_	Distal Intergen	11	17257618	17269176	11559	1	57316	uc007ice.1	259650	ENSMUSG000	C1d	C1D nuclear receptor co-repressor
chr11	17517350	17517462	113	114	17517400	30	7.24477	2.85875	4.21917	Adipose_R2_peak_	Distal Intergen	11	17257618	17269176	11559	1	57316	uc007ice.1	259732	ENSMUSG000	C1d	C1D nuclear receptor co-repressor
chr11	17580655	17580825	171	172	17580744	21	8.43286	3.95423	5.34896	Adipose_R1_peak_	Distal Intergen	11	17257618	17269176	11559	1	57316	uc007ice.1	323037	ENSMUSG000	C1d	C1D nuclear receptor co-repressor
chr11	18049924	18050089	166	167	18050003	22	9.13959	4.13397	6.01526	Adipose_R1_peak_	Distal Intergen	11	18095986	18103385	7400	1	327872	uc056yjw.1	-45897	ENSMUSG000	Gm12018	predicted gene 12018
chr11	18234718	18234901	184	185	18234781	38	22.52591	7.00978	18.96	Adipose_R1_peak_	Distal Intergen	11	18095986	18103385	7400	1	327872	uc056yjw.1	138732	ENSMUSG000	Gm12018	predicted gene 12018

chr11	18237335	18237480	146	147	18237412	19	7.07687	3.59476	4.08458	Adipose_R1_peak_	Distal Interger	11	18095986	18103385	7400	1	327872	uc056yjw.1	141349	ENSMUSG000	Gm12018	predicted gene 12018
chr11	18297477	18297640	164	165	18297565	21	8.43286	3.95423	5.34896	Adipose_R1_peak_	Distal Interger	11	18095986	18103385	7400	1	327872	uc056yjw.1	201491	ENSMUSG000	Gm12018	predicted gene 12018
chr11	18298698	18298964	267	268	18298819	43	27.35048	7.90847	23.68561	Adipose_R1_peak_	Distal Interger	11	18095986	18103385	7400	1	327872	uc056yjw.1	202712	ENSMUSG000	Gm12018	predicted gene 12018
chr11	18300804	18301075	272	273	18300945	61	46.56983	11.14375	42.62347	Adipose_R1_peak_	Distal Interger	11	18095986	18103385	7400	1	327872	uc056yjw.1	204818	ENSMUSG000	Gm12018	predicted gene 12018
chr11	18300862	18301066	205	206	18300948	45	16.22858	4.24202	12.70278	Adipose_R2_peak_	Distal Interger	11	18095986	18103385	7400	1	327872	uc056yjw.1	204876	ENSMUSG000	Gm12018	predicted gene 12018
chr11	18301363	18301635	273	274	18301471	42	26.36523	7.72873	22.71906	Adipose_R1_peak_	Distal Interger	11	18095986	18103385	7400	1	327872	uc056yjw.1	205377	ENSMUSG000	Gm12018	predicted gene 12018
chr11	18301390	18301578	189	190	18301457	40	12.96465	3.78093	9.57654	Adipose_R2_peak_	Distal Interger	11	18095986	18103385	7400	1	327872	uc056yjw.1	205404	ENSMUSG000	Gm12018	predicted gene 12018
chr11	18331744	18332290	547	548	18332030	75	38.80889	6.74672	34.79588	Adipose_R2_peak_	Distal Interger	11	18095986	18103385	7400	1	327872	uc056yjw.1	235758	ENSMUSG000	Gm12018	predicted gene 12018
chr11	18331750	18332147	398	399	18332005	44	14.12372	3.80773	10.79891	Adipose_R1_peak_	Distal Interger	11	18095986	18103385	7400	1	327872	uc056yjw.1	235764	ENSMUSG000	Gm12018	predicted gene 12018
chr11	18340459	18340702	244	245	18340586	49	33.45876	8.98689	29.68925	Adipose_R1_peak_	Distal Interger	11	18095986	18103385	7400	1	327872	uc056yjw.1	244473	ENSMUSG000	Gm12018	predicted gene 12018
chr11	18340512	18340651	140	141	18340583	33	8.83596	3.1354	5.68652	Adipose_R2_peak_	Distal Interger	11	18095986	18103385	7400	1	327872	uc056yjw.1	244526	ENSMUSG000	Gm12018	predicted gene 12018
chr11	18457484	18457658	175	176	18457593	20	7.74501	3.7745	4.70467	Adipose_R1_peak_	Distal Interger	11	18095986	18103385	7400	1	327872	uc056yjw.1	361498	ENSMUSG000	Gm12018	predicted gene 12018
chr11	18457485	18457630	146	147	18457530	29	6.74062	2.76653	3.75989	Adipose_R2_peak_	Distal Interger	11	18095986	18103385	7400	1	327872	uc056yjw.1	361499	ENSMUSG000	Gm12018	predicted gene 12018
chr11	20242538	20242707	170	171	20242586	16	5.20017	3.05554	2.38734	Adipose_R1_peak_	Promoter (<=	11	20238386	20242246	3861	2	216543	uc007icx.1	-292	ENSMUSG000	Cep68	centrosomal protein 68
chr11	20250136	20250290	155	156	20250181	30	7.24477	2.85875	4.21917	Adipose_R2_peak_	Promoter (<=	11	20227037	20249424	22388	2	216543	uc007icw.1	-712	ENSMUSG000	Cep68	centrosomal protein 68
chr11	21482505	21482866	362	363	21482683	102	82.69836	13.63083	78.45358	Adipose_R1_peak_	Distal Interger	11	21494730	21511180	16451	2	237694	uc011xsa.1	28314	ENSMUSG000	4932414J04Ril	RIKEN cDNA 4932414J04 gene
chr11	21482578	21482798	221	222	21482687	44	15.55653	4.1498	12.05625	Adipose_R2_peak_	Distal Interger	11	21494730	21511180	16451	2	237694	uc011xsa.1	28382	ENSMUSG000	4932414J04Ril	RIKEN cDNA 4932414J04 gene
chr11	23523351	23523635	285	286	23523503	31	16.23981	5.75161	12.83549	Adipose_R1_peak_	Distal Interger	11	23516203	23519942	3740	2	67358	uc011xsd.1	-3409	ENSMUSG000	1700093K21Ri	RIKEN cDNA 1700093K21 gene
chr11	23523444	23523582	139	140	23523538	32	8.29282	3.04319	5.18352	Adipose_R2_peak_	Distal Interger	11	23516203	23519942	3740	2	67358	uc011xsd.1	-3502	ENSMUSG000	1700093K21Ri	RIKEN cDNA 1700093K21 gene
chr11	24658648	24658835	188	189	24658725	28	13.73721	5.2124	10.41463	Adipose_R1_peak_	Distal Interger	11	24721006	24730722	9717	2	1.00E+08	uc011xsh.1	71887	ENSMUSG000	Gm10466	predicted gene 10466
chr11	24878036	24878194	159	160	24878116	32	8.29282	3.04319	5.18352	Adipose_R2_peak_	Distal Interger	11	24721006	24730722	9717	2	1.00E+08	uc011xsh.1	-147314	ENSMUSG000	Gm10466	predicted gene 10466
chr11	24878045	24878251	207	208	24878143	33	17.97508	6.11109	14.52164	Adipose_R1_peak_	Distal Interger	11	24721006	24730722	9717	2	1.00E+08	uc011xsh.1	-147323	ENSMUSG000	Gm10466	predicted gene 10466
chr11	25066464	25066651	188	189	25066530	25	11.36566	4.67319	8.13482	Adipose_R1_peak_	Distal Interger	11	25326107	25367137	41031	1	71234	uc007iga.2	-259456	ENSMUSG000	4933427E13Ri	RIKEN cDNA 4933427E13 gene
chr11	25201312	25201608	297	298	25201455	74	61.90123	13.48034	57.80368	Adipose_R1_peak_	Distal Interger	11	25326107	25367137	41031	1	71234	uc007iga.2	-124499	ENSMUSG000	4933427E13Ri	RIKEN cDNA 4933427E13 gene
chr11	25201323	25201589	267	268	25201446	64	30.56565	5.99415	26.6807	Adipose_R2_peak_	Distal Interger	11	25326107	25367137	41031	1	71234	uc007iga.2	-124518	ENSMUSG000	4933427E13Ri	RIKEN cDNA 4933427E13 gene
chr11	25216347	25216558	212	213	25216443	37	21.59274	6.83004	18.04766	Adipose_R1_peak_	Distal Interger	11	25326107	25367137	41031	1	71234	uc007iga.2	-109549	ENSMUSG000	4933427E13Ri	RIKEN cDNA 4933427E13 gene
chr11	25374090	25374356	267	268	25374224	40	24.42476	7.36925	20.81738	Adipose_R1_peak_	Distal Interger	11	25326107	25367137	41031	1	71234	uc007iga.2	47983	ENSMUSG000	4933427E13Ri	RIKEN cDNA 4933427E13 gene
chr11	25375240	25375516	277	278	25375384	63	48.85777	11.50323	44.88581	Adipose_R1_peak_	Distal Interger	11	25326107	25367137	41031	1	71234	uc007iga.2	49133	ENSMUSG000	4933427E13Ri	RIKEN cDNA 4933427E13 gene
chr11	25375256	25375500	245	246	25375370	39	12.34187	3.68871	8.98501	Adipose_R2_peak_	Distal Interger	11	25326107	25367137	41031	1	71234	uc007iga.2	49149	ENSMUSG000	4933427E13Ri	RIKEN cDNA 4933427E13 gene
chr11	25483532	25483976	445	446	25483596	19	7.07687	3.59476	4.08458	Adipose_R1_peak_	Distal Interger	11	25326107	25367137	41031	1	71234	uc007iga.2	157425	ENSMUSG000	4933427E13Ri	RIKEN cDNA 4933427E13 gene
chr11	25970599	25970853	255	256	25970707	46	16.90986	4.33423	13.35898	Adipose_R2_peak_	Intron (uc011x	11	26202240	26202348	109	1	1.02E+08	uc033fuc.1	-231387	ENSMUSG000	Mir6372	microRNA 6372
chr11	25970609	25970840	232	233	25970721	26	12.14079	4.85292	8.87838	Adipose_R1_peak_	Intron (uc011x	11	26202240	26202348	109	1	1.02E+08	uc033fuc.1	-231400	ENSMUSG000	Mir6372	microRNA 6372
chr11	26461646	26461793	148	149	26461727	20	7.74501	3.7745	4.70467	Adipose_R1_peak_	Intron (uc007i	11	26387084	26469124	82041	1	67030	uc007igd.1	74562	ENSMUSG000	Fancl	Fanconi anemia, complementation group L
chr11	26462924	26463259	336	337	26463083	73	60.68511	13.3006	56.59814	Adipose_R1_peak_	Intron (uc007i	11	26387084	26469124	82041	1	67030	uc007igd.1	75840	ENSMUSG000	Fancl	Fanconi anemia, complementation group L
chr11	26462972	26463226	255	256	26463097	74	39.12209	6.91633	35.10124	Adipose_R2_peak_	Intron (uc007i	11	26387084	26469124	82041	1	67030	uc007igd.1	75888	ENSMUSG000	Fancl	Fanconi anemia, complementation group L
chr11	26464961	26465102	142	143	26465024	17	5.80341	3.23528	2.92378	Adipose_R1_peak_	Intron (uc007i	11	26387084	26469124	82041	1	67030	uc007igd.1	77877	ENSMUSG000	Fancl	Fanconi anemia, complementation group L
chr11	26466343	26466550	208	209	26466380	18	6.42934	3.41502	3.48999	Adipose_R1_peak_	Intron (uc007i	11	26387084	26469124	82041	1	67030	uc007igd.1	79259	ENSMUSG000	Fancl	Fanconi anemia, complementation group L
chr11	26671629	26671985	357	358	26671813	108	105.1994	19.21912	100.8428	Adipose_R1_peak_	Distal Interger	11	26471402	26593920	122519	2	69922	uc007igf.2	-77709	ENSMUSG000	Vrk2	vaccinia related kinase 2
chr11	26671670	26671985	316	317	26671806	103	66.95583	9.59064	62.63862	Adipose_R2_peak_	Distal Interger	11	26471402	26593920	122519	2	69922	uc007igf.2	-77750	ENSMUSG000	Vrk2	vaccinia related kinase 2
chr11	26898732	26898912	181	182	26898838	29	14.55748	5.39214	11.20652	Adipose_R1_peak_	Distal Interger	11	26785108	26787859	2752	1	654472	uc007igj.1	113624	NA	Gm12070	glyceraldehyde-3-phosphate dehydrogenase pseudogene
chr11	26898733	26898920	188	189	26898849	37	11.12817	3.50427	7.83613	Adipose_R2_peak_	Distal Interger	11	26785108	26787859	2752	1	654472	uc007igj.1	113625	NA	Gm12070	glyceraldehyde-3-phosphate dehydrogenase pseudogene
chr11	27020022	27020209	188	189	27020118	30	15.39182	5.57188	12.01394	Adipose_R1_peak_	Distal Interger	11	26785108	26787859	2752	1	654472	uc007igj.1	234914	NA	Gm12070	glyceraldehyde-3-phosphate dehydrogenase pseudogene
chr11	27362304	27362548	245	246	27362424	37	21.59274	6.83004	18.04766	Adipose_R1_peak_	Distal Interger	11	26785108	26787859	2752	1	654472	uc007igj.1	577196	NA	Gm12070	glyceraldehyde-3-phosphate dehydrogenase pseudogene
chr11	27362366	27362536	171	172	27362443	38	11.72962	3.59649	8.4047	Adipose_R2_peak_	Distal Interger	11	26785108	26787859	2752	1	654472	uc007igj.1	577258	NA	Gm12070	glyceraldehyde-3-phosphate dehydrogenase pseudogene
chr11	27535579	27535830	252	253	27535709	42	26.36523	7.72873	22.71906	Adipose_R1_peak_	Distal Interger	11	26785108	26787859	2752	1	654472	uc007igj.1	750471	NA	Gm12070	glyceraldehyde-3-phosphate dehydrogenase pseudogene
chr11	27535606	27535782	177	178	27535697	33	8.83596	3.1354	5.68652	Adipose_R2_peak_	Distal Interger	11	26785108	26787859	2752	1	654472	uc007igj.1	750498	NA	Gm12070	glyceraldehyde-3-phosphate dehydrogenase pseudogene
chr11	27536238	27536566	329	330	27536381	44	28.34544	8.08821	24.66239	Adipose_R1_peak_	Distal Interger	11	26785108	26787859	2752	1	654472	uc007igj.1	751130	NA	Gm12070	glyceraldehyde-3-phosphate dehydrogenase pseudogene
chr11	27536294	27536514	221	222	27536376	44	15.55653	4.1498	12.05625	Adipose_R2_peak_	Distal Interger	11	26785108	26787859	2752	1	654472	uc007igj.1	751186	NA	Gm12070	glyceraldehyde-3-phosphate dehydrogenase pseudogene
chr11	27540736	27540976	241	242	27540880	40	24.42476	7.36925	20.81738	Adipose_R1_peak_	Distal Interger	11	26785108	26787859	2752	1	654472	uc007igj.1	755628	NA	Gm12070	glyceraldehyde-3-phosphate dehydrogenase pseudogene
chr11	27540774	27540975	202	203	27540899	47	17.60017	4.42645	14.02531	Adipose_R2_peak_	Distal Interger	11	26785108	26787859	2752	1	654472	uc007igj.1	755666	NA	Gm12070	glyceraldehyde-3-phosphate dehydrogenase pseudogene
chr11	27574553	27574823	271	272	27574666	46	30.36364	8.44768	26.64488	Adipose_R1_peak_	Distal Interger	11	26785108	26787859	2752	1	654472	uc007igj.1	789445	NA	Gm12070	glyceraldehyde-3-phosphate dehydrogenase pseudogene
chr11	27574567	27574767	201	202	27574665	37	11.12817	3.50427	7.83613	Adipose_R2_peak_	Distal Interger	11	26785108	26787859	2752	1	654472	uc007igj.1	789459	NA	Gm12070	glyceraldehyde-3-phosphate dehydrogenase pseudogene
chr11	27619407	27619555	149	150	27619547	31	7.7623	2.95097	4.69388	Adipose_R2_peak_	Distal Interger	11	26785108	26787859	2752	1	654472	uc007igj.1	834299	NA	Gm12070	glyceraldehyde-3-phosphate dehydrogenase pseudogene
chr11	27685643	27685918	276	277	27685782	46	30.36364	8.44768	26.64488	Adipose_R1_peak_	Distal Interger	11	28385684	28584324	198641	2	216613	uc007igk.3	898406	ENSMUSG000	Ccdc85a	coiled-coil domain containing 85A
chr11	27685695	27685856	162	163	27685781	30	7.24477	2.85875	4.21917	Adipose_R2_peak_	Distal Interger	11	28385684	28584324	198641	2	216613	uc007igk.3	898468	ENSMUSG000	Ccdc85a	coiled-coil domain containing 85A
chr11	27687225	27687518	294	295	27687372	50	34.50784	9.16663	30.72138	Adipose_R1_peak_	Distal Interger	11	28385684	28584324	198641	2	216613	uc007igk.3	896806	ENSMUSG000	Ccdc85a	coiled-coil domain containing 85A
chr11	27687287	27687487	201	202	27687372	38	11.72962	3.59649	8.4047	Adipose_R2_peak_	Distal Interger	11	28385684	28584324	198641	2	216613	uc007igk.3	896837	ENSMUSG000	Ccdc85a	coiled-coil domain containing 85A
chr11	27688941	27689110	170	171	27689032	20	7.74501	3.7745	4.70467	Adipose_R1_peak_	Distal Interger	11	28385684	28584324	198641	2	216613	uc007igk.3	895214	ENSMUSG000	Ccdc85a	coiled-coil domain containing 85A
chr11	27823402	27823728	327	328	27823562	109	107.6402	19.77117	103.273	Adipose_R1_peak_	Distal Interger	11	28385684	28584324	198641	2	216613	uc007igk.3	760596	ENSMUSG000	Ccdc85a	coiled-coil domain containing 85A
chr11	27823420	27823715	296	297	27823572	115	79.5602	10.69726	75.15137	Adipose_R2_peak_	Distal Interger	11	28385684	28584324	198641	2	216613	uc007igk.3	760609	ENSMUSG000	Ccdc85a	coiled-coil domain containing 85A
chr11	27851967	27852133	167	168	27852032	26	5.31252	2.48988	2.47999	Adipose_R2_peak_	Distal Interger	11	28385684	28584324	198641	2	216613	uc007igk.3	732191	ENSMUSG000	Ccdc85a	coiled-coil domain containing 85A
chr11	27852970	27853272	303	304	27853195	31	7.7623	2.95097	4.69388	Adipose_R2_peak_	Distal Interger	11	28385684	28584324	198641	2	216613	uc007igk.3	731052	ENSMUSG000	Ccdc85a	coiled-coil domain containing 85A
chr11	27854580	27854767	188	189	27854625	27	5.77405	2.5821	2.88921	Adipose_R2_peak_	Distal Interger	11	28385684	28584324	198641	2	216613	uc007igk.3	729557	ENSMUSG000	Ccdc85a	coiled-coil domain containing 85A
chr11	27856000	27856115	116	117	27856077	30	7.24477	2.85875	4.21917	Adipose_R2_peak_	Distal Interger	11	28385684	28584324	198641	2	216613	uc007igk.3	728209	ENSMUSG000	Ccdc85a	coiled-coil domain containing 85A
chr11	27860958	27861323	366	367	27861204	32	8.29282	3.04319	5.18352	Adipose_R2_peak_	Distal Interger	11	28385684	28584324	198641	2	216613	uc007igk.3	723001	ENSMUSG000	Ccdc85a	coiled-coil domain containing 85A
chr11	27863650	27863763	114	115	27863720	26	5.31252	2.48988	2.47999	Adipose_R2_peak_	Distal Interger	11	28385684	28584324	198641	2	216613	uc007igk.3	720561	ENSMUSG000	Ccdc85a	coiled-coil domain containing 85A
chr11	27865225	27865372	148	149	27865276	29	6.74062	2.76653	3.75989	Adipose_R2_peak_	Distal Interger	11	28385684	28584324	198641	2	216613	uc007igk.3	718952	ENSMUSG000	Ccdc85a	coiled-coil domain containing 85A
chr11	27872324	27872459	136	137	27872368	29	6.74062	2.76653	3.75989	Adipose_R2_peak_	Distal Interger	11	28385684	28584324	198641	2	216613	uc007igk.3	711865	ENSMUSG000	Ccdc85a	coiled-coil domain containing 85A
chr11	27873980	27874111	132	133	27874078	27	5.77405	2.5821	2.88921	Adipose_R2_peak_	Distal Interger	11	28385684	28584324	198641	2	216613	uc007igk.3	710213	ENSMUSG000	Ccdc85a	coiled-coil domain containing 85A
chr11	28102703	28102817	115	116	28102755	29	6.74062	2.76653	3.75989	Adipose_R2_peak_	Distal Interger	11	28385684	28584324	198641	2	216613	uc007igk.3	481507	ENSMUSG000	Ccdc85a	coiled-coil domain containing 85A
chr11	28102705	28102853	149	150	28102768	21	8.43286	3.95423	5.34896	Adipose_R1_peak_	Distal Interger	11	28385684	28584324	198641	2	216613	uc007igk.3	481471	ENSMUSG000	Ccdc85a	coiled-coil domain containing 85A
chr11	28237805	28237976	172	173	28237891	24	10.60668	4.49345	7.40961	Adipose_R1_peak_	Distal Interger	11	28385684	28584324	198641	2	216613	uc007igk.3	346348	ENSMUSG000	Ccdc85a	coiled-coil domain containing 85A
chr11	28294455	28294670	216	217	28294573	30	15.39182	5.57188	12.01394	Adipose_R1_peak_	Distal Interger	11	28385684	28584324	198641	2	216613	uc007igk.3	289654	ENSMUSG000	Ccdc85a	coiled-coil domain containing 85A
chr11	28294486	28294642	157	158	28294583	31	7.7623	2.95097	4.69388	Adipose_R2_peak_	Distal Interger	11	28385684	28584324	198641	2	216613	uc007igk.3	289682	ENSMUSG000	Ccdc85a	coiled-coil domain containing 85A
chr11	28315411	28315619	209	210	28315508	33	17.97508	6.11109	14.52164	Adipose_R1_peak_	Distal Interger	11	28385684	28584324	198641	2	216613	uc007igk.3	268705	ENSMUSG000	Ccdc85a	coiled-coil domain containing 85A
chr11	28315456	28315579	124	125	28315525	29	6.74062	2.76653	3.75989	Adipose_R2_peak_	Distal Interger	11	28385684	28584324	198641	2	216613	uc007igk.3	268745	ENSMUSG000	Ccdc85a	coiled-coil domain containing 85A
chr11	28473935	28474199	265	266	28474085	33	17.97508	6.11109	14.52164	Adipose_R1_peak_	Intron (uc007i	11	28385684	28584324	198641	2	216613	uc007igk.3	110125	ENSMUSG000	Ccdc85a	coiled-coil domain containing 85A
chr11	28473992	28474193	202	203	28474095	46	16.90986	4.33423	13.35898	Adipose_R2_peak_	Intron (uc007i	11	28385684	28584324	198641	2	216613	uc007igk.3	110131	ENSMUSG000	Ccdc85a	coiled-coil domain containing 85A
chr11	29082703	29082935	233	234	29082814	31	16.23981	5.75161	12.83549	Adipose_R1_peak_	Distal Interger	11	29130751	29161828	31078	1	71701	uc007igp.1	-47816	ENSMUSG000	Pnpt1	polyribonucleotide nucleotidyltransferase 1
chr11	29082735	29082886	152	153	29082811	34	9.39139	3.22762	6.20474	Adipose_R2_peak_	Distal Interger	11	29130751	29161828	31078	1	71701	uc007igp.1	-47865	ENSMUSG000	Pnpt1	polyribonucleotide nucleotidyltransferase 1
chr11	29087350	29087558	209	210	29087444	31	16.23981	5.75161	12.83549	Adipose_R1_peak_	Distal Interger	11	29130751	29161828	31078	1	71701	uc007igp.1	-43193	ENSMUSG000	Pnpt1	polyribonucleotide nucleotidyltransferase 1
chr11	29087435	29087542	108	109	29087518	28	6.25023	2.67431	3.31626	Adipose_R2_peak_	Distal Interger	11	29130751	29161828	31078	1	71701	uc007igp.1	-43209	ENSMUSG000	Pnpt1	polyribonucleotide nucleotidyltransferase 1
chr11	29261186	29261323	138	139	29261247	19	7.07687	3.59476	4.08458	Adipose_R1_peak_	Distal Interger	11	29221536	29247272	25737	2	216618	uc007igu.1	-13914	ENSMUSG000	Cfap36	cilia and flagella associated protein 36
chr11	29263149	29263424	276	277	29263296	56	40.97333	10.24506	37.09402	Adipose_R1_peak_	Distal Interger	11	29221536	29247272	25737	2	216618	uc007igu.1	-15877	ENSMUSG000	Cfap36	cilia and flagella associated protein 36
chr11	29263167	29263360	194	195	29263287	47	17.60017	4.42645	14.02531	Adipose_R2_peak_	Distal Interger	11	29221536	29247272	25737	2	216618	uc007igu.1	-15895	ENSMUSG000	Cfap36	cilia and flagella associated protein 36
chr11	29591506	29591759	254	255	29591627	38	22.52591	7.00978	18.96	Adipose_R1_peak_	Distal Interger	11	29545842	29548040	2199	2	78294	uc007ihg.1	-43466	ENSMUSG000	Rps27a	ribosomal protein S27A
chr11	29591574	29591673	100	101	29591619	26	5.31252	2.48988	2.47999	Adipose_R2_peak_	Distal Interger	11	29545842	29548040	2199	2	78294	uc007ihg.1	-43534	ENSMUSG000	Rps27a	ribosomal protein S27A
chr11	29964892	29965150	259	260	29965025	45	29.34988	8.26794	25.64927	Adipose_R1_peak_	Intron (uc007il	11	29743051	30026033	282983	2	237711	uc007ihq.1	60883	ENSMUSG000	Eml6	echinoderm microtubule associated protein like 6
chr11	29964937	29965129	193	194	29965004	38	11.72962	3.59649	8.4047	Adipose_R2_peak_	Intron (uc007il	11	29743051	30026033	282983	2	237711	uc007ihq.1	60904	ENSMUSG000	Eml6	echinoderm microtubule associated protein like 6
chr11	30037408	30037608	201	202	30037505	28	13.73721	5.2124	10.41463	Adipose_R1_peak_	Distal Interger	11	29743051	30026033	282983	2	237711	uc007ihq.1	-11375	ENSMUSG000	Eml6	echinoderm microtubule associated protein like 6
chr11	30037471	30037635	165	166	30037552	30	7.24477	2.85875	4.21917	Adipose_R2_peak_	Distal Interger	11	29743051	30026033	282983	2	237711	uc007ihq.1	-11438	ENSMUSG000	Eml6	echinoderm microtubule associated protein like 6
chr11	30067556	30067790	235	236	30067679	45	29.34988	8.26794	25.64927	Adipose_R1_peak_	Distal Interger	11	29743051	30026033	282983	2	237711	uc007ihq.1	-41523	ENSMUSG000	Eml6	echinoderm microtubule associated protein like 6
chr11	30067578	30067781	204	205	30067682	46	16.90986	4.33423	13.35898	Adipose_R2_peak_	Distal Interger	11	29743051	30026033	282983	2	237711	uc007ihq.1	-41545	ENSMUSG000	Eml6	echinoderm microtubule associated protein like 6
chr11	30069767	30070251	485	486	30069909	38	22.52591	7.00978	18.96	Adipose_R1_peak_	Distal Interger	11	29743051	30026033	282983	2	237711	uc007ihq.1	-43734	ENSMUSG000	Eml6	echinoderm microtubule associated protein like 6

chr11	30069831	30069964	134	135	30069910	27	5.77405	2.5821	2.88921	Adipose_R2_peak_	Distal Interger	11	29743051	30026033	282983	2	237711	uc007ihq.1	-43798	ENSMUSG000	Eml6	echinoderm microtubule associated protein like 6
chr11	30071023	30071291	269	270	30071167	45	29.34988	8.26794	25.64927	Adipose_R1_peak_	Distal Interger	11	29743051	30026033	282983	2	237711	uc007ihq.1	-44990	ENSMUSG000	Eml6	echinoderm microtubule associated protein like 6
chr11	30071054	30071245	192	193	30071142	40	12.96465	3.78093	9.57654	Adipose_R2_peak_	Distal Interger	11	29743051	30026033	282983	2	237711	uc007ihq.1	-45021	ENSMUSG000	Eml6	echinoderm microtubule associated protein like 6
chr11	30072395	30072832	438	439	30072557	43	14.8939	4.05758	11.42017	Adipose_R2_peak_	Distal Interger	11	29743051	30026033	282983	2	237711	uc007ihq.1	-46362	ENSMUSG000	Eml6	echinoderm microtubule associated protein like 6
chr11	30072400	30072701	302	303	30072554	44	28.34544	8.08821	24.66239	Adipose_R1_peak_	Distal Interger	11	29743051	30026033	282983	2	237711	uc007ihq.1	-46367	ENSMUSG000	Eml6	echinoderm microtubule associated protein like 6
chr11	30610890	30611142	253	254	30611024	68	54.69342	12.40192	50.66183	Adipose_R1_peak_	Intron (uc007il	11	30505992	30649396	143405	2	75572	uc007ihw.1	38254	ENSMUSG000	Acyp2	acylphosphatase 2, muscle type
chr11	30610895	30611102	208	209	30610987	53	21.92215	4.97976	18.21755	Adipose_R2_peak_	Intron (uc007il	11	30505992	30649396	143405	2	75572	uc007ihw.1	38294	ENSMUSG000	Acyp2	acylphosphatase 2, muscle type
chr11	30688943	30689113	171	172	30689007	29	14.55748	5.39214	11.20652	Adipose_R1_peak_	Distal Interger	11	30505992	30649396	143405	2	75572	uc007ihw.1	-39547	ENSMUSG000	Acyp2	acylphosphatase 2, muscle type
chr11	30688949	30689081	133	134	30688987	28	6.25023	2.67431	3.31626	Adipose_R2_peak_	Distal Interger	11	30505992	30649396	143405	2	75572	uc007ihw.1	-39553	ENSMUSG000	Acyp2	acylphosphatase 2, muscle type
chr11	31668686	31668844	159	160	31668729	16	5.20017	3.05554	2.38734	Adipose_R1_peak_	Intron (uc007ii	11	31665150	31671862	6713	2	69556	uc007iim.1	3018	ENSMUSG000	Bod1	biorientation of chromosomes in cell division 1
chr11	31695614	31695793	180	181	31695685	31	7.7623	2.95097	4.69388	Adipose_R2_peak_	Distal Interger	11	31665150	31671862	6713	2	69556	uc007iim.1	-23752	ENSMUSG000	Bod1	biorientation of chromosomes in cell division 1
chr11	31774251	31774496	246	247	31774346	28	6.25023	2.67431	3.31626	Adipose_R2_peak_	Distal Interger	11	31800688	31824547	23860	2	414116	uc007iin.1	50051	ENSMUSG000	D630024D03R	RIKEN cDNA D630024D03 gene
chr11	31813515	31813683	169	170	31813592	22	9.13959	4.13397	6.01526	Adipose_R1_peak_	Intron (uc007ii	11	31800688	31824547	23860	2	414116	uc007iin.1	10864	ENSMUSG000	D630024D03R	RIKEN cDNA D630024D03 gene
chr11	32936489	32936716	228	229	32936510	16	5.20017	3.05554	2.38734	Adipose_R1_peak_	Distal Interger	11	32820376	32824594	4219	2	69351	uc007ikb.2	-111895	ENSMUSG000	Smim23	small integral membrane protein 23
chr11	32954208	32954422	215	216	32954325	47	31.38649	8.62742	27.65048	Adipose_R1_peak_	Distal Interger	11	32820376	32824594	4219	2	69351	uc007ikb.2	-129614	ENSMUSG000	Smim23	small integral membrane protein 23
chr11	32954262	32954357	96	97	32954307	30	7.24477	2.85875	4.21917	Adipose_R2_peak_	Distal Interger	11	32820376	32824594	4219	2	69351	uc007ikb.2	-129668	ENSMUSG000	Smim23	small integral membrane protein 23
chr11	33447293	33447630	338	339	33447460	34	18.86162	6.29083	15.38482	Adipose_R1_peak_	Intron (uc007il	11	33211794	33513746	301953	2	66011	uc007ikh.2	66116	ENSMUSG000	Ranbp17	RAN binding protein 17
chr11	33447425	33447523	99	100	33447511	27	5.77405	2.5821	2.88921	Adipose_R2_peak_	Intron (uc007il	11	33211794	33513746	301953	2	66011	uc007ikh.2	66223	ENSMUSG000	Ranbp17	RAN binding protein 17
chr11	33788990	33789182	193	194	33789065	19	7.07687	3.59476	4.08458	Adipose_R1_peak_	Intron (uc007il	11	33629341	33843585	214245	2	70357	uc007iko.2	54403	ENSMUSG000	Kcnip1	Kv channel-interacting protein 1
chr11	33899994	33900143	150	151	33900080	28	6.25023	2.67431	3.31626	Adipose_R2_peak_	Intron (uc007il	11	33629341	33843585	214245	2	70357	uc007iko.2	-56409	ENSMUSG000	Kcnip1	Kv channel-interacting protein 1
chr11	34853745	34854025	281	282	34853875	47	31.38649	8.62742	27.65048	Adipose_R1_peak_	Distal Interger	11	34809185	34833641	24457	2	70385	uc007ilc.2	-20104	ENSMUSG000	Spdl1	spindle apparatus coiled-coil protein 1
chr11	34853779	34853978	200	201	34853855	46	16.90986	4.33423	13.35898	Adipose_R2_peak_	Distal Interger	11	34809185	34833641	24457	2	70385	uc007ilc.2	-20138	ENSMUSG000	Spdl1	spindle apparatus coiled-coil protein 1
chr11	34990474	34991149	676	677	34990612	58	43.19021	10.60454	39.28351	Adipose_R1_peak_	Distal Interger	11	35121456	35708507	587052	1	20564	uc007ild.2	-130307	ENSMUSG000	Slit3	slit homolog 3 (Drosophila)
chr11	34990520	34990743	224	225	34990605	54	22.67101	5.07198	18.94758	Adipose_R2_peak_	Distal Interger	11	35121456	35708507	587052	1	20564	uc007ild.2	-130713	ENSMUSG000	Slit3	slit homolog 3 (Drosophila)
chr11	34990876	34991093	218	219	34990988	43	14.8939	4.05758	11.42017	Adipose_R2_peak_	Distal Interger	11	35121456	35708507	587052	1	20564	uc007ild.2	-130363	ENSMUSG000	Slit3	slit homolog 3 (Drosophila)
chr11	35059866	35060015	150	151	35059914	26	5.31252	2.48988	2.47999	Adipose_R2_peak_	Distal Interger	11	35121456	35708507	587052	1	20564	uc007ild.2	-61441	ENSMUSG000	Slit3	slit homolog 3 (Drosophila)
chr11	35247463	35247626	164	165	35247544	19	7.03841	3.57815	4.08458	Adipose_R1_peak_	Intron (uc007il	11	35121456	35708507	587052	1	20564	uc007ild.2	126007	ENSMUSG000	Slit3	slit homolog 3 (Drosophila)
chr11	35392542	35392787	246	247	35392604	19	7.07687	3.59476	4.08458	Adipose_R1_peak_	Intron (uc007il	11	35616816	35616925	110	1	723924	uc011xta.1	-224029	ENSMUSG000	Mir218-2	microRNA 218-2
chr11	35690448	35690600	153	154	35690512	39	21.57909	6.49017	18.04766	Adipose_R1_peak_	Intron (uc007il	11	35616816	35616925	110	1	723924	uc011xta.1	73632	ENSMUSG000	Mir218-2	microRNA 218-2
chr11	35690450	35690598	149	150	35690508	39	12.34187	3.68871	8.98501	Adipose_R2_peak_	Intron (uc007il	11	35616816	35616925	110	1	723924	uc011xta.1	73634	ENSMUSG000	Mir218-2	microRNA 218-2
chr11	37753097	37753486	390	391	37753353	65	51.17258	11.8627	47.17609	Adipose_R1_peak_	Distal Interger	11	37658989	37673166	14178	1	1.00E+08	uc056ykl.1	94108	ENSMUSG000	Gm12128	predicted gene 12128
chr11	37753230	37753485	256	257	37753361	57	24.96362	5.34863	21.18596	Adipose_R2_peak_	Distal Interger	11	37658989	37673166	14178	1	1.00E+08	uc056ykl.1	94241	ENSMUSG000	Gm12128	predicted gene 12128
chr11	37923401	37923553	153	154	37923464	19	7.07687	3.59476	4.08458	Adipose_R1_peak_	Distal Interger	11	37658989	37673166	14178	1	1.00E+08	uc056ykl.1	264412	ENSMUSG000	Gm12128	predicted gene 12128
chr11	38013280	38013404	125	126	38013324	27	5.77405	2.5821	2.88921	Adipose_R2_peak_	Distal Interger	11	37658989	37673166	14178	1	1.00E+08	uc056ykl.1	354291	ENSMUSG000	Gm12128	predicted gene 12128
chr11	38181367	38181561	195	196	38181458	27	12.93148	5.03266	9.63827	Adipose_R1_peak_	Distal Interger	11	38491548	38520045	28498	2	1.01E+08	uc007iln.2	338484	NA	Gm12130	predicted gene 12130
chr11	38196395	38196579	185	186	38196449	31	7.7623	2.95097	4.69388	Adipose_R2_peak_	Distal Interger	11	38491548	38520045	28498	2	1.01E+08	uc007iln.2	323466	NA	Gm12130	predicted gene 12130
chr11	38196407	38196597	191	192	38196501	26	12.14079	4.85292	8.87838	Adipose_R1_peak_	Distal Interger	11	38491548	38520045	28498	2	1.01E+08	uc007iln.2	323448	NA	Gm12130	predicted gene 12130
chr11	38198140	38198293	154	155	38198204	20	7.74501	3.7745	4.70467	Adipose_R1_peak_	Distal Interger	11	38491548	38520045	28498	2	1.01E+08	uc007iln.2	321752	NA	Gm12130	predicted gene 12130
chr11	39137429	39137626	198	199	39137482	20	7.74501	3.7745	4.70467	Adipose_R1_peak_	Distal Interger	11	39307703	39390225	82523	2	75315	uc007ilo.1	252599	NA	4930553C11R	RIKEN cDNA 4930553C11 gene
chr11	39137442	39137626	185	186	39137504	30	7.24477	2.85875	4.21917	Adipose_R2_peak_	Distal Interger	11	39307703	39390225	82523	2	75315	uc007ilo.1	252599	NA	4930553C11R	RIKEN cDNA 4930553C11 gene
chr11	39660704	39660845	142	143	39660765	19	7.07687	3.59476	4.08458	Adipose_R1_peak_	Distal Interger	11	39307703	39390225	82523	2	75315	uc007ilo.1	-270479	NA	4930553C11R	RIKEN cDNA 4930553C11 gene
chr11	39718979	39719103	125	126	39719024	30	7.24477	2.85875	4.21917	Adipose_R2_peak_	Distal Interger	11	39307703	39390225	82523	2	75315	uc007ilo.1	-328754	NA	4930553C11R	RIKEN cDNA 4930553C11 gene
chr11	40064372	40064576	205	206	40064485	32	8.29282	3.04319	5.18352	Adipose_R2_peak_	Distal Interger	11	40679314	40692682	13369	2	108645	uc007ilp.2	628106	ENSMUSG000	Mat2b	methionine adenosyltransferase II, beta
chr11	40064468	40064638	171	172	40064558	20	7.74501	3.7745	4.70467	Adipose_R1_peak_	Distal Interger	11	40679314	40692682	13369	2	108645	uc007ilp.2	628044	ENSMUSG000	Mat2b	methionine adenosyltransferase II, beta
chr11	40065713	40065860	148	149	40065787	21	8.43286	3.95423	5.34896	Adipose_R1_peak_	Distal Interger	11	40679314	40692682	13369	2	108645	uc007ilp.2	626822	ENSMUSG000	Mat2b	methionine adenosyltransferase II, beta
chr11	40258803	40258955	153	154	40258883	19	7.07687	3.59476	4.08458	Adipose_R1_peak_	Distal Interger	11	40679314	40692682	13369	2	108645	uc007ilp.2	433727	ENSMUSG000	Mat2b	methionine adenosyltransferase II, beta
chr11	40413559	40413751	193	194	40413637	31	16.23981	5.75161	12.83549	Adipose_R1_peak_	Distal Interger	11	40679314	40692682	13369	2	108645	uc007ilp.2	278931	ENSMUSG000	Mat2b	methionine adenosyltransferase II, beta
chr11	40415407	40415660	254	255	40415528	52	35.41556	9.08258	31.62513	Adipose_R1_peak_	Distal Interger	11	40679314	40692682	13369	2	108645	uc007ilp.2	277022	ENSMUSG000	Mat2b	methionine adenosyltransferase II, beta
chr11	40415441	40415635	195	196	40415537	43	14.8939	4.05758	11.42017	Adipose_R2_peak_	Distal Interger	11	40679314	40692682	13369	2	108645	uc007ilp.2	277047	ENSMUSG000	Mat2b	methionine adenosyltransferase II, beta
chr11	40419381	40419530	150	151	40419450	21	8.43286	3.95423	5.34896	Adipose_R1_peak_	Distal Interger	11	40679314	40692682	13369	2	108645	uc007ilp.2	273152	ENSMUSG000	Mat2b	methionine adenosyltransferase II, beta
chr11	40599607	40599782	176	177	40599686	18	6.42934	3.41502	3.48999	Adipose_R1_peak_	Distal Interger	11	40679314	40692682	13369	2	108645	uc007ilp.2	92900	ENSMUSG000	Mat2b	methionine adenosyltransferase II, beta
chr11	40639189	40639546	358	359	40639376	186	183.5036	21.68917	178.9088	Adipose_R1_peak_	Distal Interger	11	40679314	40692682	13369	2	108645	uc007ilp.2	53136	ENSMUSG000	Mat2b	methionine adenosyltransferase II, beta
chr11	40639209	40639527	319	320	40639377	159	130.0144	14.75484	125.4087	Adipose_R2_peak_	Distal Interger	11	40679314	40692682	13369	2	108645	uc007ilp.2	53155	ENSMUSG000	Mat2b	methionine adenosyltransferase II, beta
chr11	40751953	40752080	128	129	40752007	27	5.77405	2.5821	2.88921	Adipose_R2_peak_	Intron (uc007il	11	40748552	40755286	6735	2	12450	uc007ilz.1	3206	ENSMUSG000	Ccng1	cyclin G1
chr11	40787896	40788043	148	149	40787984	18	6.42934	3.41502	3.48999	Adipose_R1_peak_	Distal Interger	11	40748552	40755286	6735	2	12450	uc007ilz.1	-32610	ENSMUSG000	Ccng1	cyclin G1
chr11	40809150	40809436	287	288	40809306	47	31.38649	8.62742	27.65048	Adipose_R1_peak_	Distal Interger	11	40748552	40755286	6735	2	12450	uc007ilz.1	-53864	ENSMUSG000	Ccng1	cyclin G1
chr11	40809224	40809367	144	145	40809304	29	6.74062	2.76653	3.75989	Adipose_R2_peak_	Distal Interger	11	40748552	40755286	6735	2	12450	uc007ilz.1	-53938	ENSMUSG000	Ccng1	cyclin G1
chr11	40907532	40907853	322	323	40907730	86	76.92245	15.6372	72.71191	Adipose_R1_peak_	Distal Interger	11	40748552	40755286	6735	2	12450	uc007ilz.1	-152246	ENSMUSG000	Ccng1	cyclin G1
chr11	40907571	40907851	281	282	40907722	66	32.22715	6.17859	28.31407	Adipose_R2_peak_	Distal Interger	11	40748552	40755286	6735	2	12450	uc007ilz.1	-152285	ENSMUSG000	Ccng1	cyclin G1
chr11	40910875	40911105	231	232	40910987	39	23.47001	7.18952	19.88325	Adipose_R1_peak_	Distal Interger	11	40748552	40755286	6735	2	12450	uc007ilz.1	-155589	ENSMUSG000	Ccng1	cyclin G1
chr11	40910913	40911056	144	145	40910980	30	7.24477	2.85875	4.21917	Adipose_R2_peak_	Distal Interger	11	40748552	40755286	6735	2	12450	uc007ilz.1	-155627	ENSMUSG000	Ccng1	cyclin G1
chr11	40934182	40934345	164	165	40934265	31	7.7623	2.95097	4.69388	Adipose_R2_peak_	Distal Interger	11	40748552	40755286	6735	2	12450	uc007ilz.1	-178896	ENSMUSG000	Ccng1	cyclin G1
chr11	40958201	40958456	256	257	40958336	58	25.74274	5.44085	21.94821	Adipose_R2_peak_	Distal Interger	11	40748552	40755286	6735	2	12450	uc007ilz.1	-202915	ENSMUSG000	Ccng1	cyclin G1
chr11	40958225	40958452	228	229	40958337	43	21.27061	5.82288	17.74478	Adipose_R1_peak_	Distal Interger	11	40748552	40755286	6735	2	12450	uc007ilz.1	-202939	ENSMUSG000	Ccng1	cyclin G1
chr11	40990664	40990989	326	327	40990845	112	111.8104	20.31038	107.4269	Adipose_R1_peak_	Distal Interger	11	40748552	40755286	6735	2	12450	uc007ilz.1	-235378	ENSMUSG000	Ccng1	cyclin G1
chr11	40990684	40990982	299	300	40990850	111	75.29631	10.32839	70.91589	Adipose_R2_peak_	Distal Interger	11	40748552	40755286	6735	2	12450	uc007ilz.1	-235398	ENSMUSG000	Ccng1	cyclin G1
chr11	41022379	41022644	266	267	41022510	50	34.50784	9.16663	30.72138	Adipose_R1_peak_	Distal Interger	11	40748552	40755286	6735	2	12450	uc007ilz.1	-267093	ENSMUSG000	Ccng1	cyclin G1
chr11	41022448	41022593	146	147	41022494	31	7.7623	2.95097	4.69388	Adipose_R2_peak_	Distal Interger	11	40748552	40755286	6735	2	12450	uc007ilz.1	-267162	ENSMUSG000	Ccng1	cyclin G1
chr11	41093396	41093588	193	194	41093503	24	10.60668	4.49345	7.40961	Adipose_R1_peak_	Distal Interger	11	40748552	40755286	6735	2	12450	uc007ilz.1	-338110	ENSMUSG000	Ccng1	cyclin G1
chr11	41102504	41102691	188	189	41102612	24	10.60668	4.49345	7.40961	Adipose_R1_peak_	Distal Interger	11	40748552	40755286	6735	2	12450	uc007ilz.1	-347218	ENSMUSG000	Ccng1	cyclin G1
chr11	41102567	41102672	106	107	41102605	31	7.7623	2.95097	4.69388	Adipose_R2_peak_	Distal Interger	11	40748552	40755286	6735	2	12450	uc007ilz.1	-347281	ENSMUSG000	Ccng1	cyclin G1
chr11	41148942	41149072	131	132	41149005	22	9.13959	4.13397	6.01526	Adipose_R1_peak_	Distal Interger	11	40748552	40755286	6735	2	12450	uc007ilz.1	-393656	ENSMUSG000	Ccng1	cyclin G1
chr11	41338674	41338862	189	190	41338703	16	5.20017	3.05554	2.38734	Adipose_R1_peak_	Distal Interger	11	40748552	40755286	6735	2	12450	uc007ilz.1	-583388	ENSMUSG000	Ccng1	cyclin G1
chr11	43285330	43285620	291	292	43285487	87	78.20788	15.81694	73.98885	Adipose_R1_peak_	Distal Interger	11	43374397	43374461	65	2	387164	uc011xtg.1	88841	ENSMUSG000	Mir146	microRNA 146
chr11	43285352	43285611	260	261	43285478	87	51.09389	8.11516	46.92713	Adipose_R2_peak_	Distal Interger	11	43374397	43374461	65	2	387164	uc011xtg.1	88850	ENSMUSG000	Mir146	microRNA 146
chr11	43308679	43308966	288	289	43308827	60	45.43621	10.96401	41.50272	Adipose_R1_peak_	Distal Interger	11	43374397	43374461	65	2	387164	uc011xtg.1	65495	ENSMUSG000	Mir146	microRNA 146
chr11	43308706	43308973	268	269	43308836	61	28.12311	5.7175	24.28203	Adipose_R2_peak_	Distal Interger	11	43374397	43374461	65	2	387164	uc011xtg.1	65488	ENSMUSG000	Mir146	microRNA 146
chr11	43310057	43310260	204	205	43310181	32	17.10103	5.93135	13.67137	Adipose_R1_peak_	Distal Interger	11	43374397	43374461	65	2	387164	uc011xtg.1	64201	ENSMUSG000	Mir146	microRNA 146
chr11	43310086	43310249	164	165	43310171	29	6.74062	2.76653	3.75989	Adipose_R2_peak_	Distal Interger	11	43374397	43374461	65	2	387164	uc011xtg.1	64212	ENSMUSG000	Mir146	microRNA 146
chr11	44307017	44307532	516	517	44307167	35	19.76029	6.47056	16.26028	Adipose_R1_peak_	Distal Interger	11	44400063	44414677	14615	1	16160	uc007inc.2	-92531	ENSMUSG000	Il12b	interleukin 12b
chr11	44307028	44307279	252	253	44307146	46	16.90986	4.33423	13.35898	Adipose_R2_peak_	Distal Interger	11	44400063	44414677	14615	1	16160	uc007inc.2	-92784	ENSMUSG000	Il12b	interleukin 12b
chr11	44308461	44308657	197	198	44308546	24	10.60668	4.49345	7.40961	Adipose_R1_peak_	Distal Interger	11	44400063	44414677	14615	1	16160	uc007inc.2	-91406	ENSMUSG000	Il12b	interleukin 12b
chr11	44308475	44308594	120	121	44308533	28	6.25023	2.67431	3.31626	Adipose_R2_peak_	Distal Interger	11	44400063	44414677	14615	1	16160	uc007inc.2	-91469	ENSMUSG000	Il12b	interleukin 12b
chr11	44914091	44914378	288	289	44914266	36	20.67077	6.6503	17.14779	Adipose_R1_peak_	Intron (uc007ii	11	45102866	45116928	14063	2	1.01E+08	uc029rlj.1	202550	ENSMUSG000	Gm12159	predicted gene 12159
chr11	45100508	45100697	190	191	45100615	29	14.55748	5.39214	11.20652	Adipose_R1_peak_	Downstream (	11	45102866	45116928	14063	2	1.01E+08	uc029rlj.1	16231	ENSMUSG000	Gm12159	predicted gene 12159
chr11	45100553	45100682	130	131	45100628	32	8.29282	3.04319	5.18352	Adipose_R2_peak_	Downstream (	11	45102866	45116928	14063	2	1.01E+08	uc029rlj.1	16246	ENSMUSG000	Gm12159	predicted gene 12159
chr11	45129523	45129697	175	176	45129632	22	9.13959	4.13397	6.01526	Adipose_R1_peak_	Distal Interger	11	45102866	45116928	14063	2	1.01E+08	uc029rlj.1	-12595	ENSMUSG000	Gm12159	predicted gene 12159
chr11	45583746	45583991	246	247	45583885	57	42.07807	10.4248	38.18469	Adipose_R1_peak_	Distal Interger	11	45808083	45842878	34796	1	1.00E+08	uc007inp.2	-224092	NA	F630206G17R	RIKEN cDNA F630206G17 gene
chr11	45583822	45583968	147	148	45583882	29	6.74062	2.76653	3.75989	Adipose_R2_peak_	Distal Interger	11	45808083	45842878	34796	1	1.00E+08	uc007inp.2	-224115	NA	F630206G17R	RIKEN cDNA F630206G17 gene
chr11	45588566	45588779	214	215	45588682	27	12.93148	5.03266	9.63827	Adipose_R1_peak_	Distal Interger	11	45808083	45842878	34796	1	1.00E+08	uc007inp.2	-219304	NA	F630206G17R	RIKEN cDNA F630206G17 gene
chr11	45588631	45588804	174	175	45588708	38	11.72962	3.59649	8.4047	Adipose_R2_peak_	Distal Interger	11	45808083	45842878	34796	1	1.00E+08	uc007inp.2	-219279	NA	F630206G17R	RIKEN cDNA F630206G17 gene
chr11	45761878	45762037	160	161	45762004	27	12.93148	5.03266	9.63827	Adipose_R1_peak_	Distal Interger	11	45808083	45842878	34796	1	1.00E+08	uc007inp.2	-46046	NA	F630206G17R	RIKEN cDNA F630206G17 gene
chr11	46492895	46493235	341	342	46493063	58	43.19021	10.60454	39.28351	Adipose_R1_peak_	Distal Interger	11	46524251	46538156	13906	1	210535	uc011xtr.1	-31016	ENSMUSG000	Gm12169	predicted gene 12169
chr11	46492900	46493173	274	275	46493046	55	23.42763	5.16419	19.68558	Adipose_R2_peak_	Distal Interger	11	46524251	46538156	13906	1	210535	uc011xtr.1	-31078	ENSMUSG000	Gm12169	predicted gene 12169

chr11	46496148	46496303	156	157	46496227	24	10.60668	4.49345	7.40961	Adipose_R1_peak_	Distal Intergen	11	46524251	46538156	13906	1	210535	uc011xtr.1	-27948	ENSMUSG000	Gm12169	predicted gene 12169
chr11	47123134	47123332	199	200	47123195	19	7.07687	3.59476	4.08458	Adipose_R1_peak_	Intron (uc007i	11	46978783	47379302	400520	2	24052	uc007ioz.1	255970	ENSMUSG000	Sgcd	sarcoglycan, delta (dystrophin-associated glycoprotein)
chr11	47407986	47408170	185	186	47408064	40	12.96465	3.78093	9.57654	Adipose_R2_peak_	Intron (uc007i	11	46978783	47379302	400520	2	24052	uc007ioz.1	-28684	ENSMUSG000	Sgcd	sarcoglycan, delta (dystrophin-associated glycoprotein)
chr11	47466610	47466900	291	292	47466758	57	39.96741	9.66787	36.10223	Adipose_R1_peak_	Intron (uc007i	11	46978783	47379302	400520	2	24052	uc007ioz.1	-87308	ENSMUSG000	Sgcd	sarcoglycan, delta (dystrophin-associated glycoprotein)
chr11	47466657	47466836	180	181	47466755	39	12.34187	3.68871	8.98501	Adipose_R2_peak_	Intron (uc007i	11	46978783	47379302	400520	2	24052	uc007ioz.1	-87355	ENSMUSG000	Sgcd	sarcoglycan, delta (dystrophin-associated glycoprotein)
chr11	47547941	47548099	159	160	47548080	20	7.74501	3.7745	4.70467	Adipose_R1_peak_	Intron (uc007i	11	46978783	47379302	400520	2	24052	uc007ioz.1	-168639	ENSMUSG000	Sgcd	sarcoglycan, delta (dystrophin-associated glycoprotein)
chr11	47797430	47797569	140	141	47797494	18	6.42934	3.41502	3.48999	Adipose_R1_peak_	Intron (uc007i	11	46896253	47988969	1092717	2	24052	uc007ioy.1	191400	ENSMUSG000	Sgcd	sarcoglycan, delta (dystrophin-associated glycoprotein)
chr11	47847840	47848093	254	255	47847963	38	22.52591	7.00978	18.96	Adipose_R1_peak_	Intron (uc007i	11	46896253	47988969	1092717	2	24052	uc007ioy.1	140876	ENSMUSG000	Sgcd	sarcoglycan, delta (dystrophin-associated glycoprotein)
chr11	47847847	47848078	232	233	47847964	32	8.29282	3.04319	5.18352	Adipose_R2_peak_	Intron (uc007i	11	46896253	47988969	1092717	2	24052	uc007ioy.1	140891	ENSMUSG000	Sgcd	sarcoglycan, delta (dystrophin-associated glycoprotein)
chr11	47848240	47848592	353	354	47848390	232	252.974	27.81774	248.2679	Adipose_R1_peak_	Intron (uc007i	11	46896253	47988969	1092717	2	24052	uc007ioy.1	140377	ENSMUSG000	Sgcd	sarcoglycan, delta (dystrophin-associated glycoprotein)
chr11	47848246	47848576	331	332	47848382	199	180.5167	18.44355	175.8057	Adipose_R2_peak_	Intron (uc007i	11	46896253	47988969	1092717	2	24052	uc007ioy.1	140393	ENSMUSG000	Sgcd	sarcoglycan, delta (dystrophin-associated glycoprotein)
chr11	47851438	47851583	146	147	47851504	20	7.74501	3.7745	4.70467	Adipose_R1_peak_	Intron (uc007i	11	46896253	47988969	1092717	2	24052	uc007ioy.1	137386	ENSMUSG000	Sgcd	sarcoglycan, delta (dystrophin-associated glycoprotein)
chr11	47857642	47858174	533	534	47857790	36	20.67077	6.6503	17.14779	Adipose_R1_peak_	Intron (uc007i	11	46896253	47988969	1092717	2	24052	uc007ioy.1	130795	ENSMUSG000	Sgcd	sarcoglycan, delta (dystrophin-associated glycoprotein)
chr11	47857680	47857922	243	244	47857798	36	10.5378	3.41206	7.27949	Adipose_R2_peak_	Intron (uc007i	11	46896253	47988969	1092717	2	24052	uc007ioy.1	131047	ENSMUSG000	Sgcd	sarcoglycan, delta (dystrophin-associated glycoprotein)
chr11	48326846	48327020	175	176	48326942	23	9.86443	4.31371	6.70247	Adipose_R1_peak_	Distal Intergen	11	46896253	47988969	1092717	2	24052	uc007ioy.1	-337877	ENSMUSG000	Sgcd	sarcoglycan, delta (dystrophin-associated glycoprotein)
chr11	48372483	48372648	166	167	48372559	26	12.14079	4.85292	8.87838	Adipose_R1_peak_	Distal Intergen	11	46896253	47988969	1092717	2	24052	uc007ioy.1	-383514	ENSMUSG000	Sgcd	sarcoglycan, delta (dystrophin-associated glycoprotein)
chr11	48452060	48452279	220	221	48452164	32	17.10103	5.93135	13.67137	Adipose_R1_peak_	Distal Intergen	11	48800360	48806241	5882	1	14694	uc007ipa.1	-348081	ENSMUSG000	Rack1	receptor for activated C kinase 1
chr11	48452116	48452239	124	125	48452170	31	7.7623	2.95097	4.69388	Adipose_R2_peak_	Distal Intergen	11	48800360	48806241	5882	1	14694	uc007ipa.1	-348121	ENSMUSG000	Rack1	receptor for activated C kinase 1
chr11	48456618	48456807	190	191	48456723	26	12.14079	4.85292	8.87838	Adipose_R1_peak_	Distal Intergen	11	48800360	48806241	5882	1	14694	uc007ipa.1	-343553	ENSMUSG000	Rack1	receptor for activated C kinase 1
chr11	48456653	48456756	104	105	48456704	28	6.25023	2.67431	3.31626	Adipose_R2_peak_	Distal Intergen	11	48800360	48806241	5882	1	14694	uc007ipa.1	-343604	ENSMUSG000	Rack1	receptor for activated C kinase 1
chr11	48467850	48468046	197	198	48467955	29	14.55748	5.39214	11.20652	Adipose_R1_peak_	Distal Intergen	11	48800360	48806241	5882	1	14694	uc007ipa.1	-332314	ENSMUSG000	Rack1	receptor for activated C kinase 1
chr11	48471195	48471384	190	191	48471269	29	14.55748	5.39214	11.20652	Adipose_R1_peak_	Distal Intergen	11	48800360	48806241	5882	1	14694	uc007ipa.1	-328976	ENSMUSG000	Rack1	receptor for activated C kinase 1
chr11	48471657	48471877	221	222	48471772	31	16.23981	5.75161	12.83549	Adipose_R1_peak_	Distal Intergen	11	48800360	48806241	5882	1	14694	uc007ipa.1	-328483	ENSMUSG000	Rack1	receptor for activated C kinase 1
chr11	48475160	48475318	159	160	48475245	18	6.42934	3.41502	3.48999	Adipose_R1_peak_	Distal Intergen	11	48800360	48806241	5882	1	14694	uc007ipa.1	-325042	ENSMUSG000	Rack1	receptor for activated C kinase 1
chr11	48719478	48719682	205	206	48719604	25	11.36566	4.67319	8.13482	Adipose_R1_peak_	Distal Intergen	11	48800360	48806241	5882	1	14694	uc007ipa.1	-80678	ENSMUSG000	Rack1	receptor for activated C kinase 1
chr11	48720970	48721229	260	261	48721085	33	17.97508	6.11109	14.52164	Adipose_R1_peak_	Distal Intergen	11	48800360	48806241	5882	1	14694	uc007ipa.1	-79131	ENSMUSG000	Rack1	receptor for activated C kinase 1
chr11	50407591	50407754	164	165	50407655	18	6.42934	3.41502	3.48999	Adipose_R1_peak_	Intron (uc007i	11	50389303	50431111	41809	2	216724	uc007isn.1	23357	ENSMUSG000	Rufy1	RUN and FYVE domain containing 1
chr11	50412440	50412589	150	151	50412493	29	6.74062	2.76653	3.75989	Adipose_R2_peak_	Intron (uc007i	11	50389303	50431111	41809	2	216724	uc007isn.1	18522	ENSMUSG000	Rufy1	RUN and FYVE domain containing 1
chr11	50464638	50464862	225	226	50464754	45	16.22858	4.24202	12.70278	Adipose_R2_peak_	Distal Intergen	11	50389303	50431111	41809	2	216724	uc007isn.1	-33527	ENSMUSG000	Rufy1	RUN and FYVE domain containing 1
chr11	50464661	50464823	163	164	50464739	21	6.93845	3.34706	3.9919	Adipose_R1_peak_	Distal Intergen	11	50389303	50431111	41809	2	216724	uc007isn.1	-33550	ENSMUSG000	Rufy1	RUN and FYVE domain containing 1
chr11	50566773	50566979	207	208	50566940	28	6.25023	2.67431	3.31626	Adipose_R2_peak_	Distal Intergen	11	50602085	50807573	205489	1	216725	uc007iso.2	-35106	ENSMUSG000	Adamts2	a disintegrin-like and metallopeptidase (reprolysin type) with thrombospondin type 1 motif, 2
chr11	50633690	50633877	188	189	50633780	27	12.93148	5.03266	9.63827	Adipose_R1_peak_	Intron (uc007i	11	50603237	50798345	195109	1	216725	uc057ayg.1	30453	ENSMUSG000	Adamts2	a disintegrin-like and metallopeptidase (reprolysin type) with thrombospondin type 1 motif, 2
chr11	50780948	50781151	204	205	50781010	23	9.86443	4.31371	6.70247	Adipose_R1_peak_	Intron (uc007i	11	50811085	50827731	16647	2	30944	uc007isq.1	46580	ENSMUSG000	Zfp354c	zinc finger protein 354C
chr11	50828152	50828582	431	432	50828394	103	63.11229	8.80009	59.00976	Adipose_R1_peak_	Promoter (<=1	11	50811085	50827731	16647	2	30944	uc007isq.1	-421	ENSMUSG000	Zfp354c	zinc finger protein 354C
chr11	50828158	50828534	377	378	50828195	27	5.77405	2.5821	2.88921	Adipose_R2_peak_	Promoter (<=1	11	50811085	50827731	16647	2	30944	uc007isq.1	-427	ENSMUSG000	Zfp354c	zinc finger protein 354C
chr11	50945510	50945617	108	109	50945566	31	7.7623	2.95097	4.69388	Adipose_R2_peak_	Distal Intergen	11	50950806	50953632	2827	2	19127	uc007itg.1	8015	ENSMUSG000	Prop1	paired like homeodomain factor 1
chr11	50945521	50945653	133	134	50945580	18	6.42934	3.41502	3.48999	Adipose_R1_peak_	Distal Intergen	11	50950806	50953632	2827	2	19127	uc007itg.1	7979	ENSMUSG000	Prop1	paired like homeodomain factor 1
chr11	50945977	50946141	165	166	50946009	17	5.80341	3.23528	2.92378	Adipose_R1_peak_	Distal Intergen	11	50950806	50953632	2827	2	19127	uc007itg.1	7491	ENSMUSG000	Prop1	paired like homeodomain factor 1
chr11	51607393	51607590	198	199	51607508	34	9.39139	3.22762	6.20474	Adipose_R2_peak_	Promoter (<=1	11	51600100	51606881	6782	2	15384	uc007itz.1	-512	ENSMUSG000	Hnrnpab	heterogeneous nuclear ribonucleoprotein A/B
chr11	51607960	51608146	187	188	51607988	25	4.86612	2.39766	2.09018	Adipose_R2_peak_	Promoter (1-2	11	51600100	51606881	6782	2	15384	uc007itz.1	-1079	ENSMUSG000	Hnrnpab	heterogeneous nuclear ribonucleoprotein A/B
chr11	51609172	51609430	259	260	51609335	20	7.74501	3.7745	4.70467	Adipose_R1_peak_	Promoter (2-3	11	51600100	51606881	6782	2	15384	uc007itz.1	-2291	ENSMUSG000	Hnrnpab	heterogeneous nuclear ribonucleoprotein A/B
chr11	51610742	51610875	134	135	51610779	26	5.31252	2.48988	2.47999	Adipose_R2_peak_	Distal Intergen	11	51600100	51606881	6782	2	15384	uc007itz.1	-3861	ENSMUSG000	Hnrnpab	heterogeneous nuclear ribonucleoprotein A/B
chr11	51611612	51611781	170	171	51611637	16	5.20017	3.05554	2.38734	Adipose_R1_peak_	Distal Intergen	11	51600100	51606881	6782	2	15384	uc007itz.1	-4731	ENSMUSG000	Hnrnpab	heterogeneous nuclear ribonucleoprotein A/B
chr11	51614794	51614910	117	118	51614811	28	6.25023	2.67431	3.31626	Adipose_R2_peak_	Distal Intergen	11	51619773	51623714	3942	1	52530	uc007iuc.1	-4863	ENSMUSG000	Nhp2	NHP2 ribonucleoprotein
chr11	51626043	51626243	201	202	51626131	26	12.14079	4.85292	8.87838	Adipose_R1_peak_	Intron (uc007i	11	51619773	51623714	3942	1	52530	uc007iuc.1	6270	ENSMUSG000	Nhp2	NHP2 ribonucleoprotein
chr11	52002656	52002907	252	253	52002850	18	6.42934	3.41502	3.48999	Adipose_R1_peak_	Promoter (1-2	11	52004221	52027559	23339	1	213084	uc007iuw.2	-1314	ENSMUSG000	Cdkl3	cyclin-dependent kinase-like 3
chr11	52002792	52003202	411	412	52002905	33	8.83596	3.1354	5.68652	Adipose_R2_peak_	Promoter (1-2	11	52004221	52027559	23339	1	213084	uc007iuw.2	-1019	ENSMUSG000	Cdkl3	cyclin-dependent kinase-like 3
chr11	52003768	52003961	194	195	52003849	26	5.31252	2.48988	2.47999	Adipose_R2_peak_	Promoter (<=1	11	52004221	52027559	23339	1	213084	uc007iuw.2	-260	ENSMUSG000	Cdkl3	cyclin-dependent kinase-like 3
chr11	54140065	54140714	650	651	54140157	222	128.2377	8.65144	123.6367	Adipose_R2_peak_	Distal Intergen	11	54127693	54131667	3975	1	18452	uc007ixl.2	12372	ENSMUSG000	P4ha2	procollagen-proline, 2-oxoglutarate 4-dioxygenase (proline 4-hydroxylase), alpha II polypeptide
chr11	54140070	54140714	645	646	54140152	117	38.79374	4.36041	34.94353	Adipose_R1_peak_	Distal Intergen	11	54127693	54131667	3975	1	18452	uc007ixl.2	12377	ENSMUSG000	P4ha2	procollagen-proline, 2-oxoglutarate 4-dioxygenase (proline 4-hydroxylase), alpha II polypeptide
chr11	54893327	54893685	359	360	54893533	29	14.55748	5.39214	11.20652	Adipose_R1_peak_	Distal Intergen	11	54902854	54910382	7529	1	14778	uc007iyk.2	-9169	ENSMUSG000	Gpx3	glutathione peroxidase 3
chr11	54893392	54893709	318	319	54893543	53	21.92215	4.97976	18.21755	Adipose_R2_peak_	Distal Intergen	11	54902854	54910382	7529	1	14778	uc007iyk.2	-9145	ENSMUSG000	Gpx3	glutathione peroxidase 3
chr11	55025345	55025503	159	160	55025415	29	6.74062	2.76653	3.75989	Adipose_R2_peak_	Intron (uc007i	11	54978962	55033471	54510	2	11749	uc007iyr.2	7968	ENSMUSG000	Anxa6	annexin A6
chr11	55026641	55026958	318	319	55026757	25	4.86612	2.39766	2.09018	Adipose_R2_peak_	Intron (uc007i	11	54978962	55033471	54510	2	11749	uc007iyr.2	6513	ENSMUSG000	Anxa6	annexin A6
chr11	55039192	55039337	146	147	55039263	20	7.74501	3.7745	4.70467	Adipose_R1_peak_	Distal Intergen	11	54978962	55033471	54510	2	11749	uc007iyr.2	-5721	ENSMUSG000	Anxa6	annexin A6
chr11	55040928	55041045	118	119	55040946	26	5.31252	2.48988	2.47999	Adipose_R2_peak_	Distal Intergen	11	54978962	55033471	54510	2	11749	uc007iyr.2	-7457	ENSMUSG000	Anxa6	annexin A6
chr11	55297337	55297592	256	257	55297475	84	74.36649	15.27772	70.17167	Adipose_R1_peak_	Intron (uc007i	11	55250610	55312257	61648	2	245827	uc007izf.1	14665	ENSMUSG000	Fat2	FAT atypical cadherin 2
chr11	55297370	55297549	180	181	55297469	60	27.32259	5.62528	23.49644	Adipose_R2_peak_	Intron (uc007i	11	55250610	55312257	61648	2	245827	uc007izf.1	14708	ENSMUSG000	Fat2	FAT atypical cadherin 2
chr11	55396286	55396891	606	607	55396429	24	10.60668	4.49345	7.40961	Adipose_R1_peak_	Intron (uc007i	11	55394159	55420080	25922	2	20692	uc007izh.3	23189	ENSMUSG000	Sparc	secreted acidic cysteine rich glycoprotein
chr11	55396381	55396678	298	299	55396426	29	6.74062	2.76653	3.75989	Adipose_R2_peak_	Intron (uc007i	11	55394159	55420080	25922	2	20692	uc007izh.3	23402	ENSMUSG000	Sparc	secreted acidic cysteine rich glycoprotein
chr11	55417210	55417436	227	228	55417232	16	5.20017	3.05554	2.38734	Adipose_R1_peak_	Promoter (2-3	11	55394159	55420080	25922	2	20692	uc007izh.3	2644	ENSMUSG000	Sparc	secreted acidic cysteine rich glycoprotein
chr11	55421394	55421563	170	171	55421519	30	7.24477	2.85875	4.21917	Adipose_R2_peak_	Promoter (1-2	11	55394159	55420080	25922	2	20692	uc007izh.3	-1314	ENSMUSG000	Sparc	secreted acidic cysteine rich glycoprotein
chr11	55461560	55461952	393	394	55461606	29	6.74062	2.76653	3.75989	Adipose_R2_peak_	Promoter (<=1	11	55446643	55461138	14496	2	11927	uc007izj.1	-422	ENSMUSG000	Atox1	antioxidant 1 copper chaperone
chr11	55462175	55462520	346	347	55462429	21	8.43286	3.95423	5.34896	Adipose_R1_peak_	Promoter (1-2	11	55446643	55461138	14496	2	11927	uc007izj.1	-1037	ENSMUSG000	Atox1	antioxidant 1 copper chaperone
chr11	55463171	55463368	198	199	55463268	31	7.7623	2.95097	4.69388	Adipose_R2_peak_	Promoter (2-3	11	55446643	55461138	14496	2	11927	uc007izj.1	-2033	ENSMUSG000	Atox1	antioxidant 1 copper chaperone
chr11	55463583	55463900	318	319	55463690	34	9.39139	3.22762	6.20474	Adipose_R2_peak_	Promoter (2-3	11	55446643	55461138	14496	2	11927	uc007izj.1	-2445	ENSMUSG000	Atox1	antioxidant 1 copper chaperone
chr11	55465099	55465293	195	196	55465197	34	9.39139	3.22762	6.20474	Adipose_R2_peak_	Distal Intergen	11	55446643	55461138	14496	2	11927	uc007izj.1	-3961	ENSMUSG000	Atox1	antioxidant 1 copper chaperone
chr11	55833079	55833366	288	289	55833237	54	38.78658	9.88558	34.93639	Adipose_R1_peak_	Distal Intergen	11	56024990	56040987	15998	2	216749	uc007izq.1	207621	ENSMUSG000	Nmur2	neuromedin U receptor 2
chr11	55833170	55833338	169	170	55833246	40	12.96465	3.78093	9.57654	Adipose_R2_peak_	Distal Intergen	11	56024990	56040987	15998	2	216749	uc007izq.1	207649	ENSMUSG000	Nmur2	neuromedin U receptor 2
chr11	55836279	55836451	173	174	55836430	34	9.39139	3.22762	6.20474	Adipose_R2_peak_	Distal Intergen	11	56024990	56040987	15998	2	216749	uc007izq.1	204536	ENSMUSG000	Nmur2	neuromedin U receptor 2
chr11	56180452	56180750	299	300	56180597	79	68.06648	14.37903	63.91661	Adipose_R1_peak_	Distal Intergen	11	56024990	56040987	15998	2	216749	uc007izq.1	-139465	ENSMUSG000	Nmur2	neuromedin U receptor 2
chr11	56180481	56180720	240	241	56180601	46	16.90986	4.33423	13.35898	Adipose_R2_peak_	Distal Intergen	11	56024990	56040987	15998	2	216749	uc007izq.1	-139494	ENSMUSG000	Nmur2	neuromedin U receptor 2
chr11	56229161	56229443	283	284	56229297	56	40.97333	10.24506	37.09402	Adipose_R1_peak_	Distal Intergen	11	56024990	56040987	15998	2	216749	uc007izq.1	-188174	ENSMUSG000	Nmur2	neuromedin U receptor 2
chr11	56229189	56229411	223	224	56229283	64	30.56565	5.99415	26.6807	Adipose_R2_peak_	Distal Intergen	11	56024990	56040987	15998	2	216749	uc007izq.1	-188202	ENSMUSG000	Nmur2	neuromedin U receptor 2
chr11	56232535	56232929	395	396	56232783	47	31.38649	8.62742	27.65048	Adipose_R1_peak_	Distal Intergen	11	56024990	56040987	15998	2	216749	uc007izq.1	-191548	ENSMUSG000	Nmur2	neuromedin U receptor 2
chr11	56232662	56232888	227	228	56232781	49	19.00719	4.61089	15.38598	Adipose_R2_peak_	Distal Intergen	11	56024990	56040987	15998	2	216749	uc007izq.1	-191675	ENSMUSG000	Nmur2	neuromedin U receptor 2
chr11	56234118	56234233	116	117	56234174	28	6.25023	2.67431	3.31626	Adipose_R2_peak_	Distal Intergen	11	56024990	56040987	15998	2	216749	uc007izq.1	-193131	ENSMUSG000	Nmur2	neuromedin U receptor 2
chr11	56234121	56234258	138	139	56234181	19	7.07687	3.59476	4.08458	Adipose_R1_peak_	Distal Intergen	11	56024990	56040987	15998	2	216749	uc007izq.1	-193134	ENSMUSG000	Nmur2	neuromedin U receptor 2
chr11	56593422	56593692	271	272	56593598	79	68.06648	14.37903	63.91661	Adipose_R1_peak_	Distal Intergen	11	57011618	57330244	318627	1	14799	uc007izr.3	-417926	ENSMUSG000	Gria1	glutamate receptor, ionotropic, AMPA1 (alpha 1)
chr11	56593468	56593689	222	223	56593613	61	28.12311	5.7175	24.28203	Adipose_R2_peak_	Distal Intergen	11	57011618	57330244	318627	1	14799	uc007izr.3	-417929	ENSMUSG000	Gria1	glutamate receptor, ionotropic, AMPA1 (alpha 1)
chr11	56696879	56697073	195	196	56696943	21	8.43286	3.95423	5.34896	Adipose_R1_peak_	Distal Intergen	11	57011618	57330244	318627	1	14799	uc007izr.3	-314545	ENSMUSG000	Gria1	glutamate receptor, ionotropic, AMPA1 (alpha 1)
chr11	56851842	56852094	253	254	56851973	23	9.86443	4.31371	6.70247	Adipose_R1_peak_	Distal Intergen	11	57011618	57330244	318627	1	14799	uc007izr.3	-159524	ENSMUSG000	Gria1	glutamate receptor, ionotropic, AMPA1 (alpha 1)
chr11	57157870	57158097	228	229	57157934	19	7.07687	3.59476	4.08458	Adipose_R1_peak_	Intron (uc007i	11	57242749	57330244	87496	1	14799	uc011xvc.1	-84652	ENSMUSG000	Gria1	glutamate receptor, ionotropic, AMPA1 (alpha 1)
chr11	57227986	57228126	141	142	57228007	16	5.20017	3.05554	2.38734	Adipose_R1_peak_	Intron (uc007i	11	57242749	57330244	87496	1	14799	uc011xvc.1	-14623	ENSMUSG000	Gria1	glutamate receptor, ionotropic, AMPA1 (alpha 1)
chr11	57350532	57350727	196	197	57350671	23	9.86443	4.31371	6.70247	Adipose_R1_peak_	Distal Intergen	11	57242749	57330244	87496	1	14799	uc011xvc.1	107783	ENSMUSG000	Gria1	glutamate receptor, ionotropic, AMPA1 (alpha 1)
chr11	58363150	58363450	301	302	58363399	28	6.25023	2.67431	3.31626	Adipose_R2_peak_	Distal Intergen	11	58379032	58390541	11510	1	70163	uc007jbj.1	-15582	ENSMUSG000	Lypd8	LY6/PLAUR domain containing 8
chr11	58378217	58378356	140	141	58378240	16	5.20017	3.05554	2.38734	Adipose_R1_peak_	Promoter (<=1	11	58379032	58390541	11510	1	70163	uc007jbj.1	-676	ENSMUSG000	Lypd8	LY6/PLAUR domain containing 8
chr11	58381477	58381749	273	274	58381528	16	5.20017	3.05554	2.38734	Adipose_R1_peak_	Promoter (2-3	11	58379032	58390541	11510	1	70163	uc007jbj.1	2445	ENSMUSG000	Lypd8	LY6/PLAUR domain containing 8
chr11	58470710	58470918	209	210	58470820	34	18.86162	6.29083	15.38482	Adipose_R1_peak_	Distal Intergen	11	58454915	58455980	1066	2	18329	uc007jbo.2	-14730	ENSMUSG000	Olfr30	olfactory receptor 30
chr11	58470730	58470901	172	173	58470835	33	8.83596	3.1354	5.68652	Adipose_R2_peak_	Distal Intergen	11	58454915	58455980	1066	2	18329	uc007jbo.2	-14750	ENSMUSG000	Olfr30	olfactory receptor 30
chr11	58956829	58956970	142	143	58956890	22	7.22949	3.37302	4.2352	Adipose_R1_peak_	Promoter (2-3	11	58954685	58955192	508	1	319162	uc007jct.2	2144	ENSMUSG000	Hist3h2a	histone cluster 3, H2a
chr11	58957042	58957181	140	141	58957073	25	4.86612	2.39766	2.09018	Adipose_R2_peak_	Promoter (2-3	11	58954685	58955192	508	1	319162	uc007jct.2	2357	ENSMUSG000	Hist3h2a	histone cluster 3, H2a
chr11	58957976	58958092	117	118	58957982	25	4.86612	2.39766	2.09018	Adipose_R2_peak_	Intron (uc007j	11	58954685	58955192	508	1	319162	uc007jct.2	3291	ENSMUSG000	Hist3h2a	histone cluster 3, H2a
chr11	60214912	60215065	154	155	60214964	19	7.07687	3.59476	4.08458	Adipose_R1_peak_	Intron (uc007j	11	60199084	60220627	21544	2	20787	uc007jfn.2	5562	ENSMUSG000	Srebf1	sterol regulatory element binding transcription factor 1

chr11	60219100	60219246	147	148	60219117	26	5.31252	2.48988	2.47999	Adipose_R2_peak_	Promoter (1-2	11	60199084	60220627	21544	2	20787	uc007jfn.2	1381	ENSMUSG000	Srebf1	sterol regulatory element binding transcription factor 1
chr11	60220008	60220206	199	200	60220121	28	6.25023	2.67431	3.31626	Adipose_R2_peak_	Promoter (<=	11	60199084	60220627	21544	2	20787	uc007jfn.2	421	ENSMUSG000	Srebf1	sterol regulatory element binding transcription factor 1
chr11	62872161	62872363	203	204	62872228	25	11.36566	4.67319	8.13482	Adipose_R1_peak_	Intron (uc007jl	11	62879490	62895184	15695	1	67510	uc007jkh.1	-7127	ENSMUSG000	Tvp23b	trans-golgi network vesicle protein 23B
chr11	62900506	62900700	195	196	62900600	28	13.73721	5.2124	10.41463	Adipose_R1_peak_	Distal Interger	11	62879490	62895184	15695	1	67510	uc007jkh.1	21016	ENSMUSG000	Tvp23b	trans-golgi network vesicle protein 23B
chr11	62900511	62900685	175	176	62900589	35	9.95877	3.31984	6.73562	Adipose_R2_peak_	Distal Interger	11	62879490	62895184	15695	1	67510	uc007jkh.1	21021	ENSMUSG000	Tvp23b	trans-golgi network vesicle protein 23B
chr11	64538755	64538865	111	112	64538799	29	6.74062	2.76653	3.75989	Adipose_R2_peak_	Distal Interger	11	64433135	64436674	3540	2	654805	uc011xwh.1	-102081	NA	F930015N05R	RIKEN cDNA F930015N05 gene
chr11	64572752	64573031	280	281	64572880	75	48.15128	8.98568	44.18997	Adipose_R1_peak_	Distal Interger	11	64433135	64436674	3540	2	654805	uc011xwh.1	-136078	NA	F930015N05R	RIKEN cDNA F930015N05 gene
chr11	64572814	64572972	159	160	64572890	43	13.48248	3.7186	10.08088	Adipose_R2_peak_	Distal Interger	11	64433135	64436674	3540	2	654805	uc011xwh.1	-136140	NA	F930015N05R	RIKEN cDNA F930015N05 gene
chr11	66196054	66196261	208	209	66196130	31	16.23981	5.75161	12.83549	Adipose_R1_peak_	Intron (uc007jl	11	65831324	66168551	337228	2	237806	uc007jlg.1	-27503	ENSMUSG000	Dnah9	dynein, axonemal, heavy chain 9
chr11	66197244	66197440	197	198	66197326	23	8.69536	3.843	5.60874	Adipose_R1_peak_	Intron (uc007jl	11	65831324	66168551	337228	2	237806	uc007jlg.1	-28693	ENSMUSG000	Dnah9	dynein, axonemal, heavy chain 9
chr11	66197256	66197475	220	221	66197353	41	13.59774	3.87315	10.1803	Adipose_R2_peak_	Intron (uc007jl	11	65831324	66168551	337228	2	237806	uc007jlg.1	-28705	ENSMUSG000	Dnah9	dynein, axonemal, heavy chain 9
chr11	66420936	66421292	357	358	66421117	93	74.15605	13.03823	69.96524	Adipose_R1_peak_	Intron (uc007jl	11	66211725	66526126	314402	2	380702	uc007jlm.2	104834	ENSMUSG000	Shisa6	shisa family member 6
chr11	66420937	66421193	257	258	66421111	74	39.12209	6.91633	35.10124	Adipose_R2_peak_	Intron (uc007jl	11	66211725	66526126	314402	2	380702	uc007jlm.2	104933	ENSMUSG000	Shisa6	shisa family member 6
chr11	66465307	66465528	222	223	66465419	36	20.67077	6.6503	17.14779	Adipose_R1_peak_	Intron (uc007jl	11	66211725	66526126	314402	2	380702	uc007jlm.2	60598	ENSMUSG000	Shisa6	shisa family member 6
chr11	66465315	66465530	216	217	66465427	48	18.29934	4.51867	14.70076	Adipose_R2_peak_	Intron (uc007jl	11	66211725	66526126	314402	2	380702	uc007jlm.2	60596	ENSMUSG000	Shisa6	shisa family member 6
chr11	66466426	66466700	275	276	66466577	59	44.30963	10.78427	40.38949	Adipose_R1_peak_	Intron (uc007jl	11	66211725	66526126	314402	2	380702	uc007jlm.2	59426	ENSMUSG000	Shisa6	shisa family member 6
chr11	66466490	66466692	203	204	66466561	37	11.12817	3.50427	7.83613	Adipose_R2_peak_	Intron (uc007jl	11	66211725	66526126	314402	2	380702	uc007jlm.2	59434	ENSMUSG000	Shisa6	shisa family member 6
chr11	66475032	66475339	308	309	66475186	72	59.47482	13.12087	55.39848	Adipose_R1_peak_	Intron (uc007jl	11	66211725	66526126	314402	2	380702	uc007jlm.2	50787	ENSMUSG000	Shisa6	shisa family member 6
chr11	66475066	66475312	247	248	66475183	60	27.32259	5.62528	23.49644	Adipose_R2_peak_	Intron (uc007jl	11	66211725	66526126	314402	2	380702	uc007jlm.2	50814	ENSMUSG000	Shisa6	shisa family member 6
chr11	66475936	66476072	137	138	66475999	17	5.80341	3.23528	2.92378	Adipose_R1_peak_	Intron (uc007jl	11	66211725	66526126	314402	2	380702	uc007jlm.2	50054	ENSMUSG000	Shisa6	shisa family member 6
chr11	66629142	66629432	291	292	66629285	55	39.87613	10.06532	36.01168	Adipose_R1_peak_	Distal Interger	11	66211725	66526126	314402	2	380702	uc007jlm.2	-103016	ENSMUSG000	Shisa6	shisa family member 6
chr11	66629180	66629412	233	234	66629295	44	15.55653	4.1498	12.05625	Adipose_R2_peak_	Distal Interger	11	66211725	66526126	314402	2	380702	uc007jlm.2	-103054	ENSMUSG000	Shisa6	shisa family member 6
chr11	66710679	66710817	139	140	66710742	19	7.07687	3.59476	4.08458	Adipose_R1_peak_	Distal Interger	11	66211725	66526126	314402	2	380702	uc007jlm.2	-184553	ENSMUSG000	Shisa6	shisa family member 6
chr11	68502193	68502437	245	246	68502337	29	14.55748	5.39214	11.20652	Adipose_R1_peak_	Promoter (<=	11	68503019	68533219	30201	1	104709	uc007jnq.1	-582	ENSMUSG000	Pik3r6	phosphoinositide-3-kinase, regulatory subunit 6
chr11	68502290	68502437	148	149	68502357	34	9.39139	3.22762	6.20474	Adipose_R2_peak_	Promoter (<=	11	68503019	68533219	30201	1	104709	uc007jnq.1	-582	ENSMUSG000	Pik3r6	phosphoinositide-3-kinase, regulatory subunit 6
chr11	69060813	69061170	358	359	69060903	26	5.31252	2.48988	2.47999	Adipose_R2_peak_	Promoter (1-2	11	69059775	69061576	1802	1	71923	uc007jpb.1	1038	ENSMUSG000	Borcs6	BLOC-1 related complex subunit 6
chr11	69062582	69062714	133	134	69062606	16	5.20017	3.05554	2.38734	Adipose_R1_peak_	Promoter (2-3	11	69059775	69061576	1802	1	71923	uc007jpb.1	2807	ENSMUSG000	Borcs6	BLOC-1 related complex subunit 6
chr11	69092393	69092716	324	325	69092547	31	7.7623	2.95097	4.69388	Adipose_R2_peak_	Promoter (2-3	11	69095254	69106458	11205	1	18626	uc007jpf.1	-2538	ENSMUSG000	Per1	period circadian clock 1
chr11	69093452	69093590	139	140	69093486	16	5.20017	3.05554	2.38734	Adipose_R1_peak_	Promoter (1-2	11	69095254	69106458	11205	1	18626	uc007jpf.1	-1664	ENSMUSG000	Per1	period circadian clock 1
chr11	69094552	69094727	176	177	69094608	28	6.19963	2.65992	3.29848	Adipose_R2_peak_	Promoter (<=	11	69095254	69106458	11205	1	18626	uc007jpf.1	-527	ENSMUSG000	Per1	period circadian clock 1
chr11	69095390	69095881	492	493	69095514	26	10.16564	4.08858	7.00117	Adipose_R1_peak_	Promoter (<=	11	69095254	69106458	11205	1	18626	uc007jpf.1	136	ENSMUSG000	Per1	period circadian clock 1
chr11	69096194	69096878	685	686	69096539	33	17.97508	6.11109	14.52164	Adipose_R1_peak_	Promoter (<=	11	69095254	69106458	11205	1	18626	uc007jpf.1	940	ENSMUSG000	Per1	period circadian clock 1
chr11	69097291	69097610	320	321	69097395	20	7.36052	3.61195	4.36469	Adipose_R1_peak_	Promoter (1-2	11	69098956	69109957	11002	1	18626	uc007jpg.2	-1346	ENSMUSG000	Per1	period circadian clock 1
chr11	69097863	69098087	225	226	69098059	31	6.56254	2.63081	3.61679	Adipose_R2_peak_	Promoter (<=	11	69098956	69109957	11002	1	18626	uc007jpg.2	-869	ENSMUSG000	Per1	period circadian clock 1
chr11	69098967	69099139	173	174	69099061	29	6.74062	2.76653	3.75989	Adipose_R2_peak_	Promoter (<=	11	69098956	69109957	11002	1	18626	uc007jpg.2	11	ENSMUSG000	Per1	period circadian clock 1
chr11	69099193	69099408	216	217	69099211	20	7.20926	3.54892	4.21524	Adipose_R1_peak_	Promoter (<=	11	69098956	69109957	11002	1	18626	uc007jpg.2	237	ENSMUSG000	Per1	period circadian clock 1
chr11	69099598	69099963	366	367	69099635	27	5.77405	2.5821	2.88921	Adipose_R2_peak_	Promoter (<=	11	69100158	69109957	9800	1	18626	uc007jph.2	-195	ENSMUSG000	Per1	period circadian clock 1
chr11	69101362	69101500	139	140	69101408	28	6.25023	2.67431	3.31626	Adipose_R2_peak_	Promoter (<=	11	69100509	69109957	9449	1	18626	uc011xwu.1	853	ENSMUSG000	Per1	period circadian clock 1
chr11	69399360	69399460	101	102	69399412	30	7.24477	2.85875	4.21917	Adipose_R2_peak_	Promoter (1-2	11	69396516	69398234	1719	2	67020	uc007jqc.2	-1126	ENSMUSG000	Tmem88	transmembrane protein 88
chr11	69400740	69400908	169	170	69400787	28	6.25023	2.67431	3.31626	Adipose_R2_peak_	Promoter (2-3	11	69396516	69398234	1719	2	67020	uc007jqc.2	-2506	ENSMUSG000	Tmem88	transmembrane protein 88
chr11	69401093	69401348	256	257	69401301	19	6.04493	3.16102	3.16151	Adipose_R1_peak_	Promoter (2-3	11	69396516	69398234	1719	2	67020	uc007jqc.2	-2859	ENSMUSG000	Tmem88	transmembrane protein 88
chr11	69401730	69401836	107	108	69401762	26	5.31252	2.48988	2.47999	Adipose_R2_peak_	Exon (uc007jq	11	69396516	69398234	1719	2	67020	uc007jqc.2	-3496	ENSMUSG000	Tmem88	transmembrane protein 88
chr11	69402286	69402553	268	269	69402421	19	6.68555	3.42739	3.7425	Adipose_R1_peak_	Intron (uc007j	11	69398518	69406173	7656	2	216850	uc007jqd.1	3620	ENSMUSG000	Kdm6b	KDM1 lysine (K)-specific demethylase 6B
chr11	69402319	69402630	312	313	69402418	29	6.74062	2.76653	3.75989	Adipose_R2_peak_	Intron (uc007j	11	69398518	69406173	7656	2	216850	uc007jqd.1	3543	ENSMUSG000	Kdm6b	KDM1 lysine (K)-specific demethylase 6B
chr11	69403676	69404005	330	331	69403869	25	4.86612	2.39766	2.09018	Adipose_R2_peak_	Promoter (2-3	11	69398518	69406173	7656	2	216850	uc007jqd.1	2168	ENSMUSG000	Kdm6b	KDM1 lysine (K)-specific demethylase 6B
chr11	69404343	69404510	168	169	69404370	27	5.77405	2.5821	2.88921	Adipose_R2_peak_	Promoter (1-2	11	69398518	69406173	7656	2	216850	uc007jqd.1	1663	ENSMUSG000	Kdm6b	KDM1 lysine (K)-specific demethylase 6B
chr11	69404426	69404622	197	198	69404497	18	6.42934	3.41502	3.48999	Adipose_R1_peak_	Promoter (1-2	11	69398518	69406173	7656	2	216850	uc007jqd.1	1551	ENSMUSG000	Kdm6b	KDM1 lysine (K)-specific demethylase 6B
chr11	69404951	69405082	132	133	69405019	28	6.25023	2.67431	3.31626	Adipose_R2_peak_	Promoter (1-2	11	69398518	69406173	7656	2	216850	uc007jqd.1	1091	ENSMUSG000	Kdm6b	KDM1 lysine (K)-specific demethylase 6B
chr11	69404977	69405118	142	143	69405001	16	5.20017	3.05554	2.38734	Adipose_R1_peak_	Promoter (1-2	11	69398518	69406173	7656	2	216850	uc007jqd.1	1055	ENSMUSG000	Kdm6b	KDM1 lysine (K)-specific demethylase 6B
chr11	69407284	69407404	121	122	69407337	30	7.24477	2.85875	4.21917	Adipose_R2_peak_	Promoter (1-2	11	69398518	69406173	7656	2	216850	uc007jqd.1	-1111	ENSMUSG000	Kdm6b	KDM1 lysine (K)-specific demethylase 6B
chr11	69409030	69409250	221	222	69409132	34	9.39139	3.22762	6.20474	Adipose_R2_peak_	Promoter (2-3	11	69398518	69406173	7656	2	216850	uc007jqd.1	-2857	ENSMUSG000	Kdm6b	KDM1 lysine (K)-specific demethylase 6B
chr11	69410014	69410659	646	647	69410059	29	6.74062	2.76653	3.75989	Adipose_R2_peak_	Intron (uc007j	11	69398518	69413673	15156	2	216850	uc007jqe.1	3014	ENSMUSG000	Kdm6b	KDM1 lysine (K)-specific demethylase 6B
chr11	69410249	69410522	274	275	69410290	17	5.38306	3.05055	2.56688	Adipose_R1_peak_	Intron (uc007j	11	69398518	69413673	15156	2	216850	uc007jqe.1	3151	ENSMUSG000	Kdm6b	KDM1 lysine (K)-specific demethylase 6B
chr11	69411435	69411649	215	216	69411552	34	9.39139	3.22762	6.20474	Adipose_R2_peak_	Promoter (2-3	11	69398518	69413673	15156	2	216850	uc007jqe.1	2024	ENSMUSG000	Kdm6b	KDM1 lysine (K)-specific demethylase 6B
chr11	69411883	69412134	252	253	69412033	26	10.49947	4.21281	7.33229	Adipose_R1_peak_	Promoter (1-2	11	69398518	69413673	15156	2	216850	uc007jqe.1	1539	ENSMUSG000	Kdm6b	KDM1 lysine (K)-specific demethylase 6B
chr11	69411973	69412105	133	134	69412039	29	6.74062	2.76653	3.75989	Adipose_R2_peak_	Promoter (1-2	11	69398518	69413673	15156	2	216850	uc007jqe.1	1568	ENSMUSG000	Kdm6b	KDM1 lysine (K)-specific demethylase 6B
chr11	69412436	69413740	1305	1306	69412561	30	7.24477	2.85875	4.21917	Adipose_R2_peak_	Promoter (<=	11	69398518	69413673	15156	2	216850	uc007jqe.1	0	ENSMUSG000	Kdm6b	KDM1 lysine (K)-specific demethylase 6B
chr11	69413578	69413849	272	273	69413684	24	9.57494	4.07979	6.44676	Adipose_R1_peak_	Promoter (<=	11	69398518	69413673	15156	2	216850	uc007jqe.1	0	ENSMUSG000	Kdm6b	KDM1 lysine (K)-specific demethylase 6B
chr11	69414069	69414228	160	161	69414217	20	6.97733	3.45326	4.03035	Adipose_R1_peak_	Promoter (<=	11	69398518	69413673	15156	2	216850	uc007jqe.1	-396	ENSMUSG000	Kdm6b	KDM1 lysine (K)-specific demethylase 6B
chr11	69414700	69414933	234	235	69414851	35	9.95877	3.31984	6.73562	Adipose_R2_peak_	Promoter (1-2	11	69398518	69413673	15156	2	216850	uc007jqe.1	-1027	ENSMUSG000	Kdm6b	KDM1 lysine (K)-specific demethylase 6B
chr11	69414866	69415199	334	335	69414987	21	7.72664	3.66132	4.70467	Adipose_R1_peak_	Promoter (1-2	11	69398518	69413673	15156	2	216850	uc007jqe.1	-1193	ENSMUSG000	Kdm6b	KDM1 lysine (K)-specific demethylase 6B
chr11	69416907	69417200	294	295	69416998	33	8.83596	3.1354	5.68652	Adipose_R2_peak_	Distal Interger	11	69398518	69413673	15156	2	216850	uc007jqe.1	-3234	ENSMUSG000	Kdm6b	KDM1 lysine (K)-specific demethylase 6B
chr11	69418344	69418551	208	209	69418450	32	8.29282	3.04319	5.18352	Adipose_R2_peak_	Downstream (	11	69398518	69413673	15156	2	216850	uc007jqe.1	-4671	ENSMUSG000	Kdm6b	KDM1 lysine (K)-specific demethylase 6B
chr11	69419159	69419343	185	186	69419274	29	6.74062	2.76653	3.75989	Adipose_R2_peak_	Downstream (	11	69398518	69413673	15156	2	216850	uc007jqe.1	-5486	ENSMUSG000	Kdm6b	KDM1 lysine (K)-specific demethylase 6B
chr11	69432439	69432620	182	183	69432613	22	9.13959	4.13397	6.01526	Adipose_R1_peak_	Intron (uc007j	11	69398518	69413673	15156	2	216850	uc007jqe.1	-18766	ENSMUSG000	Kdm6b	KDM1 lysine (K)-specific demethylase 6B
chr11	69665278	69665426	149	150	69665358	20	7.20926	3.54892	4.21524	Adipose_R1_peak_	Promoter (<=	11	69664213	69666170	1958	2	12514	uc007jqy.2	744	ENSMUSG000	Cd68	CD68 antigen
chr11	69665790	69665887	98	99	69665859	26	5.31252	2.48988	2.47999	Adipose_R2_peak_	Promoter (<=	11	69664213	69666170	1958	2	12514	uc007jqy.2	283	ENSMUSG000	Cd68	CD68 antigen
chr11	69666162	69666420	259	260	69666218	18	6.42934	3.41502	3.48999	Adipose_R1_peak_	Promoter (<=	11	69664213	69666170	1958	2	12514	uc007jqy.2	0	ENSMUSG000	Cd68	CD68 antigen
chr11	69666615	69666770	156	157	69666726	25	4.86612	2.39766	2.09018	Adipose_R2_peak_	Promoter (<=	11	69664213	69666170	1958	2	12514	uc007jqy.2	-445	ENSMUSG000	Cd68	CD68 antigen
chr11	69667850	69668113	264	265	69667971	33	8.83596	3.1354	5.68652	Adipose_R2_peak_	Promoter (1-2	11	69664213	69666170	1958	2	12514	uc007jqy.2	-1680	ENSMUSG000	Cd68	CD68 antigen
chr11	69668282	69668687	406	407	69668366	31	7.7623	2.95097	4.69388	Adipose_R2_peak_	Promoter (2-3	11	69664213	69666170	1958	2	12514	uc007jqy.2	-2112	ENSMUSG000	Cd68	CD68 antigen
chr11	69669126	69669381	256	257	69669149	16	5.09452	3.00791	2.34798	Adipose_R1_peak_	Promoter (2-3	11	69664213	69666170	1958	2	12514	uc007jqy.2	-2956	ENSMUSG000	Cd68	CD68 antigen
chr11	69669640	69669759	120	121	69669646	27	5.77405	2.5821	2.88921	Adipose_R2_peak_	Promoter (2-3	11	69666936	69672423	5488	2	13681	uc007jqz.2	2664	ENSMUSG000	Eif4a1	eukaryotic translation initiation factor 4A1
chr11	69670602	69671127	526	527	69670790	30	7.24477	2.85875	4.21917	Adipose_R2_peak_	Promoter (1-2	11	69666936	69672423	5488	2	13681	uc007jqz.2	1296	ENSMUSG000	Eif4a1	eukaryotic translation initiation factor 4A1
chr11	69670703	69670925	223	224	69670824	25	11.36566	4.67319	8.13482	Adipose_R1_peak_	Promoter (1-2	11	69666936	69672423	5488	2	13681	uc007jqz.2	1498	ENSMUSG000	Eif4a1	eukaryotic translation initiation factor 4A1
chr11	69672205	69672359	155	156	69672313	21	8.43286	3.95423	5.34896	Adipose_R1_peak_	Promoter (<=	11	69666936	69672423	5488	2	13681	uc007jqz.2	64	ENSMUSG000	Eif4a1	eukaryotic translation initiation factor 4A1
chr11	69674698	69674990	293	294	69674827	35	9.95877	3.31984	6.73562	Adipose_R2_peak_	Promoter (2-3	11	69666936	69672423	5488	2	13681	uc007jqz.2	-2275	ENSMUSG000	Eif4a1	eukaryotic translation initiation factor 4A1
chr11	69678316	69678421	106	107	69678403	27	5.77405	2.5821	2.88921	Adipose_R2_peak_	Intron (uc007j	11	69673110	69681970	8861	2	80886	uc007jrb.2	3549	ENSMUSG000	Senp3	SUMO/sentrin specific peptidase 3
chr11	69679832	69679936	105	106	69679838	25	4.86612	2.39766	2.09018	Adipose_R2_peak_	Promoter (2-3	11	69673110	69681970	8861	2	80886	uc007jrb.2	2034	ENSMUSG000	Senp3	SUMO/sentrin specific peptidase 3
chr11	69682543	69682796	254	255	69682699	18	6.42934	3.41502	3.48999	Adipose_R1_peak_	Promoter (<=	11	69673110	69682084	8975	2	80886	uc007jrc.2	-459	ENSMUSG000	Senp3	SUMO/sentrin specific peptidase 3
chr11	69682683	69682893	211	212	69682835	33	8.83596	3.1354	5.68652	Adipose_R2_peak_	Promoter (<=	11	69673110	69682084	8975	2	80886	uc007jrc.2	-599	ENSMUSG000	Senp3	SUMO/sentrin specific peptidase 3
chr11	69839105	69839280	176	177	69839146	16	5.20017	3.05554	2.38734	Adipose_R1_peak_	Promoter (<=	11	69838525	69839558	1034	1	69186	uc007jry.1	580	ENSMUSG000	Tmem256	transmembrane protein 256
chr11	69842376	69842475	100	101	69842443	29	6.74062	2.76653	3.75989	Adipose_R2_peak_	Distal Interger	11	69838525	69839558	1034	1	69186	uc007jry.1	3851	ENSMUSG000	Tmem256	transmembrane protein 256
chr11	69908010	69908124	115	116	69908048	25	4.86612	2.39766	2.09018	Adipose_R2_peak_	Exon (uc011xx	11	69914050	69915390	1341	1	56310	uc007jso.1	-5926	ENSMUSG000	Gps2	G protein pathway suppressor 2
chr11	69909358	69909539	182	183	69909429	29	6.74062	2.76653	3.75989	Adipose_R2_peak_	Exon (uc011xx	11	69914050	69915390	1341	1	56310	uc007jso.1	-4511	ENSMUSG000	Gps2	G protein pathway suppressor 2
chr11	69910912	69911015	104	105	69910918	25	4.86612	2.39766	2.09018	Adipose_R2_peak_	Exon (uc011xx	11	69914050	69915390	1341	1	56310	uc007jso.1	-3035	ENSMUSG000	Gps2	G protein pathway suppressor 2
chr11	69914953	69915169	217	218	69915112	30	7.24477	2.85875	4.21917	Adipose_R2_peak_	Promoter (<=	11	69914192	69916591	2400	1	56310	uc007jsp.1	761	ENSMUSG000	Gps2	G protein pathway suppressor 2
chr11	69915214	69915514	301	302	69915341	16	5.0436	2.98505	2.29833	Adipose_R1_peak_	Promoter (1-2	11	69914192	69916591	2400	1	56310	uc007jsp.1	1022	ENSMUSG000	Gps2	G protein pathway suppressor 2
chr11	69915486	69915958	473	474	69915622	31	7.7623	2.95097	4.69388	Adipose_R2_peak_	Promoter (1-2	11	69914192	69916591	2400	1	56310	uc007jsp.1	1294	ENSMUSG000	Gps2	G protein pathway suppressor 2
chr11	69916145	69916343	199	200	69916310	29	6.74062	2.76653	3.75989	Adipose_R2_peak_	Promoter (1-2	11	69914192	69916591	2400	1	56310	uc007jsp.1	1953	ENSMUSG000	Gps2	G protein pathway suppressor 2
chr11	69981622	69981772	151	152	69981693	27	5.77405	2.5821	2.88921	Adipose_R2_peak_	Promoter (<=	11	69968226	69981235	13010	2	54351	uc007jte.3	-387	ENSMUSG000	Elp5	elongator acetyltransferase complex subunit 5
chr11	69981959	69982372	414	415	69982006	28	6.25023	2.67431	3.31626	Adipose_R2_peak_	Promoter (<=	11	69968226	69981235	13010	2	54351	uc007jte.3	-724	ENSMUSG000	Elp5	elongator acetyltransferase complex subunit 5
chr11	69983809	69984056	248	249	69983938	28	6.25023	2.67431	3.31626	Adipose_R2_peak_	Promoter (2-3	11	69968226	69981235	13010	2	54351	uc007jte.3	-2574	ENSMUSG000	Elp5	elongator acetyltransferase complex subunit 5
chr11	69984972	69985131	160	161	69985023	27	5.77405	2.5821	2.88921	Adipose_R2_peak_	Intron (uc007j	11	69968226	69981235	13010	2	54351	uc007jte.3	-3737	ENSMUSG000	Elp5	elongator acetyltransferase complex subunit 5

chr11	69985476	69985958	483	484	69985604	34	9.39139	3.22762	6.20474	Adipose_R2_peak_	Intron (uc007j	11	69968226	69981235	13010	2	54351	uc007jte.3	-4241	ENSMUSG000	Elp5	elongator acetyltransferase complex subunit 5
chr11	69985857	69986000	144	145	69985915	18	6.42934	3.41502	3.48999	Adipose_R1_peak_	Intron (uc007j	11	69968226	69981235	13010	2	54351	uc007jte.3	-4622	ENSMUSG000	Elp5	elongator acetyltransferase complex subunit 5
chr11	69994374	69994699	326	327	69994560	30	7.24477	2.85875	4.21917	Adipose_R2_peak_	Promoter (1-2	11	69995766	70000011	4246	1	78246	uc007jth.3	-1067	ENSMUSG000	Phf23	PHD finger protein 23
chr11	69994797	69994935	139	140	69994828	28	6.25023	2.67431	3.31626	Adipose_R2_peak_	Promoter (<=	11	69995766	70000011	4246	1	78246	uc007jth.3	-831	ENSMUSG000	Phf23	PHD finger protein 23
chr11	69996220	69996457	238	239	69996303	29	6.74062	2.76653	3.75989	Adipose_R2_peak_	Promoter (<=	11	69995766	70000011	4246	1	78246	uc007jth.3	454	ENSMUSG000	Phf23	PHD finger protein 23
chr11	69998209	69998462	254	255	69998282	18	6.42934	3.41502	3.48999	Adipose_R1_peak_	Promoter (<=	11	69997462	70000011	2550	1	78246	uc007jtk.2	747	ENSMUSG000	Phf23	PHD finger protein 23
chr11	70236844	70236939	96	97	70236888	25	4.86612	2.39766	2.09018	Adipose_R2_peak_	Promoter (1-2	11	70235204	70238470	3267	2	104457	uc007jud.2	1531	ENSMUSG000	0610010K14Ri	RIKEN cDNA 0610010K14 gene
chr11	70238413	70238819	407	408	70238490	19	7.07687	3.59476	4.08458	Adipose_R1_peak_	Promoter (<=	11	70235204	70238470	3267	2	104457	uc007jud.2	0	ENSMUSG000	0610010K14Ri	RIKEN cDNA 0610010K14 gene
chr11	70239133	70239404	272	273	70239291	34	9.39139	3.22762	6.20474	Adipose_R2_peak_	Promoter (<=	11	70235204	70239830	4627	2	104457	uc007juf.2	426	ENSMUSG000	0610010K14Ri	RIKEN cDNA 0610010K14 gene
chr11	70239223	70239542	320	321	70239404	19	7.07687	3.59476	4.08458	Adipose_R1_peak_	Promoter (<=	11	70235204	70239830	4627	2	104457	uc007juf.2	288	ENSMUSG000	0610010K14Ri	RIKEN cDNA 0610010K14 gene
chr11	70647293	70647475	183	184	70647389	30	7.24477	2.85875	4.21917	Adipose_R2_peak_	Promoter (<=	11	70647287	70650334	3048	1	70510	uc007jvs.2	6	ENSMUSG000	Rnf167	ring finger protein 167
chr11	70647690	70647842	153	154	70647734	28	6.25023	2.67431	3.31626	Adipose_R2_peak_	Promoter (<=	11	70647287	70650334	3048	1	70510	uc007jvs.2	403	ENSMUSG000	Rnf167	ring finger protein 167
chr11	70648081	70648655	575	576	70648180	32	8.29282	3.04319	5.18352	Adipose_R2_peak_	Promoter (<=	11	70647287	70650334	3048	1	70510	uc007jvs.2	794	ENSMUSG000	Rnf167	ring finger protein 167
chr11	70648278	70648444	167	168	70648339	19	7.07687	3.59476	4.08458	Adipose_R1_peak_	Promoter (<=	11	70647287	70650334	3048	1	70510	uc007jvs.2	991	ENSMUSG000	Rnf167	ring finger protein 167
chr11	70648965	70649474	510	511	70649080	32	8.29282	3.04319	5.18352	Adipose_R2_peak_	Promoter (1-2	11	70647287	70650334	3048	1	70510	uc007jvs.2	1678	ENSMUSG000	Rnf167	ring finger protein 167
chr11	70649061	70649420	360	361	70649159	22	9.13959	4.13397	6.01526	Adipose_R1_peak_	Promoter (1-2	11	70647287	70650334	3048	1	70510	uc007jvs.2	1774	ENSMUSG000	Rnf167	ring finger protein 167
chr11	70650193	70650496	304	305	70650255	16	5.20017	3.05554	2.38734	Adipose_R1_peak_	Promoter (2-3	11	70647287	70650334	3048	1	70510	uc007jvs.2	2906	ENSMUSG000	Rnf167	ring finger protein 167
chr11	70650346	70650591	246	247	70650516	37	11.12817	3.50427	7.83613	Adipose_R2_peak_	Intron (uc007j	11	70647287	70650334	3048	1	70510	uc007jvs.2	3059	ENSMUSG000	Rnf167	ring finger protein 167
chr11	70660008	70660174	167	168	70660072	23	9.86443	4.31371	6.70247	Adipose_R1_peak_	Promoter (2-3	11	70657176	70662513	5338	1	13808	uc007jvx.3	2832	ENSMUSG000	Eno3	enolase 3, beta muscle
chr11	70660133	70660250	118	119	70660177	27	5.77405	2.5821	2.88921	Adipose_R2_peak_	Promoter (2-3	11	70657176	70662513	5338	1	13808	uc007jvx.3	2957	ENSMUSG000	Eno3	enolase 3, beta muscle
chr11	70660581	70660738	158	159	70660691	28	6.25023	2.67431	3.31626	Adipose_R2_peak_	Exon (uc007jv	11	70657176	70662513	5338	1	13808	uc007jvx.3	3405	ENSMUSG000	Eno3	enolase 3, beta muscle
chr11	70661113	70661286	174	175	70661249	27	5.77405	2.5821	2.88921	Adipose_R2_peak_	Exon (uc007jv	11	70657176	70662513	5338	1	13808	uc007jvx.3	3937	ENSMUSG000	Eno3	enolase 3, beta muscle
chr11	70662805	70663262	458	459	70663000	27	5.77405	2.5821	2.88921	Adipose_R2_peak_	Downstream (	11	70657176	70662513	5338	1	13808	uc007jvx.3	5629	ENSMUSG000	Eno3	enolase 3, beta muscle
chr11	70663537	70663667	131	132	70663643	20	7.74501	3.7745	4.70467	Adipose_R1_peak_	Downstream (	11	70663769	70669416	5648	2	216873	uc007jvz.2	5749	ENSMUSG000	Spag7	sperm associated antigen 7
chr11	70665397	70665575	179	180	70665481	26	5.31252	2.48988	2.47999	Adipose_R2_peak_	Intron (uc007j	11	70663769	70669416	5648	2	216873	uc007jvz.2	3841	ENSMUSG000	Spag7	sperm associated antigen 7
chr11	70666674	70666777	104	105	70666706	25	4.86612	2.39766	2.09018	Adipose_R2_peak_	Promoter (2-3	11	70663769	70669416	5648	2	216873	uc007jvz.2	2639	ENSMUSG000	Spag7	sperm associated antigen 7
chr11	70979414	70979544	131	132	70979542	35	14.46153	4.61404	11.13475	Adipose_R1_peak_	Intron (uc007j	11	70977846	70983026	5181	2	12261	uc007jxf.1	3482	ENSMUSG000	C1qbp	complement component 1, q subcomponent binding protein
chr11	70979447	70979544	98	99	70979532	32	7.45866	2.82165	4.42524	Adipose_R2_peak_	Intron (uc007j	11	70977846	70983026	5181	2	12261	uc007jxf.1	3482	ENSMUSG000	C1qbp	complement component 1, q subcomponent binding protein
chr11	73016312	73016601	290	291	73016402	28	6.25023	2.67431	3.31626	Adipose_R2_peak_	Promoter (2-3	11	73019008	73042065	23058	1	55984	uc007jzt.1	-2407	ENSMUSG000	Camkk1	calcium/calmodulin-dependent protein kinase kinase 1, alpha
chr11	73016585	73016732	148	149	73016608	18	6.42934	3.41502	3.48999	Adipose_R1_peak_	Promoter (2-3	11	73019008	73042065	23058	1	55984	uc007jzt.1	-2276	ENSMUSG000	Camkk1	calcium/calmodulin-dependent protein kinase kinase 1, alpha
chr11	73017865	73018010	146	147	73017976	16	5.20017	3.05554	2.38734	Adipose_R1_peak_	Promoter (<=	11	73019008	73042065	23058	1	55984	uc007jzt.1	-998	ENSMUSG000	Camkk1	calcium/calmodulin-dependent protein kinase kinase 1, alpha
chr11	73368012	73368203	192	193	73368093	44	15.55653	4.1498	12.05625	Adipose_R2_peak_	Distal Intergen	11	73371246	73375704	4459	1	258924	uc007kao.2	-3043	ENSMUSG000	Olfr376	olfactory receptor 376
chr11	73368015	73368215	201	202	73368098	47	31.38649	8.62742	27.65048	Adipose_R1_peak_	Distal Intergen	11	73371246	73375704	4459	1	258924	uc007kao.2	-3031	ENSMUSG000	Olfr376	olfactory receptor 376
chr11	73729411	73729619	209	210	73729496	30	15.39182	5.57188	12.01394	Adipose_R1_peak_	Distal Intergen	11	73688778	73713808	25031	1	76390	uc007kaz.2	40633	ENSMUSG000	Zfp735	zinc finger protein 735
chr11	73732673	73732890	218	219	73732786	32	17.10103	5.93135	13.67137	Adipose_R1_peak_	Distal Intergen	11	73688778	73713808	25031	1	76390	uc007kaz.2	43895	ENSMUSG000	Zfp735	zinc finger protein 735
chr11	73732737	73732848	112	113	73732792	26	5.31252	2.48988	2.47999	Adipose_R2_peak_	Distal Intergen	11	73688778	73713808	25031	1	76390	uc007kaz.2	43959	ENSMUSG000	Zfp735	zinc finger protein 735
chr11	73860918	73861217	300	301	73861075	63	48.85777	11.50323	44.88581	Adipose_R1_peak_	Distal Intergen	11	73847185	73848123	939	2	259010	uc007kbd.1	-12795	ENSMUSG000	Olfr393	olfactory receptor 393
chr11	73860925	73861215	291	292	73861086	74	39.12209	6.91633	35.10124	Adipose_R2_peak_	Distal Intergen	11	73847185	73848123	939	2	259010	uc007kbd.1	-12802	ENSMUSG000	Olfr393	olfactory receptor 393
chr11	74302566	74302842	277	278	74302706	55	39.87613	10.06532	36.01168	Adipose_R1_peak_	Distal Intergen	11	74283809	74289646	5838	1	18359	uc007kbr.2	18757	ENSMUSG000	Olfr59	olfactory receptor 59
chr11	74302590	74302835	246	247	74302696	67	33.06755	6.27081	29.1399	Adipose_R2_peak_	Distal Intergen	11	74283809	74289646	5838	1	18359	uc007kbr.2	18781	ENSMUSG000	Olfr59	olfactory receptor 59
chr11	74830574	74830726	153	154	74830619	30	7.24477	2.85875	4.21917	Adipose_R2_peak_	Promoter (<=	11	74830924	74845725	14802	1	17428	uc011xzb.1	-198	ENSMUSG000	Mnt	max binding protein
chr11	74830602	74830759	158	159	74830665	19	7.07687	3.59476	4.08458	Adipose_R1_peak_	Promoter (<=	11	74830924	74845725	14802	1	17428	uc011xzb.1	-165	ENSMUSG000	Mnt	max binding protein
chr11	74832527	74832844	318	319	74832617	29	6.74062	2.76653	3.75989	Adipose_R2_peak_	Promoter (1-2	11	74830924	74845725	14802	1	17428	uc011xzb.1	1603	ENSMUSG000	Mnt	max binding protein
chr11	74833079	74833269	191	192	74833140	32	8.29282	3.04319	5.18352	Adipose_R2_peak_	Promoter (2-3	11	74830924	74845725	14802	1	17428	uc011xzb.1	2155	ENSMUSG000	Mnt	max binding protein
chr11	74833213	74833361	149	150	74833282	18	6.42934	3.41502	3.48999	Adipose_R1_peak_	Promoter (2-3	11	74830924	74845725	14802	1	17428	uc011xzb.1	2289	ENSMUSG000	Mnt	max binding protein
chr11	74833503	74833657	155	156	74833548	26	5.31252	2.48988	2.47999	Adipose_R2_peak_	Promoter (2-3	11	74830924	74845725	14802	1	17428	uc011xzb.1	2579	ENSMUSG000	Mnt	max binding protein
chr11	74833955	74834517	563	564	74834048	30	7.24477	2.85875	4.21917	Adipose_R2_peak_	Intron (uc011x	11	74830924	74845725	14802	1	17428	uc011xzb.1	3031	ENSMUSG000	Mnt	max binding protein
chr11	74834148	74834322	175	176	74834292	22	8.44503	3.84853	5.36095	Adipose_R1_peak_	Intron (uc011x	11	74830924	74845725	14802	1	17428	uc011xzb.1	3224	ENSMUSG000	Mnt	max binding protein
chr11	74834653	74834916	264	265	74834853	29	6.74062	2.76653	3.75989	Adipose_R2_peak_	Intron (uc011x	11	74830924	74845725	14802	1	17428	uc011xzb.1	3729	ENSMUSG000	Mnt	max binding protein
chr11	74835108	74835252	145	146	74835217	17	5.55374	3.12503	2.73464	Adipose_R1_peak_	Intron (uc011x	11	74830924	74845725	14802	1	17428	uc011xzb.1	4184	ENSMUSG000	Mnt	max binding protein
chr11	74835265	74835476	212	213	74835292	25	4.86612	2.39766	2.09018	Adipose_R2_peak_	Intron (uc011x	11	74830924	74845725	14802	1	17428	uc011xzb.1	4341	ENSMUSG000	Mnt	max binding protein
chr11	74835809	74836075	267	268	74835966	30	7.24477	2.85875	4.21917	Adipose_R2_peak_	Intron (uc011x	11	74830924	74845725	14802	1	17428	uc011xzb.1	4885	ENSMUSG000	Mnt	max binding protein
chr11	74836254	74836613	360	361	74836392	34	9.39139	3.22762	6.20474	Adipose_R2_peak_	Exon (uc011xz	11	74830924	74845725	14802	1	17428	uc011xzb.1	5330	ENSMUSG000	Mnt	max binding protein
chr11	74836567	74836716	150	151	74836619	18	6.21948	3.32361	3.33334	Adipose_R1_peak_	Exon (uc011xz	11	74830924	74845725	14802	1	17428	uc011xzb.1	5643	ENSMUSG000	Mnt	max binding protein
chr11	74837073	74837371	299	300	74837130	19	6.24956	3.24509	3.36288	Adipose_R1_peak_	Intron (uc011x	11	74830924	74845725	14802	1	17428	uc011xzb.1	6149	ENSMUSG000	Mnt	max binding protein
chr11	74837331	74837636	306	307	74837475	31	7.7623	2.95097	4.69388	Adipose_R2_peak_	Exon (uc011xz	11	74830924	74845725	14802	1	17428	uc011xzb.1	6407	ENSMUSG000	Mnt	max binding protein
chr11	74838008	74838180	173	174	74838103	30	7.24477	2.85875	4.21917	Adipose_R2_peak_	Intron (uc011x	11	74830924	74845725	14802	1	17428	uc011xzb.1	7084	ENSMUSG000	Mnt	max binding protein
chr11	74889899	74890098	200	201	74890018	43	14.8939	4.05758	11.42017	Adipose_R2_peak_	Intron (uc007k	11	74849264	74897080	47817	2	97761	uc007kck.1	6982	ENSMUSG000	Sgsm2	small G protein signaling modulator 2
chr11	74889964	74890097	134	135	74890044	22	9.13959	4.13397	6.01526	Adipose_R1_peak_	Intron (uc007k	11	74849264	74897080	47817	2	97761	uc007kck.1	6983	ENSMUSG000	Sgsm2	small G protein signaling modulator 2
chr11	75458605	75458743	139	140	75458679	19	7.07687	3.59476	4.08458	Adipose_R1_peak_	Promoter (2-3	11	75461539	75466690	5152	1	1.00E+08	uc007kdv.1	-2796	ENSMUSG000	Mir22hg	Mir22 host gene (non-protein coding)
chr11	75460238	75460434	197	198	75460296	25	4.86612	2.39766	2.09018	Adipose_R2_peak_	Promoter (1-2	11	75461539	75466690	5152	1	1.00E+08	uc007kdv.1	-1105	ENSMUSG000	Mir22hg	Mir22 host gene (non-protein coding)
chr11	75460668	75460841	174	175	75460738	19	7.07687	3.59476	4.08458	Adipose_R1_peak_	Promoter (<=	11	75461539	75466690	5152	1	1.00E+08	uc007kdv.1	-698	ENSMUSG000	Mir22hg	Mir22 host gene (non-protein coding)
chr11	76828368	76828478	111	112	76828444	28	6.25023	2.67431	3.31626	Adipose_R2_peak_	Intron (uc007k	11	76777208	76847008	69801	2	12874	uc007kga.1	18530	ENSMUSG000	Cpd	carboxypeptidase D
chr11	76865246	76865387	142	143	76865322	21	8.43286	3.95423	5.34896	Adipose_R1_peak_	Distal Intergen	11	76777208	76847008	69801	2	12874	uc007kga.1	-18238	ENSMUSG000	Cpd	carboxypeptidase D
chr11	81043509	81043683	175	176	81043602	34	9.39139	3.22762	6.20474	Adipose_R2_peak_	Intron (uc007k	11	80880163	81152903	272741	2	11418	uc007kml.2	109220	ENSMUSG000	Asic2	acid-sensing (proton-gated) ion channel 2
chr11	81202244	81202396	153	154	81202306	16	5.20017	3.05554	2.38734	Adipose_R1_peak_	Intron (uc007k	11	80880163	81152903	272741	2	11418	uc007kml.2	-49341	ENSMUSG000	Asic2	acid-sensing (proton-gated) ion channel 2
chr11	81304621	81304779	159	160	81304750	21	8.43286	3.95423	5.34896	Adipose_R1_peak_	Intron (uc007k	11	80880163	81152903	272741	2	11418	uc007kml.2	-151718	ENSMUSG000	Asic2	acid-sensing (proton-gated) ion channel 2
chr11	82128161	82128477	317	318	82128302	126	131.7124	22.82671	127.2552	Adipose_R1_peak_	Distal Intergen	11	82115185	82116799	1615	1	20307	uc007kmt.2	12976	ENSMUSG000	Ccl8	chemokine (C-C motif) ligand 8
chr11	82128186	82128421	236	237	82128306	52	21.18119	4.88754	17.49655	Adipose_R2_peak_	Distal Intergen	11	82115185	82116799	1615	1	20307	uc007kmt.2	13001	ENSMUSG000	Ccl8	chemokine (C-C motif) ligand 8
chr11	82316929	82317028	100	101	82316986	27	5.77405	2.5821	2.88921	Adipose_R2_peak_	Distal Intergen	11	82388900	82446361	57462	1	270893	uc007kmv.2	-71872	ENSMUSG000	Tmem132e	transmembrane protein 132E
chr11	82468786	82469029	244	245	82468919	47	31.38649	8.62742	27.65048	Adipose_R1_peak_	Distal Intergen	11	82388900	82446361	57462	1	270893	uc007kmv.2	79886	ENSMUSG000	Tmem132e	transmembrane protein 132E
chr11	82468789	82469039	251	252	82468917	60	27.32259	5.62528	23.49644	Adipose_R2_peak_	Distal Intergen	11	82388900	82446361	57462	1	270893	uc007kmv.2	79889	ENSMUSG000	Tmem132e	transmembrane protein 132E
chr11	82469246	82469457	212	213	82469384	18	6.42934	3.41502	3.48999	Adipose_R1_peak_	Distal Intergen	11	82388900	82446361	57462	1	270893	uc007kmv.2	80346	ENSMUSG000	Tmem132e	transmembrane protein 132E
chr11	83075623	83075840	218	219	83075731	38	11.72962	3.59649	8.4047	Adipose_R2_peak_	Distal Intergen	11	83065112	83070678	5567	1	20556	uc007kog.1	10511	ENSMUSG000	Slfn2	schlafen 2
chr11	83076349	83076739	391	392	83076598	48	32.41826	8.80716	28.66523	Adipose_R1_peak_	Distal Intergen	11	83065112	83070678	5567	1	20556	uc007kog.1	11237	ENSMUSG000	Slfn2	schlafen 2
chr11	83076475	83076730	256	257	83076621	47	17.60017	4.42645	14.02531	Adipose_R2_peak_	Distal Intergen	11	83065112	83070678	5567	1	20556	uc007kog.1	11363	ENSMUSG000	Slfn2	schlafen 2
chr11	83552298	83552499	202	203	83552426	46	16.90986	4.33423	13.35898	Adipose_R2_peak_	Distal Intergen	11	83525779	83530518	4740	2	20304	uc007kpi.2	-21780	ENSMUSG000	Ccl5	chemokine (C-C motif) ligand 5
chr11	83552311	83552479	169	170	83552408	28	13.73721	5.2124	10.41463	Adipose_R1_peak_	Distal Intergen	11	83525779	83530518	4740	2	20304	uc007kpi.2	-21793	ENSMUSG000	Ccl5	chemokine (C-C motif) ligand 5
chr11	85504399	85505138	740	741	85504560	19	7.07687	3.59476	4.08458	Adipose_R1_peak_	Intron (uc007k	11	85640704	85826058	185355	1	192197	uc007kru.2	-135566	ENSMUSG000	Bcas3	breast carcinoma amplified sequence 3
chr11	85665616	85665861	246	247	85665739	35	19.76029	6.47056	16.26028	Adipose_R1_peak_	Intron (uc007k	11	85640704	85826058	185355	1	192197	uc007kru.2	24912	ENSMUSG000	Bcas3	breast carcinoma amplified sequence 3
chr11	85665620	85665819	200	201	85665736	44	15.55653	4.1498	12.05625	Adipose_R2_peak_	Intron (uc007k	11	85640704	85826058	185355	1	192197	uc007kru.2	24916	ENSMUSG000	Bcas3	breast carcinoma amplified sequence 3
chr11	85674091	85674418	328	329	85674267	34	18.32225	6.08337	14.86709	Adipose_R1_peak_	Intron (uc007k	11	85640704	85826058	185355	1	192197	uc007kru.2	33387	ENSMUSG000	Bcas3	breast carcinoma amplified sequence 3
chr11	85674109	85674393	285	286	85674268	45	16.22858	4.24202	12.70278	Adipose_R2_peak_	Intron (uc007k	11	85640704	85826058	185355	1	192197	uc007kru.2	33405	ENSMUSG000	Bcas3	breast carcinoma amplified sequence 3
chr11	86826797	86826987	191	192	86826868	18	6.42934	3.41502	3.48999	Adipose_R1_peak_	Distal Intergen	11	86768849	86807660	38812	2	67487	uc007ktc.1	-19137	ENSMUSG000	Dhx40	DEAH (Asp-Glu-Ala-His) box polypeptide 40
chr11	86848900	86848997	98	99	86848949	28	6.25023	2.67431	3.31626	Adipose_R2_peak_	Distal Intergen	11	86768849	86807660	38812	2	67487	uc007ktc.1	-41240	ENSMUSG000	Dhx40	DEAH (Asp-Glu-Ala-His) box polypeptide 40
chr11	87440985	87441206	222	223	87441088	18	6.42934	3.41502	3.48999	Adipose_R1_peak_	Intron (uc007k	11	87427591	87555823	128233	1	83560	uc011yby.1	13394	ENSMUSG000	Tex14	testis expressed gene 14
chr11	87443045	87443360	316	317	87443193	35	9.50295	3.2016	6.37552	Adipose_R1_peak_	Intron (uc007k	11	87427591	87555823	128233	1	83560	uc011yby.1	15454	ENSMUSG000	Tex14	testis expressed gene 14
chr11	87443057	87443373	317	318	87443167	60	19.38378	3.99728	15.7565	Adipose_R2_peak_	Intron (uc007k	11	87427591	87555823	128233	1	83560	uc011yby.1	15466	ENSMUSG000	Tex14	testis expressed gene 14
chr11	87446787	87446918	132	133	87446797	25	4.86612	2.39766	2.09018	Adipose_R2_peak_	Intron (uc007k	11	87427591	87555823	128233	1	83560	uc011yby.1	19196	ENSMUSG000	Tex14	testis expressed gene 14
chr11	87464758	87464896	139	140	87464808	18	6.42934	3.41502	3.48999	Adipose_R1_peak_	Intron (uc007k	11	87427591	87555823	128233	1	83560	uc011yby.1	37167	ENSMUSG000	Tex14	testis expressed gene 14
chr11	87466140	87466288	149	150	87466178	29	6.74062	2.76653	3.75989	Adipose_R2_peak_	Intron (uc007k	11	87427591	87555823	128233	1	83560	uc011yby.1	38549	ENSMUSG000	Tex14	testis expressed gene 14
chr11	87468930	87469060	131	132	87468981	26	5.31252	2.48988	2.47999	Adipose_R2_peak_	Intron (uc007k	11	87427591	87555823	128233	1	83560	uc011yby.1	41339	ENSMUSG000	Tex14	testis expressed gene 14
chr11	87469432	87469646	215	216	87469476	27	5.77405	2.5821	2.88921	Adipose_R2_peak_	Intron (uc007k	11	87427591	87555823	128233	1	83560	uc011yby.1	41841	ENSMUSG000	Tex14	testis expressed gene 14
chr11	87586685	87586880	196	197	87586762	21	8.43286	3.95423	5.34896	Adipose_R1_peak_	Intron (uc007k	11	87592217	87616296	24080	1	170749	uc007ktz.1	-5337	ENSMUSG000	Mtmr4	myotubularin related protein 4

chr11	87588284	87588380	97	98	87588302	26	5.31252	2.48988	2.47999	Adipose_R2_peak_	Intron (uc007k	11	87592217	87616296	24080	1	170749	uc007ktz.1	-3837	ENSMUSG000	Mtmr4	myotubularin related protein 4
chr11	87590930	87591074	145	146	87590967	27	5.77405	2.5821	2.88921	Adipose_R2_peak_	Promoter (1-2	11	87592217	87616296	24080	1	170749	uc007ktz.1	-1143	ENSMUSG000	Mtmr4	myotubularin related protein 4
chr11	87593032	87593127	96	97	87593045	25	4.86612	2.39766	2.09018	Adipose_R2_peak_	Promoter (<=	11	87592978	87616296	23319	1	170749	uc007kub.1	54	ENSMUSG000	Mtmr4	myotubularin related protein 4
chr11	87593192	87593370	179	180	87593276	19	7.07687	3.59476	4.08458	Adipose_R1_peak_	Promoter (<=	11	87592978	87616296	23319	1	170749	uc007kub.1	214	ENSMUSG000	Mtmr4	myotubularin related protein 4
chr11	87593834	87593978	145	146	87593879	27	5.77405	2.5821	2.88921	Adipose_R2_peak_	Promoter (<=	11	87592978	87616296	23319	1	170749	uc007kub.1	856	ENSMUSG000	Mtmr4	myotubularin related protein 4
chr11	87594691	87594847	157	158	87594745	30	7.24477	2.85875	4.21917	Adipose_R2_peak_	Promoter (1-2	11	87592978	87616296	23319	1	170749	uc007kub.1	1713	ENSMUSG000	Mtmr4	myotubularin related protein 4
chr11	88823949	88824173	225	226	88823974	16	5.20017	3.05554	2.38734	Adipose_R1_peak_	Distal Intergen	11	88830792	88851512	20721	2	11640	uc007kvu.2	27339	ENSMUSG000	Akap1	A kinase (PRKA) anchor protein 1
chr11	88850217	88850397	181	182	88850280	27	5.77405	2.5821	2.88921	Adipose_R2_peak_	Promoter (1-2	11	88830792	88851512	20721	2	11640	uc007kvu.2	1115	ENSMUSG000	Akap1	A kinase (PRKA) anchor protein 1
chr11	88850673	88850846	174	175	88850753	21	8.43286	3.95423	5.34896	Adipose_R1_peak_	Promoter (<=	11	88830792	88851512	20721	2	11640	uc007kvu.2	666	ENSMUSG000	Akap1	A kinase (PRKA) anchor protein 1
chr11	88967786	88969177	1392	1393	88967985	77	16.74916	3.00512	13.21312	Adipose_R2_peak_	Promoter (<=	11	88964666	88966917	2252	2	217066	uc007kvy.2	-869	ENSMUSG000	Gm15698	transcription elongation factor B (SIII), polypeptide 2 pseudogene
chr11	88967807	88967942	136	137	88967920	37	6.9215	2.50359	3.97519	Adipose_R1_peak_	Promoter (<=	11	88964666	88966917	2252	2	217066	uc007kvy.2	-890	ENSMUSG000	Gm15698	transcription elongation factor B (SIII), polypeptide 2 pseudogene
chr11	88968450	88968622	173	174	88968481	40	5.71475	2.1732	2.89268	Adipose_R1_peak_	Promoter (1-2	11	88964666	88966917	2252	2	217066	uc007kvy.2	-1533	ENSMUSG000	Gm15698	transcription elongation factor B (SIII), polypeptide 2 pseudogene
chr11	88969338	88970272	935	936	88969496	98	38.70568	5.10995	34.69368	Adipose_R2_peak_	Promoter (2-3	11	88972638	88973014	377	1	72381	uc011yck.1	-2366	ENSMUSG000	2210409E12Ri	transcription elongation factor B (SIII), polypeptide 2 pseudogene
chr11	88969377	88969743	367	368	88969515	53	18.54894	4.22864	15.09282	Adipose_R1_peak_	Promoter (2-3	11	88964666	88966917	2252	2	217066	uc007kvy.2	-2460	ENSMUSG000	Gm15698	transcription elongation factor B (SIII), polypeptide 2 pseudogene
chr11	88969879	88970037	159	160	88970024	36	5.07751	2.12754	2.33131	Adipose_R1_peak_	Promoter (2-3	11	88972638	88973014	377	1	72381	uc011yck.1	-2601	ENSMUSG000	2210409E12Ri	transcription elongation factor B (SIII), polypeptide 2 pseudogene
chr11	88970394	88970770	377	378	88970570	69	15.43074	3.03343	11.9454	Adipose_R2_peak_	Promoter (1-2	11	88972638	88973014	377	1	72381	uc011yck.1	-1868	ENSMUSG000	2210409E12Ri	transcription elongation factor B (SIII), polypeptide 2 pseudogene
chr11	88970893	88971091	199	200	88970963	52	18.15829	4.20855	14.57382	Adipose_R2_peak_	Promoter (1-2	11	88972638	88973014	377	1	72381	uc011yck.1	-1547	ENSMUSG000	2210409E12Ri	transcription elongation factor B (SIII), polypeptide 2 pseudogene
chr11	89500672	89500840	169	170	89500717	27	5.77405	2.5821	2.88921	Adipose_R2_peak_	Intron (uc007k	11	89421086	89538555	117470	2	382543	uc007kwj.1	37715	ENSMUSG000	Ankfn1	ankyrin-repeat and fibronectin type III domain containing 1
chr11	89560246	89560534	289	290	89560409	70	57.07198	12.76139	53.01762	Adipose_R1_peak_	Distal Intergen	11	89421086	89538555	117470	2	382543	uc007kwj.1	-21691	ENSMUSG000	Ankfn1	ankyrin-repeat and fibronectin type III domain containing 1
chr11	89560262	89560533	272	273	89560390	71	36.49133	6.63968	32.5091	Adipose_R2_peak_	Distal Intergen	11	89421086	89538555	117470	2	382543	uc007kwj.1	-21707	ENSMUSG000	Ankfn1	ankyrin-repeat and fibronectin type III domain containing 1
chr11	89700829	89701213	385	386	89700998	23	9.86443	4.31371	6.70247	Adipose_R1_peak_	Intron (uc007k	11	89421086	89538555	117470	2	382543	uc007kwj.1	-162274	ENSMUSG000	Ankfn1	ankyrin-repeat and fibronectin type III domain containing 1
chr11	90383473	90383735	263	264	90383537	21	8.43286	3.95423	5.34896	Adipose_R1_peak_	Intron (uc007k	11	90339154	90388207	49054	2	217082	uc007kwt.2	4472	ENSMUSG000	Hlf	hepatic leukemia factor
chr11	90386342	90386436	95	96	90386348	25	4.86612	2.39766	2.09018	Adipose_R2_peak_	Promoter (1-2	11	90339154	90388207	49054	2	217082	uc007kwt.2	1771	ENSMUSG000	Hlf	hepatic leukemia factor
chr11	90865273	90865418	146	147	90865380	18	6.42934	3.41502	3.48999	Adipose_R1_peak_	Intron (uc007k	11	90883320	90895152	11833	1	73922	uc007kxe.1	-17902	ENSMUSG000	4930405D11R	RIKEN cDNA 4930405D11 gene
chr11	90936711	90936864	154	155	90936772	23	9.86443	4.31371	6.70247	Adipose_R1_peak_	Distal Intergen	11	90883320	90895152	11833	1	73922	uc007kxe.1	53391	ENSMUSG000	4930405D11R	RIKEN cDNA 4930405D11 gene
chr11	90936731	90936828	98	99	90936764	26	5.31252	2.48988	2.47999	Adipose_R2_peak_	Distal Intergen	11	90883320	90895152	11833	1	73922	uc007kxe.1	53411	ENSMUSG000	4930405D11R	RIKEN cDNA 4930405D11 gene
chr11	90988211	90988406	196	197	90988297	25	11.36566	4.67319	8.13482	Adipose_R1_peak_	Distal Intergen	11	90883320	90895152	11833	1	73922	uc007kxe.1	104891	ENSMUSG000	4930405D11R	RIKEN cDNA 4930405D11 gene
chr11	91002053	91002311	259	260	91002172	38	22.52591	7.00978	18.96	Adipose_R1_peak_	Distal Intergen	11	90883320	90895152	11833	1	73922	uc007kxe.1	118733	ENSMUSG000	4930405D11R	RIKEN cDNA 4930405D11 gene
chr11	91002081	91002236	156	157	91002164	29	6.74062	2.76653	3.75989	Adipose_R2_peak_	Distal Intergen	11	90883320	90895152	11833	1	73922	uc007kxe.1	118761	ENSMUSG000	4930405D11R	RIKEN cDNA 4930405D11 gene
chr11	91024781	91025056	276	277	91024902	51	35.56531	9.34637	31.76235	Adipose_R1_peak_	Distal Intergen	11	90883320	90895152	11833	1	73922	uc007kxe.1	141461	ENSMUSG000	4930405D11R	RIKEN cDNA 4930405D11 gene
chr11	91024796	91024967	172	173	91024896	36	10.5378	3.41206	7.27949	Adipose_R2_peak_	Distal Intergen	11	90883320	90895152	11833	1	73922	uc007kxe.1	141476	ENSMUSG000	4930405D11R	RIKEN cDNA 4930405D11 gene
chr11	91266266	91266440	175	176	91266427	29	6.74062	2.76653	3.75989	Adipose_R2_peak_	Distal Intergen	11	91575286	91577555	2270	2	73470	uc007kxf.1	311115	ENSMUSG000	Kif2b	kinesin family member 2B
chr11	92054208	92054410	203	204	92054271	16	5.20017	3.05554	2.38734	Adipose_R1_peak_	Distal Intergen	11	91575286	91577555	2270	2	73470	uc007kxf.1	-476653	ENSMUSG000	Kif2b	kinesin family member 2B
chr11	92107183	92107320	138	139	92107264	19	7.07687	3.59476	4.08458	Adipose_R1_peak_	Distal Intergen	11	91575286	91577555	2270	2	73470	uc007kxf.1	-529628	ENSMUSG000	Kif2b	kinesin family member 2B
chr11	92243854	92244067	214	215	92243948	28	13.73721	5.2124	10.41463	Adipose_R1_peak_	Distal Intergen	11	91575286	91577555	2270	2	73470	uc007kxf.1	-666299	ENSMUSG000	Kif2b	kinesin family member 2B
chr11	92249289	92249489	201	202	92249398	23	9.86443	4.31371	6.70247	Adipose_R1_peak_	Distal Intergen	11	91575286	91577555	2270	2	73470	uc007kxf.1	-671734	ENSMUSG000	Kif2b	kinesin family member 2B
chr11	92598863	92599200	338	339	92599038	119	119.632	20.85727	115.218	Adipose_R1_peak_	Distal Intergen	11	93098410	93491923	393514	1	72605	uc007kxg.1	-499210	ENSMUSG000	Car10	carbonic anhydrase 10
chr11	92598899	92599180	282	283	92599041	104	67.98428	9.68286	63.65822	Adipose_R2_peak_	Distal Intergen	11	93098410	93491923	393514	1	72605	uc007kxg.1	-499230	ENSMUSG000	Car10	carbonic anhydrase 10
chr11	92878611	92878862	252	253	92878737	43	27.35048	7.90847	23.68561	Adipose_R1_peak_	Distal Intergen	11	93098410	93491923	393514	1	72605	uc007kxg.1	-219548	ENSMUSG000	Car10	carbonic anhydrase 10
chr11	92878619	92878857	239	240	92878738	54	22.67101	5.07198	18.94758	Adipose_R2_peak_	Distal Intergen	11	93098410	93491923	393514	1	72605	uc007kxg.1	-219553	ENSMUSG000	Car10	carbonic anhydrase 10
chr11	92917493	92917849	357	358	92917642	137	120.895	16.63854	116.4767	Adipose_R1_peak_	Distal Intergen	11	93098410	93491923	393514	1	72605	uc007kxg.1	-180561	ENSMUSG000	Car10	carbonic anhydrase 10
chr11	92917515	92917794	280	281	92917648	136	102.8792	12.63383	98.36011	Adipose_R2_peak_	Distal Intergen	11	93098410	93491923	393514	1	72605	uc007kxg.1	-180616	ENSMUSG000	Car10	carbonic anhydrase 10
chr11	93031824	93031974	151	152	93031957	18	6.42934	3.41502	3.48999	Adipose_R1_peak_	Distal Intergen	11	93098410	93491923	393514	1	72605	uc007kxg.1	-66436	ENSMUSG000	Car10	carbonic anhydrase 10
chr11	93223882	93224056	175	176	93224013	26	12.14079	4.85292	8.87838	Adipose_R1_peak_	Intron (uc007k	11	93099290	93601751	502462	1	72605	uc007kxj.2	124592	ENSMUSG000	Car10	carbonic anhydrase 10
chr11	93400666	93400845	180	181	93400776	19	7.07687	3.59476	4.08458	Adipose_R1_peak_	Intron (uc007k	11	93099290	93601751	502462	1	72605	uc007kxj.2	301376	ENSMUSG000	Car10	carbonic anhydrase 10
chr11	93428788	93429013	226	227	93428905	33	17.97508	6.11109	14.52164	Adipose_R1_peak_	Intron (uc007k	11	93099290	93601751	502462	1	72605	uc007kxj.2	329498	ENSMUSG000	Car10	carbonic anhydrase 10
chr11	93544208	93544389	182	183	93544278	29	14.55748	5.39214	11.20652	Adipose_R1_peak_	Intron (uc007k	11	93859243	93885766	26524	2	217109	uc007kxk.1	341377	ENSMUSG000	Utp18	UTP18 small subunit processome component
chr11	93558079	93558197	119	120	93558125	30	7.24477	2.85875	4.21917	Adipose_R2_peak_	Intron (uc007k	11	93859243	93885766	26524	2	217109	uc007kxk.1	327569	ENSMUSG000	Utp18	UTP18 small subunit processome component
chr11	93711610	93711816	207	208	93711732	26	12.14079	4.85292	8.87838	Adipose_R1_peak_	Distal Intergen	11	93859243	93885766	26524	2	217109	uc007kxk.1	173950	ENSMUSG000	Utp18	UTP18 small subunit processome component
chr11	93714571	93714870	300	301	93714743	39	23.47001	7.18952	19.88325	Adipose_R1_peak_	Distal Intergen	11	93859243	93885766	26524	2	217109	uc007kxk.1	170896	ENSMUSG000	Utp18	UTP18 small subunit processome component
chr11	93714630	93714786	157	158	93714641	25	4.86612	2.39766	2.09018	Adipose_R2_peak_	Distal Intergen	11	93859243	93885766	26524	2	217109	uc007kxk.1	170980	ENSMUSG000	Utp18	UTP18 small subunit processome component
chr11	93727859	93728009	151	152	93727923	18	6.42934	3.41502	3.48999	Adipose_R1_peak_	Distal Intergen	11	93859243	93885766	26524	2	217109	uc007kxk.1	157757	ENSMUSG000	Utp18	UTP18 small subunit processome component
chr11	93740406	93740566	161	162	93740475	19	7.07687	3.59476	4.08458	Adipose_R1_peak_	Distal Intergen	11	93859243	93885766	26524	2	217109	uc007kxk.1	145200	ENSMUSG000	Utp18	UTP18 small subunit processome component
chr11	93796870	93797021	152	153	93796902	16	5.20017	3.05554	2.38734	Adipose_R1_peak_	Distal Intergen	11	93859243	93885766	26524	2	217109	uc007kxk.1	88745	ENSMUSG000	Utp18	UTP18 small subunit processome component
chr11	93799954	93800101	148	149	93800045	18	6.42934	3.41502	3.48999	Adipose_R1_peak_	Distal Intergen	11	93859243	93885766	26524	2	217109	uc007kxk.1	85665	ENSMUSG000	Utp18	UTP18 small subunit processome component
chr11	94158990	94159137	148	149	94159072	32	8.29282	3.04319	5.18352	Adipose_R2_peak_	Distal Intergen	11	94211454	94215492	4039	1	22057	uc007kyf.1	-52317	ENSMUSG000	Tob1	transducer of ErbB-2.1
chr11	94165794	94165931	138	139	94165865	28	6.25023	2.67431	3.31626	Adipose_R2_peak_	Distal Intergen	11	94211454	94215492	4039	1	22057	uc007kyf.1	-45523	ENSMUSG000	Tob1	transducer of ErbB-2.1
chr11	94206491	94206645	155	156	94206548	27	5.77405	2.5821	2.88921	Adipose_R2_peak_	Distal Intergen	11	94211454	94215492	4039	1	22057	uc007kyf.1	-4809	ENSMUSG000	Tob1	transducer of ErbB-2.1
chr11	94207510	94207653	144	145	94207519	25	4.86612	2.39766	2.09018	Adipose_R2_peak_	Distal Intergen	11	94211454	94215492	4039	1	22057	uc007kyf.1	-3801	ENSMUSG000	Tob1	transducer of ErbB-2.1
chr11	94210659	94210857	199	200	94210731	19	7.03841	3.57815	4.08458	Adipose_R1_peak_	Promoter (<=	11	94211454	94215492	4039	1	22057	uc007kyf.1	-597	ENSMUSG000	Tob1	transducer of ErbB-2.1
chr11	94981057	94981162	106	107	94981126	26	5.31252	2.48988	2.47999	Adipose_R2_peak_	Distal Intergen	11	94962777	94976327	13551	2	20391	uc007kzo.3	-4730	ENSMUSG000	Sgca	sarcoglycan, alpha (dystrophin-associated glycoprotein)
chr11	94982675	94982853	179	180	94982769	22	9.13959	4.13397	6.01526	Adipose_R1_peak_	Distal Intergen	11	94962777	94976327	13551	2	20391	uc007kzo.3	-6348	ENSMUSG000	Sgca	sarcoglycan, alpha (dystrophin-associated glycoprotein)
chr11	94982739	94982962	224	225	94982814	29	6.74062	2.76653	3.75989	Adipose_R2_peak_	Distal Intergen	11	94962777	94976327	13551	2	20391	uc007kzo.3	-6412	ENSMUSG000	Sgca	sarcoglycan, alpha (dystrophin-associated glycoprotein)
chr11	94984882	94985142	261	262	94984979	27	5.77405	2.5821	2.88921	Adipose_R2_peak_	Distal Intergen	11	94991212	95006898	15687	1	217124	uc007kzr.2	-6070	ENSMUSG000	Ppp1r9b	protein phosphatase 1, regulatory subunit 9B
chr11	94995188	94995421	234	235	94995277	19	7.07687	3.59476	4.08458	Adipose_R1_peak_	Promoter (<=	11	94996064	95006898	10835	1	217124	uc007kzs.2	-643	ENSMUSG000	Ppp1r9b	protein phosphatase 1, regulatory subunit 9B
chr11	95799260	95799785	526	527	95799644	28	13.73721	5.2124	10.41463	Adipose_R1_peak_	Intron (uc007l	11	95824500	95832140	7641	1	237928	uc007las.2	-24715	ENSMUSG000	Phospho1	phosphatase, orphan 1
chr11	95826176	95826283	108	109	95826215	27	5.77405	2.5821	2.88921	Adipose_R2_peak_	Promoter (1-2	11	95824500	95832140	7641	1	237928	uc007las.2	1676	ENSMUSG000	Phospho1	phosphatase, orphan 1
chr11	95826407	95826504	98	99	95826494	28	6.25023	2.67431	3.31626	Adipose_R2_peak_	Promoter (1-2	11	95824500	95832140	7641	1	237928	uc007las.2	1907	ENSMUSG000	Phospho1	phosphatase, orphan 1
chr11	95827882	95828014	133	134	95827973	16	5.20017	3.05554	2.38734	Adipose_R1_peak_	Intron (uc007l	11	95824500	95832140	7641	1	237928	uc007las.2	3382	ENSMUSG000	Phospho1	phosphatase, orphan 1
chr11	95829416	95829646	231	232	95829486	27	5.77405	2.5821	2.88921	Adipose_R2_peak_	Intron (uc007l	11	95824500	95832140	7641	1	237928	uc007las.2	4916	ENSMUSG000	Phospho1	phosphatase, orphan 1
chr11	96649990	96650131	142	143	96650088	21	8.43286	3.95423	5.34896	Adipose_R1_peak_	Intron (uc007l	11	96767549	96777555	10007	2	74479	uc007lci.2	127424	ENSMUSG000	Snx11	sorting nexin 11
chr11	96764066	96764194	129	130	96764149	28	6.25023	2.67431	3.31626	Adipose_R2_peak_	Distal Intergen	11	96767549	96777555	10007	2	74479	uc007lci.2	13361	ENSMUSG000	Snx11	sorting nexin 11
chr11	96916678	96916802	125	126	96916757	29	6.74062	2.76653	3.75989	Adipose_R2_peak_	Promoter (<=	11	96907425	96916514	9090	2	80280	uc007lcv.2	-164	ENSMUSG000	Cdk5rap3	CDK5 regulatory subunit associated protein 3
chr11	96917139	96917306	168	169	96917203	20	7.74501	3.7745	4.70467	Adipose_R1_peak_	Promoter (<=	11	96907425	96916514	9090	2	80280	uc007lcv.2	-625	ENSMUSG000	Cdk5rap3	CDK5 regulatory subunit associated protein 3
chr11	96917140	96917323	184	185	96917216	31	7.7623	2.95097	4.69388	Adipose_R2_peak_	Promoter (<=	11	96907425	96916514	9090	2	80280	uc007lcv.2	-626	ENSMUSG000	Cdk5rap3	CDK5 regulatory subunit associated protein 3
chr11	97700975	97701187	213	214	97701042	23	9.86443	4.31371	6.70247	Adipose_R1_peak_	Promoter (<=	11	97688823	97700497	11675	2	22658	uc007len.2	-478	ENSMUSG000	Pcgf2	polycomb group ring finger 2
chr11	97701502	97701751	250	251	97701651	26	5.31252	2.48988	2.47999	Adipose_R2_peak_	Promoter (1-2	11	97688823	97700497	11675	2	22658	uc007len.2	-1005	ENSMUSG000	Pcgf2	polycomb group ring finger 2
chr11	98650126	98650348	223	224	98650261	20	7.74501	3.7745	4.70467	Adipose_R1_peak_	Intron (uc007l	11	98646759	98650123	3365	1	76758	uc007lgt.1	3367	ENSMUSG000	Gsdma2	gasdermin A2
chr11	98653108	98653261	154	155	98653170	56	17.3305	3.81722	13.89967	Adipose_R1_peak_	Intron (uc007l	11	98646759	98650123	3365	1	76758	uc007lgt.1	6349	ENSMUSG000	Gsdma2	gasdermin A2
chr11	98739727	98739878	152	153	98739823	22	9.13959	4.13397	6.01526	Adipose_R1_peak_	Promoter (1-2	11	98741785	98757695	15911	1	21833	uc007lhd.2	-1907	ENSMUSG000	Thra	thyroid hormone receptor alpha
chr11	98747716	98747864	149	150	98747738	17	5.80341	3.23528	2.92378	Adipose_R1_peak_	Intron (uc007l	11	98753290	98768403	15114	1	21833	uc007lhg.1	-5426	ENSMUSG000	Thra	thyroid hormone receptor alpha
chr11	98753791	98753922	132	133	98753862	27	5.77405	2.5821	2.88921	Adipose_R2_peak_	Promoter (<=	11	98753290	98768403	15114	1	21833	uc007lhg.1	501	ENSMUSG000	Thra	thyroid hormone receptor alpha
chr11	98755465	98755622	158	159	98755543	20	7.74501	3.7745	4.70467	Adipose_R1_peak_	Promoter (2-3	11	98753290	98768403	15114	1	21833	uc007lhg.1	2175	ENSMUSG000	Thra	thyroid hormone receptor alpha
chr11	98755469	98755562	94	95	98755514	26	5.31252	2.48988	2.47999	Adipose_R2_peak_	Promoter (2-3	11	98753290	98768403	15114	1	21833	uc007lhg.1	2179	ENSMUSG000	Thra	thyroid hormone receptor alpha
chr11	98763119	98763269	151	152	98763205	17	5.80341	3.23528	2.92378	Adipose_R1_peak_	Intron (uc007l	11	98753290	98768403	15114	1	21833	uc007lhg.1	9829	ENSMUSG000	Thra	thyroid hormone receptor alpha
chr11	98771143	98771464	322	323	98771189	28	6.25023	2.67431	3.31626	Adipose_R2_peak_	Exon (uc056yc	11	98767932	98775377	7446	2	217166	uc007lhh.2	3913	ENSMUSG000	Nr1d1	nuclear receptor subfamily 1, group D, member 1
chr11	98771296	98771555	260	261	98771420	17	5.46142	3.08465	2.64381	Adipose_R1_peak_	Exon (uc056yc	11	98767932	98775377	7446	2	217166	uc007lhh.2	3822	ENSMUSG000	Nr1d1	nuclear receptor subfamily 1, group D, member 1
chr11	98771892	98772132	241	242	98771979	26	5.31252	2.48988	2.47999	Adipose_R2_peak_	Exon (uc056yc	11	98767932	98775377	7446	2	217166	uc007lhh.2	3245	ENSMUSG000	Nr1d1	nuclear receptor subfamily 1, group D, member 1
chr11	98772812	98773001	190	191	98772962	30	7.24477	2.85875	4.21917	Adipose_R2_peak_	Promoter (2-3	11	98767932	98775377	7446	2	217166	uc007lhh.2	2376	ENSMUSG000	Nr1d1	nuclear receptor subfamily 1, group D, member 1
chr11	98773105	98773335	231	232	98773217	28	6.25023	2.67431	3.31626	Adipose_R2_peak_	Promoter (2-3	11	98767932	98775377	7446	2	217166	uc007lhh.2	2042	ENSMUSG000	Nr1d1	nuclear receptor subfamily 1, group D, member 1
chr11	98773302	98773500	199	200	98773367	23	9.86443	4.31371	6.70247	Adipose_R1_peak_	Promoter (1-2	11	98767932	98775377	7446	2	217166	uc007lhh.2	1877	ENSMUSG000	Nr1d1	nuclear receptor subfamily 1, group D, member 1
chr11	98774911	98775122	212	213	98774933	17	5.24366	2.99024	2.43022	Adipose_R1_peak_	Promoter (<=	11	98767932	98775377	7446	2	217166	uc007lhh.2	255	ENSMUSG000	Nr1d1	nuclear receptor subfamily 1, group D, member 1
chr11	98776102	98776331	230	231	98776211	35	9.95877	3.31984	6.73562	Adipose_R2_peak_	Promoter (<=	11	98767932	98775377	7446	2	217166	uc007lhh.2	-725	ENSMUSG000	Nr1d1	nuclear receptor subfamily 1, group D, member 1
chr11	98776661	98776926	266	267	98776735	33	8.83596	3.1354	5.68652	Adipose_R2_peak_	Promoter (1-2	11	98767932	98775377	7446	2	217166	uc007lhh.2	-1284	ENSMUSG000	Nr1d1	nuclear receptor subfamily 1, group D, member 1

chr11	98777187	98777466	280	281	98777297	25	4.86612	2.39766	2.09018	Adipose_R2_peak_	Promoter (1-2	11	98767932	98775377	7446	2	217166	uc007lhh.2	-1810	ENSMUSG000	Nr1d1	nuclear receptor subfamily 1, group D, member 1
chr11	98777629	98777804	176	177	98777758	26	12.14079	4.85292	8.87838	Adipose_R1_peak_	Promoter (2-3	11	98767932	98775377	7446	2	217166	uc007lhh.2	-2252	ENSMUSG000	Nr1d1	nuclear receptor subfamily 1, group D, member 1
chr11	98777724	98777926	203	204	98777808	29	6.74062	2.76653	3.75989	Adipose_R2_peak_	Promoter (2-3	11	98767932	98775377	7446	2	217166	uc007lhh.2	-2347	ENSMUSG000	Nr1d1	nuclear receptor subfamily 1, group D, member 1
chr11	98778395	98778551	157	158	98778469	28	6.25023	2.67431	3.31626	Adipose_R2_peak_	Distal Interger	11	98767932	98775377	7446	2	217166	uc007lhh.2	-3018	ENSMUSG000	Nr1d1	nuclear receptor subfamily 1, group D, member 1
chr11	98779374	98779610	237	238	98779517	31	7.7623	2.95097	4.69388	Adipose_R2_peak_	Distal Interger	11	98767932	98775377	7446	2	217166	uc007lhh.2	-3997	ENSMUSG000	Nr1d1	nuclear receptor subfamily 1, group D, member 1
chr11	98779739	98779885	147	148	98779840	20	7.32897	3.59876	4.33346	Adipose_R1_peak_	Distal Interger	11	98767932	98775377	7446	2	217166	uc007lhh.2	-4362	ENSMUSG000	Nr1d1	nuclear receptor subfamily 1, group D, member 1
chr11	98779990	98780683	694	695	98780091	34	9.39139	3.22762	6.20474	Adipose_R2_peak_	Distal Interger	11	98767932	98775377	7446	2	217166	uc007lhh.2	-4613	ENSMUSG000	Nr1d1	nuclear receptor subfamily 1, group D, member 1
chr11	98781108	98781723	616	617	98781309	22	9.13959	4.13397	6.01526	Adipose_R1_peak_	Distal Interger	11	98767932	98775377	7446	2	217166	uc007lhh.2	-5731	ENSMUSG000	Nr1d1	nuclear receptor subfamily 1, group D, member 1
chr11	98782484	98782595	112	113	98782493	25	4.86612	2.39766	2.09018	Adipose_R2_peak_	Distal Interger	11	98767932	98775377	7446	2	217166	uc007lhh.2	-7107	ENSMUSG000	Nr1d1	nuclear receptor subfamily 1, group D, member 1
chr11	98782832	98782948	117	118	98782866	25	4.86612	2.39766	2.09018	Adipose_R2_peak_	Distal Interger	11	98767932	98775377	7446	2	217166	uc007lhh.2	-7455	ENSMUSG000	Nr1d1	nuclear receptor subfamily 1, group D, member 1
chr11	98783624	98784367	744	745	98783760	33	8.83596	3.1354	5.68652	Adipose_R2_peak_	Distal Interger	11	98767932	98775377	7446	2	217166	uc007lhh.2	-8247	ENSMUSG000	Nr1d1	nuclear receptor subfamily 1, group D, member 1
chr11	98785627	98785806	180	181	98785705	35	9.95877	3.31984	6.73562	Adipose_R2_peak_	Distal Interger	11	98795769	98801038	5270	1	74026	uc007lhi.2	-9963	ENSMUSG000	Msl1	male-specific lethal 1 homolog (Drosophila)
chr11	98786062	98786201	140	141	98786123	21	8.43286	3.95423	5.34896	Adipose_R1_peak_	Distal Interger	11	98795769	98801038	5270	1	74026	uc007lhi.2	-9568	ENSMUSG000	Msl1	male-specific lethal 1 homolog (Drosophila)
chr11	98787117	98787461	345	346	98787336	18	5.37063	2.96457	2.55464	Adipose_R1_peak_	Distal Interger	11	98795769	98801038	5270	1	74026	uc007lhi.2	-8308	ENSMUSG000	Msl1	male-specific lethal 1 homolog (Drosophila)
chr11	98787206	98787705	500	501	98787429	29	6.74062	2.76653	3.75989	Adipose_R2_peak_	Distal Interger	11	98795769	98801038	5270	1	74026	uc007lhi.2	-8064	ENSMUSG000	Msl1	male-specific lethal 1 homolog (Drosophila)
chr11	98788125	98788662	538	539	98788171	29	6.74062	2.76653	3.75989	Adipose_R2_peak_	Distal Interger	11	98795769	98801038	5270	1	74026	uc007lhi.2	-7107	ENSMUSG000	Msl1	male-specific lethal 1 homolog (Drosophila)
chr11	98788761	98788906	146	147	98788844	30	7.24477	2.85875	4.21917	Adipose_R2_peak_	Distal Interger	11	98795769	98801038	5270	1	74026	uc007lhi.2	-6863	ENSMUSG000	Msl1	male-specific lethal 1 homolog (Drosophila)
chr11	98789107	98789245	139	140	98789171	19	7.07687	3.59476	4.08458	Adipose_R1_peak_	Distal Interger	11	98795769	98801038	5270	1	74026	uc007lhi.2	-6524	ENSMUSG000	Msl1	male-specific lethal 1 homolog (Drosophila)
chr11	98789411	98790035	625	626	98789521	31	7.7623	2.95097	4.69388	Adipose_R2_peak_	Distal Interger	11	98795769	98801038	5270	1	74026	uc007lhi.2	-5734	ENSMUSG000	Msl1	male-specific lethal 1 homolog (Drosophila)
chr11	98789416	98789552	137	138	98789516	18	6.42934	3.41502	3.48999	Adipose_R1_peak_	Distal Interger	11	98795769	98801038	5270	1	74026	uc007lhi.2	-6217	ENSMUSG000	Msl1	male-specific lethal 1 homolog (Drosophila)
chr11	98790749	98790981	233	234	98790848	27	5.77405	2.5821	2.88921	Adipose_R2_peak_	Distal Interger	11	98795769	98801038	5270	1	74026	uc007lhi.2	-4788	ENSMUSG000	Msl1	male-specific lethal 1 homolog (Drosophila)
chr11	98791368	98791624	257	258	98791472	29	6.74062	2.76653	3.75989	Adipose_R2_peak_	Distal Interger	11	98795769	98801038	5270	1	74026	uc007lhi.2	-4145	ENSMUSG000	Msl1	male-specific lethal 1 homolog (Drosophila)
chr11	98791633	98791979	347	348	98791852	19	7.07687	3.59476	4.08458	Adipose_R1_peak_	Distal Interger	11	98795769	98801038	5270	1	74026	uc007lhi.2	-3790	ENSMUSG000	Msl1	male-specific lethal 1 homolog (Drosophila)
chr11	98792762	98792973	212	213	98792825	20	7.70414	3.75706	4.70412	Adipose_R1_peak_	Promoter (2-3	11	98795769	98801038	5270	1	74026	uc007lhi.2	-2796	ENSMUSG000	Msl1	male-specific lethal 1 homolog (Drosophila)
chr11	98793380	98793507	128	129	98793386	25	4.86612	2.39766	2.09018	Adipose_R2_peak_	Promoter (2-3	11	98795769	98801038	5270	1	74026	uc007lhi.2	-2262	ENSMUSG000	Msl1	male-specific lethal 1 homolog (Drosophila)
chr11	98793696	98794182	487	488	98793781	31	7.7623	2.95097	4.69388	Adipose_R2_peak_	Promoter (1-2	11	98795769	98801038	5270	1	74026	uc007lhi.2	-1587	ENSMUSG000	Msl1	male-specific lethal 1 homolog (Drosophila)
chr11	98793726	98794433	708	709	98793816	19	7.07687	3.59476	4.08458	Adipose_R1_peak_	Promoter (1-2	11	98795769	98801038	5270	1	74026	uc007lhi.2	-1336	ENSMUSG000	Msl1	male-specific lethal 1 homolog (Drosophila)
chr11	98806525	98806634	110	111	98806554	27	5.77405	2.5821	2.88921	Adipose_R2_peak_	3' UTR	11	98809808	98833807	24000	1	192160	uc011yeh.1	-3174	ENSMUSG000	Casc3	cancer susceptibility candidate 3
chr11	98806995	98807172	178	179	98807036	18	6.42934	3.41502	3.48999	Adipose_R1_peak_	Promoter (2-3	11	98809808	98833807	24000	1	192160	uc011yeh.1	-2636	ENSMUSG000	Casc3	cancer susceptibility candidate 3
chr11	98936865	98936964	100	101	98936877	26	5.31252	2.48988	2.47999	Adipose_R2_peak_	Promoter (<=	11	98937696	98974942	37247	1	19401	uc007lhx.1	-732	ENSMUSG000	Rara	retinoic acid receptor, alpha
chr11	98939926	98940156	231	232	98939989	16	5.20017	3.05554	2.38734	Adipose_R1_peak_	Promoter (<=	11	98939711	98974942	35232	1	19401	uc011yei.1	215	ENSMUSG000	Rara	retinoic acid receptor, alpha
chr11	98940016	98940126	111	112	98940088	29	6.74062	2.76653	3.75989	Adipose_R2_peak_	Promoter (<=	11	98939711	98974942	35232	1	19401	uc011yei.1	305	ENSMUSG000	Rara	retinoic acid receptor, alpha
chr11	98940508	98940658	151	152	98940636	17	5.80341	3.23528	2.92378	Adipose_R1_peak_	Promoter (<=	11	98939711	98974942	35232	1	19401	uc011yei.1	797	ENSMUSG000	Rara	retinoic acid receptor, alpha
chr11	98946105	98946314	210	211	98946238	31	7.7623	2.95097	4.69388	Adipose_R2_peak_	Intron (uc007ll	11	98939711	98974942	35232	1	19401	uc011yei.1	6394	ENSMUSG000	Rara	retinoic acid receptor, alpha
chr11	98948505	98948641	137	138	98948515	25	4.86612	2.39766	2.09018	Adipose_R2_peak_	Intron (uc007ll	11	98939711	98974942	35232	1	19401	uc011yei.1	8794	ENSMUSG000	Rara	retinoic acid receptor, alpha
chr11	98949121	98949477	357	358	98949297	26	5.31252	2.48988	2.47999	Adipose_R2_peak_	Intron (uc007ll	11	98939711	98974942	35232	1	19401	uc011yei.1	9410	ENSMUSG000	Rara	retinoic acid receptor, alpha
chr11	98955457	98955556	100	101	98955501	25	4.86612	2.39766	2.09018	Adipose_R2_peak_	Intron (uc007ll	11	98960471	98974942	14472	1	19401	uc007lhz.2	-4915	ENSMUSG000	Rara	retinoic acid receptor, alpha
chr11	98958024	98958248	225	226	98958047	16	5.20017	3.05554	2.38734	Adipose_R1_peak_	Promoter (2-3	11	98960471	98974942	14472	1	19401	uc007lhz.2	-2223	ENSMUSG000	Rara	retinoic acid receptor, alpha
chr11	98961904	98962099	196	197	98961949	28	6.25023	2.67431	3.31626	Adipose_R2_peak_	Promoter (1-2	11	98960471	98974942	14472	1	19401	uc007lhz.2	1433	ENSMUSG000	Rara	retinoic acid receptor, alpha
chr11	98966214	98966321	108	109	98966261	28	6.25023	2.67431	3.31626	Adipose_R2_peak_	Promoter (1-2	11	98964872	98974942	10071	1	19401	uc007lia.1	1342	ENSMUSG000	Rara	retinoic acid receptor, alpha
chr11	99024444	99024591	148	149	99024504	26	5.31252	2.48988	2.47999	Adipose_R2_peak_	Promoter (<=	11	98992947	99024189	31243	2	21973	uc007lid.1	-255	ENSMUSG000	Top2a	topoisomerase (DNA) II alpha
chr11	99024768	99025017	250	251	99024860	31	7.7623	2.95097	4.69388	Adipose_R2_peak_	Promoter (<=	11	98992947	99024189	31243	2	21973	uc007lid.1	-579	ENSMUSG000	Top2a	topoisomerase (DNA) II alpha
chr11	99032230	99032495	266	267	99032435	22	9.13959	4.13397	6.01526	Adipose_R1_peak_	Distal Interger	11	98992947	99024189	31243	2	21973	uc007lid.1	-8041	ENSMUSG000	Top2a	topoisomerase (DNA) II alpha
chr11	99883846	99884063	218	219	99883963	32	17.10103	5.93135	13.67137	Adipose_R1_peak_	Distal Interger	11	99879187	99880229	1043	1	670533	uc011yev.1	4659	ENSMUSG000	Gm11567	predicted gene 11567
chr11	99883875	99883988	114	115	99883967	30	7.24477	2.85875	4.21917	Adipose_R2_peak_	Distal Interger	11	99879187	99880229	1043	1	670533	uc011yev.1	4688	ENSMUSG000	Gm11567	predicted gene 11567
chr11	99922387	99922719	333	334	99922552	126	114.5892	17.37219	110.1952	Adipose_R1_peak_	Distal Interger	11	99914751	99915671	921	1	670550	uc011yew.1	7636	ENSMUSG000	Gm11565	predicted gene 11565
chr11	99922412	99922690	279	280	99922541	87	51.09389	8.11516	46.92713	Adipose_R2_peak_	Distal Interger	11	99914751	99915671	921	1	670550	uc011yew.1	7661	ENSMUSG000	Gm11565	predicted gene 11565
chr11	101059278	101059404	127	128	101059383	26	5.31252	2.48988	2.47999	Adipose_R2_peak_	Intron (uc007l	11	1.01E+08	1.01E+08	7795	1	27419	uc007lmz.2	-10473	ENSMUSG000	Naglu	alpha-N-acetylglucosaminidase (Sanfilippo disease IIIB)
chr11	101065738	101065872	135	136	101065815	25	4.86612	2.39766	2.09018	Adipose_R2_peak_	Distal Interger	11	1.01E+08	1.01E+08	7795	1	27419	uc007lmz.2	-4005	ENSMUSG000	Naglu	alpha-N-acetylglucosaminidase (Sanfilippo disease IIIB)
chr11	101067687	101067930	244	245	101067698	18	6.42934	3.41502	3.48999	Adipose_R1_peak_	Promoter (1-2	11	1.01E+08	1.01E+08	7795	1	27419	uc007lmz.2	-1947	ENSMUSG000	Naglu	alpha-N-acetylglucosaminidase (Sanfilippo disease IIIB)
chr11	101069390	101069593	204	205	101069428	17	5.80341	3.23528	2.92378	Adipose_R1_peak_	Promoter (<=	11	1.01E+08	1.01E+08	7795	1	27419	uc007lmz.2	-284	ENSMUSG000	Naglu	alpha-N-acetylglucosaminidase (Sanfilippo disease IIIB)
chr11	101072375	101072646	272	273	101072522	30	7.24477	2.85875	4.21917	Adipose_R2_peak_	Promoter (2-3	11	1.01E+08	1.01E+08	7578	1	27419	uc007lna.2	2281	ENSMUSG000	Naglu	alpha-N-acetylglucosaminidase (Sanfilippo disease IIIB)
chr11	101072936	101073102	167	168	101072997	20	7.74501	3.7745	4.70467	Adipose_R1_peak_	Promoter (2-3	11	1.01E+08	1.01E+08	7578	1	27419	uc007lna.2	2842	ENSMUSG000	Naglu	alpha-N-acetylglucosaminidase (Sanfilippo disease IIIB)
chr11	101074677	101074799	123	124	101074717	27	5.77405	2.5821	2.88921	Adipose_R2_peak_	Intron (uc007l	11	1.01E+08	1.01E+08	2095	1	15485	uc007lnb.1	-3612	ENSMUSG000	Hsd17b1	hydroxysteroid (17-beta) dehydrogenase 1
chr11	101075839	101075996	158	159	101075885	31	7.7623	2.95097	4.69388	Adipose_R2_peak_	Promoter (2-3	11	1.01E+08	1.01E+08	2095	1	15485	uc007lnb.1	-2415	ENSMUSG000	Hsd17b1	hydroxysteroid (17-beta) dehydrogenase 1
chr11	101077831	101078116	286	287	101077959	26	5.31252	2.48988	2.47999	Adipose_R2_peak_	Promoter (<=	11	1.01E+08	1.01E+08	2095	1	15485	uc007lnb.1	-295	ENSMUSG000	Hsd17b1	hydroxysteroid (17-beta) dehydrogenase 1
chr11	101079848	101079983	136	137	101079961	18	6.42934	3.41502	3.48999	Adipose_R1_peak_	Promoter (1-2	11	1.01E+08	1.01E+08	2095	1	15485	uc007lnb.1	1437	ENSMUSG000	Hsd17b1	hydroxysteroid (17-beta) dehydrogenase 1
chr11	101080499	101080659	161	162	101080551	27	5.77405	2.5821	2.88921	Adipose_R2_peak_	Promoter (1-2	11	1.01E+08	1.01E+08	4055	1	71743	uc007lnc.2	-1906	ENSMUSG000	Coasy	Coenzyme A synthase
chr11	101083202	101083376	175	176	101083334	21	5.7033	2.8802	2.88138	Adipose_R1_peak_	Promoter (<=	11	1.01E+08	1.01E+08	4004	1	71743	uc007lnd.2	586	ENSMUSG000	Coasy	Coenzyme A synthase
chr11	101083692	101083790	99	100	101083735	25	4.86612	2.39766	2.09018	Adipose_R2_peak_	Promoter (1-2	11	1.01E+08	1.01E+08	4004	1	71743	uc007lnd.2	1076	ENSMUSG000	Coasy	Coenzyme A synthase
chr11	101083997	101084140	144	145	101084062	29	6.74062	2.76653	3.75989	Adipose_R2_peak_	Promoter (1-2	11	1.01E+08	1.01E+08	4004	1	71743	uc007lnd.2	1381	ENSMUSG000	Coasy	Coenzyme A synthase
chr11	101084577	101084788	212	213	101084676	30	7.24477	2.85875	4.21917	Adipose_R2_peak_	Promoter (1-2	11	1.01E+08	1.01E+08	4004	1	71743	uc007lnd.2	1961	ENSMUSG000	Coasy	Coenzyme A synthase
chr11	101739989	101740595	607	608	101740180	69	27.0941	4.89492	23.27763	Adipose_R2_peak_	Intron (uc007l	11	1.02E+08	1.02E+08	34402	1	217207	uc007lpr.1	7033	ENSMUSG000	Dhx8	DEAH (Asp-Glu-Ala-His) box polypeptide 8
chr11	101739999	101740463	465	466	101740164	39	12.35437	3.69188	9.09049	Adipose_R1_peak_	Intron (uc007l	11	1.02E+08	1.02E+08	34402	1	217207	uc007lpr.1	7043	ENSMUSG000	Dhx8	DEAH (Asp-Glu-Ala-His) box polypeptide 8
chr11	102217242	102217394	153	154	102217308	27	5.77405	2.5821	2.88921	Adipose_R2_peak_	Intron (uc007l	11	1.02E+08	1.02E+08	111	2	1.02E+08	uc033gai.1	12756	ENSMUSG000	Mir8101	microRNA 8101
chr11	102220844	102221033	190	191	102220943	17	5.80341	3.23528	2.92378	Adipose_R1_peak_	Intron (uc007l	11	1.02E+08	1.02E+08	111	2	1.02E+08	uc033gai.1	9117	ENSMUSG000	Mir8101	microRNA 8101
chr11	102222827	102223042	216	217	102222936	26	5.31252	2.48988	2.47999	Adipose_R2_peak_	Intron (uc007l	11	1.02E+08	1.02E+08	111	2	1.02E+08	uc033gai.1	7108	ENSMUSG000	Mir8101	microRNA 8101
chr11	102285757	102285960	204	205	102285842	27	5.77405	2.5821	2.88921	Adipose_R2_peak_	Promoter (<=	11	1.02E+08	1.02E+08	4489	1	72053	uc007lrd.2	818	ENSMUSG000	Tmub2	transmembrane and ubiquitin-like domain containing 2
chr11	102285803	102285958	156	157	102285827	17	5.80341	3.23528	2.92378	Adipose_R1_peak_	Promoter (<=	11	1.02E+08	1.02E+08	4489	1	72053	uc007lrd.2	864	ENSMUSG000	Tmub2	transmembrane and ubiquitin-like domain containing 2
chr11	102286292	102286559	268	269	102286422	31	7.7623	2.95097	4.69388	Adipose_R2_peak_	Promoter (1-2	11	1.02E+08	1.02E+08	4489	1	72053	uc007lrd.2	1353	ENSMUSG000	Tmub2	transmembrane and ubiquitin-like domain containing 2
chr11	102287997	102288335	339	340	102288253	26	5.31252	2.48988	2.47999	Adipose_R2_peak_	Exon (uc007lrt	11	1.02E+08	1.02E+08	4489	1	72053	uc007lrd.2	3058	ENSMUSG000	Tmub2	transmembrane and ubiquitin-like domain containing 2
chr11	102290399	102290581	183	184	102290479	31	7.7623	2.95097	4.69388	Adipose_R2_peak_	Promoter (2-3	11	1.02E+08	1.02E+08	4145	2	217218	uc007lre.1	2863	ENSMUSG000	Atxn7l3	ataxin 7-like 3
chr11	102292296	102292431	136	137	102292333	29	6.74062	2.76653	3.75989	Adipose_R2_peak_	Promoter (1-2	11	1.02E+08	1.02E+08	4145	2	217218	uc007lre.1	1013	ENSMUSG000	Atxn7l3	ataxin 7-like 3
chr11	102294168	102294362	195	196	102294203	27	5.77405	2.5821	2.88921	Adipose_R2_peak_	Promoter (<=	11	1.02E+08	1.02E+08	5760	2	217218	uc007lrf.1	697	ENSMUSG000	Atxn7l3	ataxin 7-like 3
chr11	102294513	102294757	245	246	102294662	29	6.74062	2.76653	3.75989	Adipose_R2_peak_	Promoter (<=	11	1.02E+08	1.02E+08	5760	2	217218	uc007lrf.1	302	ENSMUSG000	Atxn7l3	ataxin 7-like 3
chr11	102298034	102298242	209	210	102298195	30	7.24477	2.85875	4.21917	Adipose_R2_peak_	Promoter (1-2	11	1.02E+08	1.02E+08	7330	2	217218	uc007lrg.1	-1405	ENSMUSG000	Atxn7l3	ataxin 7-like 3
chr11	102298755	102299001	247	248	102298889	19	7.07687	3.59476	4.08458	Adipose_R1_peak_	Promoter (2-3	11	1.02E+08	1.02E+08	7330	2	217218	uc007lrg.1	-2126	ENSMUSG000	Atxn7l3	ataxin 7-like 3
chr11	102298830	102298974	145	146	102298839	25	4.86612	2.39766	2.09018	Adipose_R2_peak_	Promoter (2-3	11	1.02E+08	1.02E+08	7330	2	217218	uc007lrg.1	-2201	ENSMUSG000	Atxn7l3	ataxin 7-like 3
chr11	102300923	102301029	107	108	102300981	26	5.31252	2.48988	2.47999	Adipose_R2_peak_	Distal Interger	11	1.02E+08	1.02E+08	7330	2	217218	uc007lrg.1	-4294	ENSMUSG000	Atxn7l3	ataxin 7-like 3
chr11	102301462	102301673	212	213	102301501	28	6.25023	2.67431	3.31626	Adipose_R2_peak_	Downstream (	11	1.02E+08	1.02E+08	7330	2	217218	uc007lrg.1	-4833	ENSMUSG000	Atxn7l3	ataxin 7-like 3
chr11	102306666	102306878	213	214	102306700	30	7.24477	2.85875	4.21917	Adipose_R2_peak_	Exon (uc007lri.	11	1.02E+08	1.02E+08	8931	2	21429	uc007lri.2	6615	ENSMUSG000	Ubtf	upstream binding transcription factor, RNA polymerase I
chr11	102307360	102307603	244	245	102307495	29	6.74062	2.76653	3.75989	Adipose_R2_peak_	Intron (uc007l	11	1.02E+08	1.02E+08	8931	2	21429	uc007lri.2	5890	ENSMUSG000	Ubtf	upstream binding transcription factor, RNA polymerase I
chr11	102313004	102313118	115	116	102313085	29	6.74062	2.76653	3.75989	Adipose_R2_peak_	Promoter (<=	11	1.02E+08	1.02E+08	8931	2	21429	uc007lri.2	375	ENSMUSG000	Ubtf	upstream binding transcription factor, RNA polymerase I
chr11	102314197	102314495	299	300	102314279	27	5.77405	2.5821	2.88921	Adipose_R2_peak_	Promoter (<=	11	1.02E+08	1.02E+08	8931	2	21429	uc007lri.2	-704	ENSMUSG000	Ubtf	upstream binding transcription factor, RNA polymerase I
chr11	102314388	102314588	201	202	102314497	19	7.07687	3.59476	4.08458	Adipose_R1_peak_	Promoter (<=	11	1.02E+08	1.02E+08	8931	2	21429	uc007lri.2	-895	ENSMUSG000	Ubtf	upstream binding transcription factor, RNA polymerase I
chr11	102314806	102315020	215	216	102314890	32	8.29282	3.04319	5.18352	Adipose_R2_peak_	Promoter (1-2	11	1.02E+08	1.02E+08	8931	2	21429	uc007lri.2	-1313	ENSMUSG000	Ubtf	upstream binding transcription factor, RNA polymerase I
chr11	102314887	102315043	157	158	102314963	22	9.13959	4.13397	6.01526	Adipose_R1_peak_	Promoter (1-2	11	1.02E+08	1.02E+08	8931	2	21429	uc007lri.2	-1394	ENSMUSG000	Ubtf	upstream binding transcription factor, RNA polymerase I
chr11	102315213	102315600	388	389	102315248	28	6.25023	2.67431	3.31626	Adipose_R2_peak_	Promoter (<=	11	1.02E+08	1.02E+08	11911	2	21429	uc007lrj.2	873	ENSMUSG000	Ubtf	upstream binding transcription factor, RNA polymerase I
chr11	102316035	102316391	357	358	102316140	31	7.7623	2.95097	4.69388	Adipose_R2_peak_	Promoter (<=	11	1.02E+08	1.02E+08	11911	2	21429	uc007lrj.2	82	ENSMUSG000	Ubtf	upstream binding transcription factor, RNA polymerase I
chr11	103013987	103014136	150	151	103014088	18	6.42934	3.41502	3.48999	Adipose_R1_peak_	Intron (uc007l	11	1.03E+08	1.03E+08	23092	2	68087	uc007ltc.1	3011	ENSMUSG000	Dcakd	dephospho-CoA kinase domain containing
chr11	103017250	103017476	227	228	103017330	20	7.74501	3.7745	4.70467	Adipose_R1_peak_	Promoter (<=	11	1.03E+08	1.03E+08	23092	2	68087	uc007ltc.1	-103	ENSMUSG000	Dcakd	dephospho-CoA kinase domain containing
chr11	103022063	103022266	204	205	103022132	29	6.74062	2.76653	3.75989	Adipose_R2_peak_	Distal Interger	11	1.03E+08	1.03E+08	23092	2	68087	uc007ltc.1	-4916	ENSMUSG000	Dcakd	dephospho-CoA kinase domain containing
chr11	103025490	103025603	114	115	103025533	28	6.25023	2.67431	3.31626	Adipose_R2_peak_	Promoter (2-3	11	1.03E+08	1.03E+08	37544	1	18107	uc007ltd.1	-2959	ENSMUSG000	Nmt1	N-myristoyltransferase 1
chr11	103028626	103028773	148	149	103028695	22	9.13959	4.13397	6.01526	Adipose_R1_peak_	Promoter (<=	11	1.03E+08	1.03E+08	37544	1	18107	uc007ltd.1	64	ENSMUSG000	Nmt1	N-myristoyltransferase 1

chr11	103048615	103048769	155	156	103048695	18	6.42934	3.41502	3.48999	Adipose_R1_peak_	Intron (uc007l	11	1.03E+08	1.03E+08	37544	1	18107	uc007ltd.1	20053	ENSMUSG000	Nmt1	N-myristoyltransferase 1
chr11	103111356	103111536	181	182	103111508	18	6.42934	3.41502	3.48999	Adipose_R1_peak_	Intron (uc007l	11	1.03E+08	1.03E+08	3400	1	192231	uc007lto.1	-4789	ENSMUSG000	Hexim1	hexamethylene bis-acetamide inducible 1
chr11	103113363	103113494	132	133	103113417	17	5.76977	3.22034	2.92378	Adipose_R1_peak_	Promoter (2-3	11	1.03E+08	1.03E+08	3400	1	192231	uc007lto.1	-2831	ENSMUSG000	Hexim1	hexamethylene bis-acetamide inducible 1
chr11	103114049	103114156	108	109	103114094	29	6.74062	2.76653	3.75989	Adipose_R2_peak_	Promoter (2-3	11	1.03E+08	1.03E+08	3400	1	192231	uc007lto.1	-2169	ENSMUSG000	Hexim1	hexamethylene bis-acetamide inducible 1
chr11	103123021	103123174	154	155	103123098	27	5.77405	2.5821	2.88921	Adipose_R2_peak_	Distal Intergen	11	1.03E+08	1.03E+08	3400	1	192231	uc007lto.1	6696	ENSMUSG000	Hexim1	hexamethylene bis-acetamide inducible 1
chr11	103938643	103938815	173	174	103938724	20	7.74501	3.7745	4.70467	Adipose_R1_peak_	Intron (uc007l	11	1.04E+08	1.04E+08	132274	2	18195	uc007lvt.1	15241	ENSMUSG000	Nsf	N-ethylmaleimide sensitive fusion protein
chr11	103959868	103960205	338	339	103960191	27	5.77405	2.5821	2.88921	Adipose_R2_peak_	Distal Intergen	11	1.04E+08	1.04E+08	132274	2	18195	uc007lvt.1	-5812	ENSMUSG000	Nsf	N-ethylmaleimide sensitive fusion protein
chr11	104494736	104494875	140	141	104494800	39	22.07031	6.66749	18.52316	Adipose_R1_peak_	Distal Intergen	11	1.04E+08	1.04E+08	114859	2	76719	uc007lwn.1	-25302	ENSMUSG000	Kansl1	KAT8 regulatory NSL complex subunit 1
chr11	104494738	104494871	134	135	104494802	37	11.12817	3.50427	7.83613	Adipose_R2_peak_	Distal Intergen	11	1.04E+08	1.04E+08	114859	2	76719	uc007lwn.1	-25304	ENSMUSG000	Kansl1	KAT8 regulatory NSL complex subunit 1
chr11	104675289	104675459	171	172	104675351	18	6.42934	3.41502	3.48999	Adipose_R1_peak_	Distal Intergen	11	1.05E+08	1.05E+08	33085	1	16416	uc007lwx.2	37902	ENSMUSG000	Itgb3	integrin beta 3
chr11	104811710	104811968	259	260	104811838	44	28.34544	8.08821	24.66239	Adipose_R1_peak_	Distal Intergen	11	1.05E+08	1.05E+08	33085	1	16416	uc007lwx.2	174323	ENSMUSG000	Itgb3	integrin beta 3
chr11	104811758	104811963	206	207	104811848	51	20.44827	4.79532	16.78434	Adipose_R2_peak_	Distal Intergen	11	1.05E+08	1.05E+08	33085	1	16416	uc007lwx.2	174371	ENSMUSG000	Itgb3	integrin beta 3
chr11	104860333	104860571	239	240	104860435	42	26.36523	7.72873	22.71906	Adipose_R1_peak_	Distal Intergen	11	1.05E+08	1.05E+08	39633	1	70894	uc007lwy.1	-206264	ENSMUSG000	Efcab3	EF-hand calcium binding domain 3
chr11	104860349	104860568	220	221	104860435	38	11.72962	3.59649	8.4047	Adipose_R2_peak_	Distal Intergen	11	1.05E+08	1.05E+08	39633	1	70894	uc007lwy.1	-206267	ENSMUSG000	Efcab3	EF-hand calcium binding domain 3
chr11	106784120	106784213	94	95	106784161	25	4.86612	2.39766	2.09018	Adipose_R2_peak_	Promoter (<=	11	1.07E+08	1.07E+08	3672	2	13207	uc007lzp.1	-93	ENSMUSG000	Ddx5	DEAD (Asp-Glu-Ala-Asp) box polypeptide 5
chr11	106788064	106788212	149	150	106788099	17	5.80341	3.23528	2.92378	Adipose_R1_peak_	Promoter (<=	11	1.07E+08	1.07E+08	8139	2	13207	uc007lzq.1	282	ENSMUSG000	Ddx5	DEAD (Asp-Glu-Ala-Asp) box polypeptide 5
chr11	106788726	106788881	156	157	106788796	20	7.74501	3.7745	4.70467	Adipose_R1_peak_	Promoter (<=	11	1.07E+08	1.07E+08	8510	2	13207	uc007lzr.1	0	ENSMUSG000	Ddx5	DEAD (Asp-Glu-Ala-Asp) box polypeptide 5
chr11	109011647	109012118	472	473	109011926	481	116.4166	3.52341	112.0144	Adipose_R1_peak_	Distal Intergen	11	1.09E+08	1.09E+08	7161	1	12006	uc007mbv.1	68026	ENSMUSG000	Axin2	axin 2
chr11	109011648	109012116	469	470	109011925	775	328.4345	6.0315	323.537	Adipose_R2_peak_	Distal Intergen	11	1.09E+08	1.09E+08	7161	1	12006	uc007mbv.1	68027	ENSMUSG000	Axin2	axin 2
chr11	109283783	109284052	270	271	109283931	45	29.34988	8.26794	25.64927	Adipose_R1_peak_	Intron (uc007r	11	1.09E+08	1.09E+08	72827	2	19739	uc007mbx.1	14129	ENSMUSG000	Rgs9	regulator of G-protein signaling 9
chr11	109283795	109284043	249	250	109283923	44	15.55653	4.1498	12.05625	Adipose_R2_peak_	Intron (uc007r	11	1.09E+08	1.09E+08	72827	2	19739	uc007mbx.1	14138	ENSMUSG000	Rgs9	regulator of G-protein signaling 9
chr11	111187564	111187828	265	266	111187692	52	36.63102	9.52611	32.81188	Adipose_R1_peak_	Distal Intergen	11	1.11E+08	1.11E+08	10662	1	16518	uc007mdw.2	121400	ENSMUSG000	Kcnj2	potassium inwardly-rectifying channel, subfamily J, member 2
chr11	111187580	111187783	204	205	111187697	47	17.60017	4.42645	14.02531	Adipose_R2_peak_	Distal Intergen	11	1.11E+08	1.11E+08	10662	1	16518	uc007mdw.2	121416	ENSMUSG000	Kcnj2	potassium inwardly-rectifying channel, subfamily J, member 2
chr11	111193850	111194081	232	233	111193966	24	10.60668	4.49345	7.40961	Adipose_R1_peak_	Distal Intergen	11	1.11E+08	1.11E+08	10662	1	16518	uc007mdw.2	127686	ENSMUSG000	Kcnj2	potassium inwardly-rectifying channel, subfamily J, member 2
chr11	111250774	111250947	174	175	111250864	21	8.43286	3.95423	5.34896	Adipose_R1_peak_	Distal Intergen	11	1.11E+08	1.11E+08	10662	1	16518	uc007mdw.2	184610	ENSMUSG000	Kcnj2	potassium inwardly-rectifying channel, subfamily J, member 2
chr11	111547471	111547647	177	178	111547524	19	7.07687	3.59476	4.08458	Adipose_R1_peak_	Distal Intergen	11	1.11E+08	1.11E+08	10662	1	16518	uc007mdw.2	481307	ENSMUSG000	Kcnj2	potassium inwardly-rectifying channel, subfamily J, member 2
chr11	115014633	115015036	404	405	115014734	32	8.29282	3.04319	5.18352	Adipose_R2_peak_	Distal Intergen	11	1.15E+08	1.15E+08	6245	2	1.00E+08	uc007mgg.3	11897	ENSMUSG000	Cd300ld5	CD300 molecule like family member D5
chr11	115015230	115015389	160	161	115015269	31	7.46713	2.87078	4.43294	Adipose_R2_peak_	Distal Intergen	11	1.15E+08	1.15E+08	6245	2	1.00E+08	uc007mgg.3	11544	ENSMUSG000	Cd300ld5	CD300 molecule like family member D5
chr11	115015538	115015904	367	368	115015774	33	8.1707	2.96008	5.08736	Adipose_R2_peak_	Distal Intergen	11	1.15E+08	1.15E+08	6245	2	1.00E+08	uc007mgg.3	11029	ENSMUSG000	Cd300ld5	CD300 molecule like family member D5
chr11	115016558	115017768	1211	1212	115016605	33	7.45652	2.77692	4.42341	Adipose_R2_peak_	Downstream (	11	1.15E+08	1.15E+08	6245	2	1.00E+08	uc007mgg.3	9165	ENSMUSG000	Cd300ld5	CD300 molecule like family member D5
chr11	115017558	115017708	151	152	115017601	22	6.07344	2.94786	3.18937	Adipose_R1_peak_	Downstream (	11	1.15E+08	1.15E+08	6245	2	1.00E+08	uc007mgg.3	9225	ENSMUSG000	Cd300ld5	CD300 molecule like family member D5
chr11	115018004	115018136	133	134	115018059	22	6.87195	3.2388	3.92638	Adipose_R1_peak_	Downstream (	11	1.15E+08	1.15E+08	6245	2	1.00E+08	uc007mgg.3	8797	ENSMUSG000	Cd300ld5	CD300 molecule like family member D5
chr11	115018111	115018280	170	171	115018192	28	6.25023	2.67431	3.31626	Adipose_R2_peak_	Downstream (	11	1.15E+08	1.15E+08	6245	2	1.00E+08	uc007mgg.3	8653	ENSMUSG000	Cd300ld5	CD300 molecule like family member D5
chr11	115018453	115018626	174	175	115018569	29	6.74062	2.76653	3.75989	Adipose_R2_peak_	Downstream (	11	1.15E+08	1.15E+08	6245	2	1.00E+08	uc007mgg.3	8307	ENSMUSG000	Cd300ld5	CD300 molecule like family member D5
chr11	115018802	115018987	186	187	115018931	27	5.77405	2.5821	2.88921	Adipose_R2_peak_	Downstream (	11	1.15E+08	1.15E+08	6245	2	1.00E+08	uc007mgg.3	7946	ENSMUSG000	Cd300ld5	CD300 molecule like family member D5
chr11	115019357	115019559	203	204	115019435	32	8.29282	3.04319	5.18352	Adipose_R2_peak_	Downstream (	11	1.15E+08	1.15E+08	6245	2	1.00E+08	uc007mgg.3	7374	ENSMUSG000	Cd300ld5	CD300 molecule like family member D5
chr11	115019718	115019885	168	169	115019808	30	6.96253	2.78106	3.97376	Adipose_R2_peak_	Downstream (	11	1.15E+08	1.15E+08	6245	2	1.00E+08	uc007mgg.3	7048	ENSMUSG000	Cd300ld5	CD300 molecule like family member D5
chr11	115020135	115020389	255	256	115020213	28	5.45352	2.4509	2.61564	Adipose_R2_peak_	Downstream (	11	1.15E+08	1.15E+08	6245	2	1.00E+08	uc007mgg.3	6544	ENSMUSG000	Cd300ld5	CD300 molecule like family member D5
chr11	115021355	115021713	359	360	115021475	40	10.84615	3.26919	7.57832	Adipose_R2_peak_	Intron (uc007r	11	1.15E+08	1.15E+08	6245	2	1.00E+08	uc007mgg.3	5220	ENSMUSG000	Cd300ld5	CD300 molecule like family member D5
chr11	115022892	115023259	368	369	115023096	30	7.24477	2.85875	4.21917	Adipose_R2_peak_	Intron (uc007r	11	1.15E+08	1.15E+08	6245	2	1.00E+08	uc007mgg.3	3674	ENSMUSG000	Cd300ld5	CD300 molecule like family member D5
chr11	115023447	115024223	777	778	115023513	41	13.59774	3.87315	10.1803	Adipose_R2_peak_	Promoter (2-3	11	1.15E+08	1.15E+08	6245	2	1.00E+08	uc007mgg.3	2710	ENSMUSG000	Cd300ld5	CD300 molecule like family member D5
chr11	115024401	115024634	234	235	115024411	27	5.77405	2.5821	2.88921	Adipose_R2_peak_	Promoter (2-3	11	1.15E+08	1.15E+08	6245	2	1.00E+08	uc007mgg.3	2299	ENSMUSG000	Cd300ld5	CD300 molecule like family member D5
chr11	115031418	115031576	159	160	115031483	24	10.60668	4.49345	7.40961	Adipose_R1_peak_	3' UTR	11	1.15E+08	1.15E+08	6245	2	1.00E+08	uc007mgg.3	-4485	ENSMUSG000	Cd300ld5	CD300 molecule like family member D5
chr11	115611456	115611616	161	162	115611478	26	5.31252	2.48988	2.47999	Adipose_R2_peak_	Promoter (<=	11	1.16E+08	1.16E+08	4585	2	69674	uc007mib.1	887	ENSMUSG000	Mif4gd	MIF4G domain containing
chr11	115616732	115616933	202	203	115616831	21	8.43286	3.95423	5.34896	Adipose_R1_peak_	Promoter (1-2	11	1.16E+08	1.16E+08	3629	2	69674	uc007mif.1	-1201	ENSMUSG000	Mif4gd	MIF4G domain containing
chr11	115619381	115619543	163	164	115619404	28	6.25023	2.67431	3.31626	Adipose_R2_peak_	Intron (uc007r	11	1.16E+08	1.16E+08	3629	2	69674	uc007mif.1	-3850	ENSMUSG000	Mif4gd	MIF4G domain containing
chr11	115766303	115766444	142	143	115766366	20	7.74501	3.7745	4.70467	Adipose_R1_peak_	Promoter (<=	11	1.16E+08	1.16E+08	29984	1	71947	uc007mim.1	861	ENSMUSG000	Tmem94	transmembrane protein 94
chr11	115772193	115772450	258	259	115772324	22	9.13959	4.13397	6.01526	Adipose_R1_peak_	Intron (uc007r	11	1.16E+08	1.16E+08	29984	1	71947	uc007mim.1	6751	ENSMUSG000	Tmem94	transmembrane protein 94
chr11	115773696	115773829	134	135	115773767	27	5.77405	2.5821	2.88921	Adipose_R2_peak_	Intron (uc007r	11	1.16E+08	1.16E+08	29984	1	71947	uc007mim.1	8254	ENSMUSG000	Tmem94	transmembrane protein 94
chr11	115790615	115790785	171	172	115790679	19	7.07687	3.59476	4.08458	Adipose_R1_peak_	Promoter (2-3	11	1.16E+08	1.16E+08	1671	1	71947	uc007min.1	-2989	ENSMUSG000	Tmem94	transmembrane protein 94
chr11	115790681	115790785	105	106	115790734	30	7.24477	2.85875	4.21917	Adipose_R2_peak_	Promoter (2-3	11	1.16E+08	1.16E+08	1671	1	71947	uc007min.1	-2989	ENSMUSG000	Tmem94	transmembrane protein 94
chr11	116025194	116025440	247	248	116025299	27	5.77405	2.5821	2.88921	Adipose_R2_peak_	Promoter (<=	11	1.16E+08	1.16E+08	2544	2	15081	uc007mjo.1	-690	ENSMUSG000	H3f3b	H3 histone, family 3B
chr11	116025742	116025906	165	166	116025853	31	7.7623	2.95097	4.69388	Adipose_R2_peak_	Promoter (1-2	11	1.16E+08	1.16E+08	2544	2	15081	uc007mjo.1	-1238	ENSMUSG000	H3f3b	H3 histone, family 3B
chr11	116026552	116026762	211	212	116026657	30	7.24477	2.85875	4.21917	Adipose_R2_peak_	Promoter (2-3	11	1.16E+08	1.16E+08	2544	2	15081	uc007mjo.1	-2048	ENSMUSG000	H3f3b	H3 histone, family 3B
chr11	116027147	116027573	427	428	116027227	30	7.24477	2.85875	4.21917	Adipose_R2_peak_	Promoter (2-3	11	1.16E+08	1.16E+08	2544	2	15081	uc007mjo.1	-2643	ENSMUSG000	H3f3b	H3 histone, family 3B
chr11	116027154	116027414	261	262	116027208	18	6.42934	3.41502	3.48999	Adipose_R1_peak_	Promoter (2-3	11	1.16E+08	1.16E+08	2544	2	15081	uc007mjo.1	-2650	ENSMUSG000	H3f3b	H3 histone, family 3B
chr11	116027791	116028208	418	419	116028112	31	7.7623	2.95097	4.69388	Adipose_R2_peak_	Promoter (2-3	11	1.16E+08	1.16E+08	30899	1	217331	uc007mjp.2	-2108	ENSMUSG000	Unk	unkempt family zinc finger
chr11	116027848	116028444	597	598	116028348	30	15.39182	5.57188	12.01394	Adipose_R1_peak_	Promoter (1-2	11	1.16E+08	1.16E+08	30899	1	217331	uc007mjp.2	-1872	ENSMUSG000	Unk	unkempt family zinc finger
chr11	116028518	116028854	337	338	116028667	29	6.74062	2.76653	3.75989	Adipose_R2_peak_	Promoter (1-2	11	1.16E+08	1.16E+08	30899	1	217331	uc007mjp.2	-1462	ENSMUSG000	Unk	unkempt family zinc finger
chr11	116029098	116029461	364	365	116029179	31	7.7623	2.95097	4.69388	Adipose_R2_peak_	Promoter (<=	11	1.16E+08	1.16E+08	30899	1	217331	uc007mjp.2	-855	ENSMUSG000	Unk	unkempt family zinc finger
chr11	116389600	116389732	133	134	116389669	33	8.83596	3.1354	5.68652	Adipose_R2_peak_	Intron (uc007r	11	1.16E+08	1.16E+08	76688	2	217340	uc007mky.2	23300	ENSMUSG000	Rnf157	ring finger protein 157
chr11	116413669	116413820	152	153	116413732	19	7.07687	3.59476	4.08458	Adipose_R1_peak_	Promoter (<=	11	1.16E+08	1.16E+08	76688	2	217340	uc007mky.2	-637	ENSMUSG000	Rnf157	ring finger protein 157
chr11	116415739	116415864	126	127	116415817	28	6.25023	2.67431	3.31626	Adipose_R2_peak_	Promoter (2-3	11	1.16E+08	1.16E+08	76688	2	217340	uc007mky.2	-2707	ENSMUSG000	Rnf157	ring finger protein 157
chr11	116848378	116848524	147	148	116848476	27	5.77405	2.5821	2.88921	Adipose_R2_peak_	Intron (uc007r	11	1.17E+08	1.17E+08	3198	2	20382	uc007mmn.2	4570	ENSMUSG000	Srsf2	serine/arginine-rich splicing factor 2
chr11	116848801	116848967	167	168	116848905	28	6.25023	2.67431	3.31626	Adipose_R2_peak_	Exon (uc007m	11	1.17E+08	1.17E+08	3198	2	20382	uc007mmn.2	4127	ENSMUSG000	Srsf2	serine/arginine-rich splicing factor 2
chr11	116850109	116850288	180	181	116850185	34	9.39139	3.22762	6.20474	Adipose_R2_peak_	Promoter (2-3	11	1.17E+08	1.17E+08	3198	2	20382	uc007mmn.2	2806	ENSMUSG000	Srsf2	serine/arginine-rich splicing factor 2
chr11	116851077	116851568	492	493	116851290	23	9.86443	4.31371	6.70247	Adipose_R1_peak_	Promoter (1-2	11	1.17E+08	1.17E+08	3198	2	20382	uc007mmn.2	1526	ENSMUSG000	Srsf2	serine/arginine-rich splicing factor 2
chr11	116851343	116851505	163	164	116851394	30	7.24477	2.85875	4.21917	Adipose_R2_peak_	Promoter (1-2	11	1.17E+08	1.17E+08	3198	2	20382	uc007mmn.2	1589	ENSMUSG000	Srsf2	serine/arginine-rich splicing factor 2
chr11	116851696	116852039	344	345	116851841	33	8.83596	3.1354	5.68652	Adipose_R2_peak_	Promoter (1-2	11	1.17E+08	1.17E+08	3198	2	20382	uc007mmn.2	1055	ENSMUSG000	Srsf2	serine/arginine-rich splicing factor 2
chr11	117067823	117067926	104	105	117067921	25	4.86612	2.39766	2.09018	Adipose_R2_peak_	Distal Intergen	11	1.17E+08	1.17E+08	2173	1	76972	uc007mmx.2	-8857	ENSMUSG000	Snhg20	small nucleolar RNA host gene 20
chr11	117067894	117068032	139	140	117067956	17	5.80341	3.23528	2.92378	Adipose_R1_peak_	Distal Intergen	11	1.17E+08	1.17E+08	2173	1	76972	uc007mmx.2	-8751	ENSMUSG000	Snhg20	small nucleolar RNA host gene 20
chr11	117108604	117108715	112	113	117108612	25	4.86612	2.39766	2.09018	Adipose_R2_peak_	Distal Intergen	11	1.17E+08	1.17E+08	13592	1	74136	uc007mmz.2	-6457	ENSMUSG000	Sec14l1	SEC14-like lipid binding 1
chr11	117108758	117108930	173	174	117108833	19	7.07687	3.59476	4.08458	Adipose_R1_peak_	Distal Intergen	11	1.17E+08	1.17E+08	13592	1	74136	uc007mmz.2	-6242	ENSMUSG000	Sec14l1	SEC14-like lipid binding 1
chr11	117111014	117111112	99	100	117111028	26	5.31252	2.48988	2.47999	Adipose_R2_peak_	Distal Intergen	11	1.17E+08	1.17E+08	13592	1	74136	uc007mmz.2	-4060	ENSMUSG000	Sec14l1	SEC14-like lipid binding 1
chr11	117111724	117111877	154	155	117111802	19	7.07687	3.59476	4.08458	Adipose_R1_peak_	Distal Intergen	11	1.17E+08	1.17E+08	13592	1	74136	uc007mmz.2	-3295	ENSMUSG000	Sec14l1	SEC14-like lipid binding 1
chr11	117113828	117114028	201	202	117113880	27	5.77405	2.5821	2.88921	Adipose_R2_peak_	Promoter (1-2	11	1.17E+08	1.17E+08	13592	1	74136	uc007mmz.2	-1144	ENSMUSG000	Sec14l1	SEC14-like lipid binding 1
chr11	117114372	117114539	168	169	117114417	28	6.25023	2.67431	3.31626	Adipose_R2_peak_	Promoter (<=	11	1.17E+08	1.17E+08	13592	1	74136	uc007mmz.2	-633	ENSMUSG000	Sec14l1	SEC14-like lipid binding 1
chr11	117114860	117114965	106	107	117114885	25	4.86612	2.39766	2.09018	Adipose_R2_peak_	Promoter (<=	11	1.17E+08	1.17E+08	13592	1	74136	uc007mmz.2	-207	ENSMUSG000	Sec14l1	SEC14-like lipid binding 1
chr11	117115990	117116187	198	199	117116098	31	7.7623	2.95097	4.69388	Adipose_R2_peak_	Promoter (<=	11	1.17E+08	1.17E+08	13592	1	74136	uc007mmz.2	818	ENSMUSG000	Sec14l1	SEC14-like lipid binding 1
chr11	117118293	117118388	96	97	117118342	27	5.77405	2.5821	2.88921	Adipose_R2_peak_	Intron (uc007r	11	1.17E+08	1.17E+08	13592	1	74136	uc007mmz.2	3121	ENSMUSG000	Sec14l1	SEC14-like lipid binding 1
chr11	117119075	117119226	152	153	117119139	18	6.42934	3.41502	3.48999	Adipose_R1_peak_	Intron (uc007r	11	1.17E+08	1.17E+08	13592	1	74136	uc007mmz.2	3903	ENSMUSG000	Sec14l1	SEC14-like lipid binding 1
chr11	117221825	117222042	218	219	117222029	21	8.43286	3.95423	5.34896	Adipose_R1_peak_	Intron (uc007r	11	1.17E+08	1.17E+08	162665	1	53860	uc007mnd.2	22164	ENSMUSG000	09-Sep	septin 9
chr11	117311975	117312083	109	110	117312040	25	4.86612	2.39766	2.09018	Adipose_R2_peak_	Intron (uc007r	11	1.17E+08	1.17E+08	30611	1	53860	uc007mnf.2	-19632	ENSMUSG000	09-Sep	septin 9
chr11	117947964	117948082	119	120	117948019	28	6.25023	2.67431	3.31626	Adipose_R2_peak_	Distal Intergen	11	1.18E+08	1.18E+08	3280	2	12702	uc007moi.2	21284	ENSMUSG000	Socs3	suppressor of cytokine signaling 3
chr11	117964127	117964288	162	163	117964183	17	5.80341	3.23528	2.92378	Adipose_R1_peak_	Downstream (	11	1.18E+08	1.18E+08	3280	2	12702	uc007moi.2	5078	ENSMUSG000	Socs3	suppressor of cytokine signaling 3
chr11	117966178	117966317	140	141	117966212	17	5.80341	3.23528	2.92378	Adipose_R1_peak_	3' UTR	11	1.18E+08	1.18E+08	3280	2	12702	uc007moi.2	3049	ENSMUSG000	Socs3	suppressor of cytokine signaling 3
chr11	118427621	118427744	124	125	118427667	31	7.7623	2.95097	4.69388	Adipose_R2_peak_	Promoter (<=	11	1.18E+08	1.18E+08	23326	1	56745	uc007mpg.2	-713	ENSMUSG000	C1qtnf1	C1q and tumor necrosis factor related protein 1
chr11	118428163	118428378	216	217	118428312	19	7.07687	3.59476	4.08458	Adipose_R1_peak_	Promoter (<=	11	1.18E+08	1.18E+08	23326	1	56745	uc007mpg.2	-79	ENSMUSG000	C1qtnf1	C1q and tumor necrosis factor related protein 1
chr11	119040267	119040411	145	146	119040356	28	6.25023	2.67431	3.31626	Adipose_R2_peak_	Promoter (<=	11	1.19E+08	1.19E+08	2478	2	30951	uc007mpu.1	502	ENSMUSG000	Cbx8	chromobox 8
chr11	119042719	119042862	144	145	119042747	25	4.86612	2.39766	2.09018	Adipose_R2_peak_	Promoter (1-2	11	1.19E+08	1.19E+08	2478	2	30951	uc007mpu.1	-1806	ENSMUSG000	Cbx8	chromobox 8
chr11	119043797	119043939	143	144	119043803	25	4.86612	2.39766	2.09018	Adipose_R2_peak_	Promoter (2-3	11	1.19E+08	1.19E+08	2478	2	30951	uc007mpu.1	-2884	ENSMUSG000	Cbx8	chromobox 8
chr11	119045737	119046063	327	328	119045934	26	5.31252	2.48988	2.47999	Adipose_R2_peak_	Distal Intergen	11	1.19E+08	1.19E+08	2478	2	30951	uc007mpu.1	-4824	ENSMUSG000	Cbx8	chromobox 8
chr11	119045930	119046236	307	308	119046071	20	7.57631	3.70277	4.57781	Adipose_R1_peak_	Distal Intergen	11	1.19E+08	1.19E+08	2478	2	30951	uc007mpu.1	-5017	ENSMUSG000	Cbx8	chromobox 8
chr11	119047059	119047155	97	98	119047136	28	6.25023	2.67431	3.31626	Adipose_R2_peak_	Distal Intergen	11	1.19E+08	1.19E+08	2478	2	30951	uc007mpu.1	-6146	ENSMUSG000	Cbx8	chromobox 8

chr11	119052318	119052483	166	167	119052391	25	4.86612	2.39766	2.09018	Adipose_R2_peak_	Distal Interger	11	1.19E+08	1.19E+08	2478	2	30951	uc007mpu.1	-11405	ENSMUSG000	Cbx8	chromobox 8
chr11	119075099	119075263	165	166	119075213	17	5.80341	3.23528	2.92378	Adipose_R1_peak_	Downstream (	11	1.19E+08	1.19E+08	5086	2	12418	uc007mpv.2	7393	ENSMUSG000	Cbx4	chromobox 4
chr11	119087033	119087320	288	289	119087104	40	12.96465	3.78093	9.57654	Adipose_R2_peak_	Promoter (<=	11	1.19E+08	1.19E+08	8667	2	12418	uc007mpw.2	-796	ENSMUSG000	Cbx4	chromobox 4
chr11	119087361	119087505	145	146	119087465	19	7.07687	3.59476	4.08458	Adipose_R1_peak_	Promoter (1-2	11	1.19E+08	1.19E+08	8667	2	12418	uc007mpw.2	-1124	ENSMUSG000	Cbx4	chromobox 4
chr11	119087969	119088187	219	220	119088059	30	7.24477	2.85875	4.21917	Adipose_R2_peak_	Promoter (1-2	11	1.19E+08	1.19E+08	8667	2	12418	uc007mpw.2	-1732	ENSMUSG000	Cbx4	chromobox 4
chr11	119113164	119113294	131	132	119113205	17	5.80341	3.23528	2.92378	Adipose_R1_peak_	Distal Interger	11	1.19E+08	1.19E+08	8667	2	12418	uc007mpw.2	-26927	ENSMUSG000	Cbx4	chromobox 4
chr11	119939020	119939136	117	118	119939026	25	4.86612	2.39766	2.09018	Adipose_R2_peak_	Intron (uc007r	11	1.20E+08	1.20E+08	59527	1	108100	uc007mrg.1	-3956	ENSMUSG000	Baiap2	brain-specific angiogenesis inhibitor 1-associated protein 2
chr11	119946485	119946650	166	167	119946510	17	5.56186	3.12859	2.74233	Adipose_R1_peak_	Intron (uc007r	11	1.20E+08	1.20E+08	59527	1	108100	uc007mrg.1	3393	ENSMUSG000	Baiap2	brain-specific angiogenesis inhibitor 1-associated protein 2
chr11	120087620	120087777	158	159	120087766	23	9.86443	4.31371	6.70247	Adipose_R1_peak_	Promoter (<=	11	1.20E+08	1.20E+08	22398	2	12009	uc007mrp.2	-793	ENSMUSG000	Cep131	centrosomal protein 131
chr11	120089646	120089865	220	221	120089786	28	6.25023	2.67431	3.31626	Adipose_R2_peak_	Promoter (2-3	11	1.20E+08	1.20E+08	22398	2	12009	uc007mrp.2	-2819	ENSMUSG000	Cep131	centrosomal protein 131
chr11	120091922	120092425	504	505	120092350	19	6.91816	3.52644	3.97188	Adipose_R1_peak_	Exon (uc007m	11	1.20E+08	1.20E+08	1436	2	78777	uc007mrt.1	4544	ENSMUSG000	Tepsin	TEPSIN, adaptor related protein complex 4 accessory protein
chr11	120093426	120093597	172	173	120093527	29	6.74062	2.76653	3.75989	Adipose_R2_peak_	Exon (uc007m	11	1.20E+08	1.20E+08	1436	2	78777	uc007mrt.1	3372	ENSMUSG000	Tepsin	TEPSIN, adaptor related protein complex 4 accessory protein
chr11	120094214	120094585	372	373	120094293	28	6.25023	2.67431	3.31626	Adipose_R2_peak_	Promoter (2-3	11	1.20E+08	1.20E+08	1436	2	78777	uc007mrt.1	2384	ENSMUSG000	Tepsin	TEPSIN, adaptor related protein complex 4 accessory protein
chr11	120097914	120098220	307	308	120098118	33	8.23553	2.97696	5.14931	Adipose_R2_peak_	Promoter (<=	11	1.20E+08	1.20E+08	8210	2	78777	uc007mrq.1	511	ENSMUSG000	Tepsin	TEPSIN, adaptor related protein complex 4 accessory protein
chr11	120098519	120098631	113	114	120098570	28	6.25023	2.67431	3.31626	Adipose_R2_peak_	Promoter (<=	11	1.20E+08	1.20E+08	8210	2	78777	uc007mrq.1	100	ENSMUSG000	Tepsin	TEPSIN, adaptor related protein complex 4 accessory protein
chr11	120172955	120173068	114	115	120173012	27	5.77405	2.5821	2.88921	Adipose_R2_peak_	Distal Interger	11	1.20E+08	1.20E+08	3207	2	1.00E+08	uc007msb.2	16788	NA	2810410L24Ri	RIKEN cDNA 2810410L24 gene
chr11	120189163	120189298	136	137	120189212	18	6.42934	3.41502	3.48999	Adipose_R1_peak_	Promoter (<=	11	1.20E+08	1.20E+08	3207	2	1.00E+08	uc007msb.2	558	NA	2810410L24Ri	RIKEN cDNA 2810410L24 gene
chr11	120204523	120205061	539	540	120204941	24	10.60668	4.49345	7.40961	Adipose_R1_peak_	Distal Interger	11	1.20E+08	1.20E+08	3207	2	1.00E+08	uc007msb.2	-14667	NA	2810410L24Ri	RIKEN cDNA 2810410L24 gene
chr11	120204600	120204924	325	326	120204709	32	8.29282	3.04319	5.18352	Adipose_R2_peak_	Distal Interger	11	1.20E+08	1.20E+08	3207	2	1.00E+08	uc007msb.2	-14744	NA	2810410L24Ri	RIKEN cDNA 2810410L24 gene
chr11	120228578	120228674	97	98	120228663	25	4.86612	2.39766	2.09018	Adipose_R2_peak_	Distal Interger	11	1.20E+08	1.20E+08	59351	1	268515	uc007msd.2	-4273	ENSMUSG000	Bahcc1	BAH domain and coiled-coil containing 1
chr11	120231754	120232044	291	292	120231852	18	6.42934	3.41502	3.48999	Adipose_R1_peak_	Promoter (<=	11	1.20E+08	1.20E+08	59351	1	268515	uc007msd.2	-903	ENSMUSG000	Bahcc1	BAH domain and coiled-coil containing 1
chr11	120231916	120232162	247	248	120231944	25	4.86612	2.39766	2.09018	Adipose_R2_peak_	Promoter (<=	11	1.20E+08	1.20E+08	59351	1	268515	uc007msd.2	-785	ENSMUSG000	Bahcc1	BAH domain and coiled-coil containing 1
chr11	120232231	120232669	439	440	120232514	23	9.86443	4.31371	6.70247	Adipose_R1_peak_	Promoter (<=	11	1.20E+08	1.20E+08	59351	1	268515	uc007msd.2	-278	ENSMUSG000	Bahcc1	BAH domain and coiled-coil containing 1
chr11	120233381	120233558	178	179	120233475	33	8.83596	3.1354	5.68652	Adipose_R2_peak_	Promoter (<=	11	1.20E+08	1.20E+08	59351	1	268515	uc007msd.2	434	ENSMUSG000	Bahcc1	BAH domain and coiled-coil containing 1
chr11	120235529	120235651	123	124	120235585	28	6.25023	2.67431	3.31626	Adipose_R2_peak_	Promoter (1-2	11	1.20E+08	1.20E+08	55599	1	268515	uc007mse.2	-1048	ENSMUSG000	Bahcc1	BAH domain and coiled-coil containing 1
chr11	120237752	120237969	218	219	120237843	29	6.74062	2.76653	3.75989	Adipose_R2_peak_	Promoter (1-2	11	1.20E+08	1.20E+08	55599	1	268515	uc007mse.2	1053	ENSMUSG000	Bahcc1	BAH domain and coiled-coil containing 1
chr11	120238399	120238520	122	123	120238424	27	5.77405	2.5821	2.88921	Adipose_R2_peak_	Promoter (1-2	11	1.20E+08	1.20E+08	55599	1	268515	uc007mse.2	1700	ENSMUSG000	Bahcc1	BAH domain and coiled-coil containing 1
chr11	120293590	120293684	95	96	120293616	27	5.77405	2.5821	2.88921	Adipose_R2_peak_	Distal Interger	11	1.20E+08	1.20E+08	17879	1	268515	uc007msf.2	19171	ENSMUSG000	Bahcc1	BAH domain and coiled-coil containing 1
chr11	120301016	120301360	345	346	120301223	27	12.93148	5.03266	9.63827	Adipose_R1_peak_	Distal Interger	11	1.20E+08	1.20E+08	17879	1	268515	uc007msf.2	26597	ENSMUSG000	Bahcc1	BAH domain and coiled-coil containing 1
chr11	120301142	120301331	190	191	120301204	26	5.31252	2.48988	2.47999	Adipose_R2_peak_	Distal Interger	11	1.20E+08	1.20E+08	17879	1	268515	uc007msf.2	26723	ENSMUSG000	Bahcc1	BAH domain and coiled-coil containing 1
chr11	120304152	120304369	218	219	120304231	30	7.24477	2.85875	4.21917	Adipose_R2_peak_	Distal Interger	11	1.20E+08	1.20E+08	17879	1	268515	uc007msf.2	29733	ENSMUSG000	Bahcc1	BAH domain and coiled-coil containing 1
chr11	120306551	120307013	463	464	120306614	22	7.22949	3.37302	4.2352	Adipose_R1_peak_	Distal Interger	11	1.20E+08	1.20E+08	17879	1	268515	uc007msf.2	32132	ENSMUSG000	Bahcc1	BAH domain and coiled-coil containing 1
chr11	120306568	120306672	105	106	120306622	34	6.4319	2.48838	3.49109	Adipose_R2_peak_	Distal Interger	11	1.20E+08	1.20E+08	17879	1	268515	uc007msf.2	32149	ENSMUSG000	Bahcc1	BAH domain and coiled-coil containing 1
chr11	120307745	120308242	498	499	120307894	20	6.70973	3.34437	3.76643	Adipose_R1_peak_	Distal Interger	11	1.20E+08	1.20E+08	17879	1	268515	uc007msf.2	33326	ENSMUSG000	Bahcc1	BAH domain and coiled-coil containing 1
chr11	120307896	120308176	281	282	120308081	37	8.13171	2.77623	5.04936	Adipose_R2_peak_	Distal Interger	11	1.20E+08	1.20E+08	17879	1	268515	uc007msf.2	33477	ENSMUSG000	Bahcc1	BAH domain and coiled-coil containing 1
chr11	120308410	120309196	787	788	120308850	38	8.75673	2.87943	5.63008	Adipose_R2_peak_	Distal Interger	11	1.20E+08	1.20E+08	17879	1	268515	uc007msf.2	33991	ENSMUSG000	Bahcc1	BAH domain and coiled-coil containing 1
chr11	120308604	120309021	418	419	120308669	20	7.04819	3.48236	4.08458	Adipose_R1_peak_	Distal Interger	11	1.20E+08	1.20E+08	17879	1	268515	uc007msf.2	34185	ENSMUSG000	Bahcc1	BAH domain and coiled-coil containing 1
chr11	120309435	120310115	681	682	120309498	25	10.1118	4.17662	6.94766	Adipose_R1_peak_	Distal Interger	11	1.20E+08	1.20E+08	17879	1	268515	uc007msf.2	35016	ENSMUSG000	Bahcc1	BAH domain and coiled-coil containing 1
chr11	120309477	120309674	198	199	120309565	29	5.30559	2.37483	2.47999	Adipose_R2_peak_	Distal Interger	11	1.20E+08	1.20E+08	17879	1	268515	uc007msf.2	35058	ENSMUSG000	Bahcc1	BAH domain and coiled-coil containing 1
chr11	120311940	120312192	253	254	120312049	32	6.97016	2.69525	3.98059	Adipose_R2_peak_	Distal Interger	11	1.20E+08	1.20E+08	64	2	1.02E+08	uc033gbw.1	35221	ENSMUSG000	Mir6935	microRNA 6935
chr11	120312573	120312785	213	214	120312664	34	8.60688	3.02348	5.48556	Adipose_R2_peak_	Distal Interger	11	1.20E+08	1.20E+08	64	2	1.02E+08	uc033gbw.1	34628	ENSMUSG000	Mir6935	microRNA 6935
chr11	120314682	120314921	240	241	120314843	19	6.80057	3.47621	3.85596	Adipose_R1_peak_	Distal Interger	11	1.20E+08	1.20E+08	64	2	1.02E+08	uc033gbw.1	32492	ENSMUSG000	Mir6935	microRNA 6935
chr11	120315173	120315449	277	278	120315226	21	8.37589	3.9302	5.32888	Adipose_R1_peak_	Distal Interger	11	1.20E+08	1.20E+08	64	2	1.02E+08	uc033gbw.1	31964	ENSMUSG000	Mir6935	microRNA 6935
chr11	120315700	120316141	442	443	120315877	24	10.58239	4.48347	7.40961	Adipose_R1_peak_	Distal Interger	11	1.20E+08	1.20E+08	64	2	1.02E+08	uc033gbw.1	31272	ENSMUSG000	Mir6935	microRNA 6935
chr11	120342195	120342343	149	150	120342222	17	5.80341	3.23528	2.92378	Adipose_R1_peak_	Distal Interger	11	1.20E+08	1.20E+08	64	2	1.02E+08	uc033gbw.1	5070	ENSMUSG000	Mir6935	microRNA 6935
chr11	120577032	120577444	413	414	120577086	28	6.11308	2.63536	3.21535	Adipose_R2_peak_	3' UTR	11	1.21E+08	1.21E+08	4013	2	192662	uc007mtj.1	3803	ENSMUSG000	Arhgdia	Rho GDP dissociation inhibitor (GDI) alpha
chr11	120578903	120579339	437	438	120579212	30	6.96253	2.78106	3.97376	Adipose_R2_peak_	Promoter (1-2	11	1.21E+08	1.21E+08	4013	2	192662	uc007mtj.1	1908	ENSMUSG000	Arhgdia	Rho GDP dissociation inhibitor (GDI) alpha
chr11	120580550	120580690	141	142	120580575	16	5.17209	3.04286	2.38734	Adipose_R1_peak_	Promoter (<=	11	1.21E+08	1.21E+08	4013	2	192662	uc007mtj.1	557	ENSMUSG000	Arhgdia	Rho GDP dissociation inhibitor (GDI) alpha
chr11	121181711	121181882	172	173	121181825	21	5.53726	2.81969	2.71836	Adipose_R1_peak_	Intron (uc007r	11	1.21E+08	1.21E+08	1703	1	217369	uc007mvj.1	21440	ENSMUSG000	Uts2r	urotensin 2 receptor
chr11	121181723	121181954	232	233	121181850	37	11.12817	3.50427	7.83613	Adipose_R2_peak_	Intron (uc007r	11	1.21E+08	1.21E+08	1703	1	217369	uc007mvj.1	21452	ENSMUSG000	Uts2r	urotensin 2 receptor
chr11	121239742	121239913	172	173	121239855	28	6.25023	2.67431	3.31626	Adipose_R2_peak_	Promoter (2-3	11	1.21E+08	1.21E+08	18621	1	67608	uc007mvq.2	2506	ENSMUSG000	Narf	nuclear prelamin A recognition factor
chr11	121240364	121240664	301	302	121240524	20	7.74501	3.7745	4.70467	Adipose_R1_peak_	Intron (uc007r	11	1.21E+08	1.21E+08	18621	1	67608	uc007mvq.2	3128	ENSMUSG000	Narf	nuclear prelamin A recognition factor
chr12	3005256	3005391	136	137	3005320	20	7.74501	3.7745	4.70467	Adipose_R1_peak_	Distal Interger	12	3235526	3250374	14849	1	74173	uc007mwj.2	-230135	ENSMUSG000	Rab10os	RAB10, member RAS oncogene family, opposite strand
chr12	3016187	3016392	206	207	3016305	34	9.39139	3.22762	6.20474	Adipose_R2_peak_	Distal Interger	12	3235526	3250374	14849	1	74173	uc007mwj.2	-219134	ENSMUSG000	Rab10os	RAB10, member RAS oncogene family, opposite strand
chr12	3018720	3018895	176	177	3018744	26	5.31252	2.48988	2.47999	Adipose_R2_peak_	Distal Interger	12	3235526	3250374	14849	1	74173	uc007mwj.2	-216631	ENSMUSG000	Rab10os	RAB10, member RAS oncogene family, opposite strand
chr12	3103476	3103656	181	182	3103575	26	12.14079	4.85292	8.87838	Adipose_R1_peak_	Distal Interger	12	3235526	3250374	14849	1	74173	uc007mwj.2	-131870	ENSMUSG000	Rab10os	RAB10, member RAS oncogene family, opposite strand
chr12	3109880	3110115	236	237	3110010	1984	1198.772	9.79322	1193.347	Adipose_R1_peak_	Distal Interger	12	3235526	3250374	14849	1	74173	uc007mwj.2	-125411	ENSMUSG000	Rab10os	RAB10, member RAS oncogene family, opposite strand
chr12	3109885	3110114	230	231	3110011	2222	1689.006	14.47138	1682.869	Adipose_R2_peak_	Distal Interger	12	3235526	3250374	14849	1	74173	uc007mwj.2	-125412	ENSMUSG000	Rab10os	RAB10, member RAS oncogene family, opposite strand
chr12	3226652	3226861	210	211	3226754	26	12.14079	4.85292	8.87838	Adipose_R1_peak_	Distal Interger	12	3235526	3250374	14849	1	74173	uc007mwj.2	-8665	ENSMUSG000	Rab10os	RAB10, member RAS oncogene family, opposite strand
chr12	4219448	4219770	323	324	4219516	29	8.34841	3.23365	5.30168	Adipose_R1_peak_	Intron (uc007r	12	4234027	4240123	6097	1	69709	uc029rqo.1	-14257	ENSMUSG000	Ptrhd1	peptidyl-tRNA hydrolase domain containing 1
chr12	4219449	4220618	1170	1171	4219502	29	6.74062	2.76653	3.75989	Adipose_R2_peak_	Intron (uc007r	12	4234027	4240123	6097	1	69709	uc029rqo.1	-13409	ENSMUSG000	Ptrhd1	peptidyl-tRNA hydrolase domain containing 1
chr12	4220159	4220547	389	390	4220432	35	9.41266	3.17843	6.28584	Adipose_R1_peak_	Intron (uc007r	12	4234027	4240123	6097	1	69709	uc029rqo.1	-13480	ENSMUSG000	Ptrhd1	peptidyl-tRNA hydrolase domain containing 1
chr12	4220729	4220833	105	106	4220774	30	7.24477	2.85875	4.21917	Adipose_R2_peak_	Intron (uc007r	12	4234027	4240123	6097	1	69709	uc029rqo.1	-13194	ENSMUSG000	Ptrhd1	peptidyl-tRNA hydrolase domain containing 1
chr12	4221011	4221328	318	319	4221180	26	8.6082	3.53479	5.52257	Adipose_R1_peak_	Intron (uc007r	12	4234027	4240123	6097	1	69709	uc029rqo.1	-12699	ENSMUSG000	Ptrhd1	peptidyl-tRNA hydrolase domain containing 1
chr12	4894936	4895136	201	202	4895014	26	12.14079	4.85292	8.87838	Adipose_R1_peak_	Intron (uc007r	12	4879032	4907520	28489	2	217379	uc007myr.1	12384	ENSMUSG000	Ubxn2a	UBX domain protein 2A
chr12	5598737	5598874	138	139	5598801	20	7.74501	3.7745	4.70467	Adipose_R1_peak_	Distal Interger	12	5376502	5416132	39631	1	72669	uc011yka.1	222235	NA	2810032G03R	RIKEN cDNA 2810032G03 gene
chr12	5621873	5622054	182	183	5621981	24	10.60668	4.49345	7.40961	Adipose_R1_peak_	Distal Interger	12	5376502	5416132	39631	1	72669	uc011yka.1	245371	NA	2810032G03R	RIKEN cDNA 2810032G03 gene
chr12	5621887	5622036	150	151	5621968	33	8.83596	3.1354	5.68652	Adipose_R2_peak_	Distal Interger	12	5376502	5416132	39631	1	72669	uc011yka.1	245385	NA	2810032G03R	RIKEN cDNA 2810032G03 gene
chr12	5623159	5623579	421	422	5623247	26	12.14079	4.85292	8.87838	Adipose_R1_peak_	Distal Interger	12	5376502	5416132	39631	1	72669	uc011yka.1	246657	NA	2810032G03R	RIKEN cDNA 2810032G03 gene
chr12	5830896	5831110	215	216	5831026	25	11.36566	4.67319	8.13482	Adipose_R1_peak_	Intron (uc007r	12	5376502	5416132	39631	1	72669	uc011yka.1	454394	NA	2810032G03R	RIKEN cDNA 2810032G03 gene
chr12	5831760	5831912	153	154	5831881	18	6.42934	3.41502	3.48999	Adipose_R1_peak_	Intron (uc007r	12	5376502	5416132	39631	1	72669	uc011yka.1	455258	NA	2810032G03R	RIKEN cDNA 2810032G03 gene
chr12	5833267	5833748	482	483	5833336	21	8.43286	3.95423	5.34896	Adipose_R1_peak_	Intron (uc007r	12	5376502	5416132	39631	1	72669	uc011yka.1	456765	NA	2810032G03R	RIKEN cDNA 2810032G03 gene
chr12	5833545	5833736	192	193	5833598	62	28.93052	5.80972	25.0743	Adipose_R2_peak_	Intron (uc007r	12	5376502	5416132	39631	1	72669	uc011yka.1	457043	NA	2810032G03R	RIKEN cDNA 2810032G03 gene
chr12	5834043	5834196	154	155	5834126	20	7.74501	3.7745	4.70467	Adipose_R1_peak_	Intron (uc007r	12	5376502	5416132	39631	1	72669	uc011yka.1	457541	NA	2810032G03R	RIKEN cDNA 2810032G03 gene
chr12	6017524	6017693	170	171	6017609	27	12.93148	5.03266	9.63827	Adipose_R1_peak_	Distal Interger	12	5376502	5416132	39631	1	72669	uc011yka.1	641022	NA	2810032G03R	RIKEN cDNA 2810032G03 gene
chr12	6078947	6079179	233	234	6079077	39	23.47001	7.18952	19.88325	Adipose_R1_peak_	Distal Interger	12	5376502	5416132	39631	1	72669	uc011yka.1	702445	NA	2810032G03R	RIKEN cDNA 2810032G03 gene
chr12	6078956	6079147	192	193	6079082	47	17.60017	4.42645	14.02531	Adipose_R2_peak_	Distal Interger	12	5376502	5416132	39631	1	72669	uc011yka.1	702454	NA	2810032G03R	RIKEN cDNA 2810032G03 gene
chr12	6079609	6079774	166	167	6079697	26	12.14079	4.85292	8.87838	Adipose_R1_peak_	Distal Interger	12	5376502	5416132	39631	1	72669	uc011yka.1	703107	NA	2810032G03R	RIKEN cDNA 2810032G03 gene
chr12	6156511	6156781	271	272	6156637	51	35.56531	9.34637	31.76235	Adipose_R1_peak_	Distal Interger	12	5376502	5416132	39631	1	72669	uc011yka.1	780009	NA	2810032G03R	RIKEN cDNA 2810032G03 gene
chr12	6156526	6156781	256	257	6156653	67	33.06755	6.27081	29.1399	Adipose_R2_peak_	Distal Interger	12	5376502	5416132	39631	1	72669	uc011yka.1	780024	NA	2810032G03R	RIKEN cDNA 2810032G03 gene
chr12	6226558	6226769	212	213	6226654	25	11.36566	4.67319	8.13482	Adipose_R1_peak_	Distal Interger	12	5376502	5416132	39631	1	72669	uc011yka.1	850056	NA	2810032G03R	RIKEN cDNA 2810032G03 gene
chr12	6237586	6237871	286	287	6237735	36	20.67077	6.6503	17.14779	Adipose_R1_peak_	Distal Interger	12	5376502	5416132	39631	1	72669	uc011yka.1	861084	NA	2810032G03R	RIKEN cDNA 2810032G03 gene
chr12	6337177	6337364	188	189	6337278	24	10.60668	4.49345	7.40961	Adipose_R1_peak_	Distal Interger	12	5376502	5416132	39631	1	72669	uc011yka.1	960675	NA	2810032G03R	RIKEN cDNA 2810032G03 gene
chr12	6524915	6525154	240	241	6525018	28	13.73721	5.2124	10.41463	Adipose_R1_peak_	Distal Interger	12	7372039	7380330	8292	2	73575	uc007mza.2	855176	ENSMUSG000	1700101O22R	RIKEN cDNA 1700101O22 gene
chr12	6609951	6610149	199	200	6610066	31	16.23981	5.75161	12.83549	Adipose_R1_peak_	Distal Interger	12	7372039	7380330	8292	2	73575	uc007mza.2	770181	ENSMUSG000	1700101O22R	RIKEN cDNA 1700101O22 gene
chr12	6667527	6667714	188	189	6667631	40	12.96465	3.78093	9.57654	Adipose_R2_peak_	Distal Interger	12	7372039	7380330	8292	2	73575	uc007mza.2	712616	ENSMUSG000	1700101O22R	RIKEN cDNA 1700101O22 gene
chr12	6667548	6667716	169	170	6667630	24	10.60668	4.49345	7.40961	Adipose_R1_peak_	Distal Interger	12	7372039	7380330	8292	2	73575	uc007mza.2	712614	ENSMUSG000	1700101O22R	RIKEN cDNA 1700101O22 gene
chr12	6668445	6668707	263	264	6668561	60	40.22202	9.16619	36.356	Adipose_R1_peak_	Distal Interger	12	7372039	7380330	8292	2	73575	uc007mza.2	711623	ENSMUSG000	1700101O22R	RIKEN cDNA 1700101O22 gene
chr12	6668453	6668670	218	219	6668573	51	20.44827	4.79532	16.78434	Adipose_R2_peak_	Distal Interger	12	7372039	7380330	8292	2	73575	uc007mza.2	711660	ENSMUSG000	1700101O22R	RIKEN cDNA 1700101O22 gene
chr12	6670155	6670318	164	165	6670219	18	6.42934	3.41502	3.48999	Adipose_R1_peak_	Distal Interger	12	7372039	7380330	8292	2	73575	uc007mza.2	710012	ENSMUSG000	1700101O22R	RIKEN cDNA 1700101O22 gene
chr12	6777034	6777271	238	239	6777143	28	13.73721	5.2124	10.41463	Adipose_R1_peak_	Distal Interger	12	7372039	7380330	8292	2	73575	uc007mza.2	603059	ENSMUSG000	1700101O22R	RIKEN cDNA 1700101O22 gene
chr12	6777101	6777211	111	112	6777151	30	7.24477	2.85875	4.21917	Adipose_R2_peak_	Distal Interger	12	7372039	7380330	8292	2	73575	uc007mza.2	603119	ENSMUSG000	1700101O22R	RIKEN cDNA 1700101O22 gene
chr12	7183004	7183164	161	162	7183104	18	6.42934	3.41502	3.48999	Adipose_R1_peak_	Distal Interger	12	7372039	7380330	8292	2	73575	uc007mza.2	197166	ENSMUSG000	1700101O22R	RIKEN cDNA 1700101O22 gene
chr12	8198900	8199180	281	282	8199026	61	46.56983	11.14375	42.62347	Adipose_R1_peak_	Distal Interger	12	8208107	8276223	68117	1	68832	uc007mzg.2	-8927	ENSMUSG000	Ldah	lipid droplet associated hydrolase

chr12	8198924	8199153	230	231	8199017	61	28.12311	5.7175	24.28203	Adipose_R2_peak_	Distal Interger	12	8208107	8276223	68117	1	68832	uc007mzg.2	-8954	ENSMUSG000	Ldah	lipid droplet associated hydrolase
chr12	8431458	8431631	174	175	8431551	21	8.43286	3.95423	5.34896	Adipose_R1_peak_	Distal Interger	12	8497759	8499985	2227	2	11852	uc007mzp.1	68354	ENSMUSG000	Rhob	ras homolog family member B
chr12	8493609	8493743	135	136	8493630	17	5.80341	3.23528	2.92378	Adipose_R1_peak_	Distal Interger	12	8497759	8499985	2227	2	11852	uc007mzp.1	6242	ENSMUSG000	Rhob	ras homolog family member B
chr12	8496642	8496827	186	187	8496683	16	5.20017	3.05554	2.38734	Adipose_R1_peak_	Downstream (	12	8497759	8499985	2227	2	11852	uc007mzp.1	3158	ENSMUSG000	Rhob	ras homolog family member B
chr12	8496862	8497034	173	174	8496957	33	8.83596	3.1354	5.68652	Adipose_R2_peak_	Promoter (2-3	12	8497759	8499985	2227	2	11852	uc007mzp.1	2951	ENSMUSG000	Rhob	ras homolog family member B
chr12	8522172	8522330	159	160	8522317	16	5.20017	3.05554	2.38734	Adipose_R1_peak_	Intron (uc007r	12	8528483	8539566	11084	2	328059	uc007mzs.2	17236	ENSMUSG000	Slc7a15	solute carrier family 7 (cationic amino acid transporter, y+ system), member 15
chr12	8557481	8557694	214	215	8557584	24	10.60668	4.49345	7.40961	Adipose_R1_peak_	Intron (uc007r	12	8528483	8539566	11084	2	328059	uc007mzs.2	-17915	ENSMUSG000	Slc7a15	solute carrier family 7 (cationic amino acid transporter, y+ system), member 15
chr12	8580489	8580713	225	226	8580594	32	17.10103	5.93135	13.67137	Adipose_R1_peak_	Intron (uc007r	12	8528483	8599066	70584	2	328059	uc007mzt.1	18353	ENSMUSG000	Slc7a15	solute carrier family 7 (cationic amino acid transporter, y+ system), member 15
chr12	8581993	8582146	154	155	8582072	33	8.83596	3.1354	5.68652	Adipose_R2_peak_	Intron (uc007r	12	8528483	8599066	70584	2	328059	uc007mzt.1	16920	ENSMUSG000	Slc7a15	solute carrier family 7 (cationic amino acid transporter, y+ system), member 15
chr12	8582005	8582214	210	211	8582099	30	15.39182	5.57188	12.01394	Adipose_R1_peak_	Intron (uc007r	12	8528483	8599066	70584	2	328059	uc007mzt.1	16852	ENSMUSG000	Slc7a15	solute carrier family 7 (cationic amino acid transporter, y+ system), member 15
chr12	8582527	8582791	265	266	8582635	58	38.50106	8.9762	34.66282	Adipose_R1_peak_	Intron (uc007r	12	8528483	8599066	70584	2	328059	uc007mzt.1	16275	ENSMUSG000	Slc7a15	solute carrier family 7 (cationic amino acid transporter, y+ system), member 15
chr12	8582536	8582752	217	218	8582663	58	25.74274	5.44085	21.94821	Adipose_R2_peak_	Intron (uc007r	12	8528483	8599066	70584	2	328059	uc007mzt.1	16314	ENSMUSG000	Slc7a15	solute carrier family 7 (cationic amino acid transporter, y+ system), member 15
chr12	8610136	8610292	157	158	8610243	30	7.24477	2.85875	4.21917	Adipose_R2_peak_	Distal Interger	12	8528483	8599066	70584	2	328059	uc007mzt.1	-11070	ENSMUSG000	Slc7a15	solute carrier family 7 (cationic amino acid transporter, y+ system), member 15
chr12	8612857	8613071	215	216	8612933	29	6.74062	2.76653	3.75989	Adipose_R2_peak_	Distal Interger	12	8528483	8599066	70584	2	328059	uc007mzt.1	-13791	ENSMUSG000	Slc7a15	solute carrier family 7 (cationic amino acid transporter, y+ system), member 15
chr12	8612931	8613095	165	166	8613028	22	9.13959	4.13397	6.01526	Adipose_R1_peak_	Distal Interger	12	8528483	8599066	70584	2	328059	uc007mzt.1	-13865	ENSMUSG000	Slc7a15	solute carrier family 7 (cationic amino acid transporter, y+ system), member 15
chr12	8614385	8614560	176	177	8614462	18	6.42934	3.41502	3.48999	Adipose_R1_peak_	Distal Interger	12	8528483	8599066	70584	2	328059	uc007mzt.1	-15319	ENSMUSG000	Slc7a15	solute carrier family 7 (cationic amino acid transporter, y+ system), member 15
chr12	9680119	9680277	159	160	9680207	32	8.29282	3.04319	5.18352	Adipose_R2_peak_	Distal Interger	12	9574442	9581500	7059	1	23967	uc007nao.2	105677	ENSMUSG000	Osr1	odd-skipped related 1 (Drosophila)
chr12	9738287	9738495	209	210	9738405	29	14.55748	5.39214	11.20652	Adipose_R1_peak_	Distal Interger	12	9574442	9581500	7059	1	23967	uc007nao.2	163845	ENSMUSG000	Osr1	odd-skipped related 1 (Drosophila)
chr12	9738343	9738457	115	116	9738409	31	7.7623	2.95097	4.69388	Adipose_R2_peak_	Distal Interger	12	9574442	9581500	7059	1	23967	uc007nao.2	163901	ENSMUSG000	Osr1	odd-skipped related 1 (Drosophila)
chr12	10185821	10186042	222	223	10185943	49	19.00719	4.61089	15.38598	Adipose_R2_peak_	Distal Interger	12	10369971	10390174	20204	1	70881	uc007nap.2	-183929	ENSMUSG000	Nt5c1b	5'-nucleotidase, cytosolic IB
chr12	10185822	10186070	249	250	10185957	52	35.77416	9.21196	31.97011	Adipose_R1_peak_	Distal Interger	12	10369971	10390174	20204	1	70881	uc007nap.2	-183901	ENSMUSG000	Nt5c1b	5'-nucleotidase, cytosolic IB
chr12	10201441	10201575	135	136	10201531	19	7.07687	3.59476	4.08458	Adipose_R1_peak_	Distal Interger	12	10369971	10390174	20204	1	70881	uc007nap.2	-168396	ENSMUSG000	Nt5c1b	5'-nucleotidase, cytosolic IB
chr12	10631391	10631644	254	255	10631521	41	25.38991	7.54899	21.76305	Adipose_R1_peak_	Distal Interger	12	10446388	10531303	84916	1	328061	uc033gcq.1	185003	NA	Gm38407	predicted gene, 38407
chr12	10631410	10631575	166	167	10631563	47	17.60017	4.42645	14.02531	Adipose_R2_peak_	Distal Interger	12	10446388	10531303	84916	1	328061	uc033gcq.1	185022	NA	Gm38407	predicted gene, 38407
chr12	10700637	10700956	320	321	10700775	95	88.66338	17.25484	84.38583	Adipose_R1_peak_	Distal Interger	12	10446388	10531303	84916	1	328061	uc033gcq.1	254249	NA	Gm38407	predicted gene, 38407
chr12	10700642	10700923	282	283	10700774	77	41.80442	7.19298	37.74731	Adipose_R2_peak_	Distal Interger	12	10446388	10531303	84916	1	328061	uc033gcq.1	254254	NA	Gm38407	predicted gene, 38407
chr12	10703435	10703600	166	167	10703512	22	9.13959	4.13397	6.01526	Adipose_R1_peak_	Distal Interger	12	10446388	10531303	84916	1	328061	uc033gcq.1	257047	NA	Gm38407	predicted gene, 38407
chr12	10705248	10705378	131	132	10705317	20	7.74501	3.7745	4.70467	Adipose_R1_peak_	Distal Interger	12	10446388	10531303	84916	1	328061	uc033gcq.1	258860	NA	Gm38407	predicted gene, 38407
chr12	10817253	10817405	153	154	10817314	18	6.42934	3.41502	3.48999	Adipose_R1_peak_	Distal Interger	12	11047813	11078741	30929	1	71457	uc029rqv.1	-230408	NA	7420701I03Ri	RIKEN cDNA 7420701I03 gene
chr12	10961507	10962170	664	665	10961954	50	34.50784	9.16663	30.72138	Adipose_R1_peak_	Distal Interger	12	11047813	11078741	30929	1	71457	uc029rqv.1	-85643	NA	7420701I03Ri	RIKEN cDNA 7420701I03 gene
chr12	10962449	10962934	486	487	10962705	45	29.34988	8.26794	25.64927	Adipose_R1_peak_	Distal Interger	12	11047813	11078741	30929	1	71457	uc029rqv.1	-84879	NA	7420701I03Ri	RIKEN cDNA 7420701I03 gene
chr12	10962477	10962869	393	394	10962591	32	8.29282	3.04319	5.18352	Adipose_R2_peak_	Distal Interger	12	11047813	11078741	30929	1	71457	uc029rqv.1	-84944	NA	7420701I03Ri	RIKEN cDNA 7420701I03 gene
chr12	10971281	10971462	182	183	10971373	32	17.10103	5.93135	13.67137	Adipose_R1_peak_	Distal Interger	12	11047813	11078741	30929	1	71457	uc029rqv.1	-76351	NA	7420701I03Ri	RIKEN cDNA 7420701I03 gene
chr12	10971311	10971466	156	157	10971389	30	7.24477	2.85875	4.21917	Adipose_R2_peak_	Distal Interger	12	11047813	11078741	30929	1	71457	uc029rqv.1	-76347	NA	7420701I03Ri	RIKEN cDNA 7420701I03 gene
chr12	10993636	10994380	745	746	10993774	99	94.0006	17.97379	89.69558	Adipose_R1_peak_	Distal Interger	12	11047813	11078741	30929	1	71457	uc029rqv.1	-53433	NA	7420701I03Ri	RIKEN cDNA 7420701I03 gene
chr12	10993651	10993905	255	256	10993769	64	30.56565	5.99415	26.6807	Adipose_R2_peak_	Distal Interger	12	11047813	11078741	30929	1	71457	uc029rqv.1	-53908	NA	7420701I03Ri	RIKEN cDNA 7420701I03 gene
chr12	10994116	10994370	255	256	10994246	53	21.92215	4.97976	18.21755	Adipose_R2_peak_	Distal Interger	12	11047813	11078741	30929	1	71457	uc029rqv.1	-53443	NA	7420701I03Ri	RIKEN cDNA 7420701I03 gene
chr12	11051361	11051685	325	326	11051534	62	47.71039	11.32349	43.751	Adipose_R1_peak_	Intron (uc029r	12	11047813	11078741	30929	1	71457	uc029rqv.1	3548	NA	7420701I03Ri	RIKEN cDNA 7420701I03 gene
chr12	11051428	11051683	256	257	11051550	66	32.22715	6.17859	28.31407	Adipose_R2_peak_	Intron (uc029r	12	11047813	11078741	30929	1	71457	uc029rqv.1	3615	NA	7420701I03Ri	RIKEN cDNA 7420701I03 gene
chr12	12094593	12094917	325	326	12094738	80	69.31596	14.55877	65.15641	Adipose_R1_peak_	Distal Interger	12	12262139	12376361	114223	1	76820	uc007nbc.2	-167222	ENSMUSG000	Fam49a	family with sequence similarity 49, member A
chr12	12094594	12094903	310	311	12094732	105	69.01683	9.77508	64.68261	Adipose_R2_peak_	Distal Interger	12	12262139	12376361	114223	1	76820	uc007nbc.2	-167236	ENSMUSG000	Fam49a	family with sequence similarity 49, member A
chr12	12097120	12097327	208	209	12097226	30	15.39182	5.57188	12.01394	Adipose_R1_peak_	Distal Interger	12	12262139	12376361	114223	1	76820	uc007nbc.2	-164812	ENSMUSG000	Fam49a	family with sequence similarity 49, member A
chr12	12236905	12237077	173	174	12236930	17	5.80341	3.23528	2.92378	Adipose_R1_peak_	Distal Interger	12	12262139	12376361	114223	1	76820	uc007nbc.2	-25062	ENSMUSG000	Fam49a	family with sequence similarity 49, member A
chr12	12862089	12862343	255	256	12862220	42	26.36523	7.72873	22.71906	Adipose_R1_peak_	Distal Interger	12	12936093	12940616	4524	2	18109	uc007nbf.2	78273	ENSMUSG000	Mycn	v-myc avian myelocytomatosis viral related oncogene, neuroblastoma derived
chr12	12862191	12862288	98	99	12862231	27	5.77405	2.5821	2.88921	Adipose_R2_peak_	Distal Interger	12	12936093	12940616	4524	2	18109	uc007nbf.2	78328	ENSMUSG000	Mycn	v-myc avian myelocytomatosis viral related oncogene, neuroblastoma derived
chr12	12862831	12862970	140	141	12862909	35	9.95877	3.31984	6.73562	Adipose_R2_peak_	Distal Interger	12	12936093	12940616	4524	2	18109	uc007nbf.2	77646	ENSMUSG000	Mycn	v-myc avian myelocytomatosis viral related oncogene, neuroblastoma derived
chr12	12862846	12863014	169	170	12862908	20	7.74501	3.7745	4.70467	Adipose_R1_peak_	Distal Interger	12	12936093	12940616	4524	2	18109	uc007nbf.2	77602	ENSMUSG000	Mycn	v-myc avian myelocytomatosis viral related oncogene, neuroblastoma derived
chr12	13034412	13034599	188	189	13034592	38	11.72962	3.59649	8.4047	Adipose_R2_peak_	Distal Interger	12	12936093	12941836	5744	2	18109	uc007nbg.2	-92576	ENSMUSG000	Mycn	v-myc avian myelocytomatosis viral related oncogene, neuroblastoma derived
chr12	13034428	13034599	172	173	13034578	22	9.13959	4.13397	6.01526	Adipose_R1_peak_	Distal Interger	12	12936093	12941836	5744	2	18109	uc007nbg.2	-92592	ENSMUSG000	Mycn	v-myc avian myelocytomatosis viral related oncogene, neuroblastoma derived
chr12	13189682	13189861	180	181	13189772	23	9.86443	4.31371	6.70247	Adipose_R1_peak_	Distal Interger	12	13219307	13249174	29868	2	104721	uc007nbh.1	59313	ENSMUSG000	Ddx1	DEAD (Asp-Glu-Ala-Asp) box polypeptide 1
chr12	13189714	13189812	99	100	13189725	27	5.77405	2.5821	2.88921	Adipose_R2_peak_	Distal Interger	12	13219307	13249174	29868	2	104721	uc007nbh.1	59362	ENSMUSG000	Ddx1	DEAD (Asp-Glu-Ala-Asp) box polypeptide 1
chr12	13761870	13762115	246	247	13761988	31	16.23981	5.75161	12.83549	Adipose_R1_peak_	Distal Interger	12	13487122	13561730	74609	1	71169	uc007nbl.1	274748	ENSMUSG000	Nbas	neuroblastoma amplified sequence
chr12	13761945	13762078	134	135	13762003	31	7.7623	2.95097	4.69388	Adipose_R2_peak_	Distal Interger	12	13487122	13561730	74609	1	71169	uc007nbl.1	274823	ENSMUSG000	Nbas	neuroblastoma amplified sequence
chr12	14389462	14389692	231	232	14389596	53	21.92215	4.97976	18.21755	Adipose_R2_peak_	Distal Interger	12	14261534	14285684	24151	1	791361	uc007nbo.2	127928	NA	Gm16497	predicted gene 16497
chr12	14392210	14392415	206	207	14392311	29	14.55748	5.39214	11.20652	Adipose_R1_peak_	Distal Interger	12	14261534	14285684	24151	1	791361	uc007nbo.2	130676	NA	Gm16497	predicted gene 16497
chr12	14392247	14392370	124	125	14392299	31	7.7623	2.95097	4.69388	Adipose_R2_peak_	Distal Interger	12	14261534	14285684	24151	1	791361	uc007nbo.2	130713	NA	Gm16497	predicted gene 16497
chr12	14597773	14597965	193	194	14597874	23	9.86443	4.31371	6.70247	Adipose_R1_peak_	Distal Interger	12	14261534	14285684	24151	1	791361	uc007nbo.2	336239	NA	Gm16497	predicted gene 16497
chr12	14792726	14792916	191	192	14792807	26	12.14079	4.85292	8.87838	Adipose_R1_peak_	Distal Interger	12	14996389	15050389	54001	2	73972	uc007nbp.1	257473	NA	4930448C13R	RIKEN cDNA 4930448C13 gene
chr12	14852390	14852586	197	198	14852486	31	16.23981	5.75161	12.83549	Adipose_R1_peak_	Distal Interger	12	14996389	15050389	54001	2	73972	uc007nbp.1	197803	NA	4930448C13R	RIKEN cDNA 4930448C13 gene
chr12	14852419	14852556	138	139	14852540	27	5.77405	2.5821	2.88921	Adipose_R2_peak_	Distal Interger	12	14996389	15050389	54001	2	73972	uc007nbp.1	197833	NA	4930448C13R	RIKEN cDNA 4930448C13 gene
chr12	14944243	14944431	189	190	14944258	20	7.74501	3.7745	4.70467	Adipose_R1_peak_	Distal Interger	12	14996389	15050389	54001	2	73972	uc007nbp.1	105958	NA	4930448C13R	RIKEN cDNA 4930448C13 gene
chr12	15031693	15031960	268	269	15031829	50	34.50784	9.16663	30.72138	Adipose_R1_peak_	Intron (uc007r	12	14996389	15050389	54001	2	73972	uc007nbp.1	18429	NA	4930448C13R	RIKEN cDNA 4930448C13 gene
chr12	15031746	15031959	214	215	15031833	43	14.8939	4.05758	11.42017	Adipose_R2_peak_	Intron (uc007r	12	14996389	15050389	54001	2	73972	uc007nbp.1	18430	NA	4930448C13R	RIKEN cDNA 4930448C13 gene
chr12	15117896	15118077	182	183	15118065	24	10.60668	4.49345	7.40961	Adipose_R1_peak_	Distal Interger	12	14996389	15050389	54001	2	73972	uc007nbp.1	-67507	NA	4930448C13R	RIKEN cDNA 4930448C13 gene
chr12	15197906	15198085	180	181	15198002	23	9.86443	4.31371	6.70247	Adipose_R1_peak_	Distal Interger	12	14996389	15050389	54001	2	73972	uc007nbp.1	-147517	NA	4930448C13R	RIKEN cDNA 4930448C13 gene
chr12	15253528	15253858	331	332	15253609	91	43.43087	6.26426	39.52295	Adipose_R1_peak_	Distal Interger	12	14996389	15050389	54001	2	73972	uc007nbp.1	-203139	NA	4930448C13R	RIKEN cDNA 4930448C13 gene
chr12	15253548	15253851	304	305	15253618	86	39.59888	5.96921	35.5741	Adipose_R2_peak_	Distal Interger	12	14996389	15050389	54001	2	73972	uc007nbp.1	-203159	NA	4930448C13R	RIKEN cDNA 4930448C13 gene
chr12	15258550	15258856	307	308	15258668	137	134.6162	20.48435	130.1496	Adipose_R1_peak_	Distal Interger	12	14996389	15050389	54001	2	73972	uc007nbp.1	-208161	NA	4930448C13R	RIKEN cDNA 4930448C13 gene
chr12	15258557	15258829	273	274	15258679	113	77.42068	10.51282	73.02626	Adipose_R2_peak_	Distal Interger	12	14996389	15050389	54001	2	73972	uc007nbp.1	-208168	NA	4930448C13R	RIKEN cDNA 4930448C13 gene
chr12	15829368	15829523	156	157	15829406	16	5.20017	3.05554	2.38734	Adipose_R1_peak_	Intron (uc007r	12	15791727	15816785	25059	2	217410	uc007nbq.1	-12583	ENSMUSG000	Trib2	tribbles pseudokinase 2
chr12	15866061	15866213	153	154	15866124	17	5.80341	3.23528	2.92378	Adipose_R1_peak_	Intron (uc007r	12	15791727	15816785	25059	2	217410	uc007nbq.1	-49276	ENSMUSG000	Trib2	tribbles pseudokinase 2
chr12	15869325	15869635	311	312	15869493	68	54.69342	12.40192	50.66183	Adipose_R1_peak_	Intron (uc007r	12	15791727	15816785	25059	2	217410	uc007nbq.1	-52540	ENSMUSG000	Trib2	tribbles pseudokinase 2
chr12	15869357	15869583	227	228	15869502	51	20.44827	4.79532	16.78434	Adipose_R2_peak_	Intron (uc007r	12	15791727	15816785	25059	2	217410	uc007nbq.1	-52572	ENSMUSG000	Trib2	tribbles pseudokinase 2
chr12	15928313	15928580	268	269	15928480	26	12.14079	4.85292	8.87838	Adipose_R1_peak_	Distal Interger	12	15791727	15816785	25059	2	217410	uc007nbq.1	-111528	ENSMUSG000	Trib2	tribbles pseudokinase 2
chr12	16013211	16013355	145	146	16013281	20	7.74501	3.7745	4.70467	Adipose_R1_peak_	Distal Interger	12	15791727	15816785	25059	2	217410	uc007nbq.1	-196426	ENSMUSG000	Trib2	tribbles pseudokinase 2
chr12	16173938	16174077	140	141	16174057	20	7.74501	3.7745	4.70467	Adipose_R1_peak_	Distal Interger	12	15791727	15816785	25059	2	217410	uc007nbq.1	-357153	ENSMUSG000	Trib2	tribbles pseudokinase 2
chr12	16627924	16628134	211	212	16628117	33	17.97508	6.11109	14.52164	Adipose_R1_peak_	Distal Interger	12	16653470	16660236	6767	1	18217	uc007nbw.1	-25336	ENSMUSG000	Ntsr2	neurotensin receptor 2
chr12	16627936	16628134	199	200	16628125	52	21.18119	4.88754	17.49655	Adipose_R2_peak_	Distal Interger	12	16653470	16660236	6767	1	18217	uc007nbw.1	-25336	ENSMUSG000	Ntsr2	neurotensin receptor 2
chr12	17124933	17125073	141	142	17124998	20	7.74501	3.7745	4.70467	Adipose_R1_peak_	Distal Interger	12	17172100	17176888	4789	2	382571	uc007ncn.2	51815	ENSMUSG000	Kcnf1	potassium voltage-gated channel, subfamily F, member 1
chr12	17127477	17127595	119	120	17127581	27	5.77405	2.5821	2.88921	Adipose_R2_peak_	Distal Interger	12	17172100	17176888	4789	2	382571	uc007ncn.2	49293	ENSMUSG000	Kcnf1	potassium voltage-gated channel, subfamily F, member 1
chr12	17159863	17160001	139	140	17159888	26	5.31252	2.48988	2.47999	Adipose_R2_peak_	Distal Interger	12	17172100	17176888	4789	2	382571	uc007ncn.2	16887	ENSMUSG000	Kcnf1	potassium voltage-gated channel, subfamily F, member 1
chr12	17246780	17247013	234	235	17246878	31	16.23981	5.75161	12.83549	Adipose_R1_peak_	Distal Interger	12	17266595	17281810	15216	1	71853	uc007nco.1	-19582	ENSMUSG000	Pdia6	protein disulfide isomerase associated 6
chr12	17246792	17246965	174	175	17246891	30	7.24477	2.85875	4.21917	Adipose_R2_peak_	Distal Interger	12	17266595	17281810	15216	1	71853	uc007nco.1	-19630	ENSMUSG000	Pdia6	protein disulfide isomerase associated 6
chr12	17821077	17821267	191	192	17821194	23	9.86443	4.31371	6.70247	Adipose_R1_peak_	Distal Interger	12	17786147	17791926	5780	1	53602	uc007ncy.1	34930	ENSMUSG000	Hpcal1	hippocalcin-like 1
chr12	17821109	17821260	152	153	17821177	26	5.31252	2.48988	2.47999	Adipose_R2_peak_	Distal Interger	12	17786147	17791926	5780	1	53602	uc007ncy.1	34962	ENSMUSG000	Hpcal1	hippocalcin-like 1
chr12	17838020	17838299	280	281	17838126	23	9.86443	4.31371	6.70247	Adipose_R1_peak_	Distal Interger	12	17786147	17791926	5780	1	53602	uc007ncy.1	51873	ENSMUSG000	Hpcal1	hippocalcin-like 1
chr12	17838104	17838275	172	173	17838150	32	8.29282	3.04319	5.18352	Adipose_R2_peak_	Distal Interger	12	17786147	17791926	5780	1	53602	uc007ncy.1	51957	ENSMUSG000	Hpcal1	hippocalcin-like 1
chr12	17869139	17869240	102	103	17869184	27	5.77405	2.5821	2.88921	Adipose_R2_peak_	Distal Interger	12	17786147	17791926	5780	1	53602	uc007ncy.1	82992	ENSMUSG000	Hpcal1	hippocalcin-like 1
chr12	17869195	17869343	149	150	17869272	21	8.43286	3.95423	5.34896	Adipose_R1_peak_	Distal Interger	12	17786147	17791926	5780	1	53602	uc007ncy.1	83048	ENSMUSG000	Hpcal1	hippocalcin-like 1
chr12	17957513	17957704	192	193	17957621	24	10.60668	4.49345	7.40961	Adipose_R1_peak_	Distal Interger	12	17786147	17791926	5780	1	53602	uc007ncy.1	171366	ENSMUSG000	Hpcal1	hippocalcin-like 1
chr12	17967297	17967630	334	335	17967487	84	74.36649	15.27772	70.17167	Adipose_R1_peak_	Distal Interger	12	17786147	17791926	5780	1	53602	uc007ncy.1	181150	ENSMUSG000	Hpcal1	hippocalcin-like 1
chr12	17967309	17967621	313	314	17967481	84	48.25301	7.83851	44.11785	Adipose_R2_peak_	Distal Interger	12	17786147	17791926	5780	1	53602	uc007ncy.1	181162	ENSMUSG000	Hpcal1	hippocalcin-like 1
chr12	17969399	17969597	199	200	17969518	42	26.36523	7.72873	22.71906	Adipose_R1_peak_	Distal Interger	12	17786147	17791926	5780	1	53602	uc007ncy.1	183252	ENSMUSG000	Hpcal1	hippocalcin-like 1
chr12	17969420	17969583	164	165	17969516	30	7.24477	2.85875	4.21917	Adipose_R2_peak_	Distal Interger	12	17786147	17791926	5780	1	53602	uc007ncy.1	183273	ENSMUSG000	Hpcal1	hippocalcin-like 1

chr12	18074204	18074436	233	234	18074296	26	12.14079	4.85292	8.87838	Adipose_R1_peak_	Distal Interger	12	17786147	17791926	5780	1	53602	uc007ncy.1	288057	ENSMUSG000	Hpcal1	hippocalcin-like 1
chr12	18095120	18095252	133	134	18095189	18	6.42934	3.41502	3.48999	Adipose_R1_peak_	Distal Interger	12	17786147	17791926	5780	1	53602	uc007ncy.1	308973	ENSMUSG000	Hpcal1	hippocalcin-like 1
chr12	18122153	18122322	170	171	18122292	20	7.74501	3.7745	4.70467	Adipose_R1_peak_	Distal Interger	12	17786147	17791926	5780	1	53602	uc007ncy.1	336006	ENSMUSG000	Hpcal1	hippocalcin-like 1
chr12	18157080	18157303	224	225	18157189	35	19.76029	6.47056	16.26028	Adipose_R1_peak_	Distal Interger	12	18514510	18535254	20745	1	236366	uc007ndb.2	-357207	ENSMUSG000	5730507C01R	RIKEN cDNA 5730507C01 gene
chr12	18157116	18157291	176	177	18157200	34	9.39139	3.22762	6.20474	Adipose_R2_peak_	Distal Interger	12	18514510	18535254	20745	1	236366	uc007ndb.2	-357219	ENSMUSG000	5730507C01R	RIKEN cDNA 5730507C01 gene
chr12	18160736	18160951	216	217	18160841	24	10.60668	4.49345	7.40961	Adipose_R1_peak_	Distal Interger	12	18514510	18535254	20745	1	236366	uc007ndb.2	-353559	ENSMUSG000	5730507C01R	RIKEN cDNA 5730507C01 gene
chr12	18197656	18197842	187	188	18197760	32	8.29282	3.04319	5.18352	Adipose_R2_peak_	Distal Interger	12	18514510	18535254	20745	1	236366	uc007ndb.2	-316668	ENSMUSG000	5730507C01R	RIKEN cDNA 5730507C01 gene
chr12	18197693	18197968	276	277	18197803	21	8.43286	3.95423	5.34896	Adipose_R1_peak_	Distal Interger	12	18514510	18535254	20745	1	236366	uc007ndb.2	-316542	ENSMUSG000	5730507C01R	RIKEN cDNA 5730507C01 gene
chr12	18199701	18199993	293	294	18199843	54	22.67101	5.07198	18.94758	Adipose_R2_peak_	Distal Interger	12	18514510	18535254	20745	1	236366	uc007ndb.2	-314517	ENSMUSG000	5730507C01R	RIKEN cDNA 5730507C01 gene
chr12	18199713	18199999	287	288	18199850	35	19.76029	6.47056	16.26028	Adipose_R1_peak_	Distal Interger	12	18514510	18535254	20745	1	236366	uc007ndb.2	-314511	ENSMUSG000	5730507C01R	RIKEN cDNA 5730507C01 gene
chr12	18277170	18277310	141	142	18277262	21	8.43286	3.95423	5.34896	Adipose_R1_peak_	Distal Interger	12	18514510	18535254	20745	1	236366	uc007ndb.2	-237200	ENSMUSG000	5730507C01R	RIKEN cDNA 5730507C01 gene
chr12	18280647	18280805	159	160	18280766	17	5.80341	3.23528	2.92378	Adipose_R1_peak_	Distal Interger	12	18514510	18535254	20745	1	236366	uc007ndb.2	-233705	ENSMUSG000	5730507C01R	RIKEN cDNA 5730507C01 gene
chr12	18340747	18340891	145	146	18340812	22	9.13959	4.13397	6.01526	Adipose_R1_peak_	Distal Interger	12	18514510	18535254	20745	1	236366	uc007ndb.2	-173619	ENSMUSG000	5730507C01R	RIKEN cDNA 5730507C01 gene
chr12	18341547	18341702	156	157	18341633	18	6.42934	3.41502	3.48999	Adipose_R1_peak_	Distal Interger	12	18514510	18535254	20745	1	236366	uc007ndb.2	-172808	ENSMUSG000	5730507C01R	RIKEN cDNA 5730507C01 gene
chr12	18345141	18345610	470	471	18345304	49	33.45876	8.98689	29.68925	Adipose_R1_peak_	Distal Interger	12	18514510	18535254	20745	1	236366	uc007ndb.2	-168900	ENSMUSG000	5730507C01R	RIKEN cDNA 5730507C01 gene
chr12	18345183	18345397	215	216	18345278	38	11.72962	3.59649	8.4047	Adipose_R2_peak_	Distal Interger	12	18514510	18535254	20745	1	236366	uc007ndb.2	-169113	ENSMUSG000	5730507C01R	RIKEN cDNA 5730507C01 gene
chr12	18484971	18485074	104	105	18485015	30	7.24477	2.85875	4.21917	Adipose_R2_peak_	Distal Interger	12	18514510	18535254	20745	1	236366	uc007ndb.2	-29436	ENSMUSG000	5730507C01R	RIKEN cDNA 5730507C01 gene
chr12	18563306	18563557	252	253	18563354	30	5.82459	2.47643	2.93775	Adipose_R2_peak_	Distal Interger	12	18514510	18535254	20745	1	236366	uc007ndb.2	48796	ENSMUSG000	5730507C01R	RIKEN cDNA 5730507C01 gene
chr12	18587885	18587979	95	96	18587930	28	6.25023	2.67431	3.31626	Adipose_R2_peak_	Distal Interger	12	18514510	18535254	20745	1	236366	uc007ndb.2	73375	ENSMUSG000	5730507C01R	RIKEN cDNA 5730507C01 gene
chr12	18589751	18589932	182	183	18589885	31	7.7623	2.95097	4.69388	Adipose_R2_peak_	Distal Interger	12	18514510	18535254	20745	1	236366	uc007ndb.2	75241	ENSMUSG000	5730507C01R	RIKEN cDNA 5730507C01 gene
chr12	18592564	18592770	207	208	18592607	29	6.74062	2.76653	3.75989	Adipose_R2_peak_	Distal Interger	12	18514510	18535254	20745	1	236366	uc007ndb.2	78054	ENSMUSG000	5730507C01R	RIKEN cDNA 5730507C01 gene
chr12	18665900	18666105	206	207	18665998	28	13.73721	5.2124	10.41463	Adipose_R1_peak_	Distal Interger	12	18514510	18535254	20745	1	236366	uc007ndb.2	151390	ENSMUSG000	5730507C01R	RIKEN cDNA 5730507C01 gene
chr12	18665930	18666044	115	116	18665975	27	5.77405	2.5821	2.88921	Adipose_R2_peak_	Distal Interger	12	18514510	18535254	20745	1	236366	uc007ndb.2	151420	ENSMUSG000	5730507C01R	RIKEN cDNA 5730507C01 gene
chr12	18676557	18676690	134	135	18676627	19	7.07687	3.59476	4.08458	Adipose_R1_peak_	Distal Interger	12	18514510	18535254	20745	1	236366	uc007ndb.2	162047	ENSMUSG000	5730507C01R	RIKEN cDNA 5730507C01 gene
chr12	18676568	18676732	165	166	18676658	37	11.12817	3.50427	7.83613	Adipose_R2_peak_	Distal Interger	12	18514510	18535254	20745	1	236366	uc007ndb.2	162058	ENSMUSG000	5730507C01R	RIKEN cDNA 5730507C01 gene
chr12	18756639	18756895	257	258	18756759	50	34.50784	9.16663	30.72138	Adipose_R1_peak_	Distal Interger	12	18514510	18535254	20745	1	236366	uc007ndb.2	242129	ENSMUSG000	5730507C01R	RIKEN cDNA 5730507C01 gene
chr12	18756681	18756882	202	203	18756753	40	12.96465	3.78093	9.57654	Adipose_R2_peak_	Distal Interger	12	18514510	18535254	20745	1	236366	uc007ndb.2	242171	ENSMUSG000	5730507C01R	RIKEN cDNA 5730507C01 gene
chr12	18783181	18783383	203	204	18783289	33	8.83596	3.1354	5.68652	Adipose_R2_peak_	Distal Interger	12	18514510	18535254	20745	1	236366	uc007ndb.2	268671	ENSMUSG000	5730507C01R	RIKEN cDNA 5730507C01 gene
chr12	18838226	18838327	102	103	18838315	29	6.74062	2.76653	3.75989	Adipose_R2_peak_	Distal Interger	12	18514510	18535254	20745	1	236366	uc007ndb.2	323716	ENSMUSG000	5730507C01R	RIKEN cDNA 5730507C01 gene
chr12	18856515	18856894	380	381	18856622	33	6.98167	2.65786	3.99161	Adipose_R2_peak_	Distal Interger	12	18514510	18535254	20745	1	236366	uc007ndb.2	342005	ENSMUSG000	5730507C01R	RIKEN cDNA 5730507C01 gene
chr12	18856750	18856921	172	173	18856812	24	5.38781	2.60274	2.57153	Adipose_R1_peak_	Distal Interger	12	18514510	18535254	20745	1	236366	uc007ndb.2	342240	ENSMUSG000	5730507C01R	RIKEN cDNA 5730507C01 gene
chr12	18859021	18859187	167	168	18859118	36	10.5378	3.41206	7.27949	Adipose_R2_peak_	Distal Interger	12	18514510	18535254	20745	1	236366	uc007ndb.2	344511	ENSMUSG000	5730507C01R	RIKEN cDNA 5730507C01 gene
chr12	18861309	18861478	170	171	18861412	37	11.12817	3.50427	7.83613	Adipose_R2_peak_	Distal Interger	12	18514510	18535254	20745	1	236366	uc007ndb.2	346799	ENSMUSG000	5730507C01R	RIKEN cDNA 5730507C01 gene
chr12	18869543	18869786	244	245	18869643	37	11.12817	3.50427	7.83613	Adipose_R2_peak_	Distal Interger	12	18514510	18535254	20745	1	236366	uc007ndb.2	355033	ENSMUSG000	5730507C01R	RIKEN cDNA 5730507C01 gene
chr12	18873761	18874078	318	319	18873910	42	11.07147	3.22322	7.79685	Adipose_R2_peak_	Distal Interger	12	18514510	18535254	20745	1	236366	uc007ndb.2	359251	ENSMUSG000	5730507C01R	RIKEN cDNA 5730507C01 gene
chr12	18879131	18879267	137	138	18879177	28	6.25023	2.67431	3.31626	Adipose_R2_peak_	Distal Interger	12	18514510	18535254	20745	1	236366	uc007ndb.2	364621	ENSMUSG000	5730507C01R	RIKEN cDNA 5730507C01 gene
chr12	18880826	18880958	133	134	18880871	28	6.25023	2.67431	3.31626	Adipose_R2_peak_	Distal Interger	12	18514510	18535254	20745	1	236366	uc007ndb.2	366316	ENSMUSG000	5730507C01R	RIKEN cDNA 5730507C01 gene
chr12	18886060	18886447	388	389	18886129	33	8.83596	3.1354	5.68652	Adipose_R2_peak_	Distal Interger	12	18514510	18535254	20745	1	236366	uc007ndb.2	371550	ENSMUSG000	5730507C01R	RIKEN cDNA 5730507C01 gene
chr12	18886558	18886661	104	105	18886624	28	6.25023	2.67431	3.31626	Adipose_R2_peak_	Distal Interger	12	18514510	18535254	20745	1	236366	uc007ndb.2	372048	ENSMUSG000	5730507C01R	RIKEN cDNA 5730507C01 gene
chr12	18891754	18891880	127	128	18891821	26	5.31252	2.48988	2.47999	Adipose_R2_peak_	Distal Interger	12	18514510	18535254	20745	1	236366	uc007ndb.2	377244	ENSMUSG000	5730507C01R	RIKEN cDNA 5730507C01 gene
chr12	18892869	18893249	381	382	18892980	37	11.09221	3.49503	7.81702	Adipose_R2_peak_	Distal Interger	12	18514510	18535254	20745	1	236366	uc007ndb.2	378359	ENSMUSG000	5730507C01R	RIKEN cDNA 5730507C01 gene
chr12	18894428	18894666	239	240	18894520	33	8.83596	3.1354	5.68652	Adipose_R2_peak_	Distal Interger	12	18514510	18535254	20745	1	236366	uc007ndb.2	379918	ENSMUSG000	5730507C01R	RIKEN cDNA 5730507C01 gene
chr12	18895333	18895432	100	101	18895399	27	5.77405	2.5821	2.88921	Adipose_R2_peak_	Distal Interger	12	18514510	18535254	20745	1	236366	uc007ndb.2	380823	ENSMUSG000	5730507C01R	RIKEN cDNA 5730507C01 gene
chr12	18896258	18896490	233	234	18896310	32	8.29282	3.04319	5.18352	Adipose_R2_peak_	Distal Interger	12	18514510	18535254	20745	1	236366	uc007ndb.2	381748	ENSMUSG000	5730507C01R	RIKEN cDNA 5730507C01 gene
chr12	18901589	18901799	211	212	18901725	31	7.59917	2.90653	4.55975	Adipose_R2_peak_	Distal Interger	12	18514510	18535254	20745	1	236366	uc007ndb.2	387079	ENSMUSG000	5730507C01R	RIKEN cDNA 5730507C01 gene
chr12	18902442	18902616	175	176	18902542	30	7.24477	2.85875	4.21917	Adipose_R2_peak_	Distal Interger	12	18514510	18535254	20745	1	236366	uc007ndb.2	387932	ENSMUSG000	5730507C01R	RIKEN cDNA 5730507C01 gene
chr12	18903213	18903408	196	197	18903294	38	11.72962	3.59649	8.4047	Adipose_R2_peak_	Distal Interger	12	18514510	18535254	20745	1	236366	uc007ndb.2	388703	ENSMUSG000	5730507C01R	RIKEN cDNA 5730507C01 gene
chr12	18907565	18908089	525	526	18907661	32	8.29282	3.04319	5.18352	Adipose_R2_peak_	Distal Interger	12	18514510	18535254	20745	1	236366	uc007ndb.2	393055	ENSMUSG000	5730507C01R	RIKEN cDNA 5730507C01 gene
chr12	18913182	18913293	112	113	18913248	27	5.77405	2.5821	2.88921	Adipose_R2_peak_	Distal Interger	12	18514510	18535254	20745	1	236366	uc007ndb.2	398672	ENSMUSG000	5730507C01R	RIKEN cDNA 5730507C01 gene
chr12	18914071	18914213	143	144	18914185	28	6.25023	2.67431	3.31626	Adipose_R2_peak_	Distal Interger	12	18514510	18535254	20745	1	236366	uc007ndb.2	399561	ENSMUSG000	5730507C01R	RIKEN cDNA 5730507C01 gene
chr12	18914584	18914857	274	275	18914654	30	7.24477	2.85875	4.21917	Adipose_R2_peak_	Distal Interger	12	18514510	18535254	20745	1	236366	uc007ndb.2	400074	ENSMUSG000	5730507C01R	RIKEN cDNA 5730507C01 gene
chr12	18915671	18915851	181	182	18915776	35	9.95877	3.31984	6.73562	Adipose_R2_peak_	Distal Interger	12	18514510	18535254	20745	1	236366	uc007ndb.2	401161	ENSMUSG000	5730507C01R	RIKEN cDNA 5730507C01 gene
chr12	18917903	18918145	243	244	18918087	34	9.39139	3.22762	6.20474	Adipose_R2_peak_	Distal Interger	12	18514510	18535254	20745	1	236366	uc007ndb.2	403393	ENSMUSG000	5730507C01R	RIKEN cDNA 5730507C01 gene
chr12	18921722	18921848	127	128	18921800	29	6.74062	2.76653	3.75989	Adipose_R2_peak_	Distal Interger	12	18514510	18535254	20745	1	236366	uc007ndb.2	407212	ENSMUSG000	5730507C01R	RIKEN cDNA 5730507C01 gene
chr12	18922740	18922949	210	211	18922844	31	7.7623	2.95097	4.69388	Adipose_R2_peak_	Distal Interger	12	18514510	18535254	20745	1	236366	uc007ndb.2	408230	ENSMUSG000	5730507C01R	RIKEN cDNA 5730507C01 gene
chr12	18923424	18923560	137	138	18923493	31	7.7623	2.95097	4.69388	Adipose_R2_peak_	Distal Interger	12	18514510	18535254	20745	1	236366	uc007ndb.2	408914	ENSMUSG000	5730507C01R	RIKEN cDNA 5730507C01 gene
chr12	18923707	18923853	147	148	18923791	29	6.74062	2.76653	3.75989	Adipose_R2_peak_	Distal Interger	12	18514510	18535254	20745	1	236366	uc007ndb.2	409197	ENSMUSG000	5730507C01R	RIKEN cDNA 5730507C01 gene
chr12	18924276	18924394	119	120	18924320	27	5.77405	2.5821	2.88921	Adipose_R2_peak_	Distal Interger	12	18514510	18535254	20745	1	236366	uc007ndb.2	409766	ENSMUSG000	5730507C01R	RIKEN cDNA 5730507C01 gene
chr12	18927069	18927374	306	307	18927230	34	7.9551	2.8586	4.87984	Adipose_R2_peak_	Distal Interger	12	18514510	18535254	20745	1	236366	uc007ndb.2	412559	ENSMUSG000	5730507C01R	RIKEN cDNA 5730507C01 gene
chr12	18927708	18928060	353	354	18927906	33	8.83596	3.1354	5.68652	Adipose_R2_peak_	Distal Interger	12	18514510	18535254	20745	1	236366	uc007ndb.2	413198	ENSMUSG000	5730507C01R	RIKEN cDNA 5730507C01 gene
chr12	18931624	18931759	136	137	18931715	27	5.77405	2.5821	2.88921	Adipose_R2_peak_	Distal Interger	12	18514510	18535254	20745	1	236366	uc007ndb.2	417114	ENSMUSG000	5730507C01R	RIKEN cDNA 5730507C01 gene
chr12	18934083	18934176	94	95	18934120	27	5.77405	2.5821	2.88921	Adipose_R2_peak_	Distal Interger	12	18514510	18535254	20745	1	236366	uc007ndb.2	419573	ENSMUSG000	5730507C01R	RIKEN cDNA 5730507C01 gene
chr12	18947120	18947290	171	172	18947236	22	9.13959	4.13397	6.01526	Adipose_R1_peak_	Distal Interger	12	18514510	18535254	20745	1	236366	uc007ndb.2	432610	ENSMUSG000	5730507C01R	RIKEN cDNA 5730507C01 gene
chr12	18949231	18949364	134	135	18949317	28	6.25023	2.67431	3.31626	Adipose_R2_peak_	Distal Interger	12	18514510	18535254	20745	1	236366	uc007ndb.2	434721	ENSMUSG000	5730507C01R	RIKEN cDNA 5730507C01 gene
chr12	18953522	18953721	200	201	18953595	29	6.74062	2.76653	3.75989	Adipose_R2_peak_	Distal Interger	12	18514510	18535254	20745	1	236366	uc007ndb.2	439012	ENSMUSG000	5730507C01R	RIKEN cDNA 5730507C01 gene
chr12	18953963	18954314	352	353	18954084	31	7.7623	2.95097	4.69388	Adipose_R2_peak_	Distal Interger	12	18514510	18535254	20745	1	236366	uc007ndb.2	439453	ENSMUSG000	5730507C01R	RIKEN cDNA 5730507C01 gene
chr12	18954719	18954935	217	218	18954857	28	9.88225	3.79663	6.72012	Adipose_R1_peak_	Distal Interger	12	18514510	18535254	20745	1	236366	uc007ndb.2	440209	ENSMUSG000	5730507C01R	RIKEN cDNA 5730507C01 gene
chr12	18954755	18954938	184	185	18954847	26	5.31252	2.48988	2.47999	Adipose_R2_peak_	Distal Interger	12	18514510	18535254	20745	1	236366	uc007ndb.2	440245	ENSMUSG000	5730507C01R	RIKEN cDNA 5730507C01 gene
chr12	18957898	18958027	130	131	18957937	26	5.31252	2.48988	2.47999	Adipose_R2_peak_	Distal Interger	12	18514510	18535254	20745	1	236366	uc007ndb.2	443388	ENSMUSG000	5730507C01R	RIKEN cDNA 5730507C01 gene
chr12	18960350	18960594	245	246	18960449	33	8.83596	3.1354	5.68652	Adipose_R2_peak_	Distal Interger	12	18514510	18535254	20745	1	236366	uc007ndb.2	445840	ENSMUSG000	5730507C01R	RIKEN cDNA 5730507C01 gene
chr12	18964960	18965313	354	355	18965131	41	13.59774	3.87315	10.1803	Adipose_R2_peak_	Distal Interger	12	18514510	18535254	20745	1	236366	uc007ndb.2	450450	ENSMUSG000	5730507C01R	RIKEN cDNA 5730507C01 gene
chr12	18965445	18965567	123	124	18965500	31	7.7623	2.95097	4.69388	Adipose_R2_peak_	Distal Interger	12	18514510	18535254	20745	1	236366	uc007ndb.2	450935	ENSMUSG000	5730507C01R	RIKEN cDNA 5730507C01 gene
chr12	18969696	18969837	142	143	18969779	28	6.25023	2.67431	3.31626	Adipose_R2_peak_	Distal Interger	12	18514510	18535254	20745	1	236366	uc007ndb.2	455186	ENSMUSG000	5730507C01R	RIKEN cDNA 5730507C01 gene
chr12	18985974	18986086	113	114	18986011	25	4.86612	2.39766	2.09018	Adipose_R2_peak_	Distal Interger	12	18514510	18535254	20745	1	236366	uc007ndb.2	471464	ENSMUSG000	5730507C01R	RIKEN cDNA 5730507C01 gene
chr12	18992248	18992380	133	134	18992338	25	7.48711	3.23056	4.4898	Adipose_R1_peak_	Distal Interger	12	18514510	18535254	20745	1	236366	uc007ndb.2	477738	ENSMUSG000	5730507C01R	RIKEN cDNA 5730507C01 gene
chr12	18992309	18992562	254	255	18992389	31	7.33762	2.83589	4.30844	Adipose_R2_peak_	Distal Interger	12	18514510	18535254	20745	1	236366	uc007ndb.2	477799	ENSMUSG000	5730507C01R	RIKEN cDNA 5730507C01 gene
chr12	18996898	18997052	155	156	18996920	16	5.20017	3.05554	2.38734	Adipose_R1_peak_	Distal Interger	12	18514510	18535254	20745	1	236366	uc007ndb.2	482388	ENSMUSG000	5730507C01R	RIKEN cDNA 5730507C01 gene
chr12	18999536	18999700	165	166	18999641	33	8.83596	3.1354	5.68652	Adipose_R2_peak_	Distal Interger	12	18514510	18535254	20745	1	236366	uc007ndb.2	485026	ENSMUSG000	5730507C01R	RIKEN cDNA 5730507C01 gene
chr12	19009804	19010141	338	339	19009934	29	6.74062	2.76653	3.75989	Adipose_R2_peak_	Distal Interger	12	18514510	18535254	20745	1	236366	uc007ndb.2	495294	ENSMUSG000	5730507C01R	RIKEN cDNA 5730507C01 gene
chr12	19012361	19012559	199	200	19012455	29	6.74062	2.76653	3.75989	Adipose_R2_peak_	Distal Interger	12	18514510	18535254	20745	1	236366	uc007ndb.2	497851	ENSMUSG000	5730507C01R	RIKEN cDNA 5730507C01 gene
chr12	19018777	19018948	172	173	19018789	26	5.31252	2.48988	2.47999	Adipose_R2_peak_	Distal Interger	12	18514510	18535254	20745	1	236366	uc007ndb.2	504267	ENSMUSG000	5730507C01R	RIKEN cDNA 5730507C01 gene
chr12	19020392	19020626	235	236	19020442	28	6.25023	2.67431	3.31626	Adipose_R2_peak_	Distal Interger	12	18514510	18535254	20745	1	236366	uc007ndb.2	505882	ENSMUSG000	5730507C01R	RIKEN cDNA 5730507C01 gene
chr12	19023454	19023615	162	163	19023514	29	6.74062	2.76653	3.75989	Adipose_R2_peak_	Distal Interger	12	18514510	18535254	20745	1	236366	uc007ndb.2	508944	ENSMUSG000	5730507C01R	RIKEN cDNA 5730507C01 gene
chr12	19025732	19025909	178	179	19025797	29	6.74062	2.76653	3.75989	Adipose_R2_peak_	Distal Interger	12	18514510	18535254	20745	1	236366	uc007ndb.2	511222	ENSMUSG000	5730507C01R	RIKEN cDNA 5730507C01 gene
chr12	19039773	19039884	112	113	19039818	31	6.72563	2.67346	3.75989	Adipose_R2_peak_	Distal Interger	12	18514510	18535254	20745	1	236366	uc007ndb.2	525263	ENSMUSG000	5730507C01R	RIKEN cDNA 5730507C01 gene
chr12	19045727	19046035	309	310	19045784	27	5.77405	2.5821	2.88921	Adipose_R2_peak_	Distal Interger	12	18514510	18535254	20745	1	236366	uc007ndb.2	531217	ENSMUSG000	5730507C01R	RIKEN cDNA 5730507C01 gene
chr12	19052600	19052757	158	159	19052661	26	5.31252	2.48988	2.47999	Adipose_R2_peak_	Distal Interger	12	18514510	18535254	20745	1	236366	uc007ndb.2	538090	ENSMUSG000	5730507C01R	RIKEN cDNA 5730507C01 gene
chr12	19053679	19053827	149	150	19053724	28	6.25023	2.67431	3.31626	Adipose_R2_peak_	Distal Interger	12	18514510	18535254	20745	1	236366	uc007ndb.2	539169	ENSMUSG000	5730507C01R	RIKEN cDNA 5730507C01 gene
chr12	19054664	19054785	122	123	19054711	34	9.39139	3.22762	6.20474	Adipose_R2_peak_	Distal Interger	12	18514510	18535254	20745	1	236366	uc007ndb.2	540154	ENSMUSG000	5730507C01R	RIKEN cDNA 5730507C01 gene
chr12	19054686	19054825	140	141	19054732	20	6.43765	3.23526	3.49812	Adipose_R1_peak_	Distal Interger	12	18514510	18535254	20745	1	236366	uc007ndb.2	540176	ENSMUSG000	5730507C01R	RIKEN cDNA 5730507C01 gene
chr12	19055016	19055179	164	165	19055128	29	6.74062	2.76653	3.75989	Adipose_R2_peak_	Distal Interger	12	18514510	18535254	20745	1	236366	uc007ndb.2	540506	ENSMUSG000	5730507C01R	RIKEN cDNA 5730507C01 gene
chr12	19055043	19055183	141	142	19055066	16	5.20017	3.05554	2.38734	Adipose_R1_peak_	Distal Interger	12	18514510	18535254	20745	1	236366	uc007ndb.2	540533	ENSMUSG000	5730507C01R	RIKEN cDNA 5730507C01 gene
chr12	19072527	19072638	112	113	19072583	25	4.86612	2.39766	2.09018	Adipose_R2_peak_	Distal Interger	12	18514510	18535254	20745	1	236366	uc007ndb.2	558017	ENSMUSG000	5730507C01R	RIKEN cDNA 5730507C01 gene
chr12	19074456	19074577	122	123	19074501	26	5.31252	2.48988	2.47999	Adipose_R2_peak_	Distal Interger	12	18514510	18535254	20745	1	236366	uc007ndb.2	559946	ENSMUSG000	5730507C01R	RIKEN cDNA 5730507C01 gene

chr12	19226637	19226898	262	263	19226737	35	9.95877	3.31984	6.73562	Adipose_R2_peak_	Distal Interger	12	18514510	18535254	20745	1	236366	uc007ndb.2	712127	ENSMUSG000	5730507C01Ri	RIKEN cDNA 5730507C01 gene
chr12	19231412	19231522	111	112	19231456	28	6.25023	2.67431	3.31626	Adipose_R2_peak_	Distal Interger	12	18514510	18535254	20745	1	236366	uc007ndb.2	716902	ENSMUSG000	5730507C01Ri	RIKEN cDNA 5730507C01 gene
chr12	19232230	19232500	271	272	19232346	37	7.49747	2.632	4.46207	Adipose_R2_peak_	Distal Interger	12	18514510	18535254	20745	1	236366	uc007ndb.2	717720	ENSMUSG000	5730507C01Ri	RIKEN cDNA 5730507C01 gene
chr12	19233299	19233455	157	158	19233348	27	5.77405	2.5821	2.88921	Adipose_R2_peak_	Distal Interger	12	18514510	18535254	20745	1	236366	uc007ndb.2	718789	ENSMUSG000	5730507C01Ri	RIKEN cDNA 5730507C01 gene
chr12	19251752	19251913	162	163	19251843	31	7.7623	2.95097	4.69388	Adipose_R2_peak_	Distal Interger	12	18514510	18535254	20745	1	236366	uc007ndb.2	737242	ENSMUSG000	5730507C01Ri	RIKEN cDNA 5730507C01 gene
chr12	19253452	19253647	196	197	19253563	32	8.29282	3.04319	5.18352	Adipose_R2_peak_	Distal Interger	12	18514510	18535254	20745	1	236366	uc007ndb.2	738942	ENSMUSG000	5730507C01Ri	RIKEN cDNA 5730507C01 gene
chr12	19254075	19254218	144	145	19254157	28	6.25023	2.67431	3.31626	Adipose_R2_peak_	Distal Interger	12	18514510	18535254	20745	1	236366	uc007ndb.2	739565	ENSMUSG000	5730507C01Ri	RIKEN cDNA 5730507C01 gene
chr12	19270756	19270858	103	104	19270809	29	6.74062	2.76653	3.75989	Adipose_R2_peak_	Distal Interger	12	18514510	18535254	20745	1	236366	uc007ndb.2	756246	ENSMUSG000	5730507C01Ri	RIKEN cDNA 5730507C01 gene
chr12	19317285	19317460	176	177	19317357	28	6.25023	2.67431	3.31626	Adipose_R2_peak_	Distal Interger	12	18514510	18535254	20745	1	236366	uc007ndb.2	802775	ENSMUSG000	5730507C01Ri	RIKEN cDNA 5730507C01 gene
chr12	19318369	19318513	145	146	19318462	29	6.74062	2.76653	3.75989	Adipose_R2_peak_	Distal Interger	12	18514510	18535254	20745	1	236366	uc007ndb.2	803859	ENSMUSG000	5730507C01Ri	RIKEN cDNA 5730507C01 gene
chr12	19320955	19321107	153	154	19321034	27	5.77405	2.5821	2.88921	Adipose_R2_peak_	Distal Interger	12	18514510	18535254	20745	1	236366	uc007ndb.2	806445	ENSMUSG000	5730507C01Ri	RIKEN cDNA 5730507C01 gene
chr12	19321640	19321860	221	222	19321751	36	10.5378	3.41206	7.27949	Adipose_R2_peak_	Distal Interger	12	18514510	18535254	20745	1	236366	uc007ndb.2	807130	ENSMUSG000	5730507C01Ri	RIKEN cDNA 5730507C01 gene
chr12	19322611	19322764	154	155	19322730	30	7.24477	2.85875	4.21917	Adipose_R2_peak_	Distal Interger	12	18514510	18535254	20745	1	236366	uc007ndb.2	808101	ENSMUSG000	5730507C01Ri	RIKEN cDNA 5730507C01 gene
chr12	19323183	19323294	112	113	19323198	25	4.86612	2.39766	2.09018	Adipose_R2_peak_	Distal Interger	12	18514510	18535254	20745	1	236366	uc007ndb.2	808673	ENSMUSG000	5730507C01Ri	RIKEN cDNA 5730507C01 gene
chr12	19324211	19324535	325	326	19324409	32	8.29282	3.04319	5.18352	Adipose_R2_peak_	Distal Interger	12	18514510	18535254	20745	1	236366	uc007ndb.2	809701	ENSMUSG000	5730507C01Ri	RIKEN cDNA 5730507C01 gene
chr12	19324958	19325369	412	413	19325015	36	8.98762	3.02195	5.83278	Adipose_R2_peak_	Distal Interger	12	18514510	18535254	20745	1	236366	uc007ndb.2	810448	ENSMUSG000	5730507C01Ri	RIKEN cDNA 5730507C01 gene
chr12	19325812	19326016	205	206	19325925	36	9.5616	3.16367	6.36886	Adipose_R2_peak_	Distal Interger	12	18514510	18535254	20745	1	236366	uc007ndb.2	811302	ENSMUSG000	5730507C01Ri	RIKEN cDNA 5730507C01 gene
chr12	19326315	19326459	145	146	19326394	30	7.24477	2.85875	4.21917	Adipose_R2_peak_	Distal Interger	12	18514510	18535254	20745	1	236366	uc007ndb.2	811805	ENSMUSG000	5730507C01Ri	RIKEN cDNA 5730507C01 gene
chr12	19329757	19329944	188	189	19329884	28	6.25023	2.67431	3.31626	Adipose_R2_peak_	Distal Interger	12	18514510	18535254	20745	1	236366	uc007ndb.2	815247	ENSMUSG000	5730507C01Ri	RIKEN cDNA 5730507C01 gene
chr12	19331321	19331462	142	143	19331423	28	6.25023	2.67431	3.31626	Adipose_R2_peak_	Distal Interger	12	18514510	18535254	20745	1	236366	uc007ndb.2	816811	ENSMUSG000	5730507C01Ri	RIKEN cDNA 5730507C01 gene
chr12	19336821	19337275	455	456	19336892	27	5.77405	2.5821	2.88921	Adipose_R2_peak_	Distal Interger	12	18514510	18535254	20745	1	236366	uc007ndb.2	822311	ENSMUSG000	5730507C01Ri	RIKEN cDNA 5730507C01 gene
chr12	19338497	19338683	187	188	19338540	26	5.31252	2.48988	2.47999	Adipose_R2_peak_	Distal Interger	12	18514510	18535254	20745	1	236366	uc007ndb.2	823987	ENSMUSG000	5730507C01Ri	RIKEN cDNA 5730507C01 gene
chr12	19339816	19339940	125	126	19339889	28	6.25023	2.67431	3.31626	Adipose_R2_peak_	Distal Interger	12	18514510	18535254	20745	1	236366	uc007ndb.2	825306	ENSMUSG000	5730507C01Ri	RIKEN cDNA 5730507C01 gene
chr12	19341028	19341148	121	122	19341073	29	6.74062	2.76653	3.75989	Adipose_R2_peak_	Distal Interger	12	18514510	18535254	20745	1	236366	uc007ndb.2	826518	ENSMUSG000	5730507C01Ri	RIKEN cDNA 5730507C01 gene
chr12	19343365	19343675	311	312	19343470	35	9.95877	3.31984	6.73562	Adipose_R2_peak_	Distal Interger	12	18514510	18535254	20745	1	236366	uc007ndb.2	828855	ENSMUSG000	5730507C01Ri	RIKEN cDNA 5730507C01 gene
chr12	19348156	19348350	195	196	19348265	27	5.77405	2.5821	2.88921	Adipose_R2_peak_	Distal Interger	12	18514510	18535254	20745	1	236366	uc007ndb.2	833646	ENSMUSG000	5730507C01Ri	RIKEN cDNA 5730507C01 gene
chr12	19352256	19352593	338	339	19352522	28	6.25023	2.67431	3.31626	Adipose_R2_peak_	Distal Interger	12	18514510	18535254	20745	1	236366	uc007ndb.2	837746	ENSMUSG000	5730507C01Ri	RIKEN cDNA 5730507C01 gene
chr12	19369552	19369669	118	119	19369563	25	4.86612	2.39766	2.09018	Adipose_R2_peak_	Distal Interger	12	18514510	18535254	20745	1	236366	uc007ndb.2	855042	ENSMUSG000	5730507C01Ri	RIKEN cDNA 5730507C01 gene
chr12	19375885	19376044	160	161	19375937	30	7.24477	2.85875	4.21917	Adipose_R2_peak_	Distal Interger	12	18514510	18535254	20745	1	236366	uc007ndb.2	861375	ENSMUSG000	5730507C01Ri	RIKEN cDNA 5730507C01 gene
chr12	19378622	19378766	145	146	19378690	29	6.74062	2.76653	3.75989	Adipose_R2_peak_	Distal Interger	12	18514510	18535254	20745	1	236366	uc007ndb.2	864112	ENSMUSG000	5730507C01Ri	RIKEN cDNA 5730507C01 gene
chr12	19380496	19380656	161	162	19380626	29	6.74062	2.76653	3.75989	Adipose_R2_peak_	Distal Interger	12	18514510	18535254	20745	1	236366	uc007ndb.2	865986	ENSMUSG000	5730507C01Ri	RIKEN cDNA 5730507C01 gene
chr12	19382913	19383012	100	101	19382926	27	5.77405	2.5821	2.88921	Adipose_R2_peak_	Distal Interger	12	18514510	18535254	20745	1	236366	uc007ndb.2	868403	ENSMUSG000	5730507C01Ri	RIKEN cDNA 5730507C01 gene
chr12	19391061	19391238	178	179	19391099	18	5.19079	2.89064	2.38734	Adipose_R1_peak_	Intron (uc007r	12	18514510	18535254	20745	1	236366	uc007ndb.2	876551	ENSMUSG000	5730507C01Ri	RIKEN cDNA 5730507C01 gene
chr12	19460859	19461019	161	162	19460904	27	5.77405	2.5821	2.88921	Adipose_R2_peak_	Distal Interger	12	18514510	18535254	20745	1	236366	uc007ndb.2	946349	ENSMUSG000	5730507C01Ri	RIKEN cDNA 5730507C01 gene
chr12	19517660	19517992	333	334	19517705	28	6.25023	2.67431	3.31626	Adipose_R2_peak_	Distal Interger	12	18514510	18535254	20745	1	236366	uc007ndb.2	1003150	ENSMUSG000	5730507C01Ri	RIKEN cDNA 5730507C01 gene
chr12	19518119	19518261	143	144	19518199	27	5.77405	2.5821	2.88921	Adipose_R2_peak_	Distal Interger	12	18514510	18535254	20745	1	236366	uc007ndb.2	1003609	ENSMUSG000	5730507C01Ri	RIKEN cDNA 5730507C01 gene
chr12	19518705	19518863	159	160	19518719	26	5.31252	2.48988	2.47999	Adipose_R2_peak_	Distal Interger	12	18514510	18535254	20745	1	236366	uc007ndb.2	1004195	ENSMUSG000	5730507C01Ri	RIKEN cDNA 5730507C01 gene
chr12	19520554	19520692	139	140	19520568	27	5.77405	2.5821	2.88921	Adipose_R2_peak_	Distal Interger	12	18514510	18535254	20745	1	236366	uc007ndb.2	1006044	ENSMUSG000	5730507C01Ri	RIKEN cDNA 5730507C01 gene
chr12	19524632	19524769	138	139	19524638	25	4.86612	2.39766	2.09018	Adipose_R2_peak_	Distal Interger	12	18514510	18535254	20745	1	236366	uc007ndb.2	1010122	ENSMUSG000	5730507C01Ri	RIKEN cDNA 5730507C01 gene
chr12	19524908	19525056	149	150	19524925	26	5.31252	2.48988	2.47999	Adipose_R2_peak_	Distal Interger	12	18514510	18535254	20745	1	236366	uc007ndb.2	1010398	ENSMUSG000	5730507C01Ri	RIKEN cDNA 5730507C01 gene
chr12	19534501	19534636	136	137	19534561	31	7.7623	2.95097	4.69388	Adipose_R2_peak_	Distal Interger	12	18514510	18535254	20745	1	236366	uc007ndb.2	1019991	ENSMUSG000	5730507C01Ri	RIKEN cDNA 5730507C01 gene
chr12	19538923	19539122	200	201	19539030	27	5.53046	2.51193	2.68965	Adipose_R2_peak_	Distal Interger	12	18514510	18535254	20745	1	236366	uc007ndb.2	1024413	ENSMUSG000	5730507C01Ri	RIKEN cDNA 5730507C01 gene
chr12	19541400	19541517	118	119	19541443	28	6.25023	2.67431	3.31626	Adipose_R2_peak_	Distal Interger	12	18514510	18535254	20745	1	236366	uc007ndb.2	1026890	ENSMUSG000	5730507C01Ri	RIKEN cDNA 5730507C01 gene
chr12	19542225	19542376	152	153	19542285	31	7.7623	2.95097	4.69388	Adipose_R2_peak_	Distal Interger	12	18514510	18535254	20745	1	236366	uc007ndb.2	1027715	ENSMUSG000	5730507C01Ri	RIKEN cDNA 5730507C01 gene
chr12	19542558	19542982	425	426	19542666	35	9.95877	3.31984	6.73562	Adipose_R2_peak_	Distal Interger	12	18514510	18535254	20745	1	236366	uc007ndb.2	1028048	ENSMUSG000	5730507C01Ri	RIKEN cDNA 5730507C01 gene
chr12	19544949	19545448	500	501	19545215	42	14.2409	3.96536	10.79479	Adipose_R2_peak_	Distal Interger	12	18514510	18535254	20745	1	236366	uc007ndb.2	1030439	ENSMUSG000	5730507C01Ri	RIKEN cDNA 5730507C01 gene
chr12	19546516	19546777	262	263	19546631	29	6.74062	2.76653	3.75989	Adipose_R2_peak_	Distal Interger	12	18514510	18535254	20745	1	236366	uc007ndb.2	1032006	ENSMUSG000	5730507C01Ri	RIKEN cDNA 5730507C01 gene
chr12	19548992	19549589	598	599	19549410	49	15.7181	3.86717	12.21451	Adipose_R2_peak_	Distal Interger	12	18514510	18535254	20745	1	236366	uc007ndb.2	1034482	ENSMUSG000	5730507C01Ri	RIKEN cDNA 5730507C01 gene
chr12	19550854	19551250	397	398	19550906	27	5.77405	2.5821	2.88921	Adipose_R2_peak_	Distal Interger	12	18514510	18535254	20745	1	236366	uc007ndb.2	1036344	ENSMUSG000	5730507C01Ri	RIKEN cDNA 5730507C01 gene
chr12	19552711	19552818	108	109	19552784	26	5.31252	2.48988	2.47999	Adipose_R2_peak_	Distal Interger	12	18514510	18535254	20745	1	236366	uc007ndb.2	1038201	ENSMUSG000	5730507C01Ri	RIKEN cDNA 5730507C01 gene
chr12	19568782	19568929	148	149	19568847	25	4.86612	2.39766	2.09018	Adipose_R2_peak_	Distal Interger	12	18514510	18535254	20745	1	236366	uc007ndb.2	1054272	ENSMUSG000	5730507C01Ri	RIKEN cDNA 5730507C01 gene
chr12	19570415	19570599	185	186	19570532	34	9.39139	3.22762	6.20474	Adipose_R2_peak_	Distal Interger	12	18514510	18535254	20745	1	236366	uc007ndb.2	1055905	ENSMUSG000	5730507C01Ri	RIKEN cDNA 5730507C01 gene
chr12	19576038	19576238	201	202	19576127	20	7.74501	3.7745	4.70467	Adipose_R1_peak_	Distal Interger	12	18514510	18535254	20745	1	236366	uc007ndb.2	1061528	ENSMUSG000	5730507C01Ri	RIKEN cDNA 5730507C01 gene
chr12	19577795	19577888	94	95	19577835	25	4.86612	2.39766	2.09018	Adipose_R2_peak_	Distal Interger	12	18514510	18535254	20745	1	236366	uc007ndb.2	1063285	ENSMUSG000	5730507C01Ri	RIKEN cDNA 5730507C01 gene
chr12	19578242	19578381	140	141	19578339	26	5.31252	2.48988	2.47999	Adipose_R2_peak_	Distal Interger	12	18514510	18535254	20745	1	236366	uc007ndb.2	1063732	ENSMUSG000	5730507C01Ri	RIKEN cDNA 5730507C01 gene
chr12	19581825	19582200	376	377	19581946	39	12.34187	3.68871	8.98501	Adipose_R2_peak_	Distal Interger	12	18514510	18535254	20745	1	236366	uc007ndb.2	1067315	ENSMUSG000	5730507C01Ri	RIKEN cDNA 5730507C01 gene
chr12	19583817	19584014	198	199	19583945	32	8.29282	3.04319	5.18352	Adipose_R2_peak_	Distal Interger	12	18514510	18535254	20745	1	236366	uc007ndb.2	1069307	ENSMUSG000	5730507C01Ri	RIKEN cDNA 5730507C01 gene
chr12	19584284	19584457	174	175	19584368	28	5.99378	2.60164	3.10061	Adipose_R2_peak_	Distal Interger	12	18514510	18535254	20745	1	236366	uc007ndb.2	1069774	ENSMUSG000	5730507C01Ri	RIKEN cDNA 5730507C01 gene
chr12	19586446	19586732	287	288	19586544	41	12.98549	3.72211	9.59661	Adipose_R2_peak_	Distal Interger	12	18514510	18535254	20745	1	236366	uc007ndb.2	1071936	ENSMUSG000	5730507C01Ri	RIKEN cDNA 5730507C01 gene
chr12	19587866	19588217	352	353	19588013	28	6.25023	2.67431	3.31626	Adipose_R2_peak_	Distal Interger	12	18514510	18535254	20745	1	236366	uc007ndb.2	1073356	ENSMUSG000	5730507C01Ri	RIKEN cDNA 5730507C01 gene
chr12	19588555	19588660	106	107	19588648	27	5.77405	2.5821	2.88921	Adipose_R2_peak_	Distal Interger	12	18514510	18535254	20745	1	236366	uc007ndb.2	1074045	ENSMUSG000	5730507C01Ri	RIKEN cDNA 5730507C01 gene
chr12	19589395	19589565	171	172	19589499	25	6.41812	2.87878	3.48999	Adipose_R1_peak_	Distal Interger	12	18514510	18535254	20745	1	236366	uc007ndb.2	1074885	ENSMUSG000	5730507C01Ri	RIKEN cDNA 5730507C01 gene
chr12	19627247	19627471	225	226	19627382	21	8.43286	3.95423	5.34896	Adipose_R1_peak_	Distal Interger	12	18514510	18535254	20745	1	236366	uc007ndb.2	1112737	ENSMUSG000	5730507C01Ri	RIKEN cDNA 5730507C01 gene
chr12	19696635	19697030	396	397	19696723	21	8.43286	3.95423	5.34896	Adipose_R1_peak_	Distal Interger	12	20804392	20815779	11388	2	69513	uc007nde.2	1118749	ENSMUSG000	1700030C10Ri	RIKEN cDNA 1700030C10 gene
chr12	19710646	19710860	215	216	19710736	31	16.23981	5.75161	12.83549	Adipose_R1_peak_	Distal Interger	12	20804392	20815779	11388	2	69513	uc007nde.2	1104919	ENSMUSG000	1700030C10Ri	RIKEN cDNA 1700030C10 gene
chr12	19711361	19711576	216	217	19711482	27	12.93148	5.03266	9.63827	Adipose_R1_peak_	Distal Interger	12	20804392	20815779	11388	2	69513	uc007nde.2	1104203	ENSMUSG000	1700030C10Ri	RIKEN cDNA 1700030C10 gene
chr12	19933967	19934130	164	165	19934048	22	9.13959	4.13397	6.01526	Adipose_R1_peak_	Distal Interger	12	20804392	20815779	11388	2	69513	uc007nde.2	881649	ENSMUSG000	1700030C10Ri	RIKEN cDNA 1700030C10 gene
chr12	19990244	19990377	134	135	19990300	30	7.24477	2.85875	4.21917	Adipose_R2_peak_	Distal Interger	12	20804392	20815779	11388	2	69513	uc007nde.2	825402	ENSMUSG000	1700030C10Ri	RIKEN cDNA 1700030C10 gene
chr12	20021082	20021241	160	161	20021187	26	5.31252	2.48988	2.47999	Adipose_R2_peak_	Distal Interger	12	20804392	20815779	11388	2	69513	uc007nde.2	794538	ENSMUSG000	1700030C10Ri	RIKEN cDNA 1700030C10 gene
chr12	20143467	20143605	139	140	20143536	29	6.74062	2.76653	3.75989	Adipose_R2_peak_	Distal Interger	12	20804392	20815779	11388	2	69513	uc007nde.2	672174	ENSMUSG000	1700030C10Ri	RIKEN cDNA 1700030C10 gene
chr12	20153669	20153871	203	204	20153796	42	14.2409	3.96536	10.79479	Adipose_R2_peak_	Distal Interger	12	20804392	20815779	11388	2	69513	uc007nde.2	661908	ENSMUSG000	1700030C10Ri	RIKEN cDNA 1700030C10 gene
chr12	20154884	20155047	164	165	20154971	30	7.24477	2.85875	4.21917	Adipose_R2_peak_	Distal Interger	12	20804392	20815779	11388	2	69513	uc007nde.2	660732	ENSMUSG000	1700030C10Ri	RIKEN cDNA 1700030C10 gene
chr12	20155281	20155480	200	201	20155454	27	5.42376	2.4814	2.58701	Adipose_R2_peak_	Distal Interger	12	20804392	20815779	11388	2	69513	uc007nde.2	660299	ENSMUSG000	1700030C10Ri	RIKEN cDNA 1700030C10 gene
chr12	20168962	20169095	134	135	20169051	29	6.74062	2.76653	3.75989	Adipose_R2_peak_	Distal Interger	12	20804392	20815779	11388	2	69513	uc007nde.2	646684	ENSMUSG000	1700030C10Ri	RIKEN cDNA 1700030C10 gene
chr12	20175544	20175674	131	132	20175617	30	7.24477	2.85875	4.21917	Adipose_R2_peak_	Distal Interger	12	20804392	20815779	11388	2	69513	uc007nde.2	640105	ENSMUSG000	1700030C10Ri	RIKEN cDNA 1700030C10 gene
chr12	20177512	20177605	94	95	20177556	32	8.29282	3.04319	5.18352	Adipose_R2_peak_	Distal Interger	12	20804392	20815779	11388	2	69513	uc007nde.2	638174	ENSMUSG000	1700030C10Ri	RIKEN cDNA 1700030C10 gene
chr12	20195346	20195454	109	110	20195430	27	5.77405	2.5821	2.88921	Adipose_R2_peak_	Distal Interger	12	20804392	20815779	11388	2	69513	uc007nde.2	620325	ENSMUSG000	1700030C10Ri	RIKEN cDNA 1700030C10 gene
chr12	20195939	20196050	112	113	20195998	27	5.77405	2.5821	2.88921	Adipose_R2_peak_	Distal Interger	12	20804392	20815779	11388	2	69513	uc007nde.2	619729	ENSMUSG000	1700030C10Ri	RIKEN cDNA 1700030C10 gene
chr12	20226642	20227918	1277	1278	20226963	61	12.48386	2.82085	9.12275	Adipose_R2_peak_	Intron (uc029r	12	20804392	20815779	11388	2	69513	uc007nde.2	587861	ENSMUSG000	1700030C10Ri	RIKEN cDNA 1700030C10 gene
chr12	20226981	20227227	247	248	20227081	32	5.32441	2.28522	2.50937	Adipose_R1_peak_	Intron (uc029r	12	20804392	20815779	11388	2	69513	uc007nde.2	588552	ENSMUSG000	1700030C10Ri	RIKEN cDNA 1700030C10 gene
chr12	20227625	20228126	502	503	20227766	31	5.18707	2.28069	2.38734	Adipose_R1_peak_	Intron (uc029r	12	20804392	20815779	11388	2	69513	uc007nde.2	587653	ENSMUSG000	1700030C10Ri	RIKEN cDNA 1700030C10 gene
chr12	20228087	20233241	5155	5156	20228141	47	8.07552	2.45695	4.9958	Adipose_R2_peak_	Intron (uc029r	12	20804392	20815779	11388	2	69513	uc007nde.2	582538	ENSMUSG000	1700030C10Ri	RIKEN cDNA 1700030C10 gene
chr12	20228561	20228749	189	190	20228625	34	5.54549	2.2813	2.72649	Adipose_R1_peak_	Intron (uc029r	12	20804392	20815779	11388	2	69513	uc007nde.2	587030	ENSMUSG000	1700030C10Ri	RIKEN cDNA 1700030C10 gene
chr12	20229605	20229826	222	223	20229680	35	7.89185	2.80012	4.84994	Adipose_R1_peak_	Intron (uc029r	12	20804392	20815779	11388	2	69513	uc007nde.2	585953	ENSMUSG000	1700030C10Ri	RIKEN cDNA 1700030C10 gene
chr12	20233376	20234177	802	803	20233649	82	24.77906	3.89788	21.01137	Adipose_R2_peak_	Intron (uc029r	12	20804392	20815779	11388	2	69513	uc007nde.2	581602	ENSMUSG000	1700030C10Ri	RIKEN cDNA 1700030C10 gene
chr12	20257837	20257934	98	99	20257882	30	7.24477	2.85875	4.21917	Adipose_R2_peak_	Intron (uc029r	12	20804392	20815779	11388	2	69513	uc007nde.2	557845	ENSMUSG000	1700030C10Ri	RIKEN cDNA 1700030C10 gene
chr12	20288798	20288899	102	103	20288868	26	5.31252	2.48988	2.47999	Adipose_R2_peak_	Intron (uc029r	12	20804392	20815779	11388	2	69513	uc007nde.2	526880	ENSMUSG000	1700030C10Ri	RIKEN cDNA 1700030C10 gene
chr12	20369571	20369725	155	156	20369601	26	5.31252	2.48988	2.47999	Adipose_R2_peak_	Intron (uc029r	12	20804392	20815779	11388	2	69513	uc007nde.2	446054	ENSMUSG000	1700030C10Ri	RIKEN cDNA 1700030C10 gene
chr12	20380671	20380812	142	143	20380682	27	5.77405	2.5821	2.88921	Adipose_R2_peak_	Intron (uc029r	12	20804392	20815779	11388	2	69513	uc007nde.2	434967	ENSMUSG000	1700030C10Ri	RIKEN cDNA 1700030C10 gene
chr12	20397230	20397360	131	132	20397277	26	5.31252	2.48988	2.47999	Adipose_R2_peak_	Intron (uc029r	12	20804392	20815779	11388	2	69513	uc007nde.2	418419	ENSMUSG000	1700030C10Ri	RIKEN cDNA 1700030C10 gene
chr12	20428376	20428539	164	165	20428444	27	5.77405	2.5821	2.88921	Adipose_R2_peak_	Intron (uc029r	12	20804392	20815779	11388	2	69513	uc007nde.2	387240	ENSMUSG000	1700030C10Ri	RIKEN cDNA 1700030C10 gene
chr12	20446027	20446235	209	210	20446231	59	26.5291	5.53306	22.7188	Adipose_R2_peak_	Intron (uc029r	12	20804392	20815779	11388	2	69513	uc007nde.2	369544	ENSMUSG000	1700030C10Ri	RIKEN cDNA 1700030C10 gene
chr12	20446070	20446235	166	167	20446216	21	8.43286	3.95423	5.34896	Adipose_R1_peak_	Intron (uc029r	12	20804392	20815779	11388	2	69513	uc007nde.2	369544	ENSMUSG000	1700030C10Ri	RIKEN cDNA 1700030C10 gene
chr12	20471175	20471333	159	160	20471244	17	5.80341	3.23528	2.92378	Adipose_R1_peak_	Intron (uc029r	12	20804392	20815779	11388	2	69513	uc007nde.2	344446	ENSMUSG000	1700030C10Ri	RIKEN cDNA 1700030C10 gene

chr12	20629748	20630042	295	296	20629882	37	21.59274	6.83004	18.04766	Adipose_R1_peak_	Intron (uc029r	12	20804392	20815779	11388	2	69513	uc007nde.2	185737	ENSMUSG000	1700030C10Ri	RIKEN cDNA 1700030C10 gene
chr12	20908038	20908256	219	220	20908167	23	9.86443	4.31371	6.70247	Adipose_R1_peak_	Intron (uc029r	12	20804392	20815779	11388	2	69513	uc007nde.2	-92259	ENSMUSG000	1700030C10Ri	RIKEN cDNA 1700030C10 gene
chr12	20944494	20944683	190	191	20944639	28	6.25023	2.67431	3.31626	Adipose_R2_peak_	Intron (uc029r	12	20804392	20815779	11388	2	69513	uc007nde.2	-128715	ENSMUSG000	1700030C10Ri	RIKEN cDNA 1700030C10 gene
chr12	21587027	21587289	263	264	21587163	34	18.86162	6.29083	15.38482	Adipose_R1_peak_	Distal Interger	12	21390329	21417581	27253	2	22630	uc007ndx.1	-169446	ENSMUSG000	Ywhaq	tyrosine 3-monooxygenase/tryptophan 5-monooxygenase activation protein, theta polypeptide
chr12	21624864	21625082	219	220	21624985	39	12.34187	3.68871	8.98501	Adipose_R2_peak_	Distal Interger	12	21390329	21417581	27253	2	22630	uc007ndx.1	-207283	ENSMUSG000	Ywhaq	tyrosine 3-monooxygenase/tryptophan 5-monooxygenase activation protein, theta polypeptide
chr12	21966817	21967201	385	386	21966968	31	16.23981	5.75161	12.83549	Adipose_R1_peak_	Distal Interger	12	21390329	21417581	27253	2	22630	uc007ndx.1	-549236	ENSMUSG000	Ywhaq	tyrosine 3-monooxygenase/tryptophan 5-monooxygenase activation protein, theta polypeptide
chr12	22027336	22027535	200	201	22027410	20	7.74501	3.7745	4.70467	Adipose_R1_peak_	Distal Interger	12	21390329	21417581	27253	2	22630	uc007ndx.1	-609755	ENSMUSG000	Ywhaq	tyrosine 3-monooxygenase/tryptophan 5-monooxygenase activation protein, theta polypeptide
chr12	22044616	22044792	177	178	22044749	27	5.77405	2.5821	2.88921	Adipose_R2_peak_	Distal Interger	12	21390329	21417581	27253	2	22630	uc007ndx.1	-627035	ENSMUSG000	Ywhaq	tyrosine 3-monooxygenase/tryptophan 5-monooxygenase activation protein, theta polypeptide
chr12	22046660	22046753	94	95	22046731	28	6.25023	2.67431	3.31626	Adipose_R2_peak_	Distal Interger	12	21390329	21417581	27253	2	22630	uc007ndx.1	-629079	ENSMUSG000	Ywhaq	tyrosine 3-monooxygenase/tryptophan 5-monooxygenase activation protein, theta polypeptide
chr12	22055951	22056087	137	138	22056002	31	7.7623	2.95097	4.69388	Adipose_R2_peak_	Distal Interger	12	21390329	21417581	27253	2	22630	uc007ndx.1	-638370	ENSMUSG000	Ywhaq	tyrosine 3-monooxygenase/tryptophan 5-monooxygenase activation protein, theta polypeptide
chr12	22064652	22064774	123	124	22064723	25	4.86612	2.39766	2.09018	Adipose_R2_peak_	Distal Interger	12	21390329	21417581	27253	2	22630	uc007ndx.1	-647071	ENSMUSG000	Ywhaq	tyrosine 3-monooxygenase/tryptophan 5-monooxygenase activation protein, theta polypeptide
chr12	22065333	22065463	131	132	22065423	27	5.77405	2.5821	2.88921	Adipose_R2_peak_	Distal Interger	12	21390329	21417581	27253	2	22630	uc007ndx.1	-647752	ENSMUSG000	Ywhaq	tyrosine 3-monooxygenase/tryptophan 5-monooxygenase activation protein, theta polypeptide
chr12	22088755	22088922	168	169	22088859	30	7.24477	2.85875	4.21917	Adipose_R2_peak_	Distal Interger	12	21390329	21417581	27253	2	22630	uc007ndx.1	-671174	ENSMUSG000	Ywhaq	tyrosine 3-monooxygenase/tryptophan 5-monooxygenase activation protein, theta polypeptide
chr12	22104329	22104510	182	183	22104434	30	7.24477	2.85875	4.21917	Adipose_R2_peak_	Distal Interger	12	21390329	21417581	27253	2	22630	uc007ndx.1	-686748	ENSMUSG000	Ywhaq	tyrosine 3-monooxygenase/tryptophan 5-monooxygenase activation protein, theta polypeptide
chr12	22132007	22132187	181	182	22132061	30	7.24477	2.85875	4.21917	Adipose_R2_peak_	Intron (uc011y	12	21390329	21417581	27253	2	22630	uc007ndx.1	-714426	ENSMUSG000	Ywhaq	tyrosine 3-monooxygenase/tryptophan 5-monooxygenase activation protein, theta polypeptide
chr12	22142380	22142646	267	268	22142515	50	34.50784	9.16663	30.72138	Adipose_R1_peak_	Intron (uc011y	12	21390329	21417581	27253	2	22630	uc007ndx.1	-724799	ENSMUSG000	Ywhaq	tyrosine 3-monooxygenase/tryptophan 5-monooxygenase activation protein, theta polypeptide
chr12	22142393	22142592	200	201	22142513	39	12.34187	3.68871	8.98501	Adipose_R2_peak_	Intron (uc011y	12	21390329	21417581	27253	2	22630	uc007ndx.1	-724812	ENSMUSG000	Ywhaq	tyrosine 3-monooxygenase/tryptophan 5-monooxygenase activation protein, theta polypeptide
chr12	22143601	22143808	208	209	22143722	30	15.39182	5.57188	12.01394	Adipose_R1_peak_	Intron (uc011y	12	21390329	21417581	27253	2	22630	uc007ndx.1	-726020	ENSMUSG000	Ywhaq	tyrosine 3-monooxygenase/tryptophan 5-monooxygenase activation protein, theta polypeptide
chr12	22157925	22158036	112	113	22157969	27	5.77405	2.5821	2.88921	Adipose_R2_peak_	Distal Interger	12	21390329	21417581	27253	2	22630	uc007ndx.1	-740344	ENSMUSG000	Ywhaq	tyrosine 3-monooxygenase/tryptophan 5-monooxygenase activation protein, theta polypeptide
chr12	22163515	22163644	130	131	22163521	26	5.31252	2.48988	2.47999	Adipose_R2_peak_	Distal Interger	12	21390329	21417581	27253	2	22630	uc007ndx.1	-745934	ENSMUSG000	Ywhaq	tyrosine 3-monooxygenase/tryptophan 5-monooxygenase activation protein, theta polypeptide
chr12	22177346	22177483	138	139	22177435	28	6.25023	2.67431	3.31626	Adipose_R2_peak_	Distal Interger	12	21390329	21417581	27253	2	22630	uc007ndx.1	-759765	ENSMUSG000	Ywhaq	tyrosine 3-monooxygenase/tryptophan 5-monooxygenase activation protein, theta polypeptide
chr12	22178266	22178375	110	111	22178333	31	7.7623	2.95097	4.69388	Adipose_R2_peak_	Distal Interger	12	21390329	21417581	27253	2	22630	uc007ndx.1	-760685	ENSMUSG000	Ywhaq	tyrosine 3-monooxygenase/tryptophan 5-monooxygenase activation protein, theta polypeptide
chr12	22186740	22186884	145	146	22186861	31	7.7623	2.95097	4.69388	Adipose_R2_peak_	Distal Interger	12	21390329	21417581	27253	2	22630	uc007ndx.1	-769159	ENSMUSG000	Ywhaq	tyrosine 3-monooxygenase/tryptophan 5-monooxygenase activation protein, theta polypeptide
chr12	22189776	22189894	119	120	22189802	26	5.31252	2.48988	2.47999	Adipose_R2_peak_	Distal Interger	12	21390329	21417581	27253	2	22630	uc007ndx.1	-772195	ENSMUSG000	Ywhaq	tyrosine 3-monooxygenase/tryptophan 5-monooxygenase activation protein, theta polypeptide
chr12	22193500	22194000	501	502	22193938	30	7.24477	2.85875	4.21917	Adipose_R2_peak_	Distal Interger	12	21390329	21417581	27253	2	22630	uc007ndx.1	-775919	ENSMUSG000	Ywhaq	tyrosine 3-monooxygenase/tryptophan 5-monooxygenase activation protein, theta polypeptide
chr12	22200984	22201081	98	99	22201057	28	6.25023	2.67431	3.31626	Adipose_R2_peak_	Distal Interger	12	21390329	21417581	27253	2	22630	uc007ndx.1	-783403	ENSMUSG000	Ywhaq	tyrosine 3-monooxygenase/tryptophan 5-monooxygenase activation protein, theta polypeptide
chr12	22202082	22202205	124	125	22202116	27	5.77405	2.5821	2.88921	Adipose_R2_peak_	Distal Interger	12	21390329	21417581	27253	2	22630	uc007ndx.1	-784501	ENSMUSG000	Ywhaq	tyrosine 3-monooxygenase/tryptophan 5-monooxygenase activation protein, theta polypeptide
chr12	22221001	22221170	170	171	22221066	21	8.43286	3.95423	5.34896	Adipose_R1_peak_	Distal Interger	12	22953997	23010243	56247	2	69732	uc007nef.2	789073	ENSMUSG000	2410018L13Ri	RIKEN cDNA 2410018L13 gene
chr12	22225747	22225947	201	202	22225849	26	12.14079	4.85292	8.87838	Adipose_R1_peak_	Distal Interger	12	22953997	23010243	56247	2	69732	uc007nef.2	784296	ENSMUSG000	2410018L13Ri	RIKEN cDNA 2410018L13 gene
chr12	22236620	22236819	200	201	22236715	30	7.24477	2.85875	4.21917	Adipose_R2_peak_	Distal Interger	12	22953997	23010243	56247	2	69732	uc007nef.2	773424	ENSMUSG000	2410018L13Ri	RIKEN cDNA 2410018L13 gene
chr12	22236857	22237009	153	154	22236879	16	5.20017	3.05554	2.38734	Adipose_R1_peak_	Distal Interger	12	22953997	23010243	56247	2	69732	uc007nef.2	773234	ENSMUSG000	2410018L13Ri	RIKEN cDNA 2410018L13 gene
chr12	22249747	22249921	175	176	22249804	31	7.7623	2.95097	4.69388	Adipose_R2_peak_	Distal Interger	12	22953997	23010243	56247	2	69732	uc007nef.2	760322	ENSMUSG000	2410018L13Ri	RIKEN cDNA 2410018L13 gene
chr12	22253138	22253373	236	237	22253245	31	16.23981	5.75161	12.83549	Adipose_R1_peak_	Distal Interger	12	22953997	23010243	56247	2	69732	uc007nef.2	756870	ENSMUSG000	2410018L13Ri	RIKEN cDNA 2410018L13 gene
chr12	22256440	22256570	131	132	22256480	29	6.74062	2.76653	3.75989	Adipose_R2_peak_	Distal Interger	12	22953997	23010243	56247	2	69732	uc007nef.2	753673	ENSMUSG000	2410018L13Ri	RIKEN cDNA 2410018L13 gene
chr12	22260875	22261177	303	304	22261003	24	9.37782	4.00313	6.25132	Adipose_R1_peak_	Distal Interger	12	22953997	23010243	56247	2	69732	uc007nef.2	749066	ENSMUSG000	2410018L13Ri	RIKEN cDNA 2410018L13 gene
chr12	22269134	22269245	112	113	22269169	26	5.31252	2.48988	2.47999	Adipose_R2_peak_	Distal Interger	12	22953997	23010243	56247	2	69732	uc007nef.2	740998	ENSMUSG000	2410018L13Ri	RIKEN cDNA 2410018L13 gene
chr12	22276426	22276601	176	177	22276587	27	5.77405	2.5821	2.88921	Adipose_R2_peak_	Distal Interger	12	22953997	23010243	56247	2	69732	uc007nef.2	733642	ENSMUSG000	2410018L13Ri	RIKEN cDNA 2410018L13 gene
chr12	22283222	22283347	126	127	22283281	38	11.72962	3.59649	8.4047	Adipose_R2_peak_	Distal Interger	12	22953997	23010243	56247	2	69732	uc007nef.2	726896	ENSMUSG000	2410018L13Ri	RIKEN cDNA 2410018L13 gene
chr12	22283250	22283476	227	228	22283296	25	6.84042	3.01561	3.89524	Adipose_R1_peak_	Distal Interger	12	22953997	23010243	56247	2	69732	uc007nef.2	726767	ENSMUSG000	2410018L13Ri	RIKEN cDNA 2410018L13 gene
chr12	22290085	22290255	171	172	22290143	29	6.74062	2.76653	3.75989	Adipose_R2_peak_	Distal Interger	12	22953997	23010243	56247	2	69732	uc007nef.2	719988	ENSMUSG000	2410018L13Ri	RIKEN cDNA 2410018L13 gene
chr12	22299341	22299551	211	212	22299343	27	5.77405	2.5821	2.88921	Adipose_R2_peak_	Distal Interger	12	22953997	23010243	56247	2	69732	uc007nef.2	710692	ENSMUSG000	2410018L13Ri	RIKEN cDNA 2410018L13 gene
chr12	22318950	22319217	268	269	22319074	24	10.60668	4.49345	7.40961	Adipose_R1_peak_	Distal Interger	12	22953997	23010243	56247	2	69732	uc007nef.2	691026	ENSMUSG000	2410018L13Ri	RIKEN cDNA 2410018L13 gene
chr12	22323051	22323310	260	261	22323155	28	13.73721	5.2124	10.41463	Adipose_R1_peak_	Distal Interger	12	22953997	23010243	56247	2	69732	uc007nef.2	686933	ENSMUSG000	2410018L13Ri	RIKEN cDNA 2410018L13 gene
chr12	22338213	22338310	98	99	22338256	29	6.74062	2.76653	3.75989	Adipose_R2_peak_	Distal Interger	12	22953997	23010243	56247	2	69732	uc007nef.2	671933	ENSMUSG000	2410018L13Ri	RIKEN cDNA 2410018L13 gene
chr12	22344135	22344321	187	188	22344197	29	6.74062	2.76653	3.75989	Adipose_R2_peak_	Distal Interger	12	22953997	23010243	56247	2	69732	uc007nef.2	665922	ENSMUSG000	2410018L13Ri	RIKEN cDNA 2410018L13 gene
chr12	22344614	22344730	117	118	22344675	29	6.74062	2.76653	3.75989	Adipose_R2_peak_	Distal Interger	12	22953997	23010243	56247	2	69732	uc007nef.2	665513	ENSMUSG000	2410018L13Ri	RIKEN cDNA 2410018L13 gene
chr12	22349404	22349603	200	201	22349467	29	6.74062	2.76653	3.75989	Adipose_R2_peak_	Distal Interger	12	22953997	23010243	56247	2	69732	uc007nef.2	660640	ENSMUSG000	2410018L13Ri	RIKEN cDNA 2410018L13 gene
chr12	22349678	22350080	403	404	22349755	27	10.77876	4.20743	7.58042	Adipose_R1_peak_	Distal Interger	12	22953997	23010243	56247	2	69732	uc007nef.2	660163	ENSMUSG000	2410018L13Ri	RIKEN cDNA 2410018L13 gene
chr12	22349707	22350221	515	516	22349849	27	5.77405	2.5821	2.88921	Adipose_R2_peak_	Distal Interger	12	22953997	23010243	56247	2	69732	uc007nef.2	660022	ENSMUSG000	2410018L13Ri	RIKEN cDNA 2410018L13 gene
chr12	22353941	22354099	159	160	22354024	21	8.43286	3.95423	5.34896	Adipose_R1_peak_	Distal Interger	12	22953997	23010243	56247	2	69732	uc007nef.2	656144	ENSMUSG000	2410018L13Ri	RIKEN cDNA 2410018L13 gene
chr12	22362493	22362590	98	99	22362523	25	4.86612	2.39766	2.09018	Adipose_R2_peak_	Distal Interger	12	22953997	23010243	56247	2	69732	uc007nef.2	647653	ENSMUSG000	2410018L13Ri	RIKEN cDNA 2410018L13 gene
chr12	22378209	22378330	122	123	22378266	28	6.25023	2.67431	3.31626	Adipose_R2_peak_	Distal Interger	12	22953997	23010243	56247	2	69732	uc007nef.2	631913	ENSMUSG000	2410018L13Ri	RIKEN cDNA 2410018L13 gene
chr12	22413940	22414040	101	102	22413972	27	5.77405	2.5821	2.88921	Adipose_R2_peak_	Distal Interger	12	22953997	23010243	56247	2	69732	uc007nef.2	596203	ENSMUSG000	2410018L13Ri	RIKEN cDNA 2410018L13 gene
chr12	22417927	22418104	178	179	22418020	29	6.74062	2.76653	3.75989	Adipose_R2_peak_	Distal Interger	12	22953997	23010243	56247	2	69732	uc007nef.2	592139	ENSMUSG000	2410018L13Ri	RIKEN cDNA 2410018L13 gene
chr12	22422787	22422910	124	125	22422793	25	4.86612	2.39766	2.09018	Adipose_R2_peak_	Distal Interger	12	22953997	23010243	56247	2	69732	uc007nef.2	587333	ENSMUSG000	2410018L13Ri	RIKEN cDNA 2410018L13 gene
chr12	22494795	22494943	149	150	22494875	30	7.24477	2.85875	4.21917	Adipose_R2_peak_	Distal Interger	12	22953997	23010243	56247	2	69732	uc007nef.2	515300	ENSMUSG000	2410018L13Ri	RIKEN cDNA 2410018L13 gene
chr12	22499875	22500066	192	193	22499902	27	5.77405	2.5821	2.88921	Adipose_R2_peak_	Distal Interger	12	22953997	23010243	56247	2	69732	uc007nef.2	510177	ENSMUSG000	2410018L13Ri	RIKEN cDNA 2410018L13 gene
chr12	22500822	22501012	191	192	22500919	32	8.29282	3.04319	5.18352	Adipose_R2_peak_	Distal Interger	12	22953997	23010243	56247	2	69732	uc007nef.2	509231	ENSMUSG000	2410018L13Ri	RIKEN cDNA 2410018L13 gene
chr12	22503368	22503726	359	360	22503464	37	11.12817	3.50427	7.83613	Adipose_R2_peak_	Distal Interger	12	22953997	23010243	56247	2	69732	uc007nef.2	506517	ENSMUSG000	2410018L13Ri	RIKEN cDNA 2410018L13 gene
chr12	22505068	22505410	343	344	22505207	42	14.2409	3.96536	10.79479	Adipose_R2_peak_	Distal Interger	12	22953997	23010243	56247	2	69732	uc007nef.2	504833	ENSMUSG000	2410018L13Ri	RIKEN cDNA 2410018L13 gene
chr12	22506509	22506732	224	225	22506604	36	10.5378	3.41206	7.27949	Adipose_R2_peak_	Distal Interger	12	22953997	23010243	56247	2	69732	uc007nef.2	503511	ENSMUSG000	2410018L13Ri	RIKEN cDNA 2410018L13 gene
chr12	22508803	22509036	234	235	22508960	38	11.51639	3.54233	8.21237	Adipose_R2_peak_	Distal Interger	12	22953997	23010243	56247	2	69732	uc007nef.2	501207	ENSMUSG000	2410018L13Ri	RIKEN cDNA 2410018L13 gene
chr12	22510513	22510739	227	228	22510637	40	12.96465	3.78093	9.57654	Adipose_R2_peak_	Distal Interger	12	22953997	23010243	56247	2	69732	uc007nef.2	499504	ENSMUSG000	2410018L13Ri	RIKEN cDNA 2410018L13 gene
chr12	22512102	22512433	332	333	22512196	29	6.74062	2.76653	3.75989	Adipose_R2_peak_	Distal Interger	12	22953997	23010243	56247	2	69732	uc007nef.2	497810	ENSMUSG000	2410018L13Ri	RIKEN cDNA 2410018L13 gene
chr12	22512595	22513048	454	455	22512641	33	5.64399	2.3328	2.79912	Adipose_R2_peak_	Distal Interger	12	22953997	23010243	56247	2	69732	uc007nef.2	497195	ENSMUSG000	2410018L13Ri	RIKEN cDNA 2410018L13 gene
chr12	22513435	22513576	142	143	22513497	27	5.77405	2.5821	2.88921	Adipose_R2_peak_	Distal Interger	12	22953997	23010243	56247	2	69732	uc007nef.2	496667	ENSMUSG000	2410018L13Ri	RIKEN cDNA 2410018L13 gene
chr12	22514394	22514537	144	145	22514439	26	5.31252	2.48988	2.47999	Adipose_R2_peak_	Distal Interger	12	22953997	23010243	56247	2	69732	uc007nef.2	495706	ENSMUSG000	2410018L13Ri	RIKEN cDNA 2410018L13 gene
chr12	22515229	22515337	109	110	22515328	27	5.75051	2.57529	2.88921	Adipose_R2_peak_	Distal Interger	12	22953997	23010243	56247	2	69732	uc007nef.2	494906	ENSMUSG000	2410018L13Ri	RIKEN cDNA 2410018L13 gene
chr12	22516289	22516536	248	249	22516438	44	14.29495	3.8477	10.84762	Adipose_R2_peak_	Distal Interger	12	22953997	23010243	56247	2	69732	uc007nef.2	493707	ENSMUSG000	2410018L13Ri	RIKEN cDNA 2410018L13 gene
chr12	22517376	22517549	174	175	22517440	19	5.65977	3.00531	2.83863	Adipose_R1_peak_	Distal Interger	12	22953997	23010243	56247	2	69732	uc007nef.2	492694	ENSMUSG000	2410018L13Ri	RIKEN cDNA 2410018L13 gene
chr12	22517386	22517651	266	267	22517510	36	10.5378	3.41206	7.27949	Adipose_R2_peak_	Distal Interger	12	22953997	23010243	56247	2	69732	uc007nef.2	492592	ENSMUSG000	2410018L13Ri	RIKEN cDNA 2410018L13 gene
chr12	22519626	22519740	115	116	22519671	25	4.86612	2.39766	2.09018	Adipose_R2_peak_	Distal Interger	12	22953997	23010243	56247	2	69732	uc007nef.2	490503	ENSMUSG000	2410018L13Ri	RIKEN cDNA 2410018L13 gene
chr12	22520457	22520593	137	138	22520531	30	7.24477	2.85875	4.21917	Adipose_R2_peak_	Distal Interger	12	22953997	23010243	56247	2	69732	uc007nef.2	489650	ENSMUSG000	2410018L13Ri	RIKEN cDNA 2410018L13 gene
chr12	22520851	22521189	339	340	22521055	39	10.02343	3.1269	6.79817	Adipose_R2_peak_	Distal Interger	12	22953997	23010243	56247	2	69732	uc007nef.2	489054	ENSMUSG000	2410018L13Ri	RIKEN cDNA 2410018L13 gene
chr12	22521363	22521497	135	136	22521415	26	5.31252	2.48988	2.47999	Adipose_R2_peak_	Distal Interger	12	22953997	23010243	56247	2	69732	uc007nef.2	488746	ENSMUSG000	2410018L13Ri	RIKEN cDNA 2410018L13 gene
chr12	22521965	22522325	361	362	22522081	34	9.39139	3.22762	6.20474	Adipose_R2_peak_	Distal Interger	12	22953997	23010243	56247	2	69732	uc007nef.2	487918	ENSMUSG000	2410018L13Ri	RIKEN cDNA 2410018L13 gene
chr12	22523110	22523516	407	408	22523186	30	7.24477	2.85875	4.21917	Adipose_R2_peak_	Distal Interger	12	22953997	23010243	56247	2	69732	uc007nef.2	486727	ENSMUSG000	2410018L13Ri	RIKEN cDNA 2410018L13 gene
chr12	22525060	22525936	877	878	22525464	33	7.70684	2.84055	4.66368	Adipose_R2_peak_	Distal Interger	12	22953997	23010243	56247	2	69732	uc007nef.2	484307	ENSMUSG000	2410018L13Ri	RIKEN cDNA 2410018L13 gene
chr12	22527268	22527444	177	178	22527352	33	8.83596	3.1354	5.68652	Adipose_R2_peak_	Distal Interger	12	22953997	23010243	56247	2	69732	uc007nef.2	482799	ENSMUSG000	2410018L13Ri	RIKEN cDNA 2410018L13 gene
chr12	22528785	22529182	398	399	22528954	35	7.35271	2.67105	4.32291	Adipose_R2_peak_	Distal Interger	12	22953997	23010243	56247	2	69732	uc007nef.2	481061	ENSMUSG000	2410018L13Ri	RIKEN cDNA 2410018L13 gene
chr12	22530115	22530338	224	225	22530258	32	8.29282	3.04319	5.18352	Adipose_R2_peak_	Distal Interger	12	22953997	23010243	56247	2	69732	uc007nef.2	479905	ENSMUSG000	2410018L13Ri	RIKEN cDNA 2410018L13 gene
chr12	22530194	22530327	134	135	22530272	21	4.92571	2.60112	2.18266	Adipose_R1_peak_	Distal Interger	12	22953997	23010243	56247	2	69732	uc007nef.2	479916	ENSMUSG000	2410018L13Ri	RIKEN cDNA 2410018L13 gene
chr12	22531548	22531651	104	105	22531631	30	7.24477	2.85875	4.21917	Adipose_R2_peak_	Distal Interger	12	22953997	23010243	56247	2	69732	uc007nef.2	478592	ENSMUSG000	2410018L13Ri	RIKEN cDNA 2410018L13 gene
chr12	22532264	22532652	389	390	22532367	35	9.95877	3.31984	6.73562	Adipose_R2_peak_	Distal Interger	12	22953997	23010243	56247	2	69732	uc007nef.2	477591	ENSMUSG000	2410018L13Ri	RIKEN cDNA 2410018L13 gene
chr12	22533045	22533466	422	423	22533164	39	12.34187	3.68871	8.98501	Adipose_R2_peak_	Distal Interger	12	22953997	23010243	56247	2	69732	uc007nef.2	476777	ENSMUSG000	2410018L13Ri	RIKEN cDNA 2410018L13 gene
chr12	22540546	22540868	323	324	22540683	53	21.92215	4.97976	18.21755	Adipose_R2_peak_	Distal Interger	12	22953997	23010243	56247	2	69732	uc007nef.2	469375	ENSMUSG000	2410018L13Ri	RIKEN cDNA 2410018L13 gene
chr12	22541214	22541323	110	111	22541262	30	7.24477	2.85875	4.21917	Adipose_R2_peak_	Distal Interger	12	22953997	23010243	56247	2	69732	uc007nef.2	468920	ENSMUSG000	2410018L13Ri	RIKEN cDNA 2410018L13 gene
chr12	22542460	22542579	120	121	22542464	28	6.25023	2.67431	3.31626	Adipose_R2_peak_	Distal Interger	12	22953997	23010243	56247	2	69732	uc007nef.2	467664	ENSMUSG000	2410018L13Ri	RIKEN cDNA 2410018L13 gene
chr12	22542999	22543192	194	195	22543078	39	12.34187	3.68871	8.98501	Adipose_R2_peak_	Distal Interger	12	22953997	23010243	56247	2	69732	uc007nef.2	467051	ENSMUSG000	2410018L13Ri	RIKEN cDNA 2410018L13 gene
chr12	22543605	22543772	168	169	22543645	27	5.77405	2.5821	2.88921	Adipose_R2_peak_	Distal Interger	12	22953997	23010243	56247	2	69732	uc007nef.2	466471	ENSMUSG000	2410018L13Ri	RIKEN cDNA 2410018L13 gene
chr12	22546338	22546706	369	370	22546603	36	10.5378	3.41206	7.27949	Adipose_R2_peak_	Distal Interger	12	22953997	23010243	56247	2	69732	uc007nef.2	463537	ENSMUSG000	2410018L13Ri	RIKEN cDNA 2410018L13 gene
chr12	22563142	22563261	120	121	22563185	25	4.86612	2.39766	2.09018	Adipose_R2_peak_	Distal Interger	12	22953997	23010243	56247	2	69732	uc007nef.2	446982	ENSMUSG000	2410018L13Ri	RIKEN cDNA 2410018L13 gene
chr12	22564068	22564176	109	110	22564148	27	5.77405	2.5821	2.88921	Adipose_R2_peak_	Distal Interger	12	22953997	23010243	56247	2	69732	uc007nef.2	446067	ENSMUSG000	2410018L13Ri	RIKEN cDNA 2410018L13 gene
chr12	22567526	22567718	193	194	22567637	29	6.74062	2.76653	3.75989	Adipose_R2_peak_	Distal Interger	12	22953997	23010243	56247	2	69732	uc007nef.2	442525	ENSMUSG000	2410018L13Ri	RIKEN cDNA 2410018L13 gene

chr12	22570074	22570215	142	143	22570128	25	4.86612	2.39766	2.09018	Adipose_R2_peak_4	Distal Intergen	12	22953997	23010243	56247	2	69732	uc007nef.2	440028	ENSMUSG000	2410018L13Ri	RIKEN cDNA 2410018L13 gene
chr12	22573075	22573183	109	110	22573088	25	4.86612	2.39766	2.09018	Adipose_R2_peak_4	Distal Intergen	12	22953997	23010243	56247	2	69732	uc007nef.2	437060	ENSMUSG000	2410018L13Ri	RIKEN cDNA 2410018L13 gene
chr12	22574603	22574757	155	156	22574684	33	8.83596	3.1354	5.68652	Adipose_R2_peak_4	Distal Intergen	12	22953997	23010243	56247	2	69732	uc007nef.2	435486	ENSMUSG000	2410018L13Ri	RIKEN cDNA 2410018L13 gene
chr12	22577702	22577837	136	137	22577726	16	5.20017	3.05554	2.38734	Adipose_R1_peak_3	Distal Intergen	12	22953997	23010243	56247	2	69732	uc007nef.2	432406	ENSMUSG000	2410018L13Ri	RIKEN cDNA 2410018L13 gene
chr12	22582615	22582761	147	148	22582731	27	5.77405	2.5821	2.88921	Adipose_R2_peak_4	Distal Intergen	12	22953997	23010243	56247	2	69732	uc007nef.2	427482	ENSMUSG000	2410018L13Ri	RIKEN cDNA 2410018L13 gene
chr12	22610481	22610635	155	156	22610547	29	6.74062	2.76653	3.75989	Adipose_R2_peak_4	Distal Intergen	12	22953997	23010243	56247	2	69732	uc007nef.2	399608	ENSMUSG000	2410018L13Ri	RIKEN cDNA 2410018L13 gene
chr12	22611927	22612199	273	274	22612059	29	6.74062	2.76653	3.75989	Adipose_R2_peak_4	Distal Intergen	12	22953997	23010243	56247	2	69732	uc007nef.2	398044	ENSMUSG000	2410018L13Ri	RIKEN cDNA 2410018L13 gene
chr12	22613778	22613904	127	128	22613787	25	4.86612	2.39766	2.09018	Adipose_R2_peak_4	Distal Intergen	12	22953997	23010243	56247	2	69732	uc007nef.2	396339	ENSMUSG000	2410018L13Ri	RIKEN cDNA 2410018L13 gene
chr12	22651533	22651670	138	139	22651581	27	5.77405	2.5821	2.88921	Adipose_R2_peak_4	Distal Intergen	12	22953997	23010243	56247	2	69732	uc007nef.2	358573	ENSMUSG000	2410018L13Ri	RIKEN cDNA 2410018L13 gene
chr12	22658741	22658890	150	151	22658749	25	4.86612	2.39766	2.09018	Adipose_R2_peak_4	Distal Intergen	12	22953997	23010243	56247	2	69732	uc007nef.2	351353	ENSMUSG000	2410018L13Ri	RIKEN cDNA 2410018L13 gene
chr12	22658990	22659164	175	176	22659078	19	7.07687	3.59476	4.08458	Adipose_R1_peak_3	Distal Intergen	12	22953997	23010243	56247	2	69732	uc007nef.2	351079	ENSMUSG000	2410018L13Ri	RIKEN cDNA 2410018L13 gene
chr12	22660957	22661070	114	115	22660995	29	6.74062	2.76653	3.75989	Adipose_R2_peak_4	Distal Intergen	12	22953997	23010243	56247	2	69732	uc007nef.2	349173	ENSMUSG000	2410018L13Ri	RIKEN cDNA 2410018L13 gene
chr12	22671138	22671250	113	114	22671183	27	5.77405	2.5821	2.88921	Adipose_R2_peak_4	Distal Intergen	12	22953997	23010243	56247	2	69732	uc007nef.2	338993	ENSMUSG000	2410018L13Ri	RIKEN cDNA 2410018L13 gene
chr12	22673671	22673849	179	180	22673747	25	4.86612	2.39766	2.09018	Adipose_R2_peak_4	Distal Intergen	12	22953997	23010243	56247	2	69732	uc007nef.2	336394	ENSMUSG000	2410018L13Ri	RIKEN cDNA 2410018L13 gene
chr12	22673969	22674070	102	103	22674050	26	5.31252	2.48988	2.47999	Adipose_R2_peak_4	Distal Intergen	12	22953997	23010243	56247	2	69732	uc007nef.2	336173	ENSMUSG000	2410018L13Ri	RIKEN cDNA 2410018L13 gene
chr12	22709114	22709290	177	178	22709216	31	7.7623	2.95097	4.69388	Adipose_R2_peak_4	Distal Intergen	12	22953997	23010243	56247	2	69732	uc007nef.2	300953	ENSMUSG000	2410018L13Ri	RIKEN cDNA 2410018L13 gene
chr12	22718148	22718292	145	146	22718245	28	6.25023	2.67431	3.31626	Adipose_R2_peak_4	Distal Intergen	12	22953997	23010243	56247	2	69732	uc007nef.2	291951	ENSMUSG000	2410018L13Ri	RIKEN cDNA 2410018L13 gene
chr12	22791770	22791871	102	103	22791795	25	4.86612	2.39766	2.09018	Adipose_R2_peak_4	Distal Intergen	12	22953997	23010243	56247	2	69732	uc007nef.2	218372	ENSMUSG000	2410018L13Ri	RIKEN cDNA 2410018L13 gene
chr12	22816401	22816621	221	222	22816514	25	11.36566	4.67319	8.13482	Adipose_R1_peak_3	Distal Intergen	12	22953997	23010243	56247	2	69732	uc007nef.2	193622	ENSMUSG000	2410018L13Ri	RIKEN cDNA 2410018L13 gene
chr12	22818574	22818832	259	260	22818693	50	34.50784	9.16663	30.72138	Adipose_R1_peak_3	Distal Intergen	12	22953997	23010243	56247	2	69732	uc007nef.2	191411	ENSMUSG000	2410018L13Ri	RIKEN cDNA 2410018L13 gene
chr12	22818623	22818805	183	184	22818741	33	8.83596	3.1354	5.68652	Adipose_R2_peak_4	Distal Intergen	12	22953997	23010243	56247	2	69732	uc007nef.2	191438	ENSMUSG000	2410018L13Ri	RIKEN cDNA 2410018L13 gene
chr12	22824973	22825111	139	140	22825022	31	7.7623	2.95097	4.69388	Adipose_R2_peak_4	Distal Intergen	12	22953997	23010243	56247	2	69732	uc007nef.2	185132	ENSMUSG000	2410018L13Ri	RIKEN cDNA 2410018L13 gene
chr12	22932283	22932427	145	146	22932393	16	5.20017	3.05554	2.38734	Adipose_R1_peak_3	Distal Intergen	12	22953997	23010243	56247	2	69732	uc007nef.2	77816	ENSMUSG000	2410018L13Ri	RIKEN cDNA 2410018L13 gene
chr12	23035604	23035798	195	196	23035702	38	11.72962	3.59649	8.4047	Adipose_R2_peak_4	Distal Intergen	12	22953997	23010243	56247	2	69732	uc007nef.2	-25361	ENSMUSG000	2410018L13Ri	RIKEN cDNA 2410018L13 gene
chr12	23089511	23089640	130	131	23089576	25	4.86612	2.39766	2.09018	Adipose_R2_peak_4	Distal Intergen	12	22953997	23010243	56247	2	69732	uc007nef.2	-79268	ENSMUSG000	2410018L13Ri	RIKEN cDNA 2410018L13 gene
chr12	23124774	23125123	350	351	23124971	61	46.56983	11.14375	42.62347	Adipose_R1_peak_3	Intron (uc007r	12	22953997	23010243	56247	2	69732	uc007nef.2	-114531	ENSMUSG000	2410018L13Ri	RIKEN cDNA 2410018L13 gene
chr12	23124875	23125084	210	211	23124894	27	5.77405	2.5821	2.88921	Adipose_R2_peak_4	Intron (uc007r	12	22953997	23010243	56247	2	69732	uc007nef.2	-114632	ENSMUSG000	2410018L13Ri	RIKEN cDNA 2410018L13 gene
chr12	23205709	23206022	314	315	23205888	51	35.56531	9.34637	31.76235	Adipose_R1_peak_3	Distal Intergen	12	22953997	23010243	56247	2	69732	uc007nef.2	-195466	ENSMUSG000	2410018L13Ri	RIKEN cDNA 2410018L13 gene
chr12	23205820	23205966	147	148	23205869	39	12.34187	3.68871	8.98501	Adipose_R2_peak_4	Distal Intergen	12	22953997	23010243	56247	2	69732	uc007nef.2	-195577	ENSMUSG000	2410018L13Ri	RIKEN cDNA 2410018L13 gene
chr12	23299393	23299661	269	270	23299553	31	16.23981	5.75161	12.83549	Adipose_R1_peak_3	Intron (uc011y	12	22953997	23010243	56247	2	69732	uc007nef.2	-289150	ENSMUSG000	2410018L13Ri	RIKEN cDNA 2410018L13 gene
chr12	23300789	23301088	300	301	23300946	70	57.07198	12.76139	53.01762	Adipose_R1_peak_3	Intron (uc011y	12	22953997	23010243	56247	2	69732	uc007nef.2	-290546	ENSMUSG000	2410018L13Ri	RIKEN cDNA 2410018L13 gene
chr12	23300873	23301019	147	148	23300954	39	12.34187	3.68871	8.98501	Adipose_R2_peak_4	Intron (uc011y	12	22953997	23010243	56247	2	69732	uc007nef.2	-290630	ENSMUSG000	2410018L13Ri	RIKEN cDNA 2410018L13 gene
chr12	23462659	23462955	297	298	23462828	53	37.70483	9.70585	33.87	Adipose_R1_peak_3	Distal Intergen	12	22953997	23010243	56247	2	69732	uc007nef.2	-452416	ENSMUSG000	2410018L13Ri	RIKEN cDNA 2410018L13 gene
chr12	23462738	23462944	207	208	23462828	46	16.90986	4.33423	13.35898	Adipose_R2_peak_4	Distal Intergen	12	22953997	23010243	56247	2	69732	uc007nef.2	-452495	ENSMUSG000	2410018L13Ri	RIKEN cDNA 2410018L13 gene
chr12	23464809	23465047	239	240	23464935	28	13.73721	5.2124	10.41463	Adipose_R1_peak_3	Distal Intergen	12	22953997	23010243	56247	2	69732	uc007nef.2	-454566	ENSMUSG000	2410018L13Ri	RIKEN cDNA 2410018L13 gene
chr12	23464854	23465002	149	150	23464921	33	8.83596	3.1354	5.68652	Adipose_R2_peak_4	Distal Intergen	12	22953997	23010243	56247	2	69732	uc007nef.2	-454611	ENSMUSG000	2410018L13Ri	RIKEN cDNA 2410018L13 gene
chr12	23478051	23478194	144	145	23478111	19	7.07687	3.59476	4.08458	Adipose_R1_peak_3	Distal Intergen	12	22953997	23010243	56247	2	69732	uc007nef.2	-467808	ENSMUSG000	2410018L13Ri	RIKEN cDNA 2410018L13 gene
chr12	23685091	23685237	147	148	23685135	16	5.20017	3.05554	2.38734	Adipose_R1_peak_3	Distal Intergen	12	24043202	24097269	54068	2	66808	uc007neh.1	412032	ENSMUSG000	9030624G23R	RIKEN cDNA 9030624G23 gene
chr12	23704348	23704530	183	184	23704418	37	11.12817	3.50427	7.83613	Adipose_R2_peak_4	Distal Intergen	12	24043202	24097269	54068	2	66808	uc007neh.1	392739	ENSMUSG000	9030624G23R	RIKEN cDNA 9030624G23 gene
chr12	23704349	23704490	142	143	23704431	20	7.74501	3.7745	4.70467	Adipose_R1_peak_3	Distal Intergen	12	24043202	24097269	54068	2	66808	uc007neh.1	392779	ENSMUSG000	9030624G23R	RIKEN cDNA 9030624G23 gene
chr12	23905738	23905851	114	115	23905803	29	6.74062	2.76653	3.75989	Adipose_R2_peak_4	Distal Intergen	12	24043202	24097269	54068	2	66808	uc007neh.1	191418	ENSMUSG000	9030624G23R	RIKEN cDNA 9030624G23 gene
chr12	24000291	24000477	187	188	24000368	27	12.93148	5.03266	9.63827	Adipose_R1_peak_3	Distal Intergen	12	24043202	24097269	54068	2	66808	uc007neh.1	96792	ENSMUSG000	9030624G23R	RIKEN cDNA 9030624G23 gene
chr12	24285897	24286091	195	196	24286023	24	10.60668	4.49345	7.40961	Adipose_R1_peak_3	Distal Intergen	12	24043202	24097269	54068	2	66808	uc007neh.1	-188628	ENSMUSG000	9030624G23R	RIKEN cDNA 9030624G23 gene
chr12	24291130	24291246	117	118	24291219	28	6.25023	2.67431	3.31626	Adipose_R2_peak_4	Distal Intergen	12	24043202	24097269	54068	2	66808	uc007neh.1	-193861	ENSMUSG000	9030624G23R	RIKEN cDNA 9030624G23 gene
chr12	25075595	25075746	152	153	25075662	21	8.43286	3.95423	5.34896	Adipose_R1_peak_3	Distal Intergen	12	25093799	25096092	2294	2	15902	uc007nfe.2	20346	ENSMUSG000	Id2	inhibitor of DNA binding 2
chr12	25097215	25097384	170	171	25097297	18	6.42934	3.41502	3.48999	Adipose_R1_peak_3	Promoter (1-2	12	25093799	25096092	2294	2	15902	uc007nfe.2	-1123	ENSMUSG000	Id2	inhibitor of DNA binding 2
chr12	25098013	25098145	133	134	25098019	25	4.86612	2.39766	2.09018	Adipose_R2_peak_4	Promoter (1-2	12	25093799	25096092	2294	2	15902	uc007nfe.2	-1921	ENSMUSG000	Id2	inhibitor of DNA binding 2
chr12	25098399	25098535	137	138	25098431	21	8.43286	3.95423	5.34896	Adipose_R1_peak_3	Promoter (2-3	12	25093799	25096092	2294	2	15902	uc007nfe.2	-2307	ENSMUSG000	Id2	inhibitor of DNA binding 2
chr12	25142025	25142170	146	147	25142099	18	6.42934	3.41502	3.48999	Adipose_R1_peak_3	Distal Intergen	12	25129174	25132274	3101	1	432641	uc029rrm.1	12851	NA	Gm17746	predicted gene, 17746
chr12	25243089	25243259	171	172	25243151	22	9.13959	4.13397	6.01526	Adipose_R1_peak_3	Distal Intergen	12	25129174	25132274	3101	1	432641	uc029rrm.1	113915	NA	Gm17746	predicted gene, 17746
chr12	25252326	25252474	149	150	25252449	20	7.74501	3.7745	4.70467	Adipose_R1_peak_3	Distal Intergen	12	25129174	25132274	3101	1	432641	uc029rrm.1	123152	NA	Gm17746	predicted gene, 17746
chr12	25308331	25308461	131	132	25308337	25	4.86612	2.39766	2.09018	Adipose_R2_peak_4	Distal Intergen	12	25129174	25132274	3101	1	432641	uc029rrm.1	179157	NA	Gm17746	predicted gene, 17746
chr12	25547784	25547997	214	215	25547884	35	19.76029	6.47056	16.26028	Adipose_R1_peak_3	Distal Intergen	12	25129174	25132274	3101	1	432641	uc029rrm.1	418610	NA	Gm17746	predicted gene, 17746
chr12	25547822	25547946	125	126	25547894	28	6.25023	2.67431	3.31626	Adipose_R2_peak_4	Distal Intergen	12	25129174	25132274	3101	1	432641	uc029rrm.1	418648	NA	Gm17746	predicted gene, 17746
chr12	25636267	25636442	176	177	25636365	24	10.60668	4.49345	7.40961	Adipose_R1_peak_3	Distal Intergen	12	25129174	25132274	3101	1	432641	uc029rrm.1	507093	NA	Gm17746	predicted gene, 17746
chr12	25726633	25726733	101	102	25726691	27	5.77405	2.5821	2.88921	Adipose_R2_peak_4	Distal Intergen	12	26091962	26210505	118544	2	629481	uc056ypw.1	483772	NA	Gm29687	predicted gene, 29687
chr12	25751358	25751635	278	279	25751487	56	40.97333	10.24506	37.09402	Adipose_R1_peak_3	Distal Intergen	12	26091962	26210505	118544	2	629481	uc056ypw.1	458870	NA	Gm29687	predicted gene, 29687
chr12	25751368	25751630	263	264	25751481	60	27.32259	5.62528	23.49644	Adipose_R2_peak_4	Distal Intergen	12	26091962	26210505	118544	2	629481	uc056ypw.1	458875	NA	Gm29687	predicted gene, 29687
chr12	25973247	25973398	152	153	25973310	18	6.42934	3.41502	3.48999	Adipose_R1_peak_3	Distal Intergen	12	26091962	26210505	118544	2	629481	uc056ypw.1	237107	NA	Gm29687	predicted gene, 29687
chr12	26041006	26041268	263	264	26041134	57	42.07807	10.4248	38.18469	Adipose_R1_peak_3	Distal Intergen	12	26091962	26210505	118544	2	629481	uc056ypw.1	169237	NA	Gm29687	predicted gene, 29687
chr12	26041049	26041225	177	178	26041115	38	11.72962	3.59649	8.4047	Adipose_R2_peak_4	Distal Intergen	12	26091962	26210505	118544	2	629481	uc056ypw.1	169280	NA	Gm29687	predicted gene, 29687
chr12	27403732	27403908	177	178	27403809	27	12.93148	5.03266	9.63827	Adipose_R1_peak_3	Distal Intergen	12	27334268	27342718	8451	2	20666	uc007nfn.2	-61014	ENSMUSG000	Sox11	SRY (sex determining region Y)-box 11
chr12	27405181	27405437	257	258	27405340	55	23.42763	5.16419	19.68558	Adipose_R2_peak_4	Distal Intergen	12	27334268	27342718	8451	2	20666	uc007nfn.2	-62463	ENSMUSG000	Sox11	SRY (sex determining region Y)-box 11
chr12	27405186	27405452	267	268	27405341	43	27.35048	7.90847	23.68561	Adipose_R1_peak_3	Distal Intergen	12	27334268	27342718	8451	2	20666	uc007nfn.2	-62468	ENSMUSG000	Sox11	SRY (sex determining region Y)-box 11
chr12	27438778	27438889	112	113	27438849	30	7.24477	2.85875	4.21917	Adipose_R2_peak_4	Distal Intergen	12	27334268	27342718	8451	2	20666	uc007nfn.2	-96060	ENSMUSG000	Sox11	SRY (sex determining region Y)-box 11
chr12	27478322	27478533	212	213	27478427	19	7.07687	3.59476	4.08458	Adipose_R1_peak_3	Distal Intergen	12	27334268	27342718	8451	2	20666	uc007nfn.2	-135604	ENSMUSG000	Sox11	SRY (sex determining region Y)-box 11
chr12	27559442	27559612	171	172	27559496	19	7.07687	3.59476	4.08458	Adipose_R1_peak_3	Distal Intergen	12	27334268	27342718	8451	2	20666	uc007nfn.2	-216724	ENSMUSG000	Sox11	SRY (sex determining region Y)-box 11
chr12	27559469	27559608	140	141	27559479	25	4.86612	2.39766	2.09018	Adipose_R2_peak_4	Distal Intergen	12	27334268	27342718	8451	2	20666	uc007nfn.2	-216751	ENSMUSG000	Sox11	SRY (sex determining region Y)-box 11
chr12	28151412	28151527	116	117	28151418	25	4.86612	2.39766	2.09018	Adipose_R2_peak_4	Distal Intergen	12	28437795	28548337	110543	2	68511	uc007nfo.1	396810	ENSMUSG000	Dcdc2c	doublecortin domain containing 2C
chr12	28291166	28291436	271	272	28291229	20	7.74501	3.7745	4.70467	Adipose_R1_peak_3	Distal Intergen	12	28437795	28548337	110543	2	68511	uc007nfo.1	256901	ENSMUSG000	Dcdc2c	doublecortin domain containing 2C
chr12	28294575	28294767	193	194	28294698	28	6.25023	2.67431	3.31626	Adipose_R2_peak_4	Distal Intergen	12	28437795	28548337	110543	2	68511	uc007nfo.1	253570	ENSMUSG000	Dcdc2c	doublecortin domain containing 2C
chr12	28390774	28390899	126	127	28390797	25	4.86612	2.39766	2.09018	Adipose_R2_peak_4	Distal Intergen	12	28437795	28548337	110543	2	68511	uc007nfo.1	157438	ENSMUSG000	Dcdc2c	doublecortin domain containing 2C
chr12	28552791	28553014	224	225	28552865	22	9.13959	4.13397	6.01526	Adipose_R1_peak_3	Downstream (	12	28437795	28548337	110543	2	68511	uc007nfo.1	-4454	ENSMUSG000	Dcdc2c	doublecortin domain containing 2C
chr12	29324685	29324841	157	158	29324795	32	8.29282	3.04319	5.18352	Adipose_R2_peak_4	Distal Intergen	12	29528384	29923209	394826	1	17933	uc007ngd.1	-203543	ENSMUSG000	Myt1l	myelin transcription factor 1-like
chr12	29386091	29386336	246	247	29386201	35	19.76029	6.47056	16.26028	Adipose_R1_peak_3	Distal Intergen	12	29528384	29923209	394826	1	17933	uc007ngd.1	-142048	ENSMUSG000	Myt1l	myelin transcription factor 1-like
chr12	29386854	29387039	186	187	29386923	23	9.86443	4.31371	6.70247	Adipose_R1_peak_3	Distal Intergen	12	29528384	29923209	394826	1	17933	uc007ngd.1	-141345	ENSMUSG000	Myt1l	myelin transcription factor 1-like
chr12	29399525	29399698	174	175	29399643	34	9.39139	3.22762	6.20474	Adipose_R2_peak_4	Distal Intergen	12	29528384	29923209	394826	1	17933	uc007ngd.1	-128686	ENSMUSG000	Myt1l	myelin transcription factor 1-like
chr12	29399526	29399777	252	253	29399651	36	20.67077	6.6503	17.14779	Adipose_R1_peak_3	Distal Intergen	12	29528384	29923209	394826	1	17933	uc007ngd.1	-128607	ENSMUSG000	Myt1l	myelin transcription factor 1-like
chr12	29819800	29819918	119	120	29819844	27	5.77405	2.5821	2.88921	Adipose_R2_peak_4	Intron (uc007r	12	29826869	29857799	30931	1	17933	uc007ngh.1	-6951	ENSMUSG000	Myt1l	myelin transcription factor 1-like
chr12	29936454	29936599	146	147	29936479	26	5.31252	2.48988	2.47999	Adipose_R2_peak_4	Promoter (1-2	12	29938036	29988994	50959	1	69675	uc007ngj.1	-1437	ENSMUSG000	Pxdn	peroxidasin
chr12	30057006	30057232	227	228	30057150	21	8.43286	3.95423	5.34896	Adipose_R1_peak_3	Intron (uc007r	12	29999964	30017658	17695	1	69675	uc007ngm.2	57042	ENSMUSG000	Pxdn	peroxidasin
chr12	30151925	30152100	176	177	30152041	24	10.60668	4.49345	7.40961	Adipose_R1_peak_3	Distal Intergen	12	30054661	30132624	77964	2	22018	uc007ngo.2	-19301	ENSMUSG000	Tpo	thyroid peroxidase
chr12	30187773	30188087	315	316	30187943	64	30.56565	5.99415	26.6807	Adipose_R2_peak_4	Intron (uc007r	12	30054661	30132624	77964	2	22018	uc007ngo.2	-55149	ENSMUSG000	Tpo	thyroid peroxidase
chr12	30187810	30188095	286	287	30187958	51	35.56531	9.34637	31.76235	Adipose_R1_peak_3	Intron (uc007r	12	30054661	30132624	77964	2	22018	uc007ngo.2	-55186	ENSMUSG000	Tpo	thyroid peroxidase
chr12	30188665	30188807	143	144	30188737	20	7.74501	3.7745	4.70467	Adipose_R1_peak_3	Intron (uc007r	12	30174557	30243351	68795	2	268534	uc007ngq.2	54544	ENSMUSG000	Sntg2	syntrophin, gamma 2
chr12	30380811	30380960	150	151	30380867	25	4.86612	2.39766	2.09018	Adipose_R2_peak_4	Distal Intergen	12	30174557	30373375	198819	2	268534	uc007ngr.2	-7436	ENSMUSG000	Sntg2	syntrophin, gamma 2
chr12	30639193	30639558	366	367	30639413	64	42.97498	9.3087	39.07775	Adipose_R1_peak_3	Distal Intergen	12	30584443	30591219	6777	1	211986	uc007ngu.2	54750	ENSMUSG000	Tmem18	transmembrane protein 18
chr12	30639211	30639539	329	330	30639388	50	19.72356	4.7031	16.08069	Adipose_R2_peak_4	Distal Intergen	12	30584443	30591219	6777	1	211986	uc007ngu.2	54768	ENSMUSG000	Tmem18	transmembrane protein 18
chr12	30709660	30709862	203	204	30709785	27	12.93148	5.03266	9.63827	Adipose_R1_peak_3	Distal Intergen	12	30584443	30591219	6777	1	211986	uc007ngu.2	125217	ENSMUSG000	Tmem18	transmembrane protein 18
chr12	30709688	30709855	168	169	30709733	25	4.86612	2.39766	2.09018	Adipose_R2_peak_4	Distal Intergen	12	30584443	30591219	6777	1	211986	uc007ngu.2	125245	ENSMUSG000	Tmem18	transmembrane protein 18
chr12	33228911	33229127	217	218	33229033	31	16.23981	5.75161	12.83549	Adipose_R1_peak_3	Intron (uc007r	12	33302515	33368277	65763	1	380753	uc007niq.2	-73388	ENSMUSG000	Atxn7l1	ataxin 7-like 1
chr12	33228913	33229153	241	242	33229032	60	27.32259	5.62528	23.49644	Adipose_R2_peak_4	Intron (uc007r	12	33302515	33368277	65763	1	380753	uc007niq.2	-73362	ENSMUSG000	Atxn7l1	ataxin 7-like 1
chr12	33506603	33506817	215	216	33506684	19	7.07687	3.59476	4.08458	Adipose_R1_peak_3	Distal Intergen	12	33429624	33439380	9757	1	28071	uc007niu.1	76979	ENSMUSG000	Twistnb	twist basic helix-loop-helix transcription factor 1 neighbor
chr12	33657658	33657792	135	136	33657732	20	7.74501	3.7745	4.70467	Adipose_R1_peak_3	Distal Intergen	12	33429624	33439380	9757	1	28071	uc007niu.1	228034	ENSMUSG000	Twistnb	twist basic helix-loop-helix transcription factor 1 neighbor

chr12	33657664	33657825	162	163	33657709	37	11.12817	3.50427	7.83613	Adipose_R2_peak_	Distal Intergen	12	33429624	33439380	9757	1	28071	uc007niu.1	228040	ENSMUSG000	Twistnb	twist basic helix-loop-helix transcription factor 1 neighbor
chr12	33767115	33767369	255	256	33767237	32	17.10103	5.93135	13.67137	Adipose_R1_peak_	Distal Intergen	12	33928425	33929309	885	1	114712	uc007niv.1	-161056	ENSMUSG000	Ferd3l	Fer3-like (Drosophila)
chr12	33767150	33767349	200	201	33767232	41	13.59774	3.87315	10.1803	Adipose_R2_peak_	Distal Intergen	12	33928425	33929309	885	1	114712	uc007niv.1	-161076	ENSMUSG000	Ferd3l	Fer3-like (Drosophila)
chr12	33769261	33769530	270	271	33769385	46	30.36364	8.44768	26.64488	Adipose_R1_peak_	Distal Intergen	12	33928425	33929309	885	1	114712	uc007niv.1	-158895	ENSMUSG000	Ferd3l	Fer3-like (Drosophila)
chr12	33769336	33769454	119	120	33769377	29	6.74062	2.76653	3.75989	Adipose_R2_peak_	Distal Intergen	12	33928425	33929309	885	1	114712	uc007niv.1	-158971	ENSMUSG000	Ferd3l	Fer3-like (Drosophila)
chr12	33805171	33805465	295	296	33805299	79	43.62032	7.37742	39.5398	Adipose_R2_peak_	Distal Intergen	12	33928425	33929309	885	1	114712	uc007niv.1	-122960	ENSMUSG000	Ferd3l	Fer3-like (Drosophila)
chr12	33805180	33805510	331	332	33805324	81	70.57077	14.73851	66.40193	Adipose_R1_peak_	Distal Intergen	12	33928425	33929309	885	1	114712	uc007niv.1	-122915	ENSMUSG000	Ferd3l	Fer3-like (Drosophila)
chr12	33880053	33880275	223	224	33880155	27	12.93148	5.03266	9.63827	Adipose_R1_peak_	Distal Intergen	12	33928425	33929309	885	1	114712	uc007niv.1	-48150	ENSMUSG000	Ferd3l	Fer3-like (Drosophila)
chr12	33880088	33880195	108	109	33880148	29	6.74062	2.76653	3.75989	Adipose_R2_peak_	Distal Intergen	12	33928425	33929309	885	1	114712	uc007niv.1	-48230	ENSMUSG000	Ferd3l	Fer3-like (Drosophila)
chr12	33964831	33965050	220	221	33964962	18	6.42934	3.41502	3.48999	Adipose_R1_peak_	Distal Intergen	12	33957671	33959831	2161	1	22160	uc007niw.1	7160	ENSMUSG000	Twist1	twist basic helix-loop-helix transcription factor 1
chr12	33995561	33995798	238	239	33995682	35	19.76029	6.47056	16.26028	Adipose_R1_peak_	Distal Intergen	12	33957671	33959831	2161	1	22160	uc007niw.1	37890	ENSMUSG000	Twist1	twist basic helix-loop-helix transcription factor 1
chr12	33995637	33995744	108	109	33995691	29	6.74062	2.76653	3.75989	Adipose_R2_peak_	Distal Intergen	12	33957671	33959831	2161	1	22160	uc007niw.1	37966	ENSMUSG000	Twist1	twist basic helix-loop-helix transcription factor 1
chr12	34025988	34026222	235	236	34026117	43	27.35048	7.90847	23.68561	Adipose_R1_peak_	Distal Intergen	12	33957671	33959831	2161	1	22160	uc007niw.1	68317	ENSMUSG000	Twist1	twist basic helix-loop-helix transcription factor 1
chr12	34026053	34026187	135	136	34026107	30	7.24477	2.85875	4.21917	Adipose_R2_peak_	Distal Intergen	12	33957671	33959831	2161	1	22160	uc007niw.1	68382	ENSMUSG000	Twist1	twist basic helix-loop-helix transcription factor 1
chr12	34028390	34028587	198	199	34028519	36	10.5378	3.41206	7.27949	Adipose_R2_peak_	Distal Intergen	12	33957671	33959831	2161	1	22160	uc007niw.1	70719	ENSMUSG000	Twist1	twist basic helix-loop-helix transcription factor 1
chr12	34028396	34028631	236	237	34028514	36	20.67077	6.6503	17.14779	Adipose_R1_peak_	Distal Intergen	12	33957671	33959831	2161	1	22160	uc007niw.1	70725	ENSMUSG000	Twist1	twist basic helix-loop-helix transcription factor 1
chr12	34131170	34131496	327	328	34131295	46	30.36364	8.44768	26.64488	Adipose_R1_peak_	Intron (uc007r	12	34047582	34248645	201064	2	79221	uc007nix.1	117149	ENSMUSG000	Hdac9	histone deacetylase 9
chr12	34131183	34131435	253	254	34131308	48	18.29934	4.51867	14.70076	Adipose_R2_peak_	Intron (uc007r	12	34047582	34248645	201064	2	79221	uc007nix.1	117210	ENSMUSG000	Hdac9	histone deacetylase 9
chr12	34653137	34653287	151	152	34653201	18	6.42934	3.41502	3.48999	Adipose_R1_peak_	Intron (uc007r	12	34371497	34528889	157393	2	79221	uc007njc.2	-124248	ENSMUSG000	Hdac9	histone deacetylase 9
chr12	35348918	35349152	235	236	35349044	48	32.41826	8.80716	28.66523	Adipose_R1_peak_	Distal Intergen	12	35497979	35534989	37011	2	11622	uc007nji.1	185837	ENSMUSG000	Ahr	aryl-hydrocarbon receptor
chr12	35348939	35349103	165	166	35349049	37	11.12817	3.50427	7.83613	Adipose_R2_peak_	Distal Intergen	12	35497979	35534989	37011	2	11622	uc007nji.1	185886	ENSMUSG000	Ahr	aryl-hydrocarbon receptor
chr12	35740047	35740240	194	195	35740161	25	11.36566	4.67319	8.13482	Adipose_R1_peak_	Distal Intergen	12	35672291	35695118	22828	2	71581	uc007njk.2	-44929	NA	9130015A21R	RIKEN cDNA 9130015A21 gene
chr12	35783198	35783363	166	167	35783302	29	6.74062	2.76653	3.75989	Adipose_R2_peak_	Distal Intergen	12	35672291	35695118	22828	2	71581	uc007njk.2	-88080	NA	9130015A21R	RIKEN cDNA 9130015A21 gene
chr12	35810636	35810897	262	263	35810754	39	23.47001	7.18952	19.88325	Adipose_R1_peak_	Distal Intergen	12	35925621	35949730	24110	1	403205	uc007njl.2	-114724	ENSMUSG000	Agr3	anterior gradient 3
chr12	35810672	35810835	164	165	35810761	32	8.29282	3.04319	5.18352	Adipose_R2_peak_	Distal Intergen	12	35925621	35949730	24110	1	403205	uc007njl.2	-114786	ENSMUSG000	Agr3	anterior gradient 3
chr12	37158831	37158947	117	118	37158896	31	7.7623	2.95097	4.69388	Adipose_R2_peak_	Intron (uc007r	12	37108546	37179528	70983	1	17286	uc007nkf.1	50285	ENSMUSG000	Meox2	mesenchyme homeobox 2
chr12	37158837	37159010	174	175	37158906	27	12.93148	5.03266	9.63827	Adipose_R1_peak_	Intron (uc007r	12	37108546	37179528	70983	1	17286	uc007nkf.1	50291	ENSMUSG000	Meox2	mesenchyme homeobox 2
chr12	37208961	37209178	218	219	37209065	40	24.42476	7.36925	20.81738	Adipose_R1_peak_	Distal Intergen	12	37241639	37404228	162590	1	319660	uc007nkg.1	-32461	ENSMUSG000	Agmo	alkylglycerol monooxygenase
chr12	37366233	37366436	204	205	37366312	28	13.73721	5.2124	10.41463	Adipose_R1_peak_	Intron (uc007r	12	37241639	37404228	162590	1	319660	uc007nkg.1	124594	ENSMUSG000	Agmo	alkylglycerol monooxygenase
chr12	37366235	37366435	201	202	37366320	42	14.2409	3.96536	10.79479	Adipose_R2_peak_	Intron (uc007r	12	37241639	37404228	162590	1	319660	uc007nkg.1	124596	ENSMUSG000	Agmo	alkylglycerol monooxygenase
chr12	37607232	37607406	175	176	37607295	19	7.07687	3.59476	4.08458	Adipose_R1_peak_	Distal Intergen	12	37880174	38136840	256667	1	217480	uc007nki.1	-272768	ENSMUSG000	Dgkb	diacylglycerol kinase, beta
chr12	37670024	37670230	207	208	37670113	33	17.97508	6.11109	14.52164	Adipose_R1_peak_	Distal Intergen	12	37880174	38136840	256667	1	217480	uc007nki.1	-209944	ENSMUSG000	Dgkb	diacylglycerol kinase, beta
chr12	37670031	37670169	139	140	37670115	34	9.39139	3.22762	6.20474	Adipose_R2_peak_	Distal Intergen	12	37880174	38136840	256667	1	217480	uc007nki.1	-210005	ENSMUSG000	Dgkb	diacylglycerol kinase, beta
chr12	37673137	37673293	157	158	37673226	20	7.74501	3.7745	4.70467	Adipose_R1_peak_	Distal Intergen	12	37880174	38136840	256667	1	217480	uc007nki.1	-206881	ENSMUSG000	Dgkb	diacylglycerol kinase, beta
chr12	37779289	37779433	145	146	37779351	24	10.60668	4.49345	7.40961	Adipose_R1_peak_	Distal Intergen	12	37880174	38136840	256667	1	217480	uc007nki.1	-100741	ENSMUSG000	Dgkb	diacylglycerol kinase, beta
chr12	37782691	37782856	166	167	37782839	23	9.86443	4.31371	6.70247	Adipose_R1_peak_	Distal Intergen	12	37880174	38136840	256667	1	217480	uc007nki.1	-97318	ENSMUSG000	Dgkb	diacylglycerol kinase, beta
chr12	37846935	37847242	308	309	37847073	52	36.63102	9.52611	32.81188	Adipose_R1_peak_	Distal Intergen	12	37880174	38136840	256667	1	217480	uc007nki.1	-32932	ENSMUSG000	Dgkb	diacylglycerol kinase, beta
chr12	37847021	37847134	114	115	37847085	30	7.24477	2.85875	4.21917	Adipose_R2_peak_	Distal Intergen	12	37880174	38136840	256667	1	217480	uc007nki.1	-33040	ENSMUSG000	Dgkb	diacylglycerol kinase, beta
chr12	38041490	38041693	204	205	38041586	26	12.14079	4.85292	8.87838	Adipose_R1_peak_	Intron (uc007r	12	38019180	38175077	155898	1	217480	uc007nkm.1	22310	ENSMUSG000	Dgkb	diacylglycerol kinase, beta
chr12	38043028	38043175	148	149	38043112	21	8.43286	3.95423	5.34896	Adipose_R1_peak_	Intron (uc007r	12	38019180	38175077	155898	1	217480	uc007nkm.1	23848	ENSMUSG000	Dgkb	diacylglycerol kinase, beta
chr12	38074694	38074891	198	199	38074783	22	9.13959	4.13397	6.01526	Adipose_R1_peak_	Intron (uc007r	12	38084167	38633410	549244	1	217480	uc007nkn.1	-9276	ENSMUSG000	Dgkb	diacylglycerol kinase, beta
chr12	38078967	38079167	201	202	38079063	29	14.55748	5.39214	11.20652	Adipose_R1_peak_	Intron (uc007r	12	38084167	38633410	549244	1	217480	uc007nkn.1	-5000	ENSMUSG000	Dgkb	diacylglycerol kinase, beta
chr12	38349448	38349656	209	210	38349550	26	12.14079	4.85292	8.87838	Adipose_R1_peak_	Intron (uc007r	12	38552809	38614521	61713	2	73867	uc007nko.2	264865	NA	4930428E07Ri	RIKEN cDNA 4930428E07 gene
chr12	38528471	38528725	255	256	38528603	40	24.42476	7.36925	20.81738	Adipose_R1_peak_	Intron (uc007r	12	38552809	38614521	61713	2	73867	uc007nko.2	85796	NA	4930428E07Ri	RIKEN cDNA 4930428E07 gene
chr12	38528536	38528664	129	130	38528607	31	7.7623	2.95097	4.69388	Adipose_R2_peak_	Intron (uc007r	12	38552809	38614521	61713	2	73867	uc007nko.2	85857	NA	4930428E07Ri	RIKEN cDNA 4930428E07 gene
chr12	38745968	38746216	249	250	38746058	25	11.36566	4.67319	8.13482	Adipose_R1_peak_	Distal Intergen	12	38780258	38868215	87958	1	14009	uc033gdb.1	-34042	ENSMUSG000	Etv1	ets variant 1
chr12	38913784	38914163	380	381	38914009	98	92.65971	17.79405	88.36157	Adipose_R1_peak_	Distal Intergen	12	38783711	38868215	84505	1	14009	uc033gdd.1	130073	ENSMUSG000	Etv1	ets variant 1
chr12	38913853	38914156	304	305	38914007	81	45.45771	7.56185	41.35481	Adipose_R2_peak_	Distal Intergen	12	38783711	38868215	84505	1	14009	uc033gdd.1	130142	ENSMUSG000	Etv1	ets variant 1
chr12	39360740	39360884	145	146	39360804	22	9.13959	4.13397	6.01526	Adipose_R1_peak_	Distal Intergen	12	38783711	38868215	84505	1	14009	uc033gdd.1	577029	ENSMUSG000	Etv1	ets variant 1
chr12	39363549	39363740	192	193	39363660	23	9.86443	4.31371	6.70247	Adipose_R1_peak_	Distal Intergen	12	38783711	38868215	84505	1	14009	uc033gdd.1	579838	ENSMUSG000	Etv1	ets variant 1
chr12	39363564	39363730	167	168	39363680	35	9.95877	3.31984	6.73562	Adipose_R2_peak_	Distal Intergen	12	38783711	38868215	84505	1	14009	uc033gdd.1	579853	ENSMUSG000	Etv1	ets variant 1
chr12	39364477	39364628	152	153	39364548	20	7.74501	3.7745	4.70467	Adipose_R1_peak_	Distal Intergen	12	38783711	38868215	84505	1	14009	uc033gdd.1	580766	ENSMUSG000	Etv1	ets variant 1
chr12	39419497	39419641	145	146	39419573	23	9.86443	4.31371	6.70247	Adipose_R1_peak_	Distal Intergen	12	40005447	40037376	31930	2	11861	uc007nkr.1	617735	ENSMUSG000	Arl4a	ADP-ribosylation factor-like 4A
chr12	39542511	39542803	293	294	39542653	61	46.56983	11.14375	42.62347	Adipose_R1_peak_	Distal Intergen	12	40005447	40037376	31930	2	11861	uc007nkr.1	494573	ENSMUSG000	Arl4a	ADP-ribosylation factor-like 4A
chr12	39542545	39542791	247	248	39542663	52	21.18119	4.88754	17.49655	Adipose_R2_peak_	Distal Intergen	12	40005447	40037376	31930	2	11861	uc007nkr.1	494585	ENSMUSG000	Arl4a	ADP-ribosylation factor-like 4A
chr12	39827008	39827148	141	142	39827077	21	8.43286	3.95423	5.34896	Adipose_R1_peak_	Distal Intergen	12	40005447	40037376	31930	2	11861	uc007nkr.1	210228	ENSMUSG000	Arl4a	ADP-ribosylation factor-like 4A
chr12	39900591	39900831	241	242	39900680	35	19.76029	6.47056	16.26028	Adipose_R1_peak_	Distal Intergen	12	40005447	40037376	31930	2	11861	uc007nkr.1	136545	ENSMUSG000	Arl4a	ADP-ribosylation factor-like 4A
chr12	39900621	39900762	142	143	39900669	34	9.39139	3.22762	6.20474	Adipose_R2_peak_	Distal Intergen	12	40005447	40037376	31930	2	11861	uc007nkr.1	136614	ENSMUSG000	Arl4a	ADP-ribosylation factor-like 4A
chr12	40952899	40953181	283	284	40953031	39	23.47001	7.18952	19.88325	Adipose_R1_peak_	Distal Intergen	12	41024090	41955588	931499	1	93757	uc029rsc.2	-70909	ENSMUSG000	Immp2l	IMP2 inner mitochondrial membrane peptidase-like (S. cerevisiae)
chr12	40952968	40953133	166	167	40953077	33	8.83596	3.1354	5.68652	Adipose_R2_peak_	Distal Intergen	12	41024090	41955588	931499	1	93757	uc029rsc.2	-70957	ENSMUSG000	Immp2l	IMP2 inner mitochondrial membrane peptidase-like (S. cerevisiae)
chr12	41137229	41137404	176	177	41137322	24	10.60668	4.49345	7.40961	Adipose_R1_peak_	Intron (uc029r	12	41024366	41110943	86578	1	93757	uc033gdg.1	112863	ENSMUSG000	Immp2l	IMP2 inner mitochondrial membrane peptidase-like (S. cerevisiae)
chr12	41507472	41507603	132	133	41507547	21	8.43286	3.95423	5.34896	Adipose_R1_peak_	Intron (uc029r	12	41451668	41486057	34390	2	16981	uc007nln.2	-21415	ENSMUSG000	Lrrn3	leucine rich repeat protein 3, neuronal
chr12	41507473	41507639	167	168	41507553	34	9.39139	3.22762	6.20474	Adipose_R2_peak_	Intron (uc029r	12	41451668	41486057	34390	2	16981	uc007nln.2	-21416	ENSMUSG000	Lrrn3	leucine rich repeat protein 3, neuronal
chr12	41777880	41778096	217	218	41777952	32	17.10103	5.93135	13.67137	Adipose_R1_peak_	Intron (uc029r	12	41451668	41486057	34390	2	16981	uc007nln.2	-291823	ENSMUSG000	Lrrn3	leucine rich repeat protein 3, neuronal
chr12	41777901	41778073	173	174	41777970	31	7.7623	2.95097	4.69388	Adipose_R2_peak_	Intron (uc029r	12	41451668	41486057	34390	2	16981	uc007nln.2	-291844	ENSMUSG000	Lrrn3	leucine rich repeat protein 3, neuronal
chr12	41813649	41813795	147	148	41813723	20	7.74501	3.7745	4.70467	Adipose_R1_peak_	Intron (uc029r	12	41451668	41486057	34390	2	16981	uc007nln.2	-327592	ENSMUSG000	Lrrn3	leucine rich repeat protein 3, neuronal
chr12	41814859	41815074	216	217	41814952	28	13.73721	5.2124	10.41463	Adipose_R1_peak_	Intron (uc029r	12	41451668	41486057	34390	2	16981	uc007nln.2	-328802	ENSMUSG000	Lrrn3	leucine rich repeat protein 3, neuronal
chr12	41814901	41815017	117	118	41814960	28	6.25023	2.67431	3.31626	Adipose_R2_peak_	Intron (uc029r	12	41451668	41486057	34390	2	16981	uc007nln.2	-328844	ENSMUSG000	Lrrn3	leucine rich repeat protein 3, neuronal
chr12	41868445	41868649	205	206	41868547	33	8.83596	3.1354	5.68652	Adipose_R2_peak_	Intron (uc029r	12	41451668	41486057	34390	2	16981	uc007nln.2	-382388	ENSMUSG000	Lrrn3	leucine rich repeat protein 3, neuronal
chr12	41973682	41973918	237	238	41973807	42	26.36523	7.72873	22.71906	Adipose_R1_peak_	Distal Intergen	12	41451668	41486057	34390	2	16981	uc007nln.2	-487625	ENSMUSG000	Lrrn3	leucine rich repeat protein 3, neuronal
chr12	41973703	41973910	208	209	41973801	48	18.29934	4.51867	14.70076	Adipose_R2_peak_	Distal Intergen	12	41451668	41486057	34390	2	16981	uc007nln.2	-487646	ENSMUSG000	Lrrn3	leucine rich repeat protein 3, neuronal
chr12	41974662	41974868	207	208	41974759	26	12.14079	4.85292	8.87838	Adipose_R1_peak_	Distal Intergen	12	41451668	41486057	34390	2	16981	uc007nln.2	-488605	ENSMUSG000	Lrrn3	leucine rich repeat protein 3, neuronal
chr12	41974679	41974830	152	153	41974751	34	9.39139	3.22762	6.20474	Adipose_R2_peak_	Distal Intergen	12	41451668	41486057	34390	2	16981	uc007nln.2	-488622	ENSMUSG000	Lrrn3	leucine rich repeat protein 3, neuronal
chr12	42225741	42225932	192	193	42225830	24	10.60668	4.49345	7.40961	Adipose_R1_peak_	Distal Intergen	12	41451668	41486057	34390	2	16981	uc007nln.2	-739684	ENSMUSG000	Lrrn3	leucine rich repeat protein 3, neuronal
chr12	42229834	42230023	190	191	42229970	19	7.07687	3.59476	4.08458	Adipose_R1_peak_	Distal Intergen	12	41451668	41486057	34390	2	16981	uc007nln.2	-743777	ENSMUSG000	Lrrn3	leucine rich repeat protein 3, neuronal
chr12	42242899	42243271	373	374	42243078	68	54.69342	12.40192	50.66183	Adipose_R1_peak_	Distal Intergen	12	41451668	41486057	34390	2	16981	uc007nln.2	-756842	ENSMUSG000	Lrrn3	leucine rich repeat protein 3, neuronal
chr12	42242973	42243184	212	213	42243087	53	21.92215	4.97976	18.21755	Adipose_R2_peak_	Distal Intergen	12	41451668	41486057	34390	2	16981	uc007nln.2	-756916	ENSMUSG000	Lrrn3	leucine rich repeat protein 3, neuronal
chr12	42246532	42246716	185	186	42246615	23	9.86443	4.31371	6.70247	Adipose_R1_peak_	Distal Intergen	12	41451668	41486057	34390	2	16981	uc007nln.2	-760475	ENSMUSG000	Lrrn3	leucine rich repeat protein 3, neuronal
chr12	42246569	42246679	111	112	42246613	27	5.77405	2.5821	2.88921	Adipose_R2_peak_	Distal Intergen	12	41451668	41486057	34390	2	16981	uc007nln.2	-760512	ENSMUSG000	Lrrn3	leucine rich repeat protein 3, neuronal
chr12	42247045	42247307	263	264	42247178	39	23.47001	7.18952	19.88325	Adipose_R1_peak_	Distal Intergen	12	41451668	41486057	34390	2	16981	uc007nln.2	-760988	ENSMUSG000	Lrrn3	leucine rich repeat protein 3, neuronal
chr12	42553514	42553749	236	237	42553631	53	31.39503	7.55601	27.65897	Adipose_R1_peak_	Distal Intergen	12	41451668	41486057	34390	2	16981	uc007nln.2	-1067457	ENSMUSG000	Lrrn3	leucine rich repeat protein 3, neuronal
chr12	42553523	42553723	201	202	42553647	41	13.59774	3.87315	10.1803	Adipose_R2_peak_	Distal Intergen	12	41451668	41486057	34390	2	16981	uc007nln.2	-1067466	ENSMUSG000	Lrrn3	leucine rich repeat protein 3, neuronal
chr12	42626070	42626257	188	189	42626188	24	10.60668	4.49345	7.40961	Adipose_R1_peak_	Distal Intergen	12	41451668	41486057	34390	2	16981	uc007nln.2	-1140013	ENSMUSG000	Lrrn3	leucine rich repeat protein 3, neuronal
chr12	42629172	42629503	332	333	42629361	82	71.83084	14.91825	67.65268	Adipose_R1_peak_	Distal Intergen	12	41451668	41486057	34390	2	16981	uc007nln.2	-1143115	ENSMUSG000	Lrrn3	leucine rich repeat protein 3, neuronal
chr12	42629201	42629495	295	296	42629364	104	67.98428	9.68286	63.65822	Adipose_R2_peak_	Distal Intergen	12	41451668	41486057	34390	2	16981	uc007nln.2	-1143144	ENSMUSG000	Lrrn3	leucine rich repeat protein 3, neuronal
chr12	42667094	42667336	243	244	42667200	32	17.10103	5.93135	13.67137	Adipose_R1_peak_	Distal Intergen	12	41451668	41486057	34390	2	16981	uc007nln.2	-1181037	ENSMUSG000	Lrrn3	leucine rich repeat protein 3, neuronal
chr12	42688313	42688444	132	133	42688375	20	7.74501	3.7745	4.70467	Adipose_R1_peak_	Distal Intergen	12	41451668	41486057	34390	2	16981	uc007nln.2	-1202256	ENSMUSG000	Lrrn3	leucine rich repeat protein 3, neuronal
chr12	42780424	42780582	159	160	42780496	20	7.74501	3.7745	4.70467	Adipose_R1_peak_	Distal Intergen	12	41451668	41486057	34390	2	16981	uc007nln.2	-1294367	ENSMUSG000	Lrrn3	leucine rich repeat protein 3, neuronal
chr12	42979962	42980112	151	152	42980024	20	7.74501	3.7745	4.70467	Adipose_R1_peak_	Distal Intergen	12	44205897	44210068	4172	2	27362	uc007nlo.1	1229956	ENSMUSG000	Dnajb9	DnaJ heat shock protein family (Hsp40) member B9
chr12	43036744	43037151	408	409	43037001	35	19.76029	6.47056	16.26028	Adipose_R1_peak_	Distal Intergen	12	44205897	44210068	4172	2	27362	uc007nlo.1	1172917	ENSMUSG000	Dnajb9	DnaJ heat shock protein family (Hsp40) member B9
chr12	43036906	43037088	183	184	43037010	32	8.29282	3.04319	5.18352	Adipose_R2_peak_	Distal Intergen	12	44205897	44210068	4172	2	27362	uc007nlo.1	1172980	ENSMUSG000	Dnajb9	DnaJ heat shock protein family (Hsp40) member B9
chr12	43343770	43343963	194	195	43343880	28	13.73721	5.2124	10.41463	Adipose_R1_peak_	Distal Intergen	12	44205897	44210068	4172	2	27362	uc007nlo.1	866105	ENSMUSG000	Dnajb9	DnaJ heat shock protein family (Hsp40) member B9
chr12	43345201	43345511	311	312	43345361	59	44.30963	10.78427	40.38949	Adipose_R1_peak_	Distal Intergen	12	44205897	44210068	4172	2	27362	uc007nlo.1	864557	ENSMUSG000	Dnajb9	DnaJ heat shock protein family (Hsp40) member B9
chr12	43345264	43345466	203	204	43345365	47	17.60017	4.42645	14.02531	Adipose_R2_peak_	Distal Intergen	12	44205897	44210068	4172	2	27362	uc007nlo.1	864602	ENSMUSG000	Dnajb9	DnaJ heat shock protein family (Hsp40) member B9

chr12	43348961	43349227	267	268	43349099	45	29.34988	8.26794	25.64927	Adipose_R1_peak_	Distal Intergen	12	44205897	44210068	4172	2	27362	uc007nlo.1	860841	ENSMUSG000	Dnajb9	DnaJ heat shock protein family (Hsp40) member B9
chr12	43349004	43349185	182	183	43349095	42	14.2409	3.96536	10.79479	Adipose_R2_peak_	Distal Intergen	12	44205897	44210068	4172	2	27362	uc007nlo.1	860883	ENSMUSG000	Dnajb9	DnaJ heat shock protein family (Hsp40) member B9
chr12	43477193	43477342	150	151	43477255	24	10.60668	4.49345	7.40961	Adipose_R1_peak_	Distal Intergen	12	44205897	44210068	4172	2	27362	uc007nlo.1	732726	ENSMUSG000	Dnajb9	DnaJ heat shock protein family (Hsp40) member B9
chr12	43477199	43477385	187	188	43477249	42	14.2409	3.96536	10.79479	Adipose_R2_peak_	Distal Intergen	12	44205897	44210068	4172	2	27362	uc007nlo.1	732683	ENSMUSG000	Dnajb9	DnaJ heat shock protein family (Hsp40) member B9
chr12	43500728	43500867	140	141	43500792	18	6.42934	3.41502	3.48999	Adipose_R1_peak_	Distal Intergen	12	44205897	44210068	4172	2	27362	uc007nlo.1	709201	ENSMUSG000	Dnajb9	DnaJ heat shock protein family (Hsp40) member B9
chr12	43660445	43660589	145	146	43660525	22	9.13959	4.13397	6.01526	Adipose_R1_peak_	Distal Intergen	12	44205897	44210068	4172	2	27362	uc007nlo.1	549479	ENSMUSG000	Dnajb9	DnaJ heat shock protein family (Hsp40) member B9
chr12	43756007	43756171	165	166	43756103	21	8.43286	3.95423	5.34896	Adipose_R1_peak_	Distal Intergen	12	44205897	44210068	4172	2	27362	uc007nlo.1	453897	ENSMUSG000	Dnajb9	DnaJ heat shock protein family (Hsp40) member B9
chr12	43930514	43930766	253	254	43930663	43	27.35048	7.90847	23.68561	Adipose_R1_peak_	Distal Intergen	12	44205897	44210068	4172	2	27362	uc007nlo.1	279302	ENSMUSG000	Dnajb9	DnaJ heat shock protein family (Hsp40) member B9
chr12	43933873	43934170	298	299	43934039	53	37.70483	9.70585	33.87	Adipose_R1_peak_	Distal Intergen	12	44205897	44210068	4172	2	27362	uc007nlo.1	275898	ENSMUSG000	Dnajb9	DnaJ heat shock protein family (Hsp40) member B9
chr12	43933962	43934207	246	247	43934032	70	35.62622	6.54746	31.65691	Adipose_R2_peak_	Distal Intergen	12	44205897	44210068	4172	2	27362	uc007nlo.1	275861	ENSMUSG000	Dnajb9	DnaJ heat shock protein family (Hsp40) member B9
chr12	43969628	43969984	357	358	43969773	45	29.34988	8.26794	25.64927	Adipose_R1_peak_	Distal Intergen	12	44205897	44210068	4172	2	27362	uc007nlo.1	240084	ENSMUSG000	Dnajb9	DnaJ heat shock protein family (Hsp40) member B9
chr12	43971390	43971650	261	262	43971504	52	36.63102	9.52611	32.81188	Adipose_R1_peak_	Distal Intergen	12	44205897	44210068	4172	2	27362	uc007nlo.1	238418	ENSMUSG000	Dnajb9	DnaJ heat shock protein family (Hsp40) member B9
chr12	43971397	43971642	246	247	43971516	52	21.18119	4.88754	17.49655	Adipose_R2_peak_	Distal Intergen	12	44205897	44210068	4172	2	27362	uc007nlo.1	238426	ENSMUSG000	Dnajb9	DnaJ heat shock protein family (Hsp40) member B9
chr12	43985755	43985988	234	235	43985894	75	63.12309	13.66008	59.01505	Adipose_R1_peak_	Distal Intergen	12	44205897	44210068	4172	2	27362	uc007nlo.1	224080	ENSMUSG000	Dnajb9	DnaJ heat shock protein family (Hsp40) member B9
chr12	43985762	43985987	226	227	43985914	82	46.38432	7.65407	42.27038	Adipose_R2_peak_	Distal Intergen	12	44205897	44210068	4172	2	27362	uc007nlo.1	224081	ENSMUSG000	Dnajb9	DnaJ heat shock protein family (Hsp40) member B9
chr12	43996011	43996147	137	138	43996043	25	4.86612	2.39766	2.09018	Adipose_R2_peak_	Distal Intergen	12	44205897	44210068	4172	2	27362	uc007nlo.1	213921	ENSMUSG000	Dnajb9	DnaJ heat shock protein family (Hsp40) member B9
chr12	44364098	44364309	212	213	44364209	37	21.59274	6.83004	18.04766	Adipose_R1_peak_	Intron (uc007r	12	44328885	44546824	217940	1	319504	uc007nls.2	35213	ENSMUSG000	Nrcam	neuronal cell adhesion molecule
chr12	44364112	44364267	156	157	44364215	36	10.5378	3.41206	7.27949	Adipose_R2_peak_	Intron (uc007r	12	44328885	44546824	217940	1	319504	uc007nls.2	35227	ENSMUSG000	Nrcam	neuronal cell adhesion molecule
chr12	44367867	44368042	176	177	44367973	24	10.60668	4.49345	7.40961	Adipose_R1_peak_	Intron (uc007r	12	44328885	44546824	217940	1	319504	uc007nls.2	38982	ENSMUSG000	Nrcam	neuronal cell adhesion molecule
chr12	44485331	44485495	165	166	44485400	22	9.13959	4.13397	6.01526	Adipose_R1_peak_	Intron (uc007r	12	44456625	44601846	145222	1	319504	uc007nlx.2	28706	ENSMUSG000	Nrcam	neuronal cell adhesion molecule
chr12	44490371	44490635	265	266	44490493	43	27.35048	7.90847	23.68561	Adipose_R1_peak_	Intron (uc007r	12	44456625	44601846	145222	1	319504	uc007nlx.2	33746	ENSMUSG000	Nrcam	neuronal cell adhesion molecule
chr12	44774275	44774384	110	111	44774337	27	5.77405	2.5821	2.88921	Adipose_R2_peak_	Distal Intergen	12	44584835	44601846	17012	1	319504	uc007nmb.2	189440	ENSMUSG000	Nrcam	neuronal cell adhesion molecule
chr12	45956592	45956827	236	237	45956690	33	17.97508	6.11109	14.52164	Adipose_R1_peak_	Distal Intergen	12	46694517	46818775	124259	2	664883	uc007nmd.2	861948	ENSMUSG000	Nova1	neuro-oncological ventral antigen 1
chr12	45956651	45956781	131	132	45956713	27	5.77405	2.5821	2.88921	Adipose_R2_peak_	Distal Intergen	12	46694517	46818775	124259	2	664883	uc007nmd.2	861994	ENSMUSG000	Nova1	neuro-oncological ventral antigen 1
chr12	46045696	46045934	239	240	46045818	32	17.10103	5.93135	13.67137	Adipose_R1_peak_	Distal Intergen	12	46694517	46818775	124259	2	664883	uc007nmd.2	772841	ENSMUSG000	Nova1	neuro-oncological ventral antigen 1
chr12	46155894	46156031	138	139	46155957	19	7.07687	3.59476	4.08458	Adipose_R1_peak_	Distal Intergen	12	46694517	46818775	124259	2	664883	uc007nmd.2	662744	ENSMUSG000	Nova1	neuro-oncological ventral antigen 1
chr12	46279477	46279705	229	230	46279589	30	15.39182	5.57188	12.01394	Adipose_R1_peak_	Distal Intergen	12	46694517	46818775	124259	2	664883	uc007nmd.2	539070	ENSMUSG000	Nova1	neuro-oncological ventral antigen 1
chr12	46407941	46408165	225	226	46408042	27	12.93148	5.03266	9.63827	Adipose_R1_peak_	Distal Intergen	12	46694517	46818775	124259	2	664883	uc007nmd.2	410610	ENSMUSG000	Nova1	neuro-oncological ventral antigen 1
chr12	46623506	46623617	112	113	46623590	27	5.77405	2.5821	2.88921	Adipose_R2_peak_	Distal Intergen	12	46694517	46818775	124259	2	664883	uc007nmd.2	195158	ENSMUSG000	Nova1	neuro-oncological ventral antigen 1
chr12	46707645	46708379	735	736	46707979	61	23.58833	4.74497	19.84412	Adipose_R2_peak_	Intron (uc007r	12	46694517	46818775	124259	2	664883	uc007nmd.2	110396	ENSMUSG000	Nova1	neuro-oncological ventral antigen 1
chr12	46707705	46708372	668	669	46707961	43	16.94051	4.58082	13.53265	Adipose_R1_peak_	Intron (uc007r	12	46694517	46818775	124259	2	664883	uc007nmd.2	110403	ENSMUSG000	Nova1	neuro-oncological ventral antigen 1
chr12	46744222	46744437	216	217	46744359	40	24.42476	7.36925	20.81738	Adipose_R1_peak_	Intron (uc007r	12	46694517	46818775	124259	2	664883	uc007nmd.2	74338	ENSMUSG000	Nova1	neuro-oncological ventral antigen 1
chr12	47073458	47073741	284	285	47073600	38	22.52591	7.00978	18.96	Adipose_R1_peak_	Distal Intergen	12	46694517	46818775	124259	2	664883	uc007nmd.2	-254683	ENSMUSG000	Nova1	neuro-oncological ventral antigen 1
chr12	47073512	47073737	226	227	47073618	38	11.72962	3.59649	8.4047	Adipose_R2_peak_	Distal Intergen	12	46694517	46818775	124259	2	664883	uc007nmd.2	-254737	ENSMUSG000	Nova1	neuro-oncological ventral antigen 1
chr12	47281299	47281564	266	267	47281433	50	34.50784	9.16663	30.72138	Adipose_R1_peak_	Distal Intergen	12	46694517	46818775	124259	2	664883	uc007nmd.2	-462524	ENSMUSG000	Nova1	neuro-oncological ventral antigen 1
chr12	47281344	47281549	206	207	47281424	42	14.2409	3.96536	10.79479	Adipose_R2_peak_	Distal Intergen	12	46694517	46818775	124259	2	664883	uc007nmd.2	-462569	ENSMUSG000	Nova1	neuro-oncological ventral antigen 1
chr12	47510571	47510826	256	257	47510689	34	18.86162	6.29083	15.38482	Adipose_R1_peak_	Distal Intergen	12	46694517	46818775	124259	2	664883	uc007nmd.2	-691796	ENSMUSG000	Nova1	neuro-oncological ventral antigen 1
chr12	47510590	47510820	231	232	47510688	52	21.18119	4.88754	17.49655	Adipose_R2_peak_	Distal Intergen	12	46694517	46818775	124259	2	664883	uc007nmd.2	-691815	ENSMUSG000	Nova1	neuro-oncological ventral antigen 1
chr12	47647812	47648005	194	195	47647925	23	9.86443	4.31371	6.70247	Adipose_R1_peak_	Distal Intergen	12	46694517	46818775	124259	2	664883	uc007nmd.2	-829037	ENSMUSG000	Nova1	neuro-oncological ventral antigen 1
chr12	47996367	47996633	267	268	47996500	61	46.56983	11.14375	42.62347	Adipose_R1_peak_	Distal Intergen	12	46694517	46818775	124259	2	664883	uc007nmd.2	-1177592	ENSMUSG000	Nova1	neuro-oncological ventral antigen 1
chr12	47996397	47996622	226	227	47996497	58	25.74274	5.44085	21.94821	Adipose_R2_peak_	Distal Intergen	12	46694517	46818775	124259	2	664883	uc007nmd.2	-1177622	ENSMUSG000	Nova1	neuro-oncological ventral antigen 1
chr12	47996825	47997052	228	229	47996932	31	16.23981	5.75161	12.83549	Adipose_R1_peak_	Distal Intergen	12	46694517	46818775	124259	2	664883	uc007nmd.2	-1178050	ENSMUSG000	Nova1	neuro-oncological ventral antigen 1
chr12	47996867	47996968	102	103	47996915	30	7.24477	2.85875	4.21917	Adipose_R2_peak_	Distal Intergen	12	46694517	46818775	124259	2	664883	uc007nmd.2	-1178092	ENSMUSG000	Nova1	neuro-oncological ventral antigen 1
chr12	48052988	48053210	223	224	48053089	34	18.86162	6.29083	15.38482	Adipose_R1_peak_	Distal Intergen	12	46694517	46818775	124259	2	664883	uc007nmd.2	-1234213	ENSMUSG000	Nova1	neuro-oncological ventral antigen 1
chr12	48134621	48134812	192	193	48134718	22	9.13959	4.13397	6.01526	Adipose_R1_peak_	Distal Intergen	12	49382883	49386867	3985	1	15228	uc011ylr.1	-1248071	ENSMUSG000	Foxg1	forkhead box G1
chr12	48247596	48247878	283	284	48247742	41	25.38991	7.54899	21.76305	Adipose_R1_peak_	Distal Intergen	12	49382883	49386867	3985	1	15228	uc011ylr.1	-1135005	ENSMUSG000	Foxg1	forkhead box G1
chr12	48247649	48247807	159	160	48247733	32	8.29282	3.04319	5.18352	Adipose_R2_peak_	Distal Intergen	12	49382883	49386867	3985	1	15228	uc011ylr.1	-1135076	ENSMUSG000	Foxg1	forkhead box G1
chr12	48250388	48250557	170	171	48250471	24	10.60668	4.49345	7.40961	Adipose_R1_peak_	Distal Intergen	12	49382883	49386867	3985	1	15228	uc011ylr.1	-1132326	ENSMUSG000	Foxg1	forkhead box G1
chr12	48341415	48341695	281	282	48341545	39	23.47001	7.18952	19.88325	Adipose_R1_peak_	Distal Intergen	12	49382883	49386867	3985	1	15228	uc011ylr.1	-1041188	ENSMUSG000	Foxg1	forkhead box G1
chr12	48341452	48341693	242	243	48341553	45	16.22858	4.24202	12.70278	Adipose_R2_peak_	Distal Intergen	12	49382883	49386867	3985	1	15228	uc011ylr.1	-1041190	ENSMUSG000	Foxg1	forkhead box G1
chr12	48514936	48515126	191	192	48515027	30	15.39182	5.57188	12.01394	Adipose_R1_peak_	Distal Intergen	12	49382883	49386867	3985	1	15228	uc011ylr.1	-867757	ENSMUSG000	Foxg1	forkhead box G1
chr12	48669766	48670031	266	267	48669885	53	36.46429	9.25395	32.65705	Adipose_R1_peak_	Distal Intergen	12	49382883	49386867	3985	1	15228	uc011ylr.1	-712852	ENSMUSG000	Foxg1	forkhead box G1
chr12	48669768	48669999	232	233	48669823	48	18.29934	4.51867	14.70076	Adipose_R2_peak_	Distal Intergen	12	49382883	49386867	3985	1	15228	uc011ylr.1	-712884	ENSMUSG000	Foxg1	forkhead box G1
chr12	48915655	48915896	242	243	48915770	59	44.30963	10.78427	40.38949	Adipose_R1_peak_	Distal Intergen	12	49382883	49386867	3985	1	15228	uc011ylr.1	-466987	ENSMUSG000	Foxg1	forkhead box G1
chr12	48915710	48915835	126	127	48915772	33	8.83596	3.1354	5.68652	Adipose_R2_peak_	Distal Intergen	12	49382883	49386867	3985	1	15228	uc011ylr.1	-467048	ENSMUSG000	Foxg1	forkhead box G1
chr12	49001251	49001534	284	285	49001396	46	30.36364	8.44768	26.64488	Adipose_R1_peak_	Distal Intergen	12	49382883	49386867	3985	1	15228	uc011ylr.1	-381349	ENSMUSG000	Foxg1	forkhead box G1
chr12	49001257	49001526	270	271	49001398	50	19.72356	4.7031	16.08069	Adipose_R2_peak_	Distal Intergen	12	49382883	49386867	3985	1	15228	uc011ylr.1	-381357	ENSMUSG000	Foxg1	forkhead box G1
chr12	49195828	49196033	206	207	49195908	23	9.86443	4.31371	6.70247	Adipose_R1_peak_	Distal Intergen	12	49382883	49386867	3985	1	15228	uc011ylr.1	-186850	ENSMUSG000	Foxg1	forkhead box G1
chr12	49215669	49215863	195	196	49215780	27	12.93148	5.03266	9.63827	Adipose_R1_peak_	Distal Intergen	12	49382883	49386867	3985	1	15228	uc011ylr.1	-167020	ENSMUSG000	Foxg1	forkhead box G1
chr12	49215681	49215839	159	160	49215779	34	9.39139	3.22762	6.20474	Adipose_R2_peak_	Distal Intergen	12	49382883	49386867	3985	1	15228	uc011ylr.1	-167044	ENSMUSG000	Foxg1	forkhead box G1
chr12	49218084	49218574	491	492	49218194	28	13.73721	5.2124	10.41463	Adipose_R1_peak_	Distal Intergen	12	49382883	49386867	3985	1	15228	uc011ylr.1	-164309	ENSMUSG000	Foxg1	forkhead box G1
chr12	49218380	49218536	157	158	49218457	34	9.39139	3.22762	6.20474	Adipose_R2_peak_	Distal Intergen	12	49382883	49386867	3985	1	15228	uc011ylr.1	-164347	ENSMUSG000	Foxg1	forkhead box G1
chr12	49250677	49250863	187	188	49250773	25	11.36566	4.67319	8.13482	Adipose_R1_peak_	Distal Intergen	12	49382883	49386867	3985	1	15228	uc011ylr.1	-132020	ENSMUSG000	Foxg1	forkhead box G1
chr12	49261120	49261397	278	279	49261253	63	48.85777	11.50323	44.88581	Adipose_R1_peak_	Distal Intergen	12	49382883	49386867	3985	1	15228	uc011ylr.1	-121486	ENSMUSG000	Foxg1	forkhead box G1
chr12	49261124	49261379	256	257	49261250	59	26.5291	5.53306	22.7188	Adipose_R2_peak_	Distal Intergen	12	49382883	49386867	3985	1	15228	uc011ylr.1	-121504	ENSMUSG000	Foxg1	forkhead box G1
chr12	49303229	49303489	261	262	49303349	76	64.35061	13.83982	60.23161	Adipose_R1_peak_	Distal Intergen	12	49382883	49386867	3985	1	15228	uc011ylr.1	-79394	ENSMUSG000	Foxg1	forkhead box G1
chr12	49303237	49303497	261	262	49303359	77	41.80442	7.19298	37.74731	Adipose_R2_peak_	Distal Intergen	12	49382883	49386867	3985	1	15228	uc011ylr.1	-79386	ENSMUSG000	Foxg1	forkhead box G1
chr12	49305750	49306034	285	286	49305891	41	25.38991	7.54899	21.76305	Adipose_R1_peak_	Distal Intergen	12	49382883	49386867	3985	1	15228	uc011ylr.1	-76849	ENSMUSG000	Foxg1	forkhead box G1
chr12	49305775	49305970	196	197	49305880	38	11.72962	3.59649	8.4047	Adipose_R2_peak_	Distal Intergen	12	49382883	49386867	3985	1	15228	uc011ylr.1	-76913	ENSMUSG000	Foxg1	forkhead box G1
chr12	49307419	49307768	350	351	49307542	39	12.34187	3.68871	8.98501	Adipose_R2_peak_	Distal Intergen	12	49382883	49386867	3985	1	15228	uc011ylr.1	-75115	ENSMUSG000	Foxg1	forkhead box G1
chr12	49307429	49307837	409	410	49307714	40	24.42476	7.36925	20.81738	Adipose_R1_peak_	Distal Intergen	12	49382883	49386867	3985	1	15228	uc011ylr.1	-75046	ENSMUSG000	Foxg1	forkhead box G1
chr12	49340989	49341169	181	182	49341103	22	9.13959	4.13397	6.01526	Adipose_R1_peak_	Distal Intergen	12	49382883	49386867	3985	1	15228	uc011ylr.1	-41714	ENSMUSG000	Foxg1	forkhead box G1
chr12	49343036	49343218	183	184	49343100	32	8.29282	3.04319	5.18352	Adipose_R2_peak_	Distal Intergen	12	49382883	49386867	3985	1	15228	uc011ylr.1	-39665	ENSMUSG000	Foxg1	forkhead box G1
chr12	49343063	49343218	156	157	49343126	19	7.07687	3.59476	4.08458	Adipose_R1_peak_	Distal Intergen	12	49382883	49386867	3985	1	15228	uc011ylr.1	-39665	ENSMUSG000	Foxg1	forkhead box G1
chr12	49353790	49354072	283	284	49353918	53	37.70483	9.70585	33.87	Adipose_R1_peak_	Distal Intergen	12	49382883	49386867	3985	1	15228	uc011ylr.1	-28811	ENSMUSG000	Foxg1	forkhead box G1
chr12	49353825	49354030	206	207	49353919	43	14.8939	4.05758	11.42017	Adipose_R2_peak_	Distal Intergen	12	49382883	49386867	3985	1	15228	uc011ylr.1	-28853	ENSMUSG000	Foxg1	forkhead box G1
chr12	49531683	49531881	199	200	49531760	27	12.93148	5.03266	9.63827	Adipose_R1_peak_	Distal Intergen	12	49476993	49480862	3870	2	69054	uc029rsk.1	-50821	ENSMUSG000	1810007C17Ri	RIKEN cDNA 1810007C17 gene
chr12	49531698	49531894	197	198	49531772	39	12.34187	3.68871	8.98501	Adipose_R2_peak_	Distal Intergen	12	49476993	49480862	3870	2	69054	uc029rsk.1	-50836	ENSMUSG000	1810007C17Ri	RIKEN cDNA 1810007C17 gene
chr12	49532476	49532731	256	257	49532592	34	18.86162	6.29083	15.38482	Adipose_R1_peak_	Distal Intergen	12	49476993	49480862	3870	2	69054	uc029rsk.1	-51614	ENSMUSG000	1810007C17Ri	RIKEN cDNA 1810007C17 gene
chr12	49532478	49532733	256	257	49532562	40	12.96465	3.78093	9.57654	Adipose_R2_peak_	Distal Intergen	12	49476993	49480862	3870	2	69054	uc029rsk.1	-51616	ENSMUSG000	1810007C17Ri	RIKEN cDNA 1810007C17 gene
chr12	49609973	49610135	163	164	49610057	23	9.86443	4.31371	6.70247	Adipose_R1_peak_	Distal Intergen	12	49476993	49480862	3870	2	69054	uc029rsk.1	-129111	ENSMUSG000	1810007C17Ri	RIKEN cDNA 1810007C17 gene
chr12	49611138	49611350	213	214	49611229	38	22.52591	7.00978	18.96	Adipose_R1_peak_	Distal Intergen	12	49476993	49480862	3870	2	69054	uc029rsk.1	-130276	ENSMUSG000	1810007C17Ri	RIKEN cDNA 1810007C17 gene
chr12	49611163	49611322	160	161	49611211	31	7.7623	2.95097	4.69388	Adipose_R2_peak_	Distal Intergen	12	49476993	49480862	3870	2	69054	uc029rsk.1	-130301	ENSMUSG000	1810007C17Ri	RIKEN cDNA 1810007C17 gene
chr12	49699379	49699549	171	172	49699454	23	9.86443	4.31371	6.70247	Adipose_R1_peak_	Distal Intergen	12	49476993	49480862	3870	2	69054	uc029rsk.1	-218517	ENSMUSG000	1810007C17Ri	RIKEN cDNA 1810007C17 gene
chr12	49757155	49757332	178	179	49757227	18	6.42934	3.41502	3.48999	Adipose_R1_peak_	Distal Intergen	12	49476993	49480862	3870	2	69054	uc029rsk.1	-276293	ENSMUSG000	1810007C17Ri	RIKEN cDNA 1810007C17 gene
chr12	50174571	50174855	285	286	50174710	64	49.39898	11.46094	45.42488	Adipose_R1_peak_	Distal Intergen	12	50341232	50649223	307992	2	18760	uc007nmj.1	474368	ENSMUSG000	Prkd1	protein kinase D1
chr12	50174592	50174853	262	263	50174720	67	33.06755	6.27081	29.1399	Adipose_R2_peak_	Distal Intergen	12	50341232	50649223	307992	2	18760	uc007nmj.1	474370	ENSMUSG000	Prkd1	protein kinase D1
chr12	50509570	50509759	190	191	50509634	21	8.43286	3.95423	5.34896	Adipose_R1_peak_	Intron (uc007r	12	50341232	50649223	307992	2	18760	uc007nmj.1	139464	ENSMUSG000	Prkd1	protein kinase D1
chr12	50689963	50690128	166	167	50690042	19	7.07687	3.59476	4.08458	Adipose_R1_peak_	Distal Intergen	12	50341232	50649223	307992	2	18760	uc007nmj.1	-40740	ENSMUSG000	Prkd1	protein kinase D1
chr12	50722542	50722783	242	243	50722606	21	8.43286	3.95423	5.34896	Adipose_R1_peak_	Distal Intergen	12	50341232	50649223	307992	2	18760	uc007nmj.1	-73319	ENSMUSG000	Prkd1	protein kinase D1
chr12	50787301	50787526	226	227	50787433	29	14.55748	5.39214	11.20652	Adipose_R1_peak_	Distal Intergen	12	50341232	50649223	307992	2	18760	uc007nmj.1	-138078	ENSMUSG000	Prkd1	protein kinase D1
chr12	50787304	50787510	207	208	50787431	41	13.59774	3.87315	10.1803	Adipose_R2_peak_	Distal Intergen	12	50341232	50649223	307992	2	18760	uc007nmj.1	-138081	ENSMUSG000	Prkd1	protein kinase D1
chr12	50863781	50864093	313	314	50863945	100	92.92603	17.30832	88.62647	Adipose_R1_peak_	Distal Intergen	12	51005156	51062543	57388	1	75494	uc056yqh.1	-141063	NA	1700008C04Ri	RIKEN cDNA 1700008C04 gene
chr12	50863803	50864070	268	269	50863939	86	50.14197	8.02294	45.98602	Adipose_R2_peak_	Distal Intergen	12	51005156	51062543	57388	1	75494	uc056yqh.1	-141086	NA	1700008C04Ri	RIKEN cDNA 1700008C04 gene

chr12	50881876	50882156	281	282	50882022	58	42.63807	10.40301	38.74227	Adipose_R1_peak_	Distal Interger	12	51005156	51062543	57388	1	75494	uc056yqh.1	-123000	NA	1700008C04Ri	RIKEN cDNA 1700008C04 gene
chr12	50881908	50882160	253	254	50882038	52	21.18119	4.88754	17.49655	Adipose_R2_peak_	Distal Interger	12	51005156	51062543	57388	1	75494	uc056yqh.1	-122996	NA	1700008C04Ri	RIKEN cDNA 1700008C04 gene
chr12	51486287	51486465	179	180	51486350	34	9.39139	3.22762	6.20474	Adipose_R2_peak_	Distal Interger	12	51593341	51605773	12433	1	12810	uc007nmr.2	-106876	ENSMUSG000	Coch	cochlin
chr12	51486813	51487010	198	199	51486934	35	9.95877	3.31984	6.73562	Adipose_R2_peak_	Distal Interger	12	51593341	51605773	12433	1	12810	uc007nmr.2	-106331	ENSMUSG000	Coch	cochlin
chr12	51487759	51487877	119	120	51487819	30	7.24477	2.85875	4.21917	Adipose_R2_peak_	Distal Interger	12	51593341	51605773	12433	1	12810	uc007nmr.2	-105464	ENSMUSG000	Coch	cochlin
chr12	51490514	51491082	569	570	51490864	56	18.6103	4.06379	15.00655	Adipose_R2_peak_	Distal Interger	12	51593341	51605773	12433	1	12810	uc007nmr.2	-102259	ENSMUSG000	Coch	cochlin
chr12	51490563	51490699	137	138	51490668	26	6.53666	2.85976	3.59571	Adipose_R1_peak_	Distal Interger	12	51593341	51605773	12433	1	12810	uc007nmr.2	-102642	ENSMUSG000	Coch	cochlin
chr12	52085342	52085551	210	211	52085440	31	16.23981	5.75161	12.83549	Adipose_R1_peak_	Distal Interger	12	52097746	52144780	47035	1	76826	uc007nng.2	-12195	ENSMUSG000	Nubpl	nucleotide binding protein-like
chr12	52085385	52085502	118	119	52085434	34	9.39139	3.22762	6.20474	Adipose_R2_peak_	Distal Interger	12	52097746	52144780	47035	1	76826	uc007nng.2	-12244	ENSMUSG000	Nubpl	nucleotide binding protein-like
chr12	52200800	52201005	206	207	52200897	28	13.73721	5.2124	10.41463	Adipose_R1_peak_	Intron (uc007r	12	52097746	52144780	47035	1	76826	uc007nng.2	103054	ENSMUSG000	Nubpl	nucleotide binding protein-like
chr12	52200847	52201002	156	157	52200902	33	8.83596	3.1354	5.68652	Adipose_R2_peak_	Intron (uc007r	12	52097746	52144780	47035	1	76826	uc007nng.2	103101	ENSMUSG000	Nubpl	nucleotide binding protein-like
chr12	54705521	54705719	199	200	54705587	31	7.7623	2.95097	4.69388	Adipose_R2_peak_	Distal Interger	12	54673467	54695865	22399	2	66266	uc007nnu.1	-9656	ENSMUSG000	Eapp	E2F-associated phosphoprotein
chr12	54706404	54706689	286	287	54706508	31	7.7623	2.95097	4.69388	Adipose_R2_peak_	Distal Interger	12	54673467	54695865	22399	2	66266	uc007nnu.1	-10539	ENSMUSG000	Eapp	E2F-associated phosphoprotein
chr12	54708317	54708531	215	216	54708362	29	6.74062	2.76653	3.75989	Adipose_R2_peak_	Distal Interger	12	54673467	54695865	22399	2	66266	uc007nnu.1	-12452	ENSMUSG000	Eapp	E2F-associated phosphoprotein
chr12	54708840	54708972	133	134	54708867	26	5.31252	2.48988	2.47999	Adipose_R2_peak_	Distal Interger	12	54673467	54695865	22399	2	66266	uc007nnu.1	-12975	ENSMUSG000	Eapp	E2F-associated phosphoprotein
chr12	54709725	54709846	122	123	54709801	30	7.24477	2.85875	4.21917	Adipose_R2_peak_	Distal Interger	12	54673467	54695865	22399	2	66266	uc007nnu.1	-13860	ENSMUSG000	Eapp	E2F-associated phosphoprotein
chr12	54710903	54711020	118	119	54710982	26	5.31252	2.48988	2.47999	Adipose_R2_peak_	Distal Interger	12	54673467	54695865	22399	2	66266	uc007nnu.1	-15038	ENSMUSG000	Eapp	E2F-associated phosphoprotein
chr12	54711840	54711998	159	160	54711924	33	8.83596	3.1354	5.68652	Adipose_R2_peak_	Distal Interger	12	54673467	54695865	22399	2	66266	uc007nnu.1	-15975	ENSMUSG000	Eapp	E2F-associated phosphoprotein
chr12	54712409	54712668	260	261	54712547	28	6.25023	2.67431	3.31626	Adipose_R2_peak_	Distal Interger	12	54673467	54695865	22399	2	66266	uc007nnu.1	-16544	ENSMUSG000	Eapp	E2F-associated phosphoprotein
chr12	54713598	54714099	502	503	54713726	32	8.29282	3.04319	5.18352	Adipose_R2_peak_	Distal Interger	12	54673467	54695865	22399	2	66266	uc007nnu.1	-17733	ENSMUSG000	Eapp	E2F-associated phosphoprotein
chr12	54716242	54716570	329	330	54716379	26	5.31252	2.48988	2.47999	Adipose_R2_peak_	Distal Interger	12	54673467	54695865	22399	2	66266	uc007nnu.1	-20377	ENSMUSG000	Eapp	E2F-associated phosphoprotein
chr12	54716787	54717091	305	306	54716875	27	5.77405	2.5821	2.88921	Adipose_R2_peak_	Distal Interger	12	54673467	54695865	22399	2	66266	uc007nnu.1	-20922	ENSMUSG000	Eapp	E2F-associated phosphoprotein
chr12	54717859	54718166	308	309	54718036	20	7.20926	3.54892	4.21524	Adipose_R1_peak_	Distal Interger	12	54673467	54695865	22399	2	66266	uc007nnu.1	-21994	ENSMUSG000	Eapp	E2F-associated phosphoprotein
chr12	54717886	54718242	357	358	54718196	32	8.29282	3.04319	5.18352	Adipose_R2_peak_	Distal Interger	12	54673467	54695865	22399	2	66266	uc007nnu.1	-22021	ENSMUSG000	Eapp	E2F-associated phosphoprotein
chr12	54718714	54719094	381	382	54718894	35	9.95877	3.31984	6.73562	Adipose_R2_peak_	Distal Interger	12	54673467	54695865	22399	2	66266	uc007nnu.1	-22849	ENSMUSG000	Eapp	E2F-associated phosphoprotein
chr12	54719318	54719441	124	125	54719363	26	5.31252	2.48988	2.47999	Adipose_R2_peak_	Distal Interger	12	54673467	54695865	22399	2	66266	uc007nnu.1	-23453	ENSMUSG000	Eapp	E2F-associated phosphoprotein
chr12	54722225	54722320	96	97	54722263	29	6.74062	2.76653	3.75989	Adipose_R2_peak_	Distal Interger	12	54673467	54695865	22399	2	66266	uc007nnu.1	-26360	ENSMUSG000	Eapp	E2F-associated phosphoprotein
chr12	54847462	54847629	168	169	54847522	16	5.20017	3.05554	2.38734	Adipose_R1_peak_	Distal Interger	12	54858819	54862877	4059	2	12632	uc007nnx.1	15248	ENSMUSG000	Cfl2	cofilin 2, muscle
chr12	54892678	54892810	133	134	54892745	30	7.24477	2.85875	4.21917	Adipose_R2_peak_	Downstream (	12	54858819	54862877	4059	2	12632	uc007nnx.1	-29801	ENSMUSG000	Cfl2	cofilin 2, muscle
chr12	55616992	55617135	144	145	55617063	22	9.13959	4.13397	6.01526	Adipose_R1_peak_	Intron (uc007r	12	55598917	55602017	3101	1	56856	uc007not.1	18075	ENSMUSG000	Insm2	insulinoma-associated 2
chr12	55617024	55617174	151	152	55617100	27	5.77405	2.5821	2.88921	Adipose_R2_peak_	Intron (uc007r	12	55598917	55602017	3101	1	56856	uc007not.1	18107	ENSMUSG000	Insm2	insulinoma-associated 2
chr12	57718339	57718553	215	216	57718507	57	24.96362	5.34863	21.18596	Adipose_R2_peak_	Intron (uc057t	12	57661458	57662911	1454	2	217601	uc056yqq.1	-55428	NA	BC042761	cDNA sequence BC042761
chr12	57718359	57718571	213	214	57718486	39	23.47001	7.18952	19.88325	Adipose_R1_peak_	Intron (uc057t	12	57661458	57662911	1454	2	217601	uc056yqq.1	-55448	NA	BC042761	cDNA sequence BC042761
chr12	57882255	57882492	238	239	57882376	35	19.76029	6.47056	16.26028	Adipose_R1_peak_	Distal Interger	12	57661458	57662911	1454	2	217601	uc056yqq.1	-219344	NA	BC042761	cDNA sequence BC042761
chr12	57882343	57882491	149	150	57882392	28	6.25023	2.67431	3.31626	Adipose_R2_peak_	Distal Interger	12	57661458	57662911	1454	2	217601	uc056yqq.1	-219432	NA	BC042761	cDNA sequence BC042761
chr12	57885101	57885269	169	170	57885185	21	8.43286	3.95423	5.34896	Adipose_R1_peak_	Distal Interger	12	57661458	57662911	1454	2	217601	uc056yqq.1	-222190	NA	BC042761	cDNA sequence BC042761
chr12	57911221	57911489	269	270	57911352	47	31.38649	8.62742	27.65048	Adipose_R1_peak_	Distal Interger	12	57661458	57662911	1454	2	217601	uc056yqq.1	-248310	NA	BC042761	cDNA sequence BC042761
chr12	57911288	57911401	114	115	57911345	30	7.24477	2.85875	4.21917	Adipose_R2_peak_	Distal Interger	12	57661458	57662911	1454	2	217601	uc056yqq.1	-248377	NA	BC042761	cDNA sequence BC042761
chr12	57913212	57913489	278	279	57913343	47	31.38649	8.62742	27.65048	Adipose_R1_peak_	Distal Interger	12	57661458	57662911	1454	2	217601	uc056yqq.1	-250301	NA	BC042761	cDNA sequence BC042761
chr12	57941226	57941523	298	299	57941400	77	41.80442	7.19298	37.74731	Adipose_R2_peak_	Distal Interger	12	58211804	58216036	4233	1	20605	uc007npu.1	-270281	ENSMUSG000	Sstr1	somatostatin receptor 1
chr12	57941235	57941535	301	302	57941398	74	61.90123	13.48034	57.80368	Adipose_R1_peak_	Distal Interger	12	58211804	58216036	4233	1	20605	uc007npu.1	-270269	ENSMUSG000	Sstr1	somatostatin receptor 1
chr12	57982800	57983008	209	210	57982916	21	8.43286	3.95423	5.34896	Adipose_R1_peak_	Distal Interger	12	58211804	58216036	4233	1	20605	uc007npu.1	-228796	ENSMUSG000	Sstr1	somatostatin receptor 1
chr12	58120692	58121002	311	312	58120829	22	9.13959	4.13397	6.01526	Adipose_R1_peak_	Distal Interger	12	58211804	58216036	4233	1	20605	uc007npu.1	-90802	ENSMUSG000	Sstr1	somatostatin receptor 1
chr12	58121572	58121794	223	224	58121682	37	21.59274	6.83004	18.04766	Adipose_R1_peak_	Distal Interger	12	58211804	58216036	4233	1	20605	uc007npu.1	-90010	ENSMUSG000	Sstr1	somatostatin receptor 1
chr12	58121586	58121754	169	170	58121675	36	10.5378	3.41206	7.27949	Adipose_R2_peak_	Distal Interger	12	58211804	58216036	4233	1	20605	uc007npu.1	-90050	ENSMUSG000	Sstr1	somatostatin receptor 1
chr12	58129442	58129604	163	164	58129512	22	9.13959	4.13397	6.01526	Adipose_R1_peak_	Distal Interger	12	58211804	58216036	4233	1	20605	uc007npu.1	-82200	ENSMUSG000	Sstr1	somatostatin receptor 1
chr12	58193635	58193866	232	233	58193770	39	23.47001	7.18952	19.88325	Adipose_R1_peak_	Distal Interger	12	58211804	58216036	4233	1	20605	uc007npu.1	-17938	ENSMUSG000	Sstr1	somatostatin receptor 1
chr12	58193660	58193828	169	170	58193820	39	12.34187	3.68871	8.98501	Adipose_R2_peak_	Distal Interger	12	58211804	58216036	4233	1	20605	uc007npu.1	-17976	ENSMUSG000	Sstr1	somatostatin receptor 1
chr12	58350157	58350397	241	242	58350270	31	16.23981	5.75161	12.83549	Adipose_R1_peak_	Distal Interger	12	58264720	58269258	4539	2	66864	uc007npv.2	-80899	ENSMUSG000	Clec14a	C-type lectin domain family 14, member a
chr12	58350182	58350397	216	217	58350296	41	13.59774	3.87315	10.1803	Adipose_R2_peak_	Distal Interger	12	58264720	58269258	4539	2	66864	uc007npv.2	-80924	ENSMUSG000	Clec14a	C-type lectin domain family 14, member a
chr12	58660654	58660835	182	183	58660742	26	12.14079	4.85292	8.87838	Adipose_R1_peak_	Distal Interger	12	58958384	59012017	53634	2	20334	uc007npw.1	351182	ENSMUSG000	Sec23a	SEC23 homolog A, COPII coat complex component
chr12	58664012	58664277	266	267	58664140	33	17.97508	6.11109	14.52164	Adipose_R1_peak_	Distal Interger	12	58958384	59012017	53634	2	20334	uc007npw.1	347740	ENSMUSG000	Sec23a	SEC23 homolog A, COPII coat complex component
chr12	58664056	58664226	171	172	58664152	45	16.22858	4.24202	12.70278	Adipose_R2_peak_	Distal Interger	12	58958384	59012017	53634	2	20334	uc007npw.1	347791	ENSMUSG000	Sec23a	SEC23 homolog A, COPII coat complex component
chr12	58665009	58665214	206	207	58665098	32	17.10103	5.93135	13.67137	Adipose_R1_peak_	Distal Interger	12	58958384	59012017	53634	2	20334	uc007npw.1	346803	ENSMUSG000	Sec23a	SEC23 homolog A, COPII coat complex component
chr12	58665020	58665162	143	144	58665103	31	7.7623	2.95097	4.69388	Adipose_R2_peak_	Distal Interger	12	58958384	59012017	53634	2	20334	uc007npw.1	346855	ENSMUSG000	Sec23a	SEC23 homolog A, COPII coat complex component
chr12	58688715	58689082	368	369	58688891	78	66.82238	14.19929	62.68234	Adipose_R1_peak_	Distal Interger	12	58958384	59012017	53634	2	20334	uc007npw.1	322935	ENSMUSG000	Sec23a	SEC23 homolog A, COPII coat complex component
chr12	58688798	58689027	230	231	58688902	45	16.22858	4.24202	12.70278	Adipose_R2_peak_	Distal Interger	12	58958384	59012017	53634	2	20334	uc007npw.1	322990	ENSMUSG000	Sec23a	SEC23 homolog A, COPII coat complex component
chr12	58823787	58824003	217	218	58823854	27	12.93148	5.03266	9.63827	Adipose_R1_peak_	Distal Interger	12	58958384	59012017	53634	2	20334	uc007npw.1	188014	ENSMUSG000	Sec23a	SEC23 homolog A, COPII coat complex component
chr12	58860806	58860966	161	162	58860890	23	9.86443	4.31371	6.70247	Adipose_R1_peak_	Distal Interger	12	58958384	59012017	53634	2	20334	uc007npw.1	151051	ENSMUSG000	Sec23a	SEC23 homolog A, COPII coat complex component
chr12	58863322	58863489	168	169	58863395	28	11.64184	4.41203	8.40903	Adipose_R1_peak_	Distal Interger	12	58958384	59012017	53634	2	20334	uc007npw.1	148528	ENSMUSG000	Sec23a	SEC23 homolog A, COPII coat complex component
chr12	58863327	58863479	153	154	58863397	32	8.29282	3.04319	5.18352	Adipose_R2_peak_	Distal Interger	12	58958384	59012017	53634	2	20334	uc007npw.1	148538	ENSMUSG000	Sec23a	SEC23 homolog A, COPII coat complex component
chr12	58865463	58865759	297	298	58865624	59	44.30963	10.78427	40.38949	Adipose_R1_peak_	Distal Interger	12	58958384	59012017	53634	2	20334	uc007npw.1	146258	ENSMUSG000	Sec23a	SEC23 homolog A, COPII coat complex component
chr12	58865566	58865685	120	121	58865624	34	9.39139	3.22762	6.20474	Adipose_R2_peak_	Distal Interger	12	58958384	59012017	53634	2	20334	uc007npw.1	146332	ENSMUSG000	Sec23a	SEC23 homolog A, COPII coat complex component
chr12	58867240	58867440	201	202	58867345	30	15.39182	5.57188	12.01394	Adipose_R1_peak_	Distal Interger	12	58958384	59012017	53634	2	20334	uc007npw.1	144577	ENSMUSG000	Sec23a	SEC23 homolog A, COPII coat complex component
chr12	58952703	58952912	210	211	58952868	19	7.07687	3.59476	4.08458	Adipose_R1_peak_	Distal Interger	12	58958384	59012017	53634	2	20334	uc007npw.1	59105	ENSMUSG000	Sec23a	SEC23 homolog A, COPII coat complex component
chr12	59123700	59123868	169	170	59123779	24	10.60668	4.49345	7.40961	Adipose_R1_peak_	Distal Interger	12	59129746	59163019	33274	1	217615	uc007nqd.2	-5878	NA	NA	NA
chr12	59272006	59272219	214	215	59272114	27	12.93148	5.03266	9.63827	Adipose_R1_peak_	Distal Interger	12	59200655	59219483	18829	2	70611	uc007nqk.2	-52523	ENSMUSG000	Fbxo33	F-box protein 33
chr12	59272036	59272246	211	212	59272121	38	11.72962	3.59649	8.4047	Adipose_R2_peak_	Distal Interger	12	59200655	59219483	18829	2	70611	uc007nqk.2	-52553	ENSMUSG000	Fbxo33	F-box protein 33
chr12	59318445	59318585	141	142	59318507	20	7.74501	3.7745	4.70467	Adipose_R1_peak_	Distal Interger	12	59200655	59219483	18829	2	70611	uc007nqk.2	-98962	ENSMUSG000	Fbxo33	F-box protein 33
chr12	59359273	59359475	203	204	59359373	33	17.97508	6.11109	14.52164	Adipose_R1_peak_	Distal Interger	12	59200655	59219483	18829	2	70611	uc007nqk.2	-139790	ENSMUSG000	Fbxo33	F-box protein 33
chr12	59359281	59359473	193	194	59359393	40	12.96465	3.78093	9.57654	Adipose_R2_peak_	Distal Interger	12	59200655	59219483	18829	2	70611	uc007nqk.2	-139798	ENSMUSG000	Fbxo33	F-box protein 33
chr12	59597365	59597561	197	198	59597464	19	7.07687	3.59476	4.08458	Adipose_R1_peak_	Distal Interger	12	59200655	59219483	18829	2	70611	uc007nqk.2	-377882	ENSMUSG000	Fbxo33	F-box protein 33
chr12	59643386	59643568	183	184	59643490	29	6.74062	2.76653	3.75989	Adipose_R2_peak_	Distal Interger	12	59200655	59219483	18829	2	70611	uc007nqk.2	-423903	ENSMUSG000	Fbxo33	F-box protein 33
chr12	59754673	59754821	149	150	59754739	20	7.74501	3.7745	4.70467	Adipose_R1_peak_	Distal Interger	12	59200655	59219483	18829	2	70611	uc007nqk.2	-535190	ENSMUSG000	Fbxo33	F-box protein 33
chr12	59948734	59948937	204	205	59948847	40	12.96465	3.78093	9.57654	Adipose_R2_peak_	Distal Interger	12	59200655	59219483	18829	2	70611	uc007nqk.2	-729251	ENSMUSG000	Fbxo33	F-box protein 33
chr12	59948747	59948945	199	200	59948839	23	9.86443	4.31371	6.70247	Adipose_R1_peak_	Distal Interger	12	59200655	59219483	18829	2	70611	uc007nqk.2	-729264	ENSMUSG000	Fbxo33	F-box protein 33
chr12	60086940	60087169	230	231	60087057	27	12.93148	5.03266	9.63827	Adipose_R1_peak_	Distal Interger	12	59200655	59219483	18829	2	70611	uc007nqk.2	-867457	ENSMUSG000	Fbxo33	F-box protein 33
chr12	60087017	60087121	105	106	60087068	31	7.7623	2.95097	4.69388	Adipose_R2_peak_	Distal Interger	12	59200655	59219483	18829	2	70611	uc007nqk.2	-867534	ENSMUSG000	Fbxo33	F-box protein 33
chr12	60161329	60161486	158	159	60161417	21	8.43286	3.95423	5.34896	Adipose_R1_peak_	Distal Interger	12	59200655	59219483	18829	2	70611	uc007nqk.2	-941846	ENSMUSG000	Fbxo33	F-box protein 33
chr12	60161339	60161486	148	149	60161403	30	7.24477	2.85875	4.21917	Adipose_R2_peak_	Distal Interger	12	59200655	59219483	18829	2	70611	uc007nqk.2	-941856	ENSMUSG000	Fbxo33	F-box protein 33
chr12	60348818	60349027	210	211	60349010	46	16.90986	4.33423	13.35898	Adipose_R2_peak_	Distal Interger	12	59200655	59219483	18829	2	70611	uc007nqk.2	-1129335	ENSMUSG000	Fbxo33	F-box protein 33
chr12	60348861	60349027	167	168	60349000	20	7.74501	3.7745	4.70467	Adipose_R1_peak_	Distal Interger	12	59200655	59219483	18829	2	70611	uc007nqk.2	-1129378	ENSMUSG000	Fbxo33	F-box protein 33
chr12	60468569	60468737	169	170	60468643	22	9.13959	4.13397	6.01526	Adipose_R1_peak_	Distal Interger	12	61499660	61521820	22161	2	1.01E+08	uc007nql.1	1053083	NA	Gm20063	predicted gene, 20063
chr12	60470426	60470670	245	246	60470544	43	27.35048	7.90847	23.68561	Adipose_R1_peak_	Distal Interger	12	61499660	61521820	22161	2	1.01E+08	uc007nql.1	1051150	NA	Gm20063	predicted gene, 20063
chr12	60470444	60470685	242	243	60470559	56	24.19188	5.25641	20.43163	Adipose_R2_peak_	Distal Interger	12	61499660	61521820	22161	2	1.01E+08	uc007nql.1	1051135	NA	Gm20063	predicted gene, 20063
chr12	60501368	60501611	244	245	60501498	37	21.59274	6.83004	18.04766	Adipose_R1_peak_	Distal Interger	12	61499660	61521820	22161	2	1.01E+08	uc007nql.1	1020209	NA	Gm20063	predicted gene, 20063
chr12	60501417	60501566	150	151	60501496	35	9.95877	3.31984	6.73562	Adipose_R2_peak_	Distal Interger	12	61499660	61521820	22161	2	1.01E+08	uc007nql.1	1020254	NA	Gm20063	predicted gene, 20063
chr12	60505550	60505842	293	294	60505681	74	39.12209	6.91633	35.10124	Adipose_R2_peak_	Distal Interger	12	61499660	61521820	22161	2	1.01E+08	uc007nql.1	1015978	NA	Gm20063	predicted gene, 20063
chr12	60505554	60505828	275	276	60505679	60	45.43621	10.96401	41.50272	Adipose_R1_peak_	Distal Interger	12	61499660	61521820	22161	2	1.01E+08	uc007nql.1	1015992	NA	Gm20063	predicted gene, 20063
chr12	60702218	60702475	258	259	60702345	42	26.36523	7.72873	22.71906	Adipose_R1_peak_	Distal Interger	12	61499660	61521820	22161	2	1.01E+08	uc007nql.1	819345	NA	Gm20063	predicted gene, 20063
chr12	60705926	60706330	405	406	60706022	21	8.43286	3.95423	5.34896	Adipose_R1_peak_	Distal Interger	12	61499660	61521820	22161	2	1.01E+08	uc007nql.1	815490	NA	Gm20063	predicted gene, 20063
chr12	60708258	60708395	138	139	60708325	33	8.83596	3.1354	5.68652	Adipose_R2_peak_	Distal Interger	12	61499660	61521820	22161	2	1.01E+08	uc007nql.1	813425	NA	Gm20063	predicted gene, 20063
chr12	60777320	60777495	176	177	60777417	24	10.60668	4.49345	7.40961	Adipose_R1_peak_	Distal Interger	12	61499660	61521820	22161	2	1.01E+08	uc007nql.1	744325	NA	Gm20063	predicted gene, 20063
chr12	61045006	61045258	253	254	61045160	59	44.30963	10.78427	40.38949	Adipose_R1_peak_	Distal Interger	12	61499660	61521820	22161	2	1.01E+08	uc007nql.1	476562	NA	Gm20063	predicted gene, 20063

chr12	61045021	61045261	241	242	61045183	72	37.3624	6.73189	33.36734	Adipose_R2_peak_4	Distal Intergen	12	61499660	61521820	22161	2	1.01E+08	uc007nql.1	476559	NA	Gm20063	predicted gene, 20063
chr12	61268604	61268773	170	171	61268693	22	9.13959	4.13397	6.01526	Adipose_R1_peak_3	Distal Intergen	12	61499660	61521820	22161	2	1.01E+08	uc007nql.1	253047	NA	Gm20063	predicted gene, 20063
chr12	61269685	61269886	202	203	61269766	26	12.14079	4.85292	8.87838	Adipose_R1_peak_3	Distal Intergen	12	61499660	61521820	22161	2	1.01E+08	uc007nql.1	251934	NA	Gm20063	predicted gene, 20063
chr12	61292248	61292479	232	233	61292381	38	22.52591	7.00978	18.96	Adipose_R1_peak_3	Distal Intergen	12	61499660	61521820	22161	2	1.01E+08	uc007nql.1	229341	NA	Gm20063	predicted gene, 20063
chr12	61292268	61292479	212	213	61292394	45	16.22858	4.24202	12.70278	Adipose_R2_peak_4	Distal Intergen	12	61499660	61521820	22161	2	1.01E+08	uc007nql.1	229341	NA	Gm20063	predicted gene, 20063
chr12	61374186	61374490	305	306	61374347	72	59.47482	13.12087	55.39848	Adipose_R1_peak_3	Distal Intergen	12	61499660	61521820	22161	2	1.01E+08	uc007nql.1	147330	NA	Gm20063	predicted gene, 20063
chr12	61374232	61374496	265	266	61374349	74	39.12209	6.91633	35.10124	Adipose_R2_peak_4	Distal Intergen	12	61499660	61521820	22161	2	1.01E+08	uc007nql.1	147324	NA	Gm20063	predicted gene, 20063
chr12	61375367	61375508	142	143	61375451	20	7.74501	3.7745	4.70467	Adipose_R1_peak_3	Distal Intergen	12	61499660	61521820	22161	2	1.01E+08	uc007nql.1	146312	NA	Gm20063	predicted gene, 20063
chr12	61584226	61584578	353	354	61584415	30	15.39182	5.57188	12.01394	Adipose_R1_peak_3	Intron (uc007r	12	61523167	61845258	322092	1	238205	uc007nqm.2	61059	ENSMUSG000	Lrfn5	leucine rich repeat and fibronectin type III domain containing 5
chr12	61634474	61634725	252	253	61634592	35	19.76029	6.47056	16.26028	Adipose_R1_peak_3	Intron (uc007r	12	61523167	61845258	322092	1	238205	uc007nqm.2	111307	ENSMUSG000	Lrfn5	leucine rich repeat and fibronectin type III domain containing 5
chr12	61969449	61969681	233	234	61969549	21	8.38957	3.93597	5.3423	Adipose_R1_peak_3	Distal Intergen	12	61523167	61845258	322092	1	238205	uc007nqm.2	446282	ENSMUSG000	Lrfn5	leucine rich repeat and fibronectin type III domain containing 5
chr12	61969455	61969567	113	114	61969550	27	5.77405	2.5821	2.88921	Adipose_R2_peak_4	Distal Intergen	12	61523167	61845258	322092	1	238205	uc007nqm.2	446288	ENSMUSG000	Lrfn5	leucine rich repeat and fibronectin type III domain containing 5
chr12	62059930	62060254	325	326	62060088	86	67.30136	12.45934	63.1593	Adipose_R1_peak_3	Distal Intergen	12	61523167	61845258	322092	1	238205	uc007nqm.2	536763	ENSMUSG000	Lrfn5	leucine rich repeat and fibronectin type III domain containing 5
chr12	62059964	62060199	236	237	62060089	63	29.74474	5.90194	25.87429	Adipose_R2_peak_4	Distal Intergen	12	61523167	61845258	322092	1	238205	uc007nqm.2	536797	ENSMUSG000	Lrfn5	leucine rich repeat and fibronectin type III domain containing 5
chr12	62060761	62061020	260	261	62060887	41	25.38991	7.54899	21.76305	Adipose_R1_peak_3	Distal Intergen	12	61523167	61845258	322092	1	238205	uc007nqm.2	537594	ENSMUSG000	Lrfn5	leucine rich repeat and fibronectin type III domain containing 5
chr12	62060812	62061002	191	192	62060927	39	12.34187	3.68871	8.98501	Adipose_R2_peak_4	Distal Intergen	12	61523167	61845258	322092	1	238205	uc007nqm.2	537645	ENSMUSG000	Lrfn5	leucine rich repeat and fibronectin type III domain containing 5
chr12	62245658	62245844	187	188	62245752	36	10.5378	3.41206	7.27949	Adipose_R2_peak_4	Distal Intergen	12	62687787	62688336	550	1	73309	uc057bbm.1	-441943	NA	Spanxn4	SPANX family, member N4
chr12	62245691	62245844	154	155	62245755	19	7.07687	3.59476	4.08458	Adipose_R1_peak_3	Distal Intergen	12	62687787	62688336	550	1	73309	uc057bbm.1	-441943	NA	Spanxn4	SPANX family, member N4
chr12	62509816	62510299	484	485	62509923	32	17.10103	5.93135	13.67137	Adipose_R1_peak_3	Distal Intergen	12	62687787	62688336	550	1	73309	uc057bbm.1	-177488	NA	Spanxn4	SPANX family, member N4
chr12	62509837	62510249	413	414	62509913	38	11.72962	3.59649	8.4047	Adipose_R2_peak_4	Distal Intergen	12	62687787	62688336	550	1	73309	uc057bbm.1	-177538	NA	Spanxn4	SPANX family, member N4
chr12	62513532	62513673	142	143	62513595	18	6.42934	3.41502	3.48999	Adipose_R1_peak_3	Distal Intergen	12	62687787	62688336	550	1	73309	uc057bbm.1	-174114	NA	Spanxn4	SPANX family, member N4
chr12	62736018	62736183	166	167	62736080	19	7.07687	3.59476	4.08458	Adipose_R1_peak_3	Distal Intergen	12	62687787	62688336	550	1	73309	uc057bbm.1	48231	NA	Spanxn4	SPANX family, member N4
chr12	62828029	62828199	171	172	62828124	26	12.14079	4.85292	8.87838	Adipose_R1_peak_3	Distal Intergen	12	62687787	62688336	550	1	73309	uc057bbm.1	140242	NA	Spanxn4	SPANX family, member N4
chr12	62828065	62828166	102	103	62828118	29	6.74062	2.76653	3.75989	Adipose_R2_peak_4	Distal Intergen	12	62687787	62688336	550	1	73309	uc057bbm.1	140278	NA	Spanxn4	SPANX family, member N4
chr12	62961013	62961304	292	293	62961165	64	50.01186	11.68296	46.0279	Adipose_R1_peak_3	Distal Intergen	12	62687787	62688336	550	1	73309	uc057bbm.1	273226	NA	Spanxn4	SPANX family, member N4
chr12	62961022	62961282	261	262	62961149	63	29.74474	5.90194	25.87429	Adipose_R2_peak_4	Distal Intergen	12	62687787	62688336	550	1	73309	uc057bbm.1	273235	NA	Spanxn4	SPANX family, member N4
chr12	62978920	62979145	226	227	62979018	32	17.10103	5.93135	13.67137	Adipose_R1_peak_3	Distal Intergen	12	62687787	62688336	550	1	73309	uc057bbm.1	291133	NA	Spanxn4	SPANX family, member N4
chr12	63043127	63043390	264	265	63043267	39	23.47001	7.18952	19.88325	Adipose_R1_peak_3	Distal Intergen	12	62687787	62688336	550	1	73309	uc057bbm.1	355340	NA	Spanxn4	SPANX family, member N4
chr12	63043190	63043311	122	123	63043261	29	6.74062	2.76653	3.75989	Adipose_R2_peak_4	Distal Intergen	12	62687787	62688336	550	1	73309	uc057bbm.1	355403	NA	Spanxn4	SPANX family, member N4
chr12	63299665	63299946	282	283	63299800	38	22.52591	7.00978	18.96	Adipose_R1_peak_3	Distal Intergen	12	62687787	62688336	550	1	73309	uc057bbm.1	611878	NA	Spanxn4	SPANX family, member N4
chr12	63299706	63299918	213	214	63299813	41	13.59774	3.87315	10.1803	Adipose_R2_peak_4	Distal Intergen	12	62687787	62688336	550	1	73309	uc057bbm.1	611919	NA	Spanxn4	SPANX family, member N4
chr12	63321043	63321265	223	224	63321172	33	17.97508	6.11109	14.52164	Adipose_R1_peak_3	Distal Intergen	12	62687787	62688336	550	1	73309	uc057bbm.1	633256	NA	Spanxn4	SPANX family, member N4
chr12	63321087	63321274	188	189	63321187	44	15.55653	4.1498	12.05625	Adipose_R2_peak_4	Distal Intergen	12	62687787	62688336	550	1	73309	uc057bbm.1	633300	NA	Spanxn4	SPANX family, member N4
chr12	63354647	63354961	315	316	63354786	57	42.07807	10.4248	38.18469	Adipose_R1_peak_3	Distal Intergen	12	62687787	62688336	550	1	73309	uc057bbm.1	666860	NA	Spanxn4	SPANX family, member N4
chr12	63354715	63354905	191	192	63354791	40	12.96465	3.78093	9.57654	Adipose_R2_peak_4	Distal Intergen	12	62687787	62688336	550	1	73309	uc057bbm.1	666928	NA	Spanxn4	SPANX family, member N4
chr12	63358176	63358390	215	216	63358322	33	17.97508	6.11109	14.52164	Adipose_R1_peak_3	Distal Intergen	12	62687787	62688336	550	1	73309	uc057bbm.1	670389	NA	Spanxn4	SPANX family, member N4
chr12	63414357	63414762	406	407	63414447	37	11.12817	3.50427	7.83613	Adipose_R2_peak_4	Distal Intergen	12	62687787	62688336	550	1	73309	uc057bbm.1	726570	NA	Spanxn4	SPANX family, member N4
chr12	63414363	63414820	458	459	63414466	25	11.36566	4.67319	8.13482	Adipose_R1_peak_3	Distal Intergen	12	62687787	62688336	550	1	73309	uc057bbm.1	726576	NA	Spanxn4	SPANX family, member N4
chr12	63453169	63453327	159	160	63453233	18	6.42934	3.41502	3.48999	Adipose_R1_peak_3	Distal Intergen	12	62687787	62688336	550	1	73309	uc057bbm.1	765382	NA	Spanxn4	SPANX family, member N4
chr12	63573270	63573622	353	354	63573435	154	145.2696	19.25913	140.7698	Adipose_R1_peak_3	Distal Intergen	12	62687787	62688336	550	1	73309	uc057bbm.1	885483	NA	Spanxn4	SPANX family, member N4
chr12	63573296	63573605	310	311	63573432	106	70.05344	9.8673	65.71089	Adipose_R2_peak_4	Distal Intergen	12	62687787	62688336	550	1	73309	uc057bbm.1	885509	NA	Spanxn4	SPANX family, member N4
chr12	63579255	63579393	139	140	63579319	19	7.07687	3.59476	4.08458	Adipose_R1_peak_3	Distal Intergen	12	62687787	62688336	550	1	73309	uc057bbm.1	891468	NA	Spanxn4	SPANX family, member N4
chr12	63693105	63693365	261	262	63693235	28	13.73721	5.2124	10.41463	Adipose_R1_peak_3	Distal Intergen	12	64471333	64474898	3566	2	623046	uc011yme.1	781533	ENSMUSG000	Fscb	fibrous sheath CABYR binding protein
chr12	63695175	63695485	311	312	63695328	48	32.41826	8.80716	28.65523	Adipose_R1_peak_3	Distal Intergen	12	64471333	64474898	3566	2	623046	uc011yme.1	779413	ENSMUSG000	Fscb	fibrous sheath CABYR binding protein
chr12	63695183	63695430	248	249	63695295	50	19.72356	4.7031	16.08069	Adipose_R2_peak_4	Distal Intergen	12	64471333	64474898	3566	2	623046	uc011yme.1	779468	ENSMUSG000	Fscb	fibrous sheath CABYR binding protein
chr12	63838792	63839312	521	522	63838858	23	9.86443	4.31371	6.70247	Adipose_R1_peak_3	Distal Intergen	12	64471333	64474898	3566	2	623046	uc011yme.1	635586	ENSMUSG000	Fscb	fibrous sheath CABYR binding protein
chr12	63909731	63910129	399	400	63909809	27	12.93148	5.03266	9.63827	Adipose_R1_peak_3	Distal Intergen	12	64471333	64474898	3566	2	623046	uc011yme.1	564769	ENSMUSG000	Fscb	fibrous sheath CABYR binding protein
chr12	63909755	63909864	110	111	63909799	27	5.77405	2.5821	2.88921	Adipose_R2_peak_4	Distal Intergen	12	64471333	64474898	3566	2	623046	uc011yme.1	565034	ENSMUSG000	Fscb	fibrous sheath CABYR binding protein
chr12	63911886	63912035	150	151	63911944	17	5.80341	3.23528	2.92378	Adipose_R1_peak_3	Distal Intergen	12	64471333	64474898	3566	2	623046	uc011yme.1	562863	ENSMUSG000	Fscb	fibrous sheath CABYR binding protein
chr12	63925933	63926083	151	152	63925998	29	6.74062	2.76653	3.75989	Adipose_R2_peak_4	Distal Intergen	12	64471333	64474898	3566	2	623046	uc011yme.1	548815	ENSMUSG000	Fscb	fibrous sheath CABYR binding protein
chr12	63925943	63926168	226	227	63926046	32	17.10103	5.93135	13.67137	Adipose_R1_peak_3	Distal Intergen	12	64471333	64474898	3566	2	623046	uc011yme.1	548730	ENSMUSG000	Fscb	fibrous sheath CABYR binding protein
chr12	64030535	64030808	274	275	64030673	32	17.10103	5.93135	13.67137	Adipose_R1_peak_3	Distal Intergen	12	64471333	64474898	3566	2	623046	uc011yme.1	444090	ENSMUSG000	Fscb	fibrous sheath CABYR binding protein
chr12	64030621	64030749	129	130	64030692	30	7.24477	2.85875	4.21917	Adipose_R2_peak_4	Distal Intergen	12	64471333	64474898	3566	2	623046	uc011yme.1	444149	ENSMUSG000	Fscb	fibrous sheath CABYR binding protein
chr12	64095584	64095789	206	207	64095719	25	11.36566	4.67319	8.13482	Adipose_R1_peak_3	Distal Intergen	12	64471333	64474898	3566	2	623046	uc011yme.1	379109	ENSMUSG000	Fscb	fibrous sheath CABYR binding protein
chr12	64461171	64461578	408	409	64461245	22	9.13959	4.13397	6.01526	Adipose_R1_peak_3	Distal Intergen	12	64471333	64474898	3566	2	623046	uc011yme.1	13320	ENSMUSG000	Fscb	fibrous sheath CABYR binding protein
chr12	64461993	64462241	249	250	64462099	37	21.59274	6.83004	18.04766	Adipose_R1_peak_3	Distal Intergen	12	64471333	64474898	3566	2	623046	uc011yme.1	12657	ENSMUSG000	Fscb	fibrous sheath CABYR binding protein
chr12	64462031	64462183	153	154	64462097	35	9.95877	3.31984	6.73562	Adipose_R2_peak_4	Distal Intergen	12	64471333	64474898	3566	2	623046	uc011yme.1	12715	ENSMUSG000	Fscb	fibrous sheath CABYR binding protein
chr12	64603316	64603586	271	272	64603448	41	25.38991	7.54899	21.76305	Adipose_R1_peak_3	Distal Intergen	12	64471333	64474898	3566	2	623046	uc011yme.1	-128418	ENSMUSG000	Fscb	fibrous sheath CABYR binding protein
chr12	64603358	64603549	192	193	64603458	44	15.55653	4.1498	12.05625	Adipose_R2_peak_4	Distal Intergen	12	64471333	64474898	3566	2	623046	uc011yme.1	-128460	ENSMUSG000	Fscb	fibrous sheath CABYR binding protein
chr12	64678623	64678824	202	203	64678734	25	11.36566	4.67319	8.13482	Adipose_R1_peak_3	Distal Intergen	12	64471333	64474898	3566	2	623046	uc011yme.1	-203725	ENSMUSG000	Fscb	fibrous sheath CABYR binding protein
chr12	64678655	64678848	194	195	64678741	39	12.34187	3.68871	8.98501	Adipose_R2_peak_4	Distal Intergen	12	64471333	64474898	3566	2	623046	uc011yme.1	-203757	ENSMUSG000	Fscb	fibrous sheath CABYR binding protein
chr12	64726765	64726945	181	182	64726829	21	8.43286	3.95423	5.34896	Adipose_R1_peak_3	Distal Intergen	12	64917911	64924591	6681	1	217648	uc007nqo.1	-190966	ENSMUSG000	Gm527	predicted gene 527
chr12	64891596	64891884	289	290	64891744	68	54.69342	12.40192	50.66183	Adipose_R1_peak_3	Distal Intergen	12	64917911	64924591	6681	1	217648	uc007nqo.1	-26027	ENSMUSG000	Gm527	predicted gene 527
chr12	64891634	64891869	236	237	64891759	59	26.5291	5.53306	22.7188	Adipose_R2_peak_4	Distal Intergen	12	64917911	64924591	6681	1	217648	uc007nqo.1	-26042	ENSMUSG000	Gm527	predicted gene 527
chr12	65178966	65179115	150	151	65179029	28	12.9448	4.90088	9.65145	Adipose_R1_peak_3	Distal Intergen	12	65132735	65172580	39846	2	217653	uc007nrg.2	-6386	ENSMUSG000	Mis18bp1	MIS18 binding protein 1
chr12	65178972	65179112	141	142	65179026	34	9.39139	3.22762	6.20474	Adipose_R2_peak_4	Distal Intergen	12	65132735	65172580	39846	2	217653	uc007nrg.2	-6392	ENSMUSG000	Mis18bp1	MIS18 binding protein 1
chr12	65260256	65260442	187	188	65260330	24	10.60668	4.49345	7.40961	Adipose_R1_peak_3	Distal Intergen	12	65225517	65228454	2938	1	70948	uc007nrk.1	34739	ENSMUSG000	Wdr20rt	WD repeat domain 20, retrogene
chr12	65263544	65263765	222	223	65263635	26	12.14079	4.85292	8.87838	Adipose_R1_peak_3	Distal Intergen	12	65225517	65228454	2938	1	70948	uc007nrk.1	38027	ENSMUSG000	Wdr20rt	WD repeat domain 20, retrogene
chr12	65266100	65266333	234	235	65266207	26	12.14079	4.85292	8.87838	Adipose_R1_peak_3	Distal Intergen	12	65225517	65228454	2938	1	70948	uc007nrk.1	40583	ENSMUSG000	Wdr20rt	WD repeat domain 20, retrogene
chr12	65266521	65266799	279	280	65266645	51	20.44827	4.79532	16.78434	Adipose_R2_peak_4	Distal Intergen	12	65225517	65228454	2938	1	70948	uc007nrk.1	41004	ENSMUSG000	Wdr20rt	WD repeat domain 20, retrogene
chr12	65266522	65266809	288	289	65266662	54	38.78658	9.88558	34.93639	Adipose_R1_peak_3	Distal Intergen	12	65225517	65228454	2938	1	70948	uc007nrk.1	41005	ENSMUSG000	Wdr20rt	WD repeat domain 20, retrogene
chr12	65287248	65287480	233	234	65287360	30	15.39182	5.57188	12.01394	Adipose_R1_peak_3	Distal Intergen	12	65225517	65228454	2938	1	70948	uc007nrk.1	61731	ENSMUSG000	Wdr20rt	WD repeat domain 20, retrogene
chr12	65912934	65913181	248	249	65913047	17	5.80341	3.23528	2.92378	Adipose_R1_peak_3	Distal Intergen	12	66283379	66284401	1023	2	238217	uc011ymh.1	371220	ENSMUSG000	Rpl10l	ribosomal protein L10-like
chr12	66109031	66109284	254	255	66109155	45	29.34988	8.26794	25.64927	Adipose_R1_peak_3	Distal Intergen	12	66283379	66284401	1023	2	238217	uc011ymh.1	175117	ENSMUSG000	Rpl10l	ribosomal protein L10-like
chr12	66109083	66109267	185	186	66109173	39	12.34187	3.68871	8.98501	Adipose_R2_peak_4	Distal Intergen	12	66283379	66284401	1023	2	238217	uc011ymh.1	175134	ENSMUSG000	Rpl10l	ribosomal protein L10-like
chr12	66166294	66166505	212	213	66166397	28	13.73721	5.2124	10.41463	Adipose_R1_peak_3	Distal Intergen	12	66283379	66284401	1023	2	238217	uc011ymh.1	117896	ENSMUSG000	Rpl10l	ribosomal protein L10-like
chr12	66171783	66171921	139	140	66171809	17	5.80341	3.23528	2.92378	Adipose_R1_peak_3	Distal Intergen	12	66283379	66284401	1023	2	238217	uc011ymh.1	112480	ENSMUSG000	Rpl10l	ribosomal protein L10-like
chr12	66414139	66414283	145	146	66414220	24	10.60668	4.49345	7.40961	Adipose_R1_peak_3	Distal Intergen	12	66283379	66284401	1023	2	238217	uc011ymh.1	-129738	ENSMUSG000	Rpl10l	ribosomal protein L10-like
chr12	66560020	66560191	172	173	66560151	19	7.07687	3.59476	4.08458	Adipose_R1_peak_3	Intron (uc007r	12	66283379	66284401	1023	2	238217	uc011ymh.1	-275619	ENSMUSG000	Rpl10l	ribosomal protein L10-like
chr12	66782736	66782990	255	256	66782867	43	27.35048	7.90847	23.68561	Adipose_R1_peak_3	Intron (uc007r	12	66466059	67222549	756491	2	320772	uc007nrl.3	439559	ENSMUSG000	Mdga2	MAM domain containing glycosylphosphatidylinositol anchor 2
chr12	66782800	66782944	145	146	66782871	34	9.39139	3.22762	6.20474	Adipose_R2_peak_4	Intron (uc007r	12	66466059	67222549	756491	2	320772	uc007nrl.3	439605	ENSMUSG000	Mdga2	MAM domain containing glycosylphosphatidylinositol anchor 2
chr12	66868664	66868854	191	192	66868727	17	5.80341	3.23528	2.92378	Adipose_R1_peak_3	5' UTR	12	66466059	67222549	756491	2	320772	uc007nrl.3	353695	ENSMUSG000	Mdga2	MAM domain containing glycosylphosphatidylinositol anchor 2
chr12	66910827	66911141	315	316	66910963	29	14.55748	5.39214	11.20652	Adipose_R1_peak_3	Intron (uc007r	12	66466059	67222549	756491	2	320772	uc007nrl.3	311408	ENSMUSG000	Mdga2	MAM domain containing glycosylphosphatidylinositol anchor 2
chr12	66945060	66945351	292	293	66945204	62	47.71039	11.32349	43.751	Adipose_R1_peak_3	Intron (uc007r	12	66466059	67222549	756491	2	320772	uc007nrl.3	277198	ENSMUSG000	Mdga2	MAM domain containing glycosylphosphatidylinositol anchor 2
chr12	66945106	66945269	164	165	66945199	38	11.72962	3.59649	8.4047	Adipose_R2_peak_4	Intron (uc007r	12	66466059	67222549	756491	2	320772	uc007nrl.3	277280	ENSMUSG000	Mdga2	MAM domain containing glycosylphosphatidylinositol anchor 2
chr12	67059256	67059702	447	448	67059401	102	21.21182	2.97738	17.52676	Adipose_R2_peak_4	Intron (uc007r	12	66466059	67222549	756491	2	320772	uc007nrl.3	162847	ENSMUSG000	Mdga2	MAM domain containing glycosylphosphatidylinositol anchor 2
chr12	67059294	67059478	185	186	67059424	65	5.55478	1.81296	2.73567	Adipose_R1_peak_3	Intron (uc007r	12	66466059	67222549	756491	2	320772	uc007nrl.3	163071	ENSMUSG000	Mdga2	MAM domain containing glycosylphosphatidylinositol anchor 2
chr12	67360165	67360353	189	190	67360247	22	9.13959	4.13397	6.01526	Adipose_R1_peak_3	Distal Intergen	12	66466059	67222549	756491	2	320772	uc007nrl.3	-137616	ENSMUSG000	Mdga2	MAM domain containing glycosylphosphatidylinositol anchor 2
chr12	67477589	67477686	98	99	67477634	26	5.31252	2.48988	2.47999	Adipose_R2_peak_4	Distal Intergen	12	67656742	67669271	12530	2	1.00E+08	uc011ymj.1	191585	NA	Gm17821	DDB1 and CUL4 associated factor 5 pseudogene
chr12	67534011	67534224	214	215	67534144	29	14.55748	5.39214	11.20652	Adipose_R1_peak_3	Distal Intergen	12	67656742	67669271	12530	2	1.00E+08	uc011ymj.1	135047	NA	Gm17821	DDB1 and CUL4 associated factor 5 pseudogene
chr12	67534084	67534225	142	143	67534156	33	8.83596	3.1354	5.68652	Adipose_R2_peak_4	Distal Intergen	12	67656742	67669271	12530	2	1.00E+08	uc011ymj.1	135046	NA	Gm17821	DDB1 and CUL4 associated factor 5 pseudogene
chr12	67538500	67538799	300	301	67538659	54	38.78658	9.88558	34.93639	Adipose_R1_peak_3	Distal Intergen	12	67656742	67669271	12530	2	1.00E+08	uc011ymj.1	130472	NA	Gm17821	DDB1 and CUL4 associated factor 5 pseudogene
chr12	67559107	67559399	293	294	67559259	70	57.07198	12.76139	53.01762	Adipose_R1_peak_3	Distal Intergen	12	67656742	67669271	12530	2	1.00E+08	uc011ymj.1	109872	NA	Gm17821	DDB1 and CUL4 associated factor 5 pseudogene
chr12	67559111	67559387	277	278	67559246	67	33.06755	6.27081	29.1399	Adipose_R2_peak_4	Distal Intergen	12	67656742	67669271	12530	2	1.00E+08	uc011ymj.1	109884	NA	Gm17821	DDB1 and CUL4 associated factor 5 pseudogene

chr12	67570259	67570396	138	139	67570326	18	6.42934	3.41502	3.48999	Adipose_R1_peak_	Distal Interger	12	67656742	67669271	12530	2	1.00E+08	uc011ymj.1	98875	NA	Gm17821	DDB1 and CUL4 associated factor 5 pseudogene
chr12	67829675	67829962	288	289	67829827	56	40.97333	10.24506	37.09402	Adipose_R1_peak_	Distal Interger	12	67656742	67669271	12530	2	1.00E+08	uc011ymj.1	-160404	NA	Gm17821	DDB1 and CUL4 associated factor 5 pseudogene
chr12	67829697	67829958	262	263	67829820	56	24.19188	5.25641	20.43163	Adipose_R2_peak_	Distal Interger	12	67656742	67669271	12530	2	1.00E+08	uc011ymj.1	-160426	NA	Gm17821	DDB1 and CUL4 associated factor 5 pseudogene
chr12	67835805	67836065	261	262	67835902	24	10.60668	4.49345	7.40961	Adipose_R1_peak_	Distal Interger	12	67656742	67669271	12530	2	1.00E+08	uc011ymj.1	-166534	NA	Gm17821	DDB1 and CUL4 associated factor 5 pseudogene
chr12	68368293	68368433	141	142	68368382	18	6.42934	3.41502	3.48999	Adipose_R1_peak_	Distal Interger	12	67656742	67669271	12530	2	1.00E+08	uc011ymj.1	-699022	NA	Gm17821	DDB1 and CUL4 associated factor 5 pseudogene
chr12	68448140	68448289	150	151	68448183	18	6.42934	3.41502	3.48999	Adipose_R1_peak_	Distal Interger	12	69157722	69159186	1465	2	20090	uc007nrp.2	710897	ENSMUSG000	Rps29	ribosomal protein S29
chr12	68623975	68624076	102	103	68624032	30	7.24477	2.85875	4.21917	Adipose_R2_peak_	Distal Interger	12	69157722	69159186	1465	2	20090	uc007nrp.2	535110	ENSMUSG000	Rps29	ribosomal protein S29
chr12	68711464	68711672	209	210	68711551	31	16.23981	5.75161	12.83549	Adipose_R1_peak_	Distal Interger	12	69157722	69159186	1465	2	20090	uc007nrp.2	447514	ENSMUSG000	Rps29	ribosomal protein S29
chr12	68711470	68711635	166	167	68711561	39	12.34187	3.68871	8.98501	Adipose_R2_peak_	Distal Interger	12	69157722	69159186	1465	2	20090	uc007nrp.2	447551	ENSMUSG000	Rps29	ribosomal protein S29
chr12	68935205	68935337	133	134	68935269	18	6.42934	3.41502	3.48999	Adipose_R1_peak_	Distal Interger	12	69157722	69159186	1465	2	20090	uc007nrp.2	223849	ENSMUSG000	Rps29	ribosomal protein S29
chr12	68961784	68961988	205	206	68961879	28	13.73721	5.2124	10.41463	Adipose_R1_peak_	Distal Interger	12	69157722	69159186	1465	2	20090	uc007nrp.2	197198	ENSMUSG000	Rps29	ribosomal protein S29
chr12	68965265	68965418	154	155	68965328	22	9.13959	4.13397	6.01526	Adipose_R1_peak_	Distal Interger	12	69157722	69159186	1465	2	20090	uc007nrp.2	193768	ENSMUSG000	Rps29	ribosomal protein S29
chr12	69023253	69023432	180	181	69023331	20	7.74501	3.7745	4.70467	Adipose_R1_peak_	Distal Interger	12	69157722	69159186	1465	2	20090	uc007nrp.2	135754	ENSMUSG000	Rps29	ribosomal protein S29
chr12	69364736	69364923	188	189	69364781	27	5.77405	2.5821	2.88921	Adipose_R2_peak_	Distal Interger	12	69372150	69375980	3831	1	11845	uc007nsj.1	-7227	ENSMUSG000	Arf6	ADP-ribosylation factor 6
chr12	69369227	69369340	114	115	69369316	27	5.77405	2.5821	2.88921	Adipose_R2_peak_	Promoter (2-3	12	69372150	69375980	3831	1	11845	uc007nsj.1	-2810	ENSMUSG000	Arf6	ADP-ribosylation factor 6
chr12	69370881	69371070	190	191	69371019	29	6.74062	2.76653	3.75989	Adipose_R2_peak_	Promoter (1-2	12	69372150	69375980	3831	1	11845	uc007nsj.1	-1080	ENSMUSG000	Arf6	ADP-ribosylation factor 6
chr12	69371426	69371633	208	209	69371462	27	5.77405	2.5821	2.88921	Adipose_R2_peak_	Promoter (<=	12	69372150	69375980	3831	1	11845	uc007nsj.1	-517	ENSMUSG000	Arf6	ADP-ribosylation factor 6
chr12	69409779	69409923	145	146	69409906	19	7.07687	3.59476	4.08458	Adipose_R1_peak_	Distal Interger	12	69372150	69375980	3831	1	11845	uc007nsj.1	37629	ENSMUSG000	Arf6	ADP-ribosylation factor 6
chr12	69608569	69608749	181	182	69608694	35	14.15812	4.51911	10.83309	Adipose_R1_peak_	Intron (uc007r	12	69583761	69586730	2970	2	20663	uc007nsn.2	-21839	ENSMUSG000	Sos2	son of sevenless homolog 2 (Drosophila)
chr12	69654081	69654292	212	213	69654177	166	101.4948	9.20623	96.98166	Adipose_R2_peak_	Intron (uc007r	12	69583761	69681852	98092	2	20663	uc007nso.2	27560	ENSMUSG000	Sos2	son of sevenless homolog 2 (Drosophila)
chr12	69654095	69654250	156	157	69654170	82	25.07059	3.93757	21.4603	Adipose_R1_peak_	Intron (uc007r	12	69583761	69681852	98092	2	20663	uc007nso.2	27602	ENSMUSG000	Sos2	son of sevenless homolog 2 (Drosophila)
chr12	70972712	70973006	295	296	70972859	34	9.39139	3.22762	6.20474	Adipose_R2_peak_	Promoter (1-2	12	70974623	70995877	21255	1	19167	uc007ntz.3	-1617	ENSMUSG000	Psma3	proteasome (prosome, macropain) subunit, alpha type 3
chr12	70973977	70974181	205	206	70974065	22	9.13959	4.13397	6.01526	Adipose_R1_peak_	Promoter (<=	12	70974623	70995877	21255	1	19167	uc007ntz.3	-442	ENSMUSG000	Psma3	proteasome (prosome, macropain) subunit, alpha type 3
chr12	70974365	70974560	196	197	70974447	34	9.39139	3.22762	6.20474	Adipose_R2_peak_	Promoter (<=	12	70974623	70995877	21255	1	19167	uc007ntz.3	-63	ENSMUSG000	Psma3	proteasome (prosome, macropain) subunit, alpha type 3
chr12	71592129	71592382	254	255	71592258	44	28.34544	8.08821	24.66239	Adipose_R1_peak_	Distal Interger	12	71540607	71556133	15527	1	73815	uc029rti.1	51522	NA	4930404H11R	RIKEN cDNA 4930404H11 gene
chr12	71592165	71592335	171	172	71592264	40	12.96465	3.78093	9.57654	Adipose_R2_peak_	Distal Interger	12	71540607	71556133	15527	1	73815	uc029rti.1	51558	NA	4930404H11R	RIKEN cDNA 4930404H11 gene
chr12	72640705	72641087	383	384	72640874	52	36.13961	9.34508	32.33373	Adipose_R1_peak_	Distal Interger	12	72650353	72664828	14476	2	66375	uc007nvs.2	23741	ENSMUSG000	Dhrs7	dehydrogenase/reductase (SDR family) member 7
chr12	72640708	72641067	360	361	72640870	51	20.44827	4.79532	16.78434	Adipose_R2_peak_	Distal Interger	12	72650353	72664828	14476	2	66375	uc007nvs.2	23761	ENSMUSG000	Dhrs7	dehydrogenase/reductase (SDR family) member 7
chr12	74635927	74636133	207	208	74635975	29	6.74062	2.76653	3.75989	Adipose_R2_peak_	Distal Interger	12	74297474	74300468	2995	1	386753	uc007nwy.1	338453	ENSMUSG000	Dbpht2	DNA binding protein with his-thr domain
chr12	74635928	74636123	196	197	74636057	20	7.74501	3.7745	4.70467	Adipose_R1_peak_	Distal Interger	12	74297474	74300468	2995	1	386753	uc007nwy.1	338454	ENSMUSG000	Dbpht2	DNA binding protein with his-thr domain
chr12	74640127	74640369	243	244	74640247	33	17.97508	6.11109	14.52164	Adipose_R1_peak_	Distal Interger	12	74297474	74300468	2995	1	386753	uc007nwy.1	342653	ENSMUSG000	Dbpht2	DNA binding protein with his-thr domain
chr12	74803861	74804427	567	568	74803948	107	32.27694	3.9783	28.53704	Adipose_R1_peak_	Distal Interger	12	74897217	75177332	280116	2	238271	uc007nwz.2	372905	ENSMUSG000	Kcnh5	potassium voltage-gated channel, subfamily H (eag-related), member 5
chr12	74803876	74804039	164	165	74803947	129	40.83431	4.20597	36.79406	Adipose_R2_peak_	Distal Interger	12	74897217	75177332	280116	2	238271	uc007nwz.2	373293	ENSMUSG000	Kcnh5	potassium voltage-gated channel, subfamily H (eag-related), member 5
chr12	74804143	74804428	286	287	74804174	89	15.50537	2.6329	12.01864	Adipose_R2_peak_	Distal Interger	12	74897217	75177332	280116	2	238271	uc007nwz.2	372904	ENSMUSG000	Kcnh5	potassium voltage-gated channel, subfamily H (eag-related), member 5
chr12	74858859	74859127	269	270	74858980	35	19.76029	6.47056	16.26028	Adipose_R1_peak_	Distal Interger	12	74897217	75177332	280116	2	238271	uc007nwz.2	318205	ENSMUSG000	Kcnh5	potassium voltage-gated channel, subfamily H (eag-related), member 5
chr12	74858873	74859061	189	190	74859003	36	10.5378	3.41206	7.27949	Adipose_R2_peak_	Distal Interger	12	74897217	75177332	280116	2	238271	uc007nwz.2	318271	ENSMUSG000	Kcnh5	potassium voltage-gated channel, subfamily H (eag-related), member 5
chr12	75242555	75242866	312	313	75242647	199	164.481	15.42564	159.9302	Adipose_R1_peak_	Distal Interger	12	74897217	75177332	280116	2	238271	uc007nwz.2	-65223	ENSMUSG000	Kcnh5	potassium voltage-gated channel, subfamily H (eag-related), member 5
chr12	75242556	75242861	306	307	75242633	164	135.9142	15.1758	131.2939	Adipose_R2_peak_	Distal Interger	12	74897217	75177332	280116	2	238271	uc007nwz.2	-65224	ENSMUSG000	Kcnh5	potassium voltage-gated channel, subfamily H (eag-related), member 5
chr12	77881905	77882071	167	168	77881967	23	9.86443	4.31371	6.70247	Adipose_R1_peak_	Distal Interger	12	78226655	78684769	458115	1	268566	uc007nzc.2	-344584	ENSMUSG000	Gphn	gephyrin
chr12	77881924	77882050	127	128	77881969	27	5.77405	2.5821	2.88921	Adipose_R2_peak_	Distal Interger	12	78226655	78684769	458115	1	268566	uc007nzc.2	-344605	ENSMUSG000	Gphn	gephyrin
chr12	78091205	78091440	236	237	78091307	32	17.10103	5.93135	13.67137	Adipose_R1_peak_	Distal Interger	12	78226655	78684769	458115	1	268566	uc007nzc.2	-135215	ENSMUSG000	Gphn	gephyrin
chr12	78350877	78351151	275	276	78351008	813	638.3223	15.13269	633.1586	Adipose_R2_peak_	Intron (uc007r	12	78226655	78684769	458115	1	268566	uc007nzc.2	124222	ENSMUSG000	Gphn	gephyrin
chr12	78350883	78351140	258	259	78351004	469	222.0514	6.82557	217.3863	Adipose_R1_peak_	Intron (uc007r	12	78226655	78684769	458115	1	268566	uc007nzc.2	124228	ENSMUSG000	Gphn	gephyrin
chr12	78372631	78372949	319	320	78372775	54	36.79449	9.1678	32.97463	Adipose_R1_peak_	Intron (uc007r	12	78226655	78684769	458115	1	268566	uc007nzc.2	145976	ENSMUSG000	Gphn	gephyrin
chr12	78372655	78372887	233	234	78372781	52	21.18119	4.88754	17.49655	Adipose_R2_peak_	Intron (uc007r	12	78226655	78684769	458115	1	268566	uc007nzc.2	146000	ENSMUSG000	Gphn	gephyrin
chr12	78430860	78431019	160	161	78430922	18	6.42934	3.41502	3.48999	Adipose_R1_peak_	Intron (uc007r	12	78226655	78684769	458115	1	268566	uc007nzc.2	204205	ENSMUSG000	Gphn	gephyrin
chr12	78462468	78462627	160	161	78462529	41	25.38991	7.54899	21.76305	Adipose_R1_peak_	Intron (uc007r	12	78691535	78734519	42985	1	70897	uc007nze.2	-228908	ENSMUSG000	Fam71d	family with sequence similarity 71, member D
chr12	78490480	78490623	144	145	78490558	20	7.74501	3.7745	4.70467	Adipose_R1_peak_	Intron (uc007r	12	78691535	78734519	42985	1	70897	uc007nze.2	-200912	ENSMUSG000	Fam71d	family with sequence similarity 71, member D
chr12	78653819	78654003	185	186	78653930	22	9.13959	4.13397	6.01526	Adipose_R1_peak_	Intron (uc007r	12	78691535	78734519	42985	1	70897	uc007nze.2	-37532	ENSMUSG000	Fam71d	family with sequence similarity 71, member D
chr12	79639517	79639884	368	369	79639650	31	16.23981	5.75161	12.83549	Adipose_R1_peak_	Intron (uc007c	12	79917019	79924733	7715	2	77290	uc007oak.1	284849	NA	9430078K24Ri	RIKEN cDNA 9430078K24 gene
chr12	79853238	79853368	131	132	79853283	26	5.31252	2.48988	2.47999	Adipose_R2_peak_	Distal Interger	12	79917019	79924733	7715	2	77290	uc007oak.1	71365	NA	9430078K24Ri	RIKEN cDNA 9430078K24 gene
chr12	79873510	79873668	159	160	79873574	19	7.07687	3.59476	4.08458	Adipose_R1_peak_	Distal Interger	12	79917019	79924733	7715	2	77290	uc007oak.1	51065	NA	9430078K24Ri	RIKEN cDNA 9430078K24 gene
chr12	79921948	79922098	151	152	79922079	19	7.07687	3.59476	4.08458	Adipose_R1_peak_	Promoter (2-3	12	79917019	79924733	7715	2	77290	uc007oak.1	2635	NA	9430078K24Ri	RIKEN cDNA 9430078K24 gene
chr12	80110591	80110712	122	123	80110674	26	5.31252	2.48988	2.47999	Adipose_R2_peak_	Promoter (2-3	12	80107760	80113013	5254	2	12192	uc007oal.1	2301	ENSMUSG000	Zfp36l1	zinc finger protein 36, C3H type-like 1
chr12	80112071	80112385	315	316	80112157	28	6.25023	2.67431	3.31626	Adipose_R2_peak_	Promoter (<=	12	80107760	80113013	5254	2	12192	uc007oal.1	628	ENSMUSG000	Zfp36l1	zinc finger protein 36, C3H type-like 1
chr12	80113183	80113579	397	398	80113218	19	7.07687	3.59476	4.08458	Adipose_R1_peak_	Promoter (<=	12	80107760	80113013	5254	2	12192	uc007oal.1	-170	ENSMUSG000	Zfp36l1	zinc finger protein 36, C3H type-like 1
chr12	80113685	80114044	360	361	80113751	29	6.74062	2.76653	3.75989	Adipose_R2_peak_	Promoter (<=	12	80107760	80113013	5254	2	12192	uc007oal.1	-672	ENSMUSG000	Zfp36l1	zinc finger protein 36, C3H type-like 1
chr12	80114054	80114507	454	455	80114147	18	6.42934	3.41502	3.48999	Adipose_R1_peak_	Promoter (1-2	12	80107760	80113013	5254	2	12192	uc007oal.1	-1041	ENSMUSG000	Zfp36l1	zinc finger protein 36, C3H type-like 1
chr12	80116411	80116804	394	395	80116496	36	10.5378	3.41206	7.27949	Adipose_R2_peak_	Distal Interger	12	80107760	80113013	5254	2	12192	uc007oal.1	-3398	ENSMUSG000	Zfp36l1	zinc finger protein 36, C3H type-like 1
chr12	80119386	80119508	123	124	80119456	31	7.7623	2.95097	4.69388	Adipose_R2_peak_	Downstream (	12	80107760	80113013	5254	2	12192	uc007oal.1	-6373	ENSMUSG000	Zfp36l1	zinc finger protein 36, C3H type-like 1
chr12	81393494	81393634	141	142	81393610	21	8.43286	3.95423	5.34896	Adipose_R1_peak_	Intron (uc007c	12	81376991	81379486	2496	2	214321	uc007ocb.1	-14008	ENSMUSG000	Gm4787	predicted gene 4787
chr12	81394663	81394948	286	287	81394820	41	13.59774	3.87315	10.1803	Adipose_R2_peak_	Intron (uc007c	12	81376991	81379486	2496	2	214321	uc007ocb.1	-15177	ENSMUSG000	Gm4787	predicted gene 4787
chr12	81394665	81394954	290	291	81394815	46	30.36364	8.44768	26.64488	Adipose_R1_peak_	Intron (uc007c	12	81376991	81379486	2496	2	214321	uc007ocb.1	-15179	ENSMUSG000	Gm4787	predicted gene 4787
chr12	81551421	81551544	124	125	81551465	28	6.25023	2.67431	3.31626	Adipose_R2_peak_	Distal Interger	12	81558584	81568474	9891	2	56622	uc007ock.1	16930	ENSMUSG000	Adam21	a disintegrin and metallopeptidase domain 21
chr12	81551433	81551563	131	132	81551496	18	6.42934	3.41502	3.48999	Adipose_R1_peak_	Distal Interger	12	81558584	81568474	9891	2	56622	uc007ock.1	16911	ENSMUSG000	Adam21	a disintegrin and metallopeptidase domain 21
chr12	81551991	81552226	236	237	81552103	30	15.39182	5.57188	12.01394	Adipose_R1_peak_	Distal Interger	12	81558584	81568474	9891	2	56622	uc007ock.1	16248	ENSMUSG000	Adam21	a disintegrin and metallopeptidase domain 21
chr12	82002467	82002751	285	286	82002584	35	7.95007	2.81421	4.87526	Adipose_R2_peak_	Distal Interger	12	81975797	82000924	25128	1	54604	uc011yon.1	26670	ENSMUSG000	Pcnx	pecanex homolog (Drosophila)
chr12	82002917	82003853	937	938	82002994	32	6.51559	2.57969	3.57128	Adipose_R2_peak_	Distal Interger	12	81975797	82000924	25128	1	54604	uc011yon.1	27120	ENSMUSG000	Pcnx	pecanex homolog (Drosophila)
chr12	82004587	82005674	1088	1089	82004776	45	14.93125	3.93321	11.45662	Adipose_R2_peak_	Distal Interger	12	81975797	82000924	25128	1	54604	uc011yon.1	28790	ENSMUSG000	Pcnx	pecanex homolog (Drosophila)
chr12	82004589	82004751	163	164	82004740	25	8.15817	3.46091	5.11339	Adipose_R1_peak_	Distal Interger	12	81975797	82000924	25128	1	54604	uc011yon.1	28792	ENSMUSG000	Pcnx	pecanex homolog (Drosophila)
chr12	82005793	82006040	248	249	82005877	32	8.29282	3.04319	5.18352	Adipose_R2_peak_	Distal Interger	12	81975797	82000924	25128	1	54604	uc011yon.1	29996	ENSMUSG000	Pcnx	pecanex homolog (Drosophila)
chr12	82006481	82006836	356	357	82006566	40	12.92494	3.77096	9.55198	Adipose_R2_peak_	Distal Interger	12	81975797	82000924	25128	1	54604	uc011yon.1	30684	ENSMUSG000	Pcnx	pecanex homolog (Drosophila)
chr12	82007023	82007200	178	179	82007137	35	9.95877	3.31984	6.73562	Adipose_R2_peak_	Distal Interger	12	81975797	82000924	25128	1	54604	uc011yon.1	31226	ENSMUSG000	Pcnx	pecanex homolog (Drosophila)
chr12	82007404	82007588	185	186	82007558	26	5.20594	2.45855	2.41767	Adipose_R2_peak_	Distal Interger	12	81975797	82000924	25128	1	54604	uc011yon.1	31607	ENSMUSG000	Pcnx	pecanex homolog (Drosophila)
chr12	82007701	82008012	312	313	82007811	38	11.63679	3.57286	8.32921	Adipose_R2_peak_	Distal Interger	12	81975797	82000924	25128	1	54604	uc011yon.1	31904	ENSMUSG000	Pcnx	pecanex homolog (Drosophila)
chr12	82008250	82008374	125	126	82008302	26	5.24186	2.4691	2.45226	Adipose_R2_peak_	Distal Interger	12	81975797	82000924	25128	1	54604	uc011yon.1	32453	ENSMUSG000	Pcnx	pecanex homolog (Drosophila)
chr12	82009122	82009225	104	105	82009176	28	5.24834	2.39443	2.45859	Adipose_R2_peak_	Distal Interger	12	81975797	82000924	25128	1	54604	uc011yon.1	33325	ENSMUSG000	Pcnx	pecanex homolog (Drosophila)
chr12	82009395	82010040	646	647	82009661	40	12.96465	3.78093	9.57654	Adipose_R2_peak_	Distal Interger	12	81975797	82000924	25128	1	54604	uc011yon.1	33598	ENSMUSG000	Pcnx	pecanex homolog (Drosophila)
chr12	82010243	82010671	429	430	82010373	31	7.7623	2.95097	4.69388	Adipose_R2_peak_	Distal Interger	12	81975797	82000924	25128	1	54604	uc011yon.1	34446	ENSMUSG000	Pcnx	pecanex homolog (Drosophila)
chr12	82011193	82011356	164	165	82011237	31	6.84348	2.70444	3.85909	Adipose_R2_peak_	Distal Interger	12	81975797	82000924	25128	1	54604	uc011yon.1	35396	ENSMUSG000	Pcnx	pecanex homolog (Drosophila)
chr12	82011541	82011723	183	184	82011600	35	7.23904	2.64415	4.21917	Adipose_R2_peak_	Distal Interger	12	81975797	82000924	25128	1	54604	uc011yon.1	35744	ENSMUSG000	Pcnx	pecanex homolog (Drosophila)
chr12	82011596	82011764	169	170	82011682	25	6.64199	2.95098	3.69964	Adipose_R1_peak_	Distal Interger	12	81975797	82000924	25128	1	54604	uc011yon.1	35799	ENSMUSG000	Pcnx	pecanex homolog (Drosophila)
chr12	82011915	82012017	103	104	82011954	32	5.69622	2.37595	2.84921	Adipose_R2_peak_	Distal Interger	12	81975797	82000924	25128	1	54604	uc011yon.1	36118	ENSMUSG000	Pcnx	pecanex homolog (Drosophila)
chr12	82012362	82012617	256	257	82012406	31	7.09206	2.77025	4.09791	Adipose_R2_peak_	Distal Interger	12	81975797	82000924	25128	1	54604	uc011yon.1	36565	ENSMUSG000	Pcnx	pecanex homolog (Drosophila)
chr12	82012991	82013147	157	158	82013078	32	6.09185	2.47363	3.19509	Adipose_R2_peak_	Distal Interger	12	81975797	82000924	25128	1	54604	uc011yon.1	37194	ENSMUSG000	Pcnx	pecanex homolog (Drosophila)
chr12	82014124	82014270	147	148	82014166	29	6.44017	2.68272	3.49901	Adipose_R2_peak_	Distal Interger	12	81975797	82000924	25128	1	54604	uc011yon.1	38327	ENSMUSG000	Pcnx	pecanex homolog (Drosophila)
chr12	82014397	82014598	202	203	82014568	29	6.45823	2.68773	3.51664	Adipose_R2_peak_	Distal Interger	12	81975797	82000924	25128	1	54604	uc011yon.1	38600	ENSMUSG000	Pcnx	pecanex homolog (Drosophila)
chr12	82014722	82015238	517	518	82015192	30	6.87815	2.75801	3.89228	Adipose_R2_peak_	Distal Interger	12	81975797	82000924	25128	1	54604	uc011yon.1	38925	ENSMUSG000	Pcnx	pecanex homolog (Drosophila)
chr12	82015618	82016179	562	563	82015722	33	8.34706	3.0061	5.2355	Adipose_R2_peak_	Distal Interger	12	81975797	82000924	25128	1	54604	uc011yon.1	39821	ENSMUSG000	Pcnx	pecanex homolog (Drosophila)
chr12	82016485	82016746	262	263	82016520	28	5.66324	2.50905	2.81749	Adipose_R2_peak_	Distal Interger	12	81975797	82000924	25128	1	54604	uc011yon.1	40688	ENSMUSG000	Pcnx	pecanex homolog (Drosophila)
chr12	82017010	82017236	227	228	82017150	36	7.36814	2.6379	4.33764	Adipose_R2_peak_	Distal Interger	12	81975797	82000924	25128	1	54604	uc011yon.1	41213	ENSMUSG000	Pcnx	pecanex homolog (Drosophila)
chr12	82017620	82018070	451	452	82017699	35	7.12721	2.61779	4.13155	Adipose_R2_peak_	Distal Interger	12	81975797	82000924	25128	1	54604	uc011yon.1	41823	ENSMUSG000	Pcnx	pecanex homolog (Drosophila)
chr12	82018550	82018650	101	102	82018632	29	6.18321	2.61183	3.28282	Adipose_R2_peak_	Distal Interger	12	81975797	82000924	25128	1	54604	uc011yon.1	42753	ENSMUSG000	Pcnx	pecanex homolog (Drosophila)
chr12	82018832	82019255	424	425	82019066	27	5.19297	2.41579	2.40527	Adipose_R2_peak_	Distal Interger	12	81975797	82000924	25128	1	54604	uc011yon.1	43035	ENSMUSG000	Pcnx	pecanex homolog (Drosophila)

chr12	82019531	82020556	1026	1027	82020050	37	9.23819	3.03563	6.07441	Adipose_R2_peak_	Distal Intergen	12	81975797	82000924	25128	1	54604	uc011yon.1	43734	ENSMUSG000	Pcnx	pecanex homolog (Drosophila)
chr12	82020683	82020889	207	208	82020773	26	4.95681	2.38582	2.17761	Adipose_R2_peak_	Distal Intergen	12	81975797	82000924	25128	1	54604	uc011yon.1	44886	ENSMUSG000	Pcnx	pecanex homolog (Drosophila)
chr12	82021255	82021710	456	457	82021452	31	5.56081	2.37427	2.71889	Adipose_R2_peak_	Distal Intergen	12	81975797	82000924	25128	1	54604	uc011yon.1	45458	ENSMUSG000	Pcnx	pecanex homolog (Drosophila)
chr12	82022079	82022290	212	213	82022105	29	6.47128	2.69135	3.52879	Adipose_R2_peak_	Distal Intergen	12	81975797	82000924	25128	1	54604	uc011yon.1	46282	ENSMUSG000	Pcnx	pecanex homolog (Drosophila)
chr12	82022562	82022817	256	257	82022696	28	6.25023	2.67431	3.31626	Adipose_R2_peak_	Distal Intergen	12	81975797	82000924	25128	1	54604	uc011yon.1	46765	ENSMUSG000	Pcnx	pecanex homolog (Drosophila)
chr12	82023846	82024470	625	626	82023891	31	7.7623	2.95097	4.69388	Adipose_R2_peak_	Distal Intergen	12	81975797	82000924	25128	1	54604	uc011yon.1	48049	ENSMUSG000	Pcnx	pecanex homolog (Drosophila)
chr12	82024801	82024988	188	189	82024865	31	6.72563	2.67346	3.75989	Adipose_R2_peak_	Distal Intergen	12	81975797	82000924	25128	1	54604	uc011yon.1	49004	ENSMUSG000	Pcnx	pecanex homolog (Drosophila)
chr12	82025304	82025578	275	276	82025357	31	6.27528	2.55632	3.34018	Adipose_R2_peak_	Distal Intergen	12	81975797	82000924	25128	1	54604	uc011yon.1	49507	ENSMUSG000	Pcnx	pecanex homolog (Drosophila)
chr12	82026822	82027062	241	242	82026973	29	6.74062	2.76653	3.75989	Adipose_R2_peak_	Distal Intergen	12	81975797	82000924	25128	1	54604	uc011yon.1	51025	ENSMUSG000	Pcnx	pecanex homolog (Drosophila)
chr12	82027877	82028211	335	336	82028011	30	7.24477	2.85875	4.21917	Adipose_R2_peak_	Distal Intergen	12	81975797	82000924	25128	1	54604	uc011yon.1	52080	ENSMUSG000	Pcnx	pecanex homolog (Drosophila)
chr12	82029019	82029812	794	795	82029130	38	11.72962	3.59649	8.4047	Adipose_R2_peak_	Distal Intergen	12	81975797	82000924	25128	1	54604	uc011yon.1	53222	ENSMUSG000	Pcnx	pecanex homolog (Drosophila)
chr12	82030026	82030262	237	238	82030130	35	9.01814	3.07816	5.86231	Adipose_R2_peak_	Distal Intergen	12	81975797	82000924	25128	1	54604	uc011yon.1	54229	ENSMUSG000	Pcnx	pecanex homolog (Drosophila)
chr12	82031289	82031382	94	95	82031321	28	6.25023	2.67431	3.31626	Adipose_R2_peak_	Distal Intergen	12	81975797	82000924	25128	1	54604	uc011yon.1	55492	ENSMUSG000	Pcnx	pecanex homolog (Drosophila)
chr12	82031522	82031810	289	290	82031727	36	10.5378	3.41206	7.27949	Adipose_R2_peak_	Distal Intergen	12	81975797	82000924	25128	1	54604	uc011yon.1	55725	ENSMUSG000	Pcnx	pecanex homolog (Drosophila)
chr12	82634832	82635088	257	258	82634952	45	29.34988	8.26794	25.64927	Adipose_R1_peak_	Intron (uc007c	12	82617499	83162056	544558	1	50779	uc007odb.3	17333	ENSMUSG000	Rgs6	regulator of G-protein signaling 6
chr12	82634856	82635068	213	214	82634977	41	13.59774	3.87315	10.1803	Adipose_R2_peak_	Intron (uc007c	12	82617499	83162056	544558	1	50779	uc007odb.3	17357	ENSMUSG000	Rgs6	regulator of G-protein signaling 6
chr12	84737176	84737450	275	276	84737309	75	60.30062	12.66824	56.22078	Adipose_R1_peak_	Distal Intergen	12	84754559	84773112	18554	2	67963	uc007oft.1	35662	ENSMUSG000	Npc2	Niemann-Pick type C2
chr12	84737180	84737440	261	262	84737300	66	32.22715	6.17859	28.31407	Adipose_R2_peak_	Distal Intergen	12	84754559	84773112	18554	2	67963	uc007oft.1	35672	ENSMUSG000	Npc2	Niemann-Pick type C2
chr12	85283386	85283599	214	215	85283407	16	5.20017	3.05554	2.38734	Adipose_R1_peak_	Promoter (2-3	12	85272398	85280435	8038	2	66204	uc007ogu.2	-2951	ENSMUSG000	Acyp1	acylphosphatase 1, erythrocyte (common) type
chr12	85283491	85283595	105	106	85283521	25	4.86612	2.39766	2.09018	Adipose_R2_peak_	Distal Intergen	12	85272398	85280435	8038	2	66204	uc007ogu.2	-3056	ENSMUSG000	Acyp1	acylphosphatase 1, erythrocyte (common) type
chr12	85459499	85459749	251	252	85459610	18	6.42934	3.41502	3.48999	Adipose_R1_peak_	Distal Intergen	12	85473901	85477270	3370	1	14281	uc007oha.2	-14152	ENSMUSG000	Fos	FBJ osteosarcoma oncogene
chr12	85468168	85468423	256	257	85468360	31	7.7623	2.95097	4.69388	Adipose_R2_peak_	Distal Intergen	12	85473901	85477270	3370	1	14281	uc007oha.2	-5478	ENSMUSG000	Fos	FBJ osteosarcoma oncogene
chr12	85469967	85470090	124	125	85470033	31	7.7623	2.95097	4.69388	Adipose_R2_peak_	Distal Intergen	12	85473901	85477270	3370	1	14281	uc007oha.2	-3811	ENSMUSG000	Fos	FBJ osteosarcoma oncogene
chr12	85472478	85472741	264	265	85472633	28	6.25023	2.67431	3.31626	Adipose_R2_peak_	Promoter (1-2	12	85473901	85477270	3370	1	14281	uc007oha.2	-1160	ENSMUSG000	Fos	FBJ osteosarcoma oncogene
chr12	85475669	85475853	185	186	85475778	28	6.25023	2.67431	3.31626	Adipose_R2_peak_	Promoter (1-2	12	85473901	85477270	3370	1	14281	uc007oha.2	1768	ENSMUSG000	Fos	FBJ osteosarcoma oncogene
chr12	85478795	85479078	284	285	85478992	35	9.95877	3.31984	6.73562	Adipose_R2_peak_	Distal Intergen	12	85473901	85477270	3370	1	14281	uc007oha.2	4894	ENSMUSG000	Fos	FBJ osteosarcoma oncogene
chr12	85480763	85481129	367	368	85480835	31	7.7623	2.95097	4.69388	Adipose_R2_peak_	Distal Intergen	12	85473901	85477270	3370	1	14281	uc007oha.2	6862	ENSMUSG000	Fos	FBJ osteosarcoma oncogene
chr12	85482690	85482797	108	109	85482781	26	5.31252	2.48988	2.47999	Adipose_R2_peak_	Distal Intergen	12	85473901	85477270	3370	1	14281	uc007oha.2	8789	ENSMUSG000	Fos	FBJ osteosarcoma oncogene
chr12	85484370	85484465	96	97	85484415	27	5.77405	2.5821	2.88921	Adipose_R2_peak_	Distal Intergen	12	85473901	85477270	3370	1	14281	uc007oha.2	10469	ENSMUSG000	Fos	FBJ osteosarcoma oncogene
chr12	85491412	85491540	129	130	85491471	29	6.74062	2.76653	3.75989	Adipose_R2_peak_	Distal Intergen	12	85473901	85477270	3370	1	14281	uc007oha.2	17511	ENSMUSG000	Fos	FBJ osteosarcoma oncogene
chr12	85733181	85733374	194	195	85733288	126	131.7124	22.82671	127.2552	Adipose_R1_peak_	Distal Intergen	12	85746539	85813585	67047	1	217721	uc007ohe.1	-13165	ENSMUSG000	Mfsd7c	major facilitator superfamily domain containing 7C
chr12	85733195	85733374	180	181	85733306	110	74.23988	10.23617	69.8667	Adipose_R2_peak_	Distal Intergen	12	85746539	85813585	67047	1	217721	uc007ohe.1	-13165	ENSMUSG000	Mfsd7c	major facilitator superfamily domain containing 7C
chr12	86828037	86828238	202	203	86828168	18	6.42934	3.41502	3.48999	Adipose_R1_peak_	Distal Intergen	12	86880703	86884814	4112	2	238330	uc007oia.2	56576	ENSMUSG000	Irf2bpl	interferon regulatory factor 2 binding protein-like
chr12	86828431	86828566	136	137	86828449	28	6.25023	2.67431	3.31626	Adipose_R2_peak_	Distal Intergen	12	86880703	86884814	4112	2	238330	uc007oia.2	56248	ENSMUSG000	Irf2bpl	interferon regulatory factor 2 binding protein-like
chr12	86834082	86834212	131	132	86834144	19	7.07687	3.59476	4.08458	Adipose_R1_peak_	Distal Intergen	12	86880703	86884814	4112	2	238330	uc007oia.2	50602	ENSMUSG000	Irf2bpl	interferon regulatory factor 2 binding protein-like
chr12	86885657	86886111	455	456	86885791	19	7.07687	3.59476	4.08458	Adipose_R1_peak_	Promoter (<=	12	86880703	86884814	4112	2	238330	uc007oia.2	-843	ENSMUSG000	Irf2bpl	interferon regulatory factor 2 binding protein-like
chr12	86886296	86886490	195	196	86886401	19	7.07687	3.59476	4.08458	Adipose_R1_peak_	Promoter (1-2	12	86880703	86884814	4112	2	238330	uc007oia.2	-1482	ENSMUSG000	Irf2bpl	interferon regulatory factor 2 binding protein-like
chr12	86886583	86886693	111	112	86886643	29	6.74062	2.76653	3.75989	Adipose_R2_peak_	Promoter (1-2	12	86880703	86884814	4112	2	238330	uc007oia.2	-1769	ENSMUSG000	Irf2bpl	interferon regulatory factor 2 binding protein-like
chr12	86886773	86887055	283	284	86886855	18	6.42934	3.41502	3.48999	Adipose_R1_peak_	Promoter (1-2	12	86880703	86884814	4112	2	238330	uc007oia.2	-1959	ENSMUSG000	Irf2bpl	interferon regulatory factor 2 binding protein-like
chr12	86886938	86887122	185	186	86886953	27	5.77405	2.5821	2.88921	Adipose_R2_peak_	Promoter (2-3	12	86880703	86884814	4112	2	238330	uc007oia.2	-2124	ENSMUSG000	Irf2bpl	interferon regulatory factor 2 binding protein-like
chr12	86887661	86888018	358	359	86887822	20	7.74501	3.7745	4.70467	Adipose_R1_peak_	Promoter (2-3	12	86880703	86884814	4112	2	238330	uc007oia.2	-2847	ENSMUSG000	Irf2bpl	interferon regulatory factor 2 binding protein-like
chr12	86887916	86888117	202	203	86888039	30	7.24477	2.85875	4.21917	Adipose_R2_peak_	Distal Intergen	12	86880703	86884814	4112	2	238330	uc007oia.2	-3102	ENSMUSG000	Irf2bpl	interferon regulatory factor 2 binding protein-like
chr12	86889004	86889241	238	239	86889066	17	5.80341	3.23528	2.92378	Adipose_R1_peak_	Distal Intergen	12	86880703	86884814	4112	2	238330	uc007oia.2	-4190	ENSMUSG000	Irf2bpl	interferon regulatory factor 2 binding protein-like
chr12	86889531	86890240	710	711	86889757	37	11.12817	3.50427	7.83613	Adipose_R2_peak_	Distal Intergen	12	86880703	86884814	4112	2	238330	uc007oia.2	-4717	ENSMUSG000	Irf2bpl	interferon regulatory factor 2 binding protein-like
chr12	86890372	86890537	166	167	86890404	26	5.31252	2.48988	2.47999	Adipose_R2_peak_	Distal Intergen	12	86880703	86884814	4112	2	238330	uc007oia.2	-5558	ENSMUSG000	Irf2bpl	interferon regulatory factor 2 binding protein-like
chr12	86891676	86891882	207	208	86891727	16	5.20017	3.05554	2.38734	Adipose_R1_peak_	Distal Intergen	12	86880703	86884814	4112	2	238330	uc007oia.2	-6862	ENSMUSG000	Irf2bpl	interferon regulatory factor 2 binding protein-like
chr12	86893717	86893922	206	207	86893833	36	10.5378	3.41206	7.27949	Adipose_R2_peak_	Distal Intergen	12	86880703	86884814	4112	2	238330	uc007oia.2	-8903	ENSMUSG000	Irf2bpl	interferon regulatory factor 2 binding protein-like
chr12	87534209	87534599	391	392	87534437	46	30.36364	8.44768	26.64488	Adipose_R1_peak_	Distal Intergen	12	87514316	87518265	3950	1	666806	uc011ypn.1	19893	ENSMUSG000	Gm8300	predicted gene 8300
chr12	87534228	87534543	316	317	87534405	39	12.34187	3.68871	8.98501	Adipose_R2_peak_	Distal Intergen	12	87514316	87518265	3950	1	666806	uc011ypn.1	19912	ENSMUSG000	Gm8300	predicted gene 8300
chr12	87602644	87602750	107	108	87602688	28	6.25023	2.67431	3.31626	Adipose_R2_peak_	Promoter (<=	12	87602665	87608850	6186	1	1.00E+08	uc007ojh.3	0	ENSMUSG000	Gm2042	predicted gene 2042
chr12	87606949	87607204	256	257	87607071	27	5.53046	2.51193	2.68965	Adipose_R2_peak_	Exon (uc007oj	12	87602665	87608850	6186	1	1.00E+08	uc007ojh.3	4284	ENSMUSG000	Gm2042	predicted gene 2042
chr12	87977866	87978146	281	282	87978005	53	37.70483	9.70585	33.87	Adipose_R1_peak_	Distal Intergen	12	87954480	87960682	6203	1	1.00E+08	uc007ojo.4	23386	ENSMUSG000	Gm2042	predicted gene 2042
chr12	87977880	87978103	224	225	87978009	51	20.44827	4.79532	16.78434	Adipose_R2_peak_	Distal Intergen	12	87954480	87960682	6203	1	1.00E+08	uc007ojo.4	23400	ENSMUSG000	Gm2042	predicted gene 2042
chr12	88057414	88057685	272	273	88057537	35	9.95877	3.31984	6.73562	Adipose_R2_peak_	Intron (uc007c	12	87954480	87960682	6203	1	1.00E+08	uc007ojo.4	102934	ENSMUSG000	Gm2042	predicted gene 2042
chr12	88685293	88685466	174	175	88685402	21	8.43286	3.95423	5.34896	Adipose_R1_peak_	Distal Intergen	12	88722876	88850849	127974	1	18191	uc007ojy.1	-37410	ENSMUSG000	Nrxn3	neurexin III
chr12	88687320	88687581	262	263	88687343	16	5.20017	3.05554	2.38734	Adipose_R1_peak_	Distal Intergen	12	88722876	88850849	127974	1	18191	uc007ojy.1	-35295	ENSMUSG000	Nrxn3	neurexin III
chr12	88906786	88906933	148	149	88906846	18	6.42934	3.41502	3.48999	Adipose_R1_peak_	Intron (uc007c	12	88953390	90334933	1381544	1	18191	uc007okc.2	-46457	ENSMUSG000	Nrxn3	neurexin III
chr12	89247240	89247492	253	254	89247373	36	20.67077	6.6503	17.14779	Adipose_R1_peak_	Intron (uc007c	12	88953390	90334933	1381544	1	18191	uc007okc.2	293850	ENSMUSG000	Nrxn3	neurexin III
chr12	89247279	89247413	135	136	89247356	29	6.74062	2.76653	3.75989	Adipose_R2_peak_	Intron (uc007c	12	88953390	90334933	1381544	1	18191	uc007okc.2	293889	ENSMUSG000	Nrxn3	neurexin III
chr12	89396758	89396909	152	153	89396854	26	5.31252	2.48988	2.47999	Adipose_R2_peak_	Intron (uc007c	12	89812483	90334933	522451	1	18191	uc007oke.3	-415574	ENSMUSG000	Nrxn3	neurexin III
chr12	89450203	89450404	202	203	89450307	23	9.86443	4.31371	6.70247	Adipose_R1_peak_	Intron (uc007c	12	89812483	90334933	522451	1	18191	uc007oke.3	-362079	ENSMUSG000	Nrxn3	neurexin III
chr12	89599197	89599437	241	242	89599277	31	16.23981	5.75161	12.83549	Adipose_R1_peak_	Intron (uc007c	12	89812483	90334933	522451	1	18191	uc007oke.3	-213046	ENSMUSG000	Nrxn3	neurexin III
chr12	89599210	89599412	203	204	89599271	42	14.2409	3.96536	10.79479	Adipose_R2_peak_	Intron (uc007c	12	89812483	90334933	522451	1	18191	uc007oke.3	-213071	ENSMUSG000	Nrxn3	neurexin III
chr12	89601214	89601425	212	213	89601283	22	9.13959	4.13397	6.01526	Adipose_R1_peak_	Intron (uc007c	12	89812483	90334933	522451	1	18191	uc007oke.3	-211058	ENSMUSG000	Nrxn3	neurexin III
chr12	90020551	90020712	162	163	90020650	22	9.13959	4.13397	6.01526	Adipose_R1_peak_	Intron (uc007c	12	89812483	90334933	522451	1	18191	uc007oke.3	208068	ENSMUSG000	Nrxn3	neurexin III
chr12	90479597	90479804	208	209	90479714	35	19.76029	6.47056	16.26028	Adipose_R1_peak_	Distal Intergen	12	90724552	90738438	13887	2	13371	uc007okf.1	258634	ENSMUSG000	Dio2	deiodinase, iodothyronine, type II
chr12	90796908	90797058	151	152	90796994	40	24.42476	7.36925	20.81738	Adipose_R1_peak_	Intron (uc007c	12	90724552	90738438	13887	2	13371	uc007okf.1	-58470	ENSMUSG000	Dio2	deiodinase, iodothyronine, type II
chr12	90796949	90797055	107	108	90797003	33	8.83596	3.1354	5.68652	Adipose_R2_peak_	Intron (uc007c	12	90724552	90738438	13887	2	13371	uc007okf.1	-58511	ENSMUSG000	Dio2	deiodinase, iodothyronine, type II
chr12	90815855	90816092	238	239	90815951	34	18.86162	6.29083	15.38482	Adipose_R1_peak_	Intron (uc007c	12	90724552	90738438	13887	2	13371	uc007okf.1	-77417	ENSMUSG000	Dio2	deiodinase, iodothyronine, type II
chr12	91331011	91331580	570	571	91331230	51	35.56531	9.34637	31.76235	Adipose_R1_peak_	Intron (uc007c	12	90998492	91384409	385918	2	75216	uc007okl.2	52829	ENSMUSG000	Cep128	centrosomal protein 128
chr12	91331175	91331766	592	593	91331232	29	6.74062	2.76653	3.75989	Adipose_R2_peak_	Intron (uc007c	12	90998492	91384409	385918	2	75216	uc007okl.2	52643	ENSMUSG000	Cep128	centrosomal protein 128
chr12	91473221	91474089	869	870	91473366	39	23.47001	7.18952	19.88325	Adipose_R1_peak_	Intron (uc007c	12	91400993	91522541	121549	1	22095	uc007okp.2	72228	ENSMUSG000	Tshr	thyroid stimulating hormone receptor
chr12	91473261	91473427	167	168	91473351	38	11.72962	3.59649	8.4047	Adipose_R2_peak_	Intron (uc007c	12	91400993	91522541	121549	1	22095	uc007okp.2	72268	ENSMUSG000	Tshr	thyroid stimulating hormone receptor
chr12	91473656	91473974	319	320	91473789	47	17.60017	4.42645	14.02531	Adipose_R2_peak_	Intron (uc007c	12	91400993	91522541	121549	1	22095	uc007okp.2	72663	ENSMUSG000	Tshr	thyroid stimulating hormone receptor
chr12	91936596	91936755	160	161	91936689	35	9.95877	3.31984	6.73562	Adipose_R2_peak_	Distal Intergen	12	91806043	91849157	43115	2	20338	uc007okx.1	-87439	ENSMUSG000	Sel1l	sel-1 suppressor of lin-12-like (C. elegans)
chr12	91936630	91936776	147	148	91936696	23	9.86443	4.31371	6.70247	Adipose_R1_peak_	Distal Intergen	12	91806043	91849157	43115	2	20338	uc007okx.1	-87473	ENSMUSG000	Sel1l	sel-1 suppressor of lin-12-like (C. elegans)
chr12	92155685	92155964	280	281	92155710	19	7.07687	3.59476	4.08458	Adipose_R1_peak_	Distal Intergen	12	91806043	91849157	43115	2	20338	uc007okx.1	-306528	ENSMUSG000	Sel1l	sel-1 suppressor of lin-12-like (C. elegans)
chr12	92210083	92210323	241	242	92210203	40	24.42476	7.36925	20.81738	Adipose_R1_peak_	Distal Intergen	12	91806043	91849157	43115	2	20338	uc007okx.1	-360926	ENSMUSG000	Sel1l	sel-1 suppressor of lin-12-like (C. elegans)
chr12	92394130	92394448	319	320	92394239	78	66.82238	14.19929	62.68234	Adipose_R1_peak_	Distal Intergen	12	91806043	91849157	43115	2	20338	uc007okx.1	-544973	ENSMUSG000	Sel1l	sel-1 suppressor of lin-12-like (C. elegans)
chr12	92585725	92585939	215	216	92585833	29	14.55748	5.39214	11.20652	Adipose_R1_peak_	Distal Intergen	12	91806043	91849157	43115	2	20338	uc007okx.1	-736568	ENSMUSG000	Sel1l	sel-1 suppressor of lin-12-like (C. elegans)
chr12	92585773	92585900	128	129	92585834	28	6.25023	2.67431	3.31626	Adipose_R2_peak_	Distal Intergen	12	91806043	91849157	43115	2	20338	uc007okx.1	-736616	ENSMUSG000	Sel1l	sel-1 suppressor of lin-12-like (C. elegans)
chr12	92594044	92594208	165	166	92594122	24	10.60668	4.49345	7.40961	Adipose_R1_peak_	Distal Intergen	12	91806043	91849157	43115	2	20338	uc007okx.1	-744887	ENSMUSG000	Sel1l	sel-1 suppressor of lin-12-like (C. elegans)
chr12	92652344	92652476	133	134	92652415	20	7.74501	3.7745	4.70467	Adipose_R1_peak_	Distal Intergen	12	93416457	93444823	28367	2	75295	uc056yrw.1	792347	NA	4930559C10R	RIKEN cDNA 4930559C10 gene
chr12	92862167	92862674	508	509	92862239	21	8.43286	3.95423	5.34896	Adipose_R1_peak_	Distal Intergen	12	93416457	93444823	28367	2	75295	uc056yrw.1	582149	NA	4930559C10R	RIKEN cDNA 4930559C10 gene
chr12	92862425	92862632	208	209	92862547	44	15.55653	4.1498	12.05625	Adipose_R2_peak_	Distal Intergen	12	93416457	93444823	28367	2	75295	uc056yrw.1	582191	NA	4930559C10R	RIKEN cDNA 4930559C10 gene
chr12	93025740	93025929	190	191	93025820	22	9.13959	4.13397	6.01526	Adipose_R1_peak_	Distal Intergen	12	93416457	93444823	28367	2	75295	uc056yrw.1	418894	NA	4930559C10R	RIKEN cDNA 4930559C10 gene
chr12	93233986	93234244	259	260	93234114	52	36.63102	9.52611	32.81188	Adipose_R1_peak_	Distal Intergen	12	93416457	93444823	28367	2	75295	uc056yrw.1	210579	NA	4930559C10R	RIKEN cDNA 4930559C10 gene
chr12	93234008	93234192	185	186	93234119	37	11.12817	3.50427	7.83613	Adipose_R2_peak_	Distal Intergen	12	93416457	93444823	28367	2	75295	uc056yrw.1	210631	NA	4930559C10R	RIKEN cDNA 4930559C10 gene
chr12	93238400	93238553	154	155	93238464	19	7.07687	3.59476	4.08458	Adipose_R1_peak_	Distal Intergen	12	93416457	93444823	28367	2	75295	uc056yrw.1	206270	NA	4930559C10R	RIKEN cDNA 4930559C10 gene
chr12	93317556	93317774	219	220	93317637	31	16.23981	5.75161	12.83549	Adipose_R1_peak_	Distal Intergen	12	93416457	93444823	28367	2	75295	uc056yrw.1	127049	NA	4930559C10R	RIKEN cDNA 4930559C10 gene
chr12	93318266	93318496	231	232	93318373	33	17.97508	6.11109	14.52164	Adipose_R1_peak_	Distal Intergen	12	93416457	93444823	28367	2	75295	uc056yrw.1	126327	NA	4930559C10R	RIKEN cDNA 4930559C10 gene
chr12	93360501	93360639	139	140	93360594	21	8.43286	3.95423	5.34896	Adipose_R1_peak_	Distal Intergen	12	93416457	93444823	28367	2	75295	uc056yrw.1	84184	NA	4930559C10R	RIKEN cDNA 4930559C10 gene
chr12	93576988	93577251	264	265	93577094	300	314.2484	26.19615	309.4803	Adipose_R1_peak_	Distal Intergen	12	93416457	93444823	28367	2	75295	uc056yrw.1	-132165	NA	4930559C10R	RIKEN cDNA 4930559C10 gene
chr12	93576992	93577249	258	259	93577101	195	175.2979	18.07468	170.5963	Adipose_R2_peak_	Distal Intergen	12	93416457	93444823	28367	2	75295	uc056yrw.1	-132169	NA	4930559C10R	RIKEN cDNA 4930559C10 gene

chr12	93588975	93589264	290	291	93589118	53	37.70483	9.70585	33.87	Adipose_R1_peak_	Distal Intergen	12	93416457	93444823	28367	2	75295	uc056yrw.1	-144152	NA	4930559C10Ri	RIKEN cDNA 4930559C10 gene
chr12	93588999	93589234	236	237	93589126	49	19.00719	4.61089	15.38598	Adipose_R2_peak_	Distal Intergen	12	93416457	93444823	28367	2	75295	uc056yrw.1	-144176	NA	4930559C10Ri	RIKEN cDNA 4930559C10 gene
chr12	94036929	94037110	182	183	94037034	27	12.93148	5.03266	9.63827	Adipose_R1_peak_	Distal Intergen	12	93927338	93927464	127	1	1.02E+08	uc033ges.1	109591	ENSMUSG000	Mir8099-1	microRNA 8099-1
chr12	94079627	94079903	277	278	94079750	65	51.17258	11.8627	47.17609	Adipose_R1_peak_	Distal Intergen	12	93927338	93927464	127	1	1.02E+08	uc033ges.1	152289	ENSMUSG000	Mir8099-1	microRNA 8099-1
chr12	94079632	94079839	208	209	94079748	53	21.92215	4.97976	18.21755	Adipose_R2_peak_	Distal Intergen	12	93927338	93927464	127	1	1.02E+08	uc033ges.1	152294	ENSMUSG000	Mir8099-1	microRNA 8099-1
chr12	94124485	94124653	169	170	94124599	19	6.91816	3.52644	3.97188	Adipose_R1_peak_	Distal Intergen	12	93927338	93927464	127	1	1.02E+08	uc033ges.1	197147	ENSMUSG000	Mir8099-1	microRNA 8099-1
chr12	94125463	94125692	230	231	94125577	29	14.55748	5.39214	11.20652	Adipose_R1_peak_	Distal Intergen	12	93927338	93927464	127	1	1.02E+08	uc033ges.1	198125	ENSMUSG000	Mir8099-1	microRNA 8099-1
chr12	94125502	94125646	145	146	94125565	32	8.29282	3.04319	5.18352	Adipose_R2_peak_	Distal Intergen	12	93927338	93927464	127	1	1.02E+08	uc033ges.1	198164	ENSMUSG000	Mir8099-1	microRNA 8099-1
chr12	94209110	94209301	192	193	94209217	26	12.14079	4.85292	8.87838	Adipose_R1_peak_	Distal Intergen	12	93927338	93927464	127	1	1.02E+08	uc033ges.1	281772	ENSMUSG000	Mir8099-1	microRNA 8099-1
chr12	94209155	94209277	123	124	94209232	26	5.31252	2.48988	2.47999	Adipose_R2_peak_	Distal Intergen	12	93927338	93927464	127	1	1.02E+08	uc033ges.1	281817	ENSMUSG000	Mir8099-1	microRNA 8099-1
chr12	94269326	94269636	311	312	94269495	55	39.87613	10.06532	36.01168	Adipose_R1_peak_	Distal Intergen	12	93927338	93927464	127	1	1.02E+08	uc033ges.1	341988	ENSMUSG000	Mir8099-1	microRNA 8099-1
chr12	94269365	94269593	229	230	94269497	64	30.56565	5.99415	26.6807	Adipose_R2_peak_	Distal Intergen	12	93927338	93927464	127	1	1.02E+08	uc033ges.1	342027	ENSMUSG000	Mir8099-1	microRNA 8099-1
chr12	94555569	94555892	324	325	94555719	76	56.76315	11.2923	52.71642	Adipose_R1_peak_	Distal Intergen	12	93927338	93927464	127	1	1.02E+08	uc033ges.1	628231	ENSMUSG000	Mir8099-1	microRNA 8099-1
chr12	94555570	94555872	303	304	94555707	98	61.87664	9.12956	57.60231	Adipose_R2_peak_	Distal Intergen	12	93927338	93927464	127	1	1.02E+08	uc033ges.1	628232	ENSMUSG000	Mir8099-1	microRNA 8099-1
chr12	94575810	94575951	142	143	94575883	32	8.29282	3.04319	5.18352	Adipose_R2_peak_	Distal Intergen	12	93927338	93927464	127	1	1.02E+08	uc033ges.1	648472	ENSMUSG000	Mir8099-1	microRNA 8099-1
chr12	94575825	94576120	296	297	94575890	24	10.60668	4.49345	7.40961	Adipose_R1_peak_	Distal Intergen	12	93927338	93927464	127	1	1.02E+08	uc033ges.1	648487	ENSMUSG000	Mir8099-1	microRNA 8099-1
chr12	94816228	94816361	134	135	94816289	20	7.74501	3.7745	4.70467	Adipose_R1_peak_	Distal Intergen	12	95692226	95785213	92988	1	399558	uc007olc.2	-875865	ENSMUSG000	Flrt2	fibronectin leucine rich transmembrane protein 2
chr12	94990638	94990910	273	274	94990770	39	23.47001	7.18952	19.88325	Adipose_R1_peak_	Distal Intergen	12	95692226	95785213	92988	1	399558	uc007olc.2	-701316	ENSMUSG000	Flrt2	fibronectin leucine rich transmembrane protein 2
chr12	95260463	95260619	157	158	95260525	28	6.25023	2.67431	3.31626	Adipose_R2_peak_	Distal Intergen	12	95692226	95785213	92988	1	399558	uc007olc.2	-431607	ENSMUSG000	Flrt2	fibronectin leucine rich transmembrane protein 2
chr12	95436503	95436630	128	129	95436552	32	8.29282	3.04319	5.18352	Adipose_R2_peak_	Distal Intergen	12	95692226	95785213	92988	1	399558	uc007olc.2	-255596	ENSMUSG000	Flrt2	fibronectin leucine rich transmembrane protein 2
chr12	95436783	95436998	216	217	95436914	75	63.12309	13.66008	59.01505	Adipose_R1_peak_	Distal Intergen	12	95692226	95785213	92988	1	399558	uc007olc.2	-255228	ENSMUSG000	Flrt2	fibronectin leucine rich transmembrane protein 2
chr12	95436798	95436984	187	188	95436922	48	18.29934	4.51867	14.70076	Adipose_R2_peak_	Distal Intergen	12	95692226	95785213	92988	1	399558	uc007olc.2	-255242	ENSMUSG000	Flrt2	fibronectin leucine rich transmembrane protein 2
chr12	95579569	95579719	151	152	95579647	19	7.07687	3.59476	4.08458	Adipose_R1_peak_	Distal Intergen	12	95692226	95785213	92988	1	399558	uc007olc.2	-112507	ENSMUSG000	Flrt2	fibronectin leucine rich transmembrane protein 2
chr12	95612962	95613454	493	494	95613078	24	10.60668	4.49345	7.40961	Adipose_R1_peak_	Distal Intergen	12	95692226	95785213	92988	1	399558	uc007olc.2	-78772	ENSMUSG000	Flrt2	fibronectin leucine rich transmembrane protein 2
chr12	95613213	95613400	188	189	95613310	41	13.59774	3.87315	10.1803	Adipose_R2_peak_	Distal Intergen	12	95692226	95785213	92988	1	399558	uc007olc.2	-78826	ENSMUSG000	Flrt2	fibronectin leucine rich transmembrane protein 2
chr12	95647152	95647324	173	174	95647244	23	9.86443	4.31371	6.70247	Adipose_R1_peak_	Distal Intergen	12	95692226	95785213	92988	1	399558	uc007olc.2	-44902	ENSMUSG000	Flrt2	fibronectin leucine rich transmembrane protein 2
chr12	95647196	95647302	107	108	95647272	27	5.77405	2.5821	2.88921	Adipose_R2_peak_	Distal Intergen	12	95692226	95785213	92988	1	399558	uc007olc.2	-44924	ENSMUSG000	Flrt2	fibronectin leucine rich transmembrane protein 2
chr12	95797460	95797680	221	222	95797569	62	47.71039	11.32349	43.751	Adipose_R1_peak_	Distal Intergen	12	95692226	95785213	92988	1	399558	uc007olc.2	105234	ENSMUSG000	Flrt2	fibronectin leucine rich transmembrane protein 2
chr12	95797483	95797662	180	181	95797608	35	9.95877	3.31984	6.73562	Adipose_R2_peak_	Distal Intergen	12	95692226	95785213	92988	1	399558	uc007olc.2	105257	ENSMUSG000	Flrt2	fibronectin leucine rich transmembrane protein 2
chr12	95977588	95977867	280	281	95977736	47	25.60941	6.64031	21.98155	Adipose_R1_peak_	Distal Intergen	12	96046621	96047222	602	2	69423	uc033geu.1	69355	NA	1700019M22R	NFX-like protein
chr12	95977625	95977843	219	220	95977717	40	12.96465	3.78093	9.57654	Adipose_R2_peak_	Distal Intergen	12	96046621	96047222	602	2	69423	uc033geu.1	69379	NA	1700019M22R	NFX-like protein
chr12	96001192	96001326	135	136	96001256	22	9.13959	4.13397	6.01526	Adipose_R1_peak_	Distal Intergen	12	96046621	96047222	602	2	69423	uc033geu.1	45896	NA	1700019M22R	NFX-like protein
chr12	96006840	96007054	215	216	96006965	29	14.55748	5.39214	11.20652	Adipose_R1_peak_	Distal Intergen	12	96046621	96047222	602	2	69423	uc033geu.1	40168	NA	1700019M22R	NFX-like protein
chr12	96020282	96020450	169	170	96020415	19	7.07687	3.59476	4.08458	Adipose_R1_peak_	Distal Intergen	12	96046621	96047222	602	2	69423	uc033geu.1	26772	NA	1700019M22R	NFX-like protein
chr12	96110333	96110434	102	103	96110371	25	4.86612	2.39766	2.09018	Adipose_R2_peak_	Distal Intergen	12	96046621	96047222	602	2	69423	uc033geu.1	-63111	NA	1700019M22R	NFX-like protein
chr12	96511133	96511560	428	429	96511396	37	21.59274	6.83004	18.04766	Adipose_R1_peak_	Distal Intergen	12	96046621	96047222	602	2	69423	uc033geu.1	-463911	NA	1700019M22R	NFX-like protein
chr12	96573109	96573358	250	251	96573245	37	21.59274	6.83004	18.04766	Adipose_R1_peak_	Distal Intergen	12	96046621	96047222	602	2	69423	uc033geu.1	-525887	NA	1700019M22R	NFX-like protein
chr12	96650805	96651221	417	418	96650868	18	6.42934	3.41502	3.48999	Adipose_R1_peak_	Exon (uc007ol	12	96046621	96047222	602	2	69423	uc033geu.1	-603583	NA	1700019M22R	NFX-like protein
chr12	97061445	97061655	211	212	97061552	1716	1113.561	10.95254	1108.048	Adipose_R1_peak_	Distal Intergen	12	96046621	96047222	602	2	69423	uc033geu.1	-1014223	NA	1700019M22R	NFX-like protein
chr12	97061452	97061649	198	199	97061553	1221	417.0805	4.81422	412.2292	Adipose_R2_peak_	Distal Intergen	12	96046621	96047222	602	2	69423	uc033geu.1	-1014230	NA	1700019M22R	NFX-like protein
chr12	97381314	97381578	265	266	97381463	30	13.28936	4.77585	9.99395	Adipose_R1_peak_	Distal Intergen	12	98202300	98259459	57160	2	14420	uc007olf.2	877881	ENSMUSG000	Galc	galactosylceramidase
chr12	97381397	97381519	123	124	97381454	29	6.74062	2.76653	3.75989	Adipose_R2_peak_	Distal Intergen	12	98202300	98259459	57160	2	14420	uc007olf.2	877940	ENSMUSG000	Galc	galactosylceramidase
chr12	97559203	97559366	164	165	97559297	18	6.42934	3.41502	3.48999	Adipose_R1_peak_	Distal Intergen	12	98202300	98259459	57160	2	14420	uc007olf.2	700093	ENSMUSG000	Galc	galactosylceramidase
chr12	97567637	97567890	254	255	97567743	33	17.97508	6.11109	14.52164	Adipose_R1_peak_	Distal Intergen	12	98202300	98259459	57160	2	14420	uc007olf.2	691569	ENSMUSG000	Galc	galactosylceramidase
chr12	97567651	97567817	167	168	97567734	34	9.39139	3.22762	6.20474	Adipose_R2_peak_	Distal Intergen	12	98202300	98259459	57160	2	14420	uc007olf.2	691642	ENSMUSG000	Galc	galactosylceramidase
chr12	97621004	97621208	205	206	97621085	22	9.13959	4.13397	6.01526	Adipose_R1_peak_	Distal Intergen	12	98202300	98259459	57160	2	14420	uc007olf.2	638251	ENSMUSG000	Galc	galactosylceramidase
chr12	97670707	97670879	173	174	97670796	26	12.14079	4.85292	8.87838	Adipose_R1_peak_	Distal Intergen	12	98202300	98259459	57160	2	14420	uc007olf.2	588580	ENSMUSG000	Galc	galactosylceramidase
chr12	97680319	97680469	151	152	97680384	23	9.86443	4.31371	6.70247	Adipose_R1_peak_	Distal Intergen	12	98202300	98259459	57160	2	14420	uc007olf.2	578990	ENSMUSG000	Galc	galactosylceramidase
chr12	97924415	97924670	256	257	97924542	45	29.34988	8.26794	25.64927	Adipose_R1_peak_	Distal Intergen	12	98202300	98259459	57160	2	14420	uc007olf.2	334789	ENSMUSG000	Galc	galactosylceramidase
chr12	97924466	97924660	195	196	97924554	41	13.59774	3.87315	10.1803	Adipose_R2_peak_	Distal Intergen	12	98202300	98259459	57160	2	14420	uc007olf.2	334799	ENSMUSG000	Galc	galactosylceramidase
chr12	97925876	97926046	171	172	97926038	22	9.13959	4.13397	6.01526	Adipose_R1_peak_	Distal Intergen	12	98202300	98259459	57160	2	14420	uc007olf.2	333413	ENSMUSG000	Galc	galactosylceramidase
chr12	98081630	98081848	219	220	98081756	21	8.43286	3.95423	5.34896	Adipose_R1_peak_	Distal Intergen	12	98202300	98259459	57160	2	14420	uc007olf.2	177611	ENSMUSG000	Galc	galactosylceramidase
chr12	98445328	98445521	194	195	98445366	17	5.80341	3.23528	2.92378	Adipose_R1_peak_	Intron (uc007c	12	98489797	98532057	42261	2	72258	uc007oqx.1	86536	ENSMUSG000	Kcnk10	potassium channel, subfamily K, member 10
chr12	98486033	98486397	365	366	98486164	78	66.82238	14.19929	62.68234	Adipose_R1_peak_	Intron (uc007c	12	98489797	98532057	42261	2	72258	uc007oqx.1	45660	ENSMUSG000	Kcnk10	potassium channel, subfamily K, member 10
chr12	98486046	98486313	268	269	98486170	66	32.22715	6.17859	28.31407	Adipose_R2_peak_	Intron (uc007c	12	98489797	98532057	42261	2	72258	uc007oqx.1	45744	ENSMUSG000	Kcnk10	potassium channel, subfamily K, member 10
chr12	98558260	98558424	165	166	98558338	28	6.25023	2.67431	3.31626	Adipose_R2_peak_	Intron (uc007c	12	98429437	98574712	145276	2	72258	uc007oqg.2	16288	ENSMUSG000	Kcnk10	potassium channel, subfamily K, member 10
chr12	99645062	99645168	107	108	99645107	44	8.40763	2.59858	5.2939	Adipose_R2_peak_	Distal Intergen	12	99626053	99627974	1922	2	73457	uc007osa.2	-17088	ENSMUSG000	1700064M15R	RIKEN cDNA 1700064M15 gene
chr12	99645468	99645675	208	209	99645601	110	74.23988	10.23617	69.8667	Adipose_R2_peak_	Distal Intergen	12	99626053	99627974	1922	2	73457	uc007osa.2	-17494	ENSMUSG000	1700064M15R	RIKEN cDNA 1700064M15 gene
chr12	99645498	99645675	178	179	99645603	70	44.73269	8.82193	40.81056	Adipose_R1_peak_	Distal Intergen	12	99626053	99627974	1922	2	73457	uc007osa.2	-17524	ENSMUSG000	1700064M15R	RIKEN cDNA 1700064M15 gene
chr12	99759483	99759705	223	224	99759597	30	15.39182	5.57188	12.01394	Adipose_R1_peak_	Intron (uc007c	12	99693047	99883442	190396	2	78767	uc007osb.1	123737	ENSMUSG000	Efcab11	EF-hand calcium binding domain 11
chr12	99759495	99759629	135	136	99759552	30	7.24477	2.85875	4.21917	Adipose_R2_peak_	Intron (uc007c	12	99693047	99883442	190396	2	78767	uc007osb.1	123813	ENSMUSG000	Efcab11	EF-hand calcium binding domain 11
chr12	100194702	100194882	181	182	100194770	19	7.07687	3.59476	4.08458	Adipose_R1_peak_	Distal Intergen	12	1.00E+08	1.00E+08	10390	1	12313	uc007osq.2	-4553	ENSMUSG000	Calm1	calmodulin 1
chr12	100198068	100198239	172	173	100198118	32	8.29282	3.04319	5.18352	Adipose_R2_peak_	Promoter (1-2	12	1.00E+08	1.00E+08	10390	1	12313	uc007osq.2	-1196	ENSMUSG000	Calm1	calmodulin 1
chr12	100967145	100967347	203	204	100967263	35	19.76029	6.47056	16.26028	Adipose_R1_peak_	Intron (uc007c	12	1.01E+08	1.01E+08	13016	2	68339	uc007otf.1	15010	ENSMUSG000	Ccdc88c	coiled-coil domain containing 88C
chr12	101212914	101213493	580	581	101213143	218	203.6115	19.76451	198.9761	Adipose_R1_peak_	Intron (uc007c	12	1.01E+08	1.01E+08	3665	1	74947	uc011yqh.1	67305	ENSMUSG000	Kif4-ps	kinesin family member 4, pseudogene
chr12	101212915	101213436	522	523	101213148	266	217.7365	15.54128	212.9698	Adipose_R2_peak_	Intron (uc007c	12	1.01E+08	1.01E+08	3665	1	74947	uc011yqh.1	67306	ENSMUSG000	Kif4-ps	kinesin family member 4, pseudogene
chr12	101619126	101619396	271	272	101619256	72	59.47482	13.12087	55.39848	Adipose_R1_peak_	Intron (uc007c	12	1.02E+08	1.02E+08	66439	2	74413	uc007otm.2	92488	ENSMUSG000	Tc2n	tandem C2 domains, nuclear
chr12	101619137	101619382	246	247	101619248	54	22.67101	5.07198	18.94758	Adipose_R2_peak_	Intron (uc007c	12	1.02E+08	1.02E+08	66439	2	74413	uc007otm.2	92502	ENSMUSG000	Tc2n	tandem C2 domains, nuclear
chr12	101641803	101641946	144	145	101641867	21	8.43286	3.95423	5.34896	Adipose_R1_peak_	Intron (uc007c	12	1.02E+08	1.02E+08	66439	2	74413	uc007otm.2	69938	ENSMUSG000	Tc2n	tandem C2 domains, nuclear
chr12	102080122	102080304	183	184	102080222	29	14.55748	5.39214	11.20652	Adipose_R1_peak_	Distal Intergen	12	1.02E+08	1.02E+08	137331	1	238384	uc007oty.1	-49115	ENSMUSG000	Slc24a4	solute carrier family 24 (sodium/potassium/calcium exchanger), member 4
chr12	102125648	102125910	263	264	102125794	71	58.27042	12.94113	54.20487	Adipose_R1_peak_	Distal Intergen	12	1.02E+08	1.02E+08	137331	1	238384	uc007oty.1	-3509	ENSMUSG000	Slc24a4	solute carrier family 24 (sodium/potassium/calcium exchanger), member 4
chr12	102125658	102125898	241	242	102125783	73	38.23935	6.82411	34.23139	Adipose_R2_peak_	Distal Intergen	12	1.02E+08	1.02E+08	137331	1	238384	uc007oty.1	-3521	ENSMUSG000	Slc24a4	solute carrier family 24 (sodium/potassium/calcium exchanger), member 4
chr12	103617788	103618029	242	243	103617926	30	14.92284	5.38813	11.56983	Adipose_R1_peak_	Intron (uc007c	12	1.04E+08	1.04E+08	14770	2	217847	uc007ovz.2	13415	ENSMUSG000	Serpina10	serine (or cysteine) peptidase inhibitor, clade A (alpha-1 antiproteinase, antitrypsin), member 10
chr12	103617873	103617983	111	112	103617958	32	8.29282	3.04319	5.18352	Adipose_R2_peak_	Intron (uc007c	12	1.04E+08	1.04E+08	14770	2	217847	uc007ovz.2	13461	ENSMUSG000	Serpina10	serine (or cysteine) peptidase inhibitor, clade A (alpha-1 antiproteinase, antitrypsin), member 10
chr12	103772849	103772983	135	136	103772917	20	7.74501	3.7745	4.70467	Adipose_R1_peak_	Promoter (<=:	12	1.04E+08	1.04E+08	10047	2	20703	uc007owg.1	650	ENSMUSG000	Serpina1d	serine (or cysteine) peptidase inhibitor, clade A, member 1D
chr12	103779196	103779363	168	169	103779298	19	7.07687	3.59476	4.08458	Adipose_R1_peak_	Intron (uc007c	12	1.04E+08	1.04E+08	10047	2	20703	uc007owg.1	-5563	ENSMUSG000	Serpina1d	serine (or cysteine) peptidase inhibitor, clade A, member 1D
chr12	103793744	103793931	188	189	103793835	41	13.59774	3.87315	10.1803	Adipose_R2_peak_	Intron (uc007c	12	1.04E+08	1.04E+08	10047	2	20703	uc007owg.1	-20111	ENSMUSG000	Serpina1d	serine (or cysteine) peptidase inhibitor, clade A, member 1D
chr12	103793749	103793930	182	183	103793821	31	16.23981	5.75161	12.83549	Adipose_R1_peak_	Intron (uc007c	12	1.04E+08	1.04E+08	10047	2	20703	uc007owg.1	-20116	ENSMUSG000	Serpina1d	serine (or cysteine) peptidase inhibitor, clade A, member 1D
chr12	103795813	103795983	171	172	103795908	24	10.60668	4.49345	7.40961	Adipose_R1_peak_	Intron (uc007c	12	1.04E+08	1.04E+08	10047	2	20703	uc007owg.1	-22180	ENSMUSG000	Serpina1d	serine (or cysteine) peptidase inhibitor, clade A, member 1D
chr12	104204195	104204497	303	304	104204355	28	10.12892	3.87989	6.96466	Adipose_R1_peak_	Distal Intergen	12	1.04E+08	1.04E+08	27391	1	20715	uc029rxs.1	-10047	NA	Serpina3g	serine (or cysteine) peptidase inhibitor, clade A, member 3G
chr12	104204287	104204420	134	135	104204358	34	9.39139	3.22762	6.20474	Adipose_R2_peak_	Distal Intergen	12	1.04E+08	1.04E+08	27391	1	20715	uc029rxs.1	-10124	NA	Serpina3g	serine (or cysteine) peptidase inhibitor, clade A, member 3G
chr12	104207523	104207681	159	160	104207608	24	10.60668	4.49345	7.40961	Adipose_R1_peak_	Distal Intergen	12	1.04E+08	1.04E+08	27391	1	20715	uc029rxs.1	-6863	NA	Serpina3g	serine (or cysteine) peptidase inhibitor, clade A, member 3G
chr12	104207534	104207678	145	146	104207604	33	8.83596	3.1354	5.68652	Adipose_R2_peak_	Distal Intergen	12	1.04E+08	1.04E+08	27391	1	20715	uc029rxs.1	-6866	NA	Serpina3g	serine (or cysteine) peptidase inhibitor, clade A, member 3G
chr12	106362481	106362647	167	168	106362601	23	9.86443	4.31371	6.70247	Adipose_R1_peak_	Distal Intergen	12	1.06E+08	1.06E+08	5015	2	76639	uc011yrn.1	85019	ENSMUSG000	1700121N20R	RIKEN cDNA 1700121N20 gene
chr12	106513179	106513615	437	438	106513320	31	16.23981	5.75161	12.83549	Adipose_R1_peak_	Distal Intergen	12	1.06E+08	1.06E+08	5015	2	76639	uc011yrn.1	-65513	ENSMUSG000	1700121N20R	RIKEN cDNA 1700121N20 gene
chr12	106513316	106513548	233	234	106513446	38	11.72962	3.59649	8.4047	Adipose_R2_peak_	Distal Intergen	12	1.06E+08	1.06E+08	5015	2	76639	uc011yrn.1	-65650	ENSMUSG000	1700121N20R	RIKEN cDNA 1700121N20 gene
chr12	107329532	107329632	101	102	107329538	25	4.86612	2.39766	2.09018	Adipose_R2_peak_	Distal Intergen	12	1.08E+08	1.08E+08	11746	2	74968	uc029rxv.1	405856	ENSMUSG000	4930465M20R	RIKEN cDNA 4930465M20 gene
chr12	107383443	107383542	100	101	107383488	25	4.86612	2.39766	2.09018	Adipose_R2_peak_	Distal Intergen	12	1.08E+08	1.08E+08	11746	2	74968	uc029rxv.1	351946	ENSMUSG000	4930465M20R	RIKEN cDNA 4930465M20 gene
chr12	107794918	107795118	201	202	107794985	22	9.13959	4.13397	6.01526	Adipose_R1_peak_	Distal Intergen	12	1.08E+08	1.08E+08	11746	2	74968	uc029rxv.1	-59430	ENSMUSG000	4930465M20R	RIKEN cDNA 4930465M20 gene
chr12	108762376	108762586	211	212	108762434	17	5.80341	3.23528	2.92378	Adipose_R1_peak_	Distal Intergen	12	1.09E+08	1.09E+08	23322	1	22632	uc007pac.1	-30725	ENSMUSG000	Yy1	YY1 transcription factor
chr12	108784152	108784276	125	126	108784156	26	5.31252	2.48988	2.47999	Adipose_R2_peak_	Distal Intergen	12	1.09E+08	1.09E+08	23322	1	22632	uc007pac.1	-9035	ENSMUSG000	Yy1	YY1 transcription factor
chr12	109002315	109002447	133	134	109002378	20	7.74501	3.7745	4.70467	Adipose_R1_peak_	Intron (uc011y	12	1.09E+08	1.09E+08	36036	2	380785	uc007pal.1	65770	ENSMUSG000	Begain	brain-enriched guanylate kinase-associated
chr12	109191883	109192030	148	149	109191964	22	9.13959	4.13397	6.01526	Adipose_R1_peak_	Distal Intergen	12	1.09E+08	1.09E+08	36036	2	380785	uc007pal.1	-123666	ENSMUSG000	Begain	brain-enriched guanylate kinase-associated
chr12	109241636	109241854	219	220	109241754	27	12.93148	5.03266	9.63827	Adipose_R1_peak_	Distal Intergen	12	1.09E+08	1.09E+08	36036	2	380785	uc007pal.1	-173419	ENSMUSG000	Begain	brain-enriched guanylate kinase-associated

chr12	109244057	109244285	229	230	109244156	37	16.62622	5.08399	13.21998	Adipose_R1_peak_3	Distal Intergen	12	1.09E+08	1.09E+08	36036	2	380785	uc007pal.1	-175840	ENSMUSG000	Begain	brain-enriched guanylate kinase-associated
chr12	109244064	109244273	210	211	109244158	45	16.22858	4.24202	12.70278	Adipose_R2_peak_4	Distal Intergen	12	1.09E+08	1.09E+08	36036	2	380785	uc007pal.1	-175847	ENSMUSG000	Begain	brain-enriched guanylate kinase-associated
chr12	109821151	109821295	145	146	109821215	32	17.10103	5.93135	13.67137	Adipose_R1_peak_3	Distal Intergen	12	1.10E+08	1.10E+08	83	1	1.00E+08	uc011yul.1	73273	ENSMUSG000	Mir3072	microRNA 3072
chr12	110012155	110012420	266	267	110012303	36	10.5378	3.41206	7.27949	Adipose_R2_peak_4	Distal Intergen	12	1.10E+08	1.10E+08	6420	2	67280	uc007pbg.2	145653	NA	3110009F21Ri	RIKEN cDNA 3110009F21 gene
chr12	110012232	110012413	182	183	110012356	25	8.10044	3.44079	5.05636	Adipose_R1_peak_3	Distal Intergen	12	1.10E+08	1.10E+08	6420	2	67280	uc007pbg.2	145660	NA	3110009F21Ri	RIKEN cDNA 3110009F21 gene
chr12	110012849	110012968	120	121	110012894	32	8.29282	3.04319	5.18352	Adipose_R2_peak_4	Distal Intergen	12	1.10E+08	1.10E+08	6420	2	67280	uc007pbg.2	145105	NA	3110009F21Ri	RIKEN cDNA 3110009F21 gene
chr12	110091511	110091767	257	258	110091628	30	15.39182	5.57188	12.01394	Adipose_R1_peak_3	Distal Intergen	12	1.10E+08	1.10E+08	6420	2	67280	uc007pbg.2	66306	NA	3110009F21Ri	RIKEN cDNA 3110009F21 gene
chr12	110091531	110091823	293	294	110091629	43	14.8939	4.05758	11.42017	Adipose_R2_peak_4	Distal Intergen	12	1.10E+08	1.10E+08	6420	2	67280	uc007pbg.2	66250	NA	3110009F21Ri	RIKEN cDNA 3110009F21 gene
chr12	110133675	110134028	354	355	110133817	20	7.74501	3.7745	4.70467	Adipose_R1_peak_4	Distal Intergen	12	1.10E+08	1.10E+08	6420	2	67280	uc007pbg.2	24045	NA	3110009F21Ri	RIKEN cDNA 3110009F21 gene
chr12	110212990	110213083	94	95	110212996	25	4.86612	2.39766	2.09018	Adipose_R2_peak_4	Distal Intergen	12	1.10E+08	1.10E+08	6420	2	67280	uc007pbg.2	-54917	NA	3110009F21Ri	RIKEN cDNA 3110009F21 gene
chr12	110719718	110719998	281	282	110719879	35	19.76029	6.47056	16.26028	Adipose_R1_peak_4	Distal Intergen	12	1.11E+08	1.11E+08	57080	1	69641	uc007pbt.1	-17951	ENSMUSG000	Wdr20	WD repeat domain 20
chr12	110719831	110719948	118	119	110719892	34	9.39139	3.22762	6.20474	Adipose_R2_peak_4	Distal Intergen	12	1.11E+08	1.11E+08	57080	1	69641	uc007pbt.1	-18001	ENSMUSG000	Wdr20	WD repeat domain 20
chr12	111380494	111380662	169	170	111380553	16	5.20017	3.05554	2.38734	Adipose_R1_peak_4	Promoter (2-3	12	1.11E+08	1.11E+08	84747	2	217866	uc007pct.2	-2776	ENSMUSG000	Cdc42bpb	CDC42 binding protein kinase beta
chr12	111380570	111380723	154	155	111380587	25	4.86612	2.39766	2.09018	Adipose_R2_peak_4	Promoter (2-3	12	1.11E+08	1.11E+08	84747	2	217866	uc007pct.2	-2852	ENSMUSG000	Cdc42bpb	CDC42 binding protein kinase beta
chr12	111900104	111900266	163	164	111900161	22	9.13959	4.13397	6.01526	Adipose_R1_peak_4	Intron (uc007p	12	1.12E+08	1.12E+08	79598	2	21981	uc007ped.1	7789	ENSMUSG000	Ppp1r13b	protein phosphatase 1, regulatory (inhibitor) subunit 13B
chr12	111903904	111904012	109	110	111903910	25	4.86612	2.39766	2.09018	Adipose_R2_peak_4	Intron (uc007p	12	1.12E+08	1.12E+08	79598	2	21981	uc007ped.1	4043	ENSMUSG000	Ppp1r13b	protein phosphatase 1, regulatory (inhibitor) subunit 13B
chr12	112148584	112148716	133	134	112148621	17	5.80341	3.23528	2.92378	Adipose_R1_peak_4	Promoter (2-3	12	1.12E+08	1.12E+08	35540	1	668303	uc007pem.1	2376	ENSMUSG000	Kif26a	kinesin family member 26A
chr12	112173748	112173865	118	119	112173805	29	6.74062	2.76653	3.75989	Adipose_R2_peak_4	Exon (uc007pe	12	1.12E+08	1.12E+08	35540	1	668303	uc007pem.1	27540	ENSMUSG000	Kif26a	kinesin family member 26A
chr12	112296584	112296694	111	112	112296634	26	5.31252	2.48988	2.47999	Adipose_R2_peak_4	Distal Intergen	12	1.12E+08	1.12E+08	12176	2	1.01E+08	uc011yve.1	126504	NA	A730018C14R	RIKEN cDNA A730018C14 gene
chr12	112419438	112419593	156	157	112419542	18	6.42934	3.41502	3.48999	Adipose_R1_peak_4	Intron (uc011y	12	1.12E+08	1.12E+08	12176	2	1.01E+08	uc011yve.1	3605	NA	A730018C14R	RIKEN cDNA A730018C14 gene
chr12	112637814	112637999	186	187	112637935	19	7.07687	3.59476	4.08458	Adipose_R1_peak_4	Intron (uc007p	12	1.13E+08	1.13E+08	4325	1	30954	uc007pev.2	-6829	ENSMUSG000	Siva1	SIVA1, apoptosis-inducing factor
chr12	112639095	112639193	99	100	112639172	28	6.25023	2.67431	3.31626	Adipose_R2_peak_4	Intron (uc007p	12	1.13E+08	1.13E+08	4325	1	30954	uc007pev.2	-5635	ENSMUSG000	Siva1	SIVA1, apoptosis-inducing factor
chr12	112643150	112643296	147	148	112643219	33	8.83596	3.1354	5.68652	Adipose_R2_peak_4	Promoter (1-2	12	1.13E+08	1.13E+08	4325	1	30954	uc007pev.2	-1532	ENSMUSG000	Siva1	SIVA1, apoptosis-inducing factor
chr12	113003596	113003724	129	130	113003691	25	4.86612	2.39766	2.09018	Adipose_R2_peak_4	Promoter (2-3	12	1.13E+08	1.13E+08	40760	2	72308	uc007pfn.1	-2975	ENSMUSG000	Brf1	BRF1, RNA polymerase III transcription initiation factor 90 kDa subunit
chr12	113004129	113004362	234	235	113004231	25	4.86612	2.39766	2.09018	Adipose_R2_peak_4	Distal Intergen	12	1.13E+08	1.13E+08	40760	2	72308	uc007pfn.1	-3508	ENSMUSG000	Brf1	BRF1, RNA polymerase III transcription initiation factor 90 kDa subunit
chr12	113007372	113007543	172	173	113007436	19	7.07687	3.59476	4.08458	Adipose_R1_peak_4	Distal Intergen	12	1.13E+08	1.13E+08	40760	2	72308	uc007pfn.1	-6751	ENSMUSG000	Brf1	BRF1, RNA polymerase III transcription initiation factor 90 kDa subunit
chr12	113010728	113010909	182	183	113010833	17	5.80341	3.23528	2.92378	Adipose_R1_peak_4	Distal Intergen	12	1.13E+08	1.13E+08	59894	1	217893	uc007pfp.2	-3599	ENSMUSG000	Pacs2	phosphofurin acidic cluster sorting protein 2
chr12	113012905	113013014	110	111	113012955	28	6.25023	2.67431	3.31626	Adipose_R2_peak_4	Promoter (1-2	12	1.13E+08	1.13E+08	59894	1	217893	uc007pfp.2	-1494	ENSMUSG000	Pacs2	phosphofurin acidic cluster sorting protein 2
chr12	113154866	113155058	193	194	113154936	27	5.77405	2.5821	2.88921	Adipose_R2_peak_4	Promoter (1-2	12	1.13E+08	1.13E+08	9038	1	104732	uc007pga.2	-1363	ENSMUSG000	4930427A07R	RIKEN cDNA 4930427A07 gene
chr12	113155327	113155467	141	142	113155402	25	4.86612	2.39766	2.09018	Adipose_R2_peak_4	Promoter (<=	12	1.13E+08	1.13E+08	9038	1	104732	uc007pga.2	-954	ENSMUSG000	4930427A07R	RIKEN cDNA 4930427A07 gene
chr12	113155700	113155999	300	301	113155944	20	7.74501	3.7745	4.70467	Adipose_R1_peak_4	Promoter (<=	12	1.13E+08	1.13E+08	9038	1	104732	uc007pga.2	-422	ENSMUSG000	4930427A07R	RIKEN cDNA 4930427A07 gene
chr12	113155717	113155851	135	136	113155787	31	7.7623	2.95097	4.69388	Adipose_R2_peak_4	Promoter (<=	12	1.13E+08	1.13E+08	9038	1	104732	uc007pga.2	-570	ENSMUSG000	4930427A07R	RIKEN cDNA 4930427A07 gene
chr12	113424408	113424869	462	463	113424535	54	22.67101	5.07198	18.94758	Adipose_R2_peak_4	Intron (uc007p	12	1.13E+08	1.13E+08	2271	1	238405	uc007pgv.1	-64696	ENSMUSG000	Adam6b	a disintegrin and metallopeptidase domain 6B
chr12	113424412	113425204	793	794	113424802	48	28.3817	7.38373	24.69862	Adipose_R1_peak_4	Intron (uc007p	12	1.13E+08	1.13E+08	2271	1	238405	uc007pgv.1	-64361	ENSMUSG000	Adam6b	a disintegrin and metallopeptidase domain 6B
chr12	113709163	113709393	231	232	113709261	18	6.42934	3.41502	3.48999	Adipose_R1_peak_4	Intron (uc057b	12	1.14E+08	1.14E+08	2507	1	238406	uc007pgw.1	165255	ENSMUSG000	Adam6a	a disintegrin and metallopeptidase domain 6A
chr12	113710577	113710772	196	197	113710676	29	6.74062	2.76653	3.75989	Adipose_R2_peak_4	Intron (uc057b	12	1.14E+08	1.14E+08	2507	1	238406	uc007pgw.1	166669	ENSMUSG000	Adam6a	a disintegrin and metallopeptidase domain 6A
chr12	113883006	113883162	157	158	113883097	27	5.77405	2.5821	2.88921	Adipose_R2_peak_4	Intron (uc057b	12	1.14E+08	1.14E+08	2507	1	238406	uc007pgw.1	339098	ENSMUSG000	Adam6a	a disintegrin and metallopeptidase domain 6A
chr12	113929777	113929903	127	128	113929822	27	5.77405	2.5821	2.88921	Adipose_R2_peak_4	Intron (uc057b	12	1.14E+08	1.14E+08	2507	1	238406	uc007pgw.1	385869	ENSMUSG000	Adam6a	a disintegrin and metallopeptidase domain 6A
chr12	114116559	114116839	281	282	114116689	82	61.77571	11.61387	57.68426	Adipose_R1_peak_4	Intron (uc057b	12	1.14E+08	1.14E+08	60	2	1.02E+08	uc033ggh.1	352627	ENSMUSG000	Mir7094-1	microRNA 7094-1
chr12	114146211	114146435	225	226	114146310	30	15.39182	5.57188	12.01394	Adipose_R1_peak_4	Intron (uc057b	12	1.14E+08	1.14E+08	60	2	1.02E+08	uc033ggh.1	323031	ENSMUSG000	Mir7094-1	microRNA 7094-1
chr12	114235689	114235956	268	269	114235831	50	34.50784	9.16663	30.72138	Adipose_R1_peak_4	Intron (uc057b	12	1.14E+08	1.14E+08	60	2	1.02E+08	uc033ggh.1	233510	ENSMUSG000	Mir7094-1	microRNA 7094-1
chr12	114235702	114235942	241	242	114235829	52	21.18119	4.88754	17.49655	Adipose_R2_peak_4	Intron (uc057b	12	1.14E+08	1.14E+08	60	2	1.02E+08	uc033ggh.1	233524	ENSMUSG000	Mir7094-1	microRNA 7094-1
chr12	114312104	114312326	223	224	114312192	24	10.60668	4.49345	7.40961	Adipose_R1_peak_4	Intron (uc057b	12	1.14E+08	1.14E+08	60	2	1.02E+08	uc033ggh.1	157140	ENSMUSG000	Mir7094-1	microRNA 7094-1
chr12	114346577	114346847	271	272	114346716	44	28.34544	8.08821	24.66239	Adipose_R1_peak_4	Intron (uc057b	12	1.14E+08	1.14E+08	60	2	1.02E+08	uc033ggh.1	122619	ENSMUSG000	Mir7094-1	microRNA 7094-1
chr12	114346627	114346801	175	176	114346694	44	15.55653	4.1498	12.05625	Adipose_R2_peak_4	Intron (uc057b	12	1.14E+08	1.14E+08	60	2	1.02E+08	uc033ggh.1	122665	ENSMUSG000	Mir7094-1	microRNA 7094-1
chr12	114438478	114438732	255	256	114438625	42	26.36523	7.72873	22.71906	Adipose_R1_peak_4	Intron (uc057b	12	1.14E+08	1.14E+08	60	2	1.02E+08	uc033ggh.1	30734	ENSMUSG000	Mir7094-1	microRNA 7094-1
chr12	114438547	114438732	186	187	114438631	37	11.12817	3.50427	7.83613	Adipose_R2_peak_4	Intron (uc057b	12	1.14E+08	1.14E+08	60	2	1.02E+08	uc033ggh.1	30734	ENSMUSG000	Mir7094-1	microRNA 7094-1
chr12	114504005	114504156	152	153	114504087	22	9.13959	4.13397	6.01526	Adipose_R1_peak_4	Intron (uc057b	12	1.15E+08	1.15E+08	60	2	1.02E+08	uc033ggi.1	9538	ENSMUSG000	Mir7094-2	microRNA 7094-2
chr12	114552217	114552565	349	350	114552420	43	27.35048	7.90847	23.68561	Adipose_R1_peak_4	Intron (uc057b	12	1.15E+08	1.15E+08	60	2	1.02E+08	uc033ggi.1	-38523	ENSMUSG000	Mir7094-2	microRNA 7094-2
chr12	114552405	114552580	176	177	114552448	42	14.2409	3.96536	10.79479	Adipose_R2_peak_4	Intron (uc057b	12	1.15E+08	1.15E+08	60	2	1.02E+08	uc033ggi.1	-38711	ENSMUSG000	Mir7094-2	microRNA 7094-2
chr12	114592185	114592394	210	211	114592309	32	17.10103	5.93135	13.67137	Adipose_R1_peak_4	Intron (uc057b	12	1.15E+08	1.15E+08	60	2	1.02E+08	uc033ggi.1	-78491	ENSMUSG000	Mir7094-2	microRNA 7094-2
chr12	114592231	114592365	135	136	114592313	30	7.24477	2.85875	4.21917	Adipose_R2_peak_4	Intron (uc057b	12	1.15E+08	1.15E+08	60	2	1.02E+08	uc033ggi.1	-78537	ENSMUSG000	Mir7094-2	microRNA 7094-2
chr12	114768607	114768701	95	96	114768652	29	6.74062	2.76653	3.75989	Adipose_R2_peak_4	Intron (uc057b	12	1.15E+08	1.15E+08	60	2	1.02E+08	uc033ggi.1	-254913	ENSMUSG000	Mir7094-2	microRNA 7094-2
chr12	114780142	114780455	314	315	114780406	27	5.77405	2.5821	2.88921	Adipose_R2_peak_4	Intron (uc057b	12	1.15E+08	1.15E+08	60	2	1.02E+08	uc033ggi.1	-266448	ENSMUSG000	Mir7094-2	microRNA 7094-2
chr12	114782802	114782941	140	141	114782866	18	6.42934	3.41502	3.48999	Adipose_R1_peak_4	Intron (uc057b	12	1.15E+08	1.15E+08	60	2	1.02E+08	uc033ggi.1	-269108	ENSMUSG000	Mir7094-2	microRNA 7094-2
chr12	114787103	114787310	208	209	114787162	34	9.39139	3.22762	6.20474	Adipose_R2_peak_4	Intron (uc057b	12	1.15E+08	1.15E+08	60	2	1.02E+08	uc033ggi.1	-273409	ENSMUSG000	Mir7094-2	microRNA 7094-2
chr12	114972076	114972317	242	243	114972189	35	19.76029	6.47056	16.26028	Adipose_R1_peak_4	Intron (uc057b	12	1.15E+08	1.15E+08	60	2	1.02E+08	uc033ggi.1	-458382	ENSMUSG000	Mir7094-2	microRNA 7094-2
chr12	114972131	114972225	95	96	114972179	28	6.25023	2.67431	3.31626	Adipose_R2_peak_4	Intron (uc057b	12	1.15E+08	1.15E+08	60	2	1.02E+08	uc033ggi.1	-458437	ENSMUSG000	Mir7094-2	microRNA 7094-2
chr12	115219569	115219771	203	204	115219675	33	17.97508	6.11109	14.52164	Adipose_R1_peak_4	Intron (uc057b	12	1.16E+08	1.16E+08	75	2	1.02E+08	uc033ggj.1	476023	ENSMUSG000	Mir6388	microRNA 6388
chr12	115423498	115423925	428	429	115423645	36	20.67077	6.6503	17.14779	Adipose_R1_peak_4	Intron (uc057b	12	1.16E+08	1.16E+08	75	2	1.02E+08	uc033ggj.1	271869	ENSMUSG000	Mir6388	microRNA 6388
chr12	115513831	115514001	171	172	115513919	26	12.14079	4.85292	8.87838	Adipose_R1_peak_4	Intron (uc057b	12	1.16E+08	1.16E+08	75	2	1.02E+08	uc033ggj.1	181793	ENSMUSG000	Mir6388	microRNA 6388
chr12	115514922	115515062	141	142	115514986	22	9.13959	4.13397	6.01526	Adipose_R1_peak_4	Intron (uc057b	12	1.16E+08	1.16E+08	75	2	1.02E+08	uc033ggj.1	180732	ENSMUSG000	Mir6388	microRNA 6388
chr12	115560955	115561208	254	255	115561061	23	9.86443	4.31371	6.70247	Adipose_R1_peak_4	Intron (uc057b	12	1.16E+08	1.16E+08	75	2	1.02E+08	uc033ggj.1	134586	ENSMUSG000	Mir6388	microRNA 6388
chr12	115561809	115562176	368	369	115562029	25	11.36566	4.67319	8.13482	Adipose_R1_peak_4	Intron (uc057b	12	1.16E+08	1.16E+08	75	2	1.02E+08	uc033ggj.1	133618	ENSMUSG000	Mir6388	microRNA 6388
chr12	115563752	115563991	240	241	115563877	29	14.55748	5.39214	11.20652	Adipose_R1_peak_4	Intron (uc057b	12	1.16E+08	1.16E+08	75	2	1.02E+08	uc033ggj.1	131803	ENSMUSG000	Mir6388	microRNA 6388
chr12	115563759	115563975	217	218	115563844	35	9.95877	3.31984	6.73562	Adipose_R2_peak_4	Intron (uc057b	12	1.16E+08	1.16E+08	75	2	1.02E+08	uc033ggj.1	131819	ENSMUSG000	Mir6388	microRNA 6388
chr12	115568253	115568494	242	243	115568376	39	23.47001	7.18952	19.88325	Adipose_R1_peak_4	Intron (uc057b	12	1.16E+08	1.16E+08	75	2	1.02E+08	uc033ggj.1	127300	ENSMUSG000	Mir6388	microRNA 6388
chr12	115568330	115568429	100	101	115568376	29	6.74062	2.76653	3.75989	Adipose_R2_peak_4	Intron (uc057b	12	1.16E+08	1.16E+08	75	2	1.02E+08	uc033ggj.1	127365	ENSMUSG000	Mir6388	microRNA 6388
chr12	115583559	115583722	164	165	115583632	32	8.29282	3.04319	5.18352	Adipose_R2_peak_4	Intron (uc057b	12	1.16E+08	1.16E+08	75	2	1.02E+08	uc033ggj.1	112072	ENSMUSG000	Mir6388	microRNA 6388
chr12	115657573	115657844	272	273	115657707	45	29.34988	8.26794	25.64927	Adipose_R1_peak_4	Intron (uc057b	12	1.16E+08	1.16E+08	75	2	1.02E+08	uc033ggj.1	37950	ENSMUSG000	Mir6388	microRNA 6388
chr12	115657583	115657834	252	253	115657715	50	19.72356	4.7031	16.08069	Adipose_R2_peak_4	Intron (uc057b	12	1.16E+08	1.16E+08	75	2	1.02E+08	uc033ggj.1	37960	ENSMUSG000	Mir6388	microRNA 6388
chr12	115678807	115678997	191	192	115678893	24	10.60668	4.49345	7.40961	Adipose_R1_peak_4	Intron (uc057b	12	1.16E+08	1.16E+08	75	2	1.02E+08	uc033ggj.1	16797	ENSMUSG000	Mir6388	microRNA 6388
chr12	115678839	115678946	108	109	115678883	29	6.74062	2.76653	3.75989	Adipose_R2_peak_4	Intron (uc057b	12	1.16E+08	1.16E+08	75	2	1.02E+08	uc033ggj.1	16848	ENSMUSG000	Mir6388	microRNA 6388
chr12	115697339	115697482	144	145	115697403	18	6.42934	3.41502	3.48999	Adipose_R1_peak_4	Promoter (1-2	12	1.16E+08	1.16E+08	75	2	1.02E+08	uc033ggj.1	-1545	ENSMUSG000	Mir6388	microRNA 6388
chr12	115726328	115726511	184	185	115726430	22	9.13959	4.13397	6.01526	Adipose_R1_peak_4	Intron (uc057b	12	1.16E+08	1.16E+08	75	2	1.02E+08	uc033ggj.1	-30534	ENSMUSG000	Mir6388	microRNA 6388
chr12	115728382	115728549	168	169	115728466	28	13.67692	5.18832	10.37889	Adipose_R1_peak_4	Intron (uc057b	12	1.16E+08	1.16E+08	75	2	1.02E+08	uc033ggj.1	-32588	ENSMUSG000	Mir6388	microRNA 6388
chr12	115729658	115729883	226	227	115729775	46	29.93291	8.28714	26.2295	Adipose_R1_peak_4	Intron (uc057b	12	1.16E+08	1.16E+08	75	2	1.02E+08	uc033ggj.1	-33864	ENSMUSG000	Mir6388	microRNA 6388
chr12	115729666	115729883	218	219	115729788	46	16.90986	4.33423	13.35898	Adipose_R2_peak_4	Intron (uc057b	12	1.16E+08	1.16E+08	75	2	1.02E+08	uc033ggj.1	-33872	ENSMUSG000	Mir6388	microRNA 6388
chr12	116018130	116018398	269	270	116018255	47	31.38649	8.62742	27.65048	Adipose_R1_peak_4	Distal Intergen	12	1.16E+08	1.16E+08	7292	1	56220	uc007pha.1	-29326	ENSMUSG000	Zfp386	zinc finger protein 386 (Kruppel-like)
chr12	116018134	116018405	272	273	116018258	59	26.5291	5.53306	22.7188	Adipose_R2_peak_4	Distal Intergen	12	1.16E+08	1.16E+08	7292	1	56220	uc007pha.1	-29319	ENSMUSG000	Zfp386	zinc finger protein 386 (Kruppel-like)
chr12	116162713	116162919	207	208	116162817	21	8.43286	3.95423	5.34896	Adipose_R1_peak_4	Distal Intergen	12	1.16E+08	1.16E+08	6821	2	217935	uc007phh.1	50951	ENSMUSG000	Wdr60	WD repeat domain 60
chr12	116249194	116249296	103	104	116249241	28	6.25023	2.67431	3.31626	Adipose_R2_peak_4	Intron (uc007p	12	1.16E+08	1.16E+08	55976	2	217935	uc007phj.1	13729	ENSMUSG000	Wdr60	WD repeat domain 60
chr12	116649643	116649775	133	134	116649755	18	6.42934	3.41502	3.48999	Adipose_R1_peak_4	Intron (uc007p	12	1.16E+08	1.17E+08	287611	1	19276	uc007phv.2	163923	ENSMUSG000	Ptprn2	protein tyrosine phosphatase, receptor type, N polypeptide 2
chr12	116700778	116701243	466	467	116701008	204	214.6311	25.21499	209.9786	Adipose_R1_peak_4	Intron (uc007p	12	1.16E+08	1.17E+08	287611	1	19276	uc007phv.2	215058	ENSMUSG000	Ptprn2	protein tyrosine phosphatase, receptor type, N polypeptide 2
chr12	116700809	116701202	394	395	116701006	181	135.7798	12.84999	131.1599	Adipose_R2_peak_4	Intron (uc007p	12	1.16E+08	1.17E+08	287611	1	19276	uc007phv.2	215089	ENSMUSG000	Ptprn2	protein tyrosine phosphatase, receptor type, N polypeptide 2
chr12	116796529	116796721	193	194	116796632	27	12.93148	5.03266	9.63827	Adipose_R1_peak_4	Intron (uc007p	12	1.16E+08	1.17E+08	287611	1	19276	uc007phv.2	310809	ENSMUSG000	Ptprn2	protein tyrosine phosphatase, receptor type, N polypeptide 2
chr12	116796538	116796714	177	178	116796649	35	9.95877	3.31984	6.73562	Adipose_R2_peak_4	Intron (uc007p	12	1.16E+08	1.17E+08	287611	1	19276	uc007phv.2	310818	ENSMUSG000	Ptprn2	protein tyrosine phosphatase, receptor type, N polypeptide 2
chr12	116849894	116850294	401	402	116850120	112	109.893	19.64059	105.5178	Adipose_R1_peak_4	Intron (uc007p	12	1.17E+08	1.17E+08	6747	2	1.00E+08	uc007phz.2	300975	ENSMUSG000	Gm10421	predicted gene 10421
chr12	116849974	116850273	300	301	116850120	91	54.95	8.48403	50.74234	Adipose_R2_peak_4	Intron (uc007p	12	1.17E+08	1.17E+08	6747	2	1.00E+08	uc007phz.2	300996	ENSMUSG000	Gm10421	predicted gene 10421
chr12	116905526	116905659	134	135	116905640	17	5.80341	3.23528	2.92378	Adipose_R1_peak_4	Intron (uc007p	12	1.17E+08	1.17E+08	6747	2	1.00E+08	uc007phz.2	245610	ENSMUSG000	Gm10421	predicted gene 10421
chr12	117126408	117126590	183	184	117126433	17	5.80341	3.23528	2.92378	Adipose_R1_peak_4	Intron (uc007p	12	1.17E+08	1.17E+08	6747	2	1.00E+08	uc007phz.2	24679	ENSMUSG000	Gm10421	predicted gene 10421
chr12	117588403	117588557	155	156	117588486	32	8.29282	3.04319	5.18352	Adipose_R2_peak_4	Intron (uc007p	12	1.18E+08	1.18E+08	124465	1	217944	uc007pic.1	54464	ENSMUSG000	Rapgef5	Rap guanine nucleotide exchange factor (GEF) 5
chr12	117588412	117588607	196	197	117588505	37	18.51455	5.71008	15.05863	Adipose_R1_peak_4	Intron (uc007p	12	1.18E+08	1.18E+08	124465	1	217944	uc007pic.1	54473	ENSMUSG000	Rapgef5	Rap guanine nucleotide exchange factor (GEF) 5
chr12	117861385	117862162	778	779	117861464	22	9.13959	4.13397	6.01526	Adipose_R1_peak_4	Intron (uc007p	12	1.18E+08	1.18E+08	34846	1	217946	uc007pid.1	17524	ENSMUSG000	Cdca7l	cell division cycle associated 7 like

chr12	117861620	117861832	213	214	117861764	46	16.90986	4.33423	13.35898	Adipose_R2_peak_	Intron (uc007p	12	1.18E+08	1.18E+08	34846	1	217946	uc007pid.1	17759	ENSMUSG000	Cdca7l	cell division cycle associated 7 like
chr12	117898562	117898764	203	204	117898683	31	16.23981	5.75161	12.83549	Adipose_R1_peak_	Intron (uc011y	12	1.18E+08	1.18E+08	55905	2	13411	uc011yvs.1	35122	ENSMUSG000	Dnah11	dynein, axonemal, heavy chain 11
chr12	117898576	117898809	234	235	117898703	44	15.55653	4.1498	12.05625	Adipose_R2_peak_	Intron (uc011y	12	1.18E+08	1.18E+08	55905	2	13411	uc011yvs.1	35077	ENSMUSG000	Dnah11	dynein, axonemal, heavy chain 11
chr12	118132120	118132350	231	232	118132196	70	57.07198	12.76139	53.01762	Adipose_R1_peak_	Intron (uc007p	12	1.18E+08	1.18E+08	321062	2	13411	uc007pie.1	66693	ENSMUSG000	Dnah11	dynein, axonemal, heavy chain 11
chr12	118132128	118132311	184	185	118132186	58	25.74274	5.44085	21.94821	Adipose_R2_peak_	Intron (uc007p	12	1.18E+08	1.18E+08	321062	2	13411	uc007pie.1	66732	ENSMUSG000	Dnah11	dynein, axonemal, heavy chain 11
chr12	118132612	118132876	265	266	118132737	35	19.76029	6.47056	16.26028	Adipose_R1_peak_	Intron (uc007p	12	1.18E+08	1.18E+08	321062	2	13411	uc007pie.1	66167	ENSMUSG000	Dnah11	dynein, axonemal, heavy chain 11
chr12	119013585	119013833	249	250	119013714	52	36.63102	9.52611	32.81188	Adipose_R1_peak_	Distal Interger	12	1.19E+08	1.19E+08	98598	2	77706	uc007pij.2	-47164	ENSMUSG000	Abcb5	ATP-binding cassette, sub-family B (MDR/TAP), member 5
chr12	119013627	119013824	198	199	119013701	40	12.96465	3.78093	9.57654	Adipose_R2_peak_	Distal Interger	12	1.19E+08	1.19E+08	98598	2	77706	uc007pij.2	-47206	ENSMUSG000	Abcb5	ATP-binding cassette, sub-family B (MDR/TAP), member 5
chr12	119014550	119014699	150	151	119014630	21	8.43286	3.95423	5.34896	Adipose_R1_peak_	Distal Interger	12	1.19E+08	1.19E+08	98598	2	77706	uc007pij.2	-48129	ENSMUSG000	Abcb5	ATP-binding cassette, sub-family B (MDR/TAP), member 5
chr12	119016429	119016619	191	192	119016536	24	10.60668	4.49345	7.40961	Adipose_R1_peak_	Distal Interger	12	1.19E+08	1.19E+08	98598	2	77706	uc007pij.2	-50008	ENSMUSG000	Abcb5	ATP-binding cassette, sub-family B (MDR/TAP), member 5
chr12	119086966	119087139	174	175	119087043	23	9.86443	4.31371	6.70247	Adipose_R1_peak_	Distal Interger	12	1.19E+08	1.19E+08	98598	2	77706	uc007pij.2	-120545	ENSMUSG000	Abcb5	ATP-binding cassette, sub-family B (MDR/TAP), member 5
chr12	119518275	119518464	190	191	119518395	19	7.07687	3.59476	4.08458	Adipose_R1_peak_	Distal Interger	12	1.19E+08	1.19E+08	23523	1	238455	uc007pin.2	74865	ENSMUSG000	Macc1	metastasis associated in colon cancer 1
chr12	119555593	119555707	115	116	119555653	37	10.27183	3.28772	7.0385	Adipose_R2_peak_	Distal Interger	12	1.19E+08	1.19E+08	23523	1	238455	uc007pin.2	112183	ENSMUSG000	Macc1	metastasis associated in colon cancer 1
chr12	119556407	119556621	215	216	119556523	39	10.93424	3.34182	7.66364	Adipose_R2_peak_	Distal Interger	12	1.19E+08	1.19E+08	23523	1	238455	uc007pin.2	112997	ENSMUSG000	Macc1	metastasis associated in colon cancer 1
chr12	119665803	119665986	184	185	119665907	37	11.12817	3.50427	7.83613	Adipose_R2_peak_	Distal Interger	12	1.19E+08	1.19E+08	23523	1	238455	uc007pin.2	222393	ENSMUSG000	Macc1	metastasis associated in colon cancer 1
chr12	119665823	119666045	223	224	119665925	20	7.74501	3.7745	4.70467	Adipose_R1_peak_	Distal Interger	12	1.19E+08	1.19E+08	23523	1	238455	uc007pin.2	222413	ENSMUSG000	Macc1	metastasis associated in colon cancer 1
chr12	119669331	119669504	174	175	119669376	31	7.7623	2.95097	4.69388	Adipose_R2_peak_	Distal Interger	12	1.19E+08	1.19E+08	23523	1	238455	uc007pin.2	225921	ENSMUSG000	Macc1	metastasis associated in colon cancer 1
chr12	119669345	119669504	160	161	119669489	20	7.74501	3.7745	4.70467	Adipose_R1_peak_	Distal Interger	12	1.19E+08	1.19E+08	23523	1	238455	uc007pin.2	225935	ENSMUSG000	Macc1	metastasis associated in colon cancer 1
chr12	119691281	119691424	144	145	119691351	21	8.43286	3.95423	5.34896	Adipose_R1_peak_	Distal Interger	12	1.19E+08	1.19E+08	23523	1	238455	uc007pin.2	247871	ENSMUSG000	Macc1	metastasis associated in colon cancer 1
chr12	119692075	119692211	137	138	119692180	17	5.80341	3.23528	2.92378	Adipose_R1_peak_	Distal Interger	12	1.19E+08	1.19E+08	23523	1	238455	uc007pin.2	248665	ENSMUSG000	Macc1	metastasis associated in colon cancer 1
chr12	119820245	119820398	154	155	119820315	20	7.74501	3.7745	4.70467	Adipose_R1_peak_	Distal Interger	12	1.20E+08	1.20E+08	75284	1	217951	uc011yvw.2	-125564	ENSMUSG000	Tmem196	transmembrane protein 196
chr12	119931686	119931955	270	271	119931809	62	28.93052	5.80972	25.0743	Adipose_R2_peak_	Distal Interger	12	1.20E+08	1.20E+08	75284	1	217951	uc011yvw.2	-14007	ENSMUSG000	Tmem196	transmembrane protein 196
chr12	119931687	119931966	280	281	119931824	49	33.45876	8.98689	29.68925	Adipose_R1_peak_	Distal Interger	12	1.20E+08	1.20E+08	75284	1	217951	uc011yvw.2	-13996	ENSMUSG000	Tmem196	transmembrane protein 196
chr12	120023831	120024004	174	175	120023915	31	16.23981	5.75161	12.83549	Adipose_R1_peak_	Distal Interger	12	1.20E+08	1.20E+08	75284	1	217951	uc011yvw.2	77869	ENSMUSG000	Tmem196	transmembrane protein 196
chr13	3003609	3003763	155	156	3003740	27	5.77405	2.5821	2.88921	Adipose_R2_peak_	Distal Interger	13	3149475	3189678	40204	1	73266	uc007pio.1	-145712	NA	Speer6-ps1	spermatogenesis associated glutamate (E)-rich protein 6, pseudogene 1
chr13	3008233	3008395	163	164	3008321	40	12.96465	3.78093	9.57654	Adipose_R2_peak_	Distal Interger	13	3149475	3189678	40204	1	73266	uc007pio.1	-141080	NA	Speer6-ps1	spermatogenesis associated glutamate (E)-rich protein 6, pseudogene 1
chr13	3034248	3034399	152	153	3034320	36	10.5378	3.41206	7.27949	Adipose_R2_peak_	Distal Interger	13	3149475	3189678	40204	1	73266	uc007pio.1	-115076	NA	Speer6-ps1	spermatogenesis associated glutamate (E)-rich protein 6, pseudogene 1
chr13	3037084	3037449	366	367	3037239	66	22.04516	4.16586	18.33853	Adipose_R2_peak_	Distal Interger	13	3149475	3189678	40204	1	73266	uc007pio.1	-112026	NA	Speer6-ps1	spermatogenesis associated glutamate (E)-rich protein 6, pseudogene 1
chr13	3114590	3114727	138	139	3114665	21	8.43286	3.95423	5.34896	Adipose_R1_peak_	Distal Interger	13	3149475	3189678	40204	1	73266	uc007pio.1	-34748	NA	Speer6-ps1	spermatogenesis associated glutamate (E)-rich protein 6, pseudogene 1
chr13	3372907	3373651	745	746	3373121	882	358.9011	5.76879	353.9728	Adipose_R2_peak_	Distal Interger	13	3478260	3485220	6961	1	76937	uc007pit.1	-104609	ENSMUSG000	2810429I04Ri	RIKEN cDNA 2810429I04 gene
chr13	3372916	3373623	708	709	3373122	439	119.3227	3.85911	114.9099	Adipose_R1_peak_	Distal Interger	13	3478260	3485220	6961	1	76937	uc007pit.1	-104637	ENSMUSG000	2810429I04Ri	RIKEN cDNA 2810429I04 gene
chr13	3406302	3406474	173	174	3406370	45	16.22858	4.24202	12.70278	Adipose_R2_peak_	Distal Interger	13	3478260	3485220	6961	1	76937	uc007pit.1	-71786	ENSMUSG000	2810429I04Ri	RIKEN cDNA 2810429I04 gene
chr13	3408516	3408620	105	106	3408522	27	4.82672	2.31278	2.09018	Adipose_R2_peak_	Distal Interger	13	3478260	3485220	6961	1	76937	uc007pit.1	-69640	ENSMUSG000	2810429I04Ri	RIKEN cDNA 2810429I04 gene
chr13	3408888	3409011	124	125	3408950	36	6.72734	2.49175	3.75989	Adipose_R2_peak_	Distal Interger	13	3478260	3485220	6961	1	76937	uc007pit.1	-69249	ENSMUSG000	2810429I04Ri	RIKEN cDNA 2810429I04 gene
chr13	3435648	3435828	181	182	3435745	47	13.94402	3.59801	10.51794	Adipose_R2_peak_	Distal Interger	13	3478260	3485220	6961	1	76937	uc007pit.1	-42432	ENSMUSG000	2810429I04Ri	RIKEN cDNA 2810429I04 gene
chr13	3436798	3436945	148	149	3436880	46	16.90986	4.33423	13.35898	Adipose_R2_peak_	Distal Interger	13	3478260	3485220	6961	1	76937	uc007pit.1	-41315	ENSMUSG000	2810429I04Ri	RIKEN cDNA 2810429I04 gene
chr13	3437222	3437364	143	144	3437309	29	6.74062	2.76653	3.75989	Adipose_R2_peak_	Distal Interger	13	3478260	3485220	6961	1	76937	uc007pit.1	-40896	ENSMUSG000	2810429I04Ri	RIKEN cDNA 2810429I04 gene
chr13	3481648	3481789	142	143	3481711	18	6.42934	3.41502	3.48999	Adipose_R1_peak_	Intron (uc007p	13	3478303	3488058	9756	1	76937	uc007pip.2	3345	ENSMUSG000	2810429I04Ri	RIKEN cDNA 2810429I04 gene
chr13	4070283	4070453	171	172	4070442	36	10.5378	3.41206	7.27949	Adipose_R2_peak_	Intron (uc007p	13	4059591	4090422	30832	1	105387	uc007pji.1	10692	ENSMUSG000	Akr1c14	aldo-keto reductase family 1, member C14
chr13	4070297	4070453	157	158	4070443	19	7.07687	3.59476	4.08458	Adipose_R1_peak_	Intron (uc007p	13	4059591	4090422	30832	1	105387	uc007pji.1	10706	ENSMUSG000	Akr1c14	aldo-keto reductase family 1, member C14
chr13	4632550	4633439	890	891	4632719	50	30.80482	7.85705	27.08404	Adipose_R1_peak_	Distal Interger	13	4592490	4609164	16675	2	56043	uc007pjs.1	-23386	ENSMUSG000	Akr1e1	aldo-keto reductase family 1, member E1
chr13	4632639	4633087	449	450	4632799	55	20.08982	4.42509	16.44137	Adipose_R2_peak_	Distal Interger	13	4592490	4609164	16675	2	56043	uc007pjs.1	-23475	ENSMUSG000	Akr1e1	aldo-keto reductase family 1, member E1
chr13	4634831	4635008	178	179	4634916	21	8.43286	3.95423	5.34896	Adipose_R1_peak_	Distal Interger	13	4592490	4609164	16675	2	56043	uc007pjs.1	-25667	ENSMUSG000	Akr1e1	aldo-keto reductase family 1, member E1
chr13	4635226	4635408	183	184	4635301	22	9.13959	4.13397	6.01526	Adipose_R1_peak_	Distal Interger	13	4592490	4609164	16675	2	56043	uc007pjs.1	-26062	ENSMUSG000	Akr1e1	aldo-keto reductase family 1, member E1
chr13	4639130	4639292	163	164	4639232	21	6.05307	3.00936	3.16948	Adipose_R1_peak_	Distal Interger	13	4592490	4609164	16675	2	56043	uc007pjs.1	-29966	ENSMUSG000	Akr1e1	aldo-keto reductase family 1, member E1
chr13	4641659	4641818	160	161	4641753	18	6.42934	3.41502	3.48999	Adipose_R1_peak_	Distal Interger	13	4592490	4609164	16675	2	56043	uc007pjs.1	-32495	ENSMUSG000	Akr1e1	aldo-keto reductase family 1, member E1
chr13	4815317	4815807	491	492	4815481	22	9.13959	4.13397	6.01526	Adipose_R1_peak_	Distal Interger	13	4592490	4609164	16675	2	56043	uc007pjs.1	-206153	ENSMUSG000	Akr1e1	aldo-keto reductase family 1, member E1
chr13	4815394	4815758	365	366	4815460	35	9.95877	3.31984	6.73562	Adipose_R2_peak_	Distal Interger	13	4592490	4609164	16675	2	56043	uc007pjs.1	-206230	ENSMUSG000	Akr1e1	aldo-keto reductase family 1, member E1
chr13	4829577	4829755	179	180	4829690	31	16.23981	5.75161	12.83549	Adipose_R1_peak_	Distal Interger	13	4592490	4609164	16675	2	56043	uc007pjs.1	-220413	ENSMUSG000	Akr1e1	aldo-keto reductase family 1, member E1
chr13	4874177	4874291	115	116	4874222	30	7.24477	2.85875	4.21917	Adipose_R2_peak_	Distal Interger	13	4592490	4609164	16675	2	56043	uc007pjs.1	-265013	ENSMUSG000	Akr1e1	aldo-keto reductase family 1, member E1
chr13	4980774	4981085	312	313	4980919	30	15.39182	5.57188	12.01394	Adipose_R1_peak_	Distal Interger	13	4592490	4609164	16675	2	56043	uc007pjs.1	-371610	ENSMUSG000	Akr1e1	aldo-keto reductase family 1, member E1
chr13	4980803	4981003	201	202	4980925	32	8.29282	3.04319	5.18352	Adipose_R2_peak_	Distal Interger	13	4592490	4609164	16675	2	56043	uc007pjs.1	-371639	ENSMUSG000	Akr1e1	aldo-keto reductase family 1, member E1
chr13	5176012	5176255	244	245	5176110	42	26.36523	7.72873	22.71906	Adipose_R1_peak_	Distal Interger	13	4592490	4609164	16675	2	56043	uc007pjs.1	-566848	ENSMUSG000	Akr1e1	aldo-keto reductase family 1, member E1
chr13	5864763	5865048	286	287	5864898	35	9.95877	3.31984	6.73562	Adipose_R2_peak_	Exon (uc007pj	13	5861489	5870393	8905	1	23849	uc007pjw.1	3274	ENSMUSG000	Klf6	Kruppel-like factor 6
chr13	5866980	5867122	143	144	5867077	27	5.77405	2.5821	2.88921	Adipose_R2_peak_	Intron (uc007p	13	5861489	5870393	8905	1	23849	uc007pjw.1	5491	ENSMUSG000	Klf6	Kruppel-like factor 6
chr13	5893066	5893255	190	191	5893129	16	5.20017	3.05554	2.38734	Adipose_R1_peak_	Distal Interger	13	5861489	5870393	8905	1	23849	uc007pjw.1	31577	ENSMUSG000	Klf6	Kruppel-like factor 6
chr13	5906039	5906213	175	176	5906103	22	9.13959	4.13397	6.01526	Adipose_R1_peak_	Distal Interger	13	5861489	5870393	8905	1	23849	uc007pjw.1	44550	ENSMUSG000	Klf6	Kruppel-like factor 6
chr13	5961364	5961603	240	241	5961476	35	19.76029	6.47056	16.26028	Adipose_R1_peak_	Distal Interger	13	5861489	5870393	8905	1	23849	uc007pjw.1	99875	ENSMUSG000	Klf6	Kruppel-like factor 6
chr13	6522606	6522752	147	148	6522670	21	8.43286	3.95423	5.34896	Adipose_R1_peak_	Distal Interger	13	6548157	6580149	31993	1	69617	uc007pjx.1	-25405	ENSMUSG000	Pitrm1	pitrilysin metallepetidase 1
chr13	6522615	6522737	123	124	6522719	30	7.24477	2.85875	4.21917	Adipose_R2_peak_	Distal Interger	13	6548157	6580149	31993	1	69617	uc007pjx.1	-25420	ENSMUSG000	Pitrm1	pitrilysin metallepetidase 1
chr13	6730885	6731112	228	229	6730992	50	34.50784	9.16663	30.72138	Adipose_R1_peak_	Distal Interger	13	6579874	6648771	68898	2	56421	uc007pjz.2	-82114	ENSMUSG000	Pfkp	phosphofructokinase, platelet
chr13	6784580	6784855	276	277	6784721	59	44.30963	10.78427	40.38949	Adipose_R1_peak_	Distal Interger	13	6579874	6648771	68898	2	56421	uc007pjz.2	-135809	ENSMUSG000	Pfkp	phosphofructokinase, platelet
chr13	6784662	6784784	123	124	6784717	32	8.29282	3.04319	5.18352	Adipose_R2_peak_	Distal Interger	13	6579874	6648771	68898	2	56421	uc007pjz.2	-135891	ENSMUSG000	Pfkp	phosphofructokinase, platelet
chr13	6821253	6821411	159	160	6821355	34	9.39139	3.22762	6.20474	Adipose_R2_peak_	Distal Interger	13	6579874	6648771	68898	2	56421	uc007pjz.2	-172482	ENSMUSG000	Pfkp	phosphofructokinase, platelet
chr13	6821272	6821420	149	150	6821353	22	9.13959	4.13397	6.01526	Adipose_R1_peak_	Distal Interger	13	6579874	6648771	68898	2	56421	uc007pjz.2	-172501	ENSMUSG000	Pfkp	phosphofructokinase, platelet
chr13	6848220	6848417	198	199	6848302	29	14.55748	5.39214	11.20652	Adipose_R1_peak_	Distal Interger	13	6579874	6648771	68898	2	56421	uc007pjz.2	-199449	ENSMUSG000	Pfkp	phosphofructokinase, platelet
chr13	6848253	6848346	94	95	6848298	28	6.25023	2.67431	3.31626	Adipose_R2_peak_	Distal Interger	13	6579874	6648771	68898	2	56421	uc007pjz.2	-199482	ENSMUSG000	Pfkp	phosphofructokinase, platelet
chr13	6881916	6882033	118	119	6881980	30	7.24477	2.85875	4.21917	Adipose_R2_peak_	Distal Interger	13	6579874	6648771	68898	2	56421	uc007pjz.2	-233145	ENSMUSG000	Pfkp	phosphofructokinase, platelet
chr13	6918215	6918392	178	179	6918293	41	13.59774	3.87315	10.1803	Adipose_R2_peak_	Distal Interger	13	6579874	6648771	68898	2	56421	uc007pjz.2	-269444	ENSMUSG000	Pfkp	phosphofructokinase, platelet
chr13	6999002	6999234	233	234	6999113	56	40.97333	10.24506	37.09402	Adipose_R1_peak_	Distal Interger	13	6579874	6648771	68898	2	56421	uc007pjz.2	-350231	ENSMUSG000	Pfkp	phosphofructokinase, platelet
chr13	6999020	6999194	175	176	6999118	41	13.59774	3.87315	10.1803	Adipose_R2_peak_	Distal Interger	13	6579874	6648771	68898	2	56421	uc007pjz.2	-350249	ENSMUSG000	Pfkp	phosphofructokinase, platelet
chr13	7299584	7299818	235	236	7299734	19	7.07687	3.59476	4.08458	Adipose_R1_peak_	Distal Interger	13	6579874	6648771	68898	2	56421	uc007pjz.2	-650813	ENSMUSG000	Pfkp	phosphofructokinase, platelet
chr13	7341133	7341383	251	252	7341258	54	38.78658	9.88558	34.93639	Adipose_R1_peak_	Distal Interger	13	6579874	6648771	68898	2	56421	uc007pjz.2	-692362	ENSMUSG000	Pfkp	phosphofructokinase, platelet
chr13	7341189	7341351	163	164	7341254	36	10.5378	3.41206	7.27949	Adipose_R2_peak_	Distal Interger	13	6579874	6648771	68898	2	56421	uc007pjz.2	-692418	ENSMUSG000	Pfkp	phosphofructokinase, platelet
chr13	7400110	7400262	153	154	7400161	17	5.80341	3.23528	2.92378	Adipose_R1_peak_	Distal Interger	13	6579874	6648771	68898	2	56421	uc007pjz.2	-751339	ENSMUSG000	Pfkp	phosphofructokinase, platelet
chr13	7602263	7602544	282	283	7602403	74	61.90123	13.48034	57.80368	Adipose_R1_peak_	Distal Interger	13	8202866	8760442	557577	1	94191	uc007pkf.2	-600322	ENSMUSG000	Adarb2	adenosine deaminase, RNA-specific, B2
chr13	7602275	7602535	261	262	7602403	95	58.88104	8.8529	54.63476	Adipose_R2_peak_	Distal Interger	13	8202866	8760442	557577	1	94191	uc007pkf.2	-600331	ENSMUSG000	Adarb2	adenosine deaminase, RNA-specific, B2
chr13	7603184	7603432	249	250	7603299	26	12.14079	4.85292	8.87838	Adipose_R1_peak_	Distal Interger	13	8202866	8760442	557577	1	94191	uc007pkf.2	-599434	ENSMUSG000	Adarb2	adenosine deaminase, RNA-specific, B2
chr13	7603570	7603827	258	259	7603715	40	24.42476	7.36925	20.81738	Adipose_R1_peak_	Distal Interger	13	8202866	8760442	557577	1	94191	uc007pkf.2	-599039	ENSMUSG000	Adarb2	adenosine deaminase, RNA-specific, B2
chr13	7603647	7603827	181	182	7603731	38	11.72962	3.59649	8.4047	Adipose_R2_peak_	Distal Interger	13	8202866	8760442	557577	1	94191	uc007pkf.2	-599039	ENSMUSG000	Adarb2	adenosine deaminase, RNA-specific, B2
chr13	7897930	7898421	492	493	7898057	71	58.27042	12.94113	54.20487	Adipose_R1_peak_	Distal Interger	13	8202866	8760442	557577	1	94191	uc007pkf.2	-304445	ENSMUSG000	Adarb2	adenosine deaminase, RNA-specific, B2
chr13	7897945	7898264	320	321	7898066	47	17.60017	4.42645	14.02531	Adipose_R2_peak_	Distal Interger	13	8202866	8760442	557577	1	94191	uc007pkf.2	-304602	ENSMUSG000	Adarb2	adenosine deaminase, RNA-specific, B2
chr13	8072765	8072937	173	174	8072892	30	7.24477	2.85875	4.21917	Adipose_R2_peak_	Distal Interger	13	8202866	8760442	557577	1	94191	uc007pkf.2	-129929	ENSMUSG000	Adarb2	adenosine deaminase, RNA-specific, B2
chr13	8167824	8168039	216	217	8167935	30	15.39182	5.57188	12.01394	Adipose_R1_peak_	Distal Interger	13	8202866	8760442	557577	1	94191	uc007pkf.2	-34827	ENSMUSG000	Adarb2	adenosine deaminase, RNA-specific, B2
chr13	8349341	8349520	180	181	8349452	32	8.29282	3.04319	5.18352	Adipose_R2_peak_	Intron (uc007p	13	8202866	8760442	557577	1	94191	uc007pkf.2	146475	ENSMUSG000	Adarb2	adenosine deaminase, RNA-specific, B2
chr13	8372994	8373250	257	258	8373119	41	25.38991	7.54899	21.76305	Adipose_R1_peak_	Intron (uc007p	13	8202866	8760442	557577	1	94191	uc007pkf.2	170128	ENSMUSG000	Adarb2	adenosine deaminase, RNA-specific, B2
chr13	8373036	8373206	171	172	8373111	38	11.72962	3.59649	8.4047	Adipose_R2_peak_	Intron (uc007p	13	8202866	8760442	557577	1	94191	uc007pkf.2	170170	ENSMUSG000	Adarb2	adenosine deaminase, RNA-specific, B2
chr13	8406846	8407065	220	221	8406932	69	55.87963	12.58165	51.83661	Adipose_R1_peak_	Intron (uc007p	13	8202866	8760442	557577	1	94191	uc007pkf.2	203980	ENSMUSG000	Adarb2	adenosine deaminase, RNA-specific, B2
chr13	8943706	8943845	140	141	8943751	25	4.86612	2.39766	2.09018	Adipose_R2_peak_	Distal Interger	13	8952863	8960935	8073	1	320581	uc007pko.1	-9018	ENSMUSG000	Idi2	isopentenyl-diphosphate delta isomerase 2
chr13	8949183	8949334	152	153	8949301	18	5.95735	3.21092	3.07512	Adipose_R1_peak_	Distal Interger	13	8952863	8960935	8073	1	320581	uc007pko.1	-3529	ENSMUSG000	Idi2	isopentenyl-diphosphate delta isomerase 2
chr13	9832926	9833138	213	214	9833050	27	12.93148	5.03266	9.63827	Adipose_R1_peak_	Distal Interger	13	9684836	9765314	80479	2	66505	uc007pky.2	-67612	ENSMUSG000	Zmynd11	zinc finger, MYND domain containing 11
chr13	9833002	9833155	154	155	9833074	31	7.7623	2.95097	4.69388	Adipose_R2_peak_	Distal Interger	13	9684836	9765314	80479	2	66505	uc007pky.2	-67688	ENSMUSG000	Zmynd11	zinc finger, MYND domain containing 11
chr13	9834307	9834505	199	200	9834379	110	82.81486	12.17967	78.56991	Adipose_R1_peak_	Distal Interger	13	9684836	9765314	80479	2	66505	uc007pky.2	-68993	ENSMUSG000	Zmynd11	zinc finger, MYND domain containing 11
chr13	9853888	9854096	209	210	9854017	27	12.93148	5.03266	9.63827	Adipose_R1_peak_	Distal Interger	13	9684836	9765314	80479	2	66505	uc007pky.2	-88574	ENSMUSG000	Zmynd11	zinc finger, MYND domain containing 11

chr13	9854713	9854916	204	205	9854804	32	8.29282	3.04319	5.18352	Adipose_R2_peak_4	Distal Intergen	13	9684836	9765314	80479	2	66505	uc007pky.2	-89399	ENSMUSG000	Zmynd11	zinc finger, MYND domain containing 11
chr13	9854723	9854968	246	247	9854845	27	12.93148	5.03266	9.63827	Adipose_R1_peak_4	Distal Intergen	13	9684836	9765314	80479	2	66505	uc007pky.2	-89409	ENSMUSG000	Zmynd11	zinc finger, MYND domain containing 11
chr13	10302131	10302224	94	95	10302161	28	6.25023	2.67431	3.31626	Adipose_R2_peak_4	Intron (uc007p	13	9876613	10360803	484191	2	12671	uc007plb.1	58579	ENSMUSG000	Chrm3	cholinergic receptor, muscarinic 3, cardiac
chr13	10302143	10302299	157	158	10302219	23	9.86443	4.31371	6.70247	Adipose_R1_peak_4	Intron (uc007p	13	9876613	10360803	484191	2	12671	uc007plb.1	58504	ENSMUSG000	Chrm3	cholinergic receptor, muscarinic 3, cardiac
chr13	10421888	10422118	231	232	10422014	37	21.59274	6.83004	18.04766	Adipose_R1_peak_4	Distal Intergen	13	9876613	10360803	484191	2	12671	uc007plb.1	-61085	ENSMUSG000	Chrm3	cholinergic receptor, muscarinic 3, cardiac
chr13	10421941	10422114	174	175	10422023	36	10.5378	3.41206	7.27949	Adipose_R2_peak_4	Distal Intergen	13	9876613	10360803	484191	2	12671	uc007plb.1	-61138	ENSMUSG000	Chrm3	cholinergic receptor, muscarinic 3, cardiac
chr13	10604150	10604426	277	278	10604281	40	24.42476	7.36925	20.81738	Adipose_R1_peak_4	Distal Intergen	13	9876613	10360803	484191	2	12671	uc007plb.1	-243347	ENSMUSG000	Chrm3	cholinergic receptor, muscarinic 3, cardiac
chr13	10605445	10605755	311	312	10605602	90	79.29556	15.37864	75.07254	Adipose_R1_peak_4	Distal Intergen	13	9876613	10360803	484191	2	12671	uc007plb.1	-244642	ENSMUSG000	Chrm3	cholinergic receptor, muscarinic 3, cardiac
chr13	10605468	10605736	269	270	10605596	71	36.49133	6.63968	32.5091	Adipose_R2_peak_4	Distal Intergen	13	9876613	10360803	484191	2	12671	uc007plb.1	-244665	ENSMUSG000	Chrm3	cholinergic receptor, muscarinic 3, cardiac
chr13	10717909	10718099	191	192	10718062	18	6.42934	3.41502	3.48999	Adipose_R1_peak_4	Distal Intergen	13	9876613	10360803	484191	2	12671	uc007plb.1	-357106	ENSMUSG000	Chrm3	cholinergic receptor, muscarinic 3, cardiac
chr13	10778782	10778954	173	174	10778888	33	8.83596	3.1354	5.68652	Adipose_R2_peak_4	Distal Intergen	13	9876613	10360803	484191	2	12671	uc007plb.1	-417979	ENSMUSG000	Chrm3	cholinergic receptor, muscarinic 3, cardiac
chr13	10778787	10778983	197	198	10778905	26	12.14079	4.85292	8.87838	Adipose_R1_peak_4	Distal Intergen	13	9876613	10360803	484191	2	12671	uc007plb.1	-417984	ENSMUSG000	Chrm3	cholinergic receptor, muscarinic 3, cardiac
chr13	10788462	10788637	176	177	10788595	37	11.12817	3.50427	7.83613	Adipose_R2_peak_4	Distal Intergen	13	9876613	10360803	484191	2	12671	uc007plb.1	-427659	ENSMUSG000	Chrm3	cholinergic receptor, muscarinic 3, cardiac
chr13	10851842	10851988	147	148	10851908	22	9.13959	4.13397	6.01526	Adipose_R1_peak_4	Distal Intergen	13	9876613	10360803	484191	2	12671	uc007plb.1	-491039	ENSMUSG000	Chrm3	cholinergic receptor, muscarinic 3, cardiac
chr13	10891287	10891434	148	149	10891363	22	9.13959	4.13397	6.01526	Adipose_R1_peak_4	Distal Intergen	13	9876613	10360803	484191	2	12671	uc007plb.1	-530484	ENSMUSG000	Chrm3	cholinergic receptor, muscarinic 3, cardiac
chr13	12443438	12443636	199	200	12443535	29	14.55748	5.39214	11.20652	Adipose_R1_peak_4	Intron (uc011y	13	12439402	12459316	19915	2	56048	uc011ywf.2	15680	ENSMUSG000	Lgals8	lectin, galactose binding, soluble 8
chr13	12548583	12548719	137	138	12548647	16	5.20017	3.05554	2.38734	Adipose_R1_peak_4	Intron (uc007p	13	12565883	12606112	40230	1	67475	uc007plr.1	-17164	ENSMUSG000	Ero1lb	ERO1-like beta (S. cerevisiae)
chr13	12603423	12603688	266	267	12603569	68	54.69342	12.40192	50.66183	Adipose_R1_peak_4	Intron (uc007p	13	12565883	12606112	40230	1	67475	uc007plr.1	37540	ENSMUSG000	Ero1lb	ERO1-like beta (S. cerevisiae)
chr13	12603426	12603686	261	262	12603565	64	30.56565	5.99415	26.6807	Adipose_R2_peak_4	Intron (uc007p	13	12565883	12606112	40230	1	67475	uc007plr.1	37543	ENSMUSG000	Ero1lb	ERO1-like beta (S. cerevisiae)
chr13	12637375	12637508	134	135	12637422	30	7.24477	2.85875	4.21917	Adipose_R2_peak_4	Intron (uc007p	13	12614065	12650395	36331	2	664862	uc007plu.2	12887	NA	Gpr137b-ps	G protein-coupled receptor 137B, pseudogene
chr13	12638829	12639044	216	217	12638880	28	6.25023	2.67431	3.31626	Adipose_R2_peak_4	Intron (uc007p	13	12614065	12650395	36331	2	664862	uc007plu.2	11351	NA	Gpr137b-ps	G protein-coupled receptor 137B, pseudogene
chr13	12647135	12647243	109	110	12647149	25	4.86612	2.39766	2.09018	Adipose_R2_peak_4	Intron (uc007p	13	12614065	12650395	36331	2	664862	uc007plu.2	3152	NA	Gpr137b-ps	G protein-coupled receptor 137B, pseudogene
chr13	12701458	12701633	176	177	12701545	23	9.86443	4.31371	6.70247	Adipose_R1_peak_4	Distal Intergen	13	12614065	12650395	36331	2	664862	uc007plu.2	-51063	NA	Gpr137b-ps	G protein-coupled receptor 137B, pseudogene
chr13	12703861	12703983	123	124	12703914	29	6.74062	2.76653	3.75989	Adipose_R2_peak_4	Distal Intergen	13	12614065	12650395	36331	2	664862	uc007plu.2	-53466	NA	Gpr137b-ps	G protein-coupled receptor 137B, pseudogene
chr13	12723569	12723723	155	156	12723655	31	7.7623	2.95097	4.69388	Adipose_R2_peak_4	Distal Intergen	13	12614065	12650395	36331	2	664862	uc007plu.2	-73174	NA	Gpr137b-ps	G protein-coupled receptor 137B, pseudogene
chr13	12731755	12731922	168	169	12731845	38	11.72962	3.59649	8.4047	Adipose_R2_peak_4	Distal Intergen	13	12790818	12800079	9262	2	18812	uc007plw.3	68157	ENSMUSG000	Prl2c3	prolactin family 2, subfamily c, member 3
chr13	12736227	12736339	113	114	12736283	33	6.18641	2.4629	3.28585	Adipose_R2_peak_4	Distal Intergen	13	12790818	12800079	9262	2	18812	uc007plw.3	63740	ENSMUSG000	Prl2c3	prolactin family 2, subfamily c, member 3
chr13	12738239	12738412	174	175	12738283	26	5.31252	2.48988	2.47999	Adipose_R2_peak_4	Distal Intergen	13	12790818	12800079	9262	2	18812	uc007plw.3	61667	ENSMUSG000	Prl2c3	prolactin family 2, subfamily c, member 3
chr13	12741199	12741430	232	233	12741374	37	8.55701	2.87472	5.43811	Adipose_R2_peak_4	Distal Intergen	13	12790818	12800079	9262	2	18812	uc007plw.3	58649	ENSMUSG000	Prl2c3	prolactin family 2, subfamily c, member 3
chr13	12742074	12742219	146	147	12742123	36	7.86316	2.75295	4.79089	Adipose_R2_peak_4	Distal Intergen	13	12790818	12800079	9262	2	18812	uc007plw.3	57860	ENSMUSG000	Prl2c3	prolactin family 2, subfamily c, member 3
chr13	12743473	12743582	110	111	12743531	37	7.8386	2.70919	4.7673	Adipose_R2_peak_4	Distal Intergen	13	12790818	12800079	9262	2	18812	uc007plw.3	56497	ENSMUSG000	Prl2c3	prolactin family 2, subfamily c, member 3
chr13	12743815	12743978	164	165	12743851	36	7.62793	2.69804	4.58754	Adipose_R2_peak_4	Distal Intergen	13	12790818	12800079	9262	2	18812	uc007plw.3	56101	ENSMUSG000	Prl2c3	prolactin family 2, subfamily c, member 3
chr13	12744325	12744522	198	199	12744403	36	8.05805	2.79879	4.97918	Adipose_R2_peak_4	Distal Intergen	13	12790818	12800079	9262	2	18812	uc007plw.3	55557	ENSMUSG000	Prl2c3	prolactin family 2, subfamily c, member 3
chr13	12745200	12745522	323	324	12745300	35	6.49265	2.47002	3.54935	Adipose_R2_peak_4	Distal Intergen	13	12790818	12800079	9262	2	18812	uc007plw.3	54557	ENSMUSG000	Prl2c3	prolactin family 2, subfamily c, member 3
chr13	12751716	12752061	346	347	12751984	35	8.54397	2.95966	5.42535	Adipose_R2_peak_4	Distal Intergen	13	12790818	12800079	9262	2	18812	uc007plw.3	48018	ENSMUSG000	Prl2c3	prolactin family 2, subfamily c, member 3
chr13	12752862	12752972	111	112	12752911	33	8.83596	3.1354	5.68652	Adipose_R2_peak_4	Distal Intergen	13	12790818	12800079	9262	2	18812	uc007plw.3	47107	ENSMUSG000	Prl2c3	prolactin family 2, subfamily c, member 3
chr13	12753821	12753928	108	109	12753860	26	5.31252	2.48988	2.47999	Adipose_R2_peak_4	Distal Intergen	13	12790818	12800079	9262	2	18812	uc007plw.3	46151	ENSMUSG000	Prl2c3	prolactin family 2, subfamily c, member 3
chr13	12762877	12763079	203	204	12762987	36	10.17775	3.31933	6.94706	Adipose_R2_peak_4	Distal Intergen	13	12790818	12800079	9262	2	18812	uc007plw.3	37000	ENSMUSG000	Prl2c3	prolactin family 2, subfamily c, member 3
chr13	12764301	12764427	127	128	12764369	28	6.25023	2.67431	3.31626	Adipose_R2_peak_4	Distal Intergen	13	12790818	12800079	9262	2	18812	uc007plw.3	35652	ENSMUSG000	Prl2c3	prolactin family 2, subfamily c, member 3
chr13	12766631	12766894	264	265	12766676	25	4.86612	2.39766	2.09018	Adipose_R2_peak_4	Distal Intergen	13	12790818	12800079	9262	2	18812	uc007plw.3	33185	ENSMUSG000	Prl2c3	prolactin family 2, subfamily c, member 3
chr13	12767422	12767899	478	479	12767546	28	6.25023	2.67431	3.31626	Adipose_R2_peak_4	Distal Intergen	13	12790818	12800079	9262	2	18812	uc007plw.3	32180	ENSMUSG000	Prl2c3	prolactin family 2, subfamily c, member 3
chr13	12767640	12767807	168	169	12767778	24	8.0285	3.49815	4.98517	Adipose_R1_peak_4	Distal Intergen	13	12790818	12800079	9262	2	18812	uc007plw.3	32272	ENSMUSG000	Prl2c3	prolactin family 2, subfamily c, member 3
chr13	12768496	12768797	302	303	12768572	34	9.39139	3.22762	6.20474	Adipose_R2_peak_4	Distal Intergen	13	12790818	12800079	9262	2	18812	uc007plw.3	31282	ENSMUSG000	Prl2c3	prolactin family 2, subfamily c, member 3
chr13	12769608	12770069	462	463	12769973	32	8.29282	3.04319	5.18352	Adipose_R2_peak_4	Distal Intergen	13	12790818	12800079	9262	2	18812	uc007plw.3	30010	ENSMUSG000	Prl2c3	prolactin family 2, subfamily c, member 3
chr13	12777234	12777487	254	255	12777386	44	13.01451	3.55454	9.62511	Adipose_R2_peak_4	Distal Intergen	13	12790818	12800079	9262	2	18812	uc007plw.3	22592	ENSMUSG000	Prl2c3	prolactin family 2, subfamily c, member 3
chr13	12777822	12778074	253	254	12777941	40	10.83299	3.26614	7.56556	Adipose_R2_peak_4	Distal Intergen	13	12790818	12800079	9262	2	18812	uc007plw.3	22005	ENSMUSG000	Prl2c3	prolactin family 2, subfamily c, member 3
chr13	12778660	12778851	192	193	12778761	32	6.62623	2.60764	3.67758	Adipose_R2_peak_4	Distal Intergen	13	12790818	12800079	9262	2	18812	uc007plw.3	21228	ENSMUSG000	Prl2c3	prolactin family 2, subfamily c, member 3
chr13	12779654	12779765	112	113	12779714	30	5.82372	2.4762	2.93718	Adipose_R2_peak_4	Distal Intergen	13	12790818	12800079	9262	2	18812	uc007plw.3	20314	ENSMUSG000	Prl2c3	prolactin family 2, subfamily c, member 3
chr13	12780214	12780633	420	421	12780272	34	7.65765	2.7847	4.61648	Adipose_R2_peak_4	Distal Intergen	13	12790818	12800079	9262	2	18812	uc007plw.3	19446	ENSMUSG000	Prl2c3	prolactin family 2, subfamily c, member 3
chr13	12781488	12781696	209	210	12781533	36	7.04613	2.56408	4.05389	Adipose_R2_peak_4	Distal Intergen	13	12790818	12800079	9262	2	18812	uc007plw.3	18383	ENSMUSG000	Prl2c3	prolactin family 2, subfamily c, member 3
chr13	12783386	12783511	126	127	12783460	31	6.21077	2.5397	3.30934	Adipose_R2_peak_4	Distal Intergen	13	12790818	12800079	9262	2	18812	uc007plw.3	16568	ENSMUSG000	Prl2c3	prolactin family 2, subfamily c, member 3
chr13	12783644	12783753	110	111	12783687	30	5.32986	2.34784	2.49645	Adipose_R2_peak_4	Distal Intergen	13	12790818	12800079	9262	2	18812	uc007plw.3	16326	ENSMUSG000	Prl2c3	prolactin family 2, subfamily c, member 3
chr13	12784151	12784839	689	690	12784307	36	7.21047	2.60166	4.21202	Adipose_R2_peak_4	Distal Intergen	13	12790818	12800079	9262	2	18812	uc007plw.3	15240	ENSMUSG000	Prl2c3	prolactin family 2, subfamily c, member 3
chr13	12784972	12785074	103	104	12784998	31	4.92467	2.21558	2.14637	Adipose_R2_peak_4	Distal Intergen	13	12790818	12800079	9262	2	18812	uc007plw.3	15005	ENSMUSG000	Prl2c3	prolactin family 2, subfamily c, member 3
chr13	12788658	12788794	137	138	12788720	32	5.41651	2.3076	2.58023	Adipose_R2_peak_4	Downstream (	13	12790818	12800079	9262	2	18812	uc007plw.3	11285	ENSMUSG000	Prl2c3	prolactin family 2, subfamily c, member 3
chr13	12789570	12789877	308	309	12789706	41	7.80299	2.56765	4.73354	Adipose_R2_peak_4	Downstream (	13	12790818	12800079	9262	2	18812	uc007plw.3	10202	ENSMUSG000	Prl2c3	prolactin family 2, subfamily c, member 3
chr13	12790511	12790605	95	96	12790547	29	5.48353	2.42229	2.64474	Adipose_R2_peak_4	Downstream (	13	12790818	12800079	9262	2	18812	uc007plw.3	9474	ENSMUSG000	Prl2c3	prolactin family 2, subfamily c, member 3
chr13	12794579	12794676	98	99	12794624	32	6.23316	2.50883	3.31626	Adipose_R2_peak_4	Intron (uc007p	13	12790818	12800079	9262	2	18812	uc007plw.3	5403	ENSMUSG000	Prl2c3	prolactin family 2, subfamily c, member 3
chr13	12795226	12795460	235	236	12795334	43	9.18188	2.78325	6.02041	Adipose_R2_peak_4	Intron (uc007p	13	12790818	12800079	9262	2	18812	uc007plw.3	4619	ENSMUSG000	Prl2c3	prolactin family 2, subfamily c, member 3
chr13	12795856	12795995	140	141	12795914	29	6.01251	2.56514	3.11855	Adipose_R2_peak_4	Intron (uc007p	13	12790818	12800079	9262	2	18812	uc007plw.3	4084	ENSMUSG000	Prl2c3	prolactin family 2, subfamily c, member 3
chr13	12796749	12797060	312	313	12796851	32	7.48461	2.82843	4.44979	Adipose_R2_peak_4	Exon (uc007pl	13	12790818	12800079	9262	2	18812	uc007plw.3	3019	ENSMUSG000	Prl2c3	prolactin family 2, subfamily c, member 3
chr13	12797526	12797701	176	177	12797621	37	10.07772	3.23962	6.85034	Adipose_R2_peak_4	Promoter (2-3	13	12790818	12800079	9262	2	18812	uc007plw.3	2378	ENSMUSG000	Prl2c3	prolactin family 2, subfamily c, member 3
chr13	12798484	12798632	149	150	12798540	32	7.69305	2.88315	4.65092	Adipose_R2_peak_4	Promoter (1-2	13	12790818	12800079	9262	2	18812	uc007plw.3	1447	ENSMUSG000	Prl2c3	prolactin family 2, subfamily c, member 3
chr13	12798934	12799404	471	472	12799124	36	9.56235	3.16385	6.36958	Adipose_R2_peak_4	Promoter (<=1	13	12790818	12800079	9262	2	18812	uc007plw.3	675	ENSMUSG000	Prl2c3	prolactin family 2, subfamily c, member 3
chr13	12800703	12800992	290	291	12800750	35	6.9089	2.5666	3.92198	Adipose_R2_peak_4	Promoter (<=1	13	12790818	12800079	9262	2	18812	uc007plw.3	-624	ENSMUSG000	Prl2c3	prolactin family 2, subfamily c, member 3
chr13	12801716	12802063	348	349	12801865	42	13.87586	3.87548	10.45133	Adipose_R2_peak_4	Promoter (1-2	13	12790818	12800079	9262	2	18812	uc007plw.3	-1637	ENSMUSG000	Prl2c3	prolactin family 2, subfamily c, member 3
chr13	12802197	12802894	698	699	12802318	33	8.60803	3.0748	5.48667	Adipose_R2_peak_4	Promoter (2-3	13	12790818	12800079	9262	2	18812	uc007plw.3	-2118	ENSMUSG000	Prl2c3	prolactin family 2, subfamily c, member 3
chr13	12806458	12806589	132	133	12806572	29	6.16166	2.60592	3.26193	Adipose_R2_peak_4	Distal Intergen	13	12790818	12800079	9262	2	18812	uc007plw.3	-6379	ENSMUSG000	Prl2c3	prolactin family 2, subfamily c, member 3
chr13	12806497	12806691	195	196	12806539	25	6.58335	2.932	3.64169	Adipose_R1_peak_4	Distal Intergen	13	12790818	12800079	9262	2	18812	uc007plw.3	-6418	ENSMUSG000	Prl2c3	prolactin family 2, subfamily c, member 3
chr13	12807928	12808185	258	259	12808090	30	6.59822	2.68209	3.65076	Adipose_R2_peak_4	Distal Intergen	13	12790818	12800079	9262	2	18812	uc007plw.3	-7849	ENSMUSG000	Prl2c3	prolactin family 2, subfamily c, member 3
chr13	12808413	12809190	778	779	12808437	28	5.35168	2.42282	2.51755	Adipose_R2_peak_4	Distal Intergen	13	12790818	12800079	9262	2	18812	uc007plw.3	-8334	ENSMUSG000	Prl2c3	prolactin family 2, subfamily c, member 3
chr13	12812685	12812793	109	110	12812758	31	6.61682	2.64497	3.66863	Adipose_R2_peak_4	Distal Intergen	13	12790818	12800079	9262	2	18812	uc007plw.3	-12606	ENSMUSG000	Prl2c3	prolactin family 2, subfamily c, member 3
chr13	12813906	12814380	475	476	12814119	30	6.68314	2.70503	3.73247	Adipose_R2_peak_4	Distal Intergen	13	12790818	12800079	9262	2	18812	uc007plw.3	-13827	ENSMUSG000	Prl2c3	prolactin family 2, subfamily c, member 3
chr13	12814864	12815257	394	395	12814942	30	6.48137	2.65064	3.53843	Adipose_R2_peak_4	Distal Intergen	13	12790818	12800079	9262	2	18812	uc007plw.3	-14785	ENSMUSG000	Prl2c3	prolactin family 2, subfamily c, member 3
chr13	12817155	12817273	119	120	12817217	30	6.98809	2.78806	3.9979	Adipose_R2_peak_4	Distal Intergen	13	12790818	12800079	9262	2	18812	uc007plw.3	-17076	ENSMUSG000	Prl2c3	prolactin family 2, subfamily c, member 3
chr13	12818185	12818333	149	150	12818317	28	5.66324	2.50905	2.81749	Adipose_R2_peak_4	Distal Intergen	13	12790818	12800079	9262	2	18812	uc007plw.3	-18106	ENSMUSG000	Prl2c3	prolactin family 2, subfamily c, member 3
chr13	12819350	12819465	116	117	12819356	27	5.05113	2.37574	2.26864	Adipose_R2_peak_4	Distal Intergen	13	12790818	12800079	9262	2	18812	uc007plw.3	-19271	ENSMUSG000	Prl2c3	prolactin family 2, subfamily c, member 3
chr13	12820394	12820559	166	167	12820506	27	5.75152	2.57558	2.88921	Adipose_R2_peak_4	Distal Intergen	13	12790818	12800079	9262	2	18812	uc007plw.3	-20315	ENSMUSG000	Prl2c3	prolactin family 2, subfamily c, member 3
chr13	12822596	12822755	160	161	12822623	28	6.25023	2.67431	3.31626	Adipose_R2_peak_4	Distal Intergen	13	12790818	12800079	9262	2	18812	uc007plw.3	-22517	ENSMUSG000	Prl2c3	prolactin family 2, subfamily c, member 3
chr13	12825831	12826058	228	229	12825874	27	5.63935	2.54321	2.79469	Adipose_R2_peak_4	Distal Intergen	13	12790818	12800079	9262	2	18812	uc007plw.3	-25752	ENSMUSG000	Prl2c3	prolactin family 2, subfamily c, member 3
chr13	12827000	12827335	336	337	12827102	36	10.5378	3.41206	7.27949	Adipose_R2_peak_4	Distal Intergen	13	12790818	12800079	9262	2	18812	uc007plw.3	-26921	ENSMUSG000	Prl2c3	prolactin family 2, subfamily c, member 3
chr13	12829108	12829260	153	154	12829181	33	8.83596	3.1354	5.68652	Adipose_R2_peak_4	Distal Intergen	13	12790818	12800079	9262	2	18812	uc007plw.3	-29029	ENSMUSG000	Prl2c3	prolactin family 2, subfamily c, member 3
chr13	12829419	12829660	242	243	12829529	32	6.73884	2.6362	3.75989	Adipose_R2_peak_4	Distal Intergen	13	12790818	12800079	9262	2	18812	uc007plw.3	-29340	ENSMUSG000	Prl2c3	prolactin family 2, subfamily c, member 3
chr13	12829857	12830038	182	183	12829883	26	5.31252	2.48988	2.47999	Adipose_R2_peak_4	Distal Intergen	13	12790818	12800079	9262	2	18812	uc007plw.3	-29778	ENSMUSG000	Prl2c3	prolactin family 2, subfamily c, member 3
chr13	12830691	12830839	149	150	12830755	29	5.79611	2.50636	2.9102	Adipose_R2_peak_4	Distal Intergen	13	12790818	12800079	9262	2	18812	uc007plw.3	-30612	ENSMUSG000	Prl2c3	prolactin family 2, subfamily c, member 3
chr13	12831280	12831918	639	640	12831387	28	6.25023	2.67431	3.31626	Adipose_R2_peak_4	Distal Intergen	13	12790818	12800079	9262	2	18812	uc007plw.3	-31201	ENSMUSG000	Prl2c3	prolactin family 2, subfamily c, member 3
chr13	12835503	12835691	189	190	12835580	30	7.24477	2.85875	4.21917	Adipose_R2_peak_4	Distal Intergen	13	12790818	12800079	9262	2	18812	uc007plw.3	-35424	ENSMUSG000	Prl2c3	prolactin family 2, subfamily c, member 3
chr13	12838083	12838246	164	165	12838084	26	5.31252	2.48988	2.47999	Adipose_R2_peak_4	Distal Intergen	13	12790818	12800079	9262	2	18812	uc007plw.3	-38004	ENSMUSG000	Prl2c3	prolactin family 2, subfamily c, member 3
chr13	12842651	12842838	188	189	12842771	34	8.21596	2.92409	5.13058	Adipose_R2_peak_4	Distal Intergen	13	12790818	12800079	9262	2	18812	uc007plw.3	-42572	ENSMUSG000	Prl2c3	prolactin family 2, subfamily c, member 3
chr13	12843273	12843481	209	210	12843443	27	5.77405	2.5821	2.88921	Adipose_R2_peak_4	Distal Intergen	13	12790818	12800079	9262	2	18812	uc007plw.3	-43194	ENSMUSG000	Prl2c3	prolactin family 2, subfamily c, member 3
chr13	12844836	12844937	102	103	12844889	30	6.71732	2.71429	3.75989	Adipose_R2_peak_4	Distal Intergen	13	12790818	12800079	9262	2	18812	uc007plw.3	-44757	ENSMUSG000	Prl2c3	prolactin family 2, subfamily c, member 3
chr13	12845428	12845964	537	538	12845564	28	5.13686	2.36392	2.35124	Adipose_R2_peak_4	Distal Intergen	13	12790818	12800079	9262	2	18812	uc007plw.3	-45349	ENSMUSG000	Prl2c3	prolactin family 2, subfamily c, member 3
chr13	12848425	12849440	1016	1017	12848470	34	8.62515	3.02816	5.5031	Adipose_R2_peak_4	Distal Intergen	13	12790818	12800079	9262	2	18812	uc007plw.3	-48346	ENSMUSG000	Prl2c3	prolactin family 2, subfamily c, member 3
chr13	12851001	12851396	396	397	12851062	29	6.63838	2.7379	3.68937	Adipose_R2_peak_4	Distal Intergen	13	12790818	12800079	9262	2	18812	uc007plw.3	-50922	ENSMUSG000	Prl2c3	prolactin family 2, subfamily c, member 3

chr13	12853881	12854027	147	148	12853944	29	6.66639	2.74573	3.71635	Adipose_R2_peak_	Distal Interger	13	12790818	12800079	9262	2	18812	uc007plw.3	-53802	ENSMUSG000	Prl2c3	prolactin family 2, subfamily c, member 3
chr13	12854621	12854923	303	304	12854734	32	7.77947	2.90597	4.71059	Adipose_R2_peak_	Distal Interger	13	12790818	12800079	9262	2	18812	uc007plw.3	-54542	ENSMUSG000	Prl2c3	prolactin family 2, subfamily c, member 3
chr13	12858594	12858810	217	218	12858725	37	8.44408	2.84842	5.32928	Adipose_R2_peak_	Distal Interger	13	12790818	12800079	9262	2	18812	uc007plw.3	-58515	ENSMUSG000	Prl2c3	prolactin family 2, subfamily c, member 3
chr13	12859488	12859916	429	430	12859561	27	5.41849	2.4799	2.58217	Adipose_R2_peak_	Distal Interger	13	12790818	12800079	9262	2	18812	uc007plw.3	-59409	ENSMUSG000	Prl2c3	prolactin family 2, subfamily c, member 3
chr13	12860450	12860652	203	204	12860510	30	6.14381	2.56058	3.24465	Adipose_R2_peak_	Distal Interger	13	12790818	12800079	9262	2	18812	uc007plw.3	-60371	ENSMUSG000	Prl2c3	prolactin family 2, subfamily c, member 3
chr13	12861626	12862096	471	472	12861778	29	6.74062	2.76653	3.75989	Adipose_R2_peak_	Distal Interger	13	12790818	12800079	9262	2	18812	uc007plw.3	-61547	ENSMUSG000	Prl2c3	prolactin family 2, subfamily c, member 3
chr13	12862300	12862455	156	157	12862384	30	7.24477	2.85875	4.21917	Adipose_R2_peak_	Distal Interger	13	12790818	12800079	9262	2	18812	uc007plw.3	-62221	ENSMUSG000	Prl2c3	prolactin family 2, subfamily c, member 3
chr13	12863182	12863384	203	204	12863275	36	10.17775	3.31933	6.94706	Adipose_R2_peak_	Distal Interger	13	12790818	12800079	9262	2	18812	uc007plw.3	-63103	ENSMUSG000	Prl2c3	prolactin family 2, subfamily c, member 3
chr13	12863901	12864063	163	164	12864036	30	6.54829	2.66863	3.60286	Adipose_R2_peak_	Distal Interger	13	12790818	12800079	9262	2	18812	uc007plw.3	-63822	ENSMUSG000	Prl2c3	prolactin family 2, subfamily c, member 3
chr13	12864722	12864867	146	147	12864756	29	5.38717	2.39655	2.55175	Adipose_R2_peak_	Distal Interger	13	12790818	12800079	9262	2	18812	uc007plw.3	-64643	ENSMUSG000	Prl2c3	prolactin family 2, subfamily c, member 3
chr13	12865305	12865844	540	541	12865401	31	5.56081	2.37427	2.71889	Adipose_R2_peak_	Distal Interger	13	12790818	12800079	9262	2	18812	uc007plw.3	-65226	ENSMUSG000	Prl2c3	prolactin family 2, subfamily c, member 3
chr13	12867257	12867596	340	341	12867383	30	5.62133	2.42334	2.77743	Adipose_R2_peak_	Distal Interger	13	12790818	12800079	9262	2	18812	uc007plw.3	-67178	ENSMUSG000	Prl2c3	prolactin family 2, subfamily c, member 3
chr13	12869202	12869355	154	155	12869274	42	11.37571	3.29086	8.07606	Adipose_R2_peak_	Distal Interger	13	12790818	12800079	9262	2	18812	uc007plw.3	-69123	ENSMUSG000	Prl2c3	prolactin family 2, subfamily c, member 3
chr13	12870486	12870699	214	215	12870641	34	8.37021	2.96313	5.25771	Adipose_R2_peak_	Distal Interger	13	12790818	12800079	9262	2	18812	uc007plw.3	-70407	ENSMUSG000	Prl2c3	prolactin family 2, subfamily c, member 3
chr13	12871113	12871275	163	164	12871164	37	9.87177	3.18898	6.6686	Adipose_R2_peak_	Distal Interger	13	12790818	12800079	9262	2	18812	uc007plw.3	-71034	ENSMUSG000	Prl2c3	prolactin family 2, subfamily c, member 3
chr13	12871796	12871975	180	181	12871883	39	11.12724	3.3883	7.83613	Adipose_R2_peak_	Distal Interger	13	12790818	12800079	9262	2	18812	uc007plw.3	-71717	ENSMUSG000	Prl2c3	prolactin family 2, subfamily c, member 3
chr13	12873645	12874050	406	407	12873815	38	10.25361	3.23052	7.02077	Adipose_R2_peak_	Distal Interger	13	12790818	12800079	9262	2	18812	uc007plw.3	-73566	ENSMUSG000	Prl2c3	prolactin family 2, subfamily c, member 3
chr13	12874551	12874734	184	185	12874633	33	6.64655	2.57506	3.69708	Adipose_R2_peak_	Distal Interger	13	12790818	12800079	9262	2	18812	uc007plw.3	-74472	ENSMUSG000	Prl2c3	prolactin family 2, subfamily c, member 3
chr13	12875233	12875519	287	288	12875319	36	9.48926	3.14563	6.29891	Adipose_R2_peak_	Distal Interger	13	12790818	12800079	9262	2	18812	uc007plw.3	-75154	ENSMUSG000	Prl2c3	prolactin family 2, subfamily c, member 3
chr13	12876711	12876846	136	137	12876748	32	6.46643	2.56731	3.52438	Adipose_R2_peak_	Distal Interger	13	12790818	12800079	9262	2	18812	uc007plw.3	-76632	ENSMUSG000	Prl2c3	prolactin family 2, subfamily c, member 3
chr13	12876997	12877104	108	109	12877052	34	7.45157	2.73398	4.41874	Adipose_R2_peak_	Distal Interger	13	12790818	12800079	9262	2	18812	uc007plw.3	-76918	ENSMUSG000	Prl2c3	prolactin family 2, subfamily c, member 3
chr13	12878009	12878135	127	128	12878020	27	5.06046	2.37837	2.27748	Adipose_R2_peak_	Distal Interger	13	12790818	12800079	9262	2	18812	uc007plw.3	-77930	ENSMUSG000	Prl2c3	prolactin family 2, subfamily c, member 3
chr13	12879767	12880254	488	489	12879869	37	9.23819	3.03563	6.07441	Adipose_R2_peak_	Distal Interger	13	12790818	12800079	9262	2	18812	uc007plw.3	-79688	ENSMUSG000	Prl2c3	prolactin family 2, subfamily c, member 3
chr13	12880474	12881397	924	925	12880539	36	8.88503	2.99694	5.73396	Adipose_R2_peak_	Distal Interger	13	12790818	12800079	9262	2	18812	uc007plw.3	-80395	ENSMUSG000	Prl2c3	prolactin family 2, subfamily c, member 3
chr13	12881689	12881972	284	285	12881762	36	9.26961	3.09118	6.10505	Adipose_R2_peak_	Distal Interger	13	12790818	12800079	9262	2	18812	uc007plw.3	-81610	ENSMUSG000	Prl2c3	prolactin family 2, subfamily c, member 3
chr13	12882328	12882585	258	259	12882455	29	5.90327	2.53541	3.01343	Adipose_R2_peak_	Distal Interger	13	12790818	12800079	9262	2	18812	uc007plw.3	-82249	ENSMUSG000	Prl2c3	prolactin family 2, subfamily c, member 3
chr13	12882692	12883030	339	340	12882816	30	6.7897	2.73393	3.80744	Adipose_R2_peak_	Distal Interger	13	12790818	12800079	9262	2	18812	uc007plw.3	-82613	ENSMUSG000	Prl2c3	prolactin family 2, subfamily c, member 3
chr13	12884314	12884561	248	249	12884425	30	6.57279	2.67523	3.62665	Adipose_R2_peak_	Distal Interger	13	12790818	12800079	9262	2	18812	uc007plw.3	-84235	ENSMUSG000	Prl2c3	prolactin family 2, subfamily c, member 3
chr13	12886236	12886360	125	126	12886303	33	4.79146	2.1322	2.06474	Adipose_R2_peak_	Distal Interger	13	12790818	12800079	9262	2	18812	uc007plw.3	-86157	ENSMUSG000	Prl2c3	prolactin family 2, subfamily c, member 3
chr13	12886991	12887099	109	110	12887070	26	4.90698	2.37136	2.1295	Adipose_R2_peak_	Distal Interger	13	12790818	12800079	9262	2	18812	uc007plw.3	-86912	ENSMUSG000	Prl2c3	prolactin family 2, subfamily c, member 3
chr13	12887642	12887880	239	240	12887741	33	7.09729	2.68666	4.1027	Adipose_R2_peak_	Distal Interger	13	12790818	12800079	9262	2	18812	uc007plw.3	-87563	ENSMUSG000	Prl2c3	prolactin family 2, subfamily c, member 3
chr13	12888684	12888862	179	180	12888769	33	7.74453	2.85018	4.69388	Adipose_R2_peak_	Distal Interger	13	12790818	12800079	9262	2	18812	uc007plw.3	-88605	ENSMUSG000	Prl2c3	prolactin family 2, subfamily c, member 3
chr13	12889016	12889864	849	850	12889129	32	6.92732	2.68428	3.93986	Adipose_R2_peak_	Distal Interger	13	12790818	12800079	9262	2	18812	uc007plw.3	-88937	ENSMUSG000	Prl2c3	prolactin family 2, subfamily c, member 3
chr13	12890136	12890391	256	257	12890340	30	6.67406	2.70257	3.72371	Adipose_R2_peak_	Distal Interger	13	12790818	12800079	9262	2	18812	uc007plw.3	-90057	ENSMUSG000	Prl2c3	prolactin family 2, subfamily c, member 3
chr13	12891596	12891918	323	324	12891640	33	7.21493	2.71609	4.21614	Adipose_R2_peak_	Distal Interger	13	12790818	12800079	9262	2	18812	uc007plw.3	-91517	ENSMUSG000	Prl2c3	prolactin family 2, subfamily c, member 3
chr13	12893244	12893761	518	519	12893301	32	7.74177	2.89601	4.69388	Adipose_R2_peak_	Distal Interger	13	12790818	12800079	9262	2	18812	uc007plw.3	-93165	ENSMUSG000	Prl2c3	prolactin family 2, subfamily c, member 3
chr13	12894620	12895193	574	575	12894689	33	8.39348	3.01827	5.28012	Adipose_R2_peak_	Distal Interger	13	12790818	12800079	9262	2	18812	uc007plw.3	-94541	ENSMUSG000	Prl2c3	prolactin family 2, subfamily c, member 3
chr13	12896115	12896269	155	156	12896160	27	5.42376	2.4814	2.58701	Adipose_R2_peak_	Distal Interger	13	12790818	12800079	9262	2	18812	uc007plw.3	-96036	ENSMUSG000	Prl2c3	prolactin family 2, subfamily c, member 3
chr13	12899040	12899267	228	229	12899108	30	6.99763	2.79068	4.00693	Adipose_R2_peak_	Distal Interger	13	12790818	12800079	9262	2	18812	uc007plw.3	-98961	ENSMUSG000	Prl2c3	prolactin family 2, subfamily c, member 3
chr13	12899428	12899655	228	229	12899497	34	9.0215	3.13057	5.8656	Adipose_R2_peak_	Distal Interger	13	12790818	12800079	9262	2	18812	uc007plw.3	-99349	ENSMUSG000	Prl2c3	prolactin family 2, subfamily c, member 3
chr13	12899976	12900483	508	509	12900204	30	6.91223	2.76731	3.92513	Adipose_R2_peak_	Distal Interger	13	12790818	12800079	9262	2	18812	uc007plw.3	-99897	ENSMUSG000	Prl2c3	prolactin family 2, subfamily c, member 3
chr13	12900768	12900914	147	148	12900789	19	4.75893	2.65345	2.01946	Adipose_R1_peak_	Distal Interger	13	12790818	12800079	9262	2	18812	uc007plw.3	-100689	ENSMUSG000	Prl2c3	prolactin family 2, subfamily c, member 3
chr13	12900793	12900926	134	135	12900905	28	5.9449	2.58787	3.05369	Adipose_R2_peak_	Distal Interger	13	12790818	12800079	9262	2	18812	uc007plw.3	-100714	ENSMUSG000	Prl2c3	prolactin family 2, subfamily c, member 3
chr13	12901733	12901873	141	142	12901823	29	4.91688	2.27211	2.13899	Adipose_R2_peak_	Distal Interger	13	12790818	12800079	9262	2	18812	uc007plw.3	-101654	ENSMUSG000	Prl2c3	prolactin family 2, subfamily c, member 3
chr13	12903252	12903449	198	199	12903373	30	6.37298	2.62159	3.4342	Adipose_R2_peak_	Distal Interger	13	12996124	13005330	9207	2	18811	uc007plx.1	101881	ENSMUSG000	Prl2c2	prolactin family 2, subfamily c, member 2
chr13	12904511	12904739	229	230	12904550	33	7.09729	2.68666	4.1027	Adipose_R2_peak_	Distal Interger	13	12996124	13005330	9207	2	18811	uc007plx.1	100591	ENSMUSG000	Prl2c2	prolactin family 2, subfamily c, member 2
chr13	12905452	12905642	191	192	12905565	34	6.35907	2.47115	3.42099	Adipose_R2_peak_	Distal Interger	13	12996124	13005330	9207	2	18811	uc007plx.1	99688	ENSMUSG000	Prl2c2	prolactin family 2, subfamily c, member 2
chr13	12912512	12912747	236	237	12912615	33	8.83596	3.1354	5.68652	Adipose_R2_peak_	Distal Interger	13	12996124	13005330	9207	2	18811	uc007plx.1	92583	ENSMUSG000	Prl2c2	prolactin family 2, subfamily c, member 2
chr13	12913161	12913637	477	478	12913203	28	5.55735	2.47963	2.71575	Adipose_R2_peak_	Distal Interger	13	12996124	13005330	9207	2	18811	uc007plx.1	91693	ENSMUSG000	Prl2c2	prolactin family 2, subfamily c, member 2
chr13	12915260	12915614	355	356	12915305	30	6.83874	2.74727	3.85468	Adipose_R2_peak_	Distal Interger	13	12996124	13005330	9207	2	18811	uc007plx.1	89716	ENSMUSG000	Prl2c2	prolactin family 2, subfamily c, member 2
chr13	12917731	12918176	446	447	12917841	39	12.34187	3.68871	8.98501	Adipose_R2_peak_	Distal Interger	13	12996124	13005330	9207	2	18811	uc007plx.1	87154	ENSMUSG000	Prl2c2	prolactin family 2, subfamily c, member 2
chr13	12918470	12918954	485	486	12918538	28	5.05769	2.34232	2.27475	Adipose_R2_peak_	Distal Interger	13	12996124	13005330	9207	2	18811	uc007plx.1	86376	ENSMUSG000	Prl2c2	prolactin family 2, subfamily c, member 2
chr13	12919134	12919601	468	469	12919307	31	6.96355	2.73614	3.97439	Adipose_R2_peak_	Distal Interger	13	12996124	13005330	9207	2	18811	uc007plx.1	85729	ENSMUSG000	Prl2c2	prolactin family 2, subfamily c, member 2
chr13	12920362	12921025	664	665	12920661	38	7.96562	2.70127	4.88978	Adipose_R2_peak_	Distal Interger	13	12996124	13005330	9207	2	18811	uc007plx.1	84305	ENSMUSG000	Prl2c2	prolactin family 2, subfamily c, member 2
chr13	12921194	12921333	140	141	12921219	26	5.31252	2.48988	2.47999	Adipose_R2_peak_	Distal Interger	13	12996124	13005330	9207	2	18811	uc007plx.1	83997	ENSMUSG000	Prl2c2	prolactin family 2, subfamily c, member 2
chr13	12923670	12924103	434	435	12923832	37	9.10033	3.00274	5.94135	Adipose_R2_peak_	Distal Interger	13	12996124	13005330	9207	2	18811	uc007plx.1	81227	ENSMUSG000	Prl2c2	prolactin family 2, subfamily c, member 2
chr13	12924462	12924655	194	195	12924544	34	8.86206	3.08918	5.7118	Adipose_R2_peak_	Distal Interger	13	12996124	13005330	9207	2	18811	uc007plx.1	80675	ENSMUSG000	Prl2c2	prolactin family 2, subfamily c, member 2
chr13	12924839	12924979	141	142	12924921	34	8.48629	2.99266	5.36945	Adipose_R2_peak_	Distal Interger	13	12996124	13005330	9207	2	18811	uc007plx.1	80351	ENSMUSG000	Prl2c2	prolactin family 2, subfamily c, member 2
chr13	12925096	12925308	213	214	12925218	37	10.39978	3.31962	7.16237	Adipose_R2_peak_	Distal Interger	13	12996124	13005330	9207	2	18811	uc007plx.1	80022	ENSMUSG000	Prl2c2	prolactin family 2, subfamily c, member 2
chr13	12925859	12926034	176	177	12925912	26	4.90978	2.37217	2.13225	Adipose_R2_peak_	Distal Interger	13	12996124	13005330	9207	2	18811	uc007plx.1	79296	ENSMUSG000	Prl2c2	prolactin family 2, subfamily c, member 2
chr13	12926778	12927380	603	604	12926925	38	10.74386	3.3498	7.47929	Adipose_R2_peak_	Distal Interger	13	12996124	13005330	9207	2	18811	uc007plx.1	77950	ENSMUSG000	Prl2c2	prolactin family 2, subfamily c, member 2
chr13	12927584	12927851	268	269	12927645	29	6.17781	2.61035	3.27753	Adipose_R2_peak_	Distal Interger	13	12996124	13005330	9207	2	18811	uc007plx.1	77479	ENSMUSG000	Prl2c2	prolactin family 2, subfamily c, member 2
chr13	12928093	12928428	336	337	12928196	34	8.53945	3.00623	5.42092	Adipose_R2_peak_	Distal Interger	13	12996124	13005330	9207	2	18811	uc007plx.1	76902	ENSMUSG000	Prl2c2	prolactin family 2, subfamily c, member 2
chr13	12929307	12929529	223	224	12929386	28	5.792	2.54498	2.90663	Adipose_R2_peak_	Distal Interger	13	12996124	13005330	9207	2	18811	uc007plx.1	75801	ENSMUSG000	Prl2c2	prolactin family 2, subfamily c, member 2
chr13	12929695	12929819	125	126	12929710	27	5.35473	2.46172	2.52051	Adipose_R2_peak_	Distal Interger	13	12996124	13005330	9207	2	18811	uc007plx.1	75511	ENSMUSG000	Prl2c2	prolactin family 2, subfamily c, member 2
chr13	12930314	12930469	156	157	12930356	27	5.27029	2.43771	2.47974	Adipose_R2_peak_	Distal Interger	13	12996124	13005330	9207	2	18811	uc007plx.1	74861	ENSMUSG000	Prl2c2	prolactin family 2, subfamily c, member 2
chr13	12930583	12930906	324	325	12930639	30	6.62659	2.68974	3.67792	Adipose_R2_peak_	Distal Interger	13	12996124	13005330	9207	2	18811	uc007plx.1	74424	ENSMUSG000	Prl2c2	prolactin family 2, subfamily c, member 2
chr13	12931027	12931303	277	278	12931115	34	8.76657	3.06452	5.63952	Adipose_R2_peak_	Distal Interger	13	12996124	13005330	9207	2	18811	uc007plx.1	74027	ENSMUSG000	Prl2c2	prolactin family 2, subfamily c, member 2
chr13	12931508	12931845	338	339	12931609	39	11.04255	3.36786	7.7687	Adipose_R2_peak_	Distal Interger	13	12996124	13005330	9207	2	18811	uc007plx.1	73485	ENSMUSG000	Prl2c2	prolactin family 2, subfamily c, member 2
chr13	12932106	12932337	232	233	12932248	33	7.58056	2.80837	4.54248	Adipose_R2_peak_	Distal Interger	13	12996124	13005330	9207	2	18811	uc007plx.1	72993	ENSMUSG000	Prl2c2	prolactin family 2, subfamily c, member 2
chr13	12932519	12932632	114	115	12932577	29	6.22653	2.62374	3.31626	Adipose_R2_peak_	Distal Interger	13	12996124	13005330	9207	2	18811	uc007plx.1	72698	ENSMUSG000	Prl2c2	prolactin family 2, subfamily c, member 2
chr13	12932972	12933206	235	236	12933007	27	5.11633	2.39413	2.33116	Adipose_R2_peak_	Distal Interger	13	12996124	13005330	9207	2	18811	uc007plx.1	72124	ENSMUSG000	Prl2c2	prolactin family 2, subfamily c, member 2
chr13	12933791	12934274	484	485	12933931	35	9.23519	3.13313	6.07165	Adipose_R2_peak_	Distal Interger	13	12996124	13005330	9207	2	18811	uc007plx.1	71056	ENSMUSG000	Prl2c2	prolactin family 2, subfamily c, member 2
chr13	12934779	12934892	114	115	12934841	32	7.58805	2.85553	4.54937	Adipose_R2_peak_	Distal Interger	13	12996124	13005330	9207	2	18811	uc007plx.1	70438	ENSMUSG000	Prl2c2	prolactin family 2, subfamily c, member 2
chr13	12935286	12935439	154	155	12935370	32	7.72299	2.89105	4.67904	Adipose_R2_peak_	Distal Interger	13	12996124	13005330	9207	2	18811	uc007plx.1	69891	ENSMUSG000	Prl2c2	prolactin family 2, subfamily c, member 2
chr13	12935900	12936049	150	151	12935993	33	7.61192	2.81635	4.57213	Adipose_R2_peak_	Distal Interger	13	12996124	13005330	9207	2	18811	uc007plx.1	69281	ENSMUSG000	Prl2c2	prolactin family 2, subfamily c, member 2
chr13	12937584	12938011	428	429	12937713	36	8.41816	2.88434	5.30426	Adipose_R2_peak_	Distal Interger	13	12996124	13005330	9207	2	18811	uc007plx.1	67319	ENSMUSG000	Prl2c2	prolactin family 2, subfamily c, member 2
chr13	12938251	12938469	219	220	12938293	29	5.32404	2.37973	2.49102	Adipose_R2_peak_	Distal Interger	13	12996124	13005330	9207	2	18811	uc007plx.1	66861	ENSMUSG000	Prl2c2	prolactin family 2, subfamily c, member 2
chr13	12938568	12938994	427	428	12938643	32	7.3164	2.78459	4.28853	Adipose_R2_peak_	Distal Interger	13	12996124	13005330	9207	2	18811	uc007plx.1	66336	ENSMUSG000	Prl2c2	prolactin family 2, subfamily c, member 2
chr13	12939172	12939277	106	107	12939229	33	8.46029	3.03582	5.34465	Adipose_R2_peak_	Distal Interger	13	12996124	13005330	9207	2	18811	uc007plx.1	66053	ENSMUSG000	Prl2c2	prolactin family 2, subfamily c, member 2
chr13	12939536	12939885	350	351	12939720	40	10.71926	3.23979	7.45526	Adipose_R2_peak_	Distal Interger	13	12996124	13005330	9207	2	18811	uc007plx.1	65445	ENSMUSG000	Prl2c2	prolactin family 2, subfamily c, member 2
chr13	12941004	12941128	125	126	12941060	30	6.71732	2.71429	3.75989	Adipose_R2_peak_	Distal Interger	13	12996124	13005330	9207	2	18811	uc007plx.1	64202	ENSMUSG000	Prl2c2	prolactin family 2, subfamily c, member 2
chr13	12941326	12941472	147	148	12941415	27	5.77405	2.5821	2.88921	Adipose_R2_peak_	Distal Interger	13	12996124	13005330	9207	2	18811	uc007plx.1	63858	ENSMUSG000	Prl2c2	prolactin family 2, subfamily c, member 2
chr13	12963586	12963710	125	126	12963648	29	6.74062	2.76653	3.75989	Adipose_R2_peak_	Distal Interger	13	12996124	13005330	9207	2	18811	uc007plx.1	41620	ENSMUSG000	Prl2c2	prolactin family 2, subfamily c, member 2
chr13	12965739	12965881	143	144	12965782	25	4.86612	2.39766	2.09018	Adipose_R2_peak_	Distal Interger	13	12996124	13005330	9207	2	18811	uc007plx.1	39449	ENSMUSG000	Prl2c2	prolactin family 2, subfamily c, member 2
chr13	12966019	12966170	152	153	12966106	32	8.29282	3.04319	5.18352	Adipose_R2_peak_	Distal Interger	13	12996124	13005330	9207	2	18811	uc007plx.1	39160	ENSMUSG000	Prl2c2	prolactin family 2, subfamily c, member 2
chr13	12967313	12967456	144	145	12967383	28	6.25023	2.67431	3.31626	Adipose_R2_peak_	Distal Interger	13	12996124	13005330	9207	2	18811	uc007plx.1	37874	ENSMUSG000	Prl2c2	prolactin family 2, subfamily c, member 2
chr13	12967804	12968074	271	272	12967945	40	12.73705	3.72399	9.36907	Adipose_R2_peak_	Distal Interger	13	12996124	13005330	9207	2	18811	uc007plx.1	37256	ENSMUSG000	Prl2c2	prolactin family 2, subfamily c, member 2
chr13	12968748	12968849	102	103	12968791	28	6.25023	2.67431	3.31626	Adipose_R2_peak_	Distal Interger	13	12996124	13005330	9207	2	18811	uc007plx.1	36481	ENSMUSG000	Prl2c2	prolactin family 2, subfamily c, member 2
chr13	13016958	13017122	165	166	13017037	27	12.93148	5.03266	9.63827	Adipose_R1_peak_	Distal Interger	13	12996124	13005330	9207	2	18811	uc007plx.1	-11628	ENSMUSG000	Prl2c2	prolactin family 2, subfamily c, member 2
chr13	13652717	13652991	275	276	13652866	46	30.36364	8.44768	26.64488	Adipose_R1_peak_	Intron (uc007p	13	13709288	13747529	38242	1	17101	uc007pmk.1	-56297	ENSMUSG000	Lyst	lysosomal trafficking regulator
chr13	13652817	13652918	102	103	13652861	28	6.25023	2.67431	3.31626	Adipose_R2_peak_	Intron (uc007p	13	13709288	13747529	38242	1	17101	uc007pmk.1	-56370	ENSMUSG000	Lyst	lysosomal trafficking regulator
chr13	13718676	13718866	191	192	13718804	17	5.80341	3.23528	2.92378	Adipose_R1_peak_	Intron (uc007p	13	13709288	13747529	38242	1	17101	uc007pmk.1	9388	ENSMUSG000	Lyst	lysosomal trafficking regulator
chr13	13753018	13753120	103	104	13753061	32	7.30463	2.78154	4.27709	Adipose_R2_peak_	Intron (uc007p	13	13784059	13827895	43837	1	14706	uc056ysx.1	-30939	ENSMUSG000	Gng4	guanine nucleotide binding protein (G protein), gamma 4

chr13	13753552	13753784	233	234	13753623	44	7.22078	2.37309	4.21917	Adipose_R2_peak_	Intron (uc007	13	13784059	13827895	43837	1	14706	uc056ysx.1	-30275	ENSMUSG000	Gng4	guanine nucleotide binding protein (G protein), gamma 4
chr13	13754007	13754193	187	188	13754115	35	9.95877	3.31984	6.73562	Adipose_R2_peak_	Intron (uc007	13	13784059	13827895	43837	1	14706	uc056ysx.1	-29866	ENSMUSG000	Gng4	guanine nucleotide binding protein (G protein), gamma 4
chr13	13754994	13755462	469	470	13755082	53	8.70315	2.42659	5.57836	Adipose_R2_peak_	Intron (uc007	13	13784059	13827895	43837	1	14706	uc056ysx.1	-28597	ENSMUSG000	Gng4	guanine nucleotide binding protein (G protein), gamma 4
chr13	13860671	13861006	336	337	13860904	918	979.5455	29.49347	974.2686	Adipose_R1_peak_	Distal Interger	13	13784307	13827895	43589	1	14706	uc007pml.2	76364	ENSMUSG000	Gng4	guanine nucleotide binding protein (G protein), gamma 4
chr13	13860680	13861001	322	323	13860895	673	617.1372	20.57503	611.9876	Adipose_R2_peak_	Distal Interger	13	13784307	13827895	43589	1	14706	uc007pml.2	76373	ENSMUSG000	Gng4	guanine nucleotide binding protein (G protein), gamma 4
chr13	13883607	13883936	330	331	13883787	125	130.2678	22.64698	125.815	Adipose_R1_peak_	Distal Interger	13	13954674	13977289	22616	1	97884	uc007pmm.1	-70738	ENSMUSG000	B3galnt2	UDP-GalNAc:betaGlcNAc beta 1,3-galactosaminyltransferase, polypeptide 2
chr13	13883706	13883855	150	151	13883762	37	11.12817	3.50427	7.83613	Adipose_R2_peak_	Distal Interger	13	13954674	13977289	22616	1	97884	uc007pmm.1	-70819	ENSMUSG000	B3galnt2	UDP-GalNAc:betaGlcNAc beta 1,3-galactosaminyltransferase, polypeptide 2
chr13	13941955	13942232	278	279	13942082	49	33.45876	8.98689	29.68925	Adipose_R1_peak_	Distal Interger	13	13954674	13977289	22616	1	97884	uc007pmm.1	-12442	ENSMUSG000	B3galnt2	UDP-GalNAc:betaGlcNAc beta 1,3-galactosaminyltransferase, polypeptide 2
chr13	13941997	13942175	179	180	13942089	39	12.34187	3.68871	8.98501	Adipose_R2_peak_	Distal Interger	13	13954674	13977289	22616	1	97884	uc007pmm.1	-12499	ENSMUSG000	B3galnt2	UDP-GalNAc:betaGlcNAc beta 1,3-galactosaminyltransferase, polypeptide 2
chr13	14037337	14037571	235	236	14037468	33	17.97508	6.11109	14.52164	Adipose_R1_peak_	Promoter (2-3	13	13997951	14039638	41688	2	70430	uc007pmq.2	2067	ENSMUSG000	Tbce	tubulin-specific chaperone E
chr13	14037342	14037584	243	244	14037459	43	14.8939	4.05758	11.42017	Adipose_R2_peak_	Promoter (2-3	13	13997951	14039638	41688	2	70430	uc007pmq.2	2054	ENSMUSG000	Tbce	tubulin-specific chaperone E
chr13	14065783	14065980	198	199	14065903	26	12.14079	4.85292	8.87838	Adipose_R1_peak_	Promoter (1-2	13	14063784	14165682	101899	1	94246	uc007pmy.2	1999	ENSMUSG000	Arid4b	AT rich interactive domain 4B (RBP1-like)
chr13	14207830	14208147	318	319	14207966	106	103.5054	19.23195	99.15611	Adipose_R1_peak_	Distal Interger	13	14177983	14191256	13274	1	94246	uc007pnc.1	29847	ENSMUSG000	Arid4b	AT rich interactive domain 4B (RBP1-like)
chr13	14207841	14208112	272	273	14207952	88	52.0507	8.20738	47.87361	Adipose_R2_peak_	Distal Interger	13	14177983	14191256	13274	1	94246	uc007pnc.1	29858	ENSMUSG000	Arid4b	AT rich interactive domain 4B (RBP1-like)
chr13	14208835	14209009	175	176	14208927	22	9.13959	4.13397	6.01526	Adipose_R1_peak_	Distal Interger	13	14177983	14191256	13274	1	94246	uc007pnc.1	30852	ENSMUSG000	Arid4b	AT rich interactive domain 4B (RBP1-like)
chr13	14208852	14209005	154	155	14208955	33	8.83596	3.1354	5.68652	Adipose_R2_peak_	Distal Interger	13	14177983	14191256	13274	1	94246	uc007pnc.1	30869	ENSMUSG000	Arid4b	AT rich interactive domain 4B (RBP1-like)
chr13	14363484	14363635	152	153	14363559	27	5.77405	2.5821	2.88921	Adipose_R2_peak_	Intron (uc007	13	14245461	14318974	73514	2	94253	uc007pnf.1	-44510	ENSMUSG000	Hecw1	HECT, C2 and WW domain containing E3 ubiquitin protein ligase 1
chr13	14471558	14471739	182	183	14471683	21	8.43286	3.95423	5.34896	Adipose_R1_peak_	Intron (uc007	13	14226438	14523226	296789	2	94253	uc007pnd.2	51487	ENSMUSG000	Hecw1	HECT, C2 and WW domain containing E3 ubiquitin protein ligase 1
chr13	14742145	14742320	176	177	14742213	24	10.60668	4.49345	7.40961	Adipose_R1_peak_	Distal Interger	13	14630258	14638202	7945	1	105351	uc007pnn.2	111887	ENSMUSG000	AW209491	expressed sequence AW209491
chr13	14743885	14744129	245	246	14744020	32	17.10103	5.93135	13.67137	Adipose_R1_peak_	Distal Interger	13	14630258	14638202	7945	1	105351	uc007pnn.2	113627	ENSMUSG000	AW209491	expressed sequence AW209491
chr13	14743891	14744068	178	179	14744002	37	11.12817	3.50427	7.83613	Adipose_R2_peak_	Distal Interger	13	14630258	14638202	7945	1	105351	uc007pnn.2	113633	ENSMUSG000	AW209491	expressed sequence AW209491
chr13	14772790	14773225	436	437	14772949	62	47.71039	11.32349	43.751	Adipose_R1_peak_	Distal Interger	13	14630258	14638202	7945	1	105351	uc007pnn.2	142532	ENSMUSG000	AW209491	expressed sequence AW209491
chr13	14772864	14773141	278	279	14772999	41	13.59774	3.87315	10.1803	Adipose_R2_peak_	Distal Interger	13	14630258	14638202	7945	1	105351	uc007pnn.2	142606	ENSMUSG000	AW209491	expressed sequence AW209491
chr13	14777036	14777195	160	161	14777112	23	9.86443	4.31371	6.70247	Adipose_R1_peak_	Distal Interger	13	14630258	14638202	7945	1	105351	uc007pnn.2	146778	ENSMUSG000	AW209491	expressed sequence AW209491
chr13	14804699	14804865	167	168	14804772	20	7.74501	3.7745	4.70467	Adipose_R1_peak_	Distal Interger	13	14630258	14638202	7945	1	105351	uc007pnn.2	174441	ENSMUSG000	AW209491	expressed sequence AW209491
chr13	15027960	15028140	181	182	15028033	24	10.60668	4.49345	7.40961	Adipose_R1_peak_	Intron (uc007	13	14630258	14638202	7945	1	105351	uc007pnn.2	397702	ENSMUSG000	AW209491	expressed sequence AW209491
chr13	15027963	15028146	184	185	15028026	40	12.96465	3.78093	9.57654	Adipose_R2_peak_	Intron (uc007	13	14630258	14638202	7945	1	105351	uc007pnn.2	397705	ENSMUSG000	AW209491	expressed sequence AW209491
chr13	15029008	15029175	168	169	15029089	26	12.14079	4.85292	8.87838	Adipose_R1_peak_	Intron (uc007	13	14630258	14638202	7945	1	105351	uc007pnn.2	398750	ENSMUSG000	AW209491	expressed sequence AW209491
chr13	15139890	15140023	134	135	15139959	34	9.39139	3.22762	6.20474	Adipose_R2_peak_	Distal Interger	13	15463235	15715114	251880	1	14634	uc007pnq.1	-323212	ENSMUSG000	Gli3	GLI-Kruppel family member GLI3
chr13	15318263	15318552	290	291	15318395	70	57.07198	12.76139	53.01762	Adipose_R1_peak_	Distal Interger	13	15463235	15715114	251880	1	14634	uc007pnq.1	-144683	ENSMUSG000	Gli3	GLI-Kruppel family member GLI3
chr13	15318297	15318508	212	213	15318391	51	20.44827	4.79532	16.78434	Adipose_R2_peak_	Distal Interger	13	15463235	15715114	251880	1	14634	uc007pnq.1	-144727	ENSMUSG000	Gli3	GLI-Kruppel family member GLI3
chr13	15833650	15833875	226	227	15833764	30	15.39182	5.57188	12.01394	Adipose_R1_peak_	Intron (uc007	13	15807243	15827537	20295	1	328190	uc007pnt.1	26407	NA	A530046M15F	RIKEN cDNA A530046M15 gene
chr13	15833694	15833886	193	194	15833798	40	12.96465	3.78093	9.57654	Adipose_R2_peak_	Intron (uc007	13	15807243	15827537	20295	1	328190	uc007pnt.1	26451	NA	A530046M15F	RIKEN cDNA A530046M15 gene
chr13	16887496	16887672	177	178	16887593	18	6.42934	3.41502	3.48999	Adipose_R1_peak_	Intron (uc007	13	16857475	17694765	837291	2	192136	uc007pnx.3	807093	ENSMUSG000	Sugct	succinyl-CoA glutarate-CoA transferase
chr13	16940935	16941113	179	180	16940997	39	23.47001	7.18952	19.88325	Adipose_R1_peak_	Intron (uc007	13	16857475	17694765	837291	2	192136	uc007pnx.3	753652	ENSMUSG000	Sugct	succinyl-CoA glutarate-CoA transferase
chr13	16940936	16941132	197	198	16940981	46	16.90986	4.33423	13.35898	Adipose_R2_peak_	Intron (uc007	13	16857475	17694765	837291	2	192136	uc007pnx.3	753633	ENSMUSG000	Sugct	succinyl-CoA glutarate-CoA transferase
chr13	17229449	17229638	190	191	17229572	31	7.7623	2.95097	4.69388	Adipose_R2_peak_	Intron (uc007	13	16857475	17694765	837291	2	192136	uc007pnx.3	465127	ENSMUSG000	Sugct	succinyl-CoA glutarate-CoA transferase
chr13	17229528	17229685	158	159	17229589	18	6.42934	3.41502	3.48999	Adipose_R1_peak_	Intron (uc007	13	16857475	17694765	837291	2	192136	uc007pnx.3	465080	ENSMUSG000	Sugct	succinyl-CoA glutarate-CoA transferase
chr13	17330462	17330651	190	191	17330547	21	8.43286	3.95423	5.34896	Adipose_R1_peak_	Intron (uc007	13	16857475	17694765	837291	2	192136	uc007pnx.3	364114	ENSMUSG000	Sugct	succinyl-CoA glutarate-CoA transferase
chr13	17444220	17444364	145	146	17444284	21	8.43286	3.95423	5.34896	Adipose_R1_peak_	Intron (uc007	13	16857475	17694765	837291	2	192136	uc007pnx.3	250401	ENSMUSG000	Sugct	succinyl-CoA glutarate-CoA transferase
chr13	17528805	17529022	218	219	17528904	53	37.70483	9.70585	33.87	Adipose_R1_peak_	Intron (uc007	13	16857475	17694765	837291	2	192136	uc007pnx.3	165743	ENSMUSG000	Sugct	succinyl-CoA glutarate-CoA transferase
chr13	17528816	17529006	191	192	17528898	54	22.67101	5.07198	18.94758	Adipose_R2_peak_	Intron (uc007	13	16857475	17694765	837291	2	192136	uc007pnx.3	165759	ENSMUSG000	Sugct	succinyl-CoA glutarate-CoA transferase
chr13	17609392	17609638	247	248	17609512	52	21.18119	4.88754	17.49655	Adipose_R2_peak_	Intron (uc007	13	16857475	17694765	837291	2	192136	uc007pnx.3	85127	ENSMUSG000	Sugct	succinyl-CoA glutarate-CoA transferase
chr13	17609394	17609644	251	252	17609540	58	43.19021	10.60454	39.28351	Adipose_R1_peak_	Intron (uc007	13	16857475	17694765	837291	2	192136	uc007pnx.3	85121	ENSMUSG000	Sugct	succinyl-CoA glutarate-CoA transferase
chr13	17923045	17923345	301	302	17923190	87	68.96129	12.75222	64.80774	Adipose_R1_peak_	Intron (uc007	13	17880575	17944217	63643	2	56044	uc007pof.1	20872	ENSMUSG000	Rala	v-ral simian leukemia viral oncogene A (ras related)
chr13	17923054	17923324	271	272	17923192	72	37.3624	6.73189	33.36734	Adipose_R2_peak_	Intron (uc007	13	17880575	17944217	63643	2	56044	uc007pof.1	20893	ENSMUSG000	Rala	v-ral simian leukemia viral oncogene A (ras related)
chr13	18083495	18083679	185	186	18083607	33	8.83596	3.1354	5.68652	Adipose_R2_peak_	Downstream (	13	18084947	18100202	15256	2	73992	uc029rzb.1	16523	NA	4930448F12Ri	RIKEN cDNA 4930448F12 gene
chr13	18083567	18083801	235	236	18083710	21	7.86515	3.71791	4.82354	Adipose_R1_peak_	Downstream (	13	18084947	18100202	15256	2	73992	uc029rzb.1	16401	NA	4930448F12Ri	RIKEN cDNA 4930448F12 gene
chr13	18101336	18101501	166	167	18101461	18	6.42934	3.41502	3.48999	Adipose_R1_peak_	Promoter (1-2	13	18084947	18100202	15256	2	73992	uc029rzb.1	-1134	NA	4930448F12Ri	RIKEN cDNA 4930448F12 gene
chr13	18113302	18113518	217	218	18113380	27	12.93148	5.03266	9.63827	Adipose_R1_peak_	Distal Interger	13	18084947	18100202	15256	2	73992	uc029rzb.1	-13100	NA	4930448F12Ri	RIKEN cDNA 4930448F12 gene
chr13	18536408	18536679	272	273	18536524	64	50.01186	11.68296	46.0279	Adipose_R1_peak_	Distal Interger	13	18124960	18382039	257080	2	218030	uc007poh.1	-154369	ENSMUSG000	Pou6f2	POU domain, class 6, transcription factor 2
chr13	18536414	18536621	208	209	18536536	50	19.72356	4.7031	16.08069	Adipose_R2_peak_	Distal Interger	13	18124960	18382039	257080	2	218030	uc007poh.1	-154375	ENSMUSG000	Pou6f2	POU domain, class 6, transcription factor 2
chr13	18961029	18961259	231	232	18961138	37	21.59274	6.83004	18.04766	Adipose_R1_peak_	Intron (uc007	13	18948351	19101626	153276	1	218038	uc007pol.2	12678	ENSMUSG000	Amph	amphiphysin
chr13	18998635	18998794	160	161	18998700	30	7.24477	2.85875	4.21917	Adipose_R2_peak_	Intron (uc007	13	18948351	19101626	153276	1	218038	uc007pol.2	50284	ENSMUSG000	Amph	amphiphysin
chr13	18998657	18998863	207	208	18998750	27	12.93148	5.03266	9.63827	Adipose_R1_peak_	Intron (uc007	13	18948351	19101626	153276	1	218038	uc007pol.2	50306	ENSMUSG000	Amph	amphiphysin
chr13	19318531	19318669	139	140	19318657	16	5.20017	3.05554	2.38734	Adipose_R1_peak_	Intron (uc007	13	19357676	19395813	38138	2	76205	uc007ppa.2	77144	ENSMUSG000	Stard3nl	STARD3 N-terminal like
chr13	19444213	19444388	176	177	19444301	37	11.12817	3.50427	7.83613	Adipose_R2_peak_	Distal Interger	13	19357676	19395813	38138	2	76205	uc007ppa.2	-48400	ENSMUSG000	Stard3nl	STARD3 N-terminal like
chr13	20176424	20176716	293	294	20176497	20	7.74501	3.7745	4.70467	Adipose_R1_peak_	Intron (uc007	13	20111512	20342888	231377	1	140580	uc007ppo.1	64912	ENSMUSG000	Elmo1	engulfment and cell motility 1
chr13	20176429	20176761	333	334	20176514	27	5.77405	2.5821	2.88921	Adipose_R2_peak_	Intron (uc007	13	20111512	20342888	231377	1	140580	uc007ppo.1	64917	ENSMUSG000	Elmo1	engulfment and cell motility 1
chr13	20530877	20531111	235	236	20531109	55	23.42763	5.16419	19.68558	Adipose_R2_peak_	Intron (uc007	13	20472990	20592444	119455	1	140580	uc007ppq.2	57887	ENSMUSG000	Elmo1	engulfment and cell motility 1
chr13	20530920	20531293	374	375	20531046	27	12.93148	5.03266	9.63827	Adipose_R1_peak_	Intron (uc007	13	20472990	20592444	119455	1	140580	uc007ppq.2	57930	ENSMUSG000	Elmo1	engulfment and cell motility 1
chr13	20534707	20534946	240	241	20534829	41	25.38991	7.54899	21.76305	Adipose_R1_peak_	Intron (uc007	13	20472990	20592444	119455	1	140580	uc007ppq.2	61717	ENSMUSG000	Elmo1	engulfment and cell motility 1
chr13	20534722	20534902	181	182	20534824	39	12.34187	3.68871	8.98501	Adipose_R2_peak_	Intron (uc007	13	20472990	20592444	119455	1	140580	uc007ppq.2	61732	ENSMUSG000	Elmo1	engulfment and cell motility 1
chr13	20673243	20673554	312	313	20673379	37	21.59274	6.83004	18.04766	Adipose_R1_peak_	Distal Interger	13	20794113	20801827	7715	1	27052	uc007pps.2	-120559	ENSMUSG000	Aoah	acyloxyacyl hydrolase
chr13	20717663	20717978	316	317	20717843	57	42.07807	10.4248	38.18469	Adipose_R1_peak_	Distal Interger	13	20794113	20801827	7715	1	27052	uc007pps.2	-76135	ENSMUSG000	Aoah	acyloxyacyl hydrolase
chr13	20717757	20717968	212	213	20717846	40	12.96465	3.78093	9.57654	Adipose_R2_peak_	Distal Interger	13	20794113	20801827	7715	1	27052	uc007pps.2	-76145	ENSMUSG000	Aoah	acyloxyacyl hydrolase
chr13	20862753	20863059	307	308	20862889	40	24.42476	7.36925	20.81738	Adipose_R1_peak_	Intron (uc007	13	20794113	20801827	7715	1	27052	uc007pps.2	68640	ENSMUSG000	Aoah	acyloxyacyl hydrolase
chr13	20862826	20862938	113	114	20862885	31	7.7623	2.95097	4.69388	Adipose_R2_peak_	Intron (uc007	13	20794113	20801827	7715	1	27052	uc007pps.2	68713	ENSMUSG000	Aoah	acyloxyacyl hydrolase
chr13	21783724	21783899	176	177	21783786	19	7.07687	3.59476	4.08458	Adipose_R1_peak_	Promoter (<=	13	21782915	21783397	483	2	319153	uc007prj.1	-327	ENSMUSG000	Hist1h3i	histone cluster 1, H3i
chr13	21783843	21784063	221	222	21783916	25	4.86612	2.39766	2.09018	Adipose_R2_peak_	Promoter (<=	13	21782915	21783397	483	2	319153	uc007prj.1	-446	ENSMUSG000	Hist1h3i	histone cluster 1, H3i
chr13	21800142	21800319	178	179	21800230	31	6.17516	2.53054	3.27492	Adipose_R2_peak_	Distal Interger	13	21806412	21810199	3788	2	665622	uc007prn.2	9880	ENSMUSG000	Hist1h2br	histone cluster 1 H2br
chr13	21801418	21801616	199	200	21801450	30	5.32986	2.34784	2.49645	Adipose_R2_peak_	Distal Interger	13	21806412	21810199	3788	2	665622	uc007prn.2	8583	ENSMUSG000	Hist1h2br	histone cluster 1 H2br
chr13	21802822	21803226	405	406	21802887	38	8.08195	2.72717	5.00179	Adipose_R2_peak_	Distal Interger	13	21806412	21810199	3788	2	665622	uc007prn.2	6973	ENSMUSG000	Hist1h2br	histone cluster 1 H2br
chr13	21805262	21805771	510	511	21805385	29	4.84354	2.25286	2.09018	Adipose_R2_peak_	Downstream (	13	21806412	21810199	3788	2	665622	uc007prn.2	4428	ENSMUSG000	Hist1h2br	histone cluster 1 H2br
chr13	21805868	21805999	132	133	21805932	19	5.30403	2.86435	2.48928	Adipose_R1_peak_	Downstream (	13	21806412	21810199	3788	2	665622	uc007prn.2	4200	ENSMUSG000	Hist1h2br	histone cluster 1 H2br
chr13	21807799	21808001	203	204	21807832	18	4.90994	2.77663	2.16721	Adipose_R1_peak_	Promoter (2-3	13	21806412	21810199	3788	2	665622	uc007prn.2	2198	ENSMUSG000	Hist1h2br	histone cluster 1 H2br
chr13	21808180	21808384	205	206	21808257	20	6.19783	3.1404	3.31195	Adipose_R1_peak_	Promoter (1-2	13	21806412	21810199	3788	2	665622	uc007prn.2	1815	ENSMUSG000	Hist1h2br	histone cluster 1 H2br
chr13	21808628	21808821	194	195	21808694	44	12.69413	3.48322	9.32732	Adipose_R2_peak_	Promoter (1-2	13	21806412	21810199	3788	2	665622	uc007prn.2	1378	ENSMUSG000	Hist1h2br	histone cluster 1 H2br
chr13	21808991	21809149	159	160	21809055	24	7.29648	3.23821	4.30147	Adipose_R1_peak_	Promoter (1-2	13	21806412	21810199	3788	2	665622	uc007prn.2	1050	ENSMUSG000	Hist1h2br	histone cluster 1 H2br
chr13	21809036	21809162	127	128	21809079	35	5.82402	2.3174	2.93737	Adipose_R2_peak_	Promoter (1-2	13	21806412	21810199	3788	2	665622	uc007prn.2	1037	ENSMUSG000	Hist1h2br	histone cluster 1 H2br
chr13	21810108	21810231	124	125	21810208	36	6.40761	2.4199	3.46759	Adipose_R2_peak_	Promoter (<=	13	21806412	21810199	3788	2	665622	uc007prn.2	0	ENSMUSG000	Hist1h2br	histone cluster 1 H2br
chr13	21811130	21811530	401	402	21811324	42	9.1787	2.81775	6.01749	Adipose_R2_peak_	Promoter (<=	13	21811746	21812150	405	1	1.00E+08	uc007prr.2	-216	ENSMUSG000	Hist1h4m	histone cluster 1, H4m
chr13	21811419	21811591	173	174	21811507	24	8.59711	3.70678	5.51158	Adipose_R1_peak_	Promoter (<=	13	21811746	21812150	405	1	1.00E+08	uc007prr.2	-155	ENSMUSG000	Hist1h4m	histone cluster 1, H4m
chr13	21820733	21820964	232	233	21820739	18	6.42934	3.41502	3.48999	Adipose_R1_peak_	Distal Interger	13	21811746	21812150	405	1	1.00E+08	uc007prr.2	8987	ENSMUSG000	Hist1h4m	histone cluster 1, H4m
chr13	22603420	22603691	272	273	22603558	61	43.89819	10.19534	39.98834	Adipose_R1_peak_	Distal Interger	13	22591980	22592930	951	2	171251	uc007psv.1	-10490	ENSMUSG000	Vmn1r205	vomeronasal 1 receptor 205
chr13	22603429	22603686	258	259	22603549	62	28.93052	5.80972	25.0743	Adipose_R2_peak_	Distal Interger	13	22591980	22592930	951	2	171251	uc007psv.1	-10499	ENSMUSG000	Vmn1r205	vomeronasal 1 receptor 205
chr13	22606417	22606619	203	204	22606503	20	7.74501	3.7745	4.70467	Adipose_R1_peak_	Distal Interger	13	22591980	22592930	951	2	171251	uc007psv.1	-13487	ENSMUSG000	Vmn1r205	vomeronasal 1 receptor 205
chr13	22899029	22899150	122	123	22899096	27	5.77405	2.5821	2.88921	Adipose_R2_peak_	Distal Interger	13	22883088	22884161	1074	2	171275	uc007ptc.1	-14868	ENSMUSG000	Vmn1r212	vomeronasal 1 receptor 212
chr13	22901290	22901445	156	157	22901377	25	4.86612	2.39766	2.09018	Adipose_R2_peak_	Distal Interger	13	22883088	22884161	1074	2	171275	uc007ptc.1	-17129	ENSMUSG000	Vmn1r212	vomeronasal 1 receptor 212
chr13	22902762	22902894	133	134	22902824	16	5.20017	3.05554	2.38734	Adipose_R1_peak_	Distal Interger	13	22883088	22884161	1074	2	171275	uc007ptc.1	-18601	ENSMUSG000	Vmn1r212	vomeronasal 1 receptor 212
chr13	22908174	22908385	212	213	22908304	36	10.5378	3.41206	7.27949	Adipose_R2_peak_	Distal Interger	13	22883088	22884161	1074	2	171275	uc007ptc.1	-24013	ENSMUSG000	Vmn1r212	vomeronasal 1 receptor 212
chr13	22908264	22908412	149	150	22908324	20	7.45129	3.65002	4.45427	Adipose_R1_peak_	Distal Interger	13	22883088	22884161	1074	2	171275	uc007ptc.1	-24103	ENSMUSG000	Vmn1r212	vomeronasal 1 receptor 212
chr13	22914883	22914984	102	103	22914964	27	5.77405	2.5821	2.88921	Adipose_R2_peak_	Distal Interger	13	22883088	22884161	1074	2	171275	uc007ptc.1	-30722	ENSMUSG000	Vmn1r212	vomeronasal 1 receptor 212
chr13	22921679	22921822	144	145	22921712	26	5.31252	2.48988	2.47999	Adipose_R2_peak_	Distal Interger	13	22883088	22884161	1074	2	171275	uc007ptc.1	-37518	ENSMUSG000	Vmn1r212	vomeronasal 1 receptor 212

chr13	22929364	22929498	135	136	22929427	19	7.07687	3.59476	4.08458	Adipose_R1_peak_4	Distal Intergen	13	22883088	22884161	1074	2	171275	uc007ptc.1	-45203	ENSMUSG000	Vmn1r212	vomeronasal 1 receptor 212
chr13	23548748	23549016	269	270	23548939	23	9.86443	4.31371	6.70247	Adipose_R1_peak_4	Promoter (2-3	13	23551286	23551643	358	2	319157	uc007pua.2	2627	ENSMUSG000	Hist1h4f	histone cluster 1, H4f
chr13	23549004	23549213	210	211	23549067	28	5.43479	2.44572	2.59758	Adipose_R2_peak_4	Promoter (2-3	13	23551286	23551643	358	2	319157	uc007pua.2	2430	ENSMUSG000	Hist1h4f	histone cluster 1, H4f
chr13	23733914	23734085	172	173	23734053	19	7.07687	3.59476	4.08458	Adipose_R1_peak_4	Distal Intergen	13	23738807	23739531	725	1	50708	uc007puq.3	-4722	ENSMUSG000	Hist1h1c	histone cluster 1, H1c
chr13	23739985	23740131	147	148	23740029	28	6.25023	2.67431	3.31626	Adipose_R2_peak_4	Promoter (1-2	13	23738807	23739531	725	1	50708	uc007puq.3	1178	ENSMUSG000	Hist1h1c	histone cluster 1, H1c
chr13	23740484	23740624	141	142	23740535	31	7.59917	2.90653	4.55975	Adipose_R2_peak_4	Promoter (1-2	13	23738807	23739531	725	1	50708	uc007puq.3	1677	ENSMUSG000	Hist1h1c	histone cluster 1, H1c
chr13	24104744	24104932	189	190	24104816	42	14.2409	3.96536	10.79479	Adipose_R2_peak_4	Intron (uc007p	13	24012481	24108594	96114	2	68732	uc007pvm.2	3662	ENSMUSG000	Carmil1	capping protein regulator and myosin 1 linker 1
chr13	24302239	24302547	309	310	24302358	41	25.38991	7.54899	21.76305	Adipose_R1_peak_4	Distal Intergen	13	24012481	24280790	268310	2	68732	uc007pvn.2	-21449	ENSMUSG000	Carmil1	capping protein regulator and myosin 1 linker 1
chr13	24302283	24302420	138	139	24302358	37	11.12817	3.50427	7.83613	Adipose_R2_peak_4	Distal Intergen	13	24012481	24280790	268310	2	68732	uc007pvn.2	-21493	ENSMUSG000	Carmil1	capping protein regulator and myosin 1 linker 1
chr13	24530014	24530347	334	335	24530182	124	105.7092	15.37499	101.3511	Adipose_R1_peak_4	Distal Intergen	13	24582189	24733806	151618	1	193385	uc033glb.1	-51842	ENSMUSG000	Fam65b	family with sequence similarity 65, member B
chr13	24530021	24530308	288	289	24530172	94	57.89143	8.76068	53.65463	Adipose_R2_peak_4	Distal Intergen	13	24582189	24733806	151618	1	193385	uc033glb.1	-51881	ENSMUSG000	Fam65b	family with sequence similarity 65, member B
chr13	24726041	24726346	306	307	24726255	61	46.56983	11.14375	42.62347	Adipose_R1_peak_4	Intron (uc033g	13	24751845	24761863	10019	2	57441	uc007pwg.1	35517	ENSMUSG000	Gmnn	geminin
chr13	24726116	24726345	230	231	24726251	57	24.96362	5.34863	21.18596	Adipose_R2_peak_4	Intron (uc033g	13	24751845	24761863	10019	2	57441	uc007pwg.1	35518	ENSMUSG000	Gmnn	geminin
chr13	24776587	24776768	182	183	24776658	32	8.29282	3.04319	5.18352	Adipose_R2_peak_4	Distal Intergen	13	24751845	24761937	10093	2	57441	uc007pwh.1	-14650	ENSMUSG000	Gmnn	geminin
chr13	25031359	25031695	337	338	25031510	103	90.19249	15.62759	85.90748	Adipose_R1_peak_4	Distal Intergen	13	24992295	25020317	28023	2	380836	uc056ytg.1	-11042	ENSMUSG000	Mrs2	MRS2 magnesium transporter
chr13	25031378	25031644	267	268	25031507	91	54.95	8.48403	50.74234	Adipose_R2_peak_4	Distal Intergen	13	24992295	25020317	28023	2	380836	uc056ytg.1	-11061	ENSMUSG000	Mrs2	MRS2 magnesium transporter
chr13	25050412	25050651	240	241	25050524	84	48.25301	7.83851	44.11785	Adipose_R2_peak_4	Distal Intergen	13	25056004	25121521	65518	1	195208	uc007pwv.1	-5353	ENSMUSG000	Dcdc2a	doublecortin domain containing 2a
chr13	25050425	25050656	232	233	25050553	58	28.45311	6.09049	24.7697	Adipose_R1_peak_4	Distal Intergen	13	25056004	25121521	65518	1	195208	uc007pwv.1	-5348	ENSMUSG000	Dcdc2a	doublecortin domain containing 2a
chr13	25191735	25191870	136	137	25191837	19	7.07687	3.59476	4.08458	Adipose_R1_peak_4	Intron (uc007p	13	25252039	25269996	17958	2	22360	uc007pwx.2	78126	ENSMUSG000	Nrsn1	neurensin 1
chr13	25231550	25231804	255	256	25231682	32	17.10103	5.93135	13.67137	Adipose_R1_peak_4	Distal Intergen	13	25252039	25269996	17958	2	22360	uc007pwx.2	38192	ENSMUSG000	Nrsn1	neurensin 1
chr13	25231600	25231763	164	165	25231666	34	9.39139	3.22762	6.20474	Adipose_R2_peak_4	Distal Intergen	13	25252039	25269996	17958	2	22360	uc007pwx.2	38233	ENSMUSG000	Nrsn1	neurensin 1
chr13	25321400	25321805	406	407	25321624	138	149.306	24.98357	144.7938	Adipose_R1_peak_4	Distal Intergen	13	25252039	25269996	17958	2	22360	uc007pwx.2	-51404	ENSMUSG000	Nrsn1	neurensin 1
chr13	25321440	25321763	324	325	25321606	84	48.25301	7.83851	44.11785	Adipose_R2_peak_4	Distal Intergen	13	25252039	25269996	17958	2	22360	uc007pwx.2	-51444	ENSMUSG000	Nrsn1	neurensin 1
chr13	25334867	25335039	173	174	25334962	21	8.43286	3.95423	5.34896	Adipose_R1_peak_4	Distal Intergen	13	25252039	25269996	17958	2	22360	uc007pwx.2	-64871	ENSMUSG000	Nrsn1	neurensin 1
chr13	25334882	25335028	147	148	25334948	30	7.24477	2.85875	4.21917	Adipose_R2_peak_4	Distal Intergen	13	25252039	25269996	17958	2	22360	uc007pwx.2	-64886	ENSMUSG000	Nrsn1	neurensin 1
chr13	25403043	25403207	165	166	25403105	21	8.43286	3.95423	5.34896	Adipose_R1_peak_4	Distal Intergen	13	25252039	25269996	17958	2	22360	uc007pwx.2	-133047	ENSMUSG000	Nrsn1	neurensin 1
chr13	25506411	25506615	205	206	25506475	21	8.43286	3.95423	5.34896	Adipose_R1_peak_4	Distal Intergen	13	25252039	25269996	17958	2	22360	uc007pwx.2	-236415	ENSMUSG000	Nrsn1	neurensin 1
chr13	25538211	25538481	271	272	25538345	77	65.58373	14.01956	61.45456	Adipose_R1_peak_4	Distal Intergen	13	25252039	25269996	17958	2	22360	uc007pwx.2	-268215	ENSMUSG000	Nrsn1	neurensin 1
chr13	25538228	25538473	246	247	25538352	69	34.76717	6.45524	30.8116	Adipose_R2_peak_4	Distal Intergen	13	25252039	25269996	17958	2	22360	uc007pwx.2	-268232	ENSMUSG000	Nrsn1	neurensin 1
chr13	25770103	25770274	172	173	25770265	20	7.74501	3.7745	4.70467	Adipose_R1_peak_4	Distal Intergen	13	25921035	25966316	45282	2	1.01E+08	uc007pwy.2	196042	ENSMUSG000	Gm11351	predicted gene 11351
chr13	25772733	25772956	224	225	25772908	73	38.23935	6.82411	34.23139	Adipose_R2_peak_4	Distal Intergen	13	25921035	25966316	45282	2	1.01E+08	uc007pwy.2	193360	ENSMUSG000	Gm11351	predicted gene 11351
chr13	25772768	25772956	189	190	25772927	37	21.59274	6.64832	18.04766	Adipose_R1_peak_4	Distal Intergen	13	25921035	25966316	45282	2	1.01E+08	uc007pwy.2	193360	ENSMUSG000	Gm11351	predicted gene 11351
chr13	25824160	25824424	265	266	25824282	49	33.45876	8.98689	29.68925	Adipose_R1_peak_4	Distal Intergen	13	25921035	25966316	45282	2	1.01E+08	uc007pwy.2	141892	ENSMUSG000	Gm11351	predicted gene 11351
chr13	25824184	25824375	192	193	25824284	40	12.96465	3.78093	9.57654	Adipose_R2_peak_4	Distal Intergen	13	25921035	25966316	45282	2	1.01E+08	uc007pwy.2	141941	ENSMUSG000	Gm11351	predicted gene 11351
chr13	26050224	26050427	204	205	26050320	27	12.93148	5.03266	9.63827	Adipose_R1_peak_4	Distal Intergen	13	25921035	25966316	45282	2	1.01E+08	uc007pwy.2	-83908	ENSMUSG000	Gm11351	predicted gene 11351
chr13	26054577	26054933	357	358	26054763	85	75.64196	15.45746	71.43914	Adipose_R1_peak_4	Distal Intergen	13	25921035	25966316	45282	2	1.01E+08	uc007pwy.2	-88261	ENSMUSG000	Gm11351	predicted gene 11351
chr13	26054598	26054906	309	310	26054770	95	58.88104	8.8529	54.63476	Adipose_R2_peak_4	Distal Intergen	13	25921035	25966316	45282	2	1.01E+08	uc007pwy.2	-88282	ENSMUSG000	Gm11351	predicted gene 11351
chr13	26181862	26182037	176	177	26181950	22	9.13959	4.13397	6.01526	Adipose_R1_peak_4	Distal Intergen	13	26283159	26312406	29248	2	73576	uc029rzk.1	130369	NA	1700092E19Ri	RIKEN cDNA 1700092E19 gene
chr13	26239886	26240427	542	543	26240032	74	61.90123	13.48034	57.80368	Adipose_R1_peak_4	Distal Intergen	13	26283159	26312406	29248	2	73576	uc029rzk.1	71979	NA	1700092E19Ri	RIKEN cDNA 1700092E19 gene
chr13	26239928	26240135	208	209	26240026	45	16.22858	4.24202	12.70278	Adipose_R2_peak_4	Distal Intergen	13	26283159	26312406	29248	2	73576	uc029rzk.1	72271	NA	1700092E19Ri	RIKEN cDNA 1700092E19 gene
chr13	26245167	26245329	163	164	26245244	22	9.13959	4.13397	6.01526	Adipose_R1_peak_4	Distal Intergen	13	26283159	26312406	29248	2	73576	uc029rzk.1	67077	NA	1700092E19Ri	RIKEN cDNA 1700092E19 gene
chr13	26372906	26373083	178	179	26372990	23	9.86443	4.31371	6.70247	Adipose_R1_peak_4	Distal Intergen	13	26283159	26312406	29248	2	73576	uc029rzk.1	-60500	NA	1700092E19Ri	RIKEN cDNA 1700092E19 gene
chr13	26372933	26373037	105	106	26372983	33	8.83596	3.1354	5.68652	Adipose_R2_peak_4	Distal Intergen	13	26283159	26312406	29248	2	73576	uc029rzk.1	-60527	NA	1700092E19Ri	RIKEN cDNA 1700092E19 gene
chr13	26442847	26443055	209	210	26442941	25	11.36566	4.67319	8.13482	Adipose_R1_peak_4	Distal Intergen	13	26283159	26312406	29248	2	73576	uc029rzk.1	-130441	NA	1700092E19Ri	RIKEN cDNA 1700092E19 gene
chr13	26547162	26547382	221	222	26547286	24	10.60668	4.49345	7.40961	Adipose_R1_peak_4	Distal Intergen	13	26768172	26770165	1994	2	15192	uc007pxa.2	222783	ENSMUSG000	Hdgfl1	hepatoma derived growth factor-like 1
chr13	26547234	26547328	95	96	26547279	26	5.31252	2.48988	2.47999	Adipose_R2_peak_4	Distal Intergen	13	26768172	26770165	1994	2	15192	uc007pxa.2	222837	ENSMUSG000	Hdgfl1	hepatoma derived growth factor-like 1
chr13	26675991	26676270	280	281	26676110	55	39.87613	10.06532	36.01168	Adipose_R1_peak_4	Distal Intergen	13	26768172	26770165	1994	2	15192	uc007pxa.2	93895	ENSMUSG000	Hdgfl1	hepatoma derived growth factor-like 1
chr13	26688807	26688944	138	139	26688868	18	6.42934	3.41502	3.48999	Adipose_R1_peak_4	Distal Intergen	13	26768172	26770165	1994	2	15192	uc007pxa.2	81221	ENSMUSG000	Hdgfl1	hepatoma derived growth factor-like 1
chr13	26767940	26768132	193	194	26768010	23	9.86443	4.31371	6.70247	Adipose_R1_peak_4	Promoter (2-3	13	26768172	26770165	1994	2	15192	uc007pxa.2	2033	ENSMUSG000	Hdgfl1	hepatoma derived growth factor-like 1
chr13	26821966	26822210	245	246	26822083	50	26.85245	6.60595	23.20434	Adipose_R1_peak_4	Distal Intergen	13	26768172	26770165	1994	2	15192	uc007pxa.2	-51801	ENSMUSG000	Hdgfl1	hepatoma derived growth factor-like 1
chr13	26821972	26822169	198	199	26822103	35	9.95877	3.31984	6.73562	Adipose_R2_peak_4	Distal Intergen	13	26768172	26770165	1994	2	15192	uc007pxa.2	-51807	ENSMUSG000	Hdgfl1	hepatoma derived growth factor-like 1
chr13	27112186	27112477	292	293	27112358	84	64.98041	12.17292	60.85855	Adipose_R1_peak_4	Distal Intergen	13	27121704	27127482	5779	1	215028	uc007pxf.1	-9227	ENSMUSG000	Prl3d2	prolactin family 3, subfamily d, member 1
chr13	27112211	27112473	263	264	27112348	83	47.3161	7.74629	43.1916	Adipose_R2_peak_4	Distal Intergen	13	27121704	27127482	5779	1	215028	uc007pxf.1	-9231	ENSMUSG000	Prl3d2	prolactin family 3, subfamily d, member 1
chr13	27133448	27133631	184	185	27133533	25	11.36566	4.67319	8.13482	Adipose_R1_peak_4	Distal Intergen	13	27121704	27127482	5779	1	215028	uc007pxf.1	11744	ENSMUSG000	Prl3d2	prolactin family 3, subfamily d, member 1
chr13	27168508	27168781	274	275	27168645	59	44.30963	10.78427	40.38949	Adipose_R1_peak_4	Distal Intergen	13	27156799	27162520	5722	1	215029	uc007pxg.1	11709	ENSMUSG000	Prl3d3	prolactin family 3, subfamily d, member 3
chr13	27168511	27168785	275	276	27168638	75	40.01057	7.00855	35.97731	Adipose_R2_peak_4	Distal Intergen	13	27156799	27162520	5722	1	215029	uc007pxg.1	11712	ENSMUSG000	Prl3d3	prolactin family 3, subfamily d, member 3
chr13	27193958	27194081	124	125	27194003	28	6.25023	2.67431	3.31626	Adipose_R2_peak_4	Promoter (2-3	13	27196660	27203750	7091	1	27372	uc007pxh.3	-2579	ENSMUSG000	Prl3c1	prolactin family 3, subfamily c, member 1
chr13	27209485	27209680	196	197	27209674	21	8.43286	3.95423	5.34896	Adipose_R1_peak_4	Distal Intergen	13	27198903	27203750	4848	1	27372	uc011yxt.2	10582	ENSMUSG000	Prl3c1	prolactin family 3, subfamily c, member 1
chr13	27368245	27368407	163	164	27368378	17	5.80341	3.23528	2.92378	Adipose_R1_peak_4	Distal Intergen	13	27383345	27390846	7502	2	66392	uc007pxn.1	22439	ENSMUSG000	Prl2b1	prolactin family 2, subfamily b, member 1
chr13	27369411	27369658	248	249	27369542	35	19.76029	6.47056	16.26028	Adipose_R1_peak_4	Distal Intergen	13	27383345	27390846	7502	2	66392	uc007pxn.1	21188	ENSMUSG000	Prl2b1	prolactin family 2, subfamily b, member 1
chr13	27369421	27369604	184	185	27369512	33	8.83596	3.1354	5.68652	Adipose_R2_peak_4	Distal Intergen	13	27383345	27390846	7502	2	66392	uc007pxn.1	21242	ENSMUSG000	Prl2b1	prolactin family 2, subfamily b, member 1
chr13	27370008	27370273	266	267	27370145	59	44.30963	10.78427	40.38949	Adipose_R1_peak_4	Distal Intergen	13	27383345	27390846	7502	2	66392	uc007pxn.1	20573	ENSMUSG000	Prl2b1	prolactin family 2, subfamily b, member 1
chr13	27370050	27370210	161	162	27370126	40	12.96465	3.78093	9.57654	Adipose_R2_peak_4	Distal Intergen	13	27383345	27390846	7502	2	66392	uc007pxn.1	20636	ENSMUSG000	Prl2b1	prolactin family 2, subfamily b, member 1
chr13	27372126	27372241	116	117	27372210	26	5.31252	2.48988	2.47999	Adipose_R2_peak_4	Distal Intergen	13	27383345	27390846	7502	2	66392	uc007pxn.1	18605	ENSMUSG000	Prl2b1	prolactin family 2, subfamily b, member 1
chr13	27786989	27787332	344	345	27787146	63	48.85777	11.50323	44.88581	Adipose_R1_peak_4	Distal Intergen	13	27773495	27780804	7310	2	67505	uc007pyd.1	-6185	ENSMUSG000	Prl7c1	prolactin family 7, subfamily c, member 1
chr13	27787003	27787258	256	257	27787141	61	28.12311	5.7175	24.28203	Adipose_R2_peak_4	Distal Intergen	13	27773495	27780804	7310	2	67505	uc007pyd.1	-6199	ENSMUSG000	Prl7c1	prolactin family 7, subfamily c, member 1
chr13	27934607	27934778	172	173	27934688	28	13.73721	5.2124	10.41463	Adipose_R1_peak_4	Distal Intergen	13	28016223	28023546	7324	1	19110	uc007pyg.1	-81445	ENSMUSG000	Prl4a1	prolactin family 4, subfamily a, member 1
chr13	27934618	27934759	142	143	27934697	31	7.7623	2.95097	4.69388	Adipose_R2_peak_4	Distal Intergen	13	28016223	28023546	7324	1	19110	uc007pyg.1	-81464	ENSMUSG000	Prl4a1	prolactin family 4, subfamily a, member 1
chr13	27983758	27984025	268	269	27983878	38	22.52591	7.00978	18.96	Adipose_R1_peak_4	Distal Intergen	13	28016223	28023546	7324	1	19110	uc007pyg.1	-32198	ENSMUSG000	Prl4a1	prolactin family 4, subfamily a, member 1
chr13	27983840	27983989	150	151	27983885	33	8.83596	3.1354	5.68652	Adipose_R2_peak_4	Distal Intergen	13	28016223	28023546	7324	1	19110	uc007pyg.1	-32234	ENSMUSG000	Prl4a1	prolactin family 4, subfamily a, member 1
chr13	27987733	27987998	266	267	27987859	67	53.51345	12.22218	49.49424	Adipose_R1_peak_4	Distal Intergen	13	28016223	28023546	7324	1	19110	uc007pyg.1	-28225	ENSMUSG000	Prl4a1	prolactin family 4, subfamily a, member 1
chr13	27987774	27987990	217	218	27987867	49	19.00719	4.61089	15.38598	Adipose_R2_peak_4	Distal Intergen	13	28016223	28023546	7324	1	19110	uc007pyg.1	-28233	ENSMUSG000	Prl4a1	prolactin family 4, subfamily a, member 1
chr13	28051040	28051277	238	239	28051175	28	13.73721	5.2124	10.41463	Adipose_R1_peak_4	Distal Intergen	13	28016223	28023546	7324	1	19110	uc007pyg.1	34817	ENSMUSG000	Prl4a1	prolactin family 4, subfamily a, member 1
chr13	28052248	28052485	238	239	28052365	30	15.39182	5.57188	12.01394	Adipose_R1_peak_4	Distal Intergen	13	28016223	28023546	7324	1	19110	uc007pyg.1	36025	ENSMUSG000	Prl4a1	prolactin family 4, subfamily a, member 1
chr13	28052316	28052436	121	122	28052361	33	8.83596	3.1354	5.68652	Adipose_R2_peak_4	Distal Intergen	13	28016223	28023546	7324	1	19110	uc007pyg.1	36093	ENSMUSG000	Prl4a1	prolactin family 4, subfamily a, member 1
chr13	28053933	28054107	175	176	28054006	25	11.36566	4.67319	8.13482	Adipose_R1_peak_4	Distal Intergen	13	28016223	28023546	7324	1	19110	uc007pyg.1	37710	ENSMUSG000	Prl4a1	prolactin family 4, subfamily a, member 1
chr13	28072972	28073158	187	188	28073061	29	14.55748	5.39214	11.20652	Adipose_R1_peak_4	Distal Intergen	13	28016223	28023546	7324	1	19110	uc007pyg.1	56749	ENSMUSG000	Prl4a1	prolactin family 4, subfamily a, member 1
chr13	28218481	28218767	287	288	28218625	72	53.14472	10.96937	49.13332	Adipose_R1_peak_4	Distal Intergen	13	28142484	28151595	9112	1	28078	uc007pyh.1	75997	ENSMUSG000	Prl5a1	prolactin family 5, subfamily a, member 1
chr13	28218549	28218749	201	202	28218640	46	16.90986	4.33423	13.35898	Adipose_R2_peak_4	Distal Intergen	13	28142484	28151595	9112	1	28078	uc007pyh.1	76065	ENSMUSG000	Prl5a1	prolactin family 5, subfamily a, member 1
chr13	28360851	28360978	128	129	28360968	30	7.24477	2.85875	4.21917	Adipose_R2_peak_4	Distal Intergen	13	28142484	28151595	9112	1	28078	uc007pyh.1	218367	ENSMUSG000	Prl5a1	prolactin family 5, subfamily a, member 1
chr13	29750301	29750446	146	147	29750368	22	9.13959	4.13397	6.01526	Adipose_R1_peak_4	Intron (uc057b	13	29191746	29855673	663928	2	68916	uc057bcz.1	105227	ENSMUSG000	Cdkal1	CDK5 regulatory subunit associated protein 1-like 1
chr13	29857935	29858095	161	162	29857991	16	5.20017	3.05554	2.38734	Adipose_R1_peak_4	Promoter (2-3	13	29191746	29855673	663928	2	68916	uc057bcz.1	-2262	ENSMUSG000	Cdkal1	CDK5 regulatory subunit associated protein 1-like 1
chr13	29881369	29881630	262	263	29881499	38	22.52591	7.00978	18.96	Adipose_R1_peak_4	Distal Intergen	13	29191746	29855673	663928	2	68916	uc057bcz.1	-25696	ENSMUSG000	Cdkal1	CDK5 regulatory subunit associated protein 1-like 1
chr13	29881380	29881608	229	230	29881503	52	21.18119	4.88754	17.49655	Adipose_R2_peak_4	Distal Intergen	13	29191746	29855673	663928	2	68916	uc057bcz.1	-25707	ENSMUSG000	Cdkal1	CDK5 regulatory subunit associated protein 1-like 1
chr13	30798879	30799083	205	206	30798967	30	15.39182	5.57188	12.01394	Adipose_R1_peak_4	Distal Intergen	13	30749258	30766927	17670	1	16364	uc007pyz.1	49621	ENSMUSG000	Irf4	interferon regulatory factor 4
chr13	30798896	30799070	175	176	30798971	35	9.95877	3.31984	6.73562	Adipose_R2_peak_4	Distal Intergen	13	30749258	30766927	17670	1	16364	uc007pyz.1	49638	ENSMUSG000	Irf4	interferon regulatory factor 4
chr13	31111216	31111477	262	263	31111334	62	43.04506	9.70577	39.14745	Adipose_R1_peak_4	Distal Intergen	13	31213410	31241086	27677	2	73924	uc029rzt.1	129609	ENSMUSG000	4930401O12R	RIKEN cDNA 4930401O12 gene
chr13	31111227	31111422	196	197	31111345	39	12.34187	3.68871	8.98501	Adipose_R2_peak_4	Distal Intergen	13	31213410	31241086	27677	2	73924	uc029rzt.1	129664	ENSMUSG000	4930401O12R	RIKEN cDNA 4930401O12 gene
chr13	32353460	32353725	266	267	32353582	40	24.42476	7.36925	20.81738	Adipose_R1_peak_4	Distal Intergen	13	31819586	32338544	518959	2	218138	uc007pzq.1	-14916	ENSMUSG000	Gmds	GDP-mannose 4, 6-dehydratase
chr13	32353521	32353645	125	126	32353574	31	7.7623	2.95097	4.69388	Adipose_R2_peak_4	Distal Intergen	13	31819586	32338544	518959	2	218138	uc007pzq.1	-14977	ENSMUSG000	Gmds	GDP-mannose 4, 6-dehydratase
chr13	32891167	32891457	291	292	32891302	46	16.90986	4.33423	13.35898	Adipose_R2_peak_4	Intron (uc007p	13	32881397	32898140	16744	2	380839	uc007pzw.2	6683	ENSMUSG000	Serpinb1c	serine (or cysteine) peptidase inhibitor, clade B, member 1c
chr13	32891174	32891803	630	631	32891320	33	17.97508	6.11109	14.52164	Adipose_R1_peak_4	Intron (uc007p	13	32881397	32898140	16744	2	380839	uc007pzw.2	6337	ENSMUSG000	Serpinb1c	serine (or cysteine) peptidase inhibitor, clade B, member 1c
chr13	32892872	32893087	216	217	32893007	35	9.30954	3.15207	6.14353	Adipose_R2_peak_4	Intron (uc007p	13	32881397	32898140	16744	2	380839	uc007pzw.2	5053	ENSMUSG000	Serpinb1c	serine (or cysteine) peptidase inhibitor, clade B, member 1c
chr13	32893924	32894253	330	331	32894063	30	14.16214	5.09767	10.83701	Adipose_R1_peak_4	Intron (uc007p	13	32881397	32898140	16744	2	380839	uc007pzw.2	3887	ENSMUSG000	Serpinb1c	serine (or cysteine) peptidase inhibitor, clade B, member 1c

chr13	32893965	32894229	265	266	32894064	49	19.00719	4.61089	15.38598	Adipose_R2_peak_4	Intron (uc007p	13	32881397	32898140	16744	2	380839	uc007pzw.2	3911	ENSMUSG000	Serpinb1c	serine (or cysteine) peptidase inhibitor, clade B, member 1c
chr13	32894502	32894636	135	136	32894590	21	8.43286	3.95423	5.34896	Adipose_R1_peak_4	Intron (uc007p	13	32881397	32898140	16744	2	380839	uc007pzw.2	3504	ENSMUSG000	Serpinb1c	serine (or cysteine) peptidase inhibitor, clade B, member 1c
chr13	33287392	33287510	119	120	33287412	27	5.77405	2.5821	2.88921	Adipose_R2_peak_4	Distal Interger	13	33324077	33335366	11290	1	20709	uc007qag.1	-36567	ENSMUSG000	Serpinb9f	serine (or cysteine) peptidase inhibitor, clade B, member 9f
chr13	33289306	33289454	149	150	33289369	18	5.55801	3.04238	2.73872	Adipose_R1_peak_4	Distal Interger	13	33324077	33335366	11290	1	20709	uc007qag.1	-34623	ENSMUSG000	Serpinb9f	serine (or cysteine) peptidase inhibitor, clade B, member 9f
chr13	33290674	33290782	109	110	33290747	32	8.29282	3.04319	5.18352	Adipose_R2_peak_4	Distal Interger	13	33324077	33335366	11290	1	20709	uc007qag.1	-33295	ENSMUSG000	Serpinb9f	serine (or cysteine) peptidase inhibitor, clade B, member 9f
chr13	33300538	33300755	218	219	33300657	36	20.67077	6.6503	17.14779	Adipose_R1_peak_4	Distal Interger	13	33324077	33335366	11290	1	20709	uc007qag.1	-23322	ENSMUSG000	Serpinb9f	serine (or cysteine) peptidase inhibitor, clade B, member 9f
chr13	33318284	33318654	371	372	33318460	110	105.5806	18.75855	101.2229	Adipose_R1_peak_4	Distal Interger	13	33324077	33335366	11290	1	20709	uc007qag.1	-5423	ENSMUSG000	Serpinb9f	serine (or cysteine) peptidase inhibitor, clade B, member 9f
chr13	33318313	33318581	269	270	33318447	73	38.23935	6.82411	34.23139	Adipose_R2_peak_4	Distal Interger	13	33324077	33335366	11290	1	20709	uc007qag.1	-5496	ENSMUSG000	Serpinb9f	serine (or cysteine) peptidase inhibitor, clade B, member 9f
chr13	33332055	33332190	136	137	33332143	31	7.7623	2.95097	4.69388	Adipose_R2_peak_4	Exon (uc007qa	13	33324077	33335366	11290	1	20709	uc007qag.1	7978	ENSMUSG000	Serpinb9f	serine (or cysteine) peptidase inhibitor, clade B, member 9f
chr13	33416136	33416347	212	213	33416157	16	5.20017	3.05554	2.38734	Adipose_R1_peak_4	Distal Interger	13	33484790	33496000	11211	1	93806	uc007qah.2	-68443	ENSMUSG000	Serpinb9g	serine (or cysteine) peptidase inhibitor, clade B, member 9g
chr13	33418752	33419113	362	363	33418966	92	84.7075	16.71563	80.45023	Adipose_R1_peak_4	Distal Interger	13	33484790	33496000	11211	1	93806	uc007qah.2	-65677	ENSMUSG000	Serpinb9g	serine (or cysteine) peptidase inhibitor, clade B, member 9g
chr13	33418795	33419103	309	310	33418942	89	53.01237	8.2996	48.82483	Adipose_R2_peak_4	Distal Interger	13	33484790	33496000	11211	1	93806	uc007qah.2	-65687	ENSMUSG000	Serpinb9g	serine (or cysteine) peptidase inhibitor, clade B, member 9g
chr13	33419249	33419490	242	243	33419374	34	18.86162	6.29083	15.38482	Adipose_R1_peak_4	Distal Interger	13	33484790	33496000	11211	1	93806	uc007qah.2	-65300	ENSMUSG000	Serpinb9g	serine (or cysteine) peptidase inhibitor, clade B, member 9g
chr13	33443993	33444235	243	244	33444107	40	24.42476	7.36925	20.81738	Adipose_R1_peak_4	Distal Interger	13	33484790	33496000	11211	1	93806	uc007qah.2	-40555	ENSMUSG000	Serpinb9g	serine (or cysteine) peptidase inhibitor, clade B, member 9g
chr13	33444035	33444227	193	194	33444114	40	12.96465	3.78093	9.57654	Adipose_R2_peak_4	Distal Interger	13	33484790	33496000	11211	1	93806	uc007qah.2	-40563	ENSMUSG000	Serpinb9g	serine (or cysteine) peptidase inhibitor, clade B, member 9g
chr13	33681465	33681679	215	216	33681583	27	12.93148	5.03266	9.63827	Adipose_R1_peak_4	Distal Interger	13	33661475	33671585	10111	1	238568	uc056yth.1	19990	ENSMUSG000	Serpinb6d	serine (or cysteine) peptidase inhibitor, clade B, member 6d
chr13	33722633	33722855	223	224	33722729	26	12.14079	4.85292	8.87838	Adipose_R1_peak_4	Distal Interger	13	33661475	33671585	10111	1	238568	uc056yth.1	61158	ENSMUSG000	Serpinb6d	serine (or cysteine) peptidase inhibitor, clade B, member 6d
chr13	33732458	33732565	108	109	33732513	26	5.31252	2.48988	2.47999	Adipose_R2_peak_4	Distal Interger	13	33661475	33671585	10111	1	238568	uc056yth.1	70983	ENSMUSG000	Serpinb6d	serine (or cysteine) peptidase inhibitor, clade B, member 6d
chr13	33732800	33733043	244	245	33732914	46	30.36364	8.44768	26.64488	Adipose_R1_peak_4	Distal Interger	13	33661475	33671585	10111	1	238568	uc056yth.1	71325	ENSMUSG000	Serpinb6d	serine (or cysteine) peptidase inhibitor, clade B, member 6d
chr13	33791941	33792158	218	219	33792058	31	16.23981	5.75161	12.83549	Adipose_R1_peak_4	Distal Interger	13	33832345	33843408	11064	2	435350	uc007qal.1	51250	ENSMUSG000	Serpinb6e	serine (or cysteine) peptidase inhibitor, clade B, member 6e
chr13	33791968	33792126	159	160	33792048	31	7.7623	2.95097	4.69388	Adipose_R2_peak_4	Distal Interger	13	33832345	33843408	11064	2	435350	uc007qal.1	51282	ENSMUSG000	Serpinb6e	serine (or cysteine) peptidase inhibitor, clade B, member 6e
chr13	34474423	34474681	259	260	34474548	51	35.56531	9.34637	31.76235	Adipose_R1_peak_4	Distal Interger	13	34179158	34345182	166025	2	73102	uc007qbh.1	-129241	ENSMUSG000	Slc22a23	solute carrier family 22, member 23
chr13	34474456	34474653	198	199	34474546	40	12.96465	3.78093	9.57654	Adipose_R2_peak_4	Distal Interger	13	34179158	34345182	166025	2	73102	uc007qbh.1	-129274	ENSMUSG000	Slc22a23	solute carrier family 22, member 23
chr13	34485158	34485321	164	165	34485266	19	7.07687	3.59476	4.08458	Adipose_R1_peak_4	Distal Interger	13	34179158	34345182	166025	2	73102	uc007qbh.1	-139976	ENSMUSG000	Slc22a23	solute carrier family 22, member 23
chr13	34542482	34542615	134	135	34542568	20	7.74501	3.7745	4.70467	Adipose_R1_peak_4	Distal Interger	13	34627841	34652681	24841	2	66895	uc007qbi.1	110066	ENSMUSG000	Pxdc1	PX domain containing 1
chr13	34616584	34616722	139	140	34616652	21	8.43286	3.95423	5.34896	Adipose_R1_peak_4	Distal Interger	13	34627841	34652681	24841	2	66895	uc007qbi.1	35959	ENSMUSG000	Pxdc1	PX domain containing 1
chr13	34637696	34637919	224	225	34637758	17	5.80341	3.23528	2.92378	Adipose_R1_peak_4	Intron (uc007c	13	34627841	34652681	24841	2	66895	uc007qbi.1	14762	ENSMUSG000	Pxdc1	PX domain containing 1
chr13	34637736	34637834	99	100	34637768	25	4.86612	2.39766	2.09018	Adipose_R2_peak_4	Intron (uc007c	13	34627841	34652681	24841	2	66895	uc007qbi.1	14847	ENSMUSG000	Pxdc1	PX domain containing 1
chr13	35056250	35056488	239	240	35056366	36	20.67077	6.6503	17.14779	Adipose_R1_peak_4	Distal Interger	13	34977748	34994144	16397	2	23986	uc007qbx.3	-62106	ENSMUSG000	Eci2	enoyl-Coenzyme A delta isomerase 2
chr13	35056285	35056437	153	154	35056357	37	11.12817	3.50427	7.83613	Adipose_R2_peak_4	Distal Interger	13	34977748	34994144	16397	2	23986	uc007qbx.3	-62141	ENSMUSG000	Eci2	enoyl-Coenzyme A delta isomerase 2
chr13	35157535	35157735	201	202	35157635	50	19.72356	4.7031	16.08069	Adipose_R2_peak_4	Distal Interger	13	35181623	35188014	6392	1	1.01E+08	uc007qcc.1	-23888	ENSMUSG000	1700011B04Ri	RIKEN cDNA 1700011B04 gene
chr13	35157543	35157735	193	194	35157622	24	10.60668	4.49345	7.40961	Adipose_R1_peak_4	Distal Interger	13	35181623	35188014	6392	1	1.01E+08	uc007qcc.1	-23888	ENSMUSG000	1700011B04Ri	RIKEN cDNA 1700011B04 gene
chr13	35563678	35563937	260	261	35563802	36	20.67077	6.6503	17.14779	Adipose_R1_peak_4	Distal Interger	13	35659862	35874064	214203	1	12593	uc007qce.2	-95925	ENSMUSG000	Cdyl	chromodomain protein, Y chromosome-like
chr13	35563699	35563892	194	195	35563800	41	13.59774	3.87315	10.1803	Adipose_R2_peak_4	Distal Interger	13	35659862	35874064	214203	1	12593	uc007qce.2	-95970	ENSMUSG000	Cdyl	chromodomain protein, Y chromosome-like
chr13	35569665	35569965	301	302	35569744	25	11.36566	4.67319	8.13482	Adipose_R1_peak_4	Distal Interger	13	35659862	35874064	214203	1	12593	uc007qce.2	-89897	ENSMUSG000	Cdyl	chromodomain protein, Y chromosome-like
chr13	35636354	35636530	177	178	35636450	26	12.14079	4.85292	8.87838	Adipose_R1_peak_4	Distal Interger	13	35659862	35874064	214203	1	12593	uc007qce.2	-23332	ENSMUSG000	Cdyl	chromodomain protein, Y chromosome-like
chr13	35695334	35695558	225	226	35695428	32	17.10103	5.93135	13.67137	Adipose_R1_peak_4	Intron (uc007c	13	35659862	35874064	214203	1	12593	uc007qce.2	35472	ENSMUSG000	Cdyl	chromodomain protein, Y chromosome-like
chr13	36008048	36008336	289	290	36008191	57	42.07807	10.4248	38.18469	Adipose_R1_peak_4	Intron (uc007c	13	35967906	35970388	2483	1	76487	uc007qci.2	40142	ENSMUSG000	Ppp1r3g	protein phosphatase 1, regulatory (inhibitor) subunit 3G
chr13	36008080	36008310	231	232	36008183	49	19.00719	4.61089	15.38598	Adipose_R2_peak_4	Intron (uc007c	13	35967906	35970388	2483	1	76487	uc007qci.2	40174	ENSMUSG000	Ppp1r3g	protein phosphatase 1, regulatory (inhibitor) subunit 3G
chr13	36542293	36542438	146	147	36542365	36	10.5378	3.41206	7.27949	Adipose_R2_peak_4	Distal Interger	13	36725622	36734477	8856	2	68404	uc007qcm.3	192039	ENSMUSG000	Nrn1	neuritin 1
chr13	36542298	36542471	174	175	36542385	26	12.14079	4.85292	8.87838	Adipose_R1_peak_4	Distal Interger	13	36725622	36734477	8856	2	68404	uc007qcm.3	192006	ENSMUSG000	Nrn1	neuritin 1
chr13	36665077	36665265	189	190	36665172	30	15.39182	5.57188	12.01394	Adipose_R1_peak_4	Distal Interger	13	36725622	36734477	8856	2	68404	uc007qcm.3	69212	ENSMUSG000	Nrn1	neuritin 1
chr13	36665106	36665244	139	140	36665176	31	7.7623	2.95097	4.69388	Adipose_R2_peak_4	Distal Interger	13	36725622	36734477	8856	2	68404	uc007qcm.3	69233	ENSMUSG000	Nrn1	neuritin 1
chr13	36795654	36796074	421	422	36795872	144	137.8402	19.6145	133.364	Adipose_R1_peak_4	Distal Interger	13	36725622	36734477	8856	2	68404	uc007qcm.3	-61177	ENSMUSG000	Nrn1	neuritin 1
chr13	36795684	36796019	336	337	36795868	108	72.13875	10.05173	67.7811	Adipose_R2_peak_4	Distal Interger	13	36725622	36734477	8856	2	68404	uc007qcm.3	-61207	ENSMUSG000	Nrn1	neuritin 1
chr13	36843468	36843648	181	182	36843575	26	12.14079	4.85292	8.87838	Adipose_R1_peak_4	Distal Interger	13	36725622	36734477	8856	2	68404	uc007qcm.3	-108991	ENSMUSG000	Nrn1	neuritin 1
chr13	36847644	36847852	209	210	36847753	28	13.73721	5.2124	10.41463	Adipose_R1_peak_4	Distal Interger	13	36725622	36734477	8856	2	68404	uc007qcm.3	-113167	ENSMUSG000	Nrn1	neuritin 1
chr13	36978383	36978908	526	527	36978636	29	11.06431	4.10367	7.86383	Adipose_R1_peak_4	Intron (uc007c	13	36867178	37049213	182036	2	74145	uc007qcn.2	70305	ENSMUSG000	F13a1	coagulation factor XIII, A1 subunit
chr13	36978557	36978908	352	353	36978650	48	18.29934	4.51867	14.70076	Adipose_R2_peak_4	Intron (uc007c	13	36867178	37049213	182036	2	74145	uc007qcn.2	70305	ENSMUSG000	F13a1	coagulation factor XIII, A1 subunit
chr13	37122742	37122971	230	231	37122831	19	7.07687	3.59476	4.08458	Adipose_R1_peak_4	Distal Interger	13	36867178	37050244	183067	2	74145	uc007qco.2	-72498	ENSMUSG000	F13a1	coagulation factor XIII, A1 subunit
chr13	37263636	37263776	141	142	37263713	19	7.07687	3.59476	4.08458	Adipose_R1_peak_4	Distal Interger	13	37345345	37419036	73692	1	17084	uc007qcr.2	-81569	ENSMUSG000	Ly86	lymphocyte antigen 86
chr13	37263640	37263771	132	133	37263704	31	7.7623	2.95097	4.69388	Adipose_R2_peak_4	Distal Interger	13	37345345	37419036	73692	1	17084	uc007qcr.2	-81574	ENSMUSG000	Ly86	lymphocyte antigen 86
chr13	37450342	37450485	144	145	37450366	16	5.20017	3.05554	2.38734	Adipose_R1_peak_4	Intron (uc007c	13	37345345	37419036	73692	1	17084	uc007qcr.2	104997	ENSMUSG000	Ly86	lymphocyte antigen 86
chr13	37473857	37474023	167	168	37473945	20	7.74501	3.7745	4.70467	Adipose_R1_peak_4	Distal Interger	13	37345345	37419036	73692	1	17084	uc007qcr.2	128512	ENSMUSG000	Ly86	lymphocyte antigen 86
chr13	37736758	37736899	142	143	37736822	21	8.43286	3.95423	5.34896	Adipose_R1_peak_4	Distal Interger	13	37826038	37935534	109497	1	68750	uc007qcw.2	-89139	ENSMUSG000	Rreb1	ras responsive element binding protein 1
chr13	37872216	37872311	96	97	37872274	26	5.31252	2.48988	2.47999	Adipose_R2_peak_4	Intron (uc007c	13	37888434	37952005	63572	1	68750	uc007qda.2	-16123	ENSMUSG000	Rreb1	ras responsive element binding protein 1
chr13	38338251	38338519	269	270	38338343	46	9.79359	2.80284	6.593	Adipose_R2_peak_4	Distal Interger	13	38345716	38500314	154599	1	12161	uc007qdp.2	-7197	ENSMUSG000	Bmp6	bone morphogenetic protein 6
chr13	38338315	38338522	208	209	38338406	30	7.90999	3.0455	4.86785	Adipose_R1_peak_4	Distal Interger	13	38345716	38500314	154599	1	12161	uc007qdp.2	-7194	ENSMUSG000	Bmp6	bone morphogenetic protein 6
chr13	38360885	38361017	133	134	38360927	27	5.77405	2.5821	2.88921	Adipose_R2_peak_4	Intron (uc007c	13	38345716	38500314	154599	1	12161	uc007qdp.2	15169	ENSMUSG000	Bmp6	bone morphogenetic protein 6
chr13	38370811	38370995	185	186	38370912	23	9.86443	4.31371	6.70247	Adipose_R1_peak_4	Intron (uc007c	13	38345716	38500314	154599	1	12161	uc007qdp.2	25095	ENSMUSG000	Bmp6	bone morphogenetic protein 6
chr13	38785719	38785995	277	278	38785858	31	7.7623	2.95097	4.69388	Adipose_R2_peak_4	Distal Interger	13	38645691	38659028	13338	2	66143	uc007qdw.1	-126691	ENSMUSG000	Eef1e1	eukaryotic translation elongation factor 1 epsilon 1
chr13	38785802	38786111	310	311	38785887	22	9.13959	4.13397	6.01526	Adipose_R1_peak_4	Distal Interger	13	38645691	38659028	13338	2	66143	uc007qdw.1	-126774	ENSMUSG000	Eef1e1	eukaryotic translation elongation factor 1 epsilon 1
chr13	38889179	38889380	202	203	38889278	35	9.95877	3.31984	6.73562	Adipose_R2_peak_4	Distal Interger	13	38932140	38960537	28398	2	108652	uc007qdy.2	71157	ENSMUSG000	Slc35b3	solute carrier family 35, member B3
chr13	38889190	38889435	246	247	38889316	36	20.67077	6.6503	17.14779	Adipose_R1_peak_4	Distal Interger	13	38932140	38960537	28398	2	108652	uc007qdy.2	71102	ENSMUSG000	Slc35b3	solute carrier family 35, member B3
chr13	39051938	39052138	201	202	39052034	31	16.23981	5.75161	12.83549	Adipose_R1_peak_4	Distal Interger	13	38932140	38960875	28736	2	108652	uc011yyn.1	-91063	ENSMUSG000	Slc35b3	solute carrier family 35, member B3
chr13	39051944	39052090	147	148	39052003	32	8.29282	3.04319	5.18352	Adipose_R2_peak_4	Distal Interger	13	38932140	38960875	28736	2	108652	uc011yyn.1	-91069	ENSMUSG000	Slc35b3	solute carrier family 35, member B3
chr13	39098302	39098413	112	113	39098348	26	5.31252	2.48988	2.47999	Adipose_R2_peak_4	Distal Interger	13	38932140	38960875	28736	2	108652	uc011yyn.1	-137427	ENSMUSG000	Slc35b3	solute carrier family 35, member B3
chr13	39396499	39396661	163	164	39396566	18	6.42934	3.41502	3.48999	Adipose_R1_peak_4	Distal Interger	13	38932140	38960875	28736	2	108652	uc011yyn.1	-435624	ENSMUSG000	Slc35b3	solute carrier family 35, member B3
chr13	39596960	39597092	133	134	39597040	19	7.07687	3.59476	4.08458	Adipose_R1_peak_4	Distal Interger	13	38932140	38960875	28736	2	108652	uc011yyn.1	-636085	ENSMUSG000	Slc35b3	solute carrier family 35, member B3
chr13	39747620	39747794	175	176	39747711	27	12.93148	5.03266	9.63827	Adipose_R1_peak_4	Distal Interger	13	40001882	40288030	286149	2	218165	uc011yyo.1	540236	ENSMUSG000	Ofcc1	orofacial cleft 1 candidate 1
chr13	39747635	39747781	147	148	39747712	31	7.7623	2.95097	4.69388	Adipose_R2_peak_4	Distal Interger	13	40001882	40288030	286149	2	218165	uc011yyo.1	540249	ENSMUSG000	Ofcc1	orofacial cleft 1 candidate 1
chr13	39749748	39750143	396	397	39749938	107	104.8797	19.41169	100.5245	Adipose_R1_peak_4	Distal Interger	13	40001882	40288030	286149	2	218165	uc011yyo.1	537887	ENSMUSG000	Ofcc1	orofacial cleft 1 candidate 1
chr13	39749778	39750129	352	353	39749942	110	74.23988	10.23617	69.8667	Adipose_R2_peak_4	Distal Interger	13	40001882	40288030	286149	2	218165	uc011yyo.1	537901	ENSMUSG000	Ofcc1	orofacial cleft 1 candidate 1
chr13	39943405	39943599	195	196	39943489	28	13.73721	5.2124	10.41463	Adipose_R1_peak_4	Distal Interger	13	40001882	40288030	286149	2	218165	uc011yyo.1	344431	ENSMUSG000	Ofcc1	orofacial cleft 1 candidate 1
chr13	39943440	39943561	122	123	39943485	28	6.25023	2.67431	3.31626	Adipose_R2_peak_4	Distal Interger	13	40001882	40288030	286149	2	218165	uc011yyo.1	344469	ENSMUSG000	Ofcc1	orofacial cleft 1 candidate 1
chr13	40053565	40053773	209	210	40053659	34	18.86162	6.29083	15.38482	Adipose_R1_peak_4	Intron (uc011y	13	40001882	40288030	286149	2	218165	uc011yyo.1	234257	ENSMUSG000	Ofcc1	orofacial cleft 1 candidate 1
chr13	40053613	40053707	95	96	40053658	29	6.74062	2.76653	3.75989	Adipose_R2_peak_4	Intron (uc011y	13	40001882	40288030	286149	2	218165	uc011yyo.1	234323	ENSMUSG000	Ofcc1	orofacial cleft 1 candidate 1
chr13	40055691	40055992	302	303	40055753	23	9.86443	4.31371	6.70247	Adipose_R1_peak_4	Intron (uc011y	13	40001882	40288030	286149	2	218165	uc011yyo.1	232038	ENSMUSG000	Ofcc1	orofacial cleft 1 candidate 1
chr13	40626854	40627051	198	199	40626933	23	9.86443	4.31371	6.70247	Adipose_R1_peak_4	Distal Interger	13	40715303	40725187	9885	2	21418	uc007qed.3	98136	ENSMUSG000	Tfap2a	transcription factor AP-2, alpha
chr13	40761557	40761708	152	153	40761621	23	9.86443	4.31371	6.70247	Adipose_R1_peak_4	Distal Interger	13	40715303	40734920	19618	2	21418	uc056ytm.1	-26637	ENSMUSG000	Tfap2a	transcription factor AP-2, alpha
chr13	40761558	40761657	100	101	40761603	29	6.74062	2.76653	3.75989	Adipose_R2_peak_4	Distal Interger	13	40715303	40734920	19618	2	21418	uc056ytm.1	-26638	ENSMUSG000	Tfap2a	transcription factor AP-2, alpha
chr13	41059745	41060050	306	307	41059874	122	125.9546	22.10776	121.5177	Adipose_R1_peak_4	Intron (uc007c	13	41025120	41070465	45346	2	17152	uc007qev.2	10415	ENSMUSG000	Mak	male germ cell-associated kinase
chr13	41059749	41060030	282	283	41059876	85	49.19499	7.93073	45.04951	Adipose_R2_peak_4	Intron (uc007c	13	41025120	41070465	45346	2	17152	uc007qev.2	10435	ENSMUSG000	Mak	male germ cell-associated kinase
chr13	44822997	44823119	123	124	44823009	25	4.86612	2.39766	2.09018	Adipose_R2_peak_4	Intron (uc007c	13	44840776	44914376	73601	1	16468	uc007qgv.1	-17657	ENSMUSG000	Jarid2	jumonji, AT rich interactive domain 2
chr13	44869573	44870014	442	443	44869644	155	80.14355	7.26305	75.91381	Adipose_R1_peak_4	Intron (uc007c	13	44840776	44914376	73601	1	16468	uc007qgv.1	28797	ENSMUSG000	Jarid2	jumonji, AT rich interactive domain 2
chr13	44869575	44870017	443	444	44869643	227	116.653	7.37974	112.0844	Adipose_R2_peak_4	Intron (uc007c	13	44840776	44914376	73601	1	16468	uc007qgv.1	28799	ENSMUSG000	Jarid2	jumonji, AT rich interactive domain 2
chr13	47226284	47226433	150	151	47226380	25	4.86612	2.39766	2.09018	Adipose_R2_peak_4	Intron (uc007c	13	47194003	47247991	53989	1	218215	uc007qhz.2	32281	ENSMUSG000	Rnf144b	ring finger protein 144B
chr13	47247589	47247734	146	147	47247611	20	7.74501	3.7745	4.70467	Adipose_R1_peak_4	3' UTR	13	47194003	47247991	53989	1	218215	uc007qhz.2	53586	ENSMUSG000	Rnf144b	ring finger protein 144B
chr13	47494637	47494844	208	209	47494733	29	14.55748	5.39214	11.20652	Adipose_R1_peak_4	Distal Interger	13	47194003	47247991	53989	1	218215	uc007qhz.2	300634	ENSMUSG000	Rnf144b	ring finger protein 144B
chr13	47526492	47526624	133	134	47526556	17	5.80341	3.23528	2.92378	Adipose_R1_peak_4	Distal Interger	13	47194003	47247991	53989	1	218215	uc007qhz.2	332489	ENSMUSG000	Rnf144b	ring finger protein 144B
chr13	48297930	48298188	259	260	48298062	39	23.47001	7.18952	19.88325	Adipose_R1_peak_4	Distal Interger	13	48272418	48273884	1467	2	320315	uc007qie.1	-24046	ENSMUSG000	A330048O09R	RIKEN cDNA A330048O09 gene
chr13	48297949	48298183	235	236	48298068	51	20.44827	4.79532	16.78434	Adipose_R2_peak_4	Distal Interger	13	48272418	48273884	1467	2	320315	uc007qie.1	-24065	ENSMUSG000	A330048O09R	RIKEN cDNA A330048O09 gene
chr13	48986594	48986757	164	165	48986655	23	9.86443	4.31371	6.70247	Adipose_R1_peak_4	Distal Interger	13	48968112	48969905	1794	1	68128	uc007qip.2	18482	NA	Fam120aos	family with sequence similarity 120A, opposite strand
chr13	48986604	48986750	147	148	48986657	33	8.83596	3.1354	5.68652	Adipose_R2_peak_4	Distal Interger	13	48968112	48969905	1794	1	68128	uc007qip.2	18492	NA	Fam120aos	family with sequence similarity 120A, opposite strand

chr13	49647140	49647383	244	245	49647290	51	35.56531	9.34637	31.76235	Adipose_R1_peak_	Intron (uc007c	13	49464059	49652731	188673	2	66336	uc007qjk.1	5348	ENSMUSG000	Cenpp	centromere protein P
chr13	49647169	49647381	213	214	49647305	53	21.92215	4.97976	18.21755	Adipose_R2_peak_	Intron (uc007c	13	49464059	49652731	188673	2	66336	uc007qjk.1	5350	ENSMUSG000	Cenpp	centromere protein P
chr13	49998082	49998196	115	116	49998124	28	6.25023	2.67431	3.31626	Adipose_R2_peak_	Distal Interger	13	50245181	50250308	5128	2	380882	uc007qka.1	252112	ENSMUSG000	Gm906	predicted gene 906
chr13	50011257	50011366	110	111	50011302	27	5.77405	2.5821	2.88921	Adipose_R2_peak_	Distal Interger	13	50245181	50250308	5128	2	380882	uc007qka.1	238942	ENSMUSG000	Gm906	predicted gene 906
chr13	50020059	50020325	267	268	50020205	33	17.97508	6.11109	14.52164	Adipose_R1_peak_	Distal Interger	13	50245181	50250308	5128	2	380882	uc007qka.1	229983	ENSMUSG000	Gm906	predicted gene 906
chr13	50020154	50020284	131	132	50020218	29	6.74062	2.76653	3.75989	Adipose_R2_peak_	Distal Interger	13	50245181	50250308	5128	2	380882	uc007qka.1	230024	ENSMUSG000	Gm906	predicted gene 906
chr13	50032741	50032848	108	109	50032841	26	5.31252	2.48988	2.47999	Adipose_R2_peak_	Distal Interger	13	50245181	50250308	5128	2	380882	uc007qka.1	217460	ENSMUSG000	Gm906	predicted gene 906
chr13	50037675	50037882	208	209	50037758	20	7.74501	3.7745	4.70467	Adipose_R1_peak_	Distal Interger	13	50245181	50250308	5128	2	380882	uc007qka.1	212426	ENSMUSG000	Gm906	predicted gene 906
chr13	50101659	50101848	190	191	50101736	32	8.29282	3.04319	5.18352	Adipose_R2_peak_	Distal Interger	13	50245181	50250308	5128	2	380882	uc007qka.1	148460	ENSMUSG000	Gm906	predicted gene 906
chr13	50134826	50135020	195	196	50134947	31	16.23981	5.75161	12.83549	Adipose_R1_peak_	Distal Interger	13	50245181	50250308	5128	2	380882	uc007qka.1	115288	ENSMUSG000	Gm906	predicted gene 906
chr13	50134877	50135020	144	145	50134943	32	8.29282	3.04319	5.18352	Adipose_R2_peak_	Distal Interger	13	50245181	50250308	5128	2	380882	uc007qka.1	115288	ENSMUSG000	Gm906	predicted gene 906
chr13	50695629	50695854	226	227	50695734	29	14.55748	5.39214	11.20652	Adipose_R1_peak_	Promoter (2-3	13	50698329	50703409	5081	1	667693	uc029sav.1	-2475	ENSMUSG000	Gm8765	predicted gene 8765
chr13	50730695	50731077	383	384	50730828	40	24.42476	7.36925	20.81738	Adipose_R1_peak_	Distal Interger	13	50698329	50703409	5081	1	667693	uc029sav.1	32366	ENSMUSG000	Gm8765	predicted gene 8765
chr13	50730755	50731070	316	317	50730818	30	7.24477	2.85875	4.21917	Adipose_R2_peak_	Distal Interger	13	50698329	50703409	5081	1	667693	uc029sav.1	32426	ENSMUSG000	Gm8765	predicted gene 8765
chr13	50803922	50804216	295	296	50804081	66	52.33981	12.04244	48.33179	Adipose_R1_peak_	Distal Interger	13	50753393	50765248	11856	2	71654	uc029sax.1	-38674	NA	4930518P08Ri	RIKEN cDNA 4930518P08 gene
chr13	50803943	50804178	236	237	50804064	52	21.18119	4.88754	17.49655	Adipose_R2_peak_	Distal Interger	13	50753393	50765248	11856	2	71654	uc029sax.1	-38695	NA	4930518P08Ri	RIKEN cDNA 4930518P08 gene
chr13	50841411	50841555	145	146	50841474	22	9.13959	4.13397	6.01526	Adipose_R1_peak_	Distal Interger	13	50753393	50765248	11856	2	71654	uc029sax.1	-76163	NA	4930518P08Ri	RIKEN cDNA 4930518P08 gene
chr13	50843493	50843663	171	172	50843599	20	7.74501	3.7745	4.70467	Adipose_R1_peak_	Distal Interger	13	50753393	50765248	11856	2	71654	uc029sax.1	-78245	NA	4930518P08Ri	RIKEN cDNA 4930518P08 gene
chr13	50873681	50873890	210	211	50873770	25	11.36566	4.67319	8.13482	Adipose_R1_peak_	Distal Interger	13	50753393	50765248	11856	2	71654	uc029sax.1	-108433	NA	4930518P08Ri	RIKEN cDNA 4930518P08 gene
chr13	50897955	50898228	274	275	50898080	41	25.38991	7.54899	21.76305	Adipose_R1_peak_	Distal Interger	13	50753393	50765248	11856	2	71654	uc029sax.1	-132707	NA	4930518P08Ri	RIKEN cDNA 4930518P08 gene
chr13	50897973	50898172	200	201	50898087	44	15.55653	4.1498	12.05625	Adipose_R2_peak_	Distal Interger	13	50753393	50765248	11856	2	71654	uc029sax.1	-132725	NA	4930518P08Ri	RIKEN cDNA 4930518P08 gene
chr13	50920256	50920642	387	388	50920430	102	93.46613	16.93742	89.16473	Adipose_R1_peak_	Distal Interger	13	50753393	50765248	11856	2	71654	uc029sax.1	-155008	NA	4930518P08Ri	RIKEN cDNA 4930518P08 gene
chr13	50920280	50920550	271	272	50920420	84	48.25301	7.83851	44.11785	Adipose_R2_peak_	Distal Interger	13	50753393	50765248	11856	2	71654	uc029sax.1	-155032	NA	4930518P08Ri	RIKEN cDNA 4930518P08 gene
chr13	51034444	51034580	137	138	51034508	19	7.07687	3.59476	4.08458	Adipose_R1_peak_	Distal Interger	13	51097738	51100727	2990	2	77428	uc007qlx.2	66147	NA	9430083A17R	RIKEN cDNA 9430083A17 gene
chr13	51055882	51056038	157	158	51055986	18	6.42934	3.41502	3.48999	Adipose_R1_peak_	Distal Interger	13	51097738	51100727	2990	2	77428	uc007qlx.2	44689	NA	9430083A17R	RIKEN cDNA 9430083A17 gene
chr13	51056824	51057101	278	279	51056959	33	17.97508	6.11109	14.52164	Adipose_R1_peak_	Distal Interger	13	51097738	51100727	2990	2	77428	uc007qlx.2	43626	NA	9430083A17R	RIKEN cDNA 9430083A17 gene
chr13	51056893	51057026	134	135	51056957	33	8.83596	3.1354	5.68652	Adipose_R2_peak_	Distal Interger	13	51097738	51100727	2990	2	77428	uc007qlx.2	43701	NA	9430083A17R	RIKEN cDNA 9430083A17 gene
chr13	51058095	51058291	197	198	51058165	25	11.36566	4.67319	8.13482	Adipose_R1_peak_	Distal Interger	13	51097738	51100727	2990	2	77428	uc007qlx.2	42436	NA	9430083A17R	RIKEN cDNA 9430083A17 gene
chr13	51061059	51061296	238	239	51061180	23	9.86443	4.31371	6.70247	Adipose_R1_peak_	Distal Interger	13	51097738	51100727	2990	2	77428	uc007qlx.2	39431	NA	9430083A17R	RIKEN cDNA 9430083A17 gene
chr13	52383352	52383594	243	244	52383487	34	18.86162	6.29083	15.38482	Adipose_R1_peak_	Distal Interger	13	52504375	52530836	26462	2	68203	uc007qmv.1	147242	ENSMUSG000	Diras2	DIRAS family, GTP-binding RAS-like 2
chr13	52383375	52383589	215	216	52383483	44	15.55653	4.1498	12.05625	Adipose_R2_peak_	Distal Interger	13	52504375	52530836	26462	2	68203	uc007qmv.1	147247	ENSMUSG000	Diras2	DIRAS family, GTP-binding RAS-like 2
chr13	52387610	52387879	270	271	52387753	39	23.47001	7.18952	19.88325	Adipose_R1_peak_	Distal Interger	13	52504375	52530836	26462	2	68203	uc007qmv.1	142957	ENSMUSG000	Diras2	DIRAS family, GTP-binding RAS-like 2
chr13	52387668	52387843	176	177	52387755	39	12.34187	3.68871	8.98501	Adipose_R2_peak_	Distal Interger	13	52504375	52530836	26462	2	68203	uc007qmv.1	142993	ENSMUSG000	Diras2	DIRAS family, GTP-binding RAS-like 2
chr13	52979560	52979653	94	95	52979632	27	5.77405	2.5821	2.88921	Adipose_R2_peak_	Promoter (1-2	13	52967209	52981039	13831	2	18030	uc007qni.1	1386	ENSMUSG000	Nfil3	nuclear factor, interleukin 3, regulated
chr13	52984425	52984523	99	100	52984469	25	4.86612	2.39766	2.09018	Adipose_R2_peak_	Distal Interger	13	52967209	52981039	13831	2	18030	uc007qni.1	-3386	ENSMUSG000	Nfil3	nuclear factor, interleukin 3, regulated
chr13	53059402	53059637	236	237	53059540	26	12.14079	4.85292	8.87838	Adipose_R1_peak_	Distal Interger	13	52967209	52981039	13831	2	18030	uc007qni.1	-78363	ENSMUSG000	Nfil3	nuclear factor, interleukin 3, regulated
chr13	54421714	54421961	248	249	54421831	38	22.52591	7.00978	18.96	Adipose_R1_peak_	Distal Interger	13	54458837	54468840	10004	2	73666	uc007qod.2	46879	ENSMUSG000	Thoc3	THO complex 3
chr13	54421788	54421886	99	100	54421833	30	7.24477	2.85875	4.21917	Adipose_R2_peak_	Distal Interger	13	54458837	54468840	10004	2	73666	uc007qod.2	46954	ENSMUSG000	Thoc3	THO complex 3
chr13	54688825	54689135	311	312	54688869	30	7.24477	2.85875	4.21917	Adipose_R2_peak_	Promoter (<=	13	54679399	54688200	8802	2	105239	uc007qow.2	-625	ENSMUSG000	Rnf44	ring finger protein 44
chr13	54689456	54689730	275	276	54689502	19	7.07687	3.59476	4.08458	Adipose_R1_peak_	Promoter (1-2	13	54679399	54688200	8802	2	105239	uc007qow.2	-1256	ENSMUSG000	Rnf44	ring finger protein 44
chr13	54689983	54690557	575	576	54690057	35	9.95877	3.31984	6.73562	Adipose_R2_peak_	Promoter (1-2	13	54679399	54688200	8802	2	105239	uc007qow.2	-1783	ENSMUSG000	Rnf44	ring finger protein 44
chr13	54690455	54691330	876	877	54690775	22	8.1538	3.73185	5.10907	Adipose_R1_peak_	Promoter (2-3	13	54679399	54688200	8802	2	105239	uc007qow.2	-2255	ENSMUSG000	Rnf44	ring finger protein 44
chr13	54691137	54691236	100	101	54691179	26	5.31252	2.48988	2.47999	Adipose_R2_peak_	Promoter (2-3	13	54679399	54693960	14562	2	105239	uc007qox.3	2724	ENSMUSG000	Rnf44	ring finger protein 44
chr13	54692073	54692245	173	174	54692176	23	9.50663	4.16671	6.37919	Adipose_R1_peak_	Promoter (1-2	13	54679399	54693960	14562	2	105239	uc007qox.3	1715	ENSMUSG000	Rnf44	ring finger protein 44
chr13	54692585	54693280	696	697	54692650	29	6.74062	2.76653	3.75989	Adipose_R2_peak_	Promoter (<=	13	54679399	54693960	14562	2	105239	uc007qox.3	680	ENSMUSG000	Rnf44	ring finger protein 44
chr13	56614935	56615128	194	195	56615025	26	5.31252	2.48988	2.47999	Adipose_R2_peak_	Intron (uc011z	13	56609603	56639339	29737	1	21810	uc011zae.1	5332	ENSMUSG000	Tgfbi	transforming growth factor, beta induced
chr13	56623847	56623980	134	135	56623904	21	8.43286	3.95423	5.34896	Adipose_R1_peak_	Intron (uc011z	13	56609603	56639339	29737	1	21810	uc011zae.1	14244	ENSMUSG000	Tgfbi	transforming growth factor, beta induced
chr13	56981551	56981710	160	161	56981686	17	5.80341	3.23528	2.92378	Adipose_R1_peak_	Distal Interger	13	56773098	56895789	122692	2	26946	uc007qtb.2	-85762	ENSMUSG000	Trpc7	transient receptor potential cation channel, subfamily C, member 7
chr13	56981561	56981710	150	151	56981683	29	6.74062	2.76653	3.75989	Adipose_R2_peak_	Distal Interger	13	56773098	56895789	122692	2	26946	uc007qtb.2	-85772	ENSMUSG000	Trpc7	transient receptor potential cation channel, subfamily C, member 7
chr13	57228289	57228476	188	189	57228353	21	8.43286	3.95423	5.34896	Adipose_R1_peak_	Distal Interger	13	56773098	56895789	122692	2	26946	uc007qtb.2	-332500	ENSMUSG000	Trpc7	transient receptor potential cation channel, subfamily C, member 7
chr13	58429080	58429262	183	184	58429165	25	11.36566	4.67319	8.13482	Adipose_R1_peak_	Distal Interger	13	58405066	58411149	6084	1	74386	uc007quc.2	24014	ENSMUSG000	Rmi1	RecQ mediated genome instability 1
chr13	58431924	58432189	266	267	58432068	58	25.74274	5.44085	21.94821	Adipose_R2_peak_	Distal Interger	13	58405066	58411149	6084	1	74386	uc007quc.2	26858	ENSMUSG000	Rmi1	RecQ mediated genome instability 1
chr13	58431934	58432142	209	210	58432058	30	14.92284	5.38813	11.56983	Adipose_R1_peak_	Distal Interger	13	58405066	58411149	6084	1	74386	uc007quc.2	26868	ENSMUSG000	Rmi1	RecQ mediated genome instability 1
chr13	60160113	60160460	348	349	60160244	60	41.29473	9.51782	37.41404	Adipose_R1_peak_	Distal Interger	13	60174405	60177535	3131	2	14451	uc007qvi.2	17075	ENSMUSG000	Gas1	growth arrest specific 1
chr13	60160121	60160465	345	346	60160322	85	49.19499	7.93073	45.04951	Adipose_R2_peak_	Distal Interger	13	60174405	60177535	3131	2	14451	uc007qvi.2	17070	ENSMUSG000	Gas1	growth arrest specific 1
chr13	60180836	60180992	157	158	60180886	33	8.83596	3.1354	5.68652	Adipose_R2_peak_	Distal Interger	13	60174405	60177535	3131	2	14451	uc007qvi.2	-3301	ENSMUSG000	Gas1	growth arrest specific 1
chr13	60391253	60391459	207	208	60391348	25	11.36566	4.67319	8.13482	Adipose_R1_peak_	Distal Interger	13	60205471	60216018	10548	1	1.01E+08	uc007qvj.1	185782	NA	Gm5084	predicted gene 5084
chr13	60394364	60394585	222	223	60394463	40	24.42476	7.36925	20.81738	Adipose_R1_peak_	Distal Interger	13	60205471	60216018	10548	1	1.01E+08	uc007qvj.1	188893	NA	Gm5084	predicted gene 5084
chr13	60394384	60394600	217	218	60394469	45	16.22858	4.24202	12.70278	Adipose_R2_peak_	Distal Interger	13	60205471	60216018	10548	1	1.01E+08	uc007qvj.1	188913	NA	Gm5084	predicted gene 5084
chr13	60923846	60924108	263	264	60923964	80	69.31596	14.55877	65.15641	Adipose_R1_peak_	Distal Interger	13	60934155	60936566	2412	2	13024	uc007qvy.2	12458	ENSMUSG000	Ctla2a	cytotoxic T lymphocyte-associated protein 2 alpha
chr13	60923864	60924065	202	203	60923958	51	20.44827	4.79532	16.78434	Adipose_R2_peak_	Distal Interger	13	60934155	60936566	2412	2	13024	uc007qvy.2	12501	ENSMUSG000	Ctla2a	cytotoxic T lymphocyte-associated protein 2 alpha
chr13	61333237	61333486	250	251	61333380	47	31.38649	8.62742	27.65048	Adipose_R1_peak_	Distal Interger	13	61352461	61358170	5710	2	56092	uc007qwi.1	24684	ENSMUSG000	Cts7	cathepsin 7
chr13	61333239	61333453	215	216	61333355	45	16.22858	4.24202	12.70278	Adipose_R2_peak_	Distal Interger	13	61352461	61358170	5710	2	56092	uc007qwi.1	24717	ENSMUSG000	Cts7	cathepsin 7
chr13	61335447	61335726	280	281	61335612	31	16.17222	5.72504	12.79011	Adipose_R1_peak_	Distal Interger	13	61352461	61358170	5710	2	56092	uc007qwi.1	22444	ENSMUSG000	Cts7	cathepsin 7
chr13	61402504	61402803	300	301	61402662	89	78.64972	15.42325	74.42919	Adipose_R1_peak_	Distal Interger	13	61352461	61358170	5710	2	56092	uc007qwi.1	-44334	ENSMUSG000	Cts7	cathepsin 7
chr13	61402527	61402789	263	264	61402662	69	34.76717	6.45524	30.8116	Adipose_R2_peak_	Distal Interger	13	61352461	61358170	5710	2	56092	uc007qwi.1	-44357	ENSMUSG000	Cts7	cathepsin 7
chr13	61409991	61410140	150	151	61410064	22	9.13959	4.13397	6.01526	Adipose_R1_peak_	Distal Interger	13	61352461	61358170	5710	2	56092	uc007qwi.1	-51821	ENSMUSG000	Cts7	cathepsin 7
chr13	61411574	61411747	174	175	61411634	32	8.29282	3.04319	5.18352	Adipose_R2_peak_	Distal Interger	13	61352461	61358170	5710	2	56092	uc007qwi.1	-53404	ENSMUSG000	Cts7	cathepsin 7
chr13	61411853	61412268	416	417	61412070	113	113.2082	20.49012	108.8188	Adipose_R1_peak_	Distal Interger	13	61352461	61358170	5710	2	56092	uc007qwi.1	-53683	ENSMUSG000	Cts7	cathepsin 7
chr13	61411918	61412204	287	288	61412056	93	56.90635	8.66847	52.67918	Adipose_R2_peak_	Distal Interger	13	61352461	61358170	5710	2	56092	uc007qwi.1	-53748	ENSMUSG000	Cts7	cathepsin 7
chr13	61422031	61422397	367	368	61422132	33	17.97508	6.11109	14.52164	Adipose_R1_peak_	Distal Interger	13	61352461	61358170	5710	2	56092	uc007qwi.1	-63861	ENSMUSG000	Cts7	cathepsin 7
chr13	61422055	61422211	157	158	61422100	30	7.24477	2.85875	4.21917	Adipose_R2_peak_	Distal Interger	13	61352461	61358170	5710	2	56092	uc007qwi.1	-63885	ENSMUSG000	Cts7	cathepsin 7
chr13	61426758	61426987	230	231	61426871	33	17.97508	6.11109	14.52164	Adipose_R1_peak_	Distal Interger	13	61352461	61358170	5710	2	56092	uc007qwi.1	-68588	ENSMUSG000	Cts7	cathepsin 7
chr13	61426790	61426913	124	125	61426846	29	6.74062	2.76653	3.75989	Adipose_R2_peak_	Distal Interger	13	61352461	61358170	5710	2	56092	uc007qwi.1	-68620	ENSMUSG000	Cts7	cathepsin 7
chr13	61465201	61465556	356	357	61465421	26	12.14079	4.85292	8.87838	Adipose_R1_peak_	Distal Interger	13	61536444	61541839	5396	2	64139	uc007qwj.1	76283	ENSMUSG000	Ctsm	cathepsin M
chr13	61468703	61468990	288	289	61468847	31	16.23981	5.75161	12.83549	Adipose_R1_peak_	Distal Interger	13	61536444	61541839	5396	2	64139	uc007qwj.1	72849	ENSMUSG000	Ctsm	cathepsin M
chr13	61468788	61468907	120	121	61468833	30	7.24477	2.85875	4.21917	Adipose_R2_peak_	Distal Interger	13	61536444	61541839	5396	2	64139	uc007qwj.1	72932	ENSMUSG000	Ctsm	cathepsin M
chr13	61516783	61517100	318	319	61516945	83	67.25892	13.10651	63.11702	Adipose_R1_peak_	Distal Interger	13	61536444	61541839	5396	2	64139	uc007qwj.1	24739	ENSMUSG000	Ctsm	cathepsin M
chr13	61516791	61517053	263	264	61516954	62	28.93052	5.80972	25.0743	Adipose_R2_peak_	Distal Interger	13	61536444	61541839	5396	2	64139	uc007qwj.1	24786	ENSMUSG000	Ctsm	cathepsin M
chr13	61518164	61518410	247	248	61518297	26	12.14079	4.85292	8.87838	Adipose_R1_peak_	Distal Interger	13	61536444	61541839	5396	2	64139	uc007qwj.1	23429	ENSMUSG000	Ctsm	cathepsin M
chr13	61587704	61587849	146	147	61587802	30	7.24477	2.85875	4.21917	Adipose_R2_peak_	Distal Interger	13	61564630	61570127	5498	2	117066	uc007qwl.2	-17577	ENSMUSG000	Cts3	cathepsin 3
chr13	61587705	61588002	298	299	61587836	28	13.73721	5.2124	10.41463	Adipose_R1_peak_	Distal Interger	13	61564630	61570127	5498	2	117066	uc007qwl.2	-17578	ENSMUSG000	Cts3	cathepsin 3
chr13	61806813	61807016	204	205	61806882	27	5.77405	2.5821	2.88921	Adipose_R2_peak_	Promoter (2-3	13	61804312	62173936	369625	1	630579	uc029sbp.1	2501	ENSMUSG000	Zfp808	zinc finger protein 80
chr13	61832644	61832931	288	289	61832777	37	21.59274	6.83004	18.04766	Adipose_R1_peak_	Intron (uc029s	13	61804312	62173936	369625	1	630579	uc029sbp.1	28332	ENSMUSG000	Zfp808	zinc finger protein 80
chr13	61851283	61851442	160	161	61851364	24	10.60668	4.49345	7.40961	Adipose_R1_peak_	Intron (uc029s	13	61804312	62173936	369625	1	630579	uc029sbp.1	46971	ENSMUSG000	Zfp808	zinc finger protein 80
chr13	62040952	62041057	106	107	62041047	27	5.77405	2.5821	2.88921	Adipose_R2_peak_	Intron (uc029s	13	62016028	62173936	157909	1	630579	uc029sbq.1	24924	ENSMUSG000	Zfp808	zinc finger protein 80
chr13	62247298	62247677	380	381	62247445	72	54.89773	11.53772	50.86523	Adipose_R1_peak_	Distal Interger	13	62129890	62173936	44047	1	630579	uc007qws.1	117408	ENSMUSG000	Zfp808	zinc finger protein 80
chr13	62247301	62247618	318	319	62247469	46	16.90986	4.33423	13.35898	Adipose_R2_peak_	Distal Interger	13	62129890	62173936	44047	1	630579	uc007qws.1	117411	ENSMUSG000	Zfp808	zinc finger protein 80
chr13	62419121	62419451	331	332	62419318	33	17.97508	6.11109	14.52164	Adipose_R1_peak_	Distal Interger	13	62367716	62383174	15459	2	1.00E+08	uc007qwu.2	-35947	ENSMUSG000	Gm3604	predicted gene 3604
chr13	62419257	62419414	158	159	62419327	32	8.29282	3.04319	5.18352	Adipose_R2_peak_	Distal Interger	13	62367716	62383174	15459	2	1.00E+08	uc007qwu.2	-36083	ENSMUSG000	Gm3604	predicted gene 3604
chr13	63021659	63021958	300	301	63021809	104	67.98428	9.68286	63.65822	Adipose_R2_peak_	Intron (uc007c	13	63014934	63302877	287944	1	72061	uc007qxi.2	6725	ENSMUSG000	2010111I01Ri	RIKEN cDNA 2010111I01 gene
chr13	63021663	63021996	334	335	63021818	126	131.7124	22.82671	127.2552	Adipose_R1_peak_	Intron (uc007c	13	63014934	63302877	287944	1	72061	uc007qxi.2	6729	ENSMUSG000	2010111I01Ri	RIKEN cDNA 2010111I01 gene
chr13	63234462	63234623	162	163	63234533	75	63.12309	13.66008	59.01505	Adipose_R1_peak_	Intron (uc007c	13	63240029	63302877	62849	1	72061	uc007qxk.2	-5406	ENSMUSG000	2010111I01Ri	RIKEN cDNA 2010111I01 gene
chr13	63234464	63234604	141	142	63234522	53	21.92215	4.97976	18.21755	Adipose_R2_peak_	Intron (uc007c	13	63240029	63302877	62849	1	72061	uc007qxk.2	-5425	ENSMUSG000	2010111I01Ri	RIKEN cDNA 2010111I01 gene

chr13	64154722	64154874	153	154	64154790	19	7.07687	3.59476	4.08458	Adipose_R1_peak_4	Promoter (1-2	13	64133057	64153199	20143	2	238673	uc007qyj.1	-1523	ENSMUSG000	Zfp367	zinc finger protein 367
chr13	64154777	64154874	98	99	64154800	25	4.86612	2.39766	2.09018	Adipose_R2_peak_4	Promoter (1-2	13	64133057	64153199	20143	2	238673	uc007qyj.1	-1578	ENSMUSG000	Zfp367	zinc finger protein 367
chr13	64156050	64156150	101	102	64156107	31	7.7623	2.95097	4.69388	Adipose_R2_peak_4	Promoter (2-3	13	64133057	64153199	20143	2	238673	uc007qyj.1	-2851	ENSMUSG000	Zfp367	zinc finger protein 367
chr13	64157678	64157773	96	97	64157684	28	5.2518	2.39538	2.46177	Adipose_R2_peak_4	Distal Interger	13	64161866	64186537	24672	1	56541	uc011zaz.1	-4093	ENSMUSG000	Habp4	hyaluronic acid binding protein 4
chr13	64158128	64158270	143	144	64158192	18	6.42934	3.41502	3.48999	Adipose_R1_peak_4	Distal Interger	13	64161866	64186537	24672	1	56541	uc011zaz.1	-3596	ENSMUSG000	Habp4	hyaluronic acid binding protein 4
chr13	64159868	64159964	97	98	64159911	31	7.08589	2.7686	4.09239	Adipose_R2_peak_4	Promoter (1-2	13	64161866	64186537	24672	1	56541	uc011zaz.1	-1902	ENSMUSG000	Habp4	hyaluronic acid binding protein 4
chr13	64160473	64160718	246	247	64160582	21	7.77542	3.6812	4.7348	Adipose_R1_peak_4	Promoter (1-2	13	64161866	64186537	24672	1	56541	uc011zaz.1	-1148	ENSMUSG000	Habp4	hyaluronic acid binding protein 4
chr13	64161081	64161365	285	286	64161280	32	8.29282	3.04319	5.18352	Adipose_R2_peak_4	Promoter (<=1	13	64161866	64186537	24672	1	56541	uc011zaz.1	-501	ENSMUSG000	Habp4	hyaluronic acid binding protein 4
chr13	64607216	64607564	349	350	64607410	119	121.6731	21.56855	117.2516	Adipose_R1_peak_4	Distal Interger	13	64481246	64497792	16547	2	68880	uc011zbb.1	-109424	ENSMUSG000	1190003K10Ri	RIKEN cDNA 1190003K10 gene
chr13	64607228	64607537	310	311	64607406	104	67.98428	9.68286	63.65822	Adipose_R2_peak_4	Distal Interger	13	64481246	64497792	16547	2	68880	uc011zbb.1	-109436	ENSMUSG000	1190003K10Ri	RIKEN cDNA 1190003K10 gene
chr13	64608873	64609066	194	195	64608995	36	10.5378	3.41206	7.27949	Adipose_R2_peak_4	Distal Interger	13	64481246	64497792	16547	2	68880	uc011zbb.1	-111081	ENSMUSG000	1190003K10Ri	RIKEN cDNA 1190003K10 gene
chr13	64690317	64690527	211	212	64690391	38	11.72962	3.59649	8.4047	Adipose_R2_peak_4	Distal Interger	13	64737591	64758461	20871	2	238680	uc007qyy.1	67934	ENSMUSG000	Cntnap3	contactin associated protein-like 3
chr13	64711530	64711706	177	178	64711696	37	11.12817	3.50427	7.83613	Adipose_R2_peak_4	Distal Interger	13	64737591	64758461	20871	2	238680	uc007qyy.1	46755	ENSMUSG000	Cntnap3	contactin associated protein-like 3
chr13	64711548	64711706	159	160	64711698	25	11.36566	4.67319	8.13482	Adipose_R1_peak_4	Distal Interger	13	64737591	64758461	20871	2	238680	uc007qyy.1	46755	ENSMUSG000	Cntnap3	contactin associated protein-like 3
chr13	64782678	64782945	268	269	64782812	57	24.96362	5.34863	21.18596	Adipose_R2_peak_4	Intron (uc007c	13	64737591	64758461	20871	2	238680	uc007qyy.1	-24217	ENSMUSG000	Cntnap3	contactin associated protein-like 3
chr13	64782693	64782994	302	303	64782820	51	35.56531	9.34637	31.76235	Adipose_R1_peak_4	Intron (uc007c	13	64737591	64758461	20871	2	238680	uc007qyy.1	-24232	ENSMUSG000	Cntnap3	contactin associated protein-like 3
chr13	64832360	64832551	192	193	64832480	36	10.5378	3.41206	7.27949	Adipose_R2_peak_4	Intron (uc007c	13	64737591	64903888	166298	2	238680	uc007qyz.1	71337	ENSMUSG000	Cntnap3	contactin associated protein-like 3
chr13	64833229	64833461	233	234	64833304	32	8.29282	3.04319	5.18352	Adipose_R2_peak_4	Intron (uc007c	13	64737591	64903888	166298	2	238680	uc007qyz.1	70427	ENSMUSG000	Cntnap3	contactin associated protein-like 3
chr13	64833694	64834437	744	745	64833806	36	8.52919	2.91094	5.41095	Adipose_R2_peak_4	Intron (uc007c	13	64737591	64903888	166298	2	238680	uc007qyz.1	69451	ENSMUSG000	Cntnap3	contactin associated protein-like 3
chr13	64834237	64834379	143	144	64834309	43	13.61249	3.74911	10.31497	Adipose_R1_peak_4	Intron (uc007c	13	64737591	64903888	166298	2	238680	uc007qyz.1	69509	ENSMUSG000	Cntnap3	contactin associated protein-like 3
chr13	65774725	65774870	146	147	65774794	21	8.43286	3.95423	5.34896	Adipose_R1_peak_4	Distal Interger	13	66113453	66122836	9384	1	628709	uc011zbc.1	-338583	ENSMUSG000	Gm10324	predicted gene 10324
chr13	65835886	65836317	432	433	65835973	25	11.36566	4.67319	8.13482	Adipose_R1_peak_4	Distal Interger	13	66113453	66122836	9384	1	628709	uc011zbc.1	-277136	ENSMUSG000	Gm10324	predicted gene 10324
chr13	65922032	65922377	346	347	65922219	56	40.97333	10.24506	37.09402	Adipose_R1_peak_4	Distal Interger	13	66113453	66122836	9384	1	628709	uc011zbc.1	-191076	ENSMUSG000	Gm10324	predicted gene 10324
chr13	65922074	65922301	228	229	65922226	35	9.95877	3.31984	6.73562	Adipose_R2_peak_4	Distal Interger	13	66113453	66122836	9384	1	628709	uc011zbc.1	-191152	ENSMUSG000	Gm10324	predicted gene 10324
chr13	66002025	66002175	151	152	66002103	18	6.42934	3.41502	3.48999	Adipose_R1_peak_4	Distal Interger	13	66113453	66122836	9384	1	628709	uc011zbc.1	-111278	ENSMUSG000	Gm10324	predicted gene 10324
chr13	66554496	66554678	183	184	66554569	32	8.29282	3.04319	5.18352	Adipose_R2_peak_4	Intron (uc029s	13	66418114	66441118	23005	2	76803	uc007qzq.1	-113378	ENSMUSG000	2410141K09Ri	RIKEN cDNA 2410141K09 gene
chr13	66566550	66566721	172	173	66566619	18	6.42934	3.41502	3.48999	Adipose_R1_peak_4	Intron (uc029s	13	66418114	66441118	23005	2	76803	uc007qzq.1	-125432	ENSMUSG000	2410141K09Ri	RIKEN cDNA 2410141K09 gene
chr13	66641143	66641376	234	235	66641242	30	7.24477	2.85875	4.21917	Adipose_R2_peak_4	Intron (uc029s	13	66369085	66752929	383845	2	76803	uc007qzp.2	111553	ENSMUSG000	2410141K09Ri	RIKEN cDNA 2410141K09 gene
chr13	66641216	66641439	224	225	66641335	23	9.86443	4.31371	6.70247	Adipose_R1_peak_4	Intron (uc029s	13	66369085	66752929	383845	2	76803	uc007qzp.2	111490	ENSMUSG000	2410141K09Ri	RIKEN cDNA 2410141K09 gene
chr13	66641582	66641766	185	186	66641667	32	8.29282	3.04319	5.18352	Adipose_R2_peak_4	Intron (uc029s	13	66369085	66752929	383845	2	76803	uc007qzp.2	111163	ENSMUSG000	2410141K09Ri	RIKEN cDNA 2410141K09 gene
chr13	66665389	66665561	173	174	66665453	35	9.95877	3.31984	6.73562	Adipose_R2_peak_4	Intron (uc029s	13	66369085	66752929	383845	2	76803	uc007qzp.2	87368	ENSMUSG000	2410141K09Ri	RIKEN cDNA 2410141K09 gene
chr13	66731230	66731555	326	327	66731410	40	24.42476	7.36925	20.81738	Adipose_R1_peak_4	Intron (uc007c	13	66369085	66752929	383845	2	76803	uc007qzp.2	21374	ENSMUSG000	2410141K09Ri	RIKEN cDNA 2410141K09 gene
chr13	66748442	66748588	147	148	66748458	28	6.25023	2.67431	3.31626	Adipose_R2_peak_4	Intron (uc007c	13	66369085	66752929	383845	2	76803	uc007qzp.2	4341	ENSMUSG000	2410141K09Ri	RIKEN cDNA 2410141K09 gene
chr13	66748762	66748870	109	110	66748828	28	6.25023	2.67431	3.31626	Adipose_R2_peak_4	Intron (uc007c	13	66369085	66752929	383845	2	76803	uc007qzp.2	4059	ENSMUSG000	2410141K09Ri	RIKEN cDNA 2410141K09 gene
chr13	66751898	66751995	98	99	66751943	26	5.31252	2.48988	2.47999	Adipose_R2_peak_4	Promoter (<=1	13	66369085	66752929	383845	2	76803	uc007qzp.2	934	ENSMUSG000	2410141K09Ri	RIKEN cDNA 2410141K09 gene
chr13	66782108	66782272	165	166	66782172	24	10.60668	4.49345	7.40961	Adipose_R1_peak_4	Distal Interger	13	66369085	66752929	383845	2	76803	uc007qzp.2	-29179	ENSMUSG000	2410141K09Ri	RIKEN cDNA 2410141K09 gene
chr13	66840238	66840499	262	263	66840358	53	37.70483	9.70585	33.87	Adipose_R1_peak_4	Distal Interger	13	66904914	66908514	3601	1	1.00E+08	uc007qzv.1	-64415	ENSMUSG000	Gm10767	predicted gene 10767
chr13	66840268	66840493	226	227	66840372	48	18.29934	4.51867	14.70076	Adipose_R2_peak_4	Distal Interger	13	66904914	66908514	3601	1	1.00E+08	uc007qzv.1	-64421	ENSMUSG000	Gm10767	predicted gene 10767
chr13	67015279	67015660	382	383	67015504	172	201.4766	31.09466	196.8451	Adipose_R1_peak_4	Intron (uc029s	13	66996818	67032405	35588	2	71226	uc029sbx.1	16745	NA	4933433G19R	RIKEN cDNA 4933433G19 gene
chr13	67015324	67015649	326	327	67015495	128	93.81911	11.89609	89.33706	Adipose_R2_peak_4	Intron (uc029s	13	66996818	67032405	35588	2	71226	uc029sbx.1	16756	NA	4933433G19R	RIKEN cDNA 4933433G19 gene
chr13	67274701	67274898	198	199	67274790	25	11.36566	4.67319	8.13482	Adipose_R1_peak_4	Intron (uc007r	13	67254918	67278466	23549	2	238690	uc007ram.1	3568	ENSMUSG000	Zfp458	zinc finger protein 458
chr13	67277463	67277746	284	285	67277596	82	71.83084	14.91825	67.65268	Adipose_R1_peak_4	Promoter (<=1	13	67254918	67278466	23549	2	238690	uc007ram.1	720	ENSMUSG000	Zfp458	zinc finger protein 458
chr13	67277471	67277710	240	241	67277597	53	21.92215	4.97976	18.21755	Adipose_R2_peak_4	Promoter (<=1	13	67254918	67278466	23549	2	238690	uc007ram.1	756	ENSMUSG000	Zfp458	zinc finger protein 458
chr13	68320289	68320461	173	174	68320400	23	9.86443	4.31371	6.70247	Adipose_R1_peak_4	Distal Interger	13	68560780	68582104	21325	2	210009	uc057bdm.1	261643	ENSMUSG000	Mtrr	5-methyltetrahydrofolate-homocysteine methyltransferase reductase
chr13	68322929	68323220	292	293	68323045	128	106.0736	14.68291	101.7144	Adipose_R1_peak_4	Distal Interger	13	68560780	68582104	21325	2	210009	uc057bdm.1	258884	ENSMUSG000	Mtrr	5-methyltetrahydrofolate-homocysteine methyltransferase reductase
chr13	68322935	68323198	264	265	68323048	86	50.14197	8.02294	45.98602	Adipose_R2_peak_4	Distal Interger	13	68560780	68582104	21325	2	210009	uc057bdm.1	258906	ENSMUSG000	Mtrr	5-methyltetrahydrofolate-homocysteine methyltransferase reductase
chr13	68323408	68323685	278	279	68323545	54	38.78658	9.88558	34.93639	Adipose_R1_peak_4	Distal Interger	13	68560780	68582104	21325	2	210009	uc057bdm.1	258419	ENSMUSG000	Mtrr	5-methyltetrahydrofolate-homocysteine methyltransferase reductase
chr13	68323434	68323645	212	213	68323552	39	12.34187	3.68871	8.98501	Adipose_R2_peak_4	Distal Interger	13	68560780	68582104	21325	2	210009	uc057bdm.1	258459	ENSMUSG000	Mtrr	5-methyltetrahydrofolate-homocysteine methyltransferase reductase
chr13	68422675	68422940	266	267	68422822	42	24.57817	7.07096	20.96999	Adipose_R1_peak_4	Distal Interger	13	68560780	68582104	21325	2	210009	uc057bdm.1	159164	ENSMUSG000	Mtrr	5-methyltetrahydrofolate-homocysteine methyltransferase reductase
chr13	68422717	68422877	161	162	68422815	34	9.39139	3.22762	6.20474	Adipose_R2_peak_4	Distal Interger	13	68560780	68582104	21325	2	210009	uc057bdm.1	159227	ENSMUSG000	Mtrr	5-methyltetrahydrofolate-homocysteine methyltransferase reductase
chr13	68442182	68442351	170	171	68442267	33	8.83596	3.1354	5.68652	Adipose_R2_peak_4	Distal Interger	13	68560780	68582104	21325	2	210009	uc057bdm.1	139753	ENSMUSG000	Mtrr	5-methyltetrahydrofolate-homocysteine methyltransferase reductase
chr13	68443239	68443458	220	221	68443371	29	6.74062	2.76653	3.75989	Adipose_R2_peak_4	Distal Interger	13	68560780	68582104	21325	2	210009	uc057bdm.1	138646	ENSMUSG000	Mtrr	5-methyltetrahydrofolate-homocysteine methyltransferase reductase
chr13	68444839	68444936	98	99	68444903	29	6.74062	2.76653	3.75989	Adipose_R2_peak_4	Distal Interger	13	68560780	68582104	21325	2	210009	uc057bdm.1	137168	ENSMUSG000	Mtrr	5-methyltetrahydrofolate-homocysteine methyltransferase reductase
chr13	68446920	68447074	155	156	68446985	20	7.74501	3.7745	4.70467	Adipose_R1_peak_4	Distal Interger	13	68560780	68582104	21325	2	210009	uc057bdm.1	135030	ENSMUSG000	Mtrr	5-methyltetrahydrofolate-homocysteine methyltransferase reductase
chr13	68505843	68506107	265	266	68505956	44	28.34544	8.08821	24.66239	Adipose_R1_peak_4	Distal Interger	13	68560780	68582104	21325	2	210009	uc057bdm.1	75997	ENSMUSG000	Mtrr	5-methyltetrahydrofolate-homocysteine methyltransferase reductase
chr13	68505858	68506026	169	170	68505938	36	10.5378	3.41206	7.27949	Adipose_R2_peak_4	Distal Interger	13	68560780	68582104	21325	2	210009	uc057bdm.1	76078	ENSMUSG000	Mtrr	5-methyltetrahydrofolate-homocysteine methyltransferase reductase
chr13	68517931	68518104	174	175	68518005	22	9.13959	4.13397	6.01526	Adipose_R1_peak_4	Distal Interger	13	68560780	68582104	21325	2	210009	uc057bdm.1	64000	ENSMUSG000	Mtrr	5-methyltetrahydrofolate-homocysteine methyltransferase reductase
chr13	68520198	68520467	270	271	68520330	50	34.50784	9.16663	30.72138	Adipose_R1_peak_4	Distal Interger	13	68560780	68582104	21325	2	210009	uc057bdm.1	61637	ENSMUSG000	Mtrr	5-methyltetrahydrofolate-homocysteine methyltransferase reductase
chr13	68520228	68520443	216	217	68520308	43	14.8939	4.05758	11.42017	Adipose_R2_peak_4	Distal Interger	13	68560780	68582104	21325	2	210009	uc057bdm.1	61661	ENSMUSG000	Mtrr	5-methyltetrahydrofolate-homocysteine methyltransferase reductase
chr13	68611004	68611122	119	120	68611084	27	5.77405	2.5821	2.88921	Adipose_R2_peak_4	Intron (uc007r	13	68597439	68614231	16793	1	69315	uc007rcd.2	13565	ENSMUSG000	1700001L19Ri	RIKEN cDNA 1700001L19 gene
chr13	68726103	68726311	209	210	68726187	24	10.60668	4.49345	7.40961	Adipose_R1_peak_4	Intron (uc007r	13	68597439	68614231	16793	1	69315	uc007rcd.2	128664	ENSMUSG000	1700001L19Ri	RIKEN cDNA 1700001L19 gene
chr13	68779298	68779538	241	242	68779436	49	33.45876	8.98689	29.68925	Adipose_R1_peak_4	Intron (uc007r	13	68597439	68614231	16793	1	69315	uc007rcd.2	181859	ENSMUSG000	1700001L19Ri	RIKEN cDNA 1700001L19 gene
chr13	68779299	68779533	235	236	68779454	64	30.56565	5.99415	26.6807	Adipose_R2_peak_4	Intron (uc007r	13	68597439	68614231	16793	1	69315	uc007rcd.2	181860	ENSMUSG000	1700001L19Ri	RIKEN cDNA 1700001L19 gene
chr13	68833357	68833493	137	138	68833487	17	5.80341	3.23528	2.92378	Adipose_R1_peak_4	Intron (uc007r	13	68620043	68999541	379499	2	210044	uc007rcf.1	166048	ENSMUSG000	Adcy2	adenylate cyclase 2
chr13	68837373	68837582	210	211	68837463	50	19.72356	4.7031	16.08069	Adipose_R2_peak_4	Intron (uc007r	13	68620043	68999541	379499	2	210044	uc007rcf.1	161959	ENSMUSG000	Adcy2	adenylate cyclase 2
chr13	68837394	68837625	232	233	68837474	48	30.98019	8.2808	27.25859	Adipose_R1_peak_4	Intron (uc007r	13	68620043	68999541	379499	2	210044	uc007rcf.1	161916	ENSMUSG000	Adcy2	adenylate cyclase 2
chr13	69040849	69041031	183	184	69040948	35	19.76029	6.47056	16.26028	Adipose_R1_peak_4	Distal Interger	13	68620043	68999541	379499	2	210044	uc007rcf.1	-41308	ENSMUSG000	Adcy2	adenylate cyclase 2
chr13	69079676	69079878	203	204	69079760	36	20.67077	6.6503	17.14779	Adipose_R1_peak_4	Distal Interger	13	68620043	68999541	379499	2	210044	uc007rcf.1	-80135	ENSMUSG000	Adcy2	adenylate cyclase 2
chr13	69079697	69079858	162	163	69079747	37	11.12817	3.50427	7.83613	Adipose_R2_peak_4	Distal Interger	13	68620043	68999541	379499	2	210044	uc007rcf.1	-80156	ENSMUSG000	Adcy2	adenylate cyclase 2
chr13	69155847	69156127	281	282	69156007	40	24.42476	7.36925	20.81738	Adipose_R1_peak_4	Distal Interger	13	68620043	68999541	379499	2	210044	uc007rcf.1	-156306	ENSMUSG000	Adcy2	adenylate cyclase 2
chr13	69155862	69156096	235	236	69156005	50	19.72356	4.7031	16.08069	Adipose_R2_peak_4	Distal Interger	13	68620043	68999541	379499	2	210044	uc007rcf.1	-156321	ENSMUSG000	Adcy2	adenylate cyclase 2
chr13	69882948	69883215	268	269	69883098	23	9.86443	4.31371	6.70247	Adipose_R1_peak_4	Distal Interger	13	69809882	69816094	6213	1	28077	uc007rcq.1	73066	ENSMUSG000	Med10	mediator complex subunit 10
chr13	69883056	69883201	146	147	69883082	25	4.86612	2.39766	2.09018	Adipose_R2_peak_4	Distal Interger	13	69809882	69816094	6213	1	28077	uc007rcq.1	73174	ENSMUSG000	Med10	mediator complex subunit 10
chr13	70032279	70032461	183	184	70032377	24	10.60668	4.49345	7.40961	Adipose_R1_peak_4	Intron (uc056y	13	70004050	70028348	24299	2	74280	uc007rcs.1	-3931	ENSMUSG000	1700084F23Ri	RIKEN cDNA 1700084F23 gene
chr13	70032310	70032470	161	162	70032355	30	7.24477	2.85875	4.21917	Adipose_R2_peak_4	Intron (uc056y	13	70004050	70028348	24299	2	74280	uc007rcs.1	-3962	ENSMUSG000	1700084F23Ri	RIKEN cDNA 1700084F23 gene
chr13	70318007	70318300	294	295	70318131	65	51.17258	11.8627	47.17609	Adipose_R1_peak_4	Distal Interger	13	70232822	70248122	15301	1	75086	uc029scb.1	85185	NA	4930520P13Ri	RIKEN cDNA 4930520P13 gene
chr13	70318008	70318248	241	242	70318136	54	22.67101	5.07198	18.94758	Adipose_R2_peak_4	Distal Interger	13	70232822	70248122	15301	1	75086	uc029scb.1	85186	NA	4930520P13Ri	RIKEN cDNA 4930520P13 gene
chr13	70389570	70389834	265	266	70389695	47	31.38649	8.62742	27.65048	Adipose_R1_peak_4	Distal Interger	13	70232822	70248122	15301	1	75086	uc029scb.1	156748	NA	4930520P13Ri	RIKEN cDNA 4930520P13 gene
chr13	70389652	70389782	131	132	70389724	30	7.24477	2.85875	4.21917	Adipose_R2_peak_4	Distal Interger	13	70232822	70248122	15301	1	75086	uc029scb.1	156830	NA	4930520P13Ri	RIKEN cDNA 4930520P13 gene
chr13	70926298	70926467	170	171	70926362	19	7.07687	3.59476	4.08458	Adipose_R1_peak_4	Distal Interger	13	70882948	70884503	1556	1	77166	uc029scc.1	43350	ENSMUSG000	8030423J24Ril	RIKEN cDNA 8030423J24 gene
chr13	71013927	71014066	140	141	71014027	19	7.07687	3.59476	4.08458	Adipose_R1_peak_4	Distal Interger	13	71107089	71107209	121	1	1.00E+08	uc056yue.1	-93023	ENSMUSG000	Mir466f-4	microRNA 466f-4
chr13	71018565	71018704	140	141	71018617	16	5.20017	3.05554	2.38734	Adipose_R1_peak_4	Distal Interger	13	71107089	71107209	121	1	1.00E+08	uc056yue.1	-88385	ENSMUSG000	Mir466f-4	microRNA 466f-4
chr13	71019509	71019908	400	401	71019558	19	7.07687	3.59476	4.08458	Adipose_R1_peak_4	Distal Interger	13	71107089	71107209	121	1	1.00E+08	uc056yue.1	-87181	ENSMUSG000	Mir466f-4	microRNA 466f-4
chr13	71065962	71066127	166	167	71066036	19	7.07687	3.59476	4.08458	Adipose_R1_peak_4	Distal Interger	13	71107089	71107209	121	1	1.00E+08	uc056yue.1	-40962	ENSMUSG000	Mir466f-4	microRNA 466f-4
chr13	71214043	71214220	178	179	71214212	45	16.22858	4.24202	12.70278	Adipose_R2_peak_4	Distal Interger	13	71107089	71107209	121	1	1.00E+08	uc056yue.1	106954	ENSMUSG000	Mir466f-4	microRNA 466f-4
chr13	71214056	71214219	164	165	71214170	26	12.14079	4.85292	8.87838	Adipose_R1_peak_4	Distal Interger	13	71107089	71107209	121	1	1.00E+08	uc056yue.1	106967	ENSMUSG000	Mir466f-4	microRNA 466f-4
chr13	71317774	71318006	233	234	71317884	48	32.41826	8.80716	28.66523	Adipose_R1_peak_4	Distal Interger	13	71107089	71107209	121	1	1.00E+08	uc056yue.1	210685	ENSMUSG000	Mir466f-4	microRNA 466f-4
chr13	71317798	71317986	189	190	71317905	37	11.12817	3.50427	7.83613	Adipose_R2_peak_4	Distal Interger	13	71107089	71107209	121	1	1.00E+08	uc056yue.1	210709	ENSMUSG000	Mir466f-4	microRNA 466f-4
chr13	71384546	71384783	238	239	71384659	32	17.10103	5.93135	13.67137	Adipose_R1_peak_4	Distal Interger	13	71107089	71107209	121	1	1.00E+08	uc056yue.1	277457	ENSMUSG000	Mir466f-4	microRNA 466f-4
chr13	71384589	71384730	142	143	71384642	27	5.77405	2.5821	2.88921	Adipose_R2_peak_4	Distal Interger	13	71107089	71107209	121	1	1.00E+08	uc056yue.1	277500	ENSMUSG000	Mir466f-4	microRNA 466f-4
chr13	71430789	71431036	248	249	71430917	54	38.78658	9.88558	34.93639	Adipose_R1_peak_4	Distal Interger	13	71107089	71107209	121	1	1.00E+08	uc056yue.1	323700	ENSMUSG000	Mir466f-4	microRNA 466f-4
chr13	71430822	71431019	198	199	71430919	46	16.90986	4.33423	13.35898	Adipose_R2_peak_4	Distal Interger	13	71107089	71107209	121	1	1.00E+08	uc056yue.1	323733	ENSMUSG000	Mir466f-4	microRNA 466f-4
chr13	71431661	71431864	204	205	71431750	34	18.86162	6.29083	15.38482	Adipose_R1_peak_4	Distal Interger	13	71107089	71107209	121	1	1.00E+08	uc056yue.1	324572	ENSMUSG000	Mir466f-4	microRNA 466f-4
chr13	71431683	71431846	164	165	71431760	33	8.83596	3.1354	5.68652	Adipose_R2_peak_4	Distal Interger	13	71107089	71107209	121	1	1.00E+08	uc056yue.1	324594	ENSMUSG000	Mir466f-4	microRNA 466f-4

chr13	71703668	71703910	243	244	71703718	19	7.07687	3.59476	4.08458	Adipose_R1_peak_4	Distal Interger	13	71958232	71963723	5492	2	16371	uc007rdd.1	259813	ENSMUSG000	Irx1	Iroquois related homeobox 1 (Drosophila)
chr13	72011394	72011604	211	212	72011506	41	25.38991	7.54899	21.76305	Adipose_R1_peak_4	Distal Interger	13	71958232	71963723	5492	2	16371	uc007rdd.1	-47671	ENSMUSG000	Irx1	Iroquois related homeobox 1 (Drosophila)
chr13	72011401	72011597	197	198	72011492	36	10.5378	3.41206	7.27949	Adipose_R2_peak_4	Distal Interger	13	71958232	71963723	5492	2	16371	uc007rdd.1	-47678	ENSMUSG000	Irx1	Iroquois related homeobox 1 (Drosophila)
chr13	72386566	72386739	174	175	72386648	27	12.93148	5.03266	9.63827	Adipose_R1_peak_4	Distal Interger	13	72623466	72628564	5099	2	328287	uc011zbn.1	241825	NA	Gm20554	predicted gene, 20554
chr13	72390936	72391206	271	272	72391086	49	33.45876	8.98689	29.68925	Adipose_R1_peak_4	Distal Interger	13	72623466	72628564	5099	2	328287	uc011zbn.1	237358	NA	Gm20554	predicted gene, 20554
chr13	72390993	72391147	155	156	72391090	28	6.25023	2.67431	3.31626	Adipose_R2_peak_4	Distal Interger	13	72623466	72628564	5099	2	328287	uc011zbn.1	237417	NA	Gm20554	predicted gene, 20554
chr13	72554064	72554279	216	217	72554166	32	17.10103	5.93135	13.67137	Adipose_R1_peak_4	Distal Interger	13	72623466	72628564	5099	2	328287	uc011zbn.1	74285	NA	Gm20554	predicted gene, 20554
chr13	72679980	72680165	186	187	72680078	27	12.93148	5.03266	9.63827	Adipose_R1_peak_4	Distal Interger	13	72628797	72634196	5400	1	16372	uc007rde.2	51183	ENSMUSG000	Irx2	Iroquois related homeobox 2 (Drosophila)
chr13	72680006	72680132	127	128	72680067	34	9.39139	3.22762	6.20474	Adipose_R2_peak_4	Distal Interger	13	72628797	72634196	5400	1	16372	uc007rde.2	51209	ENSMUSG000	Irx2	Iroquois related homeobox 2 (Drosophila)
chr13	72683449	72683765	317	318	72683614	93	84.44014	16.33819	80.18662	Adipose_R1_peak_4	Distal Interger	13	72628797	72634196	5400	1	16372	uc007rde.2	54652	ENSMUSG000	Irx2	Iroquois related homeobox 2 (Drosophila)
chr13	72683478	72683763	286	287	72683617	102	65.93154	9.49843	61.62263	Adipose_R2_peak_4	Distal Interger	13	72628797	72634196	5400	1	16372	uc007rde.2	54681	ENSMUSG000	Irx2	Iroquois related homeobox 2 (Drosophila)
chr13	72715731	72715901	171	172	72715818	29	14.55748	5.39214	11.20652	Adipose_R1_peak_4	Distal Interger	13	72628797	72634196	5400	1	16372	uc007rde.2	86934	ENSMUSG000	Irx2	Iroquois related homeobox 2 (Drosophila)
chr13	72989300	72989558	259	260	72989431	43	27.35048	7.90847	23.68561	Adipose_R1_peak_4	Distal Interger	13	72809601	72816763	7163	2	78446	uc029sce.1	-172537	NA	D730050B12R	RIKEN cDNA D730050B12 gene
chr13	72989379	72989539	161	162	72989442	33	8.83596	3.1354	5.68652	Adipose_R2_peak_4	Distal Interger	13	72809601	72816763	7163	2	78446	uc029sce.1	-172616	NA	D730050B12R	RIKEN cDNA D730050B12 gene
chr13	74528500	74530872	2373	2374	74528612	47	21.13116	5.33016	17.60602	Adipose_R1_peak_4	Distal Interger	13	74480057	74493950	13894	2	235956	uc007rfg.1	-34550	ENSMUSG000	Zfp825	zinc finger protein 825
chr13	74528523	74528823	301	302	74528610	46	16.19267	4.16269	12.67956	Adipose_R2_peak_4	Distal Interger	13	74480057	74493950	13894	2	235956	uc007rfg.1	-34573	ENSMUSG000	Zfp825	zinc finger protein 825
chr13	74528982	74529199	218	219	74529056	78	26.53524	4.31412	22.72483	Adipose_R2_peak_4	Distal Interger	13	74480057	74493950	13894	2	235956	uc007rfg.1	-35032	ENSMUSG000	Zfp825	zinc finger protein 825
chr13	74529366	74529766	401	402	74529400	47	5.84679	2.06778	2.95929	Adipose_R2_peak_4	Distal Interger	13	74480057	74493950	13894	2	235956	uc007rfg.1	-35416	ENSMUSG000	Zfp825	zinc finger protein 825
chr13	74529889	74530893	1005	1006	74530099	57	14.23876	3.22173	10.79479	Adipose_R2_peak_4	Distal Interger	13	74480057	74493950	13894	2	235956	uc007rfg.1	-35939	ENSMUSG000	Zfp825	zinc finger protein 825
chr13	75026771	75026953	183	184	75026867	24	10.60668	4.49345	7.40961	Adipose_R1_peak_4	Distal Interger	13	75089987	75106603	16617	1	18548	uc007rfr.1	-63034	ENSMUSG000	Pcsk1	proprotein convertase subtilisin/kexin type 1
chr13	75172445	75172684	240	241	75172569	29	14.55748	5.39214	11.20652	Adipose_R1_peak_4	Distal Interger	13	75089987	75106603	16617	1	18548	uc007rfr.1	82458	ENSMUSG000	Pcsk1	proprotein convertase subtilisin/kexin type 1
chr13	75175672	75175793	122	123	75175701	25	4.86612	2.39766	2.09018	Adipose_R2_peak_4	Distal Interger	13	75089987	75106603	16617	1	18548	uc007rfr.1	85685	ENSMUSG000	Pcsk1	proprotein convertase subtilisin/kexin type 1
chr13	76079113	76079307	195	196	76079229	38	11.72962	3.59649	8.4047	Adipose_R2_peak_4	Promoter (1-2	13	76069485	76077403	7919	2	77041	uc007rgi.1	-1710	ENSMUSG000	Arsk	arylsulfatase K
chr13	76079125	76079375	251	252	76079257	35	19.76029	6.47056	16.26028	Adipose_R1_peak_4	Promoter (1-2	13	76069485	76077403	7919	2	77041	uc007rgi.1	-1722	ENSMUSG000	Arsk	arylsulfatase K
chr13	76171079	76171271	193	194	76171142	27	12.93148	5.03266	9.63827	Adipose_R1_peak_4	Intron (uc007r	13	76131208	76190106	58899	1	218343	uc007rgl.1	39871	ENSMUSG000	Ttc37	tetratricopeptide repeat domain 37
chr13	76206655	76206973	319	320	76206822	91	83.39809	16.53589	79.14825	Adipose_R1_peak_4	Distal Interger	13	76131208	76190106	58899	1	218343	uc007rgl.1	75447	ENSMUSG000	Ttc37	tetratricopeptide repeat domain 37
chr13	76206675	76206959	285	286	76206817	76	40.9047	7.10077	36.85923	Adipose_R2_peak_4	Distal Interger	13	76131208	76190106	58899	1	218343	uc007rgl.1	75467	ENSMUSG000	Ttc37	tetratricopeptide repeat domain 37
chr13	76263052	76263243	192	193	76263227	23	9.86443	4.31371	6.70247	Adipose_R1_peak_4	Intron (uc007r	13	76384961	77031810	646850	1	78771	uc007rgn.1	-121718	ENSMUSG000	Mctp1	multiple C2 domains, transmembrane 1
chr13	76717823	76718034	212	213	76717914	31	16.23981	5.75161	12.83549	Adipose_R1_peak_4	Intron (uc007r	13	76723668	76828840	105173	1	78771	uc007rgq.1	-5634	ENSMUSG000	Mctp1	multiple C2 domains, transmembrane 1
chr13	76717860	76717976	117	118	76717917	27	5.77405	2.5821	2.88921	Adipose_R2_peak_4	Intron (uc007r	13	76723668	76828840	105173	1	78771	uc007rgq.1	-5692	ENSMUSG000	Mctp1	multiple C2 domains, transmembrane 1
chr13	76719420	76719702	283	284	76719562	65	51.17258	11.8627	47.17609	Adipose_R1_peak_4	Intron (uc007r	13	76723668	76828840	105173	1	78771	uc007rgq.1	-3966	ENSMUSG000	Mctp1	multiple C2 domains, transmembrane 1
chr13	76719430	76719665	236	237	76719554	50	19.72356	4.7031	16.08069	Adipose_R2_peak_4	Intron (uc007r	13	76723668	76828840	105173	1	78771	uc007rgq.1	-4003	ENSMUSG000	Mctp1	multiple C2 domains, transmembrane 1
chr13	76722785	76722930	146	147	76722865	20	7.74501	3.7745	4.70467	Adipose_R1_peak_4	Promoter (<=:	13	76723668	76828840	105173	1	78771	uc007rgq.1	-738	ENSMUSG000	Mctp1	multiple C2 domains, transmembrane 1
chr13	76903734	76904008	275	276	76903869	54	38.78658	9.88558	34.93639	Adipose_R1_peak_4	Intron (uc007r	13	76825446	77031810	206365	1	78771	uc007rgr.1	78288	ENSMUSG000	Mctp1	multiple C2 domains, transmembrane 1
chr13	76903748	76904146	399	400	76903873	53	21.92215	4.97976	18.21755	Adipose_R2_peak_4	Intron (uc007r	13	76825446	77031810	206365	1	78771	uc007rgr.1	78302	ENSMUSG000	Mctp1	multiple C2 domains, transmembrane 1
chr13	76926906	76927022	117	118	76926912	25	4.86612	2.39766	2.09018	Adipose_R2_peak_5	Intron (uc007r	13	76825446	77031810	206365	1	78771	uc007rgr.1	101460	ENSMUSG000	Mctp1	multiple C2 domains, transmembrane 1
chr13	77255409	77255590	182	183	77255489	26	12.14079	4.85292	8.87838	Adipose_R1_peak_4	Intron (uc007r	13	77135536	77193905	58370	1	72371	uc007rgw.1	119873	ENSMUSG000	2210408I21Ri	RIKEN cDNA 2210408I21 gene
chr13	77369513	77369713	201	202	77369554	18	6.42934	3.41502	3.48999	Adipose_R1_peak_4	Intron (uc007r	13	77135536	77193905	58370	1	72371	uc007rgw.1	233977	ENSMUSG000	2210408I21Ri	RIKEN cDNA 2210408I21 gene
chr13	77376110	77376307	198	199	77376193	28	13.73721	5.2124	10.41463	Adipose_R1_peak_4	Intron (uc007r	13	77135536	77193905	58370	1	72371	uc007rgw.1	240574	ENSMUSG000	2210408I21Ri	RIKEN cDNA 2210408I21 gene
chr13	77376117	77376313	197	198	77376231	40	12.96465	3.78093	9.57654	Adipose_R2_peak_5	Intron (uc007r	13	77135536	77193905	58370	1	72371	uc007rgw.1	240581	ENSMUSG000	2210408I21Ri	RIKEN cDNA 2210408I21 gene
chr13	77430864	77431113	250	251	77430987	32	17.10103	5.93135	13.67137	Adipose_R1_peak_4	Intron (uc007r	13	77708690	78166240	457551	1	68675	uc007rgy.2	-277577	ENSMUSG000	Fam172a	family with sequence similarity 172, member A
chr13	77430922	77431047	126	127	77430987	32	8.29282	3.04319	5.18352	Adipose_R2_peak_5	Intron (uc007r	13	77708690	78166240	457551	1	68675	uc007rgy.2	-277643	ENSMUSG000	Fam172a	family with sequence similarity 172, member A
chr13	77431849	77432167	319	320	77432021	25	11.36566	4.67319	8.13482	Adipose_R1_peak_4	Intron (uc007r	13	77708690	78166240	457551	1	68675	uc007rgy.2	-276523	ENSMUSG000	Fam172a	family with sequence similarity 172, member A
chr13	77432030	77432227	198	199	77432166	37	11.12817	3.50427	7.83613	Adipose_R2_peak_5	Intron (uc007r	13	77708690	78166240	457551	1	68675	uc007rgy.2	-276463	ENSMUSG000	Fam172a	family with sequence similarity 172, member A
chr13	77438920	77439052	133	134	77438986	78	32.1563	5.22374	28.25	Adipose_R2_peak_5	Intron (uc007r	13	77708690	78166240	457551	1	68675	uc007rgy.2	-269638	ENSMUSG000	Fam172a	family with sequence similarity 172, member A
chr13	77540241	77540487	247	248	77540366	36	20.67077	6.6503	17.14779	Adipose_R1_peak_4	Intron (uc007r	13	77708690	78166240	457551	1	68675	uc007rgy.2	-168203	ENSMUSG000	Fam172a	family with sequence similarity 172, member A
chr13	77540846	77541238	393	394	77541027	124	128.8266	22.46724	124.3789	Adipose_R1_peak_4	Intron (uc007r	13	77708690	78166240	457551	1	68675	uc007rgy.2	-167452	ENSMUSG000	Fam172a	family with sequence similarity 172, member A
chr13	77540853	77541240	388	389	77541027	154	123.9861	14.29375	119.3959	Adipose_R2_peak_5	Intron (uc007r	13	77708690	78166240	457551	1	68675	uc007rgy.2	-167450	ENSMUSG000	Fam172a	family with sequence similarity 172, member A
chr13	77574478	77574742	265	266	77574603	44	28.34544	8.08821	24.66239	Adipose_R1_peak_4	Intron (uc007r	13	77708690	78166240	457551	1	68675	uc007rgy.2	-133948	ENSMUSG000	Fam172a	family with sequence similarity 172, member A
chr13	77946123	77946788	666	667	77946252	32	17.10103	5.93135	13.67137	Adipose_R1_peak_4	Intron (uc007r	13	78024902	78026296	1395	1	75507	uc011zcd.1	-78114	ENSMUSG000	Pou5f2	POU domain class 5, transcription factor 2
chr13	77946473	77946762	290	291	77946645	59	26.5291	5.53306	22.7188	Adipose_R2_peak_5	Intron (uc007r	13	78024902	78026296	1395	1	75507	uc011zcd.1	-78140	ENSMUSG000	Pou5f2	POU domain class 5, transcription factor 2
chr13	77948433	77948634	202	203	77948552	23	9.86443	4.31371	6.70247	Adipose_R1_peak_4	Intron (uc007r	13	78024902	78026296	1395	1	75507	uc011zcd.1	-76268	ENSMUSG000	Pou5f2	POU domain class 5, transcription factor 2
chr13	78054580	78054732	153	154	78054684	23	9.86443	4.31371	6.70247	Adipose_R1_peak_4	Intron (uc007r	13	78024902	78026296	1395	1	75507	uc011zcd.1	29678	ENSMUSG000	Pou5f2	POU domain class 5, transcription factor 2
chr13	78133764	78134150	387	388	78133932	167	161.3618	20.45777	156.8181	Adipose_R1_peak_4	Intron (uc007r	13	78198017	78236564	38548	1	320174	uc029sch.1	-63867	NA	A830082K12R	RIKEN cDNA A830082K12 gene
chr13	78133828	78134063	236	237	78133926	119	80.80696	10.4355	76.39034	Adipose_R2_peak_5	Intron (uc007r	13	78198017	78236564	38548	1	320174	uc029sch.1	-63954	NA	A830082K12R	RIKEN cDNA A830082K12 gene
chr13	78137074	78137253	180	181	78137160	25	11.36566	4.67319	8.13482	Adipose_R1_peak_4	Intron (uc007r	13	78198017	78236564	38548	1	320174	uc029sch.1	-60764	NA	A830082K12R	RIKEN cDNA A830082K12 gene
chr13	78142115	78142334	220	221	78142232	35	19.76029	6.47056	16.26028	Adipose_R1_peak_4	Intron (uc007r	13	78198017	78236564	38548	1	320174	uc029sch.1	-55683	NA	A830082K12R	RIKEN cDNA A830082K12 gene
chr13	78142130	78142258	129	130	78142194	30	7.24477	2.85875	4.21917	Adipose_R2_peak_5	Intron (uc007r	13	78198017	78236564	38548	1	320174	uc029sch.1	-55759	NA	A830082K12R	RIKEN cDNA A830082K12 gene
chr13	78262736	78262894	159	160	78262803	21	8.43286	3.95423	5.34896	Adipose_R1_peak_4	Intron (uc007r	13	78188973	78198982	10010	2	13865	uc007rhe.2	-63754	ENSMUSG000	Nr2f1	nuclear receptor subfamily 2, group F, member 1
chr13	78263728	78263925	198	199	78263816	23	9.86443	4.31371	6.70247	Adipose_R1_peak_4	Intron (uc007r	13	78188973	78198982	10010	2	13865	uc007rhe.2	-64746	ENSMUSG000	Nr2f1	nuclear receptor subfamily 2, group F, member 1
chr13	78264186	78264645	460	461	78264267	28	13.73721	5.2124	10.41463	Adipose_R1_peak_4	Intron (uc007r	13	78188973	78198982	10010	2	13865	uc007rhe.2	-65204	ENSMUSG000	Nr2f1	nuclear receptor subfamily 2, group F, member 1
chr13	78264201	78264624	424	425	78264265	38	11.72962	3.59649	8.4047	Adipose_R2_peak_5	Intron (uc007r	13	78188973	78198982	10010	2	13865	uc007rhe.2	-65219	ENSMUSG000	Nr2f1	nuclear receptor subfamily 2, group F, member 1
chr13	78357626	78357786	161	162	78357705	22	9.13959	4.13397	6.01526	Adipose_R1_peak_4	Distal Interger	13	78188973	78198982	10010	2	13865	uc007rhe.2	-158644	ENSMUSG000	Nr2f1	nuclear receptor subfamily 2, group F, member 1
chr13	78462969	78463156	188	189	78463050	25	11.36566	4.67319	8.13482	Adipose_R1_peak_4	Distal Interger	13	78188973	78198982	10010	2	13865	uc007rhe.2	-263987	ENSMUSG000	Nr2f1	nuclear receptor subfamily 2, group F, member 1
chr13	78462996	78463111	116	117	78463055	28	6.25023	2.67431	3.31626	Adipose_R2_peak_5	Distal Interger	13	78188973	78198982	10010	2	13865	uc007rhe.2	-264014	ENSMUSG000	Nr2f1	nuclear receptor subfamily 2, group F, member 1
chr13	78788372	78788526	155	156	78788432	16	5.20017	3.05554	2.38734	Adipose_R1_peak_4	Distal Interger	13	78188973	78198982	10010	2	13865	uc007rhe.2	-589390	ENSMUSG000	Nr2f1	nuclear receptor subfamily 2, group F, member 1
chr13	78841359	78841566	208	209	78841442	26	12.14079	4.85292	8.87838	Adipose_R1_peak_4	Distal Interger	13	78188973	78198982	10010	2	13865	uc007rhe.2	-642377	ENSMUSG000	Nr2f1	nuclear receptor subfamily 2, group F, member 1
chr13	78948786	78949072	287	288	78948917	77	62.16646	12.82639	58.06779	Adipose_R1_peak_4	Distal Interger	13	78188973	78198982	10010	2	13865	uc007rhe.2	-749804	ENSMUSG000	Nr2f1	nuclear receptor subfamily 2, group F, member 1
chr13	78948809	78949039	231	232	78948904	47	17.60017	4.42645	14.02531	Adipose_R2_peak_5	Distal Interger	13	78188973	78198982	10010	2	13865	uc007rhe.2	-749827	ENSMUSG000	Nr2f1	nuclear receptor subfamily 2, group F, member 1
chr13	79127369	79127680	312	313	79127500	43	25.24586	7.13919	21.63479	Adipose_R1_peak_4	Distal Interger	13	78188973	78198982	10010	2	13865	uc007rhe.2	-928387	ENSMUSG000	Nr2f1	nuclear receptor subfamily 2, group F, member 1
chr13	79127443	79127578	136	137	79127510	33	8.83596	3.1354	5.68652	Adipose_R2_peak_5	Distal Interger	13	78188973	78198982	10010	2	13865	uc007rhe.2	-928461	ENSMUSG000	Nr2f1	nuclear receptor subfamily 2, group F, member 1
chr13	79481787	79482092	306	307	79481897	55	39.87613	10.06532	36.01168	Adipose_R1_peak_4	Distal Interger	13	78188973	78198982	10010	2	13865	uc007rhe.2	-1282805	ENSMUSG000	Nr2f1	nuclear receptor subfamily 2, group F, member 1
chr13	79481821	79482049	229	230	79481900	59	26.5291	5.53306	22.7188	Adipose_R2_peak_5	Distal Interger	13	78188973	78198982	10010	2	13865	uc007rhe.2	-1282839	ENSMUSG000	Nr2f1	nuclear receptor subfamily 2, group F, member 1
chr13	79537109	79537239	131	132	79537179	19	7.07687	3.59476	4.08458	Adipose_R1_peak_4	Distal Interger	13	78188973	78198982	10010	2	13865	uc007rhe.2	-1338127	ENSMUSG000	Nr2f1	nuclear receptor subfamily 2, group F, member 1
chr13	79803114	79803370	257	258	79803242	37	21.59274	6.83004	18.04766	Adipose_R1_peak_4	Distal Interger	13	80883422	80889643	6222	1	105171	uc007rhh.1	-1080052	ENSMUSG000	Arrdc3	arrestin domain containing 3
chr13	79803162	79803368	207	208	79803244	39	12.34187	3.68871	8.98501	Adipose_R2_peak_5	Distal Interger	13	80883422	80889643	6222	1	105171	uc007rhh.1	-1080054	ENSMUSG000	Arrdc3	arrestin domain containing 3
chr13	80026153	80026371	219	220	80026246	25	11.36566	4.67319	8.13482	Adipose_R1_peak_4	Distal Interger	13	80883422	80889643	6222	1	105171	uc007rhh.1	-857051	ENSMUSG000	Arrdc3	arrestin domain containing 3
chr13	80026210	80026361	152	153	80026253	32	8.29282	3.04319	5.18352	Adipose_R2_peak_5	Distal Interger	13	80883422	80889643	6222	1	105171	uc007rhh.1	-857061	ENSMUSG000	Arrdc3	arrestin domain containing 3
chr13	80027792	80027959	168	169	80027886	23	9.86443	4.31371	6.70247	Adipose_R1_peak_4	Distal Interger	13	80883422	80889643	6222	1	105171	uc007rhh.1	-855463	ENSMUSG000	Arrdc3	arrestin domain containing 3
chr13	80052682	80053049	368	369	80052887	112	107.5239	18.83567	103.1587	Adipose_R1_peak_4	Distal Interger	13	80883422	80889643	6222	1	105171	uc007rhh.1	-830373	ENSMUSG000	Arrdc3	arrestin domain containing 3
chr13	80052725	80053041	317	318	80052895	107	71.0941	9.95952	66.74427	Adipose_R2_peak_5	Distal Interger	13	80883422	80889643	6222	1	105171	uc007rhh.1	-830381	ENSMUSG000	Arrdc3	arrestin domain containing 3
chr13	80182484	80182645	162	163	80182612	23	9.86443	4.31371	6.70247	Adipose_R1_peak_4	Distal Interger	13	80883422	80889643	6222	1	105171	uc007rhh.1	-700777	ENSMUSG000	Arrdc3	arrestin domain containing 3
chr13	80359534	80359739	206	207	80359635	45	29.34988	8.26794	25.64927	Adipose_R1_peak_4	Distal Interger	13	80883422	80889643	6222	1	105171	uc007rhh.1	-523683	ENSMUSG000	Arrdc3	arrestin domain containing 3
chr13	80633776	80634065	290	291	80633929	55	37.84452	9.33449	34.0089	Adipose_R1_peak_4	Distal Interger	13	80883422	80889643	6222	1	105171	uc007rhh.1	-249357	ENSMUSG000	Arrdc3	arrestin domain containing 3
chr13	80633783	80634011	229	230	80633917	52	21.18119	4.88754	17.49655	Adipose_R2_peak_5	Distal Interger	13	80883422	80889643	6222	1	105171	uc007rhh.1	-249411	ENSMUSG000	Arrdc3	arrestin domain containing 3
chr13	80735384	80735540	157	158	80735446	20	7.74501	3.7745	4.70467	Adipose_R1_peak_4	Distal Interger	13	80883422	80889643	6222	1	105171	uc007rhh.1	-147882	ENSMUSG000	Arrdc3	arrestin domain containing 3
chr13	80883924	80884061	138	139	80883992	18	6.42934	3.41502	3.48999	Adipose_R1_peak_4	Promoter (<=:	13	80883422	80889643	6222	1	105171	uc007rhh.1	502	ENSMUSG000	Arrdc3	arrestin domain containing 3
chr13	80884600	80884776	177	178	80884664	16	5.20017	3.05554	2.38734	Adipose_R1_peak_4	Promoter (1-2	13	80883422	80889643	6222	1	105171	uc007rhh.1	1178	ENSMUSG000	Arrdc3	arrestin domain containing 3
chr13	81304939	81305081	143	144	81304983	18	6.42934	3.41502	3.48999	Adipose_R1_peak_4	Intron (uc011z	13	81095068	81444578	349511	2	110789	uc011zcf.1	139497	ENSMUSG000	Adgrv1	adhesion G protein-coupled receptor V1
chr13	81453212	81453491	280	281	81453361	57	24.96362	5.34863	21.18596	Adipose_R2_peak_5	Intron (uc007r	13	81095068	81444578	349511	2	110789	uc011zcf.1	-8634	ENSMUSG000	Adgrv1	adhesion G protein-coupled receptor V1
chr13	81453242	81453510	269	270	81453373	57	42.07807	10.4248	38.18469	Adipose_R1_peak_4	Intron (uc007r	13	81095068	81444578	349511	2	110789	uc011zcf.1	-8664	ENSMUSG000	Adgrv1	adhesion G protein-coupled receptor V1
chr13	81586308	81586533	226	227	81586421	32	17.10103	5.93135	13.67137	Adipose_R1_peak_4	Promoter (1-2	13	81505732	81584856	79125	2	110789	uc007rhq.1	-1452	ENSMUSG000	Adgrv1	adhesion G protein-coupled receptor V1
chr13	81586339	81586522	184	185	81586427	39	12.34187	3.68871	8.98501	Adipose_R2_peak_5	Promoter (1-2	13	81505732	81584856	79125	2	110789	uc007rhq.1	-1483	ENSMUSG000	Adgrv1	adhesion G protein-coupled receptor V1
chr13	81588090	81588323	234	235	81588203	34	18.86162	6.29083	15.38482	Adipose_R1_peak_4	Intron (uc007r	13	81505732	81584856	79125	2	110789	uc007rhq.1	-3234	ENSMUSG000	Adgrv1	adhesion G protein-coupled receptor V1

chr13	82020601	82020750	150	151	82020698	27	5.77405	2.5821	2.88921	Adipose_R2_peak_5	Distal Interger	13	81783292	81797157	13866	1	12626	uc007rid.1	237309	ENSMUSG000	Cetn3	centrin 3
chr13	82051539	82051646	108	109	82051621	28	6.25023	2.67431	3.31626	Adipose_R2_peak_5	Distal Interger	13	81783292	81797157	13866	1	12626	uc007rid.1	268247	ENSMUSG000	Cetn3	centrin 3
chr13	82111731	82111959	229	230	82111848	32	17.10103	5.93135	13.67137	Adipose_R1_peak_4	Distal Interger	13	81783292	81797157	13866	1	12626	uc007rid.1	328439	ENSMUSG000	Cetn3	centrin 3
chr13	82111754	82111904	151	152	82111845	33	8.83596	3.1354	5.68652	Adipose_R2_peak_5	Distal Interger	13	81783292	81797157	13866	1	12626	uc007rid.1	328462	ENSMUSG000	Cetn3	centrin 3
chr13	82149166	82149545	380	381	82149349	98	92.65971	17.79405	88.36157	Adipose_R1_peak_4	Distal Interger	13	81783292	81797157	13866	1	12626	uc007rid.1	365874	ENSMUSG000	Cetn3	centrin 3
chr13	82149223	82149485	263	264	82149341	68	33.91425	6.36302	29.97245	Adipose_R2_peak_5	Distal Interger	13	81783292	81797157	13866	1	12626	uc007rid.1	365931	ENSMUSG000	Cetn3	centrin 3
chr13	82817337	82817496	160	161	82817408	26	12.14079	4.85292	8.87838	Adipose_R1_peak_4	Distal Interger	13	82698275	82698333	59	2	1.01E+08	uc056yul.1	-119004	ENSMUSG000	Mir3961	microRNA 3961
chr13	82817359	82817470	112	113	82817407	31	7.7623	2.95097	4.69388	Adipose_R2_peak_5	Distal Interger	13	82698275	82698333	59	2	1.01E+08	uc056yul.1	-119026	ENSMUSG000	Mir3961	microRNA 3961
chr13	82829864	82830055	192	193	82829893	17	5.80341	3.23528	2.92378	Adipose_R1_peak_4	Distal Interger	13	82698275	82698333	59	2	1.01E+08	uc056yul.1	-131531	ENSMUSG000	Mir3961	microRNA 3961
chr13	83049368	83049600	233	234	83049470	32	17.10103	5.93135	13.67137	Adipose_R1_peak_4	Distal Interger	13	82698275	82698333	59	2	1.01E+08	uc056yul.1	-351035	ENSMUSG000	Mir3961	microRNA 3961
chr13	83423681	83423927	247	248	83423822	33	17.97508	6.11109	14.52164	Adipose_R1_peak_4	Distal Interger	13	83504034	83667079	163046	1	17260	uc007rie.2	-80107	ENSMUSG000	Mef2c	myocyte enhancer factor 2C
chr13	83470935	83471159	225	226	83471052	37	11.12817	3.50427	7.83613	Adipose_R2_peak_5	Distal Interger	13	83504034	83667079	163046	1	17260	uc007rie.2	-32875	ENSMUSG000	Mef2c	myocyte enhancer factor 2C
chr13	84037058	84037260	203	204	84037080	16	5.20017	3.05554	2.38734	Adipose_R1_peak_4	Intron (uc007r	13	84025297	84064774	39478	2	553095	uc007rin.2	27514	ENSMUSG000	Gm17750	predicted gene, 17750
chr13	84110784	84111211	428	429	84110988	171	188.6731	27.18476	184.0656	Adipose_R1_peak_4	Distal Interger	13	84025297	84064774	39478	2	553095	uc007rin.2	-46010	ENSMUSG000	Gm17750	predicted gene, 17750
chr13	84110817	84111170	354	355	84110996	154	123.9861	14.29375	119.3959	Adipose_R2_peak_5	Distal Interger	13	84025297	84064774	39478	2	553095	uc007rin.2	-46043	ENSMUSG000	Gm17750	predicted gene, 17750
chr13	84359774	84360015	242	243	84359905	28	13.73721	5.2124	10.41463	Adipose_R1_peak_4	Distal Interger	13	84222296	84295966	73671	1	72745	uc007rip.2	137478	ENSMUSG000	Tmem161b	transmembrane protein 161B
chr13	84359848	84359962	115	116	84359902	29	6.74062	2.76653	3.75989	Adipose_R2_peak_5	Distal Interger	13	84222296	84295966	73671	1	72745	uc007rip.2	137552	ENSMUSG000	Tmem161b	transmembrane protein 161B
chr13	84506021	84506255	235	236	84506150	43	27.35048	7.90847	23.68561	Adipose_R1_peak_4	Distal Interger	13	84222296	84295966	73671	1	72745	uc007rip.2	283725	ENSMUSG000	Tmem161b	transmembrane protein 161B
chr13	84648752	84648939	188	189	84648825	21	8.43286	3.95423	5.34896	Adipose_R1_peak_4	Distal Interger	13	84222296	84295966	73671	1	72745	uc007rip.2	426456	ENSMUSG000	Tmem161b	transmembrane protein 161B
chr13	84912672	84912988	317	318	84912849	71	58.27042	12.94113	54.20487	Adipose_R1_peak_4	Distal Interger	13	85189477	85202496	13020	1	66671	uc007ris.1	-276489	ENSMUSG000	Ccnh	cyclin H
chr13	84912706	84912971	266	267	84912836	61	28.12311	5.7175	24.28203	Adipose_R2_peak_5	Distal Interger	13	85189477	85202496	13020	1	66671	uc007ris.1	-276506	ENSMUSG000	Ccnh	cyclin H
chr13	84963294	84963532	239	240	84963405	37	21.59274	6.83004	18.04766	Adipose_R1_peak_4	Distal Interger	13	85189477	85202496	13020	1	66671	uc007ris.1	-225945	ENSMUSG000	Ccnh	cyclin H
chr13	84963322	84963521	200	201	84963394	41	13.59774	3.87315	10.1803	Adipose_R2_peak_5	Distal Interger	13	85189477	85202496	13020	1	66671	uc007ris.1	-225956	ENSMUSG000	Ccnh	cyclin H
chr13	84964048	84964410	363	364	84964273	49	33.3472	8.94538	29.59006	Adipose_R1_peak_4	Distal Interger	13	85189477	85202496	13020	1	66671	uc007ris.1	-225067	ENSMUSG000	Ccnh	cyclin H
chr13	84964150	84964401	252	253	84964272	55	23.42763	5.16419	19.68558	Adipose_R2_peak_5	Distal Interger	13	85189477	85202496	13020	1	66671	uc007ris.1	-225076	ENSMUSG000	Ccnh	cyclin H
chr13	85179081	85179304	224	225	85179194	20	7.74501	3.7745	4.70467	Adipose_R1_peak_4	Distal Interger	13	85189477	85202496	13020	1	66671	uc007ris.1	-10173	ENSMUSG000	Ccnh	cyclin H
chr13	85405344	85405547	204	205	85405430	29	14.55748	5.39214	11.20652	Adipose_R1_peak_4	Distal Interger	13	85214699	85289486	74788	2	218397	uc007rix.2	-115858	ENSMUSG000	Rasa1	RAS p21 protein activator 1
chr13	85468124	85468332	209	210	85468233	31	16.23981	5.75161	12.83549	Adipose_R1_peak_4	Distal Interger	13	85214699	85289486	74788	2	218397	uc007rix.2	-178638	ENSMUSG000	Rasa1	RAS p21 protein activator 1
chr13	85469073	85469500	428	429	85469237	58	43.19021	10.60454	39.28351	Adipose_R1_peak_4	Distal Interger	13	85214699	85289486	74788	2	218397	uc007rix.2	-179587	ENSMUSG000	Rasa1	RAS p21 protein activator 1
chr13	85469080	85469496	417	418	85469333	60	27.32259	5.62528	23.49644	Adipose_R2_peak_5	Distal Interger	13	85214699	85289486	74788	2	218397	uc007rix.2	-179594	ENSMUSG000	Rasa1	RAS p21 protein activator 1
chr13	85672734	85673191	458	459	85672867	50	34.50784	9.16663	30.72138	Adipose_R1_peak_4	Distal Interger	13	86044798	86046795	1998	2	12867	uc007riy.1	373604	ENSMUSG000	Cox7c	cytochrome c oxidase subunit VIIc
chr13	85672788	85672992	205	206	85672879	45	16.22858	4.24202	12.70278	Adipose_R2_peak_5	Distal Interger	13	86044798	86046795	1998	2	12867	uc007riy.1	373803	ENSMUSG000	Cox7c	cytochrome c oxidase subunit VIIc
chr13	85727123	85727273	151	152	85727205	23	9.86443	4.31371	6.70247	Adipose_R1_peak_4	Distal Interger	13	86044798	86046795	1998	2	12867	uc007riy.1	319522	ENSMUSG000	Cox7c	cytochrome c oxidase subunit VIIc
chr13	85728305	85728442	138	139	85728412	18	6.42934	3.41502	3.48999	Adipose_R1_peak_4	Distal Interger	13	86044798	86046795	1998	2	12867	uc007riy.1	318353	ENSMUSG000	Cox7c	cytochrome c oxidase subunit VIIc
chr13	85730068	85730316	249	250	85730190	36	20.67077	6.6503	17.14779	Adipose_R1_peak_4	Distal Interger	13	86044798	86046795	1998	2	12867	uc007riy.1	316479	ENSMUSG000	Cox7c	cytochrome c oxidase subunit VIIc
chr13	85730119	85730258	140	141	85730185	30	7.24477	2.85875	4.21917	Adipose_R2_peak_5	Distal Interger	13	86044798	86046795	1998	2	12867	uc007riy.1	316537	ENSMUSG000	Cox7c	cytochrome c oxidase subunit VIIc
chr13	85879440	85879705	266	267	85879568	51	35.56531	9.34637	31.76235	Adipose_R1_peak_4	Distal Interger	13	86044798	86046795	1998	2	12867	uc007riy.1	167090	ENSMUSG000	Cox7c	cytochrome c oxidase subunit VIIc
chr13	85879443	85879674	232	233	85879571	47	17.60017	4.42645	14.02531	Adipose_R2_peak_5	Distal Interger	13	86044798	86046795	1998	2	12867	uc007riy.1	167121	ENSMUSG000	Cox7c	cytochrome c oxidase subunit VIIc
chr13	85946673	85946986	314	315	85946815	76	64.35061	13.83982	60.23161	Adipose_R1_peak_4	Distal Interger	13	86044798	86046795	1998	2	12867	uc007riy.1	99809	ENSMUSG000	Cox7c	cytochrome c oxidase subunit VIIc
chr13	85946675	85946924	250	251	85946821	72	37.3624	6.73189	33.36734	Adipose_R2_peak_5	Distal Interger	13	86044798	86046795	1998	2	12867	uc007riy.1	99871	ENSMUSG000	Cox7c	cytochrome c oxidase subunit VIIc
chr13	85963954	85964160	207	208	85964140	23	9.86443	4.31371	6.70247	Adipose_R1_peak_4	Distal Interger	13	86044798	86046795	1998	2	12867	uc007riy.1	82635	ENSMUSG000	Cox7c	cytochrome c oxidase subunit VIIc
chr13	86166387	86166638	252	253	86166516	26	12.14079	4.85292	8.87838	Adipose_R1_peak_4	Distal Interger	13	86044798	86046795	1998	2	12867	uc007riy.1	-119592	ENSMUSG000	Cox7c	cytochrome c oxidase subunit VIIc
chr13	86166406	86166634	229	230	86166523	36	10.5378	3.41206	7.27949	Adipose_R2_peak_5	Distal Interger	13	86044798	86046795	1998	2	12867	uc007riy.1	-119611	ENSMUSG000	Cox7c	cytochrome c oxidase subunit VIIc
chr13	86168686	86169136	451	452	86168860	132	87.24852	10.12998	82.97955	Adipose_R1_peak_4	Distal Interger	13	86044798	86046795	1998	2	12867	uc007riy.1	-121891	ENSMUSG000	Cox7c	cytochrome c oxidase subunit VIIc
chr13	86168727	86168998	272	273	86168856	99	44.64793	5.91512	40.55559	Adipose_R2_peak_5	Distal Interger	13	86044798	86046795	1998	2	12867	uc007riy.1	-121932	ENSMUSG000	Cox7c	cytochrome c oxidase subunit VIIc
chr13	86186221	86186382	162	163	86186293	20	7.74501	3.7745	4.70467	Adipose_R1_peak_4	Distal Interger	13	86044798	86046795	1998	2	12867	uc007riy.1	-139426	ENSMUSG000	Cox7c	cytochrome c oxidase subunit VIIc
chr13	86228038	86228225	188	189	86228126	23	9.86443	4.31371	6.70247	Adipose_R1_peak_4	Distal Interger	13	86044798	86046795	1998	2	12867	uc007riy.1	-181243	ENSMUSG000	Cox7c	cytochrome c oxidase subunit VIIc
chr13	86228683	86228929	247	248	86228820	37	21.59274	6.83004	18.04766	Adipose_R1_peak_4	Distal Interger	13	86044798	86046795	1998	2	12867	uc007riy.1	-181888	ENSMUSG000	Cox7c	cytochrome c oxidase subunit VIIc
chr13	86228692	86228896	205	206	86228815	34	9.39139	3.22762	6.20474	Adipose_R2_peak_5	Distal Interger	13	86044798	86046795	1998	2	12867	uc007riy.1	-181897	ENSMUSG000	Cox7c	cytochrome c oxidase subunit VIIc
chr13	86258955	86259126	172	173	86259049	28	13.73721	5.2124	10.41463	Adipose_R1_peak_4	Distal Interger	13	86044798	86046795	1998	2	12867	uc007riy.1	-212160	ENSMUSG000	Cox7c	cytochrome c oxidase subunit VIIc
chr13	86288695	86289293	599	600	86288803	31	16.12273	5.70563	12.741	Adipose_R1_peak_4	Distal Interger	13	86044798	86046795	1998	2	12867	uc007riy.1	-241900	ENSMUSG000	Cox7c	cytochrome c oxidase subunit VIIc
chr13	86288702	86289281	580	581	86288791	41	13.59774	3.87315	10.1803	Adipose_R2_peak_5	Distal Interger	13	86044798	86046795	1998	2	12867	uc007riy.1	-241907	ENSMUSG000	Cox7c	cytochrome c oxidase subunit VIIc
chr13	86294200	86294427	228	229	86294306	35	19.76029	6.47056	16.26028	Adipose_R1_peak_4	Distal Interger	13	86044798	86046795	1998	2	12867	uc007riy.1	-247405	ENSMUSG000	Cox7c	cytochrome c oxidase subunit VIIc
chr13	86294251	86294368	118	119	86294311	33	8.83596	3.1354	5.68652	Adipose_R2_peak_5	Distal Interger	13	86044798	86046795	1998	2	12867	uc007riy.1	-247456	ENSMUSG000	Cox7c	cytochrome c oxidase subunit VIIc
chr13	86302173	86302356	184	185	86302268	25	11.36566	4.67319	8.13482	Adipose_R1_peak_4	Distal Interger	13	86044798	86046795	1998	2	12867	uc007riy.1	-255378	ENSMUSG000	Cox7c	cytochrome c oxidase subunit VIIc
chr13	86636662	86636805	144	145	86636725	20	7.74501	3.7745	4.70467	Adipose_R1_peak_4	Distal Interger	13	86044798	86046795	1998	2	12867	uc007riy.1	-589867	ENSMUSG000	Cox7c	cytochrome c oxidase subunit VIIc
chr13	86687019	86687149	131	132	86687083	19	7.07687	3.59476	4.08458	Adipose_R1_peak_4	Distal Interger	13	86044798	86046795	1998	2	12867	uc007riy.1	-640224	ENSMUSG000	Cox7c	cytochrome c oxidase subunit VIIc
chr13	86732539	86732760	222	223	86732665	31	16.23981	5.75161	12.83549	Adipose_R1_peak_4	Distal Interger	13	86044798	86046795	1998	2	12867	uc007riy.1	-685744	ENSMUSG000	Cox7c	cytochrome c oxidase subunit VIIc
chr13	86732606	86732733	128	129	86732663	27	5.77405	2.5821	2.88921	Adipose_R2_peak_5	Distal Interger	13	86044798	86046795	1998	2	12867	uc007riy.1	-685811	ENSMUSG000	Cox7c	cytochrome c oxidase subunit VIIc
chr13	86754342	86754536	195	196	86754448	29	14.55748	5.39214	11.20652	Adipose_R1_peak_4	Distal Interger	13	86044798	86046795	1998	2	12867	uc007riy.1	-707547	ENSMUSG000	Cox7c	cytochrome c oxidase subunit VIIc
chr13	87054500	87054670	171	172	87054565	21	8.43286	3.95423	5.34896	Adipose_R1_peak_4	Distal Interger	13	86044798	86046795	1998	2	12867	uc007riy.1	-1007705	ENSMUSG000	Cox7c	cytochrome c oxidase subunit VIIc
chr13	87065180	87065467	288	289	87065314	43	27.35048	7.90847	23.68561	Adipose_R1_peak_4	Distal Interger	13	86044798	86046795	1998	2	12867	uc007riy.1	-1018385	ENSMUSG000	Cox7c	cytochrome c oxidase subunit VIIc
chr13	87065204	87065412	209	210	87065295	43	14.8939	4.05758	11.42017	Adipose_R2_peak_5	Distal Interger	13	86044798	86046795	1998	2	12867	uc007riy.1	-1018409	ENSMUSG000	Cox7c	cytochrome c oxidase subunit VIIc
chr13	87140771	87140910	140	141	87140843	25	11.36566	4.67319	8.13482	Adipose_R1_peak_4	Distal Interger	13	86044798	86046795	1998	2	12867	uc007riy.1	-1093976	ENSMUSG000	Cox7c	cytochrome c oxidase subunit VIIc
chr13	87141176	87141374	199	200	87141264	29	14.55748	5.39214	11.20652	Adipose_R1_peak_4	Distal Interger	13	86044798	86046795	1998	2	12867	uc007riy.1	-1094381	ENSMUSG000	Cox7c	cytochrome c oxidase subunit VIIc
chr13	87188861	87189260	400	401	87189043	151	164.0965	25.68739	159.5468	Adipose_R1_peak_4	Distal Interger	13	86044798	86046795	1998	2	12867	uc007riy.1	-1142066	ENSMUSG000	Cox7c	cytochrome c oxidase subunit VIIc
chr13	87188892	87189225	334	335	87189047	146	114.4869	13.55601	109.9246	Adipose_R2_peak_5	Distal Interger	13	86044798	86046795	1998	2	12867	uc007riy.1	-1142097	ENSMUSG000	Cox7c	cytochrome c oxidase subunit VIIc
chr13	87272640	87272825	186	187	87272702	28	6.25023	2.67431	3.31626	Adipose_R2_peak_5	Distal Interger	13	86044798	86046795	1998	2	12867	uc007riy.1	-1225845	ENSMUSG000	Cox7c	cytochrome c oxidase subunit VIIc
chr13	87272686	87273128	443	444	87272828	28	13.73721	5.2124	10.41463	Adipose_R1_peak_4	Distal Interger	13	86044798	86046795	1998	2	12867	uc007riy.1	-1225891	ENSMUSG000	Cox7c	cytochrome c oxidase subunit VIIc
chr13	87282389	87282565	177	178	87282444	36	10.5378	3.41206	7.27949	Adipose_R2_peak_5	Distal Interger	13	86044798	86046795	1998	2	12867	uc007riy.1	-1235594	ENSMUSG000	Cox7c	cytochrome c oxidase subunit VIIc
chr13	87356551	87356808	258	259	87356686	38	22.52591	7.00978	18.96	Adipose_R1_peak_4	Distal Interger	13	86044798	86046795	1998	2	12867	uc007riy.1	-1309756	ENSMUSG000	Cox7c	cytochrome c oxidase subunit VIIc
chr13	87356591	87356747	157	158	87356678	36	10.5378	3.41206	7.27949	Adipose_R2_peak_5	Distal Interger	13	86044798	86046795	1998	2	12867	uc007riy.1	-1309796	ENSMUSG000	Cox7c	cytochrome c oxidase subunit VIIc
chr13	87503849	87504106	258	259	87503982	53	37.70483	9.70585	33.87	Adipose_R1_peak_4	Distal Interger	13	88821472	88978240	156769	1	13612	uc007riz.2	-1317366	ENSMUSG000	Edil3	EGF-like repeats and discoidin I-like domains 3
chr13	87503887	87504106	220	221	87504025	51	20.44827	4.79532	16.78434	Adipose_R2_peak_5	Distal Interger	13	88821472	88978240	156769	1	13612	uc007riz.2	-1317366	ENSMUSG000	Edil3	EGF-like repeats and discoidin I-like domains 3
chr13	87506308	87506553	246	247	87506447	31	16.23981	5.75161	12.83549	Adipose_R1_peak_4	Distal Interger	13	88821472	88978240	156769	1	13612	uc007riz.2	-1314919	ENSMUSG000	Edil3	EGF-like repeats and discoidin I-like domains 3
chr13	87525082	87525219	138	139	87525101	18	6.42934	3.41502	3.48999	Adipose_R1_peak_4	Distal Interger	13	88821472	88978240	156769	1	13612	uc007riz.2	-1296253	ENSMUSG000	Edil3	EGF-like repeats and discoidin I-like domains 3
chr13	87684122	87684313	192	193	87684223	26	12.14079	4.85292	8.87838	Adipose_R1_peak_4	Distal Interger	13	88821472	88978240	156769	1	13612	uc007riz.2	-1137159	ENSMUSG000	Edil3	EGF-like repeats and discoidin I-like domains 3
chr13	87753735	87753932	198	199	87753781	16	5.20017	3.05554	2.38734	Adipose_R1_peak_4	Distal Interger	13	88821472	88978240	156769	1	13612	uc007riz.2	-1067540	ENSMUSG000	Edil3	EGF-like repeats and discoidin I-like domains 3
chr13	87891468	87891744	277	278	87891598	30	15.39182	5.57188	12.01394	Adipose_R1_peak_4	Distal Interger	13	88821472	88978240	156769	1	13612	uc007riz.2	-929728	ENSMUSG000	Edil3	EGF-like repeats and discoidin I-like domains 3
chr13	87934076	87934439	364	365	87934334	22	9.13959	4.13397	6.01526	Adipose_R1_peak_4	Distal Interger	13	88821472	88978240	156769	1	13612	uc007riz.2	-887033	ENSMUSG000	Edil3	EGF-like repeats and discoidin I-like domains 3
chr13	88045880	88046201	322	323	88046042	104	87.30187	14.52567	83.03276	Adipose_R1_peak_4	Distal Interger	13	88821472	88978240	156769	1	13612	uc007riz.2	-775271	ENSMUSG000	Edil3	EGF-like repeats and discoidin I-like domains 3
chr13	88238015	88238192	178	179	88238120	24	10.60668	4.49345	7.40961	Adipose_R1_peak_4	Distal Interger	13	88821472	88978240	156769	1	13612	uc007riz.2	-583280	ENSMUSG000	Edil3	EGF-like repeats and discoidin I-like domains 3
chr13	88491746	88491884	139	140	88491805	68	33.91425	6.36302	29.97245	Adipose_R2_peak_5	Distal Interger	13	88821472	88978240	156769	1	13612	uc007riz.2	-329588	ENSMUSG000	Edil3	EGF-like repeats and discoidin I-like domains 3
chr13	88991595	88991861	267	268	88991723	48	32.41826	8.80716	28.66523	Adipose_R1_peak_4	Intron (uc007r	13	88821472	88978240	156769	1	13612	uc007riz.2	170123	ENSMUSG000	Edil3	EGF-like repeats and discoidin I-like domains 3
chr13	88991601	88991808	208	209	88991719	47	17.60017	4.42645	14.02531	Adipose_R2_peak_5	Intron (uc007r	13	88821472	88978240	156769	1	13612	uc007riz.2	170129	ENSMUSG000	Edil3	EGF-like repeats and discoidin I-like domains 3
chr13	89219777	89220030	254	255	89219855	29	14.55748	5.39214	11.20652	Adipose_R1_peak_4	Intron (uc007r	13	89540636	89611832	71197	1	12950	uc007rjf.2	-320606	ENSMUSG000	Hapln1	hyaluronan and proteoglycan link protein 1
chr13	89219813	89219923	111	112	89219858	29	6.74062	2.76653	3.75989	Adipose_R2_peak_5	Intron (uc007r	13	89540636	89611832	71197	1	12950	uc007rjf.2	-320713	ENSMUSG000	Hapln1	hyaluronan and proteoglycan link protein 1
chr13	89448666	89448856	191	192	89448757	25	11.36566	4.67319	8.13482	Adipose_R1_peak_4	Distal Interger	13	89540636	89611832	71197	1	12950	uc007rjf.2	-91780	ENSMUSG000	Hapln1	hyaluronan and proteoglycan link protein 1
chr13	89448696	89448815	120	121	89448748	32	8.29282	3.04319	5.18352	Adipose_R2_peak_5	Distal Interger	13	89540636	89611832	71197	1	12950	uc007rjf.2	-91821	ENSMUSG000	Hapln1	hyaluronan and proteoglycan link protein 1
chr13	89628516	89628784	269	270	89628645	58	43.19021	10.60454	39.28351	Adipose_R1_peak_4	Distal Interger	13	89540636	89611832	71197	1	12950	uc007rjf.2	87880	ENSMUSG000	Hapln1	hyaluronan and proteoglycan link protein 1
chr13	89628524	89628724	201	202	89628648	48	18.29934	4.51867	14.70076	Adipose_R2_peak_5	Distal Interger	13	89540636	89611832	71197	1	12950	uc007rjf.2	87888	ENSMUSG000	Hapln1	hyaluronan and proteoglycan link protein 1
chr13	90215715	90215892	178	179	90215793	20	7.74501	3.7745	4.70467	Adipose_R1_peak_4	Intron (uc007r	13	90089667	90114921	25255	1	66074	uc007rjo.2	126048	ENSMUSG000	Tmem167	transmembrane protein 167
chr13	90321797	90321976	180	181	90321878	23	9.86443	4.31371	6.70247	Adipose_R1_peak_4	Distal Interger	13	90089667	90114921	25255	1	66074	uc007rjo.2	232130	ENSMUSG000	Tmem167	transmembrane protein 167
chr13	90321827	90321979	153	154	90321890	29	6.74062	2.76653	3.75989	Adipose_R2_peak_5	Distal Interger	13	90089667	90114921	25255	1	66074	uc007rjo.2	232160	ENSMUSG000	Tmem167	transmembrane protein 167

chr13	90329093	90329224	132	133	90329157	21	8.43286	3.95423	5.34896	Adipose_R1_peak_	Distal Interger	13	90089667	90114921	25255	1	66074	uc007rjo.2	239426	ENSMUSG000	Tmem167	transmembrane protein 167
chr13	90329096	90329202	107	108	90329146	29	6.74062	2.76653	3.75989	Adipose_R2_peak_	Distal Interger	13	90089667	90114921	25255	1	66074	uc007rjo.2	239429	ENSMUSG000	Tmem167	transmembrane protein 167
chr13	90417087	90417399	313	314	90417264	26	12.14079	4.85292	8.87838	Adipose_R1_peak_	Distal Interger	13	90089667	90114921	25255	1	66074	uc007rjo.2	327420	ENSMUSG000	Tmem167	transmembrane protein 167
chr13	90449390	90449649	260	261	90449512	29	14.55748	5.39214	11.20652	Adipose_R1_peak_	Distal Interger	13	90089667	90114921	25255	1	66074	uc007rjo.2	359723	ENSMUSG000	Tmem167	transmembrane protein 167
chr13	91734884	91735056	173	174	91734987	25	10.36747	4.27547	7.2013	Adipose_R1_peak_	Intron (uc011z	13	91741521	91786148	44628	1	74156	uc007rka.1	-6465	ENSMUSG000	Acot12	acyl-CoA thioesterase 12
chr13	91734943	91735084	142	143	91734996	34	9.39139	3.22762	6.20474	Adipose_R2_peak_	Intron (uc011z	13	91741521	91786148	44628	1	74156	uc007rka.1	-6437	ENSMUSG000	Acot12	acyl-CoA thioesterase 12
chr13	91963827	91964009	183	184	91963906	29	14.55748	5.39214	11.20652	Adipose_R1_peak_	Intron (uc029s	13	91880407	91988042	107636	2	19418	uc029sco.1	24033	ENSMUSG000	Rasgrf2	RAS protein-specific guanine nucleotide-releasing factor 2
chr13	92269059	92269304	246	247	92269161	46	30.36364	8.44768	26.64488	Adipose_R1_peak_	Promoter (2-3	13	92211878	92266072	54195	2	17686	uc007rkg.2	-2987	ENSMUSG000	Msh3	mutS homolog 3
chr13	92269094	92269212	119	120	92269155	30	7.24477	2.85875	4.21917	Adipose_R2_peak_	Intron (uc007r	13	92211878	92266072	54195	2	17686	uc007rkg.2	-3022	ENSMUSG000	Msh3	mutS homolog 3
chr13	92364765	92364955	191	192	92364849	29	6.74062	2.76653	3.75989	Adipose_R2_peak_	Intron (uc007r	13	92211878	92355003	143126	2	17686	uc007rkh.2	-9762	ENSMUSG000	Msh3	mutS homolog 3
chr13	92836651	92836800	150	151	92836719	23	9.86443	4.31371	6.70247	Adipose_R1_peak_	Distal Interger	13	92844787	92858230	13444	1	382793	uc011zct.1	-7987	ENSMUSG000	Mtx3	metaxin 3
chr13	92836675	92836793	119	120	92836737	31	7.7623	2.95097	4.69388	Adipose_R2_peak_	Distal Interger	13	92844787	92858230	13444	1	382793	uc011zct.1	-7994	ENSMUSG000	Mtx3	metaxin 3
chr13	92838755	92838984	230	231	92838877	46	30.36364	8.44768	26.64488	Adipose_R1_peak_	Distal Interger	13	92844787	92858230	13444	1	382793	uc011zct.1	-5803	ENSMUSG000	Mtx3	metaxin 3
chr13	92838771	92838990	220	221	92838868	46	16.90986	4.33423	13.35898	Adipose_R2_peak_	Distal Interger	13	92844787	92858230	13444	1	382793	uc011zct.1	-5797	ENSMUSG000	Mtx3	metaxin 3
chr13	94017443	94017811	369	370	94017628	150	146.0371	20.42614	141.5353	Adipose_R1_peak_	Distal Interger	13	94057796	94195409	137614	1	218454	uc007rlp.1	-39985	ENSMUSG000	Lhfpl2	lipoma HMGIC fusion partner-like 2
chr13	94017487	94017758	272	273	94017628	103	66.95583	9.59064	62.63862	Adipose_R2_peak_	Distal Interger	13	94057796	94195409	137614	1	218454	uc007rlp.1	-40038	ENSMUSG000	Lhfpl2	lipoma HMGIC fusion partner-like 2
chr13	94018754	94018889	136	137	94018861	21	8.43286	3.95423	5.34896	Adipose_R1_peak_	Distal Interger	13	94057796	94195409	137614	1	218454	uc007rlp.1	-38907	ENSMUSG000	Lhfpl2	lipoma HMGIC fusion partner-like 2
chr13	95654340	95654546	207	208	95654424	23	9.86443	4.31371	6.70247	Adipose_R1_peak_	Intron (uc007r	13	95661402	95683052	21651	2	544963	uc007rmp.1	28506	ENSMUSG000	Iqgap2	IQ motif containing GTPase activating protein 2
chr13	95654511	95654637	127	128	95654529	28	6.25023	2.67431	3.31626	Adipose_R2_peak_	Intron (uc007r	13	95661402	95683052	21651	2	544963	uc007rmp.1	28415	ENSMUSG000	Iqgap2	IQ motif containing GTPase activating protein 2
chr13	95845749	95846033	285	286	95845946	83	73.09611	15.09798	68.90916	Adipose_R1_peak_	Intron (uc007r	13	95627177	95891922	264746	2	544963	uc007rmo.1	45889	ENSMUSG000	Iqgap2	IQ motif containing GTPase activating protein 2
chr13	95845750	95846029	280	281	95845955	70	35.62622	6.54746	31.65691	Adipose_R2_peak_	Intron (uc007r	13	95627177	95891922	264746	2	544963	uc007rmo.1	45893	ENSMUSG000	Iqgap2	IQ motif containing GTPase activating protein 2
chr13	95931789	95931955	167	168	95931853	18	6.42934	3.41502	3.48999	Adipose_R1_peak_	Distal Interger	13	95627177	95891922	264746	2	544963	uc007rmo.1	-39867	ENSMUSG000	Iqgap2	IQ motif containing GTPase activating protein 2
chr13	99347224	99347380	157	158	99347285	18	6.42934	3.41502	3.48999	Adipose_R1_peak_	Promoter (2-3	13	99344786	99415561	70776	1	218506	uc007rpn.1	2438	ENSMUSG000	Mrps27	mitochondrial ribosomal protein S27
chr13	99350919	99351012	94	95	99350962	26	5.31252	2.48988	2.47999	Adipose_R2_peak_	Intron (uc007r	13	99344786	99415561	70776	1	218506	uc007rpn.1	6133	ENSMUSG000	Mrps27	mitochondrial ribosomal protein S27
chr13	100225580	100225710	131	132	100225643	18	6.42934	3.41502	3.48999	Adipose_R1_peak_	Intron (uc011z	13	1.00E+08	1.00E+08	35554	2	17951	uc011zdr.1	21583	ENSMUSG000	Naip5	NLR family, apoptosis inhibitory protein 5
chr13	100237432	100237691	260	261	100237565	40	24.42476	7.36925	20.81738	Adipose_R1_peak_	Intron (uc011z	13	1.00E+08	1.00E+08	35554	2	17951	uc011zdr.1	9602	ENSMUSG000	Naip5	NLR family, apoptosis inhibitory protein 5
chr13	100276606	100276717	112	113	100276612	25	4.86612	2.39766	2.09018	Adipose_R2_peak_	Distal Interger	13	1.00E+08	1.00E+08	35597	2	17951	uc007rql.1	-29270	ENSMUSG000	Naip5	NLR family, apoptosis inhibitory protein 5
chr13	100384803	100385104	302	303	100384964	163	134.8857	15.12371	130.2675	Adipose_R2_peak_	Distal Interger	13	1.00E+08	1.00E+08	38742	2	17940	uc007rqr.1	61407	ENSMUSG000	Naip1	NLR family, apoptosis inhibitory protein 1
chr13	100384807	100385121	315	316	100384978	208	237.1328	30.28652	232.4469	Adipose_R1_peak_	Distal Interger	13	1.00E+08	1.00E+08	38742	2	17940	uc007rqr.1	61390	ENSMUSG000	Naip1	NLR family, apoptosis inhibitory protein 1
chr13	100921488	100922081	594	595	100921625	60	41.29473	9.51782	37.41404	Adipose_R1_peak_	Distal Interger	13	1.01E+08	1.01E+08	30780	2	69048	uc007rrr.1	-88061	ENSMUSG000	Slc30a5	solute carrier family 30 (zinc transporter), member 5
chr13	100921525	100921794	270	271	100921624	65	31.39315	6.08637	27.49408	Adipose_R2_peak_	Distal Interger	13	1.01E+08	1.01E+08	30780	2	69048	uc007rrr.1	-88098	ENSMUSG000	Slc30a5	solute carrier family 30 (zinc transporter), member 5
chr13	100984632	100984871	240	241	100984758	34	18.86162	6.29083	15.38482	Adipose_R1_peak_	Distal Interger	13	1.01E+08	1.01E+08	30780	2	69048	uc007rrr.1	-151205	ENSMUSG000	Slc30a5	solute carrier family 30 (zinc transporter), member 5
chr13	101550676	101550864	189	190	101550802	20	7.74501	3.7745	4.70467	Adipose_R1_peak_	Distal Interger	13	1.02E+08	1.02E+08	11870	2	18708	uc007rrs.3	141766	ENSMUSG000	Pik3r1	phosphatidylinositol 3-kinase, regulatory subunit, polypeptide 1 (p85 alpha)
chr13	102158346	102158534	189	190	102158461	27	12.93148	5.03266	9.63827	Adipose_R1_peak_	Distal Interger	13	1.02E+08	1.02E+08	87457	2	18708	uc007rrt.3	-390129	ENSMUSG000	Pik3r1	phosphatidylinositol 3-kinase, regulatory subunit, polypeptide 1 (p85 alpha)
chr13	102158399	102158507	109	110	102158446	28	6.25023	2.67431	3.31626	Adipose_R2_peak_	Distal Interger	13	1.02E+08	1.02E+08	87457	2	18708	uc007rrt.3	-390182	ENSMUSG000	Pik3r1	phosphatidylinositol 3-kinase, regulatory subunit, polypeptide 1 (p85 alpha)
chr13	102384921	102385111	191	192	102385015	25	11.36566	4.67319	8.13482	Adipose_R1_peak_	Distal Interger	13	1.03E+08	1.03E+08	10705	1	17079	uc007rrx.1	-308447	ENSMUSG000	Cd180	CD180 antigen
chr13	102386483	102387208	726	727	102386617	83	51.99998	8.81924	47.99997	Adipose_R1_peak_	Distal Interger	13	1.03E+08	1.03E+08	10705	1	17079	uc007rrx.1	-306350	ENSMUSG000	Cd180	CD180 antigen
chr13	102386530	102387194	665	666	102386639	66	27.99788	5.27658	24.16489	Adipose_R2_peak_	Distal Interger	13	1.03E+08	1.03E+08	10705	1	17079	uc007rrx.1	-306364	ENSMUSG000	Cd180	CD180 antigen
chr13	102607847	102607990	144	145	102607925	22	9.13959	4.13397	6.01526	Adipose_R1_peak_	Distal Interger	13	1.03E+08	1.03E+08	10705	1	17079	uc007rrx.1	-85568	ENSMUSG000	Cd180	CD180 antigen
chr13	102784996	102785130	135	136	102785039	34	9.39139	3.22762	6.20474	Adipose_R2_peak_	Promoter (2-3	13	1.03E+08	1.03E+08	53052	2	328329	uc007rsa.1	2319	ENSMUSG000	Mast4	microtubule associated serine/threonine kinase family member 4
chr13	103275889	103276150	262	263	103276054	35	19.76029	6.47056	16.26028	Adipose_R1_peak_	Intron (uc007r	13	1.03E+08	1.03E+08	602004	2	328329	uc007rsb.2	58342	ENSMUSG000	Mast4	microtubule associated serine/threonine kinase family member 4
chr13	103403191	103403326	136	137	103403254	21	8.43286	3.95423	5.34896	Adipose_R1_peak_	Distal Interger	13	1.03E+08	1.03E+08	602004	2	328329	uc007rsb.2	-68699	ENSMUSG000	Mast4	microtubule associated serine/threonine kinase family member 4
chr13	103406830	103407142	313	314	103406996	93	86.02155	16.89536	81.75695	Adipose_R1_peak_	Distal Interger	13	1.03E+08	1.03E+08	602004	2	328329	uc007rsb.2	-72338	ENSMUSG000	Mast4	microtubule associated serine/threonine kinase family member 4
chr13	103406864	103407136	273	274	103407003	82	46.38432	7.65407	42.27038	Adipose_R2_peak_	Distal Interger	13	1.03E+08	1.03E+08	602004	2	328329	uc007rsb.2	-72372	ENSMUSG000	Mast4	microtubule associated serine/threonine kinase family member 4
chr13	103484721	103484858	138	139	103484782	18	6.42934	3.41502	3.48999	Adipose_R1_peak_	Distal Interger	13	1.03E+08	1.03E+08	602004	2	328329	uc007rsb.2	-150229	ENSMUSG000	Mast4	microtubule associated serine/threonine kinase family member 4
chr13	103585803	103586143	341	342	103585977	168	175.9171	24.20262	171.3401	Adipose_R1_peak_	Distal Interger	13	1.04E+08	1.04E+08	22968	2	218543	uc007rsh.1	178439	ENSMUSG000	Srek1	splicing regulatory glutamine/lysine-rich protein 1
chr13	103585855	103586139	285	286	103585986	117	81.71461	10.88169	77.29195	Adipose_R2_peak_	Distal Interger	13	1.04E+08	1.04E+08	22968	2	218543	uc007rsh.1	178443	ENSMUSG000	Srek1	splicing regulatory glutamine/lysine-rich protein 1
chr13	103756365	103756757	393	394	103756494	30	7.24477	2.85875	4.21917	Adipose_R2_peak_	Intron (uc007r	13	1.04E+08	1.04E+08	22968	2	218543	uc007rsh.1	7825	ENSMUSG000	Srek1	splicing regulatory glutamine/lysine-rich protein 1
chr13	103756463	103756864	402	403	103756636	34	18.86162	6.29083	15.38482	Adipose_R1_peak_	Intron (uc007r	13	1.04E+08	1.04E+08	22968	2	218543	uc007rsh.1	7718	ENSMUSG000	Srek1	splicing regulatory glutamine/lysine-rich protein 1
chr13	103990749	103991362	614	615	103990883	24	10.60668	4.49345	7.40961	Adipose_R1_peak_	Distal Interger	13	1.04E+08	1.04E+08	13129	2	75805	uc007rso.1	45205	ENSMUSG000	Nln	neurolysin (metallopeptidase M3 family)
chr13	103990806	103991172	367	368	103990967	31	7.7623	2.95097	4.69388	Adipose_R2_peak_	Distal Interger	13	1.04E+08	1.04E+08	13129	2	75805	uc007rso.1	45395	ENSMUSG000	Nln	neurolysin (metallopeptidase M3 family)
chr13	104002115	104002385	271	272	104002257	79	62.52428	12.48239	58.42426	Adipose_R1_peak_	Distal Interger	13	1.04E+08	1.04E+08	13129	2	75805	uc007rso.1	34182	ENSMUSG000	Nln	neurolysin (metallopeptidase M3 family)
chr13	104002123	104002382	260	261	104002247	64	30.56565	5.99415	26.6807	Adipose_R2_peak_	Distal Interger	13	1.04E+08	1.04E+08	13129	2	75805	uc007rso.1	34185	ENSMUSG000	Nln	neurolysin (metallopeptidase M3 family)
chr13	104562045	104562447	403	404	104562258	71	58.27042	12.94113	54.20487	Adipose_R1_peak_	Distal Interger	13	1.05E+08	1.05E+08	185627	2	67285	uc007rtj.1	254506	ENSMUSG000	Cwc27	CWC27 spliceosome-associated protein
chr13	104562085	104562387	303	304	104562279	63	29.74474	5.90194	25.87429	Adipose_R2_peak_	Distal Interger	13	1.05E+08	1.05E+08	185627	2	67285	uc007rtj.1	254566	ENSMUSG000	Cwc27	CWC27 spliceosome-associated protein
chr13	104775277	104775524	248	249	104775392	49	33.45876	8.98689	29.68925	Adipose_R1_peak_	Intron (uc007r	13	1.05E+08	1.05E+08	185627	2	67285	uc007rtj.1	41429	ENSMUSG000	Cwc27	CWC27 spliceosome-associated protein
chr13	104775301	104775513	213	214	104775377	47	17.60017	4.42645	14.02531	Adipose_R2_peak_	Intron (uc007r	13	1.05E+08	1.05E+08	185627	2	67285	uc007rtj.1	41440	ENSMUSG000	Cwc27	CWC27 spliceosome-associated protein
chr13	105407608	105407812	205	206	105407637	17	5.80341	3.23528	2.92378	Adipose_R1_peak_	Distal Interger	13	1.05E+08	1.05E+08	4441	1	15550	uc007rtu.2	-35881	ENSMUSG000	Htr1a	5-hydroxytryptamine (serotonin) receptor 1A
chr13	105713132	105713305	174	175	105713227	36	10.5378	3.41206	7.27949	Adipose_R2_peak_	Distal Interger	13	1.05E+08	1.05E+08	4441	1	15550	uc007rtu.2	269439	ENSMUSG000	Htr1a	5-hydroxytryptamine (serotonin) receptor 1A
chr13	105786137	105786394	258	259	105786301	40	24.42476	7.36925	20.81738	Adipose_R1_peak_	Distal Interger	13	1.05E+08	1.05E+08	4441	1	15550	uc007rtu.2	342444	ENSMUSG000	Htr1a	5-hydroxytryptamine (serotonin) receptor 1A
chr13	105786238	105786388	151	152	105786326	30	7.24477	2.85875	4.21917	Adipose_R2_peak_	Distal Interger	13	1.05E+08	1.05E+08	4441	1	15550	uc007rtu.2	342545	ENSMUSG000	Htr1a	5-hydroxytryptamine (serotonin) receptor 1A
chr13	105982528	105982721	194	195	105982592	17	5.80341	3.23528	2.92378	Adipose_R1_peak_	Distal Interger	13	1.05E+08	1.05E+08	4441	1	15550	uc007rtu.2	538835	ENSMUSG000	Htr1a	5-hydroxytryptamine (serotonin) receptor 1A
chr13	105998548	105998679	132	133	105998612	18	6.42934	3.41502	3.48999	Adipose_R1_peak_	Distal Interger	13	1.05E+08	1.05E+08	4441	1	15550	uc007rtu.2	554855	ENSMUSG000	Htr1a	5-hydroxytryptamine (serotonin) receptor 1A
chr13	106060910	106061054	145	146	106060974	21	8.43286	3.95423	5.34896	Adipose_R1_peak_	Distal Interger	13	1.05E+08	1.05E+08	4441	1	15550	uc007rtu.2	617217	ENSMUSG000	Htr1a	5-hydroxytryptamine (serotonin) receptor 1A
chr13	106297883	106298164	282	283	106298007	48	32.41826	8.80716	28.66523	Adipose_R1_peak_	Distal Interger	13	1.07E+08	1.07E+08	41988	2	76582	uc007rtv.1	538262	ENSMUSG000	Ipo11	importin 11
chr13	106297988	106298103	116	117	106298033	31	7.7623	2.95097	4.69388	Adipose_R2_peak_	Distal Interger	13	1.07E+08	1.07E+08	41988	2	76582	uc007rtv.1	538323	ENSMUSG000	Ipo11	importin 11
chr13	106315500	106315617	118	119	106315518	25	4.86612	2.39766	2.09018	Adipose_R2_peak_	Distal Interger	13	1.07E+08	1.07E+08	41988	2	76582	uc007rtv.1	520809	ENSMUSG000	Ipo11	importin 11
chr13	106332453	106332585	133	134	106332536	29	6.74062	2.76653	3.75989	Adipose_R2_peak_	Distal Interger	13	1.07E+08	1.07E+08	41988	2	76582	uc007rtv.1	503841	ENSMUSG000	Ipo11	importin 11
chr13	106356663	106356892	230	231	106356767	30	15.39182	5.57188	12.01394	Adipose_R1_peak_	Distal Interger	13	1.07E+08	1.07E+08	41988	2	76582	uc007rtv.1	479534	ENSMUSG000	Ipo11	importin 11
chr13	106377726	106377964	239	240	106377845	37	21.59274	6.83004	18.04766	Adipose_R1_peak_	Distal Interger	13	1.07E+08	1.07E+08	41988	2	76582	uc007rtv.1	458462	ENSMUSG000	Ipo11	importin 11
chr13	106530397	106530660	264	265	106530532	51	35.56531	9.34637	31.76235	Adipose_R1_peak_	Distal Interger	13	1.07E+08	1.07E+08	41988	2	76582	uc007rtv.1	305766	ENSMUSG000	Ipo11	importin 11
chr13	106530431	106530645	215	216	106530521	43	14.8939	4.05758	11.42017	Adipose_R2_peak_	Distal Interger	13	1.07E+08	1.07E+08	41988	2	76582	uc007rtv.1	305781	ENSMUSG000	Ipo11	importin 11
chr13	106546282	106546487	206	207	106546408	27	12.93148	5.03266	9.63827	Adipose_R1_peak_	Distal Interger	13	1.07E+08	1.07E+08	41988	2	76582	uc007rtv.1	289939	ENSMUSG000	Ipo11	importin 11
chr13	106607898	106608074	177	178	106607960	27	12.93148	5.03266	9.63827	Adipose_R1_peak_	Distal Interger	13	1.07E+08	1.07E+08	41988	2	76582	uc007rtv.1	228352	ENSMUSG000	Ipo11	importin 11
chr13	106619148	106619310	163	164	106619211	20	7.74501	3.7745	4.70467	Adipose_R1_peak_	Distal Interger	13	1.07E+08	1.07E+08	41988	2	76582	uc007rtv.1	217116	ENSMUSG000	Ipo11	importin 11
chr13	106619149	106619284	136	137	106619194	26	5.31252	2.48988	2.47999	Adipose_R2_peak_	Distal Interger	13	1.07E+08	1.07E+08	41988	2	76582	uc007rtv.1	217142	ENSMUSG000	Ipo11	importin 11
chr13	106630163	106630310	148	149	106630243	29	6.74062	2.76653	3.75989	Adipose_R2_peak_	Distal Interger	13	1.07E+08	1.07E+08	41988	2	76582	uc007rtv.1	206116	ENSMUSG000	Ipo11	importin 11
chr13	106630166	106630352	187	188	106630246	26	12.14079	4.85292	8.87838	Adipose_R1_peak_	Distal Interger	13	1.07E+08	1.07E+08	41988	2	76582	uc007rtv.1	206074	ENSMUSG000	Ipo11	importin 11
chr13	106665363	106665556	194	195	106665453	31	16.23981	5.75161	12.83549	Adipose_R1_peak_	Distal Interger	13	1.07E+08	1.07E+08	41988	2	76582	uc007rtv.1	170870	ENSMUSG000	Ipo11	importin 11
chr13	106665386	106665510	125	126	106665450	30	7.24477	2.85875	4.21917	Adipose_R2_peak_	Distal Interger	13	1.07E+08	1.07E+08	41988	2	76582	uc007rtv.1	170916	ENSMUSG000	Ipo11	importin 11
chr13	106681351	106681565	215	216	106681451	28	13.73721	5.2124	10.41463	Adipose_R1_peak_	Distal Interger	13	1.07E+08	1.07E+08	41988	2	76582	uc007rtv.1	154861	ENSMUSG000	Ipo11	importin 11
chr13	106681766	106682037	272	273	106681898	62	47.71039	11.32349	43.751	Adipose_R1_peak_	Distal Interger	13	1.07E+08	1.07E+08	41988	2	76582	uc007rtv.1	154389	ENSMUSG000	Ipo11	importin 11
chr13	106681773	106682030	258	259	106681888	63	29.74474	5.90194	25.87429	Adipose_R2_peak_	Distal Interger	13	1.07E+08	1.07E+08	41988	2	76582	uc007rtv.1	154396	ENSMUSG000	Ipo11	importin 11
chr13	106682452	106682717	266	267	106682592	35	19.76029	6.47056	16.26028	Adipose_R1_peak_	Distal Interger	13	1.07E+08	1.07E+08	41988	2	76582	uc007rtv.1	153709	ENSMUSG000	Ipo11	importin 11
chr13	106682511	106682660	150	151	106682565	31	7.7623	2.95097	4.69388	Adipose_R2_peak_	Distal Interger	13	1.07E+08	1.07E+08	41988	2	76582	uc007rtv.1	153766	ENSMUSG000	Ipo11	importin 11
chr13	106770022	106770162	141	142	106770093	22	9.13959	4.13397	6.01526	Adipose_R1_peak_	Distal Interger	13	1.07E+08	1.07E+08	41988	2	76582	uc007rtv.1	66264	ENSMUSG000	Ipo11	importin 11
chr13	106786097	106786219	123	124	106786173	28	6.25023	2.67431	3.31626	Adipose_R2_peak_	Distal Interger	13	1.07E+08	1.07E+08	41988	2	76582	uc007rtv.1	50207	ENSMUSG000	Ipo11	importin 11
chr13	106787673	106787940	268	269	106787826	24	10.60668	4.49345	7.40961	Adipose_R1_peak_	Distal Interger	13	1.07E+08	1.07E+08	41988	2	76582	uc007rtv.1	48486	ENSMUSG000	Ipo11	importin 11
chr13	106787682	106787929	248	249	106787791	43	14.8939	4.05758	11.42017	Adipose_R2_peak_	Distal Interger	13	1.07E+08	1.07E+08	41988	2	76582	uc007rtv.1	48497	ENSMUSG000	Ipo11	importin 11
chr13	108112782	108112973	192	193	108112846	26	5.31252	2.48988	2.47999	Adipose_R2_peak_	Intron (uc007r	13	1.08E+08	1.08E+08	106037	2	75597	uc007ruu.2	45652	ENSMUSG000	Ndufaf2	NADH dehydrogenase (ubiquinone) 1 alpha subcomplex, assembly factor 2
chr13	108112820	108113027	208	209	108112946	22	9.13959	4.13397	6.01526	Adipose_R1_peak_	Intron (uc007r	13	1.08E+08	1.08E+08	106037	2	75597	uc007ruu.2	45598	ENSMUSG000	Ndufaf2	NADH dehydrogenase (ubiquinone) 1 alpha subcomplex, assembly factor 2
chr13	108382339	108382469	131	132	108382397	16	5.20017	3.05554	2.38734	Adipose_R1_peak_	Intron (uc007r	13	1.08E+08	1.08E+08	73221	1	218581	uc007rvc.1	66002	ENSMUSG000	Depdc1b	DEP domain containing 1B
chr13	108394682	108394962	281	282	108394812	75	60.8111	12.84369	56.72375	Adipose_R1_peak_	Distal Interger	13	1.08E+08	1.08E+08	73221	1	218581	uc007rvc.1	78345	ENSMUSG000	Depdc1b	DEP domain containing 1B
chr13	108394691	108394936	246	247	108394824	74	39.12209	6.91633	35.10124	Adipose_R2_peak_	Distal Interger	13	1.08E+08	1.08E+08	73221	1	218581	uc007rvc.1	78354	ENSMUSG000	Depdc1b	DEP domain containing 1B

chr13	108399055	108399177	123	124	108399124	29	6.74062	2.76653	3.75989	Adipose_R2_peak_5	Distal Interger	13	1.08E+08	1.08E+08	73221	1	218581	uc007rvc.1	82718	ENSMUSG000	Depdc1b	DEP domain containing 1B
chr13	108399068	108399240	173	174	108399139	25	11.36566	4.67319	8.13482	Adipose_R1_peak_4	Distal Interger	13	1.08E+08	1.08E+08	73221	1	218581	uc007rvc.1	82731	ENSMUSG000	Depdc1b	DEP domain containing 1B
chr13	108966684	108966803	120	121	108966767	28	6.25023	2.67431	3.31626	Adipose_R2_peak_5	Intron (uc007r	13	1.09E+08	1.10E+08	673150	1	238871	uc007rvh.1	-294009	ENSMUSG000	Pde4d	phosphodiesterase 4D, cAMP specific
chr13	109181889	109182021	133	134	109181951	17	5.80341	3.23528	2.92378	Adipose_R1_peak_4	Intron (uc007r	13	1.09E+08	1.10E+08	673150	1	238871	uc007rvh.1	-78791	ENSMUSG000	Pde4d	phosphodiesterase 4D, cAMP specific
chr13	109653997	109654140	144	145	109654025	18	6.42934	3.41502	3.48999	Adipose_R1_peak_4	Intron (uc007r	13	1.10E+08	1.10E+08	87286	1	238871	uc007rvi.1	-32016	ENSMUSG000	Pde4d	phosphodiesterase 4D, cAMP specific
chr13	109811362	109811995	634	635	109811444	22	9.13959	4.13397	6.01526	Adipose_R1_peak_4	Intron (uc007r	13	1.10E+08	1.10E+08	52668	1	238871	uc007rvl.2	-91307	ENSMUSG000	Pde4d	phosphodiesterase 4D, cAMP specific
chr13	109811657	109812003	347	348	109811839	116	80.63555	10.78948	76.21979	Adipose_R2_peak_5	Intron (uc007r	13	1.10E+08	1.10E+08	52668	1	238871	uc007rvl.2	-91299	ENSMUSG000	Pde4d	phosphodiesterase 4D, cAMP specific
chr13	110148535	110148775	241	242	110148671	75	40.01057	7.00855	35.97731	Adipose_R2_peak_5	Intron (uc007r	13	1.10E+08	1.10E+08	226020	2	67295	uc007rvo.3	131431	ENSMUSG000	Rab3c	RAB3C, member RAS oncogene family
chr13	110148542	110148789	248	249	110148675	72	55.3542	11.68913	51.31976	Adipose_R1_peak_4	Intron (uc007r	13	1.10E+08	1.10E+08	226020	2	67295	uc007rvo.3	131417	ENSMUSG000	Rab3c	RAB3C, member RAS oncogene family
chr13	110312348	110312649	302	303	110312509	28	13.73721	5.2124	10.41463	Adipose_R1_peak_4	Distal Interger	13	1.10E+08	1.10E+08	226810	2	67295	uc007rvp.3	-31352	ENSMUSG000	Rab3c	RAB3C, member RAS oncogene family
chr13	110744029	110744541	513	514	110744136	30	15.39182	5.57188	12.01394	Adipose_R1_peak_4	Distal Interger	13	1.11E+08	1.11E+08	4782	1	75139	uc056yve.1	-158958	NA	Gm38397	predicted gene, 38397
chr13	110748823	110748979	157	158	110748868	28	6.25023	2.67431	3.31626	Adipose_R2_peak_5	Distal Interger	13	1.11E+08	1.11E+08	4782	1	75139	uc056yve.1	-154520	NA	Gm38397	predicted gene, 38397
chr13	111070813	111070956	144	145	111070888	22	9.13959	4.13397	6.01526	Adipose_R1_peak_4	Distal Interger	13	1.11E+08	1.11E+08	17963	2	73129	uc029sde.1	158475	NA	3110015C05Ri	RIKEN cDNA 3110015C05 gene
chr13	111188172	111188431	260	261	111188308	31	16.23981	5.75161	12.83549	Adipose_R1_peak_4	Distal Interger	13	1.11E+08	1.11E+08	17963	2	73129	uc029sde.1	41000	NA	3110015C05Ri	RIKEN cDNA 3110015C05 gene
chr13	111188278	111188433	156	157	111188328	38	11.72962	3.59649	8.4047	Adipose_R2_peak_5	Distal Interger	13	1.11E+08	1.11E+08	17963	2	73129	uc029sde.1	40998	NA	3110015C05Ri	RIKEN cDNA 3110015C05 gene
chr13	111220704	111220869	166	167	111220767	17	5.80341	3.23528	2.92378	Adipose_R1_peak_4	Intron (uc029s	13	1.11E+08	1.11E+08	17963	2	73129	uc029sde.1	8562	NA	3110015C05Ri	RIKEN cDNA 3110015C05 gene
chr13	111221575	111221912	338	339	111221724	106	103.5054	19.23195	99.15611	Adipose_R1_peak_4	Intron (uc029s	13	1.11E+08	1.11E+08	17963	2	73129	uc029sde.1	7519	NA	3110015C05Ri	RIKEN cDNA 3110015C05 gene
chr13	111221579	111221865	287	288	111221713	75	40.01057	7.00855	35.97731	Adipose_R2_peak_5	Intron (uc029s	13	1.11E+08	1.11E+08	17963	2	73129	uc029sde.1	7566	NA	3110015C05Ri	RIKEN cDNA 3110015C05 gene
chr13	111222900	111223124	225	226	111223020	32	17.10103	5.93135	13.67137	Adipose_R1_peak_4	Intron (uc029s	13	1.11E+08	1.11E+08	17963	2	73129	uc029sde.1	6307	NA	3110015C05Ri	RIKEN cDNA 3110015C05 gene
chr13	111875762	111875856	95	96	111875840	31	7.7623	2.95097	4.69388	Adipose_R2_peak_5	Distal Interger	13	1.12E+08	1.12E+08	4002	1	1.03E+08	uc007rwb.1	7826	ENSMUSG000	Gm15326	predicted gene 15326
chr13	111892957	111893145	189	190	111893101	17	5.80341	3.23528	2.92378	Adipose_R1_peak_4	Distal Interger	13	1.12E+08	1.12E+08	4002	1	1.03E+08	uc007rwb.1	25021	ENSMUSG000	Gm15326	predicted gene 15326
chr13	112566489	112566629	141	142	112566553	23	9.86443	4.31371	6.70247	Adipose_R1_peak_4	Promoter (<=1	13	1.13E+08	1.13E+08	43095	2	218624	uc011zep.2	-588	ENSMUSG000	Il31ra	interleukin 31 receptor A
chr13	112578197	112578343	147	148	112578280	34	9.39139	3.22762	6.20474	Adipose_R2_peak_5	Promoter (2-3	13	1.13E+08	1.13E+08	57856	2	218624	uc007rwi.2	2319	ENSMUSG000	Il31ra	interleukin 31 receptor A
chr13	112578201	112578336	136	137	112578269	40	11.39761	3.39856	8.16652	Adipose_R1_peak_4	Promoter (2-3	13	1.13E+08	1.13E+08	57856	2	218624	uc007rwi.2	2326	ENSMUSG000	Il31ra	interleukin 31 receptor A
chr13	113281360	113281540	181	182	113281439	27	12.93148	5.03266	9.63827	Adipose_R1_peak_4	Distal Interger	13	1.13E+08	1.13E+08	8446	1	71690	uc007rxe.1	71701	ENSMUSG000	Esm1	endothelial cell-specific molecule 1
chr13	113302708	113302941	234	235	113302824	46	28.09865	7.62595	24.43024	Adipose_R1_peak_4	Distal Interger	13	1.13E+08	1.13E+08	8446	1	71690	uc007rxe.1	93049	ENSMUSG000	Esm1	endothelial cell-specific molecule 1
chr13	113302724	113302933	210	211	113302806	49	19.00719	4.61089	15.38598	Adipose_R2_peak_5	Distal Interger	13	1.13E+08	1.13E+08	8446	1	71690	uc007rxe.1	93065	ENSMUSG000	Esm1	endothelial cell-specific molecule 1
chr13	113390900	113390997	98	99	113390949	30	7.24477	2.85875	4.21917	Adipose_R2_peak_5	Distal Interger	13	1.13E+08	1.14E+08	56557	2	70917	uc029sdg.1	111847	NA	4921509O07R	RIKEN cDNA 4921509O07 gene
chr13	113507589	113507759	171	172	113507727	27	12.93148	5.03266	9.63827	Adipose_R1_peak_4	Distal Interger	13	1.13E+08	1.14E+08	56557	2	70917	uc029sdg.1	-4745	NA	4921509O07R	RIKEN cDNA 4921509O07 gene
chr13	114842164	114842452	289	290	114842325	43	27.35048	7.90847	23.68561	Adipose_R1_peak_4	Intron (uc007r	13	1.15E+08	1.15E+08	11184	1	17434	uc007rxu.2	23927	ENSMUSG000	Mocs2	molybdenum cofactor synthesis 2
chr13	114842219	114842432	214	215	114842315	41	13.59774	3.87315	10.1803	Adipose_R2_peak_5	Intron (uc007r	13	1.15E+08	1.15E+08	11184	1	17434	uc007rxu.2	23982	ENSMUSG000	Mocs2	molybdenum cofactor synthesis 2
chr13	115081388	115081541	154	155	115081464	20	7.74501	3.7745	4.70467	Adipose_R1_peak_4	Intron (uc007r	13	1.15E+08	1.15E+08	1804	2	105083	uc007ryb.2	8617	ENSMUSG000	Pelo	pelota homolog (Drosophila)
chr13	115204976	115205232	257	258	115205084	39	23.47001	7.18952	19.88325	Adipose_R1_peak_4	Distal Interger	13	1.15E+08	1.15E+08	13610	2	105083	uc007ryd.2	-103012	ENSMUSG000	Pelo	pelota homolog (Drosophila)
chr13	115204981	115205234	254	255	115205092	46	16.90986	4.33423	13.35898	Adipose_R2_peak_5	Distal Interger	13	1.15E+08	1.15E+08	13610	2	105083	uc007ryd.2	-103017	ENSMUSG000	Pelo	pelota homolog (Drosophila)
chr13	115502004	115502306	303	304	115502160	82	71.83084	14.91825	67.65268	Adipose_R1_peak_4	Distal Interger	13	1.15E+08	1.15E+08	13610	2	105083	uc007ryd.2	-400040	ENSMUSG000	Pelo	pelota homolog (Drosophila)
chr13	115502009	115502304	296	297	115502173	86	50.14197	8.02294	45.98602	Adipose_R2_peak_5	Distal Interger	13	1.15E+08	1.15E+08	13610	2	105083	uc007ryd.2	-400045	ENSMUSG000	Pelo	pelota homolog (Drosophila)
chr13	115889118	115889328	211	212	115889242	42	14.2409	3.96536	10.79479	Adipose_R2_peak_5	Distal Interger	13	1.16E+08	1.16E+08	11419	2	16392	uc007rye.2	420360	ENSMUSG000	Isl1	ISL1 transcription factor, LIM/homeodomain
chr13	115889130	115889324	195	196	115889235	29	14.55748	5.39214	11.20652	Adipose_R1_peak_4	Distal Interger	13	1.16E+08	1.16E+08	11419	2	16392	uc007rye.2	420364	ENSMUSG000	Isl1	ISL1 transcription factor, LIM/homeodomain
chr13	116466431	116466587	157	158	116466488	19	7.07687	3.59476	4.08458	Adipose_R1_peak_4	Distal Interger	13	1.16E+08	1.16E+08	11419	2	16392	uc007rye.2	-156743	ENSMUSG000	Isl1	ISL1 transcription factor, LIM/homeodomain
chr13	116466432	116466623	192	193	116466474	42	14.2409	3.96536	10.79479	Adipose_R2_peak_5	Distal Interger	13	1.16E+08	1.16E+08	11419	2	16392	uc007rye.2	-156744	ENSMUSG000	Isl1	ISL1 transcription factor, LIM/homeodomain
chr13	116582828	116582990	163	164	116582904	25	11.36566	4.67319	8.13482	Adipose_R1_peak_4	Distal Interger	13	1.16E+08	1.16E+08	11419	2	16392	uc007rye.2	-273140	ENSMUSG000	Isl1	ISL1 transcription factor, LIM/homeodomain
chr13	116728187	116728427	241	242	116728299	26	12.14079	4.85292	8.87838	Adipose_R1_peak_4	Distal Interger	13	1.17E+08	1.17E+08	24511	2	52552	uc007ryh.1	150907	ENSMUSG000	Parp8	poly (ADP-ribose) polymerase family, member 8
chr13	116728195	116728408	214	215	116728309	38	11.72962	3.59649	8.4047	Adipose_R2_peak_5	Distal Interger	13	1.17E+08	1.17E+08	24511	2	52552	uc007ryh.1	150926	ENSMUSG000	Parp8	poly (ADP-ribose) polymerase family, member 8
chr13	116842005	116842183	179	180	116842085	20	7.74501	3.7745	4.70467	Adipose_R1_peak_4	Distal Interger	13	1.17E+08	1.17E+08	24511	2	52552	uc007ryh.1	37151	ENSMUSG000	Parp8	poly (ADP-ribose) polymerase family, member 8
chr13	116842389	116842545	157	158	116842469	20	7.74501	3.7745	4.70467	Adipose_R1_peak_4	Distal Interger	13	1.17E+08	1.17E+08	24511	2	52552	uc007ryh.1	36789	ENSMUSG000	Parp8	poly (ADP-ribose) polymerase family, member 8
chr13	116842737	116842976	240	241	116842845	39	23.47001	7.18952	19.88325	Adipose_R1_peak_4	Distal Interger	13	1.17E+08	1.17E+08	24511	2	52552	uc007ryh.1	36358	ENSMUSG000	Parp8	poly (ADP-ribose) polymerase family, member 8
chr13	116842777	116842922	146	147	116842829	31	7.7623	2.95097	4.69388	Adipose_R2_peak_5	Distal Interger	13	1.17E+08	1.17E+08	24511	2	52552	uc007ryh.1	36412	ENSMUSG000	Parp8	poly (ADP-ribose) polymerase family, member 8
chr13	117038885	117039049	165	166	117038996	29	6.74062	2.76653	3.75989	Adipose_R2_peak_5	Distal Interger	13	1.17E+08	1.17E+08	170693	2	52552	uc007ryk.1	-13369	ENSMUSG000	Parp8	poly (ADP-ribose) polymerase family, member 8
chr13	117038902	117039119	218	219	117039017	27	12.93148	5.03266	9.63827	Adipose_R1_peak_4	Distal Interger	13	1.17E+08	1.17E+08	170693	2	52552	uc007ryk.1	-13386	ENSMUSG000	Parp8	poly (ADP-ribose) polymerase family, member 8
chr13	117376847	117377110	264	265	117376966	95	76.92828	13.43291	72.71765	Adipose_R1_peak_4	Distal Interger	13	1.17E+08	1.17E+08	53843	1	13723	uc007rym.2	156274	ENSMUSG000	Emb	embigin
chr13	117376884	117377105	222	223	117376968	69	34.76717	6.45524	30.8116	Adipose_R2_peak_5	Distal Interger	13	1.17E+08	1.17E+08	53843	1	13723	uc007rym.2	156311	ENSMUSG000	Emb	embigin
chr13	117450789	117450976	188	189	117450906	36	10.5378	3.41206	7.27949	Adipose_R2_peak_5	Distal Interger	13	1.18E+08	1.18E+08	58529	1	15165	uc007ryn.2	-151344	ENSMUSG000	Hcn1	hyperpolarization-activated, cyclic nucleotide-gated K+ 1
chr13	117450794	117450961	168	169	117450892	20	7.74501	3.7745	4.70467	Adipose_R1_peak_4	Distal Interger	13	1.18E+08	1.18E+08	58529	1	15165	uc007ryn.2	-151359	ENSMUSG000	Hcn1	hyperpolarization-activated, cyclic nucleotide-gated K+ 1
chr13	117610074	117610274	201	202	117610180	18	6.42934	3.41502	3.48999	Adipose_R1_peak_4	Intron (uc007r	13	1.18E+08	1.18E+08	58529	1	15165	uc007ryn.2	7754	ENSMUSG000	Hcn1	hyperpolarization-activated, cyclic nucleotide-gated K+ 1
chr13	118058580	118058730	151	152	118058659	27	5.77405	2.5821	2.88921	Adipose_R2_peak_5	Distal Interger	13	1.18E+08	1.18E+08	7143	2	59054	uc007ryr.1	328522	ENSMUSG000	Mrps30	mitochondrial ribosomal protein S30
chr13	118058600	118058793	194	195	118058697	29	13.73288	5.06988	10.41463	Adipose_R1_peak_4	Distal Interger	13	1.18E+08	1.18E+08	7143	2	59054	uc007ryr.1	328459	ENSMUSG000	Mrps30	mitochondrial ribosomal protein S30
chr13	118073676	118073891	216	217	118073809	25	11.36566	4.67319	8.13482	Adipose_R1_peak_4	Distal Interger	13	1.18E+08	1.18E+08	7143	2	59054	uc007ryr.1	313361	ENSMUSG000	Mrps30	mitochondrial ribosomal protein S30
chr13	118106613	118106780	168	169	118106675	19	7.07687	3.59476	4.08458	Adipose_R1_peak_4	Distal Interger	13	1.18E+08	1.18E+08	7143	2	59054	uc007ryr.1	280472	ENSMUSG000	Mrps30	mitochondrial ribosomal protein S30
chr13	118108720	118108894	175	176	118108778	21	8.43286	3.95423	5.34896	Adipose_R1_peak_4	Distal Interger	13	1.18E+08	1.18E+08	7143	2	59054	uc007ryr.1	278358	ENSMUSG000	Mrps30	mitochondrial ribosomal protein S30
chr13	118191986	118192294	309	310	118192164	40	12.96465	3.78093	9.57654	Adipose_R2_peak_5	Distal Interger	13	1.18E+08	1.18E+08	7143	2	59054	uc007ryr.1	194958	ENSMUSG000	Mrps30	mitochondrial ribosomal protein S30
chr13	118191987	118192393	407	408	118192189	38	22.52591	7.00978	18.96	Adipose_R1_peak_4	Distal Interger	13	1.18E+08	1.18E+08	7143	2	59054	uc007ryr.1	194859	ENSMUSG000	Mrps30	mitochondrial ribosomal protein S30
chr13	118345463	118345677	215	216	118345578	37	21.59274	6.83004	18.04766	Adipose_R1_peak_4	Distal Interger	13	1.18E+08	1.18E+08	7143	2	59054	uc007ryr.1	41575	ENSMUSG000	Mrps30	mitochondrial ribosomal protein S30
chr13	118345473	118345645	173	174	118345571	40	12.96465	3.78093	9.57654	Adipose_R2_peak_5	Distal Interger	13	1.18E+08	1.18E+08	7143	2	59054	uc007ryr.1	41607	ENSMUSG000	Mrps30	mitochondrial ribosomal protein S30
chr13	118408864	118409142	279	280	118408954	137	115.4395	15.2797	111.0414	Adipose_R1_peak_4	Distal Interger	13	1.18E+08	1.18E+08	7143	2	59054	uc007ryr.1	-21612	ENSMUSG000	Mrps30	mitochondrial ribosomal protein S30
chr13	119070464	119070655	192	193	119070565	20	7.74501	3.7745	4.70467	Adipose_R1_peak_4	Distal Interger	13	1.19E+08	1.19E+08	21044	2	18115	uc007ryy.1	304119	ENSMUSG000	Nnt	nicotinamide nucleotide transhydrogenase
chr13	119099575	119099737	163	164	119099706	24	10.60668	4.49345	7.40961	Adipose_R1_peak_4	Distal Interger	13	1.19E+08	1.19E+08	21044	2	18115	uc007ryy.1	275037	ENSMUSG000	Nnt	nicotinamide nucleotide transhydrogenase
chr13	119193229	119193401	173	174	119193301	37	11.12817	3.50427	7.83613	Adipose_R2_peak_5	Distal Interger	13	1.19E+08	1.19E+08	21044	2	18115	uc007ryy.1	181373	ENSMUSG000	Nnt	nicotinamide nucleotide transhydrogenase
chr13	119193231	119193397	167	168	119193313	23	9.86443	4.31371	6.70247	Adipose_R1_peak_4	Distal Interger	13	1.19E+08	1.19E+08	21044	2	18115	uc007ryy.1	181377	ENSMUSG000	Nnt	nicotinamide nucleotide transhydrogenase
chr13	119213028	119213235	208	209	119213151	44	28.34544	8.08821	24.66239	Adipose_R1_peak_4	Distal Interger	13	1.19E+08	1.19E+08	21044	2	18115	uc007ryy.1	161539	ENSMUSG000	Nnt	nicotinamide nucleotide transhydrogenase
chr13	119213042	119213237	196	197	119213163	47	17.60017	4.42645	14.02531	Adipose_R2_peak_5	Distal Interger	13	1.19E+08	1.19E+08	21044	2	18115	uc007ryy.1	161537	ENSMUSG000	Nnt	nicotinamide nucleotide transhydrogenase
chr13	119214447	119214717	271	272	119214585	42	26.36523	7.72873	22.71906	Adipose_R1_peak_4	Distal Interger	13	1.19E+08	1.19E+08	21044	2	18115	uc007ryy.1	160057	ENSMUSG000	Nnt	nicotinamide nucleotide transhydrogenase
chr13	119214493	119214638	146	147	119214577	34	9.39139	3.22762	6.20474	Adipose_R2_peak_5	Distal Interger	13	1.19E+08	1.19E+08	21044	2	18115	uc007ryy.1	160136	ENSMUSG000	Nnt	nicotinamide nucleotide transhydrogenase
chr13	119225851	119226061	211	212	119225940	28	13.73721	5.2124	10.41463	Adipose_R1_peak_4	Distal Interger	13	1.19E+08	1.19E+08	21044	2	18115	uc007ryy.1	148713	ENSMUSG000	Nnt	nicotinamide nucleotide transhydrogenase
chr13	119225891	119226010	120	121	119225943	28	6.25023	2.67431	3.31626	Adipose_R2_peak_5	Distal Interger	13	1.19E+08	1.19E+08	21044	2	18115	uc007ryy.1	148764	ENSMUSG000	Nnt	nicotinamide nucleotide transhydrogenase
chr13	119488745	119489054	310	311	119488957	72	15.15271	2.92049	11.6734	Adipose_R2_peak_5	Promoter (<=1	13	1.19E+08	1.20E+08	121600	1	633640	uc029sdl.1	0	ENSMUSG000	Tmem267	transmembrane protein 267
chr13	119488804	119489023	220	221	119488882	39	5.30126	2.11102	2.48665	Adipose_R1_peak_4	Promoter (<=1	13	1.19E+08	1.20E+08	121600	1	633640	uc029sdl.1	0	ENSMUSG000	Tmem267	transmembrane protein 267
chr13	119490138	119490299	162	163	119490219	61	12.02157	2.75217	8.68784	Adipose_R2_peak_5	Promoter (1-2	13	1.19E+08	1.20E+08	121600	1	633640	uc029sdl.1	1278	ENSMUSG000	Tmem267	transmembrane protein 267
chr13	119490943	119491173	231	232	119491072	75	13.48644	2.64826	10.08477	Adipose_R2_peak_5	Promoter (1-2	13	1.19E+08	1.19E+08	2837	1	633640	uc033gnh.1	-1461	ENSMUSG000	Tmem267	transmembrane protein 267
chr13	119491769	119492092	324	325	119492035	70	11.81778	2.53459	8.49023	Adipose_R2_peak_5	Promoter (<=1	13	1.19E+08	1.19E+08	2837	1	633640	uc033gnh.1	-542	ENSMUSG000	Tmem267	transmembrane protein 267
chr13	119492269	119492442	174	175	119492386	65	9.22629	2.28561	6.063	Adipose_R2_peak_5	Promoter (<=1	13	1.19E+08	1.19E+08	2837	1	633640	uc033gnh.1	-192	ENSMUSG000	Tmem267	transmembrane protein 267
chr13	119492668	119493343	676	677	119492815	82	12.73756	2.44776	9.3695	Adipose_R2_peak_5	Promoter (<=1	13	1.19E+08	1.19E+08	2837	1	633640	uc033gnh.1	34	ENSMUSG000	Tmem267	transmembrane protein 267
chr13	119493714	119493869	156	157	119493789	78	11.80348	2.40301	8.47616	Adipose_R2_peak_5	Promoter (1-2	13	1.19E+08	1.19E+08	2837	1	633640	uc033gnh.1	1080	ENSMUSG000	Tmem267	transmembrane protein 267
chr13	119494125	119494534	410	411	119494238	77	11.62313	2.39724	8.31627	Adipose_R2_peak_5	Promoter (1-2	13	1.19E+08	1.19E+08	2837	1	633640	uc033gnh.1	1491	ENSMUSG000	Tmem267	transmembrane protein 267
chr13	119494989	119495415	427	428	119495125	65	15.94441	3.22409	12.43627	Adipose_R2_peak_5	Promoter (2-3	13	1.19E+08	1.19E+08	2837	1	633640	uc033gnh.1	2355	ENSMUSG000	Tmem267	transmembrane protein 267
chr13	119595670	119596275	606	607	119595772	77	19.70129	3.38372	16.22036	Adipose_R1_peak_4	Intron (uc029s	13	1.20E+08	1.20E+08	3812	1	633640	uc029sdm.1	-10373	ENSMUSG000	Tmem267	transmembrane protein 267
chr13	119595688	119595829	142	143	119595767	79	11.93001	2.40268	8.59908	Adipose_R2_peak_5	Intron (uc029s	13	1.20E+08	1.20E+08	3812	1	633640	uc029sdm.1	-10819	ENSMUSG000	Tmem267	transmembrane protein 267
chr13	119596019	119596136	118	119	119596073	69	7.89917	2.06518	4.82588	Adipose_R2_peak_5	Intron (uc029s	13	1.20E+08	1.20E+08	3812	1	633640	uc029sdm.1	-10512	ENSMUSG000	Tmem267	transmembrane protein 267
chr13	119596636	119597190	555	556	119596823	510	94.64237	2.92681	90.15784	Adipose_R2_peak_5	Intron (uc029s	13	1.20E+08	1.20E+08	3812	1	633640	uc029sdm.1	-9458	ENSMUSG000	Tmem267	transmembrane protein 267
chr13	119596645	119597197	553	554	119596841	305	35.7059	2.24492	31.90258	Adipose_R1_peak_4	Intron (uc029s	13	1.20E+08	1.20E+08	3812	1	633640	uc029sdm.1	-9451	ENSMUSG000	Tmem267	transmembrane protein 267
chr13	119597900	119598032	133	134	119597948	91	6.98913	1.78635	3.99892	Adipose_R2_peak_5	Intron (uc029s	13	1.20E+08	1.20E+08	3812	1	633640	uc029sdm.1	-8616	ENSMUSG000	Tmem267	transmembrane protein 267
chr13	119598222	119599325	1104	1105	119598332	274	127.6887	6.57681	123.2457	Adipose_R1_peak_4	Intron (uc029s	13	1.20E+08	1.20E+08	3812	1	633640	uc029sdm.1	-7323	ENSMUSG000	Tmem267	transmembrane protein 267
chr13	119598228	119598657	430	431	119598311	327	121.3631	5.11902	116.7813	Adipose_R2_peak_5	Intron (uc029s	13	1.20E+08	1.20E+08	3812	1	633640	uc029sdm.1	-7991	ENSMUSG000	Tmem267	transmembrane protein 267
chr13	119598896	119599344	449	450	119599055	388	90.24715	3.40451	85.78181	Adipose_R2_peak_5	Intron (uc029s	13	1.20E+08	1.20E+08	3812	1	633640	uc029sdm.1	-7304	ENSMUSG000	Tmem267	transmembrane protein 267
chr13	119599743	119600598	856	857	119599902	308	67.244	3.23303	63.10227	Adipose_R1_peak_4	Intron (uc029s	13	1.20E+08	1.20E+08	3812	1	633640	uc029sdm.1	-6050	ENSMUSG000	Tmem267	transmembrane protein 267

chr13	119599768	119600584	817	818	119599957	387	93.10468	3.49214	88.62606	Adipose_R2_peak_5	Intron (uc029s	13	1.20E+08	1.20E+08	3812	1	633640	uc029sdm.1	-6064	ENSMUSG000	Tmem267	transmembrane protein 267
chr13	119601036	119601578	543	544	119601305	145	22.17148	2.51193	18.62398	Adipose_R1_peak_4	Intron (uc029s	13	1.20E+08	1.20E+08	3812	1	633640	uc029sdm.1	-5070	ENSMUSG000	Tmem267	transmembrane protein 267
chr13	119601167	119601561	395	396	119601307	217	27.15744	2.29651	23.34032	Adipose_R2_peak_5	Intron (uc029s	13	1.20E+08	1.20E+08	3812	1	633640	uc029sdm.1	-5087	ENSMUSG000	Tmem267	transmembrane protein 267
chr13	119601855	119603029	1175	1176	119602149	513	101.586	3.05434	97.07259	Adipose_R2_peak_5	Intron (uc029s	13	1.20E+08	1.20E+08	3812	1	633640	uc029sdm.1	-3619	ENSMUSG000	Tmem267	transmembrane protein 267
chr13	119601862	119603040	1179	1180	119602148	391	66.59852	2.76129	62.46499	Adipose_R1_peak_4	Intron (uc029s	13	1.20E+08	1.20E+08	3812	1	633640	uc029sdm.1	-3608	ENSMUSG000	Tmem267	transmembrane protein 267
chr13	119609478	119609853	376	377	119609679	75	9.77742	2.21446	6.57741	Adipose_R2_peak_5	Promoter (2-3	13	1.20E+08	1.20E+08	3812	1	633640	uc029sdm.1	2830	ENSMUSG000	Tmem267	transmembrane protein 267
chr13	119609529	119609709	181	182	119609584	39	5.30126	2.11102	2.48665	Adipose_R1_peak_4	Promoter (2-3	13	1.20E+08	1.20E+08	3812	1	633640	uc029sdm.1	2881	ENSMUSG000	Tmem267	transmembrane protein 267
chr13	119610213	119610750	538	539	119610273	66	7.55169	2.05674	4.51482	Adipose_R2_peak_5	3' UTR	13	1.20E+08	1.20E+08	3812	1	633640	uc029sdm.1	3565	ENSMUSG000	Tmem267	transmembrane protein 267
chr13	119610230	119610467	238	239	119610372	46	7.47118	2.37223	4.474	Adipose_R1_peak_4	3' UTR	13	1.20E+08	1.20E+08	3812	1	633640	uc029sdm.1	3582	ENSMUSG000	Tmem267	transmembrane protein 267
chr13	119611360	119611532	173	174	119611491	48	5.04991	1.91824	2.26744	Adipose_R2_peak_5	Distal Intergen	13	1.20E+08	1.20E+08	3812	1	633640	uc029sdm.1	4712	ENSMUSG000	Tmem267	transmembrane protein 267
chr13	119612144	119612253	110	111	119612196	59	9.95104	2.4925	6.73562	Adipose_R2_peak_5	Distal Intergen	13	1.20E+08	1.20E+08	3812	1	633640	uc029sdm.1	5496	ENSMUSG000	Tmem267	transmembrane protein 267
chr13	119612859	119613394	536	537	119613020	91	19.40928	3.0048	15.78139	Adipose_R2_peak_5	Distal Intergen	13	1.20E+08	1.20E+08	3812	1	633640	uc029sdm.1	6211	ENSMUSG000	Tmem267	transmembrane protein 267
chr13	119613555	119614808	1254	1255	119613671	90	16.92351	2.76255	13.37243	Adipose_R2_peak_5	Distal Intergen	13	1.20E+08	1.20E+08	3812	1	633640	uc029sdm.1	6907	ENSMUSG000	Tmem267	transmembrane protein 267
chr13	119614451	119614770	320	321	119614515	47	6.96476	2.26098	4.01801	Adipose_R1_peak_4	Distal Intergen	13	1.20E+08	1.20E+08	3812	1	633640	uc029sdm.1	7803	ENSMUSG000	Tmem267	transmembrane protein 267
chr13	119615156	119617598	2443	2444	119615403	91	17.38539	2.79291	13.82575	Adipose_R2_peak_5	Distal Intergen	13	1.20E+08	1.20E+08	30540	1	56838	uc007sal.1	-6221	ENSMUSG000	Ccl28	chemokine (C-C motif) ligand 28
chr13	119615378	119615630	253	254	119615550	47	7.69114	2.38859	4.69144	Adipose_R1_peak_4	Distal Intergen	13	1.20E+08	1.20E+08	30540	1	56838	uc007sal.1	-8189	ENSMUSG000	Ccl28	chemokine (C-C motif) ligand 28
chr13	119616782	119617160	379	380	119616848	57	8.79052	2.36036	5.7031	Adipose_R1_peak_4	Distal Intergen	13	1.20E+08	1.20E+08	30540	1	56838	uc007sal.1	-6659	ENSMUSG000	Ccl28	chemokine (C-C motif) ligand 28
chr13	119636490	119636707	218	219	119636601	32	8.29282	3.04319	5.18352	Adipose_R2_peak_5	Intron (uc007s	13	1.20E+08	1.20E+08	30540	1	56838	uc007sal.1	12671	ENSMUSG000	Ccl28	chemokine (C-C motif) ligand 28
chr13	119934240	119934616	377	378	119934299	19	7.07687	3.59476	4.08458	Adipose_R1_peak_4	Distal Intergen	13	1.20E+08	1.20E+08	1450	2	333467	uc029sdq.1	16190	ENSMUSG000	B020031M17R	RIKEN cDNA B020031M17 gene
chr13	119934469	119934589	121	122	119934530	29	6.74062	2.76653	3.75989	Adipose_R2_peak_5	Distal Intergen	13	1.20E+08	1.20E+08	1450	2	333467	uc029sdq.1	16217	ENSMUSG000	B020031M17R	RIKEN cDNA B020031M17 gene
chr13	119941880	119942304	425	426	119942082	26	5.31252	2.48988	2.47999	Adipose_R2_peak_5	Distal Intergen	13	1.20E+08	1.20E+08	1450	2	333467	uc029sdq.1	8502	ENSMUSG000	B020031M17R	RIKEN cDNA B020031M17 gene
chr13	119962769	119962900	132	133	119962897	27	5.77405	2.5821	2.88921	Adipose_R2_peak_5	Distal Intergen	13	1.20E+08	1.20E+08	1450	2	333467	uc029sdq.1	-11963	ENSMUSG000	B020031M17R	RIKEN cDNA B020031M17 gene
chr13	119965847	119965940	94	95	119965892	28	6.25023	2.67431	3.31626	Adipose_R2_peak_5	Distal Intergen	13	1.20E+08	1.20E+08	1450	2	333467	uc029sdq.1	-15041	ENSMUSG000	B020031M17R	RIKEN cDNA B020031M17 gene
chr13	119969359	119969527	169	170	119969412	26	5.31252	2.48988	2.47999	Adipose_R2_peak_5	Distal Intergen	13	1.20E+08	1.20E+08	1450	2	333467	uc029sdq.1	-18553	ENSMUSG000	B020031M17R	RIKEN cDNA B020031M17 gene
chr13	119969943	119970072	130	131	119969977	27	5.77405	2.5821	2.88921	Adipose_R2_peak_5	Distal Intergen	13	1.20E+08	1.20E+08	1450	2	333467	uc029sdq.1	-19137	ENSMUSG000	B020031M17R	RIKEN cDNA B020031M17 gene
chr13	119976926	119977040	115	116	119976979	27	5.77405	2.5821	2.88921	Adipose_R2_peak_5	Distal Intergen	13	1.20E+08	1.20E+08	1450	2	333467	uc029sdq.1	-26120	ENSMUSG000	B020031M17R	RIKEN cDNA B020031M17 gene
chr13	119991645	119991891	247	248	119991790	33	8.83596	3.1354	5.68652	Adipose_R2_peak_5	Distal Intergen	13	1.20E+08	1.20E+08	1450	2	333467	uc029sdq.1	-40839	ENSMUSG000	B020031M17R	RIKEN cDNA B020031M17 gene
chr13	119992582	119992840	259	260	119992677	27	5.77405	2.5821	2.88921	Adipose_R2_peak_5	Distal Intergen	13	1.20E+08	1.20E+08	1450	2	333467	uc029sdq.1	-41776	ENSMUSG000	B020031M17R	RIKEN cDNA B020031M17 gene
chr13	120000765	120000964	200	201	120000918	27	5.77405	2.5821	2.88921	Adipose_R2_peak_5	Distal Intergen	13	1.20E+08	1.20E+08	1450	2	333467	uc029sdq.1	-49959	ENSMUSG000	B020031M17R	RIKEN cDNA B020031M17 gene
chr13	120028167	120028349	183	184	120028276	19	7.03841	3.57815	4.08458	Adipose_R1_peak_4	Distal Intergen	13	1.20E+08	1.20E+08	17600	2	1.01E+08	uc029sds.2	23855	ENSMUSG000	Gm21188	predicted gene, 21188
chr13	120028179	120028337	159	160	120028265	33	8.83596	3.1354	5.68652	Adipose_R2_peak_5	Distal Intergen	13	1.20E+08	1.20E+08	17600	2	1.01E+08	uc029sds.2	23867	ENSMUSG000	Gm21188	predicted gene, 21188
chr13	120296267	120296600	334	335	120296441	106	103.5054	19.23195	99.15611	Adipose_R1_peak_4	Distal Intergen	13	1.20E+08	1.20E+08	8450	1	625360	uc009shv.3	-3792	ENSMUSG000	BC147527	cDNA sequence BC147527
chr13	120296303	120296588	286	287	120296436	76	40.9047	7.10077	36.85923	Adipose_R2_peak_5	Distal Intergen	13	1.20E+08	1.20E+08	8450	1	625360	uc009shv.3	-3804	ENSMUSG000	BC147527	cDNA sequence BC147527
chr14	3054047	3054251	205	206	3054113	27	5.77405	2.5821	2.88921	Adipose_R2_peak_5	Intron (uc007s	14	3049285	3076838	27554	1	1.00E+08	uc007sam.3	4762	ENSMUSG000	Gm2897	predicted gene 2897
chr14	3061953	3062086	134	135	3062004	26	5.31252	2.48988	2.47999	Adipose_R2_peak_5	Intron (uc007s	14	3066066	3071340	5275	1	1.00E+08	uc007san.1	-3980	ENSMUSG000	Gm2897	predicted gene 2897
chr14	3106245	3106403	159	160	3106305	24	10.60668	4.49345	7.40961	Adipose_R1_peak_4	Intron (uc029s	14	3070096	3136967	66872	1	1.00E+08	uc029sdz.3	36149	ENSMUSG000	Gm2897	predicted gene 2897
chr14	3123446	3123589	144	145	3123530	43	10.74042	3.10659	7.47593	Adipose_R2_peak_5	Intron (uc029s	14	3070096	3136967	66872	1	1.00E+08	uc029sdz.3	53350	ENSMUSG000	Gm2897	predicted gene 2897
chr14	3123696	3123850	155	156	3123743	40	11.18614	3.34864	7.89193	Adipose_R2_peak_5	Intron (uc029s	14	3070096	3136967	66872	1	1.00E+08	uc029sdz.3	53600	ENSMUSG000	Gm2897	predicted gene 2897
chr14	3126365	3126515	151	152	3126388	25	4.86612	2.39766	2.09018	Adipose_R2_peak_5	Intron (uc029s	14	3070096	3136967	66872	1	1.00E+08	uc029sdz.3	56269	ENSMUSG000	Gm2897	predicted gene 2897
chr14	3147163	3147323	161	162	3147235	31	7.7623	2.95097	4.69388	Adipose_R2_peak_5	Distal Intergen	14	3188284	3196683	8400	1	544990	uc033gnp.1	-40961	ENSMUSG000	Gm5795	predicted gene 5795
chr14	3175506	3175738	233	234	3175614	28	13.73721	5.2124	10.41463	Adipose_R1_peak_4	Distal Intergen	14	3188284	3196683	8400	1	544990	uc033gnp.1	-12546	ENSMUSG000	Gm5795	predicted gene 5795
chr14	3179910	3180005	96	97	3179916	26	4.78485	2.33601	2.05846	Adipose_R2_peak_5	Distal Intergen	14	3188284	3196683	8400	1	544990	uc033gnp.1	-8279	ENSMUSG000	Gm5795	predicted gene 5795
chr14	3182110	3182206	97	98	3182173	26	5.31252	2.48988	2.47999	Adipose_R2_peak_5	Distal Intergen	14	3188284	3196683	8400	1	544990	uc033gnp.1	-6078	ENSMUSG000	Gm5795	predicted gene 5795
chr14	3202007	3202116	110	111	3202051	27	5.77405	2.5821	2.88921	Adipose_R2_peak_5	Intron (uc007s	14	3208493	3222029	13537	1	320333	uc007sar.1	-6377	ENSMUSG000	D830030K20R	RIKEN cDNA D830030K20 gene
chr14	3225606	3225770	165	166	3225636	28	6.25023	2.67431	3.31626	Adipose_R2_peak_5	Promoter (1-2	14	3224440	3234562	10123	1	320333	uc029seb.2	1166	ENSMUSG000	D830030K20R	RIKEN cDNA D830030K20 gene
chr14	3228648	3228923	276	277	3228714	35	9.76705	3.26985	6.56751	Adipose_R2_peak_5	Intron (uc007s	14	3224440	3234562	10123	1	320333	uc029seb.2	4208	ENSMUSG000	D830030K20R	RIKEN cDNA D830030K20 gene
chr14	3238132	3238326	195	196	3238231	41	13.59774	3.87315	10.1803	Adipose_R2_peak_5	Intron (uc007s	14	3224440	3234562	10123	1	320333	uc029seb.2	13692	ENSMUSG000	D830030K20R	RIKEN cDNA D830030K20 gene
chr14	3239822	3240128	307	308	3239977	49	9.82157	2.71531	6.61986	Adipose_R2_peak_5	Intron (uc007s	14	3224440	3234562	10123	1	320333	uc029seb.2	15382	ENSMUSG000	D830030K20R	RIKEN cDNA D830030K20 gene
chr14	3240568	3240718	151	152	3240629	39	6.2761	2.30985	3.34092	Adipose_R2_peak_5	Intron (uc007s	14	3224440	3234562	10123	1	320333	uc029seb.2	16128	ENSMUSG000	D830030K20R	RIKEN cDNA D830030K20 gene
chr14	3241068	3241304	237	238	3241194	40	12.96465	3.78093	9.57654	Adipose_R2_peak_5	Intron (uc007s	14	3224440	3234562	10123	1	320333	uc029seb.2	16628	ENSMUSG000	D830030K20R	RIKEN cDNA D830030K20 gene
chr14	3242384	3242564	181	182	3242444	39	12.34187	3.68871	8.98501	Adipose_R2_peak_5	Intron (uc007s	14	3224440	3234562	10123	1	320333	uc029seb.2	17944	ENSMUSG000	D830030K20R	RIKEN cDNA D830030K20 gene
chr14	3246714	3247175	462	463	3246878	30	7.24477	2.85875	4.21917	Adipose_R2_peak_5	Intron (uc007s	14	3262402	3263717	1316	1	73549	uc007sau.2	-15227	ENSMUSG000	1700110I01Ri	RIKEN cDNA 1700110I01 gene
chr14	3247364	3247504	141	142	3247425	32	8.29282	3.04319	5.18352	Adipose_R2_peak_5	Intron (uc007s	14	3262402	3263717	1316	1	73549	uc007sau.2	-14898	ENSMUSG000	1700110I01Ri	RIKEN cDNA 1700110I01 gene
chr14	3249287	3249686	400	401	3249338	29	6.74062	2.76653	3.75989	Adipose_R2_peak_5	Intron (uc007s	14	3262402	3263717	1316	1	73549	uc007sau.2	-12716	ENSMUSG000	1700110I01Ri	RIKEN cDNA 1700110I01 gene
chr14	3250273	3250411	139	140	3250329	33	8.83596	3.1354	5.68652	Adipose_R2_peak_5	Intron (uc007s	14	3262402	3263717	1316	1	73549	uc007sau.2	-11991	ENSMUSG000	1700110I01Ri	RIKEN cDNA 1700110I01 gene
chr14	3251663	3251874	212	213	3251745	40	8.56935	2.76112	5.44972	Adipose_R2_peak_5	Intron (uc007s	14	3262402	3263717	1316	1	73549	uc007sau.2	-10528	ENSMUSG000	1700110I01Ri	RIKEN cDNA 1700110I01 gene
chr14	3252634	3252729	96	97	3252640	25	4.86612	2.39766	2.09018	Adipose_R2_peak_5	Intron (uc007s	14	3262402	3263717	1316	1	73549	uc007sau.2	-9673	ENSMUSG000	1700110I01Ri	RIKEN cDNA 1700110I01 gene
chr14	3252828	3253021	194	195	3252936	37	8.07505	2.76322	4.99539	Adipose_R2_peak_5	Intron (uc007s	14	3262402	3263717	1316	1	73549	uc007sau.2	-9381	ENSMUSG000	1700110I01Ri	RIKEN cDNA 1700110I01 gene
chr14	3253360	3253466	107	108	3253421	36	10.50308	3.40306	7.26218	Adipose_R2_peak_5	Intron (uc007s	14	3262402	3263717	1316	1	73549	uc007sau.2	-8936	ENSMUSG000	1700110I01Ri	RIKEN cDNA 1700110I01 gene
chr14	3254858	3255186	329	330	3255050	54	19.18502	4.29948	15.56073	Adipose_R2_peak_5	Intron (uc007s	14	3262402	3263717	1316	1	73549	uc007sau.2	-7216	ENSMUSG000	1700110I01Ri	RIKEN cDNA 1700110I01 gene
chr14	3255332	3255461	130	131	3255387	39	11.76723	3.54486	8.44095	Adipose_R2_peak_5	Intron (uc007s	14	3262402	3263717	1316	1	73549	uc007sau.2	-6941	ENSMUSG000	1700110I01Ri	RIKEN cDNA 1700110I01 gene
chr14	3255743	3255875	133	134	3255802	33	8.83596	3.1354	5.68652	Adipose_R2_peak_5	Intron (uc007s	14	3262402	3263717	1316	1	73549	uc007sau.2	-6527	ENSMUSG000	1700110I01Ri	RIKEN cDNA 1700110I01 gene
chr14	3257709	3257889	181	182	3257794	39	10.16904	3.16078	6.93887	Adipose_R2_peak_5	Intron (uc007s	14	3262402	3263717	1316	1	73549	uc007sau.2	-4513	ENSMUSG000	1700110I01Ri	RIKEN cDNA 1700110I01 gene
chr14	3260078	3260350	273	274	3260229	38	7.1985	2.53288	4.20065	Adipose_R2_peak_5	Promoter (2-3	14	3262402	3263717	1316	1	73549	uc007sau.2	-2052	ENSMUSG000	1700110I01Ri	RIKEN cDNA 1700110I01 gene
chr14	3260694	3260834	141	142	3260768	42	8.45433	2.66906	5.33891	Adipose_R2_peak_5	Promoter (1-2	14	3262402	3263717	1316	1	73549	uc007sau.2	-1568	ENSMUSG000	1700110I01Ri	RIKEN cDNA 1700110I01 gene
chr14	3261033	3261223	191	192	3261096	39	6.92825	2.44539	3.9407	Adipose_R2_peak_5	Promoter (1-2	14	3262402	3263717	1316	1	73549	uc007sau.2	-1179	ENSMUSG000	1700110I01Ri	RIKEN cDNA 1700110I01 gene
chr14	3263412	3263507	96	97	3263436	36	5.95957	2.32033	3.06774	Adipose_R2_peak_5	Promoter (1-2	14	3262402	3263717	1316	1	73549	uc007sau.2	1010	ENSMUSG000	1700110I01Ri	RIKEN cDNA 1700110I01 gene
chr14	3263775	3263948	174	175	3263873	37	10.43006	3.32719	7.19119	Adipose_R2_peak_5	Promoter (1-2	14	3262402	3263717	1316	1	73549	uc007sau.2	1373	ENSMUSG000	1700110I01Ri	RIKEN cDNA 1700110I01 gene
chr14	3266932	3267176	245	246	3267070	39	10.77605	3.30397	7.51045	Adipose_R2_peak_5	Intron (uc007s	14	3262402	3263717	1316	1	73549	uc007sau.2	4530	ENSMUSG000	1700110I01Ri	RIKEN cDNA 1700110I01 gene
chr14	3268673	3269016	344	345	3268916	39	12.34187	3.68871	8.98501	Adipose_R2_peak_5	Intron (uc007s	14	3262402	3263717	1316	1	73549	uc007sau.2	6271	ENSMUSG000	1700110I01Ri	RIKEN cDNA 1700110I01 gene
chr14	3269337	3269519	183	184	3269400	26	7.87393	3.28786	4.83222	Adipose_R1_peak_4	Intron (uc007s	14	3262402	3263717	1316	1	73549	uc007sau.2	6935	ENSMUSG000	1700110I01Ri	RIKEN cDNA 1700110I01 gene
chr14	3269351	3269568	218	219	3269473	37	8.3191	2.81944	5.20898	Adipose_R2_peak_5	Intron (uc007s	14	3262402	3263717	1316	1	73549	uc007sau.2	6949	ENSMUSG000	1700110I01Ri	RIKEN cDNA 1700110I01 gene
chr14	3269934	3270088	155	156	3270014	33	8.83596	3.1354	5.68652	Adipose_R2_peak_5	Exon (uc007sa	14	3262402	3263717	1316	1	73549	uc007sau.2	7532	ENSMUSG000	1700110I01Ri	RIKEN cDNA 1700110I01 gene
chr14	3270280	3270461	182	183	3270344	35	9.95877	3.31984	6.73562	Adipose_R2_peak_5	Intron (uc007s	14	3262402	3263717	1316	1	73549	uc007sau.2	7878	ENSMUSG000	1700110I01Ri	RIKEN cDNA 1700110I01 gene
chr14	3270824	3271412	589	590	3270901	33	8.77548	3.11927	5.64822	Adipose_R2_peak_5	Exon (uc007sa	14	3262402	3263717	1316	1	73549	uc007sau.2	8422	ENSMUSG000	1700110I01Ri	RIKEN cDNA 1700110I01 gene
chr14	3273940	3274470	531	532	3273979	29	6.38074	2.66626	3.44184	Adipose_R2_peak_5	Intron (uc007s	14	3262402	3263717	1316	1	73549	uc007sau.2	11538	ENSMUSG000	1700110I01Ri	RIKEN cDNA 1700110I01 gene
chr14	3274631	3274857	227	228	3274737	35	9.95877	3.31984	6.73562	Adipose_R2_peak_5	Intron (uc007s	14	3262402	3263717	1316	1	73549	uc007sau.2	12229	ENSMUSG000	1700110I01Ri	RIKEN cDNA 1700110I01 gene
chr14	3275828	3276433	606	607	3276293	41	6.91004	2.38753	3.92302	Adipose_R2_peak_5	Intron (uc007s	14	3262402	3263717	1316	1	73549	uc007sau.2	13426	ENSMUSG000	1700110I01Ri	RIKEN cDNA 1700110I01 gene
chr14	3276671	3276946	276	277	3276762	47	13.26929	3.45592	9.87321	Adipose_R2_peak_5	Intron (uc007s	14	3262402	3263717	1316	1	73549	uc007sau.2	14269	ENSMUSG000	1700110I01Ri	RIKEN cDNA 1700110I01 gene
chr14	3277838	3278127	290	291	3277960	36	7.36814	2.6379	4.33764	Adipose_R2_peak_5	Intron (uc007s	14	3262402	3263717	1316	1	73549	uc007sau.2	15436	ENSMUSG000	1700110I01Ri	RIKEN cDNA 1700110I01 gene
chr14	3278885	3279212	328	329	3278996	39	10.62049	3.26697	7.35962	Adipose_R2_peak_5	Intron (uc007s	14	3262402	3263717	1316	1	73549	uc007sau.2	16483	ENSMUSG000	1700110I01Ri	RIKEN cDNA 1700110I01 gene
chr14	3280188	3280358	171	172	3280264	37	11.12817	3.50427	7.83613	Adipose_R2_peak_5	Intron (uc007s	14	3262402	3263717	1316	1	73549	uc007sau.2	17786	ENSMUSG000	1700110I01Ri	RIKEN cDNA 1700110I01 gene
chr14	3281463	3281576	114	115	3281508	33	8.83596	3.1354	5.68652	Adipose_R2_peak_5	Intron (uc007s	14	3262402	3263717	1316	1	73549	uc007sau.2	19061	ENSMUSG000	1700110I01Ri	RIKEN cDNA 1700110I01 gene
chr14	3281959	3282122	164	165	3282079	36	7.95054	2.77346	4.87557	Adipose_R2_peak_5	Intron (uc007s	14	3262402	3263717	1316	1	73549	uc007sau.2	19557	ENSMUSG000	1700110I01Ri	RIKEN cDNA 1700110I01 gene
chr14	3282231	3282440	210	211	3282298	32	8.29282	3.04319	5.18352	Adipose_R2_peak_5	Intron (uc007s	14	3262402	3263717	1316	1	73549	uc007sau.2	19829	ENSMUSG000	1700110I01Ri	RIKEN cDNA 1700110I01 gene
chr14	3300152	3300274	123	124	3300199	29	6.12387	2.59557	3.22565	Adipose_R2_peak_5	Intron (uc007s	14	3262402	3263717	1316	1	73549	uc007sau.2	37750	ENSMUSG000	1700110I01Ri	RIKEN cDNA 1700110I01 gene
chr14	3301270	3301408	139	140	3301326	27	5.77405	2.5821	2.88921	Adipose_R2_peak_5	Intron (uc007s	14	3262402	3263717	1316	1	73549	uc007sau.2	38868	ENSMUSG000	1700110I01Ri	RIKEN cDNA 1700110I01 gene
chr14	3301739	3302395	657	658	3301857	39	11.94276	3.58847	8.61122	Adipose_R2_peak_5	Intron (uc007s	14	3262402	3263717	1316	1	73549	uc007sau.2	39337	ENSMUSG000	1700110I01Ri	RIKEN cDNA 1700110I01 gene
chr14	3304810	3305031	222	223	3304909	43	14.8939	4.05758	11.42017	Adipose_R2_peak_5	Exon (uc007sa	14	3262402	3263717	1316	1	73549	uc007sau.2	42408	ENSMUSG000	1700110I01Ri	RIKEN cDNA 1700110I01 gene
chr14	3305573	3305709	137	138	3305650	25	9.14272	3.81298	6.01833	Adipose_R1_peak_4	Exon (uc007sa	14	3262402	3263717	1316	1	73549	uc007sau.2	43171	ENSMUSG000	1700110I01Ri	RIKEN cDNA 1700110I01 gene
chr14	3306398	3306492	95	96	3306444	26	5.31252	2.48988	2.47999	Adipose_R2_peak_5	Intron (uc007s	14	3262402	3263717	1316	1	73549	uc007sau.2	43996	ENSMUSG000	1700110I01Ri	RIKEN cDNA 1700110I01 gene
chr14	3307724	3307914	191	192	3307836	26	5.29021	2.48331	2.47999	Adipose_R2_peak_5	Intron (uc007s	14	3262402	3263717	1316	1	73549	uc007sau.2	45322	ENSMUSG000	1700110I01Ri	RIKEN cDNA 1700110I01 gene
chr14	3309768	3309994	227	228	3309869	40	12.55251	3.67818	9.18941	Adipose_R2_peak_5	Intron (uc007s	14	3262402	3263717	1316	1	73549	uc007sau.2	47366	ENSMUSG000	1700110I01Ri	RIKEN cDNA 1700110I01 gene
chr14	3310661	3310885	225	226	3310727	33	8.83596	3.1354	5.68652	Adipose_R2_peak_5	Intron (uc007s	14	3262402	3263717	1316	1	73549	uc007sau.2	48259	ENSMUSG000	1700110I01Ri	RIKEN cDNA 1700110I01 gene

chr14	3311658	3312170	513	514	3311761	27	5.75051	2.57529	2.88921	Adipose_R2_peak_	Intron (uc007s	14	3262402	3263717	1316	1	73549	uc007sau.2	49256	ENSMUSG000	1700110I01Ri	RIKEN cDNA 1700110I01 gene
chr14	3313056	3313347	292	293	3313184	27	5.77405	2.5821	2.88921	Adipose_R2_peak_	Intron (uc007s	14	3262402	3263717	1316	1	73549	uc007sau.2	50654	ENSMUSG000	1700110I01Ri	RIKEN cDNA 1700110I01 gene
chr14	3315617	3315927	311	312	3315790	32	7.9847	2.96049	4.90786	Adipose_R2_peak_	Intron (uc007s	14	3262402	3263717	1316	1	73549	uc007sau.2	53215	ENSMUSG000	1700110I01Ri	RIKEN cDNA 1700110I01 gene
chr14	3316836	3316976	141	142	3316926	30	7.21753	2.85121	4.21838	Adipose_R2_peak_	Intron (uc007s	14	3262402	3263717	1316	1	73549	uc007sau.2	54434	ENSMUSG000	1700110I01Ri	RIKEN cDNA 1700110I01 gene
chr14	3317283	3317386	104	105	3317344	34	8.62515	3.02816	5.5031	Adipose_R2_peak_	Intron (uc007s	14	3262402	3263717	1316	1	73549	uc007sau.2	54881	ENSMUSG000	1700110I01Ri	RIKEN cDNA 1700110I01 gene
chr14	3319841	3320075	235	236	3319957	32	7.71675	2.8894	4.67293	Adipose_R2_peak_	Intron (uc007s	14	3262402	3263717	1316	1	73549	uc007sau.2	57439	ENSMUSG000	1700110I01Ri	RIKEN cDNA 1700110I01 gene
chr14	3320362	3320501	140	141	3320403	17	5.26716	3.00037	2.45312	Adipose_R1_peak_	Intron (uc007s	14	3262402	3263717	1316	1	73549	uc007sau.2	57960	ENSMUSG000	1700110I01Ri	RIKEN cDNA 1700110I01 gene
chr14	3321109	3321262	154	155	3321183	36	10.5378	3.41206	7.27949	Adipose_R2_peak_	Intron (uc007s	14	3262402	3263717	1316	1	73549	uc007sau.2	58707	ENSMUSG000	1700110I01Ri	RIKEN cDNA 1700110I01 gene
chr14	3321488	3321631	144	145	3321548	34	9.39139	3.22762	6.20474	Adipose_R2_peak_	Intron (uc007s	14	3262402	3263717	1316	1	73549	uc007sau.2	59086	ENSMUSG000	1700110I01Ri	RIKEN cDNA 1700110I01 gene
chr14	3322079	3322344	266	267	3322260	38	9.08038	2.95374	5.92212	Adipose_R2_peak_	Intron (uc007s	14	3262402	3263717	1316	1	73549	uc007sau.2	59677	ENSMUSG000	1700110I01Ri	RIKEN cDNA 1700110I01 gene
chr14	3336894	3337103	210	211	3337016	57	16.61365	3.63728	13.08038	Adipose_R2_peak_	Intron (uc007s	14	3395093	3403534	8442	1	1.00E+08	uc011zfd.1	-57990	ENSMUSG000	Gm10413	predicted gene 10413
chr14	3338571	3338760	190	191	3338691	48	18.29934	4.51867	14.70076	Adipose_R2_peak_	Intron (uc007s	14	3395093	3403534	8442	1	1.00E+08	uc011zfd.1	-56333	ENSMUSG000	Gm10413	predicted gene 10413
chr14	3338626	3338762	137	138	3338694	30	8.35439	3.17324	5.30759	Adipose_R1_peak_	Intron (uc007s	14	3395093	3403534	8442	1	1.00E+08	uc011zfd.1	-56331	ENSMUSG000	Gm10413	predicted gene 10413
chr14	3339962	3340148	187	188	3340063	48	14.02189	3.5631	10.59392	Adipose_R2_peak_	Intron (uc007s	14	3395093	3403534	8442	1	1.00E+08	uc011zfd.1	-54945	ENSMUSG000	Gm10413	predicted gene 10413
chr14	3346446	3346636	191	192	3346560	45	15.96501	4.17814	12.45636	Adipose_R2_peak_	Intron (uc007s	14	3395093	3403534	8442	1	1.00E+08	uc011zfd.1	-48457	ENSMUSG000	Gm10413	predicted gene 10413
chr14	3347474	3347581	108	109	3347517	33	8.83596	3.1354	5.68652	Adipose_R2_peak_	Intron (uc007s	14	3395093	3403534	8442	1	1.00E+08	uc011zfd.1	-47512	ENSMUSG000	Gm10413	predicted gene 10413
chr14	3348916	3349012	97	98	3348964	30	7.24477	2.85875	4.21917	Adipose_R2_peak_	Intron (uc007s	14	3395093	3403534	8442	1	1.00E+08	uc011zfd.1	-46081	ENSMUSG000	Gm10413	predicted gene 10413
chr14	3354049	3354210	162	163	3354118	38	11.72962	3.59649	8.4047	Adipose_R2_peak_	Intron (uc007s	14	3395093	3403534	8442	1	1.00E+08	uc011zfd.1	-40883	ENSMUSG000	Gm10413	predicted gene 10413
chr14	3382531	3382705	175	176	3382578	31	7.7623	2.95097	4.69388	Adipose_R2_peak_	Intron (uc007s	14	3395093	3403534	8442	1	1.00E+08	uc011zfd.1	-12388	ENSMUSG000	Gm10413	predicted gene 10413
chr14	3385803	3385948	146	147	3385939	26	5.31252	2.48988	2.47999	Adipose_R2_peak_	Intron (uc007s	14	3395093	3403534	8442	1	1.00E+08	uc011zfd.1	-9145	ENSMUSG000	Gm10413	predicted gene 10413
chr14	3397775	3397926	152	153	3397859	41	13.59774	3.87315	10.1803	Adipose_R2_peak_	Promoter (2-3	14	3395093	3403534	8442	1	1.00E+08	uc011zfd.1	2682	ENSMUSG000	Gm10413	predicted gene 10413
chr14	3405117	3405505	389	390	3405191	34	6.46117	2.49531	3.51933	Adipose_R2_peak_	Intron (uc007s	14	3412614	3433857	21244	1	1.00E+08	uc007sba.2	-7109	ENSMUSG000	Gm10409	predicted pseudogene 8348
chr14	3405284	3405440	157	158	3405366	35	10.46345	3.4533	7.2965	Adipose_R1_peak_	Intron (uc007s	14	3412614	3433857	21244	1	1.00E+08	uc007sba.2	-7174	ENSMUSG000	Gm10409	predicted pseudogene 8348
chr14	3414743	3414848	106	107	3414787	28	6.25023	2.67431	3.31626	Adipose_R2_peak_	Promoter (2-3	14	3412614	3433857	21244	1	1.00E+08	uc007sba.2	2129	ENSMUSG000	Gm10409	predicted pseudogene 8348
chr14	3439174	3439392	219	220	3439271	29	6.74062	2.76653	3.75989	Adipose_R2_peak_	Intron (uc007s	14	3449312	3459279	9968	1	1.00E+08	uc029sec.1	-9920	ENSMUSG000	Gm10408	predicted gene 10408
chr14	3440324	3440511	188	189	3440387	35	9.95877	3.31984	6.73562	Adipose_R2_peak_	Intron (uc007s	14	3449312	3459279	9968	1	1.00E+08	uc029sec.1	-8801	ENSMUSG000	Gm10408	predicted gene 10408
chr14	3441089	3441346	258	259	3441186	26	5.31252	2.48988	2.47999	Adipose_R2_peak_	Intron (uc007s	14	3449312	3459279	9968	1	1.00E+08	uc029sec.1	-7966	ENSMUSG000	Gm10408	predicted gene 10408
chr14	3441468	3442060	593	594	3441572	31	7.06862	2.76401	4.07575	Adipose_R2_peak_	Intron (uc007s	14	3449312	3459279	9968	1	1.00E+08	uc029sec.1	-7252	ENSMUSG000	Gm10408	predicted gene 10408
chr14	3443998	3444097	100	101	3444091	26	5.31252	2.48988	2.47999	Adipose_R2_peak_	Intron (uc007s	14	3449312	3459279	9968	1	1.00E+08	uc029sec.1	-5215	ENSMUSG000	Gm10408	predicted gene 10408
chr14	3444309	3444440	132	133	3444371	27	5.77405	2.5821	2.88921	Adipose_R2_peak_	Intron (uc007s	14	3449312	3459279	9968	1	1.00E+08	uc029sec.1	-4872	ENSMUSG000	Gm10408	predicted gene 10408
chr14	3444690	3444850	161	162	3444765	32	8.12256	2.99736	5.04058	Adipose_R2_peak_	Intron (uc007s	14	3449312	3459279	9968	1	1.00E+08	uc029sec.1	-4462	ENSMUSG000	Gm10408	predicted gene 10408
chr14	3447581	3447958	378	379	3447862	35	9.95877	3.31984	6.73562	Adipose_R2_peak_	Promoter (1-2	14	3449312	3459279	9968	1	1.00E+08	uc029sec.1	-1354	ENSMUSG000	Gm10408	predicted gene 10408
chr14	3449477	3449669	193	194	3449591	44	15.09217	4.03703	11.61408	Adipose_R2_peak_	Promoter (<=	14	3449312	3459279	9968	1	1.00E+08	uc029sec.1	165	ENSMUSG000	Gm10408	predicted gene 10408
chr14	3455166	3455305	140	141	3455264	31	7.7623	2.95097	4.69388	Adipose_R2_peak_	Intron (uc007s	14	3449312	3459279	9968	1	1.00E+08	uc029sec.1	5854	ENSMUSG000	Gm10408	predicted gene 10408
chr14	3456897	3457059	163	164	3456961	23	9.86443	4.31371	6.70247	Adipose_R1_peak_	Intron (uc007s	14	3449312	3459279	9968	1	1.00E+08	uc029sec.1	7585	ENSMUSG000	Gm10408	predicted gene 10408
chr14	3457394	3457672	279	280	3457602	37	11.12817	3.50427	7.83613	Adipose_R2_peak_	Intron (uc007s	14	3449312	3459279	9968	1	1.00E+08	uc029sec.1	8082	ENSMUSG000	Gm10408	predicted gene 10408
chr14	3462180	3462448	269	270	3462209	31	5.37224	2.32692	2.53739	Adipose_R2_peak_	Intron (uc007s	14	3449312	3459279	9968	1	1.00E+08	uc029sec.1	12868	ENSMUSG000	Gm10408	predicted gene 10408
chr14	3463072	3463260	189	190	3463160	37	8.83136	2.93905	5.68652	Adipose_R2_peak_	Intron (uc007s	14	3449312	3459279	9968	1	1.00E+08	uc029sec.1	13760	ENSMUSG000	Gm10408	predicted gene 10408
chr14	3463781	3464054	274	275	3463966	35	9.01814	3.07816	5.86231	Adipose_R2_peak_	Intron (uc007s	14	3449312	3459279	9968	1	1.00E+08	uc029sec.1	14469	ENSMUSG000	Gm10408	predicted gene 10408
chr14	3464475	3464652	178	179	3464581	40	11.18614	3.34864	7.89193	Adipose_R2_peak_	Intron (uc007s	14	3449312	3459279	9968	1	1.00E+08	uc029sec.1	15163	ENSMUSG000	Gm10408	predicted gene 10408
chr14	3464751	3465072	322	323	3464805	36	8.32333	2.8617	5.21282	Adipose_R2_peak_	Intron (uc007s	14	3449312	3459279	9968	1	1.00E+08	uc029sec.1	15439	ENSMUSG000	Gm10408	predicted gene 10408
chr14	3464798	3464949	152	153	3464873	27	7.58971	3.12862	4.59104	Adipose_R1_peak_	Intron (uc007s	14	3449312	3459279	9968	1	1.00E+08	uc029sec.1	15486	ENSMUSG000	Gm10408	predicted gene 10408
chr14	3465319	3465424	106	107	3465349	29	5.80726	2.50938	2.92113	Adipose_R2_peak_	Intron (uc007s	14	3449312	3459279	9968	1	1.00E+08	uc029sec.1	16007	ENSMUSG000	Gm10408	predicted gene 10408
chr14	3477532	3477647	116	117	3477601	33	8.83596	3.1354	5.68652	Adipose_R2_peak_	Intron (uc007s	14	3504100	3505415	1316	1	73549	uc007sbc.2	-26453	ENSMUSG000	1700110I01Ri	RIKEN cDNA 1700110I01 gene
chr14	3479550	3479713	164	165	3479635	30	7.24477	2.85875	4.21917	Adipose_R2_peak_	Intron (uc007s	14	3504100	3505415	1316	1	73549	uc007sbc.2	-24387	ENSMUSG000	1700110I01Ri	RIKEN cDNA 1700110I01 gene
chr14	3480240	3480399	160	161	3480318	32	8.29282	3.04319	5.18352	Adipose_R2_peak_	Intron (uc007s	14	3504100	3505415	1316	1	73549	uc007sbc.2	-23701	ENSMUSG000	1700110I01Ri	RIKEN cDNA 1700110I01 gene
chr14	3484674	3484823	150	151	3484758	30	6.48137	2.65064	3.53843	Adipose_R2_peak_	Intron (uc007s	14	3504100	3505415	1316	1	73549	uc007sbc.2	-19277	ENSMUSG000	1700110I01Ri	RIKEN cDNA 1700110I01 gene
chr14	3497320	3497470	151	152	3497374	26	5.31252	2.48988	2.47999	Adipose_R2_peak_	Intron (uc007s	14	3504100	3505415	1316	1	73549	uc007sbc.2	-6630	ENSMUSG000	1700110I01Ri	RIKEN cDNA 1700110I01 gene
chr14	3500644	3500810	167	168	3500730	20	7.32897	3.59876	4.33346	Adipose_R1_peak_	Intron (uc007s	14	3504100	3505415	1316	1	73549	uc007sbc.2	-3290	ENSMUSG000	1700110I01Ri	RIKEN cDNA 1700110I01 gene
chr14	3506412	3506561	150	151	3506468	32	8.29282	3.04319	5.18352	Adipose_R2_peak_	Promoter (2-3	14	3504100	3505415	1316	1	73549	uc007sbc.2	2312	ENSMUSG000	1700110I01Ri	RIKEN cDNA 1700110I01 gene
chr14	3509562	3509916	355	356	3509611	30	7.24477	2.85875	4.21917	Adipose_R2_peak_	Intron (uc007s	14	3504100	3505415	1316	1	73549	uc007sbc.2	5462	ENSMUSG000	1700110I01Ri	RIKEN cDNA 1700110I01 gene
chr14	3511809	3511929	121	122	3511876	39	12.34187	3.68871	8.98501	Adipose_R2_peak_	Intron (uc007s	14	3504100	3505415	1316	1	73549	uc007sbc.2	7709	ENSMUSG000	1700110I01Ri	RIKEN cDNA 1700110I01 gene
chr14	3519185	3519342	158	159	3519228	28	6.25023	2.67431	3.31626	Adipose_R2_peak_	Intron (uc007s	14	3504100	3505415	1316	1	73549	uc007sbc.2	15085	ENSMUSG000	1700110I01Ri	RIKEN cDNA 1700110I01 gene
chr14	3545571	3545917	347	348	3545858	54	18.96709	4.25388	15.35709	Adipose_R2_peak_	Intron (uc007s	14	3504100	3505415	1316	1	73549	uc007sbc.2	41471	ENSMUSG000	1700110I01Ri	RIKEN cDNA 1700110I01 gene
chr14	3545743	3546089	347	348	3545841	42	16.37363	4.51523	12.96881	Adipose_R1_peak_	Intron (uc007s	14	3504100	3505415	1316	1	73549	uc007sbc.2	41643	ENSMUSG000	1700110I01Ri	RIKEN cDNA 1700110I01 gene
chr14	3546018	3546154	137	138	3546062	35	9.95877	3.31984	6.73562	Adipose_R2_peak_	Intron (uc007s	14	3504100	3505415	1316	1	73549	uc007sbc.2	41918	ENSMUSG000	1700110I01Ri	RIKEN cDNA 1700110I01 gene
chr14	3566322	3566418	97	98	3566399	28	6.25023	2.67431	3.31626	Adipose_R2_peak_	Intron (uc007s	14	3504100	3505415	1316	1	73549	uc007sbc.2	62222	ENSMUSG000	1700110I01Ri	RIKEN cDNA 1700110I01 gene
chr14	3570780	3570924	145	146	3570793	25	4.86612	2.39766	2.09018	Adipose_R2_peak_	Intron (uc007s	14	3634512	3642952	8441	1	1.00E+08	uc011zfe.1	-63588	ENSMUSG000	Gm10413	predicted gene 10413
chr14	3857970	3858122	153	154	3858022	25	7.01824	3.07405	4.07086	Adipose_R1_peak_	Intron (uc007s	14	3846245	3856308	10064	1	320333	uc057bea.1	11725	ENSMUSG000	D830030K20R	RIKEN cDNA D830030K20 gene
chr14	3858011	3858143	133	134	3858055	36	10.5378	3.41206	7.27949	Adipose_R2_peak_	Intron (uc007s	14	3846245	3856308	10064	1	320333	uc057bea.1	11766	ENSMUSG000	D830030K20R	RIKEN cDNA D830030K20 gene
chr14	3860699	3860878	180	181	3860784	41	10.97089	3.24842	7.69925	Adipose_R2_peak_	Intron (uc007s	14	3846245	3856308	10064	1	320333	uc057bea.1	14454	ENSMUSG000	D830030K20R	RIKEN cDNA D830030K20 gene
chr14	3866371	3866524	154	155	3866451	38	11.72962	3.59649	8.4047	Adipose_R2_peak_	Intron (uc007s	14	3846245	3856308	10064	1	320333	uc057bea.1	20126	ENSMUSG000	D830030K20R	RIKEN cDNA D830030K20 gene
chr14	3867572	3867691	120	121	3867628	29	6.74062	2.76653	3.75989	Adipose_R2_peak_	Intron (uc007s	14	3846245	3856308	10064	1	320333	uc057bea.1	21327	ENSMUSG000	D830030K20R	RIKEN cDNA D830030K20 gene
chr14	3868187	3868352	166	167	3868253	41	13.59774	3.87315	10.1803	Adipose_R2_peak_	Intron (uc007s	14	3846245	3856308	10064	1	320333	uc057bea.1	21942	ENSMUSG000	D830030K20R	RIKEN cDNA D830030K20 gene
chr14	3869602	3869763	162	163	3869696	40	11.18614	3.34864	7.89193	Adipose_R2_peak_	Intron (uc007s	14	3846245	3856308	10064	1	320333	uc057bea.1	23357	ENSMUSG000	D830030K20R	RIKEN cDNA D830030K20 gene
chr14	3881376	3881769	394	395	3881462	30	5.72204	2.4496	2.87422	Adipose_R2_peak_	Intron (uc007s	14	3846245	3856308	10064	1	320333	uc057bea.1	35131	ENSMUSG000	D830030K20R	RIKEN cDNA D830030K20 gene
chr14	3893014	3893129	116	117	3893020	25	4.86612	2.39766	2.09018	Adipose_R2_peak_	Intron (uc007s	14	3846245	3856308	10064	1	320333	uc057bea.1	46769	ENSMUSG000	D830030K20R	RIKEN cDNA D830030K20 gene
chr14	3894756	3895130	375	376	3894871	31	7.7623	2.95097	4.69388	Adipose_R2_peak_	Intron (uc007s	14	3846245	3856308	10064	1	320333	uc057bea.1	48511	ENSMUSG000	D830030K20R	RIKEN cDNA D830030K20 gene
chr14	3895328	3895523	196	197	3895399	36	10.5378	3.41206	7.27949	Adipose_R2_peak_	Intron (uc007s	14	3846245	3856308	10064	1	320333	uc057bea.1	49083	ENSMUSG000	D830030K20R	RIKEN cDNA D830030K20 gene
chr14	3895767	3895880	114	115	3895810	27	5.77405	2.5821	2.88921	Adipose_R2_peak_	Intron (uc007s	14	3846245	3856308	10064	1	320333	uc057bea.1	49522	ENSMUSG000	D830030K20R	RIKEN cDNA D830030K20 gene
chr14	3896203	3896330	128	129	3896261	27	5.77405	2.5821	2.88921	Adipose_R2_peak_	Intron (uc007s	14	3846245	3856308	10064	1	320333	uc057bea.1	49958	ENSMUSG000	D830030K20R	RIKEN cDNA D830030K20 gene
chr14	3896849	3897319	471	472	3896894	27	5.77405	2.5821	2.88921	Adipose_R2_peak_	Intron (uc007s	14	3846245	3856308	10064	1	320333	uc057bea.1	50604	ENSMUSG000	D830030K20R	RIKEN cDNA D830030K20 gene
chr14	3899863	3900379	517	518	3900089	41	13.59774	3.87315	10.1803	Adipose_R2_peak_	Intron (uc007s	14	3846245	3856308	10064	1	320333	uc057bea.1	53618	ENSMUSG000	D830030K20R	RIKEN cDNA D830030K20 gene
chr14	3900948	3901088	141	142	3901022	29	6.74062	2.76653	3.75989	Adipose_R2_peak_	Intron (uc007s	14	3846245	3856308	10064	1	320333	uc057bea.1	54703	ENSMUSG000	D830030K20R	RIKEN cDNA D830030K20 gene
chr14	3901911	3902028	118	119	3901975	27	5.53046	2.51193	2.68965	Adipose_R2_peak_	Intron (uc007s	14	3846245	3856308	10064	1	320333	uc057bea.1	55666	ENSMUSG000	D830030K20R	RIKEN cDNA D830030K20 gene
chr14	3912406	3912678	273	274	3912478	37	11.12817	3.50427	7.83613	Adipose_R2_peak_	Intron (uc007s	14	3963004	4131858	168855	1	545007	uc007sbk.1	-50326	ENSMUSG000	Gm5796	predicted gene 5796
chr14	3913165	3913391	227	228	3913291	34	9.39139	3.22762	6.20474	Adipose_R2_peak_	Intron (uc007s	14	3963004	4131858	168855	1	545007	uc007sbk.1	-49613	ENSMUSG000	Gm5796	predicted gene 5796
chr14	3922388	3922841	454	455	3922573	66	20.09756	3.8386	16.44891	Adipose_R2_peak_	Intron (uc007s	14	3963004	4131858	168855	1	545007	uc007sbk.1	-40163	ENSMUSG000	Gm5796	predicted gene 5796
chr14	3922434	3922822	389	390	3922575	32	7.65072	2.872	4.65141	Adipose_R1_peak_	Intron (uc007s	14	3963004	4131858	168855	1	545007	uc007sbk.1	-40182	ENSMUSG000	Gm5796	predicted gene 5796
chr14	3928707	3928879	173	174	3928789	31	6.60994	2.64318	3.66189	Adipose_R2_peak_	Intron (uc007s	14	3963004	4131858	168855	1	545007	uc007sbk.1	-34125	ENSMUSG000	Gm5796	predicted gene 5796
chr14	3932860	3933055	196	197	3932951	38	11.72962	3.59649	8.4047	Adipose_R2_peak_	Intron (uc007s	14	3963004	4131858	168855	1	545007	uc007sbk.1	-29949	ENSMUSG000	Gm5796	predicted gene 5796
chr14	3934663	3934759	97	98	3934702	28	6.25023	2.67431	3.31626	Adipose_R2_peak_	Intron (uc007s	14	3963004	4131858	168855	1	545007	uc007sbk.1	-28245	ENSMUSG000	Gm5796	predicted gene 5796
chr14	3935151	3935325	175	176	3935219	32	8.29282	3.04319	5.18352	Adipose_R2_peak_	Intron (uc007s	14	3963004	4131858	168855	1	545007	uc007sbk.1	-27679	ENSMUSG000	Gm5796	predicted gene 5796
chr14	3936583	3936723	141	142	3936649	32	8.29282	3.04319	5.18352	Adipose_R2_peak_	Intron (uc007s	14	3963004	4131858	168855	1	545007	uc007sbk.1	-26281	ENSMUSG000	Gm5796	predicted gene 5796
chr14	3940469	3940610	142	143	3940526	33	8.83596	3.1354	5.68652	Adipose_R2_peak_	Intron (uc007s	14	3963004	4131858	168855	1	545007	uc007sbk.1	-22394	ENSMUSG000	Gm5796	predicted gene 5796
chr14	3950818	3951034	217	218	3950890	37	11.12817	3.50427	7.83613	Adipose_R2_peak_	Intron (uc007s	14	3963004	4131858	168855	1	545007	uc007sbk.1	-11970	ENSMUSG000	Gm5796	predicted gene 5796
chr14	3952915	3953025	111	112	3952957	37	11.12817	3.50427	7.83613	Adipose_R2_peak_	Intron (uc007s	14	3963004	4131858	168855	1	545007	uc007sbk.1	-9979	ENSMUSG000	Gm5796	predicted gene 5796
chr14	3967744	3967878	135	136	3967801	29	6.74062	2.76653	3.75989	Adipose_R2_peak_	Intron (uc007s	14	3963547	4516826	553280	1	1.00E+08	uc007sbo.2	4197	ENSMUSG000	Gm3264	predicted gene 3264
chr14	3973215	3973405	191	192	3973312	32	8.29282	3.04319	5.18352	Adipose_R2_peak_	Intron (uc007s	14	3963547	4516826	553280	1	1.00E+08	uc007sbo.2	9668	ENSMUSG000	Gm3264	predicted gene 3264
chr14	3975734	3976014	281	282	3975866	35	7.95007	2.81421	4.87526	Adipose_R2_peak_	Intron (uc007s	14	3963547	4516826	553280	1	1.00E+08	uc007sbo.2	12187	ENSMUSG000	Gm3264	predicted gene 3264
chr14	3978777	3979018	242	243	3978847	34	7.22482	2.67861	4.21917	Adipose_R2_peak_	Intron (uc007s	14	3963547	4516826	553280	1	1.00E+08	uc007sbo.2	15230	ENSMUSG000	Gm3264	predicted gene 3264
chr14	3979501	3979682	182	183	3979571	40	12.96465	3.78093	9.57654	Adipose_R2_peak_	Intron (uc007s	14	3963547	4516826	553280	1	1.00E+08	uc007sbo.2	15954	ENSMUSG000	Gm3264	predicted gene 3264
chr14	3980724	3980959	236	237	3980818	29	6.74062	2.76653	3.75989	Adipose_R2_peak_	Intron (uc007s	14	3963547	4516826	553280	1	1.00E+08	uc007sbo.2	17177	ENSMUSG000	Gm3264	predicted gene 3264
chr14	3986146	3986359	214	215	3986262	30	7.24477	2.85875	4.21917	Adipose_R2_peak_	Intron (uc007s	14	3963547	4516826	553280	1	1.00E+08	uc007sbo.2	22599	ENSMUSG000	Gm3264	predicted gene 3264

chr14	3986491	3986650	160	161	3986594	18	5.94424	3.20533	3.06251	Adipose_R1_peak_4	Intron (uc007s	14	3963547	4516826	553280	1	1.00E+08	uc007sbo.2	22944	ENSMUSG000	Gm3264	predicted gene 3264
chr14	3986509	3986672	164	165	3986557	29	6.74062	2.76653	3.75989	Adipose_R2_peak_5	Intron (uc007s	14	3963547	4516826	553280	1	1.00E+08	uc007sbo.2	22962	ENSMUSG000	Gm3264	predicted gene 3264
chr14	3987085	3987231	147	148	3987147	28	6.25023	2.67431	3.31626	Adipose_R2_peak_5	Intron (uc007s	14	3963547	4516826	553280	1	1.00E+08	uc007sbo.2	23538	ENSMUSG000	Gm3264	predicted gene 3264
chr14	3987534	3987682	149	150	3987632	39	12.34187	3.68871	8.98501	Adipose_R2_peak_5	Intron (uc007s	14	3963547	4516826	553280	1	1.00E+08	uc007sbo.2	23987	ENSMUSG000	Gm3264	predicted gene 3264
chr14	3989226	3989435	210	211	3989352	39	8.09297	2.69378	5.01232	Adipose_R2_peak_5	Intron (uc007s	14	3963547	4516826	553280	1	1.00E+08	uc007sbo.2	25679	ENSMUSG000	Gm3264	predicted gene 3264
chr14	3989816	3990033	218	219	3989932	37	11.12817	3.50427	7.83613	Adipose_R2_peak_5	Intron (uc007s	14	3963547	4516826	553280	1	1.00E+08	uc007sbo.2	26269	ENSMUSG000	Gm3264	predicted gene 3264
chr14	3991873	3992005	133	134	3991904	25	4.86612	2.39766	2.09018	Adipose_R2_peak_5	Intron (uc007s	14	3963547	4516826	553280	1	1.00E+08	uc007sbo.2	28326	ENSMUSG000	Gm3264	predicted gene 3264
chr14	3992408	3992543	136	137	3992491	32	8.29282	3.04319	5.18352	Adipose_R2_peak_5	Intron (uc007s	14	3963547	4516826	553280	1	1.00E+08	uc007sbo.2	28861	ENSMUSG000	Gm3264	predicted gene 3264
chr14	3993800	3993952	153	154	3993867	27	5.63935	2.54321	2.79469	Adipose_R2_peak_5	Intron (uc007s	14	4023941	4041368	17428	1	545007	uc007sbl.1	-29989	ENSMUSG000	Gm5796	predicted gene 5796
chr14	3995335	3995792	458	459	3995398	35	6.49265	2.47002	3.54935	Adipose_R2_peak_5	Intron (uc007s	14	4023941	4041368	17428	1	545007	uc007sbl.1	-28149	ENSMUSG000	Gm5796	predicted gene 5796
chr14	3996928	3997249	322	323	3997119	41	12.80165	3.67742	9.43177	Adipose_R2_peak_5	Intron (uc007s	14	4023941	4041368	17428	1	545007	uc007sbl.1	-26692	ENSMUSG000	Gm5796	predicted gene 5796
chr14	3997556	3997799	244	245	3997602	30	7.24477	2.85875	4.21917	Adipose_R2_peak_5	Intron (uc007s	14	4023941	4041368	17428	1	545007	uc007sbl.1	-26142	ENSMUSG000	Gm5796	predicted gene 5796
chr14	3999526	3999709	184	185	3999591	32	8.29282	3.04319	5.18352	Adipose_R2_peak_5	Intron (uc007s	14	4023941	4041368	17428	1	545007	uc007sbl.1	-24232	ENSMUSG000	Gm5796	predicted gene 5796
chr14	4000905	4001066	162	163	4000970	33	8.83596	3.1354	5.68652	Adipose_R2_peak_5	Intron (uc007s	14	4023941	4041368	17428	1	545007	uc007sbl.1	-22875	ENSMUSG000	Gm5796	predicted gene 5796
chr14	4003250	4003419	170	171	4003362	36	10.5378	3.41206	7.27949	Adipose_R2_peak_5	Intron (uc007s	14	4023941	4041368	17428	1	545007	uc007sbl.1	-20522	ENSMUSG000	Gm5796	predicted gene 5796
chr14	4007069	4007219	151	152	4007126	31	7.7623	2.95097	4.69388	Adipose_R2_peak_5	Intron (uc007s	14	4023941	4041368	17428	1	545007	uc007sbl.1	-16722	ENSMUSG000	Gm5796	predicted gene 5796
chr14	4017676	4017860	185	186	4017783	29	6.74062	2.76653	3.75989	Adipose_R2_peak_5	Intron (uc007s	14	4023941	4041368	17428	1	545007	uc007sbl.1	-6081	ENSMUSG000	Gm5796	predicted gene 5796
chr14	4022379	4022515	137	138	4022424	36	10.5378	3.41206	7.27949	Adipose_R2_peak_5	Promoter (1-2	14	4023941	4041368	17428	1	545007	uc007sbl.1	-1426	ENSMUSG000	Gm5796	predicted gene 5796
chr14	4070708	4070832	125	126	4070771	30	7.24477	2.85875	4.21917	Adipose_R2_peak_5	Intron (uc007s	14	4023941	4041368	17428	1	545007	uc007sbl.1	46767	ENSMUSG000	Gm5796	predicted gene 5796
chr14	4093579	4093738	160	161	4093697	25	11.36566	4.67319	8.13482	Adipose_R1_peak_4	Intron (uc007s	14	4023941	4041368	17428	1	545007	uc007sbl.1	69638	ENSMUSG000	Gm5796	predicted gene 5796
chr14	4100093	4100249	157	158	4100173	33	8.83596	3.1354	5.68652	Adipose_R2_peak_5	Intron (uc007s	14	4023941	4041368	17428	1	545007	uc007sbl.1	76152	ENSMUSG000	Gm5796	predicted gene 5796
chr14	4101141	4101259	119	120	4101199	34	9.05733	3.13991	5.90027	Adipose_R2_peak_5	Intron (uc007s	14	4023941	4041368	17428	1	545007	uc007sbl.1	77200	ENSMUSG000	Gm5796	predicted gene 5796
chr14	4107520	4107816	297	298	4107627	30	7.24477	2.85875	4.21917	Adipose_R2_peak_5	Intron (uc007s	14	4023941	4041368	17428	1	545007	uc007sbl.1	83579	ENSMUSG000	Gm5796	predicted gene 5796
[truncated: 1,801,423 more chars]
